# Supplementary material for: Distribution of the four type VI secretion systems in Pseudomonas aeruginosa and classification of their core and accessory effectors
Source: Nat Commun. 2025 Jan 21;16:888. doi: 10.1038/s41467-024-54649-5 (PMC11751169; doi:10.1038/s41467-024-54649-5)
Supplement: Supplementary file 1 — Supplementary Information [file 41467_2024_54649_MOESM1_ESM.pdf]

Distribution of the four type VI secretion systems in *Pseudomonas aeruginosa*

and classification of their core and accessory effectors

Supplementary Figures

This file includes:

Supplementary Figures 1-39

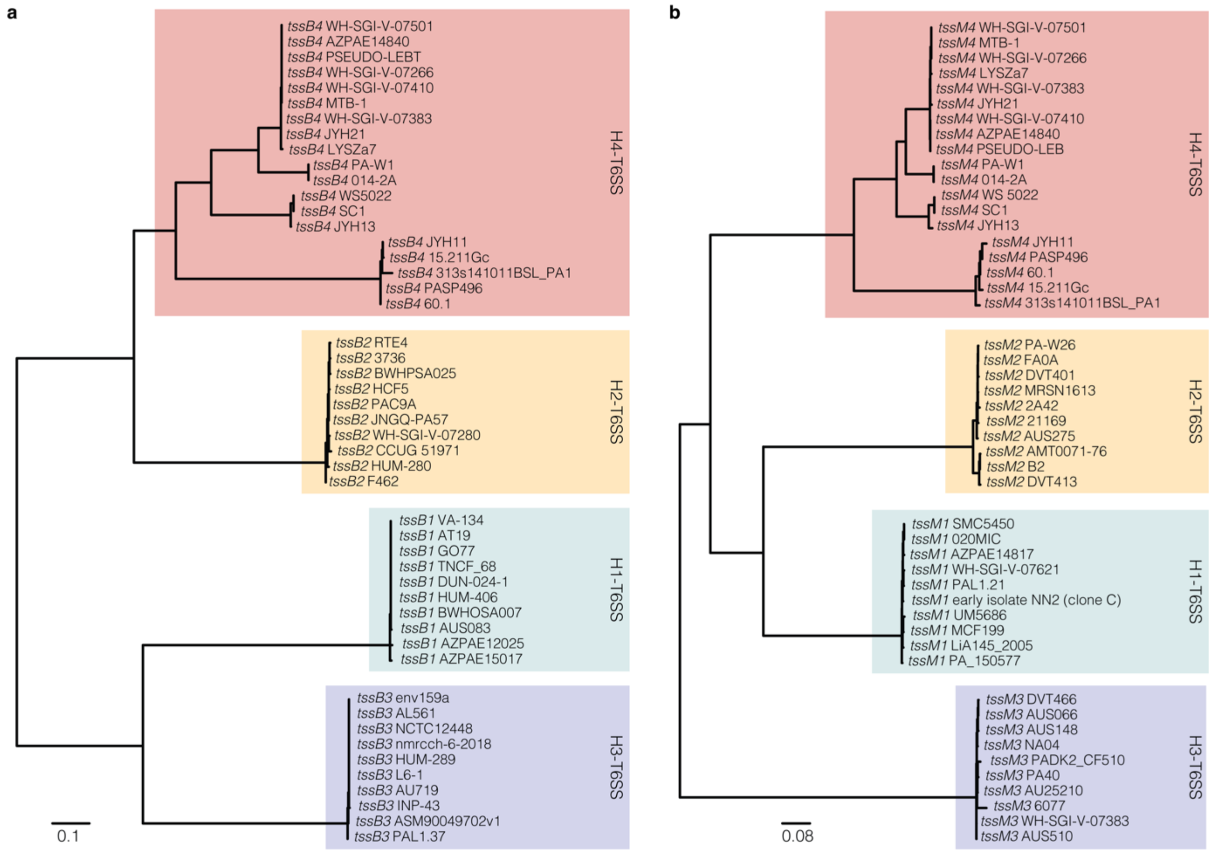

Supplementary Fig. 1. Four distinct clades of the *tssB* and *tssM* sequences of the H1-, H2-, H3-, and H4-T6SSs. Maximum-likelihood phylogenetic trees of *tssB* (**a**) and *tssM* (**b**) genes from the H1-, H2-, H3-, H4-T6SSs show separation into four separate clades, as indicated by the coloured boxes, demonstrating sequence diversity. Sequences of the *tssB* and *tssM* genes of up to 10 randomly chosen strains per H1-, H2-, and H3-T6SS and all available *tssB* and *tssM* sequences of the H4-T6SS were used. Strain names are indicated in the tree. Both trees are computed with the TPM3u+F+R2 model and are midpoint rooted. Distances are shown in substitutions per site.

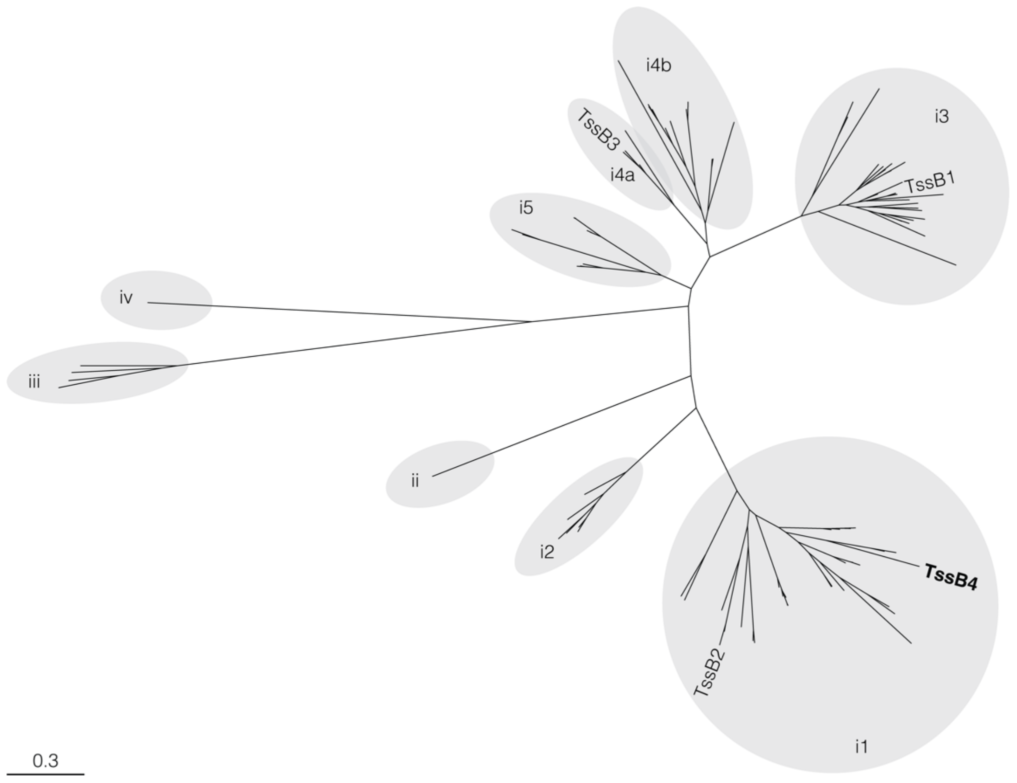

Supplementary Fig. 2. The H4-T6SS belongs to T6SS subgroup i1. Maximum likelihood tree of amino acid sequences of experimentally validated TssB proteins[1] and the TssB4 sequence of strain 60.1 (indicated in bold). TssB proteins from *P. aeruginosa* reference strain PAO1 are labelled. T6SS subgroups are indicated in grey. Distances are shown in substitutions per site. The tree was based on a multiple sequence alignment calculated with MAFFT (v7.475) and inferred with FastTree (v2.1.11) on the SeCreT6 website.

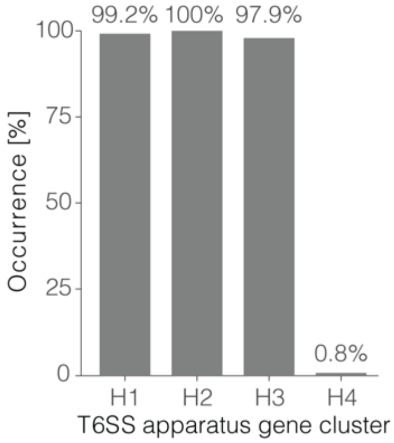

Supplementary Fig. 3. Bar graph indicating the occurrence of the four T6SS apparatus gene clusters among complete *P. aeruginosa* genomes (n=239).

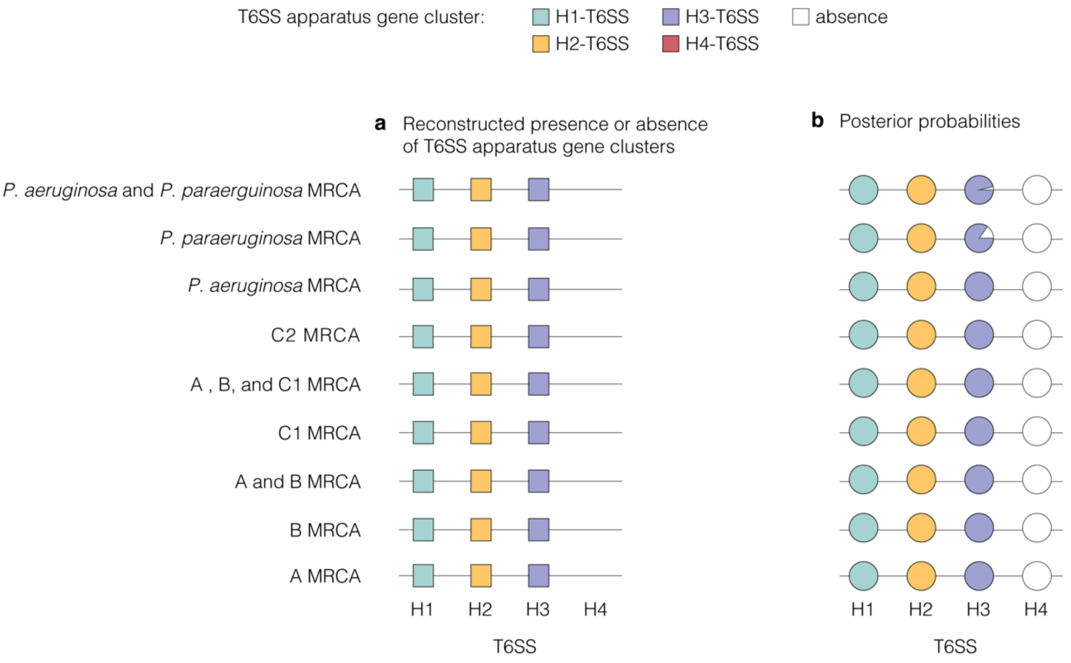

Supplementary Fig. 4. Reconstruction of T6SS apparatus gene clusters in the indicated most recent common ancestors (MRCAs). a, Graphical depiction summarising the results of the stochastic mapping. Raw data with posterior probabilities is shown in panel **b**. **b**, Posterior probabilities from stochastic character mapping for ancestral nodes of indicated MRCAs. Posterior probabilities were extracted from respective nodes from Supplementary Data 8.

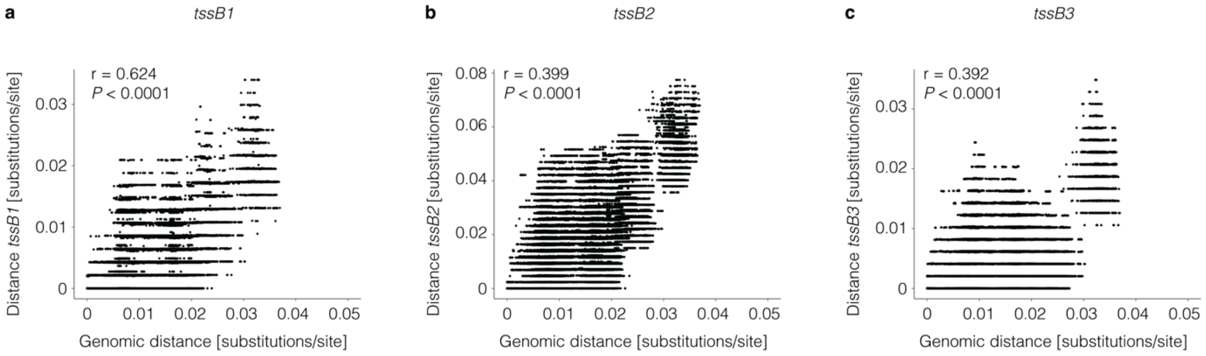

Supplementary Fig. 5. Pairwise comparison of phylogenetic distance of *tssB1* (**a**), *tssB2* (**b**), or *tssB3* (**c**), against genomic distance. Distances are calculated from maximum-likelihood phylogenetic trees. The species genomic distance was inferred using the HKY+F+I model. The *tssB* trees were calculated with the TN+F+R2 (*tssB1*), TN+F+I+G4 (*tssB2*), or TPM2+F+G4 (*tssb3*) model. Correlation analysis was performed using Spearman's rank correlation.

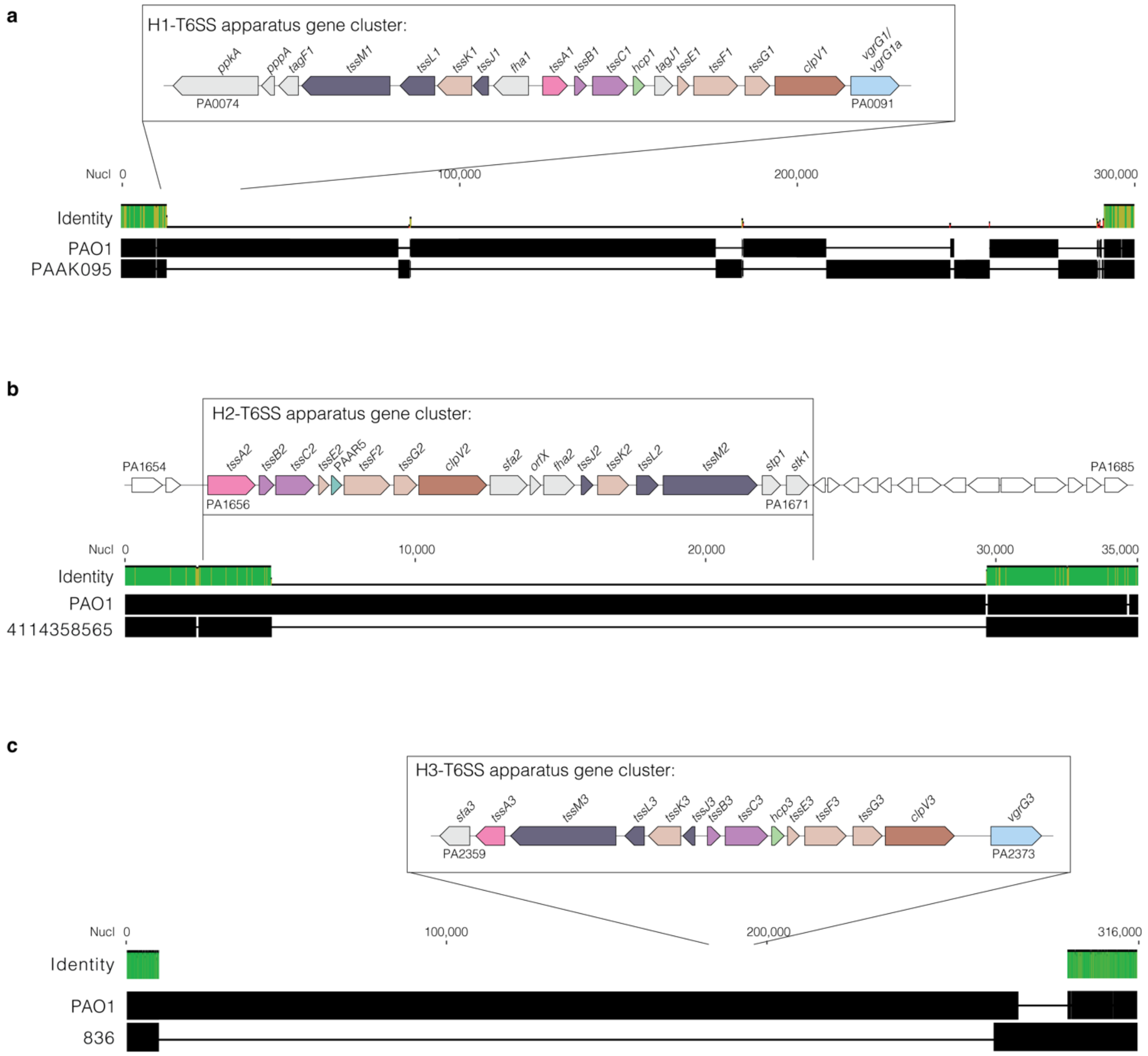

Supplementary Fig. 6. Examples of genomic deletions including genes of the H1-, H2-, and H3-T6SS apparatus gene clusters. Nucleotide alignments of the laboratory reference strain PAO1 and an exemplary strain missing either the H1- (**a**), H2- (**b**), or the H3-T6SS (**c**). Sequences were aligned with the progressiveMAUVE algorithm in Geneious (v2019.2.3).

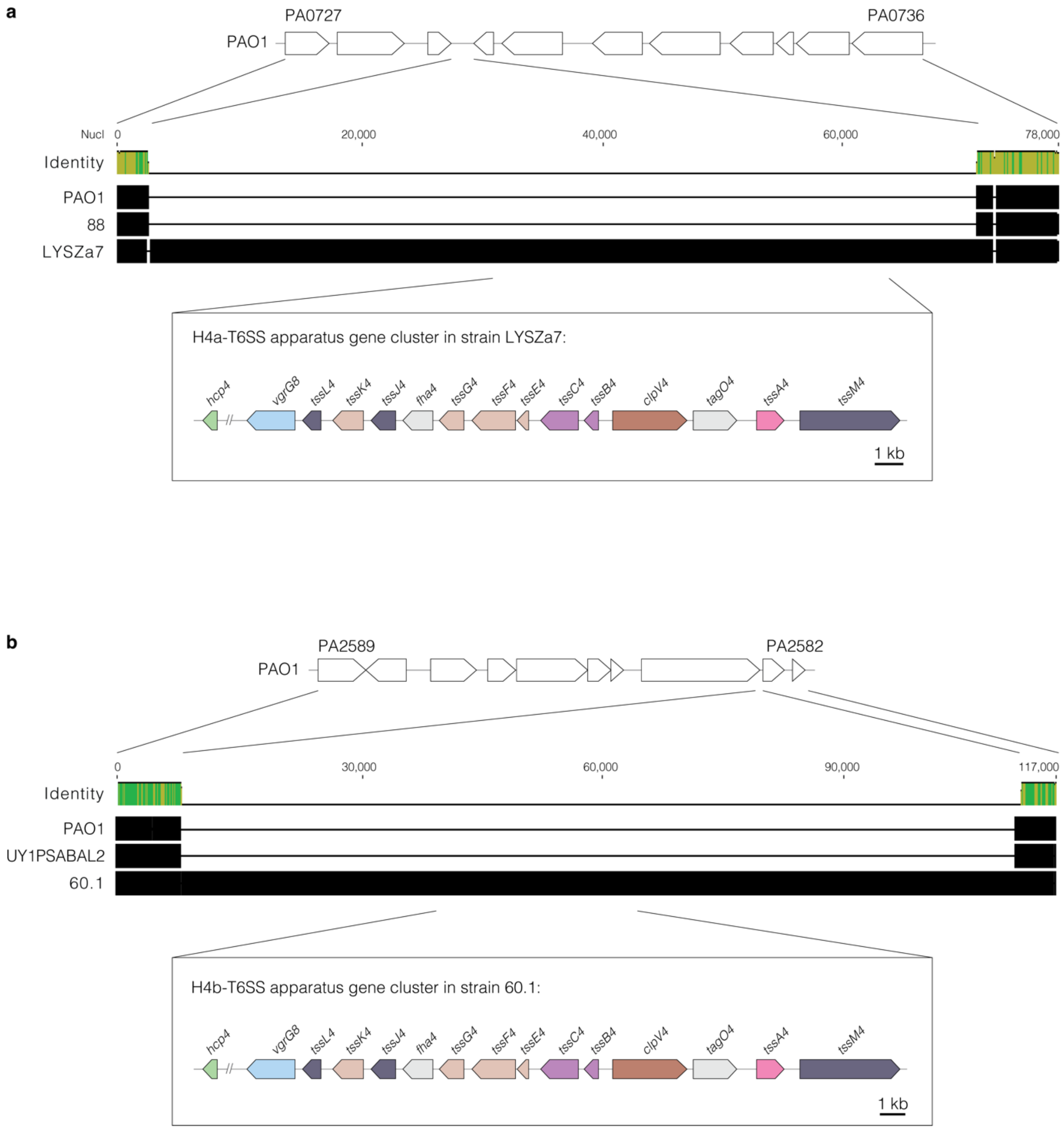

Supplementary Fig. 7. Genomic insertions at two genomic loci including genes of the H4-T6SS apparatus gene clusters. Nucleotide alignments of the indicated genome regions of exemplary strains with an H4-T6SS, their closest relative strain in the population, and the laboratory reference strain PAO1 indicating an insertion sequence that includes the H4a- (**a**) and H4b-T6SS apparatus gene clusters (**b**). Sequences were aligned with the progressiveMAUVE algorithm in Geneious (v2019.2.3).

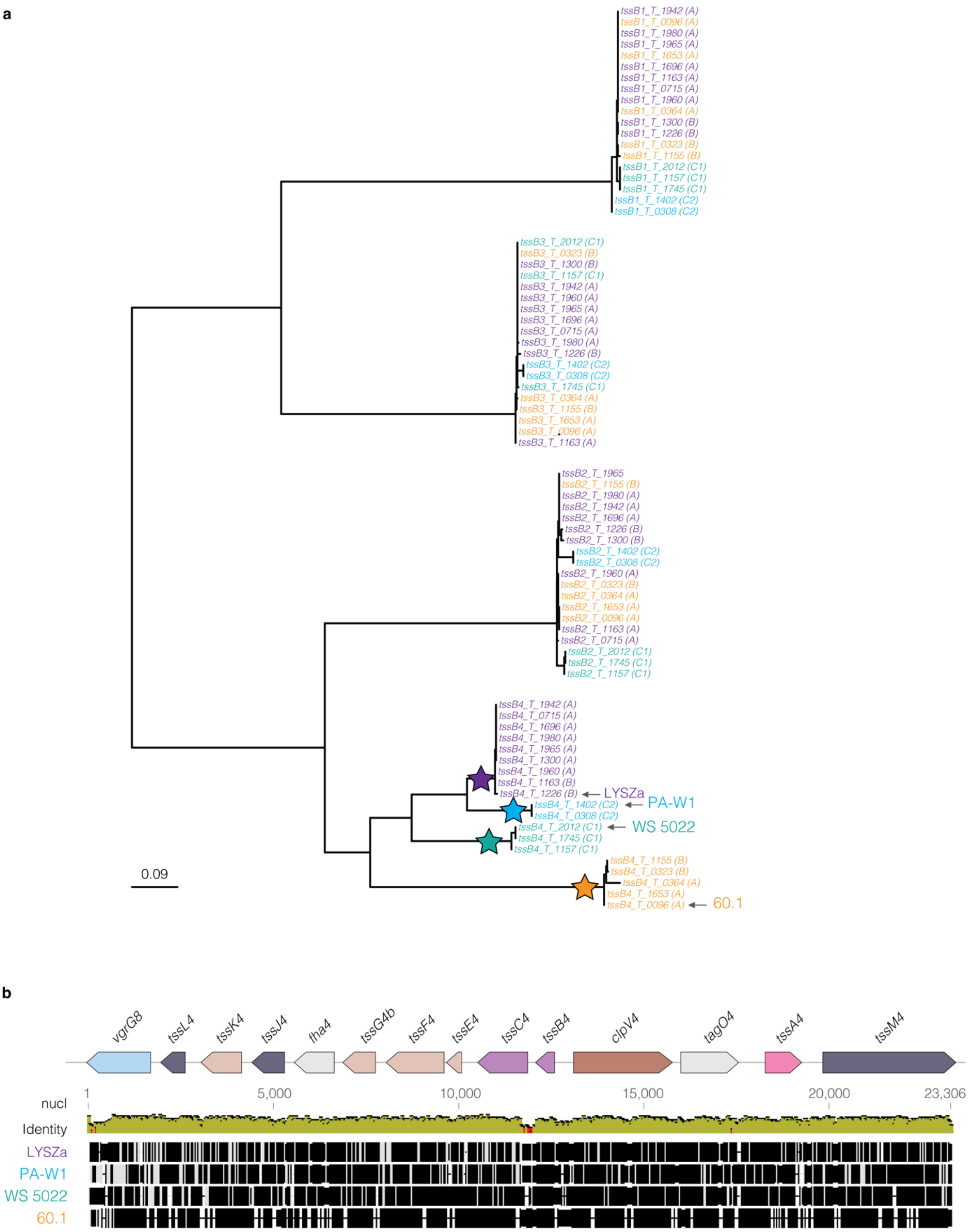

Supplementary Fig. 8. The *tssB* homologues of *P. aeruginosa* phylogenetically clade into four main branches corresponding to *tssB1* (H1-T6SS), *tssB2* (H2-T6SS), *tssB3* (H3-T6SS), and *tssB4* (H4-T6SS) across the phylogroups. a, Colouring same as in Figure 3. Phylogroups are indicated in brackets (A-C2). The tree was calculated using the TPM3+F+G4 model and 1000 ultrafast bootstraps using *tssB1, tssB2, tssB3, and tssB4* sequences in *P. aeruginosa* strains that have a H4-T6SS. This Maximum-likelihood tree is midpoint rooted

and distances are shown in substitutions per site. Stars indicate the four sub-branches of the *tssB4* clade and relate directly to Figure 3. **b,** Nucleotide alignment of the H4-T6SS apparatus gene clusters of one representative strain of each of the four clades with differing *tssB* sequences, showing no difference in synteny and only minor differences the genes' nucleotide sequences. Sequences were aligned in Geneious (v2019.2.3) using MUSCLE (v3.8.425).

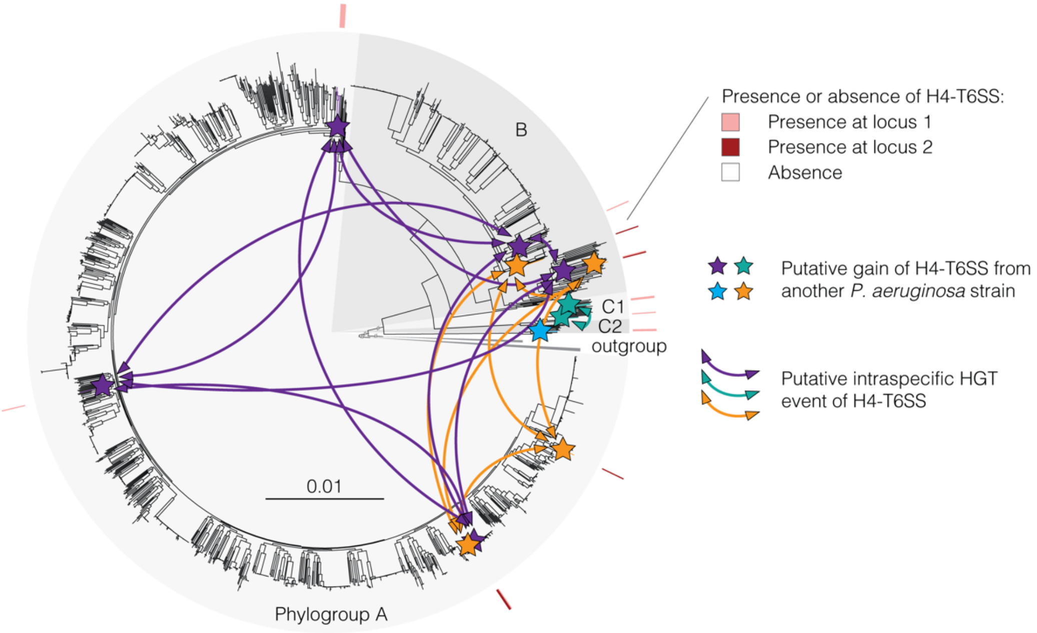

Supplementary Fig. 9. Demonstration of lateral transfer of H4-T6SS apparatus gene clusters among distantly related strains. Phylogenetic tree of 1960 *P. aeruginosa* strains based on a core-genome alignment (as shown in Figure 2). Phylogroups A, B, C1, and C2 are highlighted with grey shading. Each star refers to an acquisition event of the H4-T6SS predicted by stochastic mapping. Stars with very similar *tssB* sequences are filled with the same colour (see Figure 3 and alignment in Supplementary Fig. 8b) and depict putative intraspecific transfer by lateral gene transfer. The lines in the outer ring indicate at which position the H4-T6SS apparatus gene cluster is found in the genome.

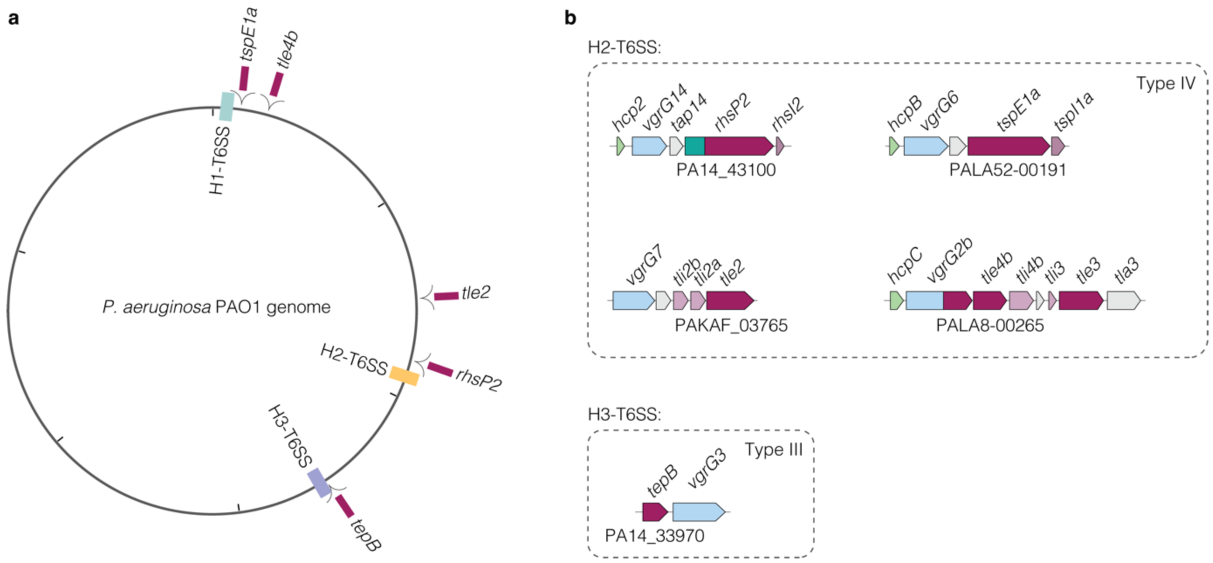

Supplementary Fig. 10. Additional T6SS effectors encoded in other *P. aeruginosa* genomes but not present in the PAO1 genome. a, Schematic of the PAO1 genome indicating the equivalent position of the individual genes (in maroon). **b**, Graphical depiction of the genomic organisation of the 5 effector loci. Dotted lines group effector genes by the type of their genomic organization (compare Figure 4c).

a

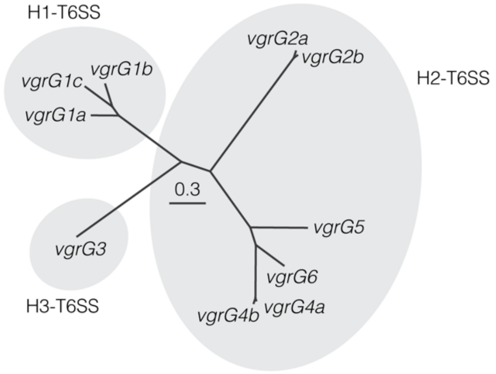

b

	vgrG1a	*vgrG1b*	*vgrG1c*	*vgrG2a*	*vgrG2b*	*vgrG3*	*vgrG4a*	*vgrG4b*	*vgrG5*	*vgrG6*
vgrG1a		70.31%	70.24%	39.62%	33.28%	47.59%	49.77%	43.23%	43.51%	49.38%
vgrG1b	70.31%		72.52%	43.51%	36.49%	48.61%	48.75%	44.17%	44.37%	50.02%
vgrG1c	70.24%	72.52%		39.57%	33.18%	46.97%	47.98%	42.84%	41.70%	47.67%
vgrG2a	39.62%	43.51%	39.57%		76.99%	38.69%	41.80%	43.10%	43.78%	42.41%
vgrG2b	33.25%	36.49%	33.18%	76.99%		32.21%	34.98%	37.25%	38.06%	35.45%
vgrG3	47.59%	48.61%	46.97%	38.69%	32.21%		48.16%	41.53%	42.35%	48.23%
vgrG4a	49.77%	48.75%	47.98%	41.80%	34.98%	48.16%		81.95%	63.11%	75.28%
vgrG4b	43.23%	44.17%	42.84%	43.10%	37.25%	41.53%	81.95%		65.48%	65.56%
vgrG5	43.51%	44.37%	41.70%	43.78%	38.06%	42.35%	63.11%	65.48%		64.08%
vgrG6	49.38%	50.02%	47.67%	42.41%	35.45%	48.23%	75.28%	65.56%	64.08%	

c

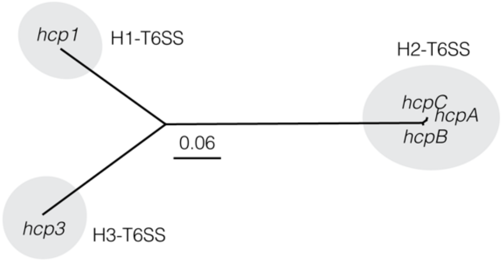

d

	hcp1	*hcpA*	*hcpB*	*hcpC*	*hcp3*
hcp1		44.75%	45.29%	44.57%	53.96%
hcpA	44.75%		98.84%	99.61%	44.58%
hcpB	45.29%	98.84%		98.46%	44.58%
hcpC	44.57%	99.61%	98.46%		44.58%
hcp3	53.96%	44.58%	44.58%	44.58%	

Supplementary Figure 11. Structural genes encoded next to effector genes share ancestry. **a**, Maximum-likelihood phylogenetic tree of *vgrG* genes of strain PAO1. The tree is inferred using the TIM+F+R3 model. The tree is unrooted and distances are shown in substitutions per site. **b**, Distance matrix indicating pairwise nucleotide similarity of *vgrG* genes of strain PAO1. **c**, Maximum-likelihood phylogenetic tree of *hcp* genes found in the genome of strain PAO1. The tree was inferred using the K3Pu+F model. The tree is unrooted and distances are shown in substitutions per site. **d**, Distance matrix indicating pairwise nucleotide similarities of *hcp* genes of strain PAO1.

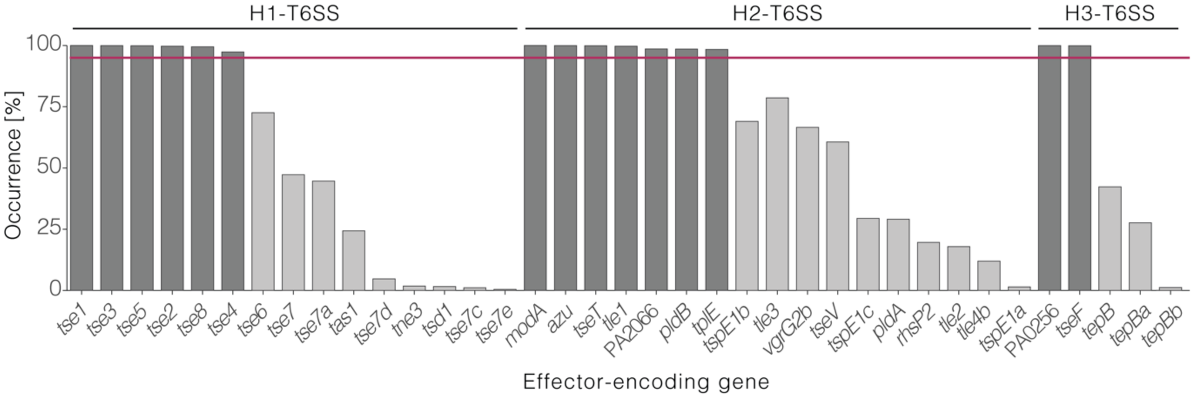

Supplementary Fig. 12. Core and accessory effector gene occurrence confirmed in complete genomes. Bar graph indicating the occurrence of the indicated effector genes among complete *P. aeruginosa* genomes (n=232). The horizontal lines indicate an occurrence of 95% and presence in the core genome.

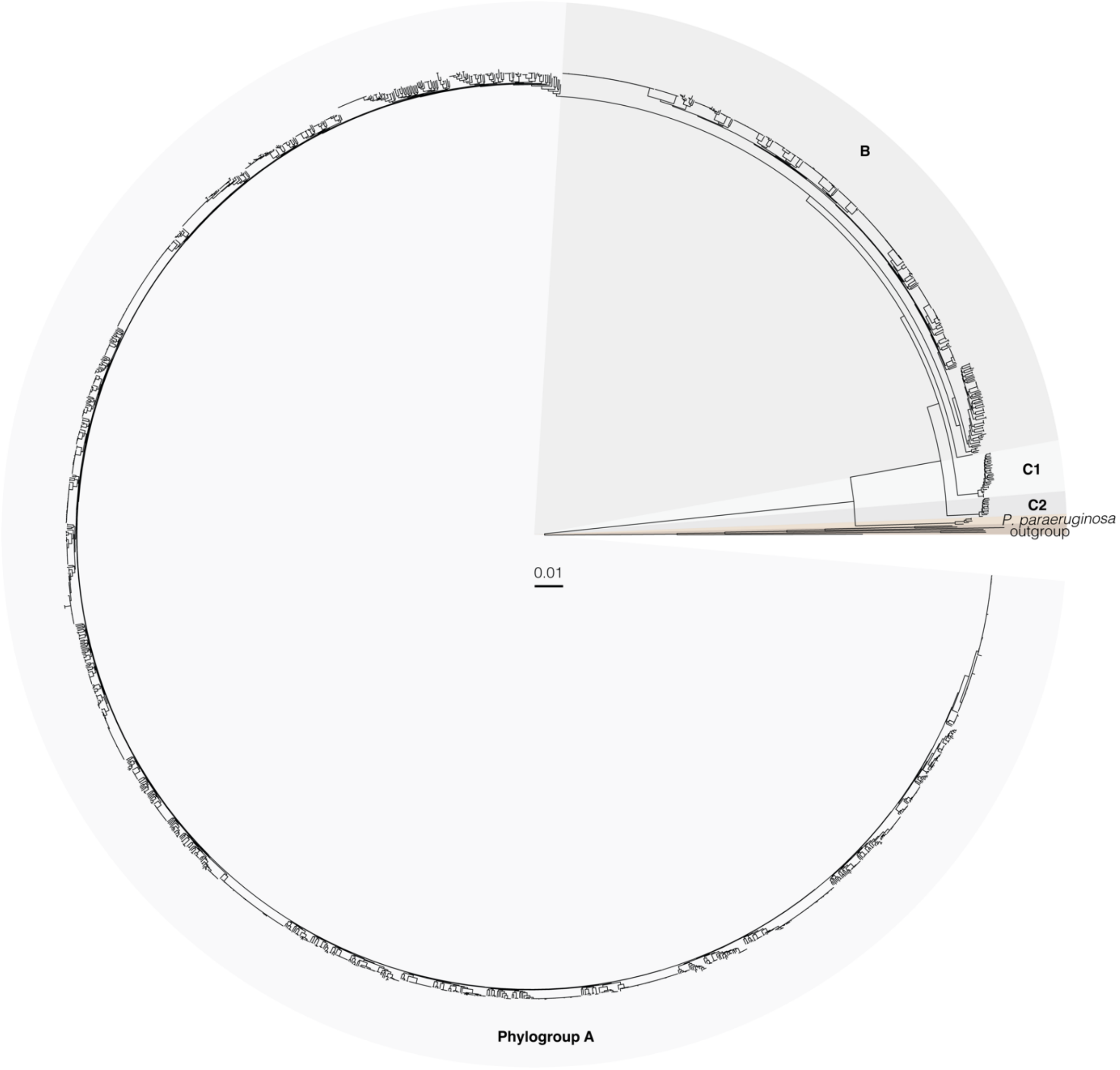

Supplementary Fig. 13. Phylogenetic tree used for the stochastic mapping. This tree includes *P. aeruginosa* genomes that encode an H1-, H2-, and H3-T6SS (n=1912), five genomes of the closely related species *P. paraeruginosa*, and seven genomes of the species *P. delhiensis, P. knackmussii, P. humi, P. jinjuensis, P. multiresinivorans, P. nitroreducens,* and *P. panipatensis* as an outgroup. The maximum-likelihood tree was inferred using the HKY+F+I model. It is rooted to the outgroup and distances are shown in substitutions per site. Accession codes for all genomes can be found in Supplementary Data 15.

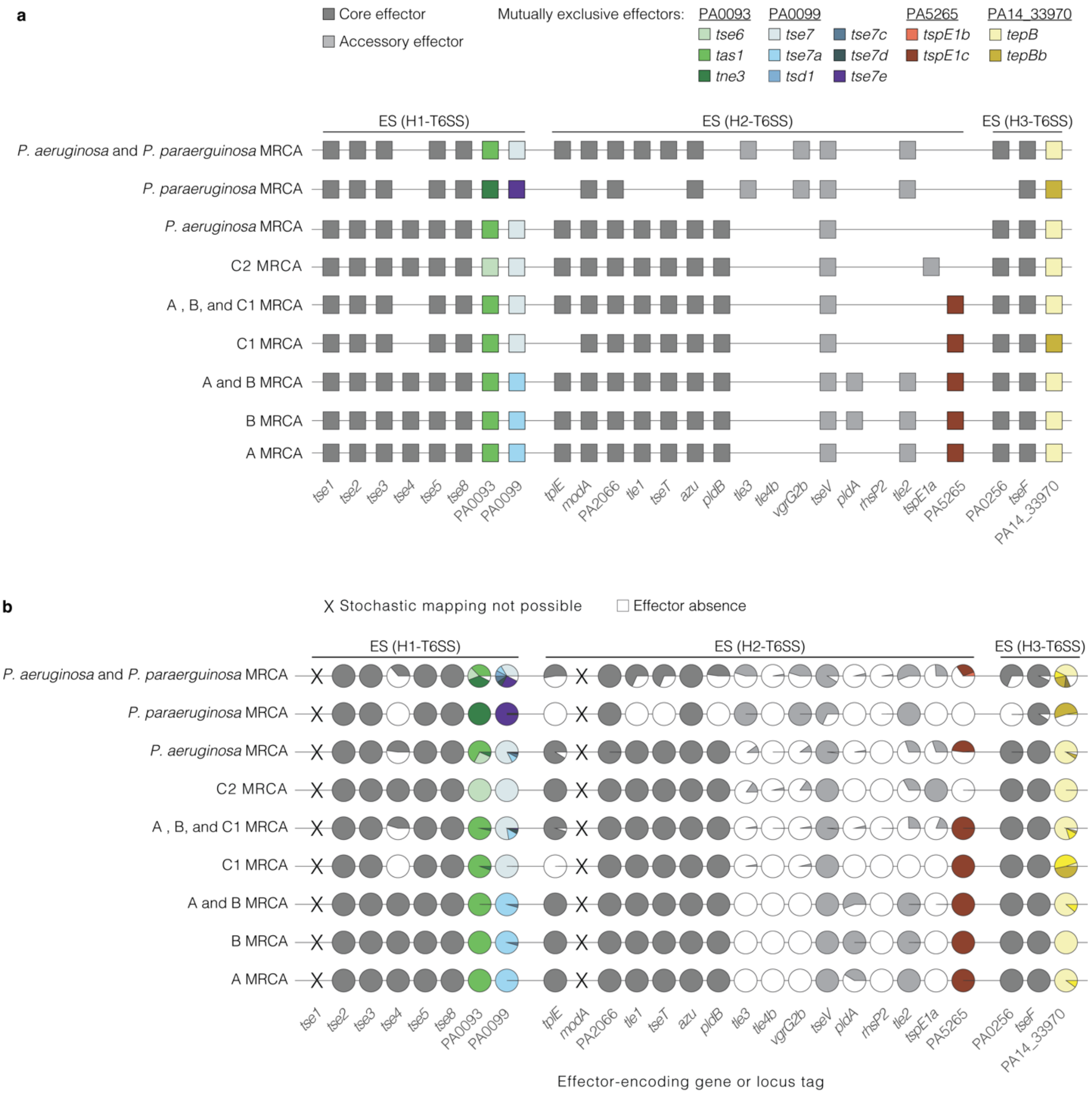

Supplementary Fig. 14. Reconstruction of the T6SS effector sets in the indicated most recent common ancestors (MRCAs). a, Graphical depiction summarizing the results of the stochastic mapping (raw data with probability values shown in panel **b**). **b,** Posterior probabilities from stochastic character mapping for ancestral nodes of effectors of indicated most recent common ancestors (MRCAs). Stochastic character mapping was not possible for two effectors (*tse1* and *modA*), because of an effector occurrence of 100% in the global population. Posterior probabilities were extracted from the respective nodes from Supplementary Data 17 and 23.

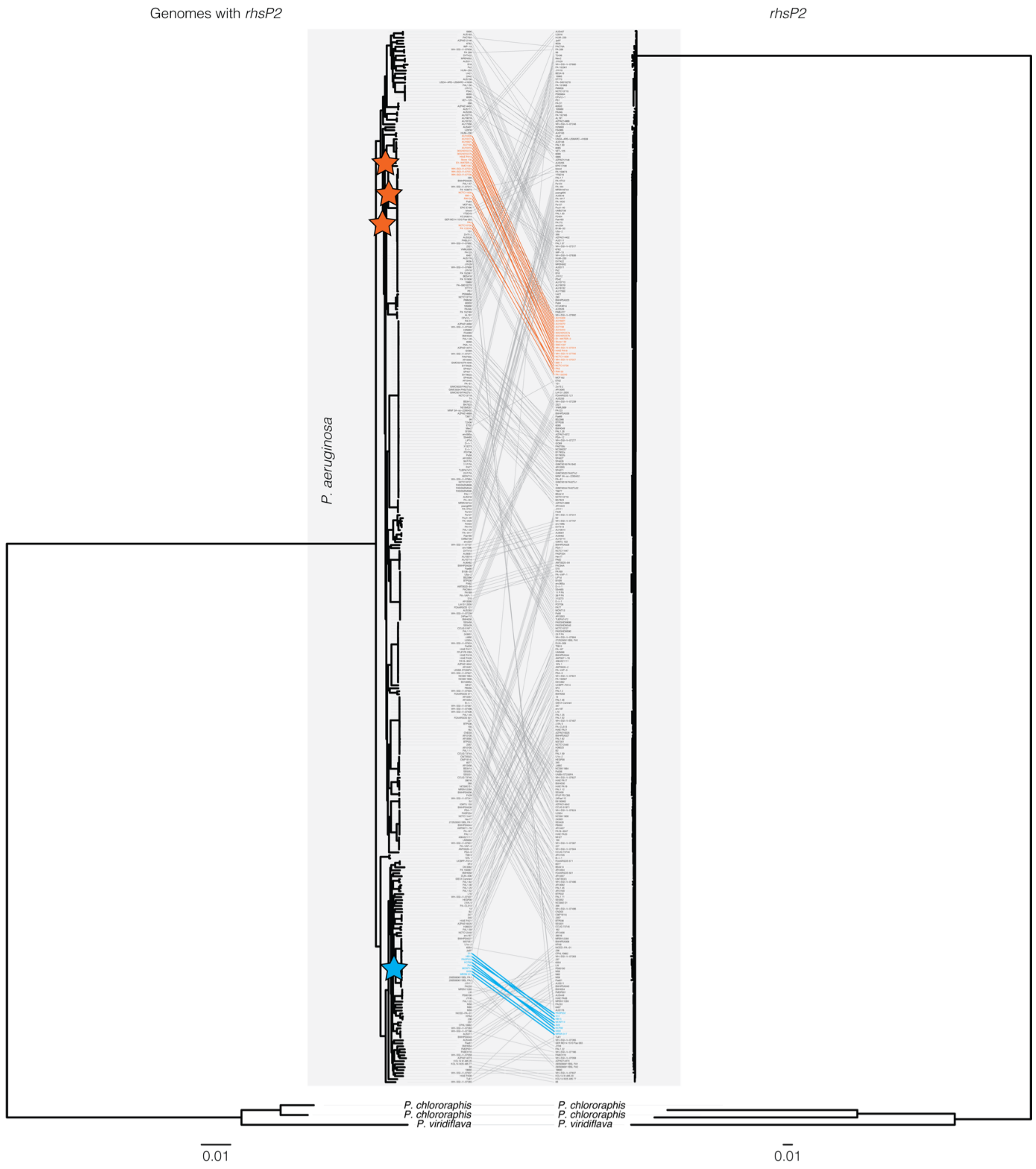

Supplementary Fig. 15. Extended figure to Figure 7b with same phylogenetic trees and branches not cut. Co-phylogenetic plot of *rhsP2* and strains harbouring *rhsP2*. The maximum-likelihood tree was calculated using the HKY+F+I model and is based on the core genome of *rhsP2+ P. aeruginosa* genomes and three strains of related species. The gene tree was calculated using the TPM2+F+R2 model and is based on a multiple sequence alignment of *rhsP2* from these strains. Both trees are midpoint rooted and distances are shown in substitutions per site. Coloured stars refer to gene gain events predicted by stochastic mapping. The coloured lines highlight examples that are shown in more detail in main figure 7.

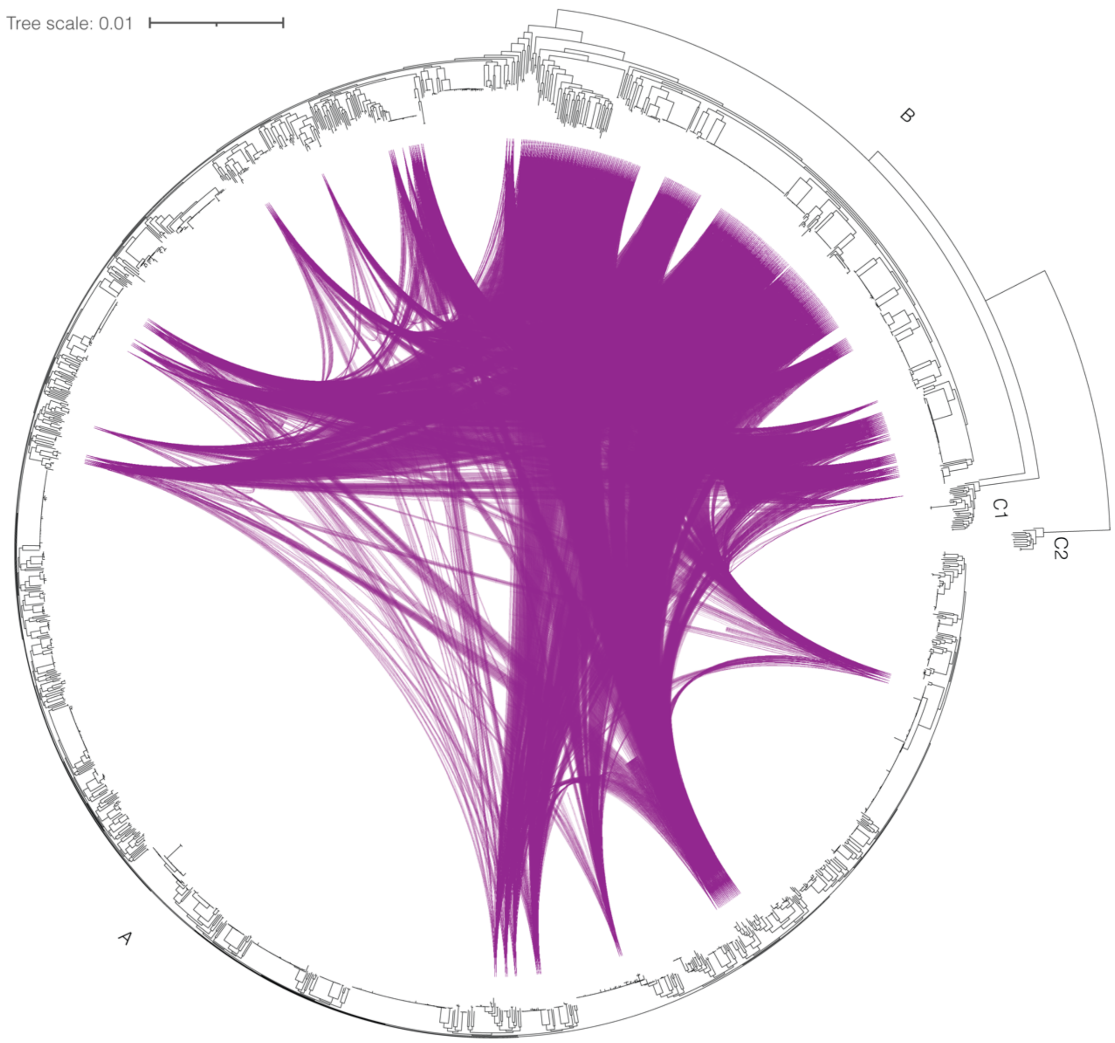

Supplementary Fig. 16. Circos plot indicating possibilities of *rhsP2* transfer between *P. aeruginosa* strains. Purple lines connect all strains that encode the effector gene *rhsP2*. The maximum-likelihood phylogenetic tree of 1912 *P.* a*eruginosa* strains is based on a core genome alignment. The tree was inferred using the HKY+F+I model and is midpoint rooted, the genetic distance is shown in substitutions per site.

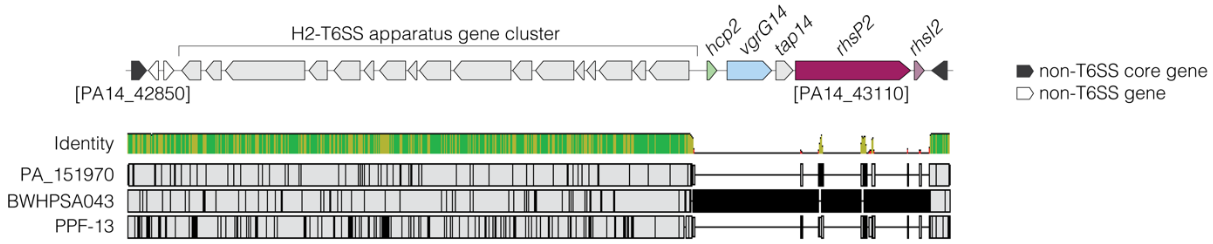

Supplementary Fig. 17. Nucleotide alignment of the genomic region between non-T6SS core genes equivalent to PA14_42850 and PA14_43110 in strain PA14 of indicated strains. If present, this genomic region includes the accessory effector *rhsP2*. Cartoon above indicates the genomic region.

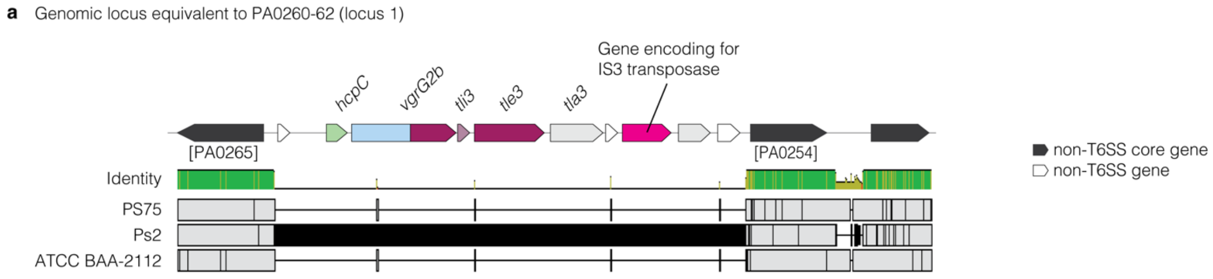

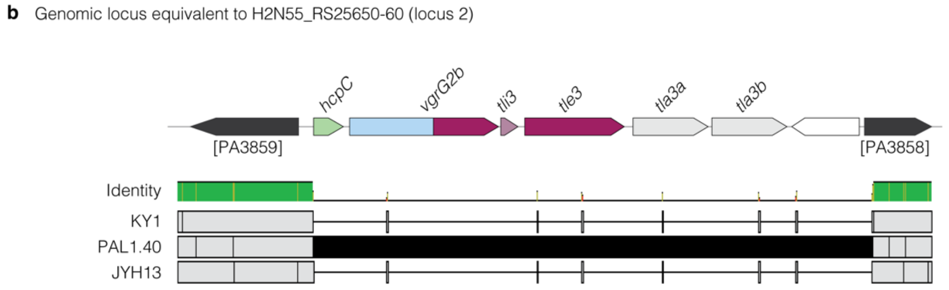

Supplementary Fig. 18. a, Nucleotide alignment of the genomic region between non-T6SS core genes equivalent to PA0265 and PA0254 in PAO1 of indicated strains. If present, this genomic region can include accessory effectors *vgrG2b* and *tle3*. Cartoon above indicates the respective genes. **b,** Nucleotide alignment of the genomic region between non-T6SS core genes equivalent to PA3859 and PA3859 in PAO1 of indicated strains. If present, this genomic region can include accessory effectors *vgrG2b* and *tle3*. Cartoon above indicates the respective genes.

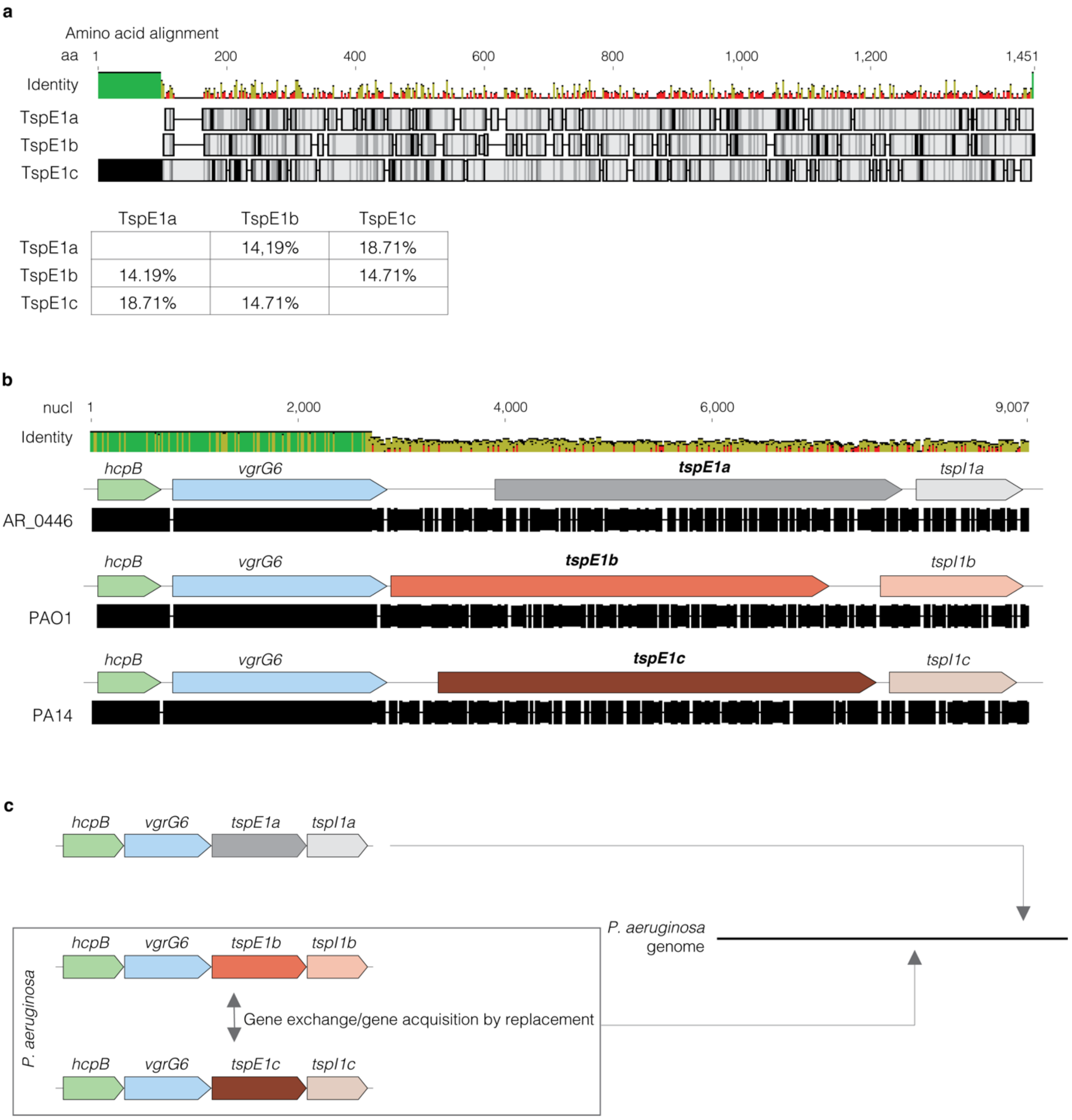

Supplementary Fig. 19. *tspE1* genes exchange by replacement. a, Amino acid alignment and distance matrix of TspE1a, TspE1b, and TspE1c showing variation. **b**, Nucleotide alignment of the genomic region including *tspE1a*, *tspE1b*, and *tspE1c* of indicated strains showing variation but conservation of *vgrG6*. **c**, Schematic of gene exchange between *tspE1b* and *tspE1c* at one genomic locus.

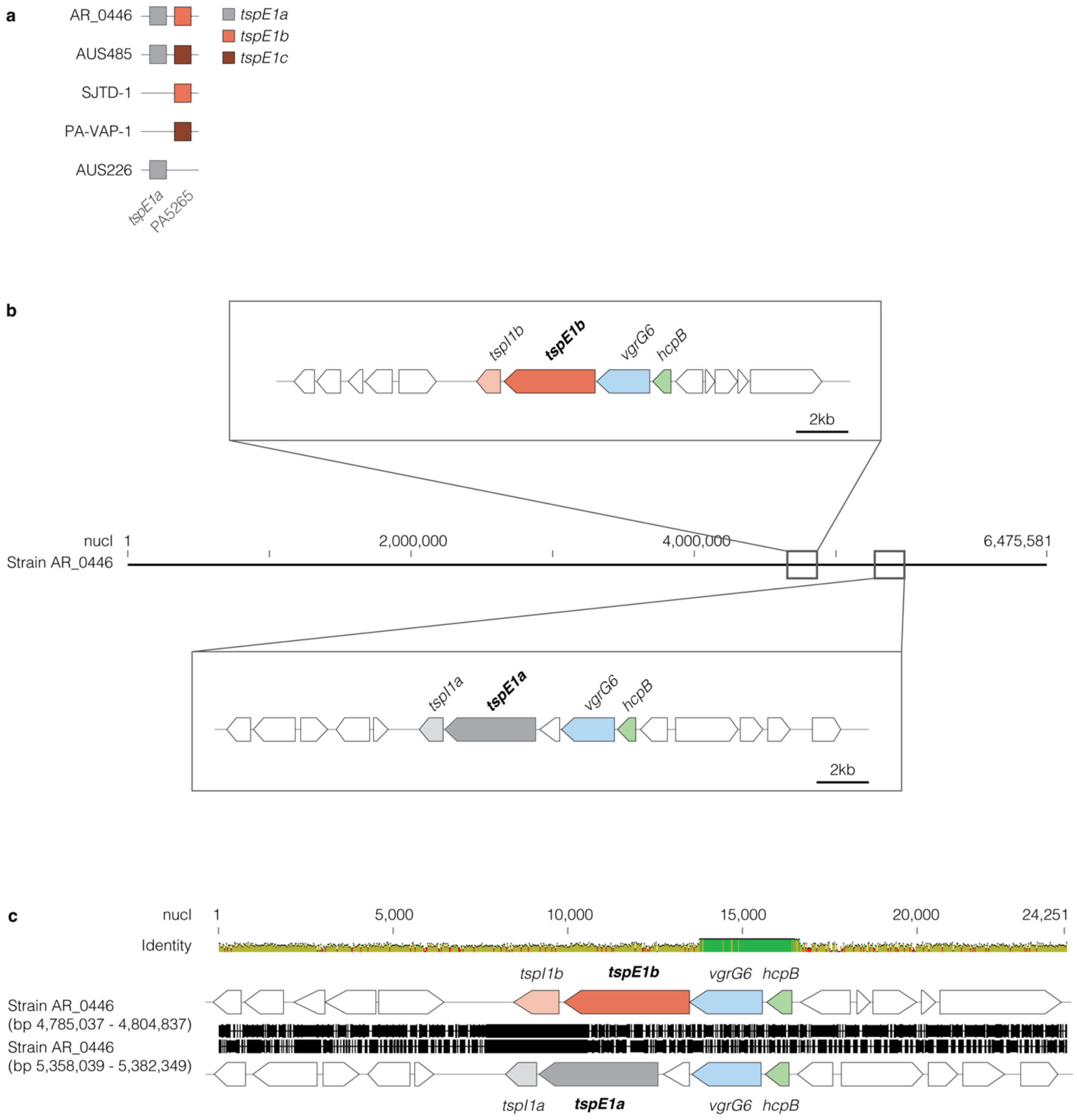

Supplementary Fig. 20. Location and comparison of *P. aeruginosa* genomic loci containing *tspE1* genes. a, Combinations of *tspE1* genes found in the dataset indicated by exemplary strains. **b**, Strains can encode two *tspE1* genes. Graphical depiction of *tspE1a* and *tspE1b* that are encoded at different positions in the genome of strain AR_0446. **c**, Nucleotide alignment of the genomic regions including *tspE1a* and *tspE1b* in strain AR_0446 demonstration variation between the two genes.

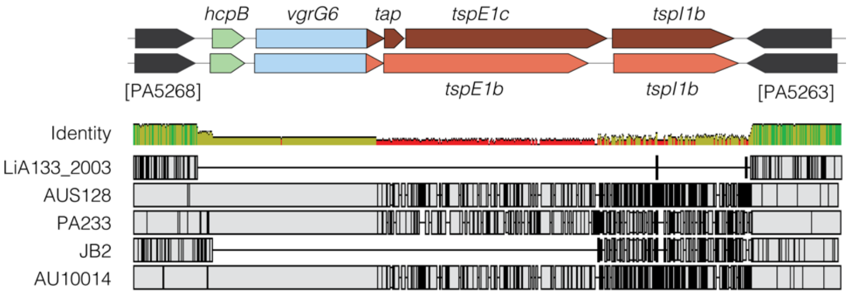

Supplementary Fig. 21. Nucleotide alignment of the genomic region between non-T6SS core genes equivalent to PA5268 and PA5263 in strain PAO1 of indicated strains. If present, this genomic region includes the accessory effector *tspE1b* and *tspE1c*. Cartoon above indicates the genomic region.

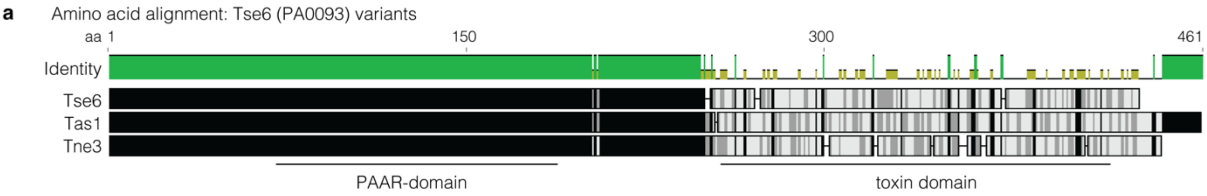

	Tse6-tox	Tas1-tox	Tne3-tox
Tse6-tox		9.58%	12.57%
Tas1-tox	9.58%		16.17%
Tne3-tox	12.57%	16.17%	

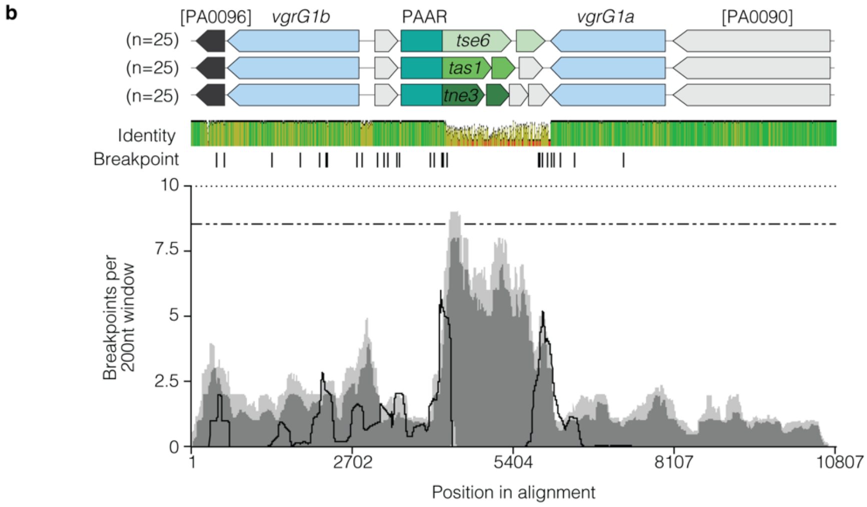

Supplementary Fig. 22. a, Amino acid alignment of mutually exclusive effectors encoded at the genomic locus equivalent to PA0093 in reference strain PAO1 and distance matrix indicating amino acid sequence similarities between the toxin domains as predicted by pfam and indicated by BLASTp. **b**, Graphical representation of the *vgrG1a* gene region with the three variant effectors encoding genes upstream. Lower panel shows evidence of altered recombination breakpoint distribution of the genomic region encoding the mutually exclusive effectors *tse6, tas1*, and *tne3*. The black line indicates the breakpoint number. Dark grey and light grey shaded areas indicate local 95% and 99% confidence intervals, respectively. Dashed lines indicate global 95% and 99% confidence intervals.

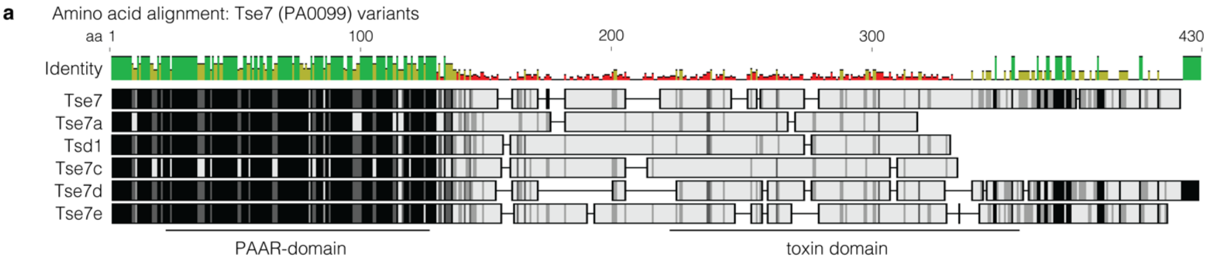

	Tse7-tox	Tse7a-tox	Tsd1-tox	Tse7c-tox	Tse7d-tox	Tse7e-tox
Tse7-tox		8.61%	13.10%	9.15%	28.57%	30.0%
Tse7a-tox	8.61%		13.46%	7.70%	7.28%	11.54%
Tsd1-tox	13.10%	13.46%		7.10%	5.95%	11.31%
Tse7c-tox	9.15%	7.70%	7.10%		6.75%	8.72%
Tse7d-tox	28.57%	7.28%	5.95%	6.75%		22.46%
Tse7e-tox	30.0%	11.54%	11.31%	8.72%	22.46%	

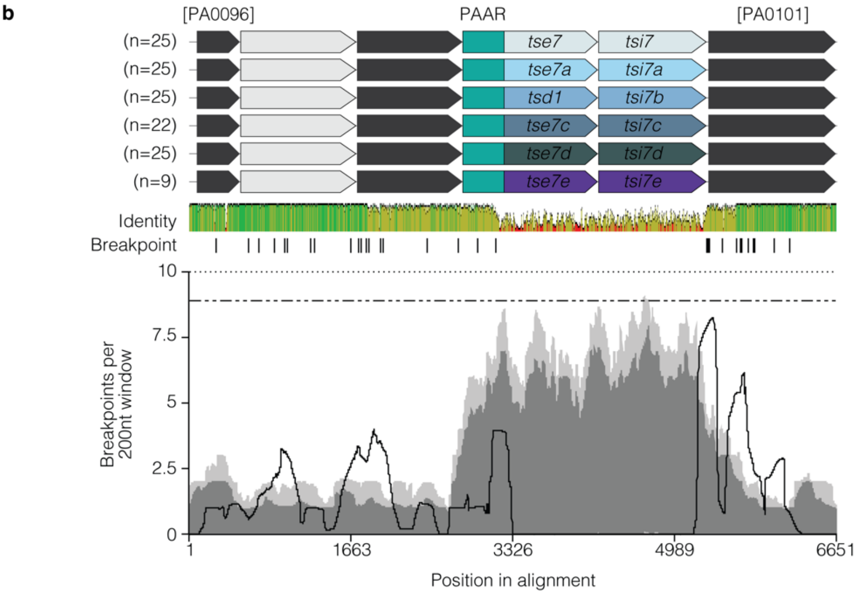

Supplementary Fig. 23. a, Amino acid alignment of mutually exclusive effectors encoded at the genomic locus equivalent to PA0099 in reference strain PAO1 and distance matrix indicating amino acid sequence similarities between the toxin domains as predicted by pfam and indicated by BLASTp. **b**, Graphical representation of the gene region equivalent to PA0099 in reference strains PAO1 with the six variant effectors encoding genes upstream. Lower panel shows evidence of altered recombination breakpoint distribution of the genomic region encoding the mutually exclusive effectors *tse7, tse7a, tsd1, tse7c, tse7d,* and *tse7e*. The black line indicates the breakpoint number. Dark grey and light grey shaded areas indicate local 95% and 99% confidence intervals, respectively. Cartoon above indicates the genomic region. Dashed lines indicate global 95% and 99% confidence intervals.

a

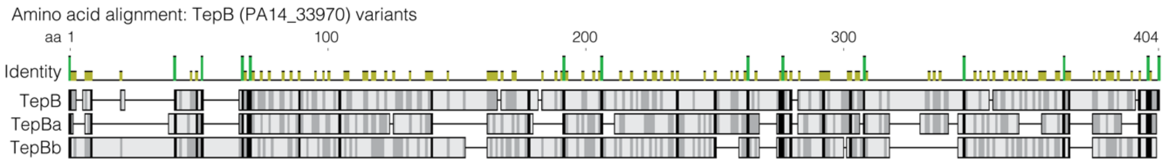

	TepB	TepBa	TepBb
TepB		12.78%	11.91%
TepBa	12.78%		11.26%
TepBb	11.91%	11.26%	

b

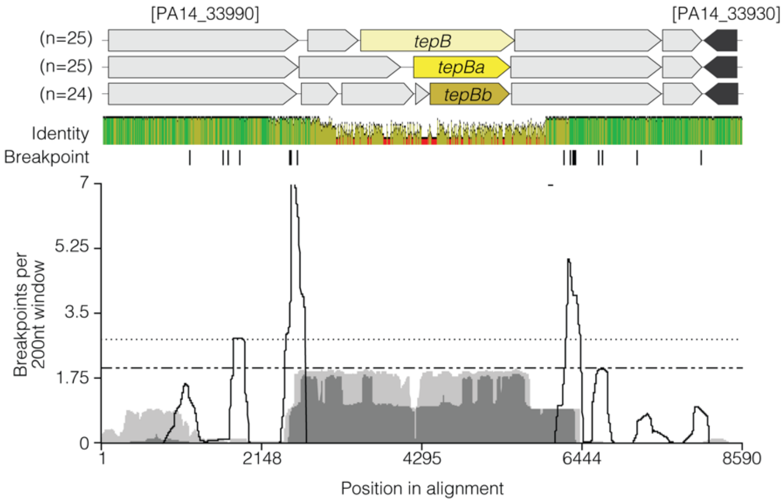

Supplementary Fig. 24. a, Amino acid alignment of mutually exclusive effectors encoded at the genomic locus equivalent to PA0099 in reference strain PAO1 and distance matrix indicating amino acid sequence similarities between the proteins. **b**, Graphical representation of the gene region equivalent to PA14_33970 in reference strain PA14 with the three variant effectors encoding genes upstream. Lower panel shows evidence of altered recombination breakpoint distribution of the genomic region encoding the mutually exclusive effectors *tepB*, *tepBa*, and *tepBb*. The black line indicates the breakpoint number. Dark grey and light grey shaded areas indicate local 95% and 99% confidence intervals, respectively. Cartoon above indicates the genomic region. Dashed lines indicate global 95% and 99% confidence intervals.

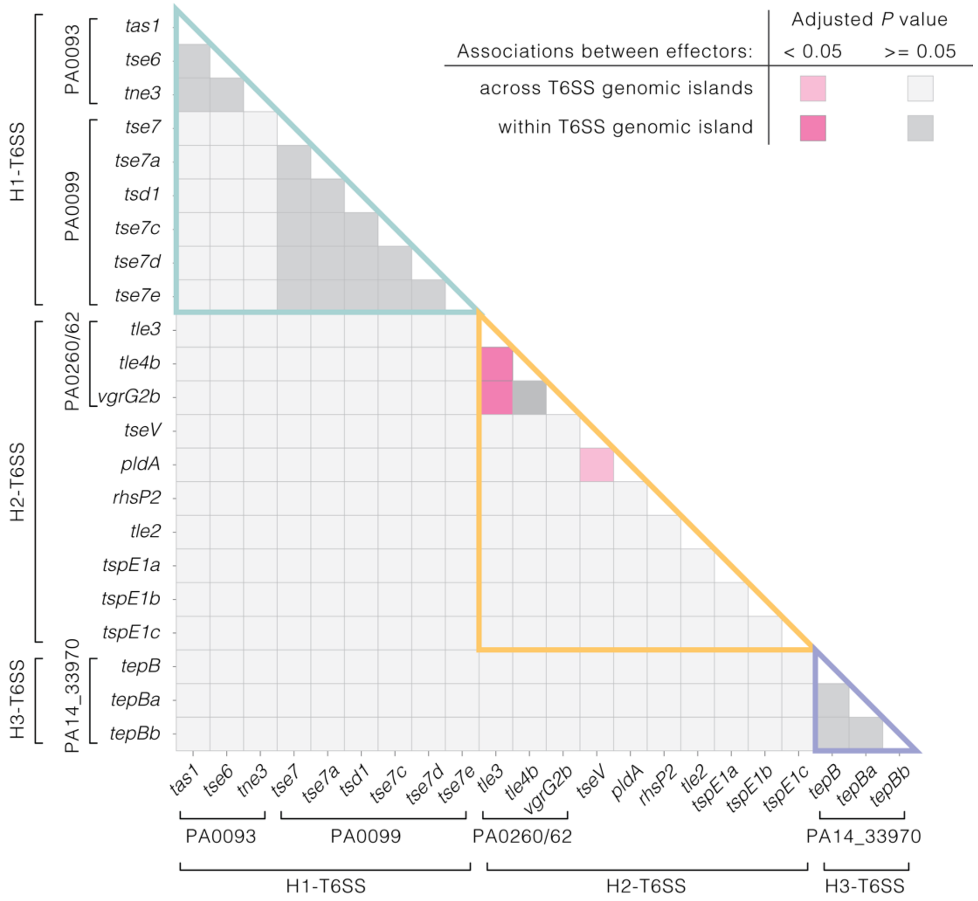

Supplementary Fig. 25. Co-occurrence between pairs of accessory effector genes. Colours indicate hypergeometric *P* values of phylogeny-corrected association study. *P* values were corrected for false-discovery rate (FDR = 0.05). Comparisons of effector pairs of the same T6SS are highlighted with triangles (green: H1-T6SS effectors, yellow: H2-T6SS effectors, purple: H3-T6SS effectors). Runs-adjusted hypergeometric *P* values as well as runs-adjusted Jaccard Coefficient and runs-adjusted Pearson Correlation coefficient are listed in Supplementary Data 25.

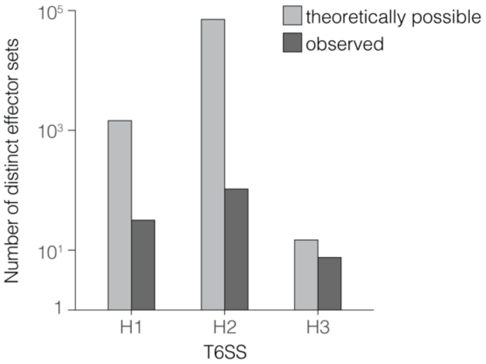

Supplementary Figure 26. Theoretically possible and observed effector sets. Bar graph indicating the theoretically possible and observed number of distinct effector sets for each T6SS.

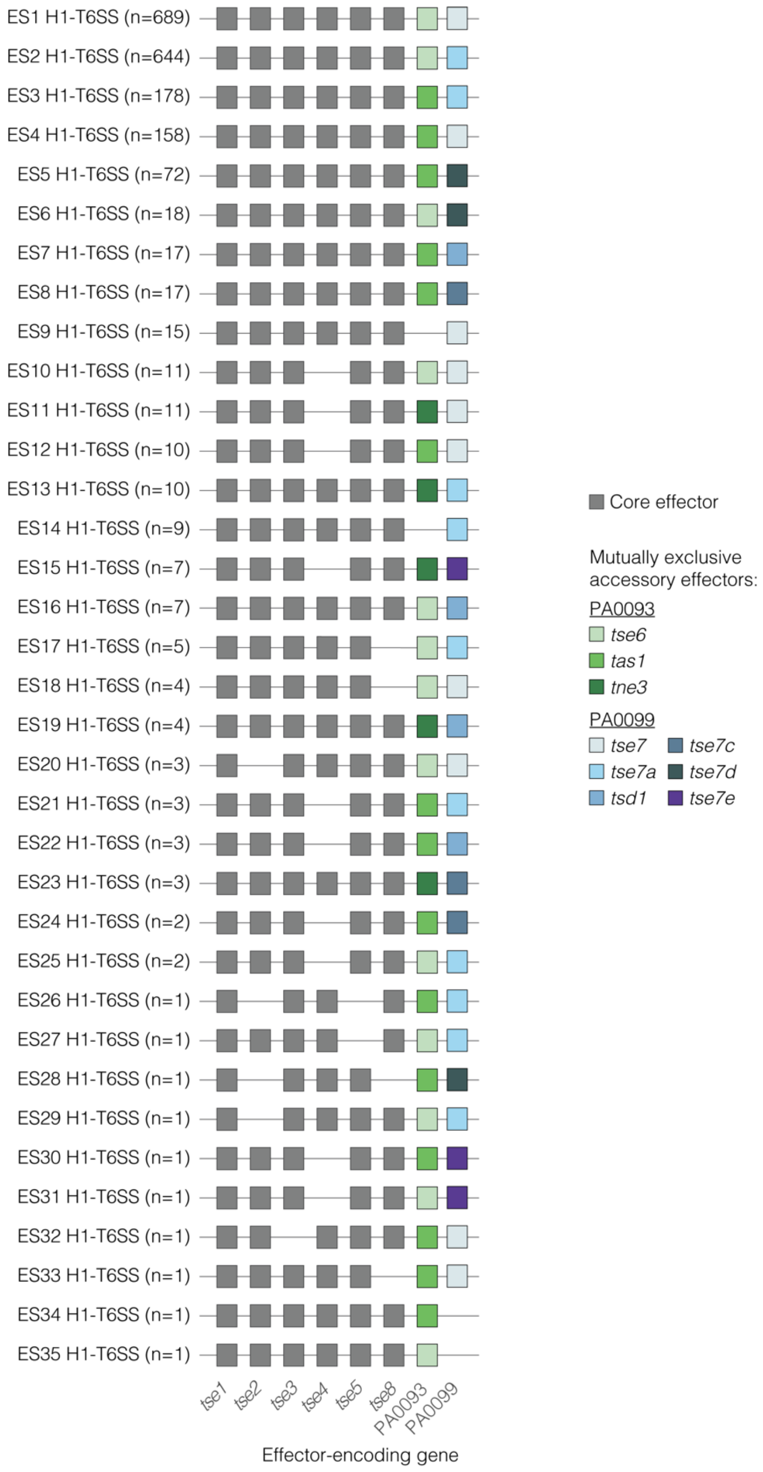

Supplementary Fig. 27. Overview of the H1-T6SS effector sets in the dataset of analysed genomes. Graphical depictions indicating the observed combinations of effectors of the H1-T6SS. The number of strains with a respective effector combination is indicated in brackets.

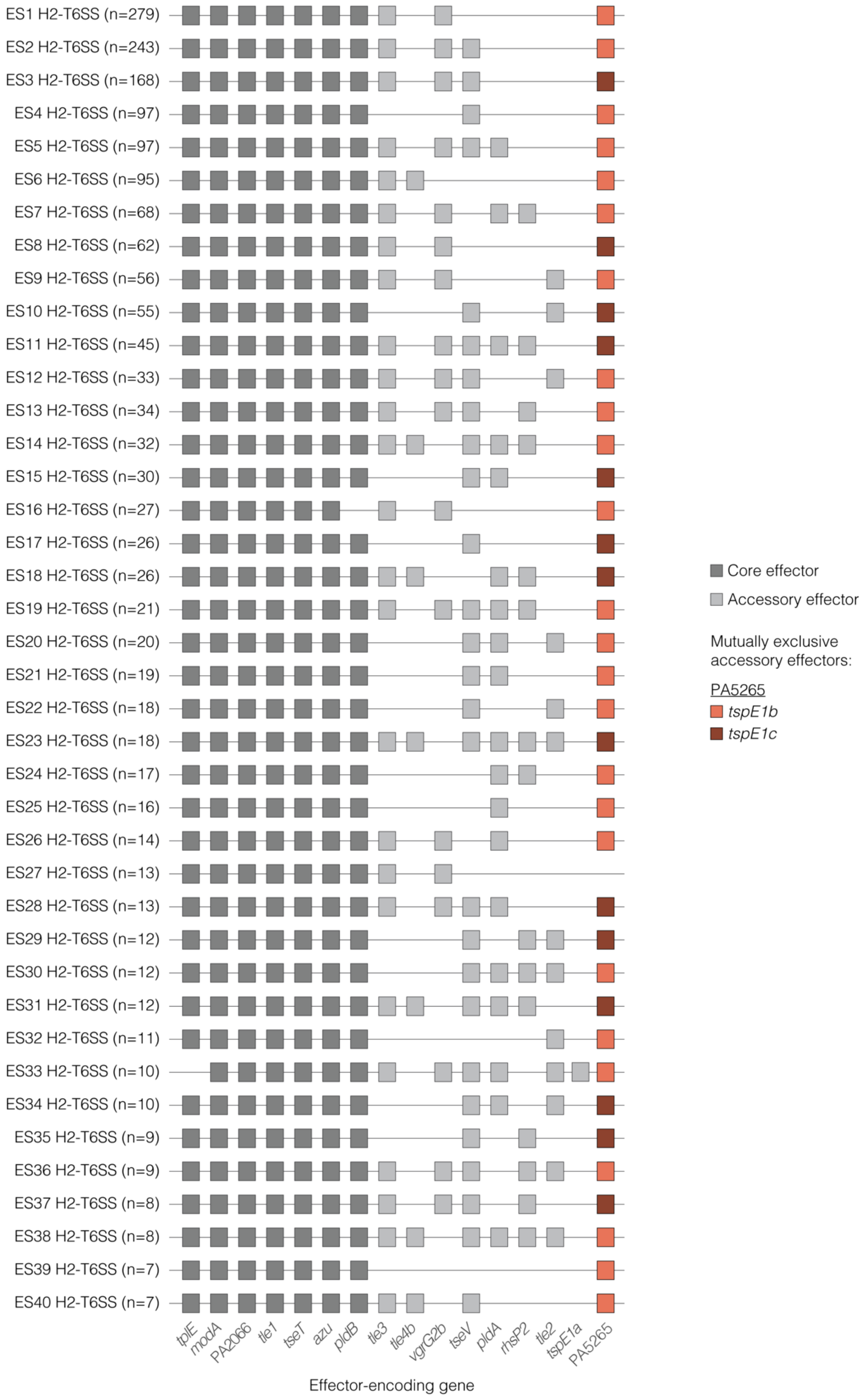

Supplementary Fig. 28. Overview of the H2-T6SS effector sets in the dataset of analysed genomes. Graphical depictions indicating the observed combinations of effectors of the H2-

T6SS. The number of strains with a respective effector combination is indicated in brackets. Continued in Supplementary Fig. 29.

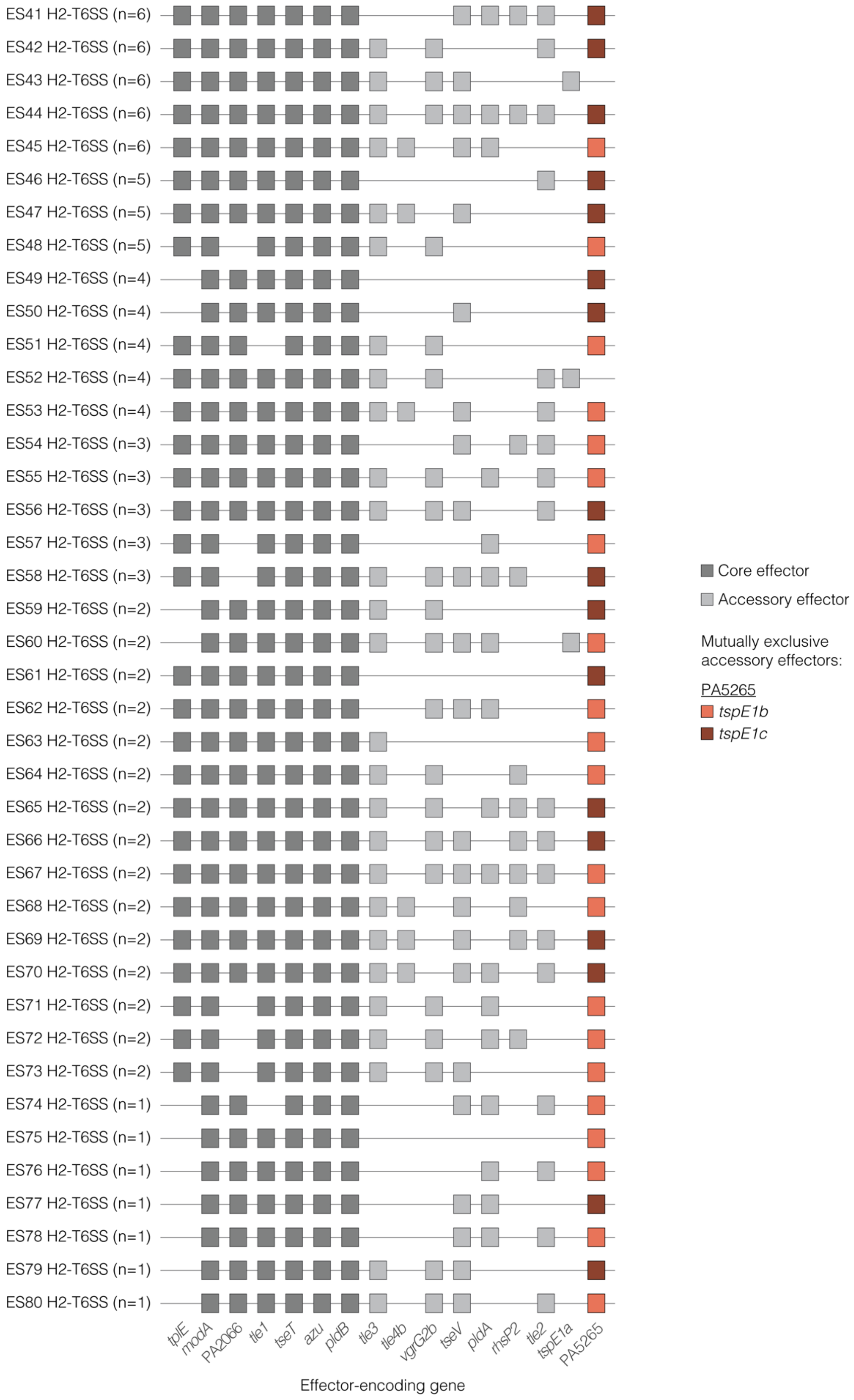

Supplementary Fig. 29. Continued from Supplementary Fig. 28. Graphical depictions indicating the observed combinations of effectors of the H2-T6SS. The number of strains

with a respective effector combination is indicated in brackets. Continued in Supplementary Fig. 30.

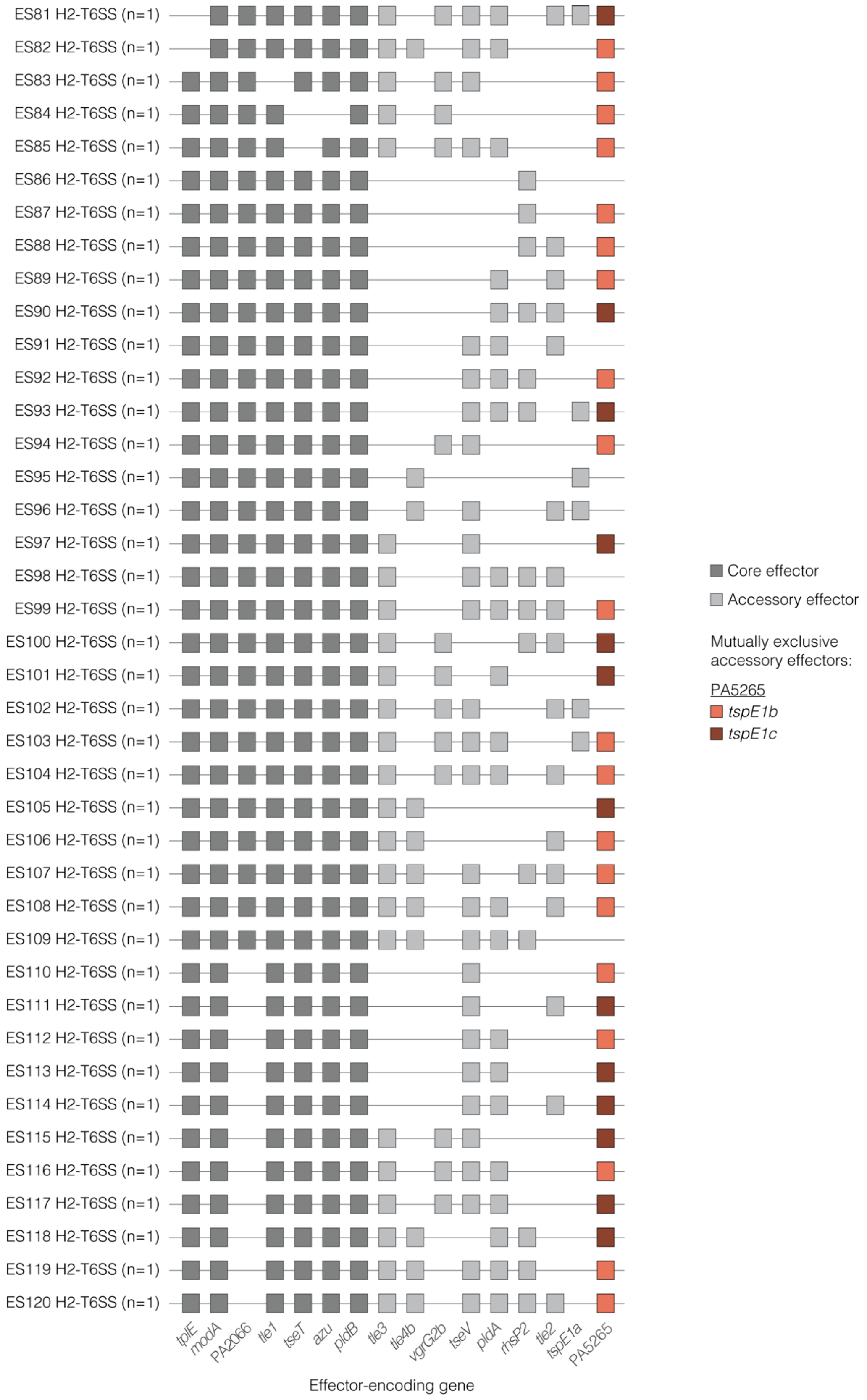

Supplementary Fig. 30. Continued from Supplementary Fig. 29. Graphical depictions indicating the observed combinations of effectors of the H2-T6SS. The number of strains with a respective effector combination is indicated in brackets.

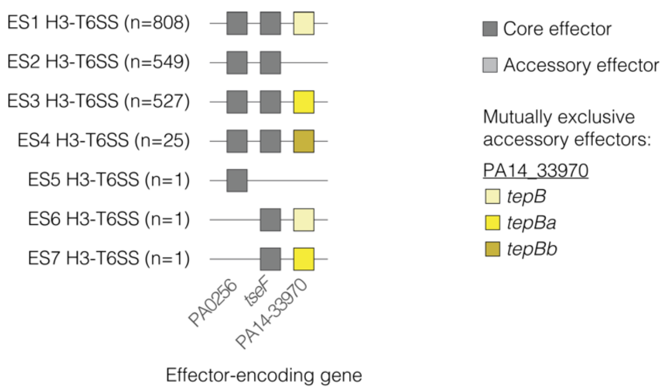

Supplementary Fig. 31. Overview of the H3-T6SS effector sets in the dataset of analysed genomes. Graphical depictions indicating the observed combinations of effectors of the H3-T6SS. The number of strains with a respective effector combination is indicated in brackets.

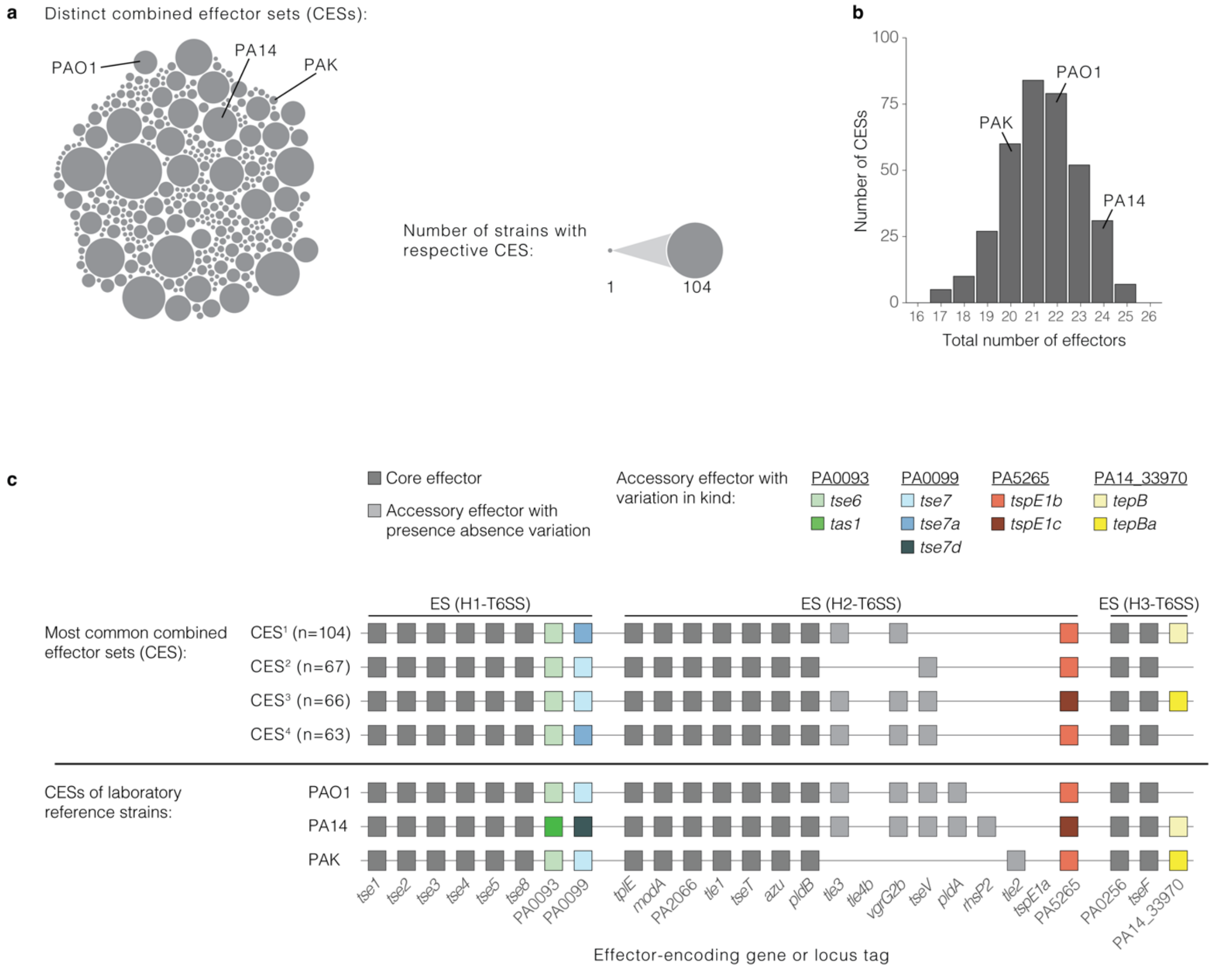

Supplementary Fig. 32. a, Bubble chart showing the large diversity of all distinct combined effector sets (CESs) within the dataset. Each bubble represents a combined effector set (referred to as the sum of H1-, H2-, and H3-T6SS effector genes in a genome). The size of the bubble is proportional to the number of strains. The most common CES is depicted. **b**, Frequency distribution of distinct combined effector sets (CESs) and the total number of effectors per combination. The names of the reference strains are indicated. **c**, Graphical depiction of the most common combined effector sets (CES) in the global *P. aeruginosa* population and effector combinations of laboratory reference strains PAO1, PA14, and PAK. The number of strains with a certain effector combination are indicated in brackets.

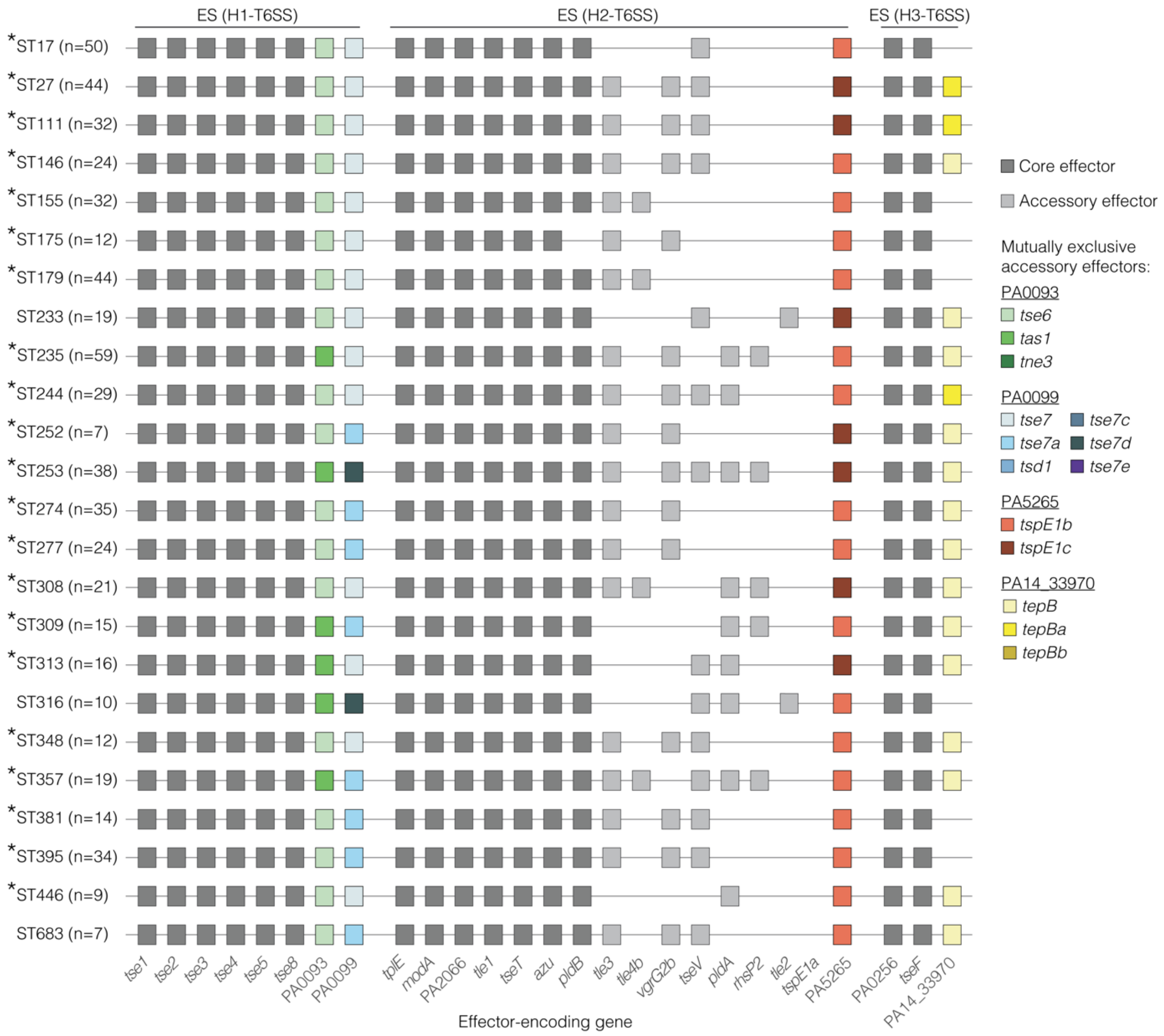

Supplementary Fig. 33. Graphical depictions of the most common effector sets of clinically relevant sequence types. Global epidemic clones recently identified by Weiman *et al.* (Weimann, A. *et al.* Evolution and host-specific adaptation of *Pseudomonas aeruginosa*. *Science (80-.).* **385**, (2024)) are indicated with a star (*). The number of strains of that sequence type with a respective effector set are indicated in brackets.

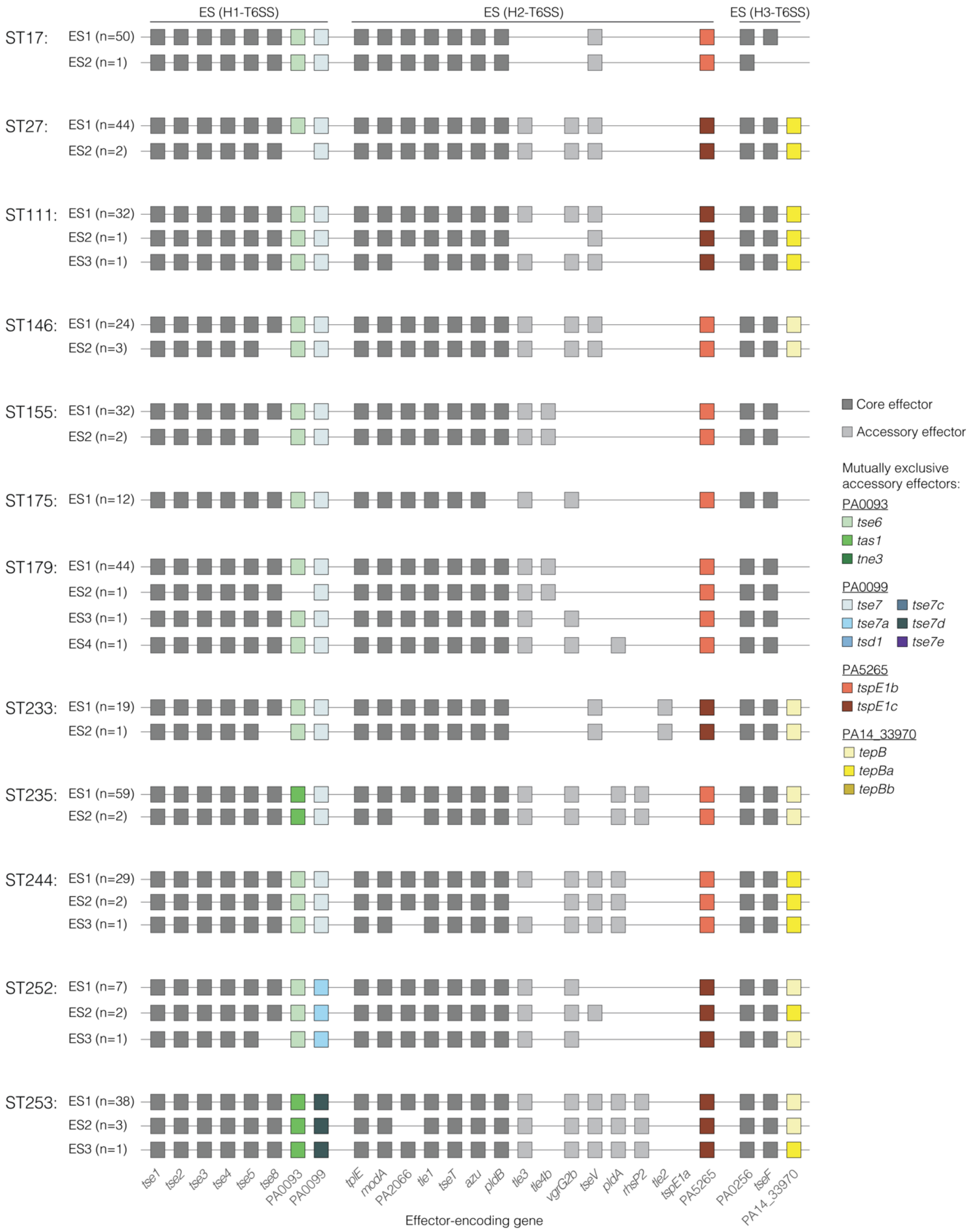

Supplementary Fig. 34. Graphical depiction of all effector sets of clinically relevant sequence types showing diversity between and within some strains from a single sequence type. The number of strains of a particular sequence type with the respective effector set is indicated in brackets. Continued in Supplementary Fig. 35.

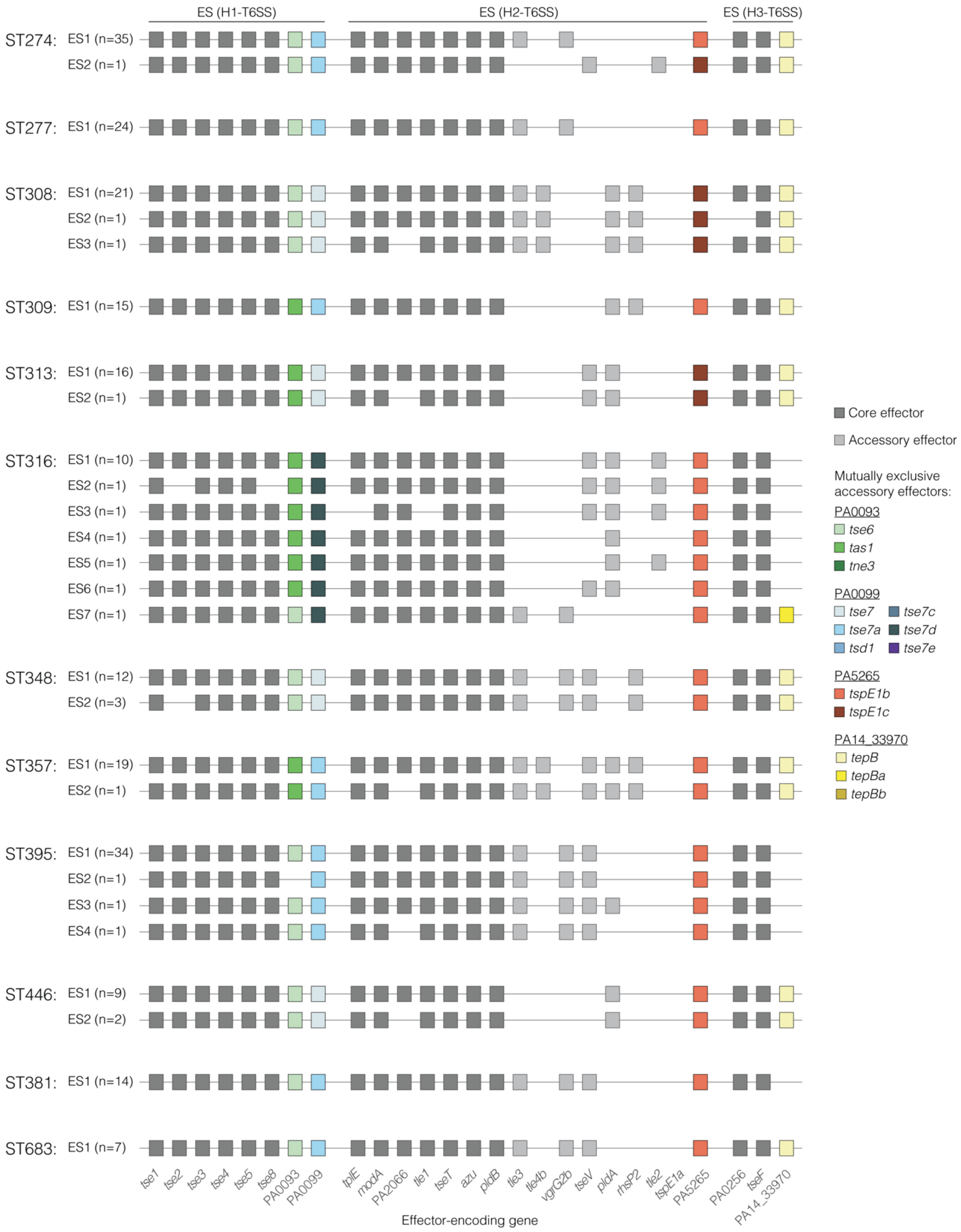

Supplementary Fig. 35. Continued from Supplementary Fig. 34. Graphical depiction of all effector sets of clinically relevant sequence types showing diversity between and within some strains from a single sequence type. The number of strains of a particular sequence type with the respective effector set is indicated in brackets.

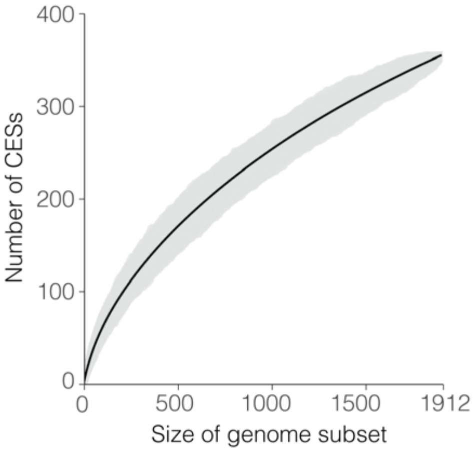

Supplementary Fig. 36. Rarefaction curve (black) of 10,000 iterations (grey) indicating that more unique CESs will be found with the addition of more genomes.

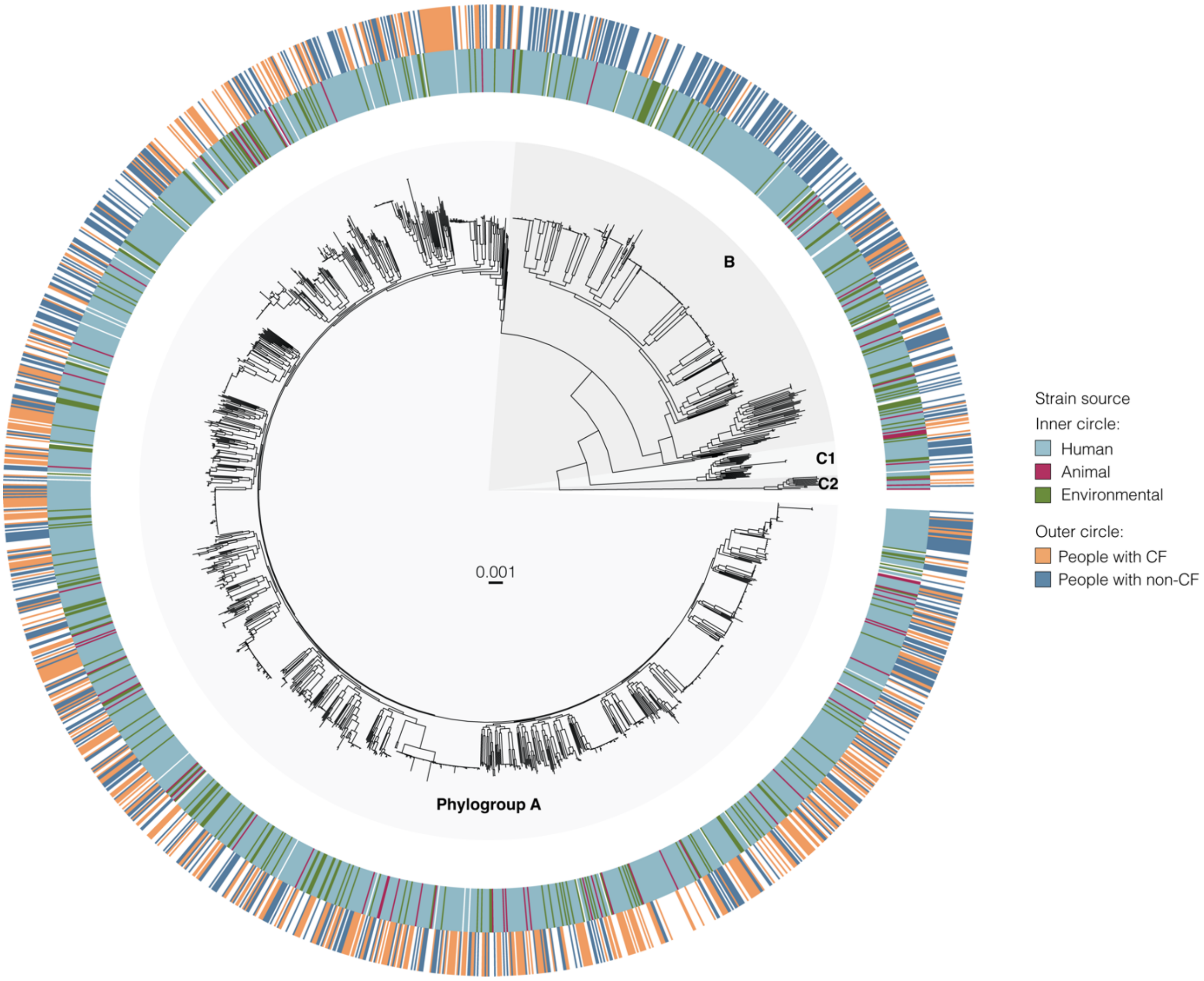

Supplementary Fig. 37. Phylogenetic relationship and the distribution of strain sources. The maximum-likelihood phylogenetic tree is based on a core genomic alignment of 1912 *P. aeruginosa* strains and computed using the HKY+F+I model. The tree is midpoint rooted. Distances are shown in substitutions per site.

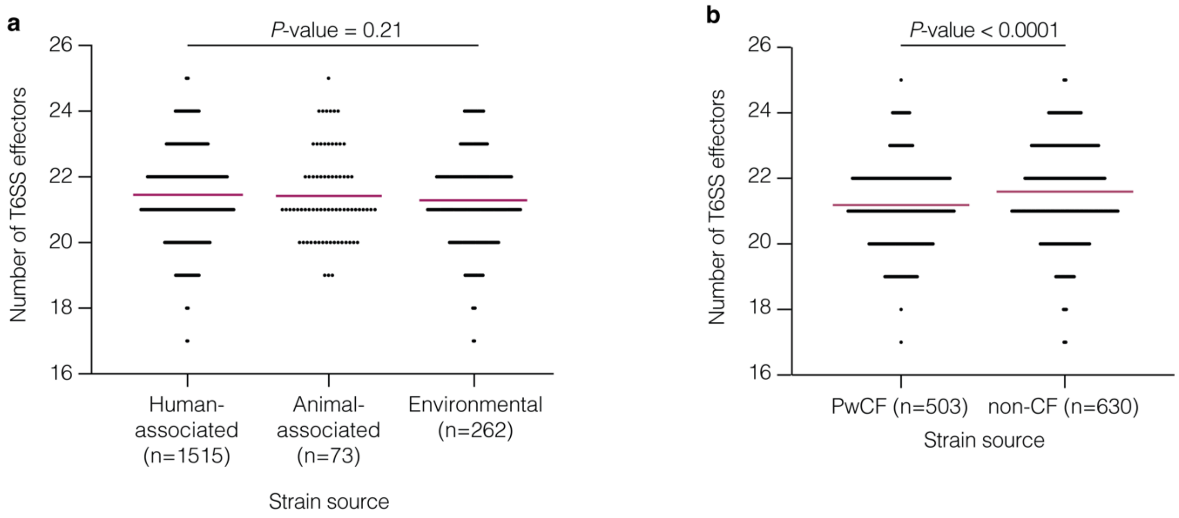

Supplementary Fig. 38. Dot plot indicating the number TSS effectors by source of isolation (**a**) and host disease (**b**). Each dot represents on strain. Red lines indicate the mean. Statistical significance was tested using the ordinary one-way ANOVA (degrees of freedom, 2; F, 1.539; R^2, 0.001664) (**a**) or two-tailed unpaired t-test (degrees of freed, 1131; t, 4.921; 95% confidence interval, 0.2442 to 0.5634) (**b**).

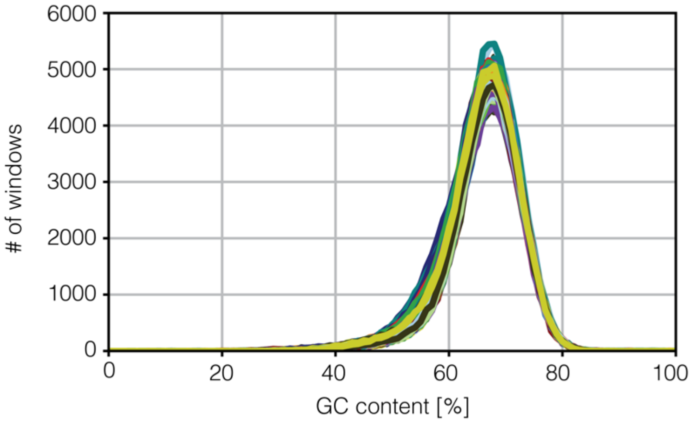

Supplementary Fig. 39. GC% content of the 1960 genomes from the dataset of *P. aeruginosa* strains. Average %GC content among all analysed genomes is 66.24%. Figure was generated by QUAST. Coloured lines indicate the different 1960 genomes. Accession codes and assembly statistics can be found in Supplementary Data 2 and 3.
