# Supplementary material for: Distribution of the four type VI secretion systems in Pseudomonas aeruginosa and classification of their core and accessory effectors
Source: Nat Commun. 2025 Jan 21;16:888. doi: 10.1038/s41467-024-54649-5 (PMC11751169; doi:10.1038/s41467-024-54649-5)
Supplement: Supplementary file 2 — Description of Additional Supplementary Files [file 41467_2024_54649_MOESM2_ESM.pdf]

Description of Additional Supplementary Files:

Supplementary Data 1: T6SS effectors known in *Pseudomonas aeruginosa*.

Supplementary Data 2: *P. aeruginosa* strains used in this study.

Supplementary Data 3: Summary of assembly statistics and pangenome of *P. aeruginosa* genomes used in this study.

Supplementary Data 4: Overview of the dataset of *P. aeruginosa* genomes.

Supplementary Data 5: Macromolecular structure and regulation of T6SSs in *P. aeruginosa*.

Supplementary Data 6: Occurrence of T6SS apparatus gene clusters.

Supplementary Data 7: Results of model comparison from ancestral reconstruction of the four T6SSs.

Supplementary Data 8: This file contains the results of the stochastic mapping of the T6SS apparatus gene clusters. Each page shows the results of one T6SS apparatus gene cluster. The respective T6SS apparatus gene cluster is indicated at the top left corner of the page. The posterior probabilities of the stochastic mapping are indicated on each node of the phylogenetic tree. The tree is based on a core genome alignment of *P. aeruginosa* (n=1960) and *P. paraeruginosa* (n=5). Seven strains of the species *P. delhiensis*, *P. knackmussii*, *P. humi*, *P. jinjuensis*, *P. multiresinivorans*, *P. nitroreducens*, and *P. panipatensis* were used as an outgroup (not shown in the tree). The tree is based on the HKY+F+I model, rooted to the outgroup and distances are shown in substitutions per site.

Supplementary Data 9: Results of amino acid blast of *tssB* genes.

Supplementary Data 10: Effector sets of *P. aeruginosa* strains.

Supplementary Data 11: Mean pairwise nucleotide identities resulting from the multiple sequence alignment of core effectors.

Supplementary Data 12: Mean pairwise nucleotide identities resulting from the multiple sequence alignment of accessory effectors.

Supplementary Data 13: Genome accession and effector sets of *Pseudomonas paraeruginosa* strains.

Supplementary Data 14: Accession codes of outgroup.

Supplementary Data 15: Accession codes of all genomes that were used for the phylogenetic tree as a basis for the stochastic mapping of effector genes.

Supplementary Data 16: Results of model comparison from stochastic mapping of effector genes.

Supplementary Data 17: To view this file, open in Preview (MacOS) or Microsoft Edge (Windows). This file contains the results of the stochastic mapping of the effector genes. Each page shows the results of one effector gene. The respective effector gene is indicated at the top left corner of the page. Effectors are ordered by their associated T6SS and are found in the following order: H1-T6SS effectors: *tas1, tse2, tse3, tse4, tse5, tse7, tse8, tne3, tse6, tse7a, tsd1, tse7c, tse7d, tse7e*; H2-T6SS effectors: *azu*, PA2066, *pldB, tle2, tplE, tseT, tseV, pldA, rhsP2, tle2, tle3, tle4b, tspE1a, tspE1b, tspE1c, vgrG2b*; H3-T6SS effectors: PA0256, *tepB, tseF, tepBa, tepBb*. The posterior probabilities of the stochastic mapping are indicated on each node of the phylogenetic tree. The tree is based on a core genome alignment of *P. aeruginosa* (n=1912) and *P. paraeruginosa* (n=5). Seven strains of the species *P. delhiensis*, *P. knackmussii*, *P. humi*, *P. jinjuensis*, *P. multiresinivorans*, *P. nitroreducens*, and *P. panipatensis* were used as an outgroup (not shown in the tree). The tree is based on the HKY+F+I model, rooted to the outgroup, and distances are shown in substitutions per site.

Supplementary Data 18: Results of nucleotide blast of effector genes.

Supplementary Data 19: Results of protein blast of effector proteins.

Supplementary Data 20: This file contains the cophylogenetic plots of accessory effector genes and bacterial strains harbouring these genes. Effectors are ordered by their associated T6SS and are found in the following order: H1-T6SS effectors: *tse6, tas1, tne3, tse7, tse7a, tsd1, tse7c, tse7d, tse7e*; H2-T6SS effectors: *tle3, tle4b, vgrG2b, tseV, pldA, tle2, tspE1a, tspE1b, tspE1c*; H3-T6SS effectors: *tepB, tepBa, tepBb*. Please note that three pages are devoted to each effector: one title page and two pages that show sequential pairs of figures with the same trees with the branches at full length to demonstrate the genetic distance (substitutions per

site) from the closest potential source of the gene, or with cut branches for ease of visually looking at the detailed branches of *P. aeruginosa* strains.

Supplementary Data 21: This file contains circos plots of accessory effectors. The plots are ordered by the effector genes' associated T6SS and are presented in the following order: H1-T6SS effectors: *tse6, tas1, tne3, tse7, tse7a, tsd1, tse7c, tse7d, tse7e*; H2-T6SS effectors: *tle3, tle4b, vgrG2b, tseV, pldA, tle2, tspE1a, tspE1b, tspE1c*; H3-T6SS effectors: *tepB, tepBa, tepBb*. The effector gene is indicated on the top left corner of each page. Purple lines connect strains that have a particular effector gene. Data on the occurrence of effector genes was used as input (Supplementary Data 10). Mean pairwise nucleotide identities between effector genes that are connected with purple lines are provided in Supplementary Table 10. The phylogenetic tree is based on a core genome alignment of *P. aeruginosa* strains (n=1912) and computed using the HKY+F+I model. The tree is midpoint rooted and distances are shown in substitutions per site.

Supplementary Data 22: To view this file, open in Preview (MacOS) or Microsoft Edge (Windows). This file contains the results of the stochastic mapping of *tle3* and *vgrG2b-C-ter* at the two different loci. The respective effector gene and the locus are indicated at the top left corner of the page. The posterior probabilities of the stochastic mapping are indicated on each node of the phylogenetic tree. The tree is based on a core genome alignment of *P. aeruginosa* (n=1912) and P. *paraeruginosa* (n=5). Seven strains of the species *P. delhiensis*, *P. knackmussii*, *P. humi*, *P. jinjuensis*, *P. multiresinivorans*, *P. nitroreducens*, and *P. panipatensis* were used as an outgroup (not shown in the tree). The tree is based on the HKY+F+I model, rooted to the outgroup and distances are shown in substitutions per site.

Supplementary Data 23: To view this file, open in Preview (MacOS) or Microsoft Edge (Windows). This file contains the results of the stochastic mapping of loci with mutually exclusive effectors. The respective effector genes and the locus are indicated at the top left corner of the page. The posterior probabilities of the stochastic mapping are indicated on each node of the phylogenetic tree. The tree is based on a core genome alignment of *P. aeruginosa* (n=1912) and *P. paraeruginosa* (n=5). Seven strains of the species *P. delhiensis*, *P. knackmussii*, *P. humi*, *P. jinjuensis*, *P. multiresinivorans*, *P. nitroreducens*, and *P. panipatensis* were used as an outgroup (not shown in the tree). The tree is based on the HKY+F+I model, rooted to the outgroup and distances are shown in substitutions per site.

Supplementary Data 24: Tse7 variants in this paper and in Pissaridou and Allsopp *et al.*, and Robinson *et al.*

Supplementary Data 25: Results from the cooccurrence analysis indicating the similarity between the phylogenetic profiles of two effector genes. Listed are the runs-adjusted Jaccard Coefficient (rJC), the runs-adjusted Pearson Correlation coefficient (rPC), and the runs-adjusted hypergeometric P value (rHyperP) as computed using the PhyloCorrelate R package.

FDR (0.05) corrected P values were calculated with the p.adjust(method = 'hochberg') function in R.

Supplementary Data 26: T6SS effectors and their characteristics as used for Figures 9 and 10a-d.

Supplementary Data 27: Results from the cooccurrence analysis indicating the similarity between two phylogenetic profiles (effector presence and indicated isolation source (human, animal, or environment)). Listed are the runs-adjusted Jaccard Coefficient (rJC), the runs-adjusted Pearson Correlation coefficient (rPC), and the runs-adjusted hypergeometric P value (rHyperP) as computed using the PhyloCorrelate R package. FDR (0.05) corrected P values were calculated with the p.adjust(method = 'hochberg') function in R.

Supplementary Data 28: Results from the cooccurrence analysis indicating the similarity between two phylogenetic profiles (effector presence and indicated isolation source (PwCF or non-CF)). Listed are the runs-adjusted Jaccard Coefficient (rJC), the runs-adjusted Pearson Correlation coefficient (rPC), and the runs-adjusted hypergeometric P value (rHyperP) as computed using the PhyloCorrelate R package. FDR (0.05) corrected P values were calculated with the p.adjust(method = 'hochberg') function in R.

Supplementary Data 29: Gene presence absence matrix of *P. aeruginosa* strains (n=1960).

Supplementary Data 30: Marker genes (n=4478) used for phylogenetic analysis of the global *P. aeruginosa* population (n=1960).

Supplementary Data 31: Marker genes (n=4566) used for phylogenetic analysis of strains with an H1-, H2-, and H3-T6SS (n=1912).

Supplementary Data 32: Marker genes (n=4271) used for phylogenetic analysis of *P. aeruginosa* strains with an H1-, H2-, and H3-T6SS (n=1912), *Pseudomonas paraeruginosa* strains (n=5) and strains of other species (n=7).

Supplementary Data 33: Number of times a model was chosen to calculate a phylogenetic tree (see details in methods section 'Phylogenetic analysis of genomes').
