# Supplementary material for: Distribution of the four type VI secretion systems in Pseudomonas aeruginosa and classification of their core and accessory effectors
Source: Nat Commun. 2025 Jan 21;16:888. doi: 10.1038/s41467-024-54649-5 (PMC11751169; doi:10.1038/s41467-024-54649-5)
Supplement: Supplementary file 22 — Supplementary Dataset 20 [file 41467_2024_54649_MOESM22_ESM.pdf]

Distribution of the four type VI secretion systems in *Pseudomonas aeruginosa* and classification of their core and accessory effectors

Supplementary Data 20

This file contains the co-phylogenetic plots of accessory effector genes and bacterial strains harbouring these genes. Effectors are ordered by their associated T6SS and are found in the following order:

H1-T6SS effectors: *tse6, tas1, tne3, tse7, tse7a, tsd1, tse7c, tse7d, tse7e*

H2-T6SS effectors: *tle3, tle4b, vgrG2b, tseV, pldA, tle2, tspE1a, tspE1b, tspE1c*

H3-T6SS effectors: *tepB, tepBa, tepBb*

Please note that three pages are devoted to each effector: one title page and two pages that show sequential pairs of figures with the same trees with the branches at full length to demonstrate the genetic distance (substitutions per site) from the closest potential source of the gene, or with cut branches for ease of visually looking at the detailed branches of *P. aeruginosa* strains.

tse6

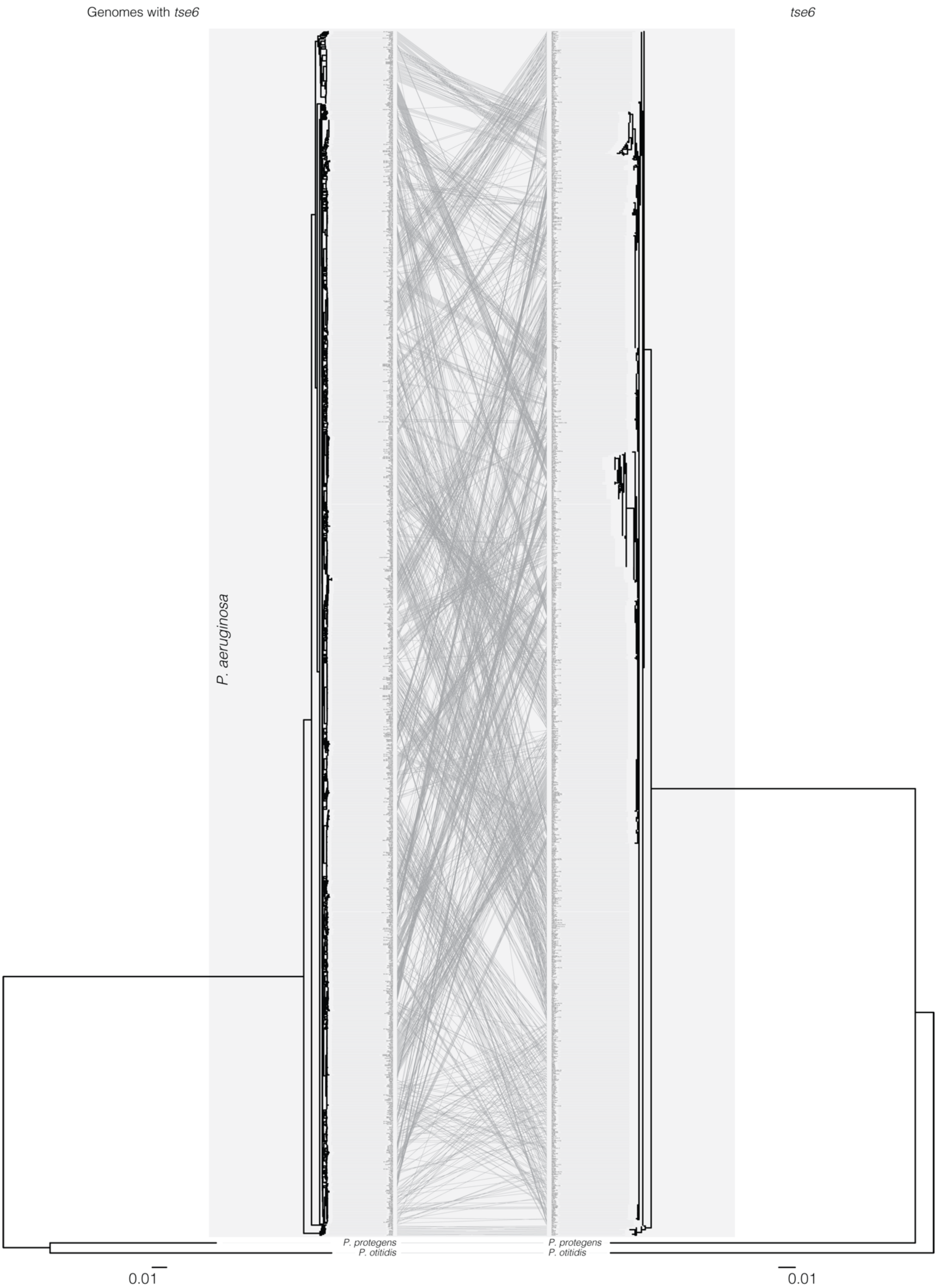

Co-phylogenetic plot of the species tree on the left and the *tse6* tree on the right. The species tree is a maximum-likelihood tree inferred with the HKY+F+I model. The gene tree is a maximum-likelihood tree inferred with the TPM3u+F+R2 model. Both trees are midpoint rooted and their distances are shown in substitutions per site.

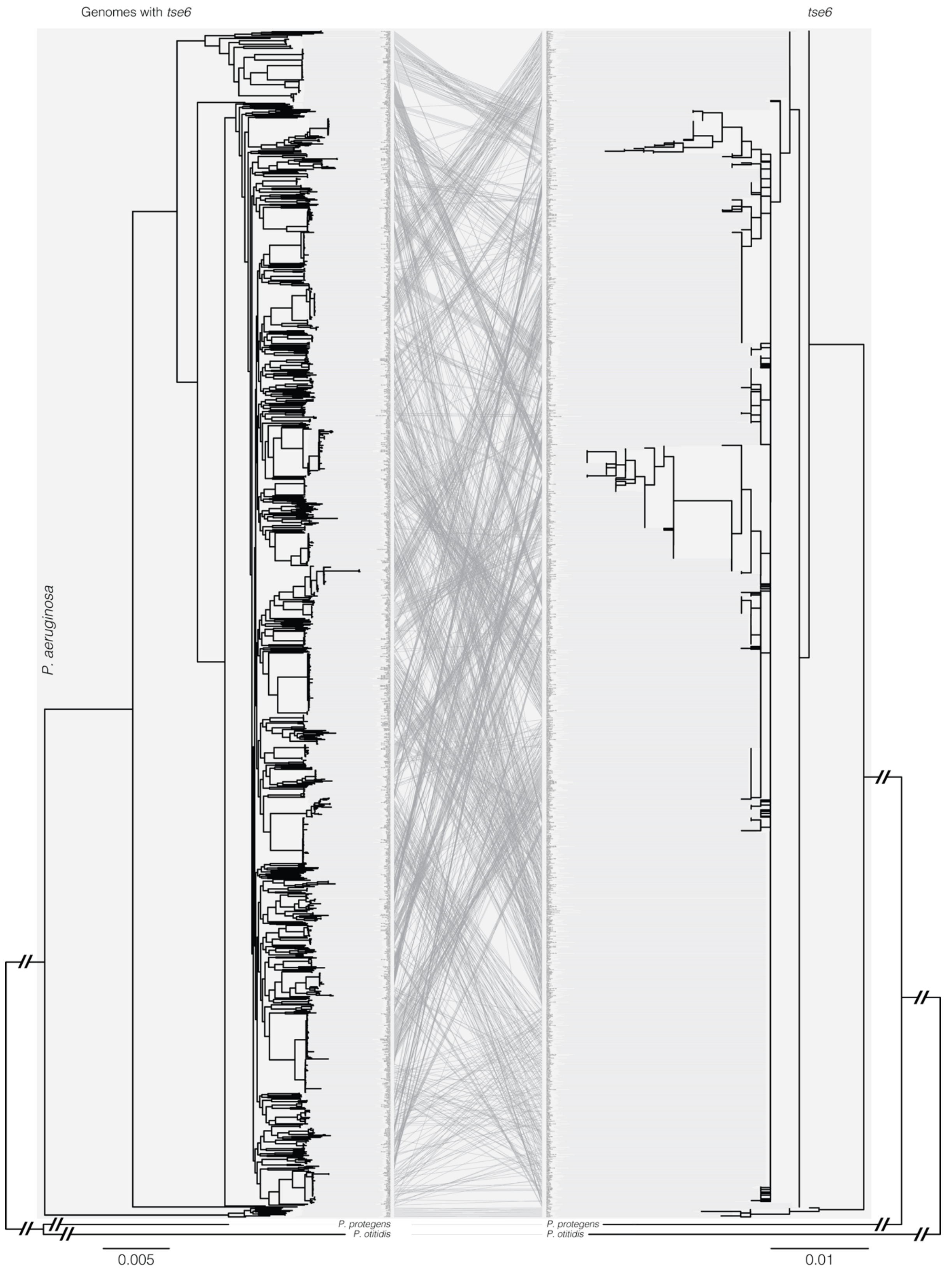

Same co-phylogenetic plot as on previous page, but with cut branches. The species tree is on the left and the *tse6* tree on the right. The species tree is a maximum-likelihood tree inferred with the HKY+F+I model. The gene tree is a maximum-likelihood tree inferred with the TPM3u+F+R2 model. Both trees are midpoint rooted and their distances are shown in substitutions per site.

tas1

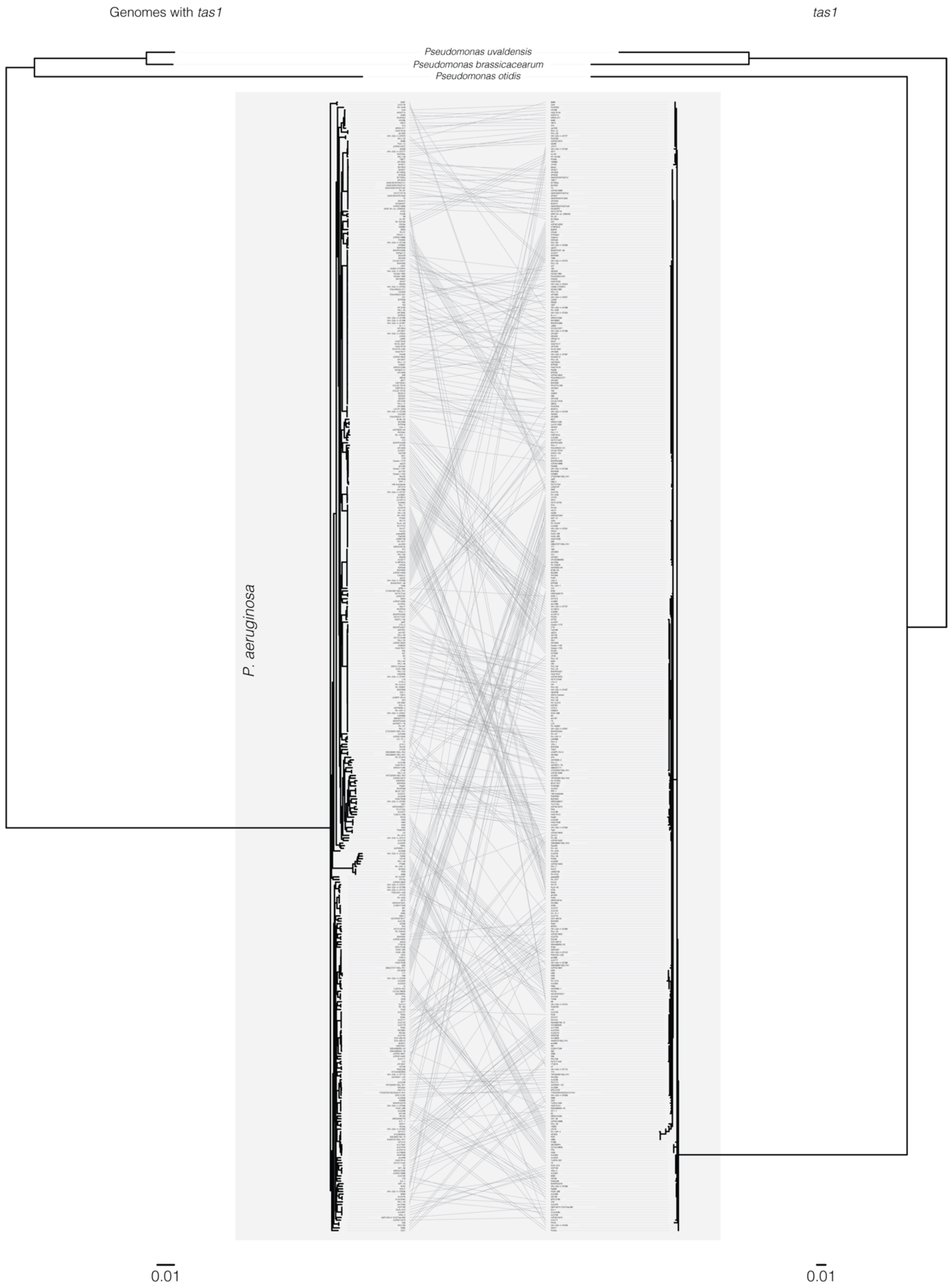

Co-phylogenetic plot of the species tree on the left and the *tas1* tree on the right. The species tree is a maximum-likelihood tree inferred with the HKY+F+I model. The gene tree is a maximum-likelihood tree inferred with the TN+F+R4 model. Both trees are midpoint rooted and their distances are shown in substitutions per site.

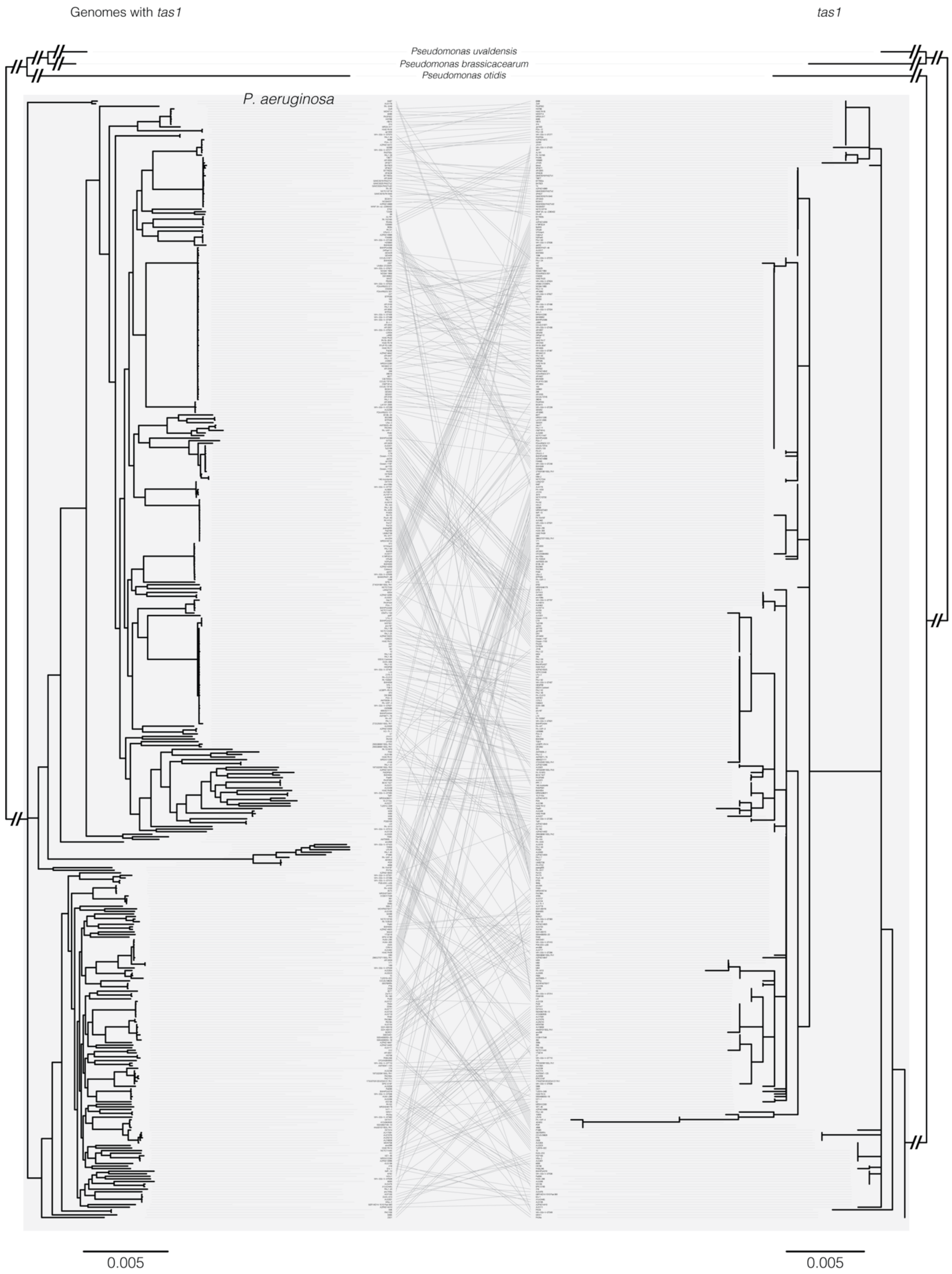

Same co-phylogenetic plot as on previous page, but with cut branches. The species tree is on the left and the *tas1* tree on the right. The species tree is a maximum-likelihood tree inferred with the HKY+F+I model. The gene tree is a maximum-likelihood tree inferred with the TN+F+R4 model. Both trees are midpoint rooted and their distances are shown in substitutions per site.

tne3

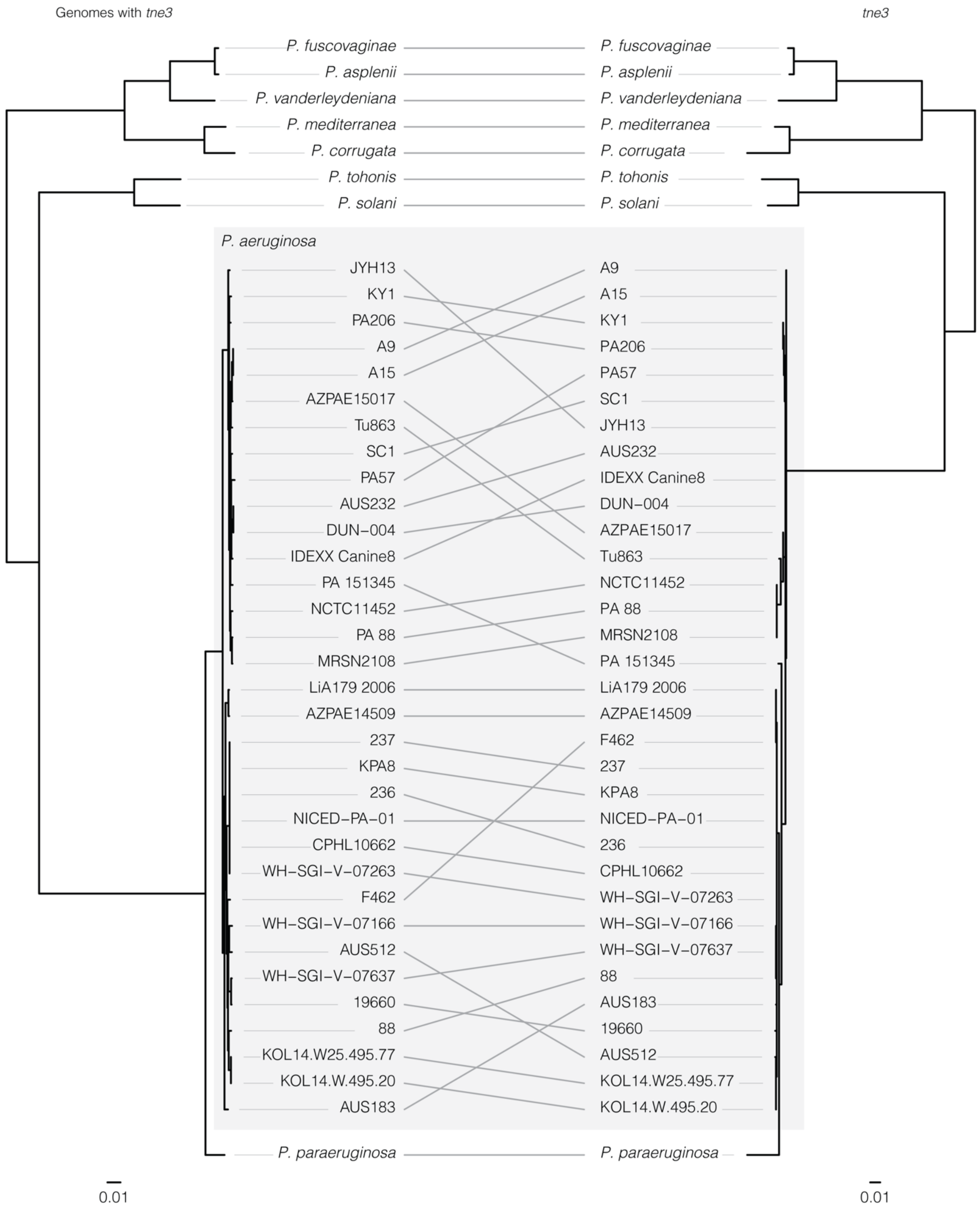

Co-phylogenetic plot of the species tree on the left and the *tne3* tree on the right. The species tree is a maximum-likelihood tree inferred with the HKY+F+I model. The gene tree is a maximum-likelihood tree inferred with the TIM3+F+G4 model. Both trees are midpoint rooted and their distances are shown in substitutions per site.

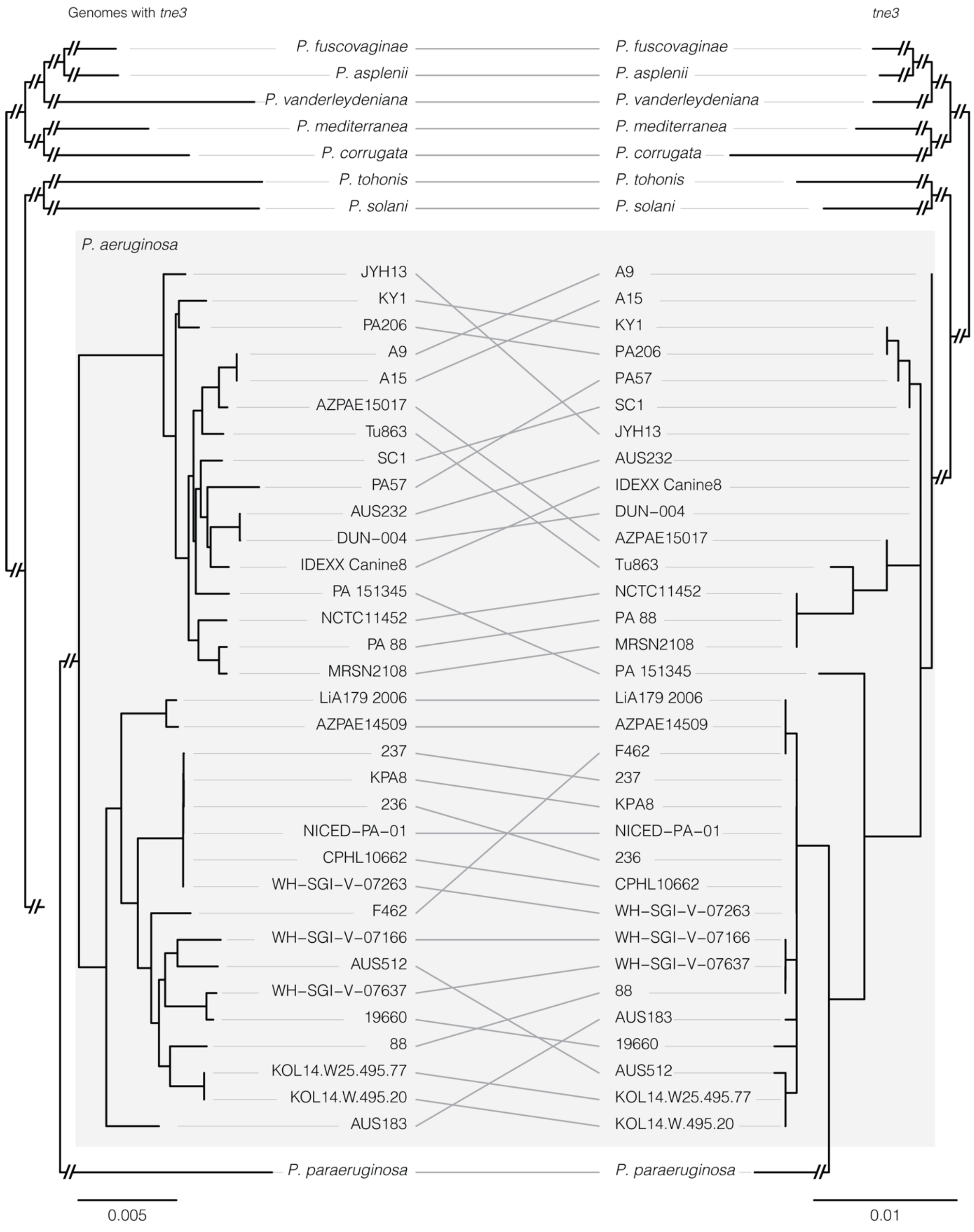

Same co-phylogenetic plot as on previous page, but with cut branches. The species tree is on the left and the *tne3* tree on the right. The species tree is a maximum-likelihood tree inferred with the HKY+F+I model. The gene tree is a maximum-likelihood tree inferred with the TIM3+F+G4 model. Both trees are midpoint rooted and their distances are shown in substitutions per site.

tse7

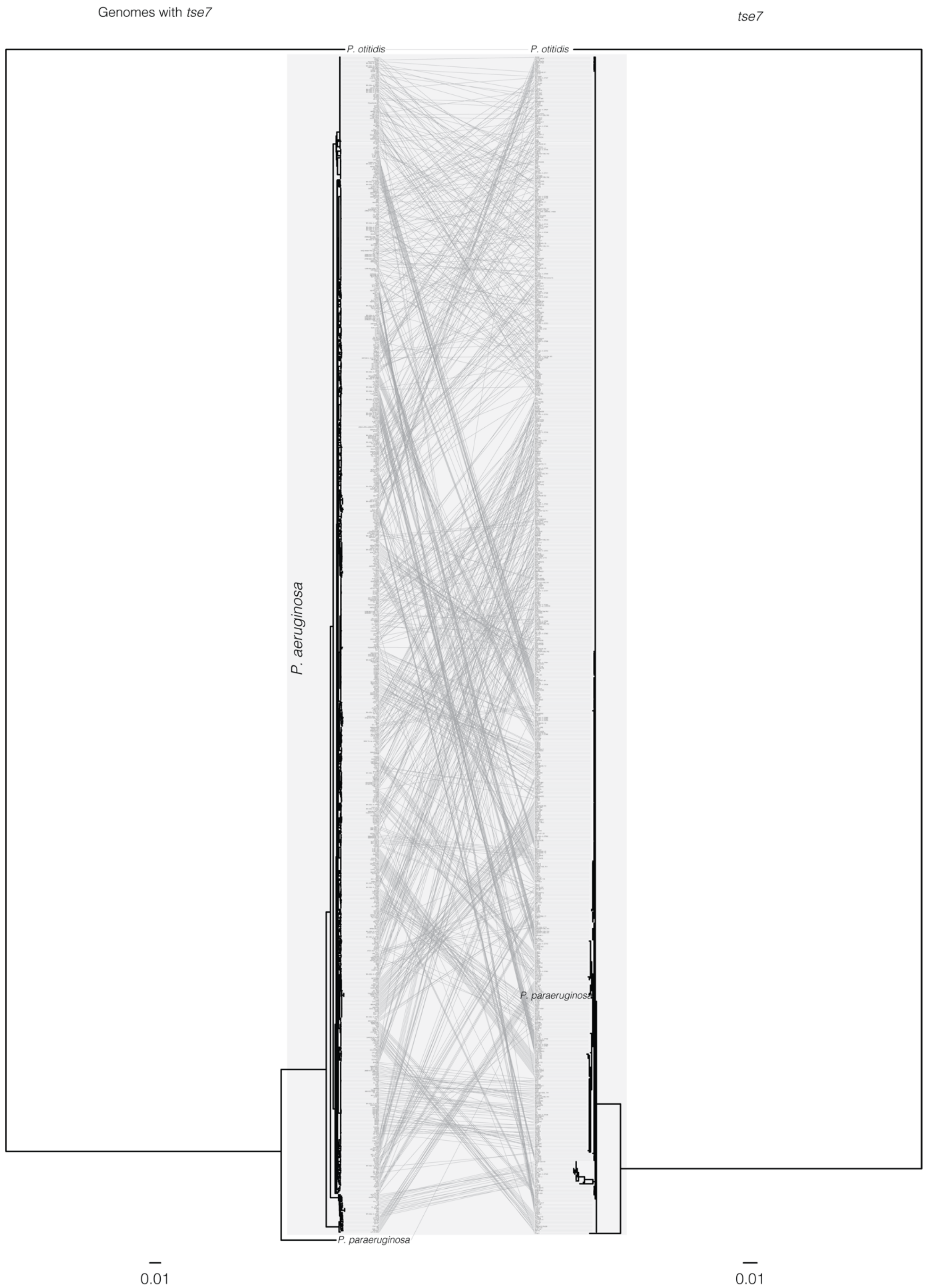

Co-phylogenetic plot of the species tree on the left and the *tse7* tree on the right. The species tree is a maximum-likelihood tree inferred with the HKY+F+I model. The gene tree is a maximum-likelihood tree inferred with the TIM2+F+I model. Both trees are midpoint rooted and their distances are shown in substitutions per site.

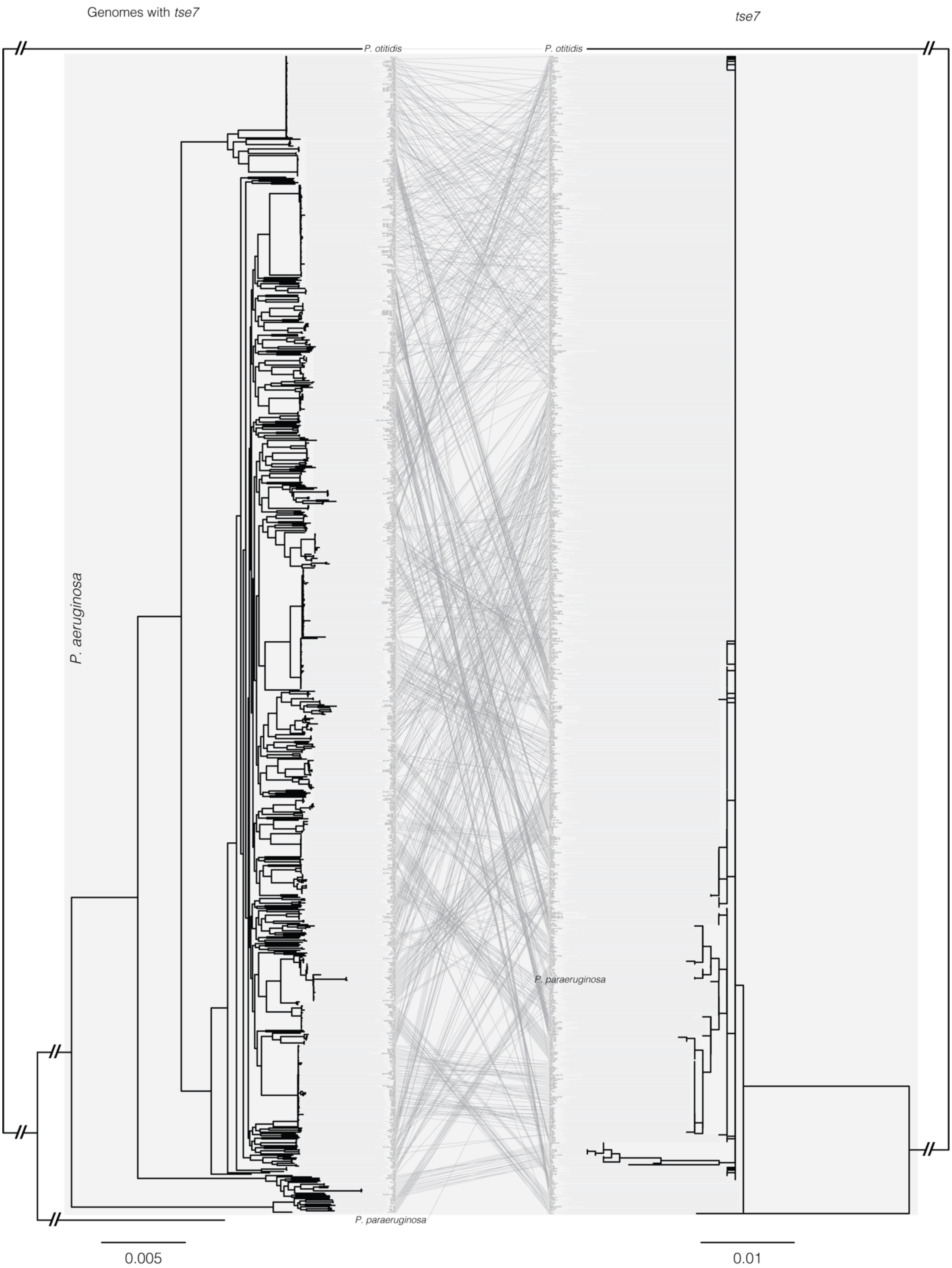

Same co-phylogenetic plot as on previous page, but with cut branches. The species tree is on the left and the *tse7* tree on the right. The species tree is a maximum-likelihood tree inferred with the HKY+F+I model. The gene tree is a maximum-likelihood tree inferred with the TIM2+F+I model. Both trees are midpoint rooted and their distances are shown in substitutions per site.

tse7a

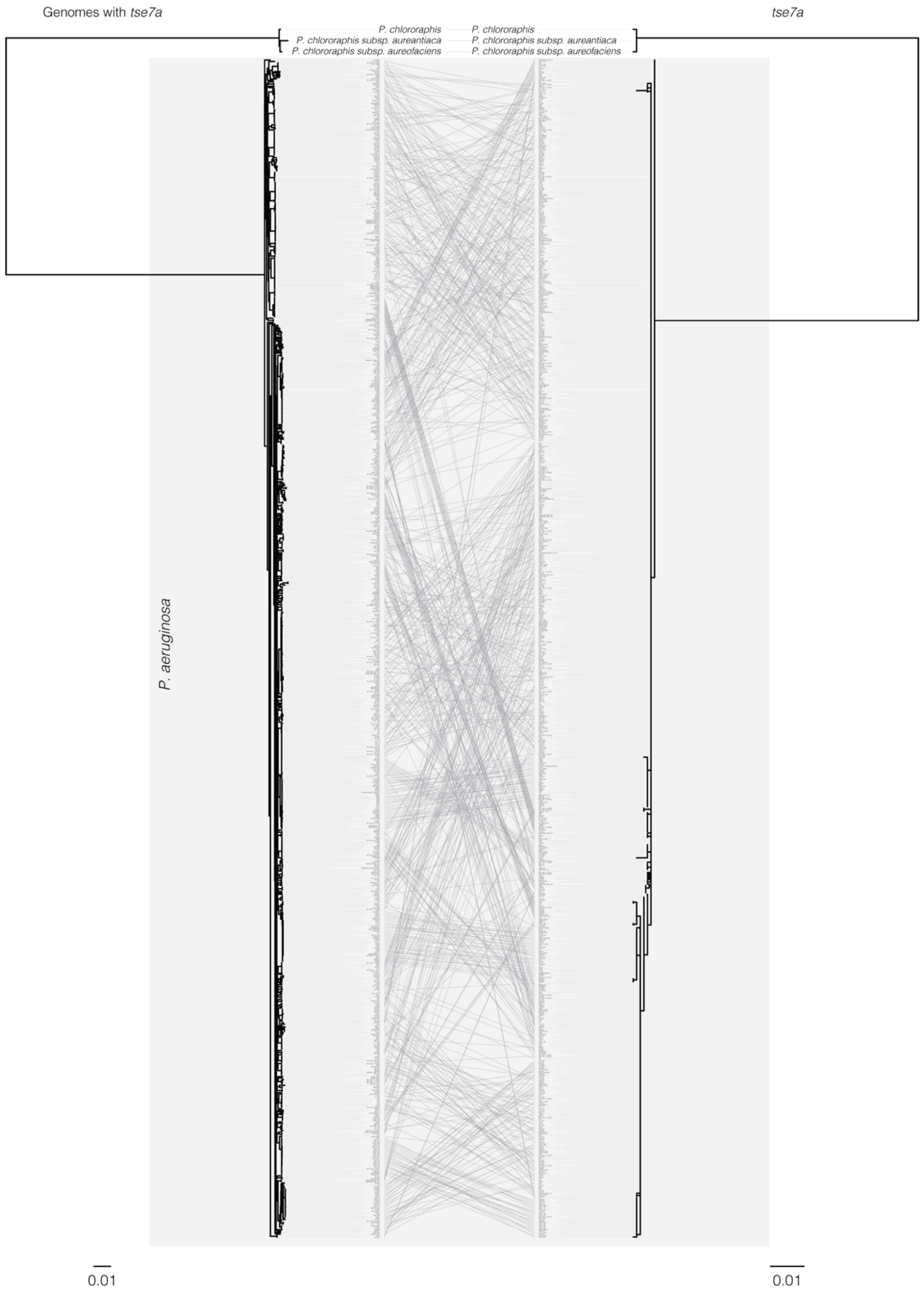

Co-phylogenetic plot of the species tree on the left and the *tse7a* tree on the right. The species tree is a maximum-likelihood tree inferred with the HKY+F+I model. The gene tree is a maximum-likelihood tree inferred with the TN+F+G4 model. Both trees are midpoint rooted and their distances are shown in substitutions per site.

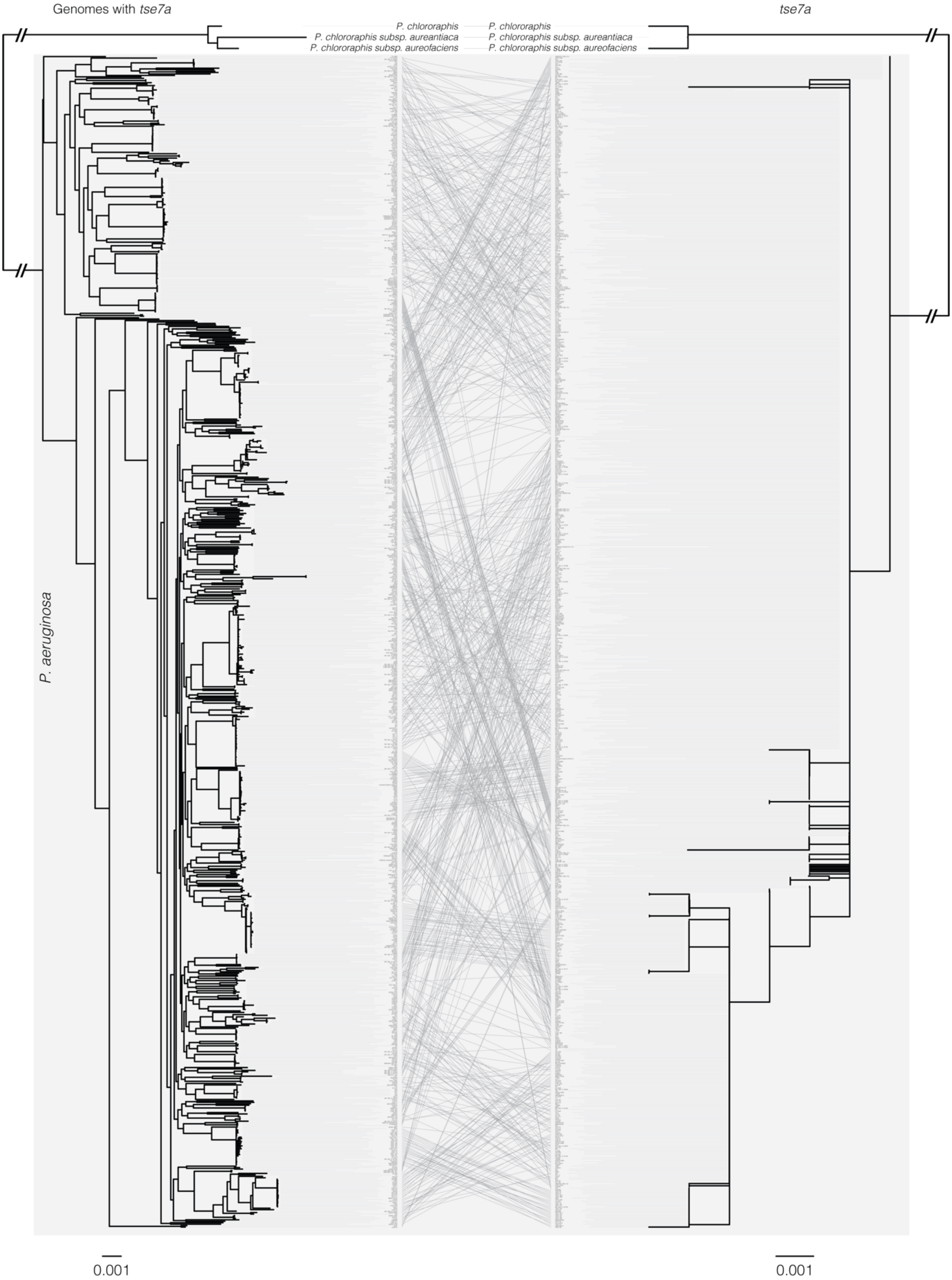

Same co-phylogenetic plot as on previous page, but with cut branches. The species tree is on the left and the *tse7a* tree on the right. The species tree is a maximum-likelihood tree inferred with the HKY+F+I model. The gene tree is a maximum-likelihood tree inferred with the TN+F+G4 model. Both trees are midpoint rooted and their distances are shown in substitutions per site.

tsd1

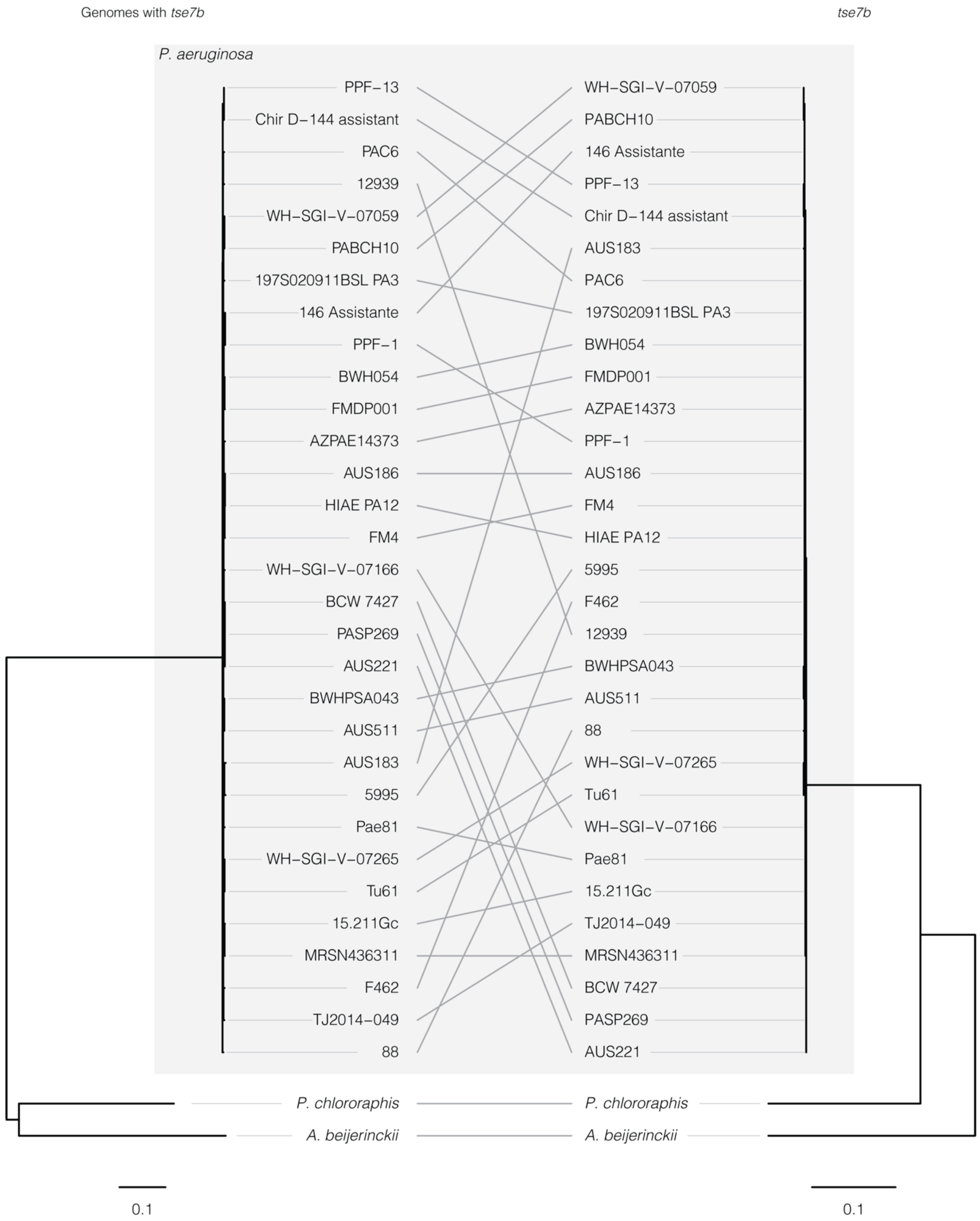

Co-phylogenetic plot of the species tree on the left and the *tsd1* tree on the right. The species tree is a maximum-likelihood tree inferred with the HKY+F+I model. The gene tree is a maximum-likelihood tree inferred with the TIM3+F model. Both trees are midpoint rooted and their distances are shown in substitutions per site.

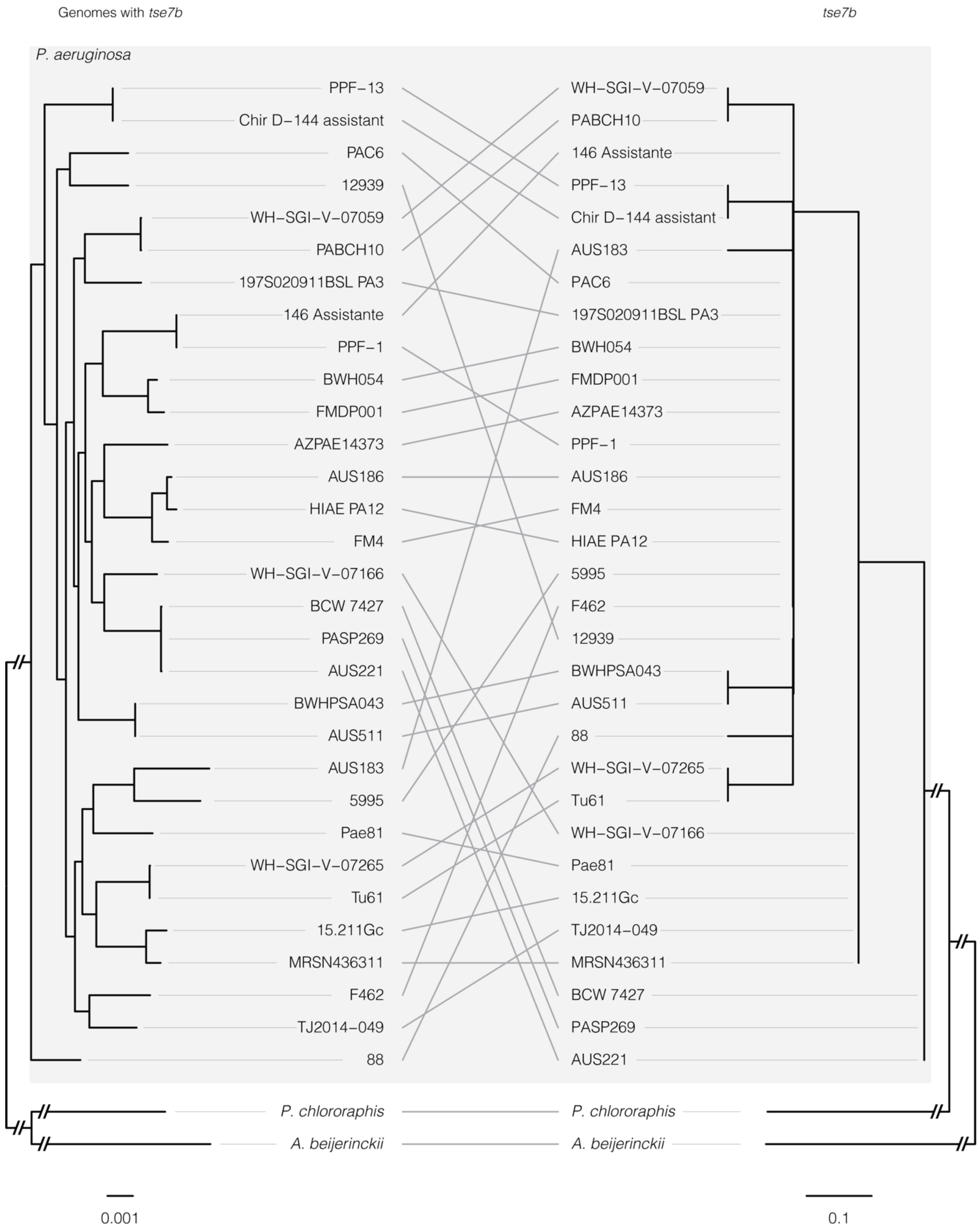

Same co-phylogenetic plot as on previous page, but with cut branches. The species tree is on the left and the *tsd1* tree on the right. The species tree is a maximum-likelihood tree inferred with the HKY+F+I model. The gene tree is a maximum-likelihood tree inferred with the TIM3+F model. Both trees are midpoint rooted and their distances are shown in substitutions per site.

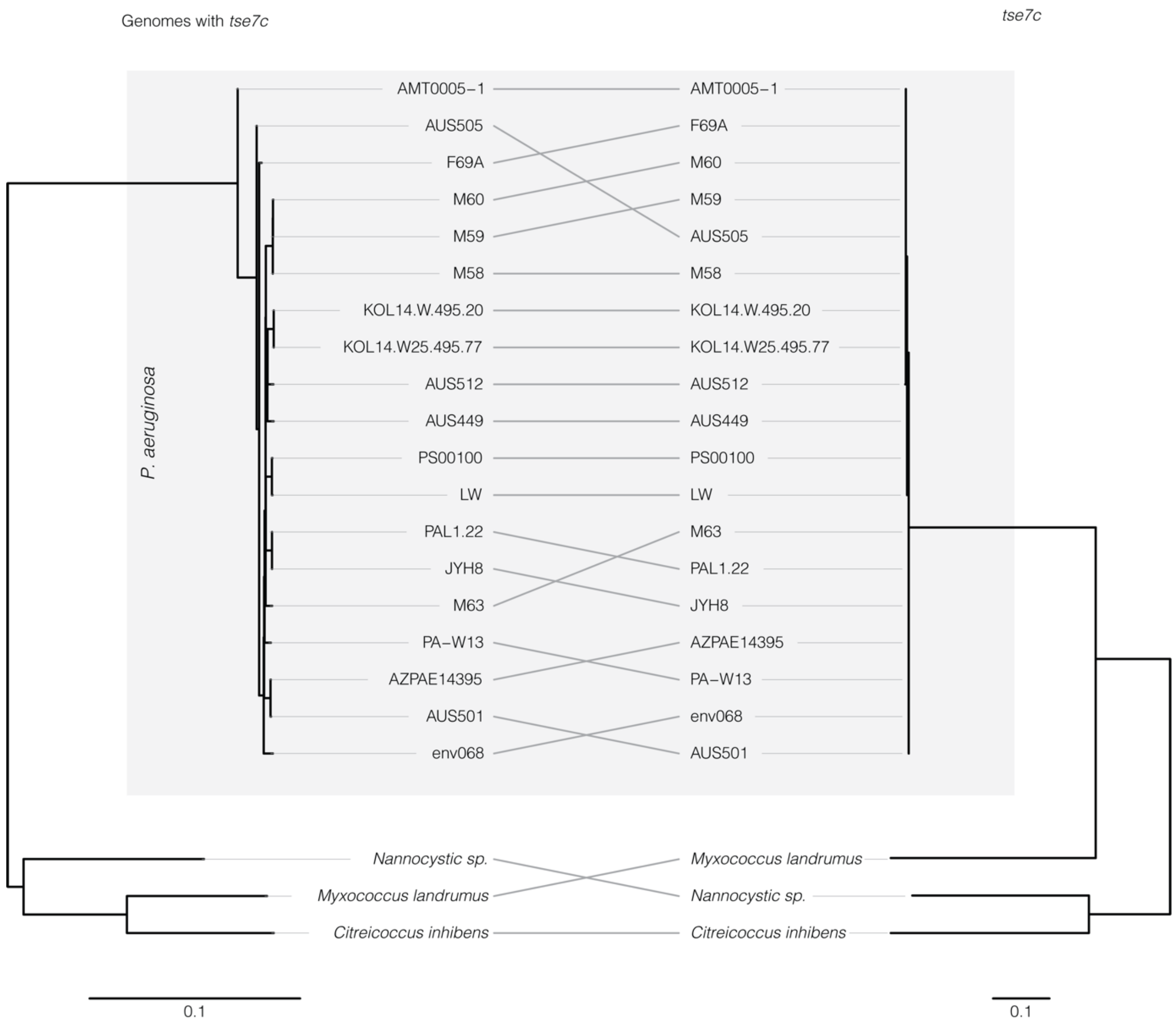

Co-phylogenetic plot of the species tree on the left and the *tse7c* tree on the right. The species tree is a maximum-likelihood tree inferred with the HKY+F+I model. The gene tree is a maximum-likelihood tree inferred with the HKY+F+G4 model. Both trees are midpoint rooted and their distances are shown in substitutions per site.

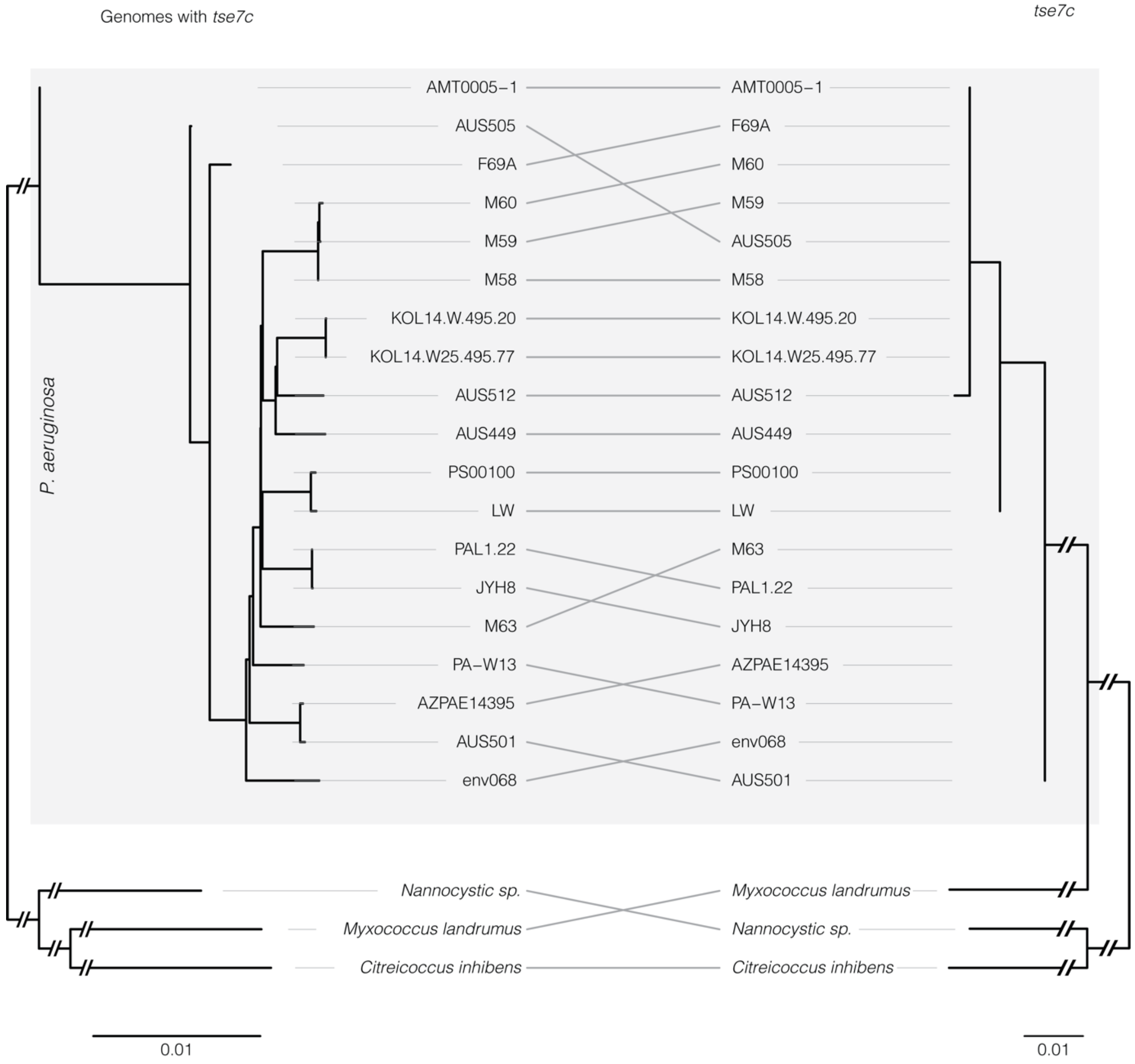

Same co-phylogenetic plot as on previous page, but with cut branches. The species tree is on the left and the *tse7c* tree on the right. The species tree is a maximum-likelihood tree inferred with the HKY+F+I model. The gene tree is a maximum-likelihood tree inferred with the HKY+F+G4 model. Both trees are midpoint rooted and their distances are shown in substitutions per site.

tse7d

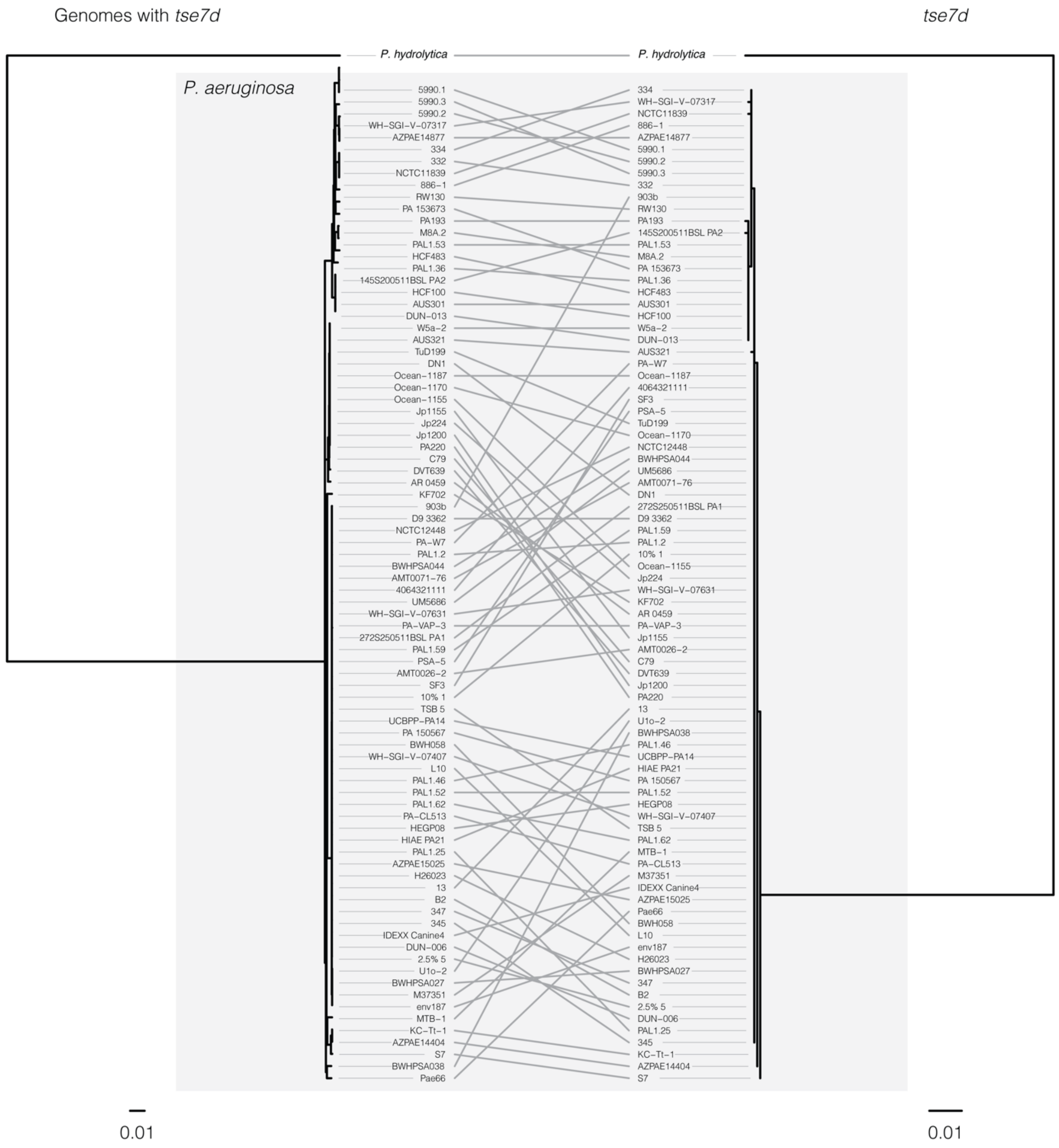

Co-phylogenetic plot of the species tree on the left and the *tse7d* tree on the right. The species tree is a maximum-likelihood tree inferred with the HKY+F+I model. The gene tree is a maximum-likelihood tree inferred with the HKY+F model. Both trees are midpoint rooted and their distances are shown in substitutions per site.

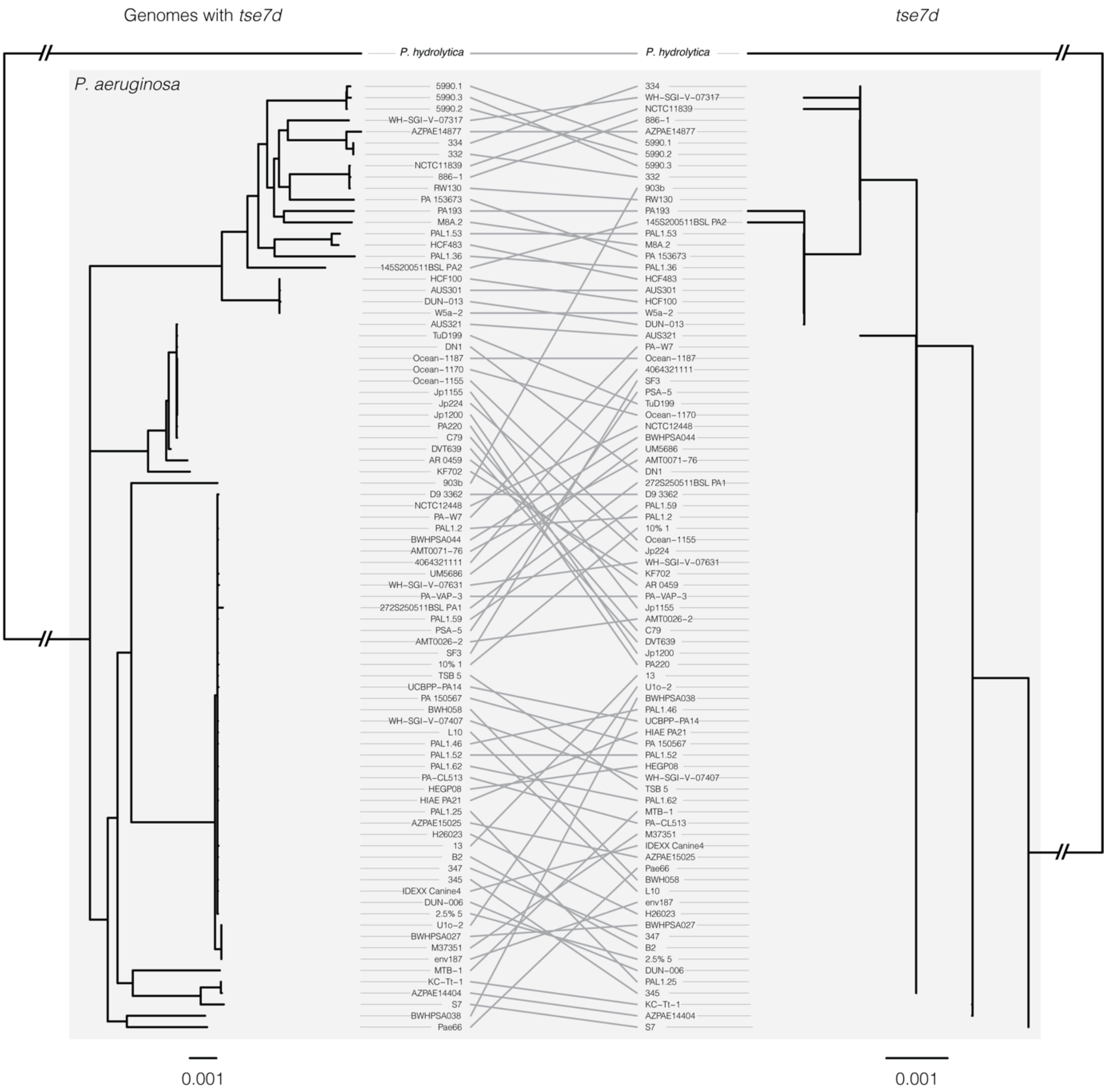

Same co-phylogenetic plot as on previous page, but with cut branches. The species tree is on the left and the *tse7d* tree on the right. The species tree is a maximum-likelihood tree inferred with the HKY+F+I model. The gene tree is a maximum-likelihood tree inferred with the HKY+F model. Both trees are midpoint rooted and their distances are shown in substitutions per site.

tse7e

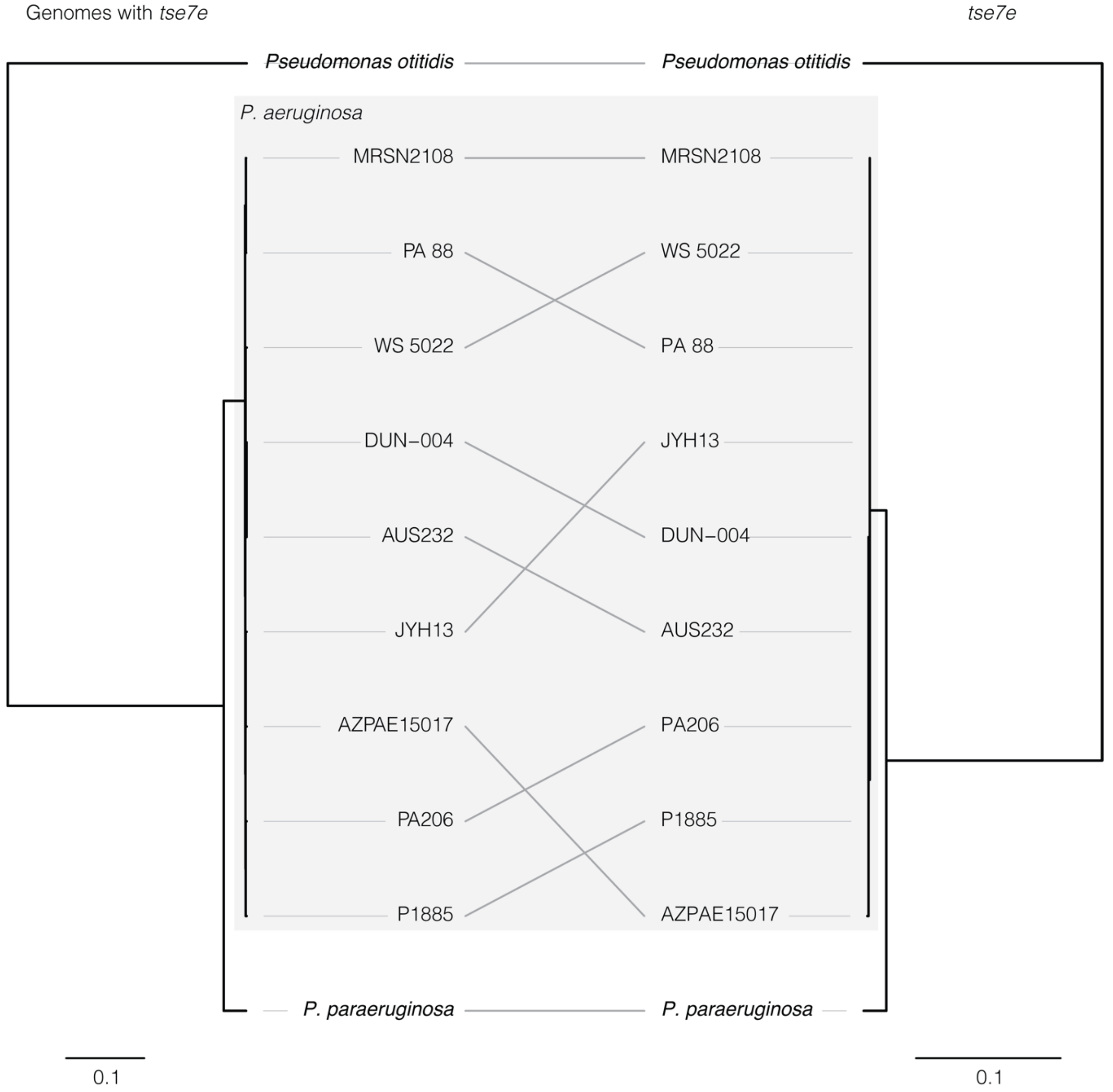

Co-phylogenetic plot of the species tree on the left and the *tse7e* tree on the right. The species tree is a maximum-likelihood tree inferred with the HKY+F+I model. The gene tree is a maximum-likelihood tree inferred with the TPM3+F model. Both trees are midpoint rooted and their distances are shown in substitutions per site.

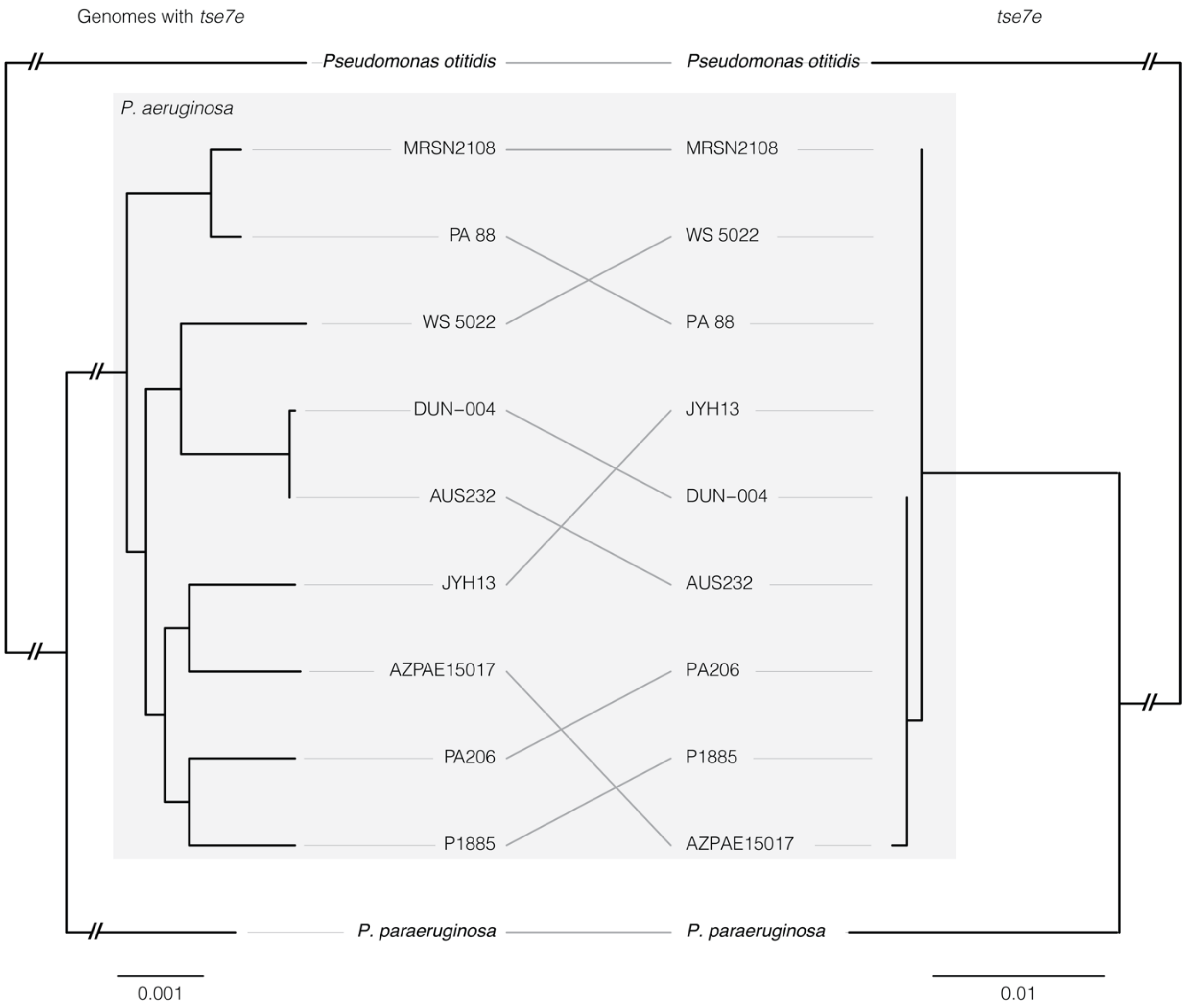

Same co-phylogenetic plot as on previous page, but with cut branches. The species tree is on the left and the *tse7e* tree on the right. The species tree is a maximum-likelihood tree inferred with the HKY+F+I model. The gene tree is a maximum-likelihood tree inferred with the TPM3+F model. Both trees are midpoint rooted and their distances are shown in substitutions per site.

tle3

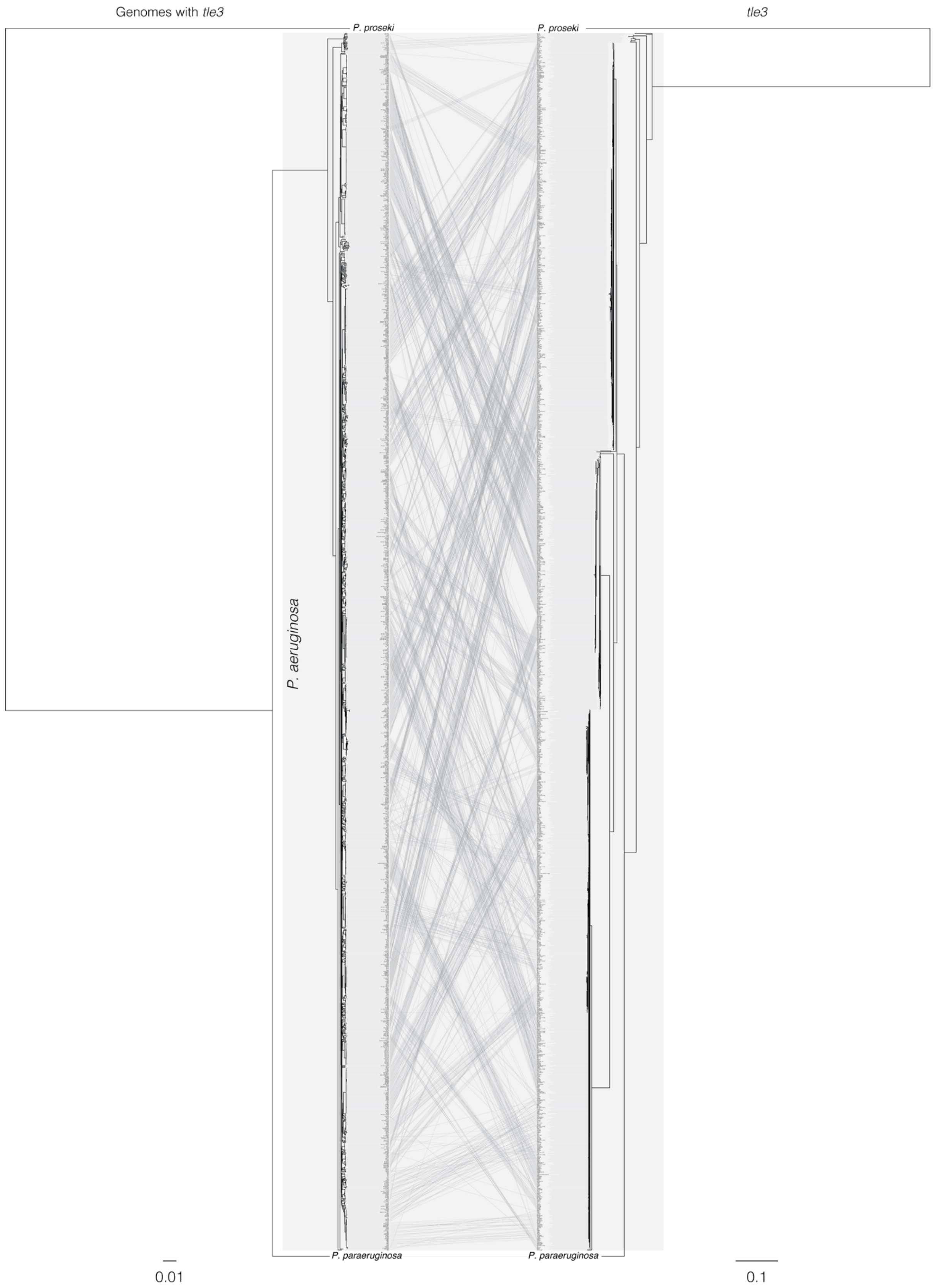

Co-phylogenetic plot of the species tree on the left and the *tle3* tree on the right. The species tree is a maximum-likelihood tree inferred with the HKY+F+I model. The gene tree is a maximum-likelihood tree inferred with the TPM2+F+R8 model. Both trees are midpoint rooted and their distances are shown in substitutions per site.

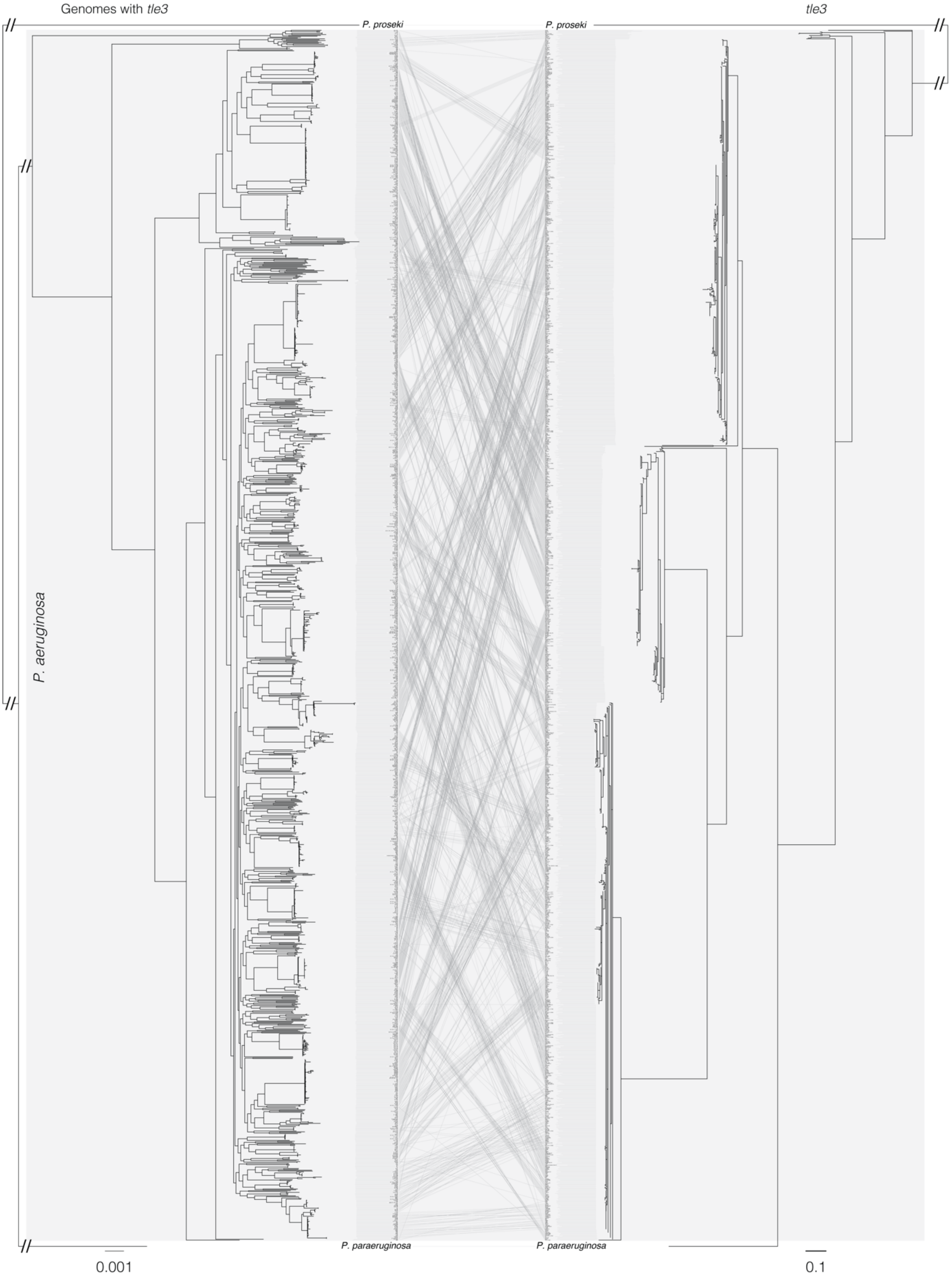

Same co-phylogenetic plot as on previous page, but with cut branches. The species tree is on the left and the *tle3* tree on the right. The species tree is a maximum-likelihood tree inferred with the HKY+F+I model. The gene tree is a maximum-likelihood tree inferred with the TPM2+F+R8 model. Both trees are midpoint rooted and their distances are shown in substitutions per site.

tle4b

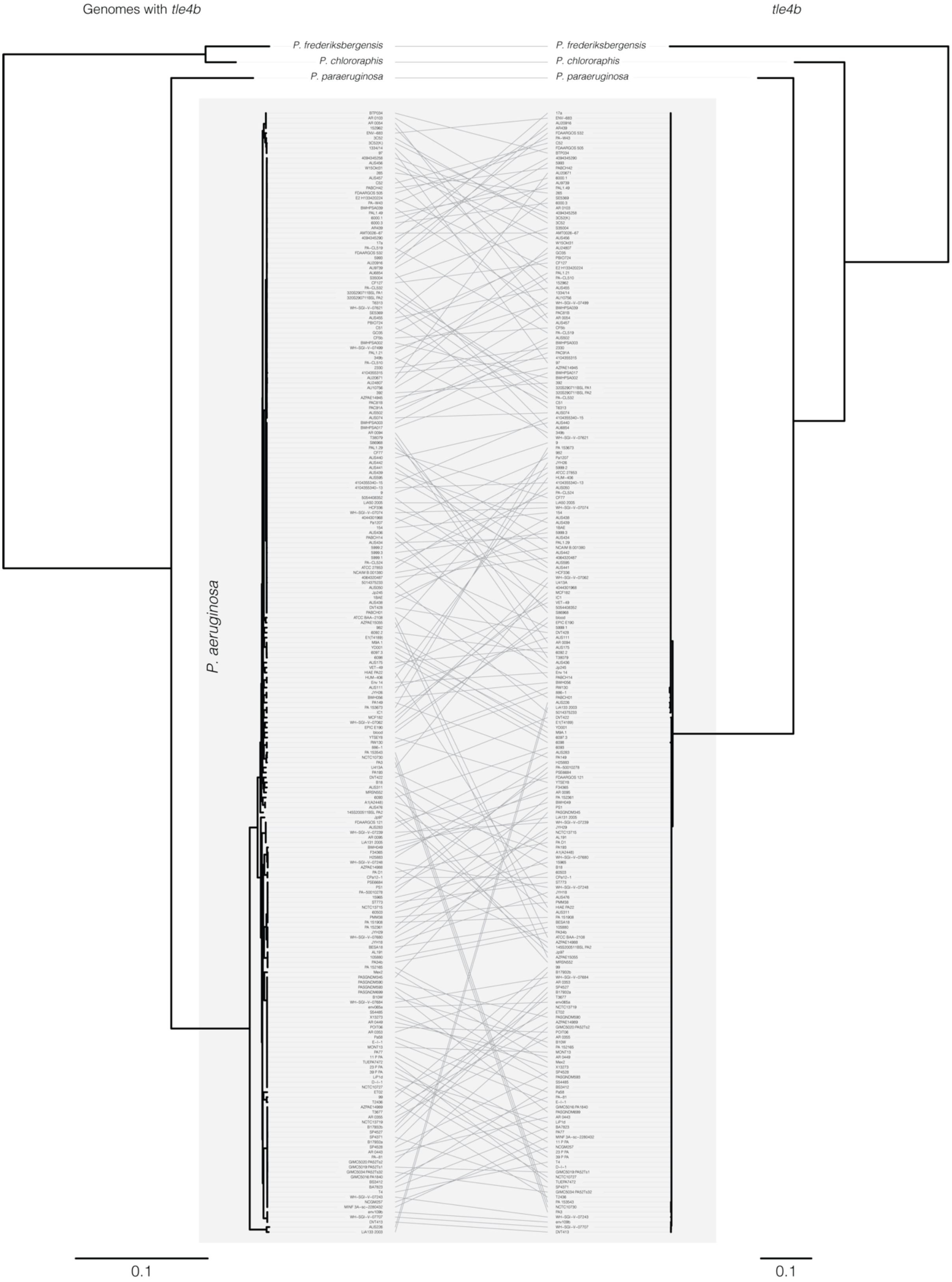

Co-phylogenetic plot of the species tree on the left and the *tle4b* tree on the right. The species tree is a maximum-likelihood tree inferred with the HKY+F+I model. The gene tree is a maximum-likelihood tree inferred with the K2P+I model. Both trees are midpoint rooted and their distances are shown in substitutions per site.

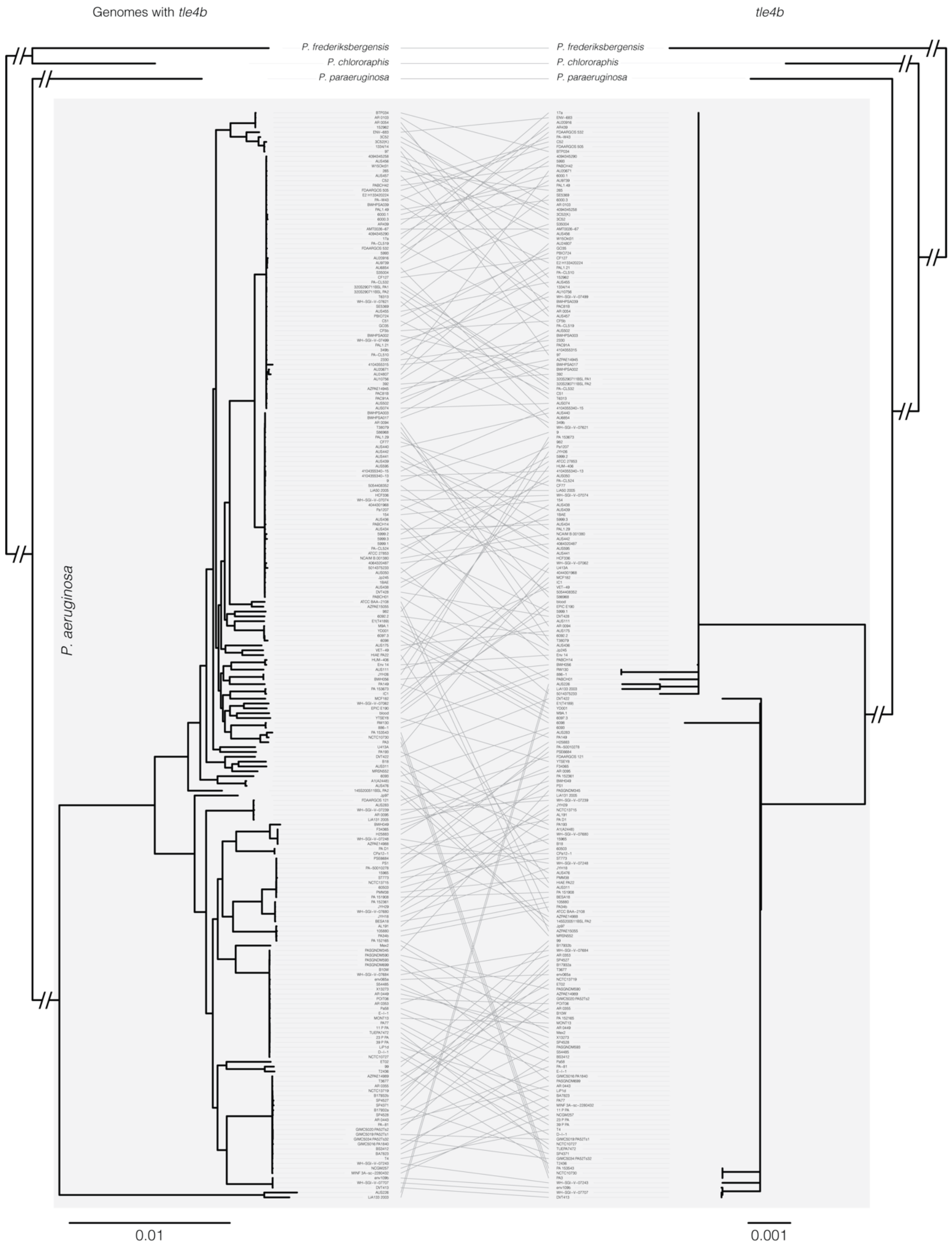

Same co-phylogenetic plot as on previous page, but with cut branches. The species tree is on the left and the *tle4b* tree on the right. The species tree is a maximum-likelihood tree inferred with the HKY+F+I model. The gene tree is a maximum-likelihood tree inferred with the K2P+I model. Both trees are midpoint rooted and their distances are shown in substitutions per site.

vgrG2b-C-ter

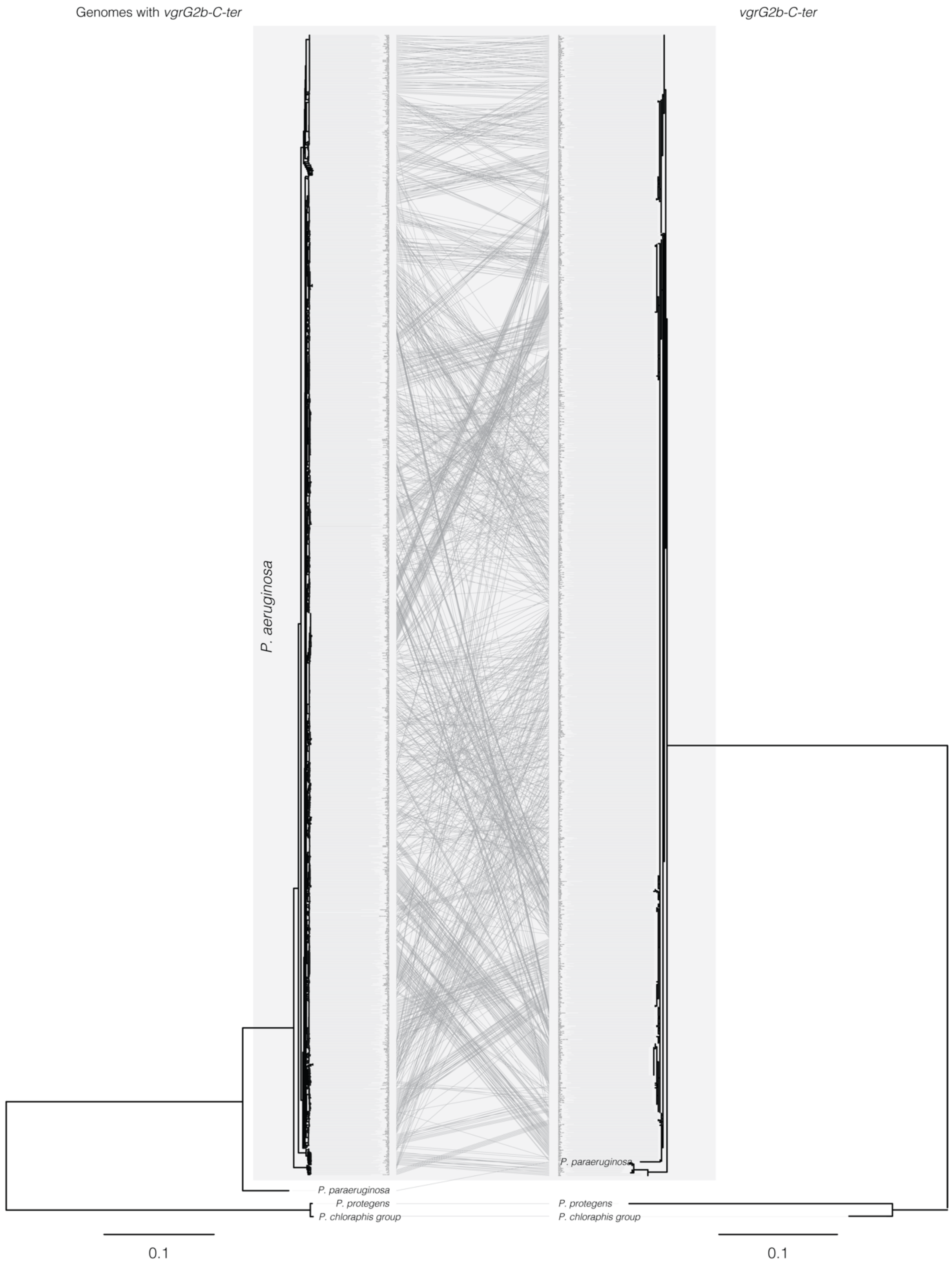

Co-phylogenetic plot of the species tree on the left and the *vgrG2b-C-ter* tree on the right. The species tree is a maximum-likelihood tree inferred with the HKY+F+I model. The gene tree is a maximum-likelihood tree inferred with the TPM3u+F+G4 model. Both trees are midpoint rooted and their distances are shown in substitutions per site.

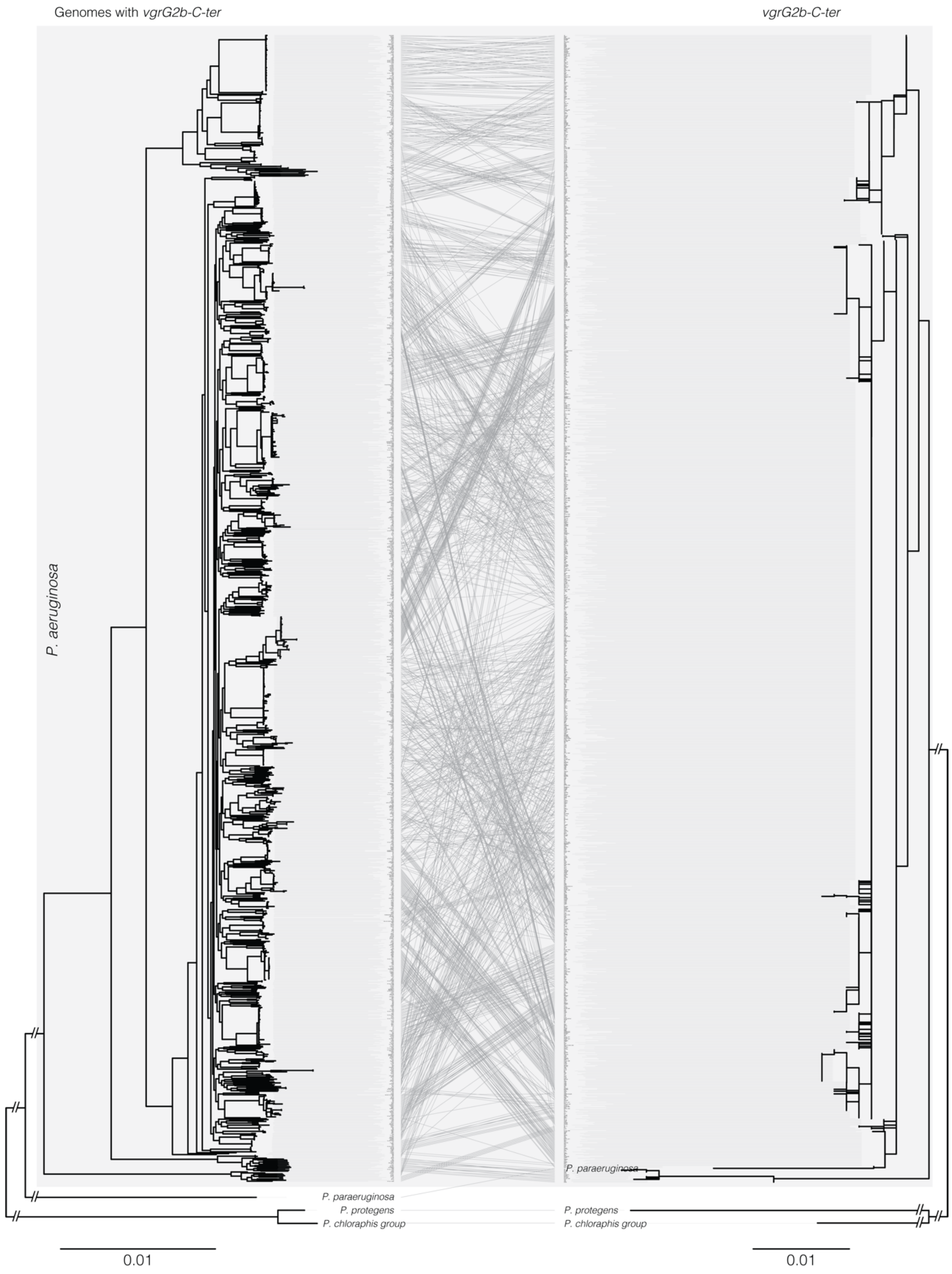

Same co-phylogenetic plot as on previous page, but with cut branches. The species tree is on the left and the *vgrG2b-C-ter* tree on the right. The species tree is a maximum-likelihood tree inferred with the HKY+F+I model. The gene tree is a maximum-likelihood tree inferred with the TPM3u+F+G4 model. Both trees are midpoint rooted and their distances are shown in substitutions per site.

tseV

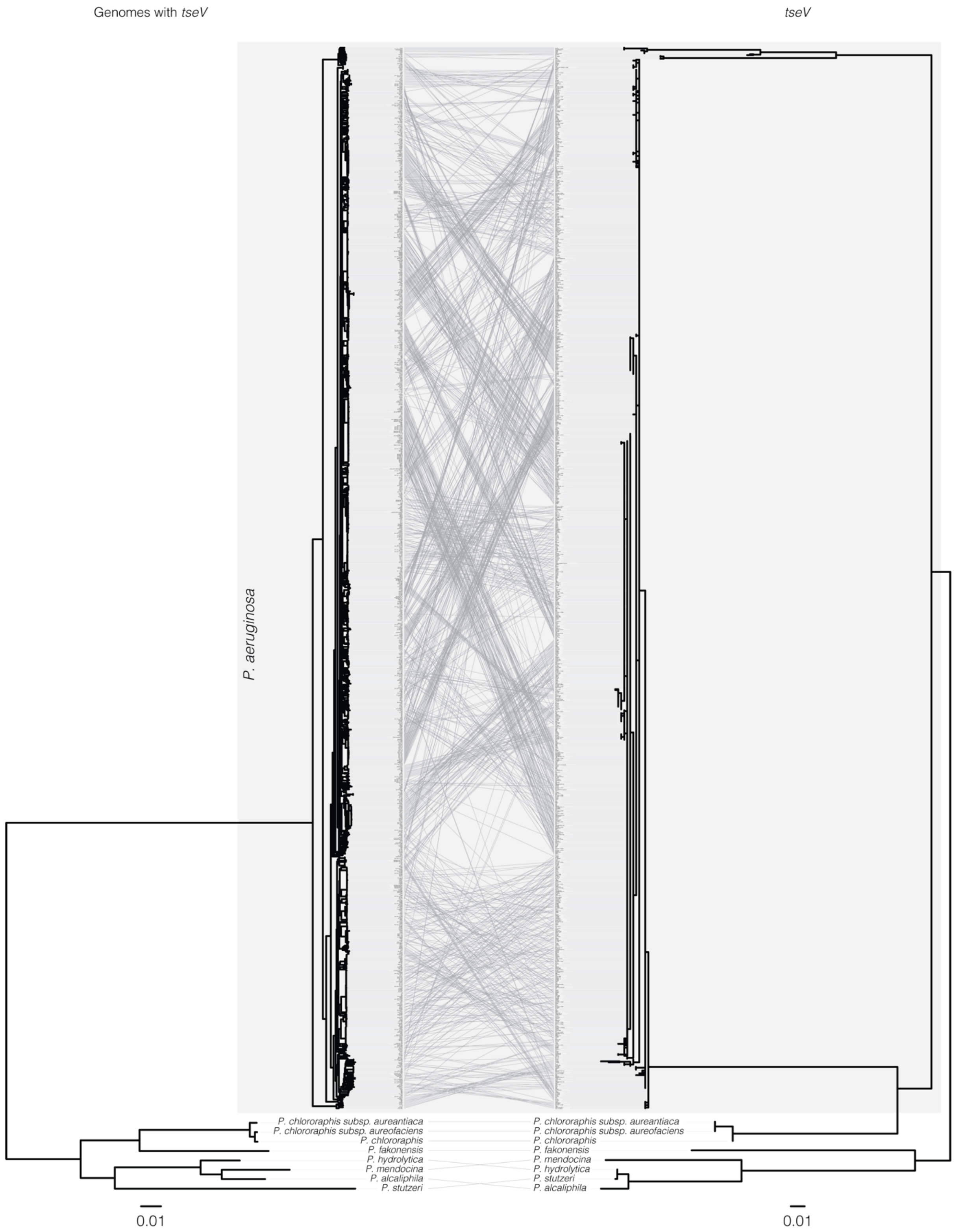

Co-phylogenetic plot of the species tree on the left and the *tseV* tree on the right. The species tree is a maximum-likelihood tree inferred with the HKY+F+I model. The gene tree is a maximum-likelihood tree inferred with the HKY+F+G4 model. Both trees are midpoint rooted and their distances are shown in substitutions per site.

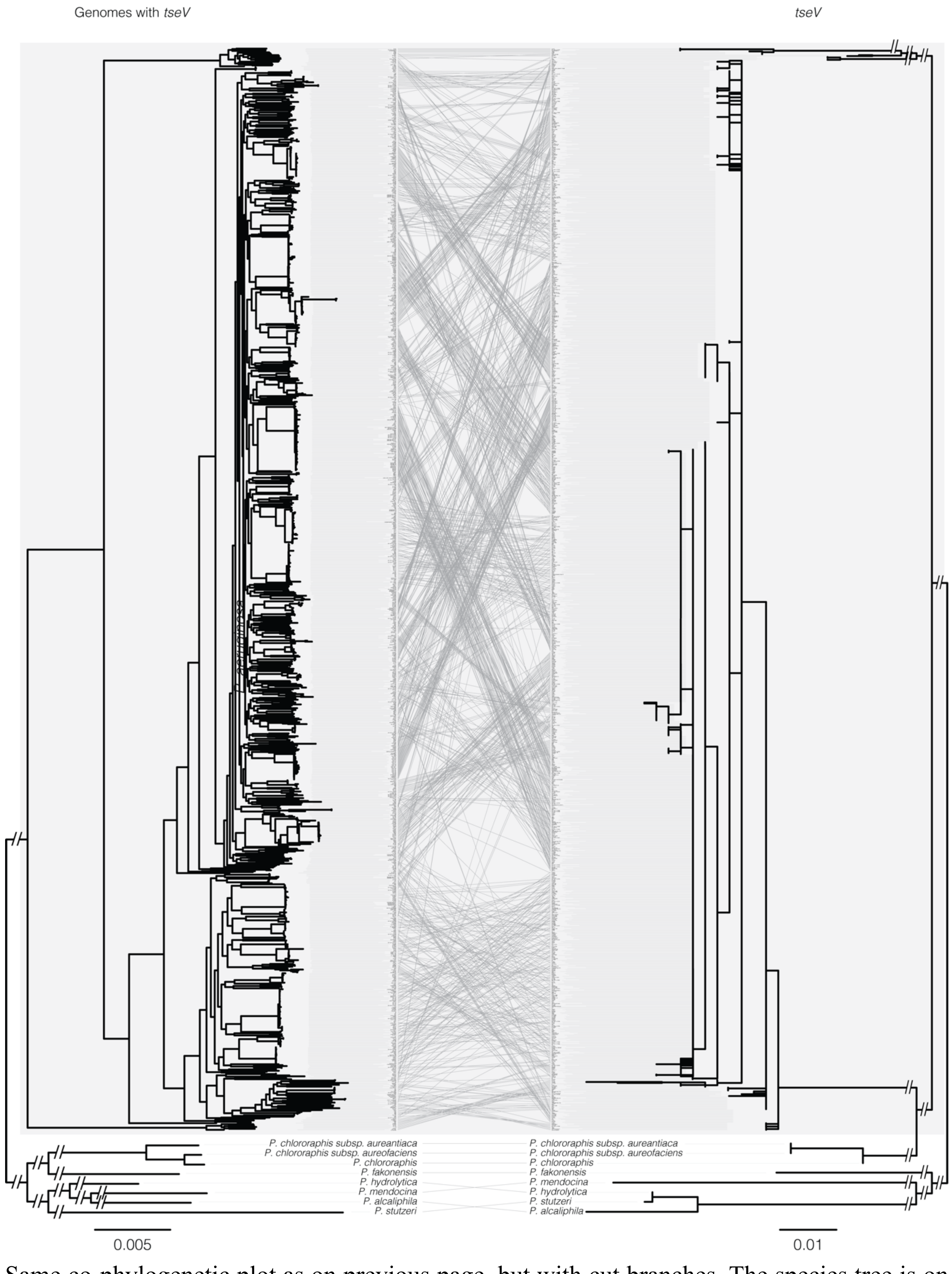

Same co-phylogenetic plot as on previous page, but with cut branches. The species tree is on the left and the *tseV* tree on the right. The species tree is a maximum-likelihood tree inferred with the HKY+F+I model. The gene tree is a maximum-likelihood tree inferred with the HKY+F+G4 model. Both trees are midpoint rooted and their distances are shown in substitutions per site.

pldA

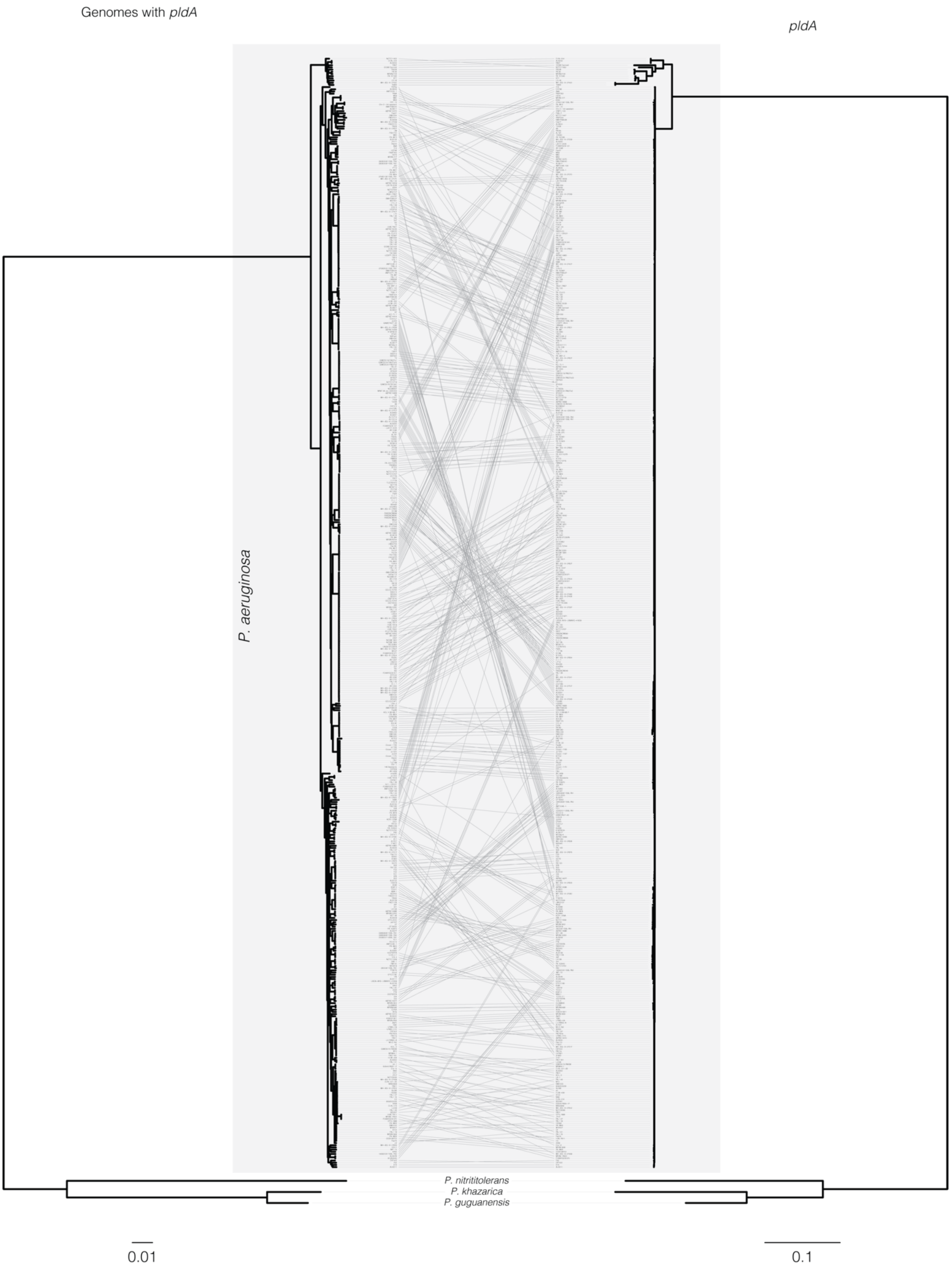

Co-phylogenetic plot of the species tree on the left and the *pldA* tree on the right. The species tree is a maximum-likelihood tree inferred with the HKY+F+I model. The gene tree is a maximum-likelihood tree inferred with the TPM2u+F+G4 model. Both trees are midpoint rooted and their distances are shown in substitutions per site.

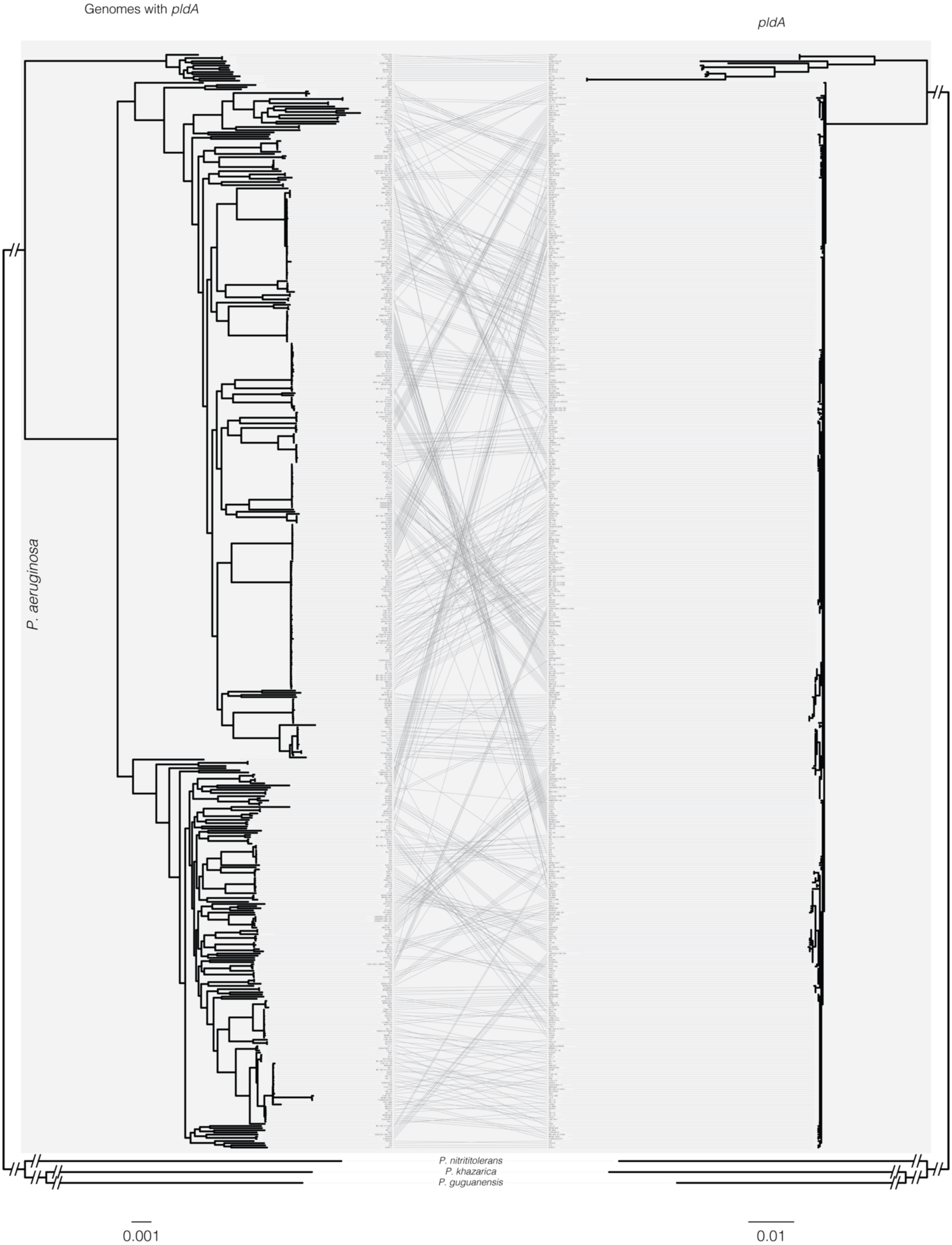

Same co-phylogenetic plot as on previous page, but with cut branches. The species tree is on the left and the *pldA* tree on the right. The species tree is a maximum-likelihood tree inferred with the HKY+F+I model. The gene tree is a maximum-likelihood tree inferred with the TPM2u+F+G4 model. Both trees are midpoint rooted and their distances are shown in substitutions per site.

tle2

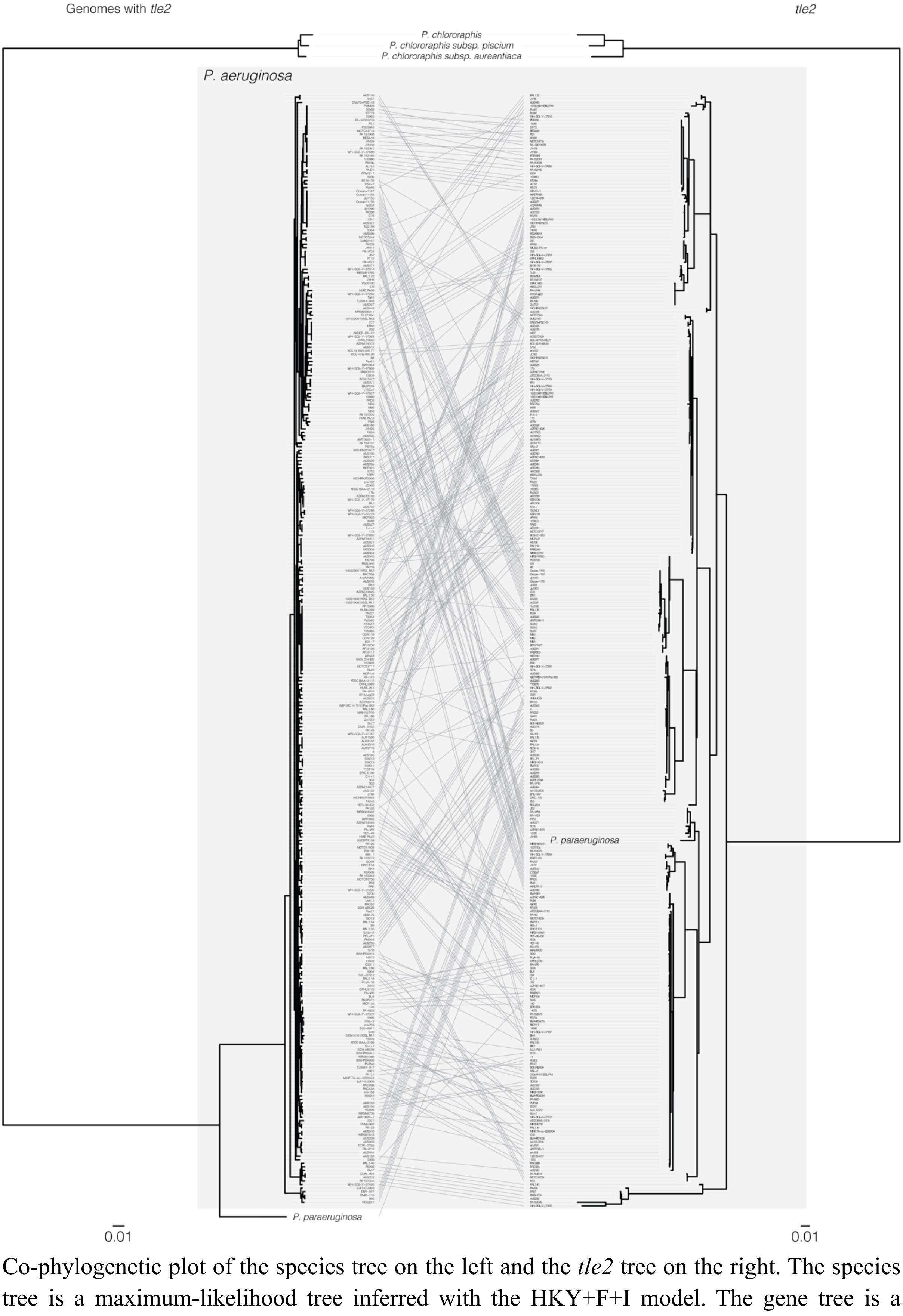

Co-phylogenetic plot of the species tree on the left and the *tle2* tree on the right. The species tree is a maximum-likelihood tree inferred with the HKY+F+I model. The gene tree is a maximum-likelihood tree inferred with the GTR+F+R5 model. Both trees are midpoint rooted and their distances are shown in substitutions per site.

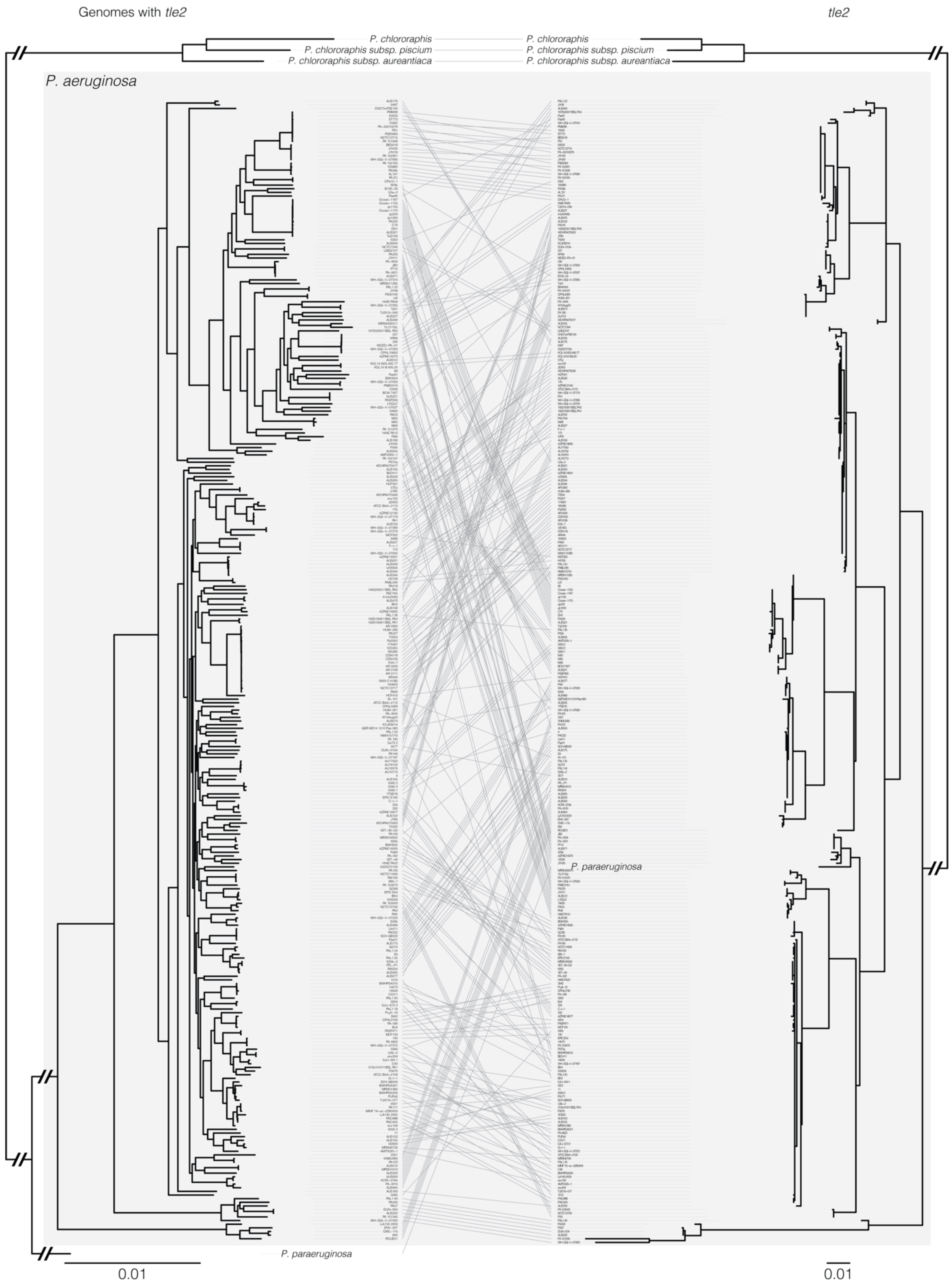

Same co-phylogenetic plot as on previous page, but with cut branches. The species tree is on the left and the *tle2* tree on the right. The species tree is a maximum-likelihood tree inferred with the HKY+F+I model. The gene tree is a maximum-likelihood tree inferred with the GTR+F+R5 model. Both trees are midpoint rooted and their distances are shown in substitutions per site.

tspE1a

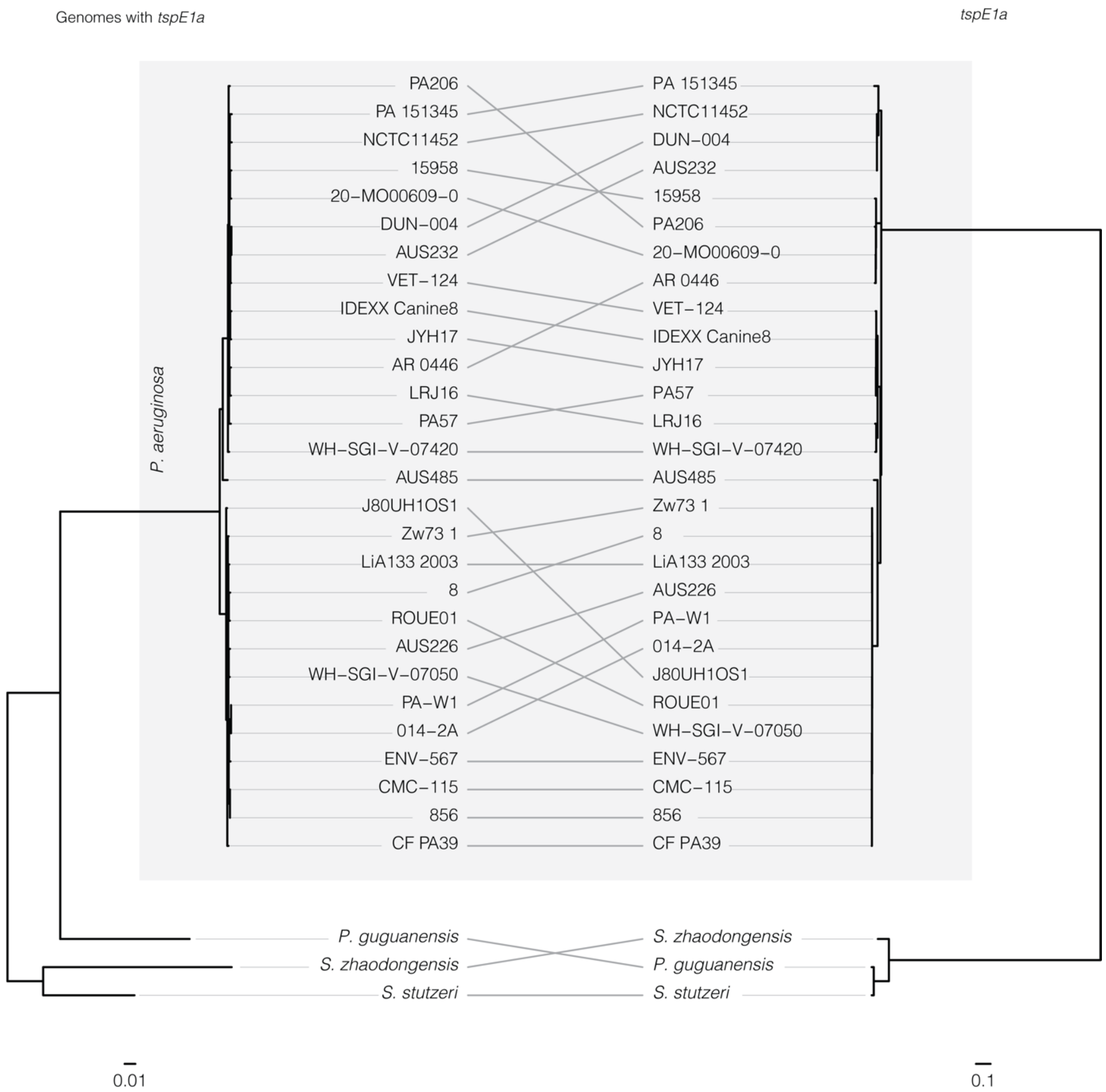

Co-phylogenetic plot of the species tree on the left and the *tspE1a* tree on the right. The species tree is a maximum-likelihood tree inferred with the HKY+F+I model. The gene tree is a maximum-likelihood tree inferred with the TPM2+F+G4 model. Both trees are midpoint rooted and their distances are shown in substitutions per site.

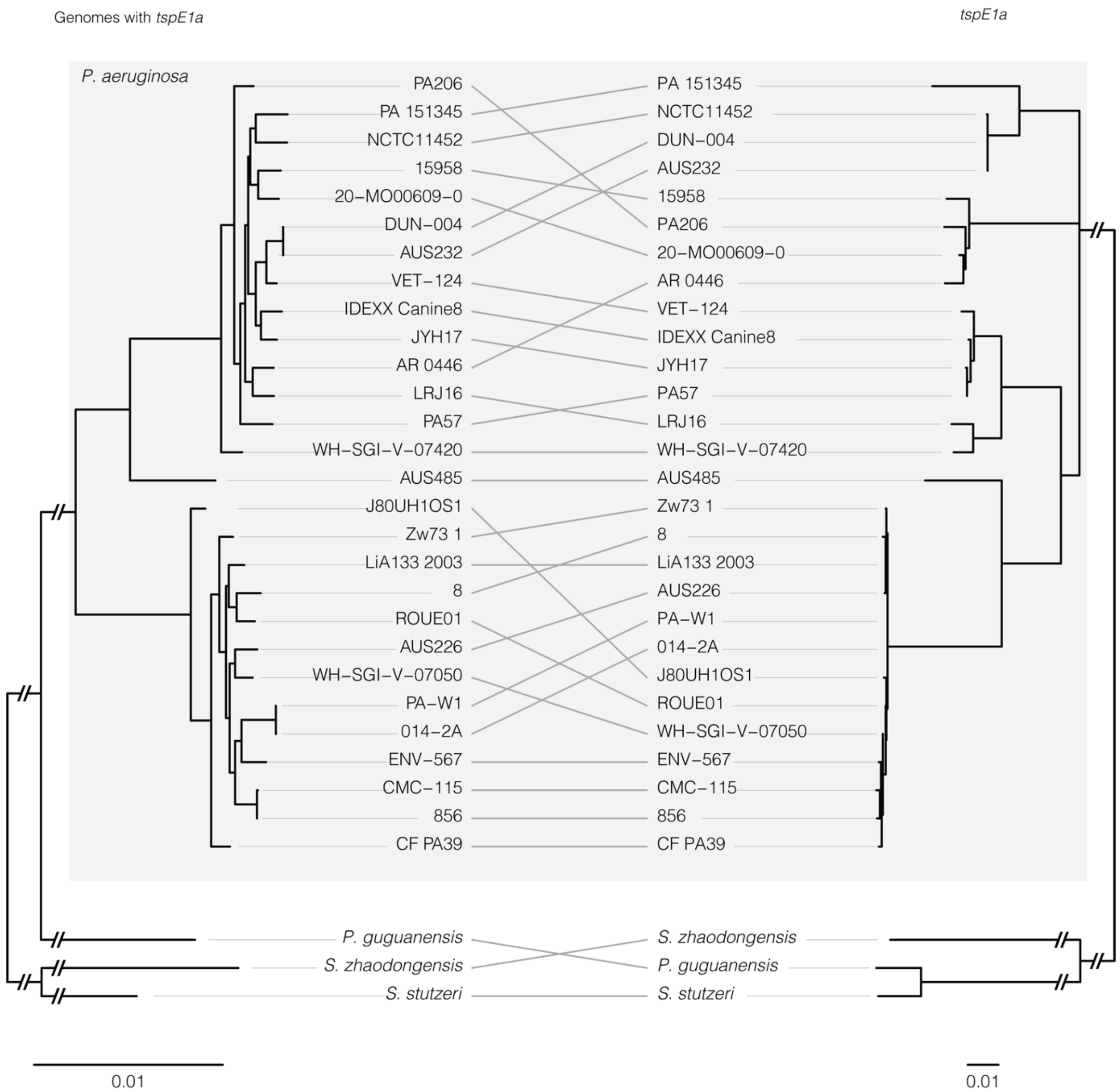

Same co-phylogenetic plot as on previous page, but with cut branches. The species tree is on the left and the *tspE1a* tree on the right. The species tree is a maximum-likelihood tree inferred with the HKY+F+I model. The gene tree is a maximum-likelihood tree inferred with the TPM2+F+G4 model. Both trees are midpoint rooted and their distances are shown in substitutions per site.

tspE1b

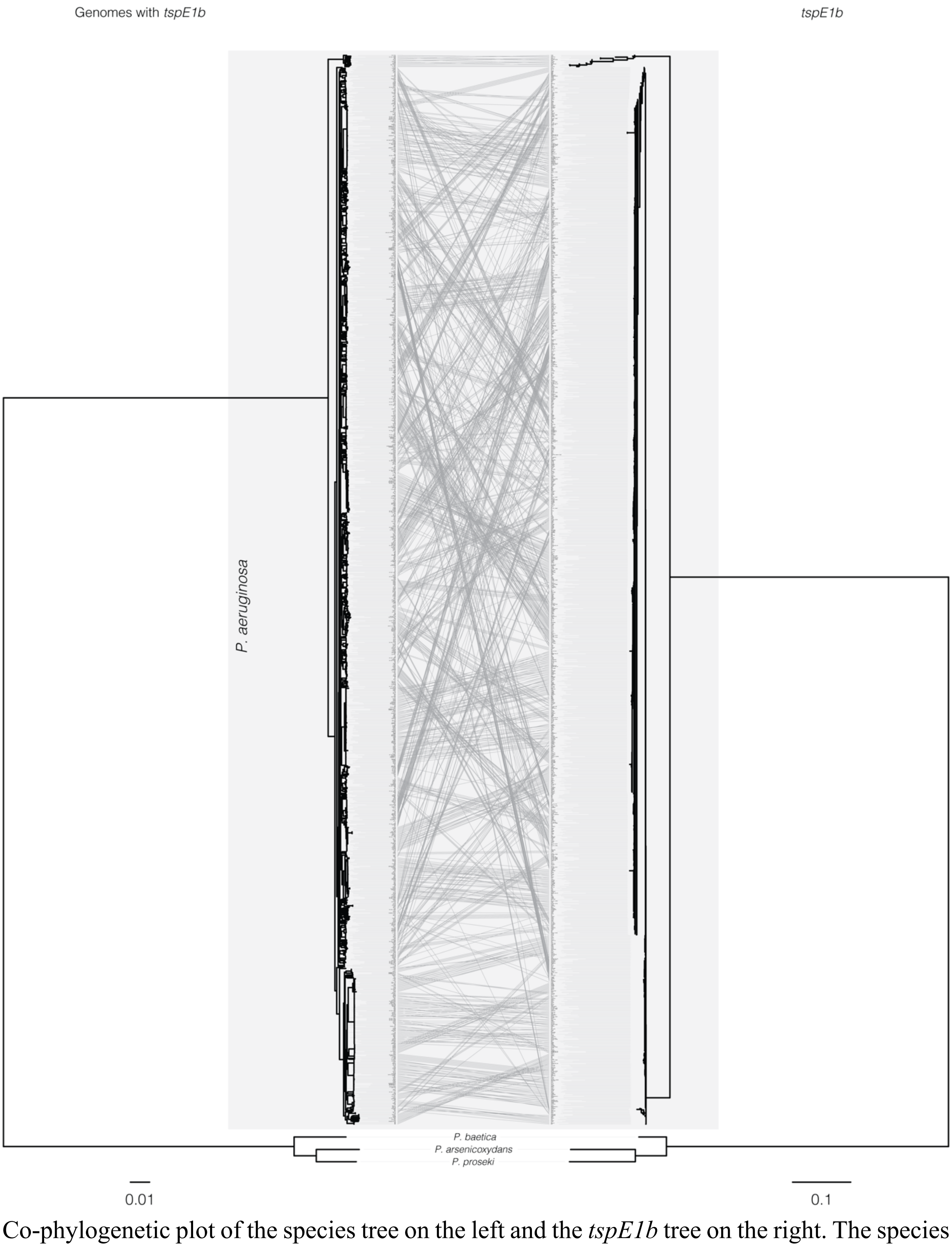

Co-phylogenetic plot of the species tree on the left and the *tspE1b* tree on the right. The species tree is a maximum-likelihood tree inferred with the HKY+F+I model. The gene tree is a maximum-likelihood tree inferred with the GTR+F+R6 model. Both trees are midpoint rooted and their distances are shown in substitutions per site.

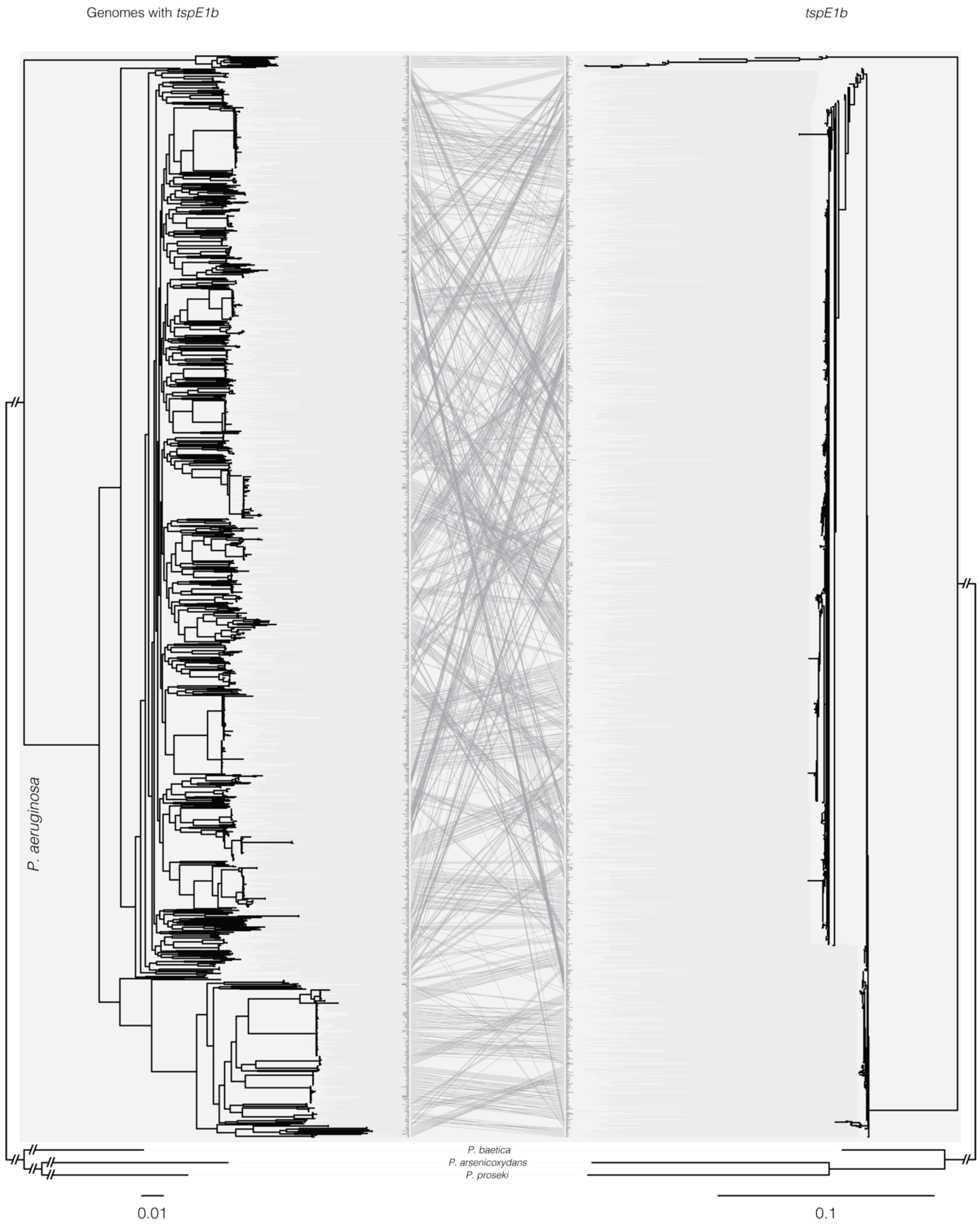

Same co-phylogenetic plot as on previous page, but with cut branches. The species tree is on the left and the *tspE1b* tree on the right. The species tree is a maximum-likelihood tree inferred with the HKY+F+I model. The gene tree is a maximum-likelihood tree inferred with the GTR+F+R6 model. Both trees are midpoint rooted and their distances are shown in substitutions per site.

tspE1c

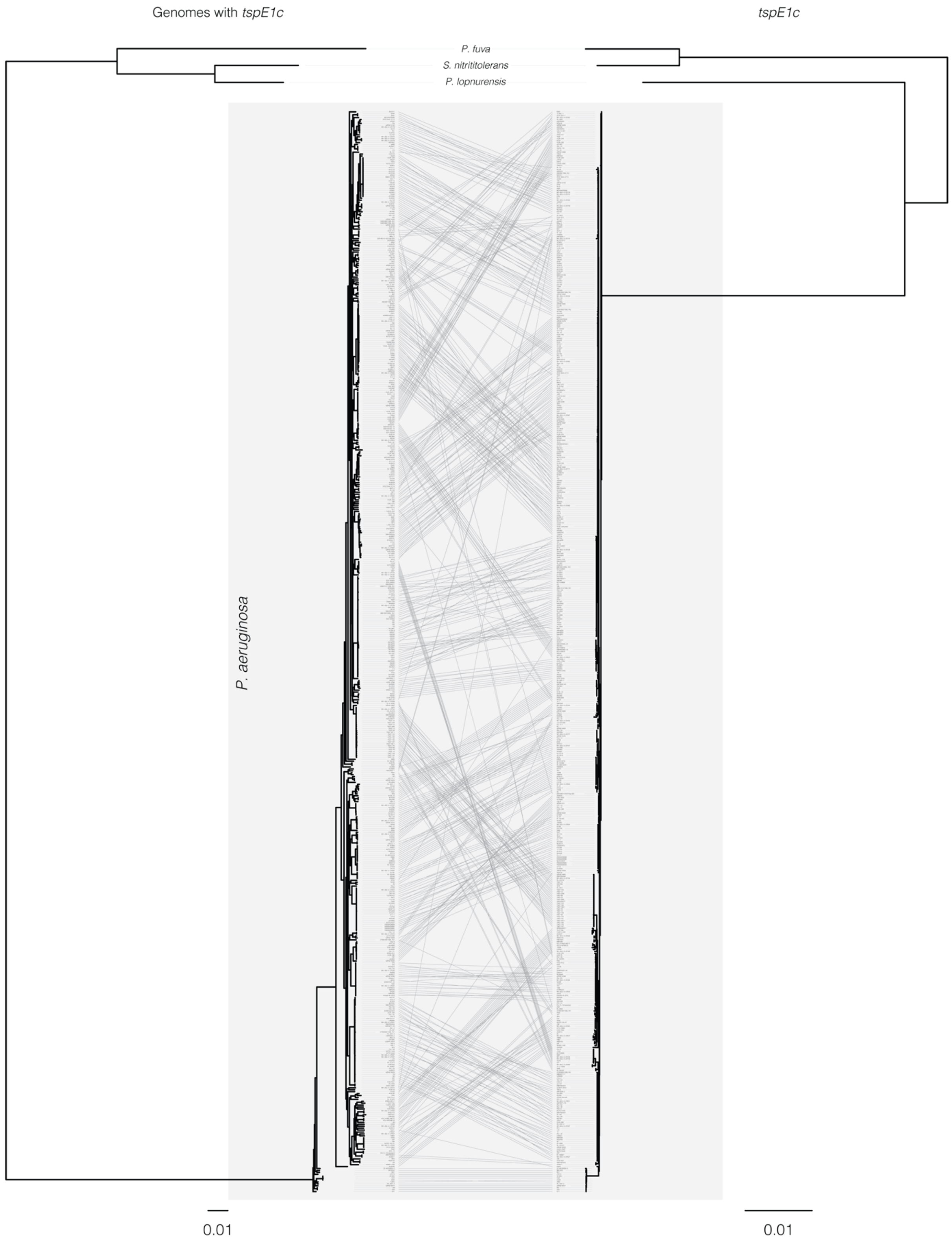

Co-phylogenetic plot of the species tree on the left and the *tspE1c* tree on the right. The species tree is a maximum-likelihood tree inferred with the HKY+F+I model. The gene tree is a maximum-likelihood tree inferred with the TN+F+R6 model. Both trees are midpoint rooted and their distances are shown in substitutions per site.

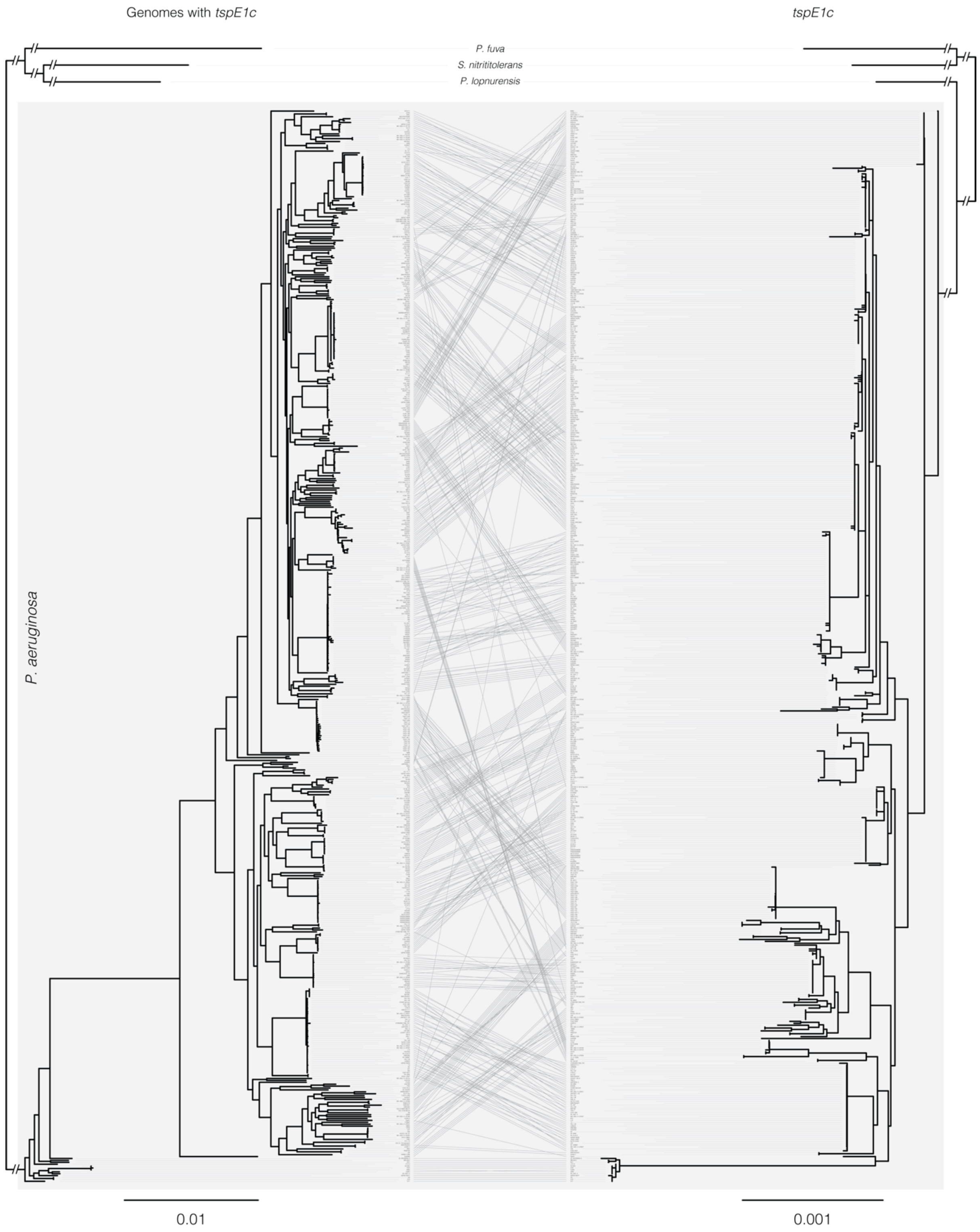

Same co-phylogenetic plot as on previous page, but with cut branches. The species tree is on the left and the *tspE1c* tree on the right. The species tree is a maximum-likelihood tree inferred with the HKY+F+I model. The gene tree is a maximum-likelihood tree inferred with the TN+F+R6 model. Both trees are midpoint rooted and their distances are shown in substitutions per site.

tepB

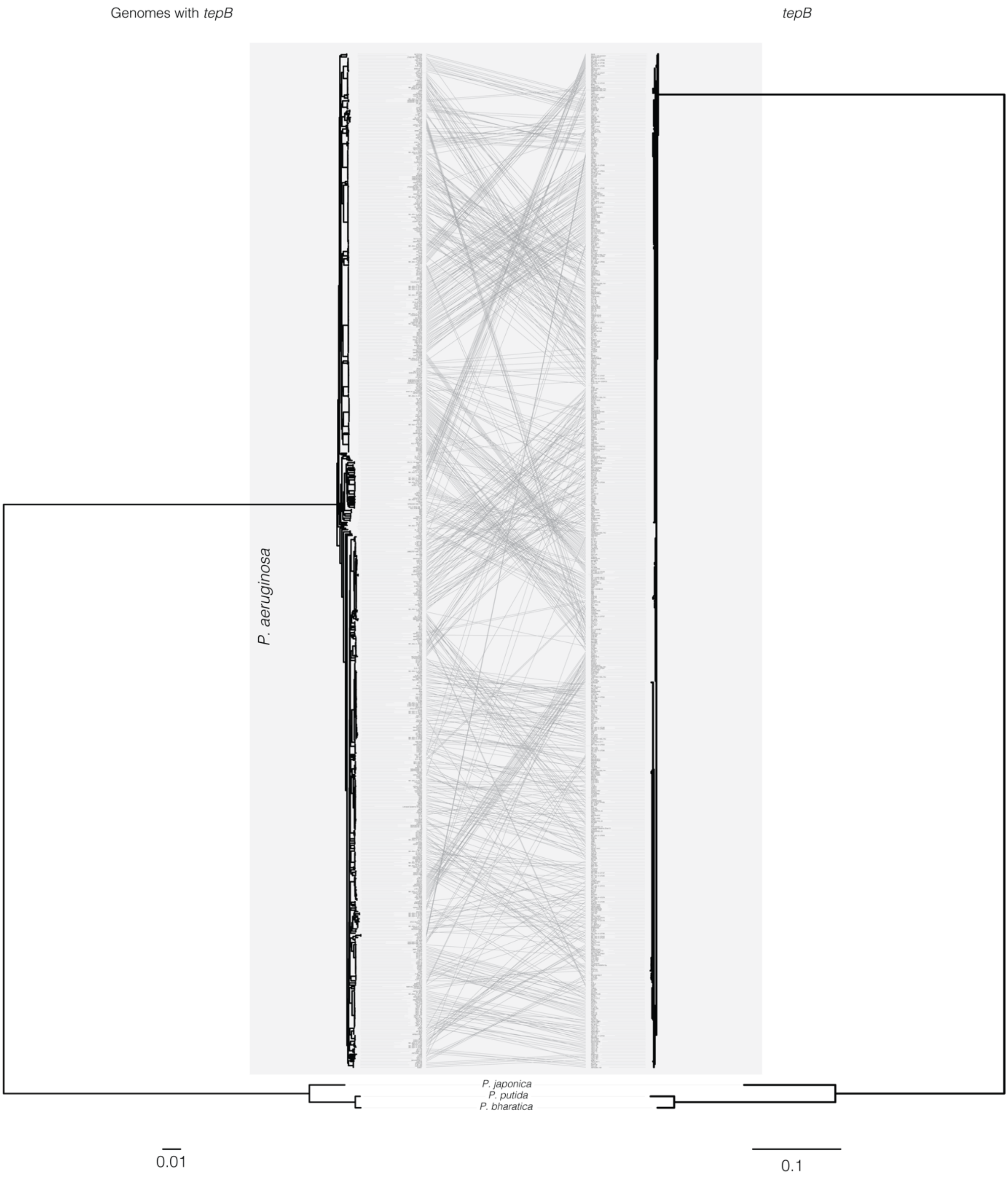

Co-phylogenetic plot of the species tree on the left and the *tepB* tree on the right. The species tree is a maximum-likelihood tree inferred with the HKY+F+I model. The gene tree is a maximum-likelihood tree inferred with the TPM2+F+G4 model. Both trees are midpoint rooted and their distances are shown in substitutions per site.

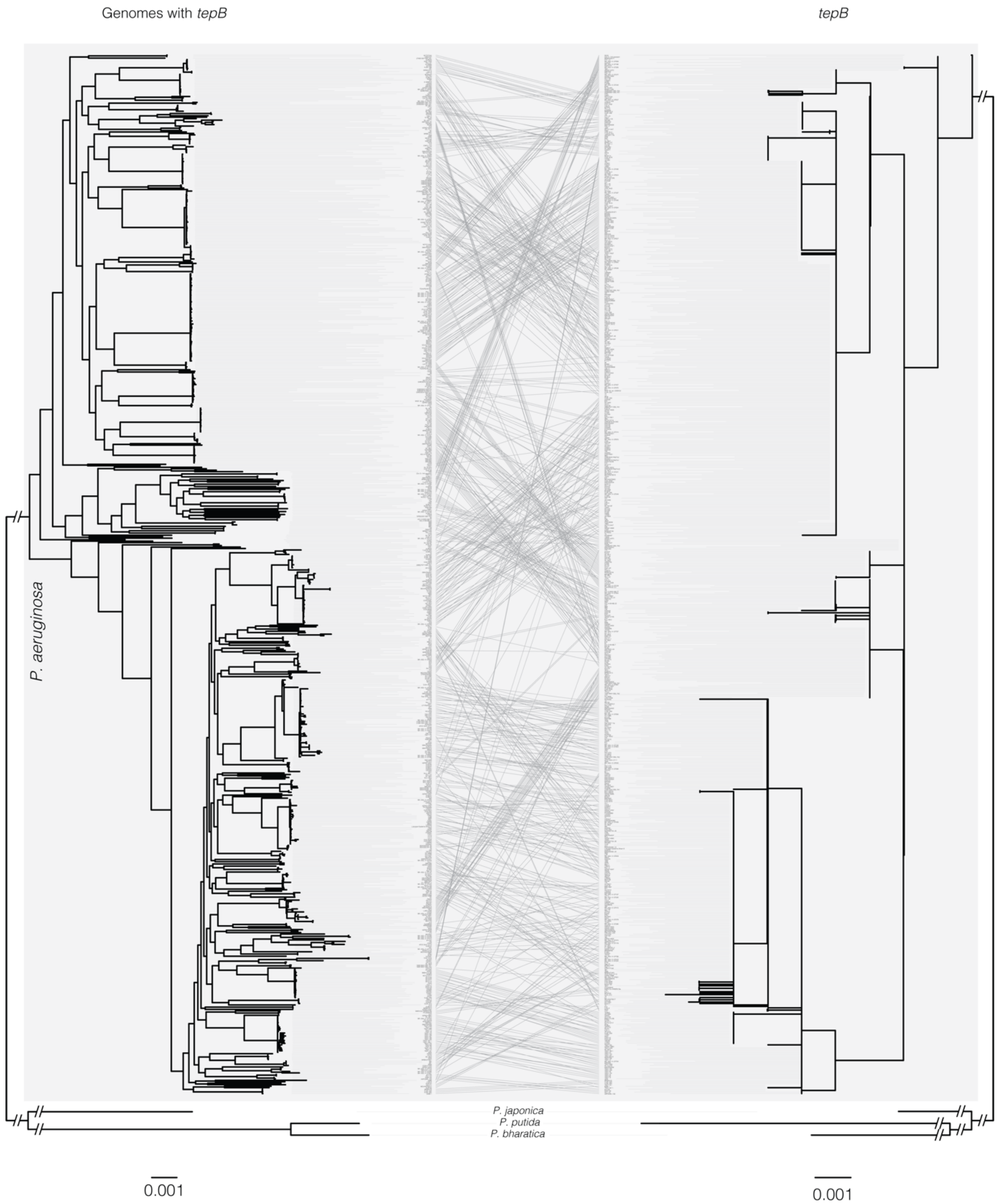

Same co-phylogenetic plot as on previous page, but with cut branches. The species tree is on the left and the *tepB* tree on the right. The species tree is a maximum-likelihood tree inferred with the HKY+F+I model. The gene tree is a maximum-likelihood tree inferred with the TPM2+F+G4 model. Both trees are midpoint rooted and their distances are shown in substitutions per site.

tepBa

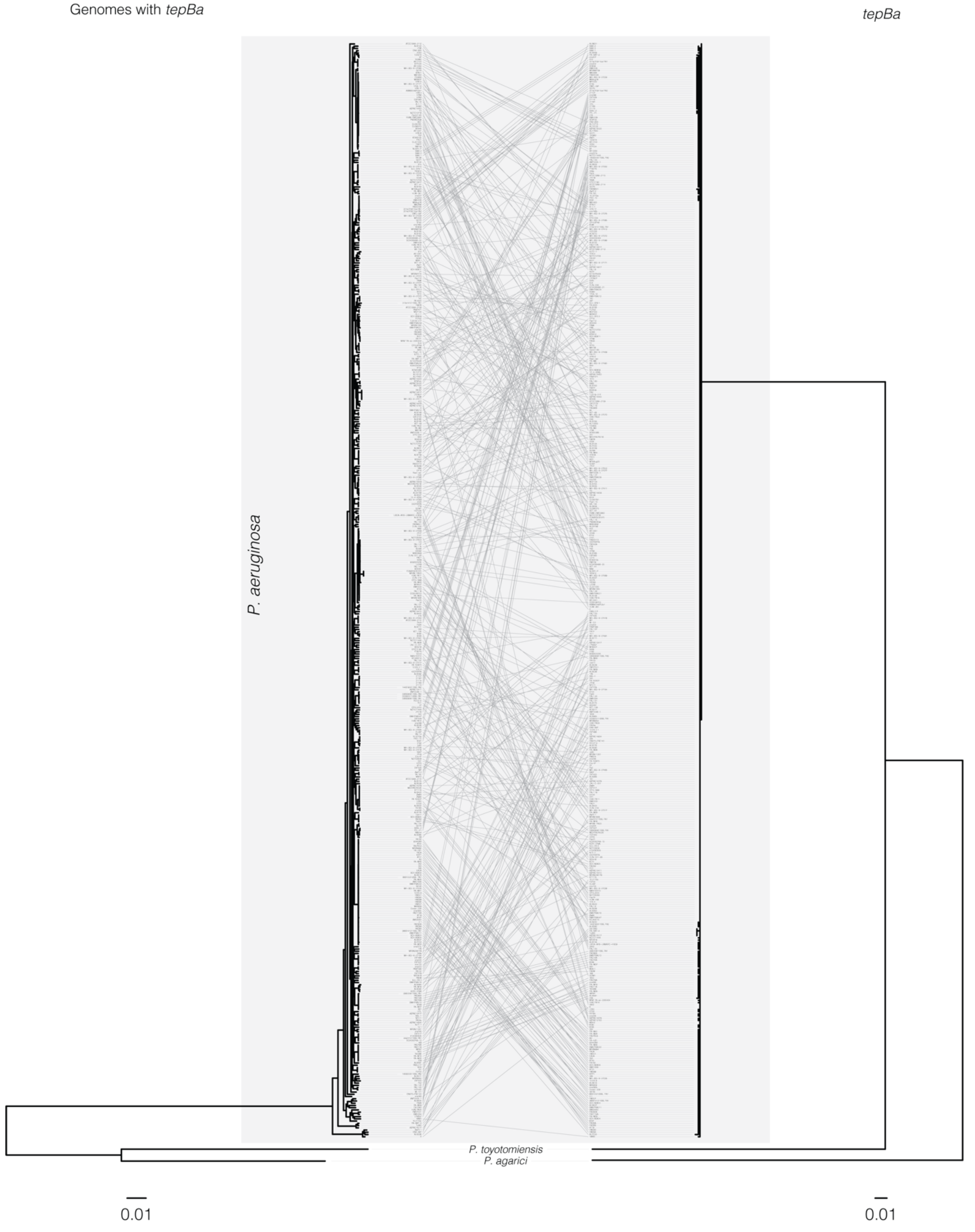

Co-phylogenetic plot of the species tree on the left and the *tepBa* tree on the right. The species tree is a maximum-likelihood tree inferred with the HKY+F+I model. The gene tree is a maximum-likelihood tree inferred with the TN+F+G4 model. Both trees are midpoint rooted and their distances are shown in substitutions per site.

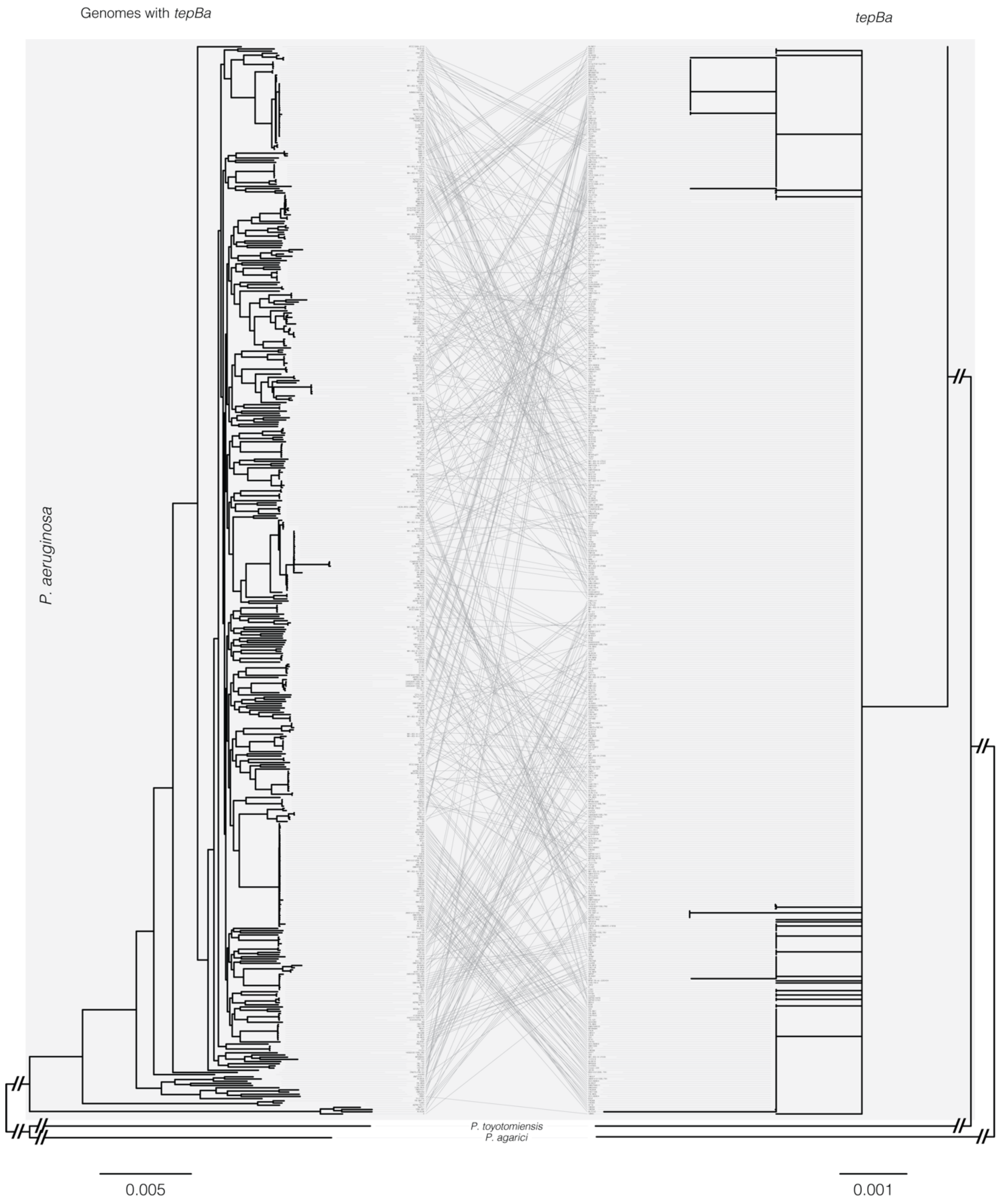

Same co-phylogenetic plot as on previous page, but with cut branches. The species tree is on the left and the *tepBa* tree on the right. The species tree is a maximum-likelihood tree inferred with the HKY+F+I model. The gene tree is a maximum-likelihood tree inferred with the TN+F+G4 model. Both trees are midpoint rooted and their distances are shown in substitutions per site.

tepBb

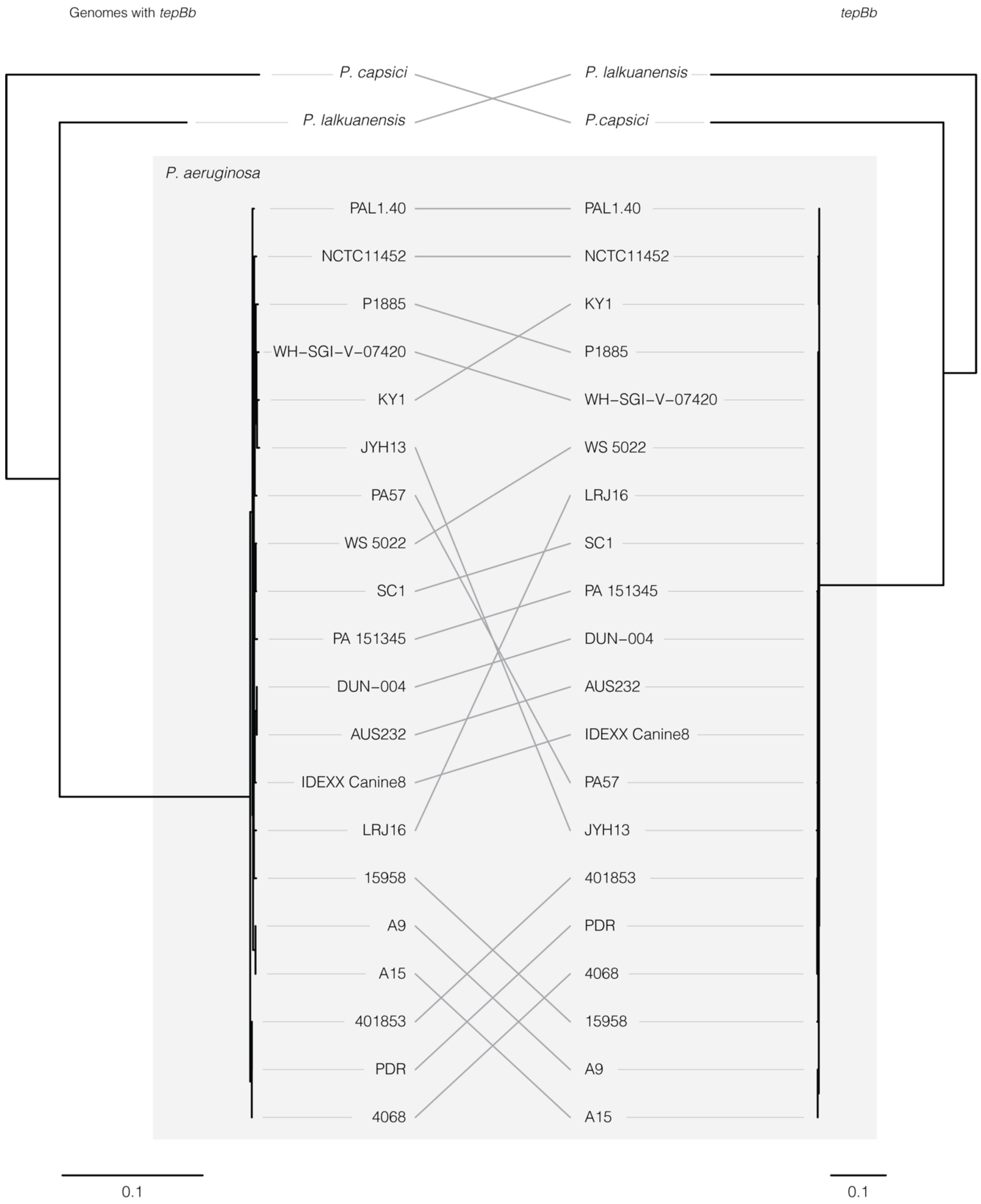

Co-phylogenetic plot of the species tree on the left and the *tepBb* tree on the right. The species tree is a maximum-likelihood tree inferred with the HKY+F+I model. The gene tree is a maximum-likelihood tree inferred with the TVMe+I model. Both trees are midpoint rooted and their distances are shown in substitutions per site.

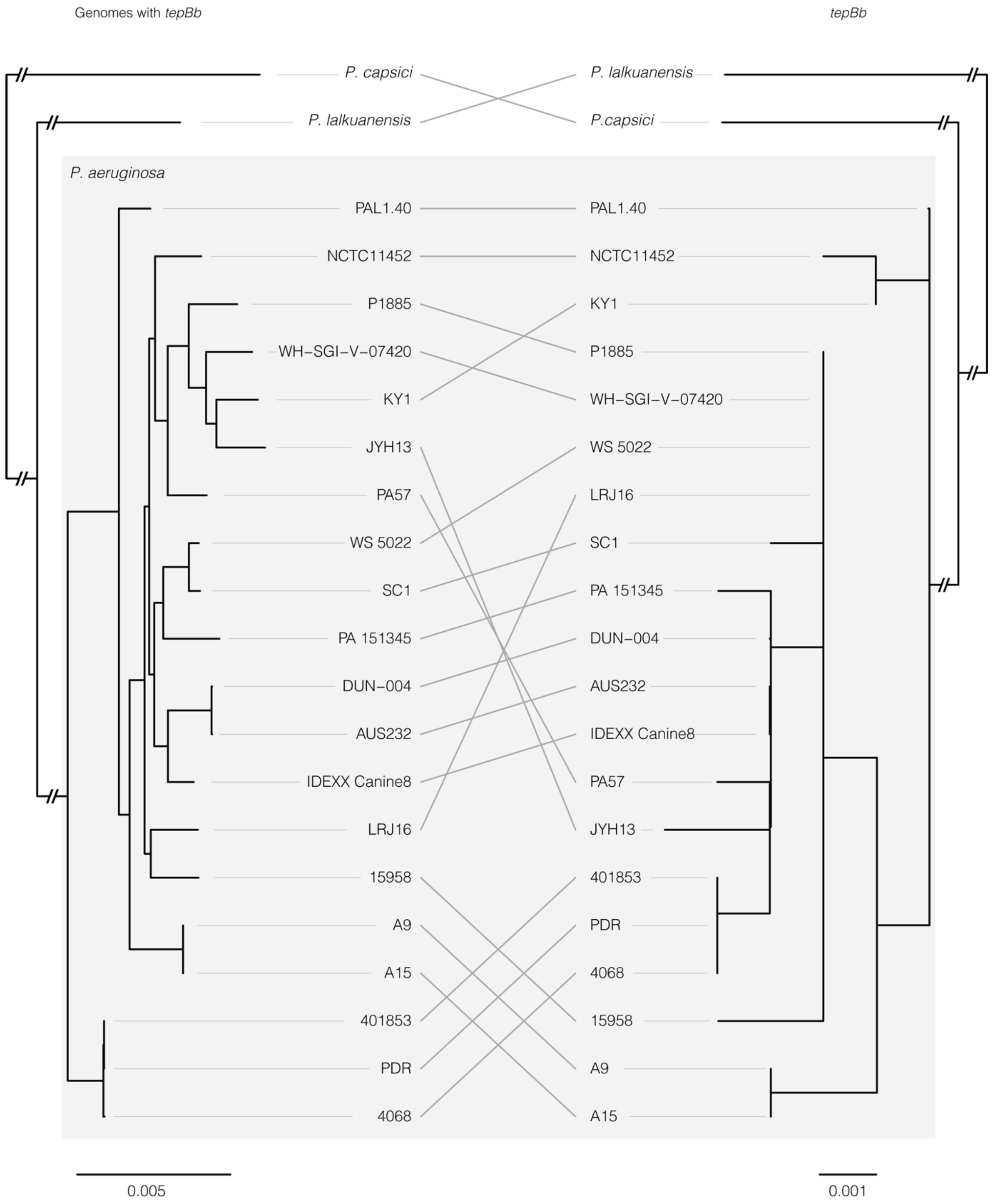

Same co-phylogenetic plot as on previous page, but with cut branches. The species tree is on the left and the *tepBb* tree on the right. The species tree is a maximum-likelihood tree inferred with the HKY+F+I model. The gene tree is a maximum-likelihood tree inferred with the TVMe+I model. Both trees are midpoint rooted and their distances are shown in substitutions per site.
