# Supplementary material for: Distribution of the four type VI secretion systems in Pseudomonas aeruginosa and classification of their core and accessory effectors
Source: Nat Commun. 2025 Jan 21;16:888. doi: 10.1038/s41467-024-54649-5 (PMC11751169; doi:10.1038/s41467-024-54649-5)
Supplement: Supplementary file 23 — Supplementary Dataset 21 [file 41467_2024_54649_MOESM23_ESM.pdf]

Distribution of the four type VI secretion systems in *Pseudomonas aeruginosa* and classification of their core and accessory effectors

Supplementary Data 21

This file contains circos plots of accessory effectors. The plots are ordered by the effector genes' associated T6SS and are presented in the following order:

H1-T6SS effectors: *tse6, tas1, tne3, tse7, tse7a, tsd1, tse7c, tse7d, tse7e*

H2-T6SS effectors: *tle3, tle4b, vgrG2b, tseV, pldA, tle2, tspE1a, tspE1b, tspE1c*

H3-T6SS effectors: *tepB, tepBa, tepBb*

The effector gene is indicated on the top left corner of each page. Purple lines connect strains that have a particular effector gene. Data on the occurrence of effector genes was used as input (Supplementary Data 10). Mean pairwise nucleotide identities between effector genes that are connected with purple lines are provided in Supplementary Table 10. The phylogenetic tree is based on a core genome alignment of *P. aeruginosa* strains (n=1912) and computed using the HKY+F+I model. The tree is midpoint rooted and distances are shown in substitutions per site.

tse6

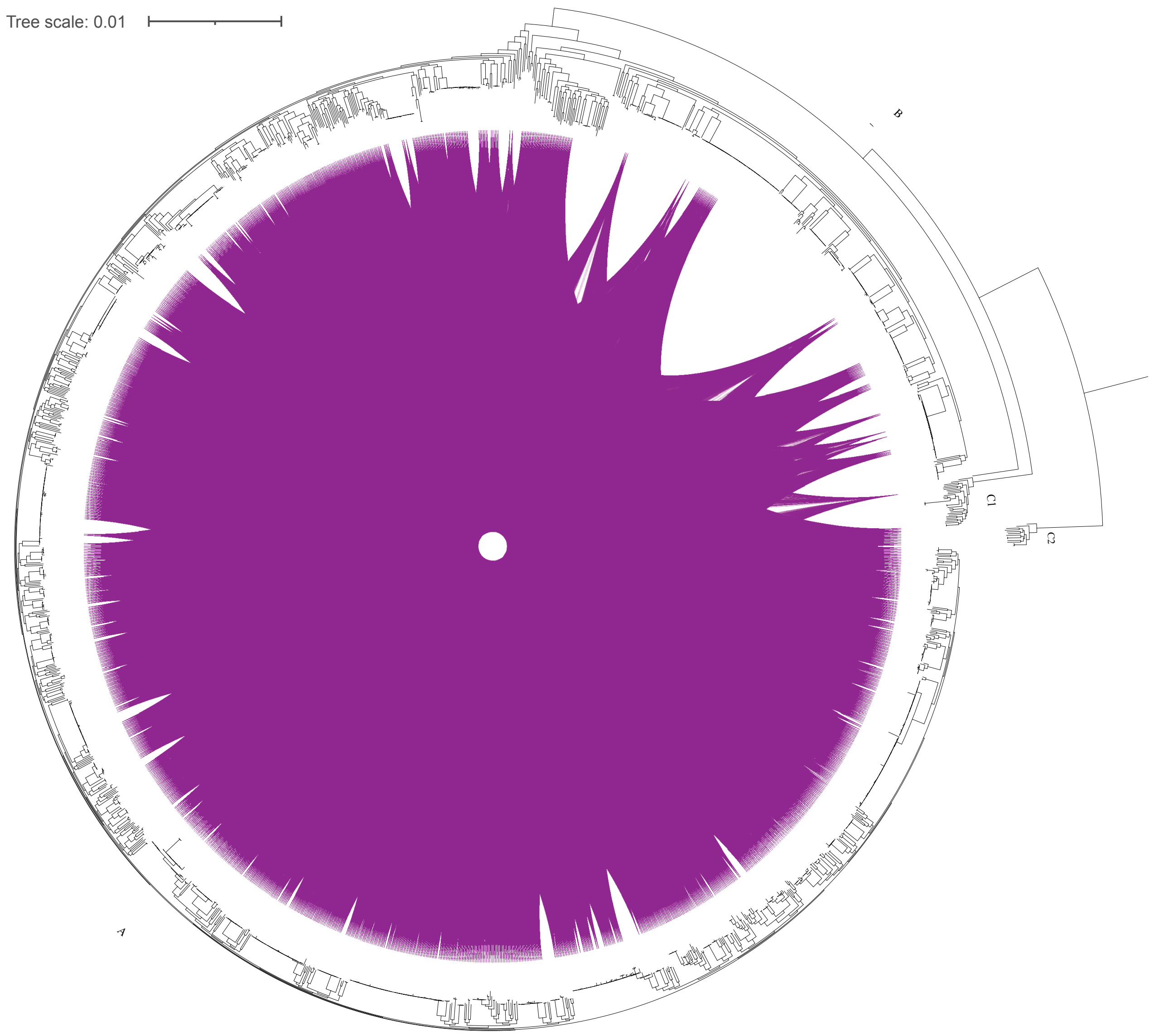

tas1

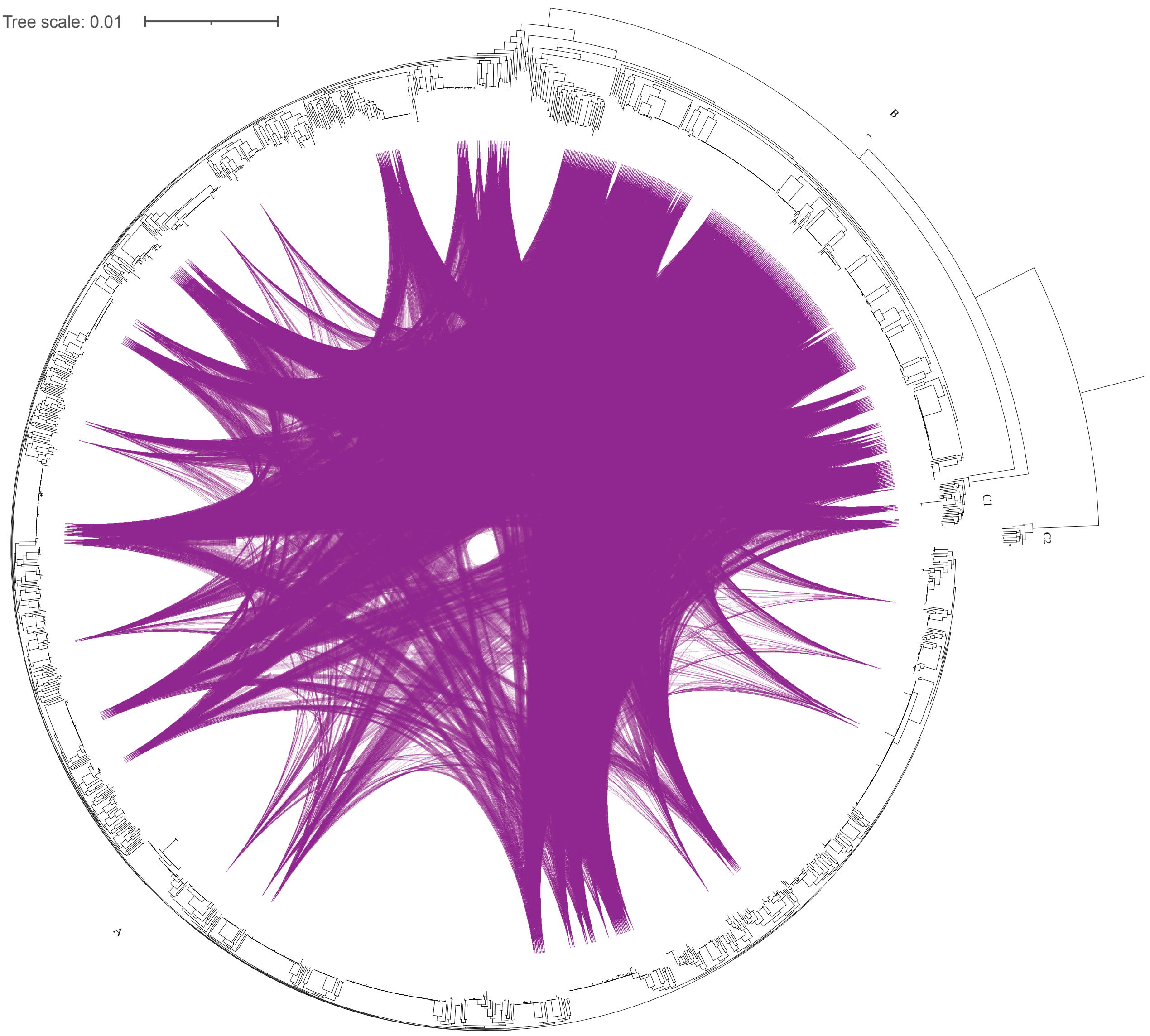

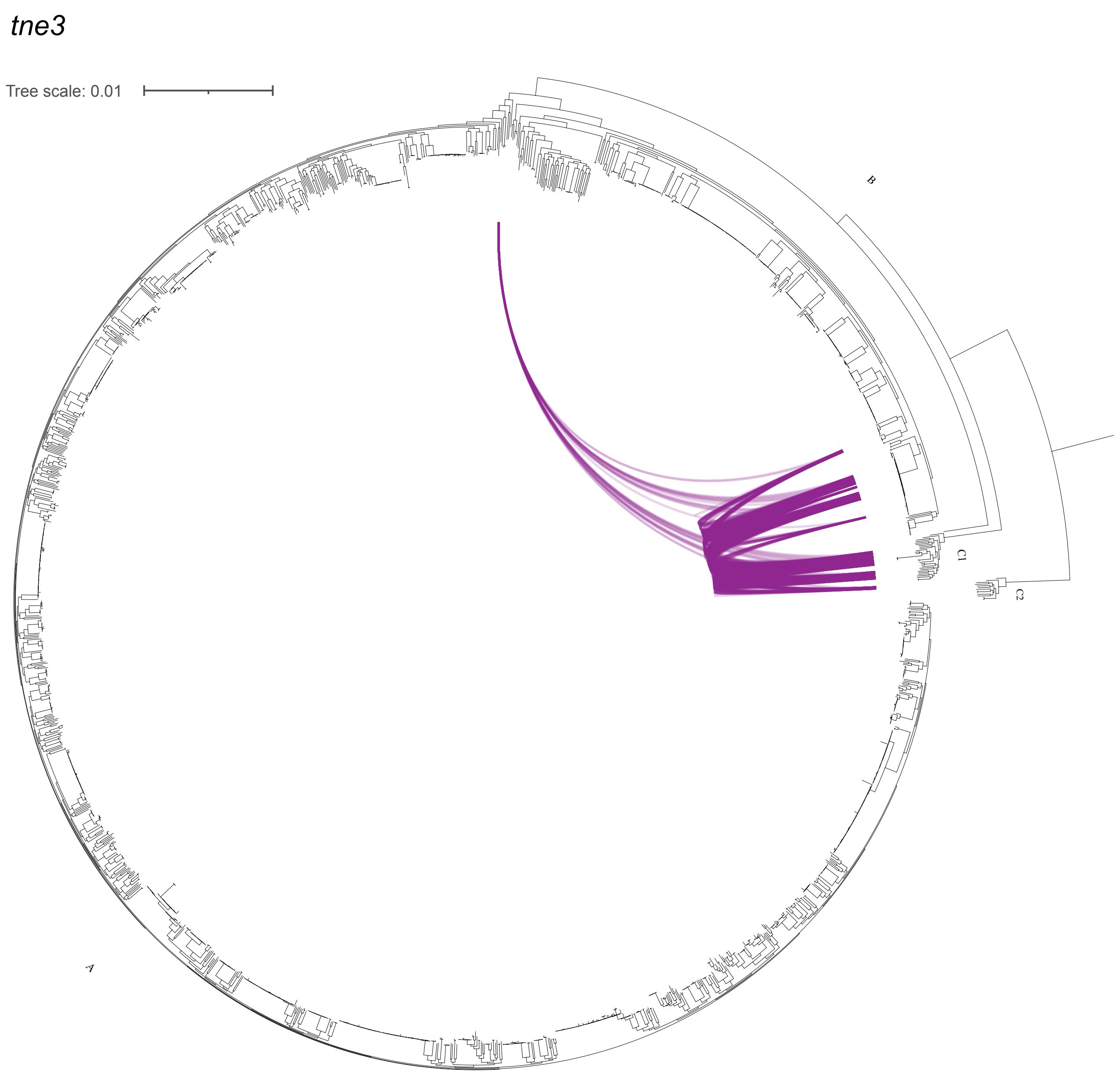
tne3
Tree scale: 0.01
B
C1
C2
A

tse7

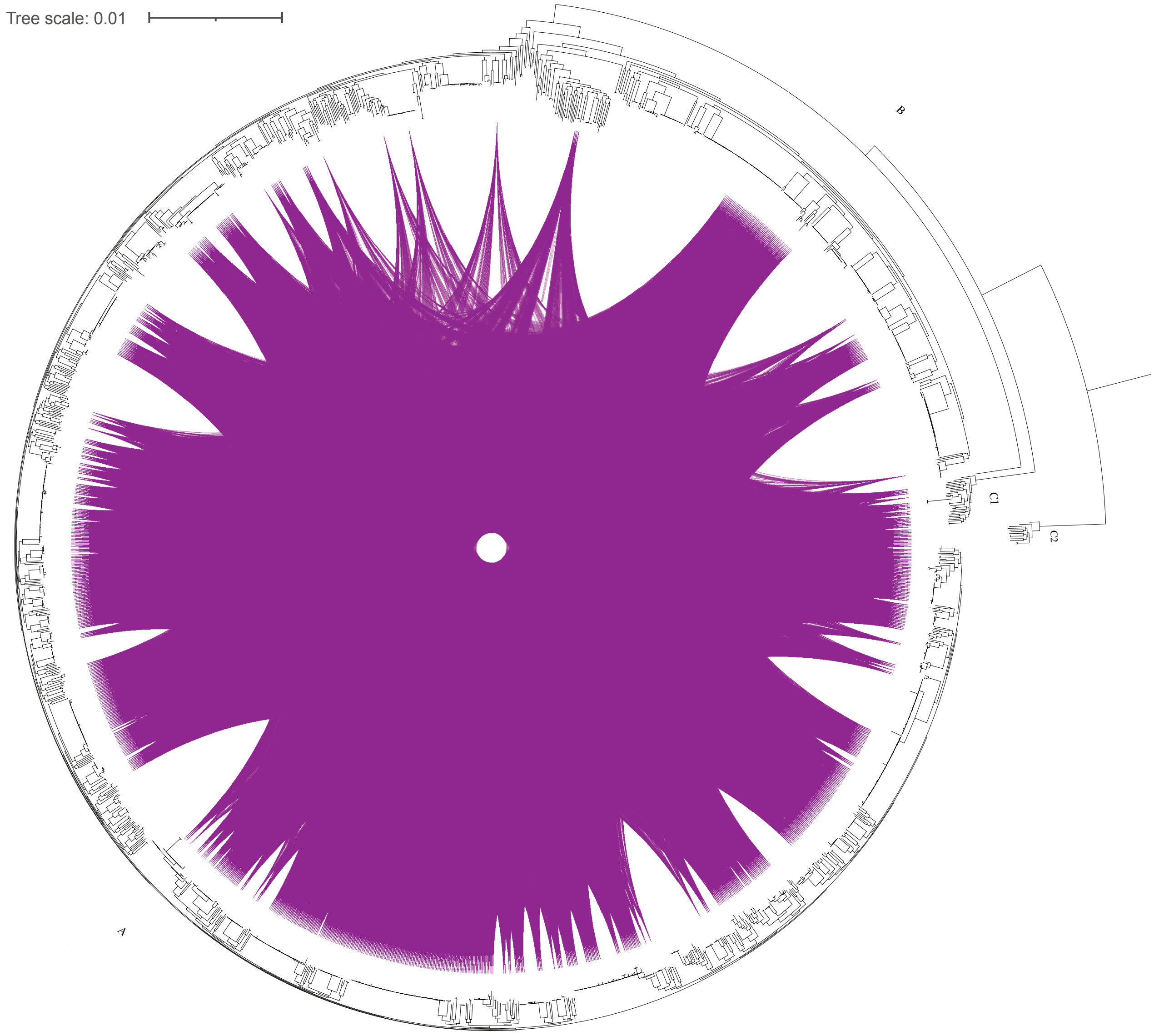

tse7a

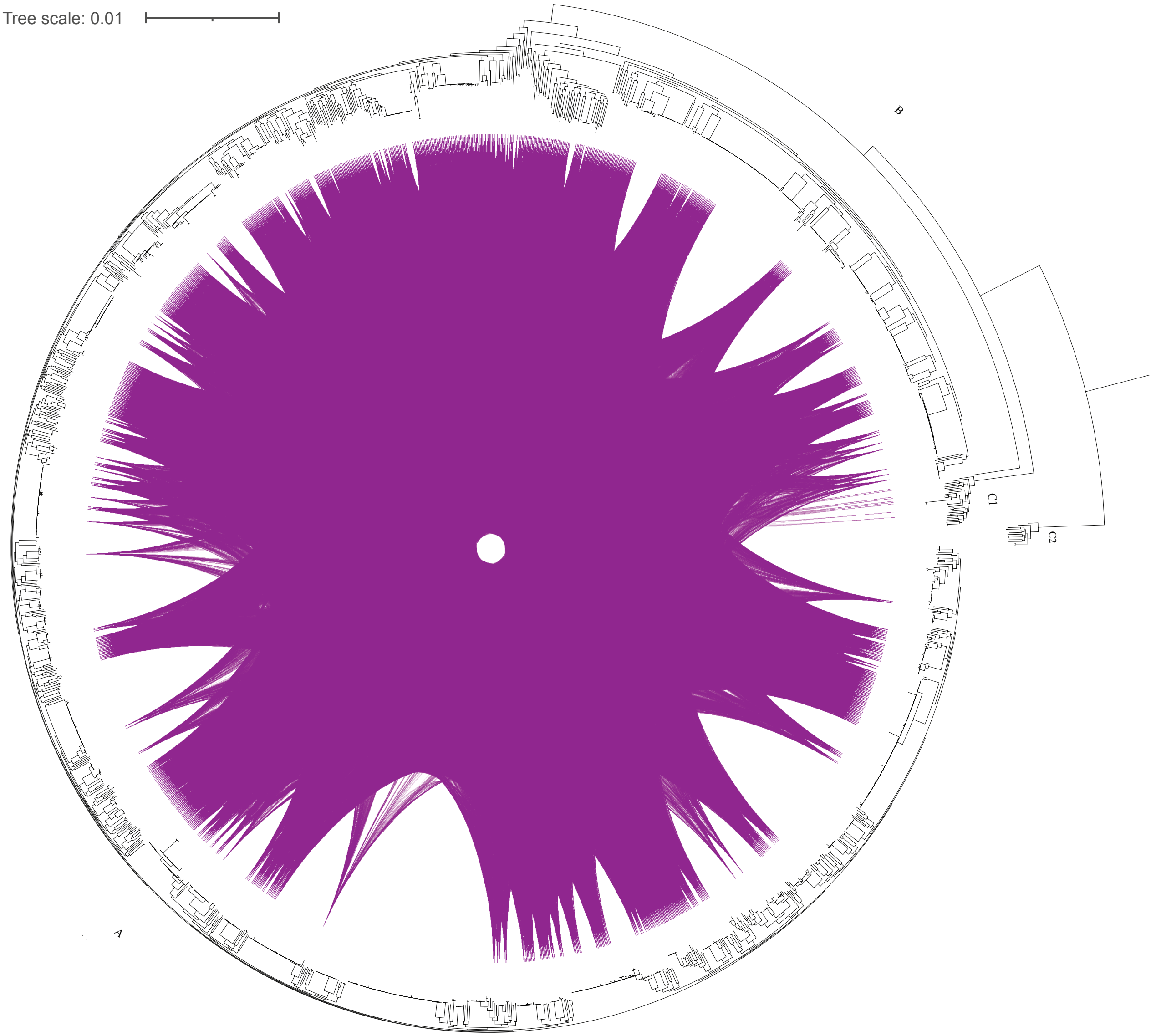

tsd1

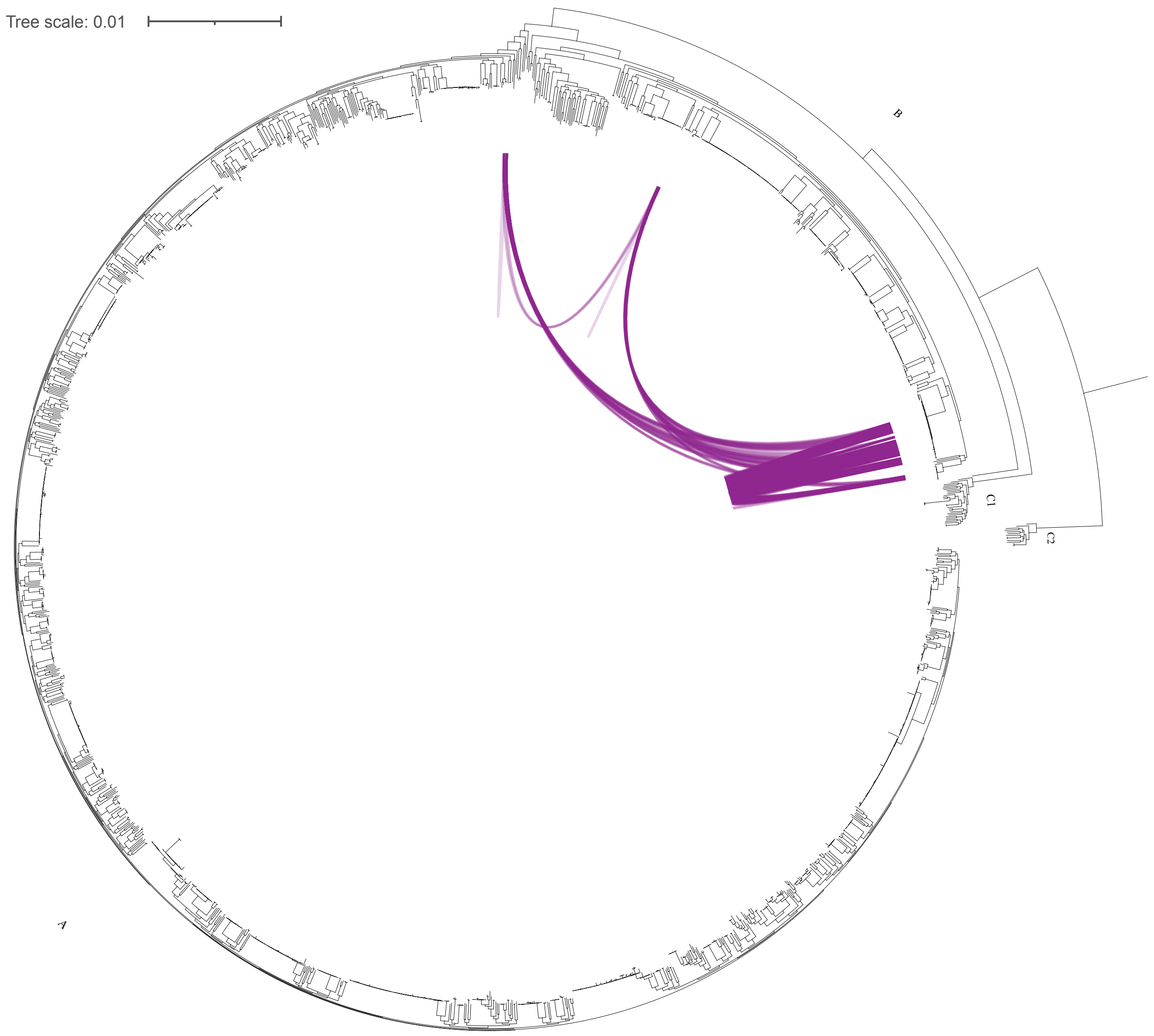

tse7c

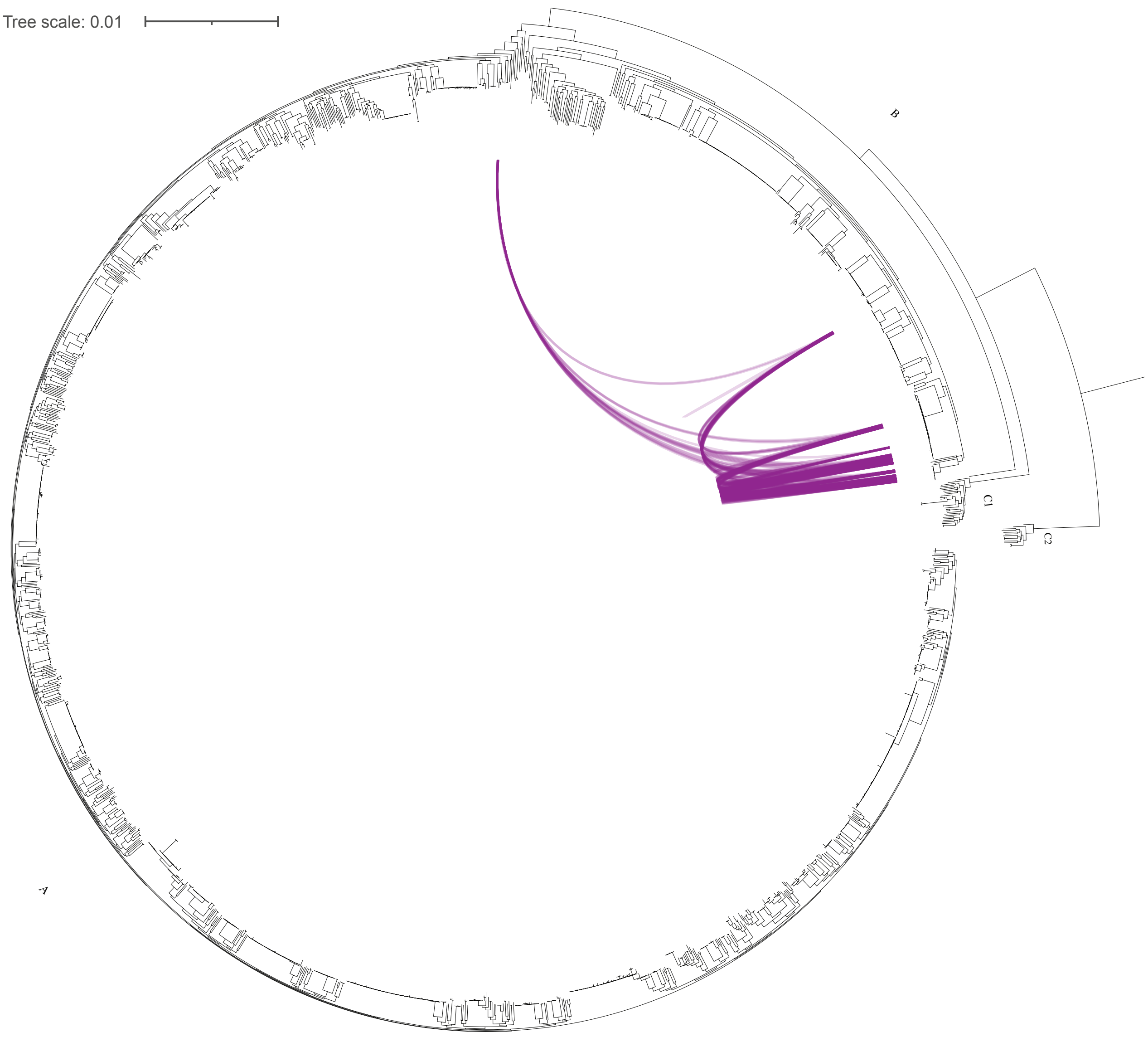

tse7d

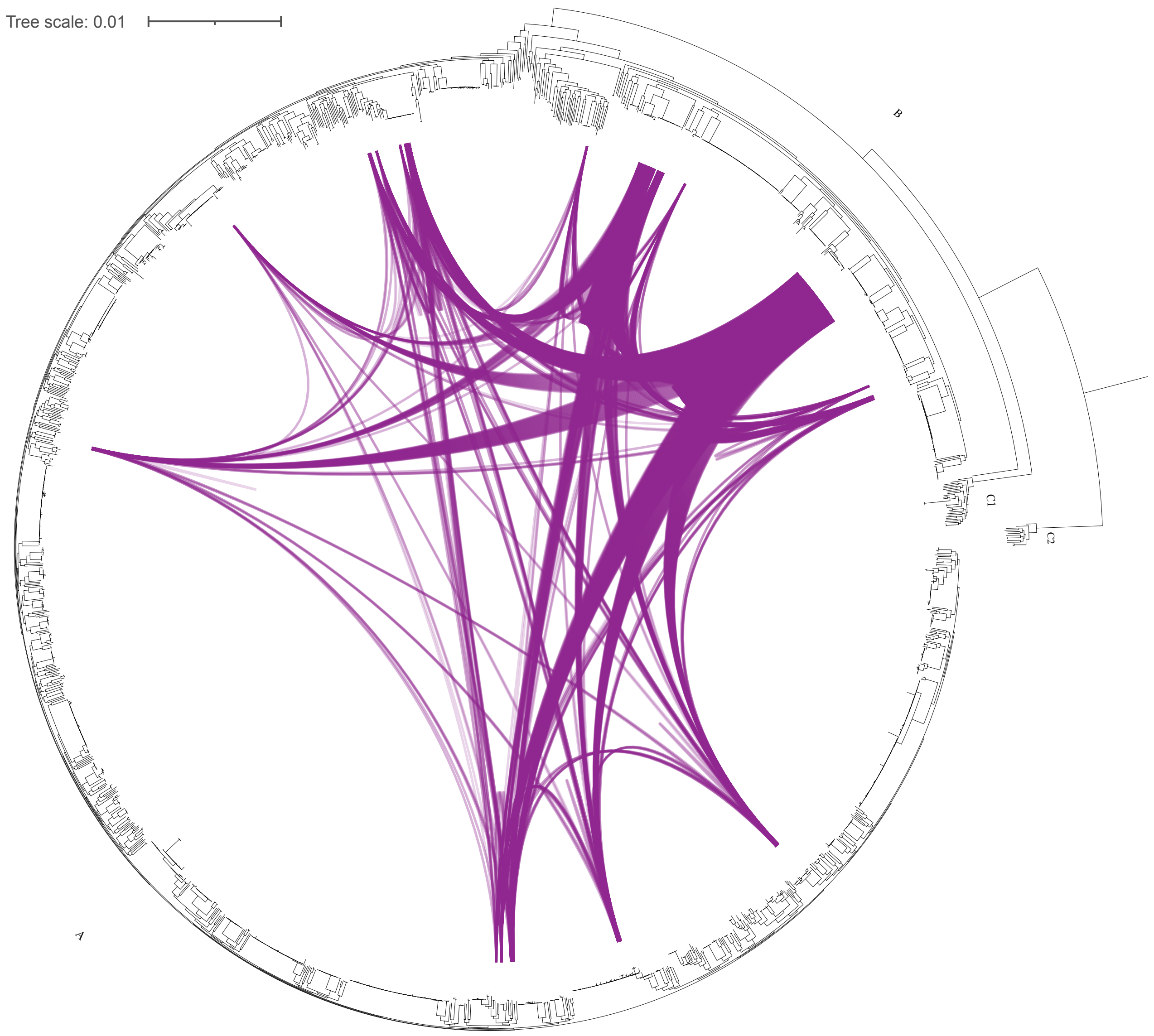

tse7e

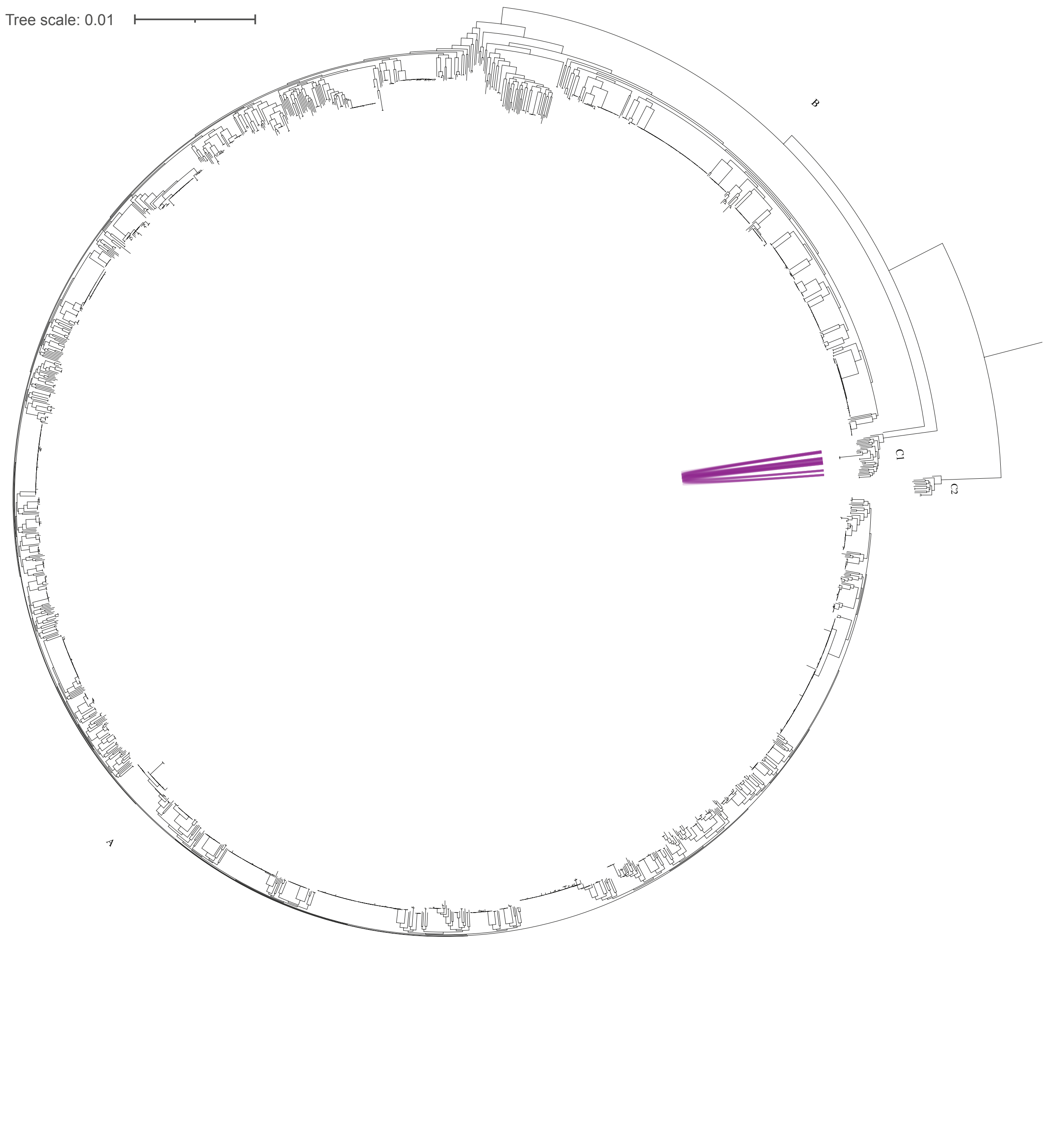

tle3

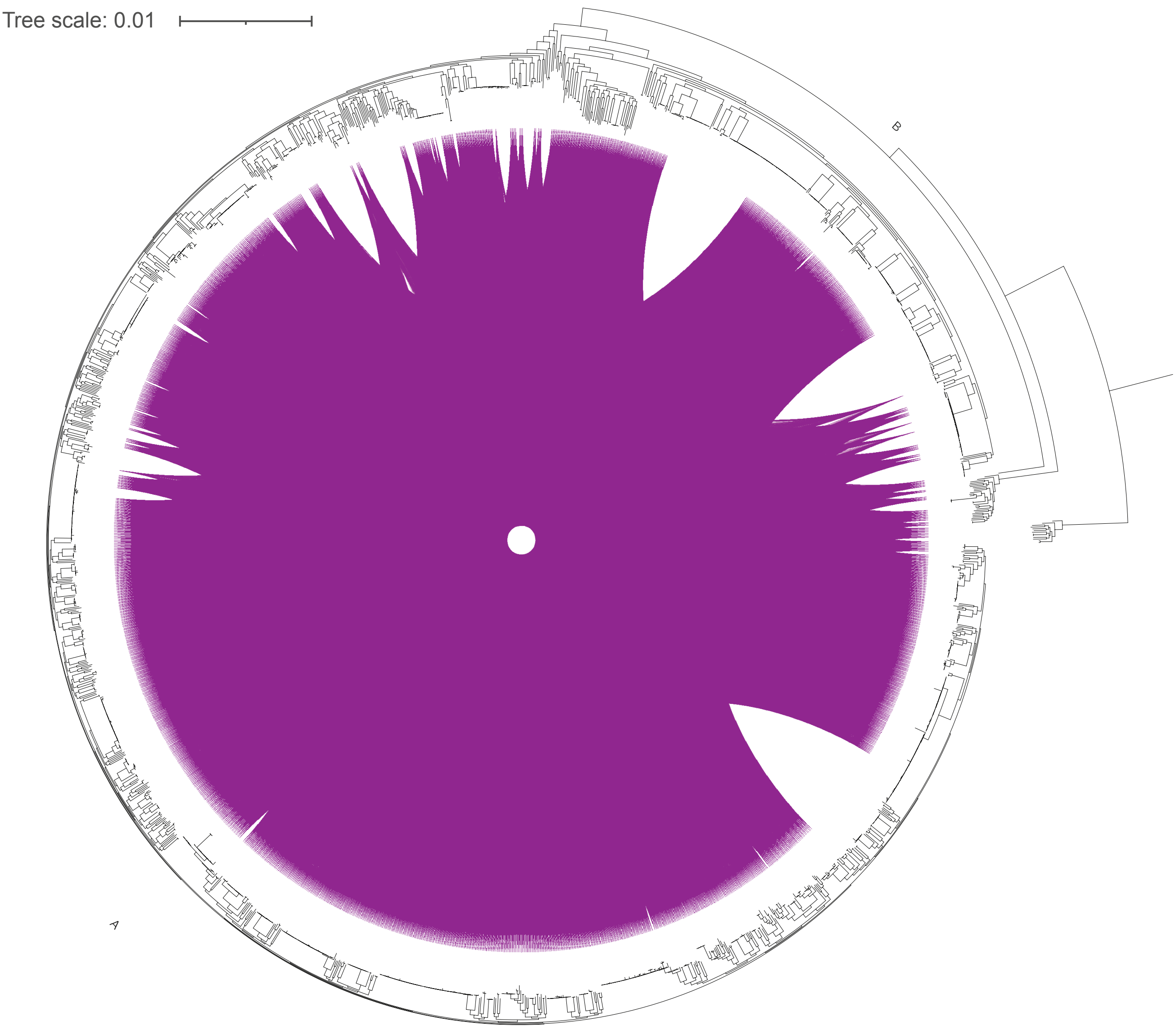

tle4b

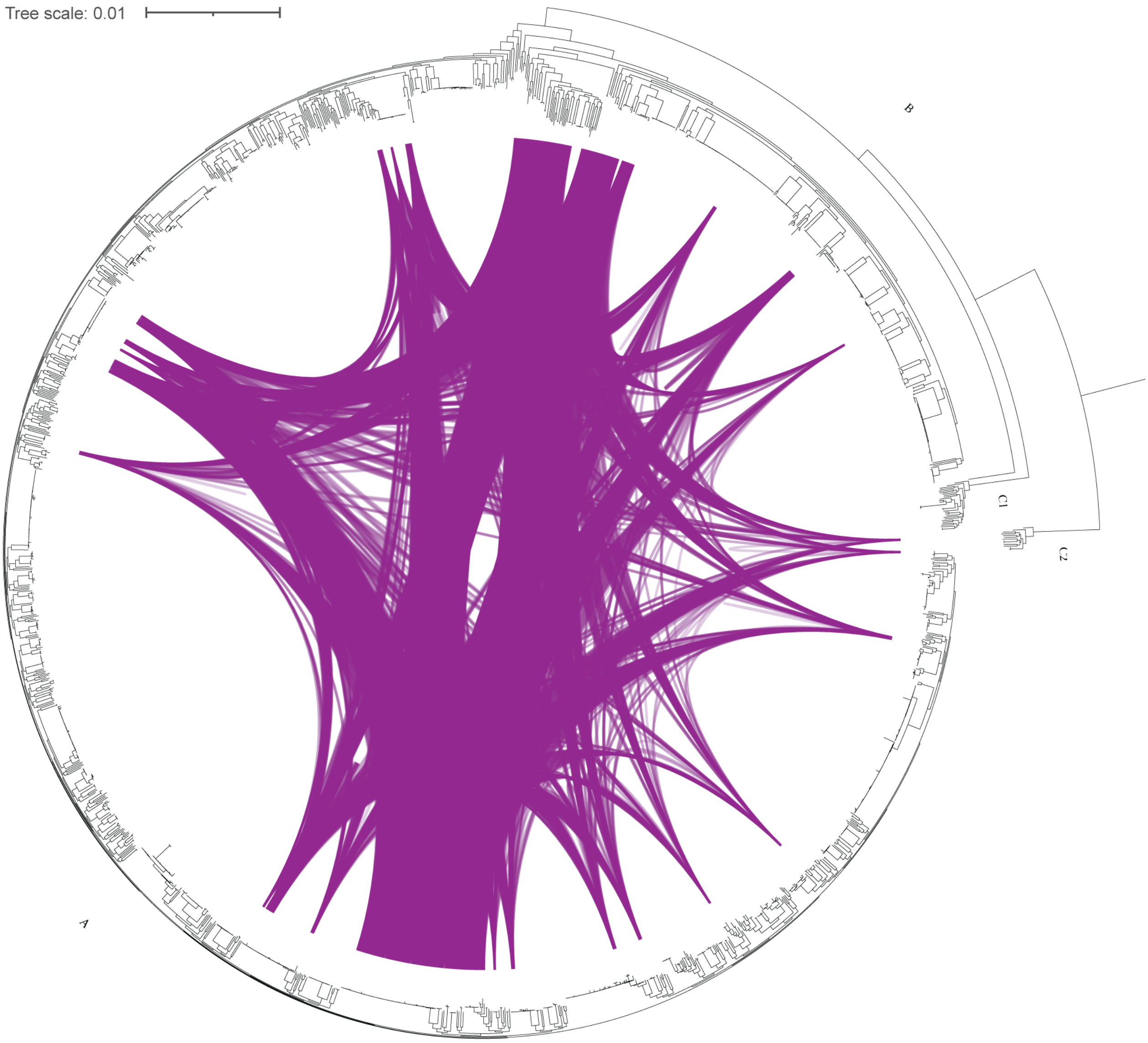

vgrG2b

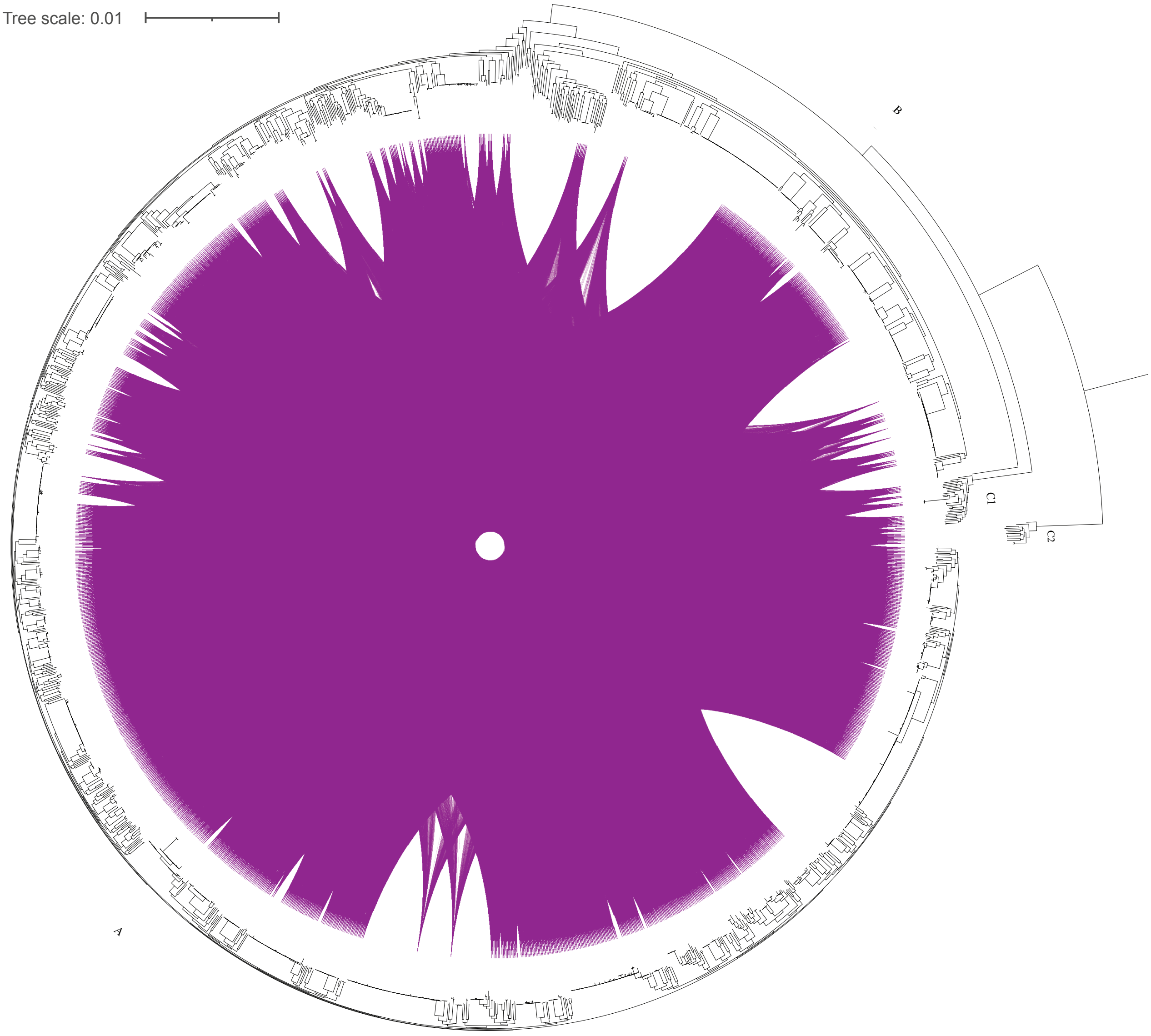

tseV

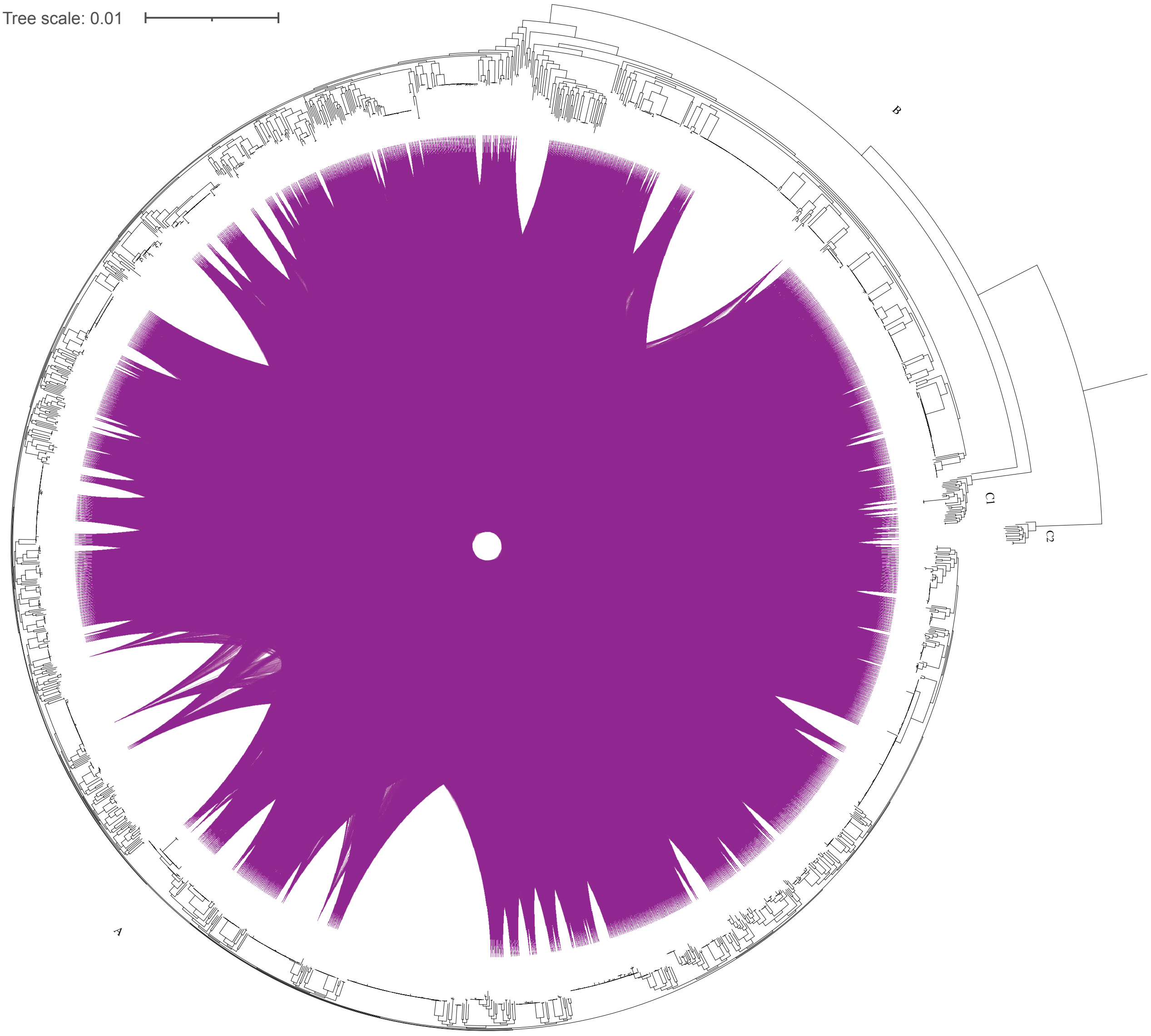

pldA

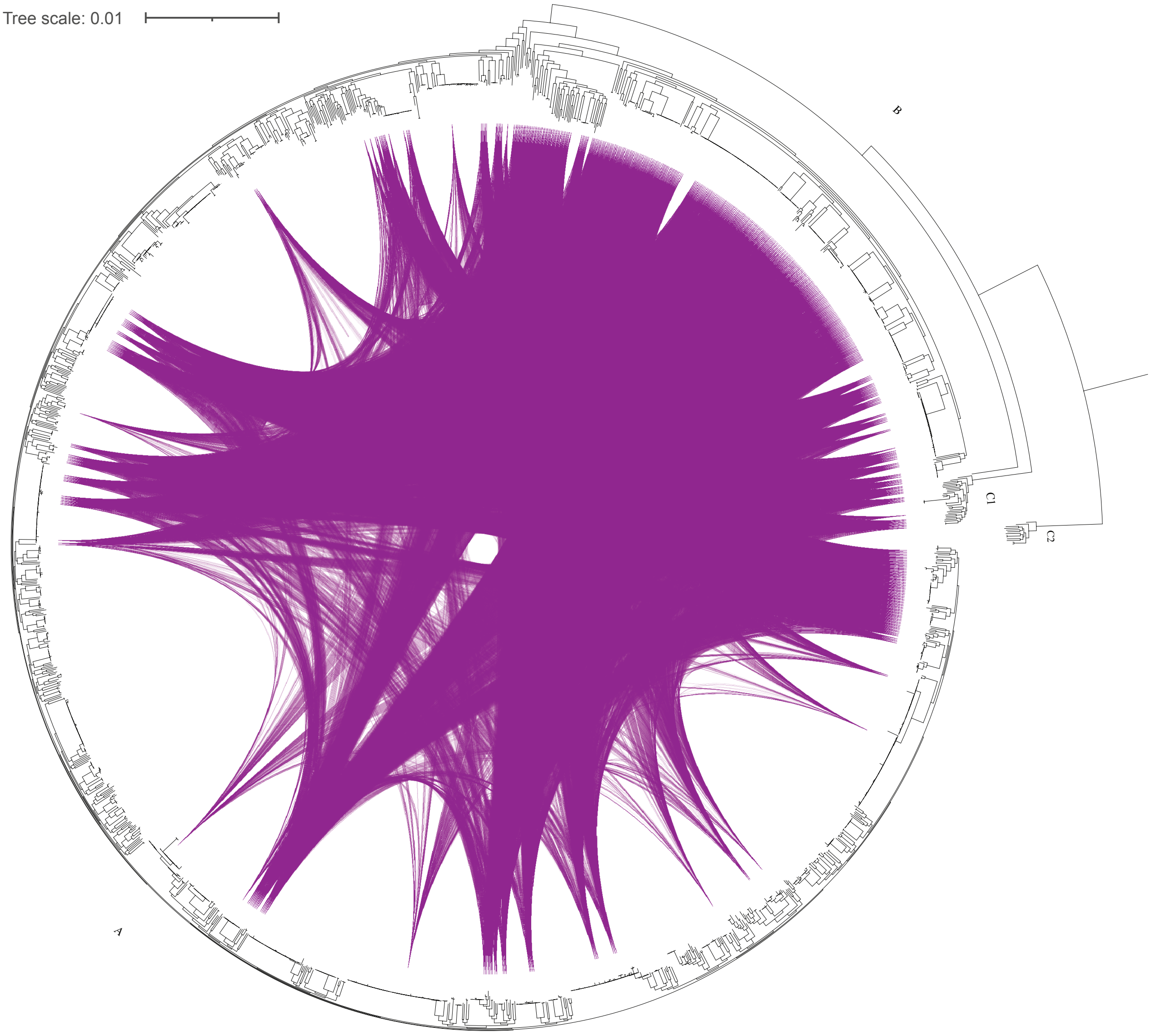

tle2

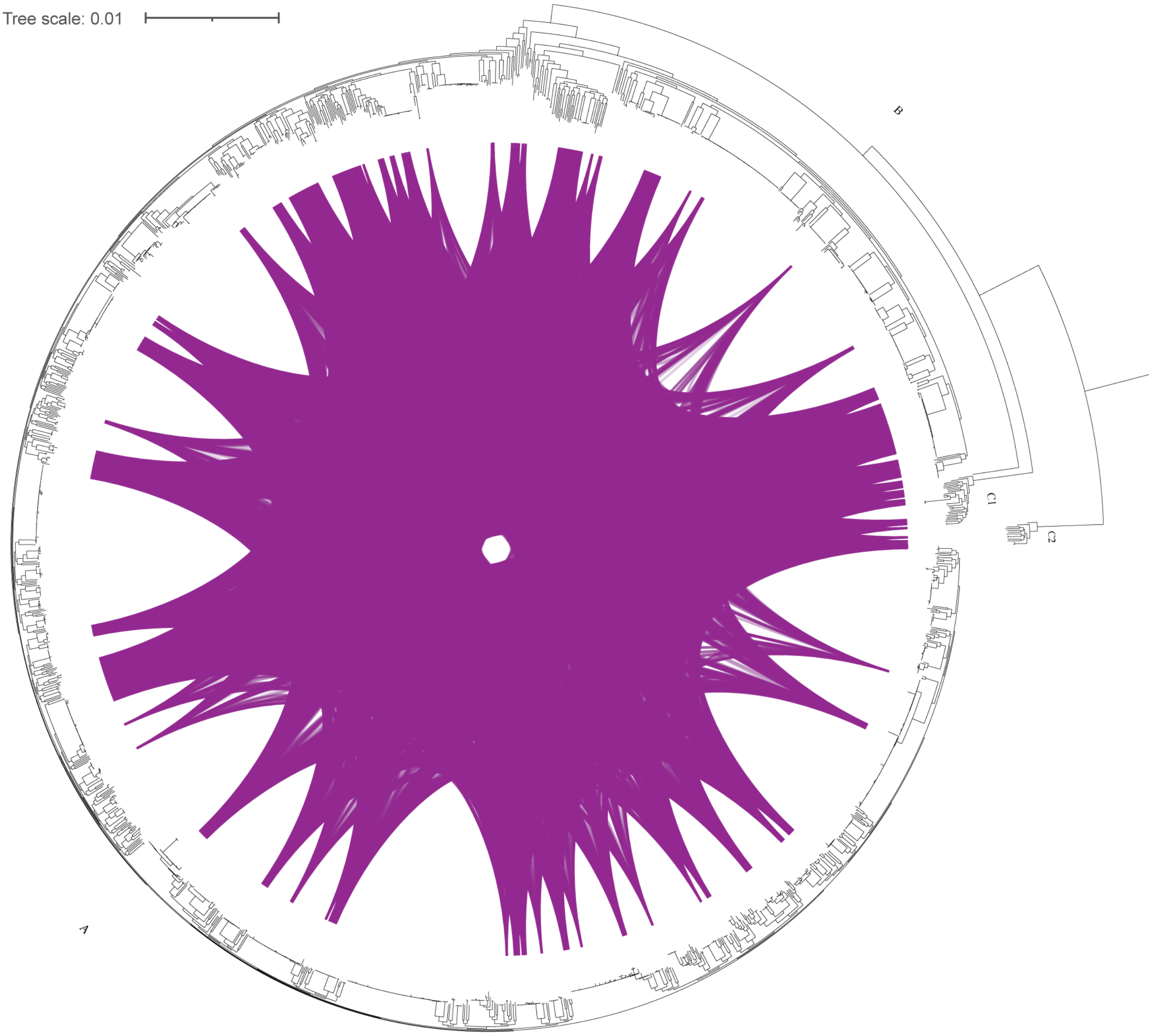

tspE1a

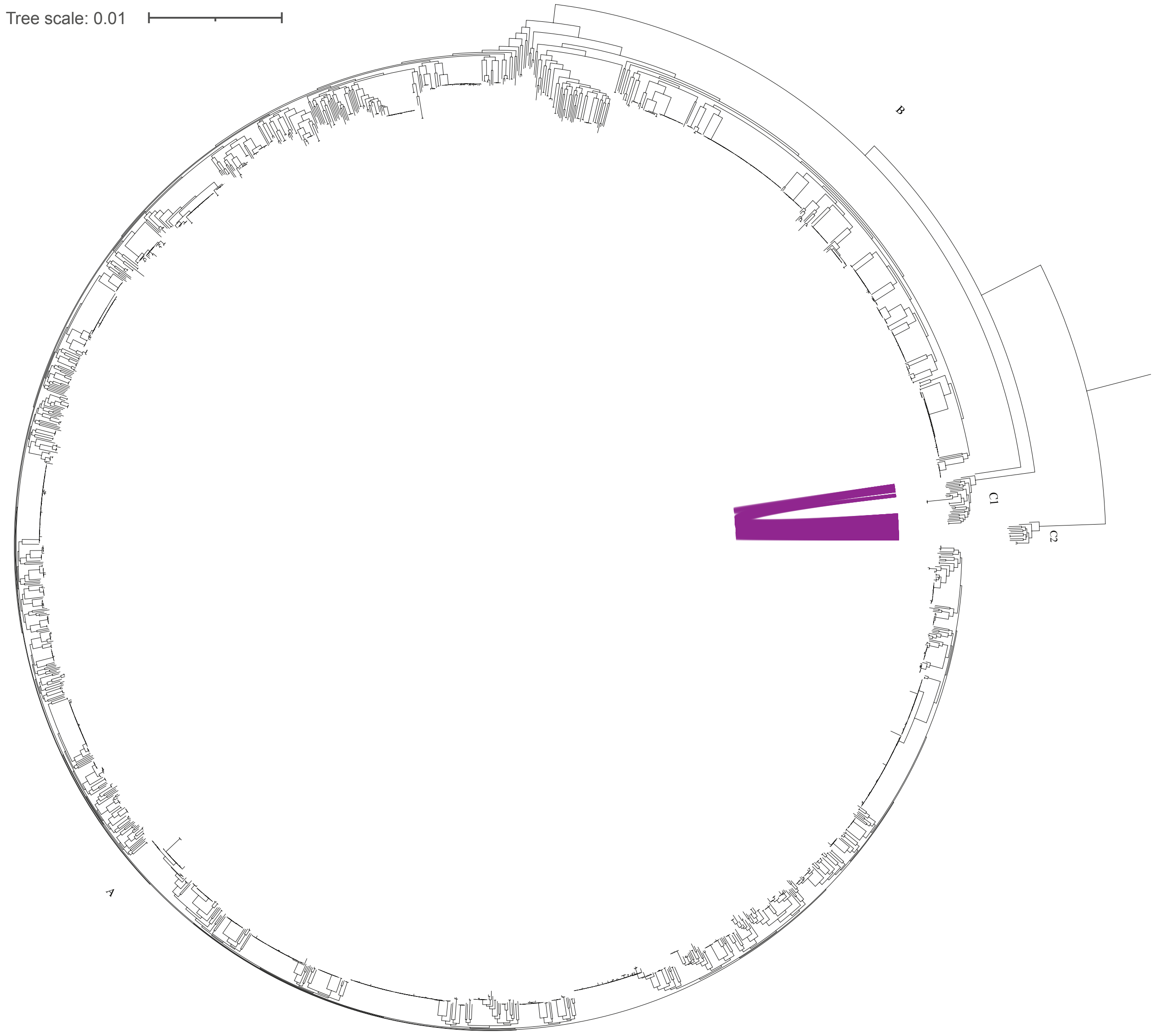

tspE1b

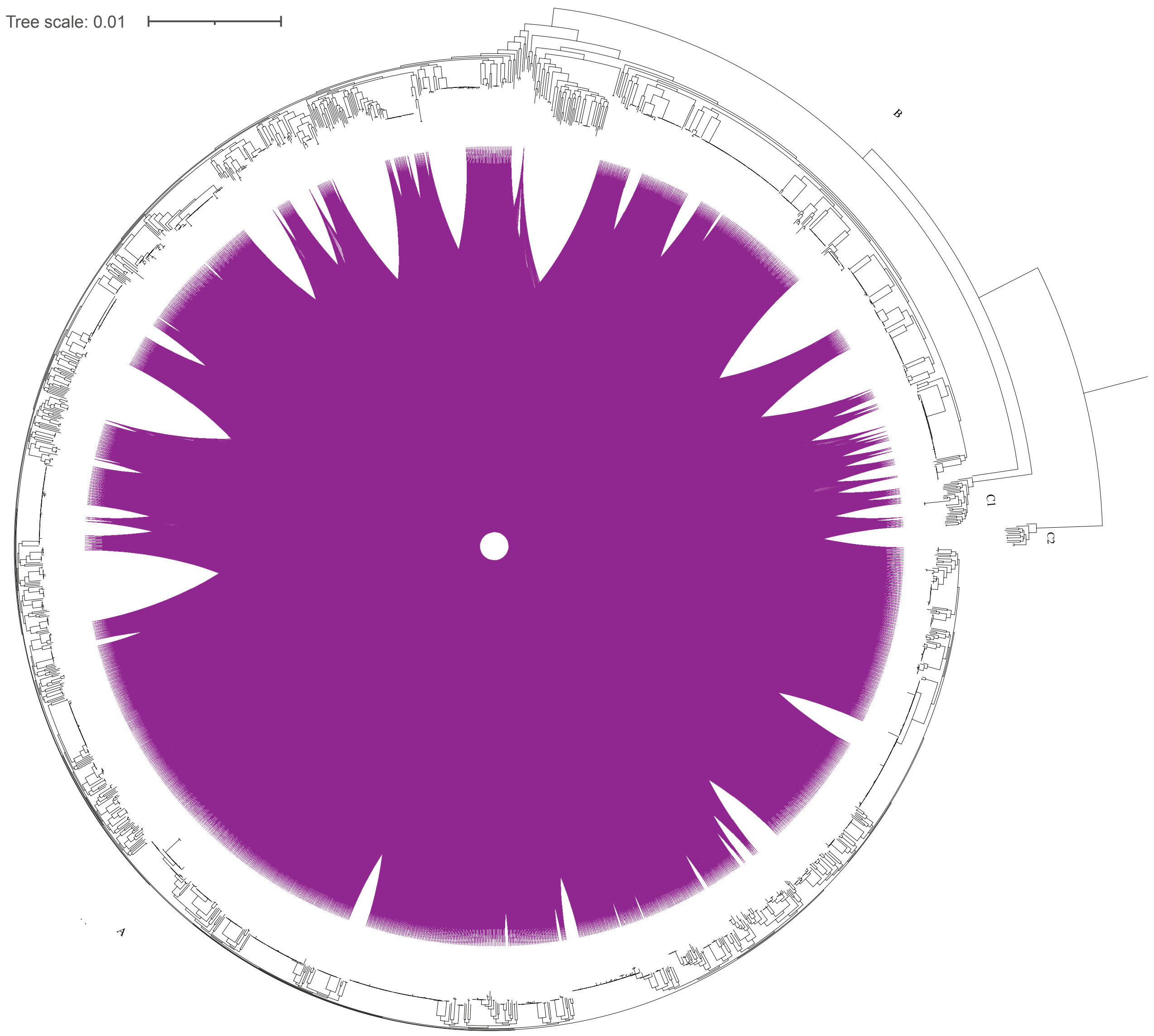

tspE1c

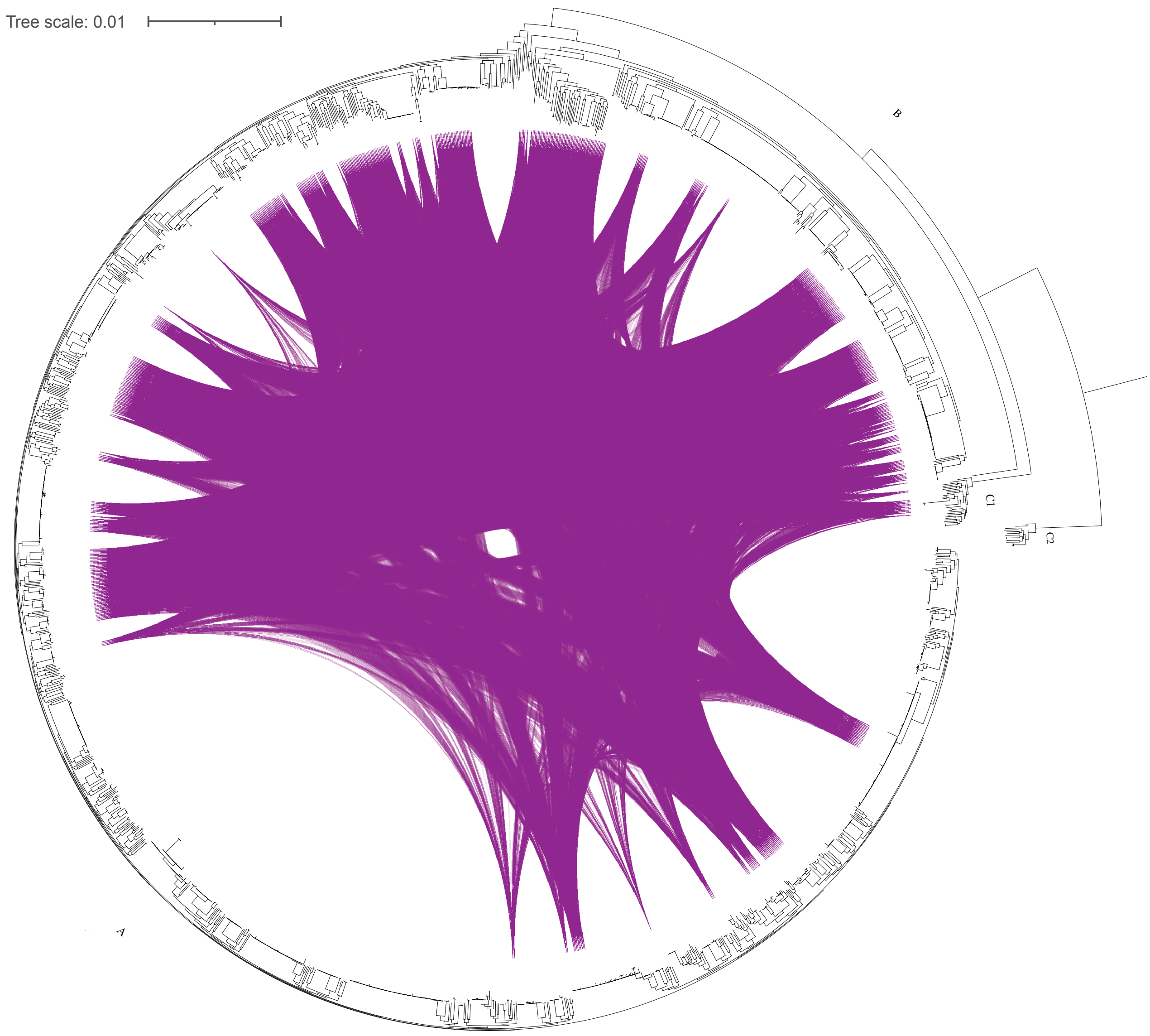

tepB

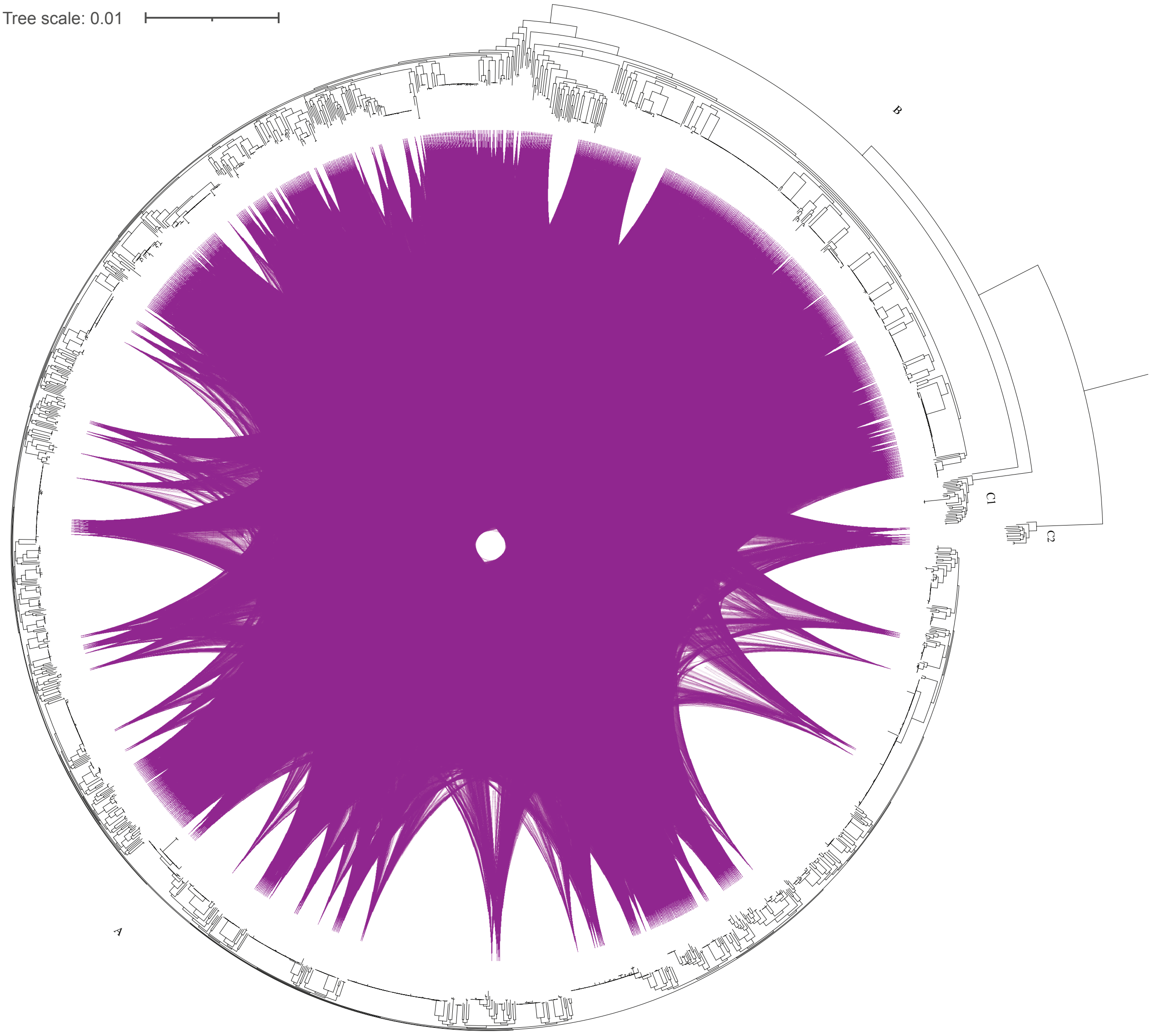

tepBa

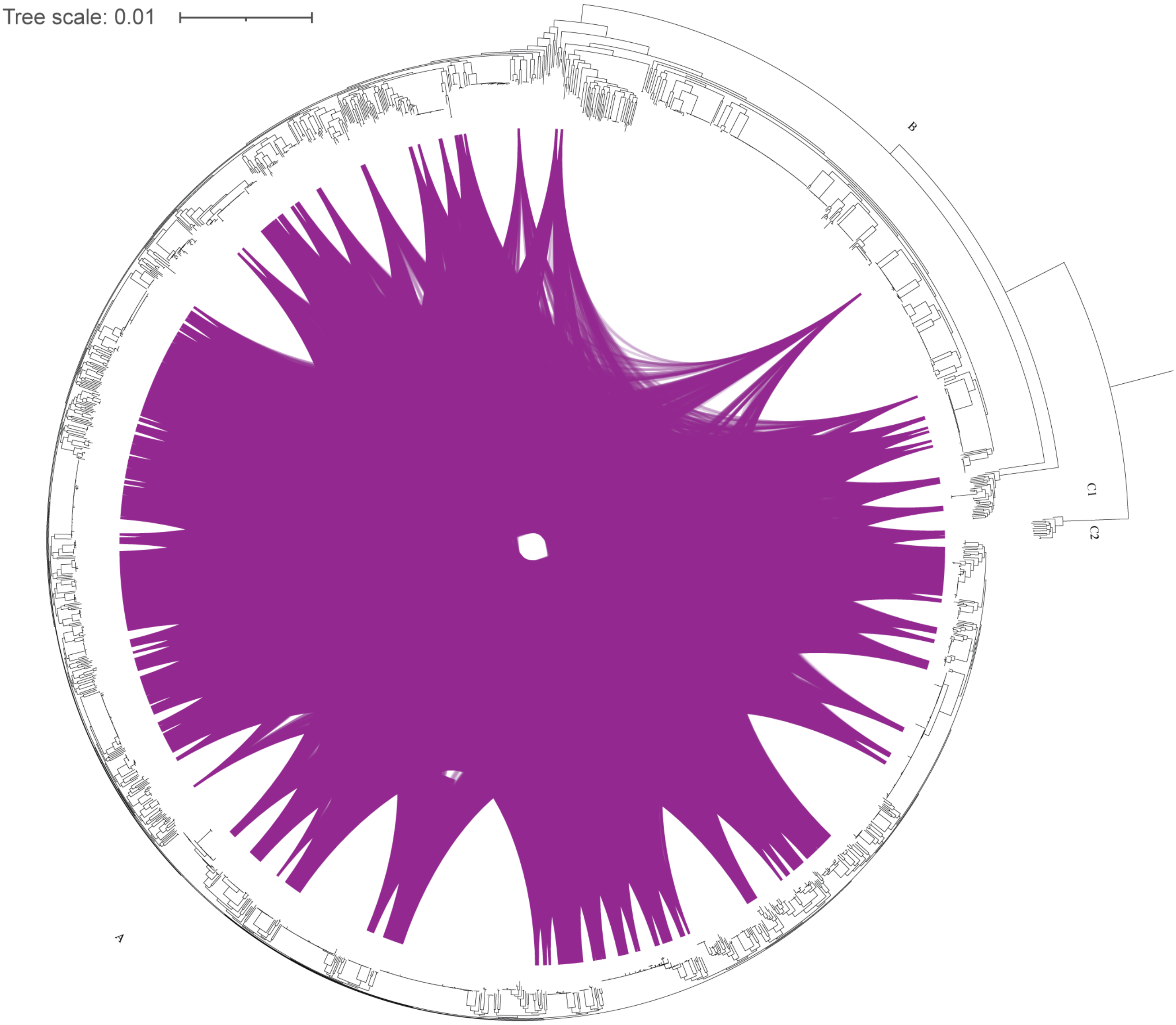

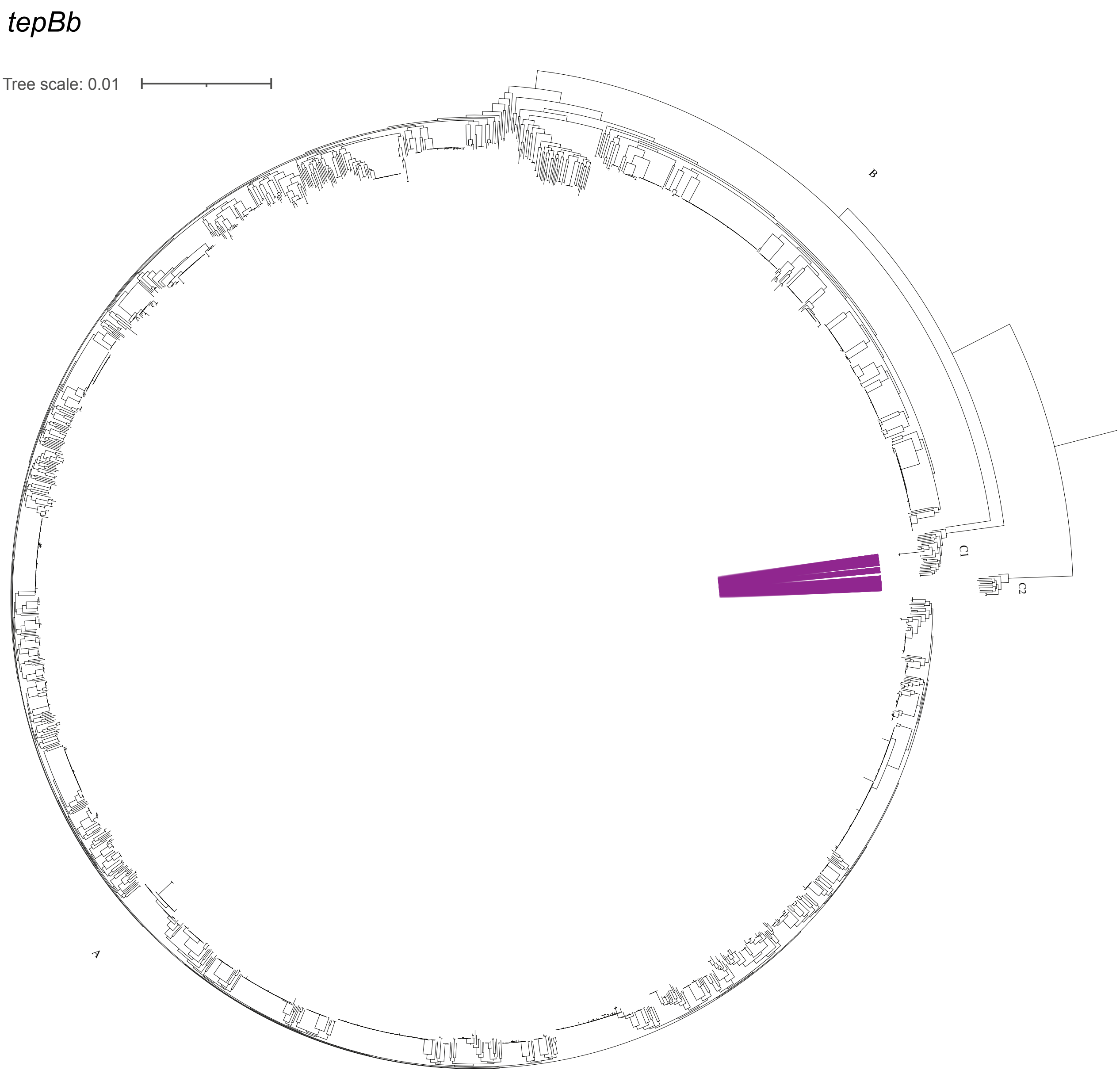
tepBb
Tree scale: 0.01
B
C1
C2
A
