# Supplementary material for: Distribution of the four type VI secretion systems in Pseudomonas aeruginosa and classification of their core and accessory effectors
Source: Nat Commun. 2025 Jan 21;16:888. doi: 10.1038/s41467-024-54649-5 (PMC11751169; doi:10.1038/s41467-024-54649-5)
Supplement: Supplementary file 36 — Reporting Summary [file 41467_2024_54649_MOESM36_ESM.pdf]

nature portfolio

Corresponding author(s): Daniel Unterweger

Last updated by author(s): 10/18/2024

Reporting Summary

Nature Portfolio wishes to improve the reproducibility of the work that we publish. This form provides structure for consistency and transparency in reporting. For further information on Nature Portfolio policies, see our Editorial Policies and the Editorial Policy Checklist.

Statistics

For all statistical analyses, confirm that the following items are present in the figure legend, table legend, main text, or Methods section.

n/a	Confirmed	
☐	☒	The exact sample size (*n*) for each experimental group/condition, given as a discrete number and unit of measurement
☒	☐	A statement on whether measurements were taken from distinct samples or whether the same sample was measured repeatedly
☐	☒	The statistical test(s) used AND whether they are one- or two-sided<br>*Only common tests should be described solely by name; describe more complex techniques in the Methods section.*
☐	☒	A description of all covariates tested
☐	☒	A description of any assumptions or corrections, such as tests of normality and adjustment for multiple comparisons
☐	☒	A full description of the statistical parameters including central tendency (e.g. means) or other basic estimates (e.g. regression coefficient) AND variation (e.g. standard deviation) or associated estimates of uncertainty (e.g. confidence intervals)
☐	☒	For null hypothesis testing, the test statistic (e.g. *F*, *t*, *r*) with confidence intervals, effect sizes, degrees of freedom and *P* value noted<br>*Give P values as exact values whenever suitable.*
☒	☐	For Bayesian analysis, information on the choice of priors and Markov chain Monte Carlo settings
☒	☐	For hierarchical and complex designs, identification of the appropriate level for tests and full reporting of outcomes
☐	☒	Estimates of effect sizes (e.g. Cohen's *d*, Pearson's *r*), indicating how they were calculated

Our web collection on statistics for biologists contains articles on many of the points above.

Software and code

Policy information about availability of computer code

Data collection	Genomes were downloaded from NCBI using Entrez direct.
Data analysis	Commercial and open source programs were used for analysis (as indicated in the Methods) including R (version 4.2.2), GraphPad Prism, Microsoft Office, blastn (version 2.10.1+), Geneious (version 2019.2.3), QUAST (version 5.2.0), BUSCO (version5.7.0), MacSyFinder (version 2.1.1), prokka (version 1.14.5), Panaroo (version 1.3.2), MUSCLE (version v3.8.31), ModelFinder, IQ-tree (version 1.6.12), RDP5, and R packages including phytools and PhyloCorrelate.

For manuscripts utilizing custom algorithms or software that are central to the research but not yet described in published literature, software must be made available to editors and reviewers. We strongly encourage code deposition in a community repository (e.g. GitHub). See the Nature Portfolio guidelines for submitting code & software for further information.

Data

Policy information about availability of data

All manuscripts must include a data availability statement. This statement should provide the following information, where applicable:
- Accession codes, unique identifiers, or web links for publicly available datasets
- A description of any restrictions on data availability
- For clinical datasets or third party data, please ensure that the statement adheres to our policy

Accession codes of analysed genomes are provided in Supplementary Data 2, Supplementary Data 13, and Supplementary Data 14.

Research involving human participants, their data, or biological material

Policy information about studies with human participants or human data. See also policy information about sex, gender (identity/presentation), and sexual orientation and race, ethnicity and racism.

Reporting on sex and gender	Not applicable.
Reporting on race, ethnicity, or other socially relevant groupings	Not applicable.
Population characteristics	Not applicable.
Recruitment	Not applicable.
Ethics oversight	Not applicable.

Note that full information on the approval of the study protocol must also be provided in the manuscript.

Field-specific reporting

Please select the one below that is the best fit for your research. If you are not sure, read the appropriate sections before making your selection.

☐ Life sciences ☐ Behavioural & social sciences ☒ Ecological, evolutionary & environmental sciences

For a reference copy of the document with all sections, see nature.com/documents/nr-reporting-summary-flat.pdf

Ecological, evolutionary & environmental sciences study design

All studies must disclose on these points even when the disclosure is negative.

Study description	Comparative genomics on Pseudomonas aeruginosa.
Research sample	This study analyzed genome sequences of Pseudomonas aeruginosa strains to test for intraspecific differences in genes of the type VI secretion systems and their effectors. The sample is meant to represent the global population of Pseudomonas aeruginosa. The genome sequences existed prior to the study and were publicly available from NCBI.
Sampling strategy	This dataset of genome sequences was chosen because the genomes cover the phylogenetic diversity of the species, belong to strains that were isolated from every continent, and include strains of clinical and environmental origin (as summarised in Supplementary Data 4). Genomes were selected based on their high quality (existing quality control and distance filtering steps in a previous analysis, doi: 10.1016/j.ebiom.2023.104532 and additional quality control performed by us using QUAST and BUSCO as described in detail in the methods section).
Data collection	Data was collected electronically by AH, VCV, and LAR during the computational analyses.
Timing and spatial scale	The genomes were downloaded on July 15, 2022 and were continuously analyzed since.
Data exclusions	For the analysis of effector occurrence, genomes that encoded less than three T6SS apparatus gene clusters were excluded to avoid a potential bias by missing apparatus gene clusters.
Reproducibility	All attempts to repeat the experiment were successful.
Randomization	Not applicable for the analyses performed.
Blinding	Not relevant for the study because external thresholds were applied.

Did the study involve field work? ☐ Yes ☒ No

Reporting for specific materials, systems and methods

We require information from authors about some types of materials, experimental systems and methods used in many studies. Here, indicate whether each material, system or method listed is relevant to your study. If you are not sure if a list item applies to your research, read the appropriate section before selecting a response.

Materials & experimental systems

n/a	Involved in the study	
☒	☐	Antibodies
☒	☐	Eukaryotic cell lines
☒	☐	Palaeontology and archaeology
☒	☐	Animals and other organisms
☐	☒	Clinical data
☒	☐	Dual use research of concern
☒	☐	Plants

Methods

n/a	Involved in the study	
☒	☐	ChIP-seq
☒	☐	Flow cytometry
☒	☐	MRI-based neuroimaging

Clinical data

Policy information about clinical studies

All manuscripts should comply with the ICMJEguidelines for publication of clinical research and a completedCONSORT checklist must be included with all submissions.

Clinical trial registration	Not applicable because not an interventional trial.
Study protocol	Not applicable because not an interventional trial.
Data collection	Not applicable because not an interventional trial.
Outcomes	Not applicable because not an interventional trial.
