# Supplementary material for: Distribution of the four type VI secretion systems in Pseudomonas aeruginosa and classification of their core and accessory effectors
Source: Nat Commun. 2025 Jan 21;16:888. doi: 10.1038/s41467-024-54649-5 (PMC11751169; doi:10.1038/s41467-024-54649-5)
Supplement: Supplementary file 10 — Supplementary Dataset 8 [file 41467_2024_54649_MOESM10_ESM.pdf]

Distribution of the four type VI secretion systems in *Pseudomonas aeruginosa* and classification of their core and accessory effectors

Supplementary Data 8

To view this file, open in Preview (MacOS) or Microsoft Edge (Windows).

This file contains the results of the stochastic mapping of the T6SS apparatus gene clusters. Each page shows the results of one T6SS apparatus gene cluster. The respective T6SS apparatus gene cluster is indicated at the top left corner of the page. The posterior probabilities of the stochastic mapping are indicated on each node of the phylogenetic tree. The tree is based on a core genome alignment of *P. aeruginosa* (n=1960) and *P. paraeruginosa* (n=5). Seven strains of the species *P. delhiensis, P. knackmussii, P. humi, P. jinjuensis, P. multiresinivorans, P. nitroreducens,* and *P. panipatensis* were used as an outgroup (not shown in the tree). The tree is based on the HKY+F+I model, rooted to the outgroup and distances are shown in substitutions per site.

H1−T6SS

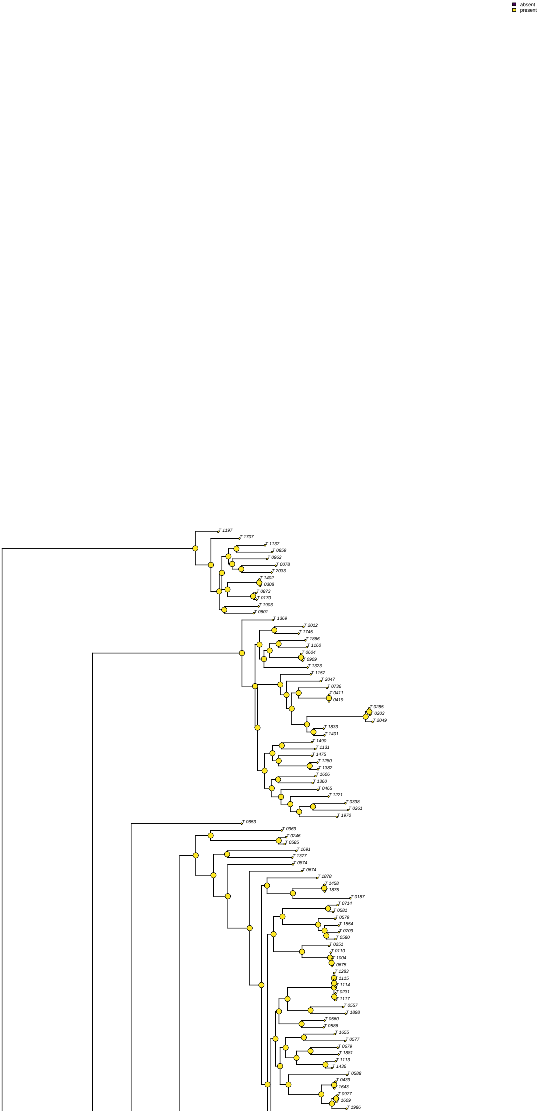

absent
present
T 1197
T 1707
T 1137
T 0859
T 0962
T 0078
T 2033
T 1402
0308
T 0873
T 0170
T 1903
T 0601
T 1369
T 2012
T 1745
T 1866
T 1160
T 0604
T 0909
T 1323
T 1157
T 2047
T 0736
T 0411
0419
T 0285
0203
T 2049
T 1833
T 1401
T 1490
T 1131
T 1475
T 1280
T 1382
T 1606
T 1360
T 0465
T 1221
T 0338
T 0261
T 1970
T 0653
T 0969
T 0246
T 0585
T 1691
T 1377
T 0874
T 0674
T 1878
T 1458
1875
T 0187
T 0714
T 0581
T 0579
T 1554
T 0709
T 0580
T 0251
T 0110
T 1004
0675
T 1283
1115
T 1114
T 0231
1117
T 0557
T 1898
T 0560
T 0586
T 1655
T 0577
T 0679
T 1881
T 1113
T 1436
T 0588
T 0439
1643
T 0977
1609
T 1986

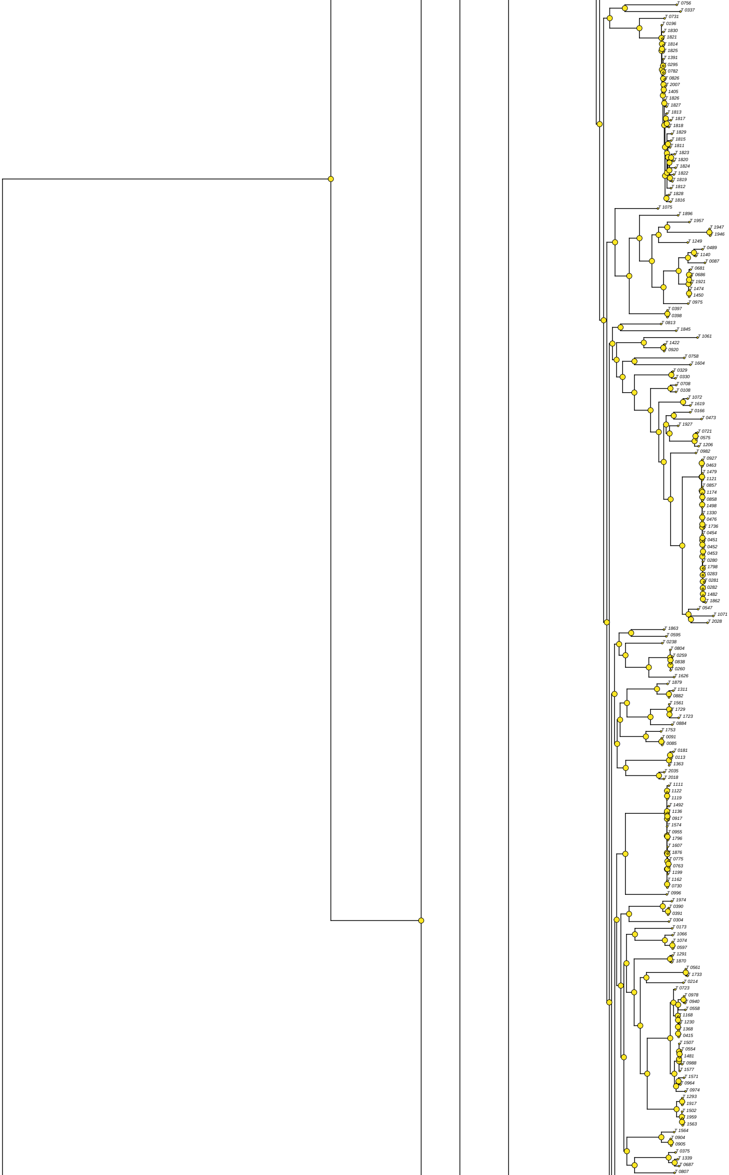

T 0756
T 0337
T 0731
T 0196
T 1830
T 1821
T 1814
T 1825
T 1391
0295
0782
T 0826
T 2007
1405
T 1826
T 1827
T 1813
T 1817
T 1818
T 1829
T 1815
T 1811
T 1823
T 1820
T 1824
T 1822
T 1819
T 1812
T 1828
T 1816
T 1075
T 1896
T 1957
T 1947
1946
T 1249
T 0489
T 1140
T 0087
T 0681
T 0686
T 1921
T 1474
1450
T 0975
T 0397
0398
T 0813
T 1845
T 1061
T 1422
0920
T 0758
T 1604
T 0329
T 0330
T 0708
T 0108
T 1072
T 1619
T 0166
T 0473
T 1927
T 0721
0575
T 1206
T 0982
T 0927
0463
T 1479
1121
T 0857
1174
0858
1498
T 1330
0476
T 1736
T 0454
0451
0452
0453
T 0280
1798
0283
T 0281
0282
1482
T 1862
T 0547
T 1071
T 2028
T 1863
T 0595
T 0238
T 0804
T 0259
0838
0260
T 1626
T 1879
T 1311
0882
T 1561
T 1729
T 1723
T 0884
T 1753
T 0091
0085
T 0181
0113
1363
T 2035
T 2018
T 1111
1122
1119
T 1492
1136
0917
T 1574
T 0955
1796
T 1607
1876
T 0775
0763
1199
T 1162
0730
T 0996
T 1974
T 0390
0391
T 0304
T 0173
T 1066
T 1074
0597
T 1291
T 1870
T 0561
T 1733
T 0214
T 0723
T 0978
T 0940
T 0558
1168
T 1230
1368
0415
T 1507
T 0554
1481
T 0988
T 1577
T 1571
T 0964
T 0974
T 1293
1917
T 1502
1959
1563
T 1564
T 0904
0905
T 0375
T 1339
T 0687
T 0807

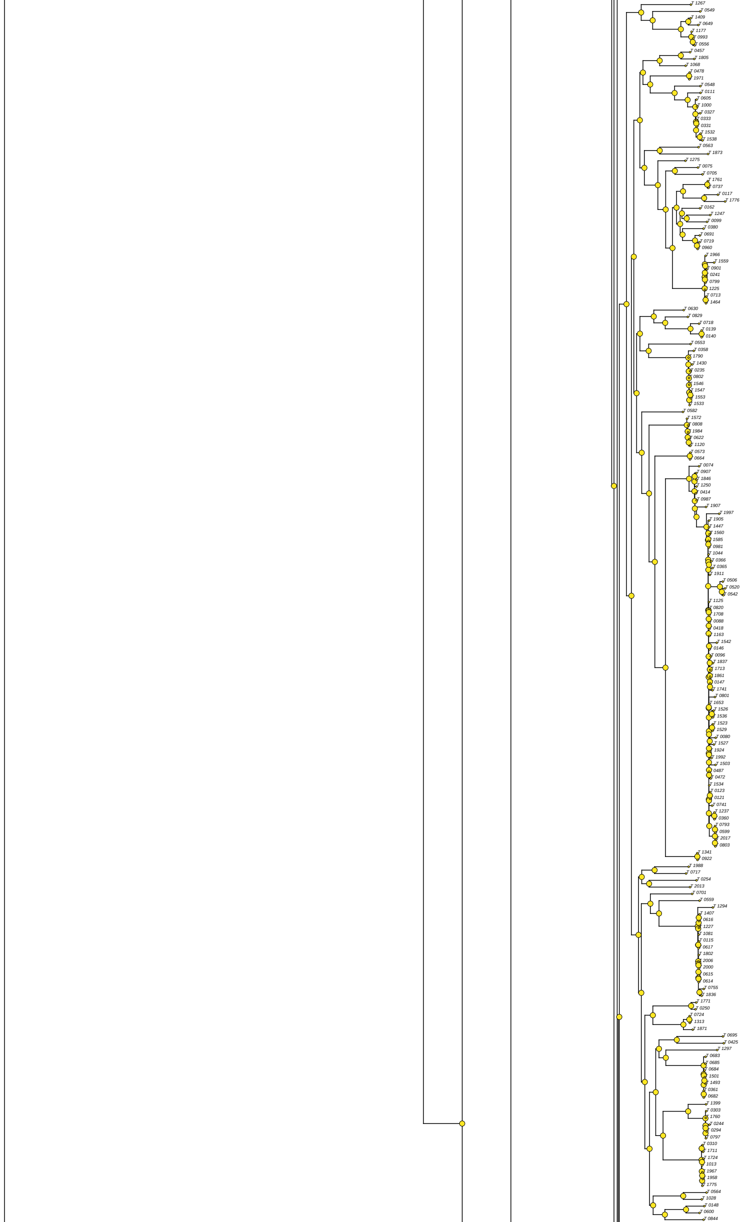
T 1267
T 0549
T 1409
T 0649
T 1177
T 0993
T 0556
T 0457
T 1805
T 1068
T 0478
T 1971
T 0548
T 0111
T 0605
T 1000
T 0327
T 0333
T 0331
T 1532
T 1538
T 0563
T 1873
T 1275
T 0075
T 0705
T 1761
T 0737
T 0117
T 1776
T 0162
T 1247
T 0099
T 0380
T 0691
T 0719
T 0960
T 1966
T 1559
T 0901
T 0241
T 0799
T 1225
T 0713
T 1464
T 0630
T 0829
T 0718
T 0139
T 0140
T 0553
T 0358
T 1790
T 1430
T 0235
T 0802
T 1546
T 1547
T 1553
T 1533
T 0582
T 1572
T 0808
T 1984
T 0622
T 1120
T 0573
T 0664
T 0074
T 0907
T 1846
T 1250
T 0414
T 0987
T 1907
T 1997
T 1905
T 1447
T 1560
T 1585
T 0981
T 1044
T 0366
T 0365
T 1911
T 0506
T 0520
T 0542
T 1125
T 0820
T 1708
T 0088
T 0418
T 1163
T 1542
T 0146
T 0096
T 1837
T 1713
T 1861
T 0147
T 1741
T 0801
T 1653
T 1526
T 1536
T 1523
T 1529
T 0080
T 1527
T 1924
T 1992
T 1503
T 0487
T 0472
T 1534
T 0123
T 0121
T 0741
T 1237
T 0360
T 0793
T 0599
T 2017
T 0803
T 1341
T 0922
T 1988
T 0717
T 0254
T 2013
T 0701
T 0559
T 1294
T 1407
T 0616
T 1227
T 1081
T 0115
T 0617
T 1802
T 2006
T 2000
T 0615
T 0614
T 0755
T 1836
T 1771
T 0250
T 0724
T 1313
T 1871
T 0695
T 0425
T 1297
T 0683
T 0685
T 0684
T 1501
T 1493
T 0361
T 0682
T 1399
T 0303
T 1760
T 0244
T 0294
T 0797
T 0310
T 1711
T 1724
T 1013
T 1967
T 1958
T 1775
T 0564
T 1028
T 0148
T 0600
T 0844

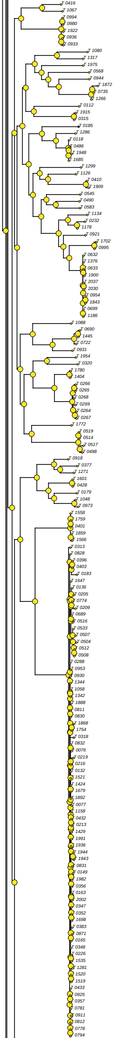
T 0416
T 1067
T 0994
0980
T 1922
0936
T 0933
T 1080
T 1317
T 1975
T 0568
T 0944
T 1872
T 0735
1266
T 0112
T 1915
0315
T 0185
T 1286
T 0118
T 0486
T 1948
T 1685
T 1299
T 1126
T 0410
T 1909
T 0545
T 0490
T 0583
T 1134
T 0232
1178
T 0921
T 1702
0995
T 0632
T 1376
0633
T 1900
T 2037
2030
T 0954
1843
T 0699
1186
T 1088
T 0690
1445
T 0722
T 0931
T 1954
T 0320
T 1780
1404
T 0266
0265
T 0268
T 0269
T 0264
T 0267
T 1772
T 0519
0514
T 0517
T 0498
T 0918
T 0377
T 1271
T 1601
0428
T 0179
T 1048
T 0973
T 1558
1759
0401
T 1859
T 1566
T 0313
T 0828
T 0396
0403
T 0183
T 1647
T 0136
T 0205
0774
T 0209
T 0689
T 0516
T 0533
T 0507
T 0504
0512
0508
T 0288
T 0953
0930
1344
T 1058
1342
T 1888
0811
0830
T 1868
1754
T 0318
T 0832
0076
T 0219
0216
T 0132
1521
T 1424
1679
1892
T 0077
T 1158
0432
0213
1429
1991
1936
T 1944
1943
0831
T 0149
1982
0356
T 0163
2002
0347
0352
1698
T 0383
0871
0165
T 0348
T 0226
1535
T 1281
1520
1519
T 0433
0925
0357
T 0781
0911
0812
0778
0794

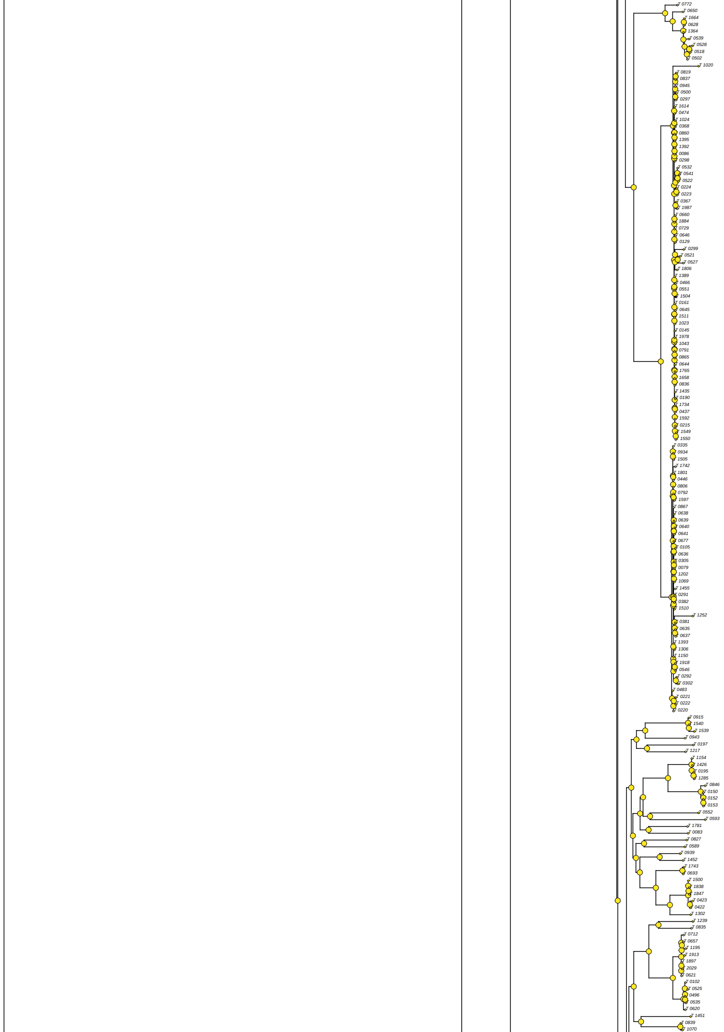
T 0772
0650
T 1664
0628
1364
T 0539
T 0528
T 0518
0502
T 1020
T 0819
0837
0945
0500
0297
T 1614
0474
T 1024
0368
0860
1395
1392
0086
0298
T 0532
T 0541
0522
T 0224
0223
T 0367
T 1987
T 0660
1884
0729
T 0646
0129
T 0299
T 0521
T 0527
T 1806
T 1389
0466
0551
1504
T 0161
0645
1511
1023
T 0145
T 1978
1043
0791
0865
0644
1765
1658
0836
T 1435
T 0190
T 1734
0437
1592
0215
T 1549
1550
T 0335
0934
1505
T 1742
T 1801
0446
0806
0792
T 1597
T 0867
T 0638
0639
T 0640
0641
T 0677
T 0105
0636
T 0305
0079
1202
1069
T 1455
T 0291
0382
T 1510
T 1252
0381
0635
0637
T 1393
1306
T 1150
T 1918
0546
T 0292
T 0302
T 0483
T 0221
T 0222
T 0220
T 0915
T 1540
T 1539
T 0943
T 0197
T 1217
T 1154
T 1426
T 0195
1285
T 0846
T 0150
0152
0153
T 0552
T 0593
T 1781
T 0083
T 0827
T 0589
T 0939
T 1452
T 1743
0693
T 1500
1838
1847
T 0423
0422
T 1302
T 1239
T 0835
T 0712
T 0657
T 1195
T 1913
T 1897
2029
0621
T 0102
T 0525
0496
0535
T 0620
T 1451
T 0839
T 1070

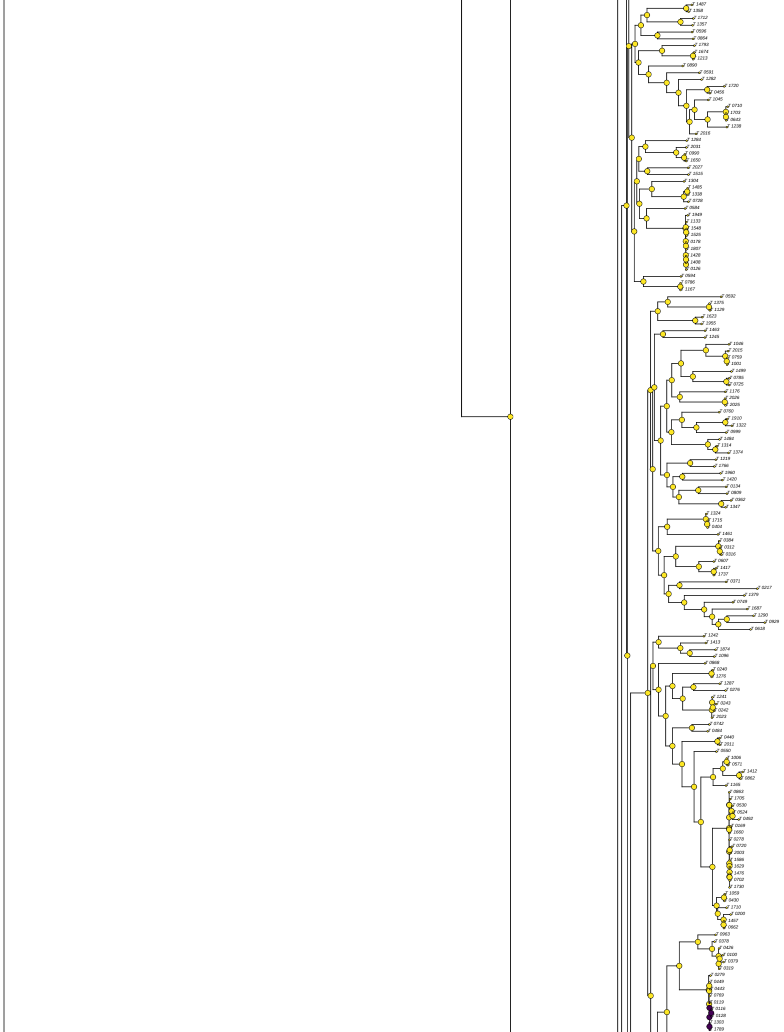
T 1487
T 1358
T 1712
T 1357
T 0596
T 0864
T 1793
T 1674
T 1213
T 0890
T 0591
T 1282
T 1720
T 0456
T 1045
T 0710
T 1703
T 0643
T 1238
T 2016
T 1284
T 2031
T 0990
T 1650
T 2027
T 1515
T 1304
T 1485
T 1338
T 0728
T 0584
T 1949
T 1133
T 1548
T 1525
T 0178
T 1807
T 1428
T 1408
T 0126
T 0594
T 0786
T 1167
T 0592
T 1375
T 1129
T 1623
T 1955
T 1463
T 1245
T 1046
T 2015
T 0759
T 1001
T 1499
T 0785
T 0725
T 1176
T 2026
T 2025
T 0760
T 1910
T 1322
T 0999
T 1484
T 1314
T 1374
T 1219
T 1766
T 1960
T 1420
T 0134
T 0809
T 0362
T 1347
T 1324
T 1715
T 0404
T 1461
T 0384
T 0312
T 0316
T 0607
T 1417
T 1737
T 0371
T 0217
T 1379
T 0749
T 1687
T 1290
T 0929
T 0618
T 1242
T 1413
T 1874
T 1096
T 0868
T 0240
T 1276
T 1287
T 0276
T 1241
T 0243
T 0242
T 2023
T 0742
T 0484
T 0440
T 2011
T 0550
T 1006
T 0571
T 1412
T 0862
T 1165
T 0863
T 1705
T 0530
T 0524
T 0492
T 0169
T 1660
T 0278
T 0720
T 2003
T 1586
T 1629
T 1476
T 0702
T 1730
T 1059
T 0430
T 1710
T 0200
T 1457
T 0662
T 0963
T 0378
T 0426
T 0100
T 0379
T 0319
T 0279
T 0449
T 0443
T 0769
T 0119
T 0116
T 0128
T 1303
T 1789

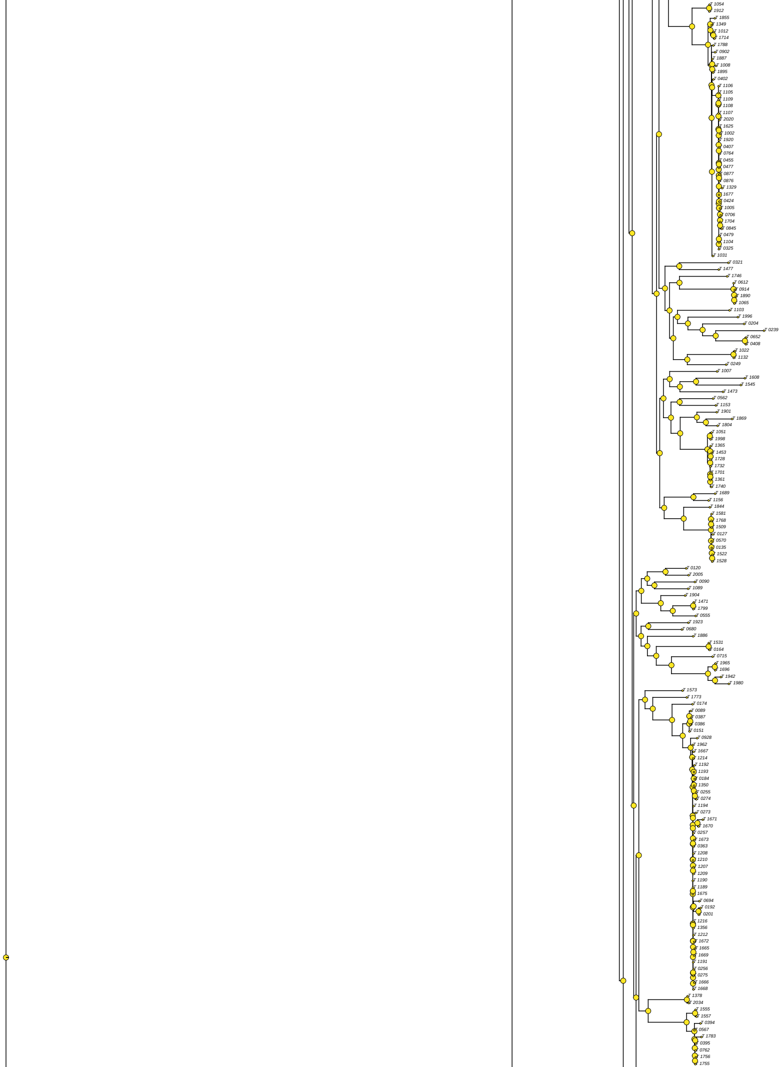
1054
1912
1855
1349
1012
1714
1788
0902
1887
1008
1895
0402
1106
1105
1109
1108
1107
2020
1625
1002
1920
0407
0764
0455
0477
0877
0876
1329
1677
0424
1005
0706
1704
0845
0479
1104
0325
1031
0321
1477
1746
0612
0914
1890
1065
1103
1996
0204
0239
0652
0408
1022
1132
0249
1007
1608
1545
1473
0562
1153
1901
1869
1804
1051
1998
1365
1453
1728
1732
1701
1361
1740
1689
1156
1844
1581
1768
1509
0127
0570
0135
1522
1528
0120
2005
0090
1089
1904
1471
1799
0555
1923
0680
1886
1531
0164
0715
1965
1696
1942
1980
1573
1773
0174
0089
0387
0386
0151
0928
1962
1667
1214
1192
1193
0184
1350
0255
0274
1194
0273
1671
1670
0257
1673
0363
1208
1210
1207
1209
1190
1189
1675
0694
0192
0201
1216
1356
1212
1672
1665
1669
1191
0256
0275
1666
1668
1378
2034
1555
1557
0394
0567
1783
0395
0762
1756
1755

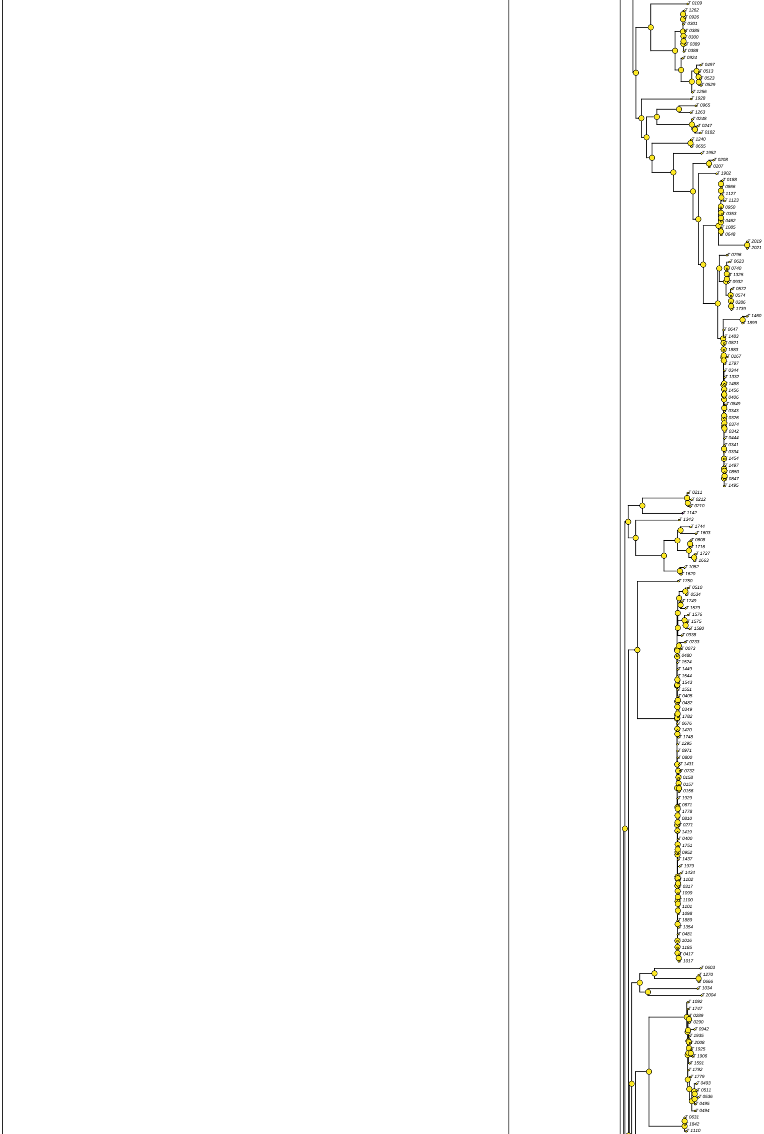
0109
1262
0926
0301
0385
0300
0389
0388
0924
0497
0513
0523
0529
1256
1928
0965
1263
0248
0247
0182
1240
0655
1952
0208
0207
1902
0188
0866
1127
1123
0950
0353
0462
1085
0648
2019
2021
0796
0623
0740
1325
0932
0572
0574
0286
1739
1460
1899
0647
1483
0821
1883
0167
1797
0344
1332
1488
1456
0406
0849
0343
0326
0374
0342
0444
0341
0334
1454
1497
0850
0847
1495
0211
0212
0210
1142
1343
1744
1603
0608
1716
1727
1663
1052
1620
1750
0510
0534
1749
1579
1576
1575
1580
0938
0233
0073
0480
1524
1449
1544
1543
1551
0405
0482
0349
1782
0676
1470
1748
1295
0971
0800
1431
0732
0158
0157
0156
1929
0671
1778
0810
0271
1419
0400
1751
0952
1437
1979
1434
1102
0317
1099
1100
1101
1098
1889
1354
0481
1016
1185
0417
1017
0603
1270
0666
1034
2004
1092
1747
0289
0290
0942
1935
2008
1925
1906
1591
1792
1779
0493
0511
0536
0495
0494
0631
1842
1110

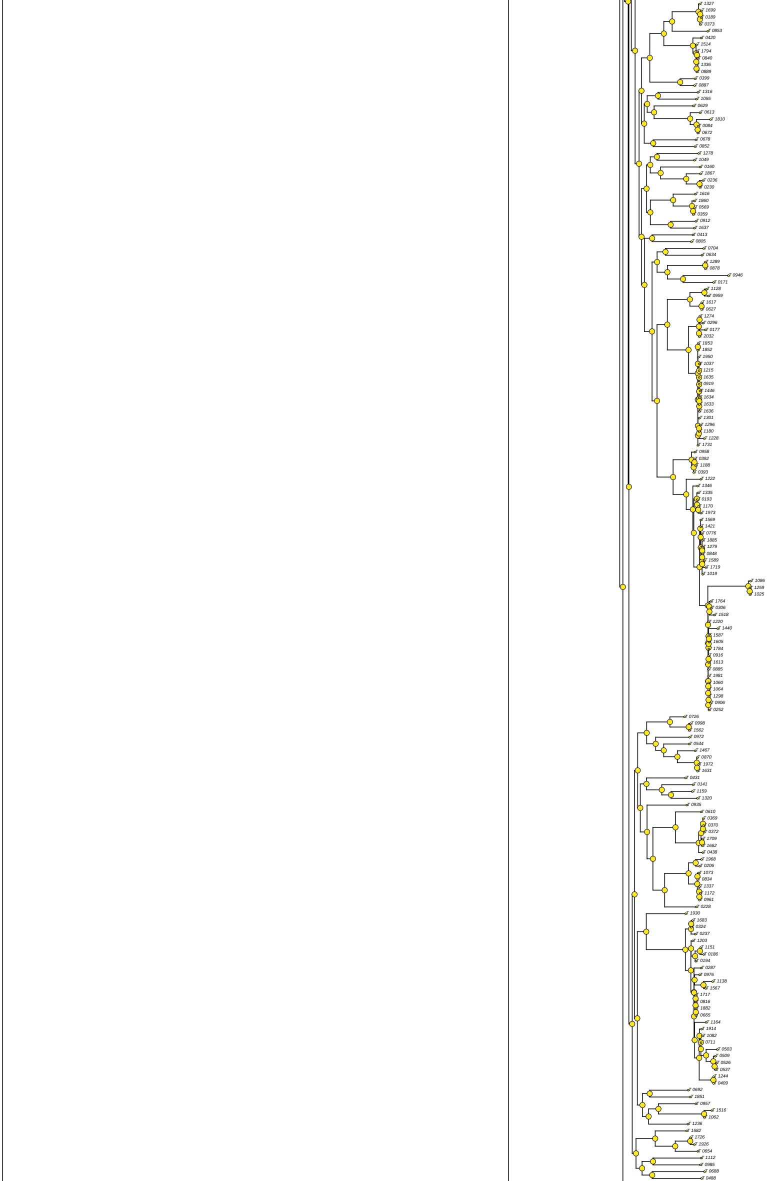
T 1327
T 1699
T 0189
T 0373
T 0853
T 0420
T 1514
T 1794
T 0840
T 1336
T 0889
T 0399
T 0887
T 1316
T 1055
T 0629
T 0613
T 1810
T 0084
T 0672
T 0678
T 0852
T 1278
T 1049
T 0160
T 1867
T 0236
T 0230
T 1616
T 1860
T 0569
T 0359
T 0912
T 1637
T 0413
T 0805
T 0704
T 0634
T 1289
T 0878
T 0946
T 0171
T 1128
T 0959
T 1617
T 0627
T 1274
T 0296
T 0177
T 2032
T 1853
T 1852
T 1950
T 1037
T 1215
T 1635
T 0919
T 1446
T 1634
T 1633
T 1636
T 1301
T 1296
T 1180
T 1228
T 1731
T 0958
T 0392
T 1188
T 0393
T 1222
T 1346
T 1335
T 0193
T 1170
T 1973
T 1569
T 1421
T 0776
T 1885
T 1279
T 0848
T 1589
T 1719
T 1019
T 1086
T 1259
T 1025
T 1764
T 0306
T 1518
T 1220
T 1440
T 1587
T 1605
T 1784
T 0916
T 1613
T 0885
T 1981
T 1060
T 1064
T 1298
T 0906
T 0252
T 0726
T 0998
T 1562
T 0972
T 0544
T 1467
T 0870
T 1972
T 1631
T 0431
T 0141
T 1159
T 1320
T 0935
T 0610
T 0369
T 0370
T 0372
T 1709
T 1662
T 0438
T 1968
T 0206
T 1073
T 0834
T 1337
T 1172
T 0961
T 0228
T 1930
T 1683
T 0324
T 0237
T 1203
T 1151
T 0186
T 0194
T 0287
T 0976
T 1138
T 1567
T 1717
T 0816
T 1882
T 0665
T 1164
T 1914
T 1082
T 0711
T 0503
T 0509
T 0526
T 0537
T 1244
T 0409
T 0692
T 1851
T 0957
T 1516
T 1062
T 1236
T 1582
T 1726
T 1926
T 0654
T 1112
T 0985
T 0688
T 0488

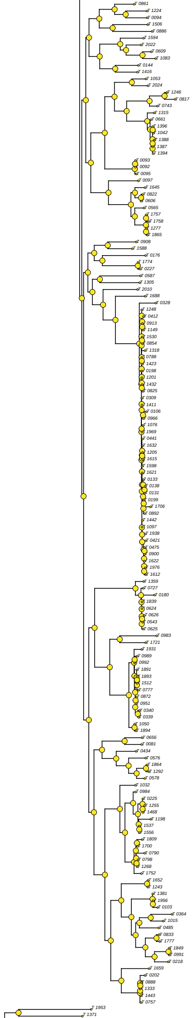
T 0861
T 1224
T 0094
T 1506
T 0886
T 1594
T 2022
T 0609
T 1083
T 0144
T 1416
T 1053
T 2024
T 1246
T 0817
T 0743
T 1315
T 0661
T 1396
1042
T 1388
1387
T 1394
T 0093
0092
T 0095
T 0097
T 1645
T 0822
0606
T 0565
T 1757
T 1758
T 1277
T 1865
T 0908
T 1588
T 0176
T 1774
T 0227
T 0587
T 1305
T 2010
T 1688
T 0328
T 1248
T 0412
0913
1149
1530
0854
T 1318
0788
1423
0198
1201
1432
T 0825
T 0309
1411
T 0106
0966
T 1076
1969
T 0441
T 1632
1205
1615
T 1598
1621
T 0133
T 0138
0131
0199
T 1706
T 0892
T 1442
1097
T 1938
T 0421
T 0475
0900
1622
T 1976
T 1612
T 1359
T 0727
T 0180
1839
0624
T 0626
0543
T 0625
T 0983
T 1721
T 1931
T 0989
0992
T 1891
T 1893
1512
T 0777
T 0872
0951
T 0340
0339
T 1050
T 1894
T 0656
T 0081
T 0434
T 0576
T 1864
T 1292
T 0578
T 1032
T 0984
T 0225
T 1255
1468
T 1198
T 1537
T 1556
T 1809
T 1700
T 0790
T 0798
T 1268
T 1752
T 1652
T 1243
T 1381
T 1956
T 0103
T 0364
T 1015
T 0485
T 0833
T 1777
T 1849
T 0991
T 0218
T 1659
T 0202
T 0888
T 1333
T 1443
T 0757
T 1953
T 1371

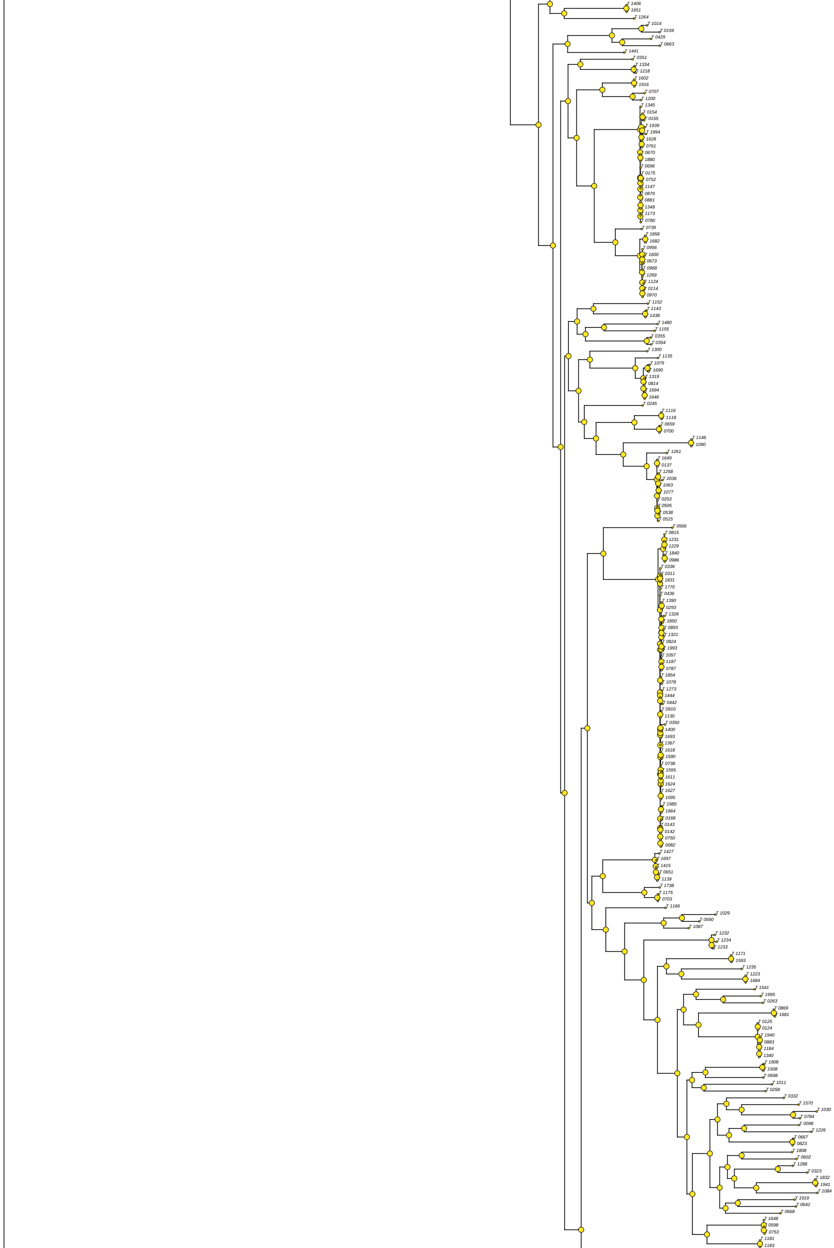

T 1406
1651
T 1264
T 1014
T 0159
T 0429
T 0663
T 1441
T 0351
T 1334
T 1218
T 1602
1916
T 0707
T 1200
T 1345
T 0154
T 0155
T 1939
T 1994
1628
0761
0670
1880
T 0696
T 0175
0752
1147
0879
0881
1348
1173
T 0780
T 0739
T 1858
1682
T 0956
T 1600
0673
T 0968
1269
T 1124
T 0114
0970
T 1152
T 1143
1438
T 1480
T 1155
T 0355
T 0354
T 1300
T 1135
T 1079
T 1690
T 1319
0814
1694
1646
T 0245
T 1116
1118
T 0659
0700
T 1146
1090
T 1261
T 1649
0137
T 1258
T 2036
1063
1077
T 0253
T 0505
T 0538
T 0515
T 0566
T 0815
1231
1229
T 1840
0986
T 0336
T 0311
1831
T 1770
T 0436
T 1390
0293
T 1326
T 1850
T 0893
T 1321
T 0824
T 1993
T 1057
T 1187
0787
T 1854
T 1078
T 1273
1444
T 0442
T 0910
1130
T 0350
1400
1693
T 1367
T 1618
1590
T 0738
1595
1611
1624
T 1627
1095
T 1985
1964
T 0168
T 0143
0142
0750
0082
T 1427
T 1697
1415
T 0651
1139
T 1738
T 1175
0703
T 1166
T 1029
T 0590
T 1087
T 1232
T 1234
T 1233
T 1171
1593
T 1235
T 1223
1684
T 1541
T 1995
T 0263
T 0869
1681
T 0125
0124
T 1940
0883
1184
1340
T 1908
1508
T 0698
T 1011
T 0258
T 0332
T 1570
T 1030
T 0784
T 0098
T 1226
T 0667
0823
T 1808
T 0602
T 1288
T 0323
T 1832
1941
T 1084
T 1919
T 0642
T 0668
T 1648
0598
0753
T 1181
1183

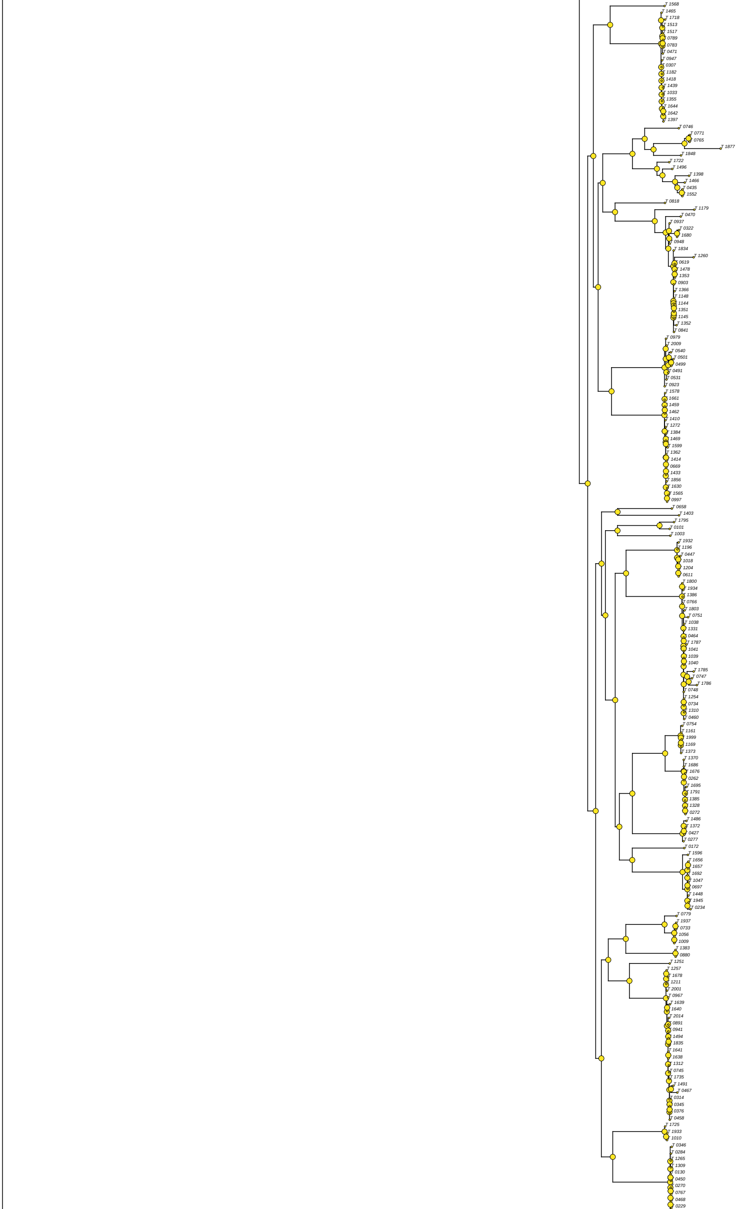

1568
1465
1718
1513
1517
0789
0783
0471
0947
0307
1182
1418
1439
1033
1355
1644
1642
1397
0746
0771
0765
1877
1848
1722
1496
1398
1466
0435
1552
0818
1179
0470
0937
0322
1680
0948
1834
1260
0619
1478
1353
0903
1366
1148
1144
1351
1145
1352
0841
0979
2009
0540
0501
0499
0491
0531
0923
1578
1661
1459
1462
1410
1272
1384
1469
1599
1362
1414
0669
1433
1856
1630
1565
0997
0658
1403
1795
0101
1003
1932
1196
0447
1018
1204
0611
1800
1934
1386
0766
1803
0751
1038
1331
0464
1787
1041
1039
1040
1785
0747
1786
0748
1254
0734
1310
0460
0754
1161
1999
1169
1373
1370
1686
1676
0262
1695
1791
1385
1328
0272
1486
1372
0427
0277
0172
1596
1656
1657
1692
1047
0697
1448
1945
0234
0779
1937
0733
1056
1009
1383
0880
1251
1257
1678
1211
2001
0967
1639
1640
2014
0891
0941
1494
1835
1641
1638
1312
0745
1735
1491
0467
0314
0345
0376
0458
1725
1933
1010
0346
0284
1265
1309
0130
0450
0270
0767
0468
0229

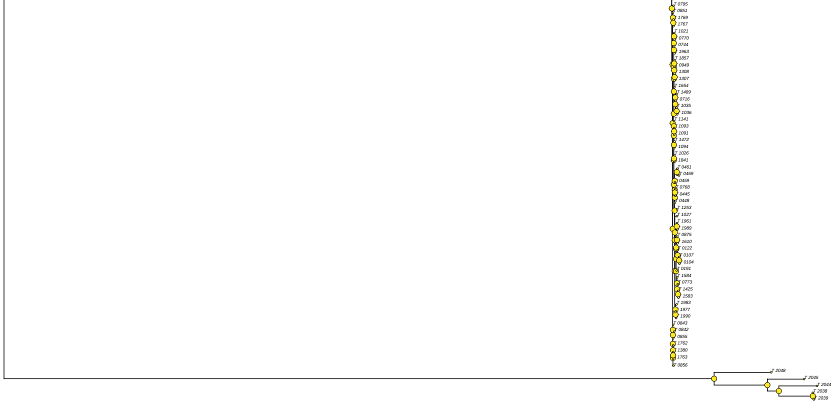
T 0795
0851
1769
1767
T 1021
0770
0744
1963
T 1857
0949
1308
1307
T 1654
T 1489
0716
T 1035
T 1036
T 1141
1093
1091
T 1472
1094
T 1026
1841
T 0461
T 0469
0459
T 0768
0445
0448
T 1253
T 1027
T 1961
1989
T 0875
1610
T 0122
T 0107
0104
T 0191
T 1584
0773
1425
1583
T 1983
1977
1990
T 0843
0842
0855
1762
1380
1763
T 0856
T 2048
T 2045
T 2044
T 2038
2039

H2−T6SS

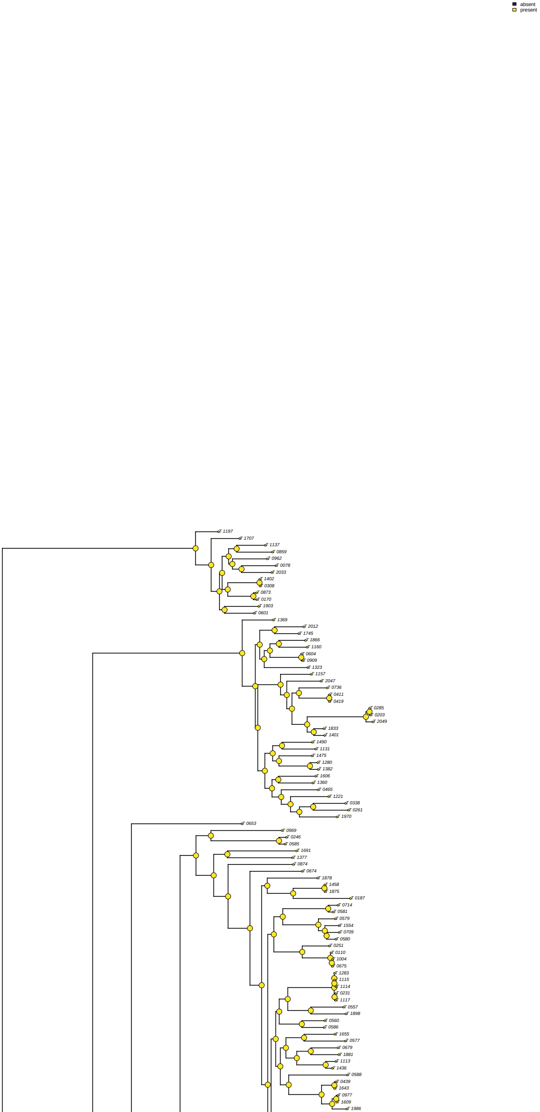

absent
present
T 1197
T 1707
T 1137
T 0859
T 0962
T 0078
T 2033
T 1402
T 0308
T 0873
T 0170
T 1903
T 0601
T 1369
T 2012
T 1745
T 1866
T 1160
T 0604
T 0909
T 1323
T 1157
T 2047
T 0736
T 0411
T 0419
T 0285
T 0203
T 2049
T 1833
T 1401
T 1490
T 1131
T 1475
T 1280
T 1382
T 1606
T 1360
T 0465
T 1221
T 0338
T 0261
T 1970
T 0653
T 0969
T 0246
T 0585
T 1691
T 1377
T 0874
T 0674
T 1878
T 1458
T 1875
T 0187
T 0714
T 0581
T 0579
T 1554
T 0709
T 0580
T 0251
T 0110
T 1004
T 0675
T 1283
T 1115
T 1114
T 0231
T 1117
T 0557
T 1898
T 0560
T 0586
T 1655
T 0577
T 0679
T 1881
T 1113
T 1436
T 0588
T 0439
T 1643
T 0977
T 1609
T 1986

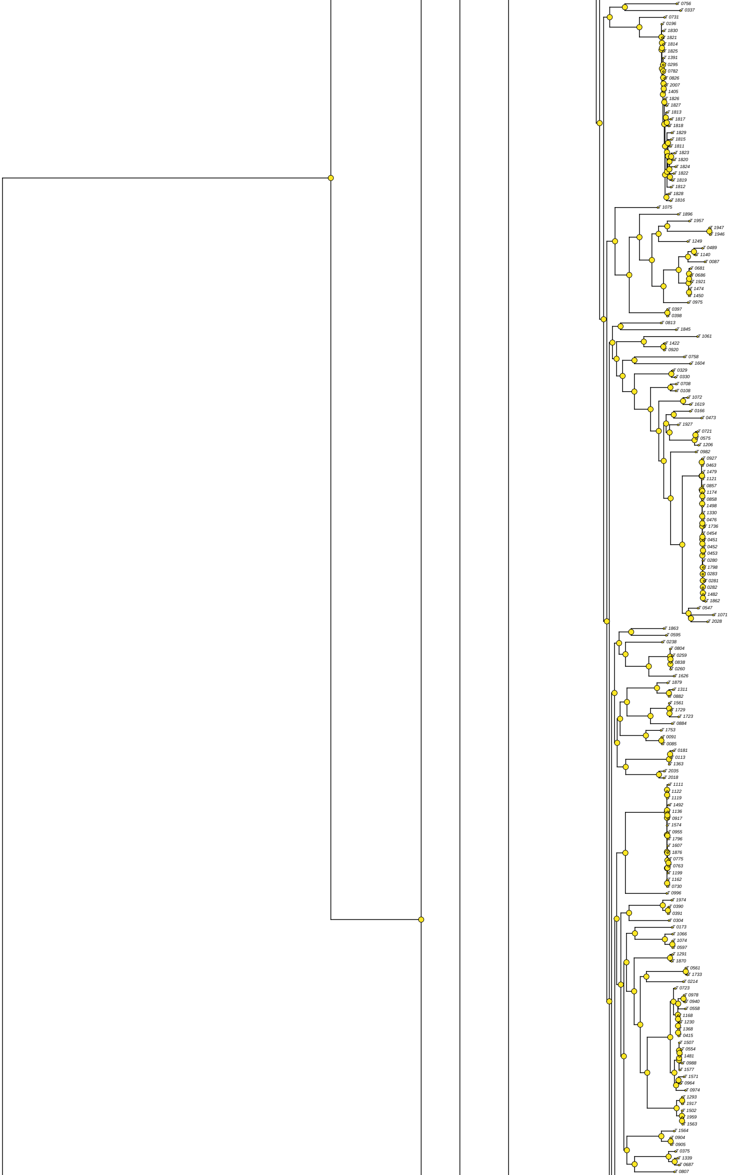
T 0756
T 0337
T 0731
T 0196
T 1830
T 1821
T 1814
T 1825
T 1391
0295
0782
T 0826
T 2007
1405
T 1826
T 1827
T 1813
T 1817
T 1818
T 1829
T 1815
T 1811
T 1823
T 1820
T 1824
T 1822
T 1819
T 1812
T 1828
T 1816
T 1075
T 1896
T 1957
T 1947
T 1946
T 1249
T 0489
T 1140
T 0087
T 0681
T 0686
T 1921
T 1474
T 1450
T 0975
T 0397
T 0398
T 0813
T 1845
T 1061
T 1422
T 0920
T 0758
T 1604
T 0329
T 0330
T 0708
T 0108
T 1072
T 1619
T 0166
T 0473
T 1927
T 0721
T 0575
T 1206
T 0982
T 0927
0463
T 1479
1121
T 0857
1174
T 0858
1498
T 1330
0476
T 1736
T 0454
0451
T 0452
0453
T 0280
1798
0283
T 0281
0282
1482
T 1862
T 0547
T 1071
T 2028
T 1863
T 0595
T 0238
T 0804
T 0259
0838
T 0260
T 1626
T 1879
T 1311
T 0882
T 1561
T 1729
T 1723
T 0884
T 1753
T 0091
T 0085
T 0181
T 0113
T 1363
T 2035
T 2018
T 1111
1122
1119
T 1492
1136
T 0917
T 1574
T 0955
T 1796
T 1607
1876
T 0775
T 0763
T 1199
T 1162
T 0730
T 0996
T 1974
T 0390
T 0391
T 0304
T 0173
T 1066
T 1074
T 0597
T 1291
T 1870
T 0561
T 1733
T 0214
T 0723
T 0978
T 0940
T 0558
T 1168
T 1230
T 1368
T 0415
T 1507
T 0554
T 1481
T 0988
T 1577
T 1571
T 0964
T 0974
T 1293
T 1917
T 1502
T 1959
T 1563
T 1564
T 0904
T 0905
T 0375
T 1339
T 0687
T 0807

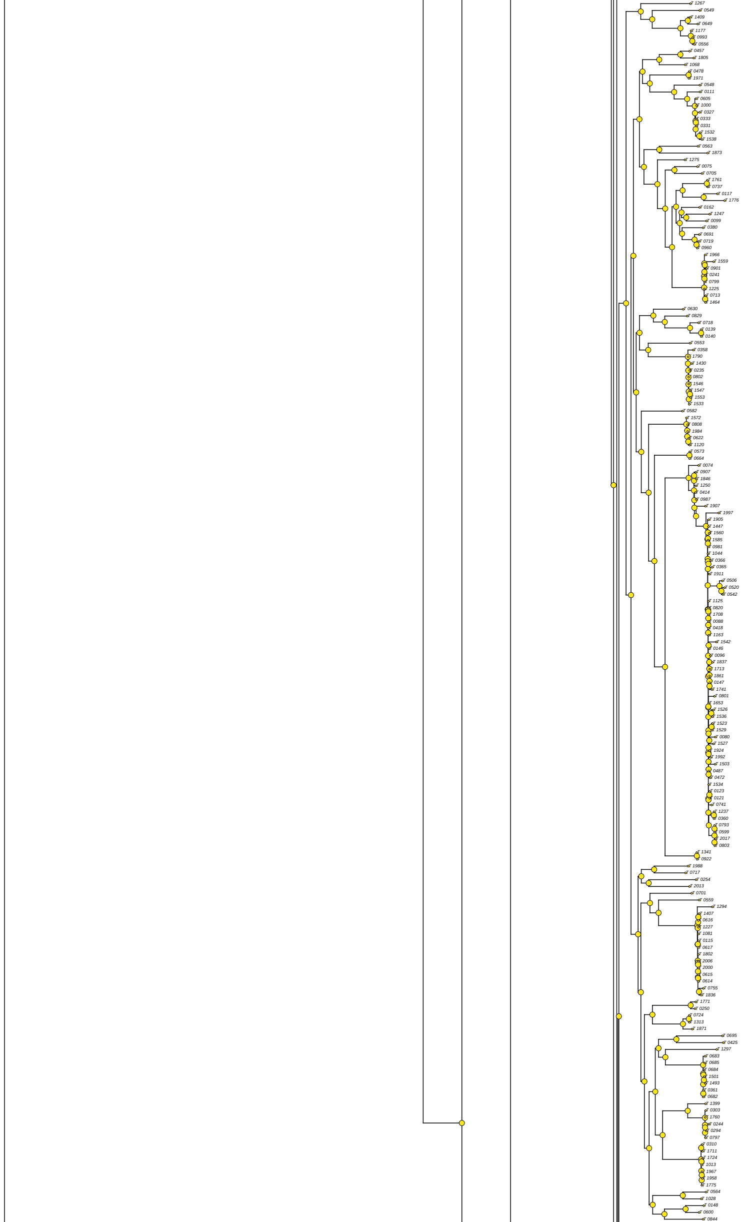

T 1267
T 0549
T 1409
T 0649
T 1177
T 0993
T 0556
T 0457
T 1805
T 1068
T 0478
T 1971
T 0548
T 0111
T 0605
T 1000
T 0327
T 0333
T 0331
T 1532
T 1538
T 0563
T 1873
T 1275
T 0075
T 0705
T 1761
T 0737
T 0117
T 1776
T 0162
T 1247
T 0099
T 0380
T 0691
T 0719
T 0960
T 1966
T 1559
T 0901
T 0241
T 0799
T 1225
T 0713
T 1464
T 0630
T 0829
T 0718
T 0139
T 0140
T 0553
T 0358
T 1790
T 1430
T 0235
T 0802
T 1546
T 1547
T 1553
T 1533
T 0582
T 1572
T 0808
T 1984
T 0622
T 1120
T 0573
T 0664
T 0074
T 0907
T 1846
T 1250
T 0414
T 0987
T 1907
T 1997
T 1905
T 1447
T 1560
T 1585
T 0981
T 1044
T 0366
T 0365
T 1911
T 0506
T 0520
T 0542
T 1125
T 0820
T 1708
T 0088
T 0418
T 1163
T 1542
T 0146
T 0096
T 1837
T 1713
T 1861
T 0147
T 1741
T 0801
T 1653
T 1526
T 1536
T 1523
T 1529
T 0080
T 1527
T 1924
T 1992
T 1503
T 0487
T 0472
T 1534
T 0123
T 0121
T 0741
T 1237
T 0360
T 0793
T 0599
T 2017
T 0803
T 1341
T 0922
T 1988
T 0717
T 0254
T 2013
T 0701
T 0559
T 1294
T 1407
T 0616
T 1227
T 1081
T 0115
T 0617
T 1802
T 2006
T 2000
T 0615
T 0614
T 0755
T 1836
T 1771
T 0250
T 0724
T 1313
T 1871
T 0695
T 0425
T 1297
T 0683
T 0685
T 0684
T 1501
T 1493
T 0361
T 0682
T 1399
T 0303
T 1760
T 0244
T 0294
T 0797
T 0310
T 1711
T 1724
T 1013
T 1967
T 1958
T 1775
T 0564
T 1028
T 0148
T 0600
T 0844

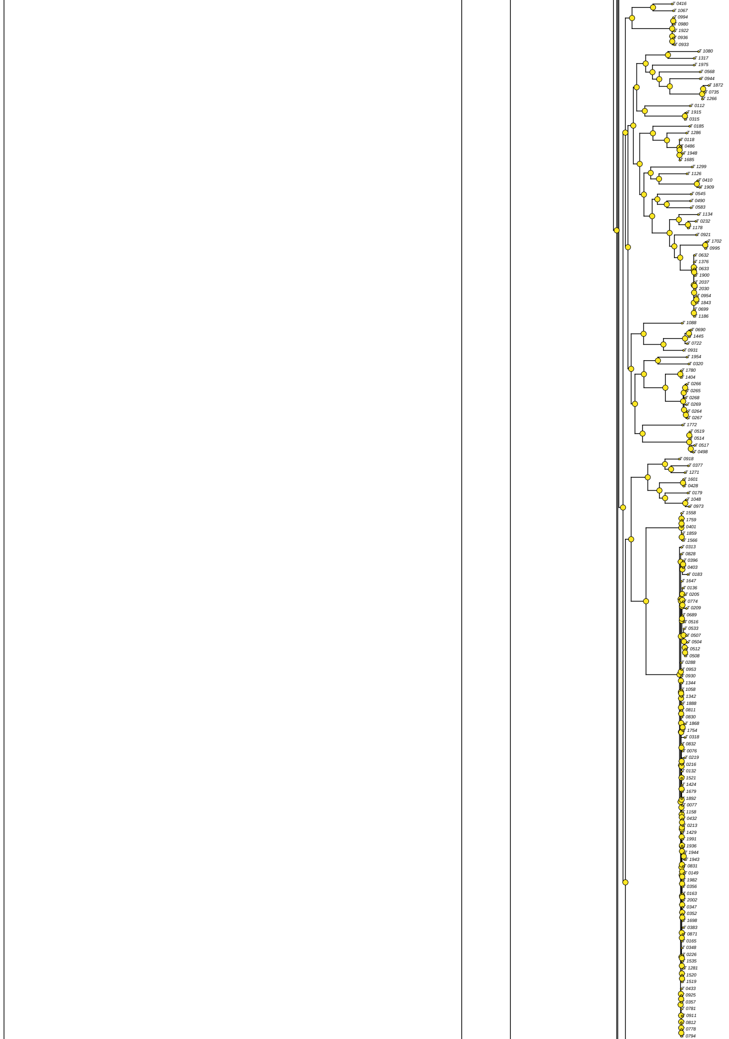
T 0416
T 1067
T 0994
T 0980
T 1922
T 0936
T 0933
T 1080
T 1317
T 1975
T 0568
T 0944
T 1872
T 0735
T 1266
T 0112
T 1915
T 0315
T 0185
T 1286
T 0118
T 0486
T 1948
T 1685
T 1299
T 1126
T 0410
T 1909
T 0545
T 0490
T 0583
T 1134
T 0232
T 1178
T 0921
T 1702
T 0995
T 0632
T 1376
T 0633
T 1900
T 2037
T 2030
T 0954
T 1843
T 0699
T 1186
T 1088
T 0690
T 1445
T 0722
T 0931
T 1954
T 0320
T 1780
T 1404
T 0266
T 0265
T 0268
T 0269
T 0264
T 0267
T 1772
T 0519
T 0514
T 0517
T 0498
T 0918
T 0377
T 1271
T 1601
T 0428
T 0179
T 1048
T 0973
T 1558
T 1759
T 0401
T 1859
T 1566
T 0313
T 0828
T 0396
T 0403
T 0183
T 1647
T 0136
T 0205
T 0774
T 0209
T 0689
T 0516
T 0533
T 0507
T 0504
T 0512
T 0508
T 0288
T 0953
T 0930
T 1344
T 1058
T 1342
T 1888
T 0811
T 0830
T 1868
T 1754
T 0318
T 0832
T 0076
T 0219
T 0216
T 0132
T 1521
T 1424
T 1679
T 1892
T 0077
T 1158
T 0432
T 0213
T 1429
T 1991
T 1936
T 1944
T 1943
T 0831
T 0149
T 1982
T 0356
T 0163
T 2002
T 0347
T 0352
T 1698
T 0383
T 0871
T 0165
T 0348
T 0226
T 1535
T 1281
T 1520
T 1519
T 0433
T 0925
T 0357
T 0781
T 0911
T 0812
T 0778
T 0794

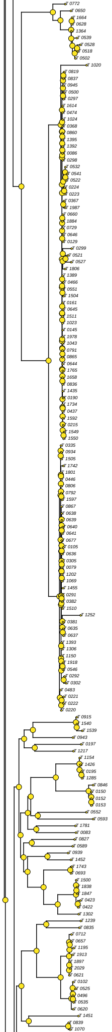

T 0772
T 0650
T 1664
T 0628
T 1364
T 0539
T 0528
T 0518
T 0502
T 1020
T 0819
T 0837
T 0945
T 0500
T 0297
T 1614
T 0474
T 1024
T 0368
T 0860
T 1395
T 1392
T 0086
T 0298
T 0532
T 0541
T 0522
T 0224
T 0223
T 0367
T 1987
T 0660
T 1884
T 0729
T 0646
T 0129
T 0299
T 0521
T 0527
T 1806
T 1389
T 0466
T 0551
T 1504
T 0161
T 0645
T 1511
T 1023
T 0145
T 1978
T 1043
T 0791
T 0865
T 0644
T 1765
T 1658
T 0836
T 1435
T 0190
T 1734
T 0437
T 1592
T 0215
T 1549
T 1550
T 0335
T 0934
T 1505
T 1742
T 1801
T 0446
T 0806
T 0792
T 1597
T 0867
T 0638
T 0639
T 0640
T 0641
T 0677
T 0105
T 0636
T 0305
T 0079
T 1202
T 1069
T 1455
T 0291
T 0382
T 1510
T 1252
T 0381
T 0635
T 0637
T 1393
T 1306
T 1150
T 1918
T 0546
T 0292
T 0302
T 0483
T 0221
T 0222
T 0220
T 0915
T 1540
T 1539
T 0943
T 0197
T 1217
T 1154
T 1426
T 0195
T 1285
T 0846
T 0150
T 0152
T 0153
T 0552
T 0593
T 1781
T 0083
T 0827
T 0589
T 0939
T 1452
T 1743
T 0693
T 1500
T 1838
T 1847
T 0423
T 0422
T 1302
T 1239
T 0835
T 0712
T 0657
T 1195
T 1913
T 1897
T 2029
T 0621
T 0102
T 0525
T 0496
T 0535
T 0620
T 1451
T 0839
T 1070

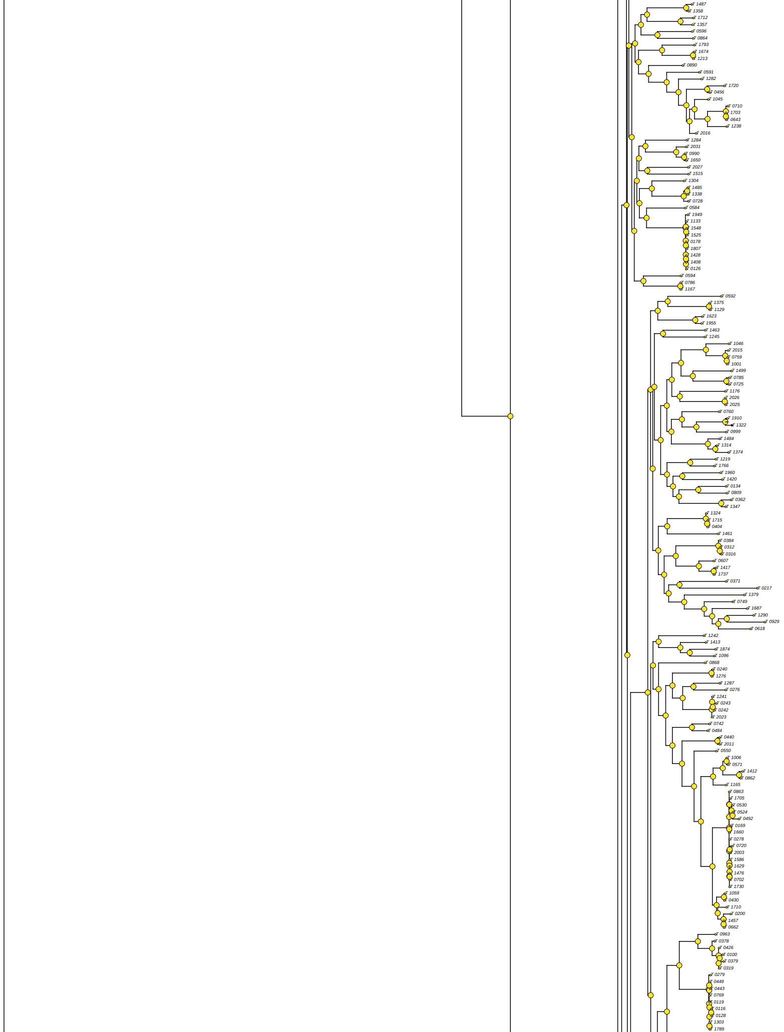

T 1487
T 1358
T 1712
T 1357
T 0596
T 0864
T 1793
T 1674
T 1213
T 0890
T 0591
T 1282
T 1720
T 0456
T 1045
T 0710
T 1703
T 0643
T 1238
T 2016
T 1284
T 2031
T 0990
T 1650
T 2027
T 1515
T 1304
T 1485
T 1338
T 0728
T 0584
T 1949
T 1133
T 1548
T 1525
T 0178
T 1807
T 1428
T 1408
T 0126
T 0594
T 0786
T 1167
T 0592
T 1375
T 1129
T 1623
T 1955
T 1463
T 1245
T 1046
T 2015
T 0759
T 1001
T 1499
T 0785
T 0725
T 1176
T 2026
T 2025
T 0760
T 1910
T 1322
T 0999
T 1484
T 1314
T 1374
T 1219
T 1766
T 1960
T 1420
T 0134
T 0809
T 0362
T 1347
T 1324
T 1715
T 0404
T 1461
T 0384
T 0312
T 0316
T 0607
T 1417
T 1737
T 0371
T 0217
T 1379
T 0749
T 1687
T 1290
T 0929
T 0618
T 1242
T 1413
T 1874
T 1096
T 0868
T 0240
T 1276
T 1287
T 0276
T 1241
T 0243
T 0242
T 2023
T 0742
T 0484
T 0440
T 2011
T 0550
T 1006
T 0571
T 1412
T 0862
T 1165
T 0863
T 1705
T 0530
T 0524
T 0492
T 0169
T 1660
T 0278
T 0720
T 2003
T 1586
T 1629
T 1476
T 0702
T 1730
T 1059
T 0430
T 1710
T 0200
T 1457
T 0662
T 0963
T 0378
T 0426
T 0100
T 0379
T 0319
T 0279
T 0449
T 0443
T 0769
T 0119
T 0116
T 0128
T 1303
T 1789

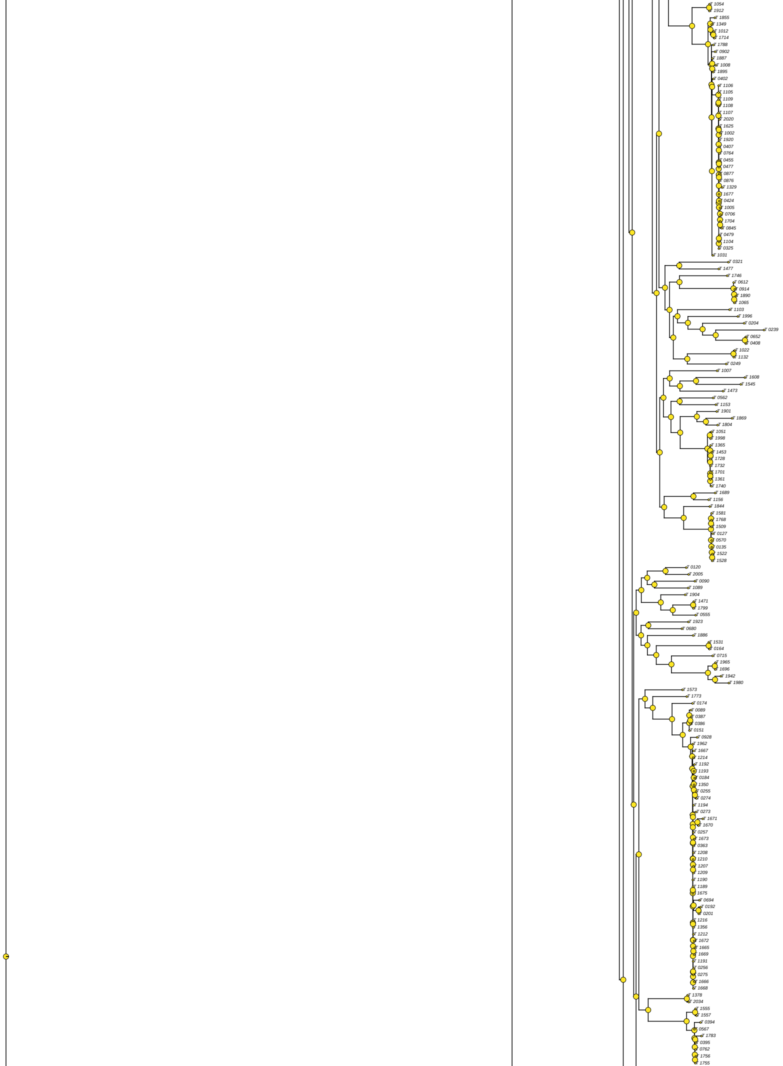
T 1054
T 1912
T 1855
T 1349
T 1012
T 1714
T 1788
T 0902
T 1887
T 1008
T 1895
T 0402
T 1106
T 1105
T 1109
T 1108
T 1107
T 2020
T 1625
T 1002
T 1920
T 0407
T 0764
T 0455
T 0477
T 0877
T 0876
T 1329
T 1677
T 0424
T 1005
T 0706
T 1704
T 0845
T 0479
T 1104
T 0325
T 1031
T 0321
T 1477
T 1746
T 0612
T 0914
T 1890
T 1065
T 1103
T 1996
T 0204
T 0239
T 0652
T 0408
T 1022
T 1132
T 0249
T 1007
T 1608
T 1545
T 1473
T 0562
T 1153
T 1901
T 1869
T 1804
T 1051
T 1998
T 1365
T 1453
T 1728
T 1732
T 1701
T 1361
T 1740
T 1689
T 1156
T 1844
T 1581
T 1768
T 1509
T 0127
T 0570
T 0135
T 1522
T 1528
T 0120
T 2005
T 0090
T 1089
T 1904
T 1471
T 1799
T 0555
T 1923
T 0680
T 1886
T 1531
T 0164
T 0715
T 1965
T 1696
T 1942
T 1980
T 1573
T 1773
T 0174
T 0089
T 0387
T 0386
T 0151
T 0928
T 1962
T 1667
T 1214
T 1192
T 1193
T 0184
T 1350
T 0255
T 0274
T 1194
T 0273
T 1671
T 1670
T 0257
T 1673
T 0363
T 1208
T 1210
T 1207
T 1209
T 1190
T 1189
T 1675
T 0694
T 0192
T 0201
T 1216
T 1356
T 1212
T 1672
T 1665
T 1669
T 1191
T 0256
T 0275
T 1666
T 1668
T 1378
T 2034
T 1555
T 1557
T 0394
T 0567
T 1783
T 0395
T 0762
T 1756
T 1755

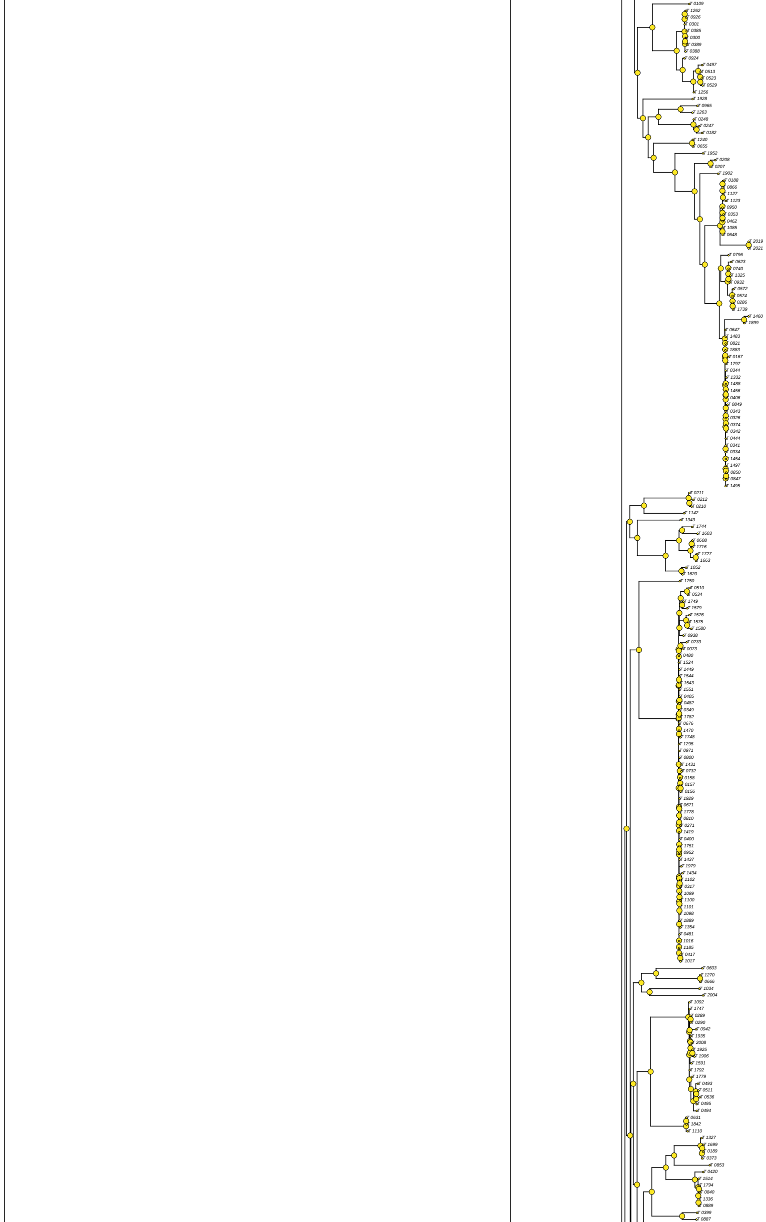
0109
1262
0926
0301
0385
0300
0389
0388
0924
0497
0513
0523
0529
1256
1928
0965
1263
0248
0247
0182
1240
0655
1952
0208
0207
1902
0188
0866
1127
1123
0950
0353
0462
1085
0648
2019
2021
0796
0623
0740
1325
0932
0572
0574
0286
1739
1460
1899
0647
1483
0821
1883
0167
1797
0344
1332
1488
1456
0406
0849
0343
0326
0374
0342
0444
0341
0334
1454
1497
0850
0847
1495
0211
0212
0210
1142
1343
1744
1603
0608
1716
1727
1663
1052
1620
1750
0510
0534
1749
1579
1576
1575
1580
0938
0233
0073
0480
1524
1449
1544
1543
1551
0405
0482
0349
1782
0676
1470
1748
1295
0971
0800
1431
0732
0158
0157
0156
1929
0671
1778
0810
0271
1419
0400
1751
0952
1437
1979
1434
1102
0317
1099
1100
1101
1098
1889
1354
0481
1016
1185
0417
1017
0603
1270
0666
1034
2004
1092
1747
0289
0290
0942
1935
2008
1925
1906
1591
1792
1779
0493
0511
0536
0495
0494
0631
1842
1110
1327
1699
0189
0373
0853
0420
1514
1794
0840
1336
0889
0399
0887

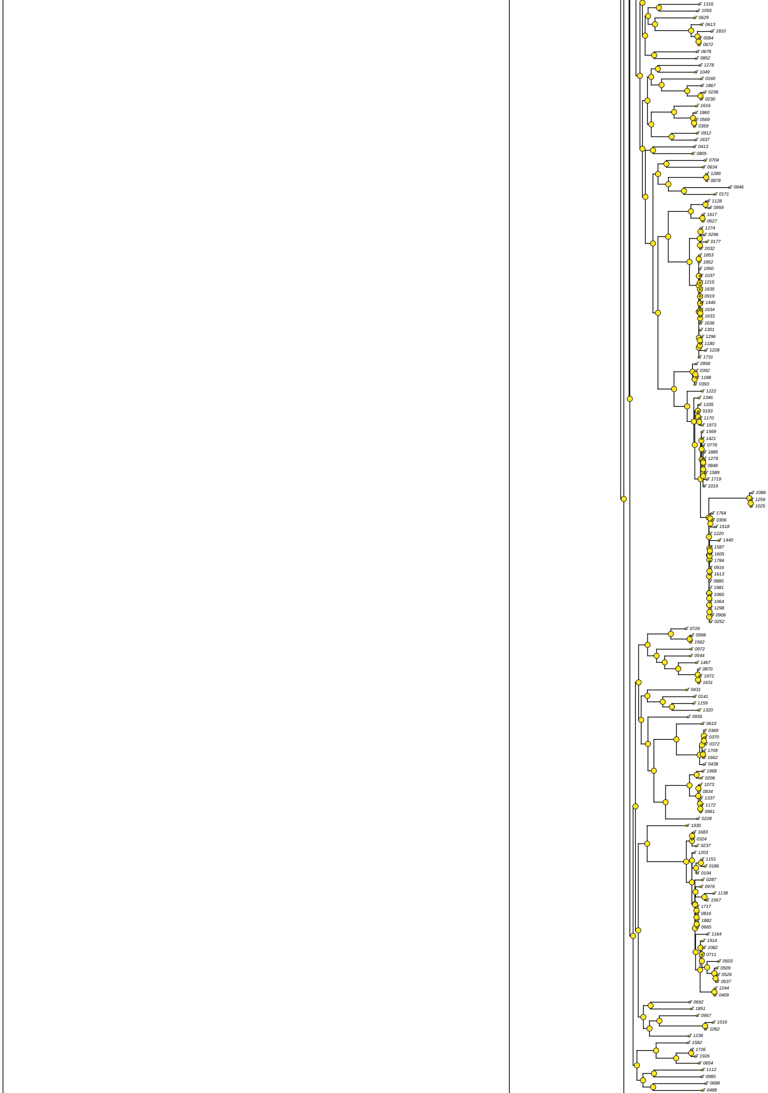

♂ 1316
♂ 1055
♂ 0629
♂ 0613
♂ 1810
♂ 0084
♂ 0672
♂ 0678
♂ 0852
♂ 1278
♂ 1049
♂ 0160
♂ 1867
♂ 0236
♂ 0230
♂ 1616
♂ 1860
♂ 0569
♂ 0359
♂ 0912
♂ 1637
♂ 0413
♂ 0805
♂ 0704
♂ 0634
♂ 1289
♂ 0878
♂ 0946
♂ 0171
♂ 1128
♂ 0959
♂ 1617
♂ 0627
♂ 1274
♂ 0296
♂ 0177
♂ 2032
♂ 1853
♂ 1852
♂ 1950
♂ 1037
♂ 1215
♂ 1635
♂ 0919
♂ 1446
♂ 1634
♂ 1633
♂ 1636
♂ 1301
♂ 1296
♂ 1180
♂ 1228
♂ 1731
♂ 0958
♂ 0392
♂ 1188
♂ 0393
♂ 1222
♂ 1346
♂ 1335
♂ 0193
♂ 1170
♂ 1973
♂ 1569
♂ 1421
♂ 0776
♂ 1885
♂ 1279
♂ 0848
♂ 1589
♂ 1719
♂ 1019
♂ 1086
♂ 1259
♂ 1025
♂ 1764
♂ 0306
♂ 1518
♂ 1220
♂ 1440
♂ 1587
♂ 1605
♂ 1784
♂ 0916
♂ 1613
♂ 0885
♂ 1981
♂ 1060
♂ 1064
♂ 1298
♂ 0906
♂ 0252
♂ 0726
♂ 0998
♂ 1562
♂ 0972
♂ 0544
♂ 1467
♂ 0870
♂ 1972
♂ 1631
♂ 0431
♂ 0141
♂ 1159
♂ 1320
♂ 0935
♂ 0610
♂ 0369
♂ 0370
♂ 0372
♂ 1709
♂ 1662
♂ 0438
♂ 1968
♂ 0206
♂ 1073
♂ 0834
♂ 1337
♂ 1172
♂ 0961
♂ 0228
♂ 1930
♂ 1683
♂ 0324
♂ 0237
♂ 1203
♂ 1151
♂ 0186
♂ 0194
♂ 0287
♂ 0976
♂ 1138
♂ 1567
♂ 1717
♂ 0816
♂ 1882
♂ 0665
♂ 1164
♂ 1914
♂ 1082
♂ 0711
♂ 0503
♂ 0509
♂ 0526
♂ 0537
♂ 1244
♂ 0409
♂ 0692
♂ 1851
♂ 0957
♂ 1516
♂ 1062
♂ 1236
♂ 1582
♂ 1726
♂ 1926
♂ 0654
♂ 1112
♂ 0985
♂ 0688
♂ 0488

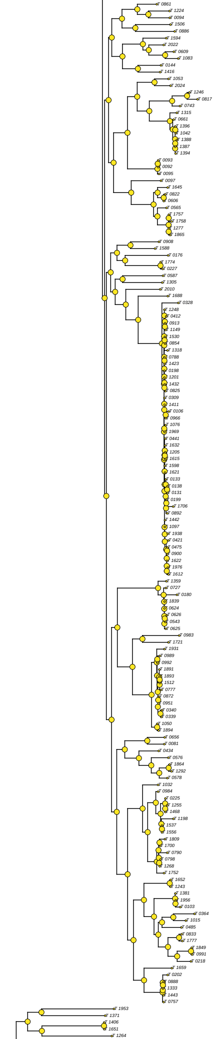
T 0861
T 1224
T 0094
T 1506
T 0886
T 1594
T 2022
T 0609
T 1083
T 0144
T 1416
T 1053
T 2024
T 1246
T 0817
T 0743
T 1315
T 0661
T 1396
T 1042
T 1388
T 1387
T 1394
T 0093
T 0092
T 0095
T 0097
T 1645
T 0822
T 0606
T 0565
T 1757
T 1758
T 1277
T 1865
T 0908
T 1588
T 0176
T 1774
T 0227
T 0587
T 1305
T 2010
T 1688
T 0328
T 1248
T 0412
T 0913
T 1149
T 1530
T 0854
T 1318
T 0788
T 1423
T 0198
T 1201
T 1432
T 0825
T 0309
T 1411
T 0106
T 0966
T 1076
T 1969
T 0441
T 1632
T 1205
T 1615
T 1598
T 1621
T 0133
T 0138
T 0131
T 0199
T 1706
T 0892
T 1442
T 1097
T 1938
T 0421
T 0475
T 0900
T 1622
T 1976
T 1612
T 1359
T 0727
T 0180
T 1839
T 0624
T 0626
T 0543
T 0625
T 0983
T 1721
T 1931
T 0989
T 0992
T 1891
T 1893
T 1512
T 0777
T 0872
T 0951
T 0340
T 0339
T 1050
T 1894
T 0656
T 0081
T 0434
T 0576
T 1864
T 1292
T 0578
T 1032
T 0984
T 0225
T 1255
T 1468
T 1198
T 1537
T 1556
T 1809
T 1700
T 0790
T 0798
T 1268
T 1752
T 1652
T 1243
T 1381
T 1956
T 0103
T 0364
T 1015
T 0485
T 0833
T 1777
T 1849
T 0991
T 0218
T 1659
T 0202
T 0888
T 1333
T 1443
T 0757
T 1953
T 1371
T 1406
T 1651
T 1264

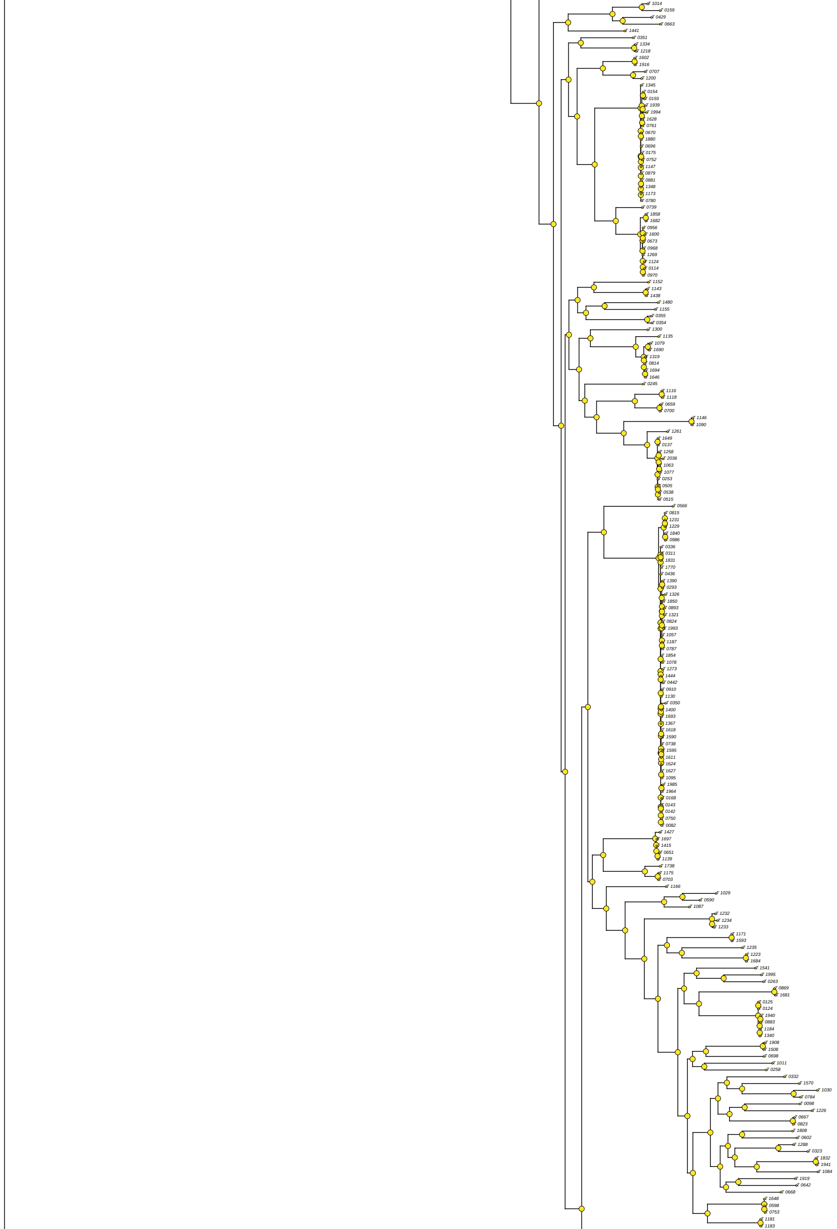
1014
0159
0429
0663
1441
0351
1334
1218
1602
1916
0707
1200
1345
0154
0155
1939
1994
1628
0761
0670
1880
0696
0175
0752
1147
0879
0881
1348
1173
0780
0739
1858
1682
0956
1600
0673
0968
1269
1124
0114
0970
1152
1143
1438
1480
1155
0355
0354
1300
1135
1079
1690
1319
0814
1694
1646
0245
1116
1118
0659
0700
1146
1090
1261
1649
0137
1258
2036
1063
1077
0253
0505
0538
0515
0566
0815
1231
1229
1840
0986
0336
0311
1831
1770
0436
1390
0293
1326
1850
0893
1321
0824
1993
1057
1187
0787
1854
1078
1273
1444
0442
0910
1130
0350
1400
1693
1367
1618
1590
0738
1595
1611
1624
1627
1095
1985
1964
0168
0143
0142
0750
0082
1427
1697
1415
0651
1139
1738
1175
0703
1166
1029
0590
1087
1232
1234
1233
1171
1593
1235
1223
1684
1541
1995
0263
0869
1681
0125
0124
1940
0883
1184
1340
1908
1508
0698
1011
0258
0332
1570
1030
0784
0098
1226
0667
0823
1808
0602
1288
0323
1832
1941
1084
1919
0642
0668
1648
0598
0753
1181
1183

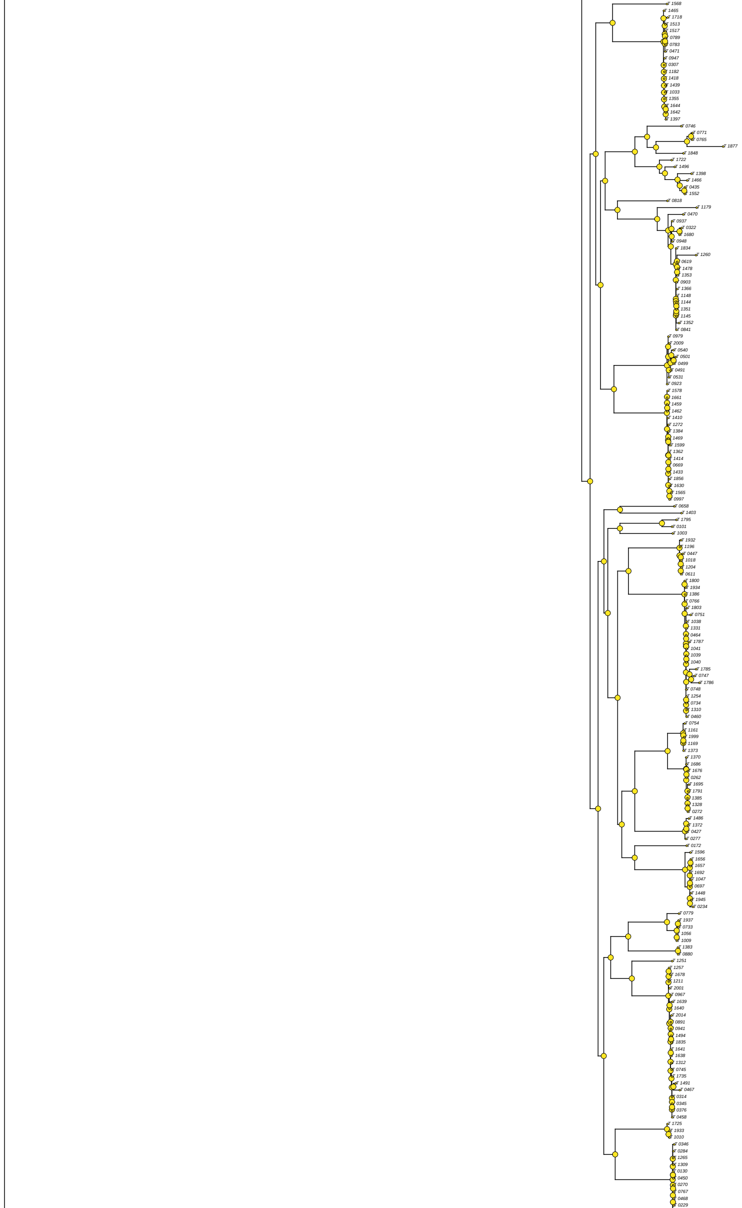
T 1568
T 1465
T 1718
T 1513
T 1517
T 0789
T 0783
T 0471
T 0947
T 0307
T 1182
T 1418
T 1439
T 1033
T 1355
T 1644
T 1642
T 1397
T 0746
T 0771
T 0765
T 1877
T 1848
T 1722
T 1496
T 1398
T 1466
T 0435
T 1552
T 0818
T 1179
T 0470
T 0937
T 0322
T 1680
T 0948
T 1834
T 1260
T 0619
T 1478
T 1353
T 0903
T 1366
T 1148
T 1144
T 1351
T 1145
T 1352
T 0841
T 0979
T 2009
T 0540
T 0501
T 0499
T 0491
T 0531
T 0923
T 1578
T 1661
T 1459
T 1462
T 1410
T 1272
T 1384
T 1469
T 1599
T 1362
T 1414
T 0669
T 1433
T 1856
T 1630
T 1565
T 0997
T 0658
T 1403
T 1795
T 0101
T 1003
T 1932
T 1196
T 0447
T 1018
T 1204
T 0611
T 1800
T 1934
T 1386
T 0766
T 1803
T 0751
T 1038
T 1331
T 0464
T 1787
T 1041
T 1039
T 1040
T 1785
T 0747
T 1786
T 0748
T 1254
T 0734
T 1310
T 0460
T 0754
T 1161
T 1999
T 1169
T 1373
T 1370
T 1686
T 1676
T 0262
T 1695
T 1791
T 1385
T 1328
T 0272
T 1486
T 1372
T 0427
T 0277
T 0172
T 1596
T 1656
T 1657
T 1692
T 1047
T 0697
T 1448
T 1945
T 0234
T 0779
T 1937
T 0733
T 1056
T 1009
T 1383
T 0880
T 1251
T 1257
T 1678
T 1211
T 2001
T 0967
T 1639
T 1640
T 2014
T 0891
T 0941
T 1494
T 1835
T 1641
T 1638
T 1312
T 0745
T 1735
T 1491
T 0467
T 0314
T 0345
T 0376
T 0458
T 1725
T 1933
T 1010
T 0346
T 0284
T 1265
T 1309
T 0130
T 0450
T 0270
T 0767
T 0468
T 0229

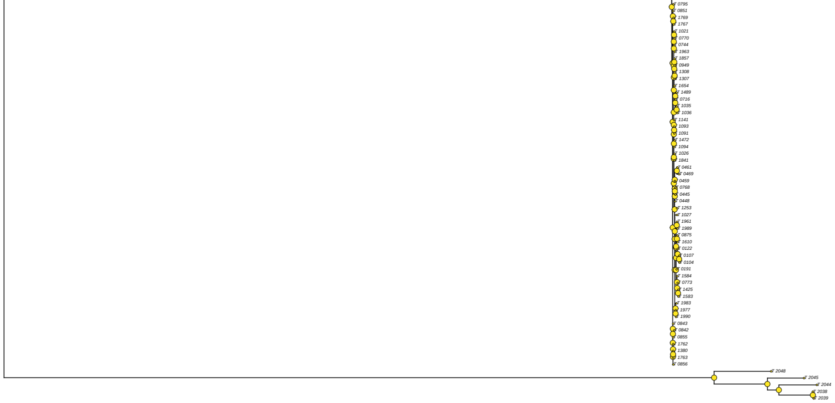
T 0795
T 0851
T 1769
T 1767
T 1021
T 0770
T 0744
T 1963
T 1857
T 0949
T 1308
T 1307
T 1654
T 1489
T 0716
T 1035
T 1036
T 1141
T 1093
T 1091
T 1472
T 1094
T 1026
T 1841
T 0461
T 0469
T 0459
T 0768
T 0445
T 0448
T 1253
T 1027
T 1961
T 1989
T 0875
T 1610
T 0122
T 0107
T 0104
T 0191
T 1584
T 0773
T 1425
T 1583
T 1983
T 1977
T 1990
T 0843
T 0842
T 0855
T 1762
T 1380
T 1763
T 0856
T 2048
T 2045
T 2044
T 2038
T 2039

H3−T6SS

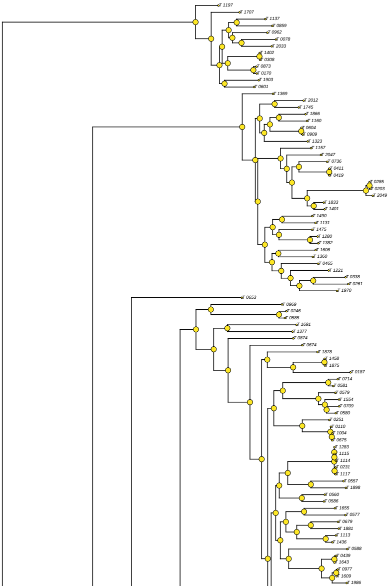

absent
present
T 1197
T 1707
T 1137
T 0859
T 0962
T 0078
T 2033
T 1402
T 0308
T 0873
T 0170
T 1903
T 0601
T 1369
T 2012
T 1745
T 1866
T 1160
T 0604
T 0909
T 1323
T 1157
T 2047
T 0736
T 0411
T 0419
T 0285
T 0203
T 2049
T 1833
T 1401
T 1490
T 1131
T 1475
T 1280
T 1382
T 1606
T 1360
T 0465
T 1221
T 0338
T 0261
T 1970
T 0653
T 0969
T 0246
T 0585
T 1691
T 1377
T 0874
T 0674
T 1878
T 1458
T 1875
T 0187
T 0714
T 0581
T 0579
T 1554
T 0709
T 0580
T 0251
T 0110
T 1004
T 0675
T 1283
T 1115
T 1114
T 0231
T 1117
T 0557
T 1898
T 0560
T 0586
T 1655
T 0577
T 0679
T 1881
T 1113
T 1436
T 0588
T 0439
T 1643
T 0977
T 1609
T 1986

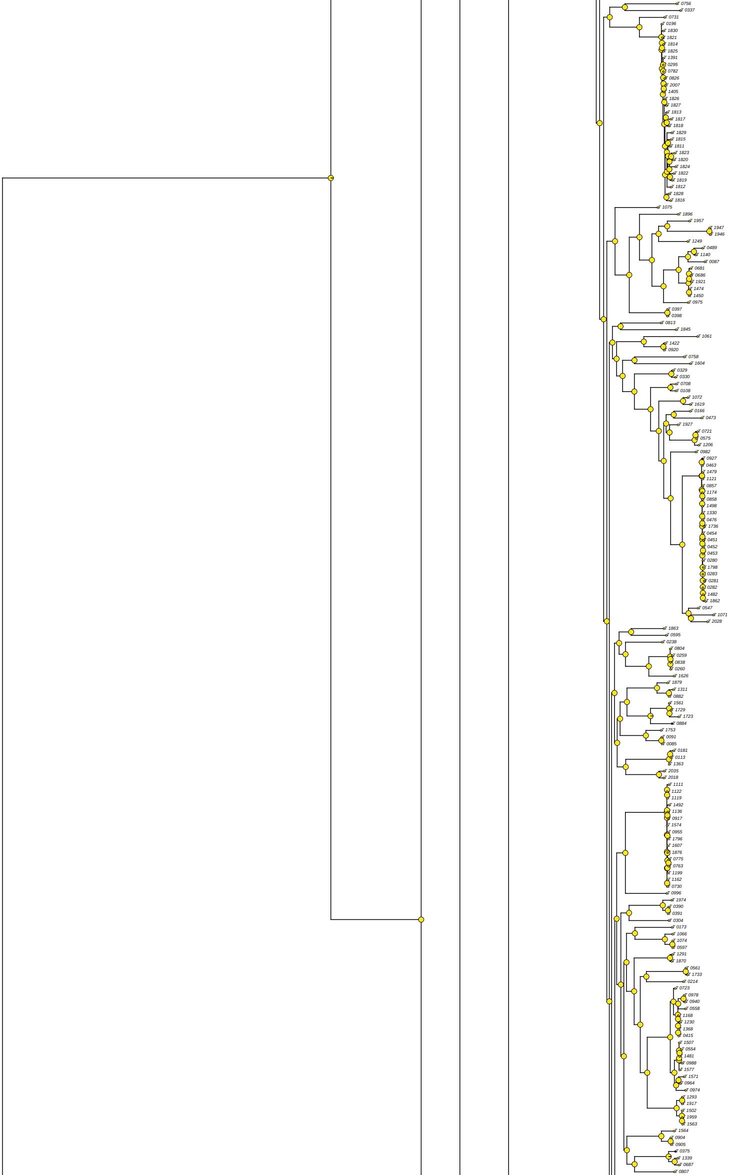

T 0756
T 0337
T 0731
T 0196
T 1830
T 1821
T 1814
T 1825
T 1391
T 0295
T 0782
T 0826
T 2007
T 1405
T 1826
T 1827
T 1813
T 1817
T 1818
T 1829
T 1815
T 1811
T 1823
T 1820
T 1824
T 1822
T 1819
T 1812
T 1828
T 1816
T 1075
T 1896
T 1957
T 1947
T 1946
T 1249
T 0489
T 1140
T 0087
T 0681
T 0686
T 1921
T 1474
T 1450
T 0975
T 0397
T 0398
T 0813
T 1845
T 1061
T 1422
T 0920
T 0758
T 1604
T 0329
T 0330
T 0708
T 0108
T 1072
T 1619
T 0166
T 0473
T 1927
T 0721
T 0575
T 1206
T 0982
T 0927
T 0463
T 1479
T 1121
T 0857
T 1174
T 0858
T 1498
T 1330
T 0476
T 1736
T 0454
T 0451
T 0452
T 0453
T 0280
T 1798
T 0283
T 0281
T 0282
T 1482
T 1862
T 0547
T 1071
T 2028
T 1863
T 0595
T 0238
T 0804
T 0259
T 0838
T 0260
T 1626
T 1879
T 1311
T 0882
T 1561
T 1729
T 1723
T 0884
T 1753
T 0091
T 0085
T 0181
T 0113
T 1363
T 2035
T 2018
T 1111
T 1122
T 1119
T 1492
T 1136
T 0917
T 1574
T 0955
T 1796
T 1607
T 1876
T 0775
T 0763
T 1199
T 1162
T 0730
T 0996
T 1974
T 0390
T 0391
T 0304
T 0173
T 1066
T 1074
T 0597
T 1291
T 1870
T 0561
T 1733
T 0214
T 0723
T 0978
T 0940
T 0558
T 1168
T 1230
T 1368
T 0415
T 1507
T 0554
T 1481
T 0988
T 1577
T 1571
T 0964
T 0974
T 1293
T 1917
T 1502
T 1959
T 1563
T 1564
T 0904
T 0905
T 0375
T 1339
T 0687
T 0807

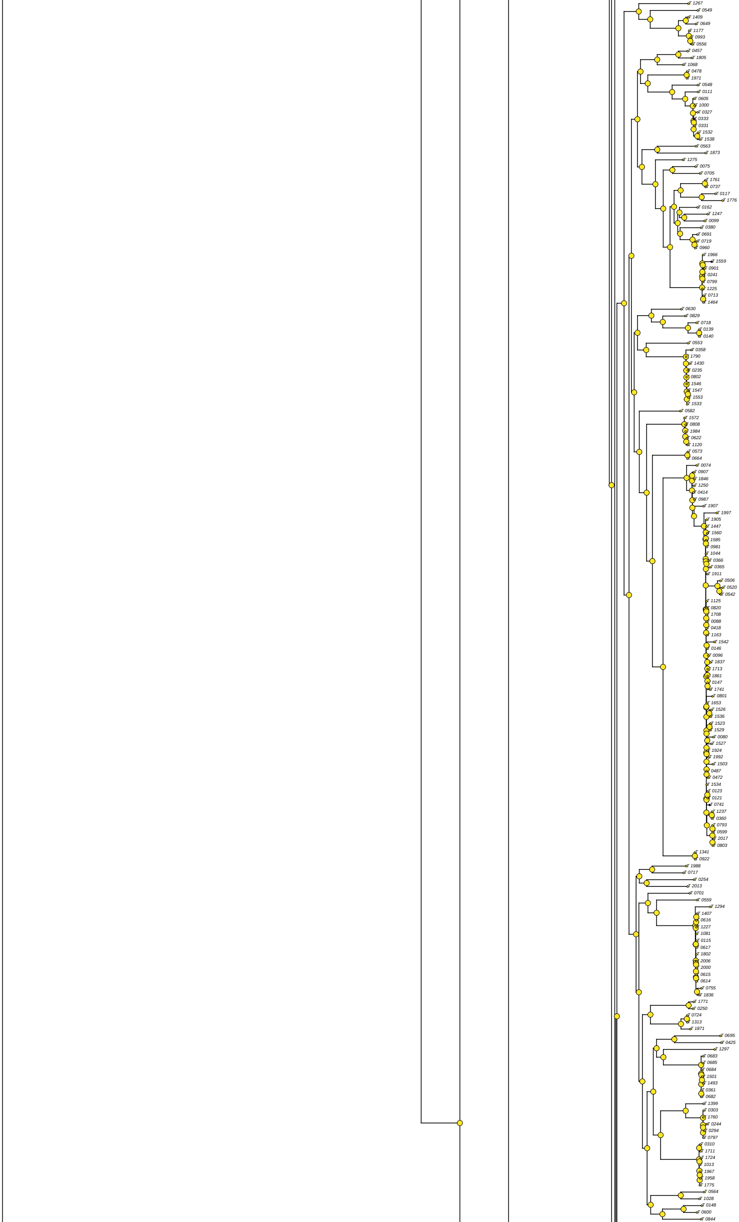
T 1267
T 0549
T 1409
T 0649
T 1177
T 0993
T 0556
T 0457
T 1805
T 1068
T 0478
T 1971
T 0548
T 0111
T 0605
T 1000
T 0327
T 0333
T 0331
T 1532
T 1538
T 0563
T 1873
T 1275
T 0075
T 0705
T 1761
T 0737
T 0117
T 1776
T 0162
T 1247
T 0099
T 0380
T 0691
T 0719
T 0960
T 1966
T 1559
T 0901
T 0241
T 0799
T 1225
T 0713
T 1464
T 0630
T 0829
T 0718
T 0139
T 0140
T 0553
T 0358
T 1790
T 1430
T 0235
T 0802
T 1546
T 1547
T 1553
T 1533
T 0582
T 1572
T 0808
T 1984
T 0622
T 1120
T 0573
T 0664
T 0074
T 0907
T 1846
T 1250
T 0414
T 0987
T 1907
T 1997
T 1905
T 1447
T 1560
T 1585
T 0981
T 1044
T 0366
T 0365
T 1911
T 0506
T 0520
T 0542
T 1125
T 0820
T 1708
T 0088
T 0418
T 1163
T 1542
T 0146
T 0096
T 1837
T 1713
T 1861
T 0147
T 1741
T 0801
T 1653
T 1526
T 1536
T 1523
T 1529
T 0080
T 1527
T 1924
T 1992
T 1503
T 0487
T 0472
T 1534
T 0123
T 0121
T 0741
T 1237
T 0360
T 0793
T 0599
T 2017
T 0803
T 1341
T 0922
T 1988
T 0717
T 0254
T 2013
T 0701
T 0559
T 1294
T 1407
T 0616
T 1227
T 1081
T 0115
T 0617
T 1802
T 2006
T 2000
T 0615
T 0614
T 0755
T 1836
T 1771
T 0250
T 0724
T 1313
T 1871
T 0695
T 0425
T 1297
T 0683
T 0685
T 0684
T 1501
T 1493
T 0361
T 0682
T 1399
T 0303
T 1760
T 0244
T 0294
T 0797
T 0310
T 1711
T 1724
T 1013
T 1967
T 1958
T 1775
T 0564
T 1028
T 0148
T 0600
T 0844

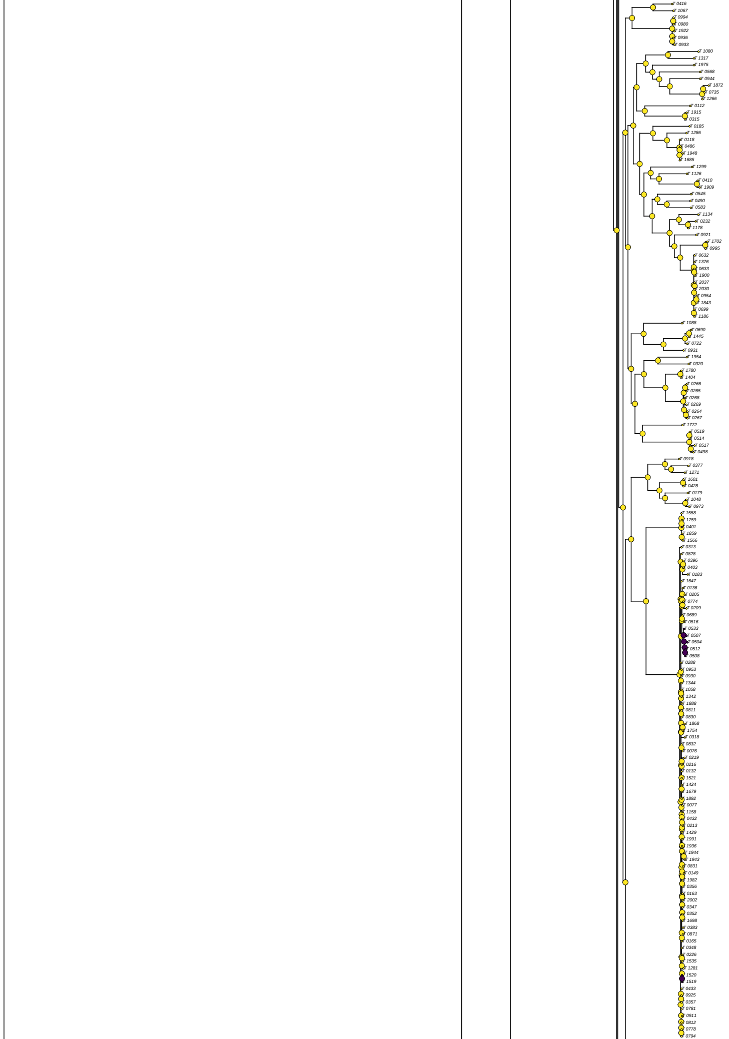
T 0416
T 1067
T 0994
T 0980
T 1922
T 0936
T 0933
T 1080
T 1317
T 1975
T 0568
T 0944
T 1872
T 0735
T 1266
T 0112
T 1915
T 0315
T 0185
T 1286
T 0118
T 0486
T 1948
T 1685
T 1299
T 1126
T 0410
T 1909
T 0545
T 0490
T 0583
T 1134
T 0232
T 1178
T 0921
T 1702
T 0995
T 0632
T 1376
T 0633
T 1900
T 2037
T 2030
T 0954
T 1843
T 0699
T 1186
T 1088
T 0690
T 1445
T 0722
T 0931
T 1954
T 0320
T 1780
T 1404
T 0266
T 0265
T 0268
T 0269
T 0264
T 0267
T 1772
T 0519
T 0514
T 0517
T 0498
T 0918
T 0377
T 1271
T 1601
T 0428
T 0179
T 1048
T 0973
T 1558
T 1759
T 0401
T 1859
T 1566
T 0313
T 0828
T 0396
T 0403
T 0183
T 1647
T 0136
T 0205
T 0774
T 0209
T 0689
T 0516
T 0533
T 0507
T 0504
T 0512
T 0508
T 0288
T 0953
T 0930
T 1344
T 1058
T 1342
T 1888
T 0811
T 0830
T 1868
T 1754
T 0318
T 0832
T 0076
T 0219
T 0216
T 0132
T 1521
T 1424
T 1679
T 1892
T 0077
T 1158
T 0432
T 0213
T 1429
T 1991
T 1936
T 1944
T 1943
T 0831
T 0149
T 1982
T 0356
T 0163
T 2002
T 0347
T 0352
T 1698
T 0383
T 0871
T 0165
T 0348
T 0226
T 1535
T 1281
T 1520
T 1519
T 0433
T 0925
T 0357
T 0781
T 0911
T 0812
T 0778
T 0794

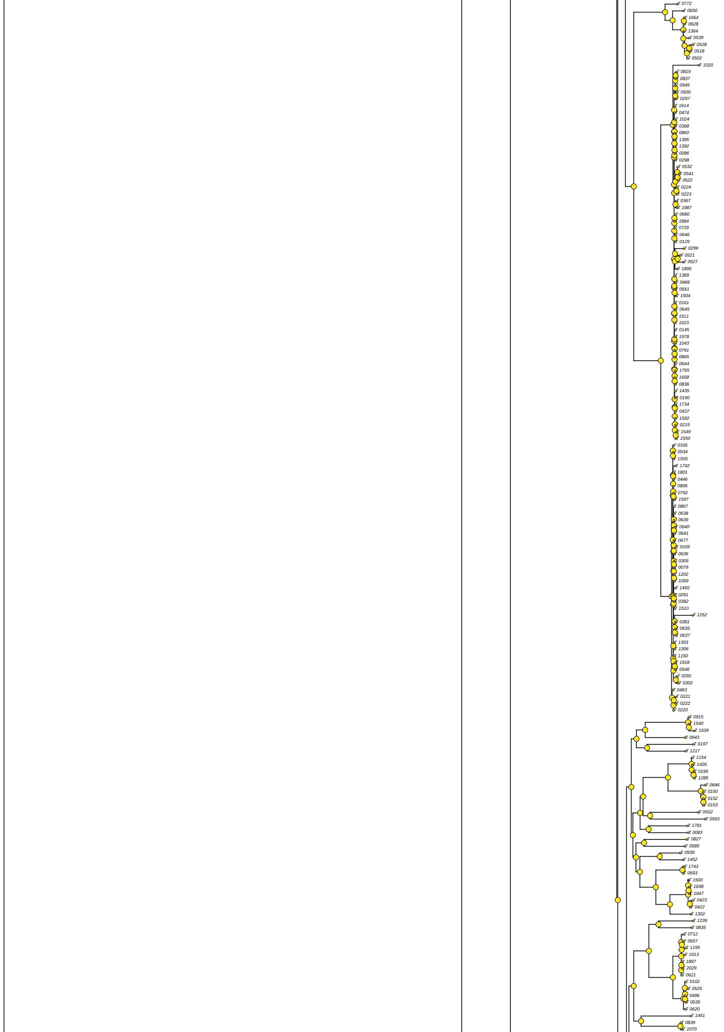
0772
0650
1664
0628
1364
0539
0528
0518
0502
1020
0819
0837
0945
0500
0297
1614
0474
1024
0368
0860
1395
1392
0086
0298
0532
0541
0522
0224
0223
0367
1987
0660
1884
0729
0646
0129
0299
0521
0527
1806
1389
0466
0551
1504
0161
0645
1511
1023
0145
1978
1043
0791
0865
0644
1765
1658
0836
1435
0190
1734
0437
1592
0215
1549
1550
0335
0934
1505
1742
1801
0446
0806
0792
1597
0867
0638
0639
0640
0641
0677
0105
0636
0305
0079
1202
1069
1455
0291
0382
1510
1252
0381
0635
0637
1393
1306
1150
1918
0546
0292
0302
0483
0221
0222
0220
0915
1540
1539
0943
0197
1217
1154
1426
0195
1285
0846
0150
0152
0153
0552
0593
1781
0083
0827
0589
0939
1452
1743
0693
1500
1838
1847
0423
0422
1302
1239
0835
0712
0657
1195
1913
1897
2029
0621
0102
0525
0496
0535
0620
1451
0839
1070

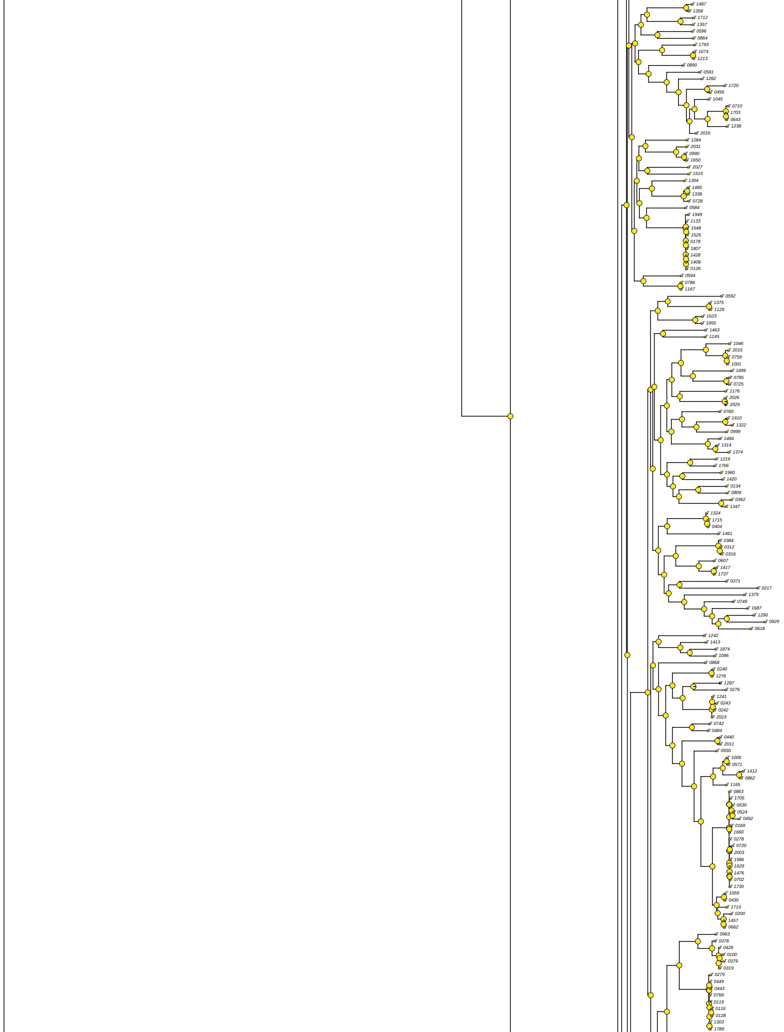

T 1487
T 1358
T 1712
T 1357
T 0596
T 0864
T 1793
T 1674
T 1213
T 0890
T 0591
T 1282
T 1720
T 0456
T 1045
T 0710
T 1703
T 0643
T 1238
T 2016
T 1284
T 2031
T 0990
T 1650
T 2027
T 1515
T 1304
T 1485
T 1338
T 0728
T 0584
T 1949
T 1133
T 1548
T 1525
T 0178
T 1807
T 1428
T 1408
T 0126
T 0594
T 0786
T 1167
T 0592
T 1375
T 1129
T 1623
T 1955
T 1463
T 1245
T 1046
T 2015
T 0759
T 1001
T 1499
T 0785
T 0725
T 1176
T 2026
T 2025
T 0760
T 1910
T 1322
T 0999
T 1484
T 1314
T 1374
T 1219
T 1766
T 1960
T 1420
T 0134
T 0809
T 0362
T 1347
T 1324
T 1715
T 0404
T 1461
T 0384
T 0312
T 0316
T 0607
T 1417
T 1737
T 0371
T 0217
T 1379
T 0749
T 1687
T 1290
T 0929
T 0618
T 1242
T 1413
T 1874
T 1096
T 0868
T 0240
T 1276
T 1287
T 0276
T 1241
T 0243
T 0242
T 2023
T 0742
T 0484
T 0440
T 2011
T 0550
T 1006
T 0571
T 1412
T 0862
T 1165
T 0863
T 1705
T 0530
T 0524
T 0492
T 0169
T 1660
T 0278
T 0720
T 2003
T 1586
T 1629
T 1476
T 0702
T 1730
T 1059
T 0430
T 1710
T 0200
T 1457
T 0662
T 0963
T 0378
T 0426
T 0100
T 0379
T 0319
T 0279
T 0449
T 0443
T 0769
T 0119
T 0116
T 0128
T 1303
T 1789

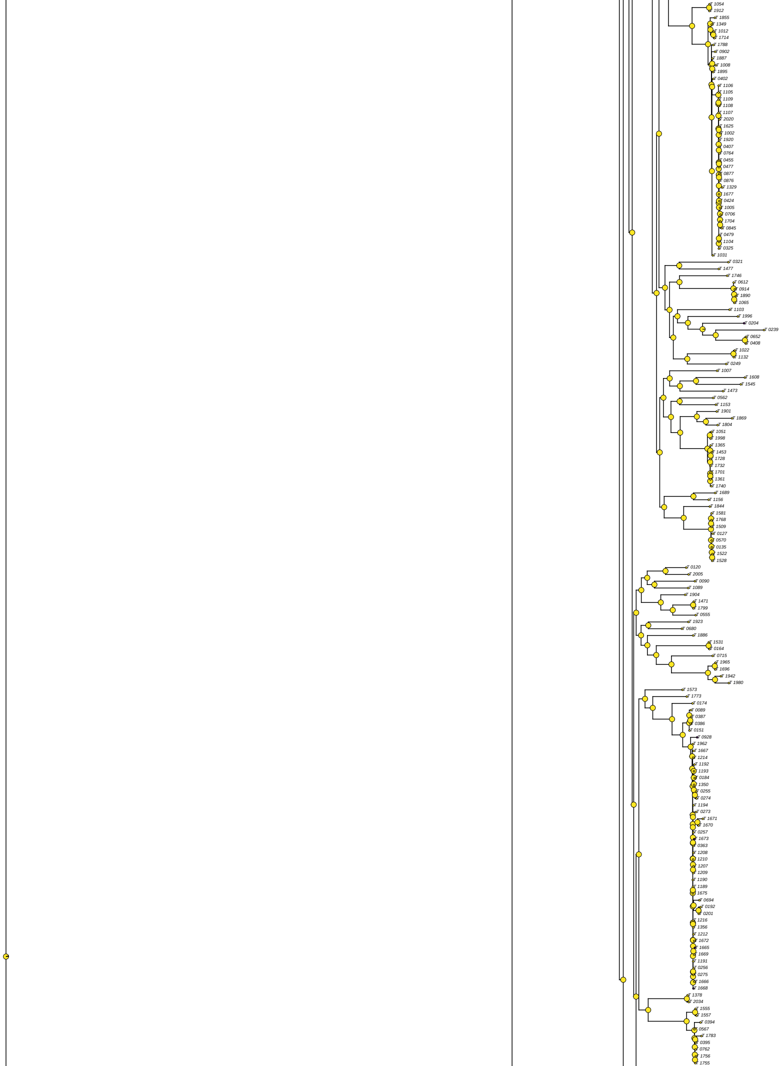

T 1054
1912
T 1855
T 1349
T 1012
T 1714
T 1788
T 0902
T 1887
T 1008
T 1895
T 0402
T 1106
T 1105
T 1109
T 1108
T 1107
T 2020
T 1625
T 1002
T 1920
0407
T 0764
T 0455
0477
0877
T 0876
T 1329
1677
0424
1005
T 0706
1704
T 0845
T 0479
1104
0325
T 1031
T 0321
T 1477
T 1746
T 0612
T 0914
T 1890
1065
T 1103
T 1996
T 0204
T 0239
T 0652
0408
T 1022
1132
T 0249
T 1007
T 1608
T 1545
T 1473
T 0562
T 1153
T 1901
T 1869
T 1804
T 1051
T 1998
T 1365
T 1453
1728
T 1732
1701
1361
T 1740
T 1689
T 1156
T 1844
T 1581
1768
1509
T 0127
0570
0135
T 1522
1528
T 0120
T 2005
T 0090
T 1089
T 1904
T 1471
T 1799
T 0555
T 1923
T 0680
T 1886
T 1531
T 0164
T 0715
T 1965
T 1696
T 1942
T 1980
T 1573
T 1773
T 0174
T 0089
T 0387
T 0386
T 0151
T 0928
T 1962
T 1667
T 1214
T 1192
T 1193
T 0184
T 1350
T 0255
T 0274
T 1194
T 0273
T 1671
T 1670
T 0257
T 1673
T 0363
T 1208
1210
T 1207
T 1209
T 1190
T 1189
T 1675
T 0694
T 0192
T 0201
T 1216
T 1356
T 1212
T 1672
T 1665
T 1669
T 1191
T 0256
T 0275
T 1666
T 1668
T 1378
T 2034
T 1555
T 1557
T 0394
T 0567
T 1783
T 0395
T 0762
T 1756
T 1755

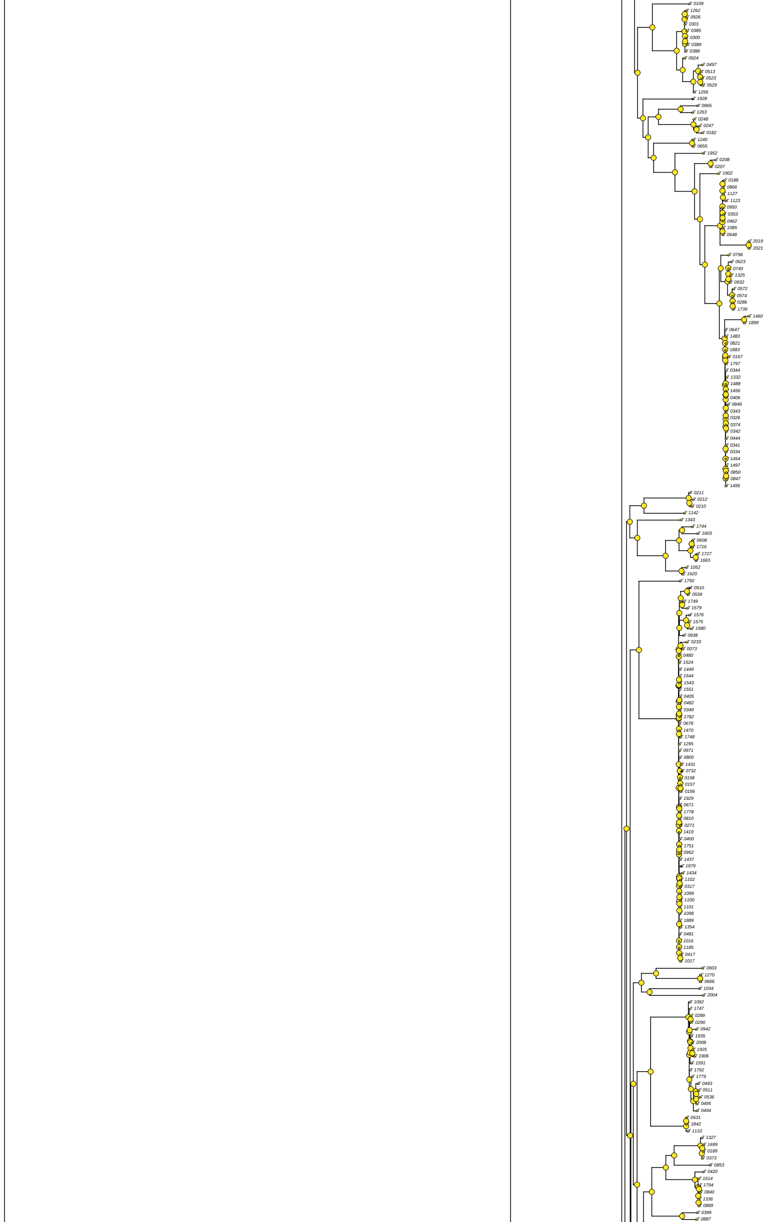
T 0109
T 1262
T 0926
T 0301
T 0385
T 0300
T 0389
T 0388
T 0924
T 0497
T 0513
T 0523
T 0529
T 1256
T 1928
T 0965
T 1263
T 0248
T 0247
T 0182
T 1240
T 0655
T 1952
T 0208
T 0207
T 1902
T 0188
0866
T 1127
T 1123
0950
T 0353
T 0462
T 1085
T 0648
T 2019
T 2021
T 0796
T 0623
T 0740
T 1325
T 0932
T 0572
0574
T 0286
T 1739
T 1460
T 1899
T 0647
T 1483
T 0821
T 1883
T 0167
T 1797
T 0344
T 1332
T 1488
T 1456
T 0406
T 0849
T 0343
T 0326
T 0374
T 0342
T 0444
T 0341
T 0334
T 1454
T 1497
T 0850
T 0847
T 1495
T 0211
T 0212
T 0210
T 1142
T 1343
T 1744
T 1603
T 0608
T 1716
T 1727
T 1663
T 1052
T 1620
T 1750
T 0510
T 0534
T 1749
T 1579
T 1576
T 1575
T 1580
T 0938
T 0233
T 0073
0480
T 1524
T 1449
T 1544
T 1543
T 1551
T 0405
0482
T 0349
T 1782
T 0676
T 1470
T 1748
T 1295
T 0971
T 0800
T 1431
T 0732
T 0158
T 0157
T 0156
T 1929
T 0671
T 1778
T 0810
T 0271
T 1419
T 0400
T 1751
T 0952
T 1437
T 1979
T 1434
T 1102
T 0317
T 1099
T 1100
T 1101
T 1098
T 1889
T 1354
T 0481
T 1016
T 1185
T 0417
T 1017
T 0603
T 1270
T 0666
T 1034
T 2004
T 1092
T 1747
T 0289
T 0290
T 0942
T 1935
T 2008
T 1925
T 1906
T 1591
T 1792
T 1779
T 0493
T 0511
T 0536
T 0495
T 0494
T 0631
T 1842
T 1110
T 1327
T 1699
T 0189
T 0373
T 0853
T 0420
T 1514
T 1794
T 0840
T 1336
T 0889
T 0399
T 0887

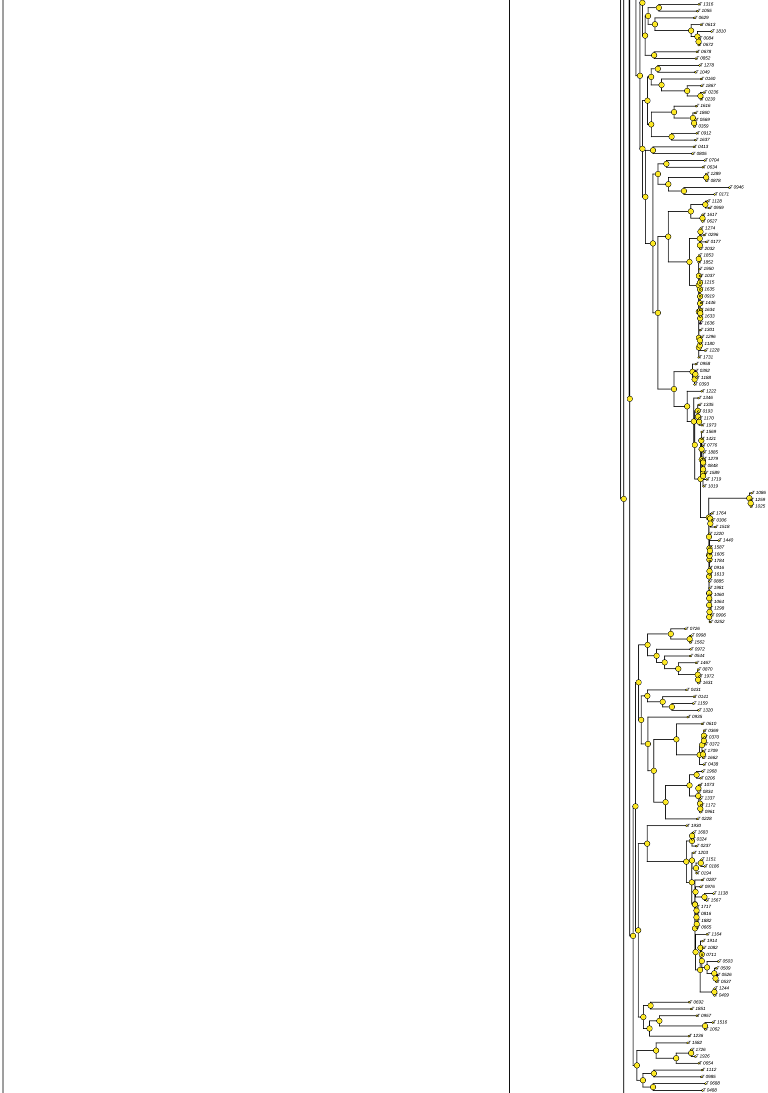
1316
1055
0629
0613
1810
0084
0672
0678
0852
1278
1049
0160
1867
0236
0230
1616
1860
0569
0359
0912
1637
0413
0805
0704
0634
1289
0878
0946
0171
1128
0959
1617
0627
1274
0296
0177
2032
1853
1852
1950
1037
1215
1635
0919
1446
1634
1633
1636
1301
1296
1180
1228
1731
0958
0392
1188
0393
1222
1346
1335
0193
1170
1973
1569
1421
0776
1885
1279
0848
1589
1719
1019
1086
1259
1025
1764
0306
1518
1220
1440
1587
1605
1784
0916
1613
0885
1981
1060
1064
1298
0906
0252
0726
0998
1562
0972
0544
1467
0870
1972
1631
0431
0141
1159
1320
0935
0610
0369
0370
0372
1709
1662
0438
1968
0206
1073
0834
1337
1172
0961
0228
1930
1683
0324
0237
1203
1151
0186
0194
0287
0976
1138
1567
1717
0816
1882
0665
1164
1914
1082
0711
0503
0509
0526
0537
1244
0409
0692
1851
0957
1516
1062
1236
1582
1726
1926
0654
1112
0985
0688
0488

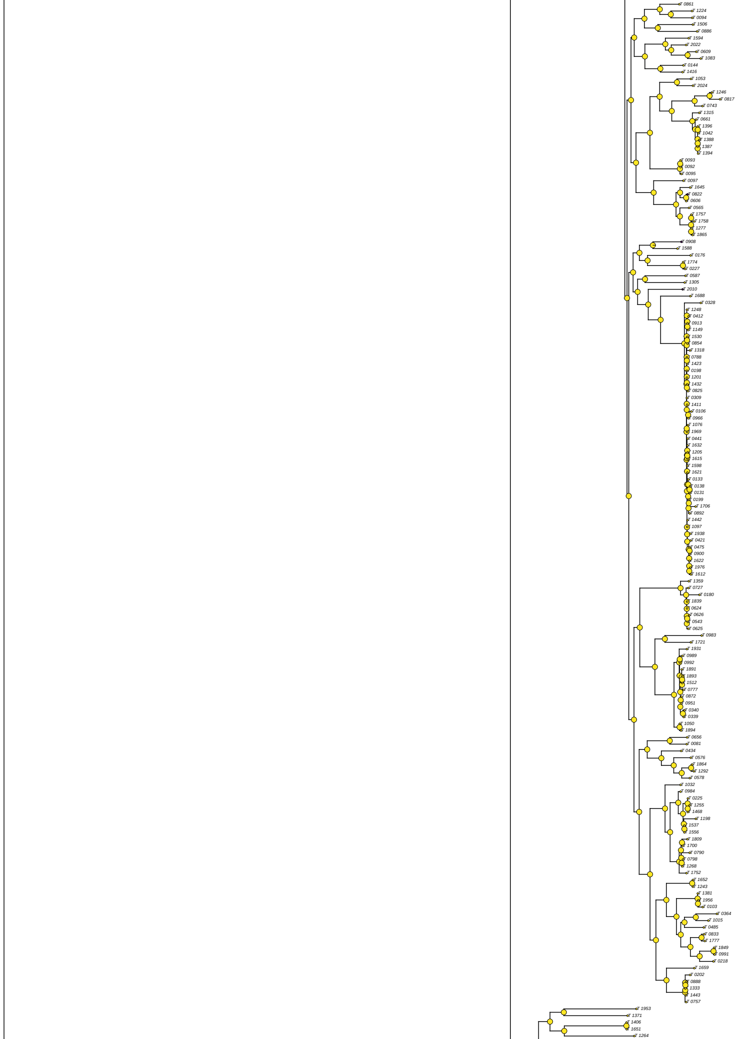
T 0861
T 1224
T 0094
T 1506
T 0886
T 1594
T 2022
T 0609
T 1083
T 0144
T 1416
T 1053
T 2024
T 1246
T 0817
T 0743
T 1315
T 0661
T 1396
T 1042
T 1388
T 1387
T 1394
T 0093
T 0092
T 0095
T 0097
T 1645
T 0822
T 0606
T 0565
T 1757
T 1758
T 1277
T 1865
T 0908
T 1588
T 0176
T 1774
T 0227
T 0587
T 1305
T 2010
T 1688
T 0328
T 1248
T 0412
T 0913
T 1149
T 1530
T 0854
T 1318
T 0788
T 1423
T 0198
T 1201
T 1432
T 0825
T 0309
T 1411
T 0106
T 0966
T 1076
T 1969
T 0441
T 1632
T 1205
T 1615
T 1598
T 1621
T 0133
T 0138
T 0131
T 0199
T 1706
T 0892
T 1442
T 1097
T 1938
T 0421
T 0475
T 0900
T 1622
T 1976
T 1612
T 1359
T 0727
T 0180
T 1839
T 0624
T 0626
T 0543
T 0625
T 0983
T 1721
T 1931
T 0989
T 0992
T 1891
T 1893
T 1512
T 0777
T 0872
T 0951
T 0340
T 0339
T 1050
T 1894
T 0656
T 0081
T 0434
T 0576
T 1864
T 1292
T 0578
T 1032
T 0984
T 0225
T 1255
T 1468
T 1198
T 1537
T 1556
T 1809
T 1700
T 0790
T 0798
T 1268
T 1752
T 1652
T 1243
T 1381
T 1956
T 0103
T 0364
T 1015
T 0485
T 0833
T 1777
T 1849
T 0991
T 0218
T 1659
T 0202
T 0888
T 1333
T 1443
T 0757
T 1953
T 1371
T 1406
T 1651
T 1264

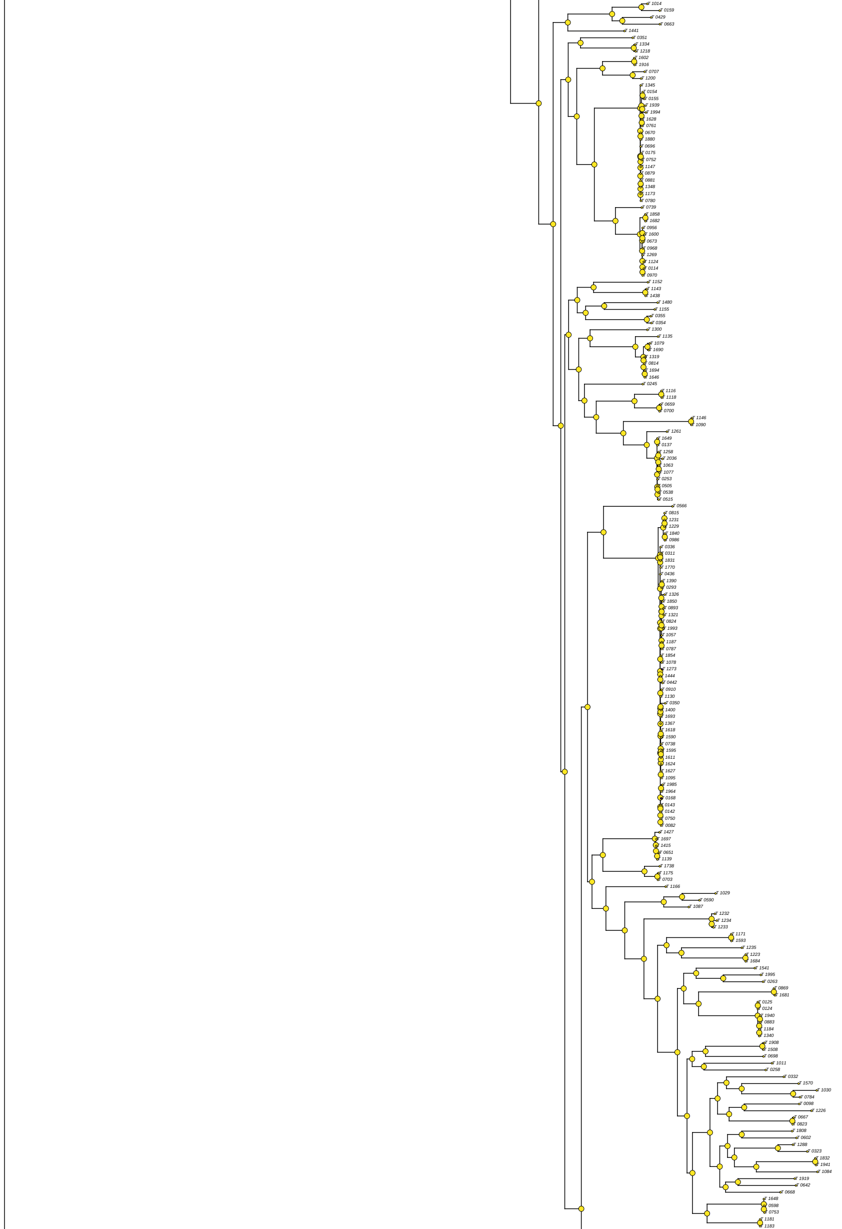

1014
0159
0429
0663
1441
0351
1334
1218
1602
1916
0707
1200
1345
0154
0155
1939
1994
1628
0761
0670
1880
0696
0175
0752
1147
0879
0881
1348
1173
0780
0739
1858
1682
0956
1600
0673
0968
1269
1124
0114
0970
1152
1143
1438
1480
1155
0355
0354
1300
1135
1079
1690
1319
0814
1694
1646
0245
1116
1118
0659
0700
1146
1090
1261
1649
0137
1258
2036
1063
1077
0253
0505
0538
0515
0566
0815
1231
1229
1840
0986
0336
0311
1831
1770
0436
1390
0293
1326
1850
0893
1321
0824
1993
1057
1187
0787
1854
1078
1273
1444
0442
0910
1130
0350
1400
1693
1367
1618
1590
0738
1595
1611
1624
1627
1095
1985
1964
0168
0143
0142
0750
0082
1427
1697
1415
0651
1139
1738
1175
0703
1166
1029
0590
1087
1232
1234
1233
1171
1593
1235
1223
1684
1541
1995
0263
0869
1681
0125
0124
1940
0883
1184
1340
1908
1508
0698
1011
0258
0332
1570
1030
0784
0098
1226
0667
0823
1808
0602
1288
0323
1832
1941
1084
1919
0642
0668
1648
0598
0753
1181
1183

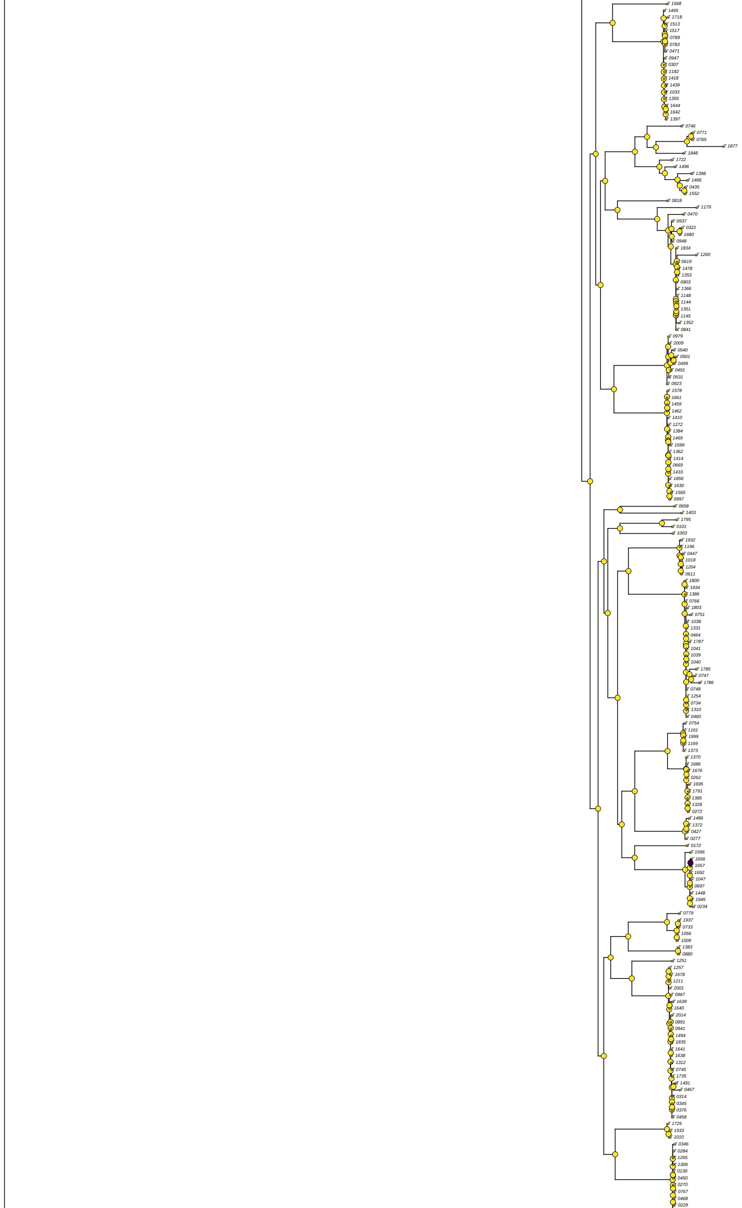
1568
1465
1718
1513
1517
0789
0783
0471
0947
0307
1182
1418
1439
1033
1355
1644
1642
1397
0746
0771
0765
1877
1848
1722
1496
1398
1466
0435
1552
0818
1179
0470
0937
0322
1680
0948
1834
1260
0619
1478
1353
0903
1366
1148
1144
1351
1145
1352
0841
0979
2009
0540
0501
0499
0491
0531
0923
1578
1661
1459
1462
1410
1272
1384
1469
1599
1362
1414
0669
1433
1856
1630
1565
0997
0658
1403
1795
0101
1003
1932
1196
0447
1018
1204
0611
1800
1934
1386
0766
1803
0751
1038
1331
0464
1787
1041
1039
1040
1785
0747
1786
0748
1254
0734
1310
0460
0754
1161
1999
1169
1373
1370
1686
1676
0262
1695
1791
1385
1328
0272
1486
1372
0427
0277
0172
1596
1656
1657
1692
1047
0697
1448
1945
0234
0779
1937
0733
1056
1009
1383
0880
1251
1257
1678
1211
2001
0967
1639
1640
2014
0891
0941
1494
1835
1641
1638
1312
0745
1735
1491
0467
0314
0345
0376
0458
1725
1933
1010
0346
0284
1265
1309
0130
0450
0270
0767
0468
0229

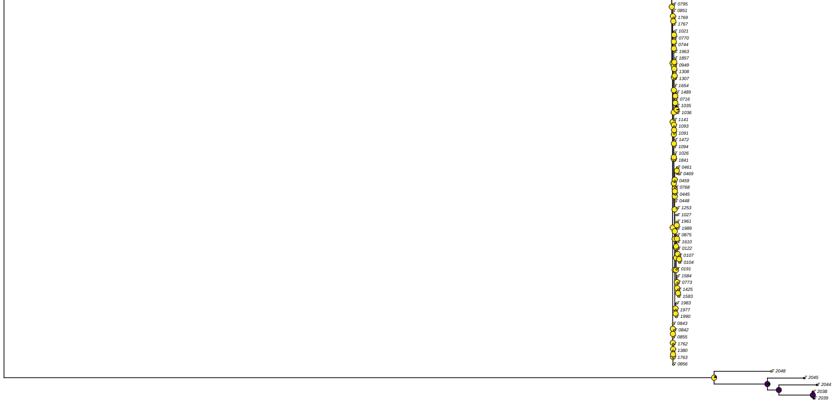
T 0795
T 0851
T 1769
T 1767
T 1021
T 0770
T 0744
T 1963
T 1857
T 0949
T 1308
T 1307
T 1654
T 1489
T 0716
T 1035
T 1036
T 1141
T 1093
T 1091
T 1472
T 1094
T 1026
T 1841
T 0461
T 0469
T 0459
T 0768
T 0445
T 0448
T 1253
T 1027
T 1961
T 1989
T 0875
T 1610
T 0122
T 0107
T 0104
T 0191
T 1584
T 0773
T 1425
T 1583
T 1983
T 1977
T 1990
T 0843
T 0842
T 0855
T 1762
T 1380
T 1763
T 0856
T 2048
T 2045
T 2044
T 2038
T 2039

H4−T6SS

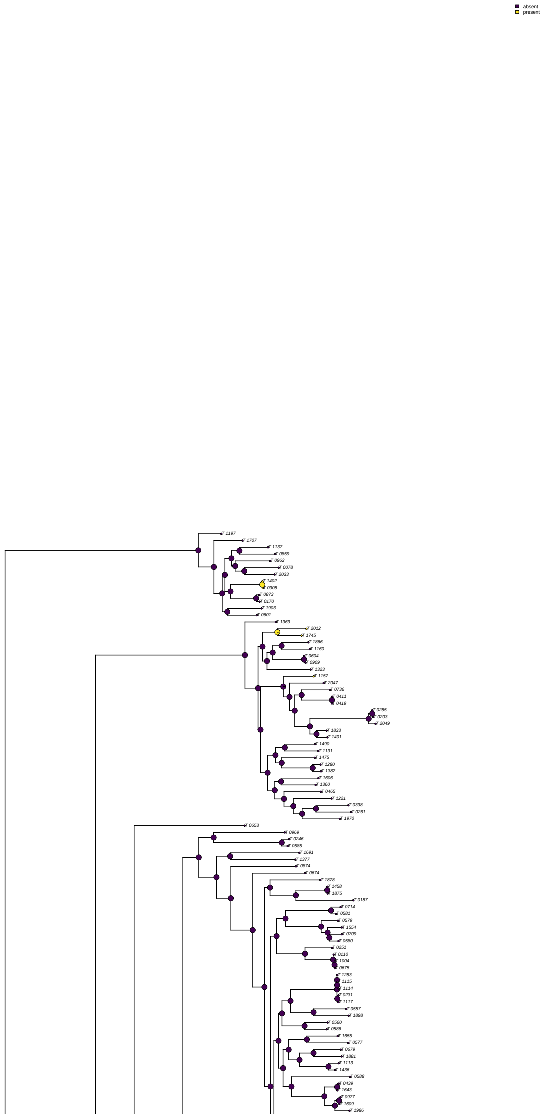
absent
present
T 1197
T 1707
T 1137
T 0859
T 0962
T 0078
T 2033
T 1402
T 0308
T 0873
T 0170
T 1903
T 0601
T 1369
T 2012
T 1745
T 1866
T 1160
T 0604
T 0909
T 1323
T 1157
T 2047
T 0736
T 0411
T 0419
T 0285
T 0203
T 2049
T 1833
T 1401
T 1490
T 1131
T 1475
T 1280
T 1382
T 1606
T 1360
T 0465
T 1221
T 0338
T 0261
T 1970
T 0653
T 0969
T 0246
T 0585
T 1691
T 1377
T 0874
T 0674
T 1878
T 1458
T 1875
T 0187
T 0714
T 0581
T 0579
T 1554
T 0709
T 0580
T 0251
T 0110
T 1004
T 0675
T 1283
T 1115
T 1114
T 0231
T 1117
T 0557
T 1898
T 0560
T 0586
T 1655
T 0577
T 0679
T 1881
T 1113
T 1436
T 0588
T 0439
T 1643
T 0977
T 1609
T 1986

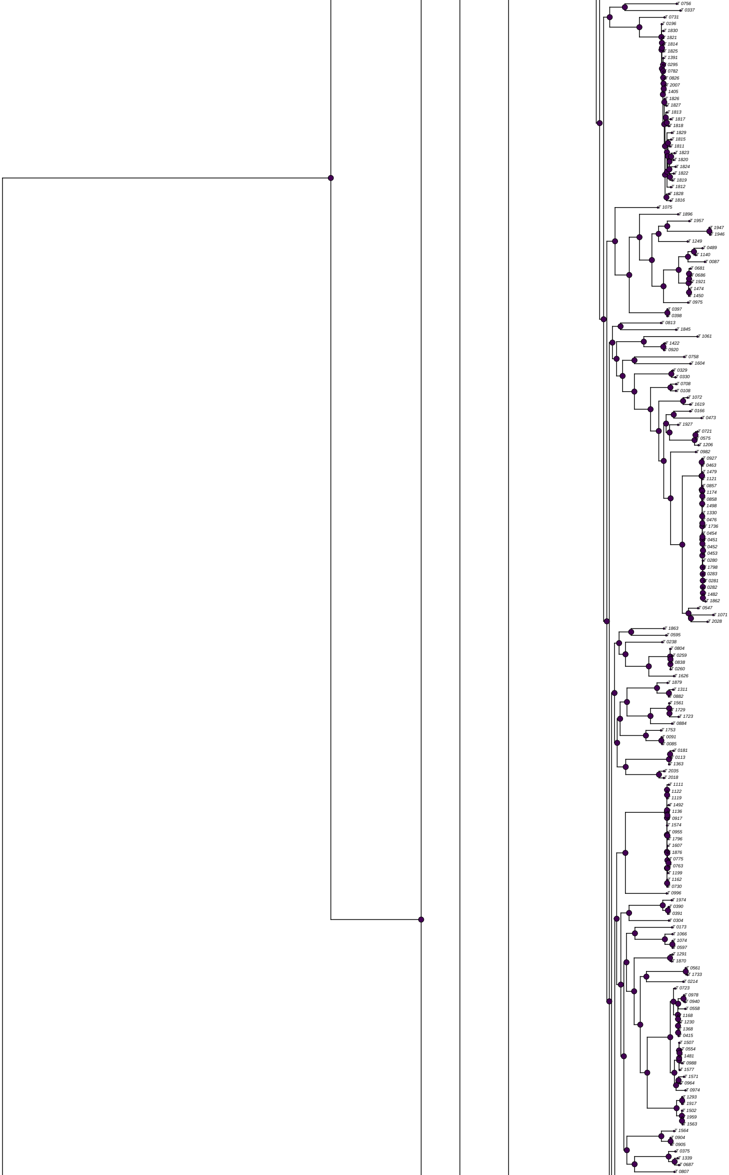
T 0756
T 0337
T 0731
T 0196
T 1830
T 1821
T 1814
T 1825
T 1391
T 0295
T 0782
T 0826
T 2007
T 1405
T 1826
T 1827
T 1813
T 1817
T 1818
T 1829
T 1815
T 1811
T 1823
T 1820
T 1824
T 1822
T 1819
T 1812
T 1828
T 1816
T 1075
T 1896
T 1957
T 1947
T 1946
T 1249
T 0489
T 1140
T 0087
T 0681
T 0686
T 1921
T 1474
T 1450
T 0975
T 0397
T 0398
T 0813
T 1845
T 1061
T 1422
T 0920
T 0758
T 1604
T 0329
T 0330
T 0708
T 0108
T 1072
T 1619
T 0166
T 0473
T 1927
T 0721
T 0575
T 1206
T 0982
T 0927
T 0463
T 1479
T 1121
T 0857
T 1174
T 0858
T 1498
T 1330
T 0476
T 1736
T 0454
T 0451
T 0452
T 0453
T 0280
T 1798
T 0283
T 0281
T 0282
T 1482
T 1862
T 0547
T 1071
T 2028
T 1863
T 0595
T 0238
T 0804
T 0259
T 0838
T 0260
T 1626
T 1879
T 1311
T 0882
T 1561
T 1729
T 1723
T 0884
T 1753
T 0091
T 0085
T 0181
T 0113
T 1363
T 2035
T 2018
T 1111
T 1122
T 1119
T 1492
T 1136
T 0917
T 1574
T 0955
T 1796
T 1607
T 1876
T 0775
T 0763
T 1199
T 1162
T 0730
T 0996
T 1974
T 0390
T 0391
T 0304
T 0173
T 1066
T 1074
T 0597
T 1291
T 1870
T 0561
T 1733
T 0214
T 0723
T 0978
T 0940
T 0558
T 1168
T 1230
T 1368
T 0415
T 1507
T 0554
T 1481
T 0988
T 1577
T 1571
T 0964
T 0974
T 1293
T 1917
T 1502
T 1959
T 1563
T 1564
T 0904
T 0905
T 0375
T 1339
T 0687
T 0807

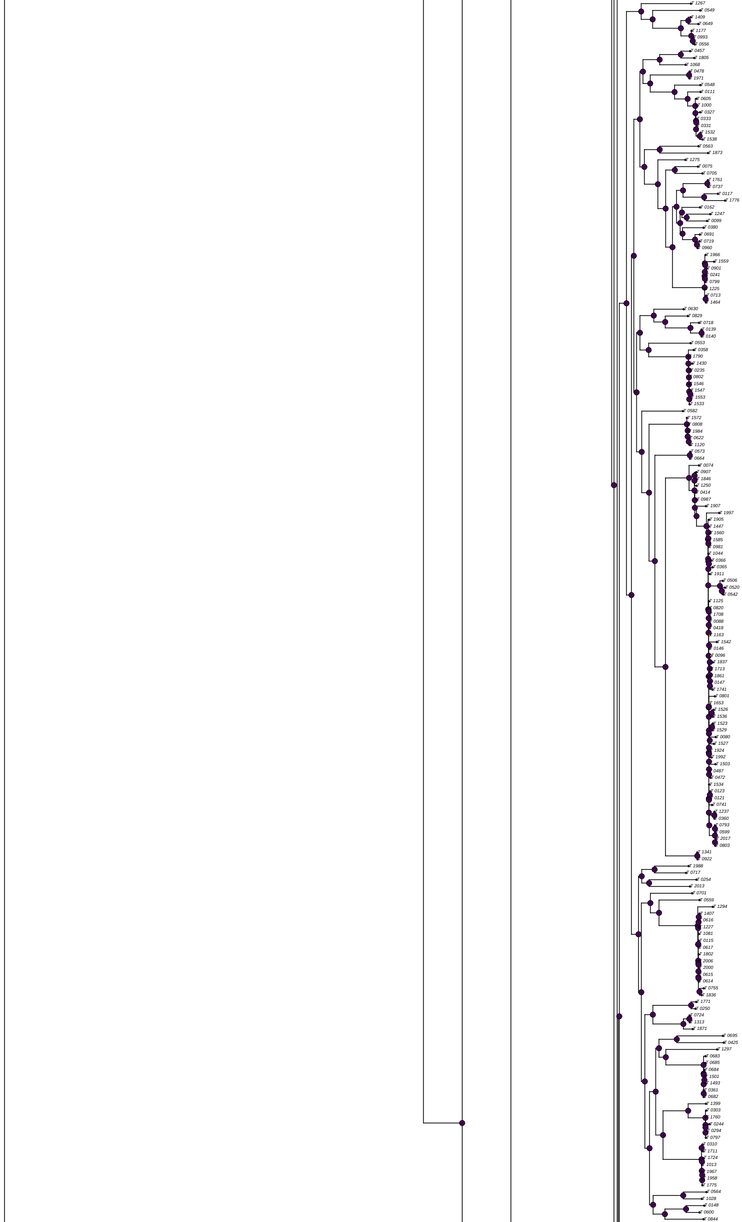

T 1267
T 0549
T 1409
T 0649
T 1177
T 0993
T 0556
T 0457
T 1805
T 1068
T 0478
T 1971
T 0548
T 0111
T 0605
T 1000
T 0327
T 0333
T 0331
T 1532
T 1538
T 0563
T 1873
T 1275
T 0075
T 0705
T 1761
T 0737
T 0117
T 1776
T 0162
T 1247
T 0099
T 0380
T 0691
T 0719
T 0960
T 1966
T 1559
T 0901
T 0241
T 0799
T 1225
T 0713
T 1464
T 0630
T 0829
T 0718
T 0139
T 0140
T 0553
T 0358
T 1790
T 1430
T 0235
T 0802
T 1546
T 1547
T 1553
T 1533
T 0582
T 1572
T 0808
T 1984
T 0622
T 1120
T 0573
T 0664
T 0074
T 0907
T 1846
T 1250
T 0414
T 0987
T 1907
T 1997
T 1905
T 1447
T 1560
T 1585
T 0981
T 1044
T 0366
T 0365
T 1911
T 0506
T 0520
T 0542
T 1125
T 0820
T 1708
T 0088
T 0418
T 1163
T 1542
T 0146
T 0096
T 1837
T 1713
T 1861
T 0147
T 1741
T 0801
T 1653
T 1526
T 1536
T 1523
T 1529
T 0080
T 1527
T 1924
T 1992
T 1503
T 0487
T 0472
T 1534
T 0123
T 0121
T 0741
T 1237
T 0360
T 0793
T 0599
T 2017
T 0803
T 1341
T 0922
T 1988
T 0717
T 0254
T 2013
T 0701
T 0559
T 1294
T 1407
T 0616
T 1227
T 1081
T 0115
T 0617
T 1802
T 2006
T 2000
T 0615
T 0614
T 0755
T 1836
T 1771
T 0250
T 0724
T 1313
T 1871
T 0695
T 0425
T 1297
T 0683
T 0685
T 0684
T 1501
T 1493
T 0361
T 0682
T 1399
T 0303
T 1760
T 0244
T 0294
T 0797
T 0310
T 1711
T 1724
T 1013
T 1967
T 1958
T 1775
T 0564
T 1028
T 0148
T 0600
T 0844

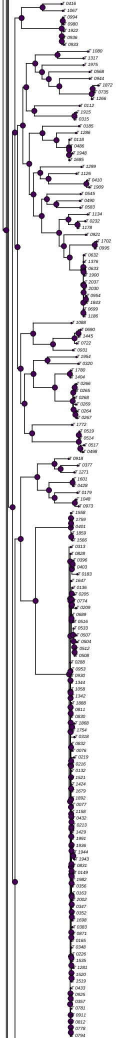

T 0416
T 1067
T 0994
T 0980
T 1922
T 0936
T 0933
T 1080
T 1317
T 1975
T 0568
T 0944
T 1872
T 0735
T 1266
T 0112
T 1915
T 0315
T 0185
T 1286
T 0118
T 0486
T 1948
T 1685
T 1299
T 1126
T 0410
T 1909
T 0545
T 0490
T 0583
T 1134
T 0232
T 1178
T 0921
T 1702
T 0995
T 0632
T 1376
T 0633
T 1900
T 2037
T 2030
T 0954
T 1843
T 0699
T 1186
T 1088
T 0690
T 1445
T 0722
T 0931
T 1954
T 0320
T 1780
T 1404
T 0266
T 0265
T 0268
T 0269
T 0264
T 0267
T 1772
T 0519
T 0514
T 0517
T 0498
T 0918
T 0377
T 1271
T 1601
T 0428
T 0179
T 1048
T 0973
T 1558
T 1759
T 0401
T 1859
T 1566
T 0313
T 0828
T 0396
T 0403
T 0183
T 1647
T 0136
T 0205
T 0774
T 0209
T 0689
T 0516
T 0533
T 0507
T 0504
T 0512
T 0508
T 0288
T 0953
T 0930
T 1344
T 1058
T 1342
T 1888
T 0811
T 0830
T 1868
T 1754
T 0318
T 0832
T 0076
T 0219
T 0216
T 0132
T 1521
T 1424
T 1679
T 1892
T 0077
T 1158
T 0432
T 0213
T 1429
T 1991
T 1936
T 1944
T 1943
T 0831
T 0149
T 1982
T 0356
T 0163
T 2002
T 0347
T 0352
T 1698
T 0383
T 0871
T 0165
T 0348
T 0226
T 1535
T 1281
T 1520
T 1519
T 0433
T 0925
T 0357
T 0781
T 0911
T 0812
T 0778
T 0794

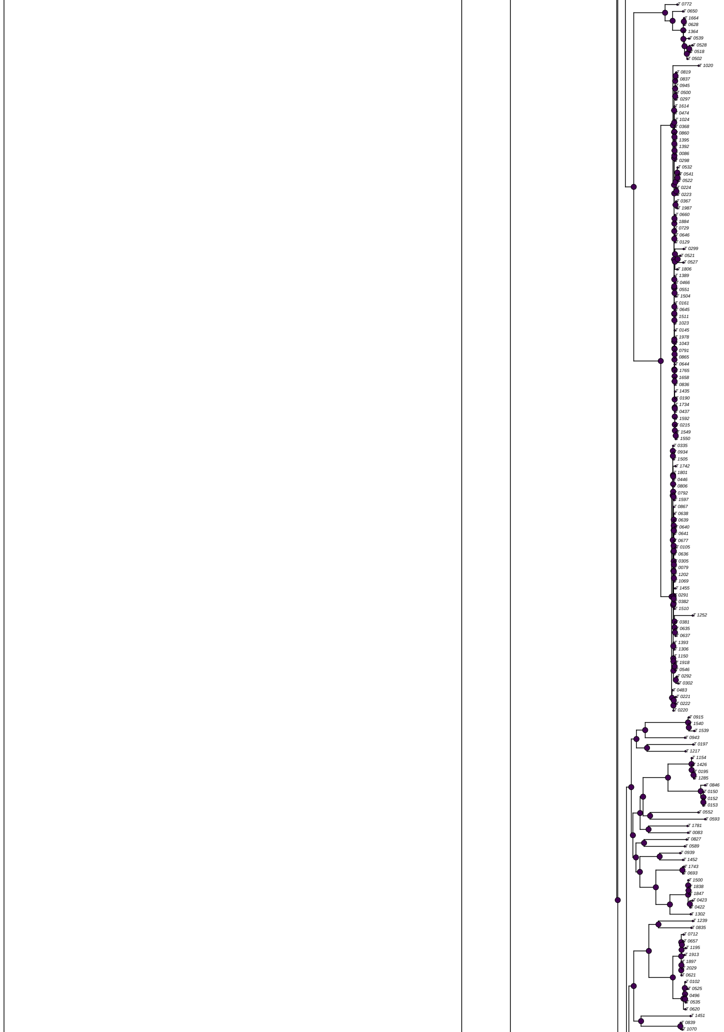
T 0772
T 0650
T 1664
T 0628
T 1364
T 0539
T 0528
T 0518
T 0502
T 1020
T 0819
T 0837
T 0945
T 0500
T 0297
T 1614
T 0474
T 1024
T 0368
T 0860
T 1395
T 1392
T 0086
T 0298
T 0532
T 0541
T 0522
T 0224
T 0223
T 0367
T 1987
T 0660
T 1884
T 0729
T 0646
T 0129
T 0299
T 0521
T 0527
T 1806
T 1389
T 0466
T 0551
T 1504
T 0161
T 0645
T 1511
T 1023
T 0145
T 1978
T 1043
T 0791
T 0865
T 0644
T 1765
T 1658
T 0836
T 1435
T 0190
T 1734
T 0437
T 1592
T 0215
T 1549
T 1550
T 0335
T 0934
T 1505
T 1742
T 1801
T 0446
T 0806
T 0792
T 1597
T 0867
T 0638
T 0639
T 0640
T 0641
T 0677
T 0105
T 0636
T 0305
T 0079
T 1202
T 1069
T 1455
T 0291
T 0382
T 1510
T 1252
T 0381
T 0635
T 0637
T 1393
T 1306
T 1150
T 1918
T 0546
T 0292
T 0302
T 0483
T 0221
T 0222
T 0220
T 0915
T 1540
T 1539
T 0943
T 0197
T 1217
T 1154
T 1426
T 0195
T 1285
T 0846
T 0150
T 0152
T 0153
T 0552
T 0593
T 1781
T 0083
T 0827
T 0589
T 0939
T 1452
T 1743
T 0693
T 1500
T 1838
T 1847
T 0423
T 0422
T 1302
T 1239
T 0835
T 0712
T 0657
T 1195
T 1913
T 1897
T 2029
T 0621
T 0102
T 0525
T 0496
T 0535
T 0620
T 1451
T 0839
T 1070

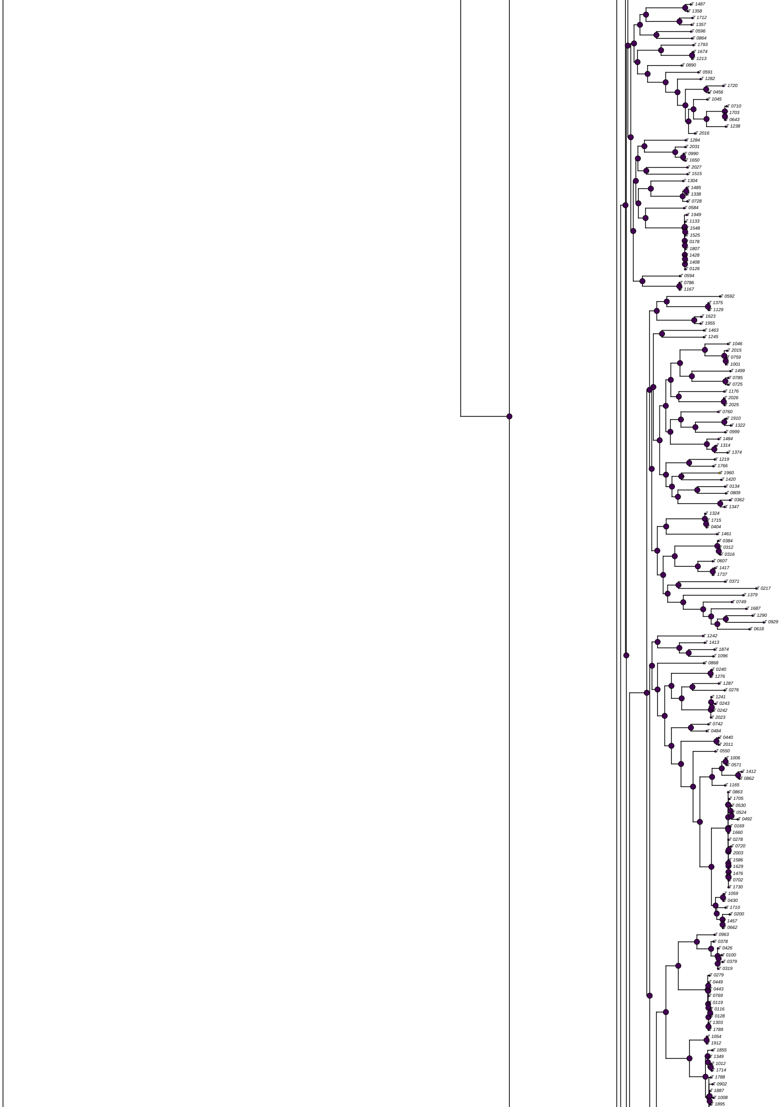
T 1487
T 1358
T 1712
T 1357
T 0596
T 0864
T 1793
T 1674
T 1213
T 0890
T 0591
T 1282
T 1720
T 0456
T 1045
T 0710
1703
T 0643
T 1238
T 2016
T 1284
T 2031
T 0990
T 1650
T 2027
T 1515
T 1304
T 1485
T 1338
T 0728
T 0584
T 1949
T 1133
1548
T 1525
T 0178
T 1807
1428
1408
T 0126
T 0594
T 0786
T 1167
T 0592
T 1375
T 1129
T 1623
T 1955
T 1463
T 1245
T 1046
T 2015
T 0759
T 1001
T 1499
T 0785
T 0725
T 1176
T 2026
T 2025
T 0760
T 1910
T 1322
T 0999
T 1484
T 1314
T 1374
T 1219
T 1766
T 1960
T 1420
T 0134
T 0809
T 0362
T 1347
T 1324
T 1715
T 0404
T 1461
T 0384
T 0312
T 0316
T 0607
T 1417
T 1737
T 0371
T 0217
T 1379
T 0749
T 1687
T 1290
T 0929
T 0618
T 1242
T 1413
T 1874
T 1096
T 0868
T 0240
T 1276
T 1287
T 0276
T 1241
T 0243
T 0242
T 2023
T 0742
T 0484
T 0440
T 2011
T 0550
T 1006
T 0571
T 1412
T 0862
T 1165
T 0863
T 1705
T 0530
T 0524
T 0492
T 0169
1660
T 0278
T 0720
2003
T 1586
1629
1476
T 0702
T 1730
T 1059
0430
T 1710
T 0200
1457
0662
T 0963
T 0378
T 0426
T 0100
T 0379
T 0319
T 0279
T 0449
T 0443
T 0769
T 0119
T 0116
T 0128
T 1303
T 1789
T 1054
T 1912
T 1855
T 1349
T 1012
T 1714
T 1788
T 0902
T 1887
T 1008
T 1895

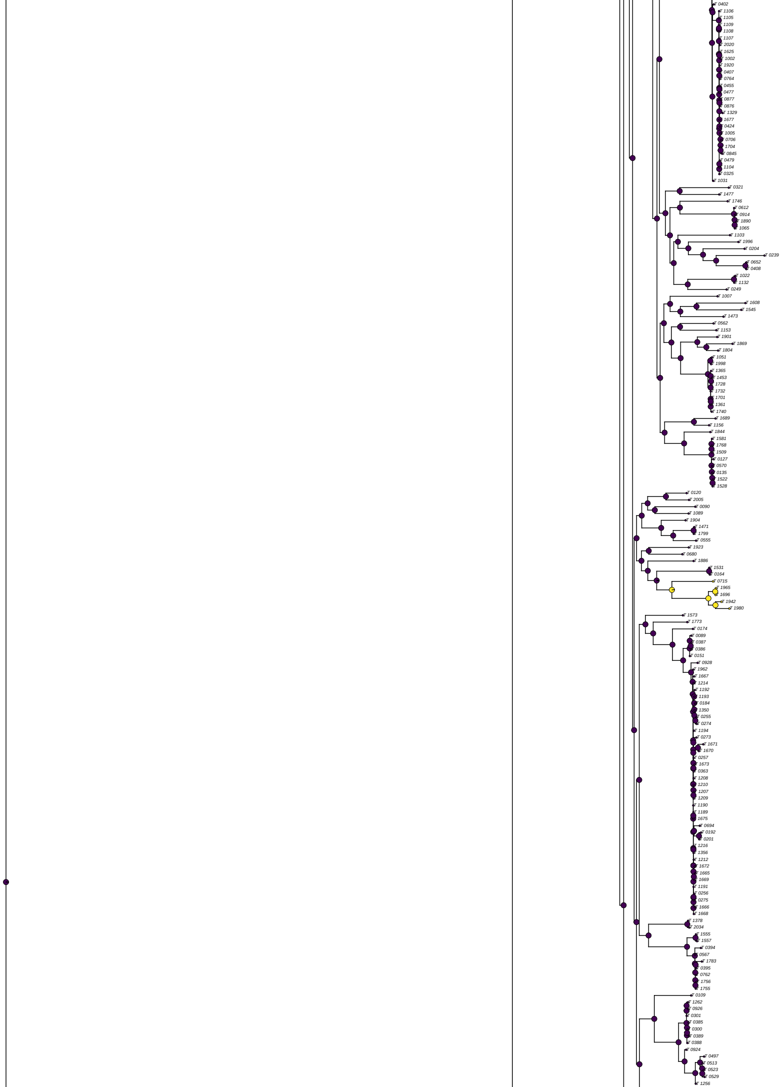
T 0402
T 1106
T 1105
T 1109
T 1108
T 1107
T 2020
T 1625
T 1002
T 1920
T 0407
T 0764
T 0455
T 0477
T 0877
T 0876
T 1329
1677
T 0424
T 1005
T 0706
1704
T 0845
T 0479
1104
T 0325
T 1031
T 0321
T 1477
T 1746
T 0612
T 0914
T 1890
T 1065
T 1103
T 1996
T 0204
T 0239
T 0652
T 0408
T 1022
T 1132
T 0249
T 1007
T 1608
T 1545
T 1473
T 0562
T 1153
T 1901
T 1869
T 1804
T 1051
T 1998
T 1365
T 1453
T 1728
T 1732
T 1701
1361
T 1740
T 1689
T 1156
T 1844
T 1581
1768
1509
T 0127
0570
0135
1522
T 1528
T 0120
T 2005
T 0090
T 1089
T 1904
T 1471
T 1799
T 0555
T 1923
T 0680
T 1886
T 1531
T 0164
T 0715
T 1965
T 1696
T 1942
T 1980
T 1573
T 1773
T 0174
T 0089
T 0387
T 0386
T 0151
T 0928
T 1962
T 1667
1214
T 1192
1193
0184
1350
T 0255
T 0274
T 1194
T 0273
T 1671
T 1670
T 0257
T 1673
0363
T 1208
1210
T 1207
T 1209
T 1190
T 1189
1675
T 0694
T 0192
T 0201
T 1216
T 1356
T 1212
T 1672
T 1665
1669
T 1191
T 0256
0275
T 1666
T 1668
T 1378
T 2034
T 1555
T 1557
T 0394
0567
T 1783
0395
0762
T 1756
T 1755
T 0109
T 1262
T 0926
T 0301
T 0385
0300
T 0389
T 0388
T 0924
T 0497
T 0513
T 0523
T 0529
T 1256

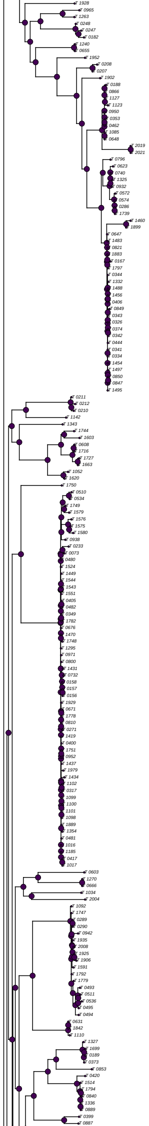
T 1928
T 0965
T 1263
T 0248
T 0247
T 0182
T 1240
T 0655
T 1952
T 0208
T 0207
T 1902
T 0188
0866
1127
T 1123
0950
0353
0462
T 1085
T 0648
T 2019
T 2021
T 0796
T 0623
0740
T 1325
T 0932
T 0572
0574
0286
T 1739
T 1460
T 1899
T 0647
T 1483
0821
1883
T 0167
T 1797
T 0344
T 1332
1488
1456
0406
T 0849
0343
0326
0374
T 0342
T 0444
T 0341
T 0334
1454
T 1497
0850
0847
T 1495
T 0211
T 0212
T 0210
T 1142
T 1343
T 1744
T 1603
T 0608
T 1716
T 1727
T 1663
T 1052
T 1620
T 1750
T 0510
T 0534
T 1749
T 1579
T 1576
T 1575
T 1580
T 0938
T 0233
T 0073
0480
T 1524
T 1449
T 1544
1543
T 1551
T 0405
0482
0349
1782
T 0676
1470
T 1748
T 1295
T 0971
T 0800
T 1431
T 0732
0158
0157
T 0156
T 1929
T 0671
T 1778
0810
T 0271
1419
T 0400
1751
0952
T 1437
T 1979
T 1434
1102
0317
1099
1100
1101
1098
T 1889
T 1354
T 0481
1016
1185
T 0417
T 1017
T 0603
T 1270
T 0666
T 1034
T 2004
T 1092
T 1747
T 0289
T 0290
T 0942
T 1935
2008
T 1925
T 1906
T 1591
T 1792
T 1779
T 0493
T 0511
T 0536
T 0495
T 0494
T 0631
1842
T 1110
T 1327
T 1699
T 0189
T 0373
T 0853
T 0420
T 1514
T 1794
T 0840
T 1336
T 0889
T 0399
T 0887

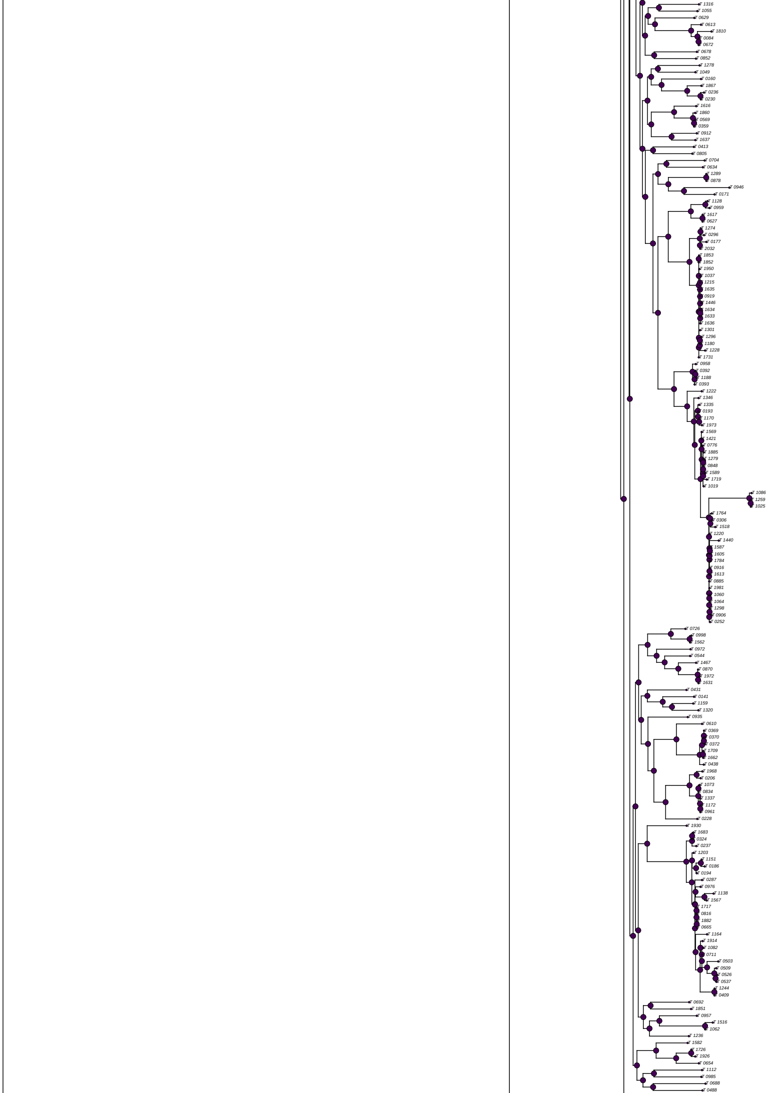
T 1316
T 1055
T 0629
T 0613
T 1810
T 0084
T 0672
T 0678
T 0852
T 1278
T 1049
T 0160
T 1867
T 0236
T 0230
T 1616
T 1860
T 0569
T 0359
T 0912
T 1637
T 0413
T 0805
T 0704
T 0634
T 1289
T 0878
T 0946
T 0171
T 1128
T 0959
T 1617
T 0627
T 1274
T 0296
T 0177
T 2032
T 1853
T 1852
T 1950
T 1037
T 1215
T 1635
T 0919
T 1446
T 1634
T 1633
T 1636
T 1301
T 1296
T 1180
T 1228
T 1731
T 0958
T 0392
T 1188
T 0393
T 1222
T 1346
T 1335
T 0193
T 1170
T 1973
T 1569
T 1421
T 0776
T 1885
T 1279
T 0848
T 1589
T 1719
T 1019
T 1086
T 1259
T 1025
T 1764
T 0306
T 1518
T 1220
T 1440
T 1587
T 1605
T 1784
T 0916
T 1613
T 0885
T 1981
T 1060
T 1064
T 1298
T 0906
T 0252
T 0726
T 0998
T 1562
T 0972
T 0544
T 1467
T 0870
T 1972
T 1631
T 0431
T 0141
T 1159
T 1320
T 0935
T 0610
T 0369
T 0370
T 0372
T 1709
T 1662
T 0438
T 1968
T 0206
T 1073
T 0834
T 1337
T 1172
T 0961
T 0228
T 1930
T 1683
T 0324
T 0237
T 1203
T 1151
T 0186
T 0194
T 0287
T 0976
T 1138
T 1567
T 1717
T 0816
T 1882
T 0665
T 1164
T 1914
T 1082
T 0711
T 0503
T 0509
T 0526
T 0537
T 1244
T 0409
T 0692
T 1851
T 0957
T 1516
T 1062
T 1236
T 1582
T 1726
T 1926
T 0654
T 1112
T 0985
T 0688
T 0488

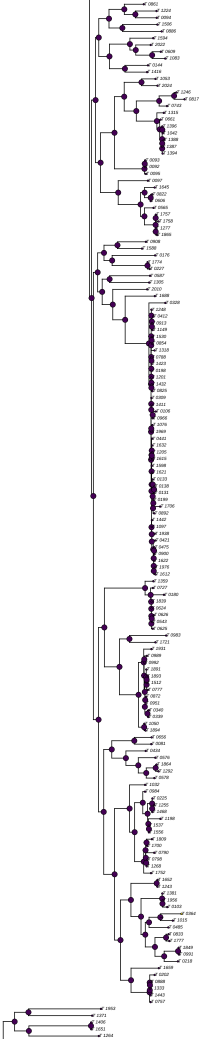
T 0861
T 1224
T 0094
T 1506
T 0886
T 1594
T 2022
T 0609
T 1083
T 0144
T 1416
T 1053
T 2024
T 1246
T 0817
T 0743
T 1315
T 0661
T 1396
T 1042
T 1388
1387
T 1394
T 0093
0092
T 0095
T 0097
T 1645
T 0822
0606
T 0565
T 1757
T 1758
1277
T 1865
T 0908
T 1588
T 0176
T 1774
T 0227
T 0587
T 1305
T 2010
T 1688
T 0328
T 1248
T 0412
0913
T 1149
T 1530
0854
T 1318
0788
T 1423
0198
1201
1432
T 0825
T 0309
1411
T 0106
T 0966
T 1076
1969
T 0441
T 1632
1205
1615
T 1598
1621
T 0133
T 0138
T 0131
T 0199
T 1706
T 0892
T 1442
T 1097
T 1938
T 0421
T 0475
T 0900
1622
T 1976
T 1612
T 1359
T 0727
T 0180
1839
0624
T 0626
T 0543
T 0625
T 0983
T 1721
T 1931
T 0989
0992
T 1891
1893
1512
T 0777
T 0872
0951
T 0340
T 0339
T 1050
T 1894
T 0656
T 0081
T 0434
T 0576
T 1864
T 1292
T 0578
T 1032
T 0984
T 0225
T 1255
T 1468
T 1198
1537
T 1556
T 1809
T 1700
T 0790
T 0798
T 1268
T 1752
T 1652
T 1243
T 1381
1956
T 0103
T 0364
T 1015
T 0485
T 0833
T 1777
T 1849
T 0991
T 0218
T 1659
T 0202
0888
1333
T 1443
T 0757
T 1953
T 1371
T 1406
T 1651
T 1264

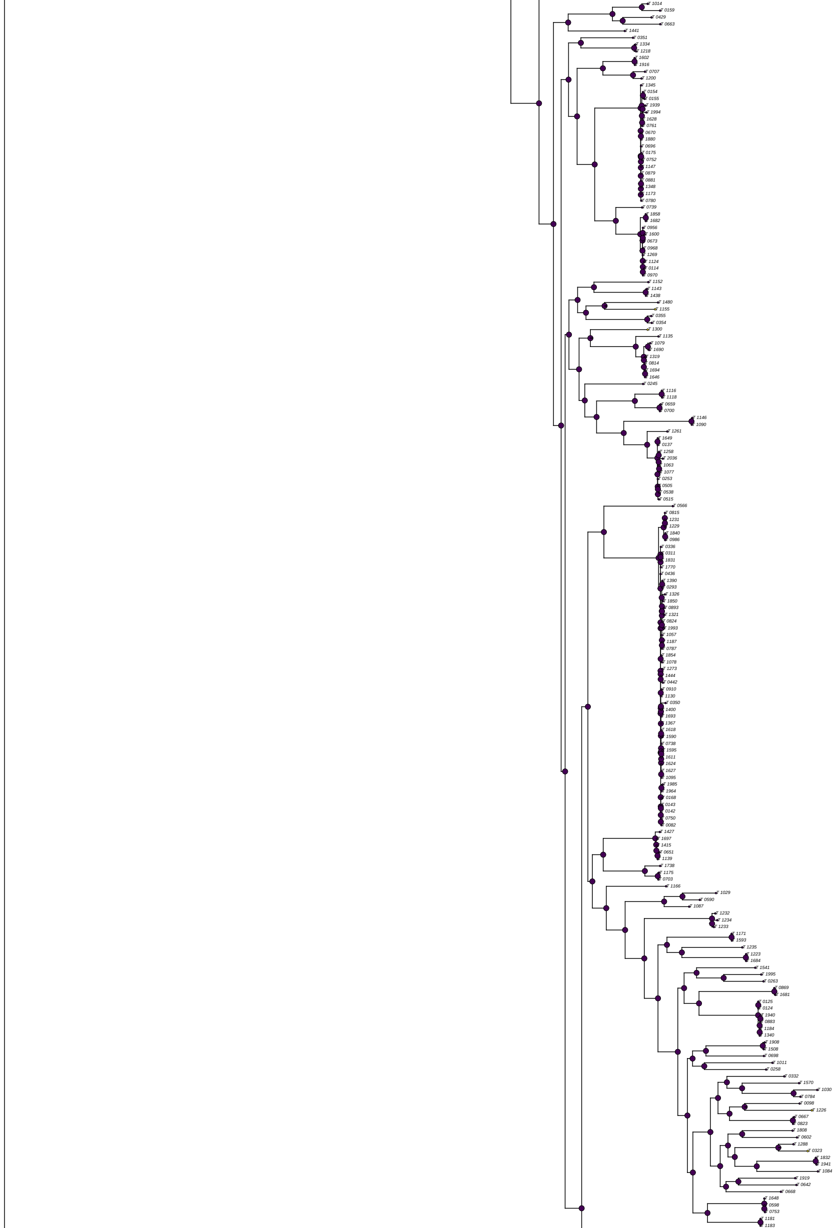

T 1014
T 0159
T 0429
T 0663
T 1441
T 0351
T 1334
T 1218
T 1602
T 1916
T 0707
T 1200
T 1345
T 0154
T 0155
T 1939
T 1994
1628
0761
0670
1880
T 0696
T 0175
T 0752
1147
0879
0881
1348
1173
T 0780
T 0739
T 1858
1682
T 0956
T 1600
0673
T 0968
T 1269
T 1124
T 0114
T 0970
T 1152
T 1143
T 1438
T 1480
T 1155
T 0355
T 0354
T 1300
T 1135
T 1079
T 1690
T 1319
T 0814
T 1694
T 1646
T 0245
T 1116
T 1118
T 0659
T 0700
T 1146
T 1090
T 1261
T 1649
T 0137
T 1258
T 2036
1063
T 1077
T 0253
0505
T 0538
T 0515
T 0566
T 0815
1231
1229
T 1840
T 0986
T 0336
T 0311
1831
T 1770
T 0436
T 1390
0293
T 1326
T 1850
T 0893
T 1321
T 0824
T 1993
T 1057
1187
T 0787
T 1854
T 1078
T 1273
1444
T 0442
T 0910
T 1130
T 0350
1400
1693
1367
T 1618
1590
T 0738
1595
1611
1624
T 1627
T 1095
T 1985
T 1964
T 0168
T 0143
T 0142
T 0750
T 0082
T 1427
T 1697
1415
T 0651
T 1139
T 1738
T 1175
T 0703
T 1166
T 1029
T 0590
T 1087
T 1232
T 1234
T 1233
T 1171
1593
T 1235
T 1223
1684
T 1541
T 1995
T 0263
T 0869
T 1681
T 0125
T 0124
T 1940
T 0883
T 1184
T 1340
T 1908
T 1508
T 0698
T 1011
T 0258
T 0332
T 1570
T 1030
T 0784
T 0098
T 1226
T 0667
T 0823
T 1808
T 0602
T 1288
T 0323
T 1832
T 1941
T 1084
T 1919
T 0642
T 0668
T 1648
T 0598
T 0753
T 1181
T 1183

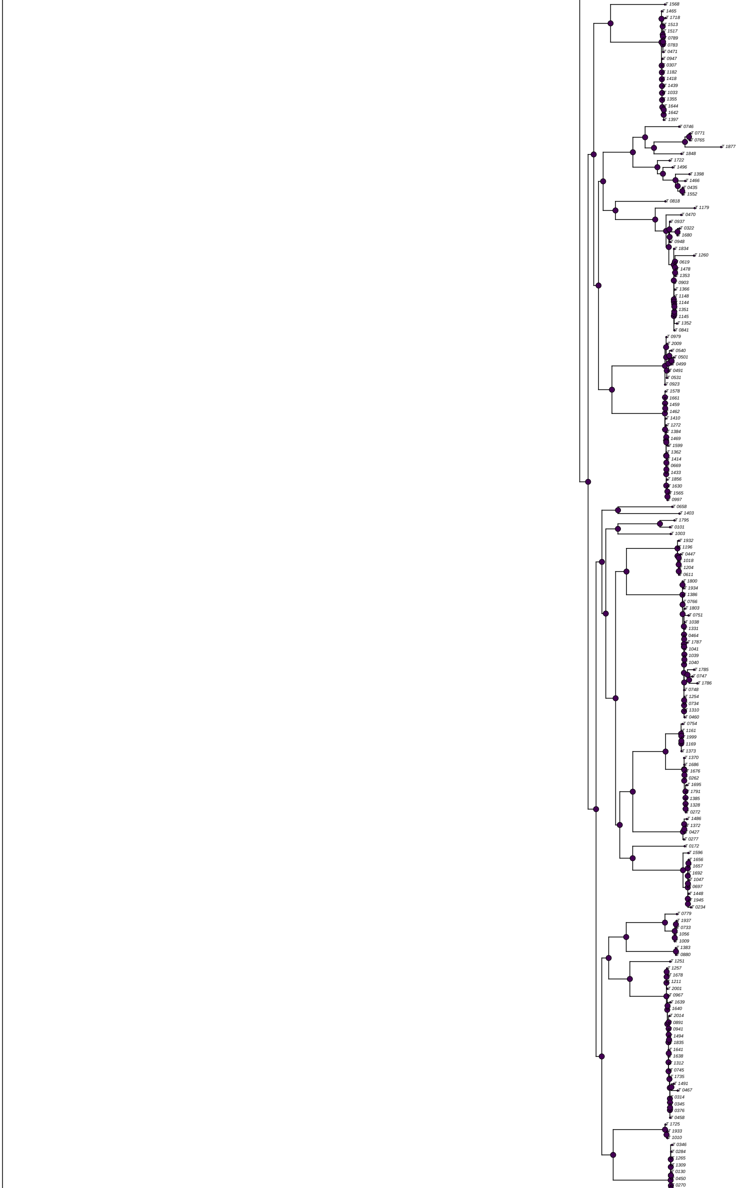
T 1568
T 1465
T 1718
1513
1517
0789
0783
T 0471
T 0947
0307
1182
1418
T 1439
1033
1355
1644
1642
T 1397
T 0746
T 0771
T 0765
T 1877
T 1848
T 1722
T 1496
T 1398
T 1466
T 0435
1552
T 0818
T 1179
T 0470
T 0937
T 0322
T 1680
T 0948
T 1834
T 1260
0619
1478
1353
0903
T 1366
T 1148
1144
1351
1145
T 1352
T 0841
T 0979
T 2009
T 0540
T 0501
T 0499
T 0491
T 0531
T 0923
T 1578
1661
1459
1462
T 1410
T 1272
T 1384
1469
T 1599
T 1362
T 1414
0669
1433
T 1856
T 1630
T 1565
0997
T 0658
T 1403
T 1795
T 0101
T 1003
T 1932
1196
T 0447
1018
1204
T 0611
T 1800
T 1934
1386
T 0766
T 1803
T 0751
T 1038
1331
0464
T 1787
1041
1039
1040
T 1785
T 0747
T 1786
T 0748
T 1254
0734
T 1310
T 0460
T 0754
T 1161
T 1999
1169
T 1373
T 1370
T 1686
T 1676
0262
T 1695
1791
1385
1328
T 0272
T 1486
T 1372
T 0427
T 0277
T 0172
T 1596
T 1656
T 1657
1692
T 1047
T 0697
T 1448
T 1945
T 0234
T 0779
T 1937
T 0733
1056
1009
T 1383
T 0880
T 1251
T 1257
T 1678
1211
T 2001
T 0967
T 1639
1640
T 2014
0891
0941
1494
1835
T 1641
T 1638
1312
T 0745
T 1735
T 1491
T 0467
T 0314
0345
0376
T 0458
T 1725
T 1933
T 1010
T 0346
T 0284
1265
T 1309
T 0130
0450
0270

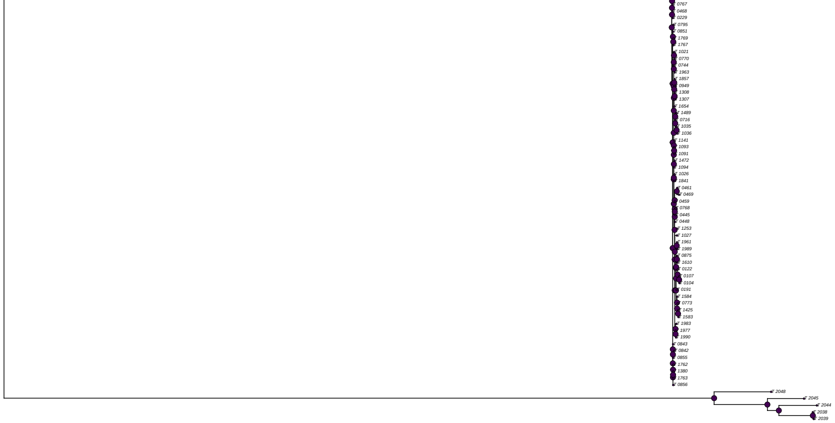
T 0767
T 0468
T 0229
T 0795
T 0851
T 1769
T 1767
T 1021
T 0770
T 0744
T 1963
T 1857
T 0949
T 1308
T 1307
T 1654
T 1489
T 0716
T 1035
T 1036
T 1141
T 1093
T 1091
T 1472
T 1094
T 1026
T 1841
T 0461
T 0469
T 0459
T 0768
T 0445
T 0448
T 1253
T 1027
T 1961
T 1989
T 0875
T 1610
T 0122
T 0107
T 0104
T 0191
T 1584
T 0773
T 1425
T 1583
T 1983
T 1977
T 1990
T 0843
T 0842
T 0855
T 1762
T 1380
T 1763
T 0856
T 2048
T 2045
T 2044
T 2038
T 2039
