# Supplementary material for: Distribution of the four type VI secretion systems in Pseudomonas aeruginosa and classification of their core and accessory effectors
Source: Nat Commun. 2025 Jan 21;16:888. doi: 10.1038/s41467-024-54649-5 (PMC11751169; doi:10.1038/s41467-024-54649-5)
Supplement: Supplementary file 19 — Supplementary Dataset 17 [file 41467_2024_54649_MOESM19_ESM.pdf]

Distribution of the four type VI secretion systems in *Pseudomonas aeruginosa* and classification of their core and accessory effectors

Supplementary Data 17

To view this file, open in Preview (MacOS) or Microsoft Edge (Windows).

This file contains the results of the stochastic mapping of the effector genes. Each page shows the results of one effector gene. The respective effector gene is indicated at the top left corner of the page. Effectors are ordered by their associated T6SS and are found in the following order:

H1-T6SS effectors: *tas1*, *tse2, tse3, tse4, tse5, tse7, tse8, tne3, tse6, tse7a, tsd1, tse7c, tse7d, tse7e*

H2-T6SS effectors: *azu,* PA2066*, pldB, tle2, tplE, tseT, tseV, pldA, rhsP2, tle2, tle3, tle4b, tspE1a, tspE1b, tspE1c, vgrG2b*

H3-T6SS effectors: PA0256*, tepB, tseF, tepBa, tepBb*

The posterior probabilities of the stochastic mapping are indicated on each node of the phylogenetic tree. The tree is based on a core genome alignment of *P. aeruginosa* (n=1912) and *P. paraeruginosa* (n=5). Seven strains of the species *P. delhiensis, P. knackmussii, P. humi, P. jinjuensis, P. multiresinivorans, P. nitroreducens,* and *P. panipatensis* were used as an outgroup (not shown in the tree). The tree is based on the HKY+F+I model, rooted to the outgroup and distances are shown in substitutions per site.

Effector gene: tas1

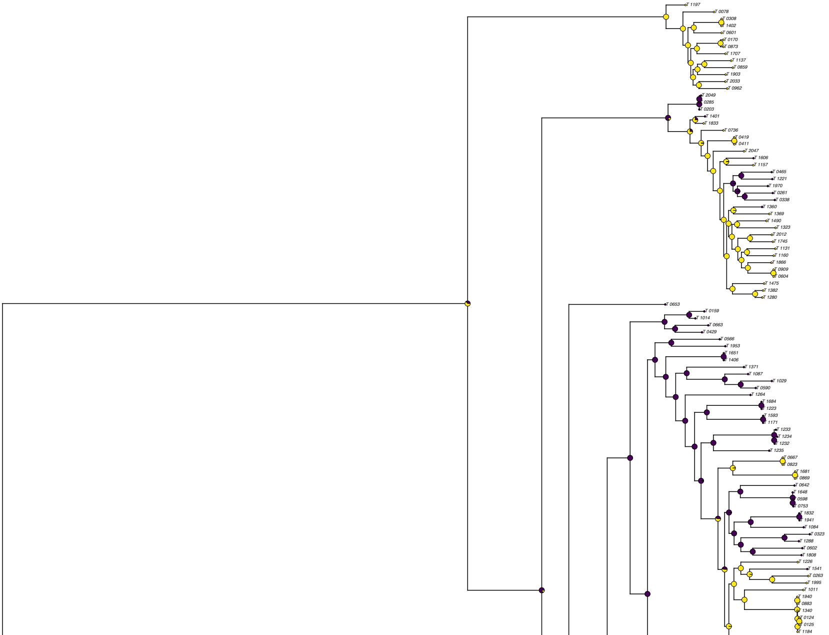

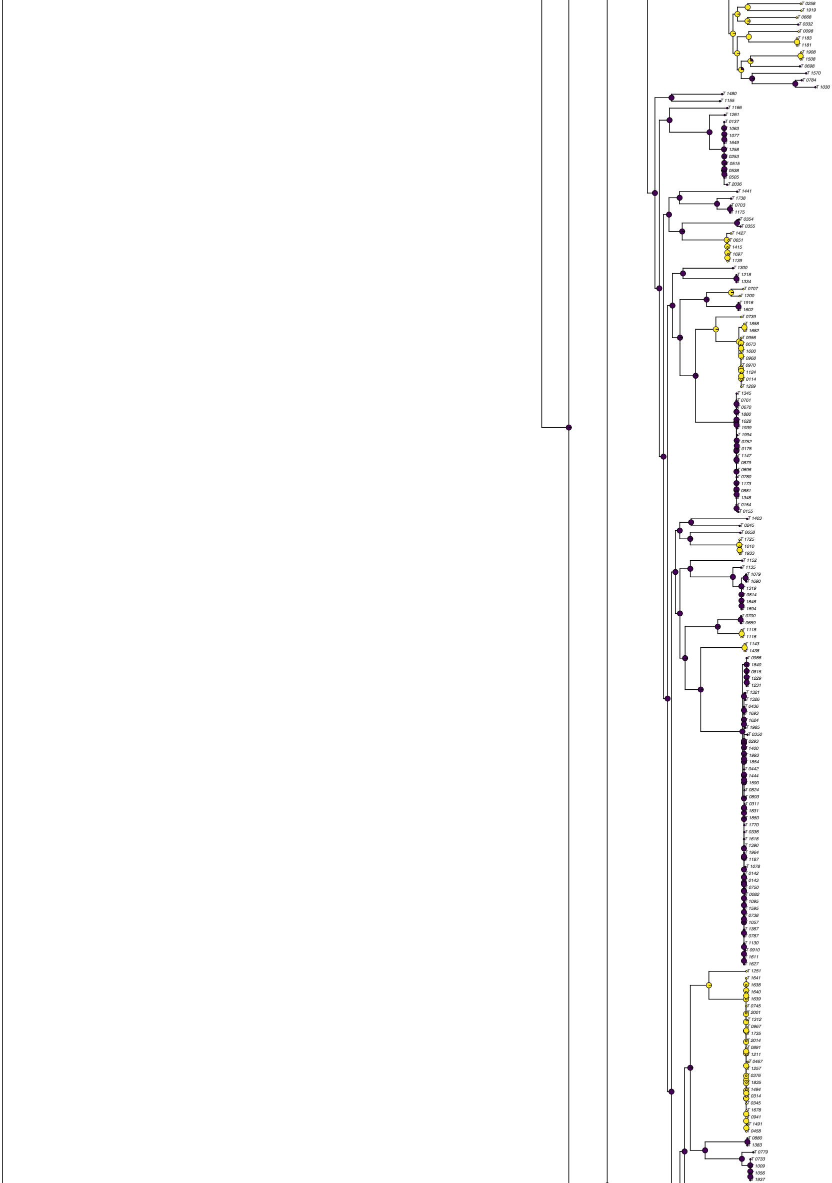

T 0258
T 1919
T 0668
T 0332
T 0098
T 1183
1181
T 1908
T 1508
T 0698
T 1570
T 0784
T 1030
T 1480
T 1155
T 1166
T 1261
T 0137
1063
1077
1649
1258
0253
0515
0538
0505
T 2036
T 1441
T 1738
T 0703
1175
T 0354
T 0355
T 1427
0651
1415
1697
1139
T 1300
T 1218
1334
T 0707
T 1200
T 1916
T 1602
T 0739
T 1858
1682
T 0956
0673
1600
0968
T 0970
1124
0114
T 1269
T 1345
T 0761
0670
1880
1628
1939
T 1994
0752
0175
T 1147
0879
0696
T 0780
1173
0881
T 1348
T 0154
T 0155
T 1403
T 0245
T 0658
T 1725
1010
1933
T 1152
T 1135
T 1079
T 1690
1319
0814
1646
1694
T 0700
T 0659
T 1118
T 1116
T 1143
1438
T 0986
1840
0815
1229
1231
T 1321
T 1326
0436
1693
1624
T 1985
T 0350
0293
1400
1993
1854
T 0442
1444
1590
T 0824
0893
T 0311
1831
1850
T 1770
T 0336
T 1618
1390
T 1964
1187
T 1078
0142
0143
0750
0082
1095
1595
0738
1057
T 1367
0787
T 1130
T 0910
1611
1627
T 1251
T 1641
1638
1640
1639
T 0745
T 2001
T 1312
T 0967
1735
2014
T 0891
1211
T 0467
1257
0376
1835
1494
0314
T 0345
T 1678
0941
T 1491
0458
T 0880
T 1383
T 0779
T 0733
1009
1056
T 1937

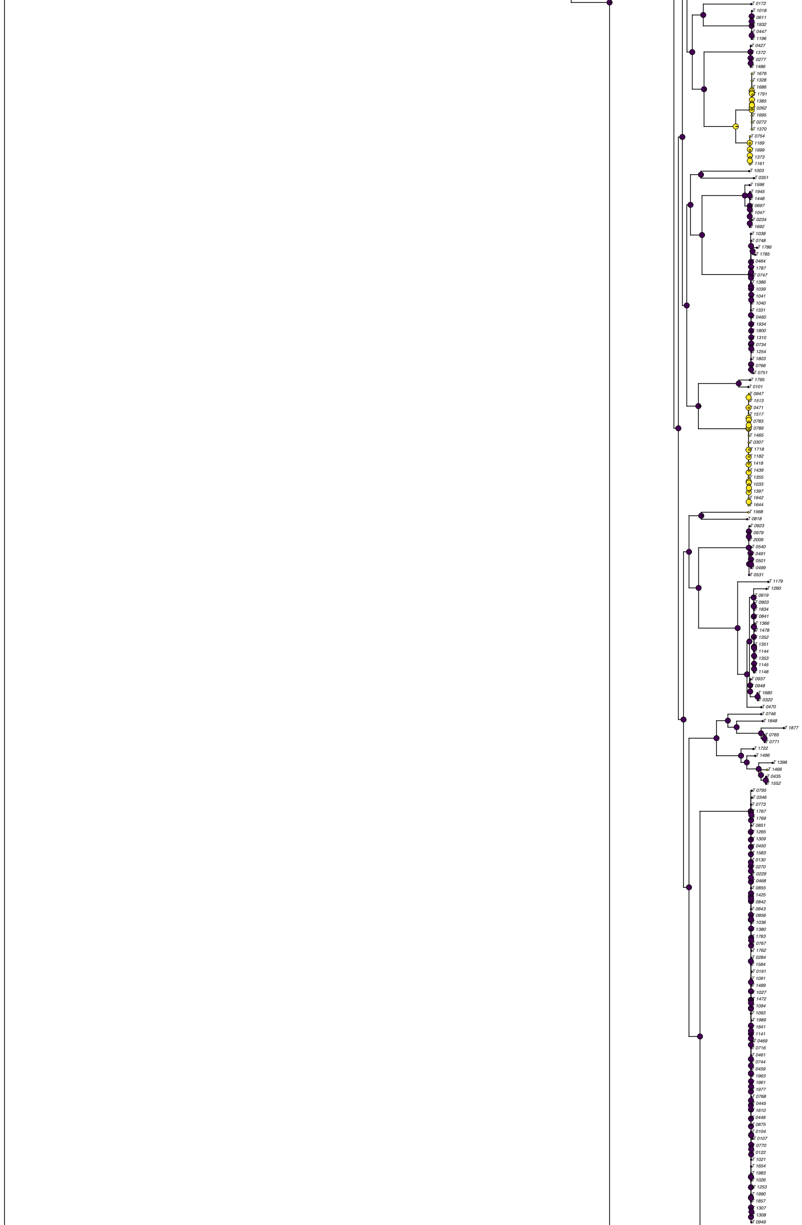
T 0172
T 1018
0611
1932
T 0447
T 1196
T 0427
1372
0277
T 1486
T 1676
T 1328
T 1686
1791
1385
0262
T 1695
T 0272
T 1370
T 0754
1169
1999
1373
1161
T 1003
T 0351
T 1596
T 1945
T 1448
0697
1047
T 0234
1692
T 1038
T 0748
T 1786
T 1785
0464
1787
T 0747
T 1386
1039
1041
1040
T 1331
0460
1934
1800
1310
0734
T 1254
T 1803
0766
T 0751
T 1795
T 0101
T 0947
T 1513
0471
T 1517
0783
0789
T 1465
T 0307
T 1718
T 1182
T 1418
T 1439
T 1355
1033
1397
T 1642
T 1644
T 1568
T 0818
T 0923
0979
T 2009
T 0540
0491
0501
T 0499
T 0531
T 1179
T 1260
T 0619
T 0903
T 1834
0841
T 1366
T 1478
1352
1351
1144
1353
1145
T 1148
T 0937
0948
T 1680
T 0322
T 0470
T 0746
T 1848
T 1877
T 0765
T 0771
T 1722
T 1496
T 1398
T 1466
T 0435
T 1552
T 0795
T 0346
T 0773
T 1767
T 1769
T 0851
T 1265
T 1309
0450
T 1583
T 0130
T 0270
T 0229
T 0468
T 0855
T 1425
0842
T 0843
0856
T 1036
T 1380
T 1763
T 0767
T 1762
T 0284
T 1584
T 0191
T 1091
T 1489
T 1027
T 1472
T 1094
T 1093
T 1989
T 1841
T 1141
T 0469
T 0716
T 0461
T 0744
T 0459
T 1963
T 1961
T 1977
T 0768
T 0445
T 1610
0448
T 0875
T 0104
T 0107
T 0770
T 0122
T 1021
T 1654
T 1983
T 1026
T 1253
T 1990
T 1857
T 1307
T 1308
T 0949

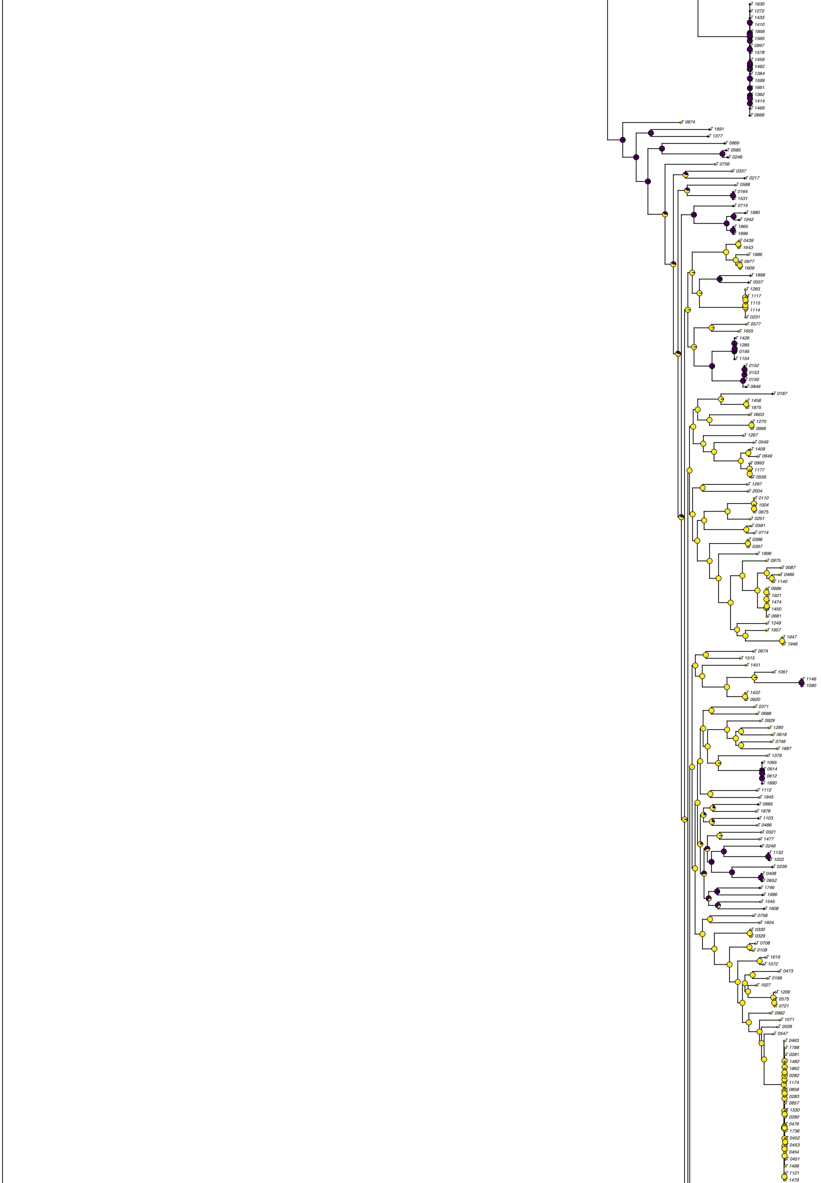

T 1630
T 1272
T 1433
T 1410
T 1856
T 1565
T 0997
T 1578
T 1459
T 1462
T 1384
T 1599
T 1661
T 1362
T 1414
T 1469
T 0669
T 0874
T 1691
T 1377
T 0969
T 0585
T 0246
T 0756
T 0337
T 0217
T 0588
T 0164
T 1531
T 0715
T 1980
T 1942
T 1965
T 1696
T 0439
T 1643
T 1986
T 0977
T 1609
T 1898
T 0557
T 1283
T 1117
T 1115
T 1114
T 0231
T 0577
T 1655
T 1426
T 1285
T 0195
T 1154
T 0152
T 0153
T 0150
T 0846
T 0187
T 1458
T 1875
T 0603
T 1270
T 0666
T 1267
T 0549
T 1409
T 0649
T 0993
T 1177
T 0556
T 1297
T 2004
T 0110
T 1004
T 0675
T 0251
T 0581
T 0714
T 0398
T 0397
T 1896
T 0975
T 0087
T 0489
T 1140
T 0686
T 1921
T 1474
T 1450
T 0681
T 1249
T 1957
T 1947
T 1946
T 0674
T 1515
T 1451
T 1061
T 1146
T 1090
T 1422
T 0920
T 0371
T 0688
T 0929
T 1290
T 0618
T 0749
T 1687
T 1379
T 1065
T 0914
T 0612
T 1890
T 1112
T 1845
T 0985
T 1878
T 1103
T 0488
T 0321
T 1477
T 0249
T 1132
T 1022
T 0239
T 0408
T 0652
T 1746
T 1996
T 1545
T 1608
T 0758
T 1604
T 0330
T 0329
T 0708
T 0108
T 1619
T 1072
T 0473
T 0166
T 1927
T 1206
T 0575
T 0721
T 0982
T 1071
T 2028
T 0547
T 0463
T 1798
T 0281
T 1482
T 1862
T 0282
T 1174
T 0858
T 0283
T 0857
T 1330
T 0280
T 0476
T 1736
T 0452
T 0453
T 0454
T 0451
T 1498
T 1121
T 1479

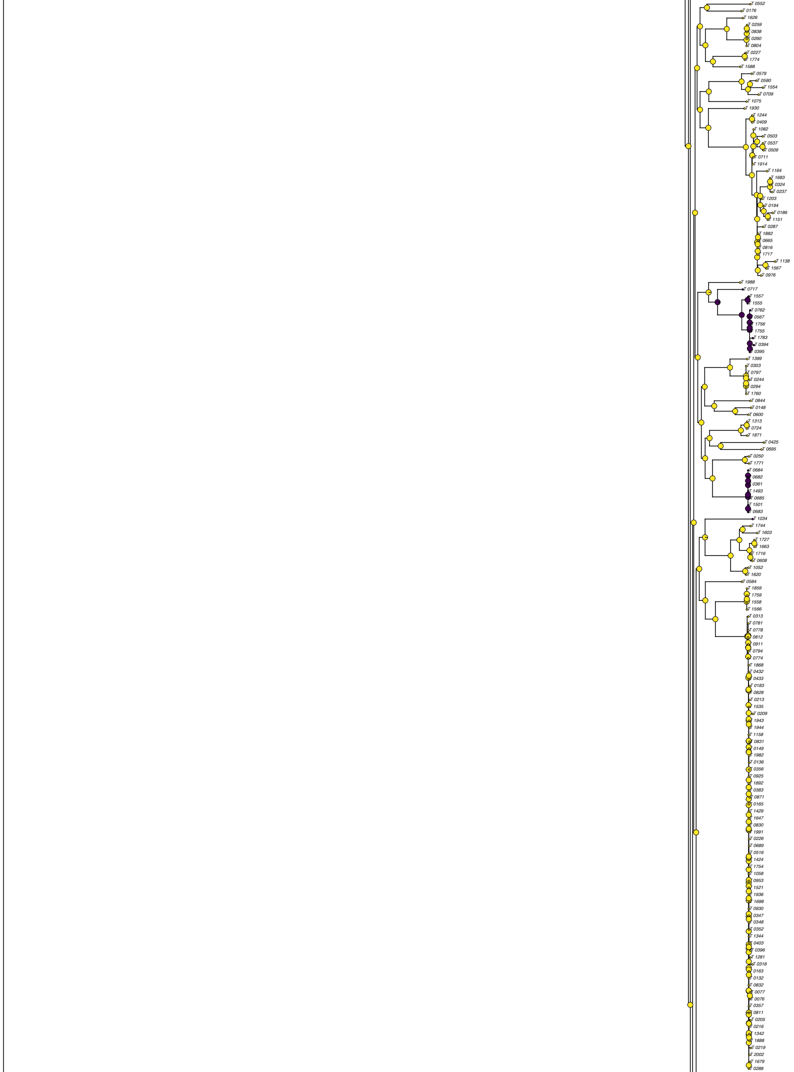
T 0552
T 0176
T 1626
T 0259
0838
0260
0804
T 0227
1774
T 1588
T 0579
T 0580
T 1554
T 0709
T 1075
T 1930
T 1244
T 0409
T 1082
T 0503
T 0537
T 0509
T 0711
T 1914
T 1164
T 1683
0324
T 0237
T 1203
T 0194
T 0186
1151
T 0287
T 1882
0665
0816
1717
T 1138
T 1567
T 0976
T 1988
T 0717
T 1557
1555
T 0762
0567
T 1756
1755
T 1783
T 0394
T 0395
T 1399
T 0303
T 0797
T 0244
0294
T 1760
T 0844
T 0148
T 0600
T 1313
T 0724
T 1871
T 0425
T 0695
T 0250
T 1771
T 0684
0682
0361
T 1493
T 0685
T 1501
T 0683
T 1034
T 1744
T 1603
T 1727
T 1663
T 1716
T 0608
T 1052
T 1620
T 0584
T 1859
1759
1558
T 1566
T 0313
T 0781
T 0778
0812
0911
T 0794
0774
T 1868
T 0432
0433
T 0183
T 0828
T 0213
1535
T 0209
1943
T 1944
T 1158
0831
0149
T 1982
T 0136
0356
T 0925
1892
0383
T 0871
0165
T 1429
T 1647
T 0830
T 1991
T 0226
T 0689
T 0516
1424
T 1754
T 1058
0953
1521
T 1936
1698
T 0930
0347
T 0348
T 0352
T 1344
T 0403
T 0396
T 1281
T 0318
0163
T 0132
T 0832
T 0077
T 0076
T 0357
0811
T 0205
T 0216
1342
T 1888
T 0219
T 2002
T 1679
0288

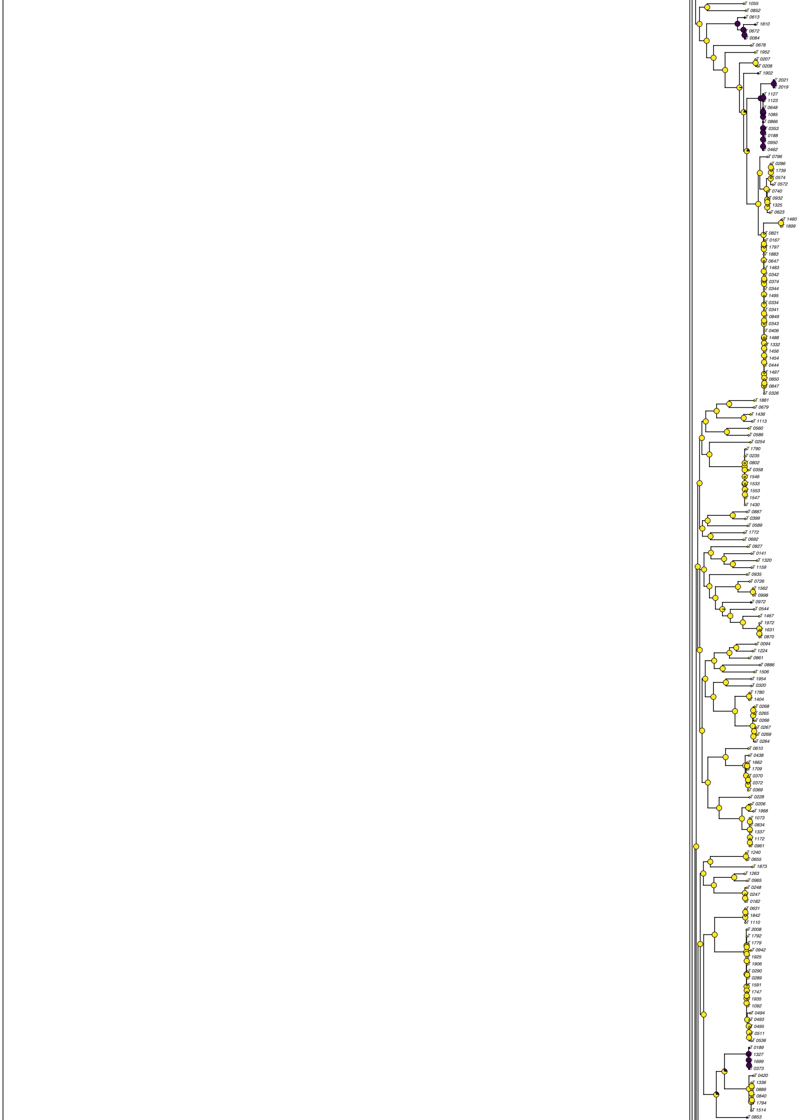
T 1055
T 0852
T 0613
T 1810
0672
T 0084
T 0678
T 1952
T 0207
T 0208
T 1902
T 2021
T 2019
T 1127
1123
T 0648
1085
T 0866
T 0353
T 0188
T 0950
T 0462
T 0796
T 0286
1739
0574
T 0572
T 0740
T 0932
1325
T 0623
T 1460
T 1899
T 0821
T 0167
1797
T 1883
0647
T 1483
0342
0374
T 0344
1495
T 0334
0341
T 0849
0343
T 0406
1488
T 1332
1456
1454
T 0444
1497
0850
0847
T 0326
T 1881
T 0679
T 1436
T 1113
T 0560
T 0586
T 0254
T 1790
T 0235
0802
T 0358
1546
1533
1553
1547
T 1430
T 0887
T 0399
T 0589
T 1772
T 0692
T 0827
T 0141
T 1320
T 1159
T 0935
T 0726
T 1562
T 0998
T 0972
T 0544
T 1467
T 1972
1631
0870
T 0094
T 1224
T 0861
T 0886
T 1506
T 1954
T 0320
T 1780
1404
T 0268
0265
T 0266
T 0267
T 0269
T 0264
T 0610
T 0438
T 1662
1709
T 0370
0372
T 0369
T 0228
T 0206
T 1968
T 1073
0834
1337
1172
0961
T 1240
T 0655
T 1873
T 1263
T 0965
T 0248
0247
T 0182
T 0631
1842
T 1110
T 2008
T 1792
T 1779
T 0942
1925
T 1906
T 0290
T 0289
T 1591
1747
1935
T 1092
T 0494
T 0493
0495
0511
T 0536
T 0189
1327
1699
T 0373
T 0420
T 1336
0889
T 0840
1794
T 1514
T 0853

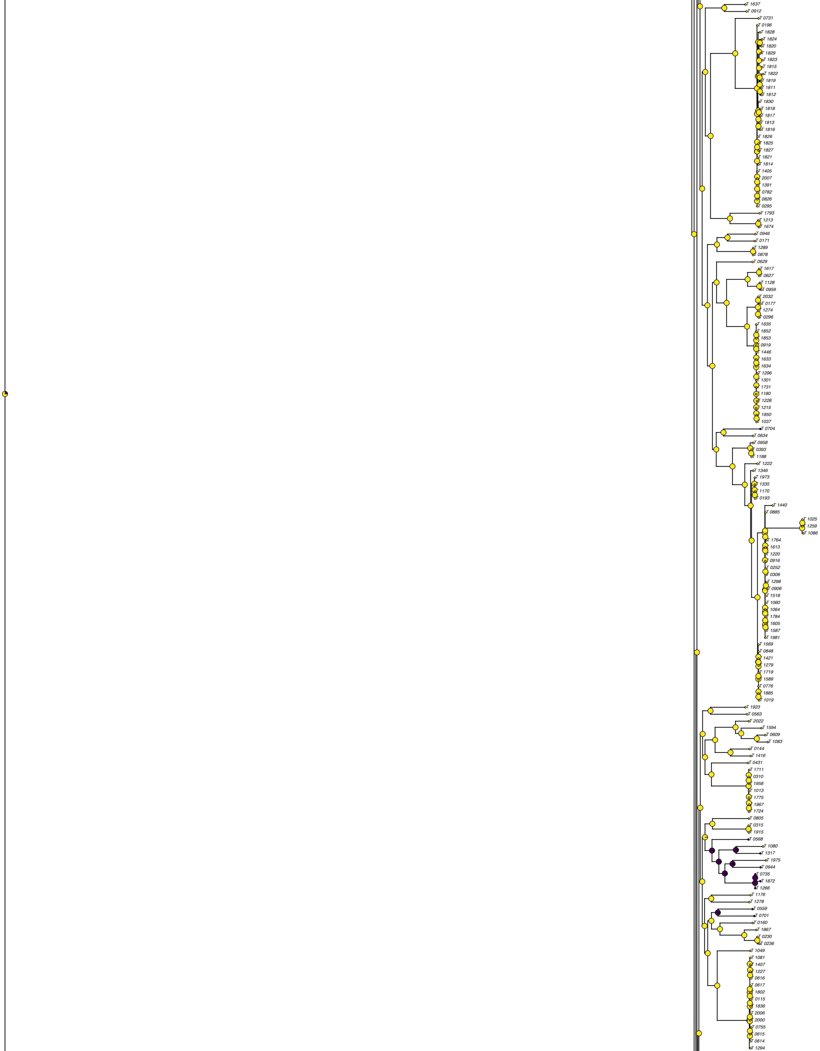
T 1637
T 0912
T 0731
T 0196
T 1828
T 1824
T 1820
T 1829
T 1823
T 1815
T 1822
T 1819
T 1811
T 1812
T 1830
T 1818
T 1817
T 1813
T 1816
T 1826
T 1825
T 1827
T 1821
T 1814
T 1405
T 2007
T 1391
T 0782
T 0826
T 0295
T 1793
T 1213
T 1674
T 0946
T 0171
T 1289
T 0878
T 0629
T 1617
T 0627
T 1128
T 0959
T 2032
T 0177
T 1274
T 0296
T 1635
T 1852
T 1853
T 0919
T 1446
T 1633
T 1634
T 1296
T 1301
T 1731
T 1180
T 1228
T 1215
T 1950
T 1037
T 0704
T 0634
T 0958
T 0393
T 1188
T 1222
T 1346
T 1973
T 1335
T 1170
T 0193
T 1440
T 0885
T 1025
T 1259
T 1086
T 1764
T 1613
T 1220
T 0916
T 0252
T 0306
T 1298
T 0906
T 1518
T 1060
T 1064
T 1784
T 1605
T 1587
T 1981
T 1569
T 0848
T 1421
T 1279
T 1719
T 1589
T 0776
T 1885
T 1019
T 1923
T 0563
T 2022
T 1594
T 0609
T 1083
T 0144
T 1416
T 0431
T 1711
T 0310
T 1958
T 1013
T 1775
T 1967
T 1724
T 0805
T 0315
T 1915
T 0568
T 1080
T 1317
T 1975
T 0944
T 0735
T 1872
T 1266
T 1176
T 1278
T 0559
T 0701
T 0160
T 1867
T 0230
T 0236
T 1049
T 1081
T 1407
T 1227
T 0616
T 0617
T 1802
T 0115
T 1836
T 2006
T 2000
T 0755
T 0615
T 0614
T 1294

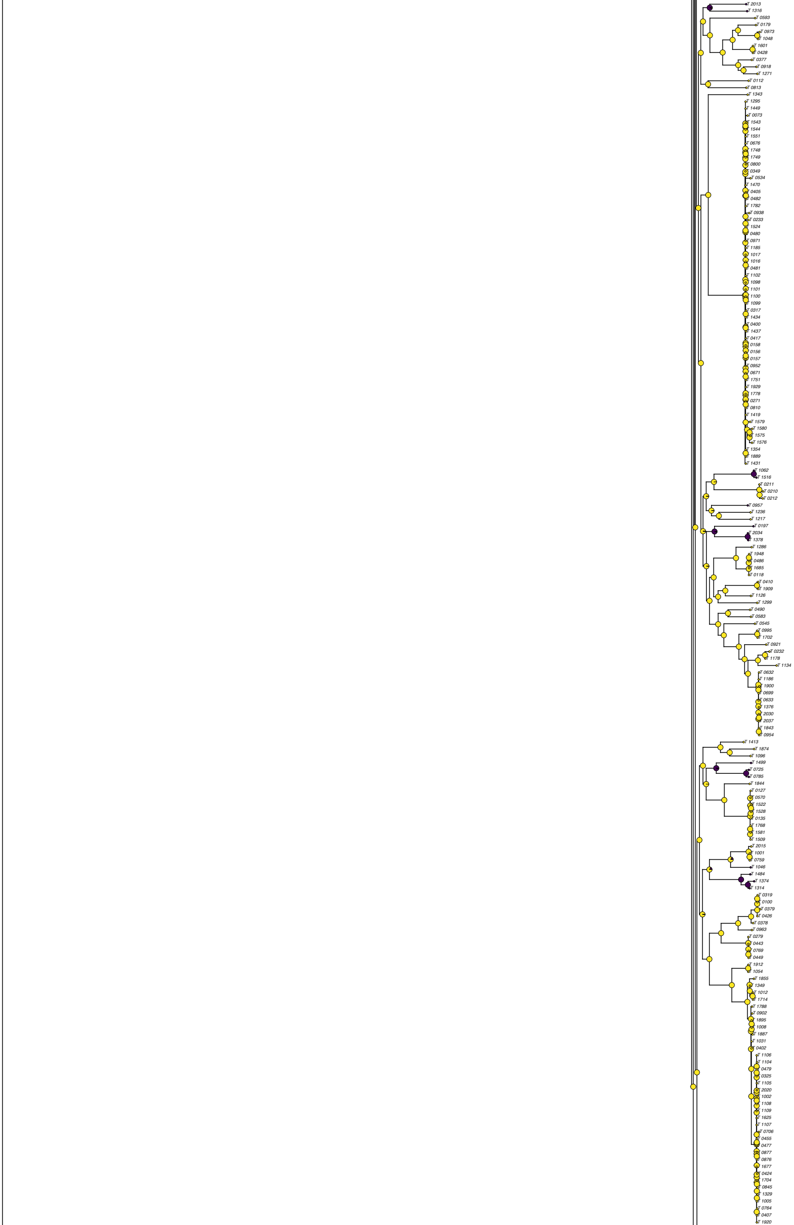
T 2013
T 1316
T 0593
T 0179
T 0973
T 1048
T 1601
T 0428
T 0377
T 0918
T 1271
T 0112
T 0813
T 1343
T 1295
T 1449
T 0073
T 1543
T 1544
T 1551
T 0676
T 1748
T 1749
T 0800
T 0349
T 0534
T 1470
T 0405
T 0482
T 1782
T 0938
T 0233
T 1524
T 0480
T 0971
T 1185
T 1017
T 1016
T 0481
T 1102
T 1098
T 1101
T 1100
T 1099
T 0317
T 1434
T 0400
T 1437
T 0417
T 0158
T 0156
T 0157
T 0952
T 0671
T 1751
T 1929
T 1778
T 0271
T 0810
T 1419
T 1579
T 1580
T 1575
T 1576
T 1354
T 1889
T 1431
T 1062
T 1516
T 0211
T 0210
T 0212
T 0957
T 1236
T 1217
T 0197
T 2034
T 1378
T 1286
T 1948
T 0486
T 1685
T 0118
T 0410
T 1909
T 1126
T 1299
T 0490
T 0583
T 0545
T 0995
T 1702
T 0921
T 0232
T 1178
T 1134
T 0632
T 1186
T 1900
T 0699
T 0633
T 1376
T 2030
T 2037
T 1843
T 0954
T 1413
T 1874
T 1096
T 1499
T 0725
T 0785
T 1844
T 0127
T 0570
T 1522
T 1528
T 0135
T 1768
T 1581
T 1509
T 2015
T 1001
T 0759
T 1046
T 1484
T 1374
T 1314
T 0319
T 0100
T 0379
T 0426
T 0378
T 0963
T 0279
T 0443
T 0769
T 0449
T 1912
T 1054
T 1855
T 1349
T 1012
T 1714
T 1788
T 0902
T 1895
T 1008
T 1887
T 1031
T 0402
T 1106
T 1104
T 0479
T 0325
T 1105
T 2020
T 1002
T 1108
T 1109
T 1625
T 1107
T 0706
T 0455
T 0477
T 0877
T 0876
T 1677
T 0424
T 1704
T 0845
T 1329
T 1005
T 0764
T 0407
T 1920

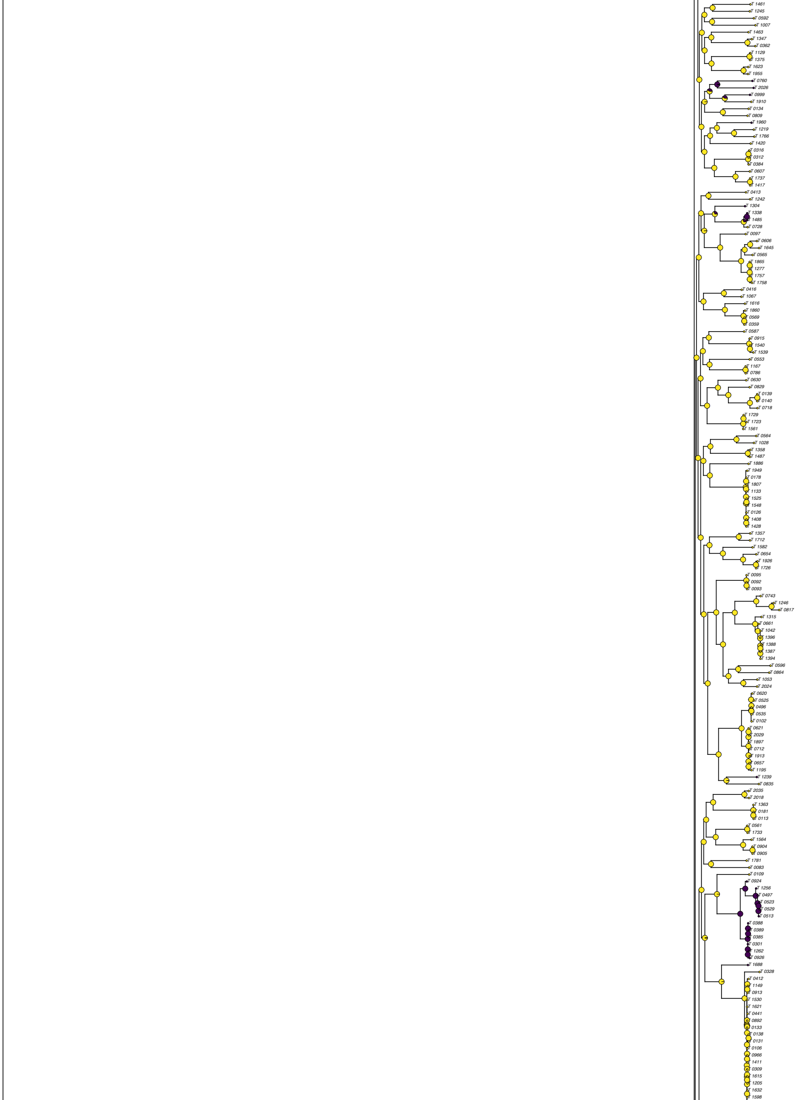
T 1461
T 1245
T 0592
T 1007
T 1463
T 1347
T 0362
T 1129
T 1375
T 1623
T 1955
T 0760
T 2026
T 0999
T 1910
T 0134
T 0809
T 1960
T 1219
T 1766
T 1420
T 0316
T 0312
T 0384
T 0607
T 1737
T 1417
T 0413
T 1242
T 1304
T 1338
T 1485
T 0728
T 0097
T 0606
T 1645
T 0565
T 1865
T 1277
T 1757
T 1758
T 0416
T 1067
T 1616
T 1860
T 0569
T 0359
T 0587
T 0915
T 1540
T 1539
T 0553
T 1167
T 0786
T 0630
T 0829
T 0139
T 0140
T 0718
T 1729
T 1723
T 1561
T 0564
T 1028
T 1358
T 1487
T 1886
T 1949
T 0178
T 1807
T 1133
T 1525
T 1548
T 0126
T 1408
T 1428
T 1357
T 1712
T 1582
T 0654
T 1926
T 1726
T 0095
T 0092
T 0093
T 0743
T 1246
T 0817
T 1315
T 0661
T 1042
T 1396
T 1388
T 1387
T 1394
T 0596
T 0864
T 1053
T 2024
T 0620
T 0525
T 0496
T 0535
T 0102
T 0621
T 2029
T 1897
T 0712
T 1913
T 0657
T 1195
T 1239
T 0835
T 2035
T 2018
T 1363
T 0181
T 0113
T 0561
T 1733
T 1564
T 0904
T 0905
T 1781
T 0083
T 0109
T 0924
T 1256
T 0497
T 0523
T 0529
T 0513
T 0388
T 0389
T 0385
T 0301
T 1262
T 0926
T 1688
T 0328
T 0412
T 1149
T 0913
T 1530
T 1621
T 0441
T 0892
T 0133
T 0138
T 0131
T 0106
T 0966
T 1411
T 0309
T 1615
T 1205
T 1632
T 1598

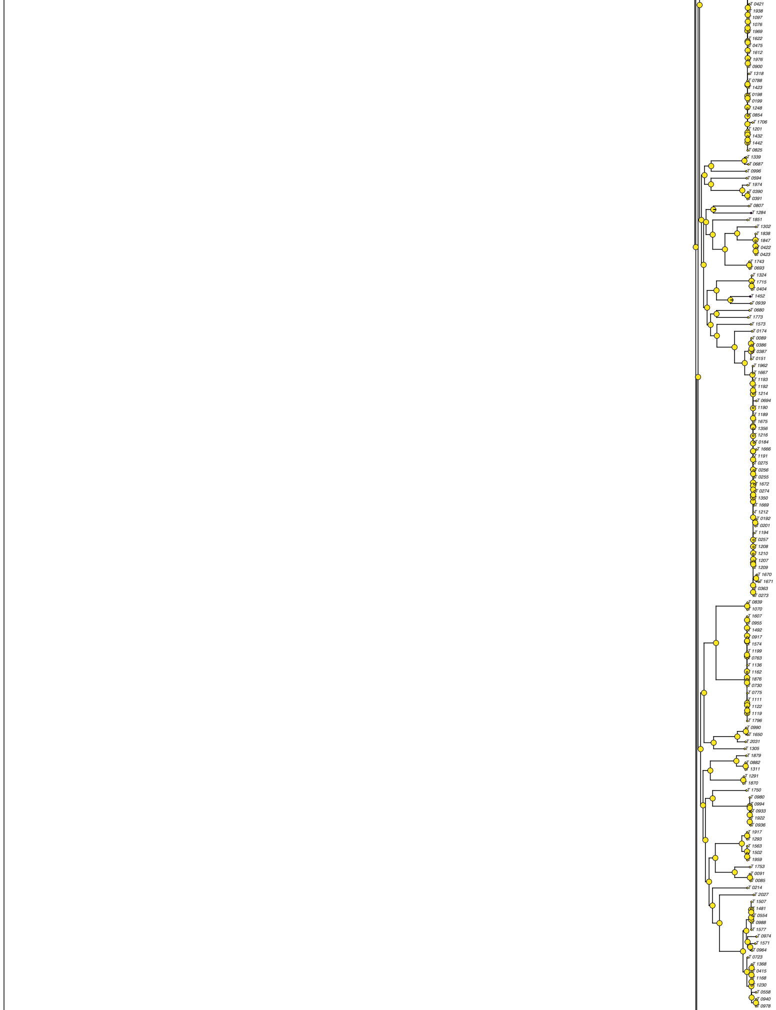

T 0421
T 1938
T 1097
T 1076
T 1969
T 1622
T 0475
T 1612
T 1976
T 0900
T 1318
T 0788
T 1423
T 0198
T 0199
T 1248
T 0854
T 1706
T 1201
T 1432
T 1442
T 0825
T 1339
T 0687
T 0996
T 0594
T 1974
T 0390
T 0391
T 0807
T 1284
T 1851
T 1302
T 1838
T 1847
T 0422
T 0423
T 1743
T 0693
T 1324
T 1715
T 0404
T 1452
T 0939
T 0680
T 1773
T 1573
T 0174
T 0089
T 0386
T 0387
T 0151
T 1962
T 1667
T 1193
T 1192
T 1214
T 0694
T 1190
T 1189
T 1675
T 1356
T 1216
T 0184
T 1666
T 1191
T 0275
T 0256
T 0255
T 1672
T 0274
T 1350
T 1669
T 1212
T 0192
T 0201
T 1194
T 0257
T 1208
T 1210
T 1207
T 1209
T 1670
T 1671
T 0363
T 0273
T 0839
T 1070
T 1607
T 0955
T 1492
T 0917
T 1574
T 1199
T 0763
T 1136
T 1162
T 1876
T 0730
T 0775
T 1111
T 1122
T 1119
T 1796
T 0990
T 1650
T 2031
T 1305
T 1879
T 0882
T 1311
T 1291
T 1870
T 1750
T 0980
T 0994
T 0933
T 1922
T 0936
T 1917
T 1293
T 1563
T 1502
T 1959
T 1753
T 0091
T 0085
T 0214
T 2027
T 1507
T 1481
T 0554
T 0988
T 1577
T 0974
T 1571
T 0964
T 0723
T 1368
T 0415
T 1168
T 1230
T 0558
T 0940
T 0978

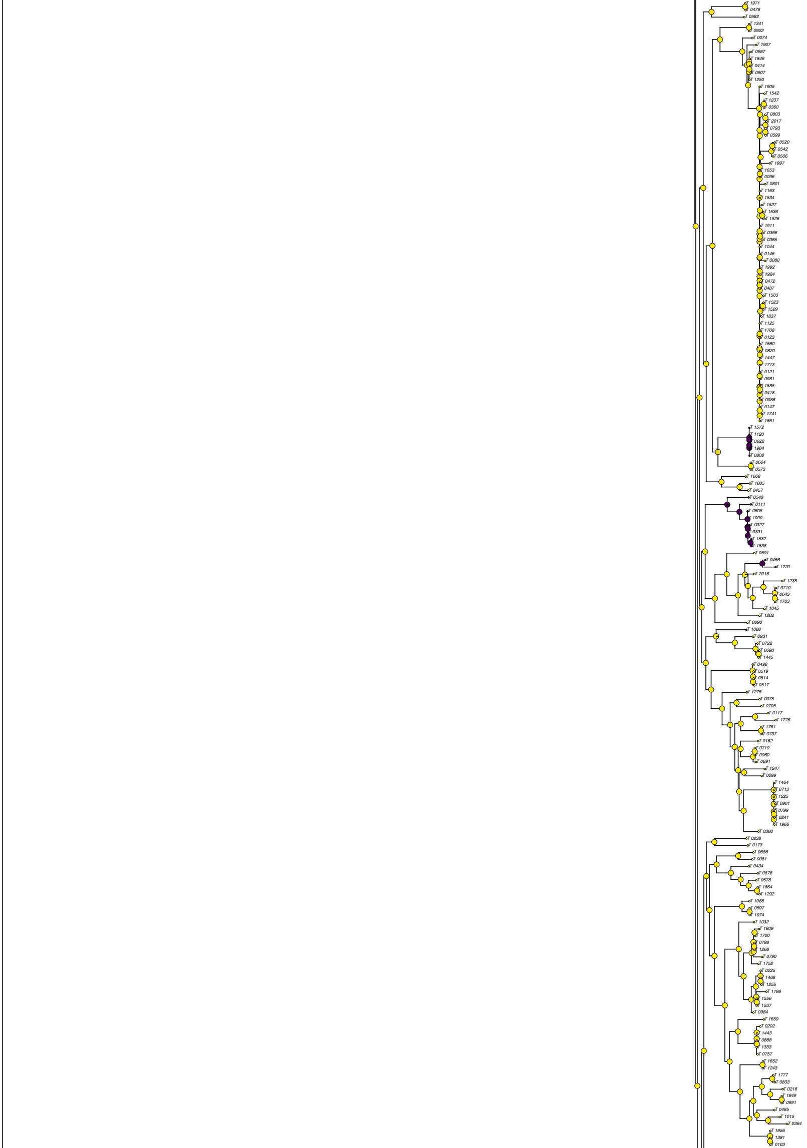
T 1971
T 0478
T 0582
T 1341
T 0922
T 0074
T 1907
T 0987
T 1846
T 0414
T 0907
T 1250
T 1905
T 1542
T 1237
T 0360
T 0803
T 2017
T 0793
T 0599
T 0520
T 0542
T 0506
T 1997
T 1653
T 0096
T 0801
T 1163
T 1534
T 1527
T 1536
T 1526
T 1911
T 0366
T 0365
T 1044
T 0146
T 0080
T 1992
T 1924
T 0472
T 0487
T 1503
T 1523
T 1529
T 1837
T 1125
T 1708
T 0123
T 1560
T 0820
T 1447
T 1713
T 0121
T 0981
T 1585
T 0418
T 0088
T 0147
T 1741
T 1861
T 1572
T 1120
T 0622
T 1984
T 0808
T 0664
T 0573
T 1068
T 1805
T 0457
T 0548
T 0111
T 0605
T 1000
T 0327
T 0331
T 1532
T 1538
T 0591
T 0456
T 1720
T 2016
T 1238
T 0710
T 0643
T 1703
T 1045
T 1282
T 0890
T 1088
T 0931
T 0722
T 0690
T 1445
T 0498
T 0519
T 0514
T 0517
T 1275
T 0075
T 0705
T 0117
T 1776
T 1761
T 0737
T 0162
T 0719
T 0960
T 0691
T 1247
T 0099
T 1464
T 0713
T 1225
T 0901
T 0799
T 0241
T 1966
T 0380
T 0238
T 0173
T 0656
T 0081
T 0434
T 0576
T 0578
T 1864
T 1292
T 1066
T 0597
T 1074
T 1032
T 1809
T 1700
T 0798
T 1268
T 0790
T 1752
T 0225
T 1468
T 1255
T 1198
T 1556
T 1537
T 0984
T 1659
T 0202
T 1443
T 0888
T 1333
T 0757
T 1652
T 1243
T 1777
T 0833
T 0218
T 1849
T 0991
T 0485
T 1015
T 0364
T 1956
T 1381
T 0103

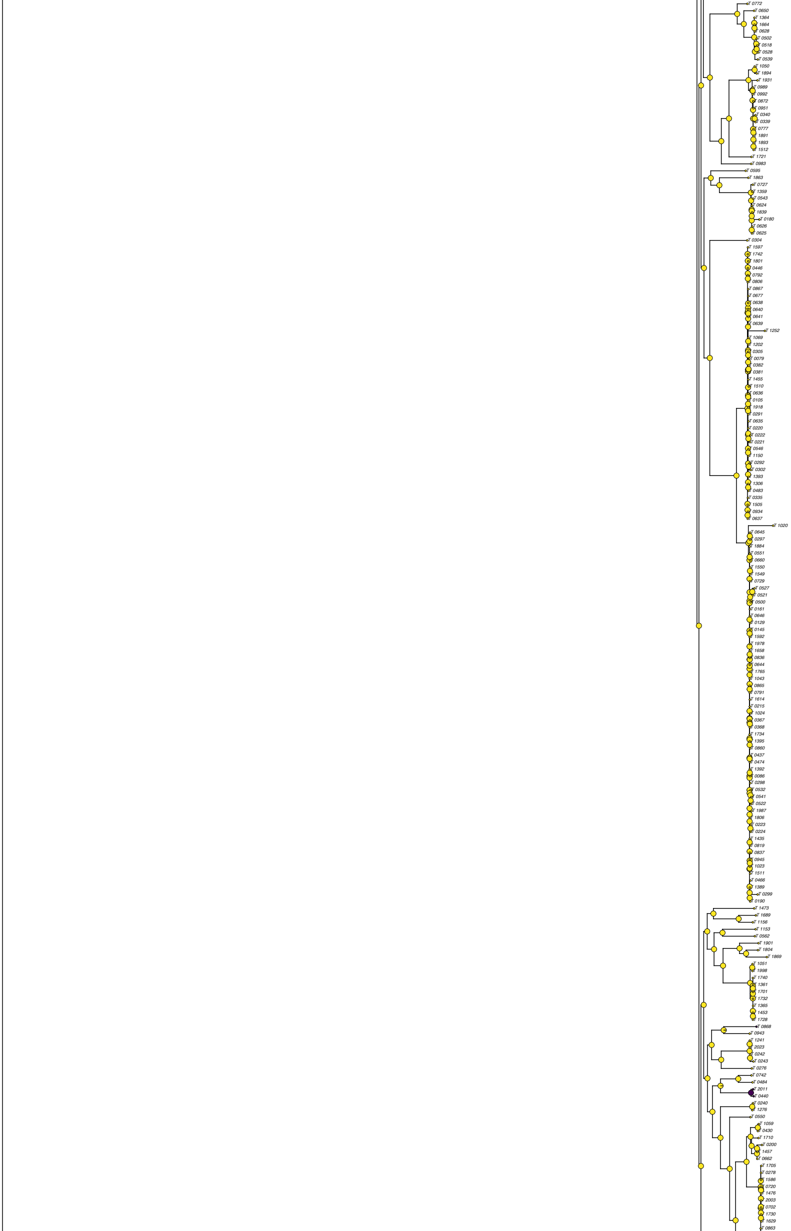
T 0772
T 0650
T 1364
T 1664
T 0628
T 0502
T 0518
T 0528
T 0539
T 1050
T 1894
T 1931
T 0989
T 0992
T 0872
T 0951
T 0340
T 0339
T 0777
T 1891
T 1893
T 1512
T 1721
T 0983
T 0595
T 1863
T 0727
T 1359
T 0543
T 0624
T 1839
T 0180
T 0626
T 0625
T 0304
T 1597
T 1742
T 1801
T 0446
T 0792
T 0806
T 0867
T 0677
T 0638
T 0640
T 0641
T 0639
T 1252
T 1069
T 1202
T 0305
T 0079
T 0382
T 0381
T 1455
T 1510
T 0636
T 0105
T 1918
T 0291
T 0635
T 0220
T 0222
T 0221
T 0546
T 1150
T 0292
T 0302
T 1393
T 1306
T 0483
T 0335
T 1505
T 0934
T 0637
T 1020
T 0645
T 0297
T 1884
T 0551
T 0660
T 1550
T 1549
T 0729
T 0527
T 0521
T 0500
T 0161
T 0646
T 0129
T 0145
T 1592
T 1978
T 1658
T 0836
T 0644
T 1765
T 1043
T 0865
T 0791
T 1614
T 0215
T 1024
T 0367
T 0368
T 1734
T 1395
T 0860
T 0437
T 0474
T 1392
T 0086
T 0298
T 0532
T 0541
T 0522
T 1987
T 1806
T 0223
T 0224
T 1435
T 0819
T 0837
T 0945
T 1023
T 1511
T 0466
T 1389
T 0299
T 0190
T 1473
T 1689
T 1156
T 1153
T 0562
T 1901
T 1804
T 1869
T 1051
T 1998
T 1740
T 1361
T 1701
T 1732
T 1365
T 1453
T 1728
T 0868
T 0943
T 1241
T 2023
T 0242
T 0243
T 0276
T 0742
T 0484
T 2011
T 0440
T 0240
T 1276
T 0550
T 1059
T 0430
T 1710
T 0200
T 1457
T 0662
T 1705
T 0278
T 1586
T 0720
T 1476
T 2003
T 0702
T 1730
T 1629
T 0863

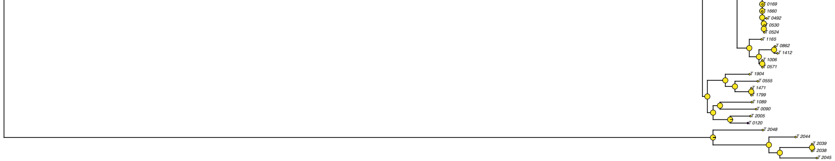
0169
1660
T 0492
T 0530
T 0524
T 1165
T 0862
T 1412
T 1006
T 0571
T 1904
T 0555
T 1471
T 1799
T 1089
T 0090
T 2005
T 0120
T 2048
T 2044
T 2039
T 2038
T 2045

Effector gene: tse2

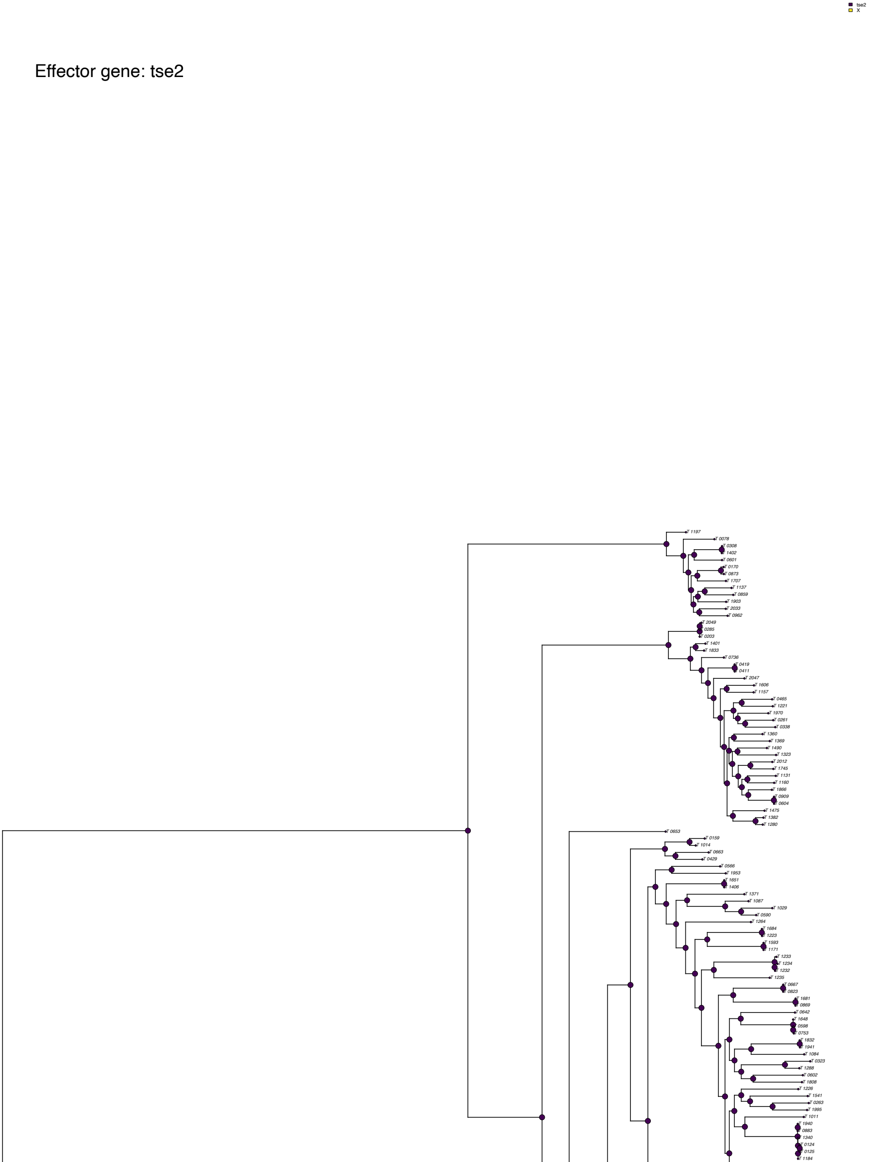

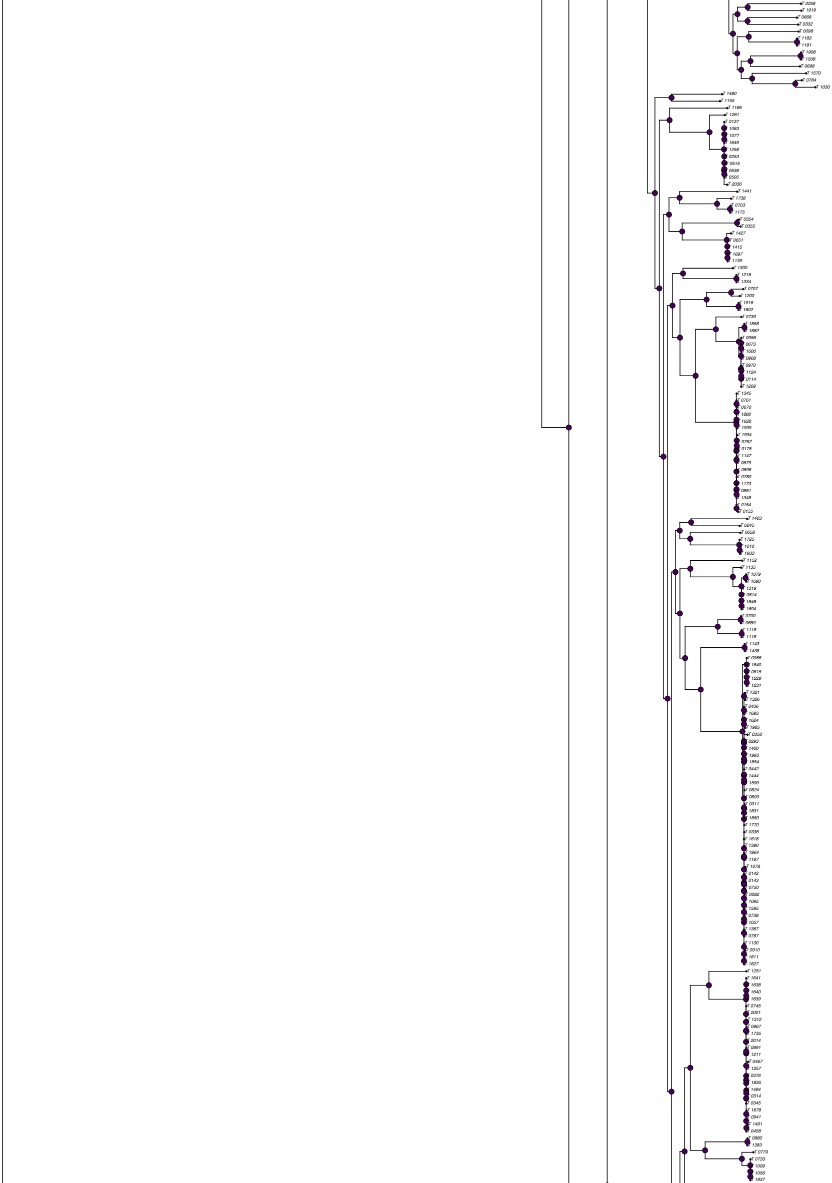

T 0258
T 1919
T 0668
T 0332
T 0098
T 1183
T 1181
T 1908
T 1508
T 0698
T 1570
T 0784
T 1030
T 1480
T 1155
T 1166
T 1261
T 0137
1063
1077
1649
1258
0253
0515
0538
0505
T 2036
T 1441
T 1738
T 0703
1175
T 0354
T 0355
T 1427
0651
1415
1697
1139
T 1300
T 1218
1334
T 0707
T 1200
T 1916
1602
T 0739
T 1858
1682
T 0956
0673
1600
0968
T 0970
1124
0114
T 1269
T 1345
T 0761
0670
1880
1628
1939
T 1994
0752
0175
T 1147
0879
T 0696
T 0780
1173
0881
T 1348
T 0154
T 0155
T 1403
T 0245
T 0658
T 1725
1010
1933
T 1152
T 1135
T 1079
1690
1319
0814
1646
1694
T 0700
0659
T 1118
1116
T 1143
1438
T 0986
1840
0815
1229
1231
T 1321
1326
0436
1693
1624
T 1985
T 0350
0293
1400
1993
1854
T 0442
1444
1590
T 0824
0893
T 0311
1831
1850
T 1770
T 0336
T 1618
1390
1964
1187
T 1078
0142
0143
0750
0082
1095
1595
0738
1057
T 1367
0787
T 1130
T 0910
1611
1627
T 1251
T 1641
1638
1640
1639
T 0745
2001
T 1312
T 0967
1735
2014
T 0891
1211
T 0467
1257
0376
1835
1494
0314
T 0345
T 1678
0941
T 1491
0458
T 0880
1383
T 0779
T 0733
1009
1056
1937

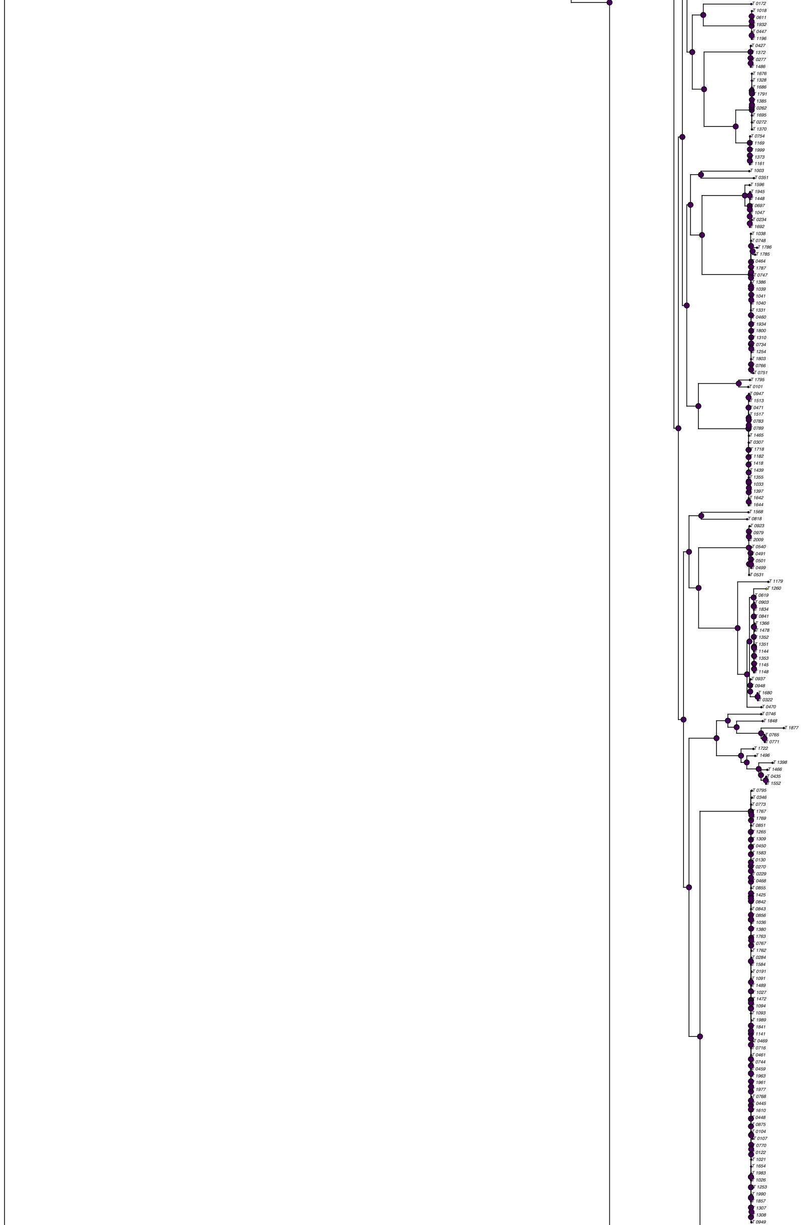
T 0172
T 1018
0611
1932
T 0447
T 1196
T 0427
1372
0277
T 1486
T 1676
T 1328
T 1686
1791
1385
T 0262
T 1695
T 0272
T 1370
T 0754
1169
1999
1373
T 1161
T 1003
T 0351
T 1596
T 1945
T 1448
0697
T 1047
T 0234
T 1692
T 1038
T 0748
T 1786
T 1785
0464
1787
T 0747
T 1386
1039
1041
1040
T 1331
0460
1934
1800
1310
0734
T 1254
T 1803
0766
T 0751
T 1795
T 0101
T 0947
T 1513
0471
T 1517
0783
0789
T 1465
T 0307
T 1718
T 1182
1418
T 1439
T 1355
1033
1397
T 1642
T 1644
T 1568
T 0818
T 0923
0979
T 2009
T 0540
0491
0501
T 0499
T 0531
T 1179
T 1260
T 0619
T 0903
T 1834
0841
T 1366
T 1478
1352
1351
1144
1353
1145
T 1148
T 0937
0948
T 1680
T 0322
T 0470
T 0746
T 1848
T 1877
T 0765
T 0771
T 1722
T 1496
T 1398
T 1466
T 0435
T 1552
T 0795
T 0346
T 0773
1767
1769
T 0851
1265
1309
0450
1583
0130
0270
T 0229
T 0468
T 0855
1425
0842
T 0843
0856
T 1036
1380
T 1763
0767
T 1762
T 0284
T 1584
T 0191
T 1091
T 1489
1027
T 1472
T 1094
T 1093
T 1989
1841
1141
T 0469
T 0716
T 0461
0744
0459
1963
1961
1977
T 0768
0445
1610
0448
T 0875
T 0104
T 0107
0770
0122
T 1021
T 1654
T 1983
1026
T 1253
1990
T 1857
T 1307
1308
T 0949

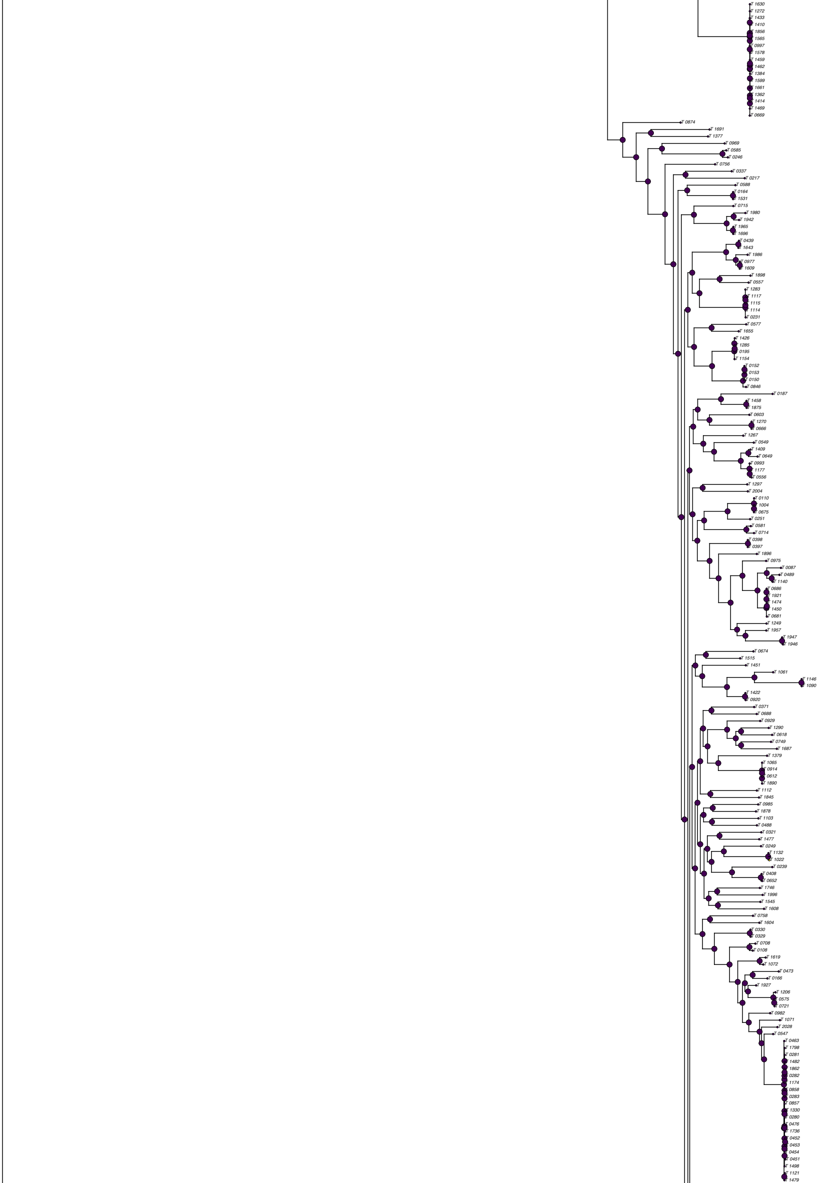
T 1630
T 1272
T 1433
T 1410
T 1856
T 1565
T 0997
T 1578
T 1459
T 1462
T 1384
T 1599
T 1661
T 1362
T 1414
T 1469
T 0669
T 0874
T 1691
T 1377
T 0969
T 0585
T 0246
T 0756
T 0337
T 0217
T 0588
T 0164
T 1531
T 0715
T 1980
T 1942
T 1965
T 1696
T 0439
T 1643
T 1986
T 0977
T 1609
T 1898
T 0557
T 1283
T 1117
T 1115
T 1114
T 0231
T 0577
T 1655
T 1426
T 1285
T 0195
T 1154
T 0152
T 0153
T 0150
T 0846
T 0187
T 1458
T 1875
T 0603
T 1270
T 0666
T 1267
T 0549
T 1409
T 0649
T 0993
T 1177
T 0556
T 1297
T 2004
T 0110
T 1004
T 0675
T 0251
T 0581
T 0714
T 0398
T 0397
T 1896
T 0975
T 0087
T 0489
T 1140
T 0686
T 1921
T 1474
T 1450
T 0681
T 1249
T 1957
T 1947
T 1946
T 0674
T 1515
T 1451
T 1061
T 1146
T 1090
T 1422
T 0920
T 0371
T 0688
T 0929
T 1290
T 0618
T 0749
T 1687
T 1379
T 1065
T 0914
T 0612
T 1890
T 1112
T 1845
T 0985
T 1878
T 1103
T 0488
T 0321
T 1477
T 0249
T 1132
T 1022
T 0239
T 0408
T 0652
T 1746
T 1996
T 1545
T 1608
T 0758
T 1604
T 0330
T 0329
T 0708
T 0108
T 1619
T 1072
T 0473
T 0166
T 1927
T 1206
T 0575
T 0721
T 0982
T 1071
T 2028
T 0547
T 0463
T 1798
T 0281
T 1482
T 1862
T 0282
T 1174
T 0858
T 0283
T 0857
T 1330
T 0280
T 0476
T 1736
T 0452
T 0453
T 0454
T 0451
T 1498
T 1121
T 1479

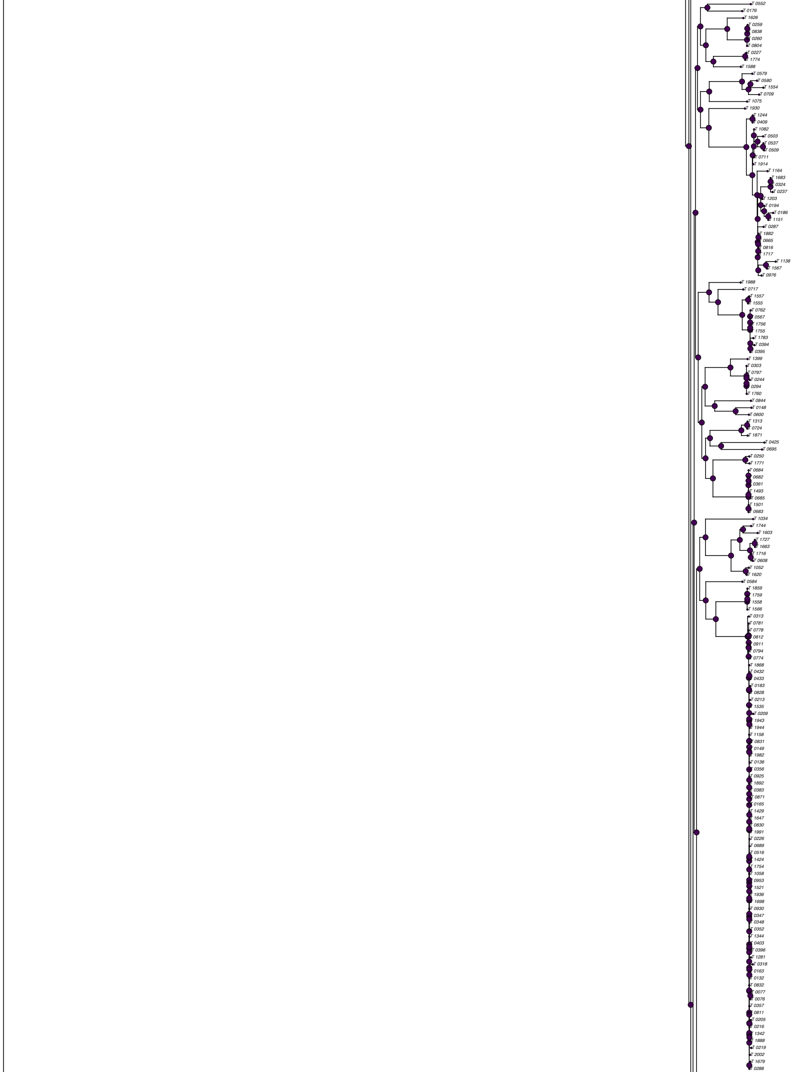
T 0552
T 0176
T 1626
T 0259
0838
0260
T 0804
T 0227
T 1774
T 1588
T 0579
T 0580
T 1554
T 0709
T 1075
T 1930
T 1244
T 0409
T 1082
T 0503
T 0537
T 0509
T 0711
T 1914
T 1164
T 1683
0324
T 0237
T 1203
T 0194
T 0186
T 1151
T 0287
T 1882
0665
T 0816
1717
T 1138
T 1567
T 0976
T 1988
T 0717
T 1557
T 1555
T 0762
0567
T 1756
T 1755
T 1783
T 0394
T 0395
T 1399
T 0303
T 0797
T 0244
0294
T 1760
T 0844
T 0148
T 0600
T 1313
T 0724
T 1871
T 0425
T 0695
T 0250
T 1771
T 0684
0682
0361
T 1493
T 0685
T 1501
T 0683
T 1034
T 1744
T 1603
T 1727
T 1663
T 1716
T 0608
T 1052
T 1620
T 0584
T 1859
1759
1558
T 1566
T 0313
T 0781
T 0778
0812
0911
T 0794
0774
T 1868
T 0432
0433
T 0183
T 0828
T 0213
1535
T 0209
1943
T 1944
T 1158
0831
0149
T 1982
T 0136
0356
T 0925
T 1892
0383
T 0871
0165
T 1429
T 1647
T 0830
1991
T 0226
T 0689
T 0516
1424
T 1754
T 1058
0953
1521
T 1936
1698
T 0930
0347
T 0348
T 0352
T 1344
T 0403
T 0396
T 1281
T 0318
0163
T 0132
T 0832
T 0077
T 0076
T 0357
0811
T 0205
T 0216
T 1342
T 1888
T 0219
T 2002
T 1679
T 0288

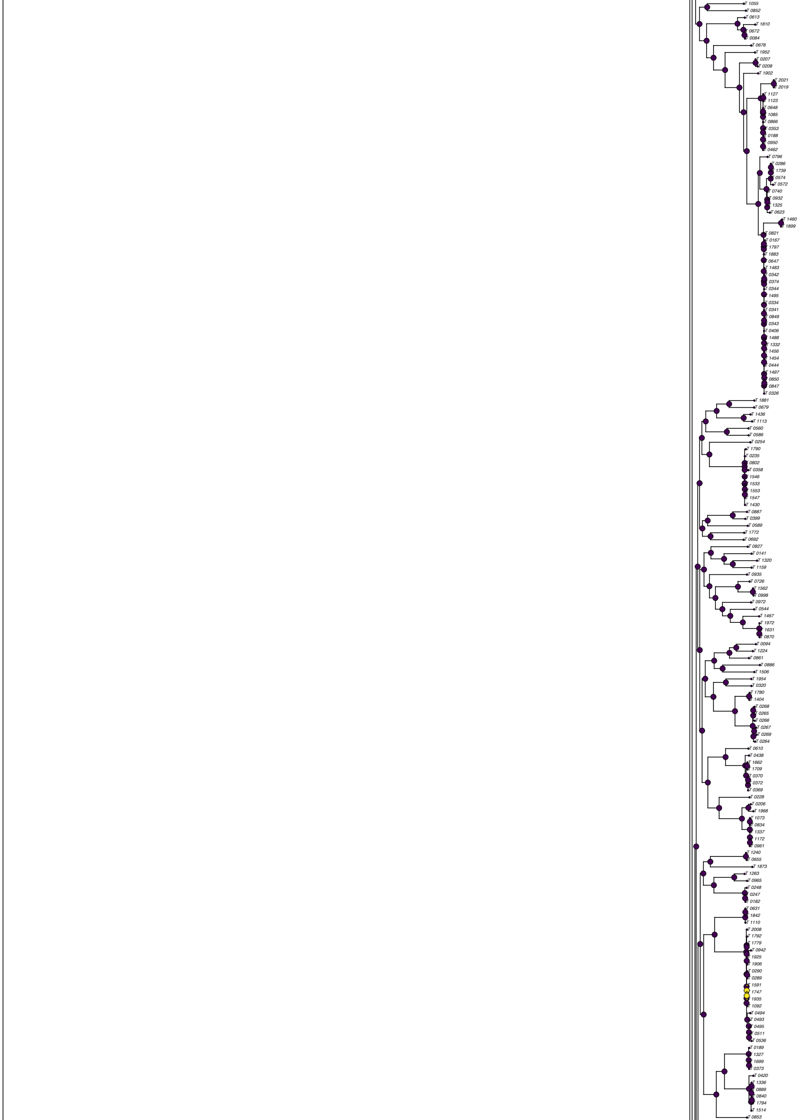
T 1055
T 0852
T 0613
T 1810
T 0672
T 0084
T 0678
T 1952
T 0207
T 0208
T 1902
T 2021
T 2019
T 1127
T 1123
T 0648
T 1085
T 0866
T 0353
T 0188
T 0950
T 0462
T 0796
T 0286
T 1739
T 0574
T 0572
T 0740
T 0932
T 1325
T 0623
T 1460
T 1899
T 0821
T 0167
T 1797
T 1883
T 0647
T 1483
T 0342
T 0374
T 0344
T 1495
T 0334
T 0341
T 0849
T 0343
T 0406
T 1488
T 1332
T 1456
T 1454
T 0444
T 1497
T 0850
T 0847
T 0326
T 1881
T 0679
T 1436
T 1113
T 0560
T 0586
T 0254
T 1790
T 0235
T 0802
T 0358
T 1546
T 1533
T 1553
T 1547
T 1430
T 0887
T 0399
T 0589
T 1772
T 0692
T 0827
T 0141
T 1320
T 1159
T 0935
T 0726
T 1562
T 0998
T 0972
T 0544
T 1467
T 1972
T 1631
T 0870
T 0094
T 1224
T 0861
T 0886
T 1506
T 1954
T 0320
T 1780
T 1404
T 0268
T 0265
T 0266
T 0267
T 0269
T 0264
T 0610
T 0438
T 1662
T 1709
T 0370
T 0372
T 0369
T 0228
T 0206
T 1968
T 1073
T 0834
T 1337
T 1172
T 0961
T 1240
T 0655
T 1873
T 1263
T 0965
T 0248
T 0247
T 0182
T 0631
T 1842
T 1110
T 2008
T 1792
T 1779
T 0942
T 1925
T 1906
T 0290
T 0289
T 1591
T 1747
T 1935
T 1092
T 0494
T 0493
T 0495
T 0511
T 0536
T 0189
T 1327
T 1699
T 0373
T 0420
T 1336
T 0889
T 0840
T 1794
T 1514
T 0853

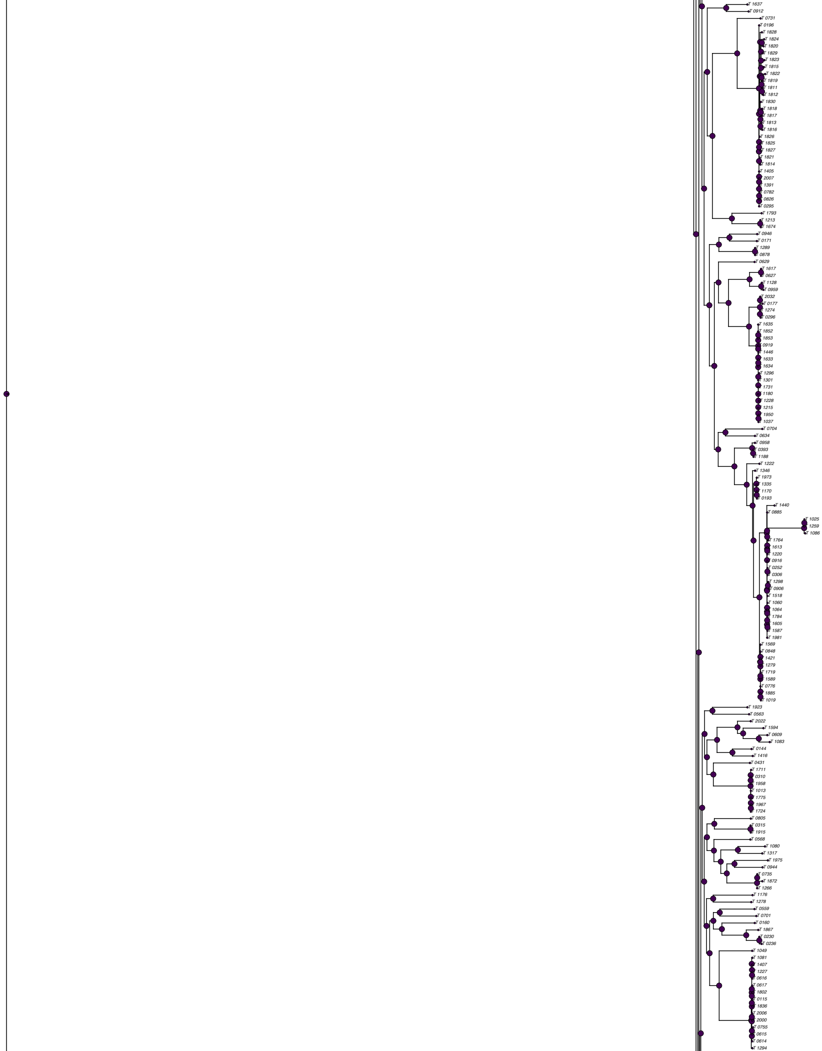
T 1637
T 0912
T 0731
T 0196
T 1828
T 1824
T 1820
T 1829
T 1823
T 1815
T 1822
T 1819
T 1811
T 1812
T 1830
T 1818
T 1817
T 1813
T 1816
T 1826
T 1825
T 1827
T 1821
T 1814
T 1405
2007
1391
0782
0826
T 0295
T 1793
T 1213
T 1674
T 0946
T 0171
T 1289
T 0878
T 0629
T 1617
T 0627
T 1128
T 0959
T 2032
T 0177
T 1274
T 0296
T 1635
T 1852
1853
0919
T 1446
1633
1634
T 1296
T 1301
1731
1180
1228
1215
1950
T 1037
T 0704
T 0634
T 0958
T 0393
T 1188
T 1222
T 1346
T 1973
1335
1170
T 0193
T 1440
T 0885
T 1025
1259
T 1086
T 1764
1613
T 1220
0916
T 0252
T 0306
T 1298
T 0906
T 1518
T 1060
1064
T 1784
1605
T 1587
T 1981
T 1569
T 0848
1421
1279
T 1719
1589
T 0776
1885
T 1019
T 1923
T 0563
T 2022
T 1594
T 0609
T 1083
T 0144
T 1416
T 0431
T 1711
0310
1958
T 1013
1775
1967
1724
T 0805
T 0315
T 1915
T 0568
T 1080
T 1317
T 1975
T 0944
T 0735
T 1872
T 1266
T 1176
T 1278
T 0559
T 0701
T 0160
T 1867
T 0230
T 0236
T 1049
T 1081
1407
1227
0616
T 0617
1802
T 0115
1836
T 2006
2000
T 0755
0615
T 0614
T 1294

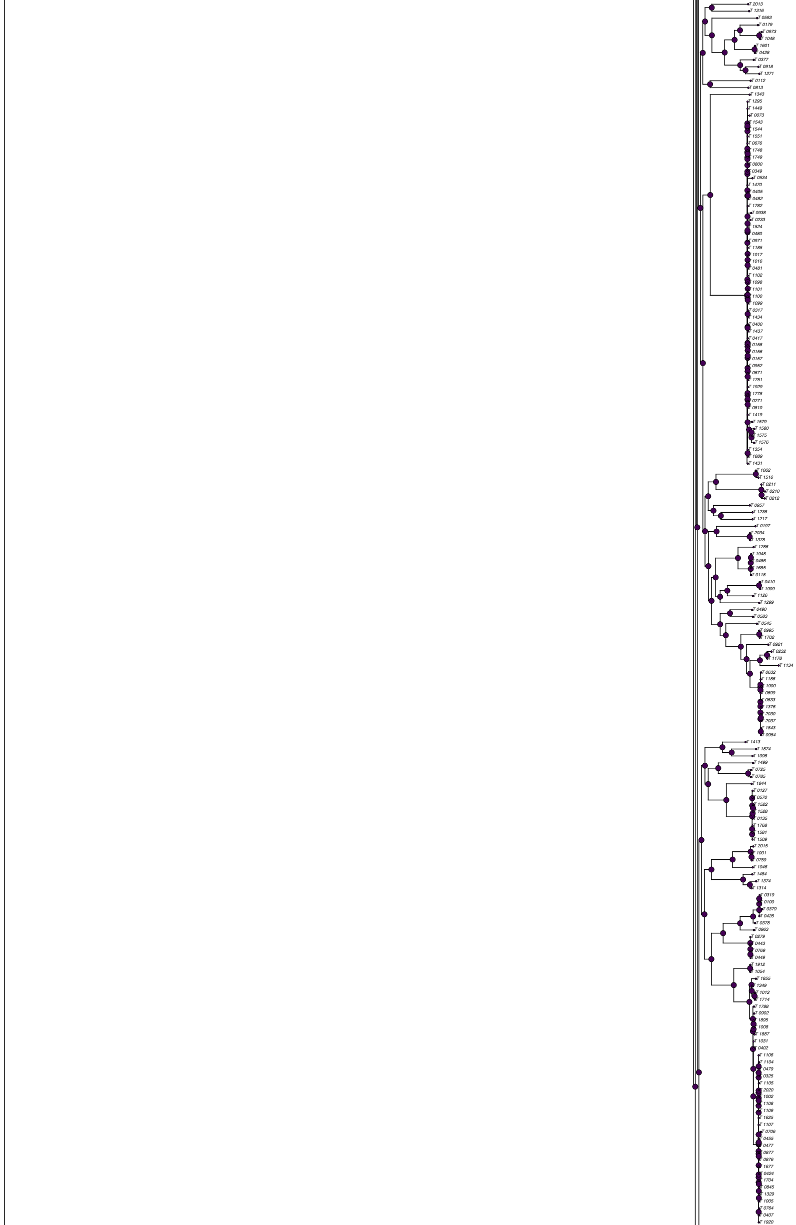

T 2013
T 1316
T 0593
T 0179
T 0973
T 1048
T 1601
T 0428
T 0377
T 0918
T 1271
T 0112
T 0813
T 1343
T 1295
T 1449
T 0073
T 1543
T 1544
T 1551
T 0676
T 1748
T 1749
T 0800
T 0349
T 0534
T 1470
T 0405
T 0482
T 1782
T 0938
T 0233
T 1524
T 0480
T 0971
T 1185
T 1017
T 1016
T 0481
T 1102
T 1098
T 1101
T 1100
T 1099
T 0317
T 1434
T 0400
T 1437
T 0417
T 0158
T 0156
T 0157
T 0952
T 0671
T 1751
T 1929
T 1778
T 0271
T 0810
T 1419
T 1579
T 1580
T 1575
T 1576
T 1354
T 1889
T 1431
T 1062
T 1516
T 0211
T 0210
T 0212
T 0957
T 1236
T 1217
T 0197
T 2034
T 1378
T 1286
T 1948
T 0486
T 1685
T 0118
T 0410
T 1909
T 1126
T 1299
T 0490
T 0583
T 0545
T 0995
T 1702
T 0921
T 0232
T 1178
T 1134
T 0632
T 1186
T 1900
T 0699
T 0633
T 1376
T 2030
T 2037
T 1843
T 0954
T 1413
T 1874
T 1096
T 1499
T 0725
T 0785
T 1844
T 0127
T 0570
T 1522
T 1528
T 0135
T 1768
T 1581
T 1509
T 2015
T 1001
T 0759
T 1046
T 1484
T 1374
T 1314
T 0319
T 0100
T 0379
T 0426
T 0378
T 0963
T 0279
T 0443
T 0769
T 0449
T 1912
T 1054
T 1855
T 1349
T 1012
T 1714
T 1788
T 0902
T 1895
T 1008
T 1887
T 1031
T 0402
T 1106
T 1104
T 0479
T 0325
T 1105
T 2020
T 1002
T 1108
T 1109
T 1625
T 1107
T 0706
T 0455
T 0477
T 0877
T 0876
T 1677
T 0424
T 1704
T 0845
T 1329
T 1005
T 0764
T 0407
T 1920

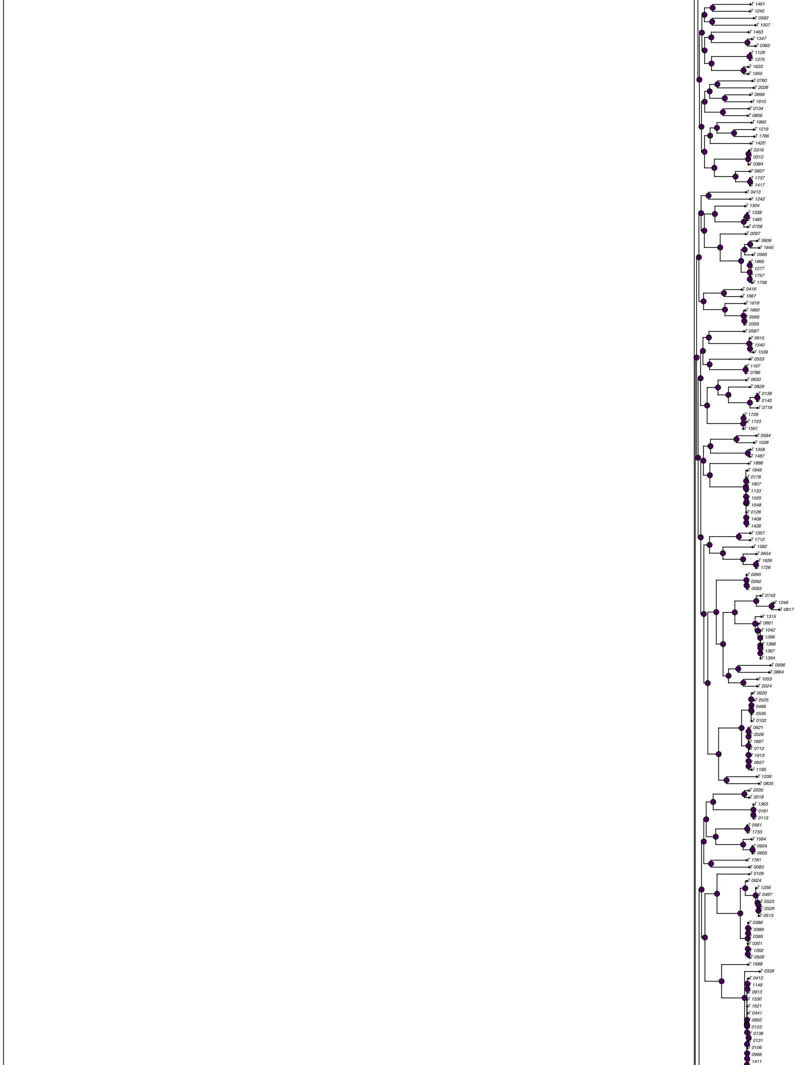
T 1461
T 1245
T 0592
T 1007
T 1463
T 1347
T 0362
T 1129
T 1375
T 1623
T 1955
T 0760
T 2026
T 0999
T 1910
T 0134
T 0809
T 1960
T 1219
T 1766
T 1420
T 0316
T 0312
T 0384
T 0607
T 1737
T 1417
T 0413
T 1242
T 1304
T 1338
T 1485
T 0728
T 0097
T 0606
T 1645
T 0565
T 1865
T 1277
T 1757
T 1758
T 0416
T 1067
T 1616
T 1860
T 0569
T 0359
T 0587
T 0915
T 1540
T 1539
T 0553
T 1167
T 0786
T 0630
T 0829
T 0139
T 0140
T 0718
T 1729
T 1723
T 1561
T 0564
T 1028
T 1358
T 1487
T 1886
T 1949
T 0178
T 1807
T 1133
T 1525
T 1548
T 0126
T 1408
T 1428
T 1357
T 1712
T 1582
T 0654
T 1926
T 1726
T 0095
T 0092
T 0093
T 0743
T 1246
T 0817
T 1315
T 0661
T 1042
T 1396
T 1388
T 1387
T 1394
T 0596
T 0864
T 1053
T 2024
T 0620
T 0525
T 0496
T 0535
T 0102
T 0621
T 2029
T 1897
T 0712
T 1913
T 0657
T 1195
T 1239
T 0835
T 2035
T 2018
T 1363
T 0181
T 0113
T 0561
T 1733
T 1564
T 0904
T 0905
T 1781
T 0083
T 0109
T 0924
T 1256
T 0497
T 0523
T 0529
T 0513
T 0388
T 0389
T 0385
T 0301
T 1262
T 0926
T 1688
T 0328
T 0412
T 1149
T 0913
T 1530
T 1621
T 0441
T 0892
T 0133
T 0138
T 0131
T 0106
T 0966
T 1411

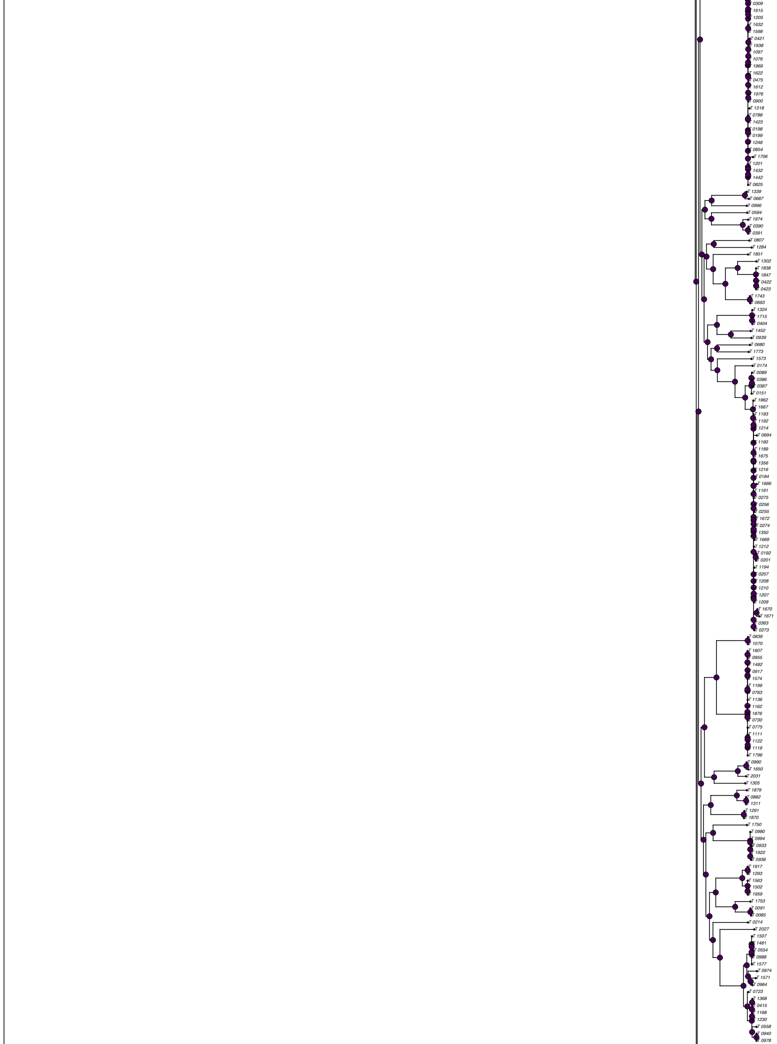

T 0309
T 1615
T 1205
T 1632
T 1598
T 0421
T 1938
T 1097
T 1076
T 1969
T 1622
T 0475
T 1612
T 1976
T 0900
T 1318
T 0788
T 1423
T 0198
T 0199
T 1248
T 0854
T 1706
T 1201
T 1432
T 1442
T 0825
T 1339
T 0687
T 0996
T 0594
T 1974
T 0390
T 0391
T 0807
T 1284
T 1851
T 1302
T 1838
T 1847
T 0422
T 0423
T 1743
T 0693
T 1324
T 1715
T 0404
T 1452
T 0939
T 0680
T 1773
T 1573
T 0174
T 0089
T 0386
T 0387
T 0151
T 1962
T 1667
T 1193
T 1192
T 1214
T 0694
T 1190
T 1189
T 1675
T 1356
T 1216
T 0184
T 1666
T 1191
T 0275
T 0256
T 0255
T 1672
T 0274
T 1350
T 1669
T 1212
T 0192
T 0201
T 1194
T 0257
T 1208
T 1210
T 1207
T 1209
T 1670
T 1671
T 0363
T 0273
T 0839
T 1070
T 1607
T 0955
T 1492
T 0917
T 1574
T 1199
T 0763
T 1136
T 1162
T 1876
T 0730
T 0775
T 1111
T 1122
T 1119
T 1796
T 0990
T 1650
T 2031
T 1305
T 1879
T 0882
T 1311
T 1291
T 1870
T 1750
T 0980
T 0994
T 0933
T 1922
T 0936
T 1917
T 1293
T 1563
T 1502
T 1959
T 1753
T 0091
T 0085
T 0214
T 2027
T 1507
T 1481
T 0554
T 0988
T 1577
T 0974
T 1571
T 0964
T 0723
T 1368
T 0415
T 1168
T 1230
T 0558
T 0940
T 0978

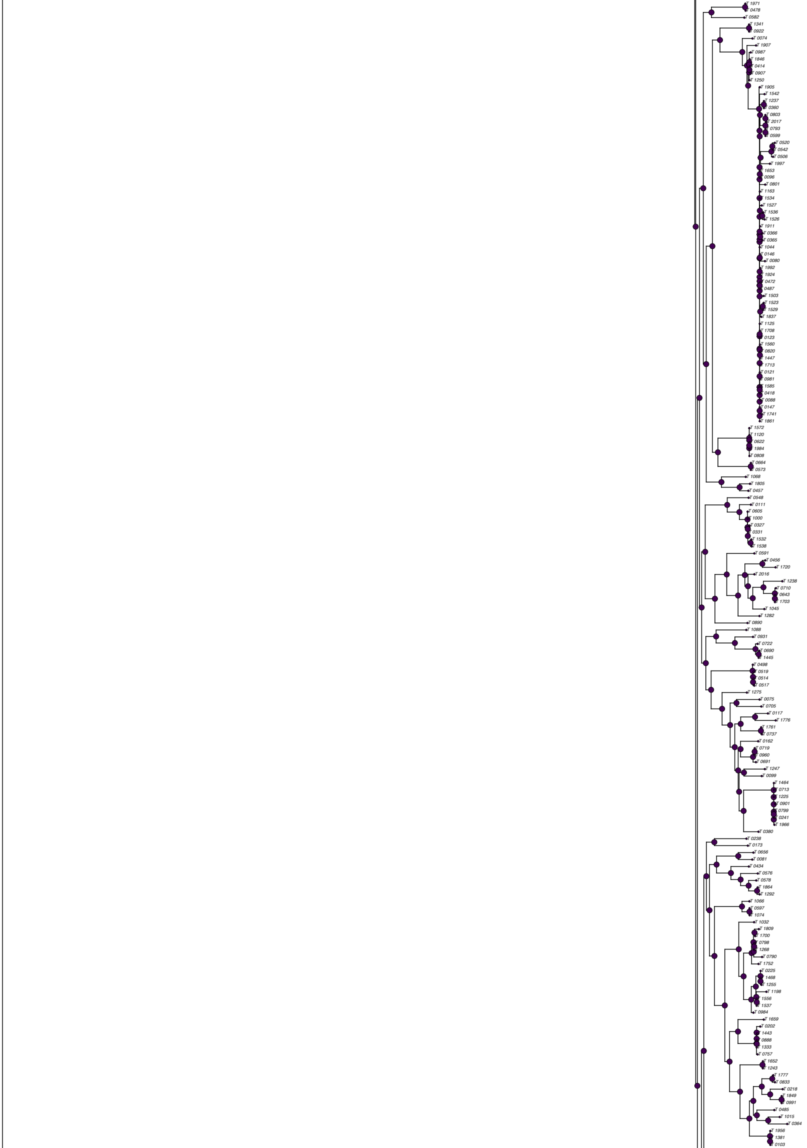
T 1971
T 0478
T 0582
T 1341
T 0922
T 0074
T 1907
T 0987
T 1846
T 0414
T 0907
T 1250
T 1905
T 1542
T 1237
T 0360
T 0803
T 2017
T 0793
T 0599
T 0520
T 0542
T 0506
T 1997
T 1653
T 0096
T 0801
T 1163
T 1534
T 1527
T 1536
T 1526
T 1911
T 0366
T 0365
T 1044
T 0146
T 0080
T 1992
T 1924
T 0472
T 0487
T 1503
T 1523
T 1529
T 1837
T 1125
T 1708
T 0123
T 1560
T 0820
T 1447
T 1713
T 0121
T 0981
T 1585
T 0418
T 0088
T 0147
T 1741
T 1861
T 1572
T 1120
T 0622
T 1984
T 0808
T 0664
T 0573
T 1068
T 1805
T 0457
T 0548
T 0111
T 0605
T 1000
T 0327
T 0331
T 1532
T 1538
T 0591
T 0456
T 1720
T 2016
T 1238
T 0710
T 0643
T 1703
T 1045
T 1282
T 0890
T 1088
T 0931
T 0722
T 0690
T 1445
T 0498
T 0519
T 0514
T 0517
T 1275
T 0075
T 0705
T 0117
T 1776
T 1761
T 0737
T 0162
T 0719
T 0960
T 0691
T 1247
T 0099
T 1464
T 0713
T 1225
T 0901
T 0799
T 0241
T 1966
T 0380
T 0238
T 0173
T 0656
T 0081
T 0434
T 0576
T 0578
T 1864
T 1292
T 1066
T 0597
T 1074
T 1032
T 1809
T 1700
T 0798
T 1268
T 0790
T 1752
T 0225
T 1468
T 1255
T 1198
T 1556
T 1537
T 0984
T 1659
T 0202
T 1443
T 0888
T 1333
T 0757
T 1652
T 1243
T 1777
T 0833
T 0218
T 1849
T 0991
T 0485
T 1015
T 0364
T 1956
T 1381
T 0103

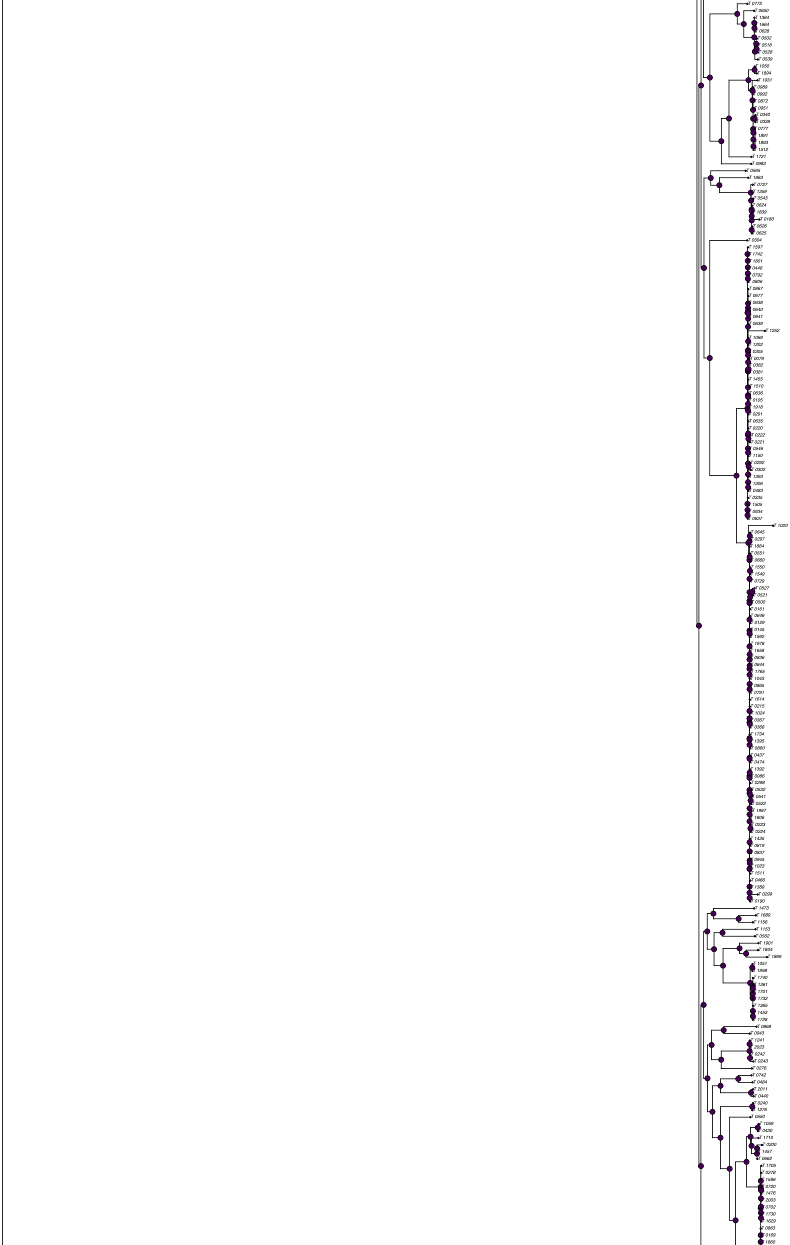
T 0772
T 0650
T 1364
1664
0628
T 0502
0518
T 0528
T 0539
T 1050
T 1894
T 1931
T 0989
0992
T 0872
T 0951
T 0340
T 0339
0777
1891
1893
T 1512
T 1721
T 0983
T 0595
T 1863
T 0727
T 1359
T 0543
T 0624
1839
T 0180
T 0626
T 0625
T 0304
T 1597
1742
1801
0446
0792
T 0806
T 0867
T 0677
0638
0640
0641
T 0639
T 1252
T 1069
T 1202
0305
T 0079
0382
0381
T 1455
1510
T 0636
0105
1918
T 0291
T 0635
T 0220
T 0222
T 0221
0546
T 1150
T 0292
T 0302
1393
1306
T 0483
T 0335
1505
0934
T 0637
T 1020
T 0645
0297
T 1884
T 0551
0660
T 1550
1549
0729
T 0527
T 0521
0500
T 0161
0646
0129
T 0145
T 1592
T 1978
T 1658
0836
0644
T 1765
T 1043
0865
T 0791
T 1614
T 0215
1024
0367
T 0368
T 1734
1395
T 0860
0437
T 0474
T 1392
0086
T 0298
T 0532
T 0541
T 0522
T 1987
1806
T 0223
T 0224
T 1435
T 0819
T 0837
0945
1023
T 1511
T 0466
1389
T 0299
T 0190
T 1473
T 1689
T 1156
T 1153
T 0562
T 1901
T 1804
T 1869
T 1051
T 1998
T 1740
1361
1701
1732
T 1365
1453
T 1728
T 0868
T 0943
T 1241
2023
0242
T 0243
T 0276
T 0742
T 0484
T 2011
T 0440
T 0240
T 1276
T 0550
T 1059
0430
T 1710
T 0200
1457
T 0662
T 1705
T 0278
1586
0720
T 1476
2003
0702
1730
T 1629
T 0863
0169
1660

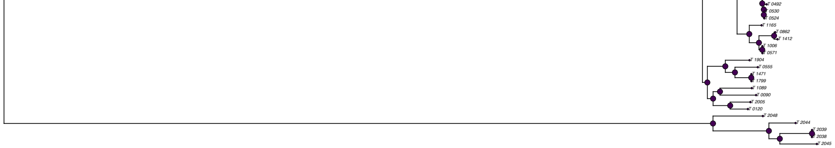
T 0492
T 0530
T 0524
T 1165
T 0862
T 1412
T 1006
T 0571
T 1904
T 0555
T 1471
T 1799
T 1089
T 0090
T 2005
T 0120
T 2048
T 2044
T 2039
T 2038
T 2045

Effector gene: tse3

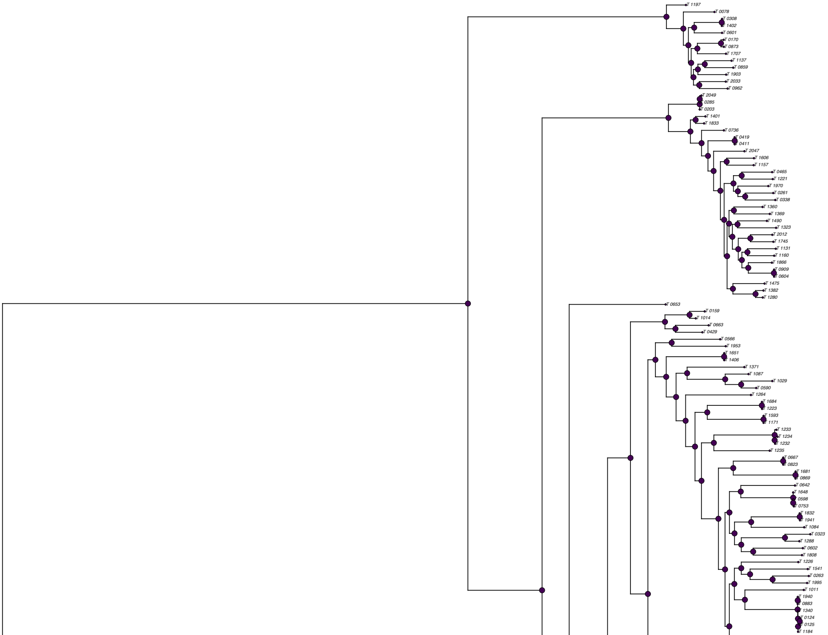

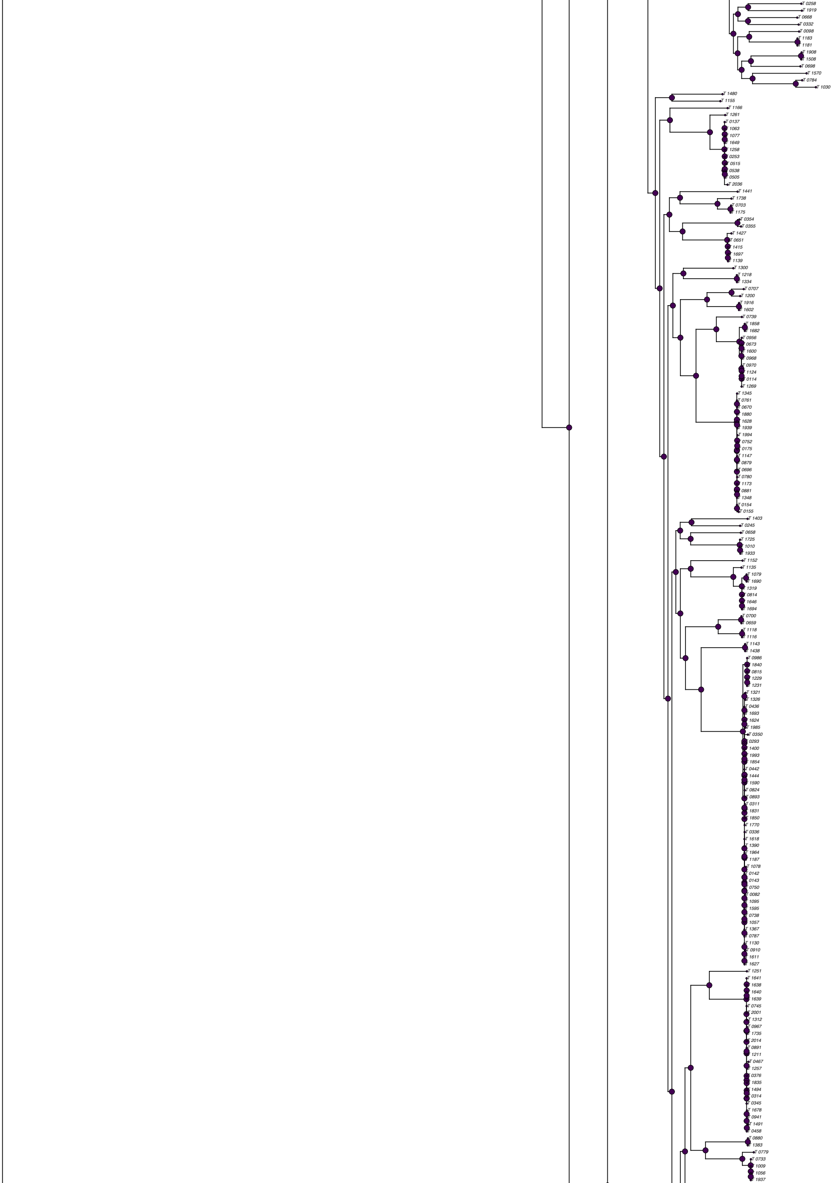

T 0258
T 1919
T 0668
T 0332
T 0098
T 1183
T 1181
T 1908
T 1508
T 0698
T 1570
T 0784
T 1030
T 1480
T 1155
T 1166
T 1261
T 0137
1063
1077
T 1649
1258
0253
0515
0538
0505
T 2036
T 1441
T 1738
T 0703
T 1175
T 0354
T 0355
T 1427
T 0651
1415
1697
T 1139
T 1300
T 1218
T 1334
T 0707
T 1200
T 1916
T 1602
T 0739
T 1858
T 1682
T 0956
0673
T 1600
T 0968
T 0970
T 1124
T 0114
T 1269
T 1345
T 0761
0670
1880
1628
T 1939
T 1994
0752
0175
T 1147
T 0879
T 0696
T 0780
1173
0881
T 1348
T 0154
T 0155
T 1403
T 0245
T 0658
T 1725
1010
T 1933
T 1152
T 1135
T 1079
T 1690
1319
0814
1646
T 1694
T 0700
T 0659
T 1118
T 1116
T 1143
T 1438
T 0986
1840
0815
1229
T 1231
T 1321
T 1326
T 0436
1693
1624
T 1985
T 0350
0293
1400
1993
1854
T 0442
1444
1590
T 0824
0893
T 0311
1831
1850
T 1770
T 0336
T 1618
T 1390
1964
1187
T 1078
T 0142
0143
0750
T 0082
1095
1595
T 0738
1057
T 1367
T 0787
T 1130
T 0910
1611
T 1627
T 1251
T 1641
1638
1640
1639
T 0745
2001
T 1312
T 0967
T 1735
2014
T 0891
1211
T 0467
1257
0376
1835
1494
0314
T 0345
T 1678
0941
T 1491
T 0458
T 0880
T 1383
T 0779
T 0733
1009
1056
T 1937

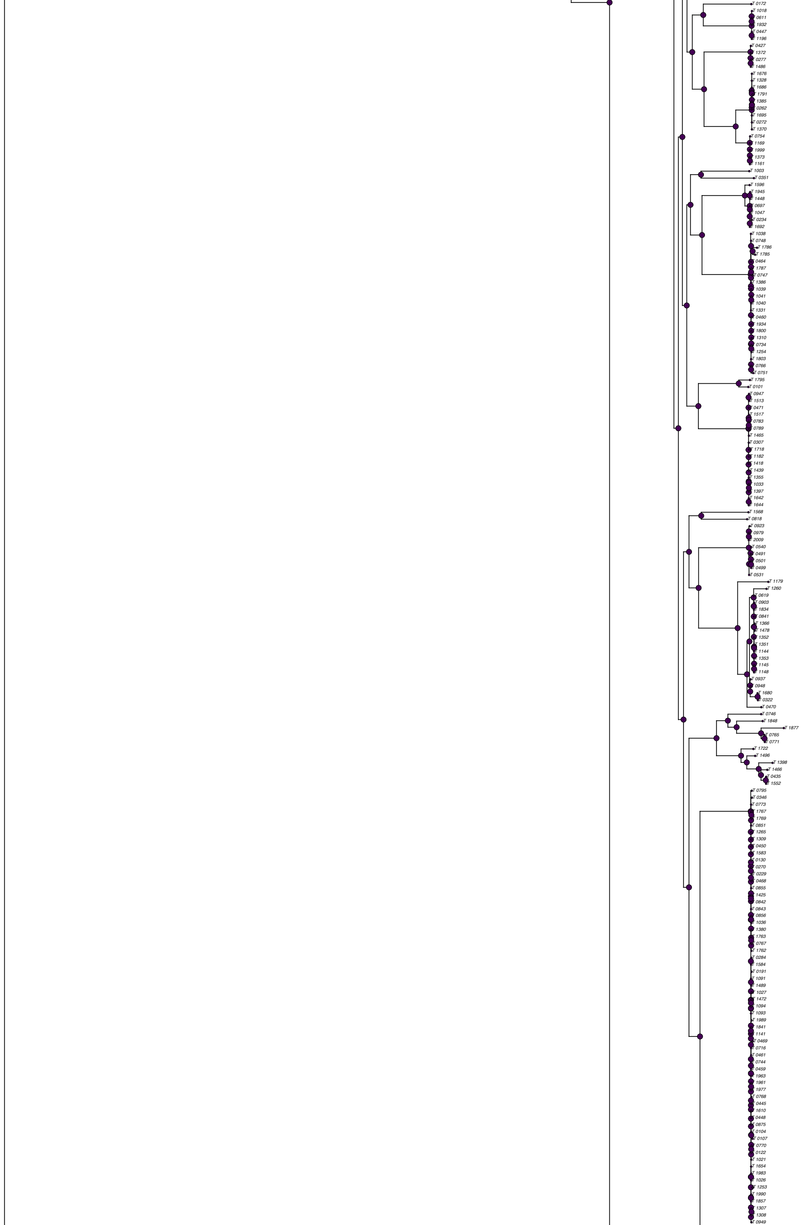

T 0172
T 1018
0611
1932
T 0447
T 1196
T 0427
1372
T 0277
T 1486
T 1676
T 1328
T 1686
1791
1385
T 0262
T 1695
T 0272
T 1370
T 0754
1169
1999
1373
T 1161
T 1003
T 0351
T 1596
T 1945
T 1448
0697
T 1047
T 0234
T 1692
T 1038
T 0748
T 1786
T 1785
0464
1787
T 0747
T 1386
1039
1041
1040
T 1331
0460
1934
1800
1310
0734
T 1254
T 1803
0766
T 0751
T 1795
T 0101
T 0947
T 1513
0471
T 1517
0783
0789
T 1465
T 0307
T 1718
T 1182
1418
T 1439
T 1355
1033
1397
T 1642
T 1644
T 1568
T 0818
T 0923
0979
T 2009
T 0540
0491
0501
T 0499
T 0531
T 1179
T 1260
0619
T 0903
T 1834
0841
T 1366
T 1478
1352
T 1351
1144
1353
1145
T 1148
T 0937
0948
T 1680
T 0322
T 0470
T 0746
T 1848
T 1877
T 0765
T 0771
T 1722
T 1496
T 1398
T 1466
T 0435
T 1552
T 0795
T 0346
T 0773
1767
1769
T 0851
1265
1309
0450
T 1583
T 0130
0270
T 0229
T 0468
T 0855
1425
0842
T 0843
0856
T 1036
1380
T 1763
0767
T 1762
T 0284
1584
T 0191
T 1091
T 1489
1027
T 1472
T 1094
T 1093
T 1989
1841
1141
T 0469
T 0716
T 0461
0744
T 0459
1963
1961
1977
T 0768
0445
1610
0448
T 0875
T 0104
T 0107
0770
0122
T 1021
T 1654
T 1983
1026
T 1253
T 1990
T 1857
T 1307
1308
T 0949

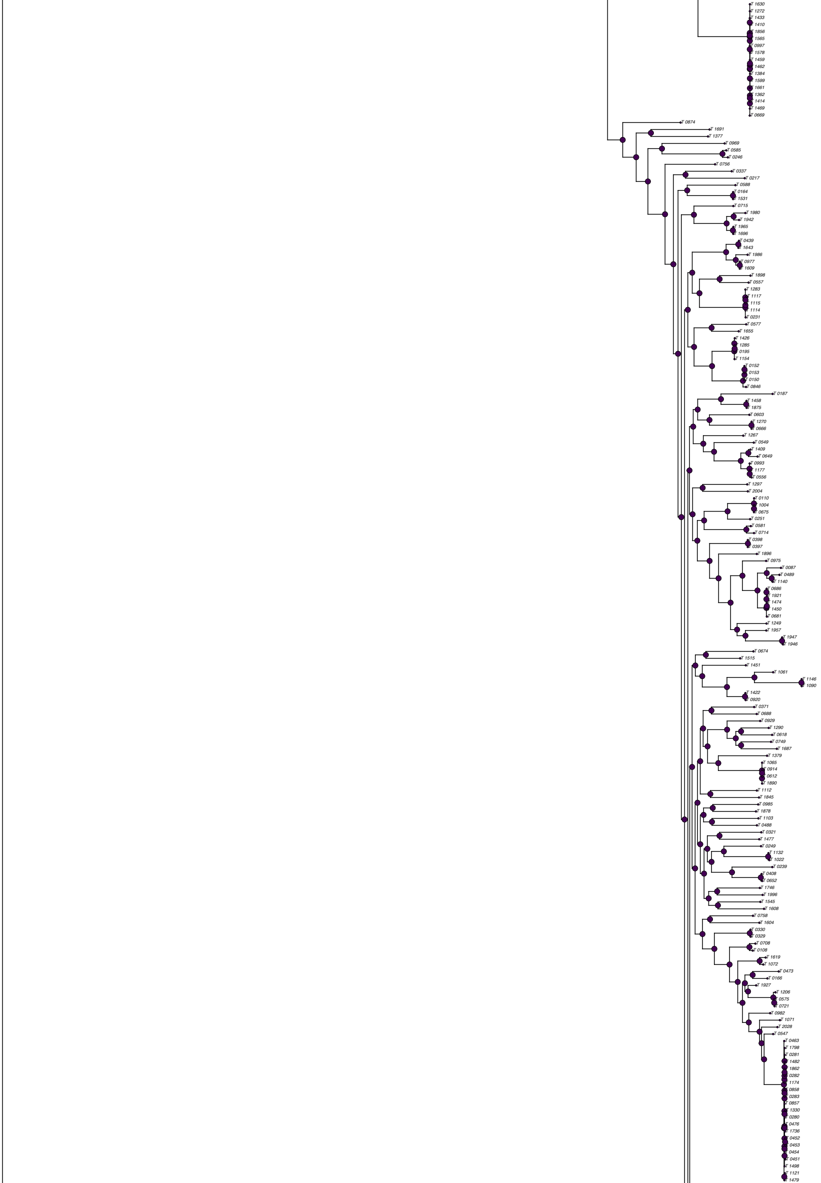
T 1630
T 1272
T 1433
T 1410
T 1856
T 1565
T 0997
T 1578
T 1459
T 1462
T 1384
T 1599
T 1661
T 1362
T 1414
T 1469
T 0669
T 0874
T 1691
T 1377
T 0969
T 0585
T 0246
T 0756
T 0337
T 0217
T 0588
T 0164
T 1531
T 0715
T 1980
T 1942
T 1965
T 1696
T 0439
T 1643
T 1986
T 0977
T 1609
T 1898
T 0557
T 1283
T 1117
T 1115
T 1114
T 0231
T 0577
T 1655
T 1426
T 1285
T 0195
T 1154
T 0152
T 0153
T 0150
T 0846
T 0187
T 1458
T 1875
T 0603
T 1270
T 0666
T 1267
T 0549
T 1409
T 0649
T 0993
T 1177
T 0556
T 1297
T 2004
T 0110
T 1004
T 0675
T 0251
T 0581
T 0714
T 0398
T 0397
T 1896
T 0975
T 0087
T 0489
T 1140
T 0686
T 1921
T 1474
T 1450
T 0681
T 1249
T 1957
T 1947
T 1946
T 0674
T 1515
T 1451
T 1061
T 1146
T 1090
T 1422
T 0920
T 0371
T 0688
T 0929
T 1290
T 0618
T 0749
T 1687
T 1379
T 1065
T 0914
T 0612
T 1890
T 1112
T 1845
T 0985
T 1878
T 1103
T 0488
T 0321
T 1477
T 0249
T 1132
T 1022
T 0239
T 0408
T 0652
T 1746
T 1996
T 1545
T 1608
T 0758
T 1604
T 0330
T 0329
T 0708
T 0108
T 1619
T 1072
T 0473
T 0166
T 1927
T 1206
T 0575
T 0721
T 0982
T 1071
T 2028
T 0547
T 0463
T 1798
T 0281
T 1482
T 1862
T 0282
T 1174
T 0858
T 0283
T 0857
T 1330
T 0280
T 0476
T 1736
T 0452
T 0453
T 0454
T 0451
T 1498
T 1121
T 1479

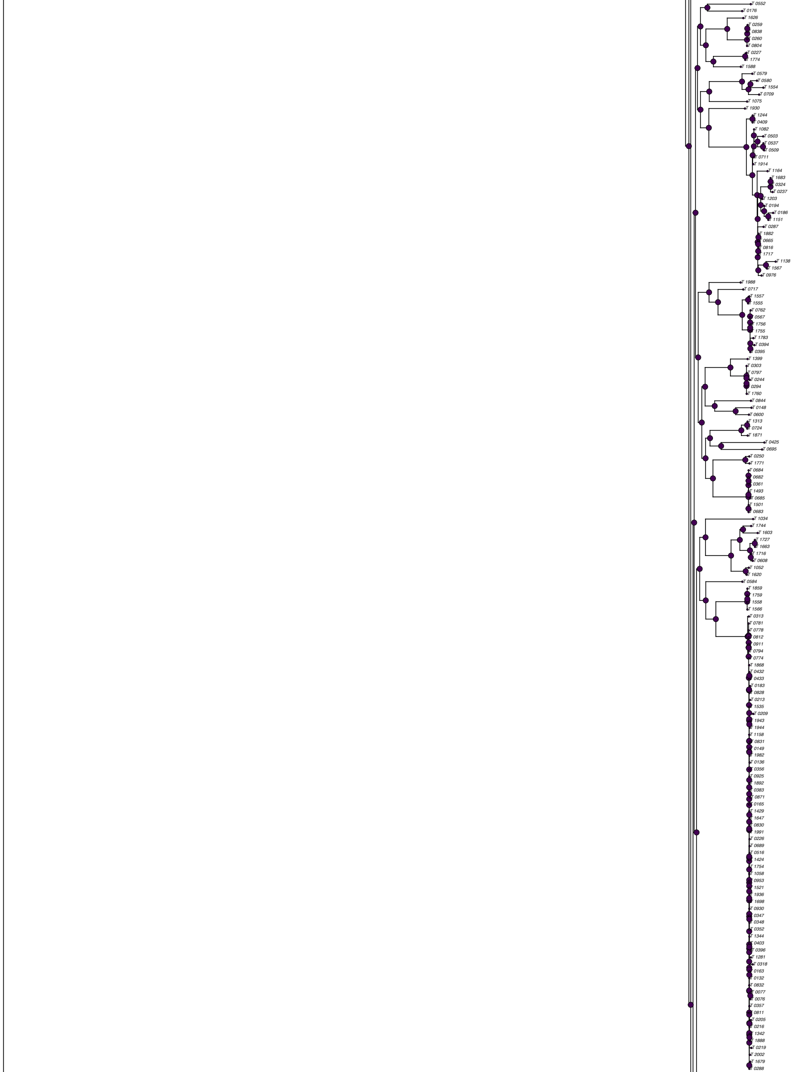

T 0552
T 0176
T 1626
T 0259
0838
0260
0804
T 0227
1774
T 1588
T 0579
T 0580
T 1554
T 0709
T 1075
T 1930
T 1244
0409
T 1082
T 0503
T 0537
T 0509
T 0711
T 1914
T 1164
T 1683
0324
T 0237
T 1203
T 0194
T 0186
T 1151
T 0287
T 1882
0665
0816
1717
T 1138
T 1567
T 0976
T 1988
T 0717
T 1557
1555
T 0762
0567
T 1756
1755
T 1783
T 0394
0395
T 1399
T 0303
0797
T 0244
0294
T 1760
T 0844
T 0148
T 0600
T 1313
0724
T 1871
T 0425
T 0695
T 0250
T 1771
T 0684
0682
0361
T 1493
T 0685
T 1501
0683
T 1034
T 1744
T 1603
T 1727
T 1663
T 1716
T 0608
T 1052
T 1620
T 0584
T 1859
1759
1558
T 1566
T 0313
T 0781
T 0778
0812
0911
T 0794
0774
T 1868
T 0432
0433
T 0183
0828
T 0213
1535
T 0209
1943
1944
T 1158
0831
0149
T 1982
T 0136
0356
T 0925
T 1892
0383
T 0871
0165
T 1429
T 1647
T 0830
1991
T 0226
T 0689
T 0516
1424
T 1754
T 1058
0953
1521
T 1936
1698
T 0930
0347
T 0348
T 0352
T 1344
0403
T 0396
T 1281
T 0318
0163
T 0132
T 0832
T 0077
T 0076
T 0357
0811
T 0205
T 0216
1342
T 1888
T 0219
T 2002
T 1679
0288

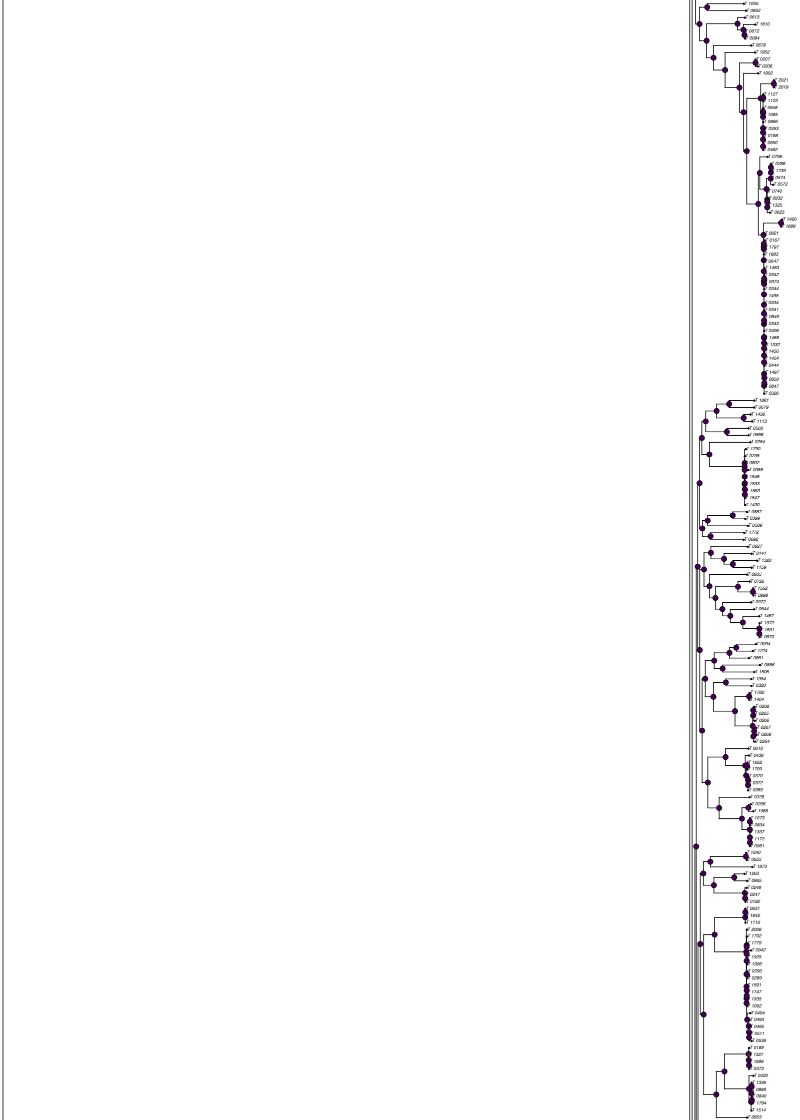
T 1055
T 0852
T 0613
T 1810
T 0672
T 0084
T 0678
T 1952
T 0207
T 0208
T 1902
T 2021
T 2019
T 1127
T 1123
T 0648
T 1085
T 0866
T 0353
T 0188
T 0950
T 0462
T 0796
T 0286
T 1739
T 0574
T 0572
T 0740
T 0932
T 1325
T 0623
T 1460
T 1899
T 0821
T 0167
T 1797
T 1883
T 0647
T 1483
T 0342
T 0374
T 0344
T 1495
T 0334
T 0341
T 0849
T 0343
T 0406
T 1488
T 1332
T 1456
T 1454
T 0444
T 1497
T 0850
T 0847
T 0326
T 1881
T 0679
T 1436
T 1113
T 0560
T 0586
T 0254
T 1790
T 0235
T 0802
T 0358
T 1546
T 1533
T 1553
T 1547
T 1430
T 0887
T 0399
T 0589
T 1772
T 0692
T 0827
T 0141
T 1320
T 1159
T 0935
T 0726
T 1562
T 0998
T 0972
T 0544
T 1467
T 1972
T 1631
T 0870
T 0094
T 1224
T 0861
T 0886
T 1506
T 1954
T 0320
T 1780
T 1404
T 0268
T 0265
T 0266
T 0267
T 0269
T 0264
T 0610
T 0438
T 1662
T 1709
T 0370
T 0372
T 0369
T 0228
T 0206
T 1968
T 1073
T 0834
T 1337
T 1172
T 0961
T 1240
T 0655
T 1873
T 1263
T 0965
T 0248
T 0247
T 0182
T 0631
T 1842
T 1110
T 2008
T 1792
T 1779
T 0942
T 1925
T 1906
T 0290
T 0289
T 1591
T 1747
T 1935
T 1092
T 0494
T 0493
T 0495
T 0511
T 0536
T 0189
T 1327
T 1699
T 0373
T 0420
T 1336
T 0889
T 0840
T 1794
T 1514
T 0853

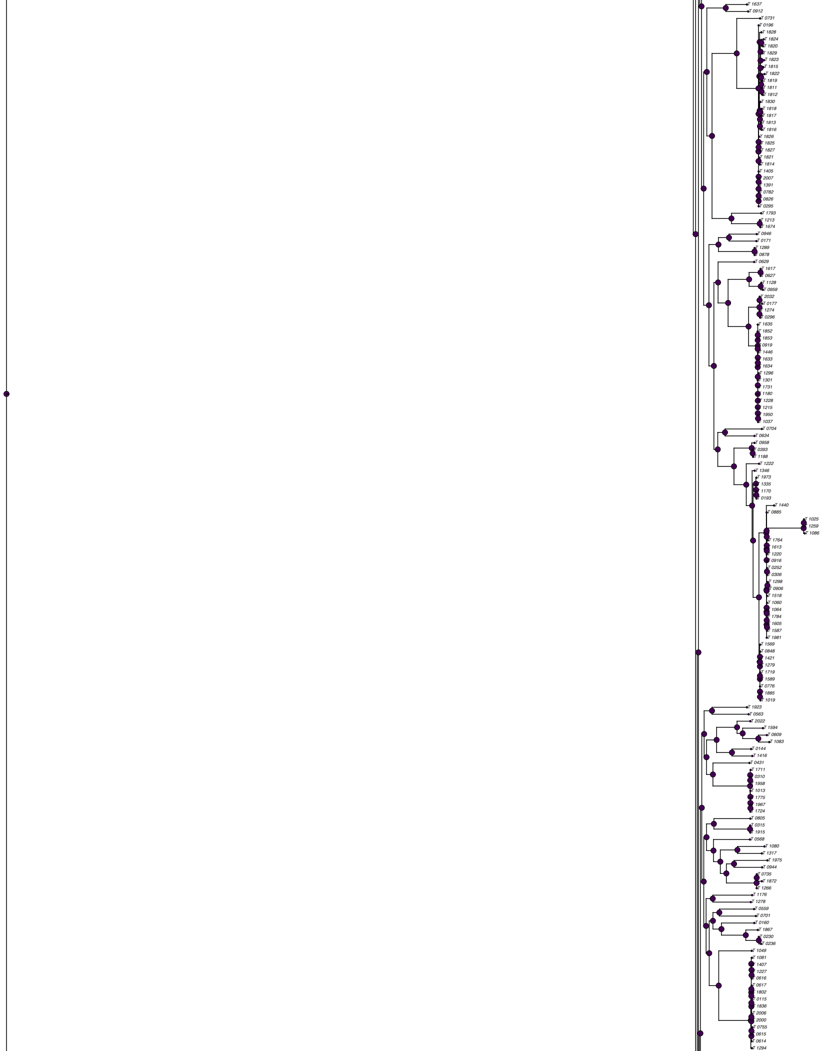
T 1637
T 0912
T 0731
T 0196
T 1828
T 1824
T 1820
T 1829
T 1823
T 1815
T 1822
T 1819
T 1811
T 1812
T 1830
T 1818
T 1817
T 1813
T 1816
T 1826
T 1825
T 1827
T 1821
T 1814
T 1405
2007
1391
0782
0826
T 0295
T 1793
T 1213
T 1674
T 0946
T 0171
T 1289
T 0878
T 0629
T 1617
T 0627
T 1128
T 0959
T 2032
T 0177
T 1274
T 0296
T 1635
T 1852
1853
0919
T 1446
1633
1634
T 1296
T 1301
1731
1180
1228
1215
1950
T 1037
T 0704
T 0634
T 0958
T 0393
T 1188
T 1222
T 1346
T 1973
1335
1170
T 0193
T 1440
T 0885
T 1025
1259
T 1086
T 1764
1613
T 1220
0916
T 0252
T 0306
T 1298
T 0906
T 1518
T 1060
1064
T 1784
1605
T 1587
T 1981
T 1569
T 0848
1421
1279
T 1719
1589
T 0776
1885
T 1019
T 1923
T 0563
T 2022
T 1594
T 0609
T 1083
T 0144
T 1416
T 0431
T 1711
0310
1958
T 1013
1775
1967
1724
T 0805
T 0315
T 1915
T 0568
T 1080
T 1317
T 1975
T 0944
T 0735
T 1872
T 1266
T 1176
T 1278
T 0559
T 0701
T 0160
T 1867
T 0230
T 0236
T 1049
T 1081
1407
1227
0616
T 0617
1802
T 0115
1836
T 2006
2000
T 0755
0615
T 0614
T 1294

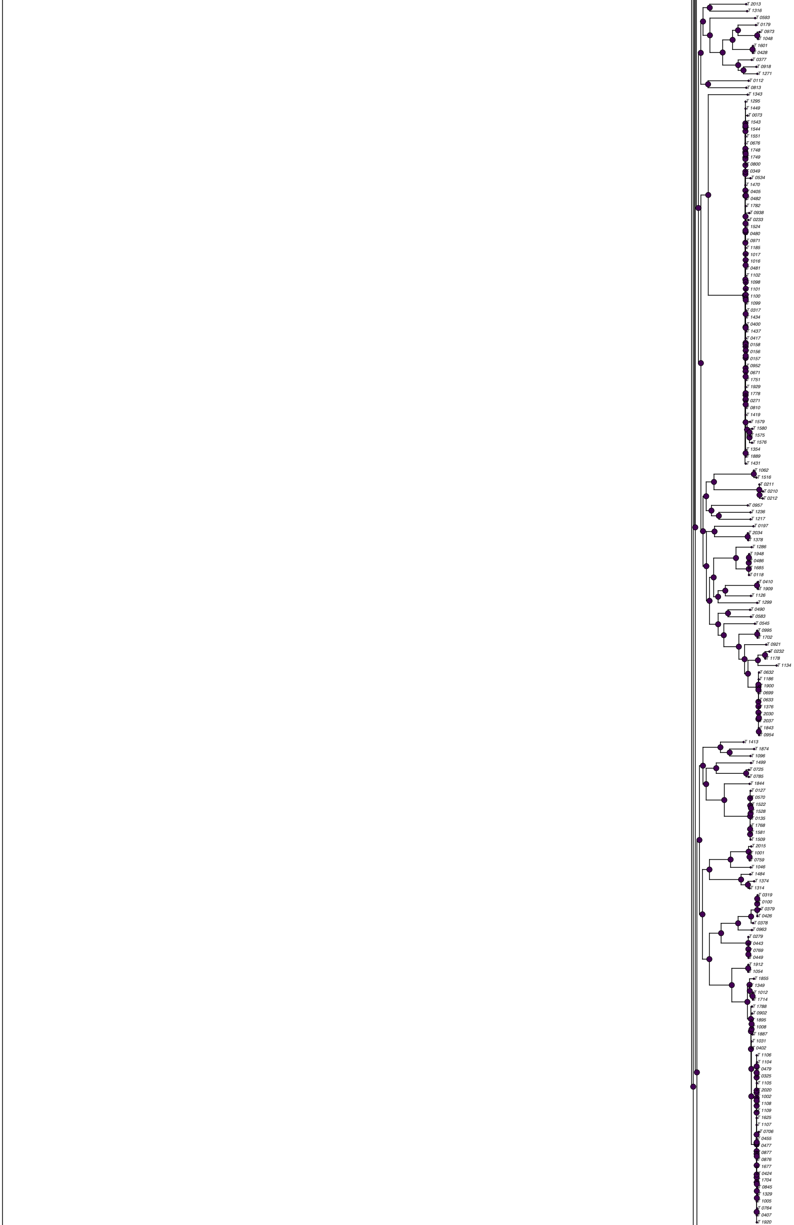
T 2013
T 1316
T 0593
T 0179
T 0973
T 1048
T 1601
T 0428
T 0377
T 0918
T 1271
T 0112
T 0813
T 1343
T 1295
T 1449
T 0073
T 1543
T 1544
T 1551
T 0676
T 1748
T 1749
T 0800
T 0349
T 0534
T 1470
T 0405
T 0482
T 1782
T 0938
T 0233
T 1524
T 0480
T 0971
T 1185
T 1017
T 1016
T 0481
T 1102
T 1098
T 1101
T 1100
T 1099
T 0317
T 1434
T 0400
T 1437
T 0417
T 0158
T 0156
T 0157
T 0952
T 0671
T 1751
T 1929
T 1778
T 0271
T 0810
T 1419
T 1579
T 1580
T 1575
T 1576
T 1354
T 1889
T 1431
T 1062
T 1516
T 0211
T 0210
T 0212
T 0957
T 1236
T 1217
T 0197
T 2034
T 1378
T 1286
T 1948
T 0486
T 1685
T 0118
T 0410
T 1909
T 1126
T 1299
T 0490
T 0583
T 0545
T 0995
T 1702
T 0921
T 0232
T 1178
T 1134
T 0632
T 1186
T 1900
T 0699
T 0633
T 1376
T 2030
T 2037
T 1843
T 0954
T 1413
T 1874
T 1096
T 1499
T 0725
T 0785
T 1844
T 0127
T 0570
T 1522
T 1528
T 0135
T 1768
T 1581
T 1509
T 2015
T 1001
T 0759
T 1046
T 1484
T 1374
T 1314
T 0319
T 0100
T 0379
T 0426
T 0378
T 0963
T 0279
T 0443
T 0769
T 0449
T 1912
T 1054
T 1855
T 1349
T 1012
T 1714
T 1788
T 0902
T 1895
T 1008
T 1887
T 1031
T 0402
T 1106
T 1104
T 0479
T 0325
T 1105
T 2020
T 1002
T 1108
T 1109
T 1625
T 1107
T 0706
T 0455
T 0477
T 0877
T 0876
T 1677
T 0424
T 1704
T 0845
T 1329
T 1005
T 0764
T 0407
T 1920

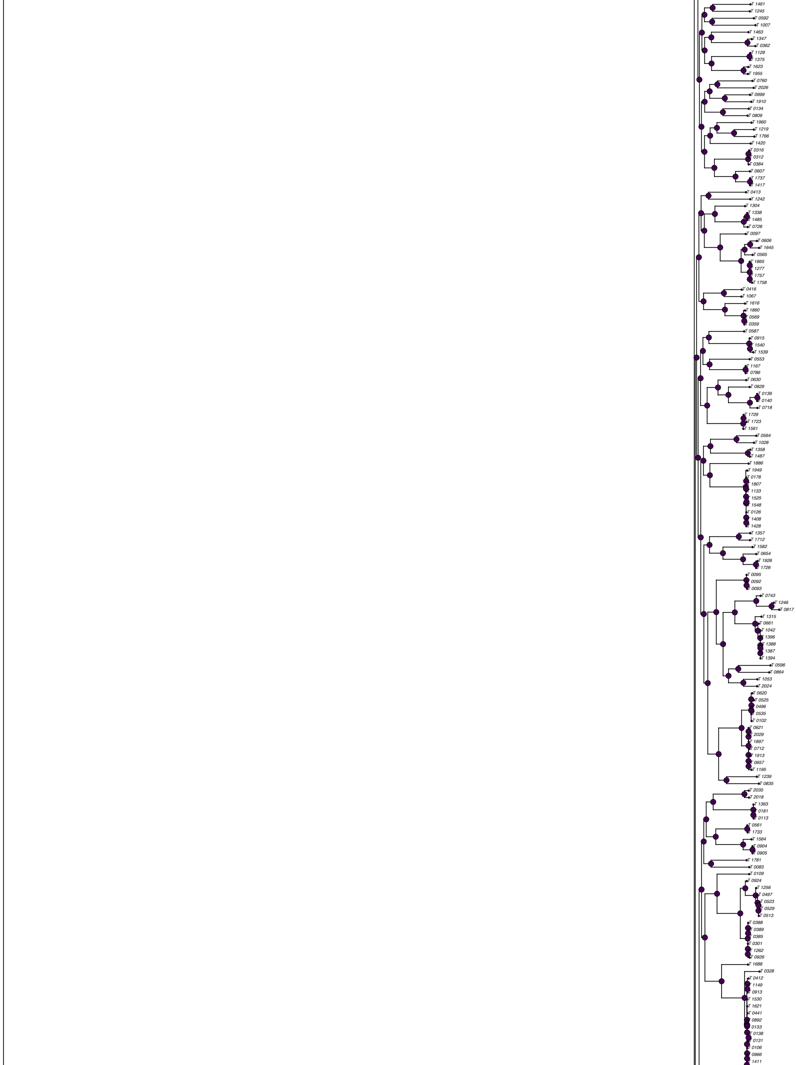
T 1461
T 1245
T 0592
T 1007
T 1463
T 1347
T 0362
T 1129
T 1375
T 1623
T 1955
T 0760
T 2026
T 0999
T 1910
T 0134
T 0809
T 1960
T 1219
T 1766
T 1420
T 0316
T 0312
T 0384
T 0607
T 1737
T 1417
T 0413
T 1242
T 1304
T 1338
T 1485
T 0728
T 0097
T 0606
T 1645
T 0565
T 1865
T 1277
T 1757
T 1758
T 0416
T 1067
T 1616
T 1860
T 0569
T 0359
T 0587
T 0915
T 1540
T 1539
T 0553
T 1167
T 0786
T 0630
T 0829
T 0139
T 0140
T 0718
T 1729
T 1723
T 1561
T 0564
T 1028
T 1358
T 1487
T 1886
T 1949
T 0178
T 1807
T 1133
T 1525
T 1548
T 0126
T 1408
T 1428
T 1357
T 1712
T 1582
T 0654
T 1926
T 1726
T 0095
T 0092
T 0093
T 0743
T 1246
T 0817
T 1315
T 0661
T 1042
T 1396
T 1388
T 1387
T 1394
T 0596
T 0864
T 1053
T 2024
T 0620
T 0525
T 0496
T 0535
T 0102
T 0621
T 2029
T 1897
T 0712
T 1913
T 0657
T 1195
T 1239
T 0835
T 2035
T 2018
T 1363
T 0181
T 0113
T 0561
T 1733
T 1564
T 0904
T 0905
T 1781
T 0083
T 0109
T 0924
T 1256
T 0497
T 0523
T 0529
T 0513
T 0388
T 0389
T 0385
T 0301
T 1262
T 0926
T 1688
T 0328
T 0412
T 1149
T 0913
T 1530
T 1621
T 0441
T 0892
T 0133
T 0138
T 0131
T 0106
T 0966
T 1411

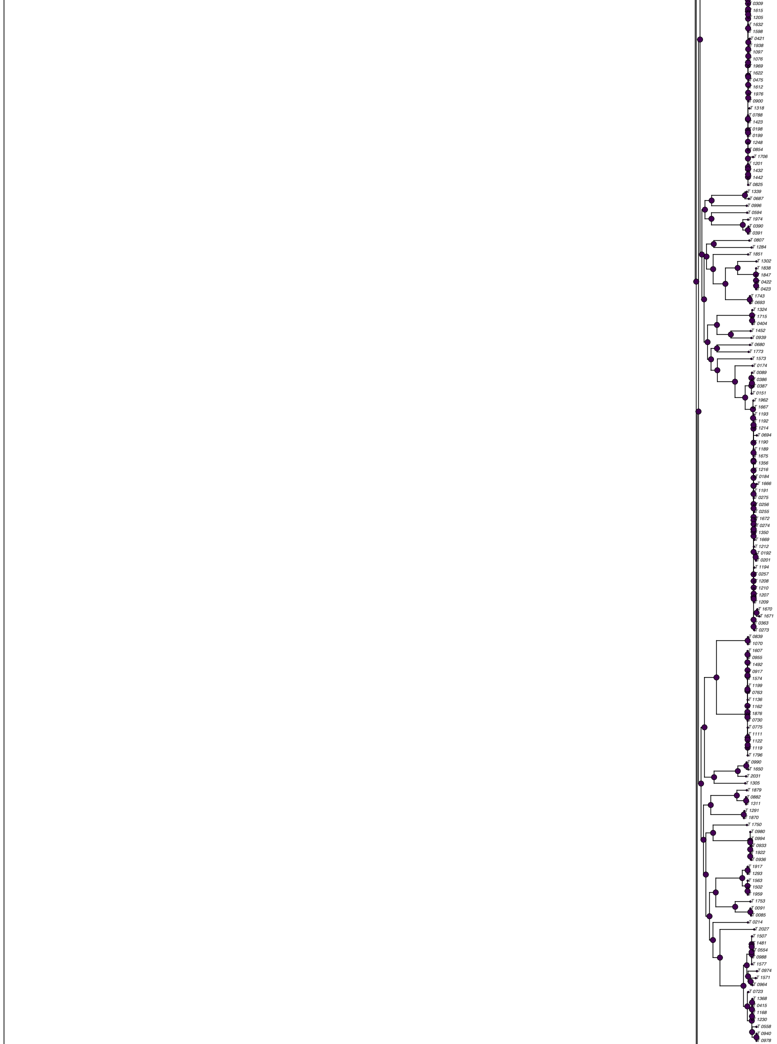
0309
1615
1205
T 1632
T 1598
T 0421
T 1938
1097
T 1076
1969
T 1622
T 0475
T 1612
1976
T 0900
T 1318
T 0788
T 1423
0198
T 0199
1248
T 0854
T 1706
T 1201
1432
1442
T 0825
T 1339
T 0687
T 0996
T 0594
T 1974
T 0390
T 0391
T 0807
T 1284
T 1851
T 1302
T 1838
1847
T 0422
T 0423
T 1743
T 0693
T 1324
1715
T 0404
T 1452
T 0939
T 0680
T 1773
T 1573
T 0174
T 0089
0386
0387
T 0151
T 1962
T 1667
T 1193
T 1192
1214
T 0694
1190
T 1189
T 1675
1356
1216
T 0184
T 1666
T 1191
T 0275
T 0256
T 0255
T 1672
T 0274
1350
T 1669
T 1212
T 0192
T 0201
T 1194
T 0257
T 1208
1210
T 1207
T 1209
T 1670
T 1671
T 0363
T 0273
T 0839
T 1070
T 1607
T 0955
T 1492
0917
T 1574
T 1199
0763
T 1136
1162
1876
T 0730
T 0775
T 1111
1122
1119
T 1796
T 0990
T 1650
T 2031
T 1305
T 1879
T 0882
T 1311
T 1291
T 1870
T 1750
T 0980
T 0994
T 0933
T 1922
T 0936
T 1917
T 1293
T 1563
1502
T 1959
T 1753
T 0091
T 0085
T 0214
T 2027
T 1507
1481
T 0554
T 0988
T 1577
T 0974
T 1571
T 0964
T 0723
T 1368
T 0415
1168
1230
T 0558
T 0940
T 0978

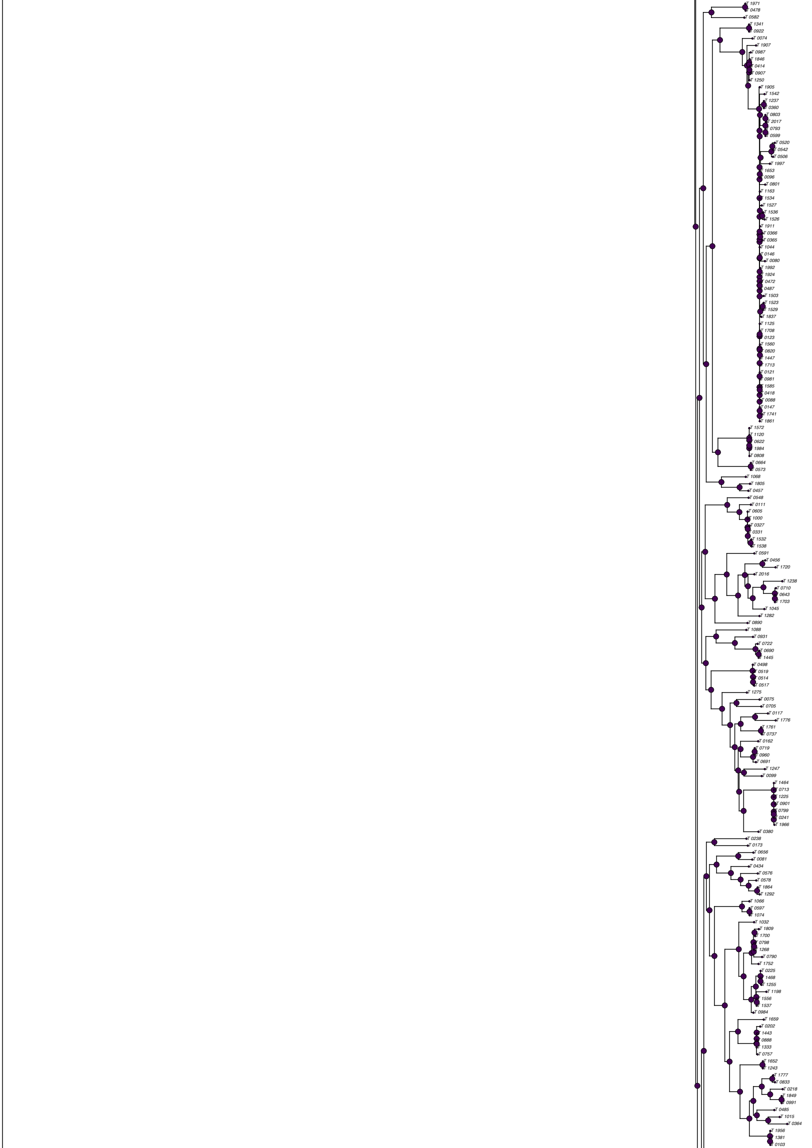
T 1971
T 0478
T 0582
T 1341
T 0922
T 0074
T 1907
T 0987
T 1846
T 0414
T 0907
T 1250
T 1905
T 1542
T 1237
T 0360
T 0803
T 2017
T 0793
T 0599
T 0520
T 0542
T 0506
T 1997
1653
0096
T 0801
T 1163
1534
T 1527
T 1536
T 1526
T 1911
T 0366
T 0365
T 1044
T 0146
T 0080
T 1992
1924
0472
0487
T 1503
T 1523
T 1529
T 1837
T 1125
T 1708
0123
T 1560
0820
1447
1713
T 0121
0981
1585
0418
T 0088
T 0147
T 1741
T 1861
T 1572
T 1120
0622
1984
T 0808
T 0664
T 0573
T 1068
T 1805
T 0457
T 0548
T 0111
T 0605
1000
T 0327
0331
T 1532
T 1538
T 0591
T 0456
T 1720
T 2016
T 1238
T 0710
0643
T 1703
T 1045
T 1282
T 0890
T 1088
T 0931
T 0722
T 0690
T 1445
T 0498
0519
0514
T 0517
T 1275
T 0075
T 0705
T 0117
T 1776
T 1761
T 0737
T 0162
T 0719
0960
T 0691
T 1247
T 0099
T 1464
0713
1225
T 0901
0799
0241
T 1966
T 0380
T 0238
T 0173
T 0656
T 0081
T 0434
T 0576
T 0578
T 1864
T 1292
T 1066
T 0597
T 1074
T 1032
T 1809
T 1700
0798
1268
T 0790
T 1752
T 0225
1468
T 1255
T 1198
1556
T 1537
T 0984
T 1659
T 0202
1443
0888
1333
T 0757
T 1652
T 1243
T 1777
T 0833
T 0218
T 1849
T 0991
T 0485
T 1015
T 0364
T 1956
1381
T 0103

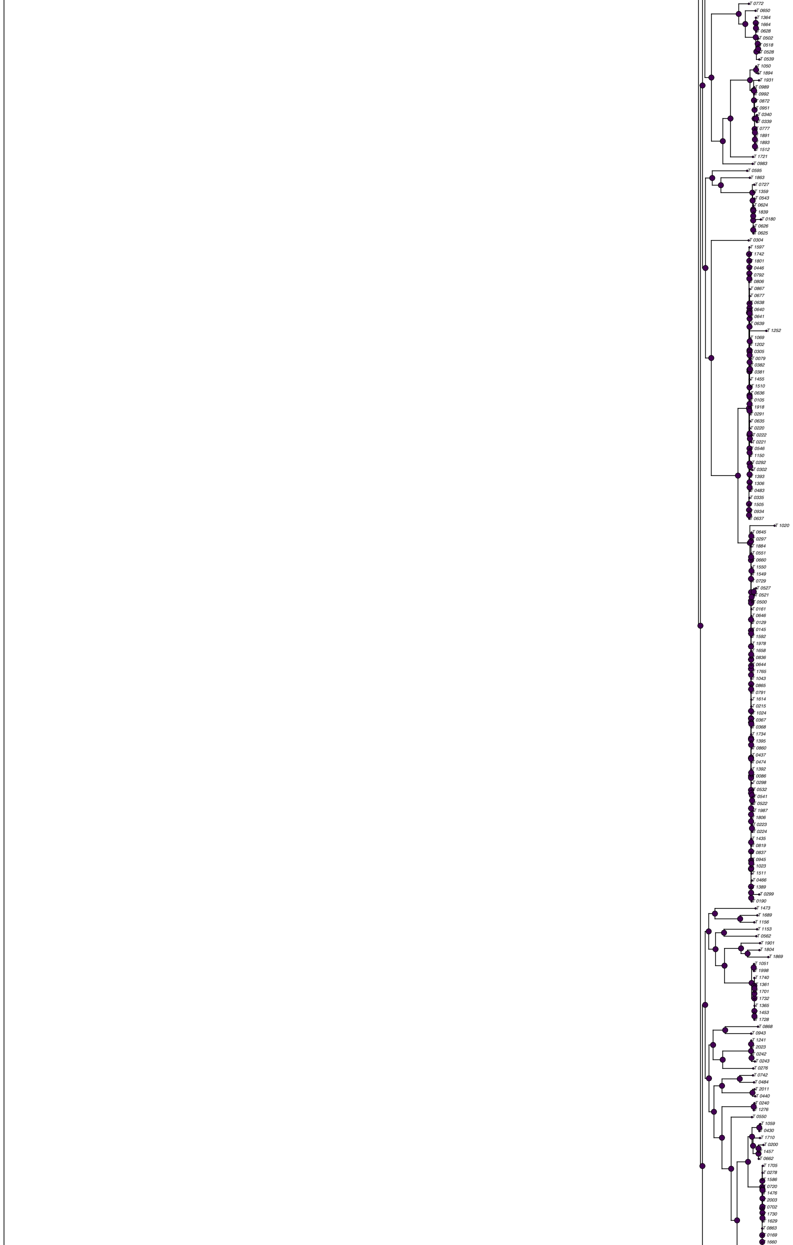
T 0772
T 0650
T 1364
T 1664
T 0628
T 0502
T 0518
T 0528
T 0539
T 1050
T 1894
T 1931
T 0989
T 0992
T 0872
T 0951
T 0340
T 0339
T 0777
T 1891
T 1893
T 1512
T 1721
T 0983
T 0595
T 1863
T 0727
T 1359
T 0543
T 0624
T 1839
T 0180
T 0626
T 0625
T 0304
T 1597
T 1742
T 1801
T 0446
T 0792
T 0806
T 0867
T 0677
T 0638
T 0640
T 0641
T 0639
T 1252
T 1069
T 1202
T 0305
T 0079
T 0382
T 0381
T 1455
T 1510
T 0636
T 0105
T 1918
T 0291
T 0635
T 0220
T 0222
T 0221
T 0546
T 1150
T 0292
T 0302
T 1393
T 1306
T 0483
T 0335
T 1505
T 0934
T 0637
T 1020
T 0645
T 0297
T 1884
T 0551
T 0660
T 1550
T 1549
T 0729
T 0527
T 0521
T 0500
T 0161
T 0646
T 0129
T 0145
T 1592
T 1978
T 1658
T 0836
T 0644
T 1765
T 1043
T 0865
T 0791
T 1614
T 0215
T 1024
T 0367
T 0368
T 1734
T 1395
T 0860
T 0437
T 0474
T 1392
T 0086
T 0298
T 0532
T 0541
T 0522
T 1987
T 1806
T 0223
T 0224
T 1435
T 0819
T 0837
T 0945
T 1023
T 1511
T 0466
T 1389
T 0299
T 0190
T 1473
T 1689
T 1156
T 1153
T 0562
T 1901
T 1804
T 1869
T 1051
T 1998
T 1740
T 1361
T 1701
T 1732
T 1365
T 1453
T 1728
T 0868
T 0943
T 1241
T 2023
T 0242
T 0243
T 0276
T 0742
T 0484
T 2011
T 0440
T 0240
T 1276
T 0550
T 1059
T 0430
T 1710
T 0200
T 1457
T 0662
T 1705
T 0278
T 1586
T 0720
T 1476
T 2003
T 0702
T 1730
T 1629
T 0863
T 0169
T 1660

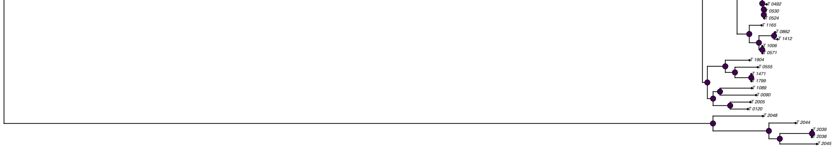
T 0492
T 0530
T 0524
T 1165
T 0862
T 1412
T 1006
T 0571
T 1904
T 0555
T 1471
T 1799
T 1089
T 0090
T 2005
T 0120
T 2048
T 2044
T 2039
T 2038
T 2045

Effector gene: tse4

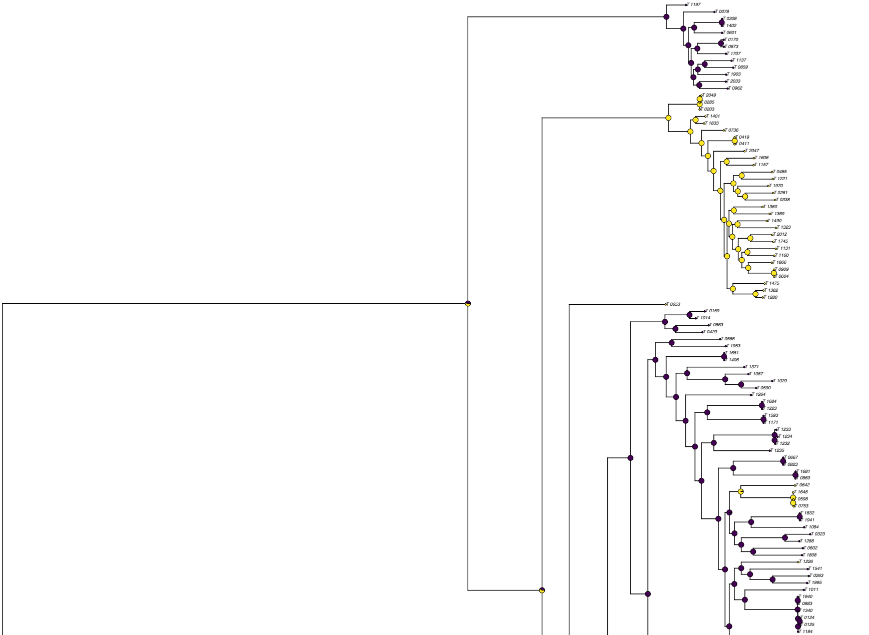

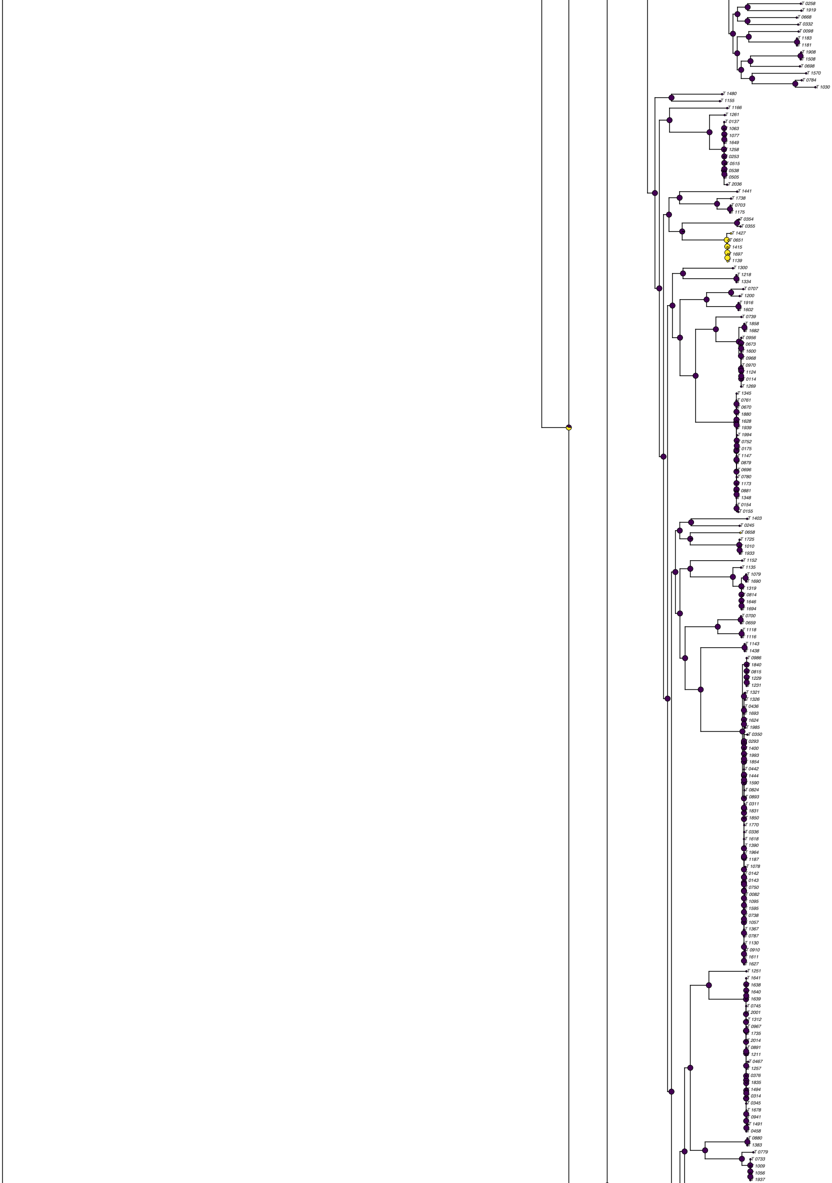
T 0258
T 1919
T 0668
T 0332
T 0098
T 1183
T 1181
T 1908
T 1508
T 0698
T 1570
T 0784
T 1030
T 1480
T 1155
T 1166
T 1261
T 0137
1063
1077
1649
1258
0253
0515
0538
0505
T 2036
T 1441
T 1738
T 0703
T 1175
T 0354
T 0355
T 1427
0651
1415
1697
1139
T 1300
T 1218
T 1334
T 0707
T 1200
T 1916
T 1602
T 0739
T 1858
T 1682
T 0956
0673
1600
0968
0970
1124
0114
T 1269
T 1345
0761
0670
1880
1628
1939
1994
0752
0175
1147
0879
0696
0780
1173
0881
1348
0154
0155
T 1403
T 0245
T 0658
T 1725
1010
1933
T 1152
T 1135
1079
1690
1319
0814
1646
1694
0700
0659
1118
1116
1143
1438
T 0986
1840
0815
1229
1231
1321
1326
0436
1693
1624
1985
T 0350
0293
1400
1993
1854
0442
1444
1590
0824
0893
0311
1831
1850
1770
0336
1618
1390
1964
1187
1078
0142
0143
0750
0082
1095
1595
0738
1057
1367
0787
1130
0910
1611
1627
T 1251
1641
1638
1640
1639
0745
2001
1312
0967
1735
2014
0891
1211
0467
1257
0376
1835
1494
0314
0345
1678
0941
1491
0458
0880
1383
T 0779
T 0733
1009
1056
1937

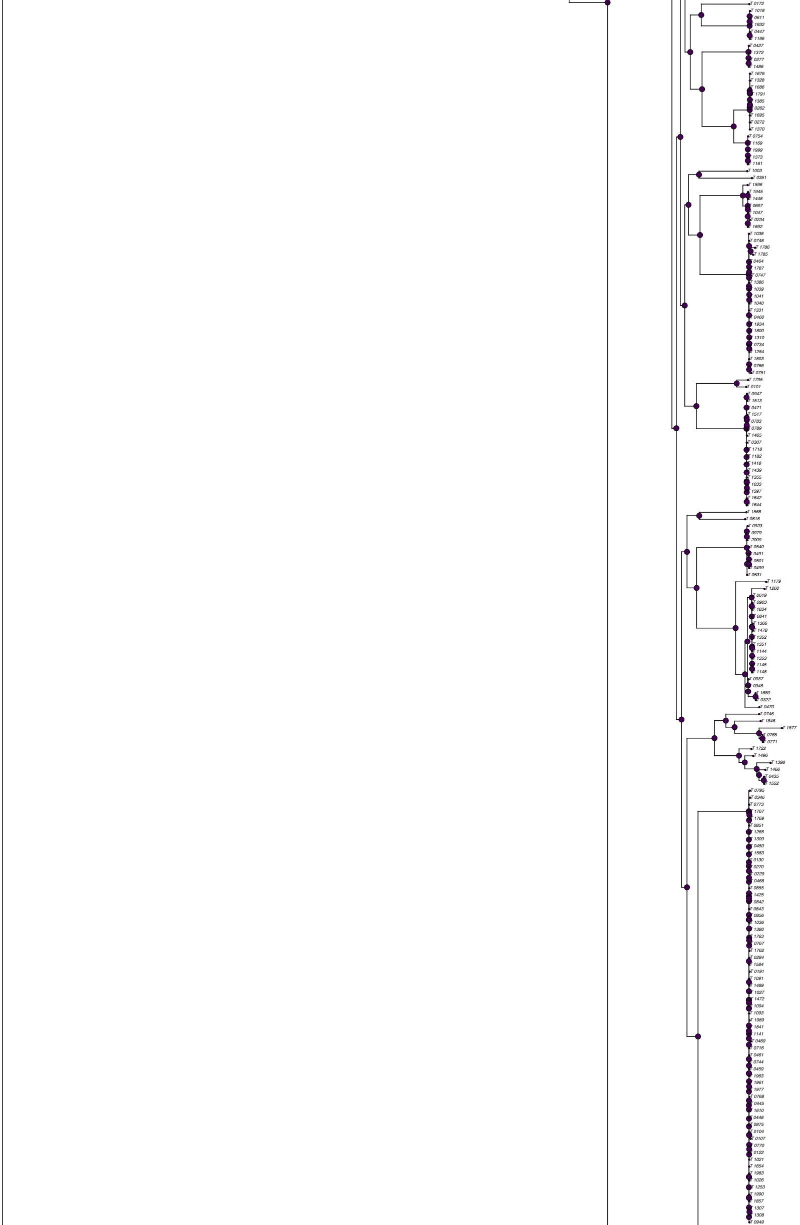
T 0172
T 1018
0611
1932
T 0447
T 1196
T 0427
1372
0277
T 1486
T 1676
T 1328
T 1686
1791
1385
0262
T 1695
T 0272
T 1370
T 0754
1169
1999
1373
T 1161
T 1003
T 0351
T 1596
T 1945
T 1448
0697
T 1047
T 0234
T 1692
T 1038
T 0748
T 1786
T 1785
0464
1787
T 0747
T 1386
1039
1041
1040
T 1331
0460
1934
1800
1310
0734
T 1254
T 1803
0766
T 0751
T 1795
T 0101
T 0947
T 1513
0471
T 1517
0783
0789
T 1465
T 0307
T 1718
T 1182
T 1418
T 1439
T 1355
1033
1397
T 1642
T 1644
T 1568
T 0818
T 0923
0979
T 2009
T 0540
0491
0501
T 0499
T 0531
T 1179
T 1260
T 0619
T 0903
T 1834
0841
T 1366
T 1478
1352
T 1351
1144
1353
1145
T 1148
T 0937
0948
T 1680
T 0322
T 0470
T 0746
T 1848
T 1877
T 0765
T 0771
T 1722
T 1496
T 1398
T 1466
T 0435
T 1552
T 0795
T 0346
T 0773
1767
1769
T 0851
1265
1309
0450
1583
0130
0270
T 0229
0468
T 0855
1425
0842
T 0843
0856
T 1036
1380
T 1763
0767
T 1762
T 0284
T 1584
T 0191
T 1091
T 1489
1027
T 1472
T 1094
T 1093
T 1989
1841
1141
T 0469
T 0716
T 0461
0744
0459
1963
1961
1977
T 0768
0445
1610
0448
T 0875
T 0104
T 0107
0770
0122
T 1021
T 1654
T 1983
1026
T 1253
T 1990
T 1857
T 1307
1308
T 0949

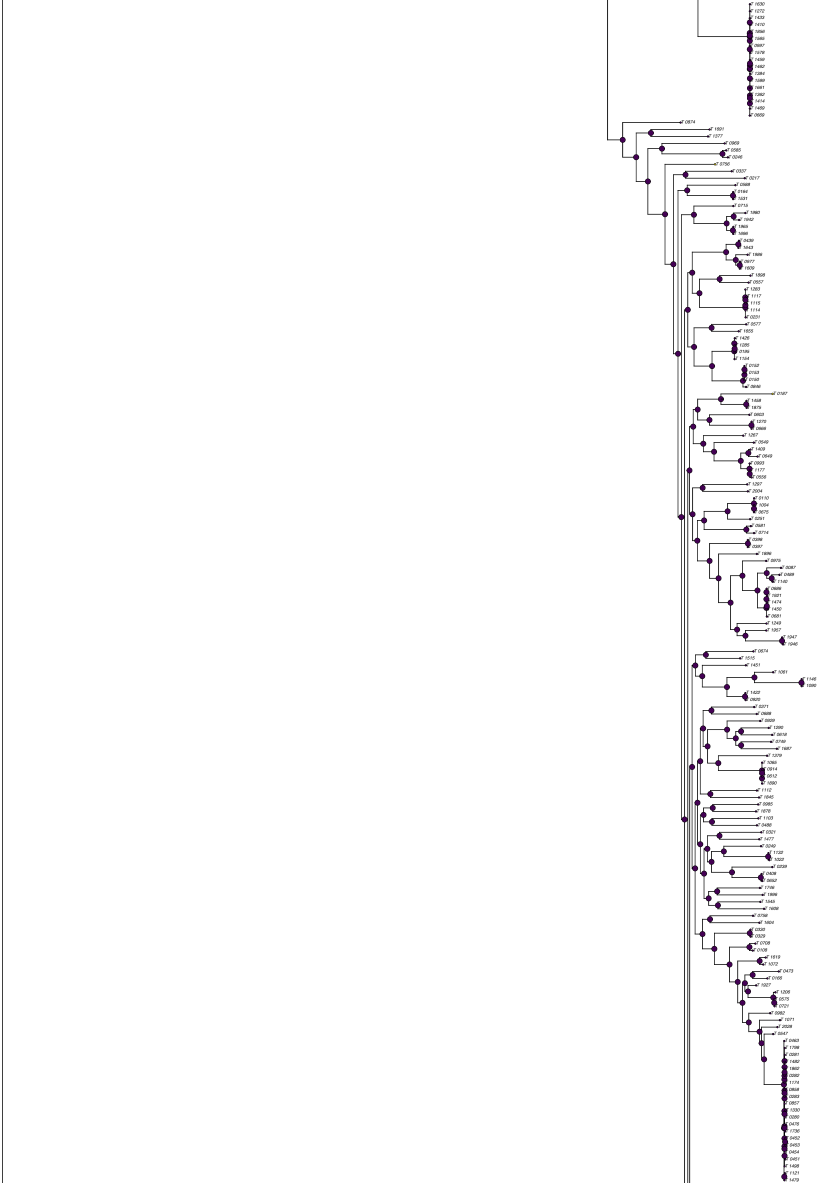
T 1630
T 1272
T 1433
T 1410
T 1856
T 1565
T 0997
T 1578
T 1459
T 1462
T 1384
T 1599
T 1661
T 1362
T 1414
T 1469
T 0669
T 0874
T 1691
T 1377
T 0969
T 0585
T 0246
T 0756
T 0337
T 0217
T 0588
T 0164
T 1531
T 0715
T 1980
T 1942
T 1965
T 1696
T 0439
T 1643
T 1986
T 0977
T 1609
T 1898
T 0557
T 1283
T 1117
T 1115
T 1114
T 0231
T 0577
T 1655
T 1426
T 1285
T 0195
T 1154
T 0152
T 0153
T 0150
T 0846
T 0187
T 1458
T 1875
T 0603
T 1270
T 0666
T 1267
T 0549
T 1409
T 0649
T 0993
T 1177
T 0556
T 1297
T 2004
T 0110
T 1004
T 0675
T 0251
T 0581
T 0714
T 0398
T 0397
T 1896
T 0975
T 0087
T 0489
T 1140
T 0686
T 1921
T 1474
T 1450
T 0681
T 1249
T 1957
T 1947
T 1946
T 0674
T 1515
T 1451
T 1061
T 1146
T 1090
T 1422
T 0920
T 0371
T 0688
T 0929
T 1290
T 0618
T 0749
T 1687
T 1379
T 1065
T 0914
T 0612
T 1890
T 1112
T 1845
T 0985
T 1878
T 1103
T 0488
T 0321
T 1477
T 0249
T 1132
T 1022
T 0239
T 0408
T 0652
T 1746
T 1996
T 1545
T 1608
T 0758
T 1604
T 0330
T 0329
T 0708
T 0108
T 1619
T 1072
T 0473
T 0166
T 1927
T 1206
T 0575
T 0721
T 0982
T 1071
T 2028
T 0547
T 0463
T 1798
T 0281
T 1482
T 1862
T 0282
T 1174
T 0858
T 0283
T 0857
T 1330
T 0280
T 0476
T 1736
T 0452
T 0453
T 0454
T 0451
T 1498
T 1121
T 1479

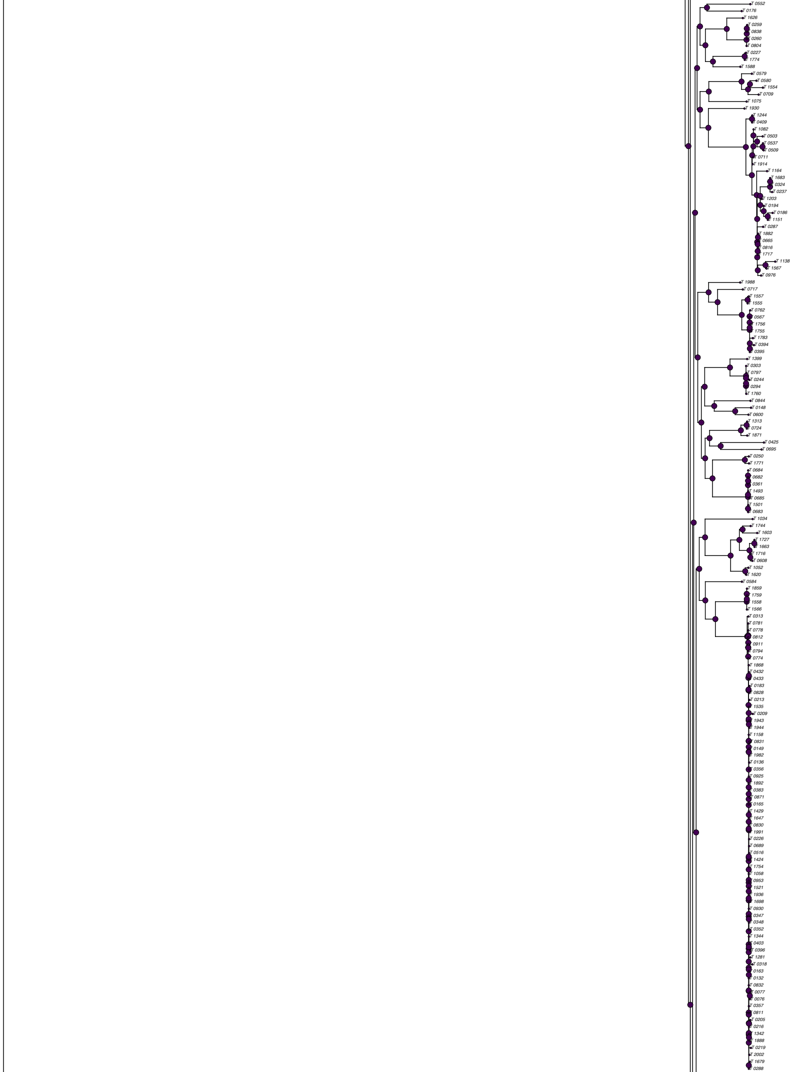
T 0552
T 0176
T 1626
T 0259
0838
0260
T 0804
T 0227
T 1774
T 1588
T 0579
T 0580
T 1554
T 0709
T 1075
T 1930
T 1244
T 0409
T 1082
T 0503
T 0537
T 0509
T 0711
T 1914
T 1164
T 1683
0324
T 0237
T 1203
T 0194
T 0186
T 1151
T 0287
T 1882
0665
T 0816
1717
T 1138
T 1567
T 0976
T 1988
T 0717
T 1557
T 1555
T 0762
0567
T 1756
1755
T 1783
T 0394
T 0395
T 1399
T 0303
T 0797
T 0244
0294
T 1760
T 0844
T 0148
T 0600
T 1313
T 0724
T 1871
T 0425
T 0695
T 0250
T 1771
T 0684
0682
0361
T 1493
T 0685
T 1501
T 0683
T 1034
T 1744
T 1603
T 1727
T 1663
T 1716
T 0608
T 1052
T 1620
T 0584
T 1859
1759
1558
T 1566
T 0313
T 0781
T 0778
0812
0911
T 0794
0774
T 1868
T 0432
0433
T 0183
T 0828
T 0213
1535
T 0209
1943
T 1944
T 1158
0831
0149
T 1982
T 0136
0356
T 0925
T 1892
0383
T 0871
0165
T 1429
T 1647
T 0830
1991
T 0226
T 0689
T 0516
1424
T 1754
T 1058
0953
1521
T 1936
1698
T 0930
0347
T 0348
T 0352
T 1344
0403
T 0396
T 1281
T 0318
0163
T 0132
T 0832
T 0077
T 0076
T 0357
0811
T 0205
T 0216
1342
T 1888
T 0219
T 2002
T 1679
T 0288

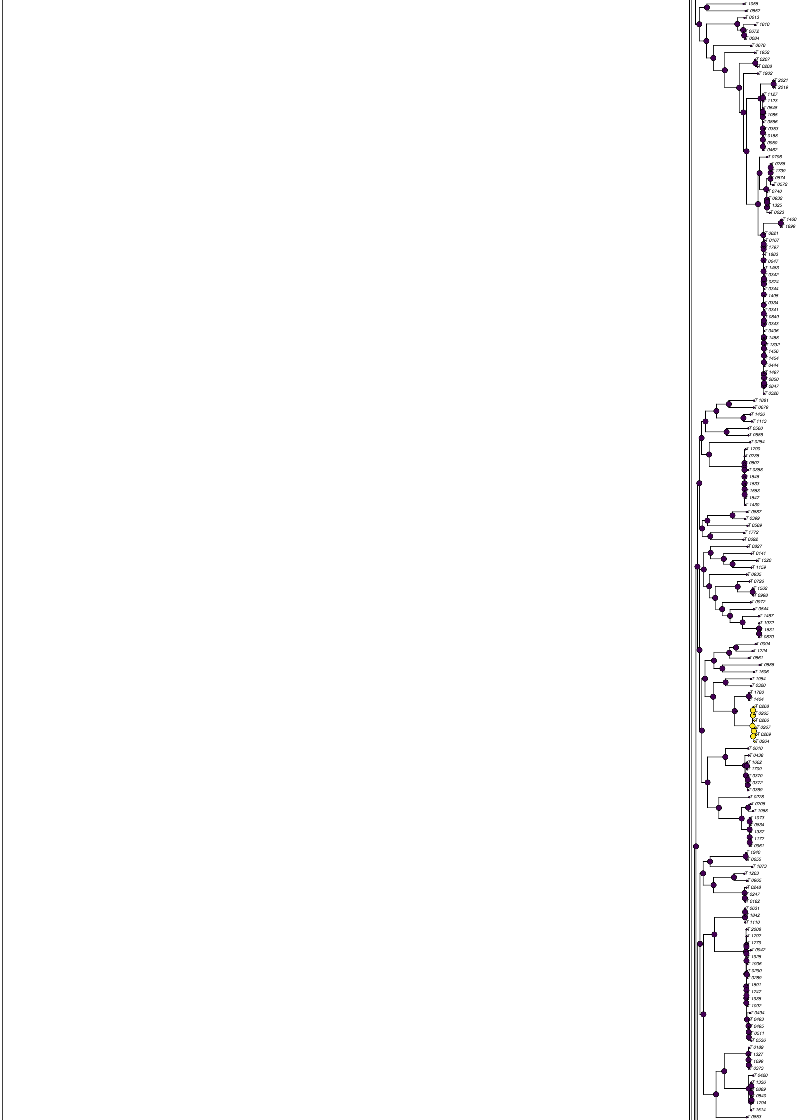
T 1055
T 0852
T 0613
T 1810
T 0672
T 0084
T 0678
T 1952
T 0207
T 0208
T 1902
T 2021
T 2019
T 1127
T 1123
T 0648
T 1085
T 0866
T 0353
T 0188
T 0950
T 0462
T 0796
T 0286
T 1739
T 0574
T 0572
T 0740
T 0932
T 1325
T 0623
T 1460
T 1899
T 0821
T 0167
T 1797
T 1883
T 0647
T 1483
T 0342
T 0374
T 0344
T 1495
T 0334
T 0341
T 0849
T 0343
T 0406
T 1488
T 1332
T 1456
T 1454
T 0444
T 1497
T 0850
T 0847
T 0326
T 1881
T 0679
T 1436
T 1113
T 0560
T 0586
T 0254
T 1790
T 0235
T 0802
T 0358
T 1546
T 1533
T 1553
T 1547
T 1430
T 0887
T 0399
T 0589
T 1772
T 0692
T 0827
T 0141
T 1320
T 1159
T 0935
T 0726
T 1562
T 0998
T 0972
T 0544
T 1467
T 1972
T 1631
T 0870
T 0094
T 1224
T 0861
T 0886
T 1506
T 1954
T 0320
T 1780
T 1404
T 0268
T 0265
T 0266
T 0267
T 0269
T 0264
T 0610
T 0438
T 1662
T 1709
T 0370
T 0372
T 0369
T 0228
T 0206
T 1968
T 1073
T 0834
T 1337
T 1172
T 0961
T 1240
T 0655
T 1873
T 1263
T 0965
T 0248
T 0247
T 0182
T 0631
T 1842
T 1110
T 2008
T 1792
T 1779
T 0942
T 1925
T 1906
T 0290
T 0289
T 1591
T 1747
T 1935
T 1092
T 0494
T 0493
T 0495
T 0511
T 0536
T 0189
T 1327
T 1699
T 0373
T 0420
T 1336
T 0889
T 0840
T 1794
T 1514
T 0853

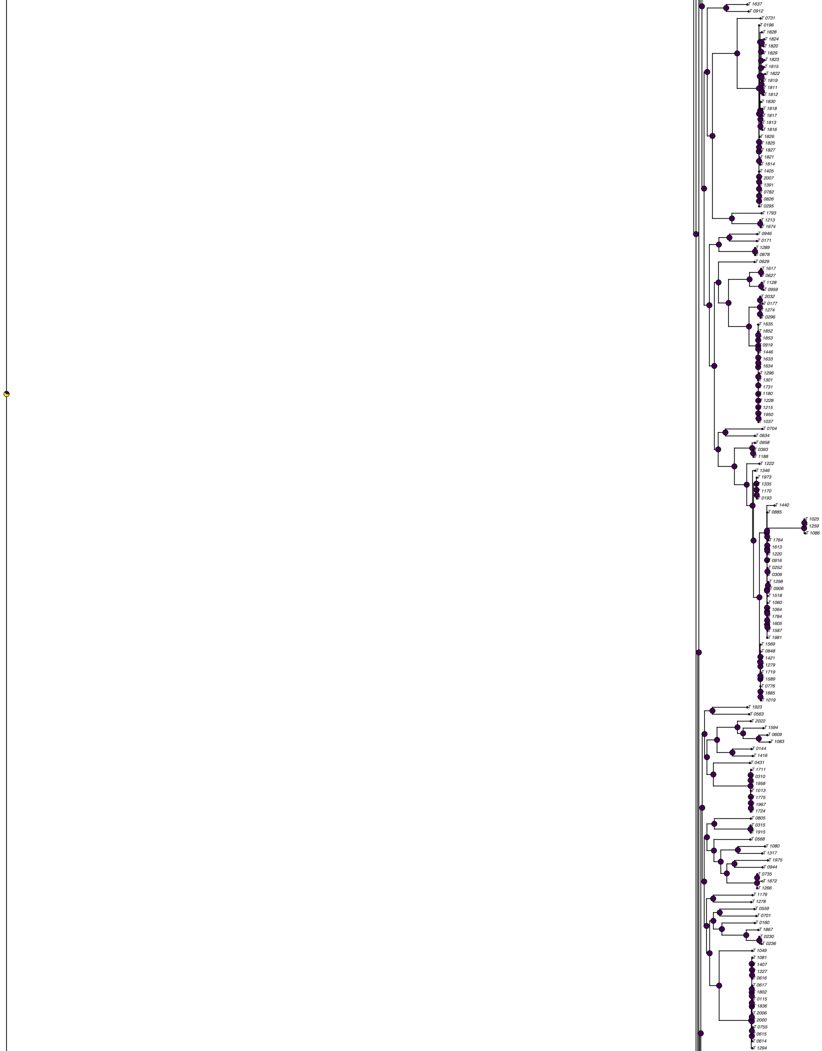
T 1637
T 0912
T 0731
T 0196
T 1828
T 1824
T 1820
T 1829
T 1823
T 1815
T 1822
T 1819
T 1811
T 1812
T 1830
T 1818
T 1817
T 1813
T 1816
T 1826
T 1825
T 1827
T 1821
T 1814
T 1405
2007
1391
0782
0826
T 0295
T 1793
T 1213
T 1674
T 0946
T 0171
T 1289
T 0878
T 0629
T 1617
T 0627
T 1128
T 0959
T 2032
T 0177
T 1274
T 0296
T 1635
T 1852
1853
0919
T 1446
1633
1634
T 1296
T 1301
1731
1180
T 1228
1215
1950
T 1037
T 0704
T 0634
T 0958
T 0393
T 1188
T 1222
T 1346
T 1973
1335
1170
T 0193
T 1440
T 0885
T 1025
1259
T 1086
T 1764
1613
T 1220
0916
T 0252
T 0306
T 1298
T 0906
T 1518
T 1060
1064
T 1784
1605
T 1587
T 1981
T 1569
T 0848
1421
1279
T 1719
1589
T 0776
1885
T 1019
T 1923
T 0563
T 2022
T 1594
T 0609
T 1083
T 0144
T 1416
T 0431
T 1711
0310
1958
T 1013
1775
1967
1724
T 0805
T 0315
T 1915
T 0568
T 1080
T 1317
T 1975
T 0944
T 0735
T 1872
T 1266
T 1176
T 1278
T 0559
T 0701
T 0160
T 1867
T 0230
T 0236
T 1049
T 1081
1407
1227
0616
T 0617
1802
T 0115
1836
T 2006
2000
T 0755
0615
T 0614
T 1294

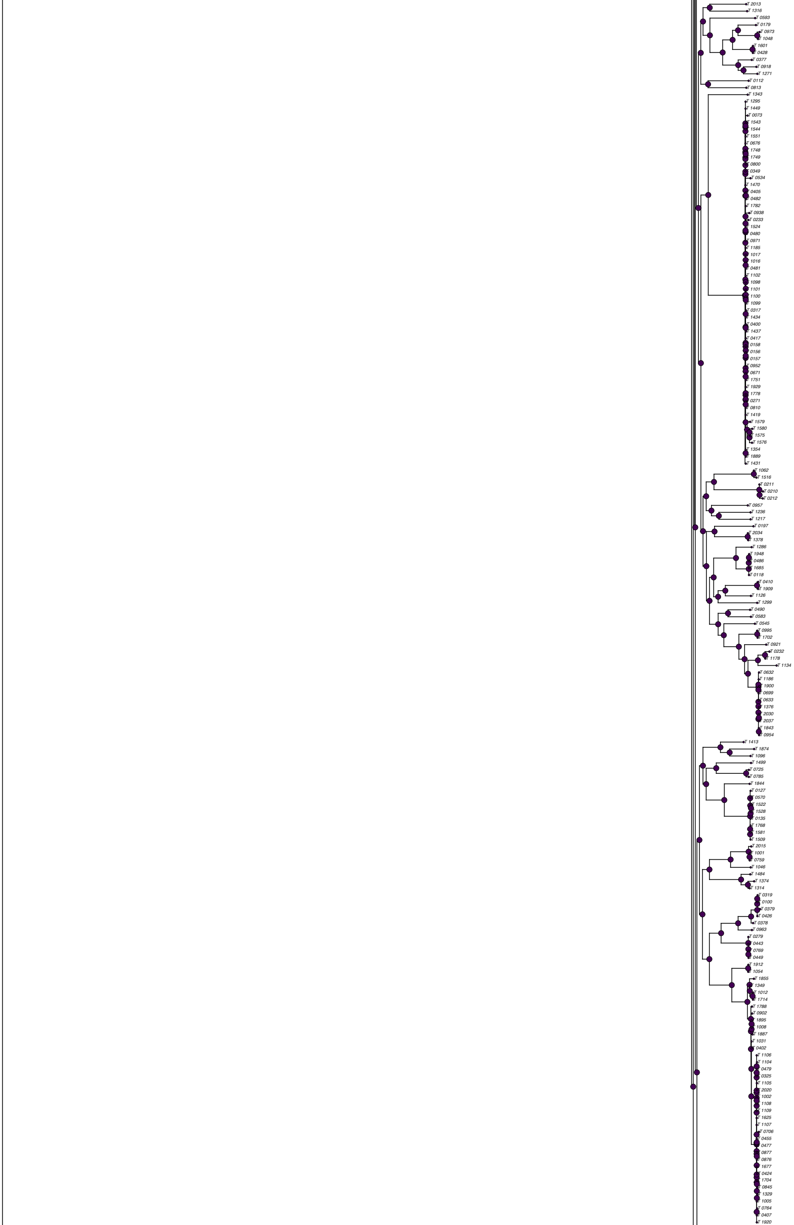

T 2013
T 1316
T 0593
T 0179
T 0973
T 1048
T 1601
T 0428
T 0377
T 0918
T 1271
T 0112
T 0813
T 1343
T 1295
T 1449
T 0073
1543
1544
T 1551
T 0676
1748
1749
0800
0349
T 0534
T 1470
0405
0482
T 1782
T 0938
T 0233
1524
0480
T 0971
T 1185
1017
1016
0481
T 1102
1098
1101
1100
T 1099
T 0317
T 1434
T 0400
T 1437
T 0417
0158
0156
0157
T 0952
0671
T 1751
T 1929
1778
0271
T 0810
T 1419
T 1579
T 1580
1575
T 1576
T 1354
T 1889
T 1431
T 1062
T 1516
T 0211
T 0210
T 0212
T 0957
T 1236
T 1217
T 0197
T 2034
T 1378
T 1286
T 1948
0486
1685
T 0118
T 0410
T 1909
T 1126
T 1299
T 0490
T 0583
T 0545
T 0995
T 1702
T 0921
T 0232
T 1178
T 1134
T 0632
T 1186
1900
T 0699
T 0633
1376
2030
2037
T 1843
T 0954
T 1413
T 1874
T 1096
T 1499
T 0725
T 0785
T 1844
T 0127
0570
1522
1528
T 0135
T 1768
1581
T 1509
T 2015
T 1001
T 0759
T 1046
T 1484
T 1374
T 1314
T 0319
0100
T 0379
T 0426
T 0378
T 0963
T 0279
0443
0769
T 0449
T 1912
T 1054
T 1855
1349
T 1012
T 1714
T 1788
T 0902
1895
1008
T 1887
T 1031
0402
T 1106
T 1104
0479
0325
T 1105
2020
1002
1108
1109
T 1625
T 1107
T 0706
T 0455
0477
0877
T 0876
1677
0424
1704
T 0845
T 1329
T 1005
T 0764
T 0407
T 1920

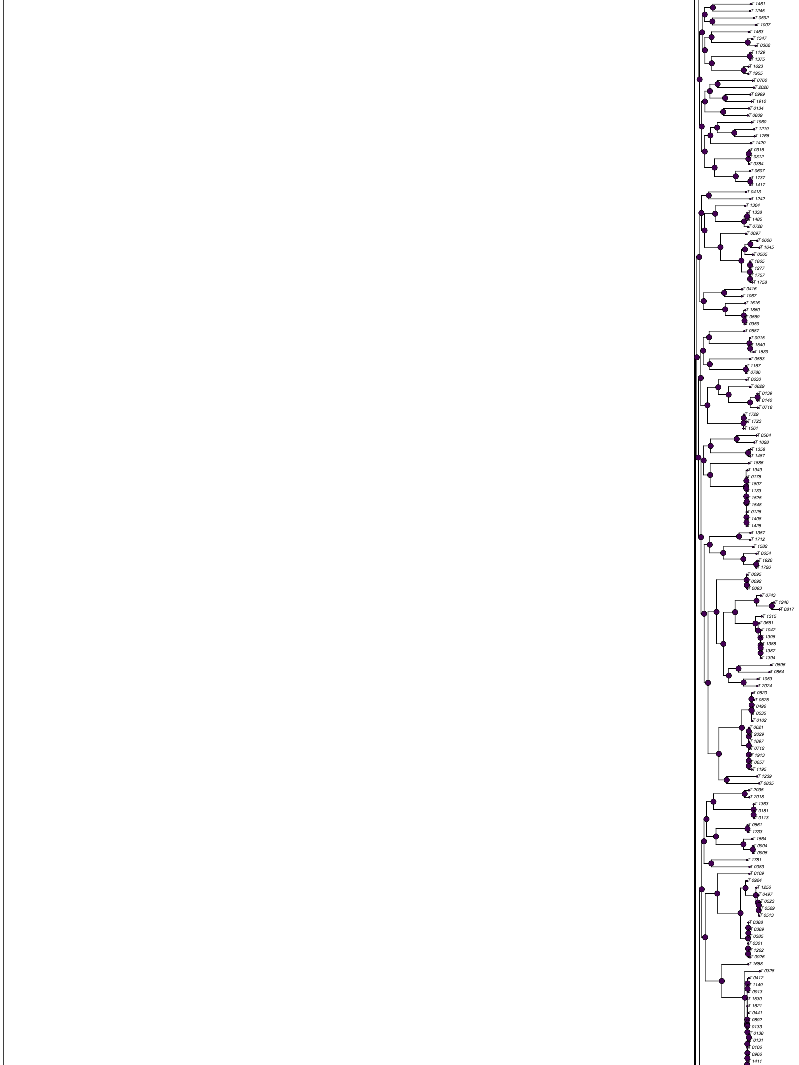
T 1461
T 1245
T 0592
T 1007
T 1463
T 1347
T 0362
T 1129
T 1375
T 1623
T 1955
T 0760
T 2026
T 0999
T 1910
T 0134
T 0809
T 1960
T 1219
T 1766
T 1420
T 0316
T 0312
T 0384
T 0607
T 1737
T 1417
T 0413
T 1242
T 1304
T 1338
T 1485
T 0728
T 0097
T 0606
T 1645
T 0565
T 1865
1277
1757
T 1758
T 0416
T 1067
T 1616
T 1860
0569
T 0359
T 0587
T 0915
1540
T 1539
T 0553
T 1167
T 0786
T 0630
T 0829
T 0139
0140
T 0718
T 1729
T 1723
T 1561
T 0564
T 1028
T 1358
T 1487
T 1886
T 1949
T 0178
1807
1133
1525
1548
T 0126
1408
T 1428
T 1357
T 1712
T 1582
T 0654
T 1926
T 1726
T 0095
0092
T 0093
T 0743
T 1246
T 0817
T 1315
T 0661
T 1042
1396
T 1388
1387
T 1394
T 0596
T 0864
T 1053
T 2024
T 0620
T 0525
0496
T 0535
T 0102
T 0621
2029
T 1897
T 0712
T 1913
T 0657
T 1195
T 1239
T 0835
T 2035
T 2018
T 1363
0181
T 0113
T 0561
T 1733
T 1564
T 0904
T 0905
T 1781
T 0083
T 0109
T 0924
T 1256
T 0497
T 0523
T 0529
T 0513
T 0388
T 0389
T 0385
T 0301
T 1262
T 0926
T 1688
T 0328
T 0412
1149
T 0913
T 1530
T 1621
T 0441
0892
0133
T 0138
0131
T 0106
0966
1411

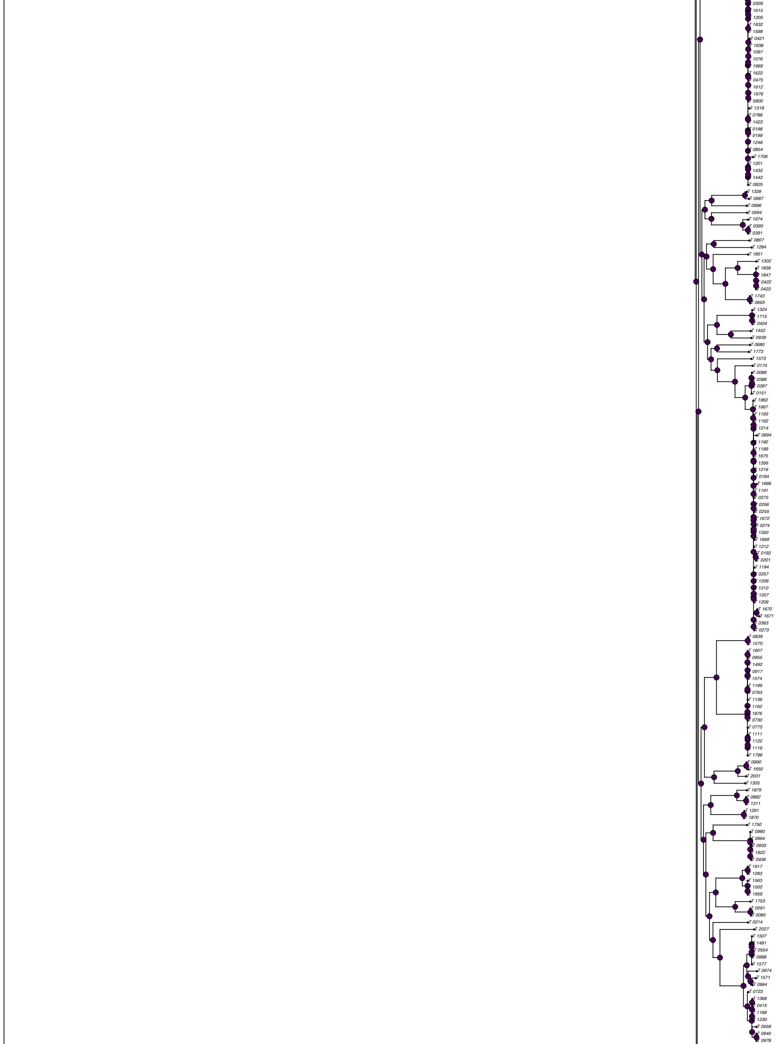
0309
1615
1205
T 1632
T 1598
T 0421
T 1938
T 1097
T 1076
1969
T 1622
T 0475
T 1612
T 1976
T 0900
T 1318
T 0788
T 1423
T 0198
T 0199
1248
T 0854
T 1706
T 1201
1432
1442
T 0825
T 1339
T 0687
T 0996
T 0594
T 1974
T 0390
T 0391
T 0807
T 1284
T 1851
T 1302
T 1838
1847
T 0422
T 0423
T 1743
T 0693
T 1324
1715
T 0404
T 1452
T 0939
T 0680
T 1773
T 1573
T 0174
T 0089
0386
0387
T 0151
T 1962
T 1667
T 1193
T 1192
1214
T 0694
1190
T 1189
T 1675
1356
1216
T 0184
T 1666
T 1191
T 0275
T 0256
T 0255
T 1672
T 0274
1350
T 1669
T 1212
T 0192
T 0201
T 1194
T 0257
T 1208
T 1210
T 1207
T 1209
T 1670
T 1671
T 0363
T 0273
T 0839
T 1070
T 1607
T 0955
T 1492
0917
T 1574
T 1199
T 0763
T 1136
1162
1876
T 0730
T 0775
T 1111
1122
1119
T 1796
T 0990
T 1650
T 2031
T 1305
T 1879
T 0882
T 1311
T 1291
T 1870
T 1750
T 0980
T 0994
T 0933
T 1922
T 0936
T 1917
T 1293
T 1563
T 1502
T 1959
T 1753
T 0091
T 0085
T 0214
T 2027
T 1507
1481
T 0554
T 0988
T 1577
T 0974
T 1571
T 0964
T 0723
T 1368
T 0415
1168
1230
T 0558
T 0940
T 0978

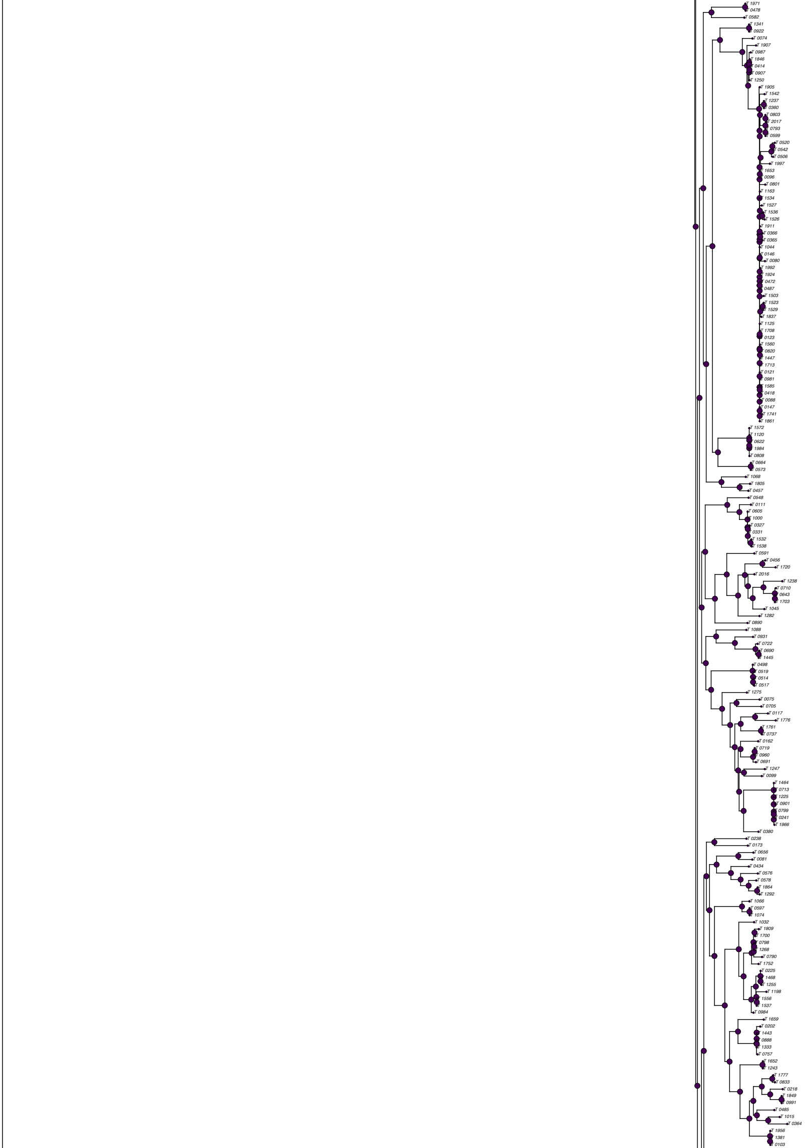
T 1971
T 0478
T 0582
T 1341
T 0922
T 0074
T 1907
T 0987
T 1846
T 0414
T 0907
T 1250
T 1905
T 1542
T 1237
T 0360
T 0803
T 2017
T 0793
T 0599
T 0520
T 0542
T 0506
T 1997
T 1653
T 0096
T 0801
T 1163
T 1534
T 1527
T 1536
T 1526
T 1911
T 0366
T 0365
T 1044
T 0146
T 0080
T 1992
T 1924
T 0472
T 0487
T 1503
T 1523
T 1529
T 1837
T 1125
T 1708
T 0123
T 1560
T 0820
T 1447
T 1713
T 0121
T 0981
T 1585
T 0418
T 0088
T 0147
T 1741
T 1861
T 1572
T 1120
T 0622
T 1984
T 0808
T 0664
T 0573
T 1068
T 1805
T 0457
T 0548
T 0111
T 0605
T 1000
T 0327
T 0331
T 1532
T 1538
T 0591
T 0456
T 1720
T 2016
T 1238
T 0710
T 0643
T 1703
T 1045
T 1282
T 0890
T 1088
T 0931
T 0722
T 0690
T 1445
T 0498
T 0519
T 0514
T 0517
T 1275
T 0075
T 0705
T 0117
T 1776
T 1761
T 0737
T 0162
T 0719
T 0960
T 0691
T 1247
T 0099
T 1464
T 0713
T 1225
T 0901
T 0799
T 0241
T 1966
T 0380
T 0238
T 0173
T 0656
T 0081
T 0434
T 0576
T 0578
T 1864
T 1292
T 1066
T 0597
T 1074
T 1032
T 1809
T 1700
T 0798
T 1268
T 0790
T 1752
T 0225
T 1468
T 1255
T 1198
T 1556
T 1537
T 0984
T 1659
T 0202
T 1443
T 0888
T 1333
T 0757
T 1652
T 1243
T 1777
T 0833
T 0218
T 1849
T 0991
T 0485
T 1015
T 0364
T 1956
T 1381
T 0103

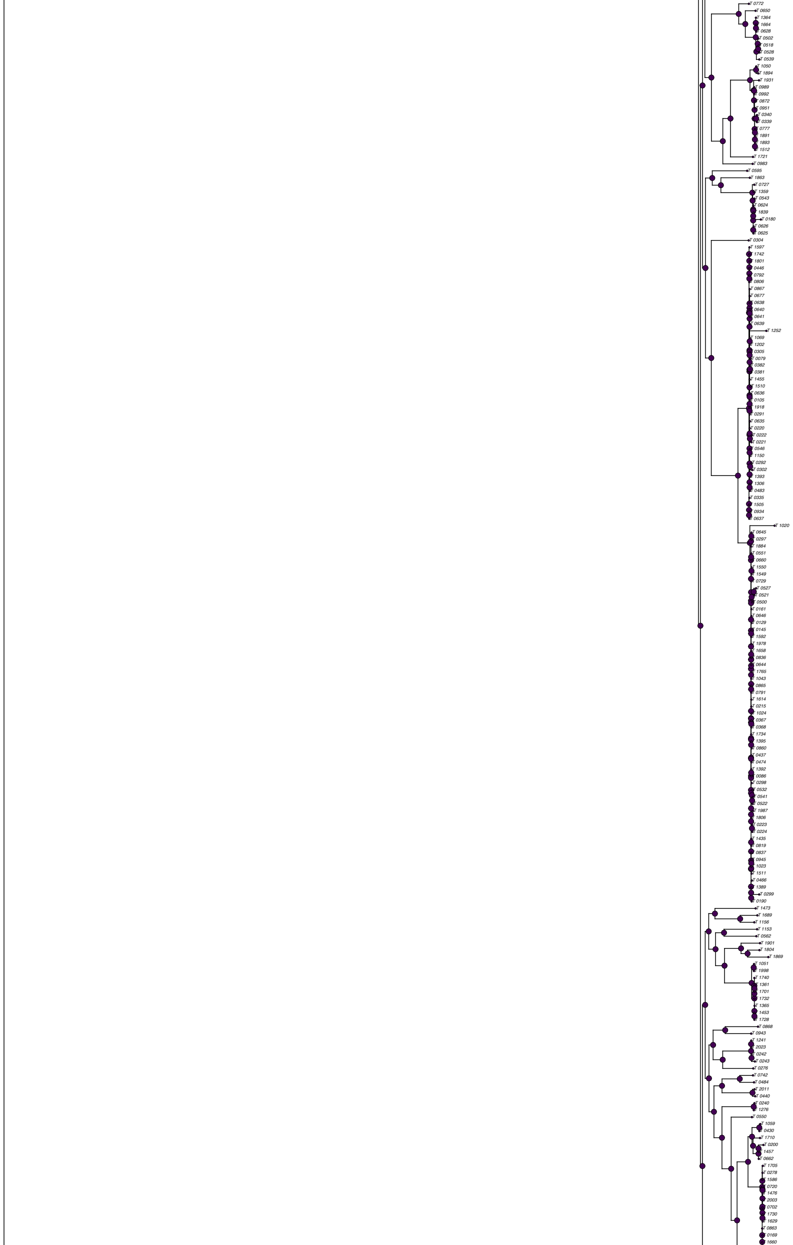

T 0772
T 0650
T 1364
1664
0628
T 0502
0518
T 0528
T 0539
T 1050
T 1894
T 1931
T 0989
0992
0872
0951
T 0340
T 0339
0777
1891
1893
1512
T 1721
T 0983
T 0595
T 1863
T 0727
T 1359
T 0543
T 0624
1839
T 0180
T 0626
T 0625
T 0304
T 1597
1742
1801
0446
0792
T 0806
T 0867
T 0677
0638
0640
0641
T 0639
T 1252
T 1069
T 1202
0305
T 0079
0382
0381
T 1455
1510
T 0636
0105
1918
T 0291
T 0635
T 0220
T 0222
T 0221
0546
1150
T 0292
T 0302
1393
1306
T 0483
T 0335
1505
0934
T 0637
T 1020
T 0645
0297
1884
T 0551
0660
T 1550
1549
0729
T 0527
T 0521
0500
T 0161
0646
0129
T 0145
T 1592
T 1978
1658
0836
0644
T 1765
T 1043
0865
T 0791
T 1614
T 0215
1024
0367
T 0368
T 1734
1395
T 0860
0437
T 0474
T 1392
0086
T 0298
T 0532
T 0541
T 0522
T 1987
1806
T 0223
T 0224
T 1435
T 0819
T 0837
0945
1023
T 1511
T 0466
1389
T 0299
T 0190
T 1473
T 1689
T 1156
T 1153
T 0562
T 1901
T 1804
T 1869
T 1051
T 1998
T 1740
1361
1701
1732
T 1365
1453
T 1728
T 0868
T 0943
T 1241
2023
0242
T 0243
T 0276
T 0742
T 0484
T 2011
T 0440
T 0240
T 1276
T 0550
T 1059
0430
T 1710
T 0200
1457
T 0662
T 1705
T 0278
1586
0720
1476
2003
0702
1730
1629
T 0863
0169
1660

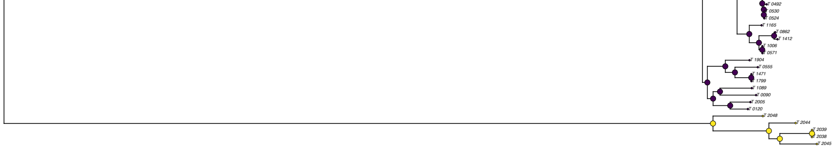

T 0492
T 0530
T 0524
T 1165
T 0862
T 1412
T 1006
T 0571
T 1904
T 0555
T 1471
T 1799
T 1089
T 0090
T 2005
T 0120
T 2048
T 2044
T 2039
T 2038
T 2045

Effector gene: tse5

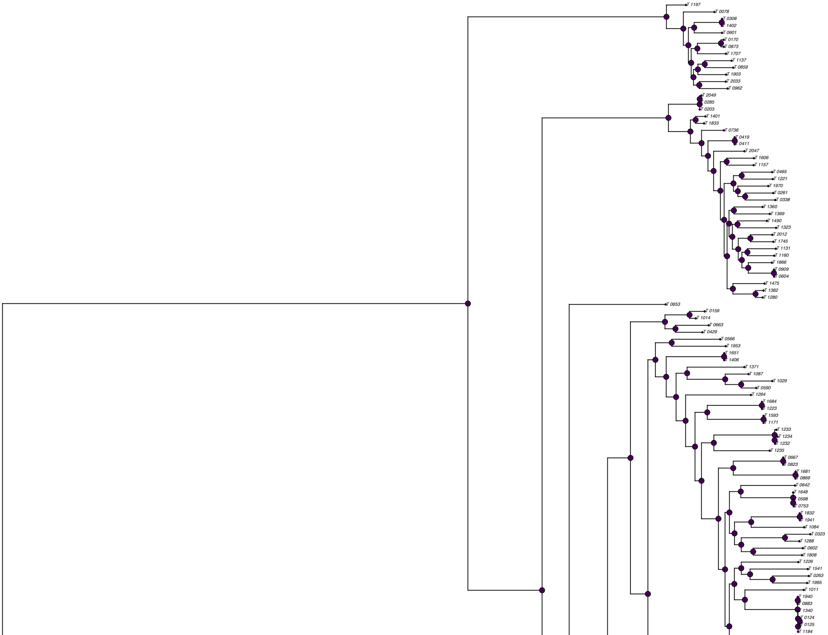

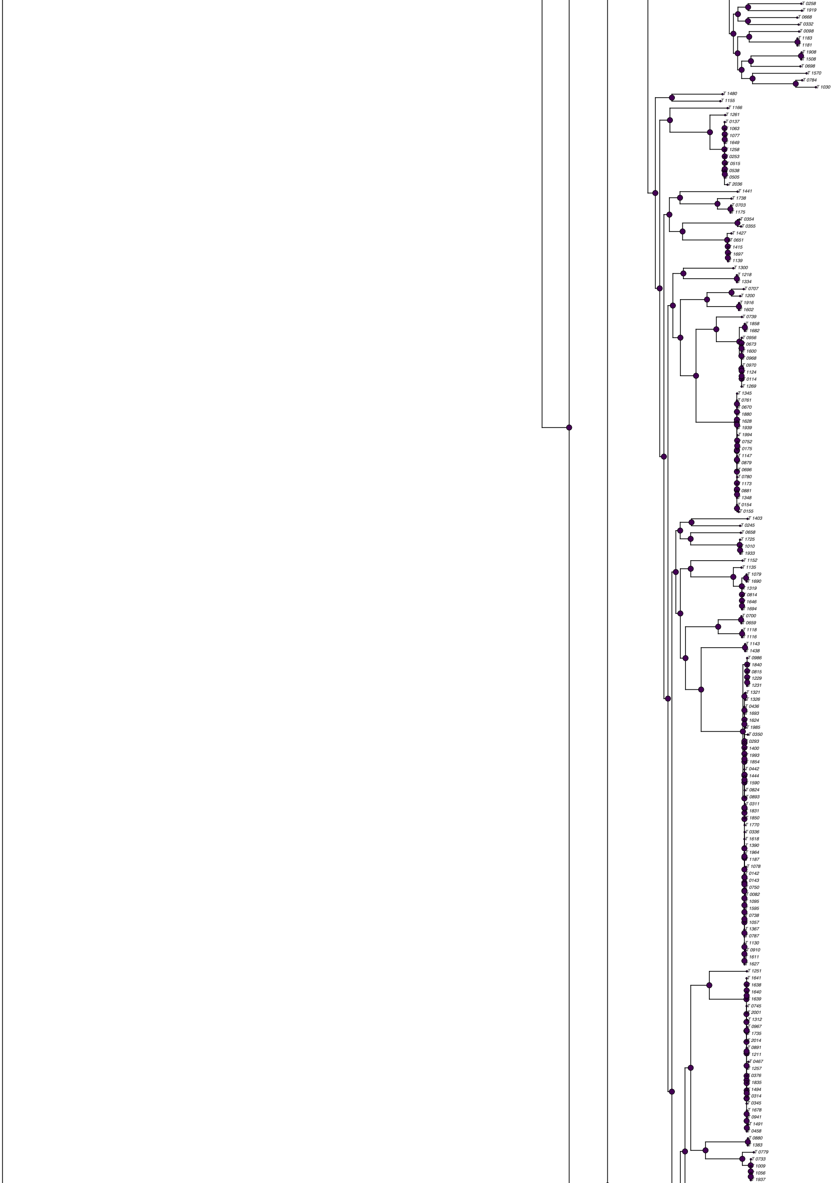

T 0258
T 1919
T 0668
T 0332
T 0098
T 1183
1181
T 1908
1508
T 0698
T 1570
T 0784
T 1030
T 1480
T 1155
T 1166
T 1261
T 0137
1063
1077
1649
1258
0253
0515
0538
0505
T 2036
T 1441
T 1738
T 0703
1175
T 0354
T 0355
T 1427
0651
1415
1697
1139
T 1300
T 1218
1334
T 0707
T 1200
T 1916
1602
T 0739
T 1858
1682
T 0956
0673
1600
0968
T 0970
1124
0114
T 1269
T 1345
T 0761
0670
1880
1628
1939
T 1994
0752
0175
T 1147
0879
T 0696
T 0780
1173
0881
T 1348
T 0154
T 0155
T 1403
T 0245
T 0658
T 1725
1010
1933
T 1152
T 1135
T 1079
1690
1319
0814
1646
1694
T 0700
T 0659
T 1118
1116
T 1143
1438
T 0986
1840
0815
1229
1231
T 1321
1326
0436
1693
1624
T 1985
T 0350
0293
1400
1993
1854
T 0442
1444
1590
T 0824
0893
T 0311
1831
1850
T 1770
T 0336
T 1618
T 1390
1964
1187
T 1078
0142
0143
0750
T 0082
1095
1595
T 0738
1057
T 1367
T 0787
T 1130
T 0910
1611
1627
T 1251
T 1641
1638
1640
1639
T 0745
2001
T 1312
T 0967
1735
2014
T 0891
1211
T 0467
1257
0376
1835
1494
0314
T 0345
T 1678
0941
T 1491
0458
T 0880
1383
T 0779
T 0733
1009
1056
1937

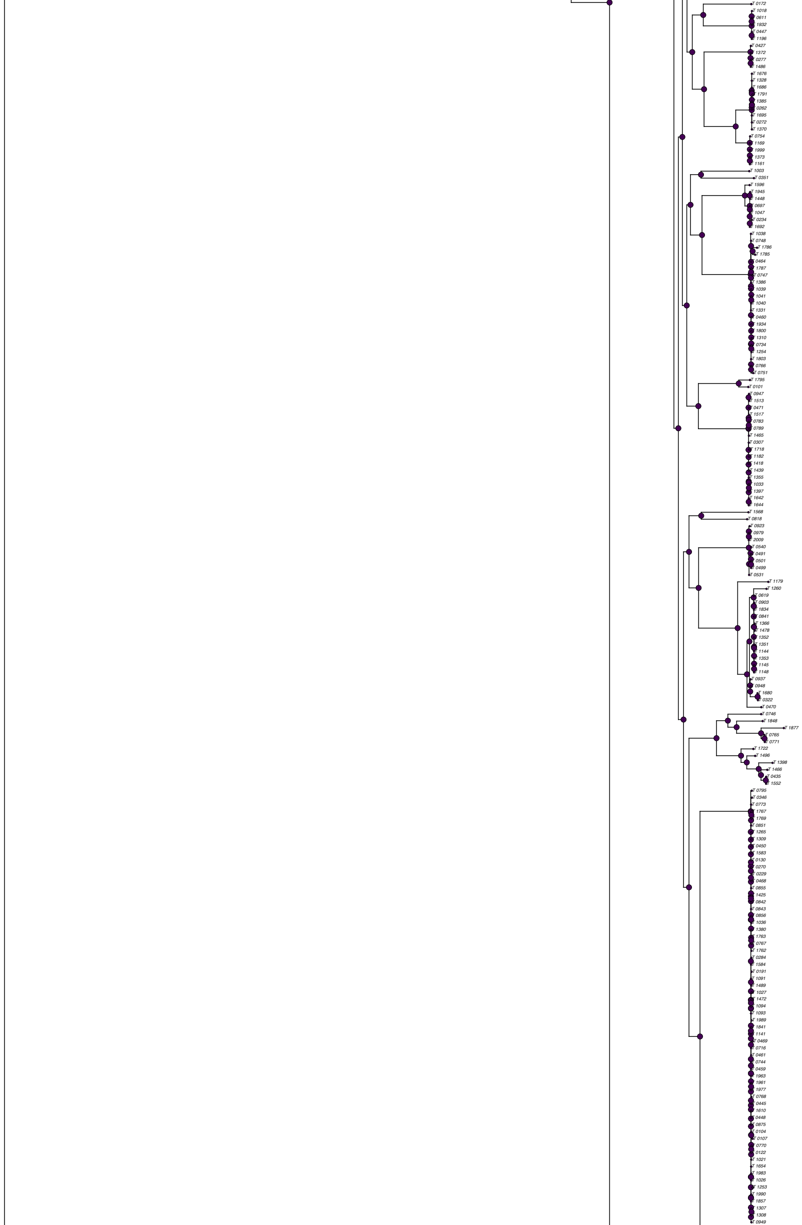
T 0172
T 1018
0611
1932
T 0447
T 1196
T 0427
1372
T 0277
T 1486
T 1676
T 1328
T 1686
1791
1385
0262
T 1695
T 0272
T 1370
T 0754
1169
1999
1373
T 1161
T 1003
T 0351
T 1596
T 1945
T 1448
0697
T 1047
T 0234
T 1692
T 1038
T 0748
T 1786
T 1785
0464
1787
T 0747
T 1386
1039
1041
1040
T 1331
0460
1934
1800
1310
0734
T 1254
T 1803
0766
T 0751
T 1795
T 0101
T 0947
T 1513
0471
T 1517
0783
0789
T 1465
T 0307
T 1718
T 1182
1418
T 1439
T 1355
1033
1397
T 1642
T 1644
T 1568
T 0818
T 0923
0979
T 2009
T 0540
0491
0501
T 0499
T 0531
T 1179
T 1260
0619
T 0903
T 1834
0841
T 1366
T 1478
1352
1351
1144
1353
1145
T 1148
T 0937
0948
T 1680
T 0322
T 0470
T 0746
T 1848
T 1877
T 0765
T 0771
T 1722
T 1496
T 1398
T 1466
T 0435
T 1552
T 0795
T 0346
T 0773
1767
1769
T 0851
1265
1309
0450
T 1583
T 0130
0270
T 0229
0468
T 0855
1425
0842
T 0843
0856
T 1036
1380
T 1763
0767
T 1762
T 0284
1584
T 0191
T 1091
T 1489
1027
T 1472
T 1094
T 1093
T 1989
1841
1141
T 0469
T 0716
T 0461
0744
T 0459
1963
1961
1977
T 0768
0445
1610
0448
T 0875
T 0104
T 0107
0770
0122
T 1021
T 1654
T 1983
1026
T 1253
T 1990
T 1857
T 1307
1308
T 0949

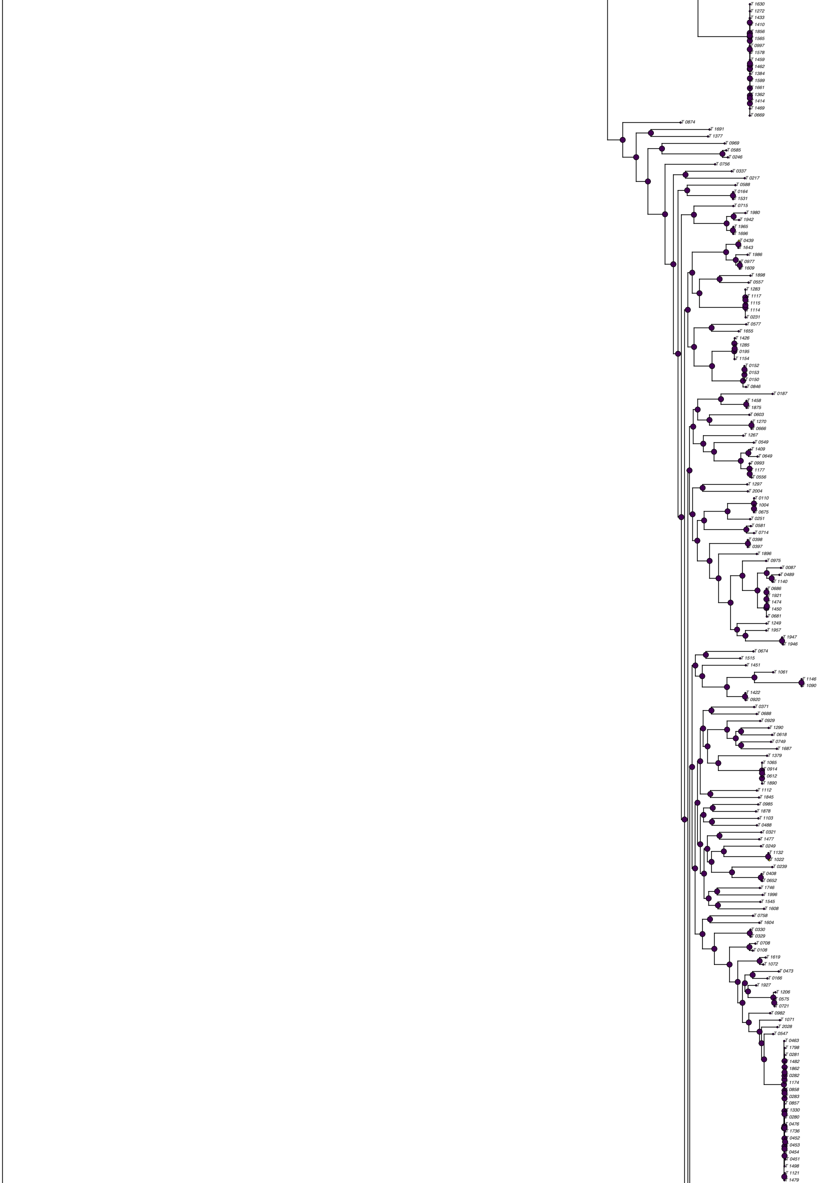

T 1630
T 1272
T 1433
T 1410
T 1856
T 1565
T 0997
T 1578
T 1459
T 1462
T 1384
T 1599
T 1661
T 1362
T 1414
T 1469
T 0669
T 0874
T 1691
T 1377
T 0969
T 0585
T 0246
T 0756
T 0337
T 0217
T 0588
T 0164
T 1531
T 0715
T 1980
T 1942
T 1965
T 1696
T 0439
T 1643
T 1986
T 0977
T 1609
T 1898
T 0557
T 1283
T 1117
T 1115
T 1114
T 0231
T 0577
T 1655
T 1426
T 1285
T 0195
T 1154
T 0152
T 0153
T 0150
T 0846
T 0187
T 1458
T 1875
T 0603
T 1270
T 0666
T 1267
T 0549
T 1409
T 0649
T 0993
T 1177
T 0556
T 1297
T 2004
T 0110
T 1004
T 0675
T 0251
T 0581
T 0714
T 0398
T 0397
T 1896
T 0975
T 0087
T 0489
T 1140
T 0686
T 1921
T 1474
T 1450
T 0681
T 1249
T 1957
T 1947
T 1946
T 0674
T 1515
T 1451
T 1061
T 1146
T 1090
T 1422
T 0920
T 0371
T 0688
T 0929
T 1290
T 0618
T 0749
T 1687
T 1379
T 1065
T 0914
T 0612
T 1890
T 1112
T 1845
T 0985
T 1878
T 1103
T 0488
T 0321
T 1477
T 0249
T 1132
T 1022
T 0239
T 0408
T 0652
T 1746
T 1996
T 1545
T 1608
T 0758
T 1604
T 0330
T 0329
T 0708
T 0108
T 1619
T 1072
T 0473
T 0166
T 1927
T 1206
T 0575
T 0721
T 0982
T 1071
T 2028
T 0547
T 0463
T 1798
T 0281
T 1482
T 1862
T 0282
T 1174
T 0858
T 0283
T 0857
T 1330
T 0280
T 0476
T 1736
T 0452
T 0453
T 0454
T 0451
T 1498
T 1121
T 1479

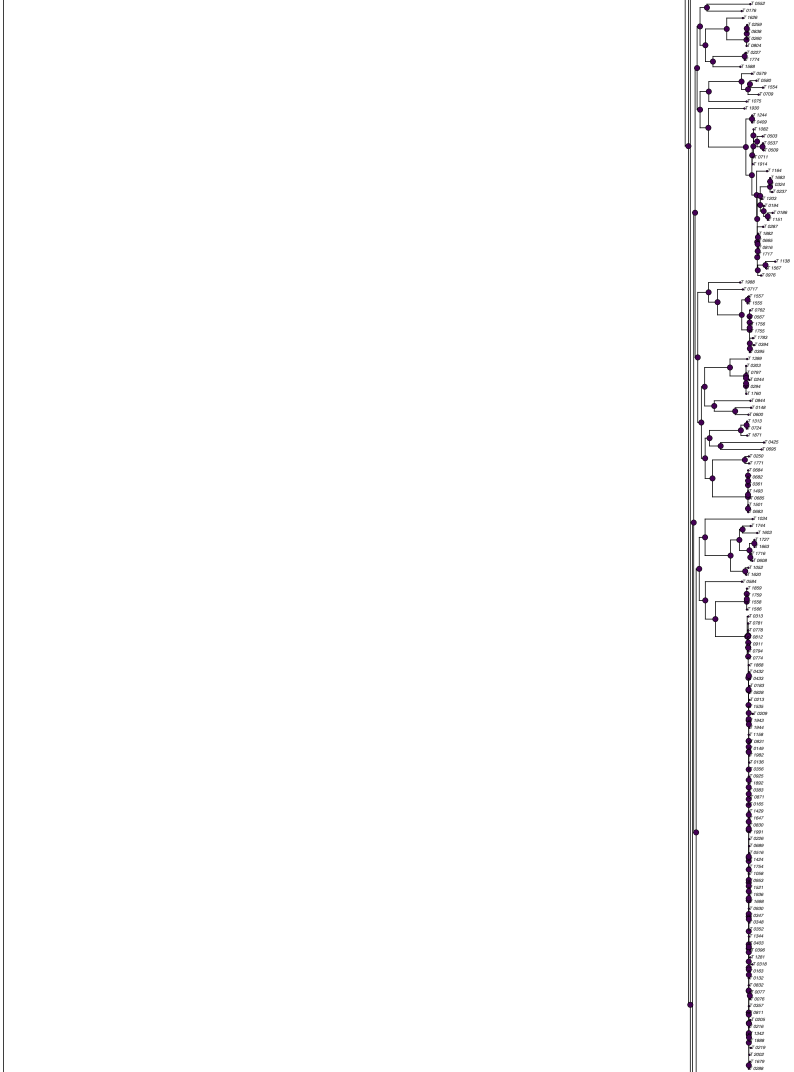
T 0552
T 0176
T 1626
T 0259
T 0838
T 0260
T 0804
T 0227
T 1774
T 1588
T 0579
T 0580
T 1554
T 0709
T 1075
T 1930
T 1244
T 0409
T 1082
T 0503
T 0537
T 0509
T 0711
T 1914
T 1164
T 1683
T 0324
T 0237
T 1203
T 0194
T 0186
T 1151
T 0287
T 1882
T 0665
T 0816
T 1717
T 1138
T 1567
T 0976
T 1988
T 0717
T 1557
T 1555
T 0762
T 0567
T 1756
T 1755
T 1783
T 0394
T 0395
T 1399
T 0303
T 0797
T 0244
T 0294
T 1760
T 0844
T 0148
T 0600
T 1313
T 0724
T 1871
T 0425
T 0695
T 0250
T 1771
T 0684
T 0682
T 0361
T 1493
T 0685
T 1501
T 0683
T 1034
T 1744
T 1603
T 1727
T 1663
T 1716
T 0608
T 1052
T 1620
T 0584
T 1859
T 1759
T 1558
T 1566
T 0313
T 0781
T 0778
T 0812
T 0911
T 0794
T 0774
T 1868
T 0432
T 0433
T 0183
T 0828
T 0213
T 1535
T 0209
T 1943
T 1944
T 1158
T 0831
T 0149
T 1982
T 0136
T 0356
T 0925
T 1892
T 0383
T 0871
T 0165
T 1429
T 1647
T 0830
T 1991
T 0226
T 0689
T 0516
T 1424
T 1754
T 1058
T 0953
T 1521
T 1936
T 1698
T 0930
T 0347
T 0348
T 0352
T 1344
T 0403
T 0396
T 1281
T 0318
T 0163
T 0132
T 0832
T 0077
T 0076
T 0357
T 0811
T 0205
T 0216
T 1342
T 1888
T 0219
T 2002
T 1679
T 0288

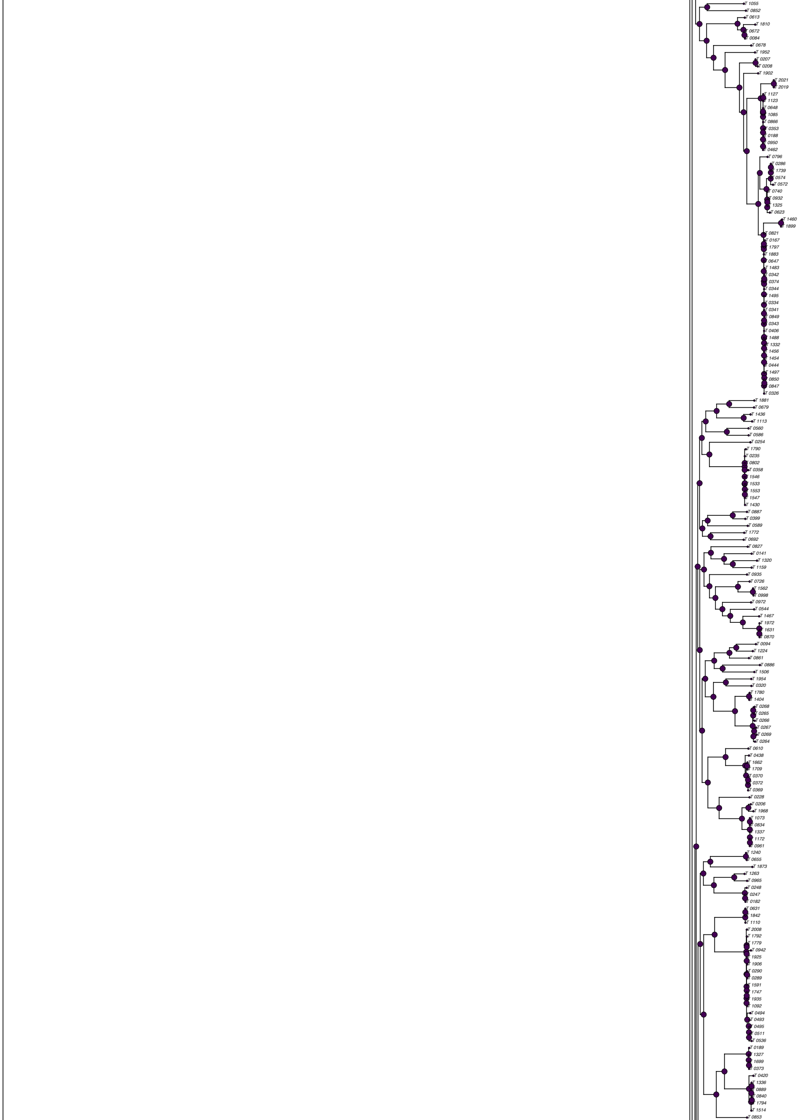

T 1055
T 0852
T 0613
T 1810
T 0672
T 0084
T 0678
T 1952
T 0207
T 0208
T 1902
T 2021
T 2019
T 1127
T 1123
T 0648
T 1085
T 0866
T 0353
T 0188
T 0950
T 0462
T 0796
T 0286
T 1739
T 0574
T 0572
T 0740
T 0932
T 1325
T 0623
T 1460
T 1899
T 0821
T 0167
T 1797
T 1883
T 0647
T 1483
T 0342
T 0374
T 0344
T 1495
T 0334
T 0341
T 0849
T 0343
T 0406
T 1488
T 1332
T 1456
T 1454
T 0444
T 1497
T 0850
T 0847
T 0326
T 1881
T 0679
T 1436
T 1113
T 0560
T 0586
T 0254
T 1790
T 0235
T 0802
T 0358
T 1546
T 1533
T 1553
T 1547
T 1430
T 0887
T 0399
T 0589
T 1772
T 0692
T 0827
T 0141
T 1320
T 1159
T 0935
T 0726
T 1562
T 0998
T 0972
T 0544
T 1467
T 1972
T 1631
T 0870
T 0094
T 1224
T 0861
T 0886
T 1506
T 1954
T 0320
T 1780
T 1404
T 0268
T 0265
T 0266
T 0267
T 0269
T 0264
T 0610
T 0438
T 1662
T 1709
T 0370
T 0372
T 0369
T 0228
T 0206
T 1968
T 1073
T 0834
T 1337
T 1172
T 0961
T 1240
T 0655
T 1873
T 1263
T 0965
T 0248
T 0247
T 0182
T 0631
T 1842
T 1110
T 2008
T 1792
T 1779
T 0942
T 1925
T 1906
T 0290
T 0289
T 1591
T 1747
T 1935
T 1092
T 0494
T 0493
T 0495
T 0511
T 0536
T 0189
T 1327
T 1699
T 0373
T 0420
T 1336
T 0889
T 0840
T 1794
T 1514
T 0853

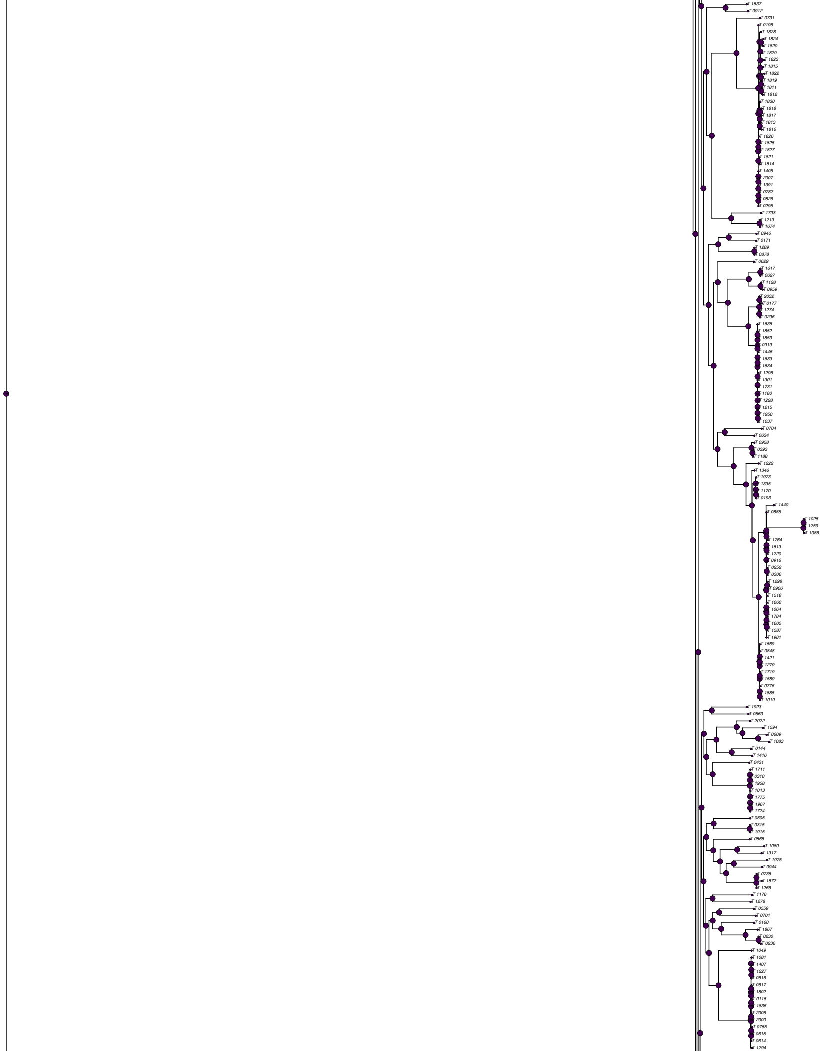
T 1637
T 0912
T 0731
T 0196
T 1828
T 1824
T 1820
T 1829
T 1823
T 1815
T 1822
T 1819
T 1811
T 1812
T 1830
T 1818
T 1817
T 1813
T 1816
T 1826
T 1825
T 1827
T 1821
T 1814
T 1405
T 2007
T 1391
T 0782
T 0826
T 0295
T 1793
T 1213
T 1674
T 0946
T 0171
T 1289
T 0878
T 0629
T 1617
T 0627
T 1128
T 0959
T 2032
T 0177
T 1274
T 0296
T 1635
T 1852
T 1853
T 0919
T 1446
T 1633
T 1634
T 1296
T 1301
T 1731
T 1180
T 1228
T 1215
T 1950
T 1037
T 0704
T 0634
T 0958
T 0393
T 1188
T 1222
T 1346
T 1973
T 1335
T 1170
T 0193
T 1440
T 0885
T 1025
T 1259
T 1086
T 1764
T 1613
T 1220
T 0916
T 0252
T 0306
T 1298
T 0906
T 1518
T 1060
T 1064
T 1784
T 1605
T 1587
T 1981
T 1569
T 0848
T 1421
T 1279
T 1719
T 1589
T 0776
T 1885
T 1019
T 1923
T 0563
T 2022
T 1594
T 0609
T 1083
T 0144
T 1416
T 0431
T 1711
T 0310
T 1958
T 1013
T 1775
T 1967
T 1724
T 0805
T 0315
T 1915
T 0568
T 1080
T 1317
T 1975
T 0944
T 0735
T 1872
T 1266
T 1176
T 1278
T 0559
T 0701
T 0160
T 1867
T 0230
T 0236
T 1049
T 1081
T 1407
T 1227
T 0616
T 0617
T 1802
T 0115
T 1836
T 2006
T 2000
T 0755
T 0615
T 0614
T 1294

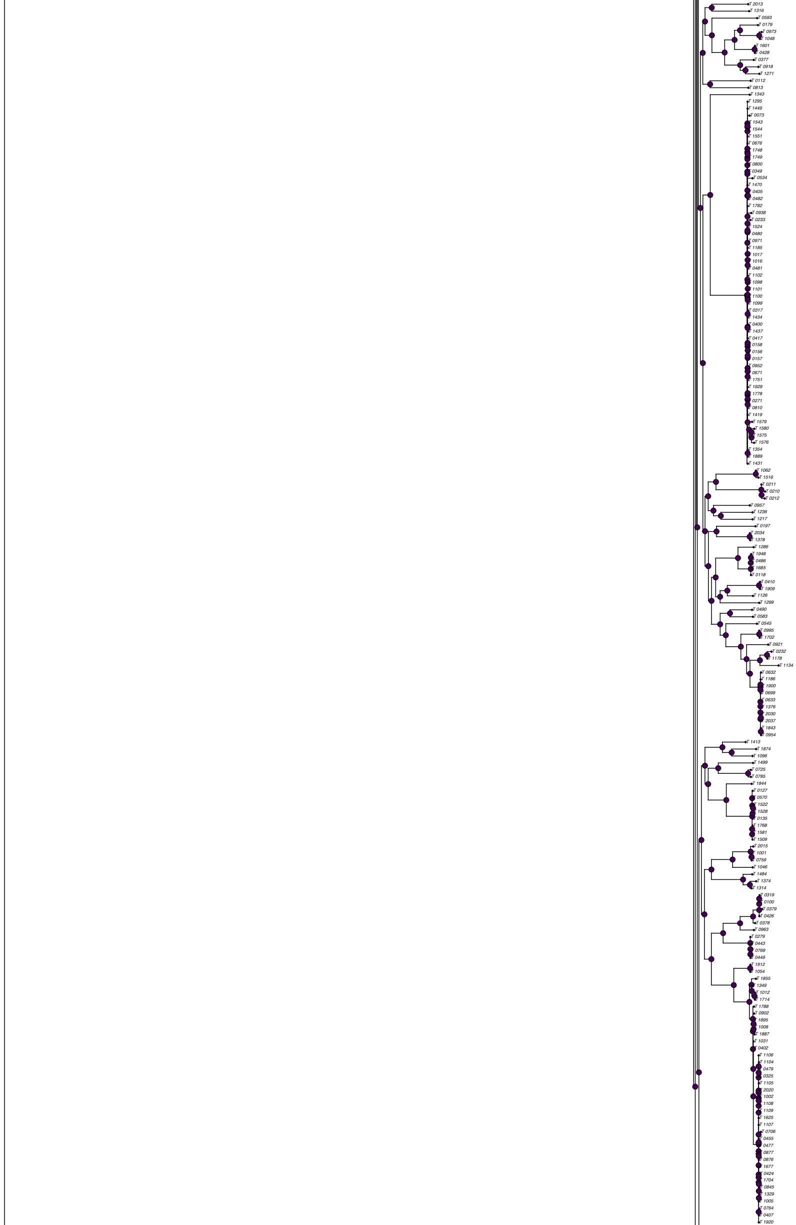
T 2013
T 1316
T 0593
T 0179
T 0973
T 1048
T 1601
T 0428
T 0377
T 0918
T 1271
T 0112
T 0813
T 1343
T 1295
T 1449
T 0073
T 1543
T 1544
T 1551
T 0676
T 1748
T 1749
T 0800
T 0349
T 0534
T 1470
T 0405
T 0482
T 1782
T 0938
T 0233
T 1524
T 0480
T 0971
T 1185
T 1017
T 1016
T 0481
T 1102
T 1098
T 1101
T 1100
T 1099
T 0317
T 1434
T 0400
T 1437
T 0417
T 0158
T 0156
T 0157
T 0952
T 0671
T 1751
T 1929
T 1778
T 0271
T 0810
T 1419
T 1579
T 1580
T 1575
T 1576
T 1354
T 1889
T 1431
T 1062
T 1516
T 0211
T 0210
T 0212
T 0957
T 1236
T 1217
T 0197
T 2034
T 1378
T 1286
T 1948
T 0486
T 1685
T 0118
T 0410
T 1909
T 1126
T 1299
T 0490
T 0583
T 0545
T 0995
T 1702
T 0921
T 0232
T 1178
T 1134
T 0632
T 1186
T 1900
T 0699
T 0633
T 1376
T 2030
T 2037
T 1843
T 0954
T 1413
T 1874
T 1096
T 1499
T 0725
T 0785
T 1844
T 0127
T 0570
T 1522
T 1528
T 0135
T 1768
T 1581
T 1509
T 2015
T 1001
T 0759
T 1046
T 1484
T 1374
T 1314
T 0319
T 0100
T 0379
T 0426
T 0378
T 0963
T 0279
T 0443
T 0769
T 0449
T 1912
T 1054
T 1855
T 1349
T 1012
T 1714
T 1788
T 0902
T 1895
T 1008
T 1887
T 1031
T 0402
T 1106
T 1104
T 0479
T 0325
T 1105
T 2020
T 1002
T 1108
T 1109
T 1625
T 1107
T 0706
T 0455
T 0477
T 0877
T 0876
T 1677
T 0424
T 1704
T 0845
T 1329
T 1005
T 0764
T 0407
T 1920

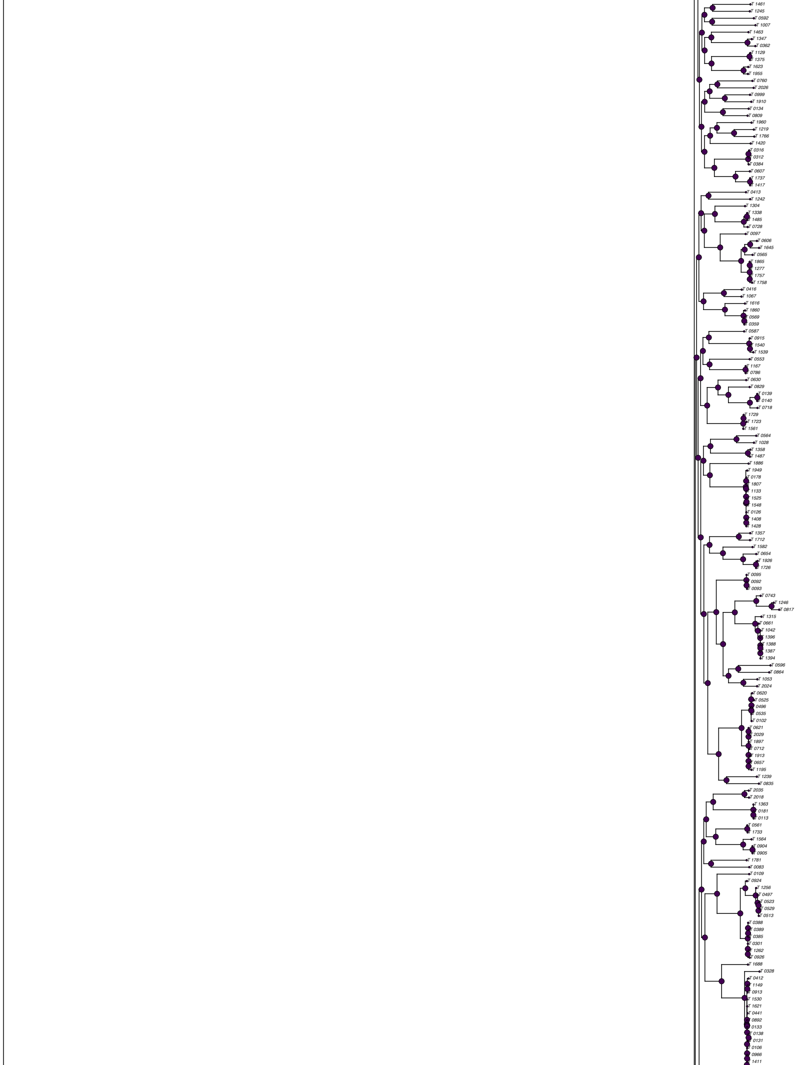
T 1461
T 1245
T 0592
T 1007
T 1463
T 1347
T 0362
T 1129
T 1375
T 1623
T 1955
T 0760
T 2026
T 0999
T 1910
T 0134
T 0809
T 1960
T 1219
T 1766
T 1420
T 0316
T 0312
T 0384
T 0607
T 1737
T 1417
T 0413
T 1242
T 1304
T 1338
T 1485
T 0728
T 0097
T 0606
T 1645
T 0565
T 1865
T 1277
T 1757
T 1758
T 0416
T 1067
T 1616
T 1860
T 0569
T 0359
T 0587
T 0915
T 1540
T 1539
T 0553
T 1167
T 0786
T 0630
T 0829
T 0139
T 0140
T 0718
T 1729
T 1723
T 1561
T 0564
T 1028
T 1358
T 1487
T 1886
T 1949
T 0178
1807
1133
1525
1548
T 0126
1408
T 1428
T 1357
T 1712
T 1582
T 0654
T 1926
T 1726
T 0095
0092
T 0093
T 0743
T 1246
T 0817
T 1315
T 0661
T 1042
1396
T 1388
1387
T 1394
T 0596
T 0864
T 1053
T 2024
T 0620
T 0525
0496
T 0535
T 0102
T 0621
2029
T 1897
T 0712
T 1913
T 0657
T 1195
T 1239
T 0835
T 2035
T 2018
T 1363
0181
T 0113
T 0561
T 1733
T 1564
T 0904
T 0905
T 1781
T 0083
T 0109
T 0924
T 1256
T 0497
T 0523
T 0529
T 0513
T 0388
T 0389
T 0385
T 0301
T 1262
T 0926
T 1688
T 0328
T 0412
1149
T 0913
T 1530
T 1621
T 0441
0892
0133
T 0138
0131
0106
0966
1411

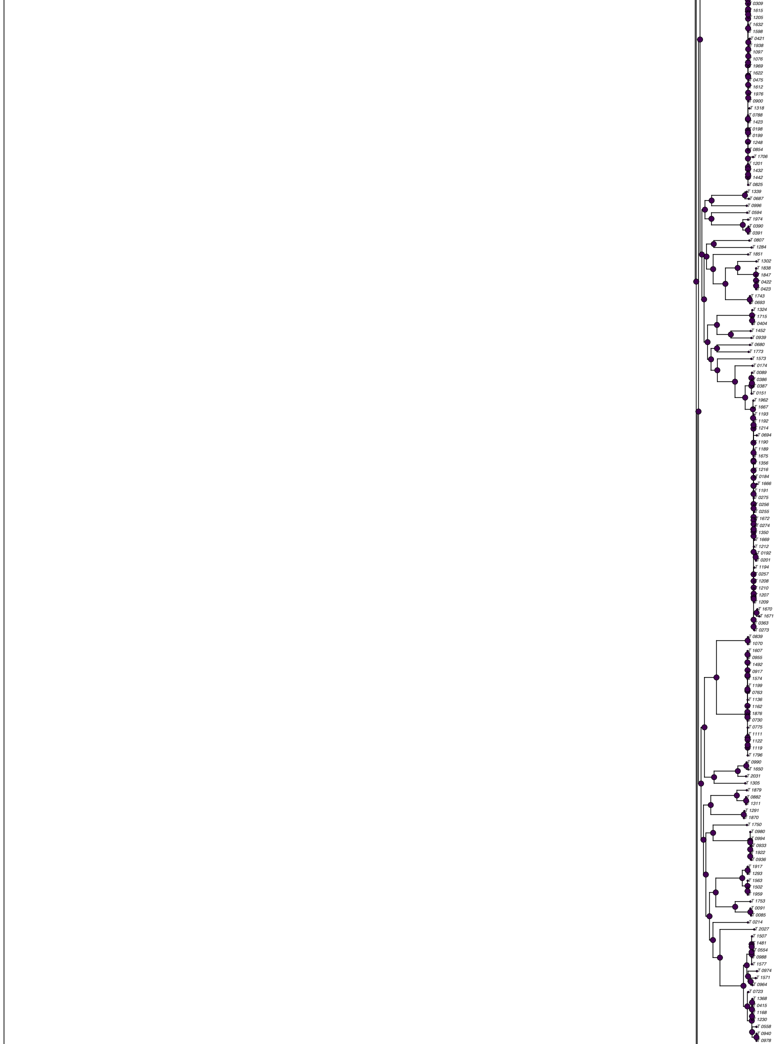
T 0309
T 1615
T 1205
T 1632
T 1598
T 0421
T 1938
T 1097
T 1076
T 1969
T 1622
T 0475
T 1612
T 1976
T 0900
T 1318
T 0788
T 1423
T 0198
T 0199
T 1248
T 0854
T 1706
T 1201
T 1432
T 1442
T 0825
T 1339
T 0687
T 0996
T 0594
T 1974
T 0390
T 0391
T 0807
T 1284
T 1851
T 1302
T 1838
T 1847
T 0422
T 0423
T 1743
T 0693
T 1324
T 1715
T 0404
T 1452
T 0939
T 0680
T 1773
T 1573
T 0174
T 0089
T 0386
T 0387
T 0151
T 1962
T 1667
T 1193
T 1192
T 1214
T 0694
T 1190
T 1189
T 1675
T 1356
T 1216
T 0184
T 1666
T 1191
T 0275
T 0256
T 0255
T 1672
T 0274
T 1350
T 1669
T 1212
T 0192
T 0201
T 1194
T 0257
T 1208
T 1210
T 1207
T 1209
T 1670
T 1671
T 0363
T 0273
T 0839
T 1070
T 1607
T 0955
T 1492
T 0917
T 1574
T 1199
T 0763
T 1136
T 1162
T 1876
T 0730
T 0775
T 1111
T 1122
T 1119
T 1796
T 0990
T 1650
T 2031
T 1305
T 1879
T 0882
T 1311
T 1291
T 1870
T 1750
T 0980
T 0994
T 0933
T 1922
T 0936
T 1917
T 1293
T 1563
T 1502
T 1959
T 1753
T 0091
T 0085
T 0214
T 2027
T 1507
T 1481
T 0554
T 0988
T 1577
T 0974
T 1571
T 0964
T 0723
T 1368
T 0415
T 1168
T 1230
T 0558
T 0940
T 0978

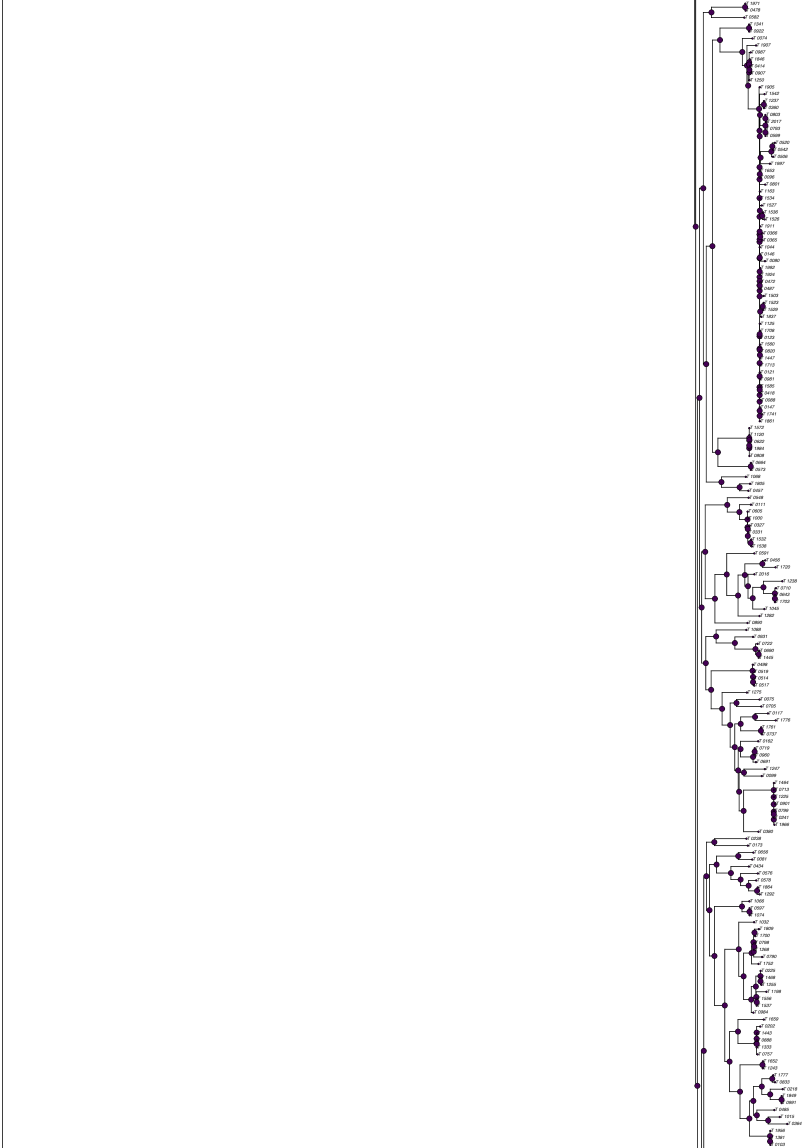
T 1971
T 0478
T 0582
T 1341
T 0922
T 0074
T 1907
T 0987
T 1846
T 0414
T 0907
T 1250
T 1905
T 1542
T 1237
T 0360
T 0803
T 2017
T 0793
T 0599
T 0520
T 0542
T 0506
T 1997
T 1653
T 0096
T 0801
T 1163
T 1534
T 1527
T 1536
T 1526
T 1911
T 0366
T 0365
T 1044
T 0146
T 0080
T 1992
T 1924
T 0472
T 0487
T 1503
T 1523
T 1529
T 1837
T 1125
T 1708
T 0123
T 1560
T 0820
T 1447
T 1713
T 0121
T 0981
T 1585
T 0418
T 0088
T 0147
T 1741
T 1861
T 1572
T 1120
T 0622
T 1984
T 0808
T 0664
T 0573
T 1068
T 1805
T 0457
T 0548
T 0111
T 0605
T 1000
T 0327
T 0331
T 1532
T 1538
T 0591
T 0456
T 1720
T 2016
T 1238
T 0710
T 0643
T 1703
T 1045
T 1282
T 0890
T 1088
T 0931
T 0722
T 0690
T 1445
T 0498
T 0519
T 0514
T 0517
T 1275
T 0075
T 0705
T 0117
T 1776
T 1761
T 0737
T 0162
T 0719
T 0960
T 0691
T 1247
T 0099
T 1464
T 0713
T 1225
T 0901
T 0799
T 0241
T 1966
T 0380
T 0238
T 0173
T 0656
T 0081
T 0434
T 0576
T 0578
T 1864
T 1292
T 1066
T 0597
T 1074
T 1032
T 1809
T 1700
T 0798
T 1268
T 0790
T 1752
T 0225
T 1468
T 1255
T 1198
T 1556
T 1537
T 0984
T 1659
T 0202
T 1443
T 0888
T 1333
T 0757
T 1652
T 1243
T 1777
T 0833
T 0218
T 1849
T 0991
T 0485
T 1015
T 0364
T 1956
T 1381
T 0103

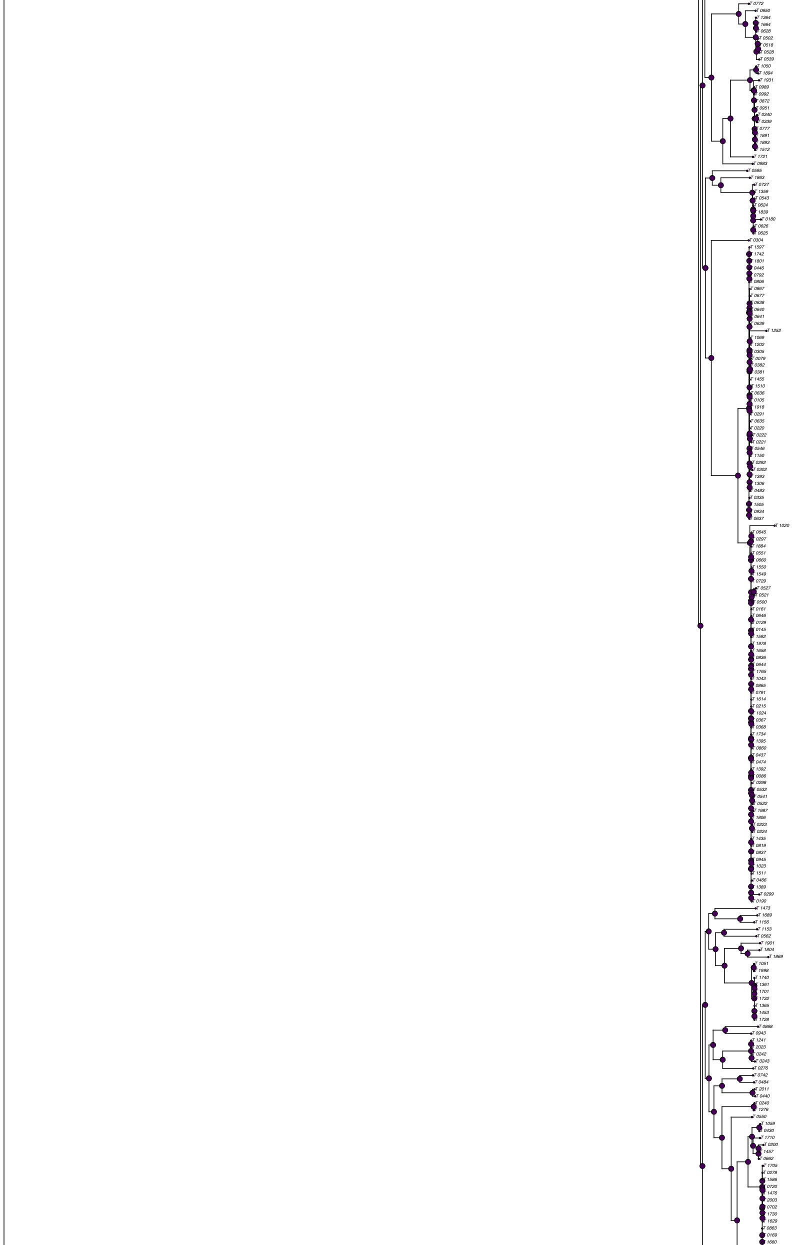

T 0772
T 0650
T 1364
1664
0628
T 0502
0518
T 0528
T 0539
T 1050
T 1894
T 1931
T 0989
0992
0872
T 0951
T 0340
T 0339
0777
1891
1893
T 1512
T 1721
T 0983
T 0595
T 1863
T 0727
T 1359
T 0543
T 0624
1839
T 0180
T 0626
T 0625
T 0304
T 1597
1742
1801
0446
0792
T 0806
T 0867
T 0677
0638
0640
0641
T 0639
T 1252
T 1069
T 1202
0305
T 0079
0382
0381
T 1455
1510
T 0636
0105
1918
T 0291
T 0635
T 0220
T 0222
T 0221
0546
T 1150
T 0292
T 0302
1393
1306
T 0483
T 0335
1505
0934
T 0637
T 1020
T 0645
0297
1884
T 0551
0660
T 1550
1549
0729
T 0527
T 0521
0500
T 0161
0646
0129
T 0145
T 1592
T 1978
1658
0836
0644
1765
T 1043
0865
T 0791
T 1614
T 0215
1024
0367
T 0368
T 1734
1395
T 0860
0437
T 0474
T 1392
0086
T 0298
T 0532
T 0541
T 0522
T 1987
1806
T 0223
T 0224
T 1435
T 0819
T 0837
0945
1023
T 1511
T 0466
1389
T 0299
T 0190
T 1473
T 1689
T 1156
T 1153
T 0562
T 1901
T 1804
T 1869
T 1051
T 1998
T 1740
1361
1701
1732
T 1365
1453
T 1728
T 0868
T 0943
T 1241
2023
0242
T 0243
T 0276
T 0742
T 0484
T 2011
T 0440
T 0240
T 1276
T 0550
T 1059
0430
T 1710
T 0200
1457
T 0662
T 1705
T 0278
1586
0720
T 1476
2003
0702
1730
T 1629
T 0863
0169
1660

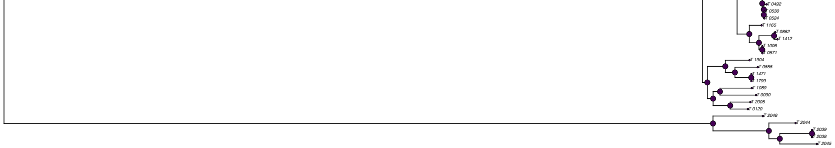
T 0492
T 0530
T 0524
T 1165
T 0862
T 1412
T 1006
T 0571
T 1904
T 0555
T 1471
T 1799
T 1089
T 0090
T 2005
T 0120
T 2048
T 2044
T 2039
T 2038
T 2045

Effector gene: tse7

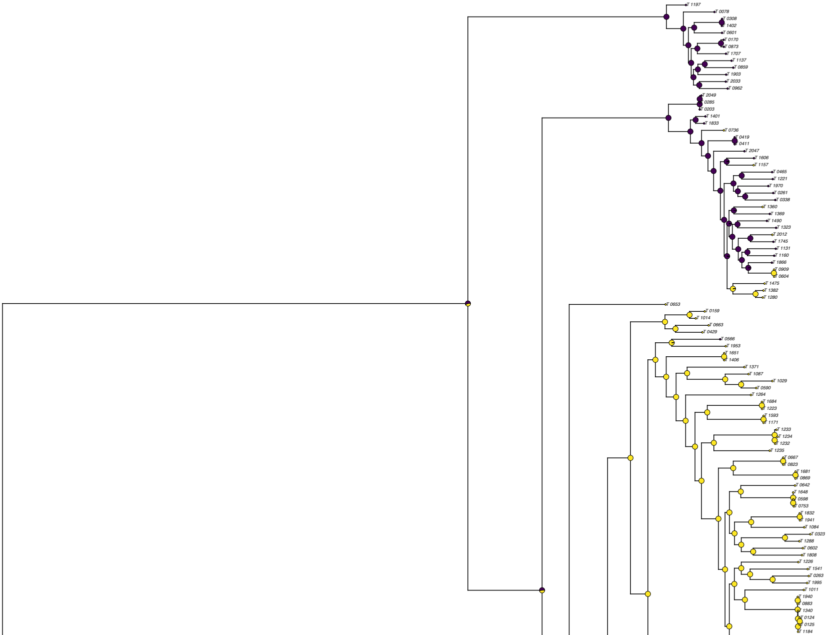

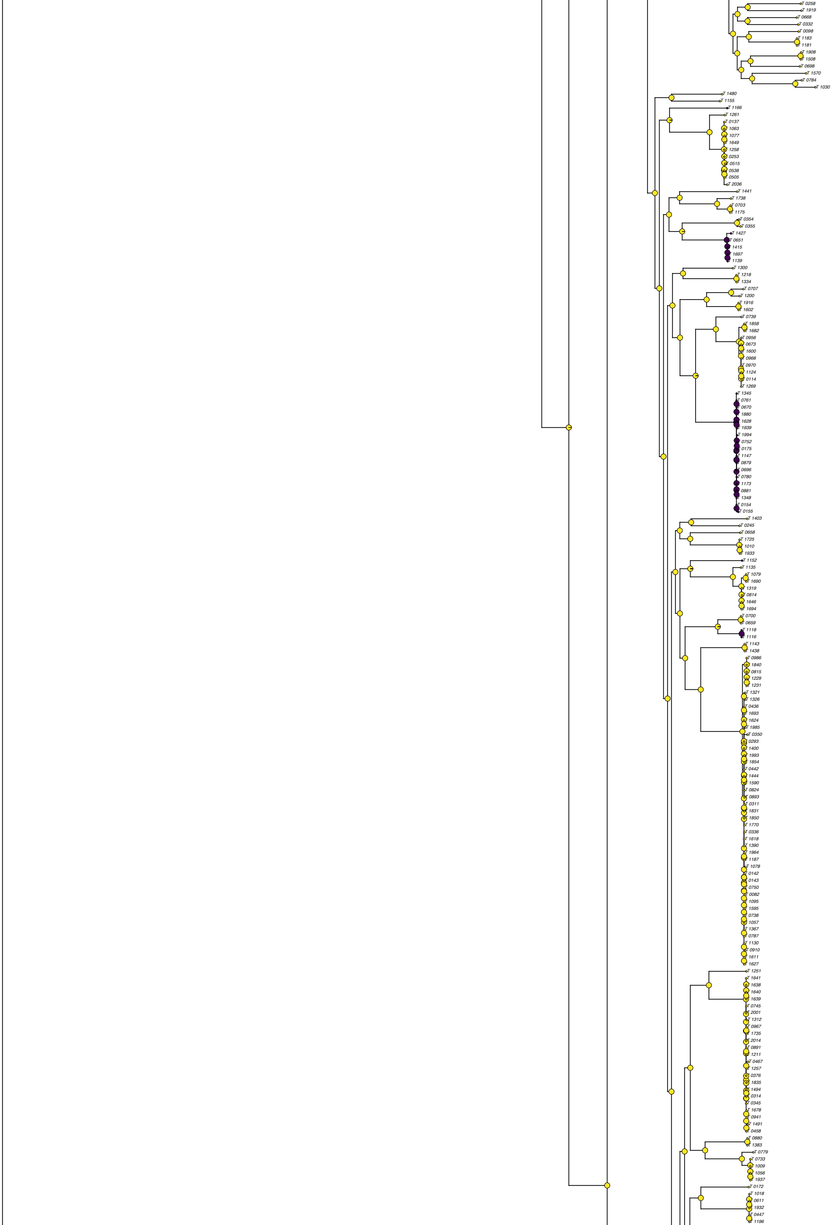

T 0258
T 1919
T 0668
T 0332
T 0098
T 1183
1181
T 1908
1508
T 0698
T 1570
T 0784
T 1030
T 1480
T 1155
T 1166
T 1261
T 0137
1063
1077
1649
1258
0253
0515
0538
0505
T 2036
T 1441
T 1738
T 0703
T 1175
T 0354
T 0355
T 1427
T 0651
1415
1697
1139
T 1300
T 1218
1334
T 0707
T 1200
T 1916
1602
T 0739
T 1858
1682
T 0956
0673
1600
0968
T 0970
1124
0114
T 1269
T 1345
T 0761
0670
1880
1628
1939
T 1994
0752
0175
T 1147
0879
T 0696
T 0780
1173
0881
1348
0154
T 0155
T 1403
T 0245
T 0658
T 1725
1010
1933
T 1152
T 1135
T 1079
1690
1319
0814
1646
1694
T 0700
0659
T 1118
1116
T 1143
1438
T 0986
1840
0815
1229
1231
T 1321
1326
T 0436
1693
1624
T 1985
T 0350
0293
1400
1993
1854
T 0442
1444
1590
T 0824
0893
T 0311
1831
1850
T 1770
T 0336
T 1618
1390
1964
1187
T 1078
0142
0143
0750
0082
1095
1595
0738
1057
T 1367
0787
T 1130
T 0910
1611
1627
T 1251
T 1641
1638
1640
1639
T 0745
T 2001
T 1312
T 0967
1735
2014
T 0891
1211
T 0467
1257
0376
1835
1494
0314
0345
T 1678
0941
T 1491
0458
T 0880
1383
T 0779
T 0733
1009
1056
1937
T 0172
T 1018
0611
1932
T 0447
1196

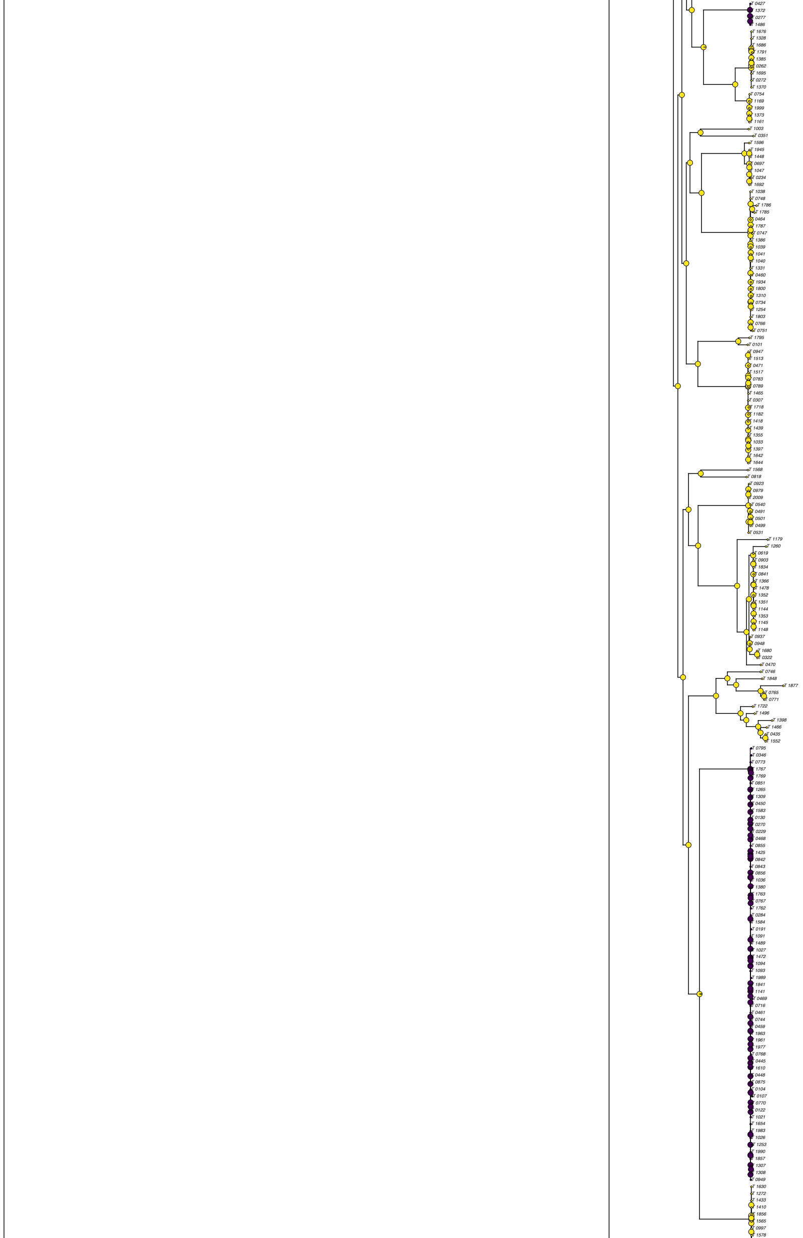

T 0427
T 1372
T 0277
T 1486
T 1676
T 1328
T 1686
T 1791
T 1385
T 0262
T 1695
T 0272
T 1370
T 0754
T 1169
T 1999
T 1373
T 1161
T 1003
T 0351
T 1596
T 1945
T 1448
T 0697
T 1047
T 0234
T 1692
T 1038
T 0748
T 1786
T 1785
T 0464
T 1787
T 0747
T 1386
T 1039
T 1041
T 1040
T 1331
T 0460
T 1934
T 1800
T 1310
T 0734
T 1254
T 1803
T 0766
T 0751
T 1795
T 0101
T 0947
T 1513
T 0471
T 1517
T 0783
T 0789
T 1465
T 0307
T 1718
T 1182
T 1418
T 1439
T 1355
T 1033
T 1397
T 1642
T 1644
T 1568
T 0818
T 0923
T 0979
T 2009
T 0540
T 0491
T 0501
T 0499
T 0531
T 1179
T 1260
T 0619
T 0903
T 1834
T 0841
T 1366
T 1478
T 1352
T 1351
T 1144
T 1353
T 1145
T 1148
T 0937
T 0948
T 1680
T 0322
T 0470
T 0746
T 1848
T 1877
T 0765
T 0771
T 1722
T 1496
T 1398
T 1466
T 0435
T 1552
T 0795
T 0346
T 0773
T 1767
T 1769
T 0851
T 1265
T 1309
T 0450
T 1583
T 0130
T 0270
T 0229
T 0468
T 0855
T 1425
T 0842
T 0843
T 0856
T 1036
T 1380
T 1763
T 0767
T 1762
T 0284
T 1584
T 0191
T 1091
T 1489
T 1027
T 1472
T 1094
T 1093
T 1989
T 1841
T 1141
T 0469
T 0716
T 0461
T 0744
T 0459
T 1963
T 1961
T 1977
T 0768
T 0445
T 1610
T 0448
T 0875
T 0104
T 0107
T 0770
T 0122
T 1021
T 1654
T 1983
T 1026
T 1253
T 1990
T 1857
T 1307
T 1308
T 0949
T 1630
T 1272
T 1433
T 1410
T 1856
T 1565
T 0997
T 1578

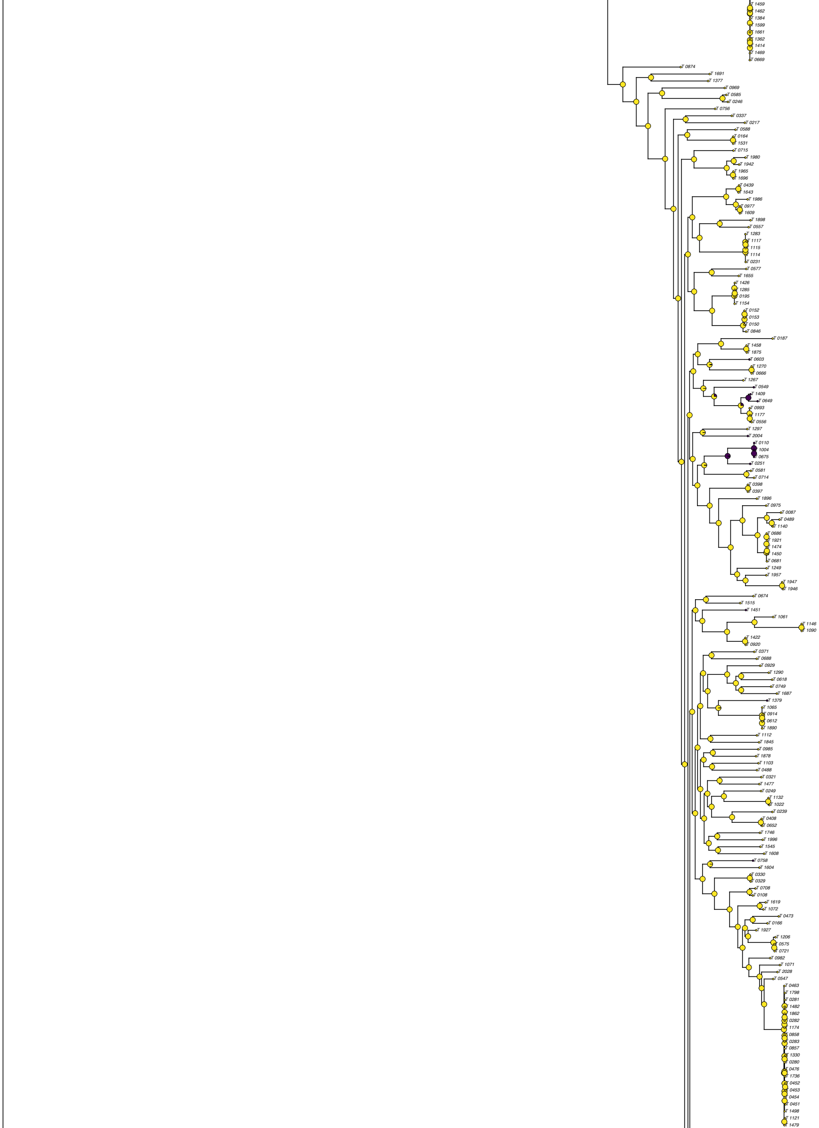
T 1459
T 1462
T 1384
T 1599
T 1661
T 1362
T 1414
T 1469
T 0669
T 0874
T 1691
T 1377
T 0969
T 0585
T 0246
T 0756
T 0337
T 0217
T 0588
T 0164
T 1531
T 0715
T 1980
T 1942
T 1965
T 1696
T 0439
T 1643
T 1986
T 0977
T 1609
T 1898
T 0557
T 1283
T 1117
T 1115
T 1114
T 0231
T 0577
T 1655
T 1426
T 1285
T 0195
T 1154
T 0152
T 0153
T 0150
T 0846
T 0187
T 1458
T 1875
T 0603
T 1270
T 0666
T 1267
T 0549
T 1409
T 0649
T 0993
T 1177
T 0556
T 1297
T 2004
T 0110
T 1004
T 0675
T 0251
T 0581
T 0714
T 0398
T 0397
T 1896
T 0975
T 0087
T 0489
T 1140
T 0686
T 1921
T 1474
T 1450
T 0681
T 1249
T 1957
T 1947
T 1946
T 0674
T 1515
T 1451
T 1061
T 1146
T 1090
T 1422
T 0920
T 0371
T 0688
T 0929
T 1290
T 0618
T 0749
T 1687
T 1379
T 1065
T 0914
T 0612
T 1890
T 1112
T 1845
T 0985
T 1878
T 1103
T 0488
T 0321
T 1477
T 0249
T 1132
T 1022
T 0239
T 0408
T 0652
T 1746
T 1996
T 1545
T 1608
T 0758
T 1604
T 0330
T 0329
T 0708
T 0108
T 1619
T 1072
T 0473
T 0166
T 1927
T 1206
T 0575
T 0721
T 0982
T 1071
T 2028
T 0547
T 0463
T 1798
T 0281
T 1482
T 1862
T 0282
T 1174
T 0858
T 0283
T 0857
T 1330
T 0280
T 0476
T 1736
T 0452
T 0453
T 0454
T 0451
T 1498
T 1121
T 1479

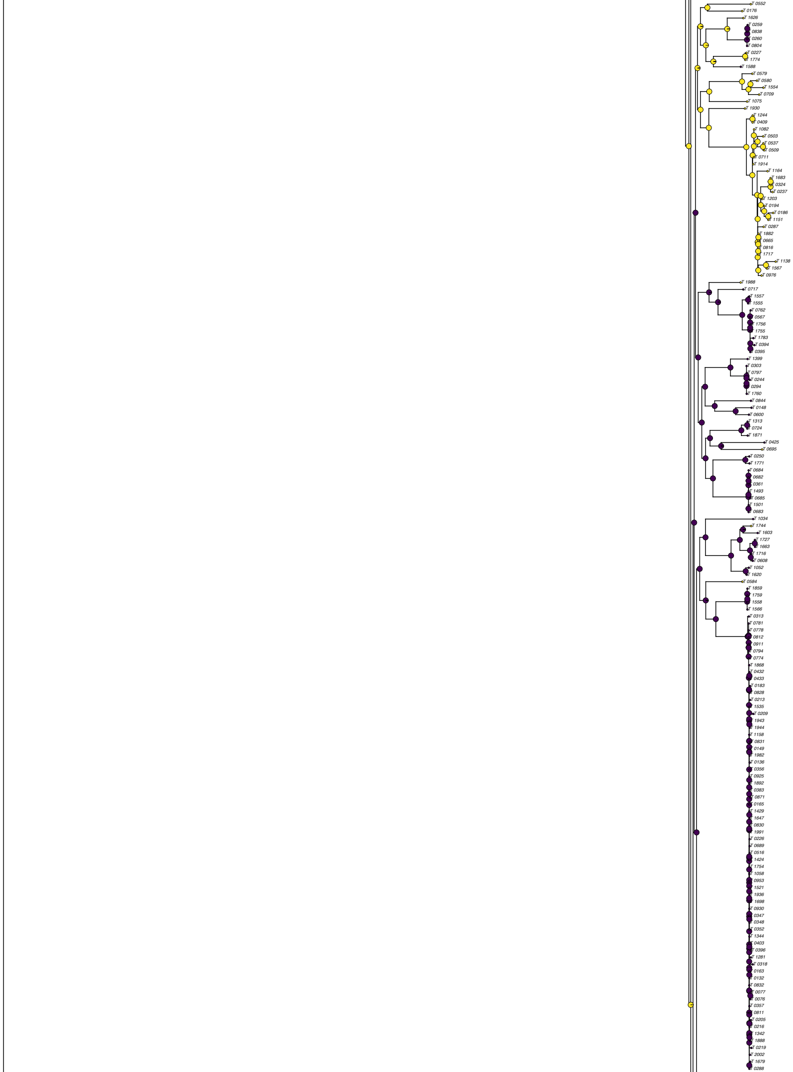
T 0552
T 0176
T 1626
T 0259
0838
0260
T 0804
T 0227
T 1774
T 1588
T 0579
T 0580
T 1554
T 0709
T 1075
T 1930
T 1244
T 0409
T 1082
T 0503
T 0537
T 0509
T 0711
T 1914
T 1164
T 1683
0324
T 0237
T 1203
T 0194
T 0186
T 1151
T 0287
T 1882
0665
T 0816
T 1717
T 1138
T 1567
T 0976
T 1988
T 0717
T 1557
T 1555
T 0762
0567
T 1756
1755
T 1783
T 0394
T 0395
T 1399
T 0303
T 0797
T 0244
0294
T 1760
T 0844
T 0148
T 0600
T 1313
T 0724
T 1871
T 0425
T 0695
T 0250
T 1771
T 0684
0682
0361
T 1493
T 0685
T 1501
T 0683
T 1034
T 1744
T 1603
T 1727
T 1663
T 1716
T 0608
T 1052
T 1620
T 0584
T 1859
1759
1558
T 1566
T 0313
T 0781
T 0778
0812
0911
T 0794
0774
T 1868
T 0432
0433
T 0183
T 0828
T 0213
1535
T 0209
1943
T 1944
T 1158
0831
0149
T 1982
T 0136
0356
T 0925
T 1892
0383
T 0871
0165
T 1429
T 1647
T 0830
1991
T 0226
T 0689
T 0516
1424
T 1754
T 1058
0953
1521
T 1936
1698
T 0930
0347
T 0348
T 0352
T 1344
T 0403
T 0396
T 1281
T 0318
0163
T 0132
T 0832
T 0077
T 0076
T 0357
0811
T 0205
T 0216
T 1342
T 1888
T 0219
T 2002
T 1679
T 0288

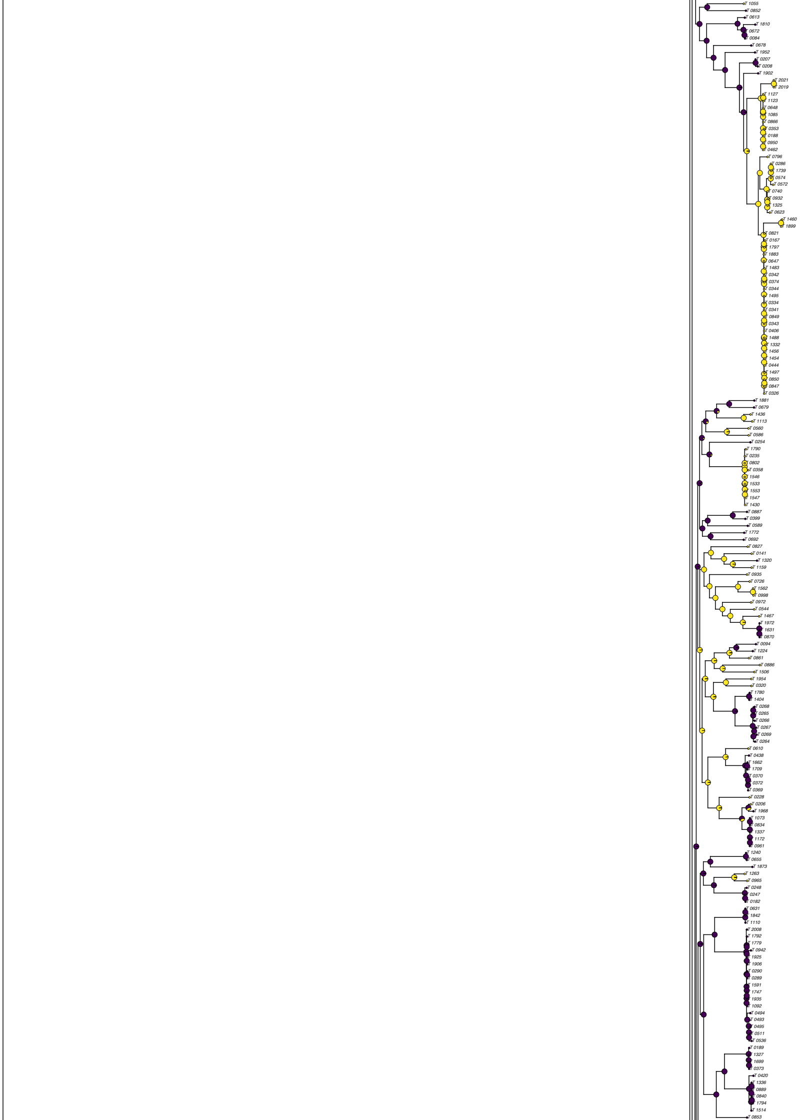

T 1055
T 0852
T 0613
T 1810
T 0672
T 0084
T 0678
T 1952
T 0207
T 0208
T 1902
T 2021
T 2019
T 1127
T 1123
T 0648
T 1085
T 0866
T 0353
T 0188
T 0950
T 0462
T 0796
T 0286
T 1739
T 0574
T 0572
T 0740
T 0932
T 1325
T 0623
T 1460
T 1899
T 0821
T 0167
T 1797
T 1883
T 0647
T 1483
T 0342
T 0374
T 0344
T 1495
T 0334
T 0341
T 0849
T 0343
T 0406
T 1488
T 1332
T 1456
T 1454
T 0444
T 1497
T 0850
T 0847
T 0326
T 1881
T 0679
T 1436
T 1113
T 0560
T 0586
T 0254
T 1790
T 0235
T 0802
T 0358
T 1546
T 1533
T 1553
T 1547
T 1430
T 0887
T 0399
T 0589
T 1772
T 0692
T 0827
T 0141
T 1320
T 1159
T 0935
T 0726
T 1562
T 0998
T 0972
T 0544
T 1467
T 1972
T 1631
T 0870
T 0094
T 1224
T 0861
T 0886
T 1506
T 1954
T 0320
T 1780
T 1404
T 0268
T 0265
T 0266
T 0267
T 0269
T 0264
T 0610
T 0438
T 1662
T 1709
T 0370
T 0372
T 0369
T 0228
T 0206
T 1968
T 1073
T 0834
T 1337
T 1172
T 0961
T 1240
T 0655
T 1873
T 1263
T 0965
T 0248
T 0247
T 0182
T 0631
T 1842
T 1110
T 2008
T 1792
T 1779
T 0942
T 1925
T 1906
T 0290
T 0289
T 1591
T 1747
T 1935
T 1092
T 0494
T 0493
T 0495
T 0511
T 0536
T 0189
T 1327
T 1699
T 0373
T 0420
T 1336
T 0889
T 0840
T 1794
T 1514
T 0853

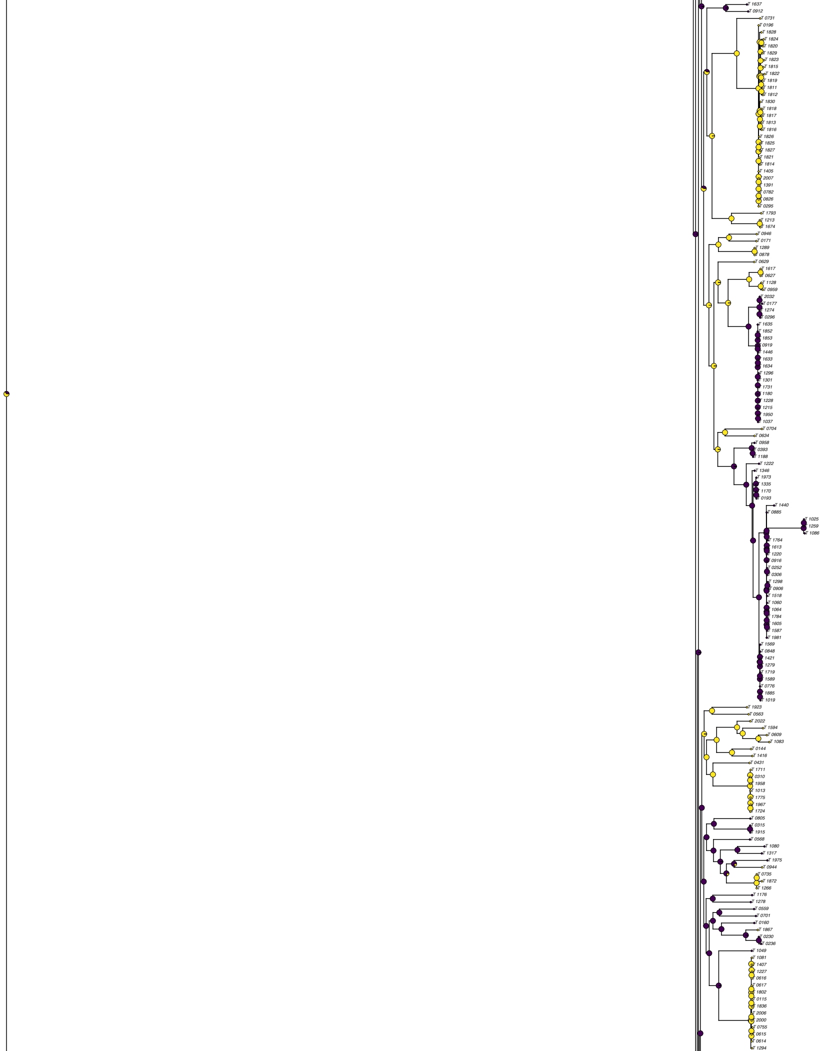
T 1637
T 0912
T 0731
T 0196
T 1828
T 1824
T 1820
T 1829
T 1823
T 1815
T 1822
T 1819
T 1811
T 1812
T 1830
T 1818
T 1817
T 1813
T 1816
T 1826
T 1825
T 1827
T 1821
T 1814
T 1405
T 2007
T 1391
T 0782
T 0826
T 0295
T 1793
T 1213
T 1674
T 0946
T 0171
T 1289
T 0878
T 0629
T 1617
T 0627
T 1128
T 0959
T 2032
T 0177
T 1274
T 0296
T 1635
T 1852
T 1853
T 0919
T 1446
T 1633
T 1634
T 1296
T 1301
T 1731
T 1180
T 1228
T 1215
T 1950
T 1037
T 0704
T 0634
T 0958
T 0393
T 1188
T 1222
T 1346
T 1973
T 1335
T 1170
T 0193
T 1440
T 0885
T 1025
T 1259
T 1086
T 1764
T 1613
T 1220
T 0916
T 0252
T 0306
T 1298
T 0906
T 1518
T 1060
T 1064
T 1784
T 1605
T 1587
T 1981
T 1569
T 0848
T 1421
T 1279
T 1719
T 1589
T 0776
T 1885
T 1019
T 1923
T 0563
T 2022
T 1594
T 0609
T 1083
T 0144
T 1416
T 0431
T 1711
T 0310
T 1958
T 1013
T 1775
T 1967
T 1724
T 0805
T 0315
T 1915
T 0568
T 1080
T 1317
T 1975
T 0944
T 0735
T 1872
T 1266
T 1176
T 1278
T 0559
T 0701
T 0160
T 1867
T 0230
T 0236
T 1049
T 1081
T 1407
T 1227
T 0616
T 0617
T 1802
T 0115
T 1836
T 2006
T 2000
T 0755
T 0615
T 0614
T 1294

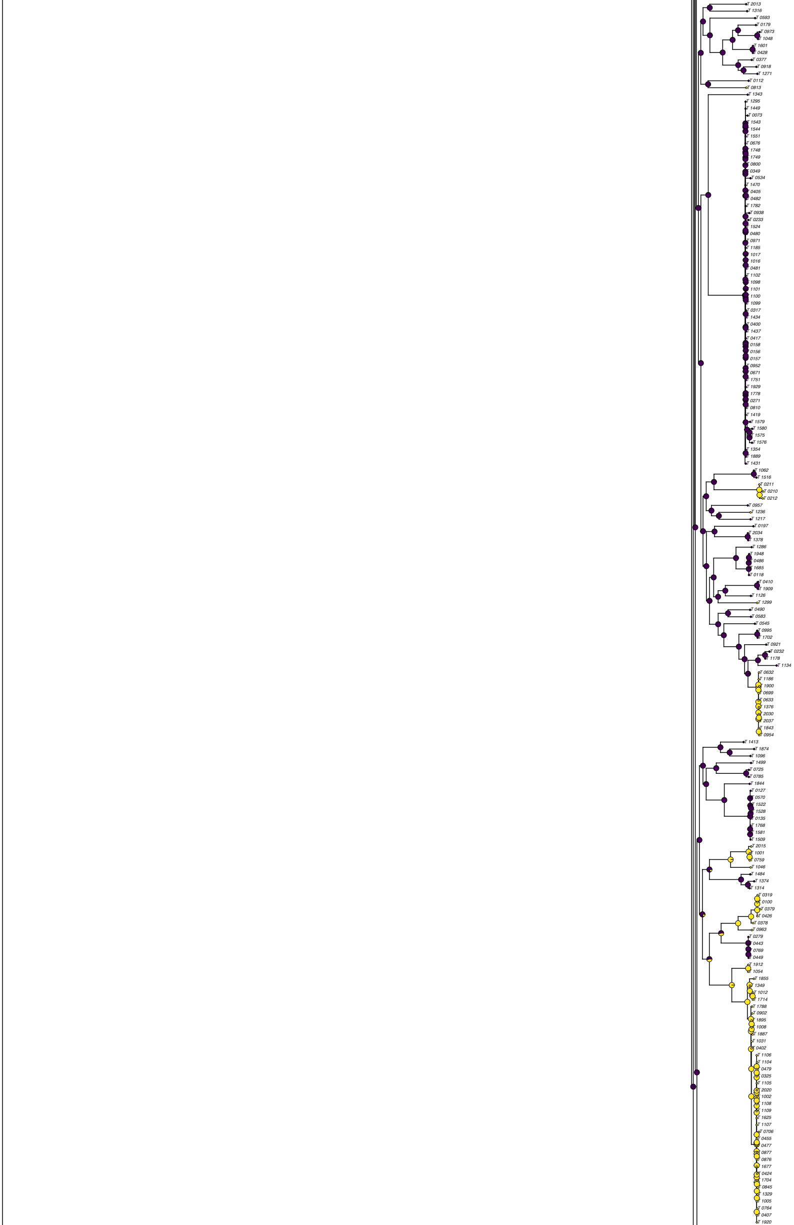
T 2013
T 1316
T 0593
T 0179
T 0973
T 1048
T 1601
T 0428
T 0377
T 0918
T 1271
T 0112
T 0813
T 1343
T 1295
T 1449
T 0073
T 1543
T 1544
T 1551
T 0676
T 1748
T 1749
T 0800
T 0349
T 0534
T 1470
T 0405
T 0482
T 1782
T 0938
T 0233
T 1524
T 0480
T 0971
T 1185
T 1017
T 1016
T 0481
T 1102
T 1098
T 1101
T 1100
T 1099
T 0317
T 1434
T 0400
T 1437
T 0417
T 0158
T 0156
T 0157
T 0952
T 0671
T 1751
T 1929
T 1778
T 0271
T 0810
T 1419
T 1579
T 1580
T 1575
T 1576
T 1354
T 1889
T 1431
T 1062
T 1516
T 0211
T 0210
T 0212
T 0957
T 1236
T 1217
T 0197
T 2034
T 1378
T 1286
T 1948
T 0486
T 1685
T 0118
T 0410
T 1909
T 1126
T 1299
T 0490
T 0583
T 0545
T 0995
T 1702
T 0921
T 0232
T 1178
T 1134
T 0632
T 1186
T 1900
T 0699
T 0633
T 1376
T 2030
T 2037
T 1843
T 0954
T 1413
T 1874
T 1096
T 1499
T 0725
T 0785
T 1844
T 0127
T 0570
T 1522
T 1528
T 0135
T 1768
T 1581
T 1509
T 2015
T 1001
T 0759
T 1046
T 1484
T 1374
T 1314
T 0319
T 0100
T 0379
T 0426
T 0378
T 0963
T 0279
T 0443
T 0769
T 0449
T 1912
T 1054
T 1855
T 1349
T 1012
T 1714
T 1788
T 0902
T 1895
T 1008
T 1887
T 1031
T 0402
T 1106
T 1104
T 0479
T 0325
T 1105
T 2020
T 1002
T 1108
T 1109
T 1625
T 1107
T 0706
T 0455
T 0477
T 0877
T 0876
T 1677
T 0424
T 1704
T 0845
T 1329
T 1005
T 0764
T 0407
T 1920

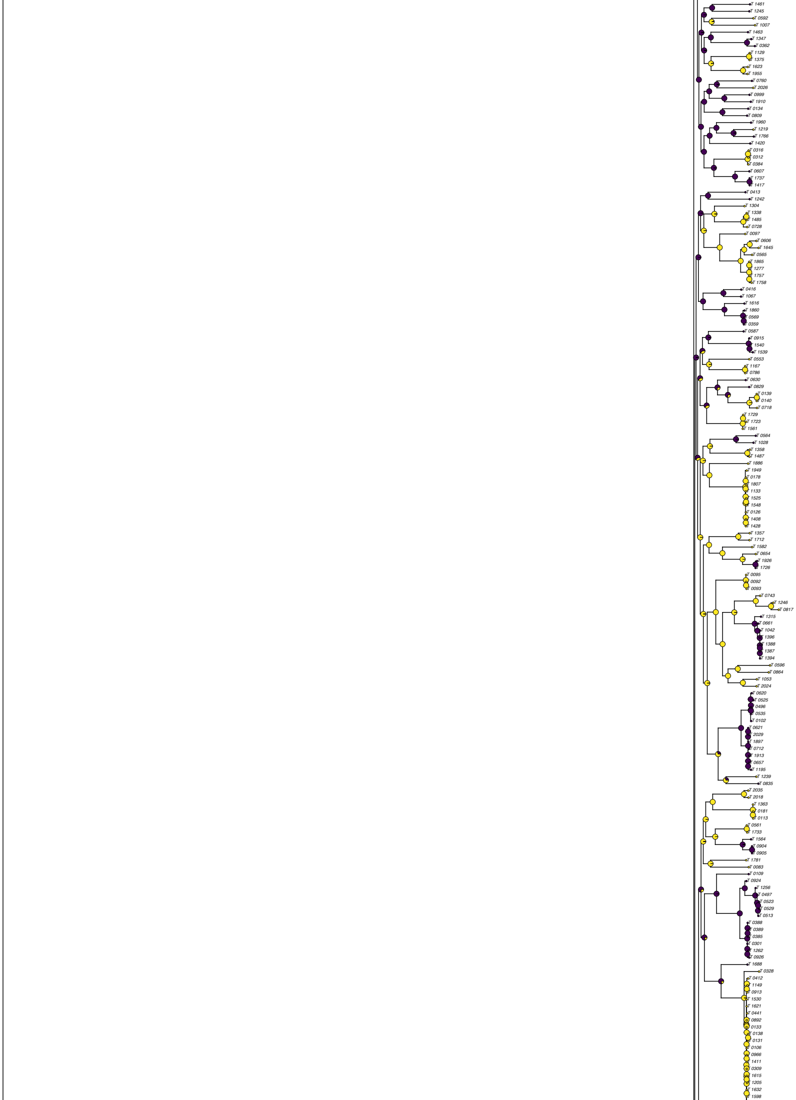
T 1461
T 1245
T 0592
T 1007
T 1463
T 1347
T 0362
T 1129
T 1375
T 1623
T 1955
T 0760
T 2026
T 0999
T 1910
T 0134
T 0809
T 1960
T 1219
T 1766
T 1420
T 0316
T 0312
T 0384
T 0607
T 1737
T 1417
T 0413
T 1242
T 1304
T 1338
T 1485
T 0728
T 0097
T 0606
T 1645
T 0565
T 1865
T 1277
T 1757
T 1758
T 0416
T 1067
T 1616
T 1860
T 0569
T 0359
T 0587
T 0915
T 1540
T 1539
T 0553
T 1167
T 0786
T 0630
T 0829
T 0139
T 0140
T 0718
T 1729
T 1723
T 1561
T 0564
T 1028
T 1358
T 1487
T 1886
T 1949
T 0178
T 1807
T 1133
T 1525
T 1548
T 0126
T 1408
T 1428
T 1357
T 1712
T 1582
T 0654
T 1926
T 1726
T 0095
T 0092
T 0093
T 0743
T 1246
T 0817
T 1315
T 0661
T 1042
T 1396
T 1388
T 1387
T 1394
T 0596
T 0864
T 1053
T 2024
T 0620
T 0525
T 0496
T 0535
T 0102
T 0621
T 2029
T 1897
T 0712
T 1913
T 0657
T 1195
T 1239
T 0835
T 2035
T 2018
T 1363
T 0181
T 0113
T 0561
T 1733
T 1564
T 0904
T 0905
T 1781
T 0083
T 0109
T 0924
T 1256
T 0497
T 0523
T 0529
T 0513
T 0388
T 0389
T 0385
T 0301
T 1262
T 0926
T 1688
T 0328
T 0412
T 1149
T 0913
T 1530
T 1621
T 0441
T 0892
T 0133
T 0138
T 0131
T 0106
T 0966
T 1411
T 0309
T 1615
T 1205
T 1632
T 1598

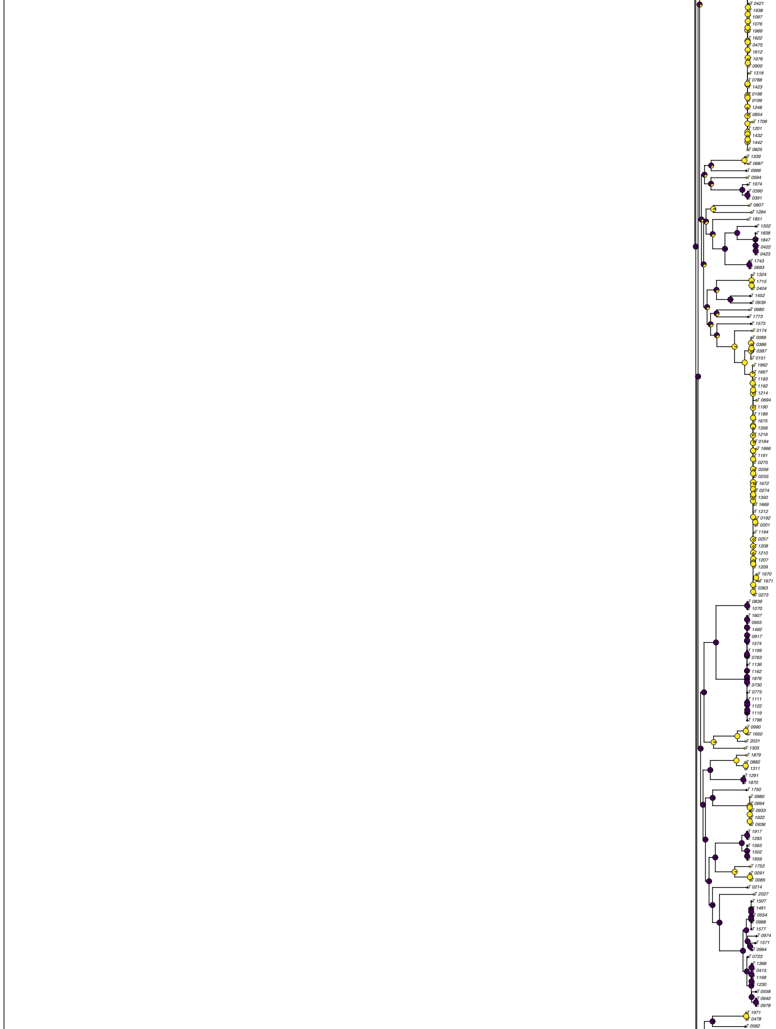
T 0421
T 1938
T 1097
T 1076
T 1969
T 1622
T 0475
T 1612
T 1976
T 0900
T 1318
T 0788
T 1423
T 0198
T 0199
T 1248
T 0854
T 1706
T 1201
T 1432
T 1442
T 0825
T 1339
T 0687
T 0996
T 0594
T 1974
T 0390
T 0391
T 0807
T 1284
T 1851
T 1302
T 1838
T 1847
T 0422
T 0423
T 1743
T 0693
T 1324
T 1715
T 0404
T 1452
T 0939
T 0680
T 1773
T 1573
T 0174
T 0089
T 0386
T 0387
T 0151
T 1962
T 1667
T 1193
T 1192
T 1214
T 0694
T 1190
T 1189
T 1675
T 1356
T 1216
T 0184
T 1666
T 1191
T 0275
T 0256
T 0255
T 1672
T 0274
T 1350
T 1669
T 1212
T 0192
T 0201
T 1194
T 0257
T 1208
T 1210
T 1207
T 1209
T 1670
T 1671
T 0363
T 0273
T 0839
T 1070
T 1607
T 0955
T 1492
T 0917
T 1574
T 1199
T 0763
T 1136
T 1162
T 1876
T 0730
T 0775
T 1111
T 1122
T 1119
T 1796
T 0990
T 1650
T 2031
T 1305
T 1879
T 0882
T 1311
T 1291
T 1870
T 1750
T 0980
T 0994
T 0933
T 1922
T 0936
T 1917
T 1293
T 1563
T 1502
T 1959
T 1753
T 0091
T 0085
T 0214
T 2027
T 1507
T 1481
T 0554
T 0988
T 1577
T 0974
T 1571
T 0964
T 0723
T 1368
T 0415
T 1168
T 1230
T 0558
T 0940
T 0978
T 1971
T 0478
T 0582

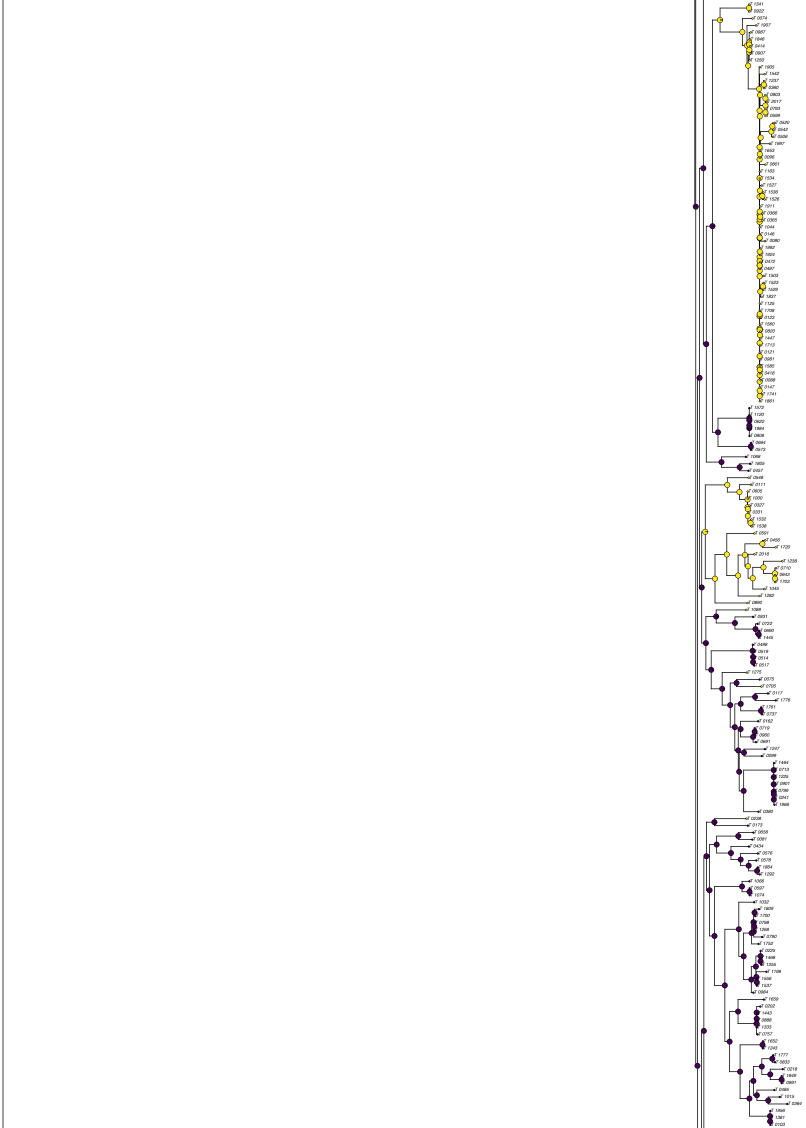
T 1341
0922
T 0074
T 1907
T 0987
T 1846
T 0414
T 0907
T 1250
T 1905
T 1542
T 1237
0360
T 0803
T 2017
0793
0599
T 0520
T 0542
T 0506
T 1997
T 1653
0096
T 0801
T 1163
1534
T 1527
T 1536
T 1526
T 1911
T 0366
T 0365
T 1044
T 0146
T 0080
T 1992
T 1924
T 0472
0487
T 1503
T 1523
T 1529
T 1837
T 1125
T 1708
0123
T 1560
0820
1447
T 1713
T 0121
0981
T 1585
0418
T 0088
T 0147
T 1741
T 1861
T 1572
T 1120
0622
1984
T 0808
T 0664
0573
T 1068
T 1805
T 0457
T 0548
T 0111
T 0605
T 1000
T 0327
0331
T 1532
T 1538
T 0591
T 0456
T 1720
T 2016
T 1238
T 0710
0643
T 1703
T 1045
T 1282
T 0890
T 1088
T 0931
T 0722
T 0690
1445
T 0498
T 0519
0514
T 0517
T 1275
T 0075
T 0705
T 0117
T 1776
T 1761
T 0737
T 0162
T 0719
T 0960
T 0691
T 1247
T 0099
T 1464
0713
1225
T 0901
T 0799
0241
T 1966
T 0380
T 0238
T 0173
T 0656
T 0081
T 0434
T 0576
T 0578
T 1864
T 1292
T 1066
T 0597
T 1074
T 1032
T 1809
T 1700
0798
T 1268
T 0790
T 1752
T 0225
T 1468
T 1255
T 1198
T 1556
T 1537
T 0984
T 1659
T 0202
1443
0888
T 1333
T 0757
T 1652
T 1243
T 1777
T 0833
T 0218
T 1849
T 0991
T 0485
T 1015
T 0364
T 1956
T 1381
T 0103

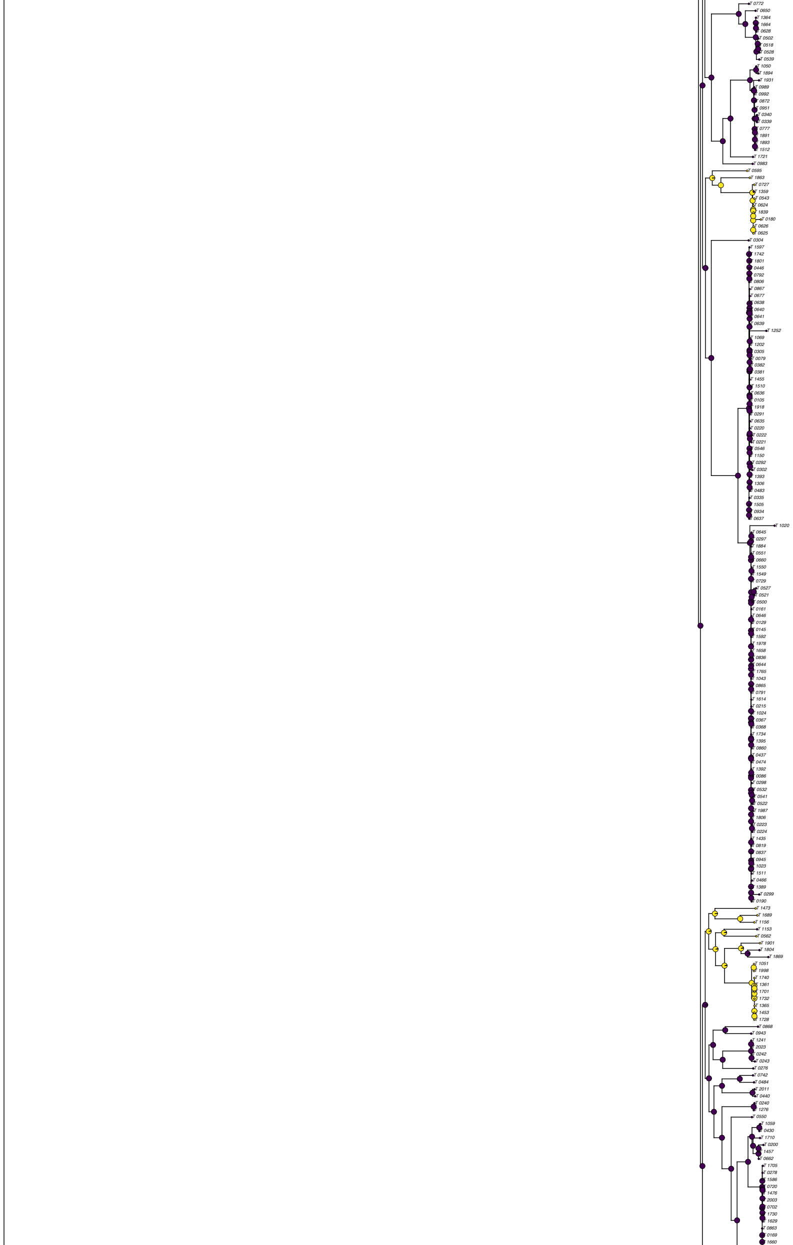

T 0772
T 0650
T 1364
T 1664
T 0628
T 0502
T 0518
T 0528
T 0539
T 1050
T 1894
T 1931
T 0989
T 0992
T 0872
T 0951
T 0340
T 0339
T 0777
T 1891
T 1893
T 1512
T 1721
T 0983
T 0595
T 1863
T 0727
T 1359
T 0543
T 0624
T 1839
T 0180
T 0626
T 0625
T 0304
T 1597
T 1742
T 1801
T 0446
T 0792
T 0806
T 0867
T 0677
T 0638
T 0640
T 0641
T 0639
T 1252
T 1069
T 1202
T 0305
T 0079
T 0382
T 0381
T 1455
T 1510
T 0636
T 0105
T 1918
T 0291
T 0635
T 0220
T 0222
T 0221
T 0546
T 1150
T 0292
T 0302
T 1393
T 1306
T 0483
T 0335
T 1505
T 0934
T 0637
T 1020
T 0645
T 0297
T 1884
T 0551
T 0660
T 1550
T 1549
T 0729
T 0527
T 0521
T 0500
T 0161
T 0646
T 0129
T 0145
T 1592
T 1978
T 1658
T 0836
T 0644
T 1765
T 1043
T 0865
T 0791
T 1614
T 0215
T 1024
T 0367
T 0368
T 1734
T 1395
T 0860
T 0437
T 0474
T 1392
T 0086
T 0298
T 0532
T 0541
T 0522
T 1987
T 1806
T 0223
T 0224
T 1435
T 0819
T 0837
T 0945
T 1023
T 1511
T 0466
T 1389
T 0299
T 0190
T 1473
T 1689
T 1156
T 1153
T 0562
T 1901
T 1804
T 1869
T 1051
T 1998
T 1740
T 1361
T 1701
T 1732
T 1365
T 1453
T 1728
T 0868
T 0943
T 1241
T 2023
T 0242
T 0243
T 0276
T 0742
T 0484
T 2011
T 0440
T 0240
T 1276
T 0550
T 1059
T 0430
T 1710
T 0200
T 1457
T 0662
T 1705
T 0278
T 1586
T 0720
T 1476
T 2003
T 0702
T 1730
T 1629
T 0863
T 0169
T 1660

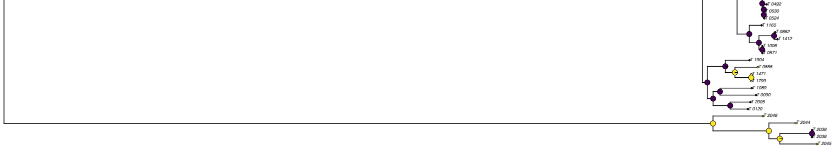
T 0492
T 0530
T 0524
T 1165
T 0862
T 1412
T 1006
T 0571
T 1904
T 0555
T 1471
T 1799
T 1089
T 0090
T 2005
T 0120
T 2048
T 2044
T 2039
T 2038
T 2045

Effector gene: tse8

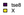

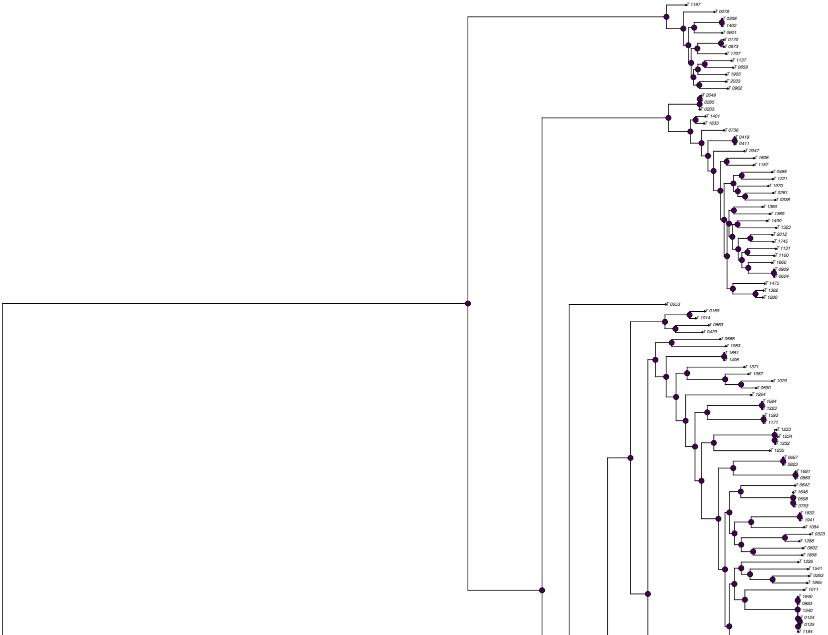

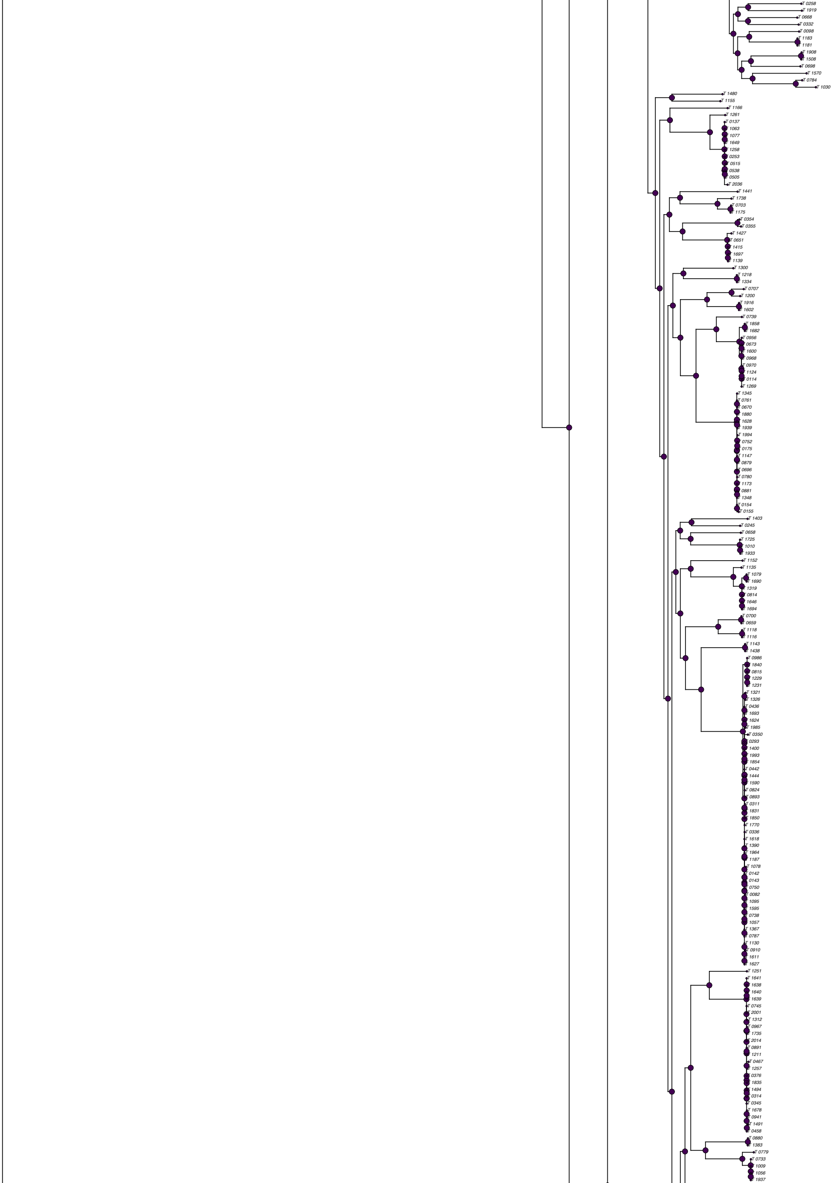
T 0258
T 1919
T 0668
T 0332
T 0098
T 1183
T 1181
T 1908
T 1508
T 0698
T 1570
T 0784
T 1030
T 1480
T 1155
T 1166
T 1261
T 0137
1063
1077
T 1649
1258
0253
0515
0538
0505
T 2036
T 1441
T 1738
T 0703
T 1175
T 0354
T 0355
T 1427
T 0651
1415
1697
1139
T 1300
T 1218
1334
T 0707
T 1200
T 1916
T 1602
T 0739
T 1858
T 1682
T 0956
0673
T 1600
T 0968
T 0970
T 1124
T 0114
T 1269
T 1345
T 0761
0670
1880
1628
1939
T 1994
0752
0175
T 1147
T 0879
T 0696
T 0780
1173
0881
T 1348
T 0154
T 0155
T 1403
T 0245
T 0658
T 1725
1010
1933
T 1152
T 1135
T 1079
T 1690
1319
0814
1646
T 1694
T 0700
T 0659
T 1118
T 1116
T 1143
1438
T 0986
1840
0815
1229
1231
T 1321
T 1326
0436
1693
1624
T 1985
T 0350
0293
1400
1993
1854
T 0442
1444
1590
T 0824
0893
T 0311
1831
1850
T 1770
T 0336
T 1618
T 1390
T 1964
1187
T 1078
0142
0143
0750
T 0082
1095
1595
T 0738
1057
T 1367
T 0787
T 1130
T 0910
1611
1627
T 1251
T 1641
1638
1640
1639
T 0745
2001
T 1312
T 0967
1735
2014
T 0891
1211
T 0467
1257
0376
1835
1494
0314
T 0345
T 1678
0941
T 1491
0458
T 0880
T 1383
T 0779
T 0733
1009
1056
T 1937

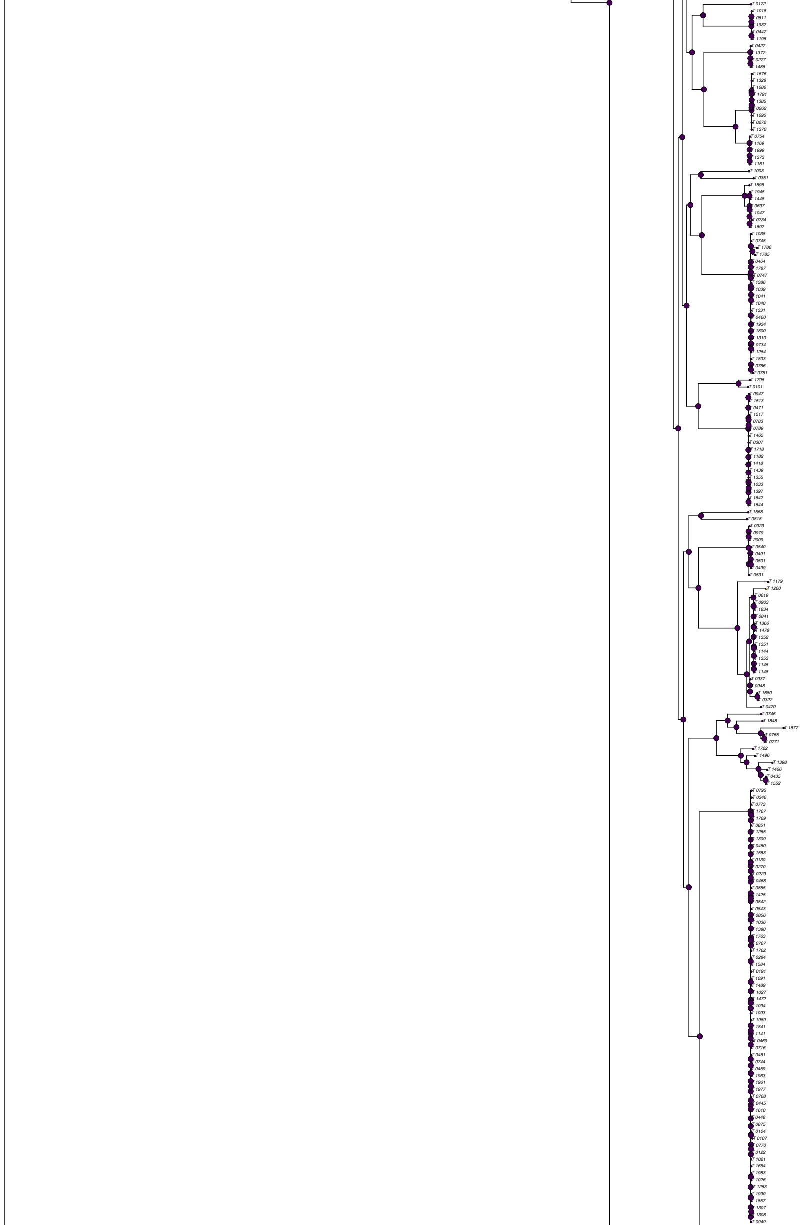
T 0172
T 1018
0611
1932
T 0447
T 1196
T 0427
1372
T 0277
T 1486
T 1676
T 1328
T 1686
1791
1385
T 0262
T 1695
T 0272
T 1370
T 0754
1169
1999
1373
T 1161
T 1003
T 0351
T 1596
T 1945
T 1448
0697
T 1047
T 0234
T 1692
T 1038
T 0748
T 1786
T 1785
0464
1787
T 0747
T 1386
1039
1041
1040
T 1331
0460
1934
1800
1310
0734
T 1254
T 1803
0766
T 0751
T 1795
T 0101
T 0947
T 1513
0471
T 1517
0783
0789
T 1465
T 0307
T 1718
T 1182
1418
T 1439
T 1355
1033
1397
T 1642
T 1644
T 1568
T 0818
T 0923
0979
T 2009
T 0540
0491
0501
T 0499
T 0531
T 1179
T 1260
T 0619
T 0903
T 1834
0841
T 1366
T 1478
1352
T 1351
1144
1353
1145
T 1148
T 0937
0948
T 1680
T 0322
T 0470
T 0746
T 1848
T 1877
T 0765
T 0771
T 1722
T 1496
T 1398
T 1466
T 0435
T 1552
T 0795
T 0346
T 0773
T 1767
1769
T 0851
1265
T 1309
0450
T 1583
T 0130
T 0270
T 0229
T 0468
T 0855
T 1425
0842
T 0843
0856
T 1036
1380
T 1763
0767
T 1762
T 0284
T 1584
T 0191
T 1091
T 1489
1027
T 1472
T 1094
T 1093
T 1989
1841
1141
T 0469
T 0716
T 0461
0744
T 0459
1963
1961
1977
T 0768
0445
1610
0448
T 0875
T 0104
T 0107
0770
0122
T 1021
T 1654
T 1983
1026
T 1253
T 1990
T 1857
T 1307
1308
T 0949

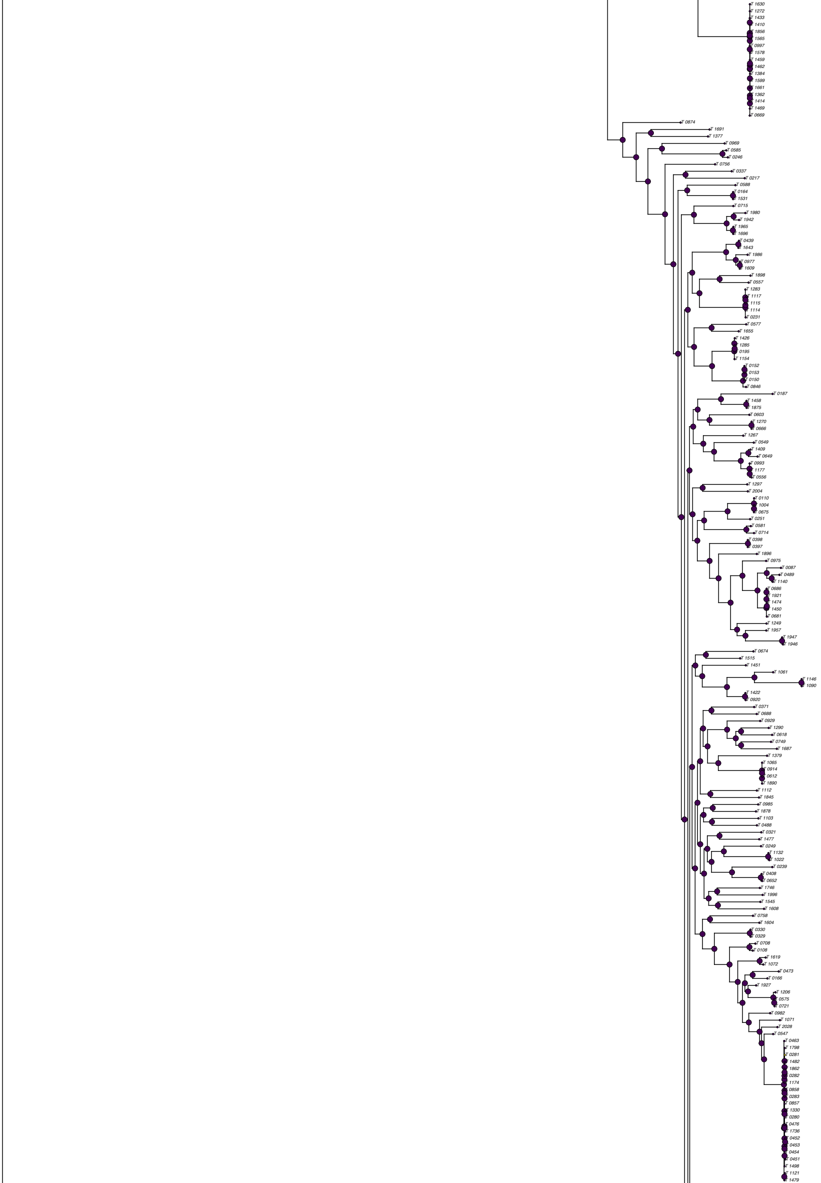

T 1630
T 1272
T 1433
T 1410
T 1856
T 1565
T 0997
T 1578
T 1459
T 1462
T 1384
T 1599
T 1661
T 1362
T 1414
T 1469
T 0669
T 0874
T 1691
T 1377
T 0969
T 0585
T 0246
T 0756
T 0337
T 0217
T 0588
T 0164
T 1531
T 0715
T 1980
T 1942
T 1965
T 1696
T 0439
T 1643
T 1986
T 0977
T 1609
T 1898
T 0557
T 1283
T 1117
T 1115
T 1114
T 0231
T 0577
T 1655
T 1426
T 1285
T 0195
T 1154
T 0152
T 0153
T 0150
T 0846
T 0187
T 1458
T 1875
T 0603
T 1270
T 0666
T 1267
T 0549
T 1409
T 0649
T 0993
T 1177
T 0556
T 1297
T 2004
T 0110
T 1004
T 0675
T 0251
T 0581
T 0714
T 0398
T 0397
T 1896
T 0975
T 0087
T 0489
T 1140
T 0686
T 1921
T 1474
T 1450
T 0681
T 1249
T 1957
T 1947
T 1946
T 0674
T 1515
T 1451
T 1061
T 1146
T 1090
T 1422
T 0920
T 0371
T 0688
T 0929
T 1290
T 0618
T 0749
T 1687
T 1379
T 1065
T 0914
T 0612
T 1890
T 1112
T 1845
T 0985
T 1878
T 1103
T 0488
T 0321
T 1477
T 0249
T 1132
T 1022
T 0239
T 0408
T 0652
T 1746
T 1996
T 1545
T 1608
T 0758
T 1604
T 0330
T 0329
T 0708
T 0108
T 1619
T 1072
T 0473
T 0166
T 1927
T 1206
T 0575
T 0721
T 0982
T 1071
T 2028
T 0547
T 0463
T 1798
T 0281
T 1482
T 1862
T 0282
T 1174
T 0858
T 0283
T 0857
T 1330
T 0280
T 0476
T 1736
T 0452
T 0453
T 0454
T 0451
T 1498
T 1121
T 1479

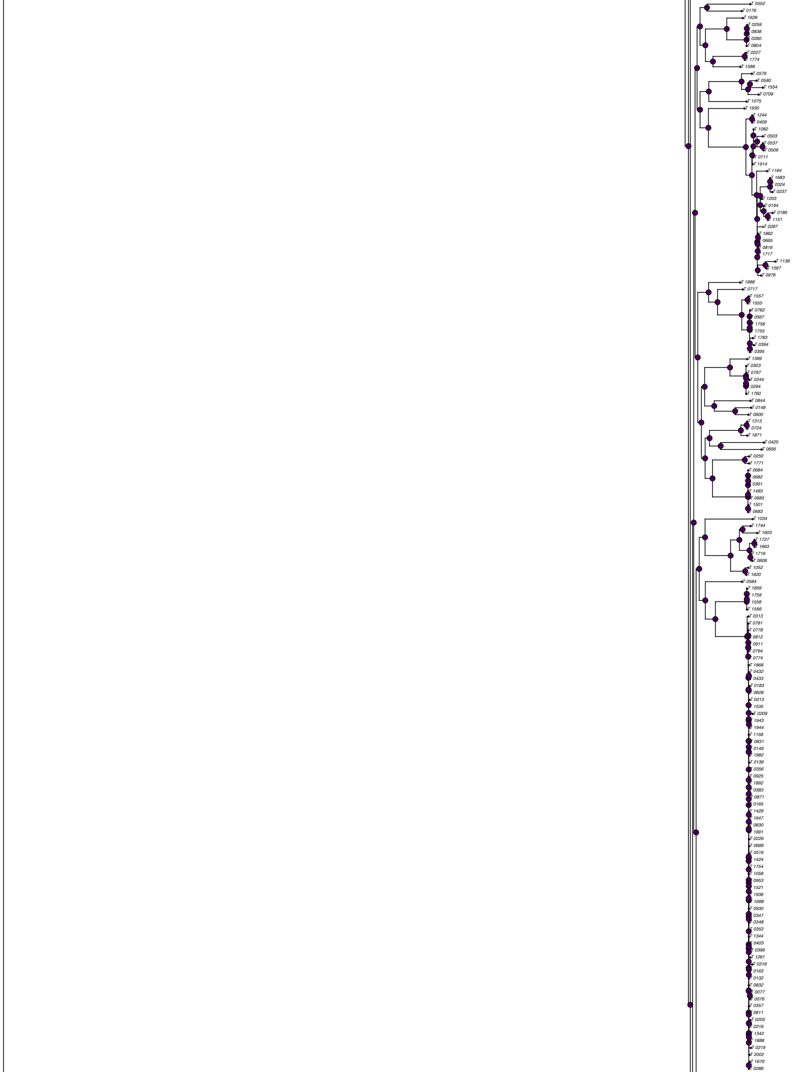
T 0552
T 0176
T 1626
T 0259
0838
0260
T 0804
T 0227
T 1774
T 1588
T 0579
T 0580
T 1554
T 0709
T 1075
T 1930
T 1244
T 0409
T 1082
T 0503
T 0537
T 0509
T 0711
T 1914
T 1164
T 1683
0324
T 0237
T 1203
T 0194
T 0186
T 1151
T 0287
T 1882
0665
T 0816
1717
T 1138
T 1567
T 0976
T 1988
T 0717
T 1557
T 1555
T 0762
0567
T 1756
1755
T 1783
T 0394
T 0395
T 1399
T 0303
0797
T 0244
0294
T 1760
T 0844
T 0148
T 0600
T 1313
T 0724
T 1871
T 0425
T 0695
T 0250
T 1771
T 0684
0682
0361
T 1493
T 0685
T 1501
T 0683
T 1034
T 1744
T 1603
T 1727
T 1663
T 1716
T 0608
T 1052
T 1620
T 0584
T 1859
1759
1558
T 1566
T 0313
T 0781
T 0778
0812
0911
T 0794
0774
T 1868
T 0432
0433
T 0183
T 0828
T 0213
1535
T 0209
1943
T 1944
T 1158
0831
0149
T 1982
T 0136
0356
T 0925
T 1892
0383
T 0871
0165
T 1429
T 1647
T 0830
1991
T 0226
T 0689
T 0516
1424
T 1754
T 1058
0953
1521
T 1936
1698
T 0930
0347
T 0348
T 0352
T 1344
0403
T 0396
T 1281
T 0318
0163
T 0132
T 0832
T 0077
T 0076
T 0357
0811
T 0205
T 0216
T 1342
T 1888
T 0219
T 2002
T 1679
T 0288

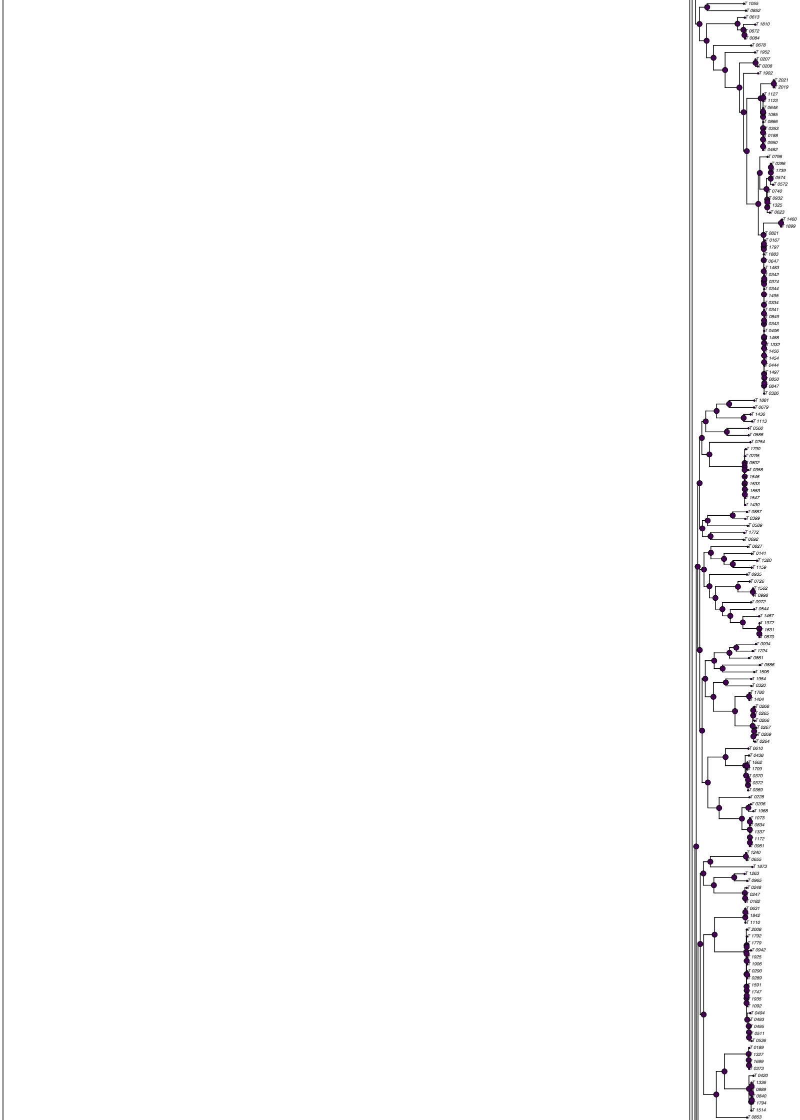
T 1055
T 0852
T 0613
T 1810
T 0672
T 0084
T 0678
T 1952
T 0207
T 0208
T 1902
T 2021
T 2019
T 1127
T 1123
T 0648
T 1085
T 0866
T 0353
T 0188
T 0950
T 0462
T 0796
T 0286
T 1739
T 0574
T 0572
T 0740
T 0932
T 1325
T 0623
T 1460
T 1899
T 0821
T 0167
T 1797
T 1883
T 0647
T 1483
T 0342
T 0374
T 0344
T 1495
T 0334
T 0341
T 0849
T 0343
T 0406
T 1488
T 1332
T 1456
T 1454
T 0444
T 1497
T 0850
T 0847
T 0326
T 1881
T 0679
T 1436
T 1113
T 0560
T 0586
T 0254
T 1790
T 0235
T 0802
T 0358
T 1546
T 1533
T 1553
T 1547
T 1430
T 0887
T 0399
T 0589
T 1772
T 0692
T 0827
T 0141
T 1320
T 1159
T 0935
T 0726
T 1562
T 0998
T 0972
T 0544
T 1467
T 1972
T 1631
T 0870
T 0094
T 1224
T 0861
T 0886
T 1506
T 1954
T 0320
T 1780
T 1404
T 0268
T 0265
T 0266
T 0267
T 0269
T 0264
T 0610
T 0438
T 1662
T 1709
T 0370
T 0372
T 0369
T 0228
T 0206
T 1968
T 1073
T 0834
T 1337
T 1172
T 0961
T 1240
T 0655
T 1873
T 1263
T 0965
T 0248
T 0247
T 0182
T 0631
T 1842
T 1110
T 2008
T 1792
T 1779
T 0942
T 1925
T 1906
T 0290
T 0289
T 1591
T 1747
T 1935
T 1092
T 0494
T 0493
T 0495
T 0511
T 0536
T 0189
T 1327
T 1699
T 0373
T 0420
T 1336
T 0889
T 0840
T 1794
T 1514
T 0853

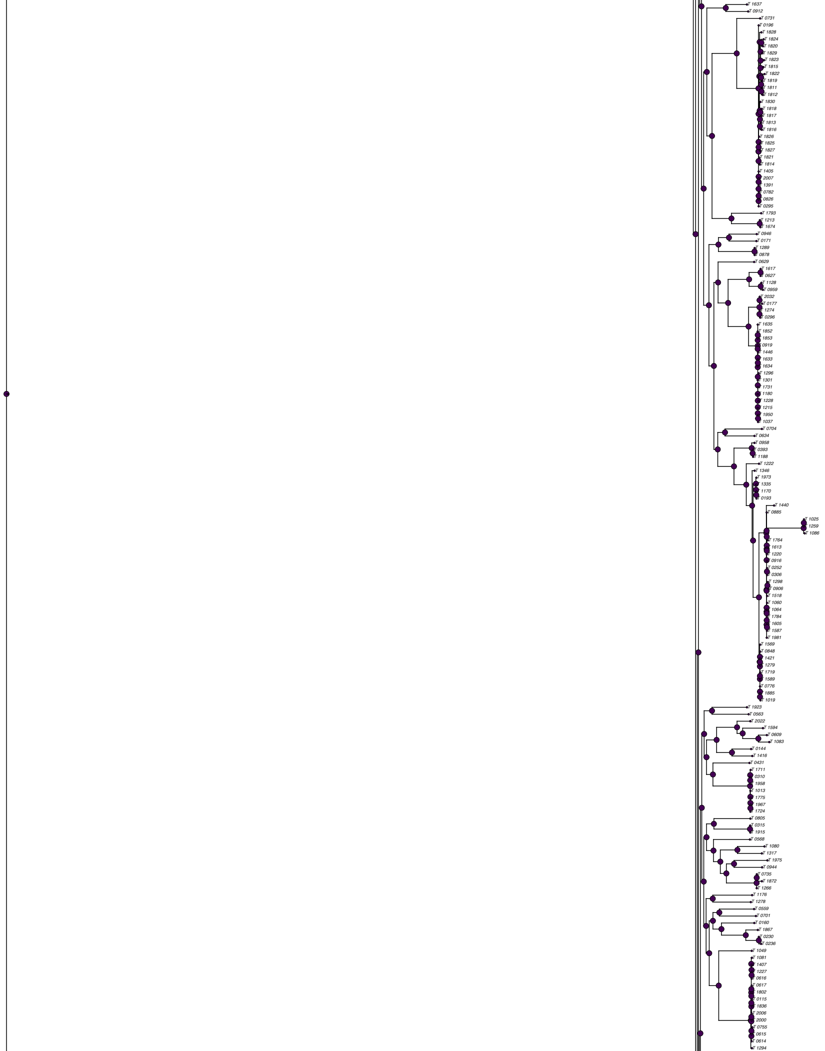

T 1637
T 0912
T 0731
T 0196
T 1828
T 1824
T 1820
T 1829
T 1823
T 1815
T 1822
T 1819
T 1811
T 1812
T 1830
T 1818
T 1817
T 1813
T 1816
T 1826
T 1825
T 1827
T 1821
T 1814
T 1405
2007
1391
0782
0826
T 0295
T 1793
T 1213
T 1674
T 0946
T 0171
T 1289
T 0878
T 0629
T 1617
T 0627
T 1128
T 0959
T 2032
T 0177
T 1274
T 0296
T 1635
T 1852
1853
0919
T 1446
1633
1634
T 1296
T 1301
1731
1180
1228
1215
1950
T 1037
T 0704
T 0634
T 0958
T 0393
T 1188
T 1222
T 1346
T 1973
1335
1170
T 0193
T 1440
T 0885
T 1025
1259
T 1086
T 1764
1613
T 1220
0916
T 0252
T 0306
T 1298
T 0906
T 1518
T 1060
1064
T 1784
1605
T 1587
T 1981
T 1569
T 0848
1421
1279
T 1719
1589
T 0776
1885
T 1019
T 1923
T 0563
T 2022
T 1594
T 0609
T 1083
T 0144
T 1416
T 0431
T 1711
0310
1958
T 1013
1775
1967
T 1724
T 0805
T 0315
T 1915
T 0568
T 1080
T 1317
T 1975
T 0944
T 0735
T 1872
T 1266
T 1176
T 1278
T 0559
T 0701
T 0160
T 1867
T 0230
T 0236
T 1049
T 1081
1407
1227
0616
T 0617
1802
T 0115
1836
T 2006
2000
T 0755
0615
T 0614
T 1294

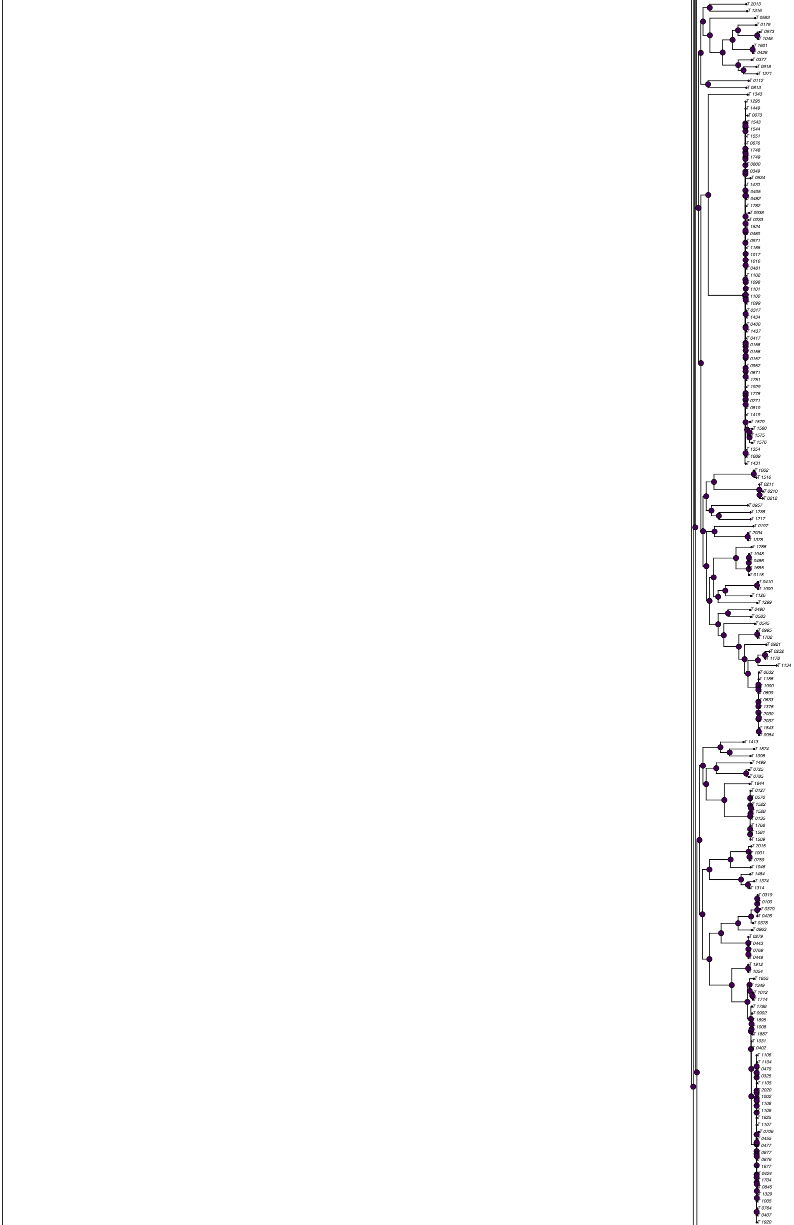
T 2013
T 1316
T 0593
T 0179
T 0973
T 1048
T 1601
T 0428
T 0377
T 0918
T 1271
T 0112
T 0813
T 1343
T 1295
T 1449
T 0073
T 1543
T 1544
T 1551
T 0676
T 1748
T 1749
T 0800
T 0349
T 0534
T 1470
T 0405
T 0482
T 1782
T 0938
T 0233
T 1524
T 0480
T 0971
T 1185
T 1017
T 1016
T 0481
T 1102
T 1098
T 1101
T 1100
T 1099
T 0317
T 1434
T 0400
T 1437
T 0417
T 0158
T 0156
T 0157
T 0952
T 0671
T 1751
T 1929
T 1778
T 0271
T 0810
T 1419
T 1579
T 1580
T 1575
T 1576
T 1354
T 1889
T 1431
T 1062
T 1516
T 0211
T 0210
T 0212
T 0957
T 1236
T 1217
T 0197
T 2034
T 1378
T 1286
T 1948
T 0486
T 1685
T 0118
T 0410
T 1909
T 1126
T 1299
T 0490
T 0583
T 0545
T 0995
T 1702
T 0921
T 0232
T 1178
T 1134
T 0632
T 1186
T 1900
T 0699
T 0633
T 1376
T 2030
T 2037
T 1843
T 0954
T 1413
T 1874
T 1096
T 1499
T 0725
T 0785
T 1844
T 0127
T 0570
T 1522
T 1528
T 0135
T 1768
T 1581
T 1509
T 2015
T 1001
T 0759
T 1046
T 1484
T 1374
T 1314
T 0319
T 0100
T 0379
T 0426
T 0378
T 0963
T 0279
T 0443
T 0769
T 0449
T 1912
T 1054
T 1855
T 1349
T 1012
T 1714
T 1788
T 0902
T 1895
T 1008
T 1887
T 1031
T 0402
T 1106
T 1104
T 0479
T 0325
T 1105
T 2020
T 1002
T 1108
T 1109
T 1625
T 1107
T 0706
T 0455
T 0477
T 0877
T 0876
T 1677
T 0424
T 1704
T 0845
T 1329
T 1005
T 0764
T 0407
T 1920

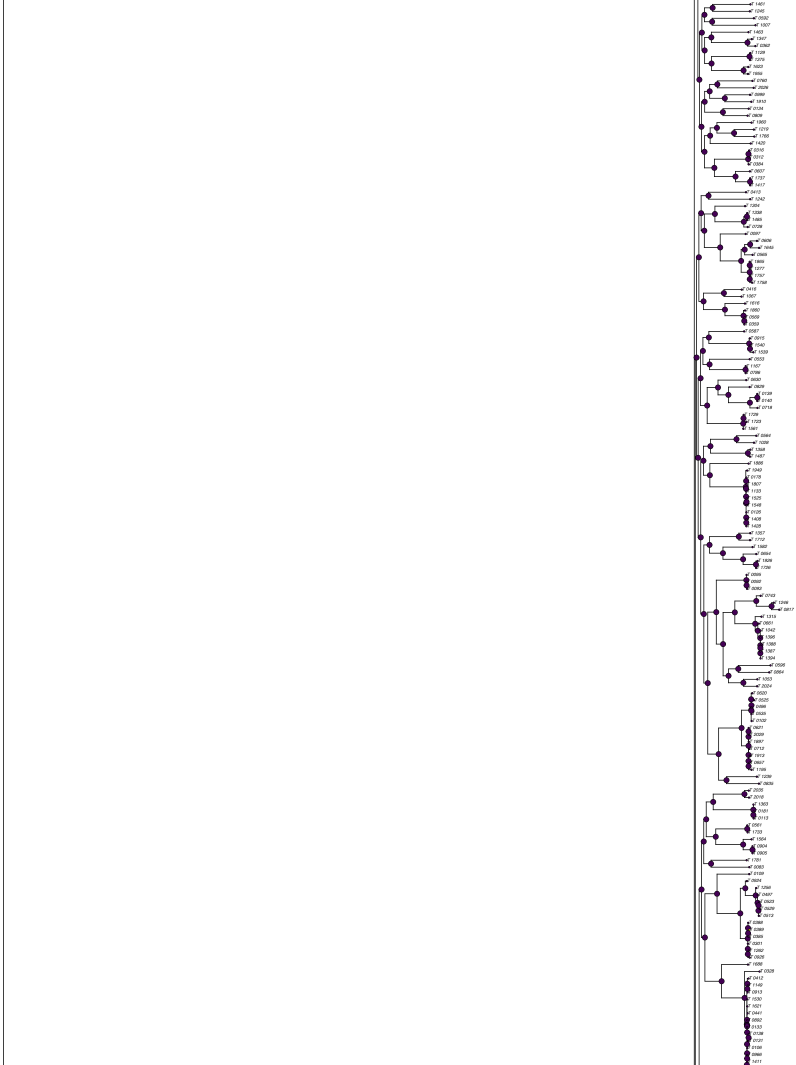
T 1461
T 1245
T 0592
T 1007
T 1463
T 1347
T 0362
T 1129
T 1375
T 1623
T 1955
T 0760
T 2026
T 0999
T 1910
T 0134
T 0809
T 1960
T 1219
T 1766
T 1420
T 0316
T 0312
T 0384
T 0607
T 1737
T 1417
T 0413
T 1242
T 1304
T 1338
T 1485
T 0728
T 0097
T 0606
T 1645
T 0565
T 1865
T 1277
T 1757
T 1758
T 0416
T 1067
T 1616
T 1860
T 0569
T 0359
T 0587
T 0915
T 1540
T 1539
T 0553
T 1167
T 0786
T 0630
T 0829
T 0139
T 0140
T 0718
T 1729
T 1723
T 1561
T 0564
T 1028
T 1358
T 1487
T 1886
T 1949
T 0178
T 1807
T 1133
T 1525
T 1548
T 0126
T 1408
T 1428
T 1357
T 1712
T 1582
T 0654
T 1926
T 1726
T 0095
T 0092
T 0093
T 0743
T 1246
T 0817
T 1315
T 0661
T 1042
T 1396
T 1388
T 1387
T 1394
T 0596
T 0864
T 1053
T 2024
T 0620
T 0525
T 0496
T 0535
T 0102
T 0621
T 2029
T 1897
T 0712
T 1913
T 0657
T 1195
T 1239
T 0835
T 2035
T 2018
T 1363
T 0181
T 0113
T 0561
T 1733
T 1564
T 0904
T 0905
T 1781
T 0083
T 0109
T 0924
T 1256
T 0497
T 0523
T 0529
T 0513
T 0388
T 0389
T 0385
T 0301
T 1262
T 0926
T 1688
T 0328
T 0412
T 1149
T 0913
T 1530
T 1621
T 0441
T 0892
T 0133
T 0138
T 0131
T 0106
T 0966
T 1411

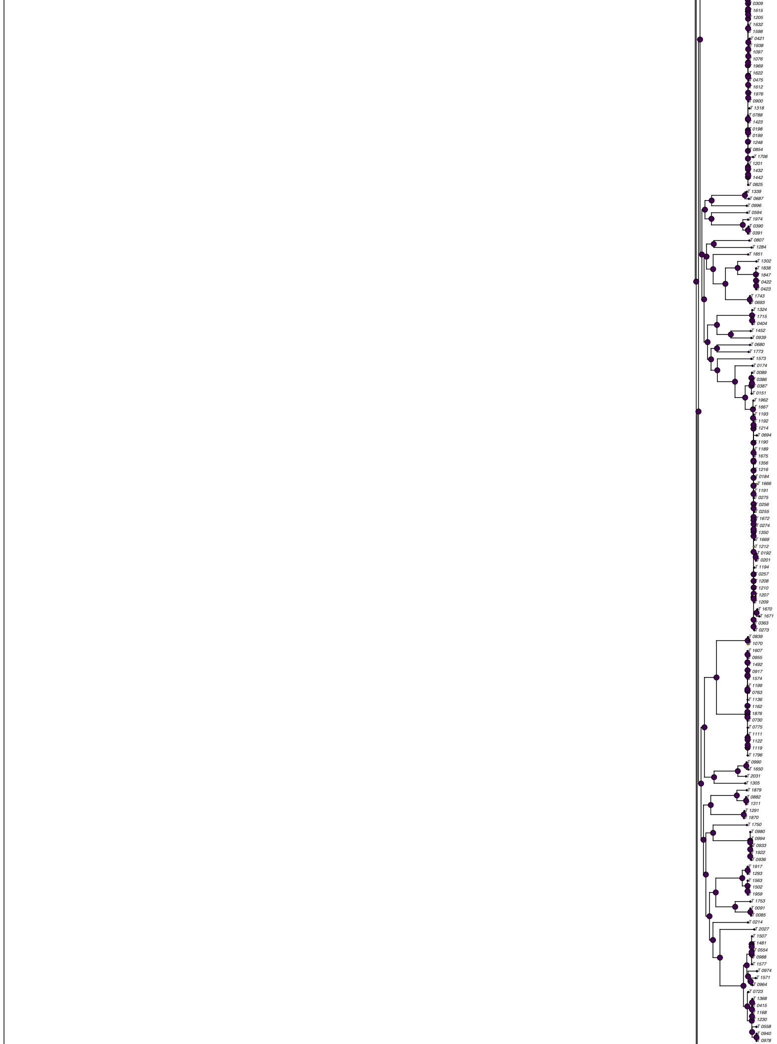

T 0309
T 1615
T 1205
T 1632
T 1598
T 0421
T 1938
T 1097
T 1076
T 1969
T 1622
T 0475
T 1612
T 1976
T 0900
T 1318
T 0788
T 1423
T 0198
T 0199
T 1248
T 0854
T 1706
T 1201
T 1432
T 1442
T 0825
T 1339
T 0687
T 0996
T 0594
T 1974
T 0390
T 0391
T 0807
T 1284
T 1851
T 1302
T 1838
T 1847
T 0422
T 0423
T 1743
T 0693
T 1324
T 1715
T 0404
T 1452
T 0939
T 0680
T 1773
T 1573
T 0174
T 0089
T 0386
T 0387
T 0151
T 1962
T 1667
T 1193
T 1192
T 1214
T 0694
T 1190
T 1189
T 1675
T 1356
T 1216
T 0184
T 1666
T 1191
T 0275
T 0256
T 0255
T 1672
T 0274
T 1350
T 1669
T 1212
T 0192
T 0201
T 1194
T 0257
T 1208
T 1210
T 1207
T 1209
T 1670
T 1671
T 0363
T 0273
T 0839
T 1070
T 1607
T 0955
T 1492
T 0917
T 1574
T 1199
T 0763
T 1136
T 1162
T 1876
T 0730
T 0775
T 1111
T 1122
T 1119
T 1796
T 0990
T 1650
T 2031
T 1305
T 1879
T 0882
T 1311
T 1291
T 1870
T 1750
T 0980
T 0994
T 0933
T 1922
T 0936
T 1917
T 1293
T 1563
T 1502
T 1959
T 1753
T 0091
T 0085
T 0214
T 2027
T 1507
T 1481
T 0554
T 0988
T 1577
T 0974
T 1571
T 0964
T 0723
T 1368
T 0415
T 1168
T 1230
T 0558
T 0940
T 0978

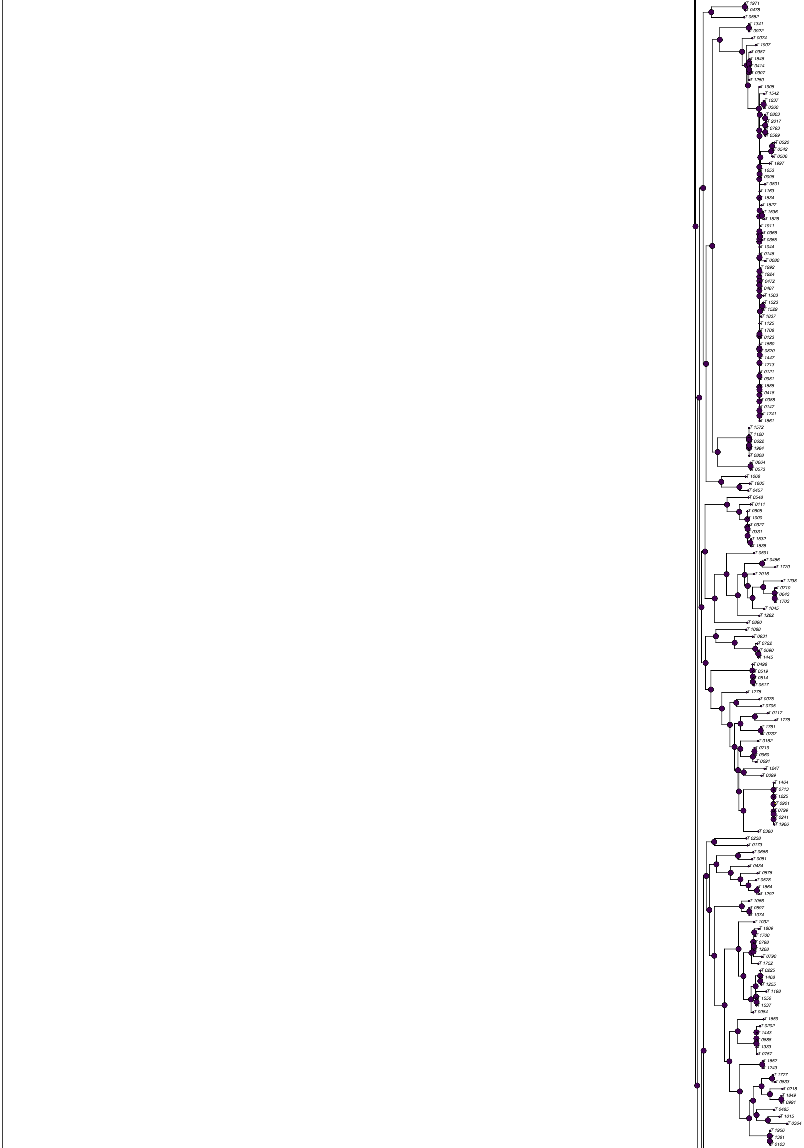
T 1971
T 0478
T 0582
T 1341
T 0922
T 0074
T 1907
T 0987
T 1846
T 0414
T 0907
T 1250
T 1905
T 1542
T 1237
T 0360
T 0803
T 2017
T 0793
T 0599
T 0520
T 0542
T 0506
T 1997
T 1653
T 0096
T 0801
T 1163
T 1534
T 1527
T 1536
T 1526
T 1911
T 0366
T 0365
T 1044
T 0146
T 0080
T 1992
T 1924
T 0472
T 0487
T 1503
T 1523
T 1529
T 1837
T 1125
T 1708
T 0123
T 1560
T 0820
T 1447
T 1713
T 0121
T 0981
T 1585
T 0418
T 0088
T 0147
T 1741
T 1861
T 1572
T 1120
T 0622
T 1984
T 0808
T 0664
T 0573
T 1068
T 1805
T 0457
T 0548
T 0111
T 0605
T 1000
T 0327
T 0331
T 1532
T 1538
T 0591
T 0456
T 1720
T 2016
T 1238
T 0710
T 0643
T 1703
T 1045
T 1282
T 0890
T 1088
T 0931
T 0722
T 0690
T 1445
T 0498
T 0519
T 0514
T 0517
T 1275
T 0075
T 0705
T 0117
T 1776
T 1761
T 0737
T 0162
T 0719
T 0960
T 0691
T 1247
T 0099
T 1464
T 0713
T 1225
T 0901
T 0799
T 0241
T 1966
T 0380
T 0238
T 0173
T 0656
T 0081
T 0434
T 0576
T 0578
T 1864
T 1292
T 1066
T 0597
T 1074
T 1032
T 1809
T 1700
T 0798
T 1268
T 0790
T 1752
T 0225
T 1468
T 1255
T 1198
T 1556
T 1537
T 0984
T 1659
T 0202
T 1443
T 0888
T 1333
T 0757
T 1652
T 1243
T 1777
T 0833
T 0218
T 1849
T 0991
T 0485
T 1015
T 0364
T 1956
T 1381
T 0103

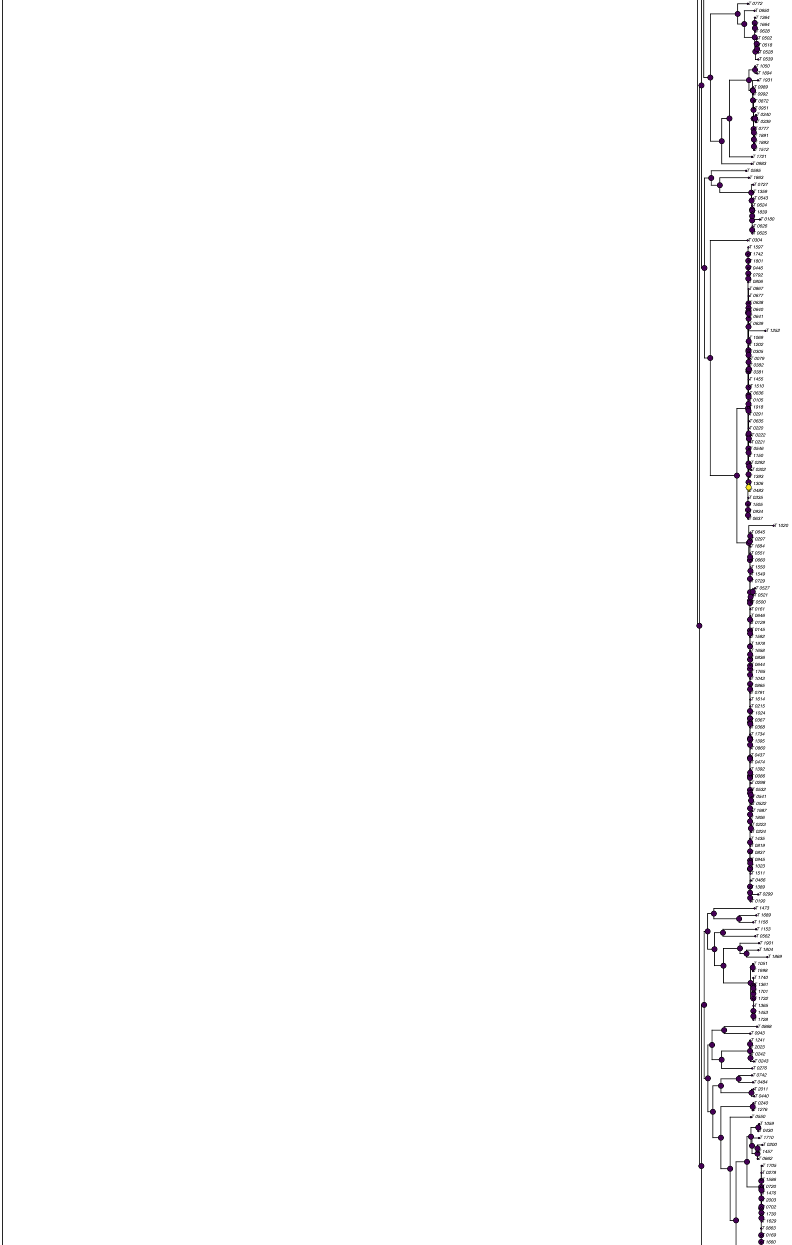
T 0772
T 0650
T 1364
1664
0628
T 0502
0518
T 0528
T 0539
T 1050
T 1894
T 1931
T 0989
0992
0872
0951
T 0340
T 0339
0777
1891
1893
1512
T 1721
T 0983
T 0595
T 1863
T 0727
T 1359
T 0543
T 0624
1839
T 0180
T 0626
0625
T 0304
T 1597
1742
1801
0446
0792
0806
T 0867
T 0677
0638
0640
0641
0639
T 1252
T 1069
1202
0305
T 0079
0382
0381
T 1455
1510
0636
0105
1918
T 0291
T 0635
T 0220
T 0222
T 0221
0546
1150
T 0292
T 0302
1393
1306
0483
T 0335
1505
0934
0637
T 1020
T 0645
0297
1884
T 0551
0660
T 1550
1549
0729
T 0527
T 0521
0500
T 0161
0646
0129
T 0145
1592
T 1978
1658
0836
0644
1765
1043
0865
T 0791
T 1614
T 0215
1024
0367
0368
T 1734
1395
0860
0437
T 0474
T 1392
0086
T 0298
0532
0541
0522
T 1987
1806
0223
0224
T 1435
T 0819
0837
0945
1023
T 1511
T 0466
1389
T 0299
T 0190
T 1473
T 1689
T 1156
T 1153
T 0562
T 1901
T 1804
T 1869
T 1051
1998
T 1740
1361
1701
1732
T 1365
1453
T 1728
T 0868
T 0943
T 1241
2023
0242
T 0243
T 0276
T 0742
T 0484
T 2011
T 0440
T 0240
1276
T 0550
T 1059
0430
T 1710
T 0200
1457
T 0662
T 1705
T 0278
1586
0720
1476
2003
0702
1730
1629
T 0863
0169
1660

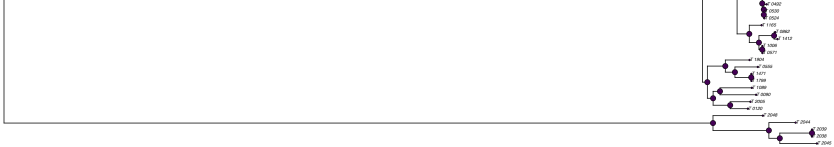
T 0492
T 0530
T 0524
T 1165
T 0862
T 1412
T 1006
T 0571
T 1904
T 0555
T 1471
T 1799
T 1089
T 0090
T 2005
T 0120
T 2048
T 2044
T 2039
T 2038
T 2045

Effector gene: tne3 (tse6a)

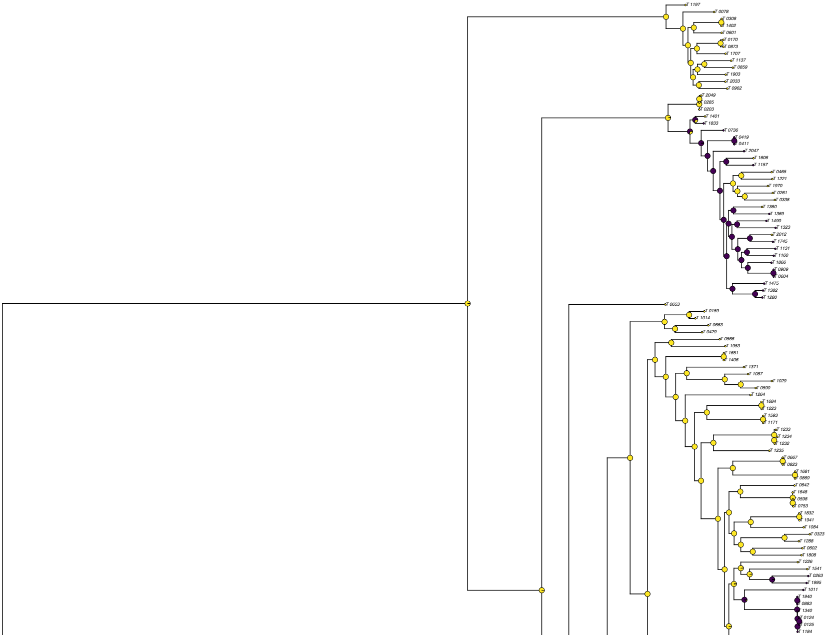

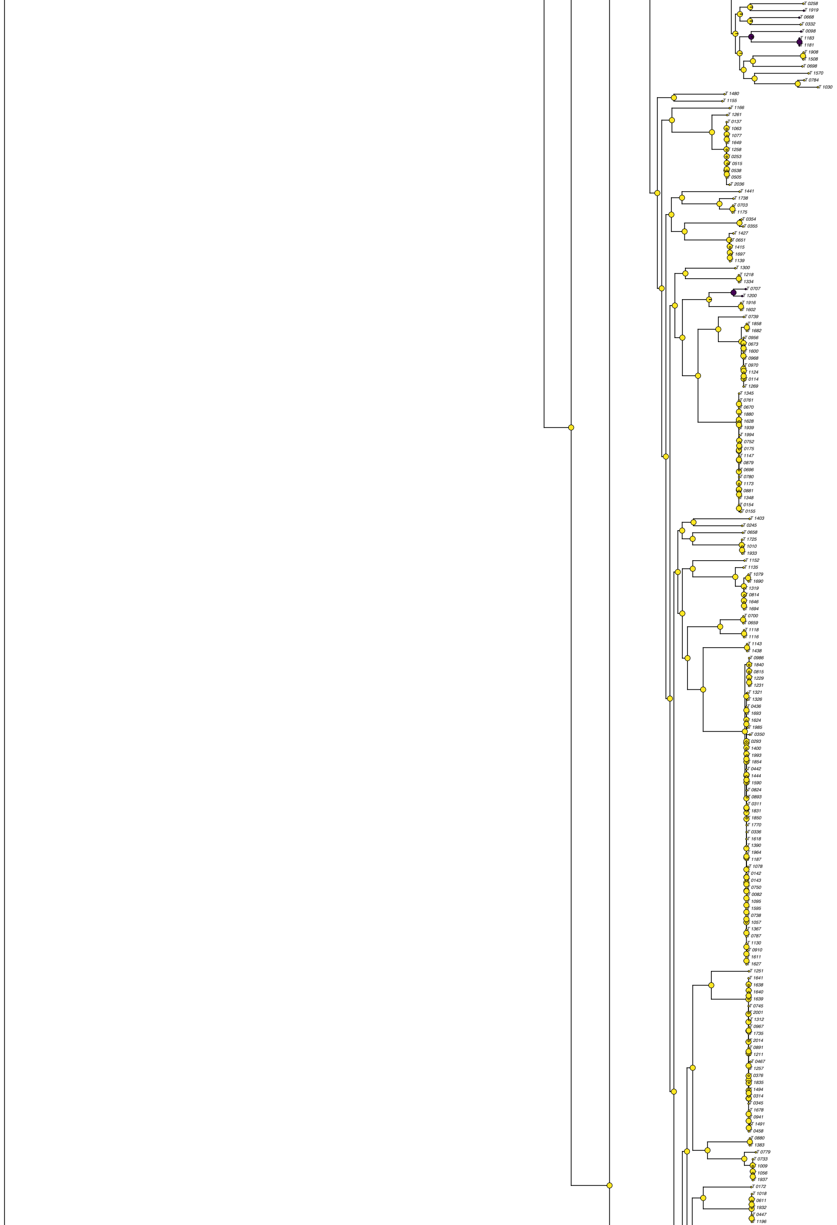

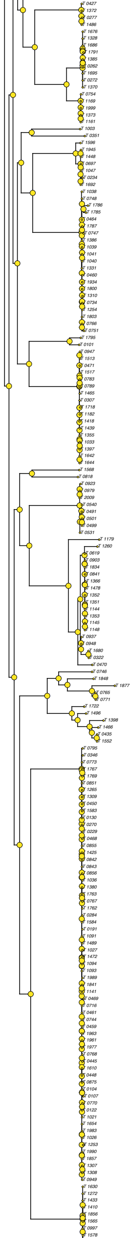
T 0427
1372
0277
1486
T 1676
T 1328
T 1686
1791
1385
0262
T 1695
T 0272
T 1370
T 0754
1169
1999
1373
T 1161
T 1003
T 0351
T 1596
T 1945
T 1448
0697
T 1047
T 0234
1692
T 1038
T 0748
T 1786
T 1785
0464
1787
T 0747
T 1386
1039
1041
T 1040
T 1331
0460
1934
1800
1310
0734
T 1254
T 1803
0766
T 0751
T 1795
T 0101
T 0947
T 1513
0471
T 1517
0783
0789
T 1465
T 0307
T 1718
T 1182
1418
T 1439
T 1355
1033
1397
T 1642
1644
T 1568
T 0818
T 0923
0979
T 2009
T 0540
0491
0501
T 0499
T 0531
T 1179
T 1260
0619
T 0903
1834
0841
T 1366
T 1478
1352
T 1351
T 1144
1353
1145
T 1148
T 0937
0948
T 1680
T 0322
T 0470
T 0746
T 1848
T 1877
T 0765
T 0771
T 1722
T 1496
T 1398
T 1466
T 0435
T 1552
T 0795
T 0346
T 0773
1767
1769
T 0851
1265
1309
0450
1583
T 0130
0270
T 0229
0468
T 0855
1425
0842
T 0843
0856
T 1036
1380
1763
0767
T 1762
T 0284
T 1584
T 0191
T 1091
T 1489
T 1027
1472
1094
T 1093
T 1989
1841
1141
T 0469
T 0716
T 0461
T 0744
0459
1963
1961
1977
T 0768
0445
1610
0448
T 0875
T 0104
T 0107
0770
0122
T 1021
T 1654
T 1983
1026
T 1253
T 1990
1857
1307
1308
T 0949
T 1630
T 1272
T 1433
1410
1856
1565
T 0997
1578

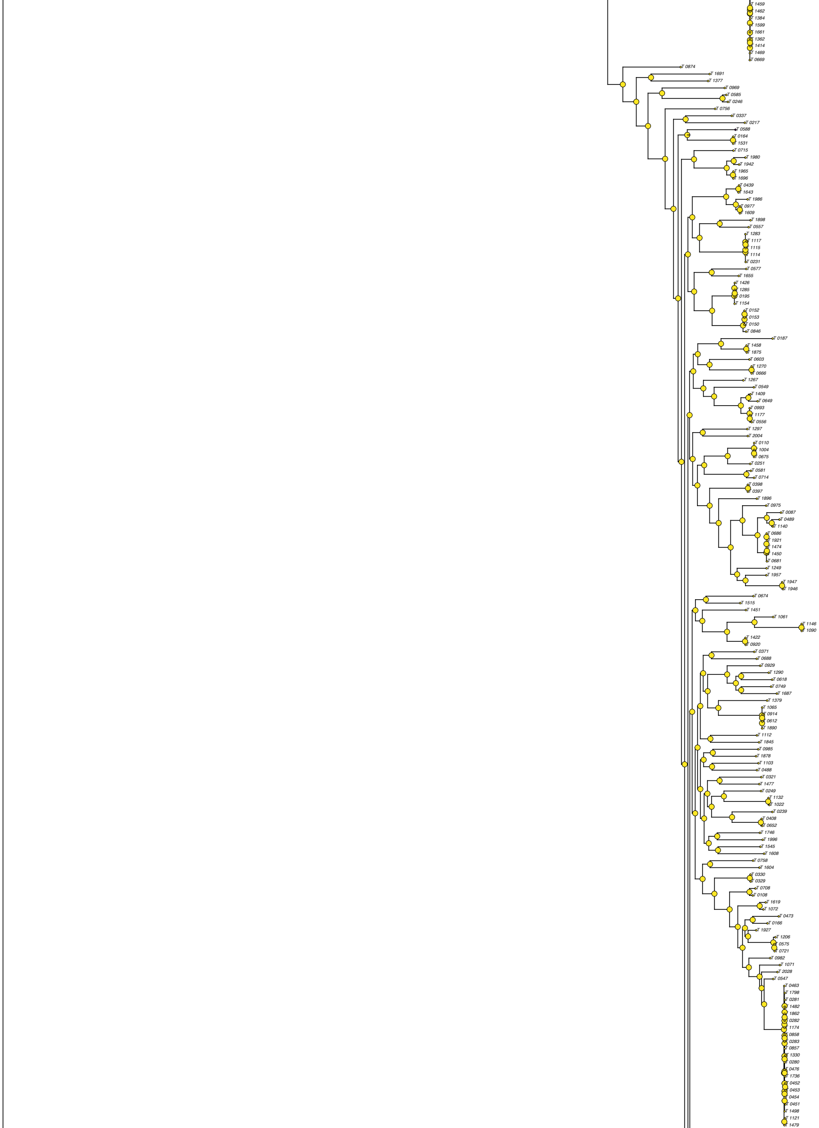
T 1459
T 1462
T 1384
T 1599
T 1661
T 1362
T 1414
T 1469
T 0669
T 0874
T 1691
T 1377
T 0969
T 0585
T 0246
T 0756
T 0337
T 0217
T 0588
T 0164
T 1531
T 0715
T 1980
T 1942
T 1965
T 1696
T 0439
T 1643
T 1986
T 0977
T 1609
T 1898
T 0557
T 1283
T 1117
T 1115
T 1114
T 0231
T 0577
T 1655
T 1426
T 1285
T 0195
T 1154
T 0152
T 0153
T 0150
T 0846
T 0187
T 1458
T 1875
T 0603
T 1270
T 0666
T 1267
T 0549
T 1409
T 0649
T 0993
T 1177
T 0556
T 1297
T 2004
T 0110
T 1004
T 0675
T 0251
T 0581
T 0714
T 0398
T 0397
T 1896
T 0975
T 0087
T 0489
T 1140
T 0686
T 1921
T 1474
T 1450
T 0681
T 1249
T 1957
T 1947
T 1946
T 0674
T 1515
T 1451
T 1061
T 1146
T 1090
T 1422
T 0920
T 0371
T 0688
T 0929
T 1290
T 0618
T 0749
T 1687
T 1379
T 1065
T 0914
T 0612
T 1890
T 1112
T 1845
T 0985
T 1878
T 1103
T 0488
T 0321
T 1477
T 0249
T 1132
T 1022
T 0239
T 0408
T 0652
T 1746
T 1996
T 1545
T 1608
T 0758
T 1604
T 0330
T 0329
T 0708
T 0108
T 1619
T 1072
T 0473
T 0166
T 1927
T 1206
T 0575
T 0721
T 0982
T 1071
T 2028
T 0547
T 0463
T 1798
T 0281
T 1482
T 1862
T 0282
T 1174
T 0858
T 0283
T 0857
T 1330
T 0280
T 0476
T 1736
T 0452
T 0453
T 0454
T 0451
T 1498
T 1121
T 1479

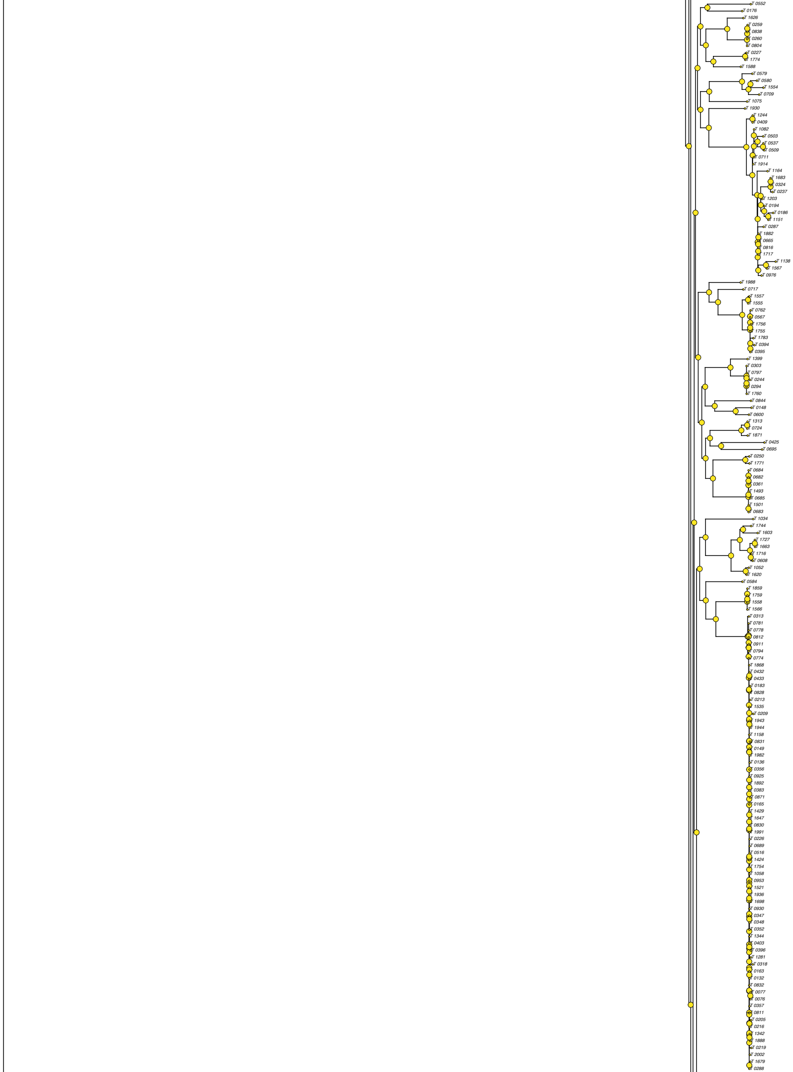

T 0552
T 0176
T 1626
T 0259
0838
0260
T 0804
T 0227
1774
T 1588
T 0579
T 0580
T 1554
T 0709
T 1075
T 1930
T 1244
T 0409
T 1082
T 0503
T 0537
T 0509
T 0711
T 1914
T 1164
T 1683
0324
T 0237
T 1203
T 0194
T 0186
1151
T 0287
T 1882
0665
T 0816
1717
T 1138
T 1567
T 0976
T 1988
T 0717
T 1557
1555
T 0762
0567
1756
1755
T 1783
T 0394
0395
T 1399
T 0303
0797
T 0244
0294
T 1760
T 0844
T 0148
T 0600
T 1313
T 0724
T 1871
T 0425
T 0695
T 0250
T 1771
T 0684
0682
0361
T 1493
T 0685
T 1501
0683
T 1034
T 1744
T 1603
T 1727
1663
T 1716
T 0608
T 1052
T 1620
T 0584
T 1859
1759
1558
T 1566
T 0313
T 0781
T 0778
0812
0911
0794
0774
T 1868
T 0432
0433
T 0183
0828
T 0213
1535
T 0209
1943
1944
T 1158
0831
0149
1982
T 0136
0356
T 0925
1892
0383
T 0871
0165
T 1429
1647
0830
1991
T 0226
T 0689
T 0516
1424
T 1754
T 1058
0953
1521
1936
1698
T 0930
0347
0348
T 0352
T 1344
T 0403
T 0396
T 1281
T 0318
0163
0132
T 0832
T 0077
T 0076
T 0357
0811
T 0205
T 0216
1342
1888
T 0219
T 2002
T 1679
0288

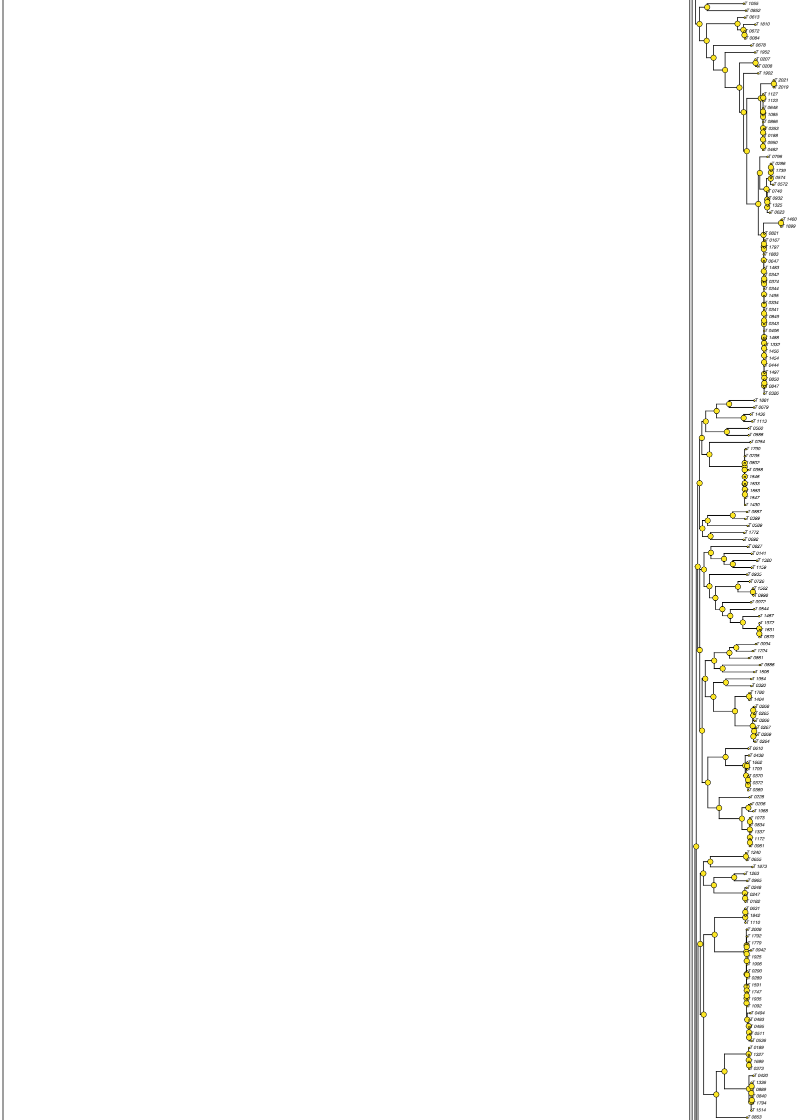
T 1055
T 0852
T 0613
T 1810
T 0672
T 0084
T 0678
T 1952
T 0207
T 0208
T 1902
T 2021
T 2019
T 1127
T 1123
T 0648
T 1085
T 0866
T 0353
T 0188
T 0950
T 0462
T 0796
T 0286
T 1739
T 0574
T 0572
T 0740
T 0932
T 1325
T 0623
T 1460
T 1899
T 0821
T 0167
T 1797
T 1883
T 0647
T 1483
T 0342
T 0374
T 0344
T 1495
T 0334
T 0341
T 0849
T 0343
T 0406
T 1488
T 1332
T 1456
T 1454
T 0444
T 1497
T 0850
T 0847
T 0326
T 1881
T 0679
T 1436
T 1113
T 0560
T 0586
T 0254
T 1790
T 0235
T 0802
T 0358
T 1546
T 1533
T 1553
T 1547
T 1430
T 0887
T 0399
T 0589
T 1772
T 0692
T 0827
T 0141
T 1320
T 1159
T 0935
T 0726
T 1562
T 0998
T 0972
T 0544
T 1467
T 1972
T 1631
T 0870
T 0094
T 1224
T 0861
T 0886
T 1506
T 1954
T 0320
T 1780
T 1404
T 0268
T 0265
T 0266
T 0267
T 0269
T 0264
T 0610
T 0438
T 1662
T 1709
T 0370
T 0372
T 0369
T 0228
T 0206
T 1968
T 1073
T 0834
T 1337
T 1172
T 0961
T 1240
T 0655
T 1873
T 1263
T 0965
T 0248
T 0247
T 0182
T 0631
T 1842
T 1110
T 2008
T 1792
T 1779
T 0942
T 1925
T 1906
T 0290
T 0289
T 1591
T 1747
T 1935
T 1092
T 0494
T 0493
T 0495
T 0511
T 0536
T 0189
T 1327
T 1699
T 0373
T 0420
T 1336
T 0889
T 0840
T 1794
T 1514
T 0853

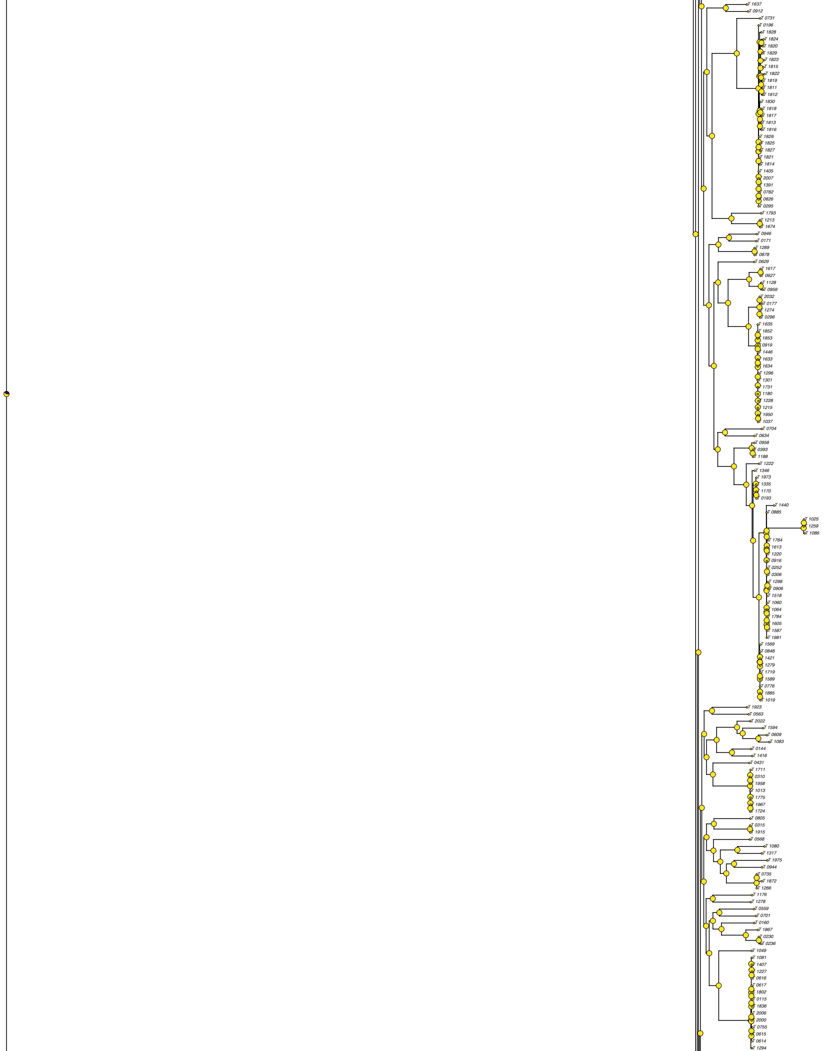
T 1637
T 0912
T 0731
T 0196
T 1828
T 1824
T 1820
T 1829
T 1823
T 1815
T 1822
T 1819
T 1811
T 1812
T 1830
T 1818
T 1817
T 1813
T 1816
T 1826
T 1825
T 1827
T 1821
T 1814
T 1405
2007
1391
0782
0826
0295
T 1793
T 1213
T 1674
T 0946
T 0171
T 1289
0878
T 0629
T 1617
0627
T 1128
T 0959
T 2032
T 0177
1274
0296
T 1635
T 1852
1853
0919
1446
1633
1634
T 1296
1301
1731
1180
1228
1215
1950
1037
T 0704
T 0634
T 0958
0393
1188
T 1222
T 1346
T 1973
1335
1170
0193
T 1440
T 0885
T 1025
1259
T 1086
T 1764
1613
T 1220
0916
T 0252
0306
T 1298
T 0906
T 1518
T 1060
1064
1784
1605
T 1587
T 1981
T 1569
T 0848
1421
1279
T 1719
1589
T 0776
1885
T 1019
T 1923
T 0563
T 2022
T 1594
T 0609
T 1083
T 0144
T 1416
T 0431
T 1711
0310
1958
T 1013
1775
1967
1724
T 0805
0315
1915
T 0568
T 1080
T 1317
T 1975
T 0944
T 0735
T 1872
T 1266
T 1176
T 1278
T 0559
T 0701
T 0160
T 1867
T 0230
T 0236
T 1049
T 1081
1407
1227
0616
T 0617
1802
T 0115
1836
T 2006
2000
T 0755
0615
T 0614
T 1294

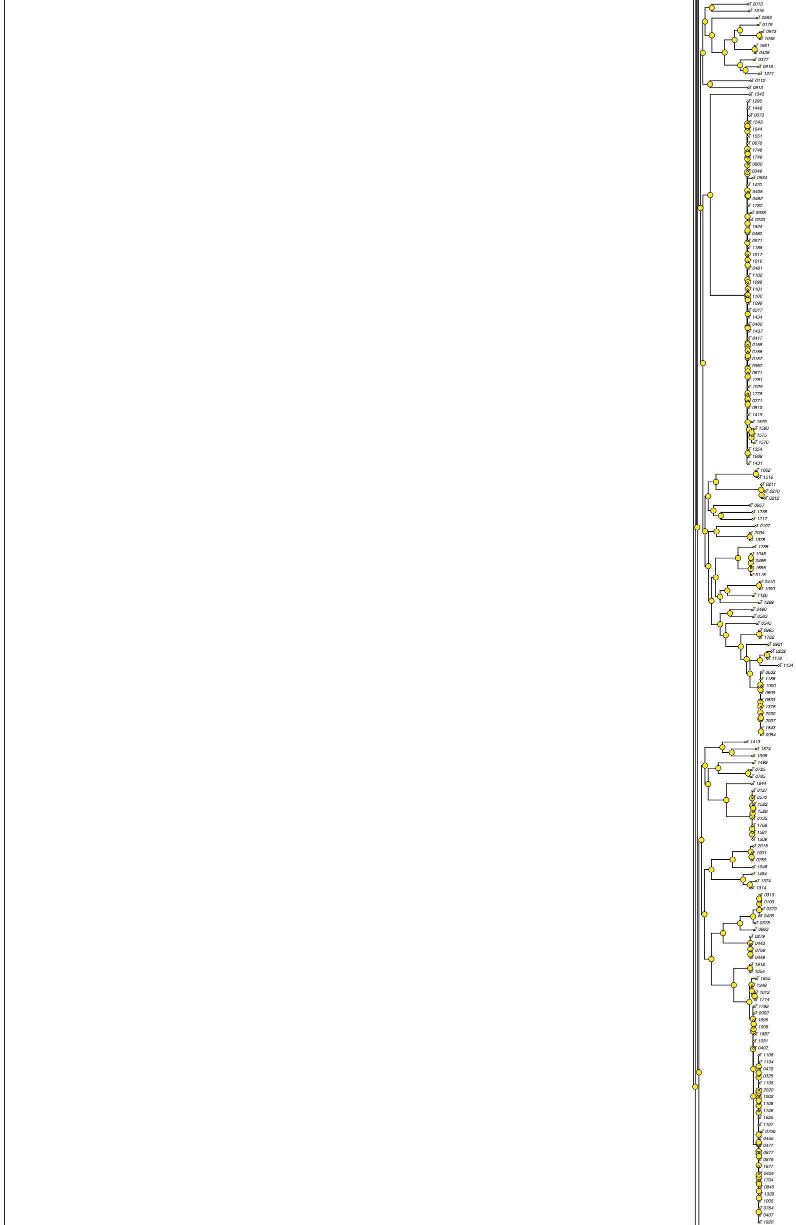
T 2013
T 1316
T 0593
T 0179
T 0973
T 1048
T 1601
T 0428
T 0377
T 0918
T 1271
T 0112
T 0813
T 1343
T 1295
T 1449
T 0073
T 1543
T 1544
T 1551
T 0676
T 1748
T 1749
T 0800
T 0349
T 0534
T 1470
T 0405
T 0482
T 1782
T 0938
T 0233
T 1524
T 0480
T 0971
T 1185
T 1017
T 1016
T 0481
T 1102
T 1098
T 1101
T 1100
T 1099
T 0317
T 1434
T 0400
T 1437
T 0417
T 0158
T 0156
T 0157
T 0952
T 0671
T 1751
T 1929
T 1778
T 0271
T 0810
T 1419
T 1579
T 1580
T 1575
T 1576
T 1354
T 1889
T 1431
T 1062
T 1516
T 0211
T 0210
T 0212
T 0957
T 1236
T 1217
T 0197
T 2034
T 1378
T 1286
T 1948
T 0486
T 1685
T 0118
T 0410
T 1909
T 1126
T 1299
T 0490
T 0583
T 0545
T 0995
T 1702
T 0921
T 0232
T 1178
T 1134
T 0632
T 1186
T 1900
T 0699
T 0633
T 1376
T 2030
T 2037
T 1843
T 0954
T 1413
T 1874
T 1096
T 1499
T 0725
T 0785
T 1844
T 0127
T 0570
T 1522
T 1528
T 0135
T 1768
T 1581
T 1509
T 2015
T 1001
T 0759
T 1046
T 1484
T 1374
T 1314
T 0319
T 0100
T 0379
T 0426
T 0378
T 0963
T 0279
T 0443
T 0769
T 0449
T 1912
T 1054
T 1855
T 1349
T 1012
T 1714
T 1788
T 0902
T 1895
T 1008
T 1887
T 1031
T 0402
T 1106
T 1104
T 0479
T 0325
T 1105
T 2020
T 1002
T 1108
T 1109
T 1625
T 1107
T 0706
T 0455
T 0477
T 0877
T 0876
T 1677
T 0424
T 1704
T 0845
T 1329
T 1005
T 0764
T 0407
T 1920

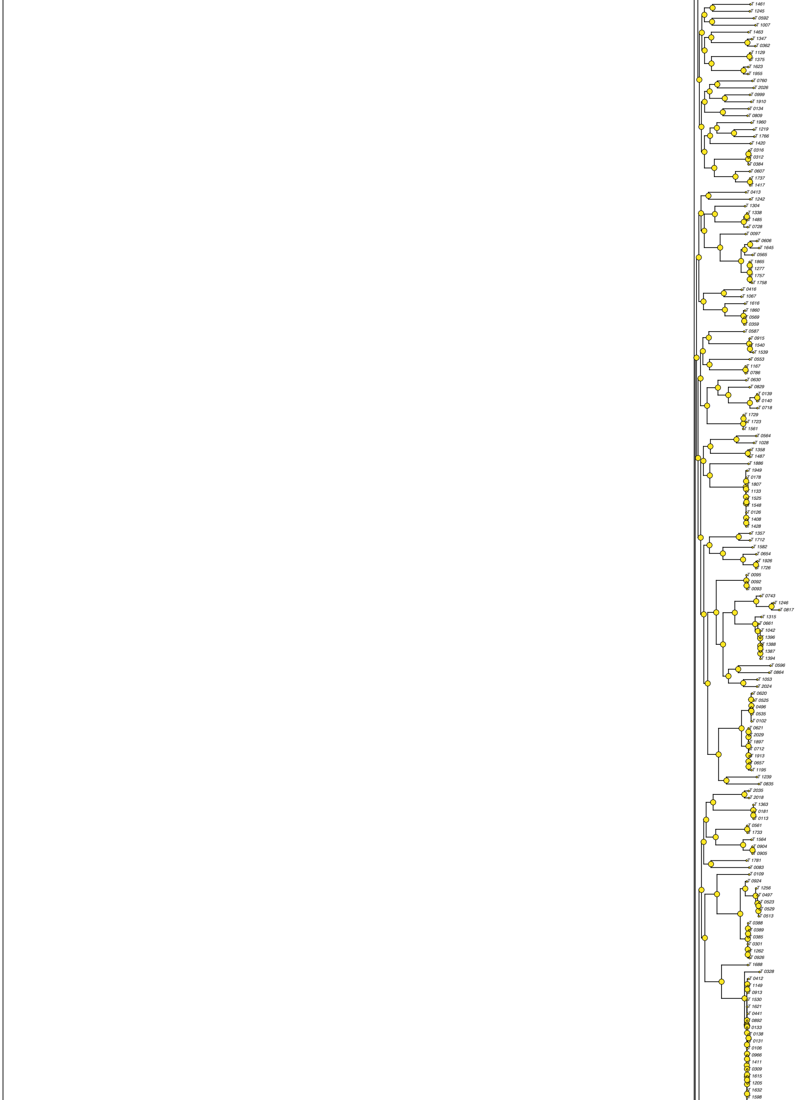
T 1461
T 1245
T 0592
T 1007
T 1463
T 1347
T 0362
T 1129
T 1375
T 1623
T 1955
T 0760
T 2026
T 0999
T 1910
T 0134
T 0809
T 1960
T 1219
T 1766
T 1420
T 0316
T 0312
T 0384
T 0607
T 1737
T 1417
T 0413
T 1242
T 1304
T 1338
T 1485
T 0728
T 0097
T 0606
T 1645
T 0565
T 1865
T 1277
T 1757
T 1758
T 0416
T 1067
T 1616
T 1860
T 0569
T 0359
T 0587
T 0915
T 1540
T 1539
T 0553
T 1167
T 0786
T 0630
T 0829
T 0139
T 0140
T 0718
T 1729
T 1723
T 1561
T 0564
T 1028
T 1358
T 1487
T 1886
T 1949
T 0178
T 1807
T 1133
T 1525
T 1548
T 0126
T 1408
T 1428
T 1357
T 1712
T 1582
T 0654
T 1926
T 1726
T 0095
T 0092
T 0093
T 0743
T 1246
T 0817
T 1315
T 0661
T 1042
T 1396
T 1388
T 1387
T 1394
T 0596
T 0864
T 1053
T 2024
T 0620
T 0525
T 0496
T 0535
T 0102
T 0621
T 2029
T 1897
T 0712
T 1913
T 0657
T 1195
T 1239
T 0835
T 2035
T 2018
T 1363
T 0181
T 0113
T 0561
T 1733
T 1564
T 0904
T 0905
T 1781
T 0083
T 0109
T 0924
T 1256
T 0497
T 0523
T 0529
T 0513
T 0388
T 0389
T 0385
T 0301
T 1262
T 0926
T 1688
T 0328
T 0412
T 1149
T 0913
T 1530
T 1621
T 0441
T 0892
T 0133
T 0138
T 0131
T 0106
T 0966
T 1411
T 0309
T 1615
T 1205
T 1632
T 1598

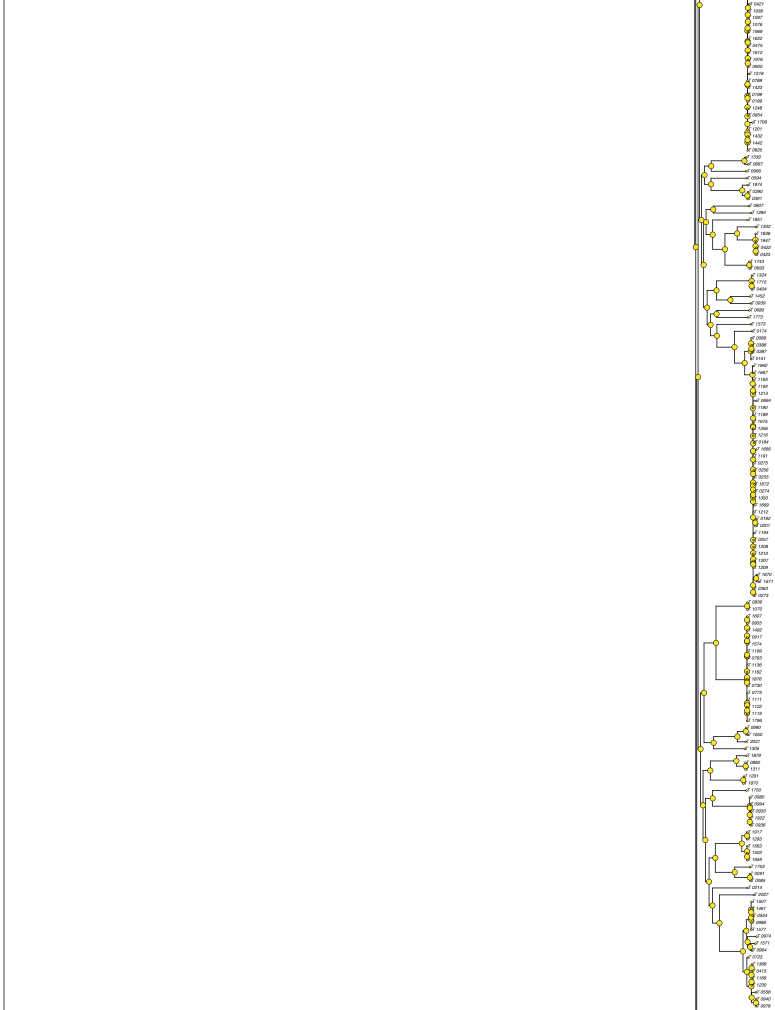
T 0421
T 1938
T 1097
T 1076
T 1969
T 1622
T 0475
T 1612
T 1976
T 0900
T 1318
T 0788
T 1423
T 0198
T 0199
T 1248
T 0854
T 1706
T 1201
T 1432
T 1442
T 0825
T 1339
T 0687
T 0996
T 0594
T 1974
T 0390
T 0391
T 0807
T 1284
T 1851
T 1302
T 1838
T 1847
T 0422
T 0423
T 1743
T 0693
T 1324
T 1715
T 0404
T 1452
T 0939
T 0680
T 1773
T 1573
T 0174
T 0089
T 0386
T 0387
T 0151
T 1962
T 1667
T 1193
T 1192
T 1214
T 0694
T 1190
T 1189
T 1675
T 1356
T 1216
T 0184
T 1666
T 1191
T 0275
T 0256
T 0255
T 1672
T 0274
T 1350
T 1669
T 1212
T 0192
T 0201
T 1194
T 0257
T 1208
T 1210
T 1207
T 1209
T 1670
T 1671
T 0363
T 0273
T 0839
T 1070
T 1607
T 0955
T 1492
T 0917
T 1574
T 1199
T 0763
T 1136
T 1162
T 1876
T 0730
T 0775
T 1111
T 1122
T 1119
T 1796
T 0990
T 1650
T 2031
T 1305
T 1879
T 0882
T 1311
T 1291
T 1870
T 1750
T 0980
T 0994
T 0933
T 1922
T 0936
T 1917
T 1293
T 1563
T 1502
T 1959
T 1753
T 0091
T 0085
T 0214
T 2027
T 1507
T 1481
T 0554
T 0988
T 1577
T 0974
T 1571
T 0964
T 0723
T 1368
T 0415
T 1168
T 1230
T 0558
T 0940
T 0978

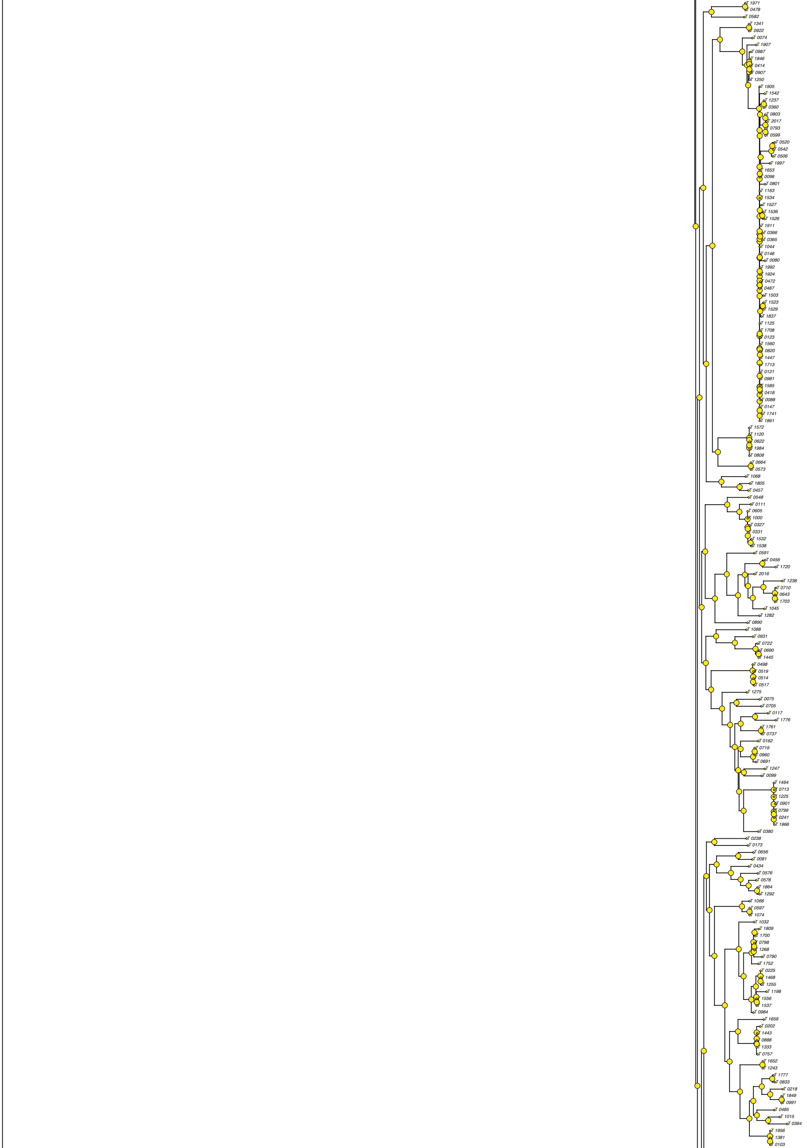
T 1971
T 0478
T 0582
T 1341
T 0922
T 0074
T 1907
T 0987
T 1846
T 0414
T 0907
T 1250
T 1905
T 1542
T 1237
T 0360
T 0803
T 2017
T 0793
T 0599
T 0520
T 0542
T 0506
T 1997
T 1653
T 0096
T 0801
T 1163
T 1534
T 1527
T 1536
T 1526
T 1911
T 0366
T 0365
T 1044
T 0146
T 0080
T 1992
T 1924
T 0472
T 0487
T 1503
T 1523
T 1529
T 1837
T 1125
T 1708
T 0123
T 1560
T 0820
T 1447
T 1713
T 0121
T 0981
T 1585
T 0418
T 0088
T 0147
T 1741
T 1861
T 1572
T 1120
T 0622
T 1984
T 0808
T 0664
T 0573
T 1068
T 1805
T 0457
T 0548
T 0111
T 0605
T 1000
T 0327
T 0331
T 1532
T 1538
T 0591
T 0456
T 1720
T 2016
T 1238
T 0710
T 0643
T 1703
T 1045
T 1282
T 0890
T 1088
T 0931
T 0722
T 0690
T 1445
T 0498
T 0519
T 0514
T 0517
T 1275
T 0075
T 0705
T 0117
T 1776
T 1761
T 0737
T 0162
T 0719
T 0960
T 0691
T 1247
T 0099
T 1464
T 0713
T 1225
T 0901
T 0799
T 0241
T 1966
T 0380
T 0238
T 0173
T 0656
T 0081
T 0434
T 0576
T 0578
T 1864
T 1292
T 1066
T 0597
T 1074
T 1032
T 1809
T 1700
T 0798
T 1268
T 0790
T 1752
T 0225
T 1468
T 1255
T 1198
T 1556
T 1537
T 0984
T 1659
T 0202
T 1443
T 0888
T 1333
T 0757
T 1652
T 1243
T 1777
T 0833
T 0218
T 1849
T 0991
T 0485
T 1015
T 0364
T 1956
T 1381
T 0103

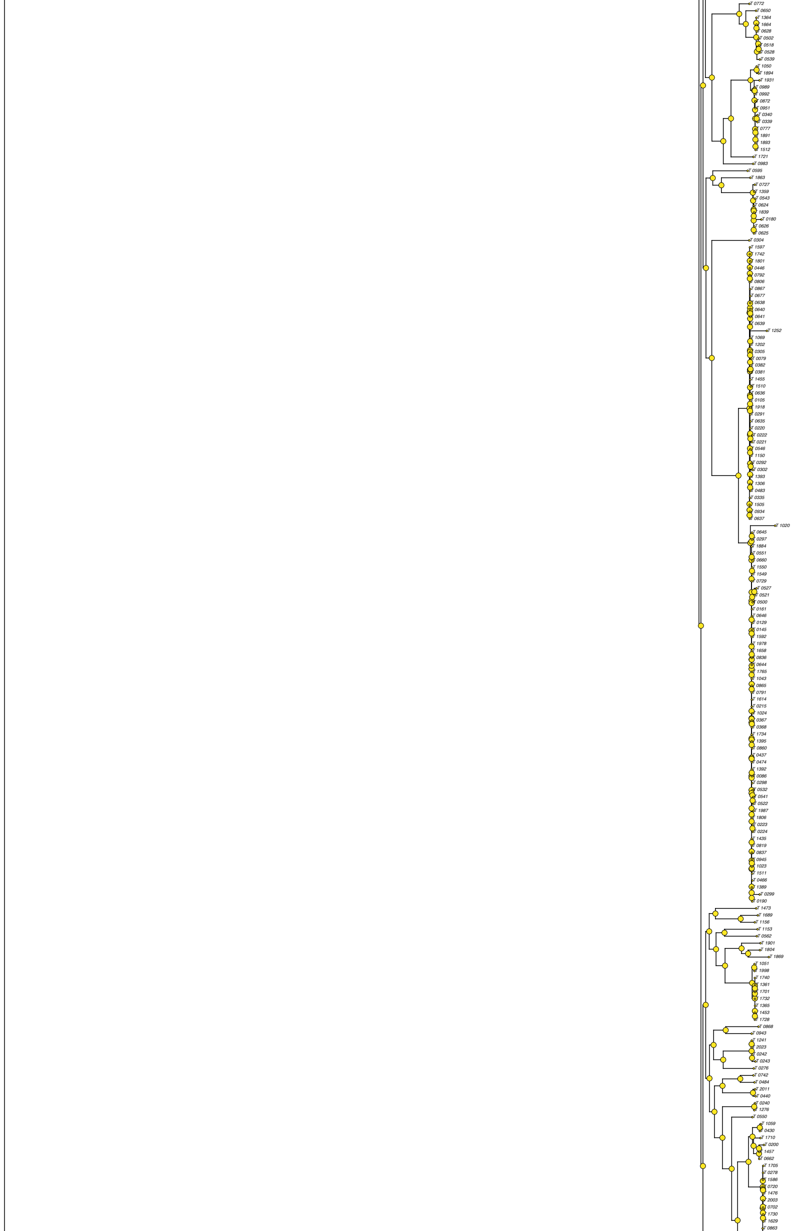
T 0772
T 0650
T 1364
T 1664
T 0628
T 0502
T 0518
T 0528
T 0539
T 1050
T 1894
T 1931
T 0989
T 0992
T 0872
T 0951
T 0340
T 0339
T 0777
T 1891
T 1893
T 1512
T 1721
T 0983
T 0595
T 1863
T 0727
T 1359
T 0543
T 0624
T 1839
T 0180
T 0626
T 0625
T 0304
T 1597
T 1742
T 1801
T 0446
T 0792
T 0806
T 0867
T 0677
T 0638
T 0640
T 0641
T 0639
T 1252
T 1069
T 1202
T 0305
T 0079
T 0382
T 0381
T 1455
T 1510
T 0636
T 0105
T 1918
T 0291
T 0635
T 0220
T 0222
T 0221
T 0546
T 1150
T 0292
T 0302
T 1393
T 1306
T 0483
T 0335
T 1505
T 0934
T 0637
T 1020
T 0645
T 0297
T 1884
T 0551
T 0660
T 1550
T 1549
T 0729
T 0527
T 0521
T 0500
T 0161
T 0646
T 0129
T 0145
T 1592
T 1978
T 1658
T 0836
T 0644
T 1765
T 1043
T 0865
T 0791
T 1614
T 0215
T 1024
T 0367
T 0368
T 1734
T 1395
T 0860
T 0437
T 0474
T 1392
T 0086
T 0298
T 0532
T 0541
T 0522
T 1987
T 1806
T 0223
T 0224
T 1435
T 0819
T 0837
T 0945
T 1023
T 1511
T 0466
T 1389
T 0299
T 0190
T 1473
T 1689
T 1156
T 1153
T 0562
T 1901
T 1804
T 1869
T 1051
T 1998
T 1740
T 1361
T 1701
T 1732
T 1365
T 1453
T 1728
T 0868
T 0943
T 1241
T 2023
T 0242
T 0243
T 0276
T 0742
T 0484
T 2011
T 0440
T 0240
T 1276
T 0550
T 1059
T 0430
T 1710
T 0200
T 1457
T 0662
T 1705
T 0278
T 1586
T 0720
T 1476
T 2003
T 0702
T 1730
T 1629
T 0863

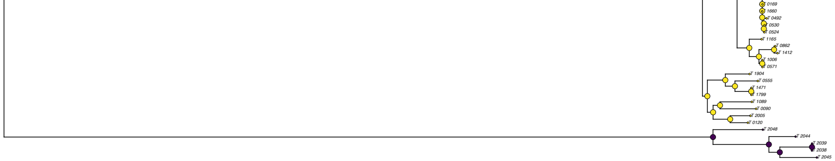
T 0169
T 1660
T 0492
T 0530
T 0524
T 1165
T 0862
T 1412
T 1006
T 0571
T 1904
T 0555
T 1471
T 1799
T 1089
T 0090
T 2005
T 0120
T 2048
T 2044
T 2039
T 2038
T 2045

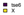

Effector gene: tse6

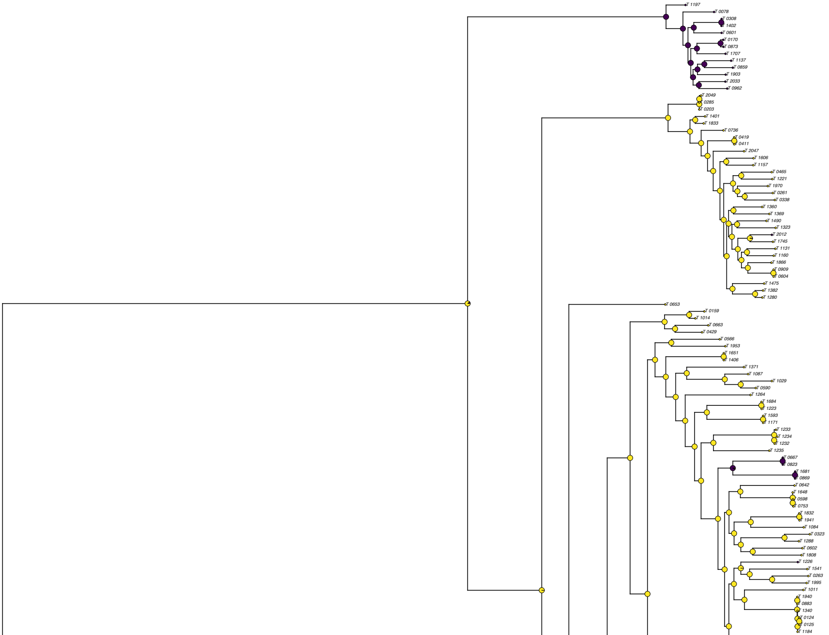

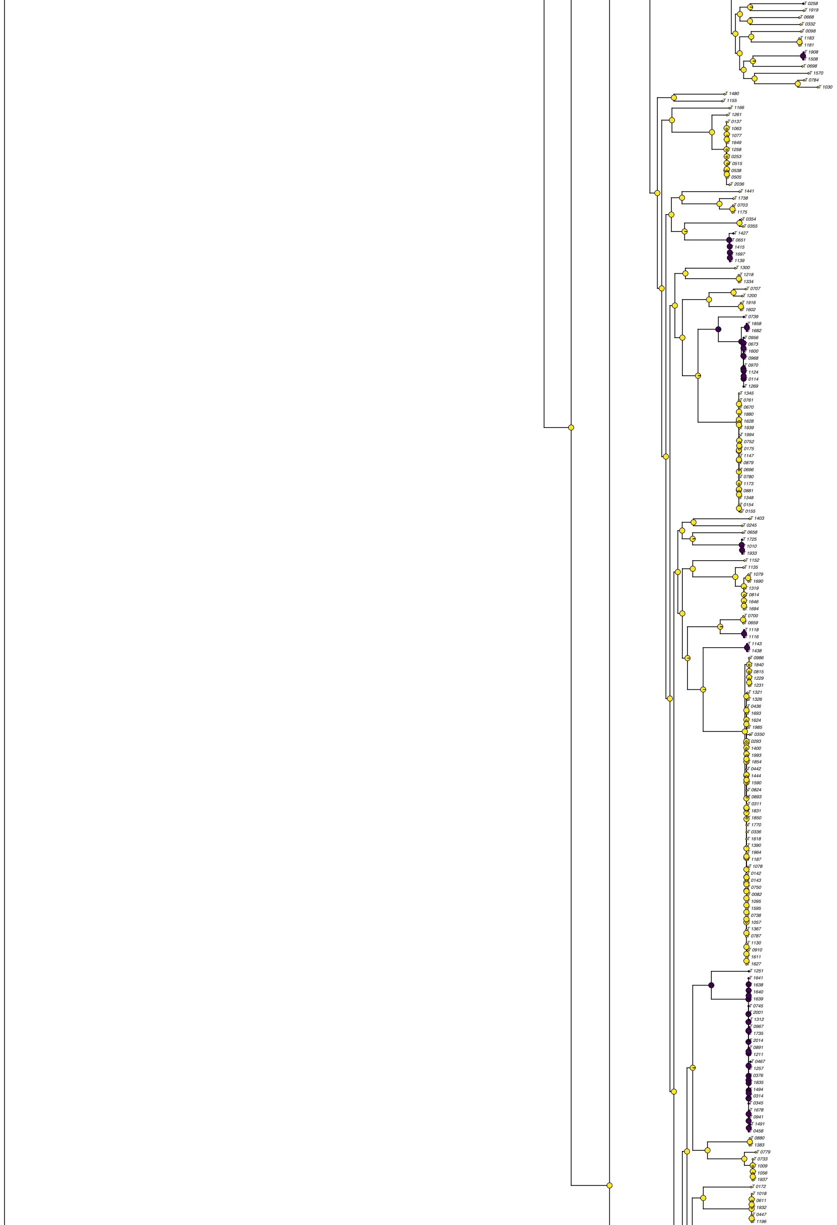

T 0258
T 1919
T 0668
T 0332
T 0098
T 1183
T 1181
T 1908
T 1508
T 0698
T 1570
T 0784
T 1030
T 1480
T 1155
T 1166
T 1261
T 0137
1063
1077
1649
1258
0253
0515
0538
0505
T 2036
T 1441
T 1738
T 0703
T 1175
T 0354
T 0355
T 1427
T 0651
1415
1697
1139
T 1300
T 1218
1334
T 0707
T 1200
T 1916
T 1602
T 0739
T 1858
T 1682
T 0956
0673
1600
0968
T 0970
1124
0114
T 1269
T 1345
T 0761
0670
1880
1628
1939
T 1994
0752
0175
T 1147
0879
T 0696
T 0780
1173
0881
T 1348
0154
T 0155
T 1403
T 0245
T 0658
T 1725
1010
T 1933
T 1152
T 1135
T 1079
T 1690
1319
0814
1646
1694
T 0700
0659
T 1118
T 1116
T 1143
T 1438
T 0986
1840
0815
1229
1231
T 1321
T 1326
T 0436
1693
1624
T 1985
T 0350
0293
1400
1993
1854
T 0442
1444
1590
T 0824
0893
T 0311
1831
1850
1770
0336
T 1618
T 1390
1964
1187
T 1078
0142
0143
0750
0082
1095
1595
T 0738
1057
T 1367
0787
T 1130
0910
1611
1627
T 1251
T 1641
1638
1640
1639
T 0745
2001
T 1312
T 0967
T 1735
2014
T 0891
1211
T 0467
1257
0376
1835
1494
0314
T 0345
T 1678
0941
T 1491
0458
T 0880
1383
T 0779
T 0733
1009
1056
1937
T 0172
T 1018
0611
1932
T 0447
1196

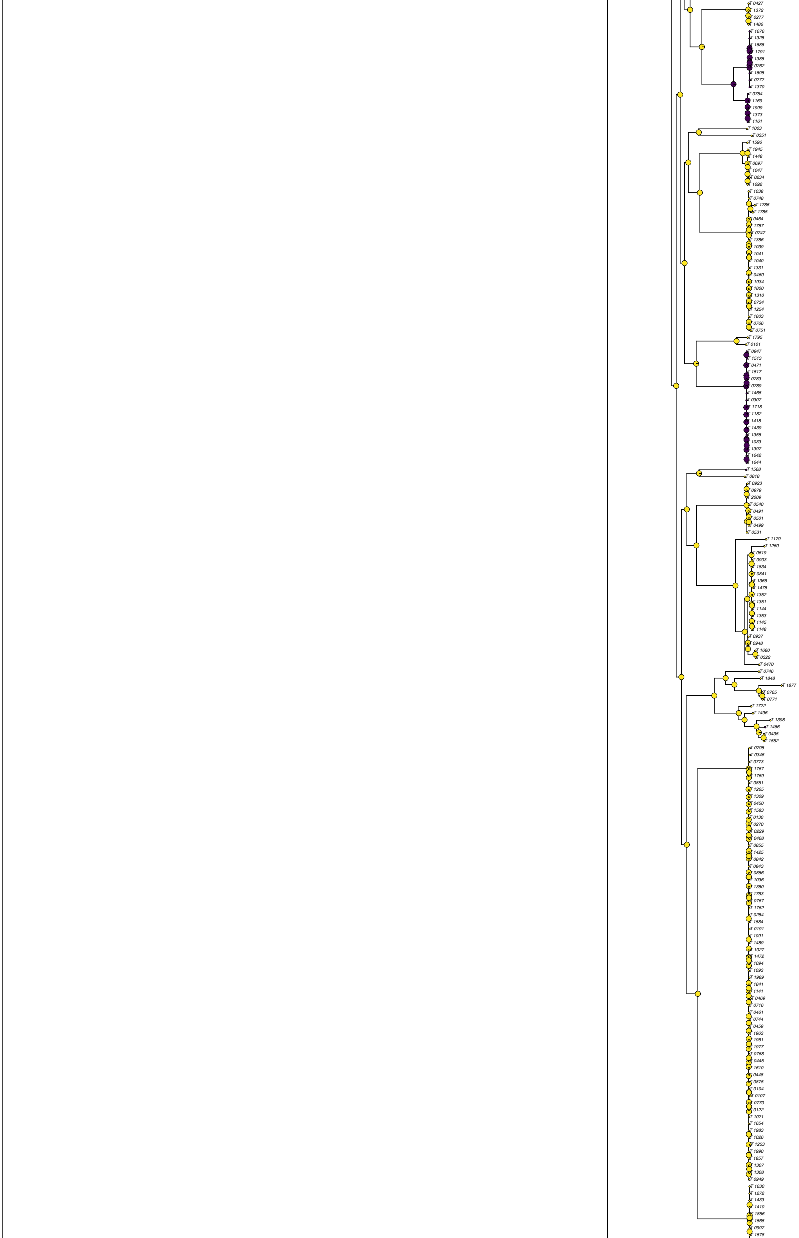
T 0427
T 1372
T 0277
T 1486
T 1676
T 1328
T 1686
T 1791
T 1385
T 0262
T 1695
T 0272
T 1370
T 0754
T 1169
T 1999
T 1373
T 1161
T 1003
T 0351
T 1596
T 1945
T 1448
T 0697
T 1047
T 0234
T 1692
T 1038
T 0748
T 1786
T 1785
T 0464
T 1787
T 0747
T 1386
T 1039
T 1041
T 1040
T 1331
T 0460
T 1934
T 1800
T 1310
T 0734
T 1254
T 1803
T 0766
T 0751
T 1795
T 0101
T 0947
T 1513
T 0471
T 1517
T 0783
T 0789
T 1465
T 0307
T 1718
T 1182
T 1418
T 1439
T 1355
T 1033
T 1397
T 1642
T 1644
T 1568
T 0818
T 0923
T 0979
T 2009
T 0540
T 0491
T 0501
T 0499
T 0531
T 1179
T 1260
T 0619
T 0903
T 1834
T 0841
T 1366
T 1478
T 1352
T 1351
T 1144
T 1353
T 1145
T 1148
T 0937
T 0948
T 1680
T 0322
T 0470
T 0746
T 1848
T 1877
T 0765
T 0771
T 1722
T 1496
T 1398
T 1466
T 0435
T 1552
T 0795
T 0346
T 0773
T 1767
T 1769
T 0851
T 1265
T 1309
T 0450
T 1583
T 0130
T 0270
T 0229
T 0468
T 0855
T 1425
T 0842
T 0843
T 0856
T 1036
T 1380
T 1763
T 0767
T 1762
T 0284
T 1584
T 0191
T 1091
T 1489
T 1027
T 1472
T 1094
T 1093
T 1989
T 1841
T 1141
T 0469
T 0716
T 0461
T 0744
T 0459
T 1963
T 1961
T 1977
T 0768
T 0445
T 1610
T 0448
T 0875
T 0104
T 0107
T 0770
T 0122
T 1021
T 1654
T 1983
T 1026
T 1253
T 1990
T 1857
T 1307
T 1308
T 0949
T 1630
T 1272
T 1433
T 1410
T 1856
T 1565
T 0997
T 1578

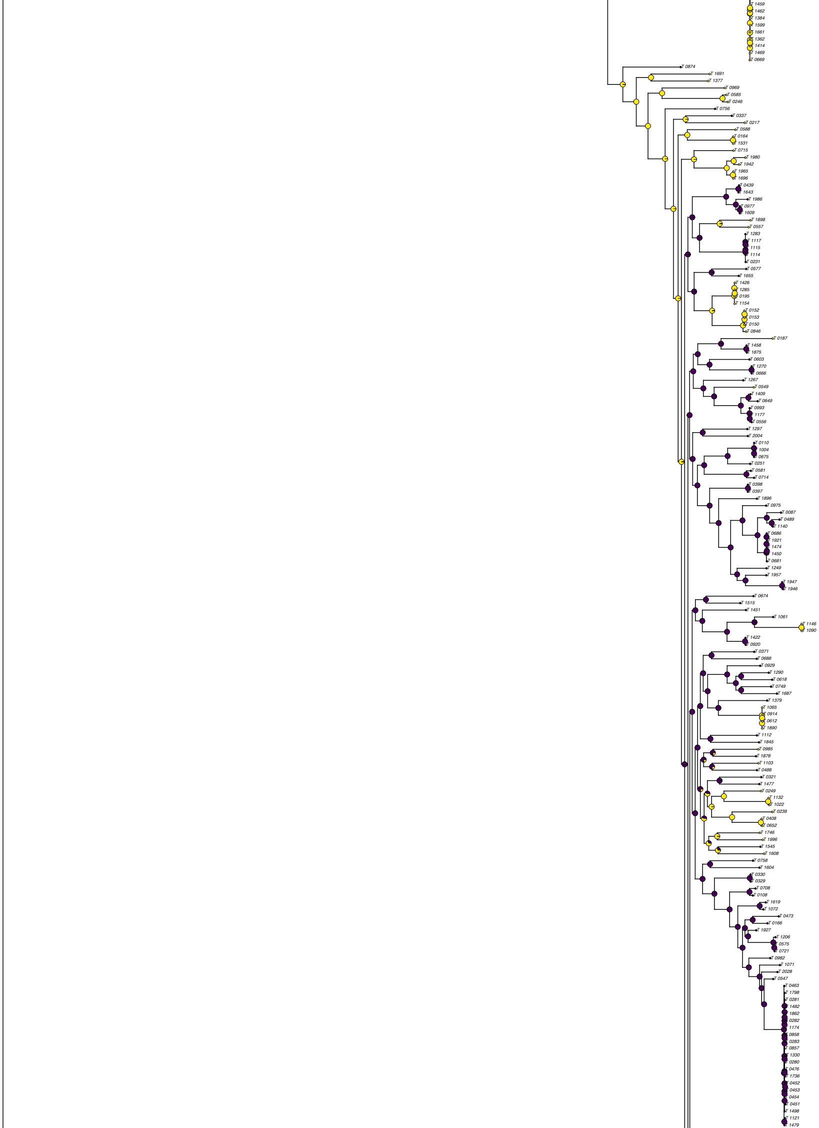
T 1459
T 1462
T 1384
T 1599
T 1661
T 1362
T 1414
T 1469
T 0669
T 0874
T 1691
T 1377
T 0969
T 0585
T 0246
T 0756
T 0337
T 0217
T 0588
T 0164
T 1531
T 0715
T 1980
T 1942
T 1965
T 1696
T 0439
T 1643
T 1986
T 0977
T 1609
T 1898
T 0557
T 1283
T 1117
T 1115
T 1114
T 0231
T 0577
T 1655
T 1426
T 1285
T 0195
T 1154
T 0152
T 0153
T 0150
T 0846
T 0187
T 1458
T 1875
T 0603
T 1270
T 0666
T 1267
T 0549
T 1409
T 0649
T 0993
T 1177
T 0556
T 1297
T 2004
T 0110
T 1004
T 0675
T 0251
T 0581
T 0714
T 0398
T 0397
T 1896
T 0975
T 0087
T 0489
T 1140
T 0686
T 1921
T 1474
T 1450
T 0681
T 1249
T 1957
T 1947
T 1946
T 0674
T 1515
T 1451
T 1061
T 1146
T 1090
T 1422
T 0920
T 0371
T 0688
T 0929
T 1290
T 0618
T 0749
T 1687
T 1379
T 1065
T 0914
T 0612
T 1890
T 1112
T 1845
T 0985
T 1878
T 1103
T 0488
T 0321
T 1477
T 0249
T 1132
T 1022
T 0239
T 0408
T 0652
T 1746
T 1996
T 1545
T 1608
T 0758
T 1604
T 0330
T 0329
T 0708
T 0108
T 1619
T 1072
T 0473
T 0166
T 1927
T 1206
T 0575
T 0721
T 0982
T 1071
T 2028
T 0547
T 0463
T 1798
T 0281
T 1482
T 1862
T 0282
T 1174
T 0858
T 0283
T 0857
T 1330
T 0280
T 0476
T 1736
T 0452
T 0453
T 0454
T 0451
T 1498
T 1121
T 1479

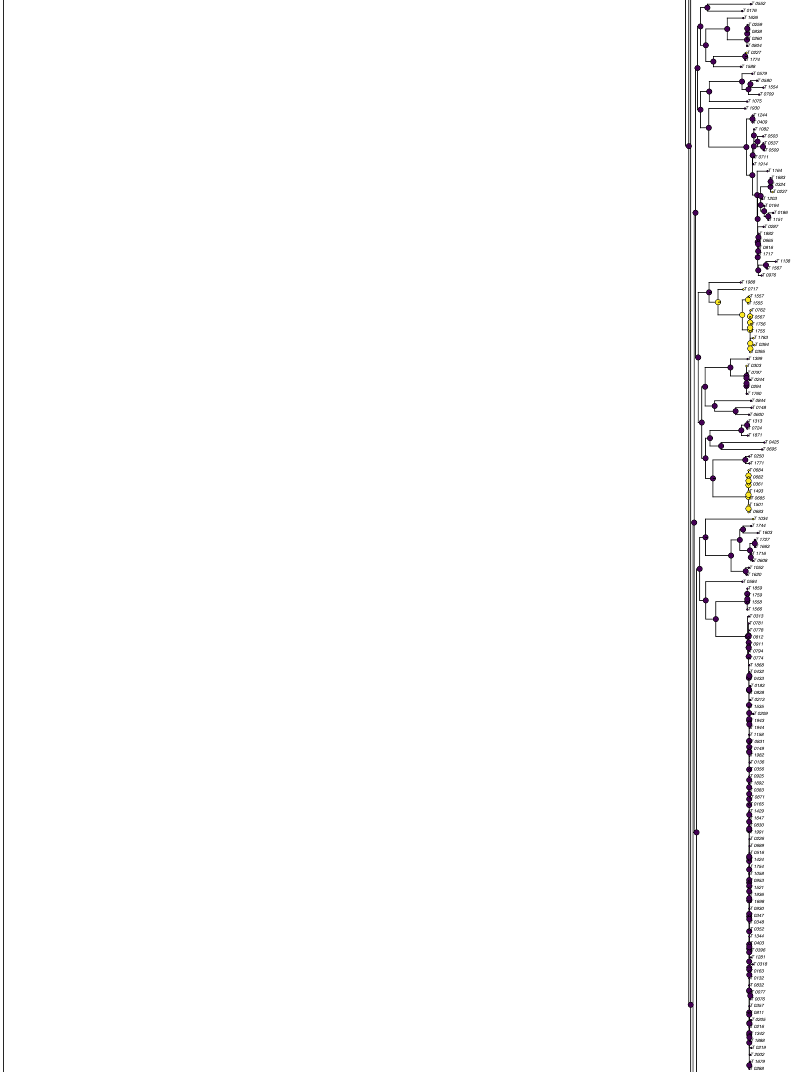
T 0552
T 0176
T 1626
T 0259
0838
0260
T 0804
T 0227
T 1774
T 1588
T 0579
T 0580
T 1554
T 0709
T 1075
T 1930
T 1244
T 0409
T 1082
T 0503
T 0537
T 0509
T 0711
T 1914
T 1164
T 1683
0324
T 0237
T 1203
T 0194
T 0186
T 1151
T 0287
T 1882
0665
T 0816
1717
T 1138
T 1567
T 0976
T 1988
T 0717
T 1557
T 1555
T 0762
0567
T 1756
T 1755
T 1783
T 0394
0395
T 1399
T 0303
T 0797
T 0244
0294
T 1760
T 0844
T 0148
T 0600
T 1313
T 0724
T 1871
T 0425
T 0695
T 0250
T 1771
T 0684
0682
T 0361
T 1493
T 0685
T 1501
T 0683
T 1034
T 1744
T 1603
T 1727
T 1663
T 1716
T 0608
T 1052
T 1620
T 0584
T 1859
1759
1558
T 1566
T 0313
T 0781
T 0778
0812
0911
T 0794
0774
T 1868
T 0432
0433
T 0183
T 0828
T 0213
1535
T 0209
1943
T 1944
T 1158
0831
0149
T 1982
T 0136
T 0356
T 0925
T 1892
0383
T 0871
0165
T 1429
T 1647
T 0830
T 1991
T 0226
T 0689
T 0516
1424
T 1754
T 1058
0953
T 1521
T 1936
T 1698
T 0930
0347
T 0348
T 0352
T 1344
T 0403
T 0396
T 1281
T 0318
0163
T 0132
T 0832
T 0077
T 0076
T 0357
0811
T 0205
T 0216
T 1342
T 1888
T 0219
T 2002
T 1679
T 0288

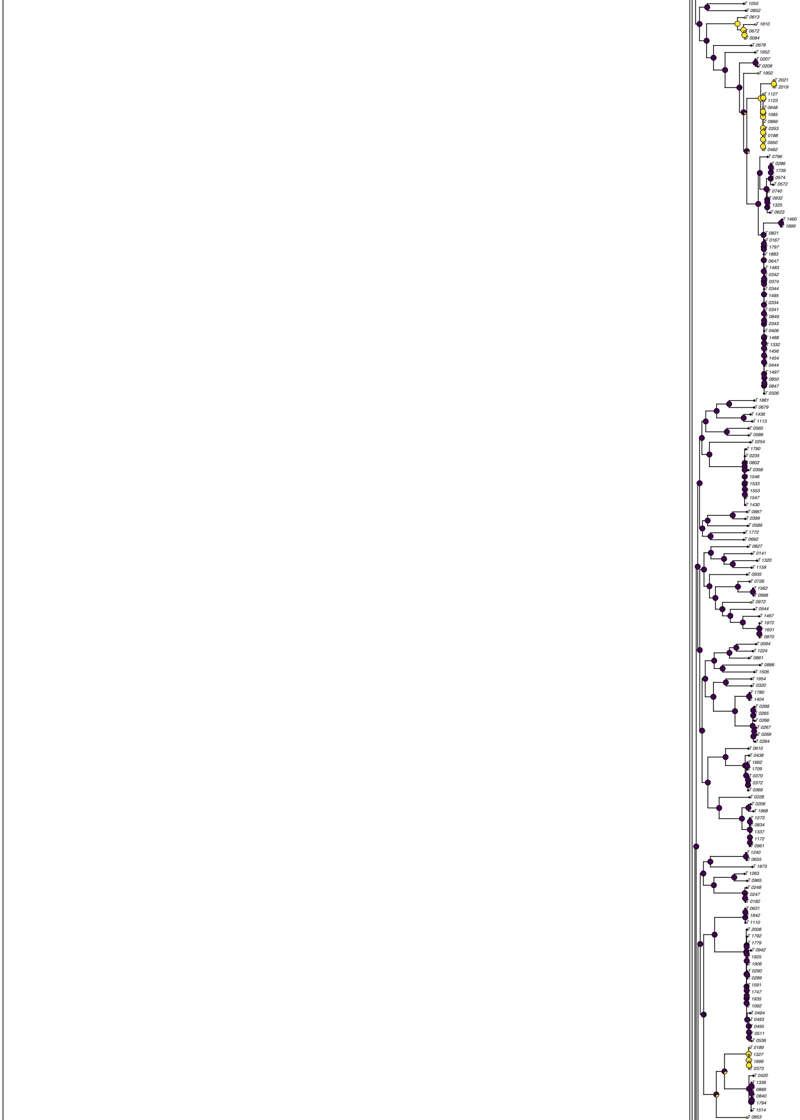
T 1055
T 0852
T 0613
T 1810
T 0672
T 0084
T 0678
T 1952
T 0207
T 0208
T 1902
T 2021
T 2019
T 1127
T 1123
T 0648
T 1085
T 0866
T 0353
T 0188
T 0950
T 0462
T 0796
T 0286
T 1739
T 0574
T 0572
T 0740
T 0932
T 1325
T 0623
T 1460
T 1899
T 0821
T 0167
T 1797
T 1883
T 0647
T 1483
T 0342
T 0374
T 0344
T 1495
T 0334
T 0341
T 0849
T 0343
T 0406
T 1488
T 1332
T 1456
T 1454
T 0444
T 1497
T 0850
T 0847
T 0326
T 1881
T 0679
T 1436
T 1113
T 0560
T 0586
T 0254
T 1790
T 0235
T 0802
T 0358
T 1546
T 1533
T 1553
T 1547
T 1430
T 0887
T 0399
T 0589
T 1772
T 0692
T 0827
T 0141
T 1320
T 1159
T 0935
T 0726
T 1562
T 0998
T 0972
T 0544
T 1467
T 1972
T 1631
T 0870
T 0094
T 1224
T 0861
T 0886
T 1506
T 1954
T 0320
T 1780
T 1404
T 0268
T 0265
T 0266
T 0267
T 0269
T 0264
T 0610
T 0438
T 1662
T 1709
T 0370
T 0372
T 0369
T 0228
T 0206
T 1968
T 1073
T 0834
T 1337
T 1172
T 0961
T 1240
T 0655
T 1873
T 1263
T 0965
T 0248
T 0247
T 0182
T 0631
T 1842
T 1110
T 2008
T 1792
T 1779
T 0942
T 1925
T 1906
T 0290
T 0289
T 1591
T 1747
T 1935
T 1092
T 0494
T 0493
T 0495
T 0511
T 0536
T 0189
T 1327
T 1699
T 0373
T 0420
T 1336
T 0889
T 0840
T 1794
T 1514
T 0853

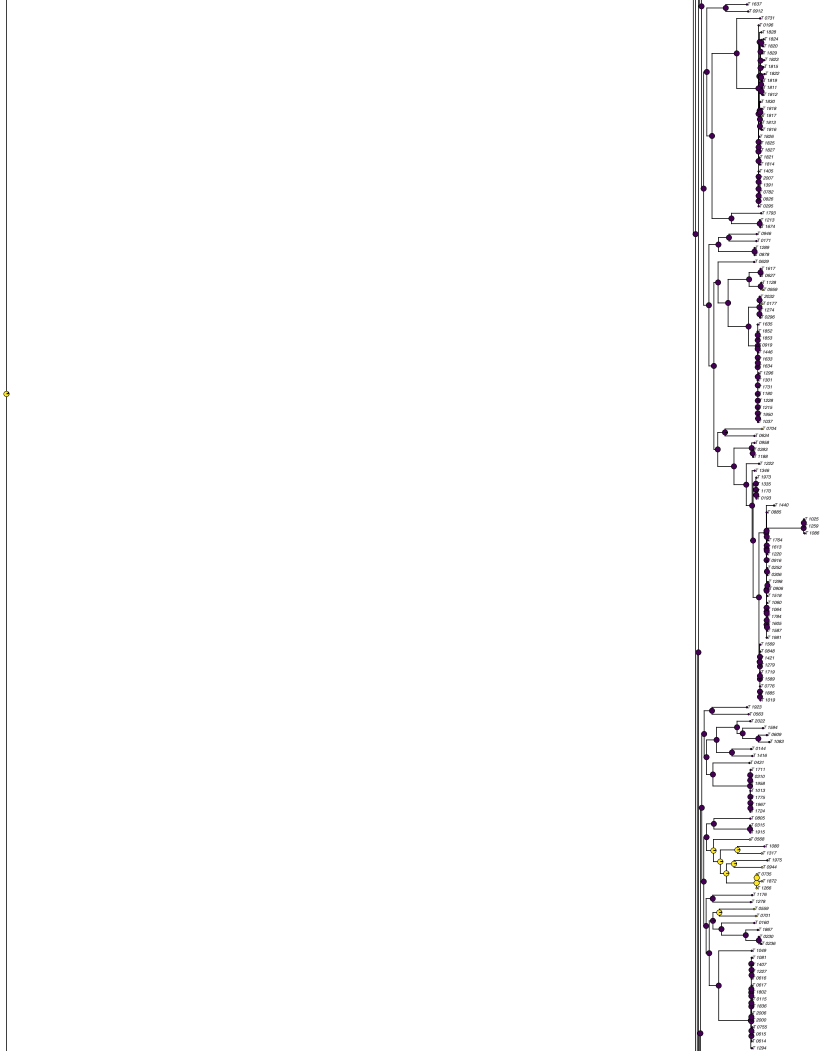
T 1637
T 0912
T 0731
T 0196
T 1828
T 1824
T 1820
T 1829
T 1823
T 1815
T 1822
T 1819
T 1811
T 1812
T 1830
T 1818
T 1817
T 1813
T 1816
T 1826
T 1825
T 1827
T 1821
T 1814
T 1405
T 2007
T 1391
T 0782
T 0826
T 0295
T 1793
T 1213
T 1674
T 0946
T 0171
T 1289
T 0878
T 0629
T 1617
T 0627
T 1128
T 0959
T 2032
T 0177
T 1274
T 0296
T 1635
T 1852
T 1853
T 0919
T 1446
T 1633
T 1634
T 1296
T 1301
T 1731
T 1180
T 1228
T 1215
T 1950
T 1037
T 0704
T 0634
T 0958
T 0393
T 1188
T 1222
T 1346
T 1973
T 1335
T 1170
T 0193
T 1440
T 0885
T 1025
T 1259
T 1086
T 1764
T 1613
T 1220
T 0916
T 0252
T 0306
T 1298
T 0906
T 1518
T 1060
T 1064
T 1784
T 1605
T 1587
T 1981
T 1569
T 0848
T 1421
T 1279
T 1719
T 1589
T 0776
T 1885
T 1019
T 1923
T 0563
T 2022
T 1594
T 0609
T 1083
T 0144
T 1416
T 0431
T 1711
T 0310
T 1958
T 1013
T 1775
T 1967
T 1724
T 0805
T 0315
T 1915
T 0568
T 1080
T 1317
T 1975
T 0944
T 0735
T 1872
T 1266
T 1176
T 1278
T 0559
T 0701
T 0160
T 1867
T 0230
T 0236
T 1049
T 1081
T 1407
T 1227
T 0616
T 0617
T 1802
T 0115
T 1836
T 2006
T 2000
T 0755
T 0615
T 0614
T 1294

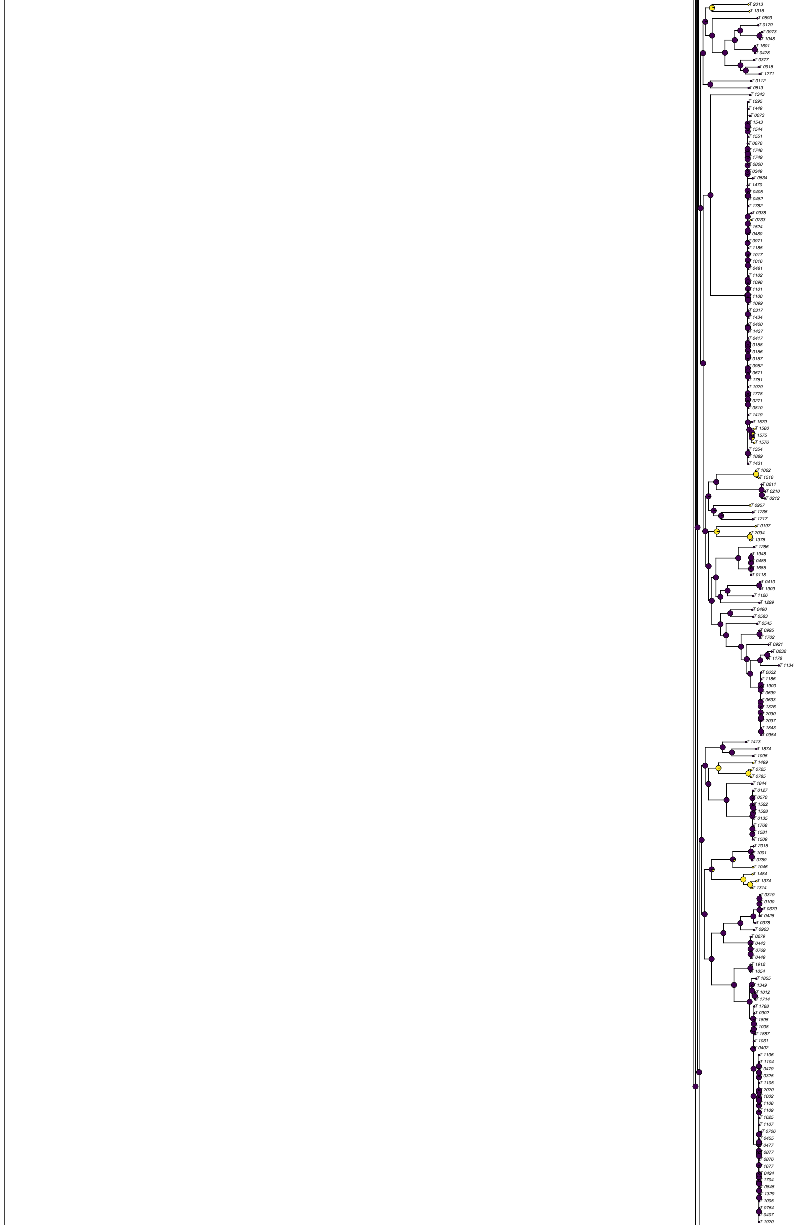
T 2013
T 1316
T 0593
T 0179
T 0973
T 1048
T 1601
T 0428
T 0377
T 0918
T 1271
T 0112
T 0813
T 1343
T 1295
T 1449
T 0073
T 1543
T 1544
T 1551
T 0676
T 1748
T 1749
T 0800
T 0349
T 0534
T 1470
T 0405
T 0482
T 1782
T 0938
T 0233
T 1524
T 0480
T 0971
T 1185
T 1017
T 1016
T 0481
T 1102
T 1098
T 1101
T 1100
T 1099
T 0317
T 1434
T 0400
T 1437
T 0417
T 0158
T 0156
T 0157
T 0952
T 0671
T 1751
T 1929
T 1778
T 0271
T 0810
T 1419
T 1579
T 1580
T 1575
T 1576
T 1354
T 1889
T 1431
T 1062
T 1516
T 0211
T 0210
T 0212
T 0957
T 1236
T 1217
T 0197
T 2034
T 1378
T 1286
T 1948
T 0486
T 1685
T 0118
T 0410
T 1909
T 1126
T 1299
T 0490
T 0583
T 0545
T 0995
T 1702
T 0921
T 0232
T 1178
T 1134
T 0632
T 1186
T 1900
T 0699
T 0633
T 1376
T 2030
T 2037
T 1843
T 0954
T 1413
T 1874
T 1096
T 1499
T 0725
T 0785
T 1844
T 0127
T 0570
T 1522
T 1528
T 0135
T 1768
T 1581
T 1509
T 2015
T 1001
T 0759
T 1046
T 1484
T 1374
T 1314
T 0319
T 0100
T 0379
T 0426
T 0378
T 0963
T 0279
T 0443
T 0769
T 0449
T 1912
T 1054
T 1855
T 1349
T 1012
T 1714
T 1788
T 0902
T 1895
T 1008
T 1887
T 1031
T 0402
T 1106
T 1104
T 0479
T 0325
T 1105
T 2020
T 1002
T 1108
T 1109
T 1625
T 1107
T 0706
T 0455
T 0477
T 0877
T 0876
T 1677
T 0424
T 1704
T 0845
T 1329
T 1005
T 0764
T 0407
T 1920

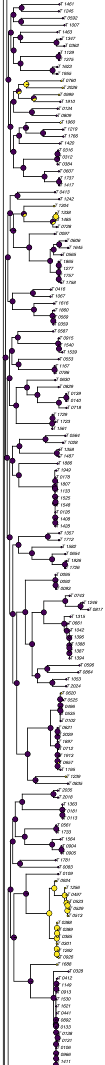
T 1461
T 1245
T 0592
T 1007
T 1463
T 1347
T 0362
T 1129
T 1375
T 1623
T 1955
T 0760
T 2026
T 0999
T 1910
T 0134
T 0809
T 1960
T 1219
T 1766
T 1420
T 0316
T 0312
T 0384
T 0607
T 1737
T 1417
T 0413
T 1242
T 1304
T 1338
T 1485
T 0728
T 0097
T 0606
T 1645
T 0565
T 1865
T 1277
T 1757
T 1758
T 0416
T 1067
T 1616
T 1860
T 0569
T 0359
T 0587
T 0915
T 1540
T 1539
T 0553
T 1167
T 0786
T 0630
T 0829
T 0139
T 0140
T 0718
T 1729
T 1723
T 1561
T 0564
T 1028
T 1358
T 1487
T 1886
T 1949
T 0178
T 1807
T 1133
T 1525
T 1548
T 0126
T 1408
T 1428
T 1357
T 1712
T 1582
T 0654
T 1926
T 1726
T 0095
T 0092
T 0093
T 0743
T 1246
T 0817
T 1315
T 0661
T 1042
T 1396
T 1388
T 1387
T 1394
T 0596
T 0864
T 1053
T 2024
T 0620
T 0525
T 0496
T 0535
T 0102
T 0621
T 2029
T 1897
T 0712
T 1913
T 0657
T 1195
T 1239
T 0835
T 2035
T 2018
T 1363
T 0181
T 0113
T 0561
T 1733
T 1564
T 0904
T 0905
T 1781
T 0083
T 0109
T 0924
T 1256
T 0497
T 0523
T 0529
T 0513
T 0388
T 0389
T 0385
T 0301
T 1262
T 0926
T 1688
T 0328
T 0412
T 1149
T 0913
T 1530
T 1621
T 0441
T 0892
T 0133
T 0138
T 0131
T 0106
T 0966
T 1411

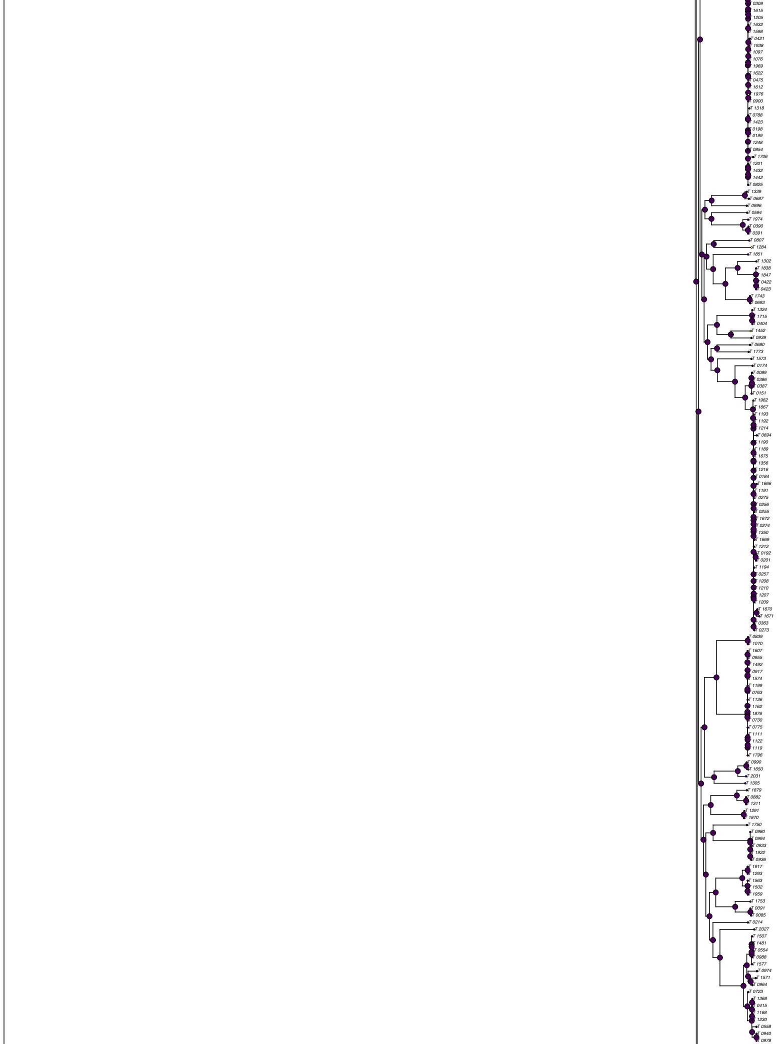
T 0309
T 1615
T 1205
T 1632
T 1598
T 0421
T 1938
T 1097
T 1076
T 1969
T 1622
T 0475
T 1612
T 1976
T 0900
T 1318
T 0788
T 1423
T 0198
T 0199
T 1248
T 0854
T 1706
T 1201
T 1432
T 1442
T 0825
T 1339
T 0687
T 0996
T 0594
T 1974
T 0390
T 0391
T 0807
T 1284
T 1851
T 1302
T 1838
T 1847
T 0422
T 0423
T 1743
T 0693
T 1324
T 1715
T 0404
T 1452
T 0939
T 0680
T 1773
T 1573
T 0174
T 0089
T 0386
T 0387
T 0151
T 1962
T 1667
T 1193
T 1192
T 1214
T 0694
T 1190
T 1189
T 1675
T 1356
T 1216
T 0184
T 1666
T 1191
T 0275
T 0256
T 0255
T 1672
T 0274
T 1350
T 1669
T 1212
T 0192
T 0201
T 1194
T 0257
T 1208
T 1210
T 1207
T 1209
T 1670
T 1671
T 0363
T 0273
T 0839
T 1070
T 1607
T 0955
T 1492
T 0917
T 1574
T 1199
T 0763
T 1136
T 1162
T 1876
T 0730
T 0775
T 1111
T 1122
T 1119
T 1796
T 0990
T 1650
T 2031
T 1305
T 1879
T 0882
T 1311
T 1291
T 1870
T 1750
T 0980
T 0994
T 0933
T 1922
T 0936
T 1917
T 1293
T 1563
T 1502
T 1959
T 1753
T 0091
T 0085
T 0214
T 2027
T 1507
T 1481
T 0554
T 0988
T 1577
T 0974
T 1571
T 0964
T 0723
T 1368
T 0415
T 1168
T 1230
T 0558
T 0940
T 0978

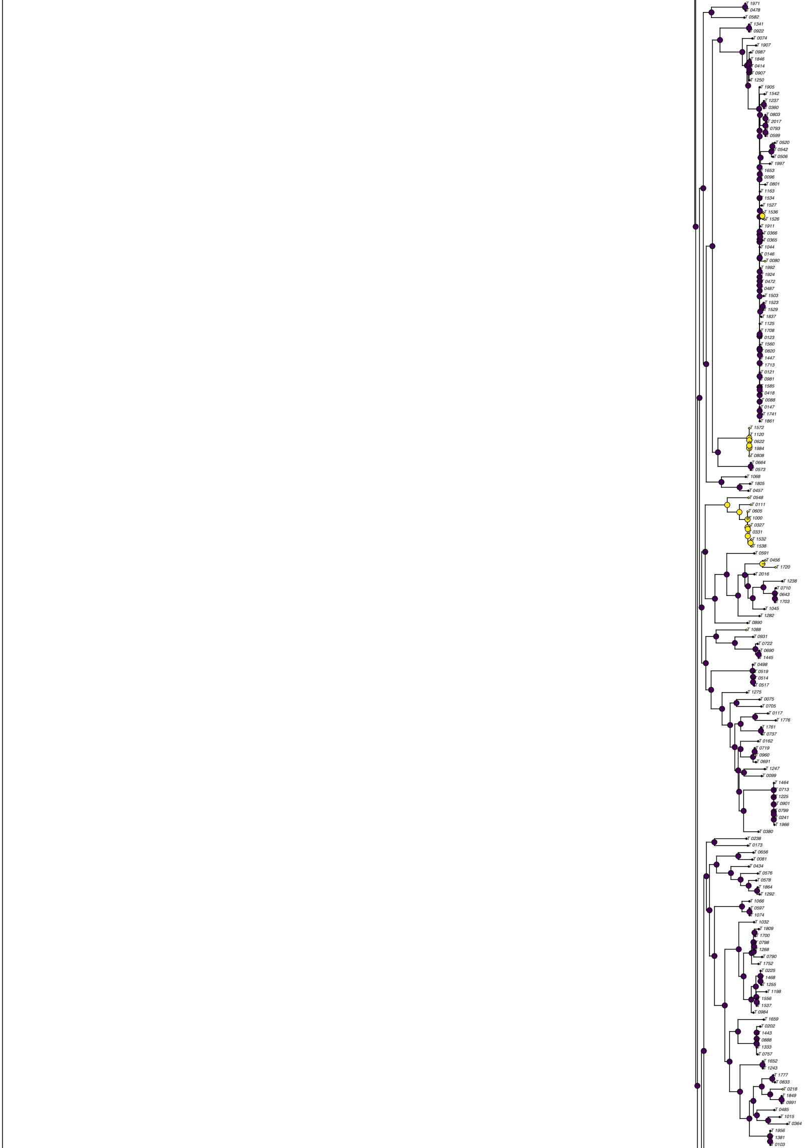
T 1971
T 0478
T 0582
T 1341
T 0922
T 0074
T 1907
T 0987
T 1846
T 0414
T 0907
T 1250
T 1905
T 1542
T 1237
T 0360
T 0803
T 2017
T 0793
T 0599
T 0520
T 0542
T 0506
T 1997
T 1653
T 0096
T 0801
T 1163
T 1534
T 1527
T 1536
T 1526
T 1911
T 0366
T 0365
T 1044
T 0146
T 0080
T 1992
T 1924
T 0472
T 0487
T 1503
T 1523
T 1529
T 1837
T 1125
T 1708
T 0123
T 1560
T 0820
T 1447
T 1713
T 0121
T 0981
T 1585
T 0418
T 0088
T 0147
T 1741
T 1861
T 1572
T 1120
T 0622
T 1984
T 0808
T 0664
T 0573
T 1068
T 1805
T 0457
T 0548
T 0111
T 0605
T 1000
T 0327
T 0331
T 1532
T 1538
T 0591
T 0456
T 1720
T 2016
T 1238
T 0710
T 0643
T 1703
T 1045
T 1282
T 0890
T 1088
T 0931
T 0722
T 0690
T 1445
T 0498
T 0519
T 0514
T 0517
T 1275
T 0075
T 0705
T 0117
T 1776
T 1761
T 0737
T 0162
T 0719
T 0960
T 0691
T 1247
T 0099
T 1464
T 0713
T 1225
T 0901
T 0799
T 0241
T 1966
T 0380
T 0238
T 0173
T 0656
T 0081
T 0434
T 0576
T 0578
T 1864
T 1292
T 1066
T 0597
T 1074
T 1032
T 1809
T 1700
T 0798
T 1268
T 0790
T 1752
T 0225
T 1468
T 1255
T 1198
T 1556
T 1537
T 0984
T 1659
T 0202
T 1443
T 0888
T 1333
T 0757
T 1652
T 1243
T 1777
T 0833
T 0218
T 1849
T 0991
T 0485
T 1015
T 0364
T 1956
T 1381
T 0103

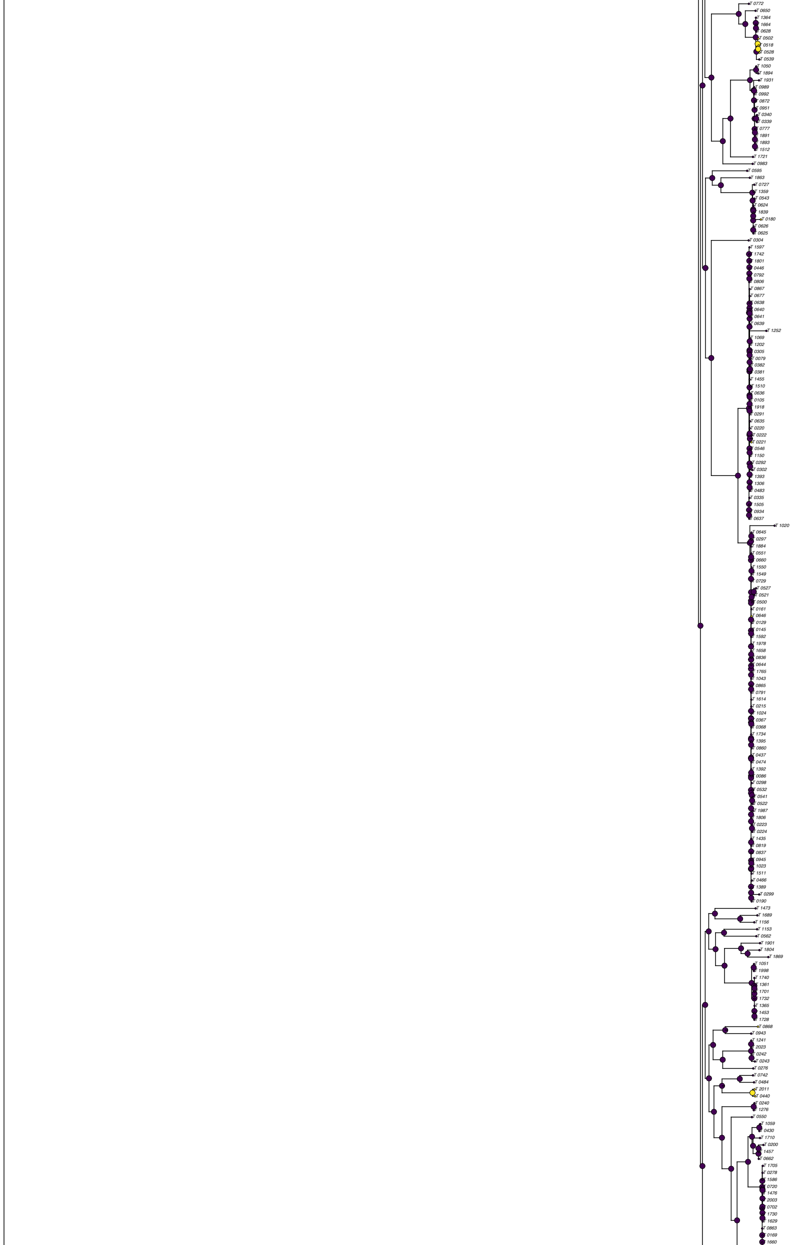

T 0772
T 0650
T 1364
T 1664
T 0628
T 0502
T 0518
T 0528
T 0539
T 1050
T 1894
T 1931
T 0989
T 0992
T 0872
T 0951
T 0340
T 0339
T 0777
T 1891
T 1893
T 1512
T 1721
T 0983
T 0595
T 1863
T 0727
T 1359
T 0543
T 0624
T 1839
T 0180
T 0626
T 0625
T 0304
T 1597
T 1742
T 1801
T 0446
T 0792
T 0806
T 0867
T 0677
T 0638
T 0640
T 0641
T 0639
T 1252
T 1069
T 1202
T 0305
T 0079
T 0382
T 0381
T 1455
T 1510
T 0636
T 0105
T 1918
T 0291
T 0635
T 0220
T 0222
T 0221
T 0546
T 1150
T 0292
T 0302
T 1393
T 1306
T 0483
T 0335
T 1505
T 0934
T 0637
T 1020
T 0645
T 0297
T 1884
T 0551
T 0660
T 1550
T 1549
T 0729
T 0527
T 0521
T 0500
T 0161
T 0646
T 0129
T 0145
T 1592
T 1978
T 1658
T 0836
T 0644
T 1765
T 1043
T 0865
T 0791
T 1614
T 0215
T 1024
T 0367
T 0368
T 1734
T 1395
T 0860
T 0437
T 0474
T 1392
T 0086
T 0298
T 0532
T 0541
T 0522
T 1987
T 1806
T 0223
T 0224
T 1435
T 0819
T 0837
T 0945
T 1023
T 1511
T 0466
T 1389
T 0299
T 0190
T 1473
T 1689
T 1156
T 1153
T 0562
T 1901
T 1804
T 1869
T 1051
T 1998
T 1740
T 1361
T 1701
T 1732
T 1365
T 1453
T 1728
T 0868
T 0943
T 1241
T 2023
T 0242
T 0243
T 0276
T 0742
T 0484
T 2011
T 0440
T 0240
T 1276
T 0550
T 1059
T 0430
T 1710
T 0200
T 1457
T 0662
T 1705
T 0278
T 1586
T 0720
T 1476
T 2003
T 0702
T 1730
T 1629
T 0863
T 0169
T 1660

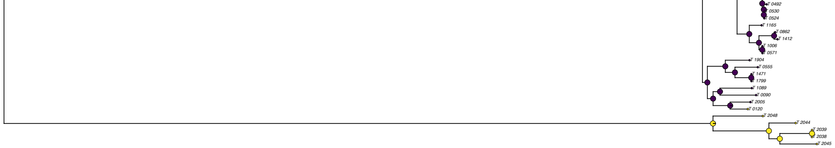
T 0492
T 0530
T 0524
T 1165
T 0862
T 1412
T 1006
T 0571
T 1904
T 0555
T 1471
T 1799
T 1089
T 0090
T 2005
T 0120
T 2048
T 2044
T 2039
T 2038
T 2045

Effector gene: tse7a

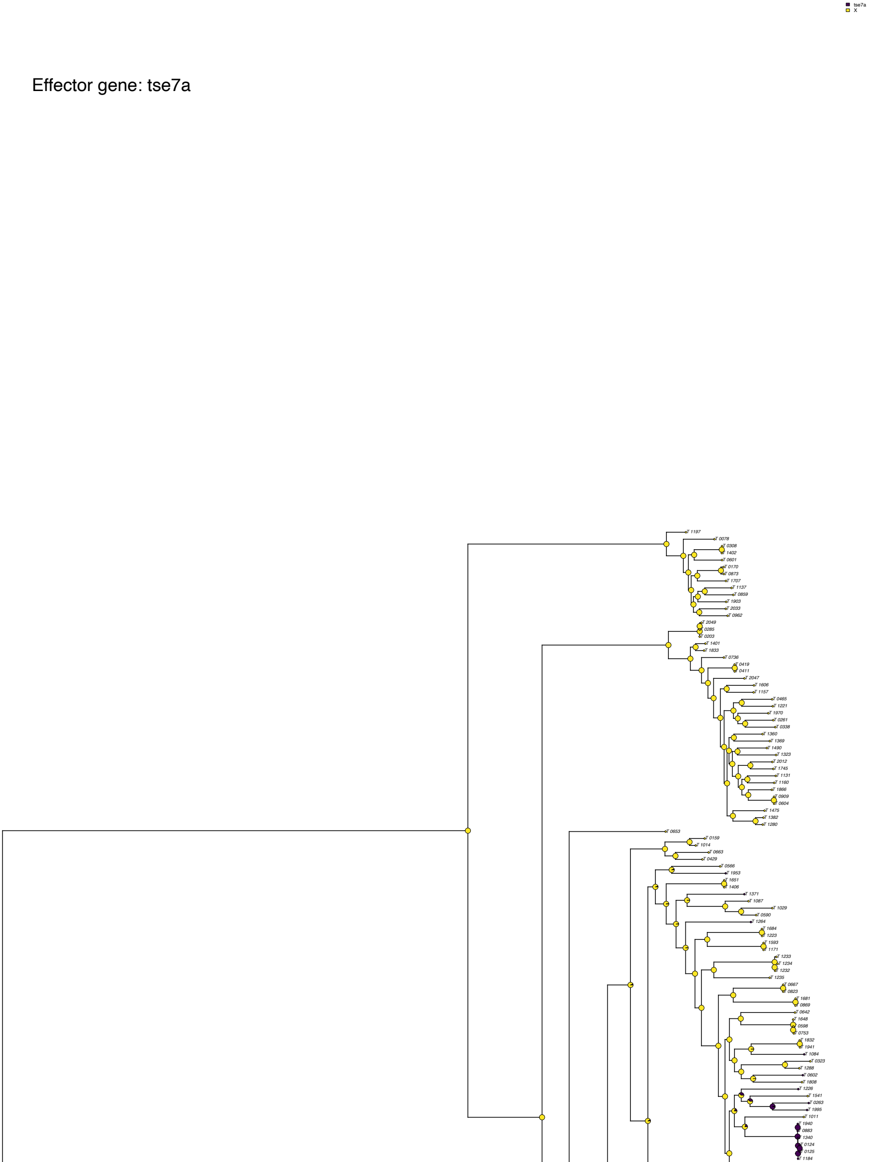

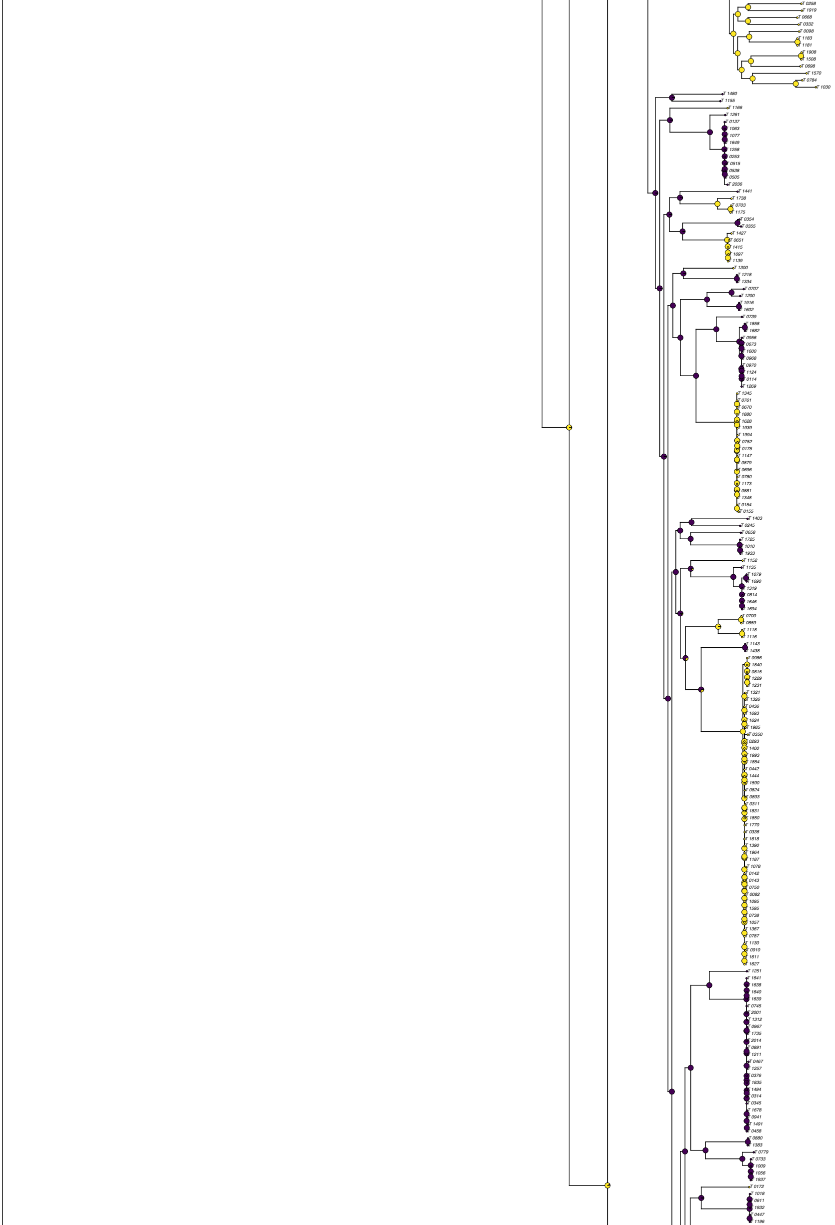
T 0258
T 1919
T 0668
T 0332
T 0098
T 1183
1181
T 1908
1508
T 0698
T 1570
T 0784
T 1030
T 1480
T 1155
T 1166
T 1261
T 0137
1063
1077
1649
1258
0253
0515
0538
0505
T 2036
T 1441
T 1738
T 0703
T 1175
T 0354
T 0355
T 1427
T 0651
1415
T 1697
T 1139
T 1300
T 1218
1334
T 0707
T 1200
T 1916
T 1602
T 0739
T 1858
T 1682
T 0956
0673
1600
0968
T 0970
1124
0114
T 1269
T 1345
T 0761
0670
1880
1628
1939
T 1994
0752
0175
T 1147
0879
T 0696
T 0780
1173
0881
1348
0154
T 0155
T 1403
T 0245
T 0658
T 1725
1010
T 1933
T 1152
T 1135
T 1079
T 1690
1319
0814
1646
1694
T 0700
T 0659
T 1118
T 1116
T 1143
T 1438
T 0986
1840
0815
1229
1231
T 1321
T 1326
T 0436
1693
1624
T 1985
T 0350
0293
1400
1993
1854
T 0442
1444
1590
T 0824
0893
T 0311
1831
1850
T 1770
T 0336
T 1618
T 1390
1964
1187
T 1078
0142
0143
0750
T 0082
1095
1595
T 0738
T 1057
T 1367
T 0787
T 1130
T 0910
1611
1627
T 1251
T 1641
1638
1640
1639
T 0745
2001
T 1312
T 0967
T 1735
2014
T 0891
1211
T 0467
T 1257
0376
1835
1494
0314
T 0345
T 1678
0941
T 1491
0458
T 0880
T 1383
T 0779
T 0733
1009
1056
T 1937
T 0172
T 1018
0611
1932
T 0447
T 1196

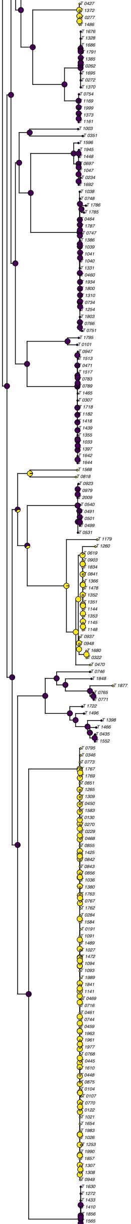
T 0427
T 1372
T 0277
T 1486
T 1676
T 1328
T 1686
T 1791
T 1385
T 0262
T 1695
T 0272
T 1370
T 0754
T 1169
T 1999
T 1373
T 1161
T 1003
T 0351
T 1596
T 1945
T 1448
T 0697
T 1047
T 0234
T 1692
T 1038
T 0748
T 1786
T 1785
T 0464
T 1787
T 0747
T 1386
T 1039
T 1041
T 1040
T 1331
T 0460
T 1934
T 1800
T 1310
T 0734
T 1254
T 1803
T 0766
T 0751
T 1795
T 0101
T 0947
T 1513
T 0471
T 1517
T 0783
T 0789
T 1465
T 0307
T 1718
T 1182
T 1418
T 1439
T 1355
T 1033
T 1397
T 1642
T 1644
T 1568
T 0818
T 0923
T 0979
T 2009
T 0540
T 0491
T 0501
T 0499
T 0531
T 1179
T 1260
T 0619
T 0903
T 1834
T 0841
T 1366
T 1478
T 1352
T 1351
T 1144
T 1353
T 1145
T 1148
T 0937
T 0948
T 1680
T 0322
T 0470
T 0746
T 1848
T 1877
T 0765
T 0771
T 1722
T 1496
T 1398
T 1466
T 0435
T 1552
T 0795
T 0346
T 0773
T 1767
T 1769
T 0851
T 1265
T 1309
T 0450
T 1583
T 0130
T 0270
T 0229
T 0468
T 0855
T 1425
T 0842
T 0843
T 0856
T 1036
T 1380
T 1763
T 0767
T 1762
T 0284
T 1584
T 0191
T 1091
T 1489
T 1027
T 1472
T 1094
T 1093
T 1989
T 1841
T 1141
T 0469
T 0716
T 0461
T 0744
T 0459
T 1963
T 1961
T 1977
T 0768
T 0445
T 1610
T 0448
T 0875
T 0104
T 0107
T 0770
T 0122
T 1021
T 1654
T 1983
T 1026
T 1253
T 1990
T 1857
T 1307
T 1308
T 0949
T 1630
T 1272
T 1433
T 1410
T 1856
T 1565

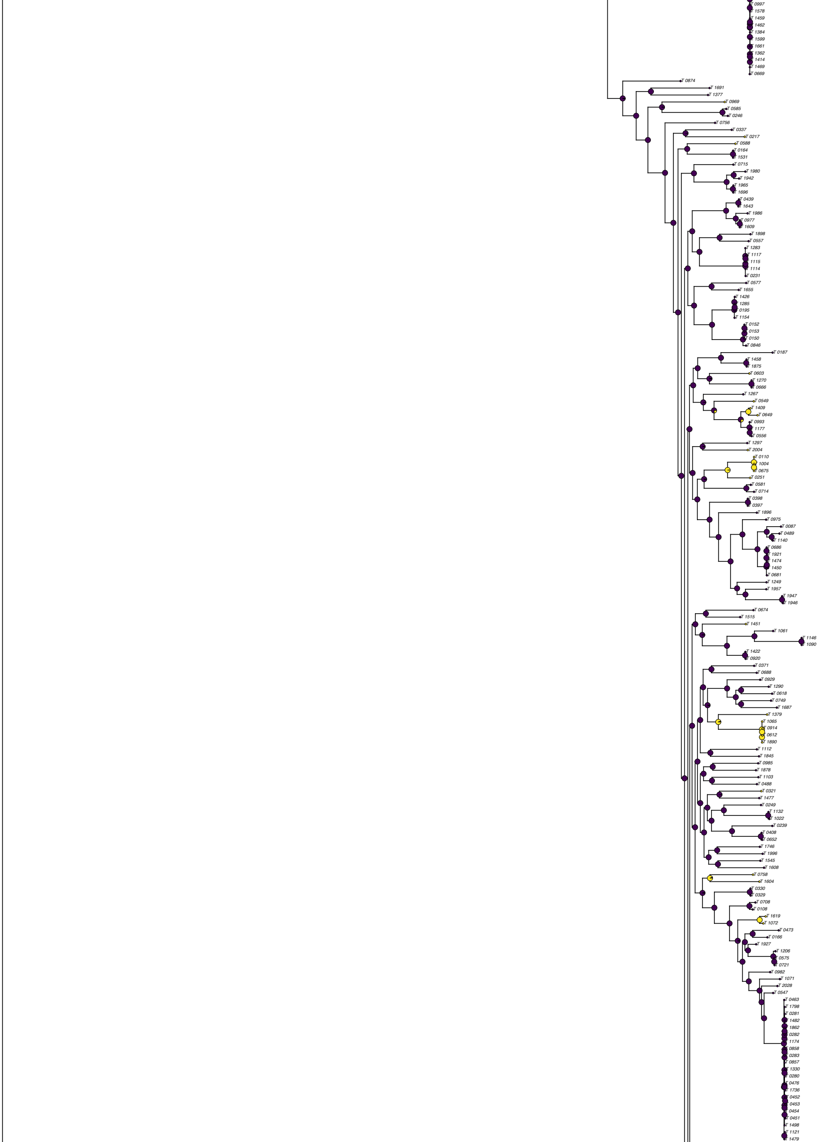
T 0997
T 1578
T 1459
T 1462
T 1384
T 1599
T 1661
T 1362
T 1414
T 1469
T 0669
T 0874
T 1691
T 1377
T 0969
T 0585
T 0246
T 0756
T 0337
T 0217
T 0588
T 0164
T 1531
T 0715
T 1980
T 1942
T 1965
T 1696
T 0439
T 1643
T 1986
T 0977
T 1609
T 1898
T 0557
T 1283
T 1117
T 1115
T 1114
T 0231
T 0577
T 1655
T 1426
T 1285
T 0195
T 1154
T 0152
T 0153
T 0150
T 0846
T 0187
T 1458
T 1875
T 0603
T 1270
T 0666
T 1267
T 0549
T 1409
T 0649
T 0993
T 1177
T 0556
T 1297
T 2004
T 0110
T 1004
T 0675
T 0251
T 0581
T 0714
T 0398
T 0397
T 1896
T 0975
T 0087
T 0489
T 1140
T 0686
T 1921
T 1474
T 1450
T 0681
T 1249
T 1957
T 1947
T 1946
T 0674
T 1515
T 1451
T 1061
T 1146
T 1090
T 1422
T 0920
T 0371
T 0688
T 0929
T 1290
T 0618
T 0749
T 1687
T 1379
T 1065
T 0914
T 0612
T 1890
T 1112
T 1845
T 0985
T 1878
T 1103
T 0488
T 0321
T 1477
T 0249
T 1132
T 1022
T 0239
T 0408
T 0652
T 1746
T 1996
T 1545
T 1608
T 0758
T 1604
T 0330
T 0329
T 0708
T 0108
T 1619
T 1072
T 0473
T 0166
T 1927
T 1206
T 0575
T 0721
T 0982
T 1071
T 2028
T 0547
T 0463
T 1798
T 0281
T 1482
T 1862
T 0282
T 1174
T 0858
T 0283
T 0857
T 1330
T 0280
T 0476
T 1736
T 0452
T 0453
T 0454
T 0451
T 1498
T 1121
T 1479

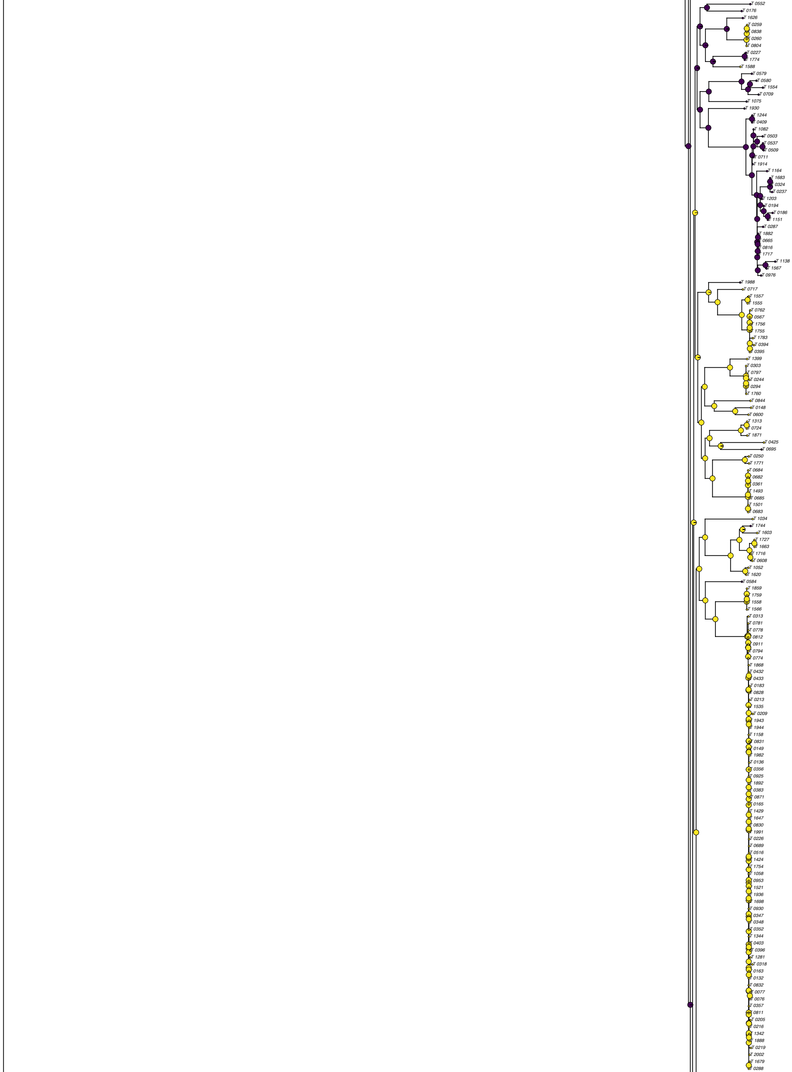
T 0552
T 0176
T 1626
T 0259
0838
0260
T 0804
T 0227
T 1774
T 1588
T 0579
T 0580
T 1554
T 0709
T 1075
T 1930
T 1244
T 0409
T 1082
T 0503
T 0537
T 0509
T 0711
T 1914
T 1164
T 1683
0324
T 0237
T 1203
T 0194
T 0186
T 1151
T 0287
T 1882
0665
T 0816
1717
T 1138
T 1567
T 0976
T 1988
T 0717
T 1557
T 1555
T 0762
0567
1756
1755
T 1783
T 0394
0395
T 1399
T 0303
T 0797
T 0244
0294
T 1760
T 0844
T 0148
T 0600
T 1313
T 0724
T 1871
T 0425
T 0695
T 0250
T 1771
T 0684
0682
0361
T 1493
T 0685
T 1501
0683
T 1034
T 1744
T 1603
T 1727
T 1663
T 1716
T 0608
T 1052
T 1620
T 0584
T 1859
1759
1558
T 1566
T 0313
T 0781
T 0778
0812
0911
T 0794
0774
T 1868
T 0432
0433
T 0183
T 0828
T 0213
1535
T 0209
1943
1944
T 1158
0831
0149
T 1982
T 0136
0356
T 0925
1892
0383
T 0871
0165
T 1429
1647
0830
1991
T 0226
T 0689
T 0516
1424
T 1754
T 1058
0953
1521
T 1936
1698
T 0930
0347
0348
T 0352
T 1344
T 0403
T 0396
T 1281
T 0318
0163
T 0132
T 0832
T 0077
T 0076
T 0357
0811
T 0205
T 0216
1342
1888
T 0219
T 2002
T 1679
0288

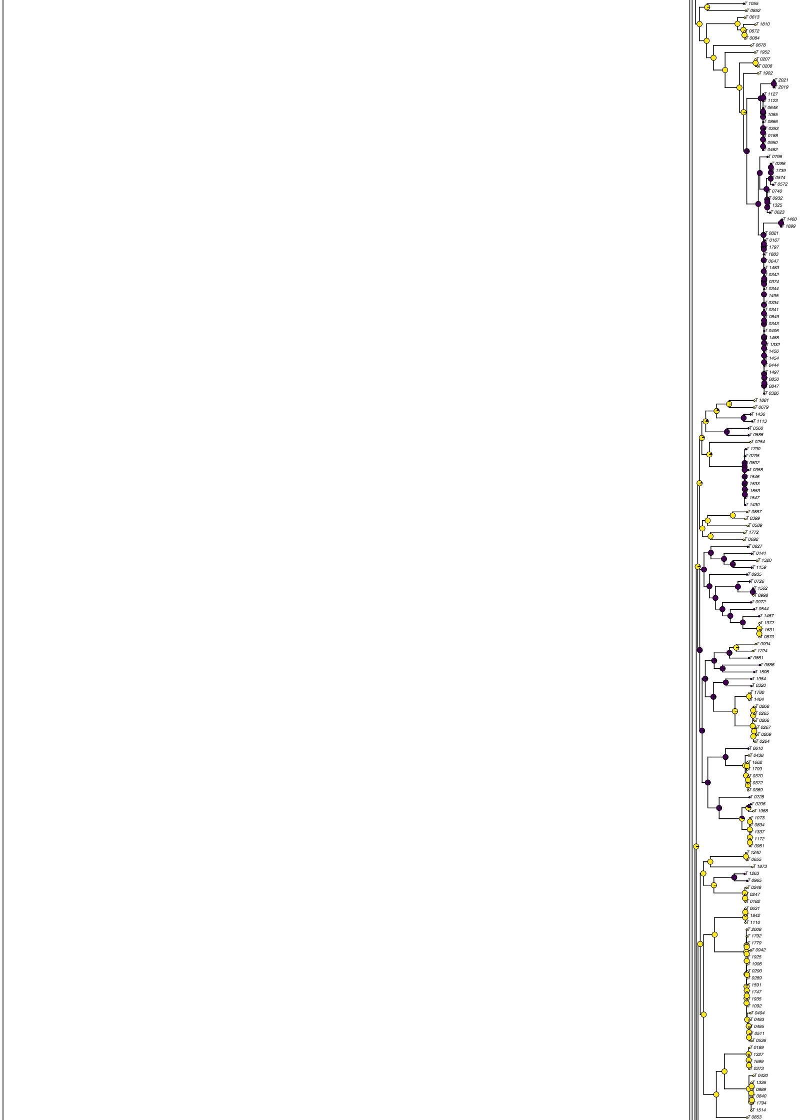
T 1055
T 0852
T 0613
T 1810
T 0672
T 0084
T 0678
T 1952
T 0207
T 0208
T 1902
T 2021
T 2019
T 1127
T 1123
T 0648
T 1085
T 0866
T 0353
T 0188
T 0950
T 0462
T 0796
T 0286
T 1739
T 0574
T 0572
T 0740
T 0932
T 1325
T 0623
T 1460
T 1899
T 0821
T 0167
T 1797
T 1883
T 0647
T 1483
T 0342
T 0374
T 0344
T 1495
T 0334
T 0341
T 0849
T 0343
T 0406
T 1488
T 1332
T 1456
T 1454
T 0444
T 1497
T 0850
T 0847
T 0326
T 1881
T 0679
T 1436
T 1113
T 0560
T 0586
T 0254
T 1790
T 0235
T 0802
T 0358
T 1546
T 1533
T 1553
T 1547
T 1430
T 0887
T 0399
T 0589
T 1772
T 0692
T 0827
T 0141
T 1320
T 1159
T 0935
T 0726
T 1562
T 0998
T 0972
T 0544
T 1467
T 1972
T 1631
T 0870
T 0094
T 1224
T 0861
T 0886
T 1506
T 1954
T 0320
T 1780
T 1404
T 0268
T 0265
T 0266
T 0267
T 0269
T 0264
T 0610
T 0438
T 1662
T 1709
T 0370
T 0372
T 0369
T 0228
T 0206
T 1968
T 1073
T 0834
T 1337
T 1172
T 0961
T 1240
T 0655
T 1873
T 1263
T 0965
T 0248
T 0247
T 0182
T 0631
T 1842
T 1110
T 2008
T 1792
T 1779
T 0942
T 1925
T 1906
T 0290
T 0289
T 1591
T 1747
T 1935
T 1092
T 0494
T 0493
T 0495
T 0511
T 0536
T 0189
T 1327
T 1699
T 0373
T 0420
T 1336
T 0889
T 0840
T 1794
T 1514
T 0853

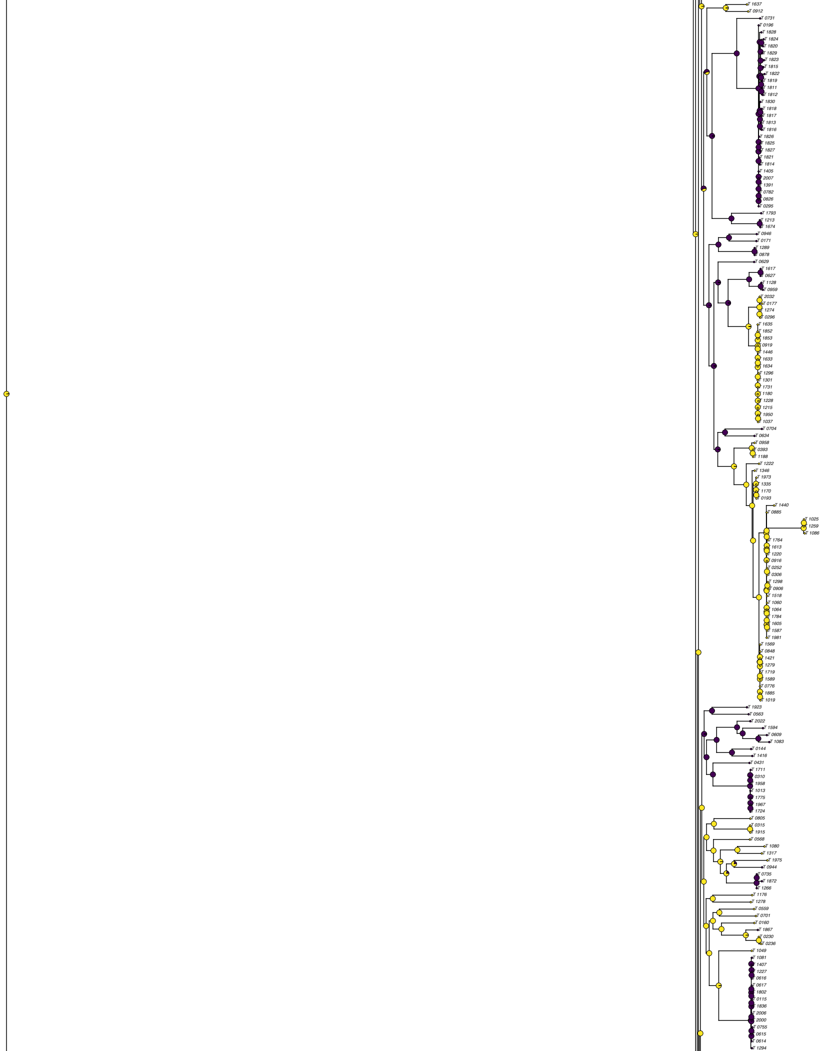
T 1637
T 0912
T 0731
T 0196
T 1828
T 1824
T 1820
T 1829
T 1823
T 1815
T 1822
T 1819
T 1811
T 1812
T 1830
T 1818
T 1817
T 1813
T 1816
T 1826
T 1825
T 1827
T 1821
T 1814
T 1405
2007
1391
0782
0826
T 0295
T 1793
T 1213
T 1674
T 0946
T 0171
T 1289
T 0878
T 0629
T 1617
T 0627
T 1128
T 0959
T 2032
T 0177
T 1274
T 0296
T 1635
T 1852
1853
0919
T 1446
1633
1634
T 1296
T 1301
T 1731
1180
1228
1215
1950
T 1037
T 0704
T 0634
T 0958
0393
T 1188
T 1222
T 1346
T 1973
1335
1170
0193
T 1440
T 0885
T 1025
1259
T 1086
T 1764
1613
T 1220
0916
T 0252
0306
T 1298
T 0906
T 1518
T 1060
1064
1784
1605
T 1587
T 1981
T 1569
T 0848
1421
1279
T 1719
1589
T 0776
1885
T 1019
T 1923
T 0563
T 2022
T 1594
T 0609
T 1083
T 0144
T 1416
T 0431
T 1711
0310
1958
T 1013
1775
1967
1724
T 0805
T 0315
T 1915
T 0568
T 1080
T 1317
T 1975
T 0944
T 0735
T 1872
T 1266
T 1176
T 1278
T 0559
T 0701
T 0160
T 1867
T 0230
T 0236
T 1049
T 1081
1407
1227
0616
T 0617
1802
0115
1836
T 2006
2000
T 0755
0615
T 0614
T 1294

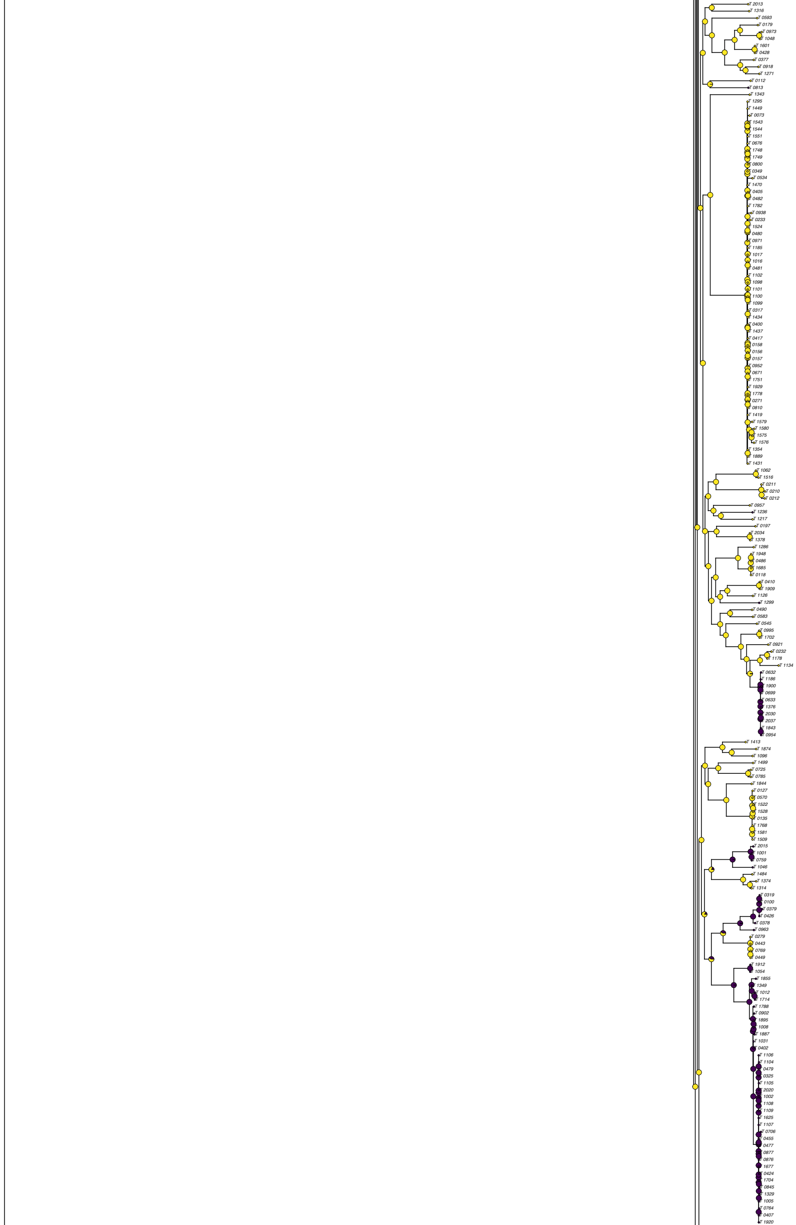
T 2013
T 1316
T 0593
T 0179
T 0973
T 1048
T 1601
T 0428
T 0377
T 0918
T 1271
T 0112
T 0813
T 1343
T 1295
T 1449
T 0073
T 1543
T 1544
T 1551
T 0676
T 1748
T 1749
T 0800
T 0349
T 0534
T 1470
T 0405
T 0482
T 1782
T 0938
T 0233
T 1524
T 0480
T 0971
T 1185
T 1017
T 1016
T 0481
T 1102
T 1098
T 1101
T 1100
T 1099
T 0317
T 1434
T 0400
T 1437
T 0417
T 0158
T 0156
T 0157
T 0952
T 0671
T 1751
T 1929
T 1778
T 0271
T 0810
T 1419
T 1579
T 1580
T 1575
T 1576
T 1354
T 1889
T 1431
T 1062
T 1516
T 0211
T 0210
T 0212
T 0957
T 1236
T 1217
T 0197
T 2034
T 1378
T 1286
T 1948
T 0486
T 1685
T 0118
T 0410
T 1909
T 1126
T 1299
T 0490
T 0583
T 0545
T 0995
T 1702
T 0921
T 0232
T 1178
T 1134
T 0632
T 1186
T 1900
T 0699
T 0633
T 1376
T 2030
T 2037
T 1843
T 0954
T 1413
T 1874
T 1096
T 1499
T 0725
T 0785
T 1844
T 0127
T 0570
T 1522
T 1528
T 0135
T 1768
T 1581
T 1509
T 2015
T 1001
T 0759
T 1046
T 1484
T 1374
T 1314
T 0319
T 0100
T 0379
T 0426
T 0378
T 0963
T 0279
T 0443
T 0769
T 0449
T 1912
T 1054
T 1855
T 1349
T 1012
T 1714
T 1788
T 0902
T 1895
T 1008
T 1887
T 1031
T 0402
T 1106
T 1104
T 0479
T 0325
T 1105
T 2020
T 1002
T 1108
T 1109
T 1625
T 1107
T 0706
T 0455
T 0477
T 0877
T 0876
T 1677
T 0424
T 1704
T 0845
T 1329
T 1005
T 0764
T 0407
T 1920

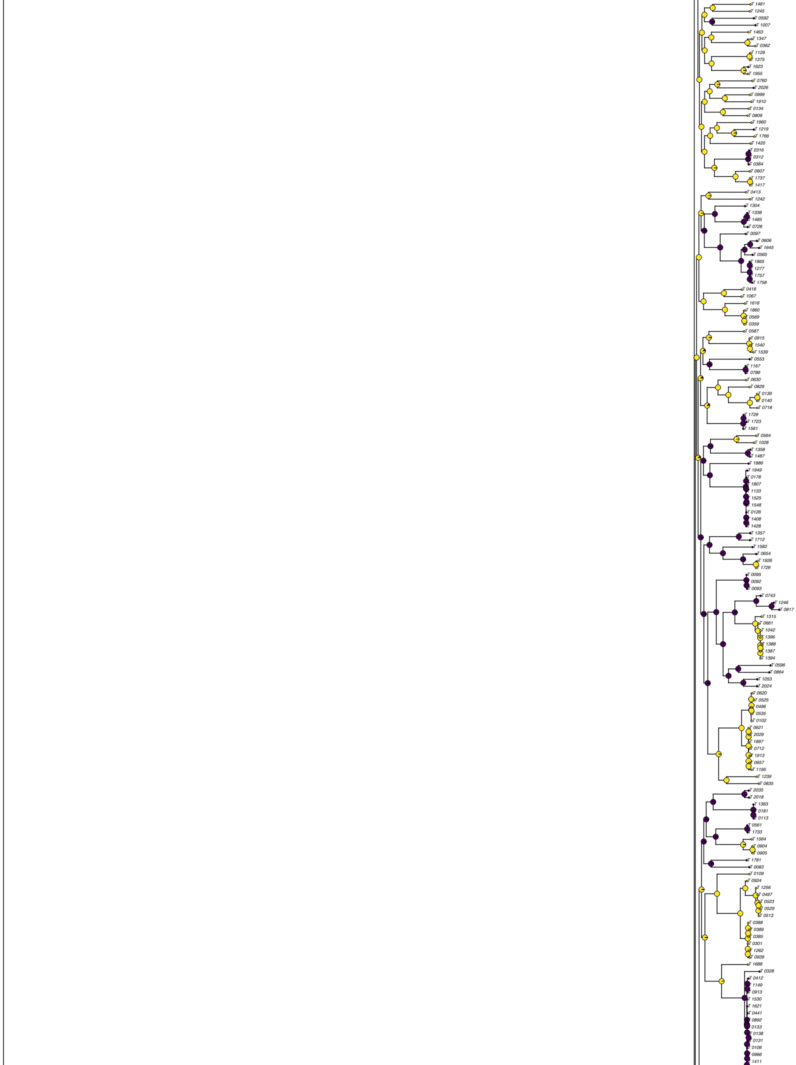
T 1461
T 1245
T 0592
T 1007
T 1463
T 1347
T 0362
T 1129
T 1375
T 1623
T 1955
T 0760
T 2026
T 0999
T 1910
T 0134
T 0809
T 1960
T 1219
T 1766
T 1420
T 0316
0312
T 0384
T 0607
T 1737
T 1417
T 0413
T 1242
T 1304
T 1338
T 1485
T 0728
T 0097
T 0606
T 1645
T 0565
T 1865
1277
1757
T 1758
T 0416
T 1067
T 1616
T 1860
0569
0359
T 0587
T 0915
1540
T 1539
T 0553
T 1167
0786
T 0630
T 0829
T 0139
0140
T 0718
T 1729
T 1723
T 1561
T 0564
T 1028
T 1358
T 1487
T 1886
T 1949
T 0178
1807
1133
1525
1548
T 0126
1408
1428
T 1357
T 1712
T 1582
T 0654
T 1926
1726
T 0095
0092
0093
T 0743
T 1246
T 0817
T 1315
0661
T 1042
1396
T 1388
1387
T 1394
T 0596
T 0864
T 1053
T 2024
T 0620
T 0525
0496
0535
T 0102
T 0621
2029
T 1897
T 0712
T 1913
T 0657
T 1195
T 1239
T 0835
T 2035
T 2018
T 1363
0181
0113
T 0561
T 1733
T 1564
T 0904
0905
T 1781
T 0083
T 0109
T 0924
T 1256
T 0497
T 0523
T 0529
T 0513
T 0388
T 0389
T 0385
T 0301
T 1262
T 0926
T 1688
T 0328
T 0412
T 1149
T 0913
T 1530
T 1621
T 0441
0892
0133
T 0138
T 0131
0106
0966
1411

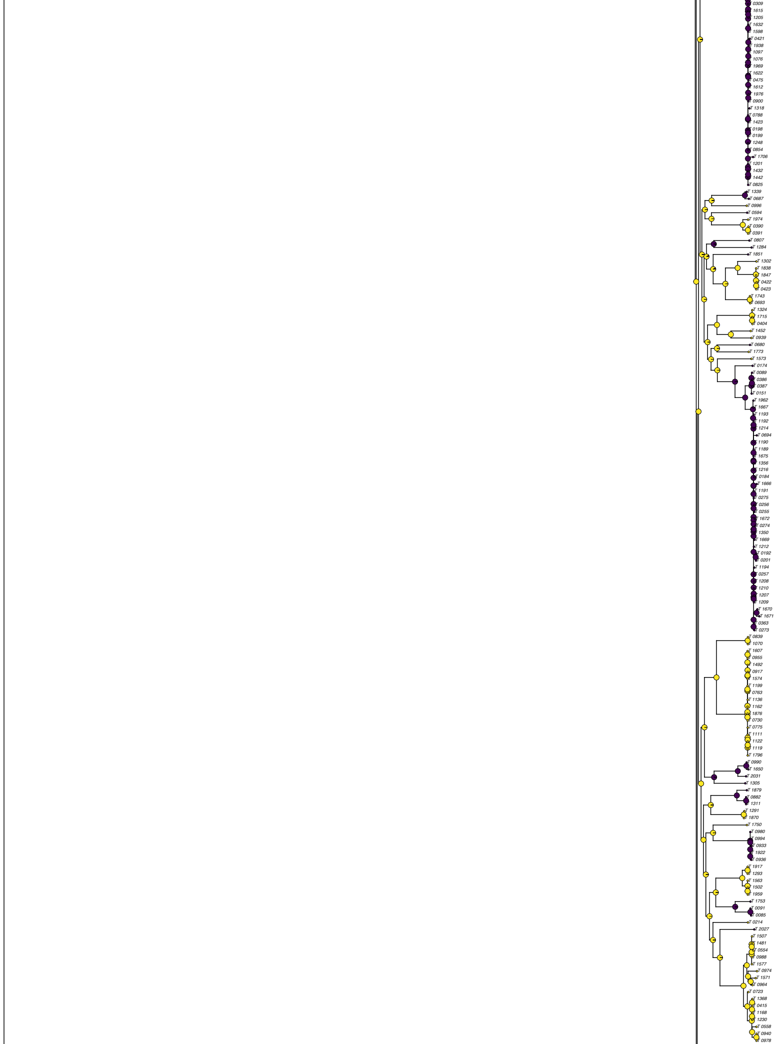

T 0309
T 1615
T 1205
T 1632
T 1598
T 0421
T 1938
T 1097
T 1076
T 1969
T 1622
T 0475
T 1612
T 1976
T 0900
T 1318
T 0788
T 1423
T 0198
T 0199
T 1248
T 0854
T 1706
T 1201
T 1432
T 1442
T 0825
T 1339
T 0687
T 0996
T 0594
T 1974
T 0390
T 0391
T 0807
T 1284
T 1851
T 1302
T 1838
T 1847
T 0422
T 0423
T 1743
T 0693
T 1324
T 1715
T 0404
T 1452
T 0939
T 0680
T 1773
T 1573
T 0174
T 0089
T 0386
T 0387
T 0151
T 1962
T 1667
T 1193
T 1192
T 1214
T 0694
T 1190
T 1189
T 1675
T 1356
T 1216
T 0184
T 1666
T 1191
T 0275
T 0256
T 0255
T 1672
T 0274
T 1350
T 1669
T 1212
T 0192
T 0201
T 1194
T 0257
T 1208
T 1210
T 1207
T 1209
T 1670
T 1671
T 0363
T 0273
T 0839
T 1070
T 1607
T 0955
T 1492
T 0917
T 1574
T 1199
T 0763
T 1136
T 1162
T 1876
T 0730
T 0775
T 1111
T 1122
T 1119
T 1796
T 0990
T 1650
T 2031
T 1305
T 1879
T 0882
T 1311
T 1291
T 1870
T 1750
T 0980
T 0994
T 0933
T 1922
T 0936
T 1917
T 1293
T 1563
T 1502
T 1959
T 1753
T 0091
T 0085
T 0214
T 2027
T 1507
T 1481
T 0554
T 0988
T 1577
T 0974
T 1571
T 0964
T 0723
T 1368
T 0415
T 1168
T 1230
T 0558
T 0940
T 0978

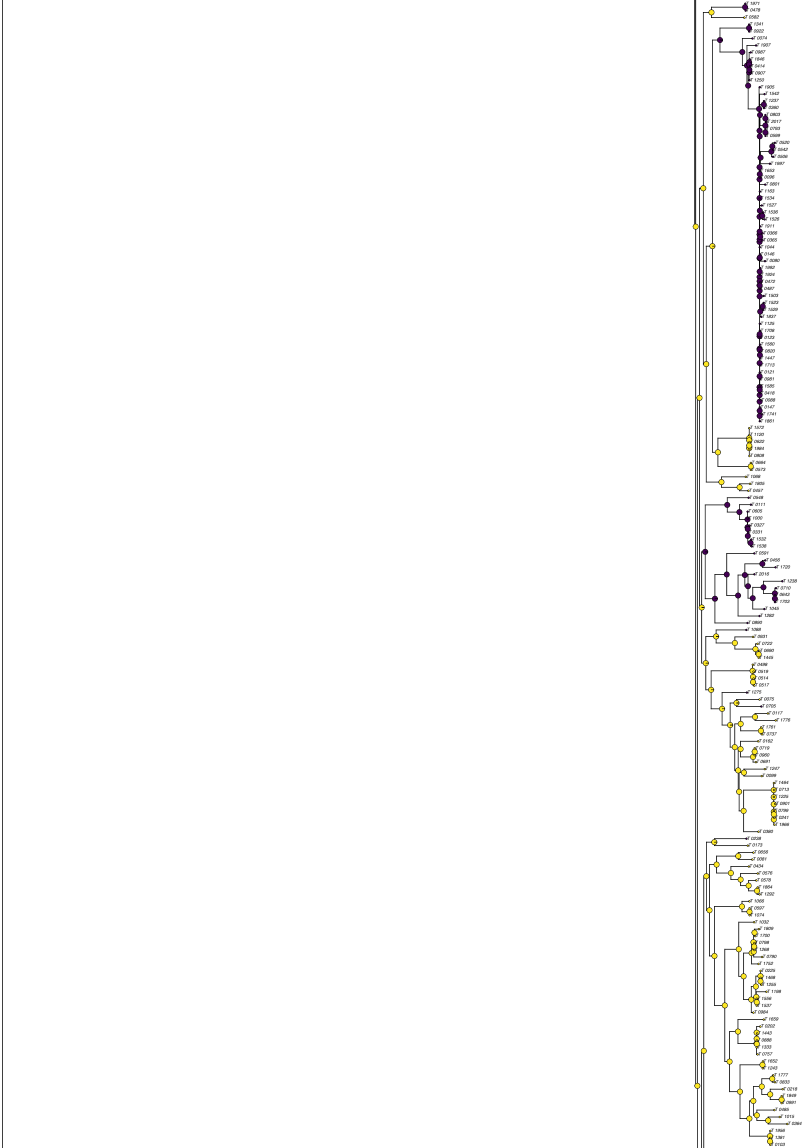
T 1971
T 0478
T 0582
T 1341
T 0922
T 0074
T 1907
T 0987
T 1846
T 0414
T 0907
T 1250
T 1905
T 1542
T 1237
T 0360
T 0803
T 2017
T 0793
T 0599
T 0520
T 0542
T 0506
T 1997
T 1653
0096
T 0801
T 1163
1534
T 1527
T 1536
T 1526
T 1911
T 0366
T 0365
T 1044
T 0146
T 0080
T 1992
T 1924
T 0472
0487
T 1503
T 1523
T 1529
T 1837
T 1125
T 1708
T 0123
T 1560
0820
T 1447
T 1713
T 0121
T 0981
T 1585
T 0418
T 0088
T 0147
T 1741
T 1861
T 1572
T 1120
T 0622
T 1984
T 0808
T 0664
T 0573
T 1068
T 1805
T 0457
T 0548
T 0111
T 0605
1000
T 0327
0331
T 1532
T 1538
T 0591
T 0456
T 1720
T 2016
T 1238
T 0710
0643
T 1703
T 1045
T 1282
T 0890
T 1088
T 0931
T 0722
T 0690
T 1445
T 0498
T 0519
T 0514
T 0517
T 1275
T 0075
T 0705
T 0117
T 1776
T 1761
T 0737
T 0162
T 0719
0960
T 0691
T 1247
T 0099
T 1464
0713
1225
T 0901
T 0799
0241
T 1966
T 0380
T 0238
T 0173
T 0656
T 0081
T 0434
T 0576
T 0578
T 1864
T 1292
T 1066
T 0597
1074
T 1032
T 1809
T 1700
T 0798
1268
T 0790
T 1752
T 0225
T 1468
T 1255
T 1198
T 1556
T 1537
T 0984
T 1659
T 0202
1443
0888
T 1333
T 0757
T 1652
T 1243
T 1777
T 0833
T 0218
T 1849
T 0991
T 0485
T 1015
T 0364
T 1956
T 1381
T 0103

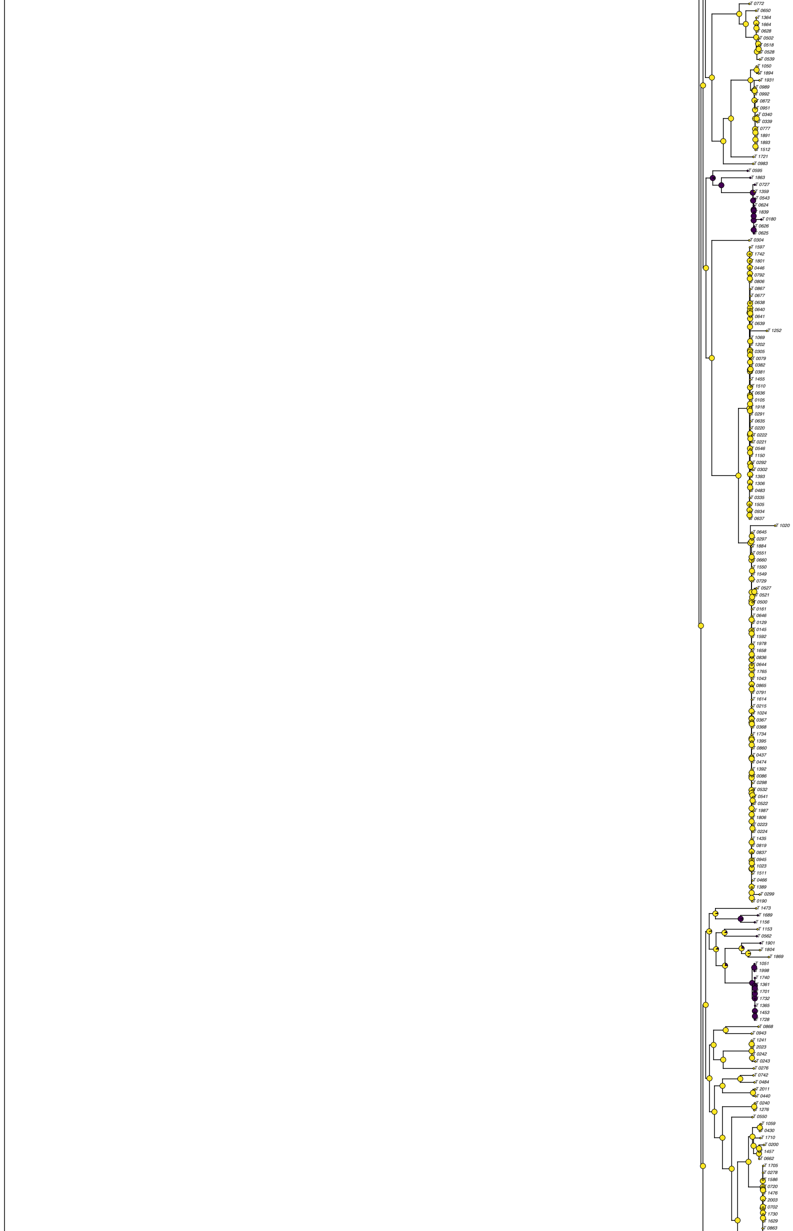

T 0772
T 0650
T 1364
T 1664
T 0628
T 0502
T 0518
T 0528
T 0539
T 1050
T 1894
T 1931
T 0989
T 0992
T 0872
T 0951
T 0340
T 0339
T 0777
T 1891
T 1893
T 1512
T 1721
T 0983
T 0595
T 1863
T 0727
T 1359
T 0543
T 0624
T 1839
T 0180
T 0626
T 0625
T 0304
T 1597
T 1742
T 1801
T 0446
T 0792
T 0806
T 0867
T 0677
T 0638
T 0640
T 0641
T 0639
T 1252
T 1069
T 1202
T 0305
T 0079
T 0382
T 0381
T 1455
T 1510
T 0636
T 0105
T 1918
T 0291
T 0635
T 0220
T 0222
T 0221
T 0546
T 1150
T 0292
T 0302
T 1393
T 1306
T 0483
T 0335
T 1505
T 0934
T 0637
T 1020
T 0645
T 0297
T 1884
T 0551
T 0660
T 1550
T 1549
T 0729
T 0527
T 0521
T 0500
T 0161
T 0646
T 0129
T 0145
T 1592
T 1978
T 1658
T 0836
T 0644
T 1765
T 1043
T 0865
T 0791
T 1614
T 0215
T 1024
T 0367
T 0368
T 1734
T 1395
T 0860
T 0437
T 0474
T 1392
T 0086
T 0298
T 0532
T 0541
T 0522
T 1987
T 1806
T 0223
T 0224
T 1435
T 0819
T 0837
T 0945
T 1023
T 1511
T 0466
T 1389
T 0299
T 0190
T 1473
T 1689
T 1156
T 1153
T 0562
T 1901
T 1804
T 1869
T 1051
T 1998
T 1740
T 1361
T 1701
T 1732
T 1365
T 1453
T 1728
T 0868
T 0943
T 1241
T 2023
T 0242
T 0243
T 0276
T 0742
T 0484
T 2011
T 0440
T 0240
T 1276
T 0550
T 1059
T 0430
T 1710
T 0200
T 1457
T 0662
T 1705
T 0278
T 1586
T 0720
T 1476
T 2003
T 0702
T 1730
T 1629
T 0863

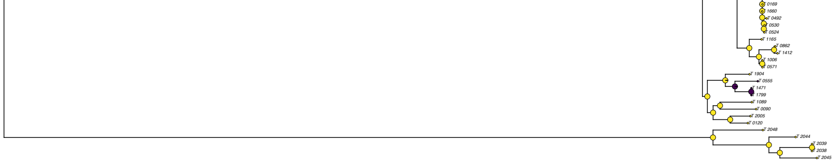

0169
1660
T 0492
T 0530
T 0524
T 1165
T 0862
T 1412
T 1006
T 0571
T 1904
T 0555
T 1471
T 1799
T 1089
T 0090
T 2005
T 0120
T 2048
T 2044
T 2039
T 2038
T 2045

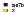

Effector gene: tsd1 (tse7b)

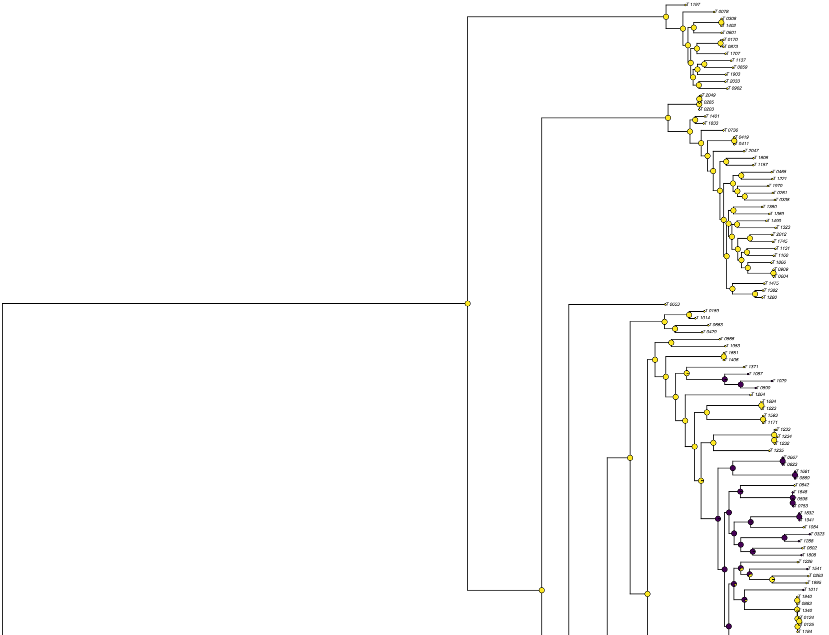

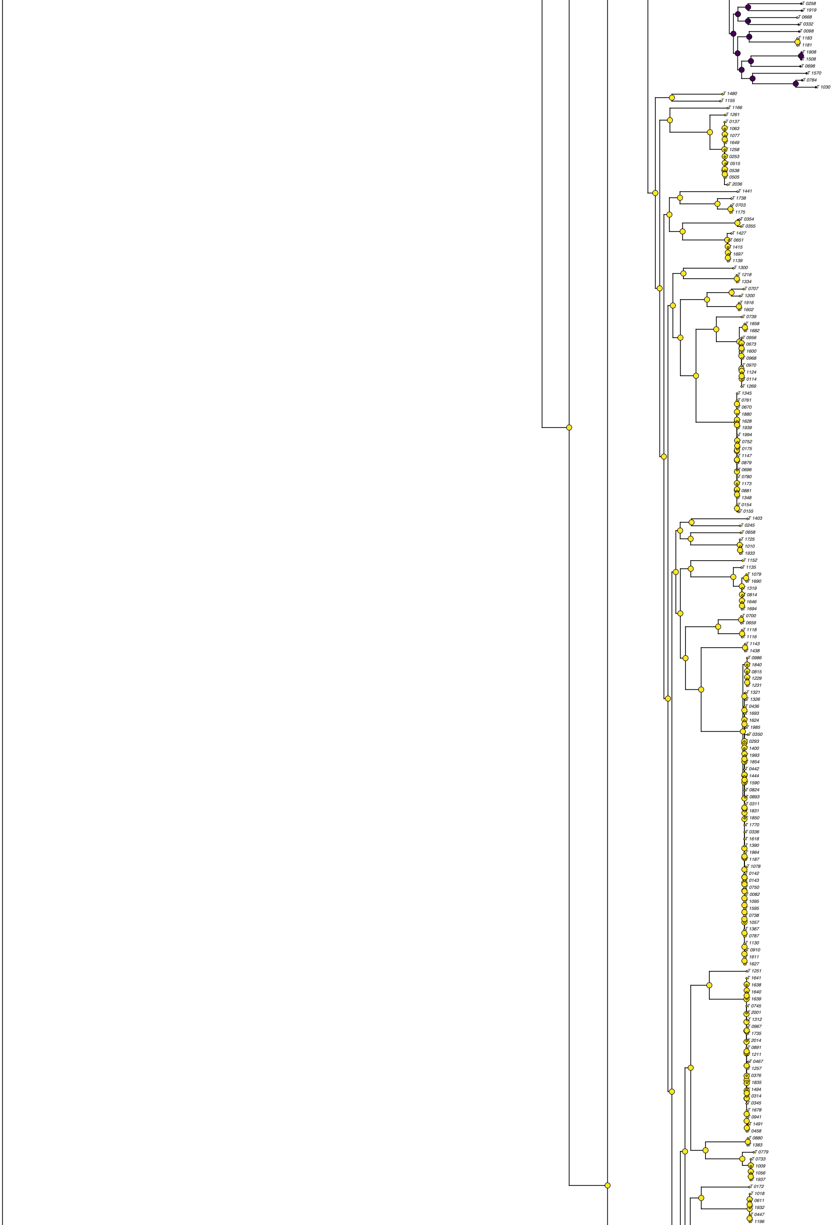
T 0258
T 1919
T 0668
T 0332
T 0098
T 1183
1181
T 1908
T 1508
T 0698
T 1570
T 0784
T 1030
T 1480
T 1155
T 1166
T 1261
T 0137
1063
1077
1649
1258
0253
0515
0538
0505
T 2036
T 1441
T 1738
T 0703
T 1175
T 0354
T 0355
T 1427
T 0651
1415
T 1697
1139
T 1300
T 1218
1334
T 0707
T 1200
T 1916
T 1602
T 0739
T 1858
T 1682
T 0956
0673
1600
0968
T 0970
1124
0114
T 1269
T 1345
T 0761
0670
1880
T 1628
1939
T 1994
0752
0175
T 1147
0879
T 0696
T 0780
1173
0881
1348
0154
T 0155
T 1403
T 0245
T 0658
T 1725
1010
1933
T 1152
T 1135
T 1079
1690
1319
T 0814
1646
1694
T 0700
0659
T 1118
1116
T 1143
1438
T 0986
1840
0815
1229
1231
T 1321
T 1326
T 0436
1693
1624
T 1985
T 0350
0293
1400
1993
1854
T 0442
1444
1590
T 0824
0893
T 0311
1831
1850
T 1770
T 0336
T 1618
T 1390
1964
1187
T 1078
0142
0143
0750
0082
1095
1595
0738
1057
T 1367
0787
T 1130
T 0910
1611
1627
T 1251
T 1641
1638
1640
1639
T 0745
2001
T 1312
T 0967
1735
2014
T 0891
1211
T 0467
1257
0376
1835
1494
0314
0345
T 1678
0941
T 1491
0458
T 0880
1383
T 0779
T 0733
1009
1056
1937
T 0172
T 1018
0611
1932
T 0447
1196

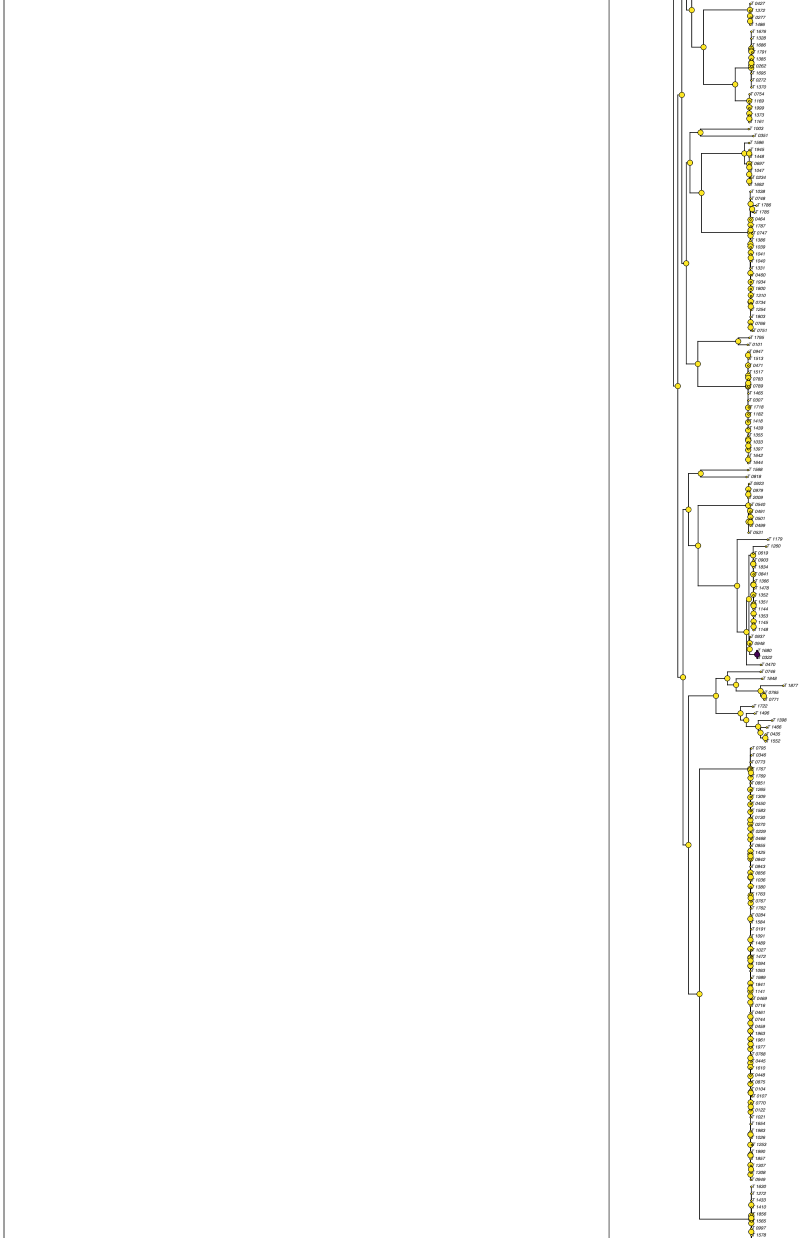
T 0427
T 1372
T 0277
T 1486
T 1676
T 1328
T 1686
T 1791
T 1385
T 0262
T 1695
T 0272
T 1370
T 0754
T 1169
T 1999
T 1373
T 1161
T 1003
T 0351
T 1596
T 1945
T 1448
T 0697
T 1047
T 0234
T 1692
T 1038
T 0748
T 1786
T 1785
T 0464
T 1787
T 0747
T 1386
T 1039
T 1041
T 1040
T 1331
T 0460
T 1934
T 1800
T 1310
T 0734
T 1254
T 1803
T 0766
T 0751
T 1795
T 0101
T 0947
T 1513
T 0471
T 1517
T 0783
T 0789
T 1465
T 0307
T 1718
T 1182
T 1418
T 1439
T 1355
T 1033
T 1397
T 1642
T 1644
T 1568
T 0818
T 0923
T 0979
T 2009
T 0540
T 0491
T 0501
T 0499
T 0531
T 1179
T 1260
T 0619
T 0903
T 1834
T 0841
T 1366
T 1478
T 1352
T 1351
T 1144
T 1353
T 1145
T 1148
T 0937
T 0948
T 1680
T 0322
T 0470
T 0746
T 1848
T 1877
T 0765
T 0771
T 1722
T 1496
T 1398
T 1466
T 0435
T 1552
T 0795
T 0346
T 0773
T 1767
T 1769
T 0851
T 1265
T 1309
T 0450
T 1583
T 0130
T 0270
T 0229
T 0468
T 0855
T 1425
T 0842
T 0843
T 0856
T 1036
T 1380
T 1763
T 0767
T 1762
T 0284
T 1584
T 0191
T 1091
T 1489
T 1027
T 1472
T 1094
T 1093
T 1989
T 1841
T 1141
T 0469
T 0716
T 0461
T 0744
T 0459
T 1963
T 1961
T 1977
T 0768
T 0445
T 1610
T 0448
T 0875
T 0104
T 0107
T 0770
T 0122
T 1021
T 1654
T 1983
T 1026
T 1253
T 1990
T 1857
T 1307
T 1308
T 0949
T 1630
T 1272
T 1433
T 1410
T 1856
T 1565
T 0997
T 1578

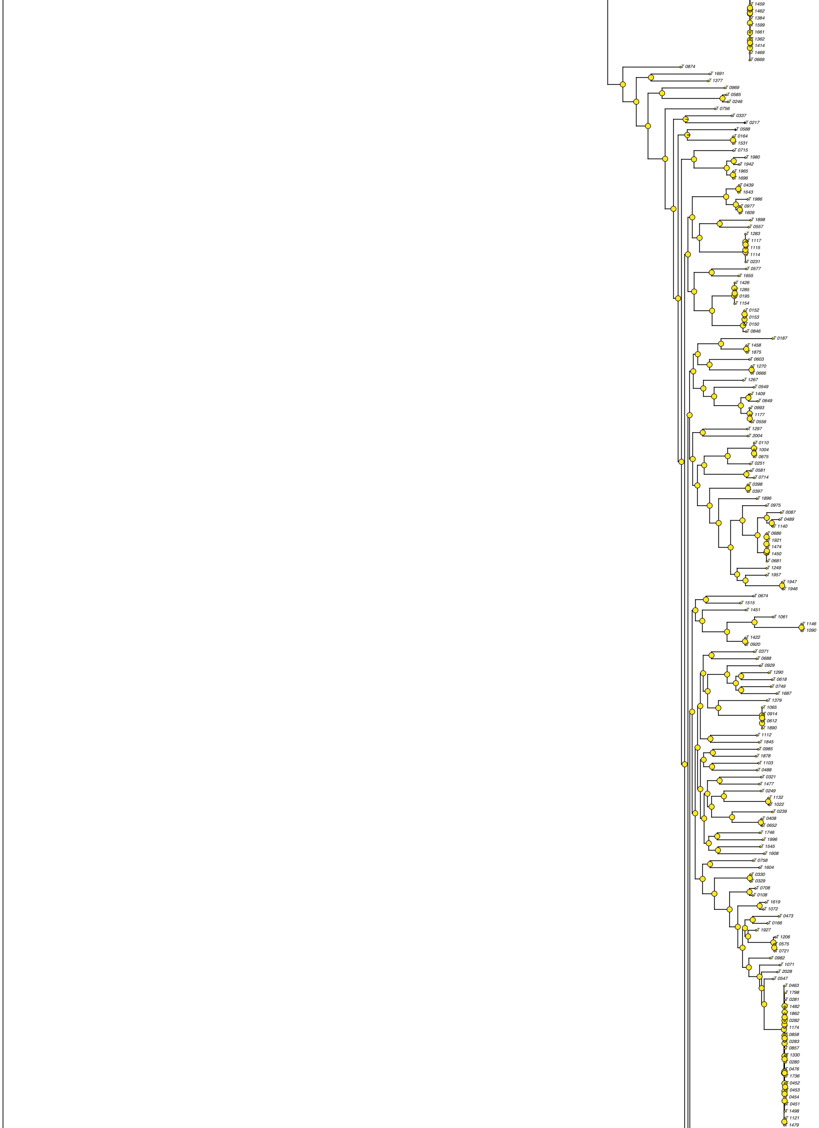
T 1459
T 1462
T 1384
T 1599
T 1661
T 1362
T 1414
T 1469
T 0669
T 0874
T 1691
T 1377
T 0969
T 0585
T 0246
T 0756
T 0337
T 0217
T 0588
T 0164
T 1531
T 0715
T 1980
T 1942
T 1965
T 1696
T 0439
T 1643
T 1986
T 0977
T 1609
T 1898
T 0557
T 1283
T 1117
T 1115
T 1114
T 0231
T 0577
T 1655
T 1426
T 1285
T 0195
T 1154
T 0152
T 0153
T 0150
T 0846
T 0187
T 1458
T 1875
T 0603
T 1270
T 0666
T 1267
T 0549
T 1409
T 0649
T 0993
T 1177
T 0556
T 1297
T 2004
T 0110
T 1004
T 0675
T 0251
T 0581
T 0714
T 0398
T 0397
T 1896
T 0975
T 0087
T 0489
T 1140
T 0686
T 1921
T 1474
T 1450
T 0681
T 1249
T 1957
T 1947
T 1946
T 0674
T 1515
T 1451
T 1061
T 1146
T 1090
T 1422
T 0920
T 0371
T 0688
T 0929
T 1290
T 0618
T 0749
T 1687
T 1379
T 1065
T 0914
T 0612
T 1890
T 1112
T 1845
T 0985
T 1878
T 1103
T 0488
T 0321
T 1477
T 0249
T 1132
T 1022
T 0239
T 0408
T 0652
T 1746
T 1996
T 1545
T 1608
T 0758
T 1604
T 0330
T 0329
T 0708
T 0108
T 1619
T 1072
T 0473
T 0166
T 1927
T 1206
T 0575
T 0721
T 0982
T 1071
T 2028
T 0547
T 0463
T 1798
T 0281
T 1482
T 1862
T 0282
T 1174
T 0858
T 0283
T 0857
T 1330
T 0280
T 0476
T 1736
T 0452
T 0453
T 0454
T 0451
T 1498
T 1121
T 1479

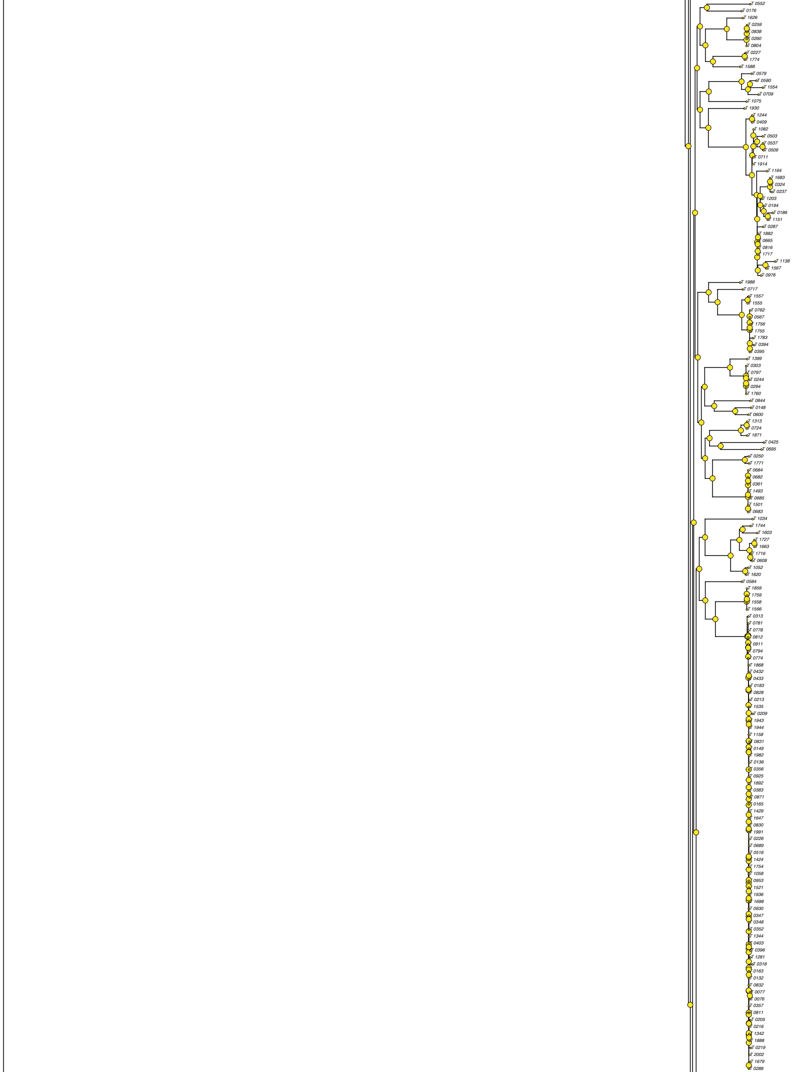
T 0552
T 0176
T 1626
T 0259
T 0838
T 0260
T 0804
T 0227
T 1774
T 1588
T 0579
T 0580
T 1554
T 0709
T 1075
T 1930
T 1244
T 0409
T 1082
T 0503
T 0537
T 0509
T 0711
T 1914
T 1164
T 1683
T 0324
T 0237
T 1203
T 0194
T 0186
T 1151
T 0287
T 1882
T 0665
T 0816
T 1717
T 1138
T 1567
T 0976
T 1988
T 0717
T 1557
T 1555
T 0762
T 0567
T 1756
T 1755
T 1783
T 0394
T 0395
T 1399
T 0303
T 0797
T 0244
T 0294
T 1760
T 0844
T 0148
T 0600
T 1313
T 0724
T 1871
T 0425
T 0695
T 0250
T 1771
T 0684
T 0682
T 0361
T 1493
T 0685
T 1501
T 0683
T 1034
T 1744
T 1603
T 1727
T 1663
T 1716
T 0608
T 1052
T 1620
T 0584
T 1859
T 1759
T 1558
T 1566
T 0313
T 0781
T 0778
T 0812
T 0911
T 0794
T 0774
T 1868
T 0432
T 0433
T 0183
T 0828
T 0213
T 1535
T 0209
T 1943
T 1944
T 1158
T 0831
T 0149
T 1982
T 0136
T 0356
T 0925
T 1892
T 0383
T 0871
T 0165
T 1429
T 1647
T 0830
T 1991
T 0226
T 0689
T 0516
T 1424
T 1754
T 1058
T 0953
T 1521
T 1936
T 1698
T 0930
T 0347
T 0348
T 0352
T 1344
T 0403
T 0396
T 1281
T 0318
T 0163
T 0132
T 0832
T 0077
T 0076
T 0357
T 0811
T 0205
T 0216
T 1342
T 1888
T 0219
T 2002
T 1679
T 0288

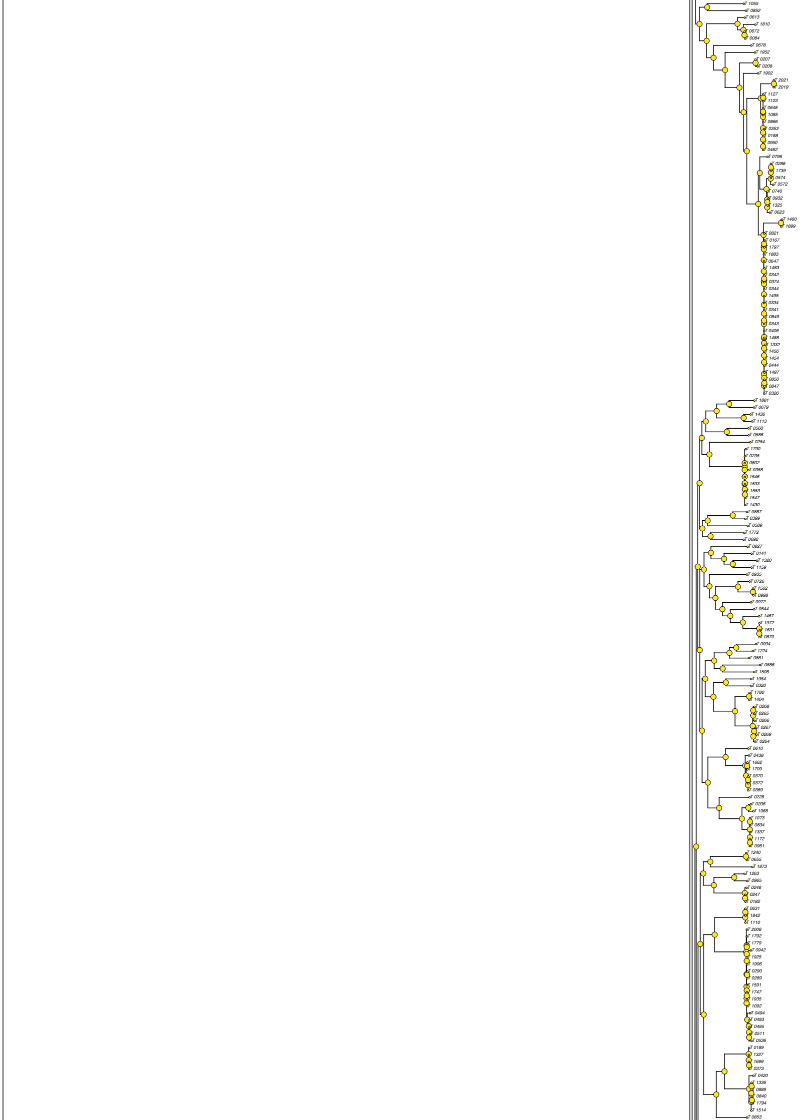

T 1055
T 0852
T 0613
T 1810
T 0672
T 0084
T 0678
T 1952
T 0207
T 0208
T 1902
T 2021
T 2019
T 1127
T 1123
T 0648
T 1085
T 0866
T 0353
T 0188
T 0950
T 0462
T 0796
T 0286
T 1739
T 0574
T 0572
T 0740
T 0932
T 1325
T 0623
T 1460
T 1899
T 0821
T 0167
T 1797
T 1883
T 0647
T 1483
T 0342
T 0374
T 0344
T 1495
T 0334
T 0341
T 0849
T 0343
T 0406
T 1488
T 1332
T 1456
T 1454
T 0444
T 1497
T 0850
T 0847
T 0326
T 1881
T 0679
T 1436
T 1113
T 0560
T 0586
T 0254
T 1790
T 0235
T 0802
T 0358
T 1546
T 1533
T 1553
T 1547
T 1430
T 0887
T 0399
T 0589
T 1772
T 0692
T 0827
T 0141
T 1320
T 1159
T 0935
T 0726
T 1562
T 0998
T 0972
T 0544
T 1467
T 1972
T 1631
T 0870
T 0094
T 1224
T 0861
T 0886
T 1506
T 1954
T 0320
T 1780
T 1404
T 0268
T 0265
T 0266
T 0267
T 0269
T 0264
T 0610
T 0438
T 1662
T 1709
T 0370
T 0372
T 0369
T 0228
T 0206
T 1968
T 1073
T 0834
T 1337
T 1172
T 0961
T 1240
T 0655
T 1873
T 1263
T 0965
T 0248
T 0247
T 0182
T 0631
T 1842
T 1110
T 2008
T 1792
T 1779
T 0942
T 1925
T 1906
T 0290
T 0289
T 1591
T 1747
T 1935
T 1092
T 0494
T 0493
T 0495
T 0511
T 0536
T 0189
T 1327
T 1699
T 0373
T 0420
T 1336
T 0889
T 0840
T 1794
T 1514
T 0853

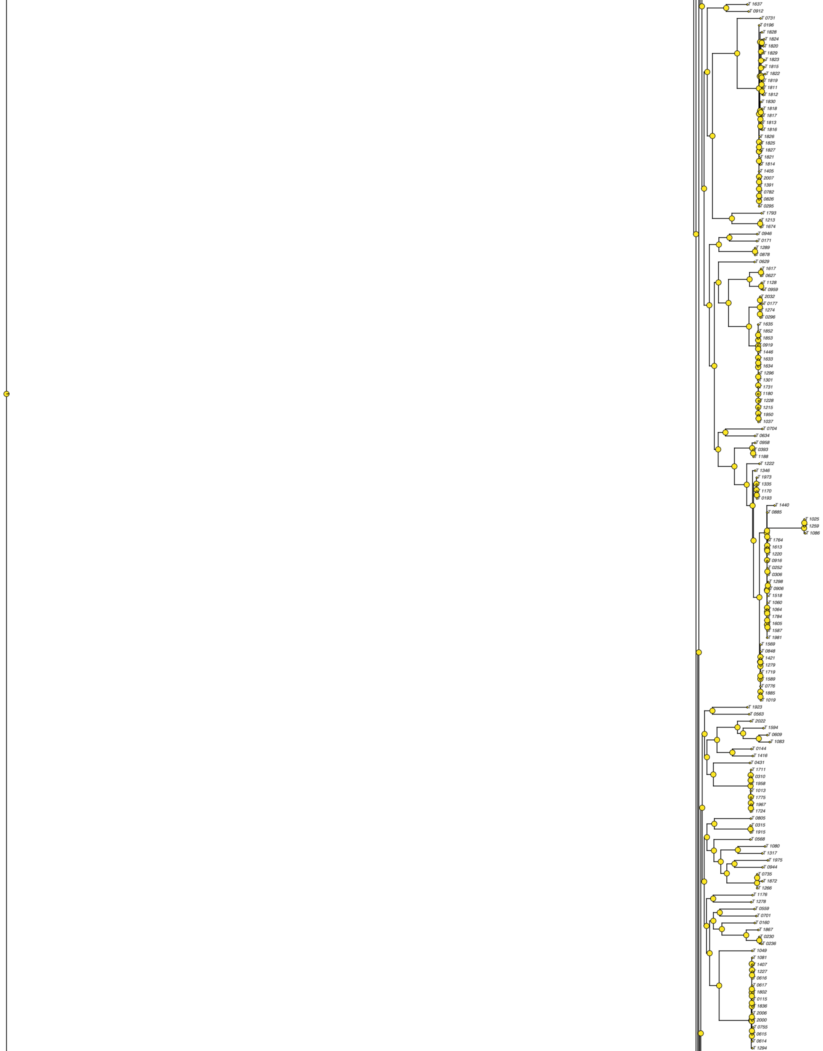
T 1637
T 0912
T 0731
T 0196
T 1828
T 1824
T 1820
T 1829
T 1823
T 1815
T 1822
T 1819
T 1811
T 1812
T 1830
T 1818
T 1817
T 1813
T 1816
T 1826
T 1825
T 1827
T 1821
T 1814
T 1405
T 2007
T 1391
T 0782
T 0826
T 0295
T 1793
T 1213
T 1674
T 0946
T 0171
T 1289
T 0878
T 0629
T 1617
T 0627
T 1128
T 0959
T 2032
T 0177
T 1274
T 0296
T 1635
T 1852
T 1853
T 0919
T 1446
T 1633
T 1634
T 1296
T 1301
T 1731
T 1180
T 1228
T 1215
T 1950
T 1037
T 0704
T 0634
T 0958
T 0393
T 1188
T 1222
T 1346
T 1973
T 1335
T 1170
T 0193
T 1440
T 0885
T 1025
T 1259
T 1086
T 1764
T 1613
T 1220
T 0916
T 0252
T 0306
T 1298
T 0906
T 1518
T 1060
T 1064
T 1784
T 1605
T 1587
T 1981
T 1569
T 0848
T 1421
T 1279
T 1719
T 1589
T 0776
T 1885
T 1019
T 1923
T 0563
T 2022
T 1594
T 0609
T 1083
T 0144
T 1416
T 0431
T 1711
T 0310
T 1958
T 1013
T 1775
T 1967
T 1724
T 0805
T 0315
T 1915
T 0568
T 1080
T 1317
T 1975
T 0944
T 0735
T 1872
T 1266
T 1176
T 1278
T 0559
T 0701
T 0160
T 1867
T 0230
T 0236
T 1049
T 1081
T 1407
T 1227
T 0616
T 0617
T 1802
T 0115
T 1836
T 2006
T 2000
T 0755
T 0615
T 0614
T 1294

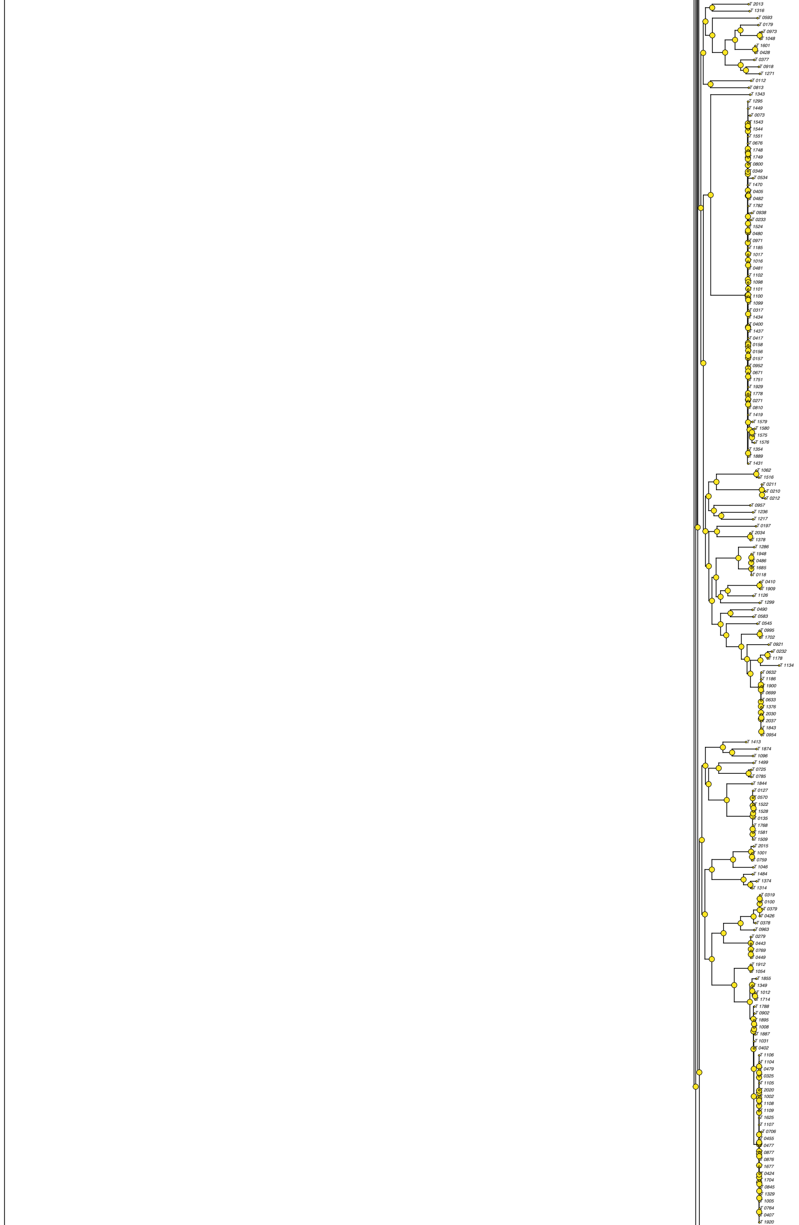
T 2013
T 1316
T 0593
T 0179
T 0973
T 1048
T 1601
T 0428
T 0377
T 0918
T 1271
T 0112
T 0813
T 1343
T 1295
T 1449
T 0073
T 1543
T 1544
T 1551
T 0676
1748
1749
0800
0349
T 0534
T 1470
0405
0482
T 1782
T 0938
T 0233
1524
0480
0971
T 1185
1017
1016
T 0481
T 1102
1098
T 1101
1100
T 1099
T 0317
1434
T 0400
T 1437
T 0417
0158
0156
0157
T 0952
0671
T 1751
T 1929
1778
0271
0810
T 1419
T 1579
T 1580
T 1575
T 1576
T 1354
T 1889
T 1431
T 1062
T 1516
T 0211
T 0210
T 0212
T 0957
T 1236
T 1217
T 0197
T 2034
T 1378
T 1286
T 1948
0486
1685
T 0118
T 0410
T 1909
T 1126
T 1299
T 0490
T 0583
T 0545
T 0995
T 1702
T 0921
T 0232
T 1178
T 1134
T 0632
T 1186
T 1900
T 0699
T 0633
T 1376
2030
T 2037
T 1843
T 0954
T 1413
T 1874
T 1096
T 1499
T 0725
T 0785
T 1844
T 0127
0570
T 1522
T 1528
T 0135
T 1768
T 1581
T 1509
T 2015
T 1001
T 0759
T 1046
T 1484
T 1374
T 1314
T 0319
0100
T 0379
T 0426
T 0378
T 0963
T 0279
0443
0769
T 0449
T 1912
T 1054
T 1855
1349
T 1012
T 1714
T 1788
T 0902
1895
1008
T 1887
T 1031
0402
T 1106
T 1104
0479
0325
T 1105
2020
1002
1108
1109
T 1625
T 1107
T 0706
0455
0477
0877
0876
1677
T 0424
1704
T 0845
T 1329
1005
T 0764
0407
T 1920

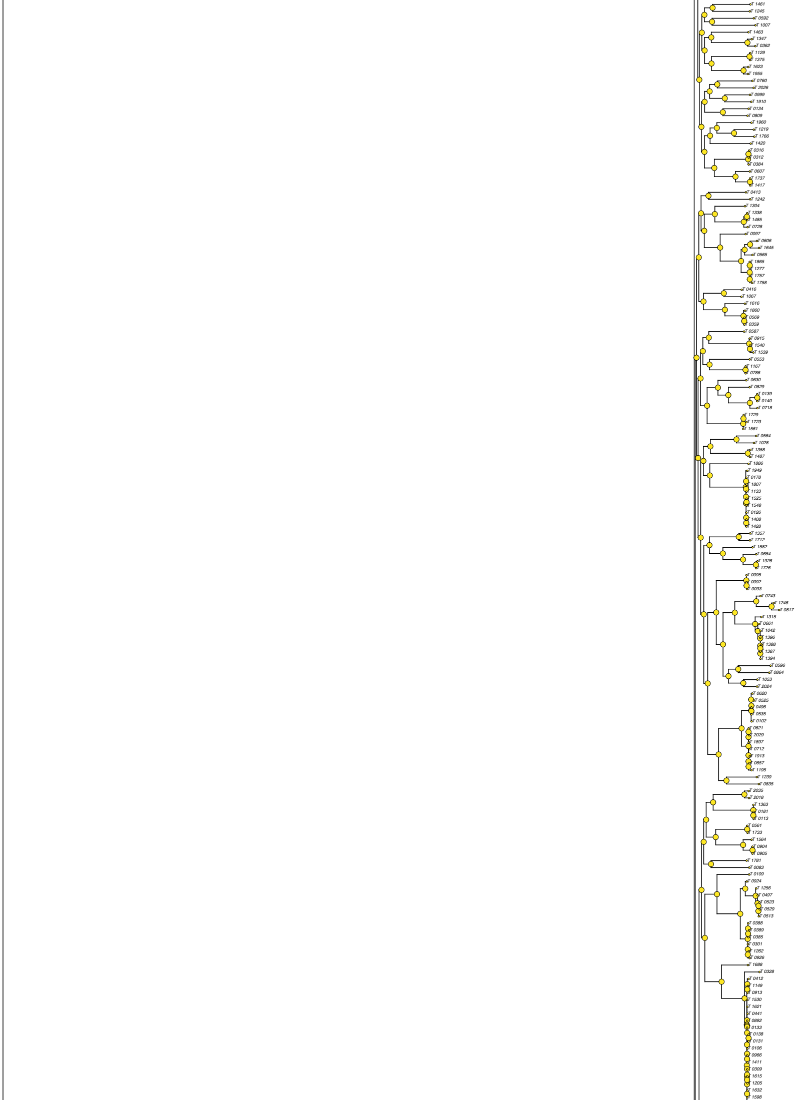
T 1461
T 1245
T 0592
T 1007
T 1463
T 1347
T 0362
T 1129
T 1375
T 1623
T 1955
T 0760
T 2026
T 0999
T 1910
T 0134
T 0809
T 1960
T 1219
T 1766
T 1420
T 0316
T 0312
T 0384
T 0607
T 1737
T 1417
T 0413
T 1242
T 1304
T 1338
T 1485
T 0728
T 0097
T 0606
T 1645
T 0565
T 1865
T 1277
T 1757
T 1758
T 0416
T 1067
T 1616
T 1860
T 0569
T 0359
T 0587
T 0915
T 1540
T 1539
T 0553
T 1167
T 0786
T 0630
T 0829
T 0139
T 0140
T 0718
T 1729
T 1723
T 1561
T 0564
T 1028
T 1358
T 1487
T 1886
T 1949
T 0178
T 1807
T 1133
T 1525
T 1548
T 0126
T 1408
T 1428
T 1357
T 1712
T 1582
T 0654
T 1926
T 1726
T 0095
T 0092
T 0093
T 0743
T 1246
T 0817
T 1315
T 0661
T 1042
T 1396
T 1388
T 1387
T 1394
T 0596
T 0864
T 1053
T 2024
T 0620
T 0525
T 0496
T 0535
T 0102
T 0621
T 2029
T 1897
T 0712
T 1913
T 0657
T 1195
T 1239
T 0835
T 2035
T 2018
T 1363
T 0181
T 0113
T 0561
T 1733
T 1564
T 0904
T 0905
T 1781
T 0083
T 0109
T 0924
T 1256
T 0497
T 0523
T 0529
T 0513
T 0388
T 0389
T 0385
T 0301
T 1262
T 0926
T 1688
T 0328
T 0412
T 1149
T 0913
T 1530
T 1621
T 0441
T 0892
T 0133
T 0138
T 0131
T 0106
T 0966
T 1411
T 0309
T 1615
T 1205
T 1632
T 1598

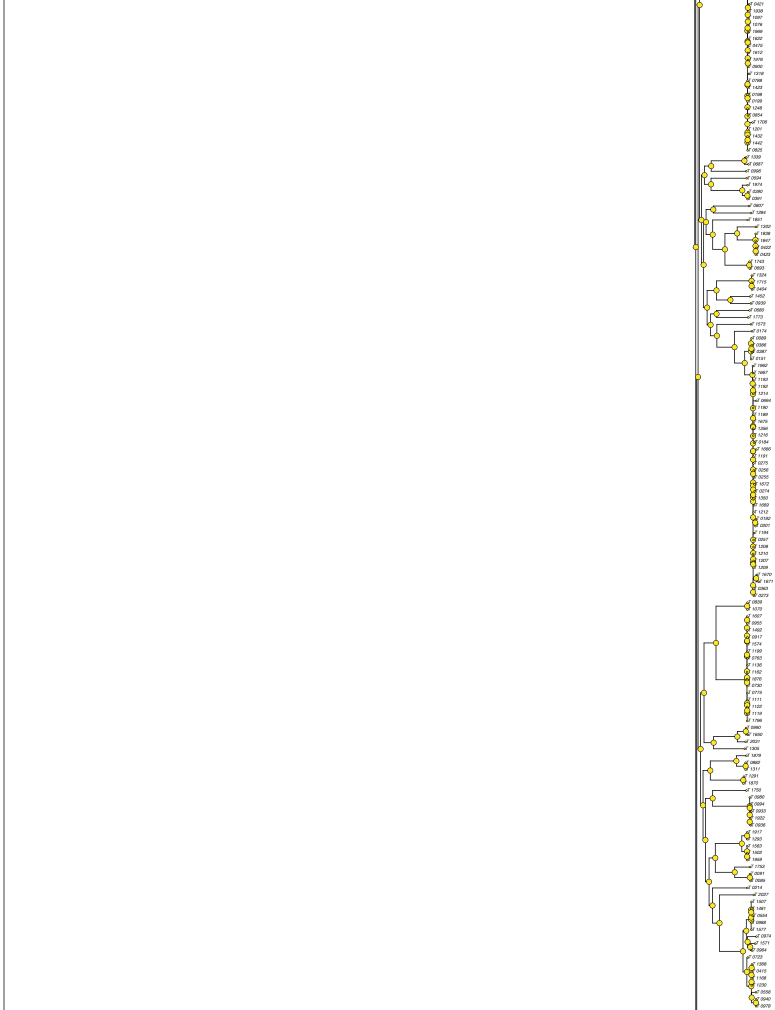

T 0421
T 1938
T 1097
T 1076
T 1969
T 1622
T 0475
T 1612
T 1976
T 0900
T 1318
T 0788
T 1423
T 0198
T 0199
T 1248
T 0854
T 1706
T 1201
T 1432
T 1442
T 0825
T 1339
T 0687
T 0996
T 0594
T 1974
T 0390
T 0391
T 0807
T 1284
T 1851
T 1302
T 1838
T 1847
T 0422
T 0423
T 1743
T 0693
T 1324
T 1715
T 0404
T 1452
T 0939
T 0680
T 1773
T 1573
T 0174
T 0089
T 0386
T 0387
T 0151
T 1962
T 1667
T 1193
T 1192
T 1214
T 0694
T 1190
T 1189
T 1675
T 1356
T 1216
T 0184
T 1666
T 1191
T 0275
T 0256
T 0255
T 1672
T 0274
T 1350
T 1669
T 1212
T 0192
T 0201
T 1194
T 0257
T 1208
T 1210
T 1207
T 1209
T 1670
T 1671
T 0363
T 0273
T 0839
T 1070
T 1607
T 0955
T 1492
T 0917
T 1574
T 1199
T 0763
T 1136
T 1162
T 1876
T 0730
T 0775
T 1111
T 1122
T 1119
T 1796
T 0990
T 1650
T 2031
T 1305
T 1879
T 0882
T 1311
T 1291
T 1870
T 1750
T 0980
T 0994
T 0933
T 1922
T 0936
T 1917
T 1293
T 1563
T 1502
T 1959
T 1753
T 0091
T 0085
T 0214
T 2027
T 1507
T 1481
T 0554
T 0988
T 1577
T 0974
T 1571
T 0964
T 0723
T 1368
T 0415
T 1168
T 1230
T 0558
T 0940
T 0978

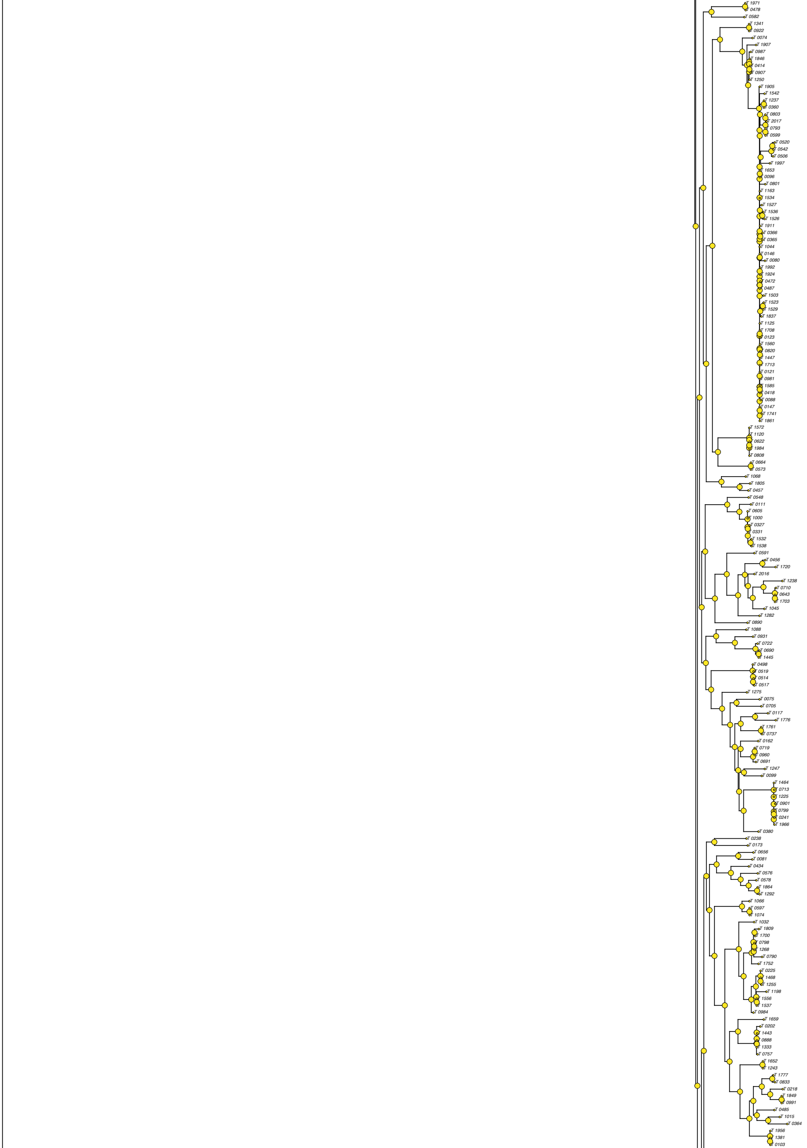
T 1971
T 0478
T 0582
T 1341
T 0922
T 0074
T 1907
T 0987
T 1846
T 0414
T 0907
T 1250
T 1905
T 1542
T 1237
T 0360
T 0803
T 2017
T 0793
T 0599
T 0520
T 0542
T 0506
T 1997
T 1653
0096
T 0801
T 1163
1534
T 1527
T 1536
T 1526
T 1911
T 0366
T 0365
T 1044
T 0146
T 0080
T 1992
T 1924
T 0472
T 0487
T 1503
T 1523
T 1529
T 1837
T 1125
T 1708
T 0123
T 1560
T 0820
T 1447
T 1713
T 0121
T 0981
T 1585
T 0418
T 0088
T 0147
T 1741
T 1861
T 1572
T 1120
T 0622
T 1984
T 0808
T 0664
T 0573
T 1068
T 1805
T 0457
T 0548
T 0111
T 0605
1000
T 0327
0331
T 1532
T 1538
T 0591
T 0456
T 1720
T 2016
T 1238
T 0710
0643
T 1703
T 1045
T 1282
T 0890
T 1088
T 0931
T 0722
T 0690
T 1445
T 0498
T 0519
T 0514
T 0517
T 1275
T 0075
T 0705
T 0117
T 1776
T 1761
T 0737
T 0162
T 0719
T 0960
T 0691
T 1247
T 0099
T 1464
T 0713
T 1225
T 0901
T 0799
T 0241
T 1966
T 0380
T 0238
T 0173
T 0656
T 0081
T 0434
T 0576
T 0578
T 1864
T 1292
T 1066
T 0597
T 1074
T 1032
T 1809
T 1700
T 0798
T 1268
T 0790
T 1752
T 0225
T 1468
T 1255
T 1198
T 1556
T 1537
T 0984
T 1659
T 0202
T 1443
T 0888
T 1333
T 0757
T 1652
T 1243
T 1777
T 0833
T 0218
T 1849
T 0991
T 0485
T 1015
T 0364
T 1956
T 1381
T 0103

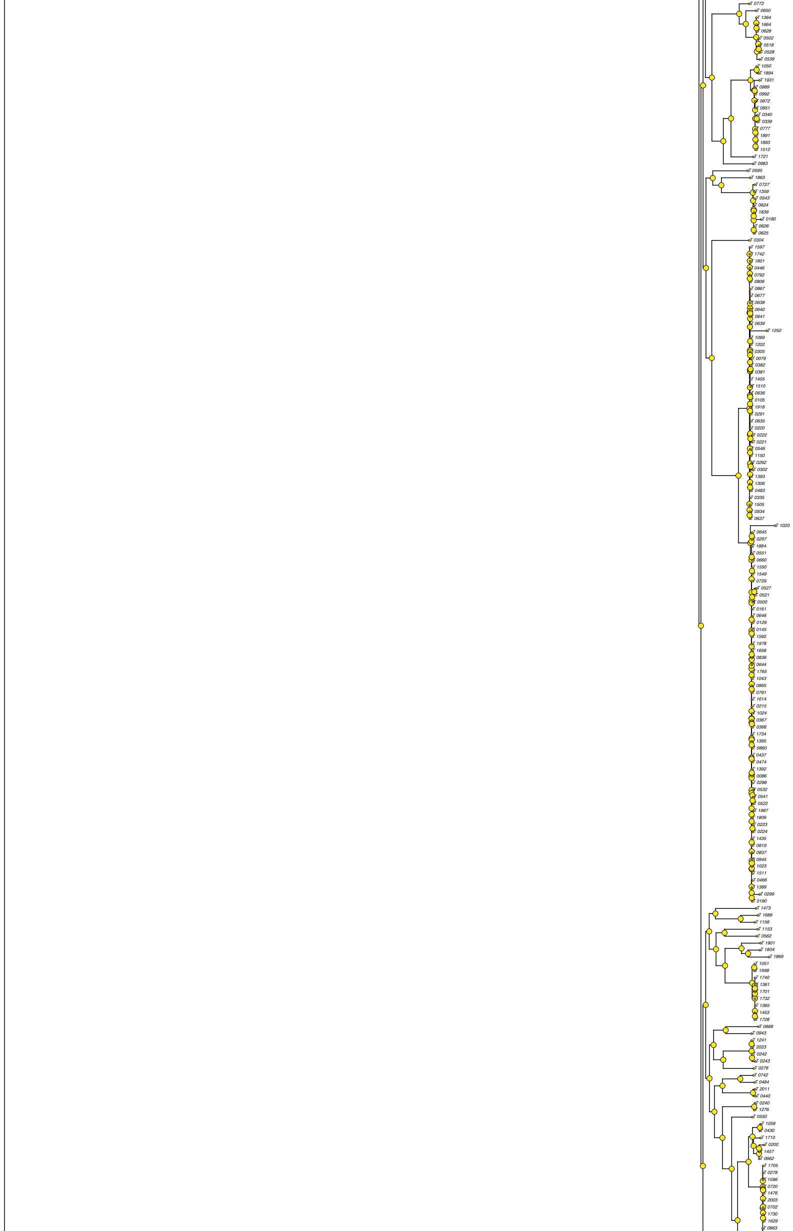

T 0772
T 0650
T 1364
T 1664
T 0628
T 0502
T 0518
T 0528
T 0539
T 1050
T 1894
T 1931
T 0989
T 0992
T 0872
T 0951
T 0340
T 0339
T 0777
T 1891
T 1893
T 1512
T 1721
T 0983
T 0595
T 1863
T 0727
T 1359
T 0543
T 0624
T 1839
T 0180
T 0626
T 0625
T 0304
T 1597
T 1742
T 1801
T 0446
T 0792
T 0806
T 0867
T 0677
T 0638
T 0640
T 0641
T 0639
T 1252
T 1069
T 1202
T 0305
T 0079
T 0382
T 0381
T 1455
T 1510
T 0636
T 0105
T 1918
T 0291
T 0635
T 0220
T 0222
T 0221
T 0546
T 1150
T 0292
T 0302
T 1393
T 1306
T 0483
T 0335
T 1505
T 0934
T 0637
T 1020
T 0645
T 0297
T 1884
T 0551
T 0660
T 1550
T 1549
T 0729
T 0527
T 0521
T 0500
T 0161
T 0646
T 0129
T 0145
T 1592
T 1978
T 1658
T 0836
T 0644
T 1765
T 1043
T 0865
T 0791
T 1614
T 0215
T 1024
T 0367
T 0368
T 1734
T 1395
T 0860
T 0437
T 0474
T 1392
T 0086
T 0298
T 0532
T 0541
T 0522
T 1987
T 1806
T 0223
T 0224
T 1435
T 0819
T 0837
T 0945
T 1023
T 1511
T 0466
T 1389
T 0299
T 0190
T 1473
T 1689
T 1156
T 1153
T 0562
T 1901
T 1804
T 1869
T 1051
T 1998
T 1740
T 1361
T 1701
T 1732
T 1365
T 1453
T 1728
T 0868
T 0943
T 1241
T 2023
T 0242
T 0243
T 0276
T 0742
T 0484
T 2011
T 0440
T 0240
T 1276
T 0550
T 1059
T 0430
T 1710
T 0200
T 1457
T 0662
T 1705
T 0278
T 1586
T 0720
T 1476
T 2003
T 0702
T 1730
T 1629
T 0863

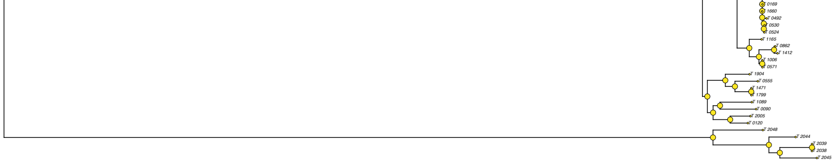
0169
1660
T 0492
T 0530
T 0524
T 1165
T 0862
T 1412
T 1006
T 0571
T 1904
T 0555
T 1471
T 1799
T 1089
T 0090
T 2005
T 0120
T 2048
T 2044
T 2039
T 2038
T 2045

tse7c
X

Effector gene: tse7c

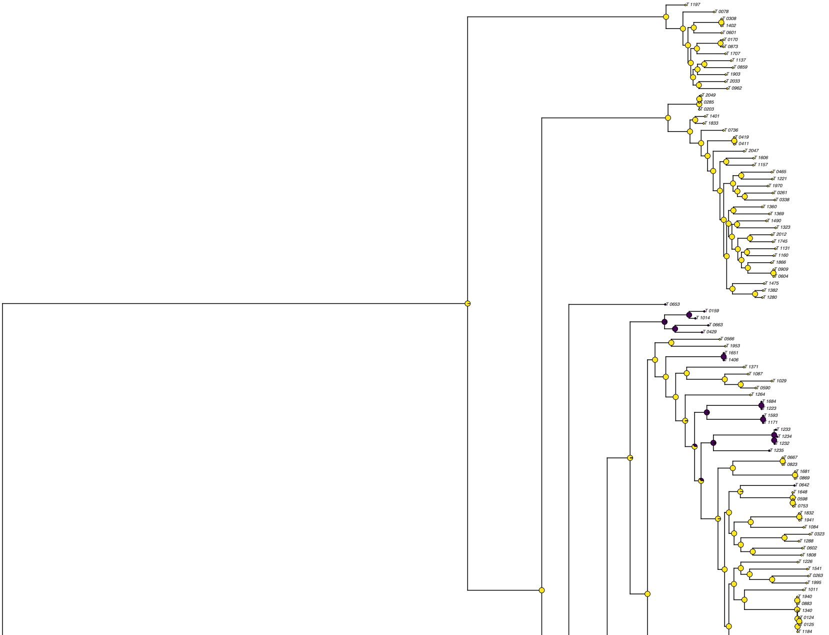

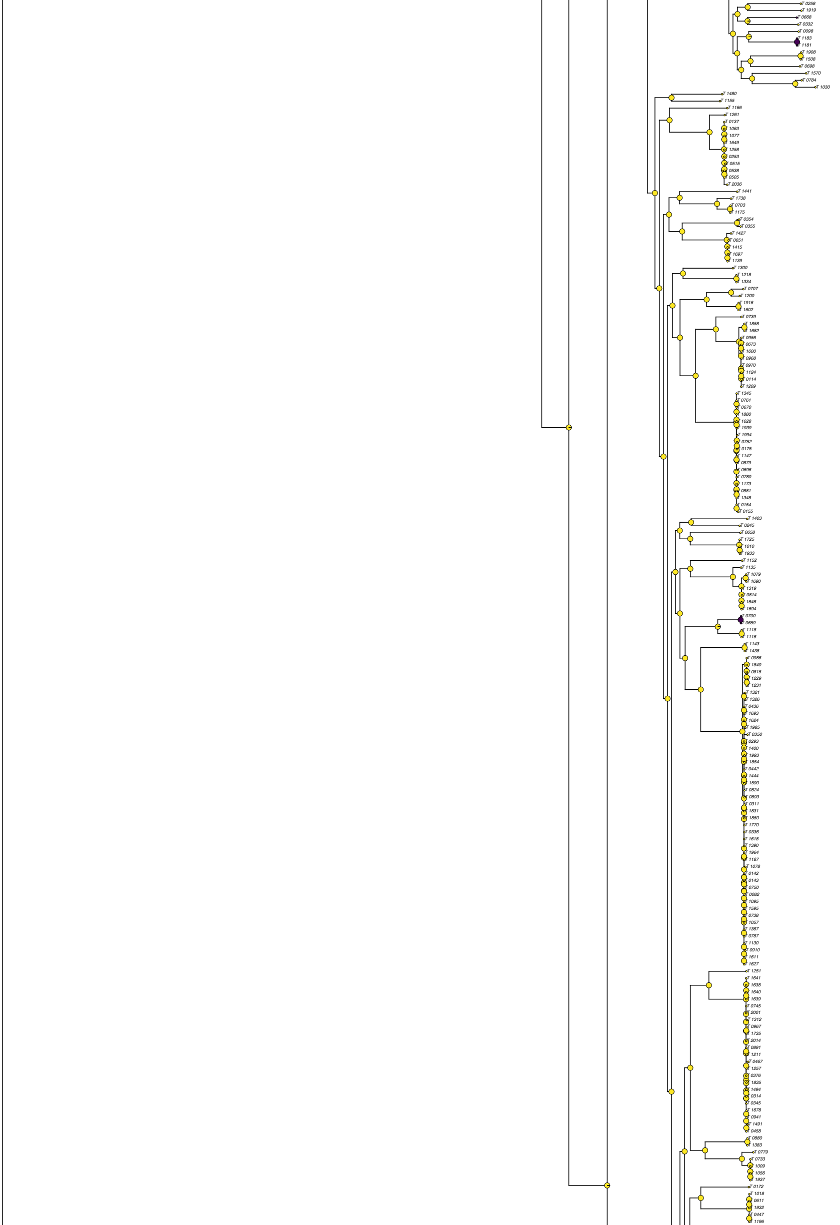
T 0258
T 1919
T 0668
T 0332
T 0098
T 1183
T 1181
T 1908
T 1508
T 0698
T 1570
T 0784
T 1030
T 1480
T 1155
T 1166
T 1261
T 0137
T 1063
T 1077
T 1649
T 1258
T 0253
T 0515
T 0538
T 0505
T 2036
T 1441
T 1738
T 0703
T 1175
T 0354
T 0355
T 1427
T 0651
T 1415
T 1697
T 1139
T 1300
T 1218
T 1334
T 0707
T 1200
T 1916
T 1602
T 0739
T 1858
T 1682
T 0956
T 0673
T 1600
T 0968
T 0970
T 1124
T 0114
T 1269
T 1345
T 0761
T 0670
T 1880
T 1628
T 1939
T 1994
T 0752
T 0175
T 1147
T 0879
T 0696
T 0780
T 1173
T 0881
T 1348
T 0154
T 0155
T 1403
T 0245
T 0658
T 1725
T 1010
T 1933
T 1152
T 1135
T 1079
T 1690
T 1319
T 0814
T 1646
T 1694
T 0700
T 0659
T 1118
T 1116
T 1143
T 1438
T 0986
T 1840
T 0815
T 1229
T 1231
T 1321
T 1326
T 0436
T 1693
T 1624
T 1985
T 0350
T 0293
T 1400
T 1993
T 1854
T 0442
T 1444
T 1590
T 0824
T 0893
T 0311
T 1831
T 1850
T 1770
T 0336
T 1618
T 1390
T 1964
T 1187
T 1078
T 0142
T 0143
T 0750
T 0082
T 1095
T 1595
T 0738
T 1057
T 1367
T 0787
T 1130
T 0910
T 1611
T 1627
T 1251
T 1641
T 1638
T 1640
T 1639
T 0745
T 2001
T 1312
T 0967
T 1735
T 2014
T 0891
T 1211
T 0467
T 1257
T 0376
T 1835
T 1494
T 0314
T 0345
T 1678
T 0941
T 1491
T 0458
T 0880
T 1383
T 0779
T 0733
T 1009
T 1056
T 1937
T 0172
T 1018
T 0611
T 1932
T 0447
T 1196

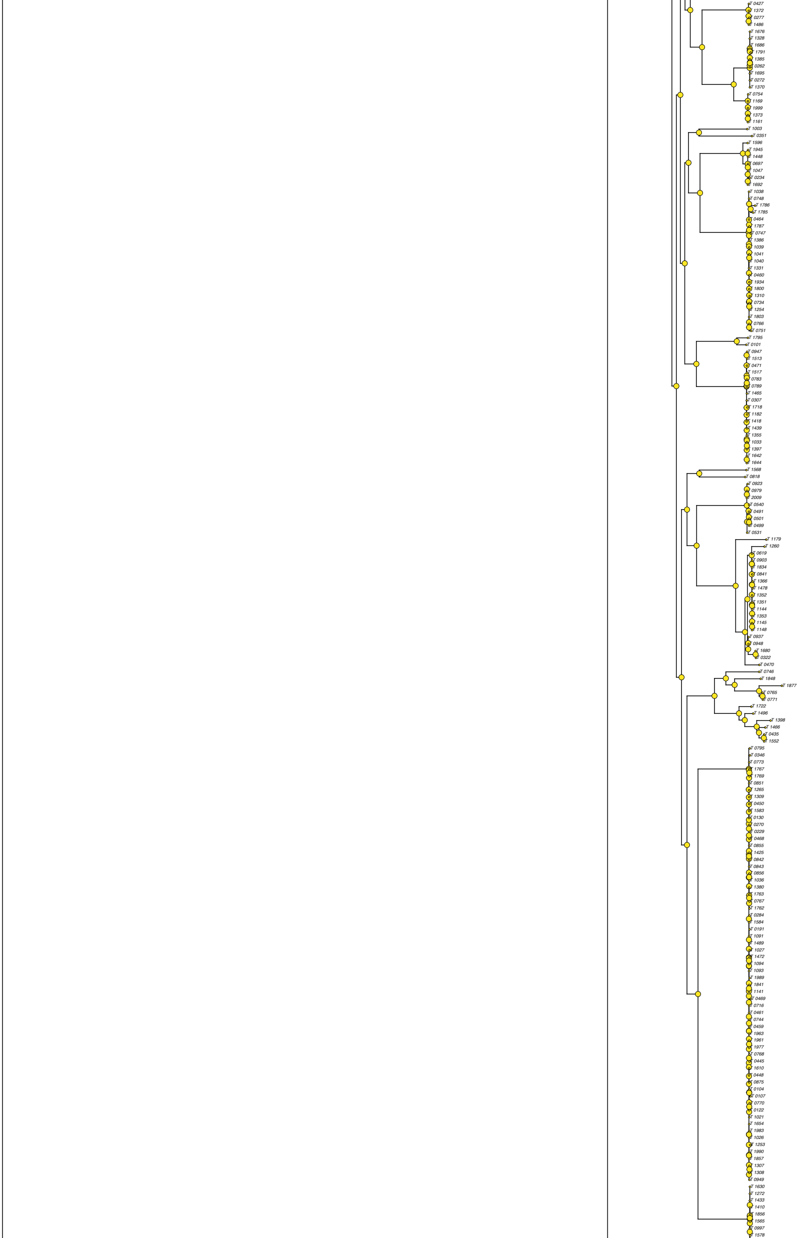

T 0427
T 1372
T 0277
T 1486
T 1676
T 1328
T 1686
T 1791
T 1385
T 0262
T 1695
T 0272
T 1370
T 0754
T 1169
T 1999
T 1373
T 1161
T 1003
T 0351
T 1596
T 1945
T 1448
T 0697
T 1047
T 0234
T 1692
T 1038
T 0748
T 1786
T 1785
T 0464
T 1787
T 0747
T 1386
T 1039
T 1041
T 1040
T 1331
T 0460
T 1934
T 1800
T 1310
T 0734
T 1254
T 1803
T 0766
T 0751
T 1795
T 0101
T 0947
T 1513
T 0471
T 1517
T 0783
T 0789
T 1465
T 0307
T 1718
T 1182
T 1418
T 1439
T 1355
T 1033
T 1397
T 1642
T 1644
T 1568
T 0818
T 0923
T 0979
T 2009
T 0540
T 0491
T 0501
T 0499
T 0531
T 1179
T 1260
T 0619
T 0903
T 1834
T 0841
T 1366
T 1478
T 1352
T 1351
T 1144
T 1353
T 1145
T 1148
T 0937
T 0948
T 1680
T 0322
T 0470
T 0746
T 1848
T 1877
T 0765
T 0771
T 1722
T 1496
T 1398
T 1466
T 0435
T 1552
T 0795
T 0346
T 0773
T 1767
T 1769
T 0851
T 1265
T 1309
T 0450
T 1583
T 0130
T 0270
T 0229
T 0468
T 0855
T 1425
T 0842
T 0843
T 0856
T 1036
T 1380
T 1763
T 0767
T 1762
T 0284
T 1584
T 0191
T 1091
T 1489
T 1027
T 1472
T 1094
T 1093
T 1989
T 1841
T 1141
T 0469
T 0716
T 0461
T 0744
T 0459
T 1963
T 1961
T 1977
T 0768
T 0445
T 1610
T 0448
T 0875
T 0104
T 0107
T 0770
T 0122
T 1021
T 1654
T 1983
T 1026
T 1253
T 1990
T 1857
T 1307
T 1308
T 0949
T 1630
T 1272
T 1433
T 1410
T 1856
T 1565
T 0997
T 1578

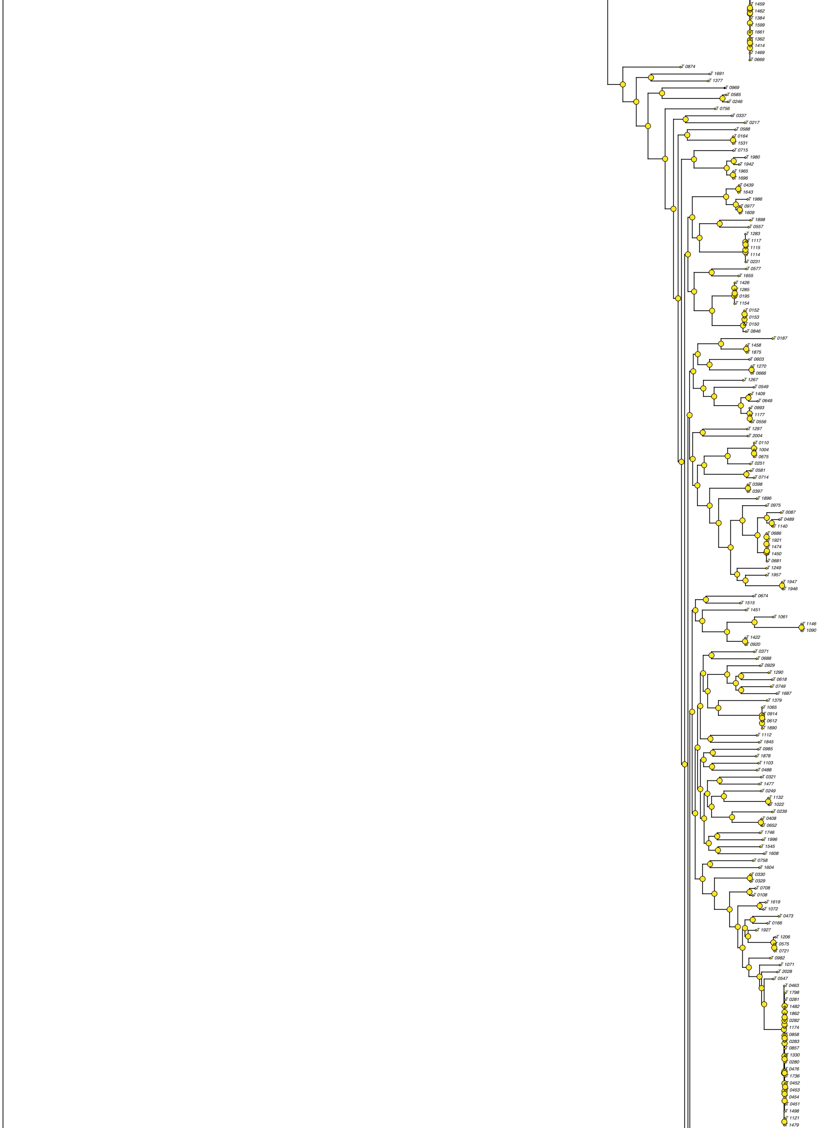
T 1459
T 1462
T 1384
T 1599
T 1661
T 1362
T 1414
T 1469
T 0669
T 0874
T 1691
T 1377
T 0969
T 0585
T 0246
T 0756
T 0337
T 0217
T 0588
T 0164
T 1531
T 0715
T 1980
T 1942
T 1965
T 1696
T 0439
T 1643
T 1986
T 0977
T 1609
T 1898
T 0557
T 1283
T 1117
T 1115
T 1114
T 0231
T 0577
T 1655
T 1426
T 1285
T 0195
T 1154
T 0152
T 0153
T 0150
T 0846
T 0187
T 1458
T 1875
T 0603
T 1270
T 0666
T 1267
T 0549
T 1409
T 0649
T 0993
T 1177
T 0556
T 1297
T 2004
T 0110
T 1004
T 0675
T 0251
T 0581
T 0714
T 0398
T 0397
T 1896
T 0975
T 0087
T 0489
T 1140
T 0686
T 1921
T 1474
T 1450
T 0681
T 1249
T 1957
T 1947
T 1946
T 0674
T 1515
T 1451
T 1061
T 1146
T 1090
T 1422
T 0920
T 0371
T 0688
T 0929
T 1290
T 0618
T 0749
T 1687
T 1379
T 1065
T 0914
T 0612
T 1890
T 1112
T 1845
T 0985
T 1878
T 1103
T 0488
T 0321
T 1477
T 0249
T 1132
T 1022
T 0239
T 0408
T 0652
T 1746
T 1996
T 1545
T 1608
T 0758
T 1604
T 0330
T 0329
T 0708
T 0108
T 1619
T 1072
T 0473
T 0166
T 1927
T 1206
T 0575
T 0721
T 0982
T 1071
T 2028
T 0547
T 0463
T 1798
T 0281
T 1482
T 1862
T 0282
T 1174
T 0858
T 0283
T 0857
T 1330
T 0280
T 0476
T 1736
T 0452
T 0453
T 0454
T 0451
T 1498
T 1121
T 1479

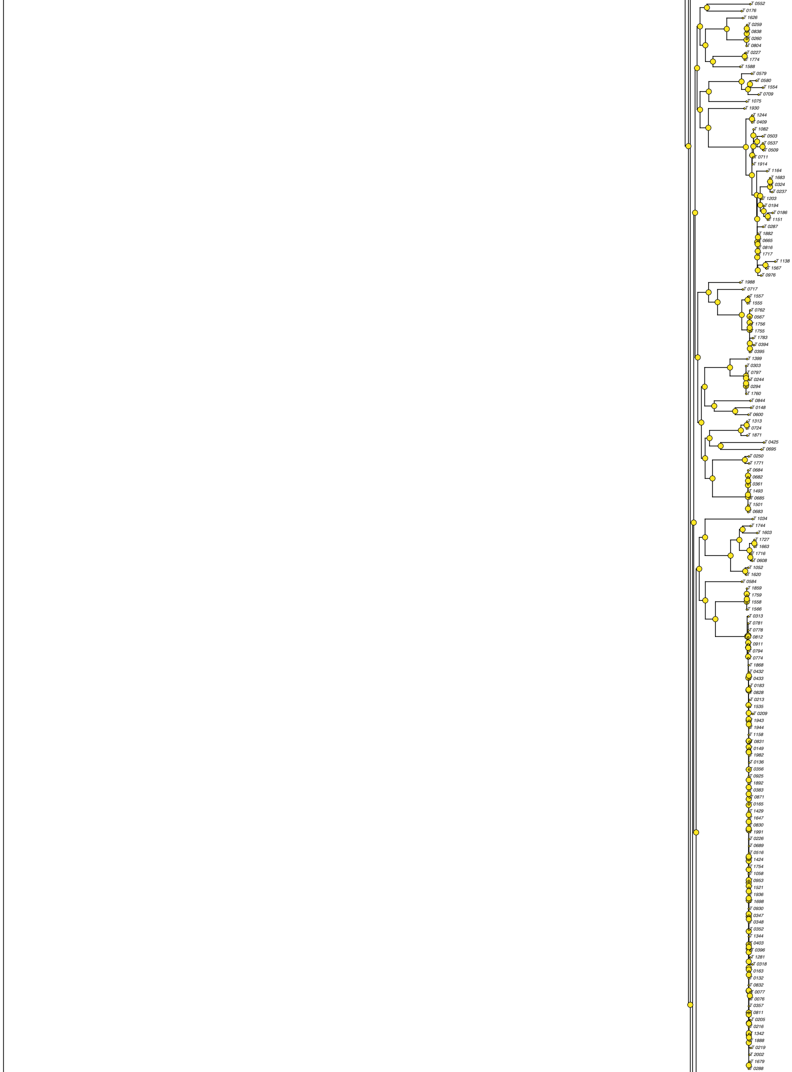

T 0552
T 0176
T 1626
T 0259
T 0838
T 0260
T 0804
T 0227
T 1774
T 1588
T 0579
T 0580
T 1554
T 0709
T 1075
T 1930
T 1244
T 0409
T 1082
T 0503
T 0537
T 0509
T 0711
T 1914
T 1164
T 1683
T 0324
T 0237
T 1203
T 0194
T 0186
T 1151
T 0287
T 1882
T 0665
T 0816
T 1717
T 1138
T 1567
T 0976
T 1988
T 0717
T 1557
T 1555
T 0762
T 0567
T 1756
T 1755
T 1783
T 0394
T 0395
T 1399
T 0303
T 0797
T 0244
T 0294
T 1760
T 0844
T 0148
T 0600
T 1313
T 0724
T 1871
T 0425
T 0695
T 0250
T 1771
T 0684
T 0682
T 0361
T 1493
T 0685
T 1501
T 0683
T 1034
T 1744
T 1603
T 1727
T 1663
T 1716
T 0608
T 1052
T 1620
T 0584
T 1859
T 1759
T 1558
T 1566
T 0313
T 0781
T 0778
T 0812
T 0911
T 0794
T 0774
T 1868
T 0432
T 0433
T 0183
T 0828
T 0213
T 1535
T 0209
T 1943
T 1944
T 1158
T 0831
T 0149
T 1982
T 0136
T 0356
T 0925
T 1892
T 0383
T 0871
T 0165
T 1429
T 1647
T 0830
T 1991
T 0226
T 0689
T 0516
T 1424
T 1754
T 1058
T 0953
T 1521
T 1936
T 1698
T 0930
T 0347
T 0348
T 0352
T 1344
T 0403
T 0396
T 1281
T 0318
T 0163
T 0132
T 0832
T 0077
T 0076
T 0357
T 0811
T 0205
T 0216
T 1342
T 1888
T 0219
T 2002
T 1679
T 0288

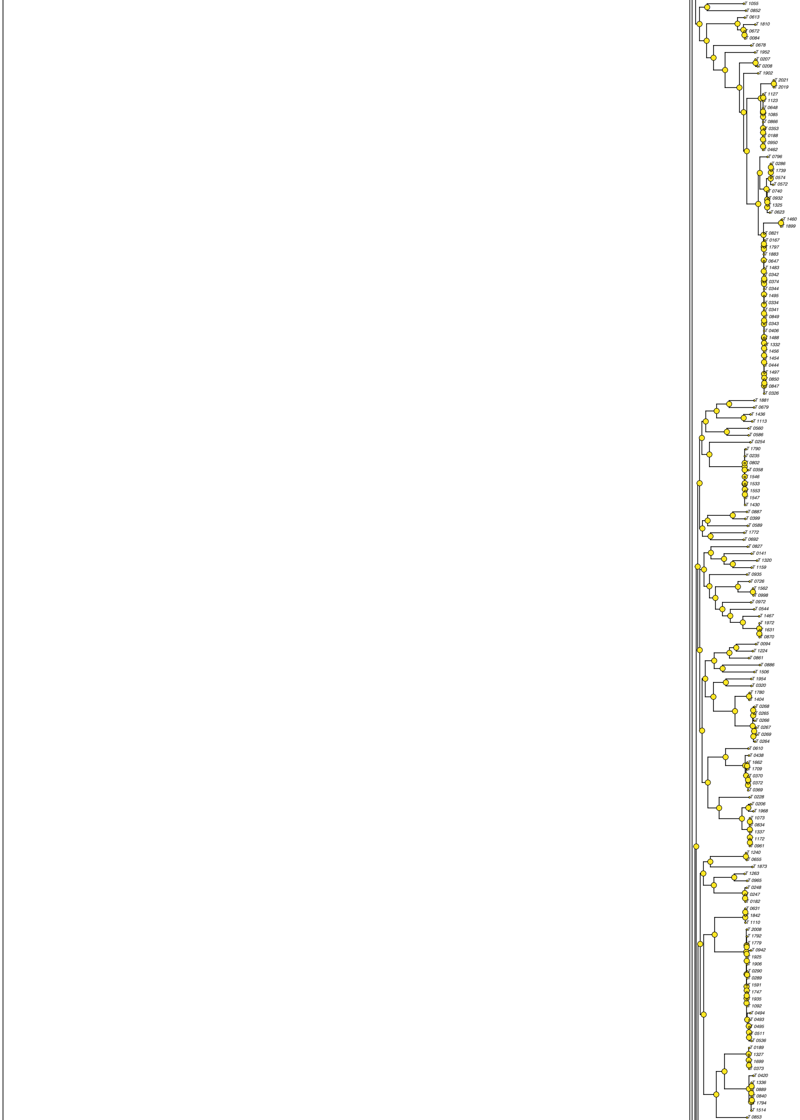
T 1055
T 0852
T 0613
T 1810
T 0672
T 0084
T 0678
T 1952
T 0207
T 0208
T 1902
T 2021
T 2019
T 1127
T 1123
T 0648
T 1085
T 0866
T 0353
T 0188
T 0950
T 0462
T 0796
T 0286
T 1739
T 0574
T 0572
T 0740
T 0932
T 1325
T 0623
T 1460
T 1899
T 0821
T 0167
T 1797
T 1883
T 0647
T 1483
T 0342
T 0374
T 0344
T 1495
T 0334
T 0341
T 0849
T 0343
T 0406
T 1488
T 1332
T 1456
T 1454
T 0444
T 1497
T 0850
T 0847
T 0326
T 1881
T 0679
T 1436
T 1113
T 0560
T 0586
T 0254
T 1790
T 0235
T 0802
T 0358
T 1546
T 1533
T 1553
T 1547
T 1430
T 0887
T 0399
T 0589
T 1772
T 0692
T 0827
T 0141
T 1320
T 1159
T 0935
T 0726
T 1562
T 0998
T 0972
T 0544
T 1467
T 1972
T 1631
T 0870
T 0094
T 1224
T 0861
T 0886
T 1506
T 1954
T 0320
T 1780
T 1404
T 0268
T 0265
T 0266
T 0267
T 0269
T 0264
T 0610
T 0438
T 1662
T 1709
T 0370
T 0372
T 0369
T 0228
T 0206
T 1968
T 1073
T 0834
T 1337
T 1172
T 0961
T 1240
T 0655
T 1873
T 1263
T 0965
T 0248
T 0247
T 0182
T 0631
T 1842
T 1110
T 2008
T 1792
T 1779
T 0942
T 1925
T 1906
T 0290
T 0289
T 1591
T 1747
T 1935
T 1092
T 0494
T 0493
T 0495
T 0511
T 0536
T 0189
T 1327
T 1699
T 0373
T 0420
T 1336
T 0889
T 0840
T 1794
T 1514
T 0853

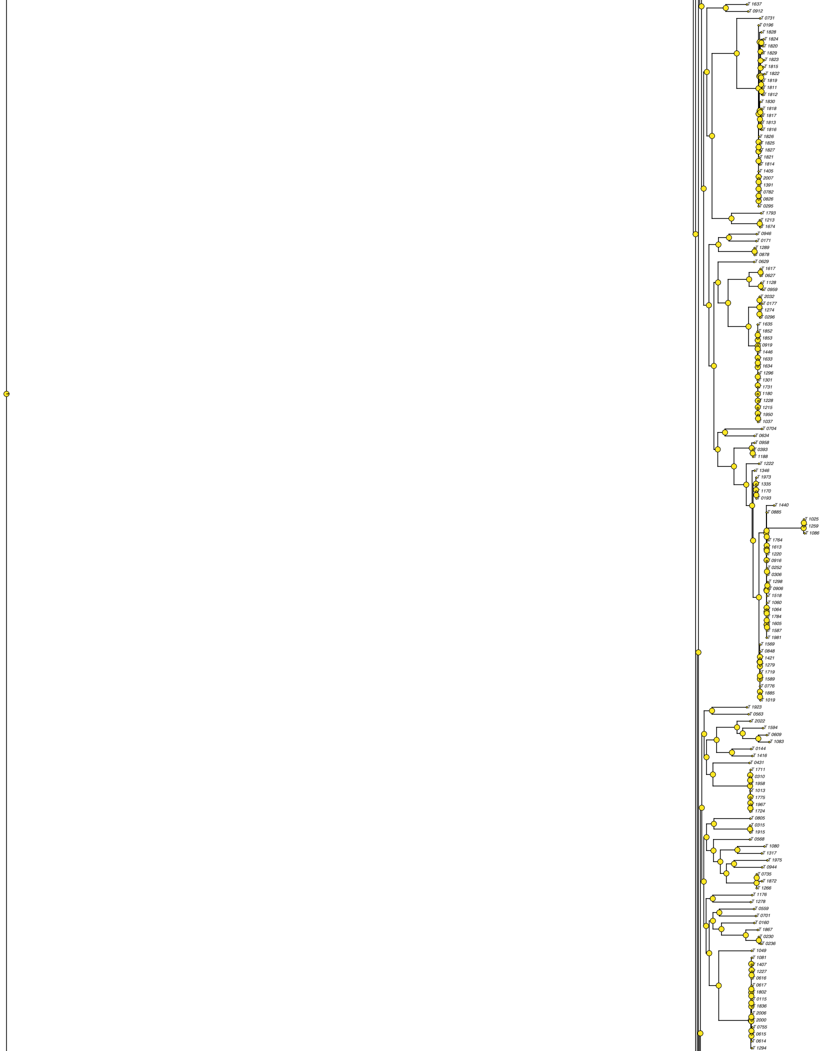

T 1637
T 0912
T 0731
T 0196
T 1828
T 1824
T 1820
T 1829
T 1823
T 1815
T 1822
T 1819
T 1811
T 1812
T 1830
T 1818
T 1817
T 1813
T 1816
T 1826
T 1825
T 1827
T 1821
T 1814
T 1405
2007
1391
0782
0826
0295
T 1793
T 1213
T 1674
T 0946
T 0171
T 1289
0878
T 0629
T 1617
0627
T 1128
T 0959
T 2032
T 0177
T 1274
T 0296
T 1635
T 1852
1853
0919
T 1446
1633
1634
T 1296
T 1301
1731
1180
1228
1215
1950
T 1037
T 0704
T 0634
T 0958
0393
T 1188
T 1222
T 1346
T 1973
1335
1170
0193
T 1440
T 0885
T 1025
1259
T 1086
T 1764
1613
T 1220
0916
T 0252
0306
T 1298
T 0906
T 1518
T 1060
1064
1784
1605
T 1587
T 1981
T 1569
T 0848
1421
1279
T 1719
1589
T 0776
1885
T 1019
T 1923
T 0563
T 2022
T 1594
T 0609
T 1083
T 0144
T 1416
T 0431
T 1711
0310
1958
T 1013
1775
1967
1724
T 0805
T 0315
T 1915
T 0568
T 1080
T 1317
T 1975
T 0944
T 0735
T 1872
T 1266
T 1176
T 1278
T 0559
T 0701
T 0160
T 1867
T 0230
T 0236
T 1049
T 1081
1407
1227
0616
T 0617
1802
T 0115
1836
T 2006
2000
T 0755
0615
T 0614
T 1294

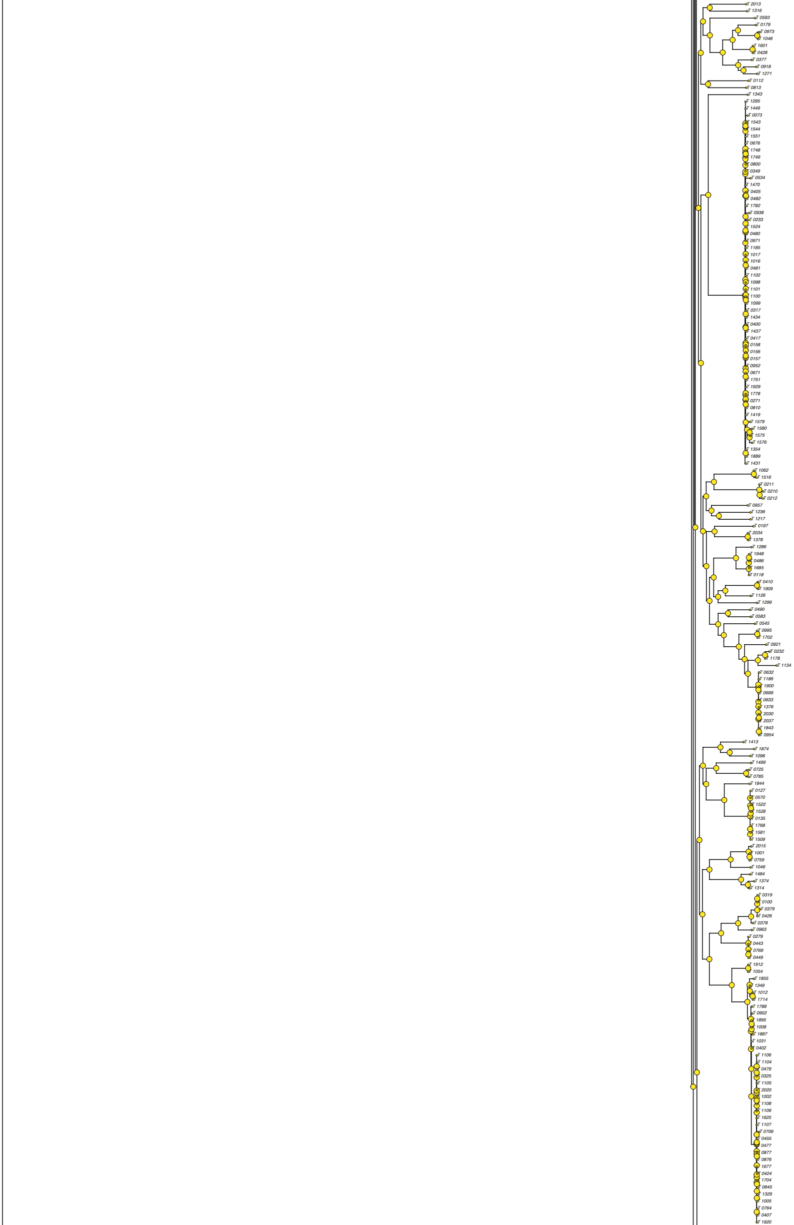
T 2013
T 1316
T 0593
T 0179
T 0973
T 1048
T 1601
T 0428
T 0377
T 0918
T 1271
T 0112
T 0813
T 1343
T 1295
T 1449
T 0073
T 1543
T 1544
T 1551
T 0676
T 1748
T 1749
T 0800
T 0349
T 0534
T 1470
T 0405
T 0482
T 1782
T 0938
T 0233
T 1524
T 0480
T 0971
T 1185
T 1017
T 1016
T 0481
T 1102
T 1098
T 1101
T 1100
T 1099
T 0317
T 1434
T 0400
T 1437
T 0417
T 0158
T 0156
T 0157
T 0952
T 0671
T 1751
T 1929
T 1778
T 0271
T 0810
T 1419
T 1579
T 1580
T 1575
T 1576
T 1354
T 1889
T 1431
T 1062
T 1516
T 0211
T 0210
T 0212
T 0957
T 1236
T 1217
T 0197
T 2034
T 1378
T 1286
T 1948
T 0486
T 1685
T 0118
T 0410
T 1909
T 1126
T 1299
T 0490
T 0583
T 0545
T 0995
T 1702
T 0921
T 0232
T 1178
T 1134
T 0632
T 1186
T 1900
T 0699
T 0633
T 1376
T 2030
T 2037
T 1843
T 0954
T 1413
T 1874
T 1096
T 1499
T 0725
T 0785
T 1844
T 0127
T 0570
T 1522
T 1528
T 0135
T 1768
T 1581
T 1509
T 2015
T 1001
T 0759
T 1046
T 1484
T 1374
T 1314
T 0319
T 0100
T 0379
T 0426
T 0378
T 0963
T 0279
T 0443
T 0769
T 0449
T 1912
T 1054
T 1855
T 1349
T 1012
T 1714
T 1788
T 0902
T 1895
T 1008
T 1887
T 1031
T 0402
T 1106
T 1104
T 0479
T 0325
T 1105
T 2020
T 1002
T 1108
T 1109
T 1625
T 1107
T 0706
T 0455
T 0477
T 0877
T 0876
T 1677
T 0424
T 1704
T 0845
T 1329
T 1005
T 0764
T 0407
T 1920

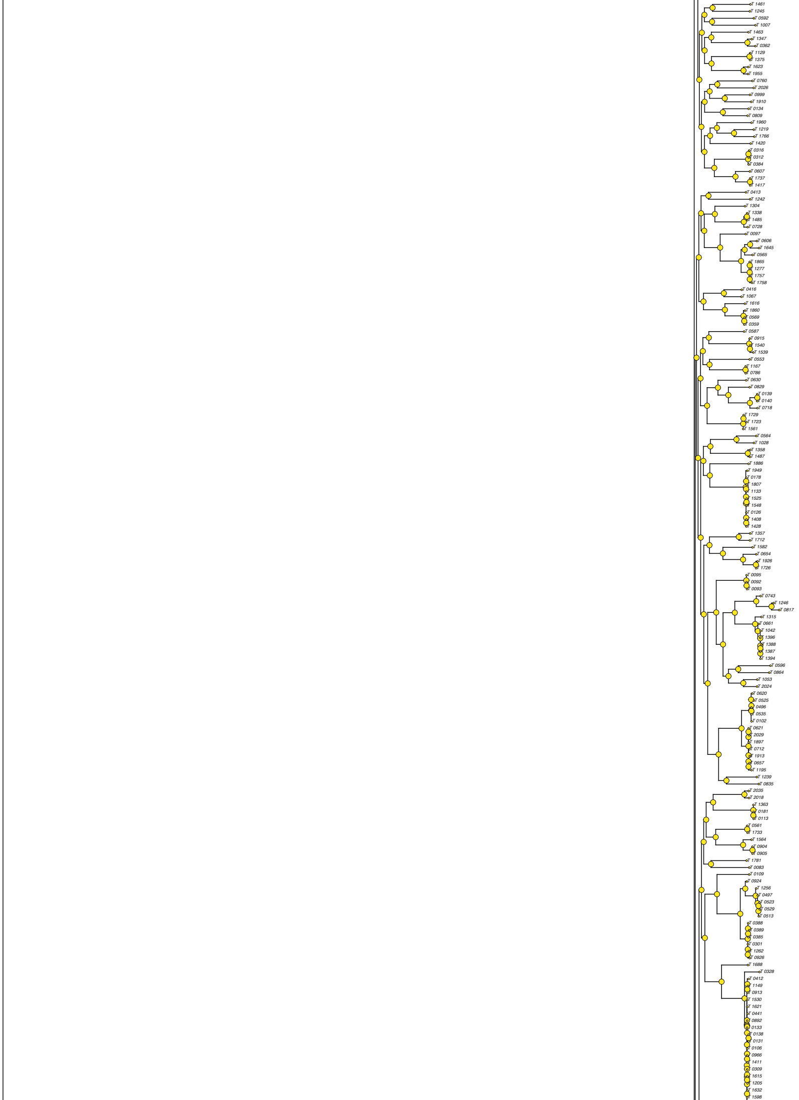
T 1461
T 1245
T 0592
T 1007
T 1463
T 1347
T 0362
T 1129
T 1375
T 1623
T 1955
T 0760
T 2026
T 0999
T 1910
T 0134
T 0809
T 1960
T 1219
T 1766
T 1420
T 0316
T 0312
T 0384
T 0607
T 1737
T 1417
T 0413
T 1242
T 1304
T 1338
T 1485
T 0728
T 0097
T 0606
T 1645
T 0565
T 1865
T 1277
T 1757
T 1758
T 0416
T 1067
T 1616
T 1860
T 0569
T 0359
T 0587
T 0915
T 1540
T 1539
T 0553
T 1167
T 0786
T 0630
T 0829
T 0139
T 0140
T 0718
T 1729
T 1723
T 1561
T 0564
T 1028
T 1358
T 1487
T 1886
T 1949
T 0178
T 1807
T 1133
T 1525
T 1548
T 0126
T 1408
T 1428
T 1357
T 1712
T 1582
T 0654
T 1926
T 1726
T 0095
T 0092
T 0093
T 0743
T 1246
T 0817
T 1315
T 0661
T 1042
T 1396
T 1388
T 1387
T 1394
T 0596
T 0864
T 1053
T 2024
T 0620
T 0525
T 0496
T 0535
T 0102
T 0621
T 2029
T 1897
T 0712
T 1913
T 0657
T 1195
T 1239
T 0835
T 2035
T 2018
T 1363
T 0181
T 0113
T 0561
T 1733
T 1564
T 0904
T 0905
T 1781
T 0083
T 0109
T 0924
T 1256
T 0497
T 0523
T 0529
T 0513
T 0388
T 0389
T 0385
T 0301
T 1262
T 0926
T 1688
T 0328
T 0412
T 1149
T 0913
T 1530
T 1621
T 0441
T 0892
T 0133
T 0138
T 0131
T 0106
T 0966
T 1411
T 0309
T 1615
T 1205
T 1632
T 1598

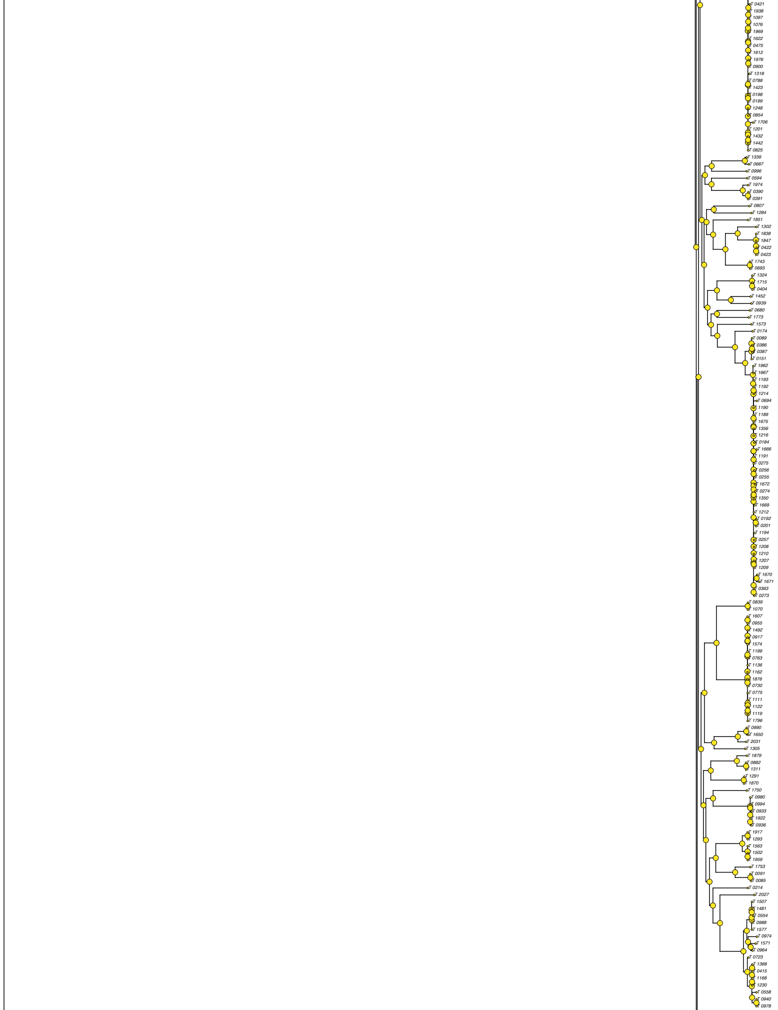
T 0421
T 1938
T 1097
T 1076
T 1969
T 1622
T 0475
T 1612
T 1976
T 0900
T 1318
T 0788
T 1423
T 0198
T 0199
T 1248
T 0854
T 1706
T 1201
T 1432
T 1442
T 0825
T 1339
T 0687
T 0996
T 0594
T 1974
T 0390
T 0391
T 0807
T 1284
T 1851
T 1302
T 1838
T 1847
T 0422
T 0423
T 1743
T 0693
T 1324
T 1715
T 0404
T 1452
T 0939
T 0680
T 1773
T 1573
T 0174
T 0089
T 0386
T 0387
T 0151
T 1962
T 1667
T 1193
T 1192
T 1214
T 0694
T 1190
T 1189
T 1675
T 1356
T 1216
T 0184
T 1666
T 1191
T 0275
T 0256
T 0255
T 1672
T 0274
T 1350
T 1669
T 1212
T 0192
T 0201
T 1194
T 0257
T 1208
T 1210
T 1207
T 1209
T 1670
T 1671
T 0363
T 0273
T 0839
T 1070
T 1607
T 0955
T 1492
T 0917
T 1574
T 1199
T 0763
T 1136
T 1162
T 1876
T 0730
T 0775
T 1111
T 1122
T 1119
T 1796
T 0990
T 1650
T 2031
T 1305
T 1879
T 0882
T 1311
T 1291
T 1870
T 1750
T 0980
T 0994
T 0933
T 1922
T 0936
T 1917
T 1293
T 1563
T 1502
T 1959
T 1753
T 0091
T 0085
T 0214
T 2027
T 1507
T 1481
T 0554
T 0988
T 1577
T 0974
T 1571
T 0964
T 0723
T 1368
T 0415
T 1168
T 1230
T 0558
T 0940
T 0978

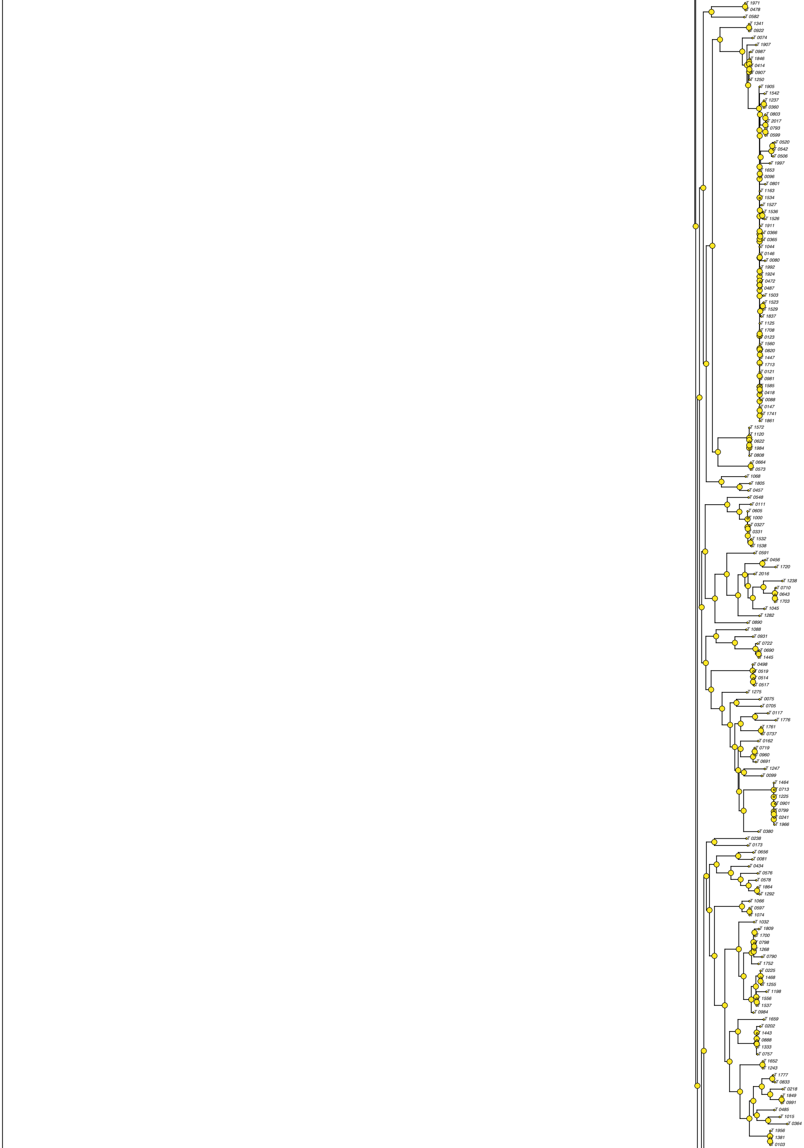
T 1971
T 0478
T 0582
T 1341
T 0922
T 0074
T 1907
T 0987
T 1846
T 0414
T 0907
T 1250
T 1905
T 1542
T 1237
T 0360
T 0803
T 2017
T 0793
T 0599
T 0520
T 0542
T 0506
T 1997
T 1653
T 0096
T 0801
T 1163
T 1534
T 1527
T 1536
T 1526
T 1911
T 0366
T 0365
T 1044
T 0146
T 0080
T 1992
T 1924
T 0472
T 0487
T 1503
T 1523
T 1529
T 1837
T 1125
T 1708
T 0123
T 1560
T 0820
T 1447
T 1713
T 0121
T 0981
T 1585
T 0418
T 0088
T 0147
T 1741
T 1861
T 1572
T 1120
T 0622
T 1984
T 0808
T 0664
T 0573
T 1068
T 1805
T 0457
T 0548
T 0111
T 0605
T 1000
T 0327
T 0331
T 1532
T 1538
T 0591
T 0456
T 1720
T 2016
T 1238
T 0710
T 0643
T 1703
T 1045
T 1282
T 0890
T 1088
T 0931
T 0722
T 0690
T 1445
T 0498
T 0519
T 0514
T 0517
T 1275
T 0075
T 0705
T 0117
T 1776
T 1761
T 0737
T 0162
T 0719
T 0960
T 0691
T 1247
T 0099
T 1464
T 0713
T 1225
T 0901
T 0799
T 0241
T 1966
T 0380
T 0238
T 0173
T 0656
T 0081
T 0434
T 0576
T 0578
T 1864
T 1292
T 1066
T 0597
T 1074
T 1032
T 1809
T 1700
T 0798
T 1268
T 0790
T 1752
T 0225
T 1468
T 1255
T 1198
T 1556
T 1537
T 0984
T 1659
T 0202
T 1443
T 0888
T 1333
T 0757
T 1652
T 1243
T 1777
T 0833
T 0218
T 1849
T 0991
T 0485
T 1015
T 0364
T 1956
T 1381
T 0103

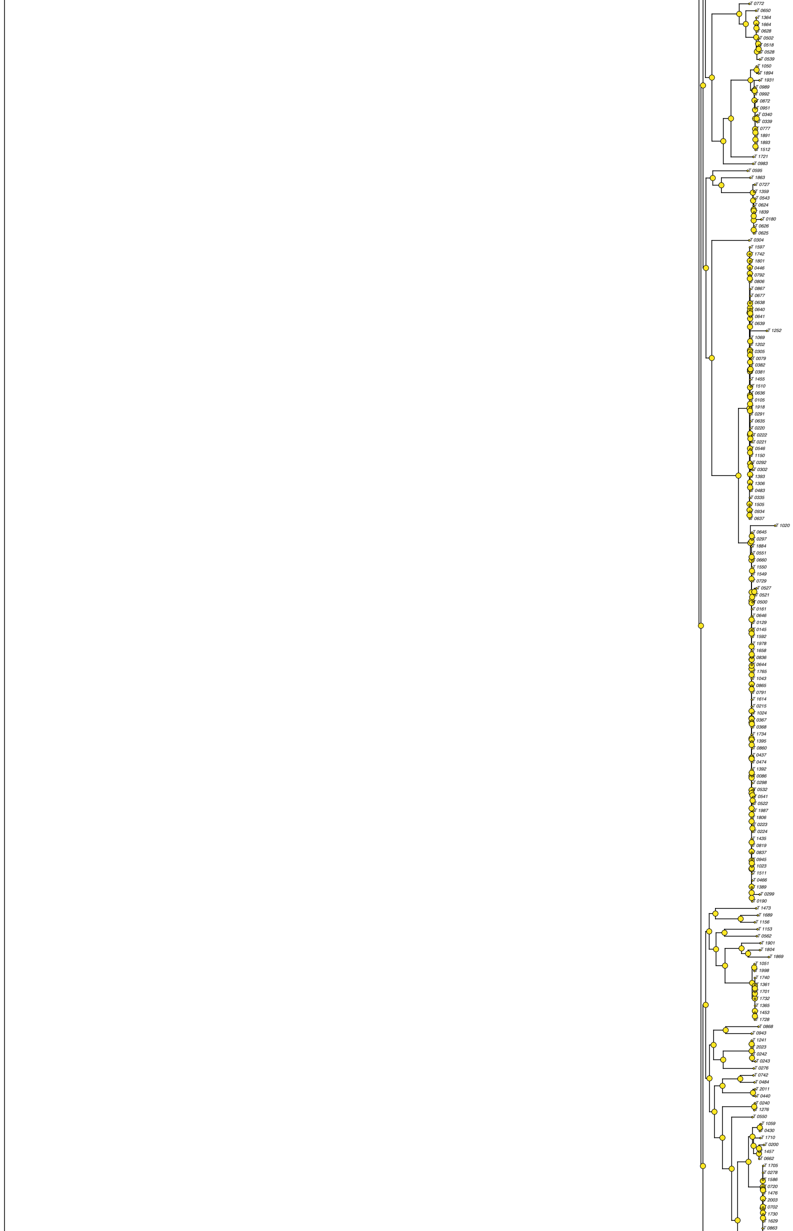
T 0772
T 0650
T 1364
T 1664
T 0628
T 0502
T 0518
T 0528
T 0539
T 1050
T 1894
T 1931
T 0989
T 0992
T 0872
T 0951
T 0340
T 0339
T 0777
T 1891
T 1893
T 1512
T 1721
T 0983
T 0595
T 1863
T 0727
T 1359
T 0543
T 0624
T 1839
T 0180
T 0626
T 0625
T 0304
T 1597
T 1742
T 1801
T 0446
T 0792
T 0806
T 0867
T 0677
T 0638
T 0640
T 0641
T 0639
T 1252
T 1069
T 1202
T 0305
T 0079
T 0382
T 0381
T 1455
T 1510
T 0636
T 0105
T 1918
T 0291
T 0635
T 0220
T 0222
T 0221
T 0546
T 1150
T 0292
T 0302
T 1393
T 1306
T 0483
T 0335
T 1505
T 0934
T 0637
T 1020
T 0645
T 0297
T 1884
T 0551
T 0660
T 1550
T 1549
T 0729
T 0527
T 0521
T 0500
T 0161
T 0646
T 0129
T 0145
T 1592
T 1978
T 1658
T 0836
T 0644
T 1765
T 1043
T 0865
T 0791
T 1614
T 0215
T 1024
T 0367
T 0368
T 1734
T 1395
T 0860
T 0437
T 0474
T 1392
T 0086
T 0298
T 0532
T 0541
T 0522
T 1987
T 1806
T 0223
T 0224
T 1435
T 0819
T 0837
T 0945
T 1023
T 1511
T 0466
T 1389
T 0299
T 0190
T 1473
T 1689
T 1156
T 1153
T 0562
T 1901
T 1804
T 1869
T 1051
T 1998
T 1740
T 1361
T 1701
T 1732
T 1365
T 1453
T 1728
T 0868
T 0943
T 1241
T 2023
T 0242
T 0243
T 0276
T 0742
T 0484
T 2011
T 0440
T 0240
T 1276
T 0550
T 1059
T 0430
T 1710
T 0200
T 1457
T 0662
T 1705
T 0278
T 1586
T 0720
T 1476
T 2003
T 0702
T 1730
T 1629
T 0863

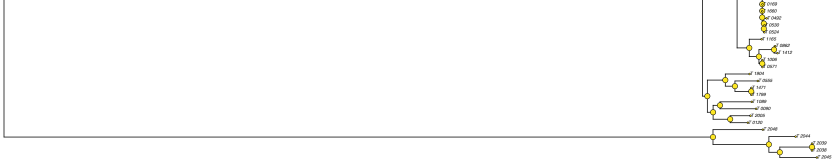
T 0169
T 1660
T 0492
T 0530
T 0524
T 1165
T 0862
T 1412
T 1006
T 0571
T 1904
T 0555
T 1471
T 1799
T 1089
T 0090
T 2005
T 0120
T 2048
T 2044
T 2039
T 2038
T 2045

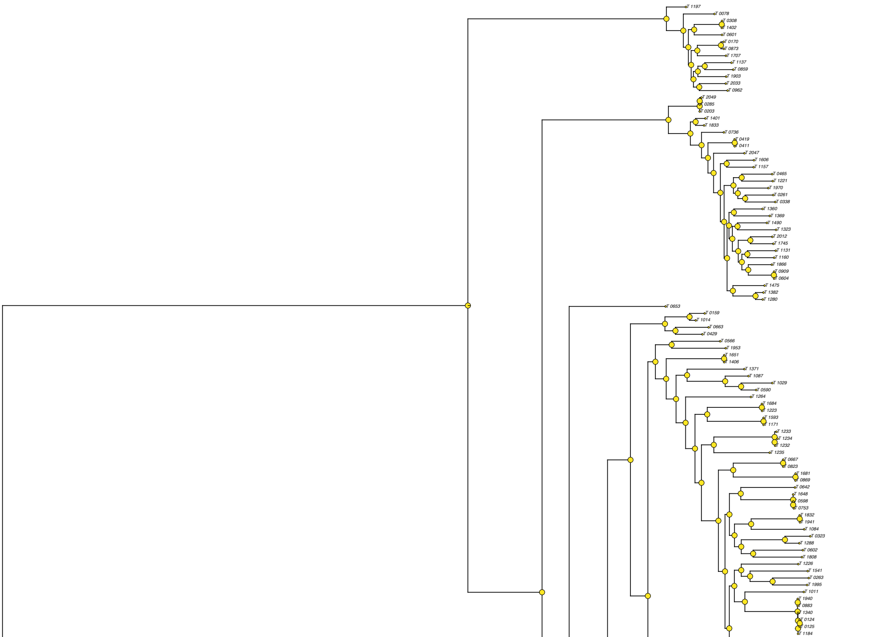

Effector gene: tse7d

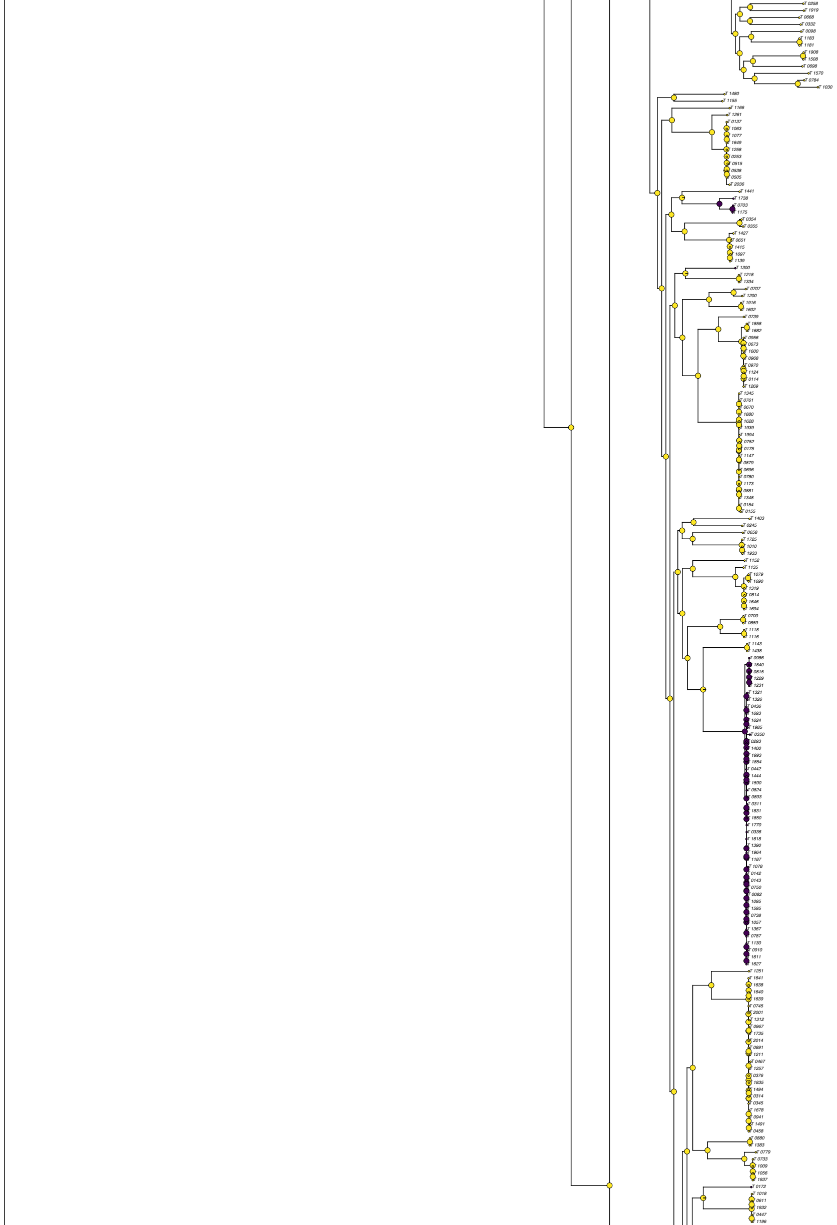
T 0258
T 1919
T 0668
T 0332
T 0098
T 1183
1181
T 1908
1508
T 0698
T 1570
T 0784
T 1030
T 1480
T 1155
T 1166
T 1261
T 0137
1063
1077
1649
1258
0253
0515
0538
0505
T 2036
T 1441
T 1738
T 0703
1175
T 0354
T 0355
T 1427
T 0651
1415
1697
1139
T 1300
T 1218
1334
T 0707
T 1200
T 1916
1602
T 0739
T 1858
1682
T 0956
0673
1600
0968
T 0970
1124
0114
T 1269
T 1345
T 0761
0670
1880
1628
1939
T 1994
0752
0175
T 1147
0879
0696
T 0780
1173
0881
1348
0154
T 0155
T 1403
T 0245
T 0658
T 1725
1010
1933
T 1152
T 1135
T 1079
1690
1319
0814
1646
1694
T 0700
0659
T 1118
1116
T 1143
1438
T 0986
1840
0815
1229
1231
T 1321
1326
T 0436
1693
1624
1985
T 0350
0293
1400
1993
1854
T 0442
1444
1590
T 0824
0893
T 0311
1831
1850
T 1770
T 0336
T 1618
T 1390
1964
1187
T 1078
0142
0143
0750
0082
1095
1595
0738
1057
T 1367
0787
T 1130
T 0910
1611
1627
T 1251
T 1641
1638
1640
1639
T 0745
2001
T 1312
T 0967
1735
2014
T 0891
1211
T 0467
1257
0376
1835
1494
0314
0345
T 1678
0941
T 1491
0458
T 0880
1383
T 0779
T 0733
1009
1056
1937
T 0172
T 1018
0611
1932
T 0447
1196

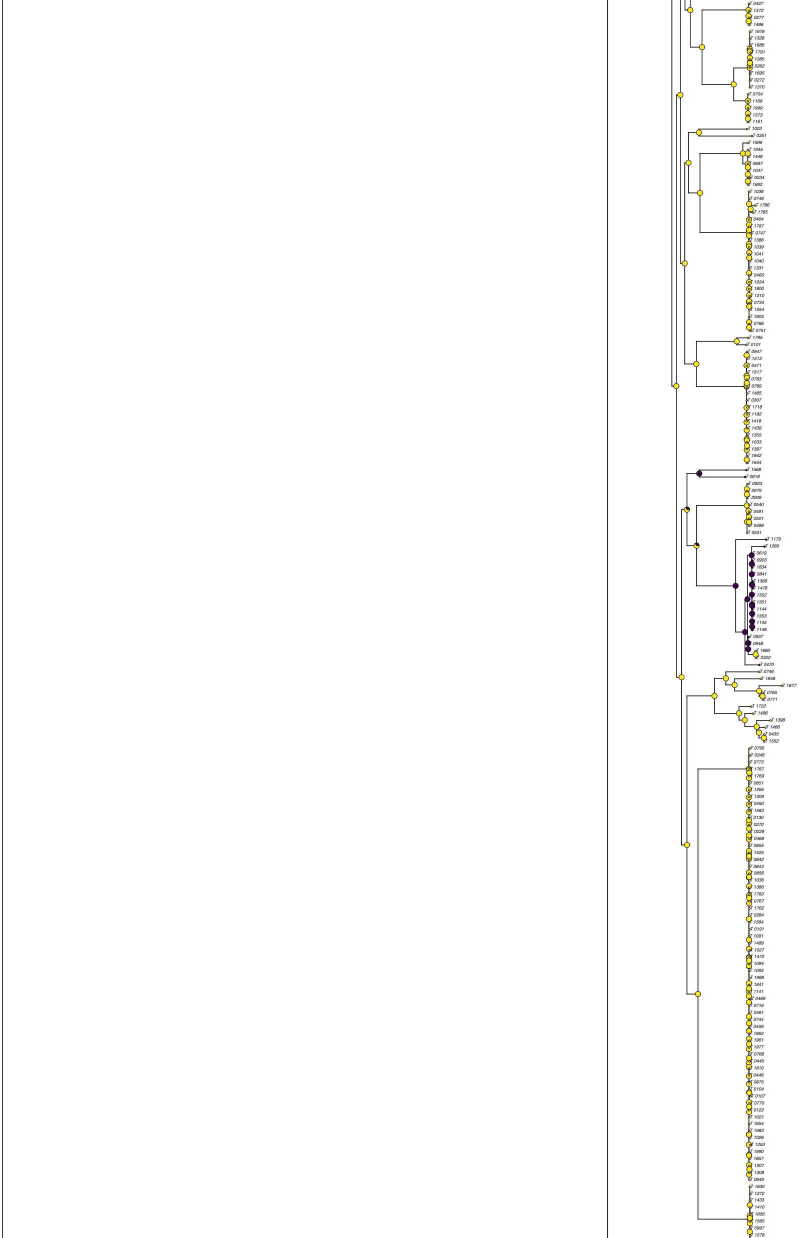
T 0427
T 1372
T 0277
T 1486
T 1676
T 1328
T 1686
T 1791
T 1385
T 0262
T 1695
T 0272
T 1370
T 0754
T 1169
T 1999
T 1373
T 1161
T 1003
T 0351
T 1596
T 1945
T 1448
T 0697
T 1047
T 0234
T 1692
T 1038
T 0748
T 1786
T 1785
T 0464
T 1787
T 0747
T 1386
T 1039
T 1041
T 1040
T 1331
T 0460
T 1934
T 1800
T 1310
T 0734
T 1254
T 1803
T 0766
T 0751
T 1795
T 0101
T 0947
T 1513
T 0471
T 1517
T 0783
T 0789
T 1465
T 0307
T 1718
T 1182
T 1418
T 1439
T 1355
T 1033
T 1397
T 1642
T 1644
T 1568
T 0818
T 0923
T 0979
T 2009
T 0540
T 0491
T 0501
T 0499
T 0531
T 1179
T 1260
T 0619
T 0903
T 1834
T 0841
T 1366
T 1478
T 1352
T 1351
T 1144
T 1353
T 1145
T 1148
T 0937
T 0948
T 1680
T 0322
T 0470
T 0746
T 1848
T 1877
T 0765
T 0771
T 1722
T 1496
T 1398
T 1466
T 0435
T 1552
T 0795
T 0346
T 0773
T 1767
T 1769
T 0851
T 1265
T 1309
T 0450
T 1583
T 0130
T 0270
T 0229
T 0468
T 0855
T 1425
T 0842
T 0843
T 0856
T 1036
T 1380
T 1763
T 0767
T 1762
T 0284
T 1584
T 0191
T 1091
T 1489
T 1027
T 1472
T 1094
T 1093
T 1989
T 1841
T 1141
T 0469
T 0716
T 0461
T 0744
T 0459
T 1963
T 1961
T 1977
T 0768
T 0445
T 1610
T 0448
T 0875
T 0104
T 0107
T 0770
T 0122
T 1021
T 1654
T 1983
T 1026
T 1253
T 1990
T 1857
T 1307
T 1308
T 0949
T 1630
T 1272
T 1433
T 1410
T 1856
T 1565
T 0997
T 1578

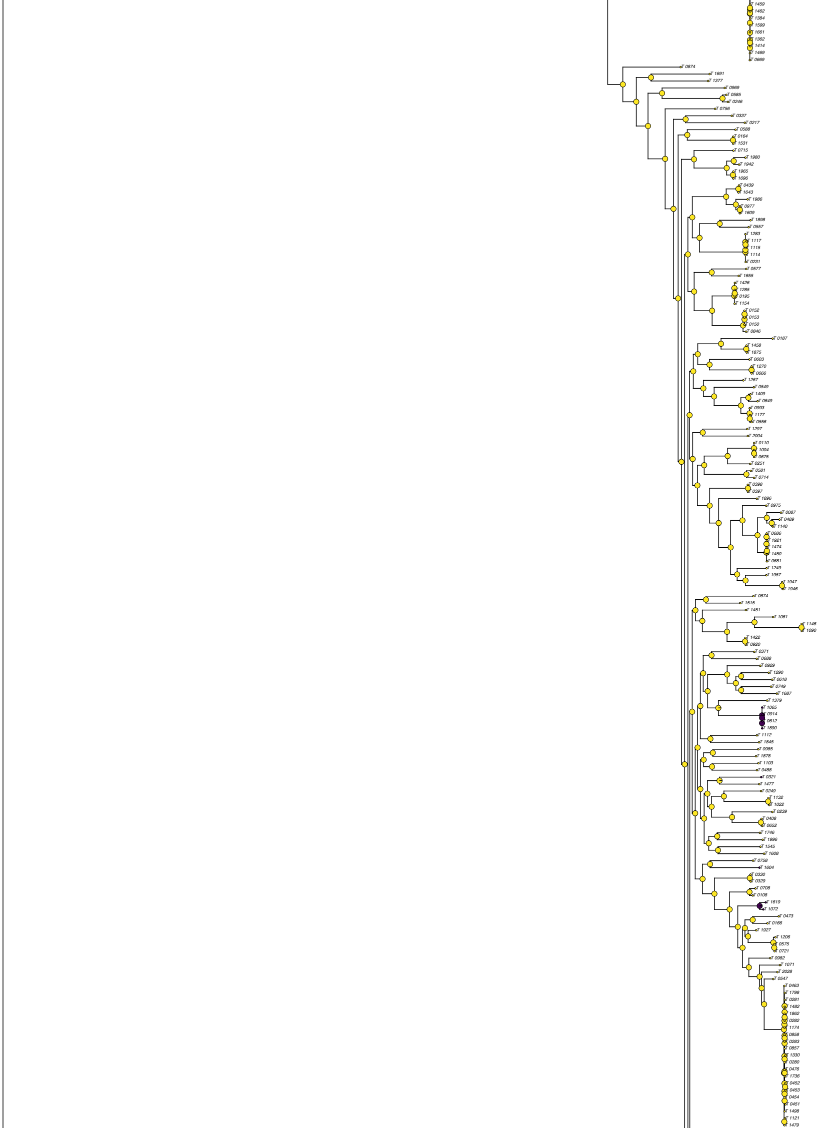

T 1459
T 1462
T 1384
T 1599
T 1661
T 1362
T 1414
T 1469
T 0669
T 0874
T 1691
T 1377
T 0969
T 0585
T 0246
T 0756
T 0337
T 0217
T 0588
T 0164
T 1531
T 0715
T 1980
T 1942
T 1965
T 1696
T 0439
T 1643
T 1986
T 0977
T 1609
T 1898
T 0557
T 1283
T 1117
T 1115
T 1114
T 0231
T 0577
T 1655
T 1426
T 1285
T 0195
T 1154
T 0152
T 0153
T 0150
T 0846
T 0187
T 1458
T 1875
T 0603
T 1270
T 0666
T 1267
T 0549
T 1409
T 0649
T 0993
T 1177
T 0556
T 1297
T 2004
T 0110
T 1004
T 0675
T 0251
T 0581
T 0714
T 0398
T 0397
T 1896
T 0975
T 0087
T 0489
T 1140
T 0686
T 1921
T 1474
T 1450
T 0681
T 1249
T 1957
T 1947
T 1946
T 0674
T 1515
T 1451
T 1061
T 1146
T 1090
T 1422
T 0920
T 0371
T 0688
T 0929
T 1290
T 0618
T 0749
T 1687
T 1379
T 1065
T 0914
T 0612
T 1890
T 1112
T 1845
T 0985
T 1878
T 1103
T 0488
T 0321
T 1477
T 0249
T 1132
T 1022
T 0239
T 0408
T 0652
T 1746
T 1996
T 1545
T 1608
T 0758
T 1604
T 0330
T 0329
T 0708
T 0108
T 1619
T 1072
T 0473
T 0166
T 1927
T 1206
T 0575
T 0721
T 0982
T 1071
T 2028
T 0547
T 0463
T 1798
T 0281
T 1482
T 1862
T 0282
T 1174
T 0858
T 0283
T 0857
T 1330
T 0280
T 0476
T 1736
T 0452
T 0453
T 0454
T 0451
T 1498
T 1121
T 1479

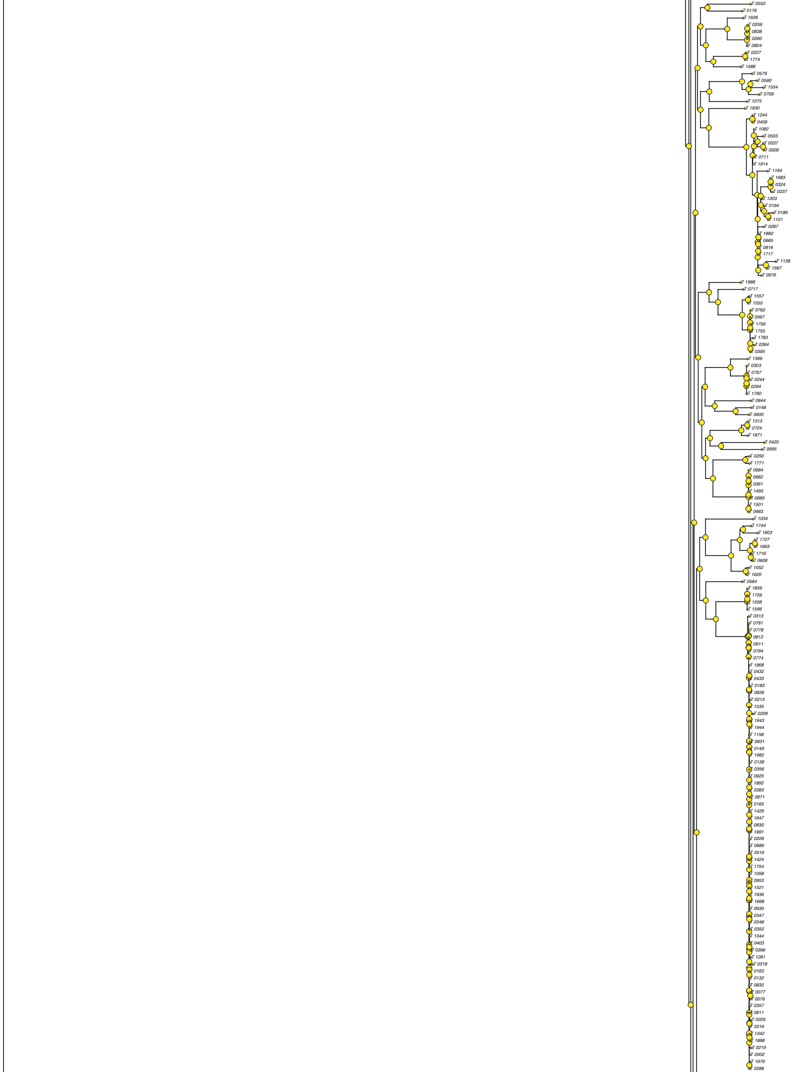
T 0552
T 0176
T 1626
T 0259
T 0838
T 0260
T 0804
T 0227
T 1774
T 1588
T 0579
T 0580
T 1554
T 0709
T 1075
T 1930
T 1244
T 0409
T 1082
T 0503
T 0537
T 0509
T 0711
T 1914
T 1164
T 1683
T 0324
T 0237
T 1203
T 0194
T 0186
T 1151
T 0287
T 1882
T 0665
T 0816
T 1717
T 1138
T 1567
T 0976
T 1988
T 0717
T 1557
T 1555
T 0762
T 0567
T 1756
T 1755
T 1783
T 0394
T 0395
T 1399
T 0303
T 0797
T 0244
T 0294
T 1760
T 0844
T 0148
T 0600
T 1313
T 0724
T 1871
T 0425
T 0695
T 0250
T 1771
T 0684
T 0682
T 0361
T 1493
T 0685
T 1501
T 0683
T 1034
T 1744
T 1603
T 1727
T 1663
T 1716
T 0608
T 1052
T 1620
T 0584
T 1859
T 1759
T 1558
T 1566
T 0313
T 0781
T 0778
T 0812
T 0911
T 0794
T 0774
T 1868
T 0432
T 0433
T 0183
T 0828
T 0213
T 1535
T 0209
T 1943
T 1944
T 1158
T 0831
T 0149
T 1982
T 0136
T 0356
T 0925
T 1892
T 0383
T 0871
T 0165
T 1429
T 1647
T 0830
T 1991
T 0226
T 0689
T 0516
T 1424
T 1754
T 1058
T 0953
T 1521
T 1936
T 1698
T 0930
T 0347
T 0348
T 0352
T 1344
T 0403
T 0396
T 1281
T 0318
T 0163
T 0132
T 0832
T 0077
T 0076
T 0357
T 0811
T 0205
T 0216
T 1342
T 1888
T 0219
T 2002
T 1679
T 0288

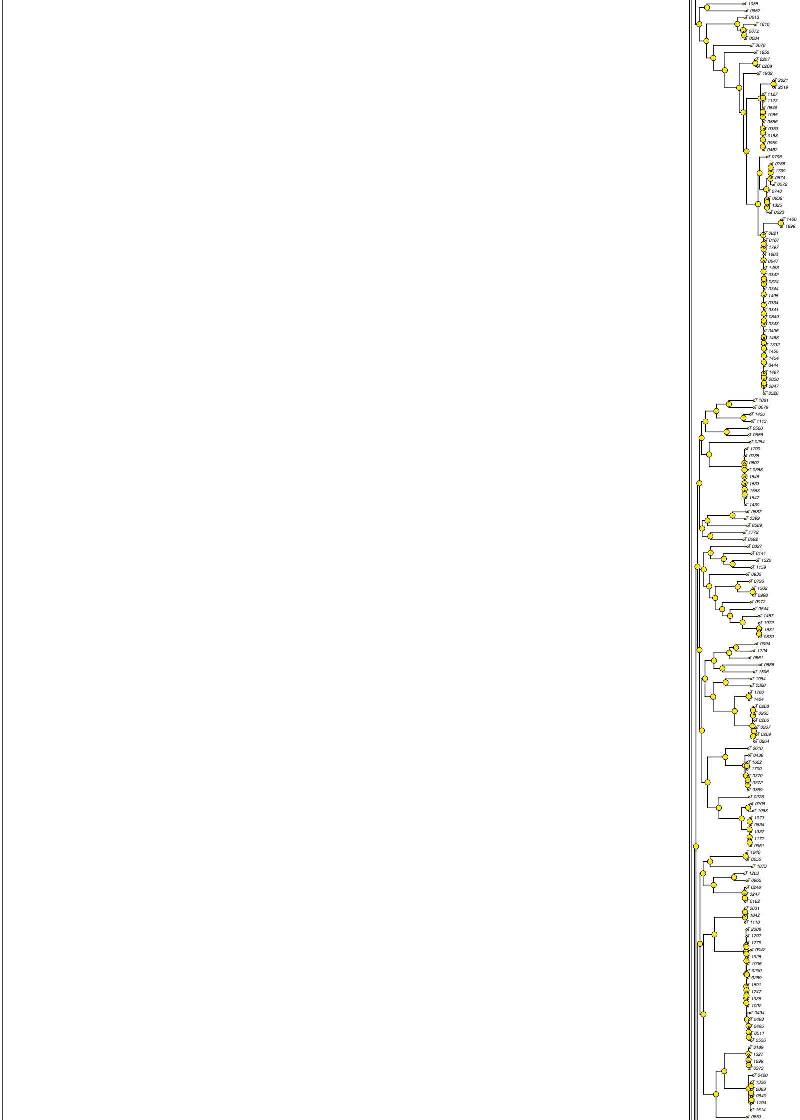
T 1055
T 0852
T 0613
T 1810
T 0672
T 0084
T 0678
T 1952
T 0207
T 0208
T 1902
T 2021
T 2019
T 1127
T 1123
T 0648
T 1085
T 0866
T 0353
T 0188
T 0950
T 0462
T 0796
T 0286
T 1739
T 0574
T 0572
T 0740
T 0932
T 1325
T 0623
T 1460
T 1899
T 0821
T 0167
T 1797
T 1883
T 0647
T 1483
T 0342
T 0374
T 0344
T 1495
T 0334
T 0341
T 0849
T 0343
T 0406
T 1488
T 1332
T 1456
T 1454
T 0444
T 1497
T 0850
T 0847
T 0326
T 1881
T 0679
T 1436
T 1113
T 0560
T 0586
T 0254
T 1790
T 0235
T 0802
T 0358
T 1546
T 1533
T 1553
T 1547
T 1430
T 0887
T 0399
T 0589
T 1772
T 0692
T 0827
T 0141
T 1320
T 1159
T 0935
T 0726
T 1562
T 0998
T 0972
T 0544
T 1467
T 1972
T 1631
T 0870
T 0094
T 1224
T 0861
T 0886
T 1506
T 1954
T 0320
T 1780
T 1404
T 0268
T 0265
T 0266
T 0267
T 0269
T 0264
T 0610
T 0438
T 1662
T 1709
T 0370
T 0372
T 0369
T 0228
T 0206
T 1968
T 1073
T 0834
T 1337
T 1172
T 0961
T 1240
T 0655
T 1873
T 1263
T 0965
T 0248
T 0247
T 0182
T 0631
T 1842
T 1110
T 2008
T 1792
T 1779
T 0942
T 1925
T 1906
T 0290
T 0289
T 1591
T 1747
T 1935
T 1092
T 0494
T 0493
T 0495
T 0511
T 0536
T 0189
T 1327
T 1699
T 0373
T 0420
T 1336
T 0889
T 0840
T 1794
T 1514
T 0853

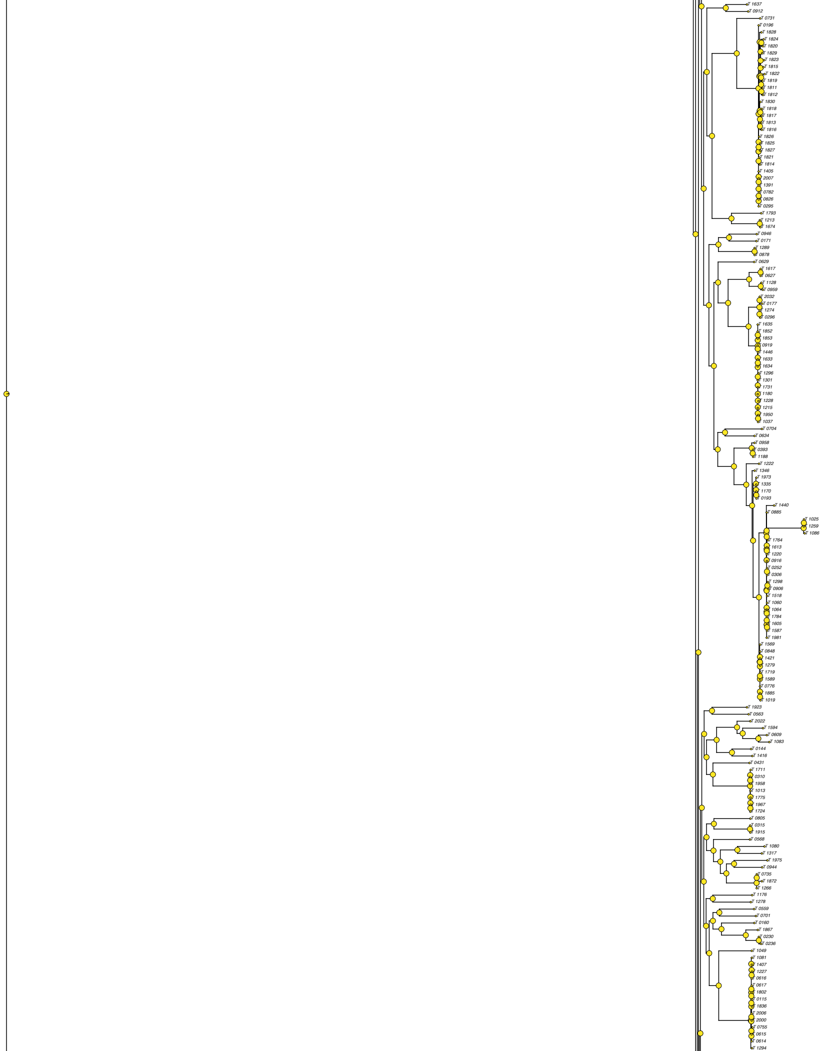
T 1637
T 0912
T 0731
T 0196
T 1828
T 1824
T 1820
T 1829
T 1823
T 1815
T 1822
T 1819
T 1811
T 1812
T 1830
T 1818
T 1817
T 1813
T 1816
T 1826
T 1825
T 1827
T 1821
T 1814
T 1405
T 2007
T 1391
T 0782
T 0826
T 0295
T 1793
T 1213
T 1674
T 0946
T 0171
T 1289
T 0878
T 0629
T 1617
T 0627
T 1128
T 0959
T 2032
T 0177
T 1274
T 0296
T 1635
T 1852
T 1853
T 0919
T 1446
T 1633
T 1634
T 1296
T 1301
T 1731
T 1180
T 1228
T 1215
T 1950
T 1037
T 0704
T 0634
T 0958
T 0393
T 1188
T 1222
T 1346
T 1973
T 1335
T 1170
T 0193
T 1440
T 0885
T 1025
T 1259
T 1086
T 1764
T 1613
T 1220
T 0916
T 0252
T 0306
T 1298
T 0906
T 1518
T 1060
T 1064
T 1784
T 1605
T 1587
T 1981
T 1569
T 0848
T 1421
T 1279
T 1719
T 1589
T 0776
T 1885
T 1019
T 1923
T 0563
T 2022
T 1594
T 0609
T 1083
T 0144
T 1416
T 0431
T 1711
T 0310
T 1958
T 1013
T 1775
T 1967
T 1724
T 0805
T 0315
T 1915
T 0568
T 1080
T 1317
T 1975
T 0944
T 0735
T 1872
T 1266
T 1176
T 1278
T 0559
T 0701
T 0160
T 1867
T 0230
T 0236
T 1049
T 1081
T 1407
T 1227
T 0616
T 0617
T 1802
T 0115
T 1836
T 2006
T 2000
T 0755
T 0615
T 0614
T 1294

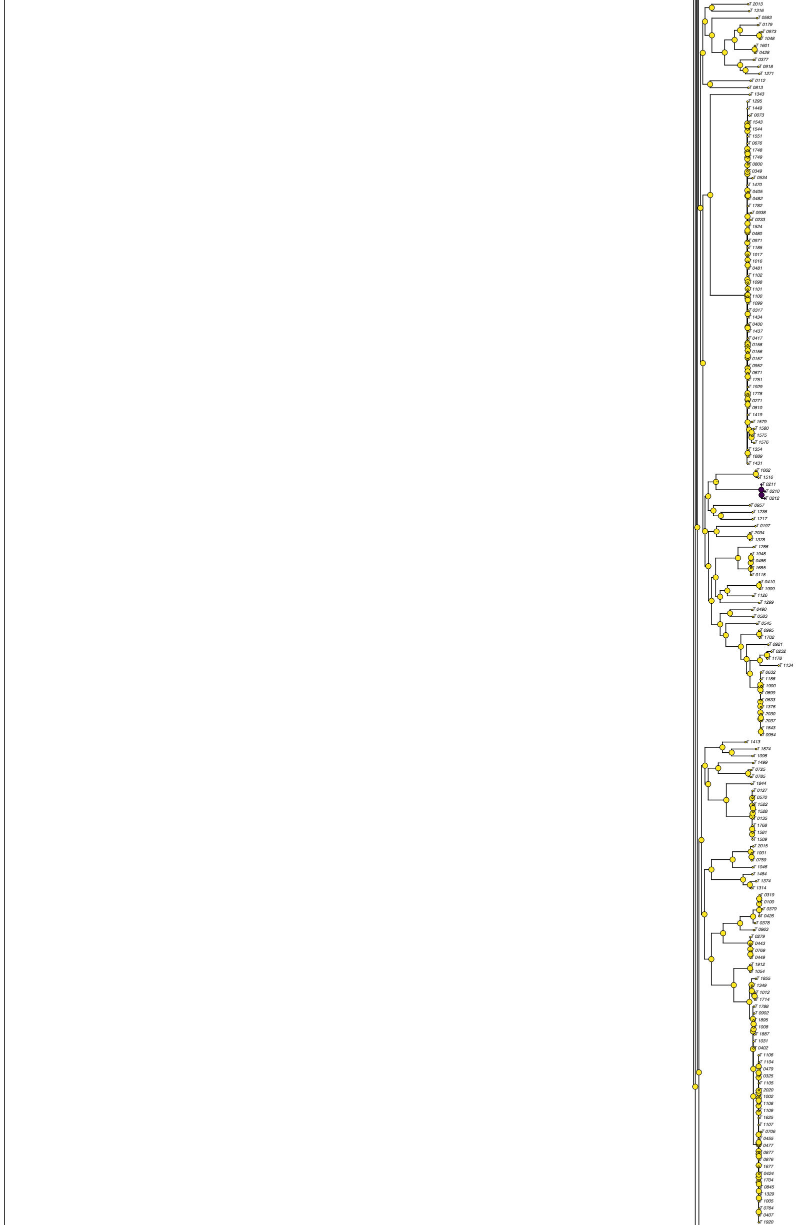
T 2013
T 1316
T 0593
T 0179
T 0973
T 1048
T 1601
T 0428
T 0377
T 0918
T 1271
T 0112
T 0813
T 1343
T 1295
T 1449
T 0073
T 1543
T 1544
T 1551
T 0676
T 1748
T 1749
T 0800
T 0349
T 0534
T 1470
T 0405
T 0482
T 1782
T 0938
T 0233
T 1524
T 0480
T 0971
T 1185
T 1017
T 1016
T 0481
T 1102
T 1098
T 1101
T 1100
T 1099
T 0317
T 1434
T 0400
T 1437
T 0417
T 0158
T 0156
T 0157
T 0952
T 0671
T 1751
T 1929
T 1778
T 0271
T 0810
T 1419
T 1579
T 1580
T 1575
T 1576
T 1354
T 1889
T 1431
T 1062
T 1516
T 0211
T 0210
T 0212
T 0957
T 1236
T 1217
T 0197
T 2034
T 1378
T 1286
T 1948
T 0486
T 1685
T 0118
T 0410
T 1909
T 1126
T 1299
T 0490
T 0583
T 0545
T 0995
T 1702
T 0921
T 0232
T 1178
T 1134
T 0632
T 1186
T 1900
T 0699
T 0633
T 1376
T 2030
T 2037
T 1843
T 0954
T 1413
T 1874
T 1096
T 1499
T 0725
T 0785
T 1844
T 0127
T 0570
T 1522
T 1528
T 0135
T 1768
T 1581
T 1509
T 2015
T 1001
T 0759
T 1046
T 1484
T 1374
T 1314
T 0319
T 0100
T 0379
T 0426
T 0378
T 0963
T 0279
T 0443
T 0769
T 0449
T 1912
T 1054
T 1855
T 1349
T 1012
T 1714
T 1788
T 0902
T 1895
T 1008
T 1887
T 1031
T 0402
T 1106
T 1104
T 0479
T 0325
T 1105
T 2020
T 1002
T 1108
T 1109
T 1625
T 1107
T 0706
T 0455
T 0477
T 0877
T 0876
T 1677
T 0424
T 1704
T 0845
T 1329
T 1005
T 0764
T 0407
T 1920

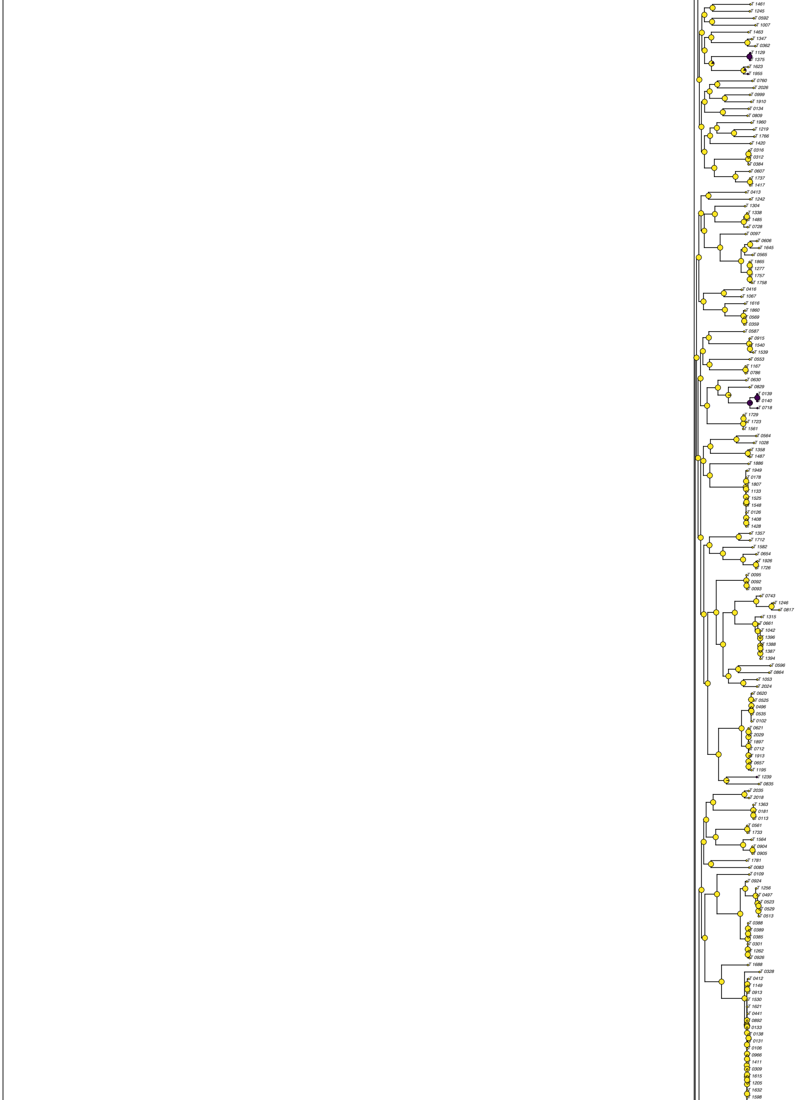
T 1461
T 1245
T 0592
T 1007
T 1463
T 1347
T 0362
T 1129
T 1375
T 1623
T 1955
T 0760
T 2026
T 0999
T 1910
T 0134
T 0809
T 1960
T 1219
T 1766
T 1420
T 0316
T 0312
T 0384
T 0607
T 1737
T 1417
T 0413
T 1242
T 1304
T 1338
T 1485
T 0728
T 0097
T 0606
T 1645
T 0565
T 1865
T 1277
T 1757
T 1758
T 0416
T 1067
T 1616
T 1860
T 0569
T 0359
T 0587
T 0915
T 1540
T 1539
T 0553
T 1167
T 0786
T 0630
T 0829
T 0139
T 0140
T 0718
T 1729
T 1723
T 1561
T 0564
T 1028
T 1358
T 1487
T 1886
T 1949
T 0178
T 1807
T 1133
T 1525
T 1548
T 0126
T 1408
T 1428
T 1357
T 1712
T 1582
T 0654
T 1926
T 1726
T 0095
T 0092
T 0093
T 0743
T 1246
T 0817
T 1315
T 0661
T 1042
T 1396
T 1388
T 1387
T 1394
T 0596
T 0864
T 1053
T 2024
T 0620
T 0525
T 0496
T 0535
T 0102
T 0621
T 2029
T 1897
T 0712
T 1913
T 0657
T 1195
T 1239
T 0835
T 2035
T 2018
T 1363
T 0181
T 0113
T 0561
T 1733
T 1564
T 0904
T 0905
T 1781
T 0083
T 0109
T 0924
T 1256
T 0497
T 0523
T 0529
T 0513
T 0388
T 0389
T 0385
T 0301
T 1262
T 0926
T 1688
T 0328
T 0412
T 1149
T 0913
T 1530
T 1621
T 0441
T 0892
T 0133
T 0138
T 0131
T 0106
T 0966
T 1411
T 0309
T 1615
T 1205
T 1632
T 1598

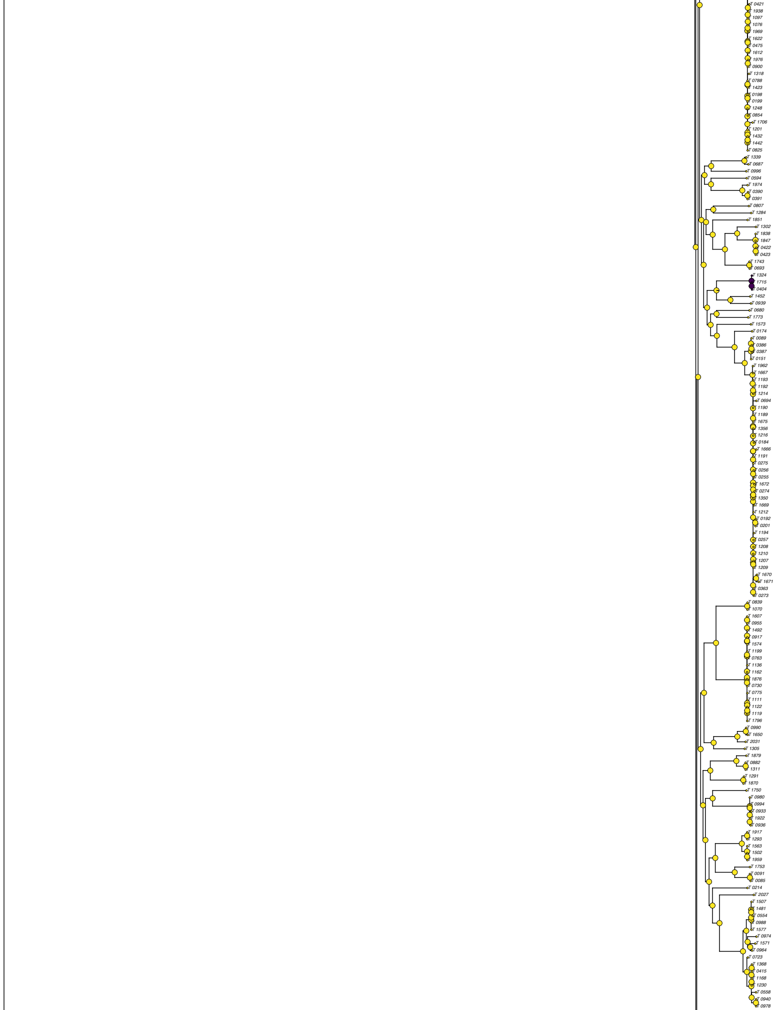
T 0421
T 1938
T 1097
T 1076
T 1969
T 1622
T 0475
T 1612
T 1976
T 0900
T 1318
T 0788
T 1423
T 0198
T 0199
T 1248
T 0854
T 1706
T 1201
T 1432
T 1442
T 0825
T 1339
T 0687
T 0996
T 0594
T 1974
T 0390
T 0391
T 0807
T 1284
T 1851
T 1302
T 1838
T 1847
T 0422
T 0423
T 1743
T 0693
T 1324
T 1715
T 0404
T 1452
T 0939
T 0680
T 1773
T 1573
T 0174
T 0089
T 0386
T 0387
T 0151
T 1962
T 1667
T 1193
T 1192
T 1214
T 0694
T 1190
T 1189
T 1675
T 1356
T 1216
T 0184
T 1666
T 1191
T 0275
T 0256
T 0255
T 1672
T 0274
T 1350
T 1669
T 1212
T 0192
T 0201
T 1194
T 0257
T 1208
T 1210
T 1207
T 1209
T 1670
T 1671
T 0363
T 0273
T 0839
T 1070
T 1607
T 0955
T 1492
T 0917
T 1574
T 1199
T 0763
T 1136
T 1162
T 1876
T 0730
T 0775
T 1111
T 1122
T 1119
T 1796
T 0990
T 1650
T 2031
T 1305
T 1879
T 0882
T 1311
T 1291
T 1870
T 1750
T 0980
T 0994
T 0933
T 1922
T 0936
T 1917
T 1293
T 1563
T 1502
T 1959
T 1753
T 0091
T 0085
T 0214
T 2027
T 1507
T 1481
T 0554
T 0988
T 1577
T 0974
T 1571
T 0964
T 0723
T 1368
T 0415
T 1168
T 1230
T 0558
T 0940
T 0978

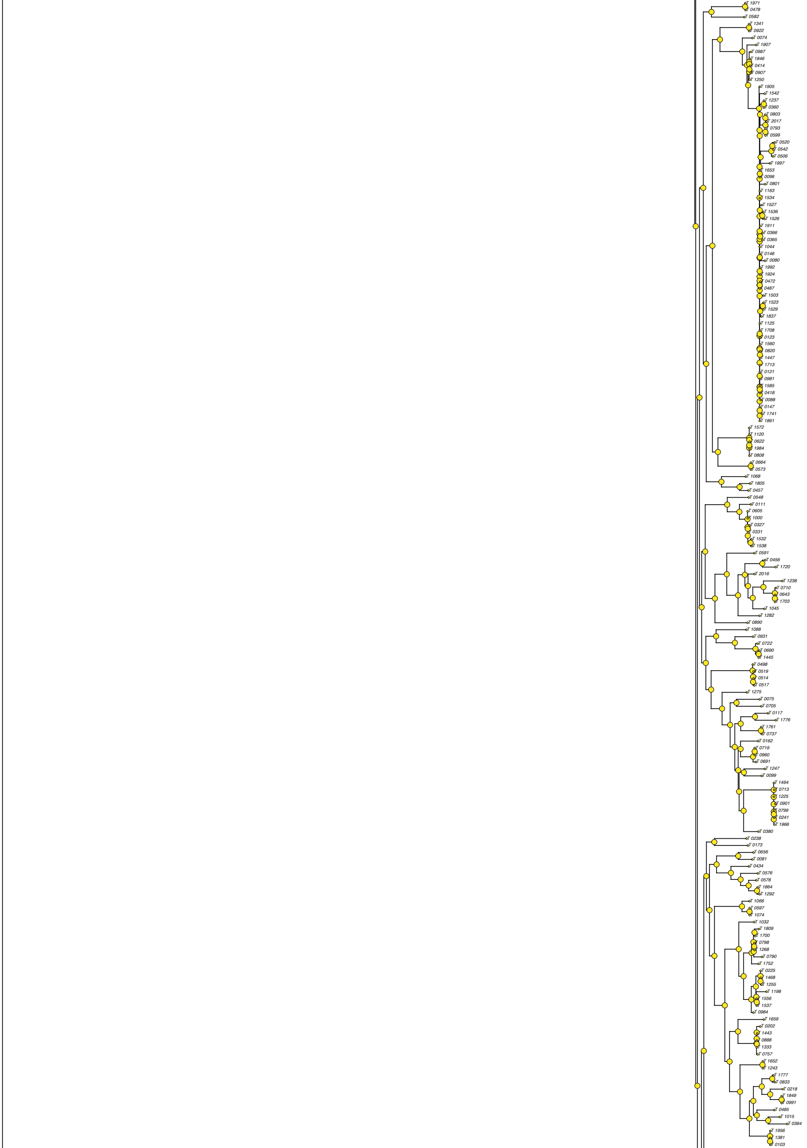
T 1971
T 0478
T 0582
T 1341
T 0922
T 0074
T 1907
T 0987
T 1846
T 0414
T 0907
T 1250
T 1905
T 1542
T 1237
T 0360
T 0803
T 2017
T 0793
T 0599
T 0520
T 0542
T 0506
T 1997
T 1653
T 0096
T 0801
T 1163
T 1534
T 1527
T 1536
T 1526
T 1911
T 0366
T 0365
T 1044
T 0146
T 0080
T 1992
T 1924
T 0472
T 0487
T 1503
T 1523
T 1529
T 1837
T 1125
T 1708
T 0123
T 1560
T 0820
T 1447
T 1713
T 0121
T 0981
T 1585
T 0418
T 0088
T 0147
T 1741
T 1861
T 1572
T 1120
T 0622
T 1984
T 0808
T 0664
T 0573
T 1068
T 1805
T 0457
T 0548
T 0111
T 0605
T 1000
T 0327
T 0331
T 1532
T 1538
T 0591
T 0456
T 1720
T 2016
T 1238
T 0710
T 0643
T 1703
T 1045
T 1282
T 0890
T 1088
T 0931
T 0722
T 0690
T 1445
T 0498
T 0519
T 0514
T 0517
T 1275
T 0075
T 0705
T 0117
T 1776
T 1761
T 0737
T 0162
T 0719
T 0960
T 0691
T 1247
T 0099
T 1464
T 0713
T 1225
T 0901
T 0799
T 0241
T 1966
T 0380
T 0238
T 0173
T 0656
T 0081
T 0434
T 0576
T 0578
T 1864
T 1292
T 1066
T 0597
T 1074
T 1032
T 1809
T 1700
T 0798
T 1268
T 0790
T 1752
T 0225
T 1468
T 1255
T 1198
T 1556
T 1537
T 0984
T 1659
T 0202
T 1443
T 0888
T 1333
T 0757
T 1652
T 1243
T 1777
T 0833
T 0218
T 1849
T 0991
T 0485
T 1015
T 0364
T 1956
T 1381
T 0103

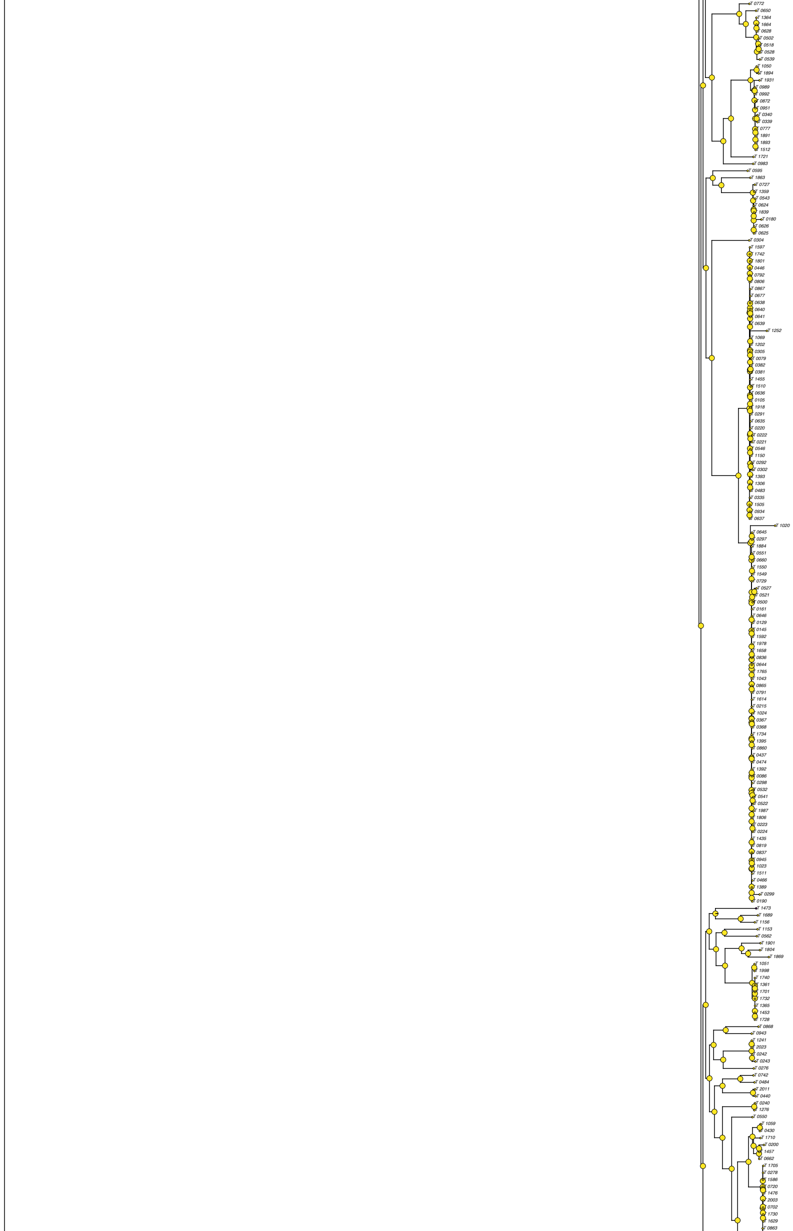
T 0772
T 0650
T 1364
T 1664
T 0628
T 0502
T 0518
T 0528
T 0539
T 1050
T 1894
T 1931
T 0989
T 0992
T 0872
T 0951
T 0340
T 0339
T 0777
T 1891
T 1893
T 1512
T 1721
T 0983
T 0595
T 1863
T 0727
T 1359
T 0543
T 0624
T 1839
T 0180
T 0626
T 0625
T 0304
T 1597
T 1742
T 1801
T 0446
T 0792
T 0806
T 0867
T 0677
T 0638
T 0640
T 0641
T 0639
T 1252
T 1069
T 1202
T 0305
T 0079
T 0382
T 0381
T 1455
T 1510
T 0636
T 0105
T 1918
T 0291
T 0635
T 0220
T 0222
T 0221
T 0546
T 1150
T 0292
T 0302
T 1393
T 1306
T 0483
T 0335
T 1505
T 0934
T 0637
T 1020
T 0645
T 0297
T 1884
T 0551
T 0660
T 1550
T 1549
T 0729
T 0527
T 0521
T 0500
T 0161
T 0646
T 0129
T 0145
T 1592
T 1978
T 1658
T 0836
T 0644
T 1765
T 1043
T 0865
T 0791
T 1614
T 0215
T 1024
T 0367
T 0368
T 1734
T 1395
T 0860
T 0437
T 0474
T 1392
T 0086
T 0298
T 0532
T 0541
T 0522
T 1987
T 1806
T 0223
T 0224
T 1435
T 0819
T 0837
T 0945
T 1023
T 1511
T 0466
T 1389
T 0299
T 0190
T 1473
T 1689
T 1156
T 1153
T 0562
T 1901
T 1804
T 1869
T 1051
T 1998
T 1740
T 1361
T 1701
T 1732
T 1365
T 1453
T 1728
T 0868
T 0943
T 1241
T 2023
T 0242
T 0243
T 0276
T 0742
T 0484
T 2011
T 0440
T 0240
T 1276
T 0550
T 1059
T 0430
T 1710
T 0200
T 1457
T 0662
T 1705
T 0278
T 1586
T 0720
T 1476
T 2003
T 0702
T 1730
T 1629
T 0863

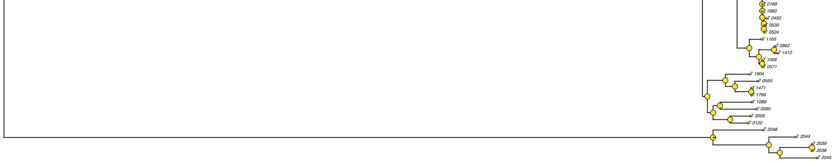

T 0169
T 1660
T 0492
T 0530
T 0524
T 1165
T 0862
T 1412
T 1006
T 0571
T 1904
T 0555
T 1471
T 1799
T 1089
T 0090
T 2005
T 0120
T 2048
T 2044
T 2039
T 2038
T 2045

tse7e
X

Effector gene: tse7e

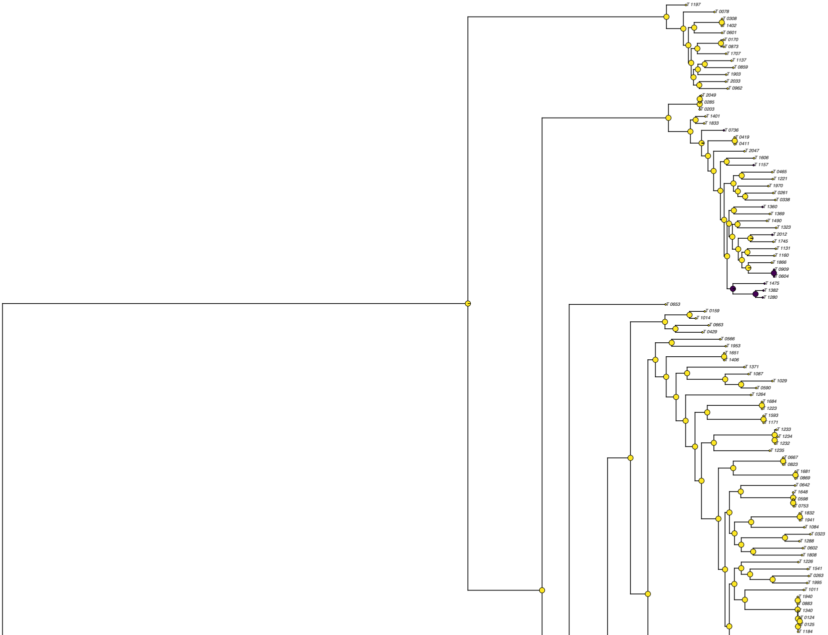

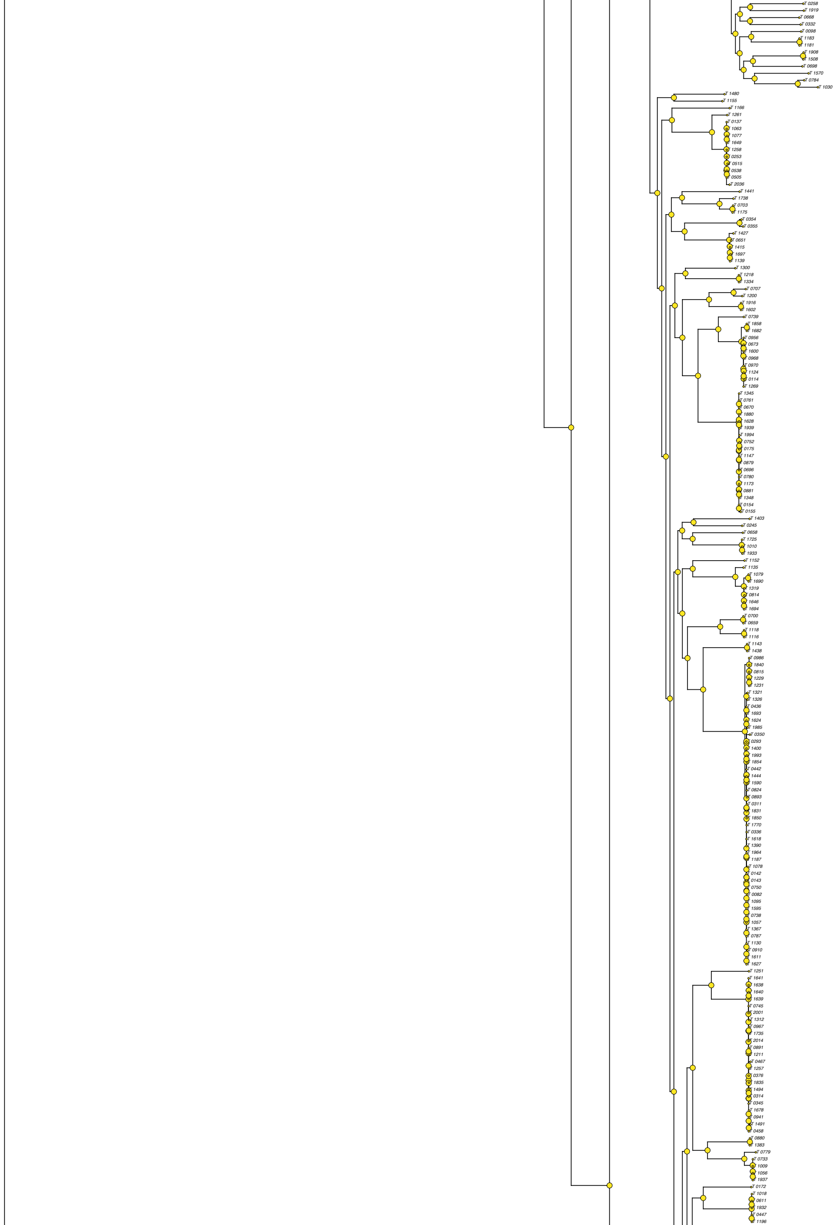
T 0258
T 1919
T 0668
T 0332
T 0098
T 1183
T 1181
T 1908
T 1508
T 0698
T 1570
T 0784
T 1030
T 1480
T 1155
T 1166
T 1261
T 0137
T 1063
T 1077
T 1649
T 1258
T 0253
T 0515
T 0538
T 0505
T 2036
T 1441
T 1738
T 0703
T 1175
T 0354
T 0355
T 1427
T 0651
T 1415
T 1697
T 1139
T 1300
T 1218
T 1334
T 0707
T 1200
T 1916
T 1602
T 0739
T 1858
T 1682
T 0956
T 0673
T 1600
T 0968
T 0970
T 1124
T 0114
T 1269
T 1345
T 0761
T 0670
T 1880
T 1628
T 1939
T 1994
T 0752
T 0175
T 1147
T 0879
T 0696
T 0780
T 1173
T 0881
T 1348
T 0154
T 0155
T 1403
T 0245
T 0658
T 1725
T 1010
T 1933
T 1152
T 1135
T 1079
T 1690
T 1319
T 0814
T 1646
T 1694
T 0700
T 0659
T 1118
T 1116
T 1143
T 1438
T 0986
T 1840
T 0815
T 1229
T 1231
T 1321
T 1326
T 0436
T 1693
T 1624
T 1985
T 0350
T 0293
T 1400
T 1993
T 1854
T 0442
T 1444
T 1590
T 0824
T 0893
T 0311
T 1831
T 1850
T 1770
T 0336
T 1618
T 1390
T 1964
T 1187
T 1078
T 0142
T 0143
T 0750
T 0082
T 1095
T 1595
T 0738
T 1057
T 1367
T 0787
T 1130
T 0910
T 1611
T 1627
T 1251
T 1641
T 1638
T 1640
T 1639
T 0745
T 2001
T 1312
T 0967
T 1735
T 2014
T 0891
T 1211
T 0467
T 1257
T 0376
T 1835
T 1494
T 0314
T 0345
T 1678
T 0941
T 1491
T 0458
T 0880
T 1383
T 0779
T 0733
T 1009
T 1056
T 1937
T 0172
T 1018
T 0611
T 1932
T 0447
T 1196

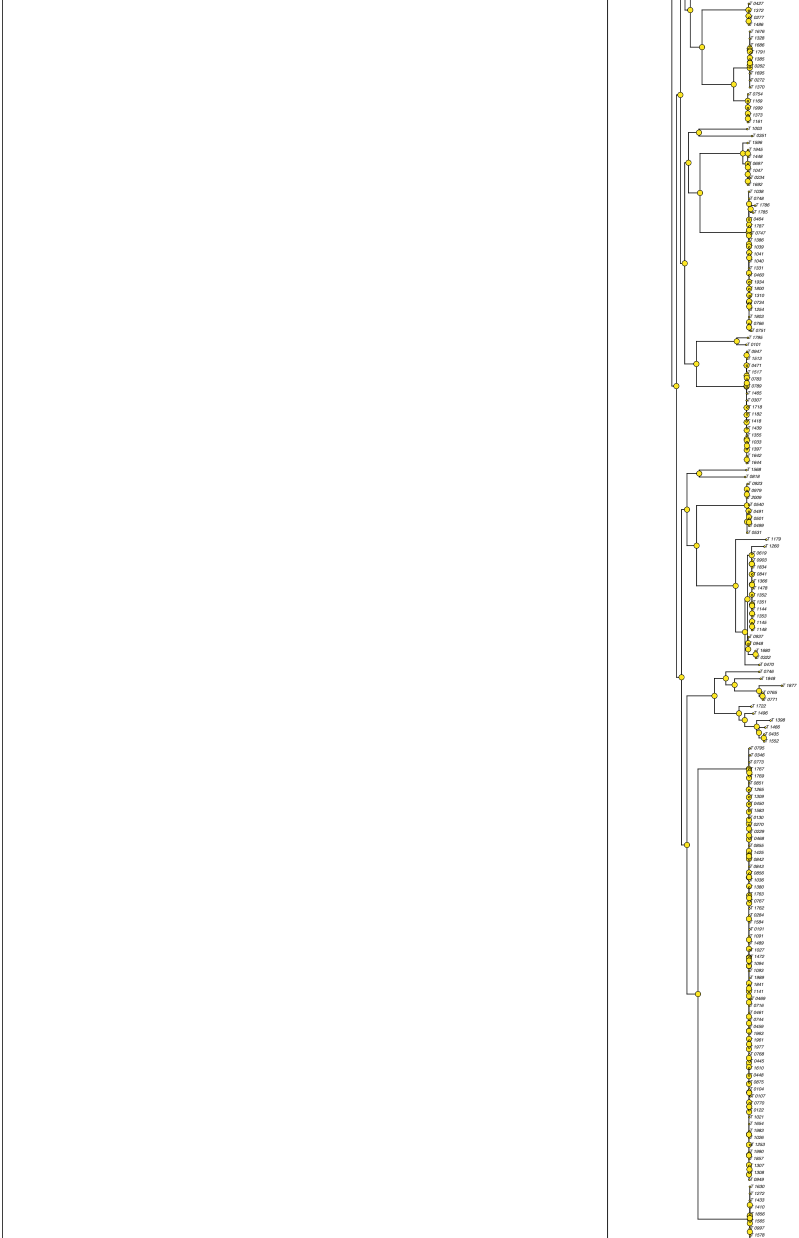
T 0427
T 1372
T 0277
T 1486
T 1676
T 1328
T 1686
T 1791
T 1385
T 0262
T 1695
T 0272
T 1370
T 0754
T 1169
T 1999
T 1373
T 1161
T 1003
T 0351
T 1596
T 1945
T 1448
T 0697
T 1047
T 0234
T 1692
T 1038
T 0748
T 1786
T 1785
T 0464
T 1787
T 0747
T 1386
T 1039
T 1041
T 1040
T 1331
T 0460
T 1934
T 1800
T 1310
T 0734
T 1254
T 1803
T 0766
T 0751
T 1795
T 0101
T 0947
T 1513
T 0471
T 1517
T 0783
T 0789
T 1465
T 0307
T 1718
T 1182
T 1418
T 1439
T 1355
T 1033
T 1397
T 1642
T 1644
T 1568
T 0818
T 0923
T 0979
T 2009
T 0540
T 0491
T 0501
T 0499
T 0531
T 1179
T 1260
T 0619
T 0903
T 1834
T 0841
T 1366
T 1478
T 1352
T 1351
T 1144
T 1353
T 1145
T 1148
T 0937
T 0948
T 1680
T 0322
T 0470
T 0746
T 1848
T 1877
T 0765
T 0771
T 1722
T 1496
T 1398
T 1466
T 0435
T 1552
T 0795
T 0346
T 0773
T 1767
T 1769
T 0851
T 1265
T 1309
T 0450
T 1583
T 0130
T 0270
T 0229
T 0468
T 0855
T 1425
T 0842
T 0843
T 0856
T 1036
T 1380
T 1763
T 0767
T 1762
T 0284
T 1584
T 0191
T 1091
T 1489
T 1027
T 1472
T 1094
T 1093
T 1989
T 1841
T 1141
T 0469
T 0716
T 0461
T 0744
T 0459
T 1963
T 1961
T 1977
T 0768
T 0445
T 1610
T 0448
T 0875
T 0104
T 0107
T 0770
T 0122
T 1021
T 1654
T 1983
T 1026
T 1253
T 1990
T 1857
T 1307
T 1308
T 0949
T 1630
T 1272
T 1433
T 1410
T 1856
T 1565
T 0997
T 1578

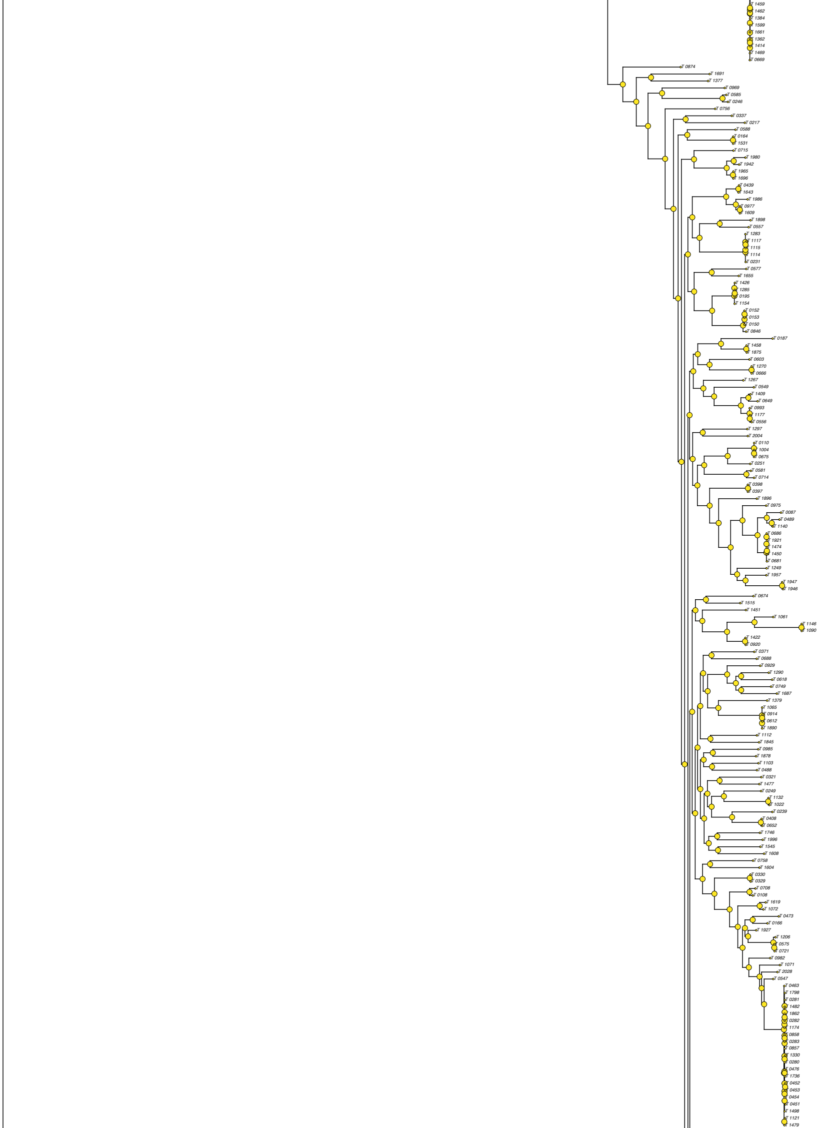
T 1459
T 1462
T 1384
T 1599
T 1661
T 1362
T 1414
T 1469
T 0669
T 0874
T 1691
T 1377
T 0969
T 0585
T 0246
T 0756
T 0337
T 0217
T 0588
T 0164
T 1531
T 0715
T 1980
T 1942
T 1965
T 1696
T 0439
T 1643
T 1986
T 0977
T 1609
T 1898
T 0557
T 1283
T 1117
T 1115
T 1114
T 0231
T 0577
T 1655
T 1426
T 1285
T 0195
T 1154
T 0152
T 0153
T 0150
T 0846
T 0187
T 1458
T 1875
T 0603
T 1270
T 0666
T 1267
T 0549
T 1409
T 0649
T 0993
T 1177
T 0556
T 1297
T 2004
T 0110
T 1004
T 0675
T 0251
T 0581
T 0714
T 0398
T 0397
T 1896
T 0975
T 0087
T 0489
T 1140
T 0686
T 1921
T 1474
T 1450
T 0681
T 1249
T 1957
T 1947
T 1946
T 0674
T 1515
T 1451
T 1061
T 1146
T 1090
T 1422
T 0920
T 0371
T 0688
T 0929
T 1290
T 0618
T 0749
T 1687
T 1379
T 1065
T 0914
T 0612
T 1890
T 1112
T 1845
T 0985
T 1878
T 1103
T 0488
T 0321
T 1477
T 0249
T 1132
T 1022
T 0239
T 0408
T 0652
T 1746
T 1996
T 1545
T 1608
T 0758
T 1604
T 0330
T 0329
T 0708
T 0108
T 1619
T 1072
T 0473
T 0166
T 1927
T 1206
T 0575
T 0721
T 0982
T 1071
T 2028
T 0547
T 0463
T 1798
T 0281
T 1482
T 1862
T 0282
T 1174
T 0858
T 0283
T 0857
T 1330
T 0280
T 0476
T 1736
T 0452
T 0453
T 0454
T 0451
T 1498
T 1121
T 1479

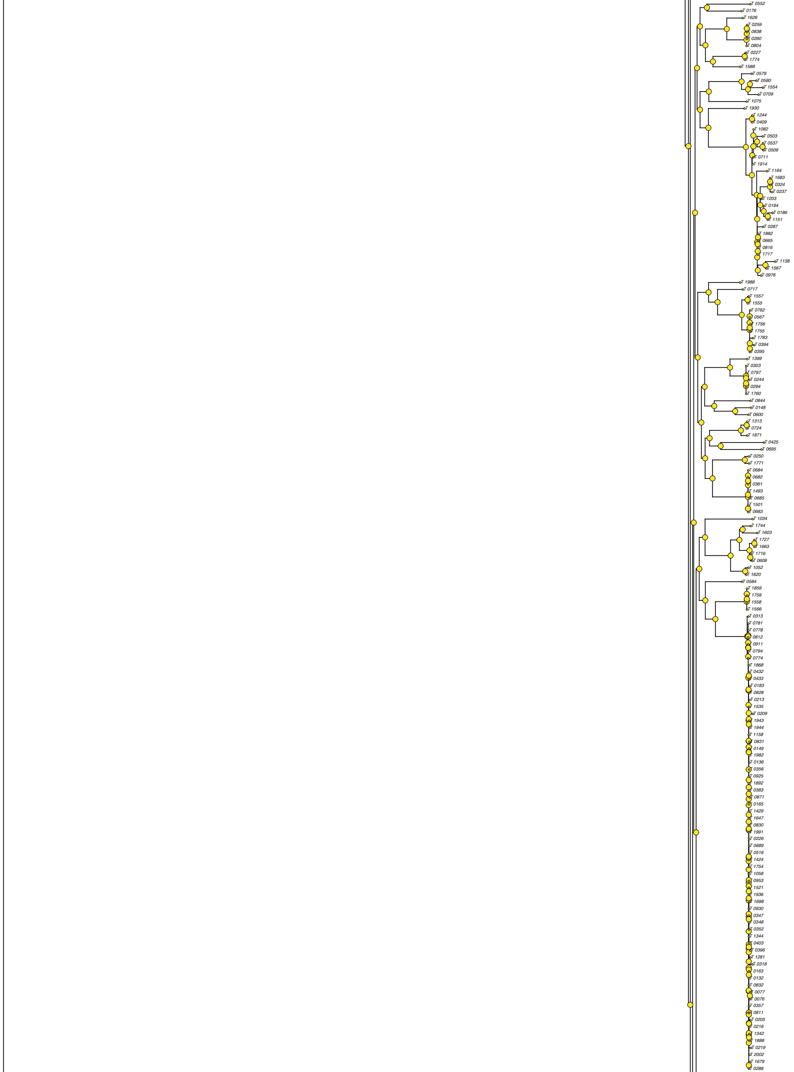
T 0552
T 0176
T 1626
T 0259
0838
T 0260
T 0804
T 0227
1774
T 1588
T 0579
T 0580
T 1554
T 0709
T 1075
T 1930
T 1244
T 0409
T 1082
T 0503
T 0537
T 0509
T 0711
T 1914
T 1164
T 1683
0324
T 0237
T 1203
T 0194
T 0186
1151
T 0287
T 1882
0665
T 0816
1717
T 1138
1567
T 0976
T 1988
T 0717
T 1557
1555
T 0762
0567
1756
1755
T 1783
T 0394
0395
T 1399
T 0303
0797
T 0244
0294
T 1760
T 0844
T 0148
T 0600
T 1313
0724
T 1871
T 0425
T 0695
T 0250
T 1771
T 0684
0682
0361
T 1493
T 0685
T 1501
0683
T 1034
T 1744
T 1603
T 1727
1663
T 1716
T 0608
T 1052
1620
T 0584
T 1859
1759
1558
1566
T 0313
T 0781
T 0778
0812
0911
0794
0774
T 1868
T 0432
0433
T 0183
0828
T 0213
1535
T 0209
1943
1944
T 1158
0831
0149
T 1982
T 0136
0356
T 0925
1892
0383
T 0871
0165
T 1429
1647
0830
T 1991
T 0226
T 0689
T 0516
1424
T 1754
T 1058
0953
1521
T 1936
1698
T 0930
0347
0348
T 0352
T 1344
T 0403
T 0396
T 1281
T 0318
0163
T 0132
T 0832
T 0077
T 0076
T 0357
0811
T 0205
T 0216
1342
1888
T 0219
T 2002
T 1679
0288

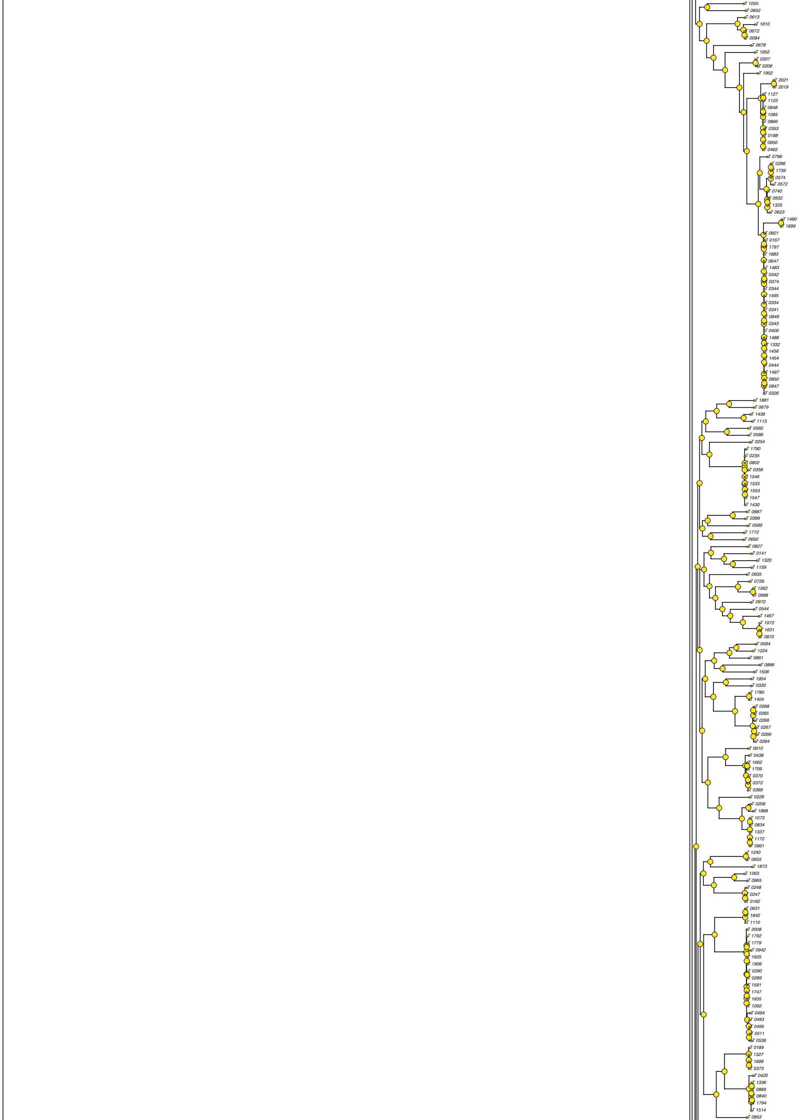
T 1055
T 0852
T 0613
T 1810
T 0672
T 0084
T 0678
T 1952
T 0207
T 0208
T 1902
T 2021
T 2019
T 1127
T 1123
T 0648
T 1085
T 0866
T 0353
T 0188
T 0950
T 0462
T 0796
T 0286
T 1739
T 0574
T 0572
T 0740
T 0932
T 1325
T 0623
T 1460
T 1899
T 0821
T 0167
T 1797
T 1883
T 0647
T 1483
T 0342
T 0374
T 0344
T 1495
T 0334
T 0341
T 0849
T 0343
T 0406
T 1488
T 1332
T 1456
T 1454
T 0444
T 1497
T 0850
T 0847
T 0326
T 1881
T 0679
T 1436
T 1113
T 0560
T 0586
T 0254
T 1790
T 0235
T 0802
T 0358
T 1546
T 1533
T 1553
T 1547
T 1430
T 0887
T 0399
T 0589
T 1772
T 0692
T 0827
T 0141
T 1320
T 1159
T 0935
T 0726
T 1562
T 0998
T 0972
T 0544
T 1467
T 1972
T 1631
T 0870
T 0094
T 1224
T 0861
T 0886
T 1506
T 1954
T 0320
T 1780
T 1404
T 0268
T 0265
T 0266
T 0267
T 0269
T 0264
T 0610
T 0438
T 1662
T 1709
T 0370
T 0372
T 0369
T 0228
T 0206
T 1968
T 1073
T 0834
T 1337
T 1172
T 0961
T 1240
T 0655
T 1873
T 1263
T 0965
T 0248
T 0247
T 0182
T 0631
T 1842
T 1110
T 2008
T 1792
T 1779
T 0942
T 1925
T 1906
T 0290
T 0289
T 1591
T 1747
T 1935
T 1092
T 0494
T 0493
T 0495
T 0511
T 0536
T 0189
T 1327
T 1699
T 0373
T 0420
T 1336
T 0889
T 0840
T 1794
T 1514
T 0853

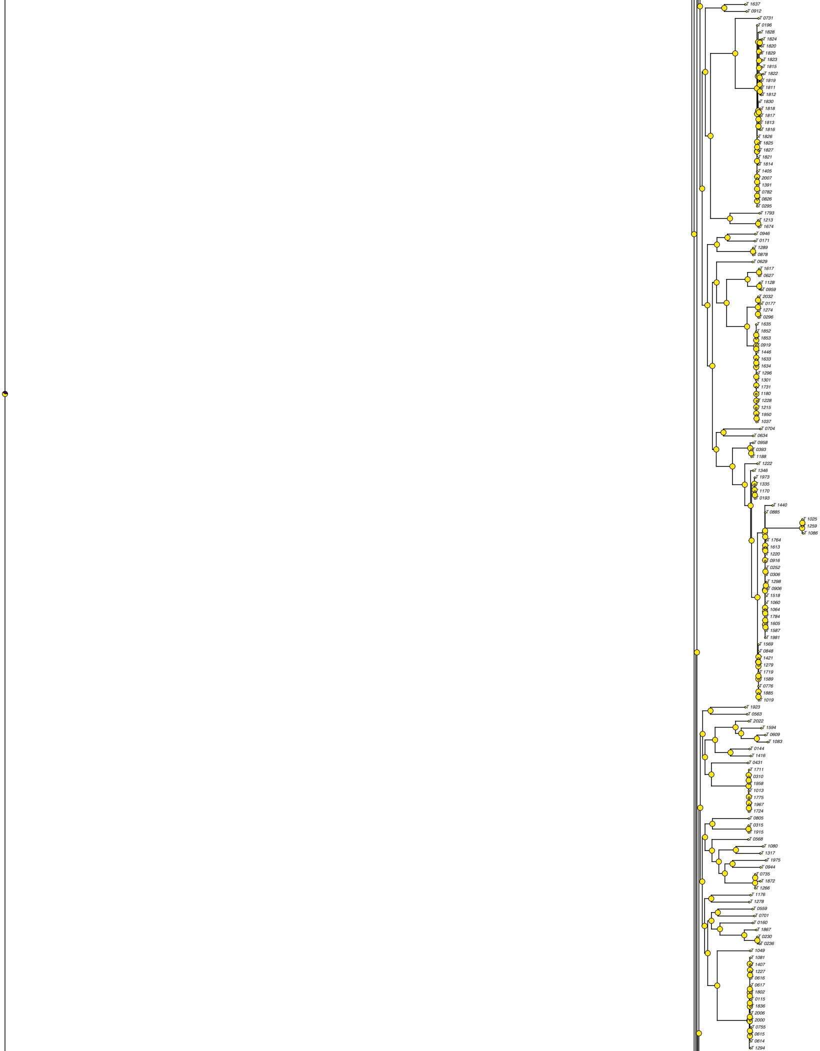
T 1637
T 0912
T 0731
T 0196
T 1828
T 1824
T 1820
T 1829
T 1823
T 1815
T 1822
T 1819
T 1811
T 1812
T 1830
T 1818
T 1817
T 1813
T 1816
T 1826
T 1825
T 1827
T 1821
T 1814
T 1405
2007
1391
0782
0826
T 0295
T 1793
T 1213
T 1674
T 0946
T 0171
T 1289
0878
T 0629
T 1617
T 0627
T 1128
T 0959
T 2032
T 0177
T 1274
T 0296
T 1635
T 1852
1853
0919
1446
1633
1634
T 1296
1301
1731
1180
1228
1215
1950
1037
T 0704
T 0634
T 0958
0393
1188
T 1222
T 1346
T 1973
1335
1170
0193
T 1440
T 0885
T 1025
1259
T 1086
T 1764
1613
1220
0916
T 0252
0306
T 1298
T 0906
T 1518
T 1060
1064
1784
1605
1587
T 1981
T 1569
T 0848
1421
1279
T 1719
1589
T 0776
1885
1019
T 1923
T 0563
T 2022
T 1594
T 0609
T 1083
T 0144
T 1416
T 0431
T 1711
0310
1958
1013
1775
1967
1724
T 0805
T 0315
1915
T 0568
T 1080
T 1317
T 1975
T 0944
T 0735
T 1872
T 1266
T 1176
T 1278
T 0559
T 0701
T 0160
T 1867
T 0230
T 0236
T 1049
T 1081
1407
1227
0616
T 0617
1802
0115
1836
T 2006
2000
T 0755
0615
T 0614
T 1294

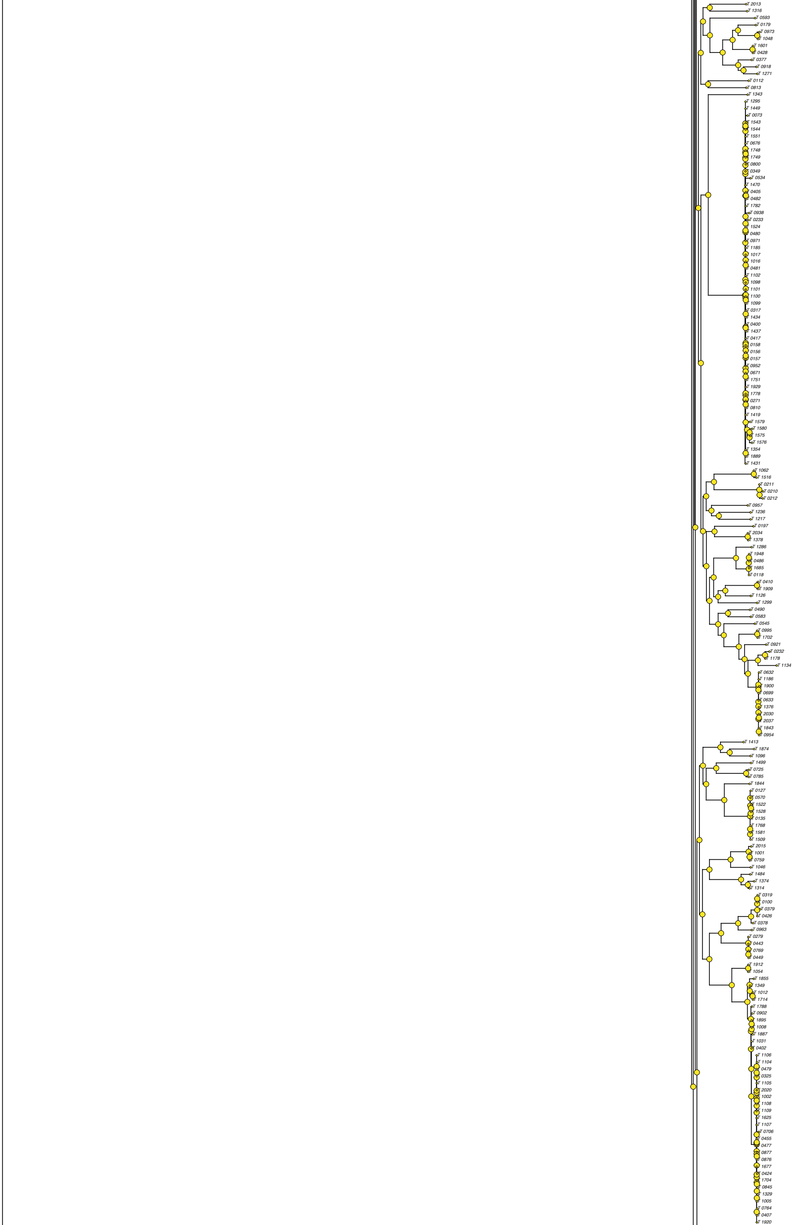
T 2013
T 1316
T 0593
T 0179
T 0973
T 1048
T 1601
T 0428
T 0377
T 0918
T 1271
T 0112
T 0813
T 1343
T 1295
T 1449
T 0073
T 1543
T 1544
T 1551
T 0676
T 1748
T 1749
T 0800
T 0349
T 0534
T 1470
T 0405
T 0482
T 1782
T 0938
T 0233
T 1524
T 0480
T 0971
T 1185
T 1017
T 1016
T 0481
T 1102
T 1098
T 1101
T 1100
T 1099
T 0317
T 1434
T 0400
T 1437
T 0417
T 0158
T 0156
T 0157
T 0952
T 0671
T 1751
T 1929
T 1778
T 0271
T 0810
T 1419
T 1579
T 1580
T 1575
T 1576
T 1354
T 1889
T 1431
T 1062
T 1516
T 0211
T 0210
T 0212
T 0957
T 1236
T 1217
T 0197
T 2034
T 1378
T 1286
T 1948
T 0486
T 1685
T 0118
T 0410
T 1909
T 1126
T 1299
T 0490
T 0583
T 0545
T 0995
T 1702
T 0921
T 0232
T 1178
T 1134
T 0632
T 1186
T 1900
T 0699
T 0633
T 1376
T 2030
T 2037
T 1843
T 0954
T 1413
T 1874
T 1096
T 1499
T 0725
T 0785
T 1844
T 0127
T 0570
T 1522
T 1528
T 0135
T 1768
T 1581
T 1509
T 2015
T 1001
T 0759
T 1046
T 1484
T 1374
T 1314
T 0319
T 0100
T 0379
T 0426
T 0378
T 0963
T 0279
T 0443
T 0769
T 0449
T 1912
T 1054
T 1855
T 1349
T 1012
T 1714
T 1788
T 0902
T 1895
T 1008
T 1887
T 1031
T 0402
T 1106
T 1104
T 0479
T 0325
T 1105
T 2020
T 1002
T 1108
T 1109
T 1625
T 1107
T 0706
T 0455
T 0477
T 0877
T 0876
T 1677
T 0424
T 1704
T 0845
T 1329
T 1005
T 0764
T 0407
T 1920

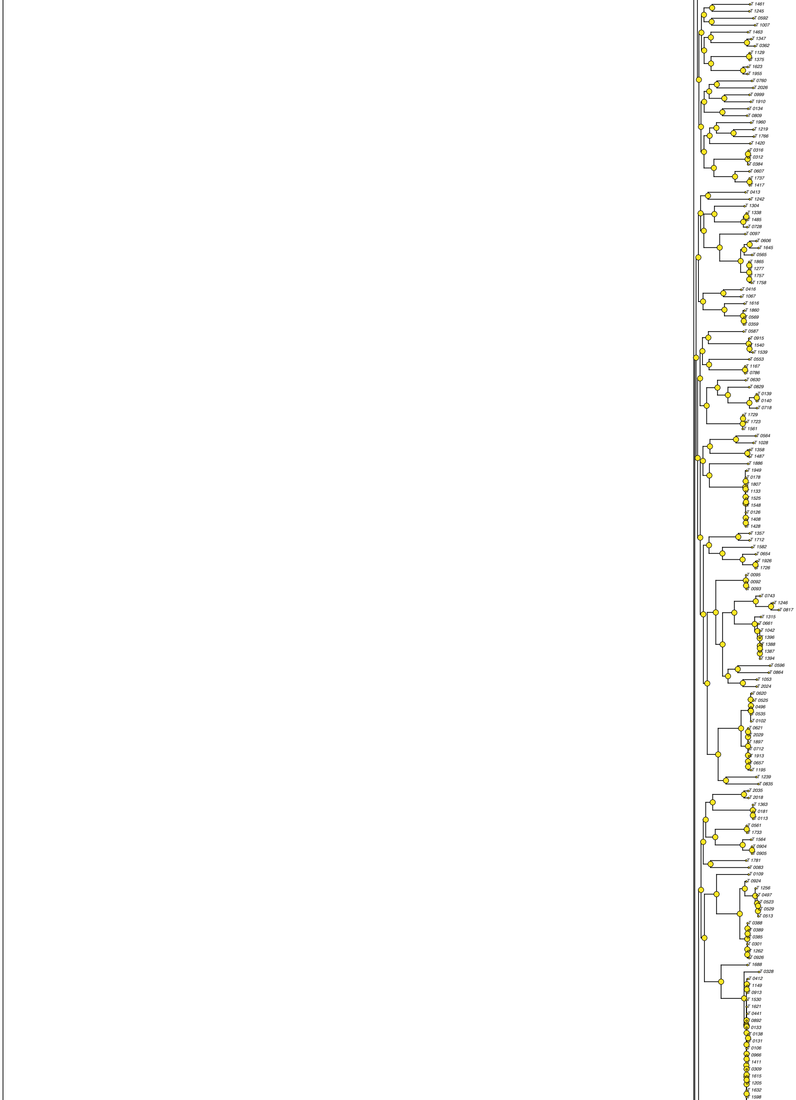
T 1461
T 1245
T 0592
T 1007
T 1463
T 1347
T 0362
T 1129
T 1375
T 1623
T 1955
T 0760
T 2026
T 0999
T 1910
T 0134
T 0809
T 1960
T 1219
T 1766
T 1420
T 0316
T 0312
T 0384
T 0607
T 1737
T 1417
T 0413
T 1242
T 1304
T 1338
T 1485
T 0728
T 0097
T 0606
T 1645
T 0565
T 1865
T 1277
T 1757
T 1758
T 0416
T 1067
T 1616
T 1860
T 0569
T 0359
T 0587
T 0915
T 1540
T 1539
T 0553
T 1167
T 0786
T 0630
T 0829
T 0139
T 0140
T 0718
T 1729
T 1723
T 1561
T 0564
T 1028
T 1358
T 1487
T 1886
T 1949
T 0178
T 1807
T 1133
T 1525
T 1548
T 0126
T 1408
T 1428
T 1357
T 1712
T 1582
T 0654
T 1926
T 1726
T 0095
T 0092
T 0093
T 0743
T 1246
T 0817
T 1315
T 0661
T 1042
T 1396
T 1388
T 1387
T 1394
T 0596
T 0864
T 1053
T 2024
T 0620
T 0525
T 0496
T 0535
T 0102
T 0621
T 2029
T 1897
T 0712
T 1913
T 0657
T 1195
T 1239
T 0835
T 2035
T 2018
T 1363
T 0181
T 0113
T 0561
T 1733
T 1564
T 0904
T 0905
T 1781
T 0083
T 0109
T 0924
T 1256
T 0497
T 0523
T 0529
T 0513
T 0388
T 0389
T 0385
T 0301
T 1262
T 0926
T 1688
T 0328
T 0412
T 1149
T 0913
T 1530
T 1621
T 0441
T 0892
T 0133
T 0138
T 0131
T 0106
T 0966
T 1411
T 0309
T 1615
T 1205
T 1632
T 1598

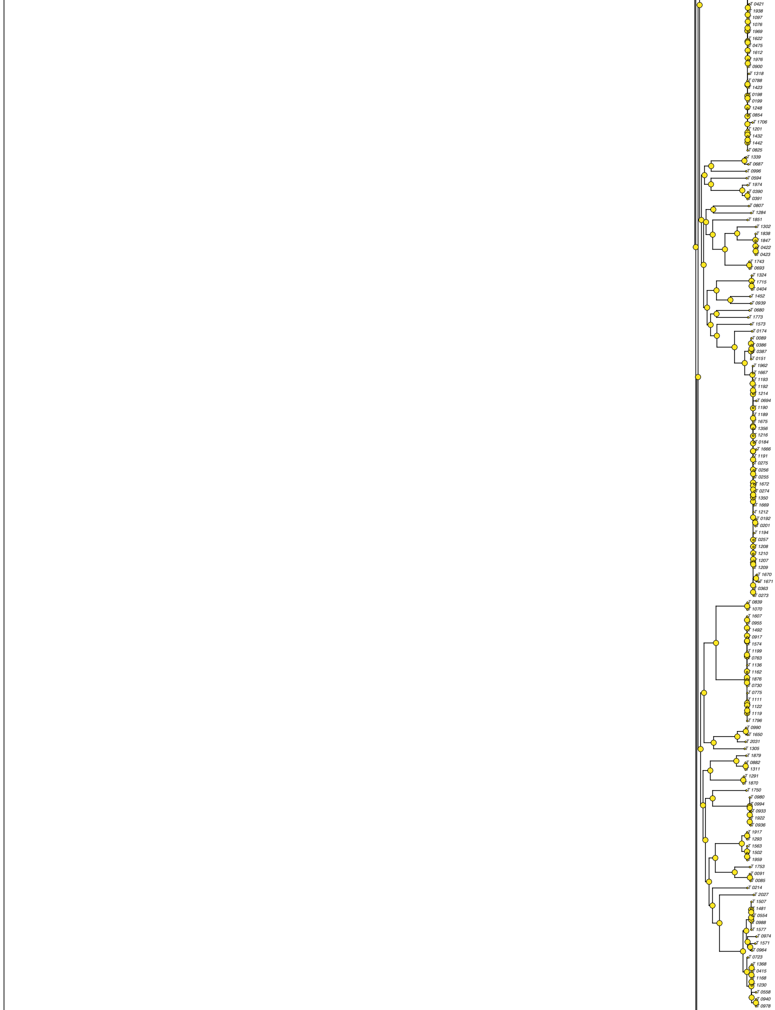
T 0421
T 1938
T 1097
T 1076
T 1969
T 1622
T 0475
T 1612
T 1976
T 0900
T 1318
T 0788
T 1423
T 0198
T 0199
T 1248
T 0854
T 1706
T 1201
T 1432
T 1442
T 0825
T 1339
T 0687
T 0996
T 0594
T 1974
T 0390
T 0391
T 0807
T 1284
T 1851
T 1302
T 1838
T 1847
T 0422
T 0423
T 1743
T 0693
T 1324
T 1715
T 0404
T 1452
T 0939
T 0680
T 1773
T 1573
T 0174
T 0089
T 0386
T 0387
T 0151
T 1962
T 1667
T 1193
T 1192
T 1214
T 0694
T 1190
T 1189
T 1675
T 1356
T 1216
T 0184
T 1666
T 1191
T 0275
T 0256
T 0255
T 1672
T 0274
T 1350
T 1669
T 1212
T 0192
T 0201
T 1194
T 0257
T 1208
T 1210
T 1207
T 1209
T 1670
T 1671
T 0363
T 0273
T 0839
T 1070
T 1607
T 0955
T 1492
T 0917
T 1574
T 1199
T 0763
T 1136
T 1162
T 1876
T 0730
T 0775
T 1111
T 1122
T 1119
T 1796
T 0990
T 1650
T 2031
T 1305
T 1879
T 0882
T 1311
T 1291
T 1870
T 1750
T 0980
T 0994
T 0933
T 1922
T 0936
T 1917
T 1293
T 1563
T 1502
T 1959
T 1753
T 0091
T 0085
T 0214
T 2027
T 1507
T 1481
T 0554
T 0988
T 1577
T 0974
T 1571
T 0964
T 0723
T 1368
T 0415
T 1168
T 1230
T 0558
T 0940
T 0978

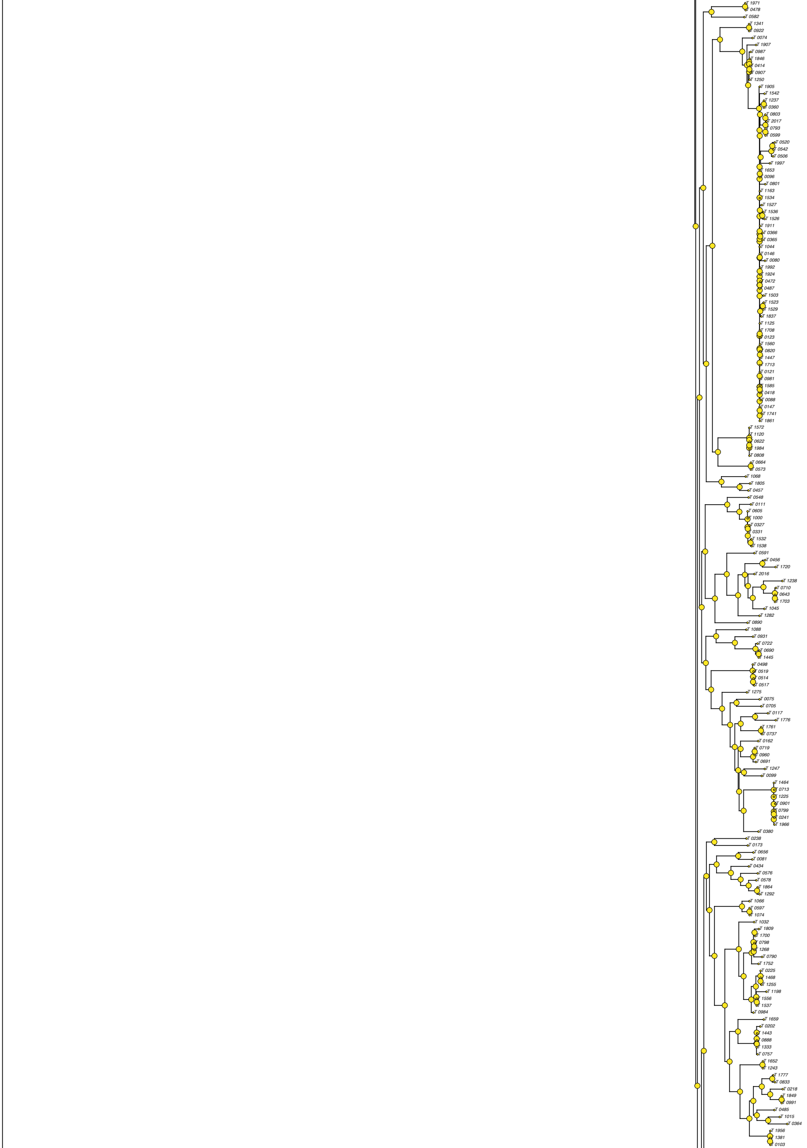
T 1971
T 0478
T 0582
T 1341
T 0922
T 0074
T 1907
T 0987
T 1846
T 0414
T 0907
T 1250
T 1905
T 1542
T 1237
T 0360
T 0803
T 2017
T 0793
T 0599
T 0520
T 0542
T 0506
T 1997
T 1653
T 0096
T 0801
T 1163
T 1534
T 1527
T 1536
T 1526
T 1911
T 0366
T 0365
T 1044
T 0146
T 0080
T 1992
T 1924
T 0472
T 0487
T 1503
T 1523
T 1529
T 1837
T 1125
T 1708
T 0123
T 1560
T 0820
T 1447
T 1713
T 0121
T 0981
T 1585
T 0418
T 0088
T 0147
T 1741
T 1861
T 1572
T 1120
T 0622
T 1984
T 0808
T 0664
T 0573
T 1068
T 1805
T 0457
T 0548
T 0111
T 0605
T 1000
T 0327
T 0331
T 1532
T 1538
T 0591
T 0456
T 1720
T 2016
T 1238
T 0710
T 0643
T 1703
T 1045
T 1282
T 0890
T 1088
T 0931
T 0722
T 0690
T 1445
T 0498
T 0519
T 0514
T 0517
T 1275
T 0075
T 0705
T 0117
T 1776
T 1761
T 0737
T 0162
T 0719
T 0960
T 0691
T 1247
T 0099
T 1464
T 0713
T 1225
T 0901
T 0799
T 0241
T 1966
T 0380
T 0238
T 0173
T 0656
T 0081
T 0434
T 0576
T 0578
T 1864
T 1292
T 1066
T 0597
T 1074
T 1032
T 1809
T 1700
T 0798
T 1268
T 0790
T 1752
T 0225
T 1468
T 1255
T 1198
T 1556
T 1537
T 0984
T 1659
T 0202
T 1443
T 0888
T 1333
T 0757
T 1652
T 1243
T 1777
T 0833
T 0218
T 1849
T 0991
T 0485
T 1015
T 0364
T 1956
T 1381
T 0103

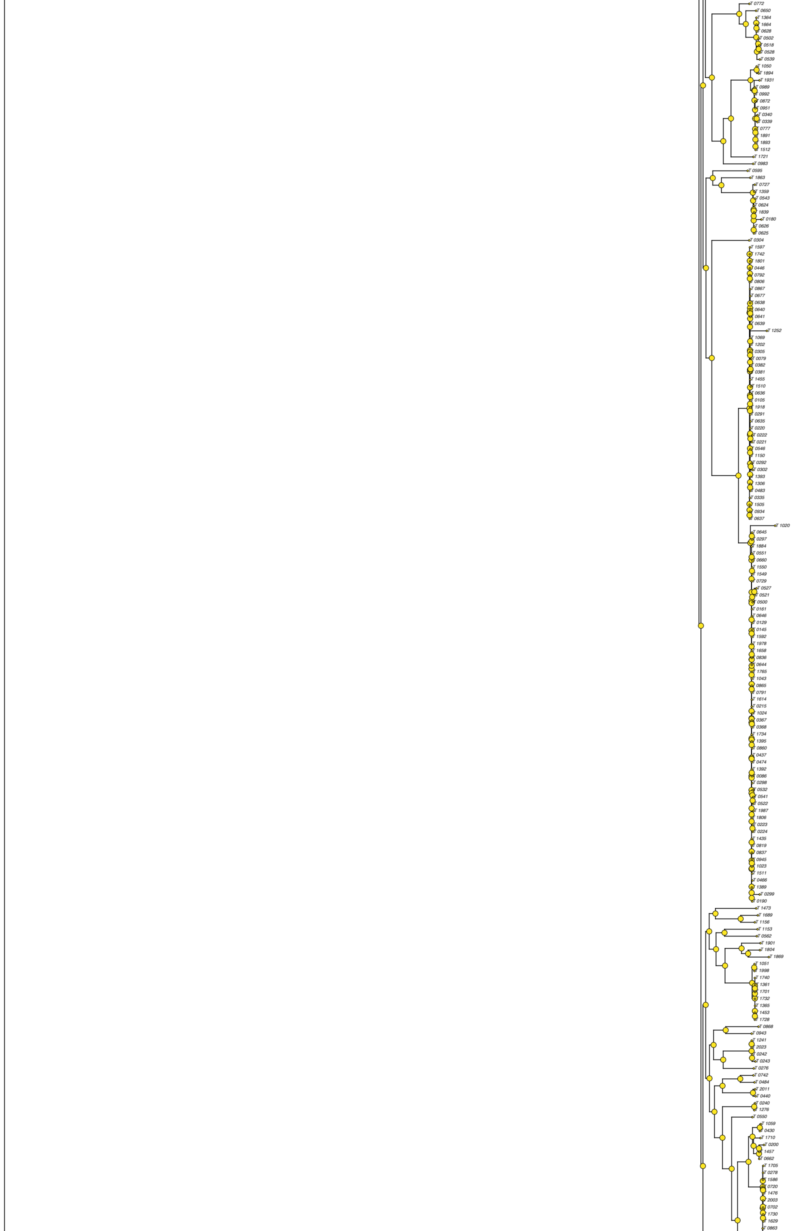

T 0772
T 0650
T 1364
T 1664
T 0628
T 0502
T 0518
T 0528
T 0539
T 1050
T 1894
T 1931
T 0989
T 0992
T 0872
T 0951
T 0340
T 0339
T 0777
T 1891
T 1893
T 1512
T 1721
T 0983
T 0595
T 1863
T 0727
T 1359
T 0543
T 0624
T 1839
T 0180
T 0626
T 0625
T 0304
T 1597
T 1742
T 1801
T 0446
T 0792
T 0806
T 0867
T 0677
T 0638
T 0640
T 0641
T 0639
T 1252
T 1069
T 1202
T 0305
T 0079
T 0382
T 0381
T 1455
T 1510
T 0636
T 0105
T 1918
T 0291
T 0635
T 0220
T 0222
T 0221
T 0546
T 1150
T 0292
T 0302
T 1393
T 1306
T 0483
T 0335
T 1505
T 0934
T 0637
T 1020
T 0645
T 0297
T 1884
T 0551
T 0660
T 1550
T 1549
T 0729
T 0527
T 0521
T 0500
T 0161
T 0646
T 0129
T 0145
T 1592
T 1978
T 1658
T 0836
T 0644
T 1765
T 1043
T 0865
T 0791
T 1614
T 0215
T 1024
T 0367
T 0368
T 1734
T 1395
T 0860
T 0437
T 0474
T 1392
T 0086
T 0298
T 0532
T 0541
T 0522
T 1987
T 1806
T 0223
T 0224
T 1435
T 0819
T 0837
T 0945
T 1023
T 1511
T 0466
T 1389
T 0299
T 0190
T 1473
T 1689
T 1156
T 1153
T 0562
T 1901
T 1804
T 1869
T 1051
T 1998
T 1740
T 1361
T 1701
T 1732
T 1365
T 1453
T 1728
T 0868
T 0943
T 1241
T 2023
T 0242
T 0243
T 0276
T 0742
T 0484
T 2011
T 0440
T 0240
T 1276
T 0550
T 1059
T 0430
T 1710
T 0200
T 1457
T 0662
T 1705
T 0278
T 1586
T 0720
T 1476
T 2003
T 0702
T 1730
T 1629
T 0863

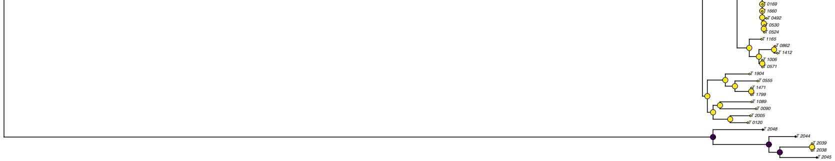

0169
1660
T 0492
T 0530
T 0524
T 1165
T 0862
T 1412
T 1006
T 0571
T 1904
T 0555
T 1471
T 1799
T 1089
T 0090
T 2005
T 0120
T 2048
T 2044
T 2039
T 2038
T 2045

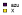

Effector gene: azu

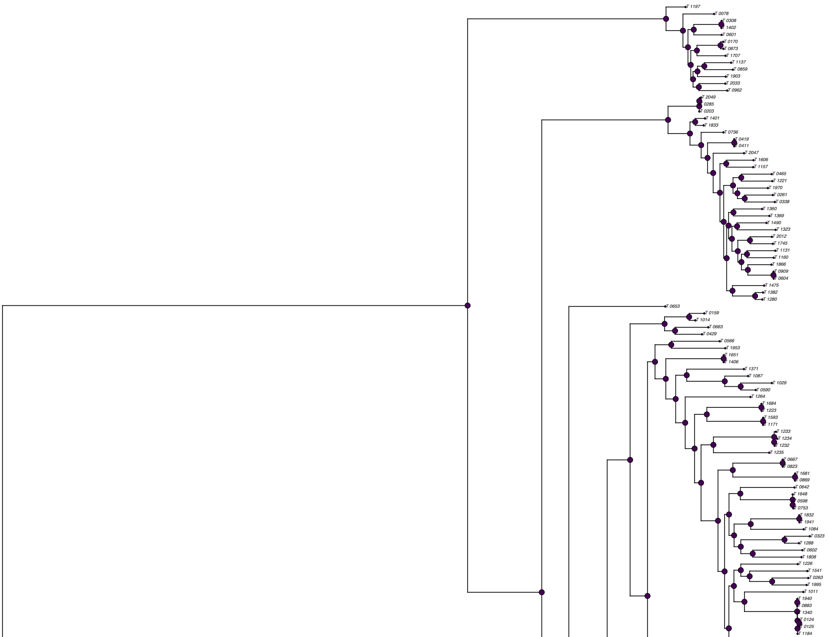

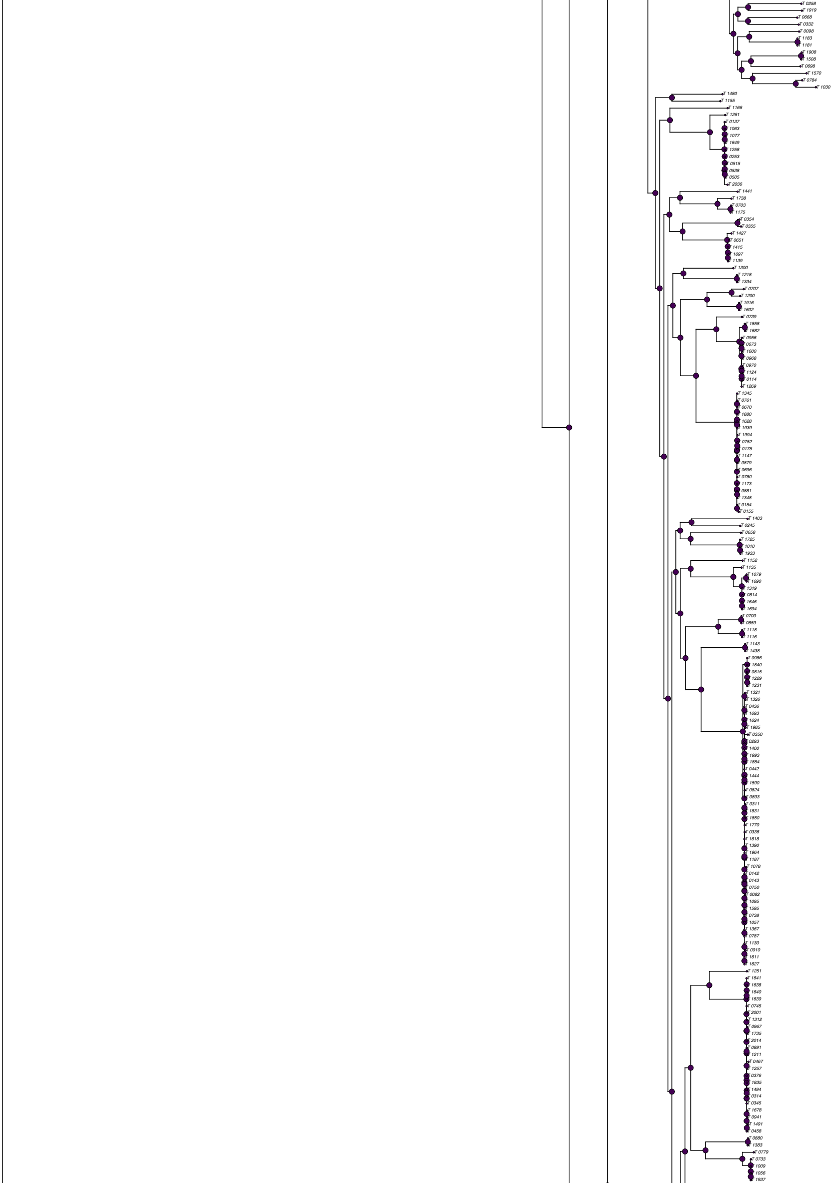
T 0258
T 1919
T 0668
T 0332
T 0098
T 1183
T 1181
T 1908
T 1508
T 0698
T 1570
T 0784
T 1030
T 1480
T 1155
T 1166
T 1261
T 0137
1063
1077
T 1649
T 1258
0253
T 0515
0538
T 0505
T 2036
T 1441
T 1738
T 0703
T 1175
T 0354
T 0355
T 1427
T 0651
1415
T 1697
T 1139
T 1300
T 1218
T 1334
T 0707
T 1200
T 1916
T 1602
T 0739
T 1858
T 1682
T 0956
0673
T 1600
T 0968
T 0970
T 1124
T 0114
T 1269
T 1345
T 0761
T 0670
1880
T 1628
T 1939
T 1994
T 0752
T 0175
T 1147
T 0879
T 0696
T 0780
1173
T 0881
T 1348
T 0154
T 0155
T 1403
T 0245
T 0658
T 1725
1010
T 1933
T 1152
T 1135
T 1079
T 1690
T 1319
T 0814
T 1646
T 1694
T 0700
T 0659
T 1118
T 1116
T 1143
T 1438
T 0986
1840
T 0815
T 1229
T 1231
T 1321
T 1326
T 0436
T 1693
T 1624
T 1985
T 0350
0293
1400
1993
T 1854
T 0442
1444
T 1590
T 0824
0893
T 0311
1831
1850
T 1770
T 0336
T 1618
T 1390
T 1964
1187
T 1078
T 0142
T 0143
T 0750
T 0082
T 1095
T 1595
T 0738
T 1057
T 1367
T 0787
T 1130
T 0910
1611
T 1627
T 1251
T 1641
1638
1640
1639
T 0745
2001
T 1312
T 0967
T 1735
2014
T 0891
1211
T 0467
T 1257
T 0376
T 1835
T 1494
T 0314
T 0345
T 1678
T 0941
T 1491
T 0458
T 0880
T 1383
T 0779
T 0733
1009
1056
T 1937

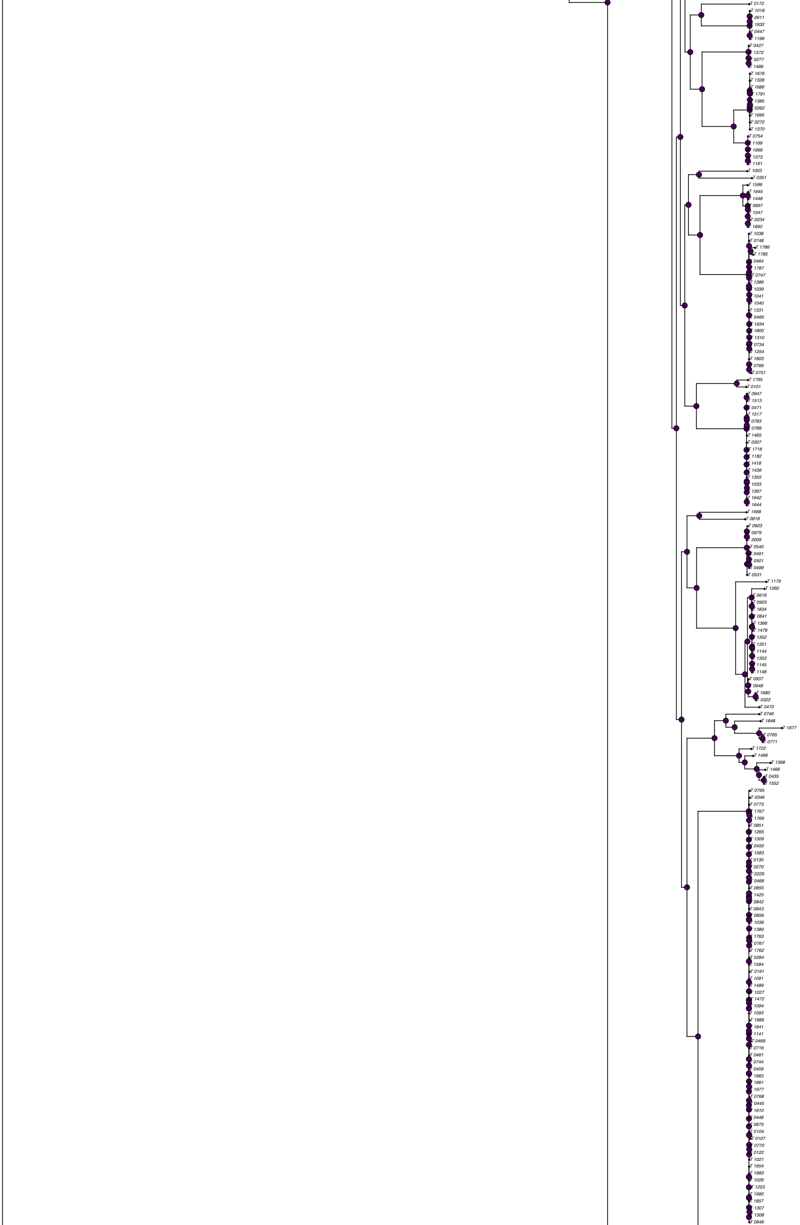
T 0172
T 1018
0611
1932
T 0447
T 1196
T 0427
1372
T 0277
T 1486
T 1676
T 1328
T 1686
1791
1385
0262
T 1695
T 0272
T 1370
T 0754
1169
1999
1373
T 1161
T 1003
T 0351
T 1596
T 1945
T 1448
0697
T 1047
T 0234
T 1692
T 1038
T 0748
T 1786
T 1785
0464
1787
T 0747
T 1386
1039
1041
1040
T 1331
0460
1934
1800
1310
0734
T 1254
T 1803
0766
T 0751
T 1795
T 0101
T 0947
T 1513
0471
T 1517
0783
0789
T 1465
T 0307
T 1718
T 1182
1418
T 1439
T 1355
1033
1397
T 1642
T 1644
T 1568
T 0818
T 0923
0979
T 2009
T 0540
0491
0501
T 0499
T 0531
T 1179
T 1260
0619
T 0903
T 1834
0841
T 1366
T 1478
1352
1351
1144
1353
1145
T 1148
T 0937
0948
T 1680
T 0322
T 0470
T 0746
T 1848
T 1877
T 0765
T 0771
T 1722
T 1496
T 1398
T 1466
T 0435
T 1552
T 0795
T 0346
T 0773
1767
1769
T 0851
1265
1309
0450
T 1583
T 0130
0270
T 0229
0468
T 0855
1425
0842
T 0843
0856
T 1036
1380
T 1763
0767
T 1762
T 0284
T 1584
T 0191
T 1091
T 1489
1027
T 1472
T 1094
T 1093
T 1989
1841
1141
T 0469
T 0716
T 0461
0744
T 0459
1963
1961
1977
T 0768
0445
1610
0448
T 0875
T 0104
T 0107
0770
0122
T 1021
T 1654
T 1983
1026
T 1253
T 1990
T 1857
T 1307
1308
T 0949

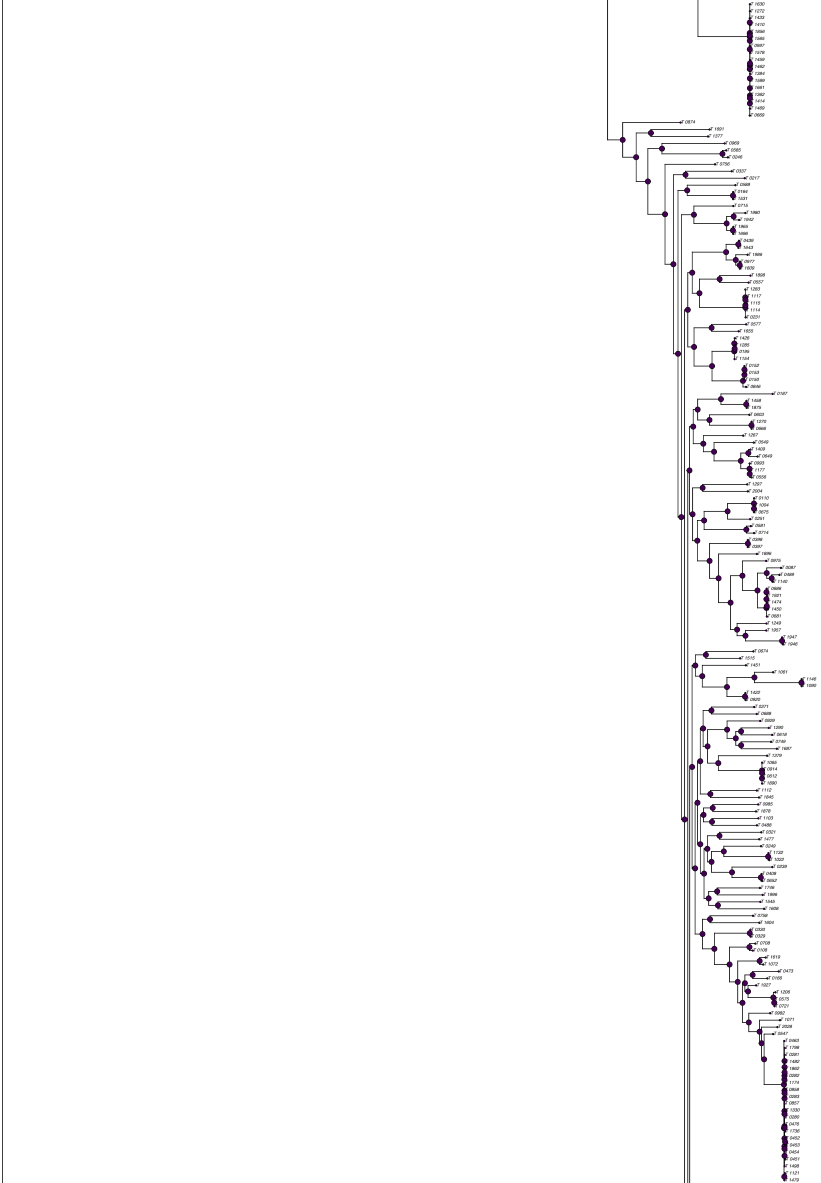
T 1630
T 1272
T 1433
T 1410
T 1856
T 1565
T 0997
T 1578
T 1459
T 1462
T 1384
T 1599
T 1661
T 1362
T 1414
T 1469
T 0669
T 0874
T 1691
T 1377
T 0969
T 0585
T 0246
T 0756
T 0337
T 0217
T 0588
T 0164
T 1531
T 0715
T 1980
T 1942
T 1965
T 1696
T 0439
T 1643
T 1986
T 0977
T 1609
T 1898
T 0557
T 1283
T 1117
T 1115
T 1114
T 0231
T 0577
T 1655
T 1426
T 1285
T 0195
T 1154
T 0152
T 0153
T 0150
T 0846
T 0187
T 1458
T 1875
T 0603
T 1270
T 0666
T 1267
T 0549
T 1409
T 0649
T 0993
T 1177
T 0556
T 1297
T 2004
T 0110
T 1004
T 0675
T 0251
T 0581
T 0714
T 0398
T 0397
T 1896
T 0975
T 0087
T 0489
T 1140
T 0686
T 1921
T 1474
T 1450
T 0681
T 1249
T 1957
T 1947
T 1946
T 0674
T 1515
T 1451
T 1061
T 1146
T 1090
T 1422
T 0920
T 0371
T 0688
T 0929
T 1290
T 0618
T 0749
T 1687
T 1379
T 1065
T 0914
T 0612
T 1890
T 1112
T 1845
T 0985
T 1878
T 1103
T 0488
T 0321
T 1477
T 0249
T 1132
T 1022
T 0239
T 0408
T 0652
T 1746
T 1996
T 1545
T 1608
T 0758
T 1604
T 0330
T 0329
T 0708
T 0108
T 1619
T 1072
T 0473
T 0166
T 1927
T 1206
T 0575
T 0721
T 0982
T 1071
T 2028
T 0547
T 0463
T 1798
T 0281
T 1482
T 1862
T 0282
T 1174
T 0858
T 0283
T 0857
T 1330
T 0280
T 0476
T 1736
T 0452
T 0453
T 0454
T 0451
T 1498
T 1121
T 1479

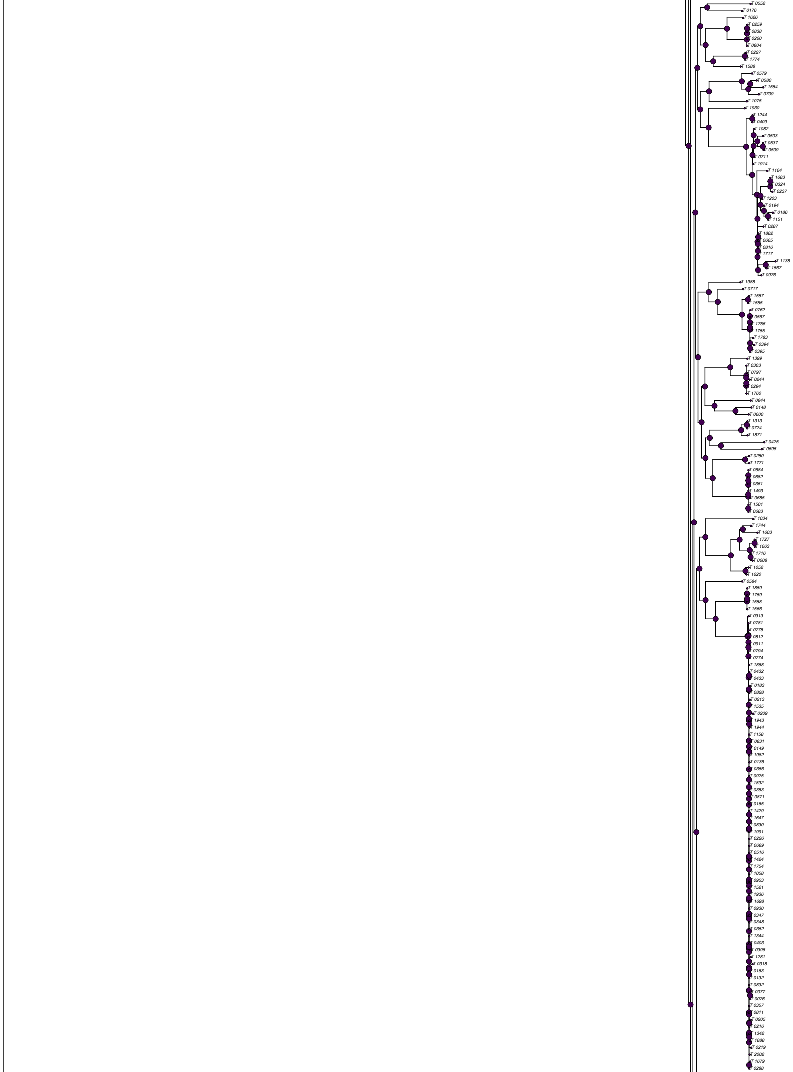
T 0552
T 0176
T 1626
T 0259
0838
0260
T 0804
T 0227
T 1774
T 1588
T 0579
T 0580
T 1554
T 0709
T 1075
T 1930
T 1244
T 0409
T 1082
T 0503
T 0537
T 0509
T 0711
T 1914
T 1164
T 1683
0324
T 0237
T 1203
T 0194
T 0186
T 1151
T 0287
T 1882
0665
T 0816
T 1717
T 1138
T 1567
T 0976
T 1988
T 0717
T 1557
T 1555
T 0762
0567
T 1756
T 1755
T 1783
T 0394
T 0395
T 1399
T 0303
T 0797
T 0244
0294
T 1760
T 0844
T 0148
T 0600
T 1313
T 0724
T 1871
T 0425
T 0695
T 0250
T 1771
T 0684
0682
0361
T 1493
T 0685
T 1501
T 0683
T 1034
T 1744
T 1603
T 1727
T 1663
T 1716
T 0608
T 1052
T 1620
T 0584
T 1859
1759
1558
T 1566
T 0313
T 0781
T 0778
0812
0911
T 0794
0774
T 1868
T 0432
0433
T 0183
T 0828
T 0213
1535
T 0209
1943
T 1944
T 1158
0831
0149
T 1982
T 0136
0356
T 0925
T 1892
0383
T 0871
0165
T 1429
T 1647
T 0830
1991
T 0226
T 0689
T 0516
1424
T 1754
T 1058
0953
1521
T 1936
1698
T 0930
0347
T 0348
T 0352
T 1344
0403
T 0396
T 1281
T 0318
0163
T 0132
T 0832
T 0077
T 0076
T 0357
0811
T 0205
T 0216
1342
T 1888
T 0219
T 2002
T 1679
T 0288

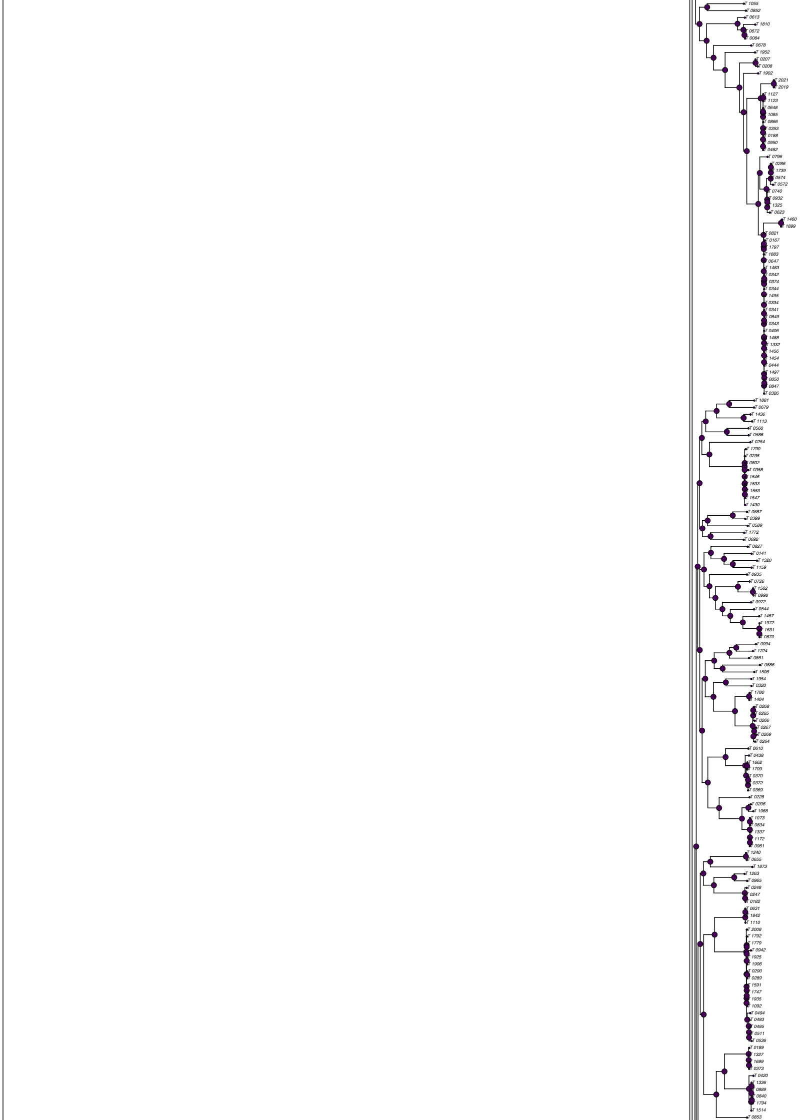
T 1055
T 0852
T 0613
T 1810
T 0672
T 0084
T 0678
T 1952
T 0207
T 0208
T 1902
T 2021
T 2019
T 1127
T 1123
T 0648
T 1085
T 0866
T 0353
T 0188
T 0950
T 0462
T 0796
T 0286
T 1739
T 0574
T 0572
T 0740
T 0932
T 1325
T 0623
T 1460
T 1899
T 0821
T 0167
T 1797
T 1883
T 0647
T 1483
T 0342
T 0374
T 0344
T 1495
T 0334
T 0341
T 0849
T 0343
T 0406
T 1488
T 1332
T 1456
T 1454
T 0444
T 1497
T 0850
T 0847
T 0326
T 1881
T 0679
T 1436
T 1113
T 0560
T 0586
T 0254
T 1790
T 0235
T 0802
T 0358
T 1546
T 1533
T 1553
T 1547
T 1430
T 0887
T 0399
T 0589
T 1772
T 0692
T 0827
T 0141
T 1320
T 1159
T 0935
T 0726
T 1562
T 0998
T 0972
T 0544
T 1467
T 1972
T 1631
T 0870
T 0094
T 1224
T 0861
T 0886
T 1506
T 1954
T 0320
T 1780
T 1404
T 0268
T 0265
T 0266
T 0267
T 0269
T 0264
T 0610
T 0438
T 1662
T 1709
T 0370
T 0372
T 0369
T 0228
T 0206
T 1968
T 1073
T 0834
T 1337
T 1172
T 0961
T 1240
T 0655
T 1873
T 1263
T 0965
T 0248
T 0247
T 0182
T 0631
T 1842
T 1110
T 2008
T 1792
T 1779
T 0942
T 1925
T 1906
T 0290
T 0289
T 1591
T 1747
T 1935
T 1092
T 0494
T 0493
T 0495
T 0511
T 0536
T 0189
T 1327
T 1699
T 0373
T 0420
T 1336
T 0889
T 0840
T 1794
T 1514
T 0853

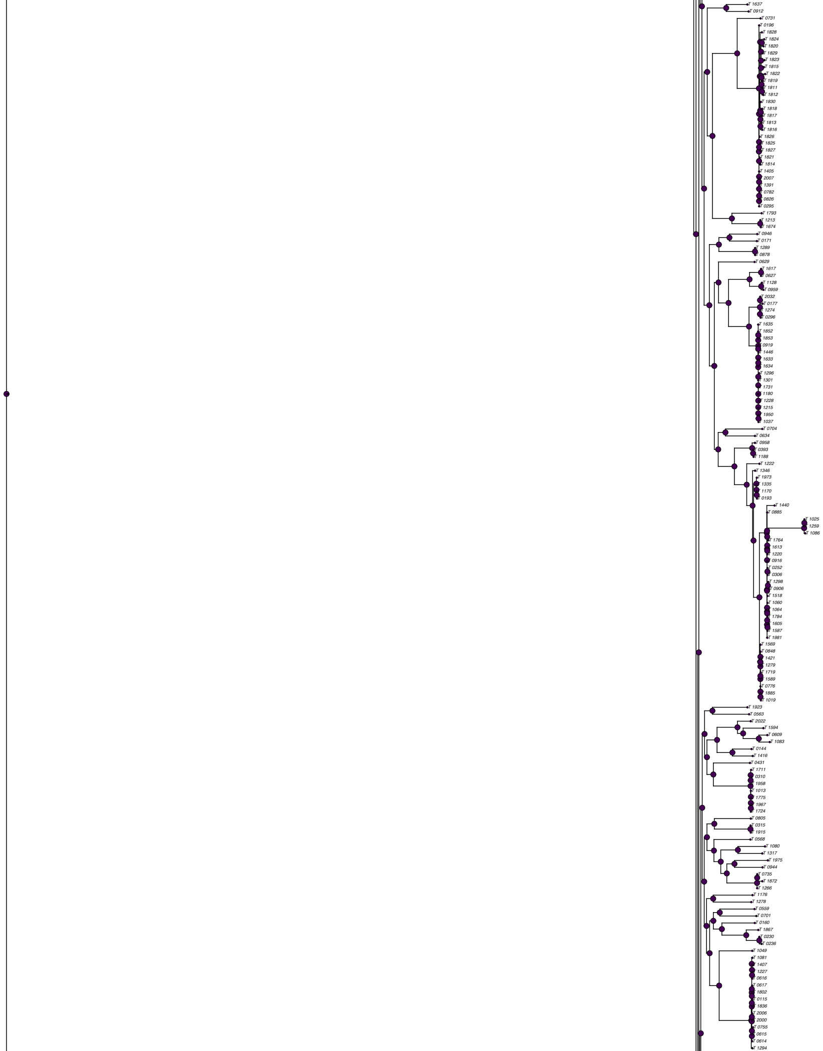
T 1637
T 0912
T 0731
T 0196
T 1828
T 1824
T 1820
T 1829
T 1823
T 1815
T 1822
T 1819
T 1811
T 1812
T 1830
T 1818
T 1817
T 1813
T 1816
T 1826
T 1825
T 1827
T 1821
T 1814
T 1405
T 2007
T 1391
T 0782
T 0826
T 0295
T 1793
T 1213
T 1674
T 0946
T 0171
T 1289
T 0878
T 0629
T 1617
T 0627
T 1128
T 0959
T 2032
T 0177
T 1274
T 0296
T 1635
T 1852
T 1853
T 0919
T 1446
T 1633
T 1634
T 1296
T 1301
T 1731
T 1180
T 1228
T 1215
T 1950
T 1037
T 0704
T 0634
T 0958
T 0393
T 1188
T 1222
T 1346
T 1973
T 1335
T 1170
T 0193
T 1440
T 0885
T 1025
T 1259
T 1086
T 1764
T 1613
T 1220
T 0916
T 0252
T 0306
T 1298
T 0906
T 1518
T 1060
T 1064
T 1784
T 1605
T 1587
T 1981
T 1569
T 0848
T 1421
T 1279
T 1719
T 1589
T 0776
T 1885
T 1019
T 1923
T 0563
T 2022
T 1594
T 0609
T 1083
T 0144
T 1416
T 0431
T 1711
T 0310
T 1958
T 1013
T 1775
T 1967
T 1724
T 0805
T 0315
T 1915
T 0568
T 1080
T 1317
T 1975
T 0944
T 0735
T 1872
T 1266
T 1176
T 1278
T 0559
T 0701
T 0160
T 1867
T 0230
T 0236
T 1049
T 1081
T 1407
T 1227
T 0616
T 0617
T 1802
T 0115
T 1836
T 2006
T 2000
T 0755
T 0615
T 0614
T 1294

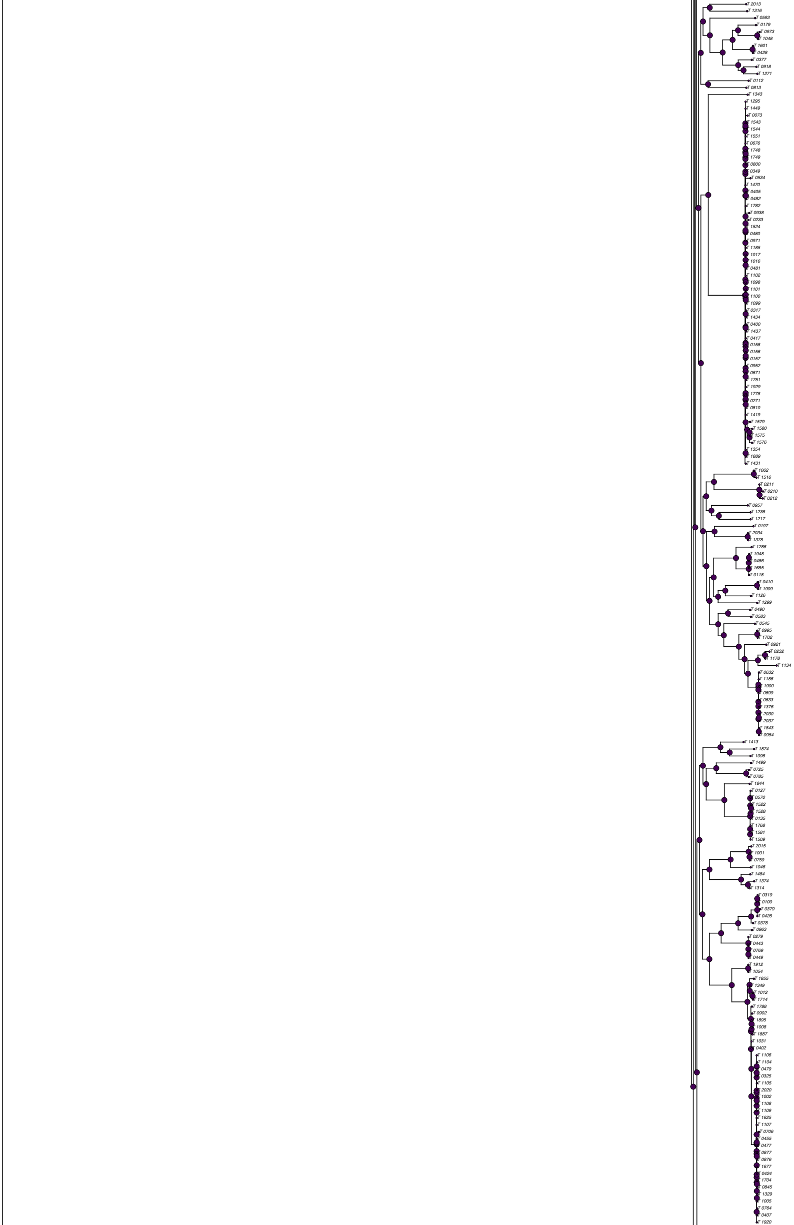

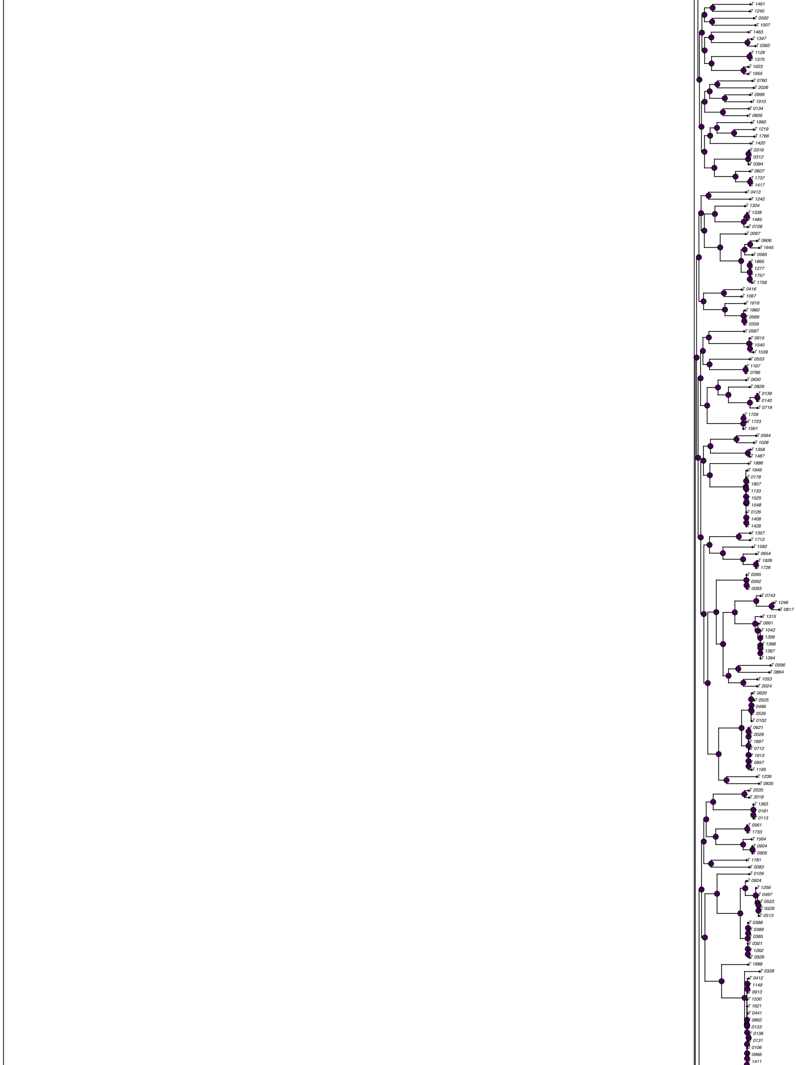
T 1461
T 1245
T 0592
T 1007
T 1463
T 1347
T 0362
T 1129
T 1375
T 1623
T 1955
T 0760
T 2026
T 0999
T 1910
T 0134
T 0809
T 1960
T 1219
T 1766
T 1420
T 0316
T 0312
T 0384
T 0607
T 1737
T 1417
T 0413
T 1242
T 1304
T 1338
T 1485
T 0728
T 0097
T 0606
T 1645
T 0565
T 1865
T 1277
T 1757
T 1758
T 0416
T 1067
T 1616
T 1860
T 0569
T 0359
T 0587
T 0915
T 1540
T 1539
T 0553
T 1167
T 0786
T 0630
T 0829
T 0139
T 0140
T 0718
T 1729
T 1723
T 1561
T 0564
T 1028
T 1358
T 1487
T 1886
T 1949
T 0178
T 1807
T 1133
T 1525
T 1548
T 0126
T 1408
T 1428
T 1357
T 1712
T 1582
T 0654
T 1926
T 1726
T 0095
T 0092
T 0093
T 0743
T 1246
T 0817
T 1315
T 0661
T 1042
T 1396
T 1388
T 1387
T 1394
T 0596
T 0864
T 1053
T 2024
T 0620
T 0525
T 0496
T 0535
T 0102
T 0621
T 2029
T 1897
T 0712
T 1913
T 0657
T 1195
T 1239
T 0835
T 2035
T 2018
T 1363
T 0181
T 0113
T 0561
T 1733
T 1564
T 0904
T 0905
T 1781
T 0083
T 0109
T 0924
T 1256
T 0497
T 0523
T 0529
T 0513
T 0388
T 0389
T 0385
T 0301
T 1262
T 0926
T 1688
T 0328
T 0412
T 1149
T 0913
T 1530
T 1621
T 0441
T 0892
T 0133
T 0138
T 0131
T 0106
T 0966
T 1411

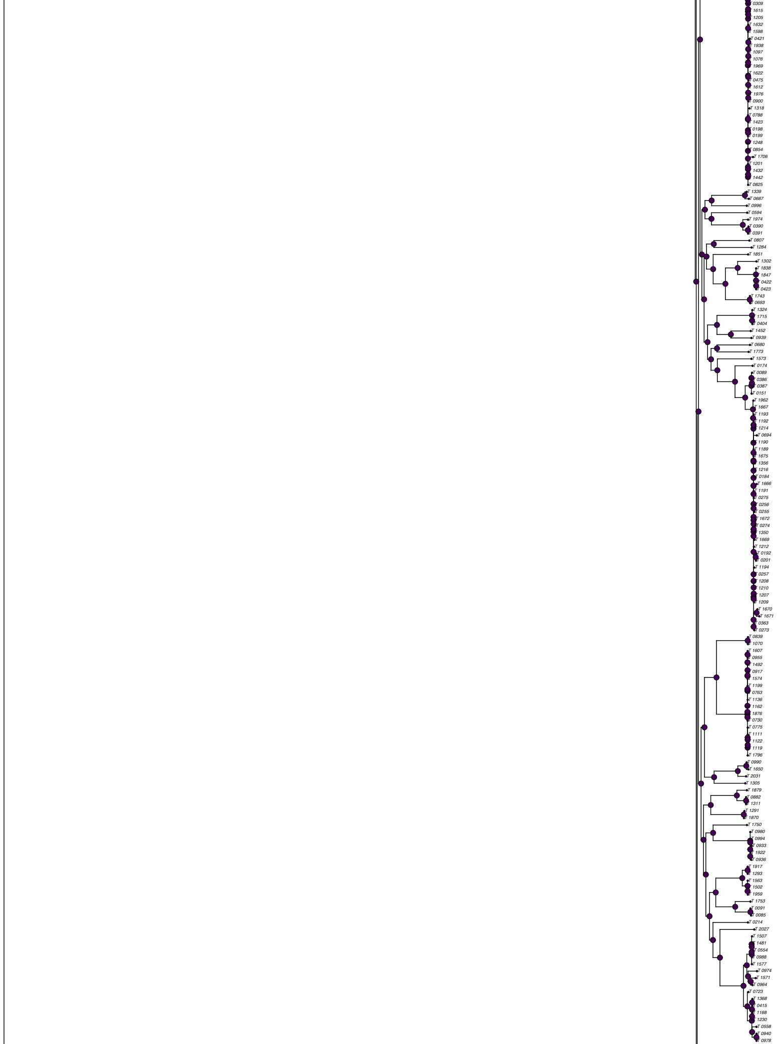

T 0309
T 1615
T 1205
T 1632
T 1598
T 0421
T 1938
T 1097
T 1076
T 1969
T 1622
T 0475
T 1612
T 1976
T 0900
T 1318
T 0788
T 1423
T 0198
T 0199
T 1248
T 0854
T 1706
T 1201
T 1432
T 1442
T 0825
T 1339
T 0687
T 0996
T 0594
T 1974
T 0390
T 0391
T 0807
T 1284
T 1851
T 1302
T 1838
T 1847
T 0422
T 0423
T 1743
T 0693
T 1324
T 1715
T 0404
T 1452
T 0939
T 0680
T 1773
T 1573
T 0174
T 0089
T 0386
T 0387
T 0151
T 1962
T 1667
T 1193
T 1192
T 1214
T 0694
T 1190
T 1189
T 1675
T 1356
T 1216
T 0184
T 1666
T 1191
T 0275
T 0256
T 0255
T 1672
T 0274
T 1350
T 1669
T 1212
T 0192
T 0201
T 1194
T 0257
T 1208
T 1210
T 1207
T 1209
T 1670
T 1671
T 0363
T 0273
T 0839
T 1070
T 1607
T 0955
T 1492
T 0917
T 1574
T 1199
T 0763
T 1136
T 1162
T 1876
T 0730
T 0775
T 1111
T 1122
T 1119
T 1796
T 0990
T 1650
T 2031
T 1305
T 1879
T 0882
T 1311
T 1291
T 1870
T 1750
T 0980
T 0994
T 0933
T 1922
T 0936
T 1917
T 1293
T 1563
T 1502
T 1959
T 1753
T 0091
T 0085
T 0214
T 2027
T 1507
T 1481
T 0554
T 0988
T 1577
T 0974
T 1571
T 0964
T 0723
T 1368
T 0415
T 1168
T 1230
T 0558
T 0940
T 0978

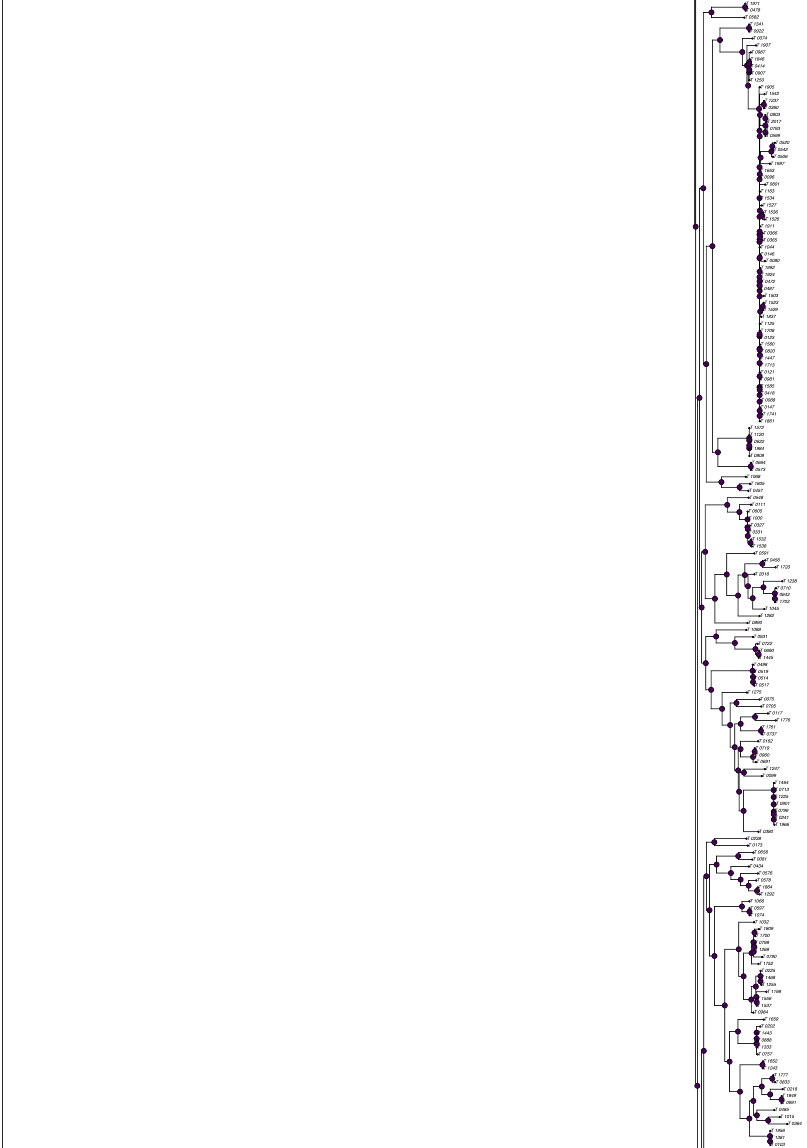
T 1971
T 0478
T 0582
T 1341
0922
T 0074
T 1907
T 0987
T 1846
0414
T 0907
T 1250
T 1905
T 1542
T 1237
0360
T 0803
T 2017
T 0793
0599
T 0520
0542
T 0506
T 1997
1653
0096
T 0801
T 1163
1534
T 1527
T 1536
T 1526
T 1911
T 0366
T 0365
T 1044
T 0146
T 0080
T 1992
1924
0472
0487
T 1503
T 1523
1529
T 1837
T 1125
T 1708
0123
T 1560
0820
T 1447
1713
T 0121
0981
1585
0418
T 0088
T 0147
T 1741
T 1861
T 1572
T 1120
0622
1984
T 0808
T 0664
0573
T 1068
T 1805
T 0457
T 0548
T 0111
T 0605
1000
T 0327
0331
T 1532
T 1538
T 0591
T 0456
T 1720
T 2016
T 1238
T 0710
0643
T 1703
T 1045
T 1282
T 0890
T 1088
T 0931
T 0722
T 0690
T 1445
T 0498
0519
0514
T 0517
T 1275
T 0075
T 0705
T 0117
T 1776
T 1761
T 0737
T 0162
T 0719
0960
T 0691
T 1247
T 0099
T 1464
0713
1225
0901
0799
0241
T 1966
T 0380
T 0238
T 0173
T 0656
T 0081
T 0434
T 0576
T 0578
T 1864
T 1292
T 1066
T 0597
1074
T 1032
T 1809
T 1700
0798
1268
T 0790
T 1752
T 0225
1468
T 1255
T 1198
1556
1537
T 0984
T 1659
T 0202
1443
0888
1333
T 0757
T 1652
T 1243
T 1777
T 0833
T 0218
T 1849
0991
T 0485
T 1015
T 0364
T 1956
1381
T 0103

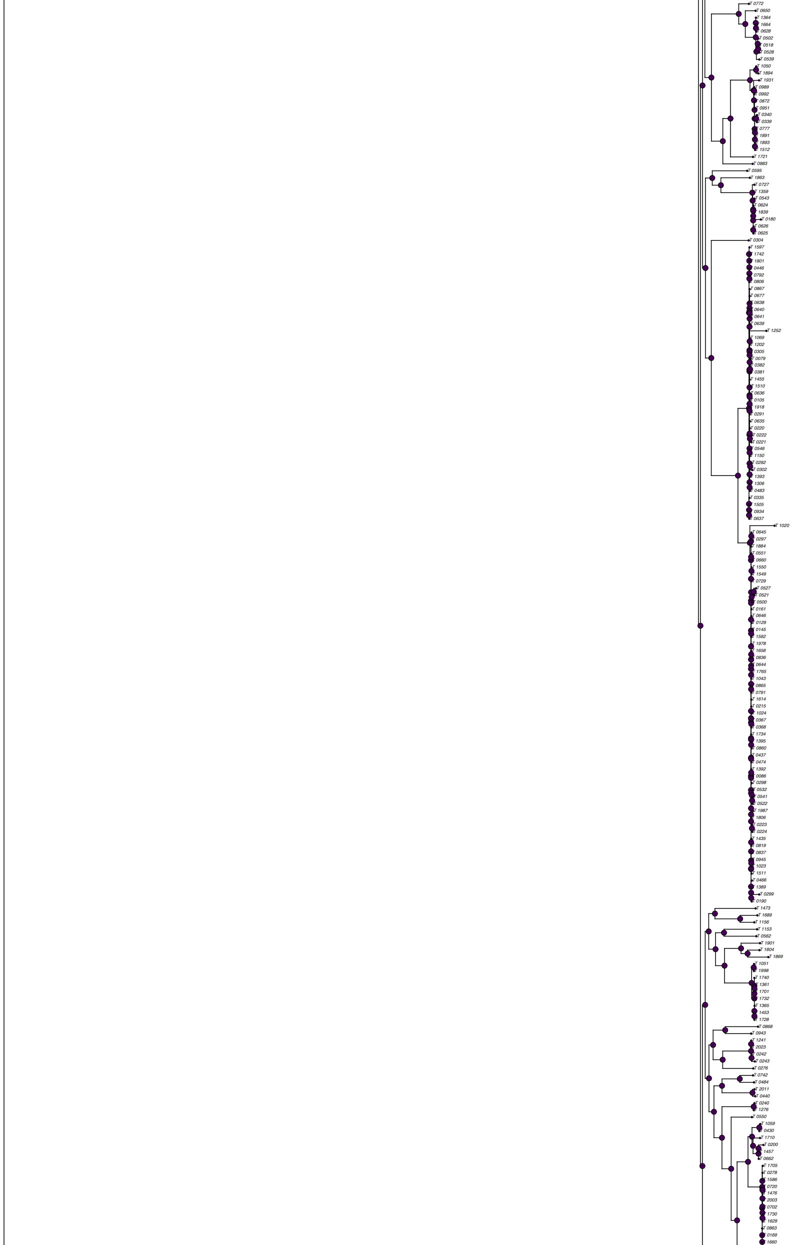
T 0772
T 0650
T 1364
1664
0628
T 0502
0518
T 0528
T 0539
T 1050
T 1894
T 1931
T 0989
0992
0872
T 0951
T 0340
T 0339
0777
1891
1893
T 1512
T 1721
T 0983
T 0595
T 1863
T 0727
T 1359
T 0543
T 0624
1839
T 0180
T 0626
T 0625
T 0304
T 1597
1742
1801
0446
0792
T 0806
T 0867
T 0677
0638
0640
0641
T 0639
T 1252
T 1069
T 1202
0305
T 0079
0382
0381
T 1455
1510
T 0636
0105
1918
T 0291
T 0635
T 0220
T 0222
T 0221
0546
T 1150
T 0292
T 0302
1393
1306
T 0483
T 0335
1505
0934
T 0637
T 1020
T 0645
0297
1884
T 0551
0660
T 1550
1549
0729
T 0527
T 0521
0500
T 0161
0646
0129
T 0145
T 1592
T 1978
1658
0836
0644
T 1765
T 1043
0865
T 0791
T 1614
T 0215
1024
0367
T 0368
T 1734
1395
T 0860
0437
T 0474
T 1392
0086
T 0298
T 0532
T 0541
T 0522
T 1987
1806
T 0223
T 0224
T 1435
T 0819
T 0837
0945
1023
T 1511
T 0466
1389
T 0299
T 0190
T 1473
T 1689
T 1156
T 1153
T 0562
T 1901
T 1804
T 1869
T 1051
T 1998
T 1740
1361
1701
1732
T 1365
1453
T 1728
T 0868
T 0943
T 1241
2023
0242
T 0243
T 0276
T 0742
T 0484
T 2011
T 0440
T 0240
T 1276
T 0550
T 1059
0430
T 1710
T 0200
1457
T 0662
T 1705
T 0278
1586
0720
T 1476
2003
0702
1730
T 1629
T 0863
0169
1660

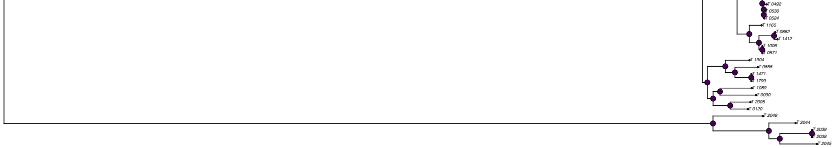
T 0492
T 0530
T 0524
T 1165
T 0862
T 1412
T 1006
T 0571
T 1904
T 0555
T 1471
T 1799
T 1089
T 0090
T 2005
T 0120
T 2048
T 2044
T 2039
T 2038
T 2045

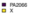

Effector gene: PA2066

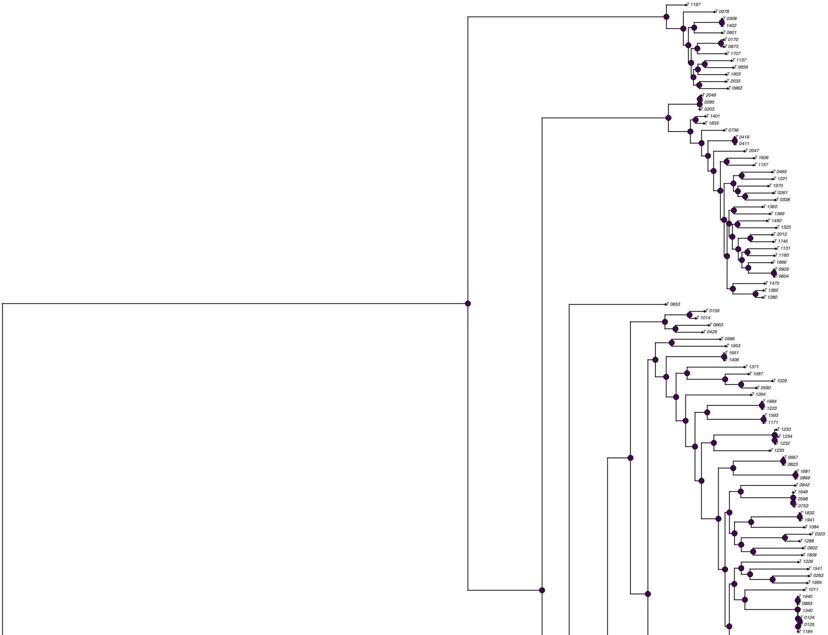

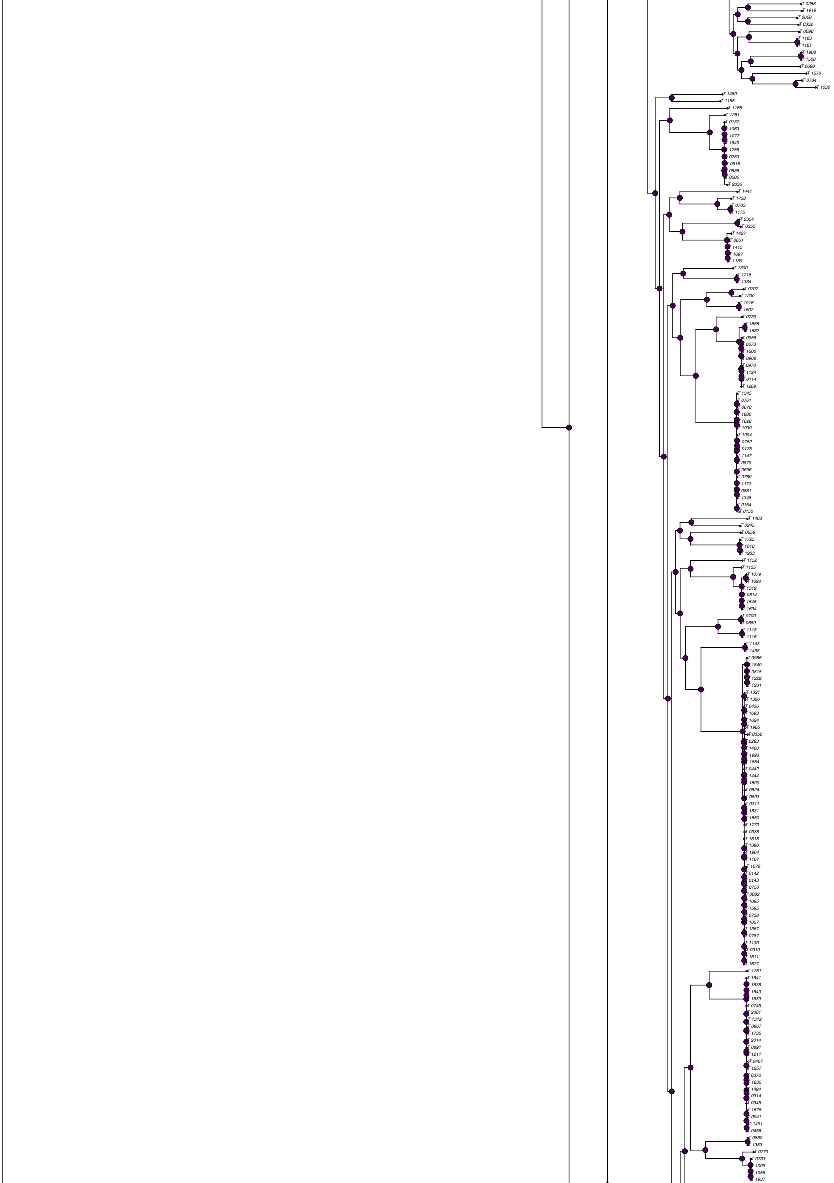
T 0258
T 1919
T 0668
T 0332
T 0098
T 1183
T 1181
T 1908
T 1508
T 0698
T 1570
T 0784
T 1030
T 1480
T 1155
T 1166
T 1261
T 0137
1063
1077
1649
1258
0253
0515
0538
0505
T 2036
T 1441
T 1738
T 0703
T 1175
T 0354
T 0355
T 1427
T 0651
1415
1697
1139
T 1300
T 1218
T 1334
T 0707
T 1200
T 1916
T 1602
T 0739
T 1858
T 1682
T 0956
0673
1600
0968
0970
1124
0114
T 1269
T 1345
T 0761
0670
1880
1628
1939
1994
0752
0175
1147
0879
0696
0780
1173
0881
1348
0154
T 0155
T 1403
T 0245
T 0658
T 1725
1010
1933
T 1152
T 1135
T 1079
T 1690
1319
0814
1646
1694
T 0700
T 0659
T 1118
T 1116
T 1143
1438
T 0986
1840
0815
1229
1231
T 1321
T 1326
0436
1693
1624
1985
T 0350
0293
1400
1993
1854
0442
1444
1590
0824
0893
0311
1831
1850
1770
0336
1618
1390
1964
1187
1078
0142
0143
0750
0082
1095
1595
0738
1057
1367
0787
1130
0910
1611
1627
T 1251
T 1641
1638
1640
1639
0745
2001
1312
0967
1735
2014
0891
1211
0467
1257
0376
1835
1494
0314
0345
1678
0941
1491
0458
T 0880
1383
T 0779
T 0733
1009
1056
1937

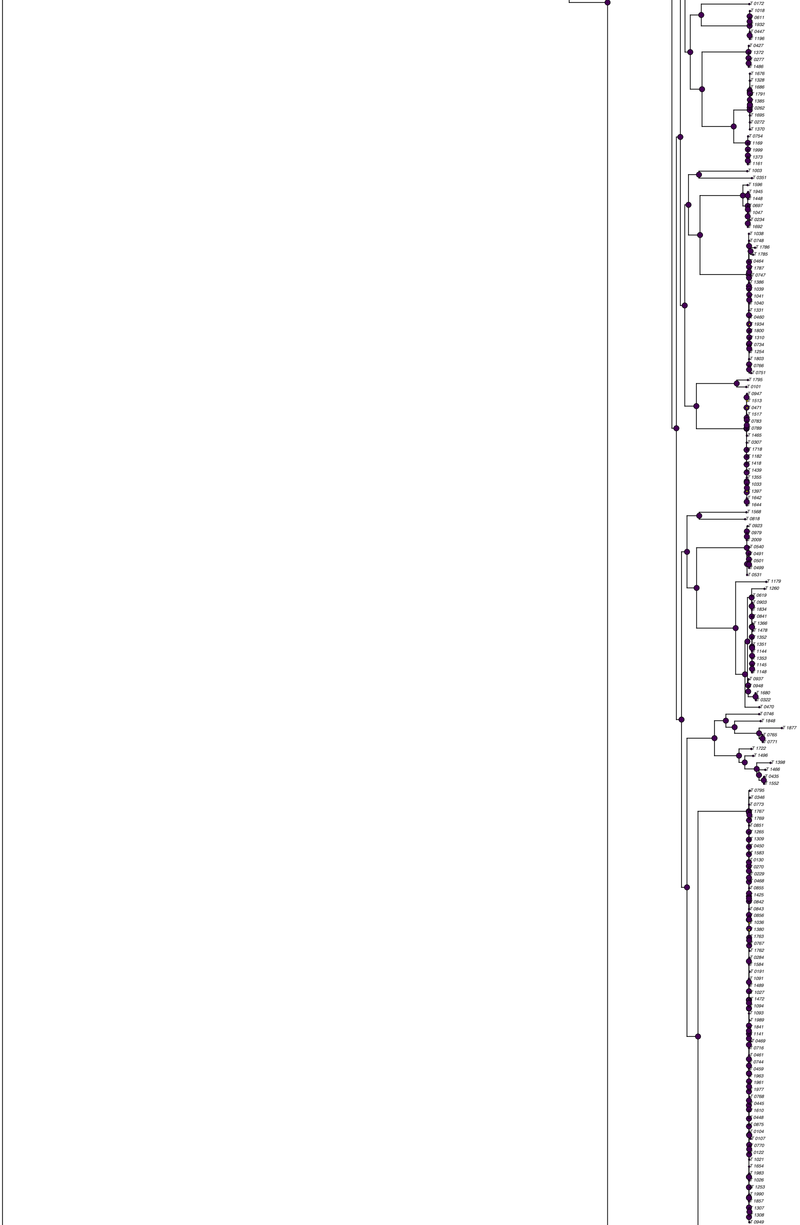

T 0172
T 1018
0611
1932
T 0447
T 1196
T 0427
1372
0277
T 1486
T 1676
T 1328
T 1686
1791
1385
0262
T 1695
T 0272
T 1370
T 0754
1169
1999
1373
T 1161
T 1003
T 0351
T 1596
T 1945
T 1448
0697
T 1047
T 0234
T 1692
T 1038
T 0748
T 1786
T 1785
0464
1787
T 0747
T 1386
1039
1041
1040
T 1331
0460
1934
1800
1310
0734
T 1254
T 1803
0766
T 0751
T 1795
T 0101
T 0947
T 1513
0471
T 1517
0783
0789
T 1465
T 0307
T 1718
T 1182
1418
T 1439
T 1355
1033
1397
T 1642
T 1644
T 1568
T 0818
T 0923
0979
T 2009
T 0540
0491
0501
T 0499
T 0531
T 1179
T 1260
0619
T 0903
T 1834
0841
T 1366
T 1478
1352
1351
1144
1353
1145
T 1148
T 0937
0948
T 1680
T 0322
T 0470
T 0746
T 1848
T 1877
T 0765
T 0771
T 1722
T 1496
T 1398
T 1466
T 0435
T 1552
T 0795
T 0346
T 0773
1767
1769
T 0851
1265
1309
0450
1583
0130
0270
T 0229
0468
T 0855
1425
0842
T 0843
0856
T 1036
1380
T 1763
0767
T 1762
T 0284
T 1584
T 0191
T 1091
T 1489
1027
T 1472
1094
T 1093
T 1989
1841
1141
T 0469
T 0716
T 0461
0744
0459
1963
1961
1977
T 0768
0445
1610
0448
T 0875
T 0104
T 0107
0770
0122
T 1021
T 1654
T 1983
1026
T 1253
T 1990
T 1857
T 1307
1308
T 0949

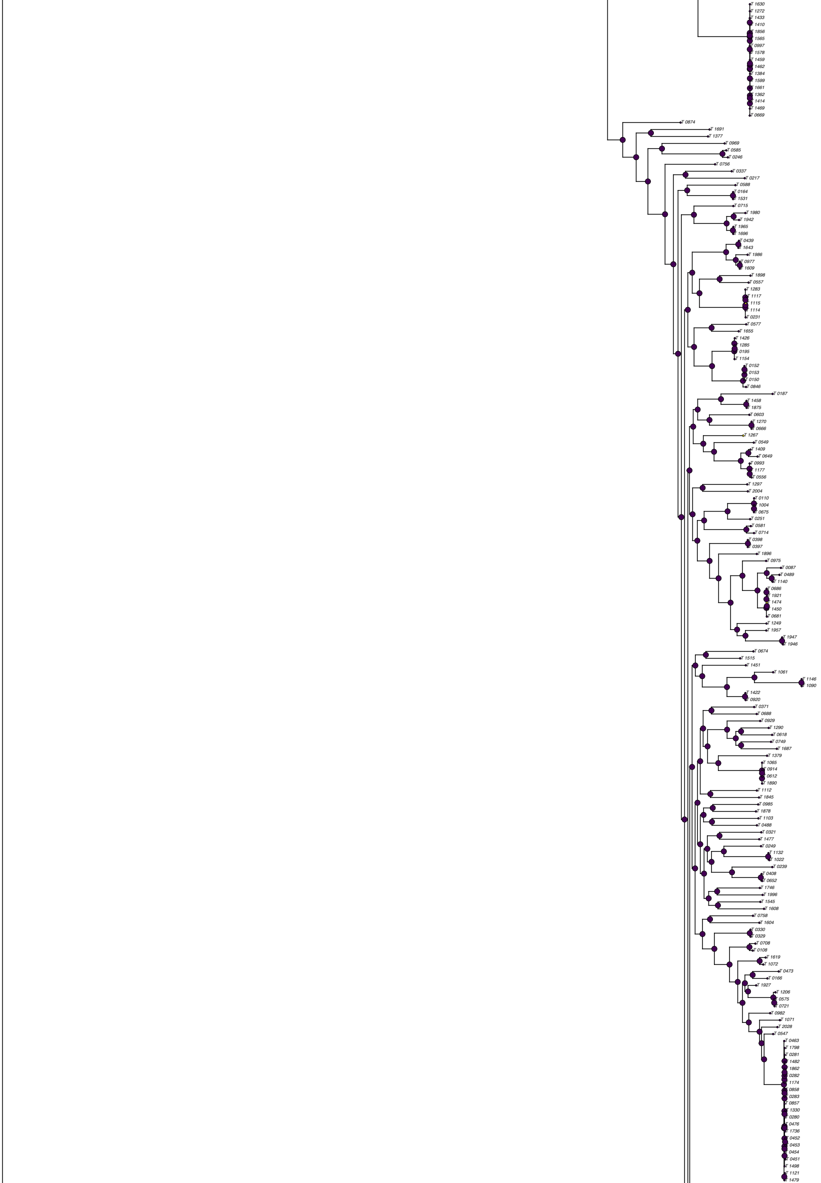
T 1630
T 1272
T 1433
T 1410
T 1856
T 1565
T 0997
T 1578
T 1459
T 1462
T 1384
T 1599
T 1661
T 1362
T 1414
T 1469
T 0669
T 0874
T 1691
T 1377
T 0969
T 0585
T 0246
T 0756
T 0337
T 0217
T 0588
T 0164
T 1531
T 0715
T 1980
T 1942
T 1965
T 1696
T 0439
T 1643
T 1986
T 0977
T 1609
T 1898
T 0557
T 1283
T 1117
T 1115
T 1114
T 0231
T 0577
T 1655
T 1426
T 1285
T 0195
T 1154
T 0152
T 0153
T 0150
T 0846
T 0187
T 1458
T 1875
T 0603
T 1270
T 0666
T 1267
T 0549
T 1409
T 0649
T 0993
T 1177
T 0556
T 1297
T 2004
T 0110
T 1004
T 0675
T 0251
T 0581
T 0714
T 0398
T 0397
T 1896
T 0975
T 0087
T 0489
T 1140
T 0686
T 1921
T 1474
T 1450
T 0681
T 1249
T 1957
T 1947
T 1946
T 0674
T 1515
T 1451
T 1061
T 1146
T 1090
T 1422
T 0920
T 0371
T 0688
T 0929
T 1290
T 0618
T 0749
T 1687
T 1379
T 1065
T 0914
T 0612
T 1890
T 1112
T 1845
T 0985
T 1878
T 1103
T 0488
T 0321
T 1477
T 0249
T 1132
T 1022
T 0239
T 0408
T 0652
T 1746
T 1996
T 1545
T 1608
T 0758
T 1604
T 0330
T 0329
T 0708
T 0108
T 1619
T 1072
T 0473
T 0166
T 1927
T 1206
T 0575
T 0721
T 0982
T 1071
T 2028
T 0547
T 0463
T 1798
T 0281
T 1482
T 1862
T 0282
T 1174
T 0858
T 0283
T 0857
T 1330
T 0280
T 0476
T 1736
T 0452
T 0453
T 0454
T 0451
T 1498
T 1121
T 1479

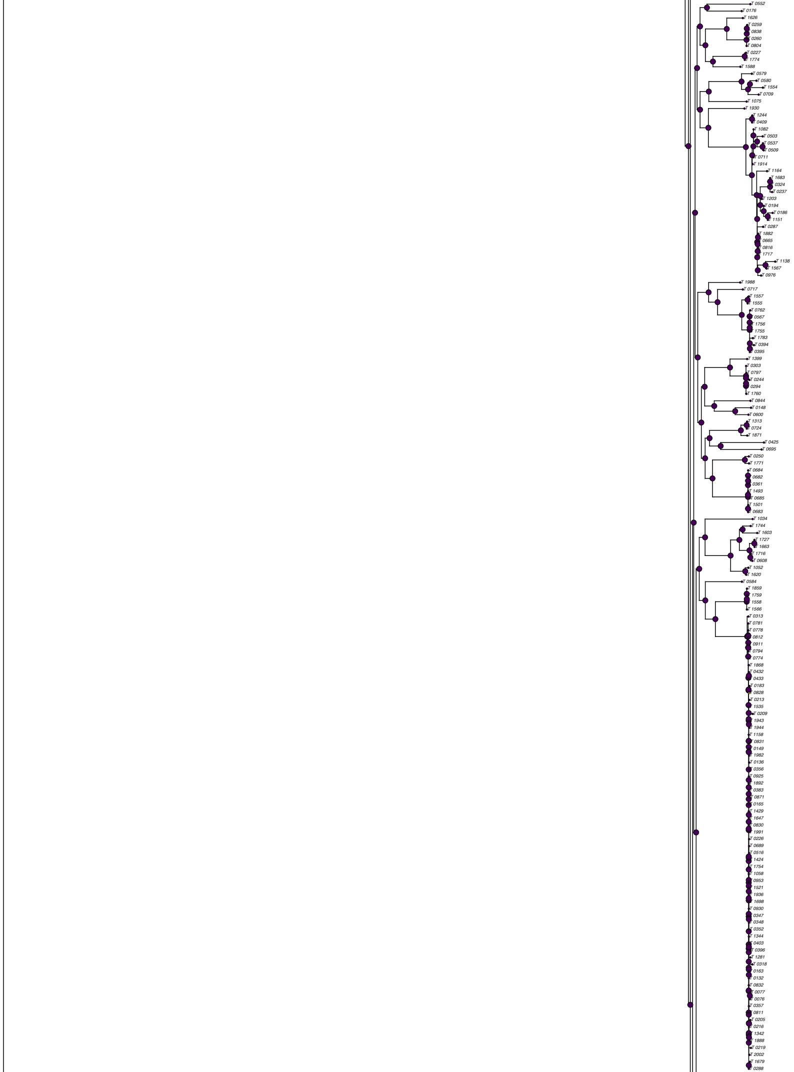

T 0552
T 0176
T 1626
T 0259
0838
0260
T 0804
T 0227
T 1774
T 1588
T 0579
T 0580
T 1554
T 0709
T 1075
T 1930
T 1244
T 0409
T 1082
T 0503
T 0537
T 0509
T 0711
T 1914
T 1164
T 1683
0324
T 0237
T 1203
T 0194
T 0186
T 1151
T 0287
T 1882
0665
T 0816
1717
T 1138
T 1567
T 0976
T 1988
T 0717
T 1557
T 1555
T 0762
0567
T 1756
1755
T 1783
T 0394
T 0395
T 1399
T 0303
T 0797
T 0244
0294
T 1760
T 0844
T 0148
T 0600
T 1313
T 0724
T 1871
T 0425
T 0695
T 0250
T 1771
T 0684
0682
0361
T 1493
T 0685
T 1501
T 0683
T 1034
T 1744
T 1603
T 1727
T 1663
T 1716
T 0608
T 1052
T 1620
T 0584
T 1859
1759
1558
T 1566
T 0313
T 0781
T 0778
0812
0911
T 0794
0774
T 1868
T 0432
0433
T 0183
T 0828
T 0213
1535
T 0209
1943
T 1944
T 1158
0831
0149
T 1982
T 0136
0356
T 0925
T 1892
0383
T 0871
0165
T 1429
T 1647
T 0830
1991
T 0226
T 0689
T 0516
1424
T 1754
T 1058
0953
1521
T 1936
1698
T 0930
0347
T 0348
T 0352
T 1344
0403
T 0396
T 1281
T 0318
0163
T 0132
T 0832
T 0077
T 0076
T 0357
0811
T 0205
T 0216
1342
T 1888
T 0219
T 2002
T 1679
T 0288

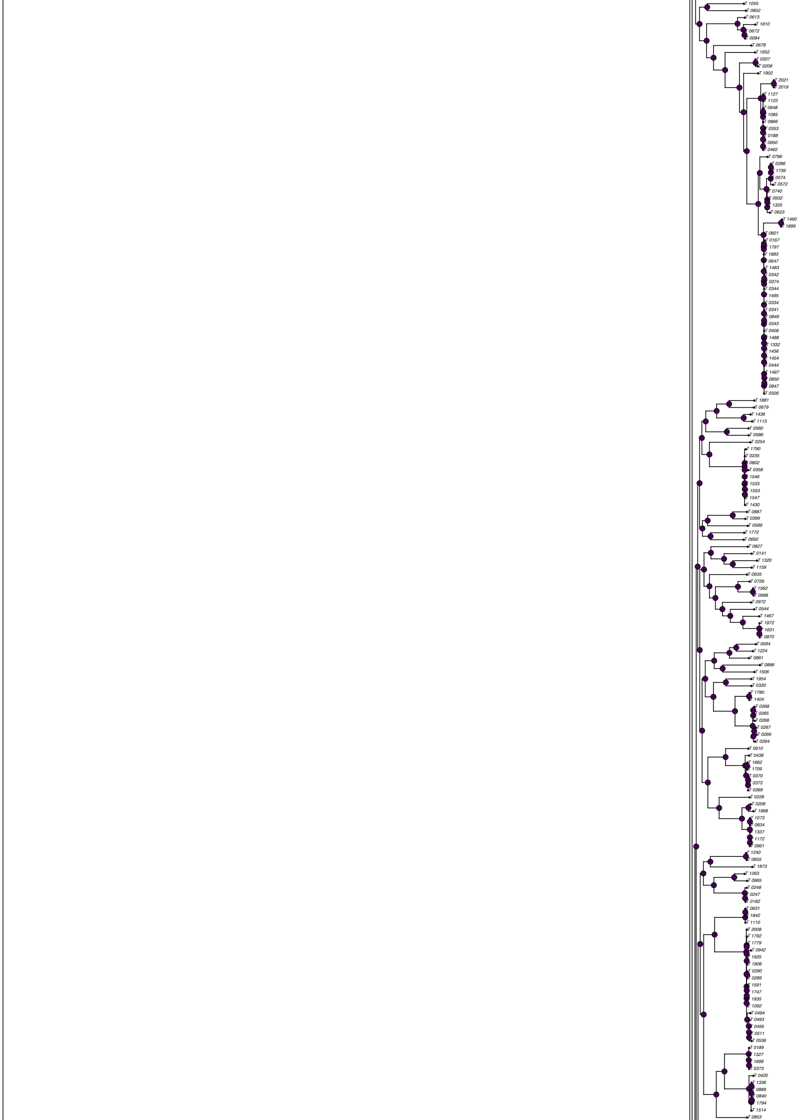
T 1055
T 0852
T 0613
T 1810
T 0672
T 0084
T 0678
T 1952
T 0207
T 0208
T 1902
T 2021
T 2019
T 1127
T 1123
T 0648
T 1085
T 0866
T 0353
T 0188
T 0950
T 0462
T 0796
T 0286
T 1739
T 0574
T 0572
T 0740
T 0932
T 1325
T 0623
T 1460
T 1899
T 0821
T 0167
T 1797
T 1883
T 0647
T 1483
T 0342
T 0374
T 0344
T 1495
T 0334
T 0341
T 0849
T 0343
T 0406
T 1488
T 1332
T 1456
T 1454
T 0444
T 1497
T 0850
T 0847
T 0326
T 1881
T 0679
T 1436
T 1113
T 0560
T 0586
T 0254
T 1790
T 0235
T 0802
T 0358
T 1546
T 1533
T 1553
T 1547
T 1430
T 0887
T 0399
T 0589
T 1772
T 0692
T 0827
T 0141
T 1320
T 1159
T 0935
T 0726
T 1562
T 0998
T 0972
T 0544
T 1467
T 1972
T 1631
T 0870
T 0094
T 1224
T 0861
T 0886
T 1506
T 1954
T 0320
T 1780
T 1404
T 0268
T 0265
T 0266
T 0267
T 0269
T 0264
T 0610
T 0438
T 1662
T 1709
T 0370
T 0372
T 0369
T 0228
T 0206
T 1968
T 1073
T 0834
T 1337
T 1172
T 0961
T 1240
T 0655
T 1873
T 1263
T 0965
T 0248
T 0247
T 0182
T 0631
T 1842
T 1110
T 2008
T 1792
T 1779
T 0942
T 1925
T 1906
T 0290
T 0289
T 1591
T 1747
T 1935
T 1092
T 0494
T 0493
T 0495
T 0511
T 0536
T 0189
T 1327
T 1699
T 0373
T 0420
T 1336
T 0889
T 0840
T 1794
T 1514
T 0853

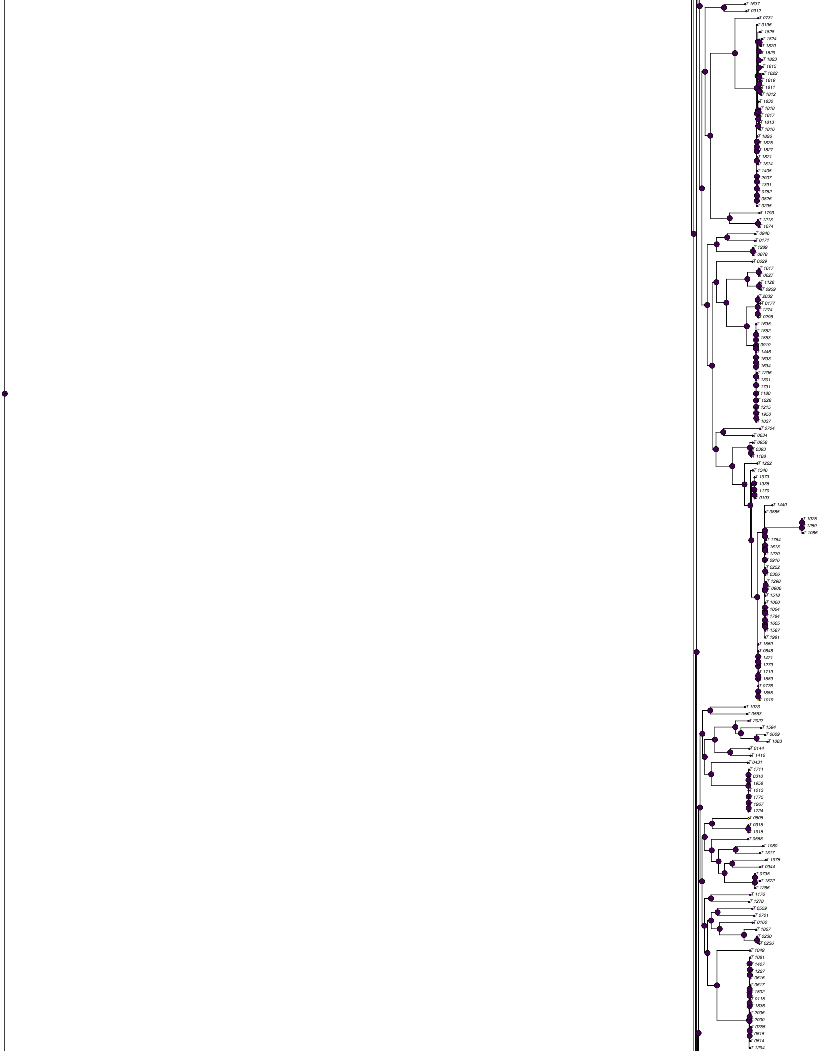
T 1637
T 0912
T 0731
T 0196
T 1828
T 1824
T 1820
T 1829
T 1823
T 1815
T 1822
T 1819
T 1811
T 1812
T 1830
T 1818
T 1817
T 1813
T 1816
T 1826
T 1825
T 1827
T 1821
T 1814
T 1405
2007
1391
0782
0826
T 0295
T 1793
T 1213
T 1674
T 0946
T 0171
T 1289
T 0878
T 0629
T 1617
T 0627
T 1128
T 0959
T 2032
T 0177
T 1274
T 0296
T 1635
T 1852
1853
0919
T 1446
1633
1634
T 1296
T 1301
1731
1180
1228
1215
1950
T 1037
T 0704
T 0634
T 0958
T 0393
T 1188
T 1222
T 1346
T 1973
1335
1170
T 0193
T 1440
T 0885
T 1025
1259
T 1086
T 1764
1613
T 1220
0916
T 0252
T 0306
T 1298
T 0906
T 1518
T 1060
1064
T 1784
1605
T 1587
T 1981
T 1569
T 0848
1421
1279
T 1719
1589
T 0776
1885
T 1019
T 1923
T 0563
T 2022
T 1594
T 0609
T 1083
T 0144
T 1416
T 0431
T 1711
0310
1958
T 1013
1775
1967
T 1724
T 0805
T 0315
T 1915
T 0568
T 1080
T 1317
T 1975
T 0944
T 0735
T 1872
T 1266
T 1176
T 1278
T 0559
T 0701
T 0160
T 1867
T 0230
T 0236
T 1049
T 1081
1407
1227
0616
T 0617
1802
T 0115
1836
T 2006
2000
T 0755
0615
T 0614
T 1294

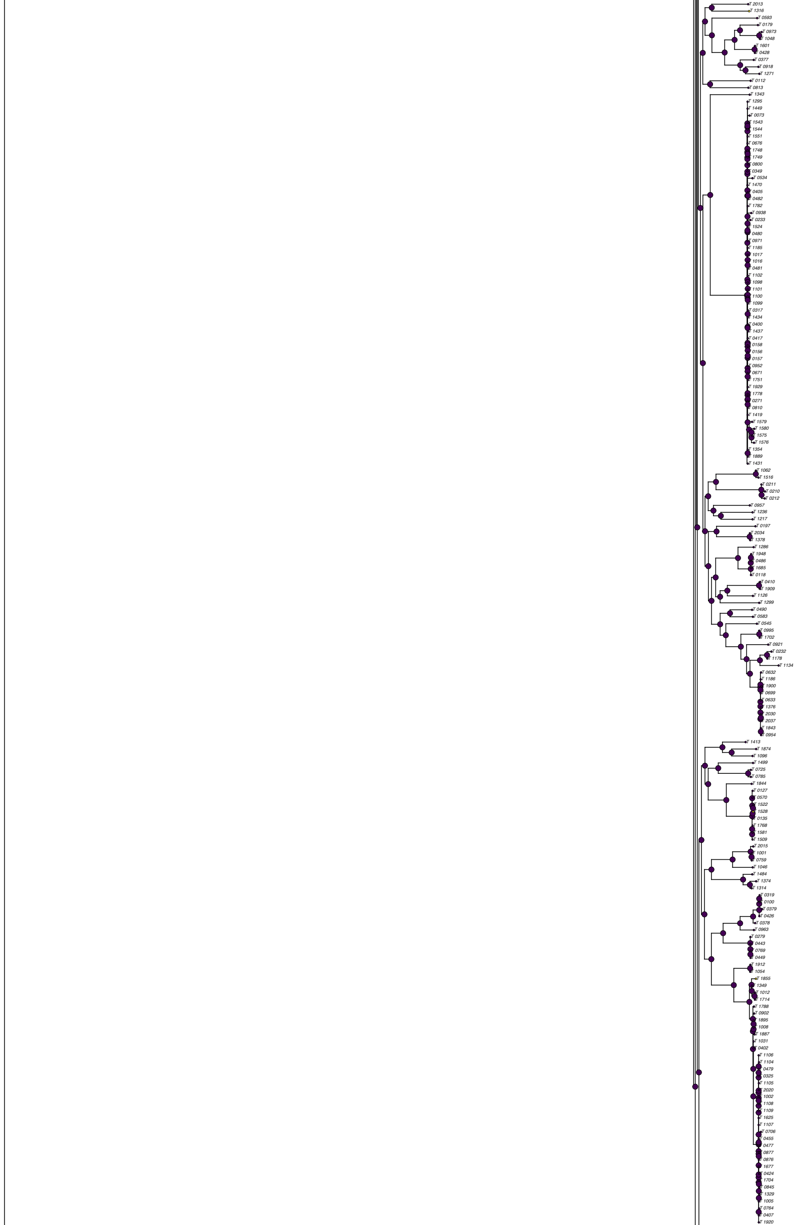
T 2013
T 1316
T 0593
T 0179
T 0973
T 1048
T 1601
T 0428
T 0377
T 0918
T 1271
T 0112
T 0813
T 1343
T 1295
T 1449
T 0073
T 1543
T 1544
T 1551
T 0676
T 1748
T 1749
T 0800
T 0349
T 0534
T 1470
T 0405
T 0482
T 1782
T 0938
T 0233
T 1524
T 0480
T 0971
T 1185
T 1017
T 1016
T 0481
T 1102
T 1098
T 1101
T 1100
T 1099
T 0317
T 1434
T 0400
T 1437
T 0417
T 0158
T 0156
T 0157
T 0952
T 0671
T 1751
T 1929
T 1778
T 0271
T 0810
T 1419
T 1579
T 1580
T 1575
T 1576
T 1354
T 1889
T 1431
T 1062
T 1516
T 0211
T 0210
T 0212
T 0957
T 1236
T 1217
T 0197
T 2034
T 1378
T 1286
T 1948
T 0486
T 1685
T 0118
T 0410
T 1909
T 1126
T 1299
T 0490
T 0583
T 0545
T 0995
T 1702
T 0921
T 0232
T 1178
T 1134
T 0632
T 1186
T 1900
T 0699
T 0633
T 1376
T 2030
T 2037
T 1843
T 0954
T 1413
T 1874
T 1096
T 1499
T 0725
T 0785
T 1844
T 0127
T 0570
T 1522
T 1528
T 0135
T 1768
T 1581
T 1509
T 2015
T 1001
T 0759
T 1046
T 1484
T 1374
T 1314
T 0319
T 0100
T 0379
T 0426
T 0378
T 0963
T 0279
T 0443
T 0769
T 0449
T 1912
T 1054
T 1855
T 1349
T 1012
T 1714
T 1788
T 0902
T 1895
T 1008
T 1887
T 1031
T 0402
T 1106
T 1104
T 0479
T 0325
T 1105
T 2020
T 1002
T 1108
T 1109
T 1625
T 1107
T 0706
T 0455
T 0477
T 0877
T 0876
T 1677
T 0424
T 1704
T 0845
T 1329
T 1005
T 0764
T 0407
T 1920

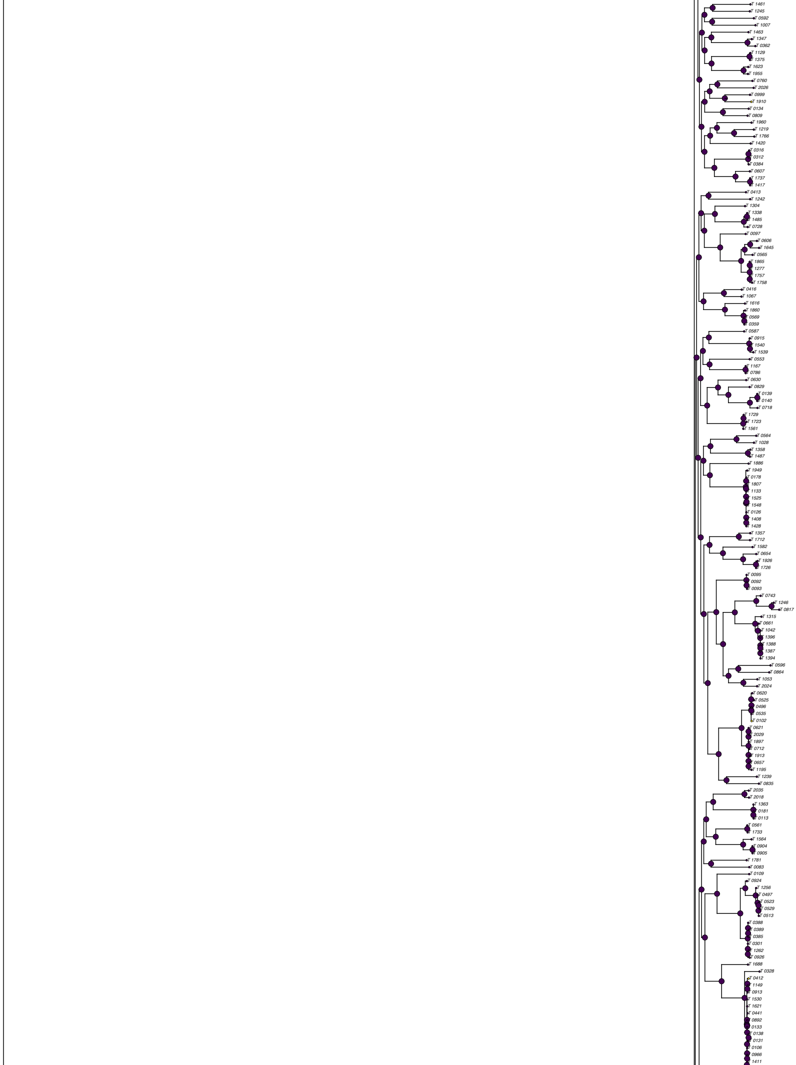
T 1461
T 1245
T 0592
T 1007
T 1463
T 1347
T 0362
T 1129
T 1375
T 1623
T 1955
T 0760
T 2026
T 0999
T 1910
T 0134
T 0809
T 1960
T 1219
T 1766
T 1420
T 0316
T 0312
T 0384
T 0607
T 1737
T 1417
T 0413
T 1242
T 1304
T 1338
T 1485
T 0728
T 0097
T 0606
T 1645
T 0565
T 1865
T 1277
T 1757
T 1758
T 0416
T 1067
T 1616
T 1860
T 0569
T 0359
T 0587
T 0915
T 1540
T 1539
T 0553
T 1167
T 0786
T 0630
T 0829
T 0139
T 0140
T 0718
T 1729
T 1723
T 1561
T 0564
T 1028
T 1358
T 1487
T 1886
T 1949
T 0178
1807
1133
1525
1548
T 0126
1408
T 1428
T 1357
T 1712
T 1582
T 0654
T 1926
T 1726
T 0095
0092
T 0093
T 0743
T 1246
T 0817
T 1315
T 0661
T 1042
1396
T 1388
1387
T 1394
T 0596
T 0864
T 1053
T 2024
T 0620
T 0525
0496
T 0535
T 0102
T 0621
2029
T 1897
T 0712
T 1913
T 0657
T 1195
T 1239
T 0835
T 2035
T 2018
T 1363
0181
0113
T 0561
T 1733
T 1564
T 0904
T 0905
T 1781
T 0083
T 0109
T 0924
T 1256
T 0497
T 0523
T 0529
T 0513
T 0388
T 0389
T 0385
T 0301
T 1262
T 0926
T 1688
T 0328
T 0412
T 1149
T 0913
T 1530
T 1621
T 0441
0892
0133
T 0138
T 0131
T 0106
0966
1411

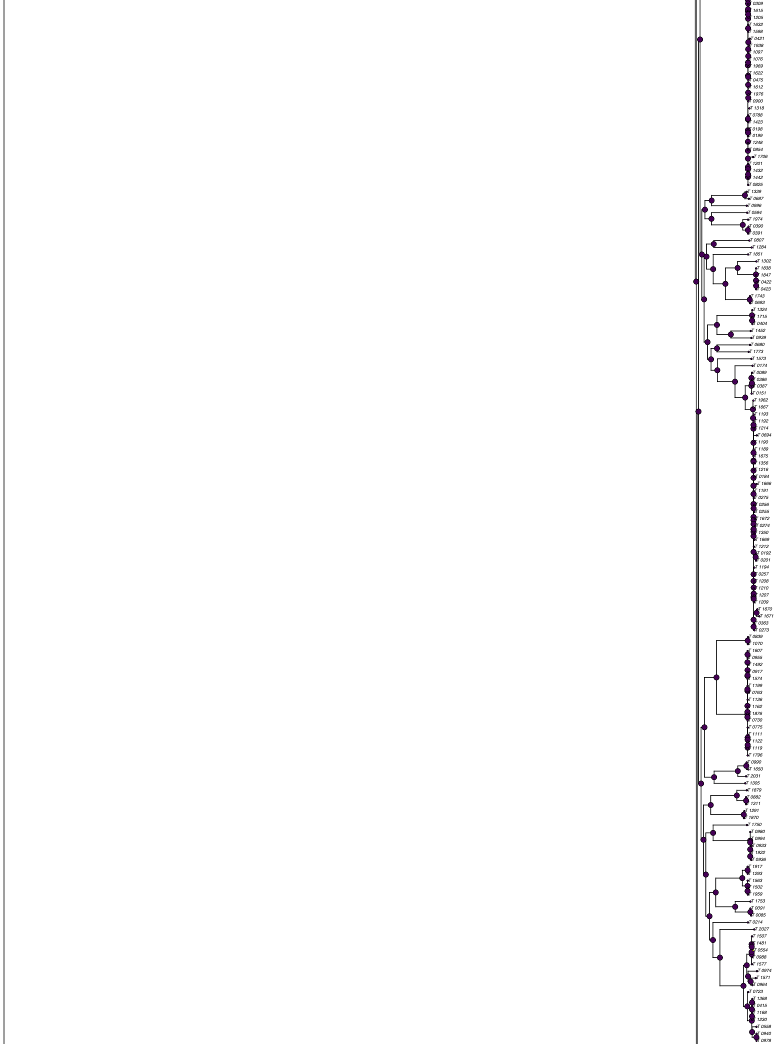
0309
1615
1205
1632
1598
0421
1938
1097
1076
1969
1622
0475
1612
1976
0900
T 1318
T 0788
1423
0198
0199
1248
0854
T 1706
T 1201
1432
1442
T 0825
T 1339
T 0687
T 0996
T 0594
T 1974
T 0390
T 0391
T 0807
T 1284
T 1851
T 1302
T 1838
1847
0422
T 0423
T 1743
T 0693
T 1324
1715
0404
T 1452
T 0939
T 0680
T 1773
T 1573
T 0174
T 0089
0386
0387
T 0151
T 1962
T 1667
T 1193
T 1192
1214
T 0694
1190
T 1189
T 1675
1356
1216
T 0184
T 1666
T 1191
T 0275
T 0256
T 0255
T 1672
T 0274
1350
T 1669
T 1212
T 0192
T 0201
T 1194
T 0257
T 1208
1210
T 1207
T 1209
T 1670
T 1671
T 0363
T 0273
T 0839
T 1070
T 1607
T 0955
T 1492
0917
T 1574
T 1199
0763
T 1136
1162
1876
T 0730
T 0775
T 1111
1122
1119
T 1796
T 0990
T 1650
T 2031
T 1305
T 1879
T 0882
T 1311
T 1291
T 1870
T 1750
T 0980
T 0994
T 0933
T 1922
T 0936
T 1917
T 1293
T 1563
1502
T 1959
T 1753
T 0091
T 0085
T 0214
T 2027
T 1507
1481
T 0554
T 0988
T 1577
T 0974
T 1571
T 0964
T 0723
T 1368
0415
1168
1230
T 0558
T 0940
T 0978

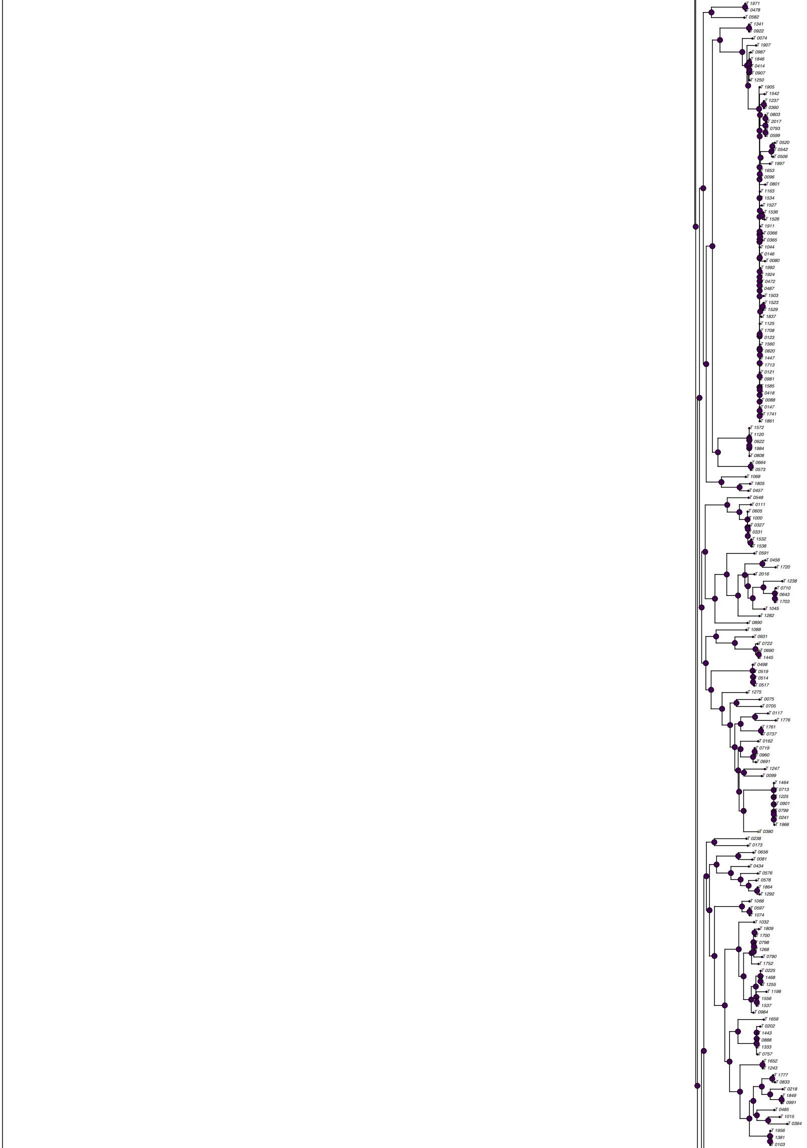
T 1971
T 0478
T 0582
T 1341
T 0922
T 0074
T 1907
T 0987
T 1846
T 0414
T 0907
T 1250
T 1905
T 1542
T 1237
T 0360
T 0803
T 2017
T 0793
T 0599
T 0520
T 0542
T 0506
T 1997
T 1653
T 0096
T 0801
T 1163
T 1534
T 1527
T 1536
T 1526
T 1911
T 0366
T 0365
T 1044
T 0146
T 0080
T 1992
T 1924
T 0472
T 0487
T 1503
T 1523
T 1529
T 1837
T 1125
T 1708
T 0123
T 1560
T 0820
T 1447
T 1713
T 0121
T 0981
T 1585
T 0418
T 0088
T 0147
T 1741
T 1861
T 1572
T 1120
T 0622
T 1984
T 0808
T 0664
T 0573
T 1068
T 1805
T 0457
T 0548
T 0111
T 0605
T 1000
T 0327
T 0331
T 1532
T 1538
T 0591
T 0456
T 1720
T 2016
T 1238
T 0710
T 0643
T 1703
T 1045
T 1282
T 0890
T 1088
T 0931
T 0722
T 0690
T 1445
T 0498
T 0519
T 0514
T 0517
T 1275
T 0075
T 0705
T 0117
T 1776
T 1761
T 0737
T 0162
T 0719
T 0960
T 0691
T 1247
T 0099
T 1464
T 0713
T 1225
T 0901
T 0799
T 0241
T 1966
T 0380
T 0238
T 0173
T 0656
T 0081
T 0434
T 0576
T 0578
T 1864
T 1292
T 1066
T 0597
T 1074
T 1032
T 1809
T 1700
T 0798
T 1268
T 0790
T 1752
T 0225
T 1468
T 1255
T 1198
T 1556
T 1537
T 0984
T 1659
T 0202
T 1443
T 0888
T 1333
T 0757
T 1652
T 1243
T 1777
T 0833
T 0218
T 1849
T 0991
T 0485
T 1015
T 0364
T 1956
T 1381
T 0103

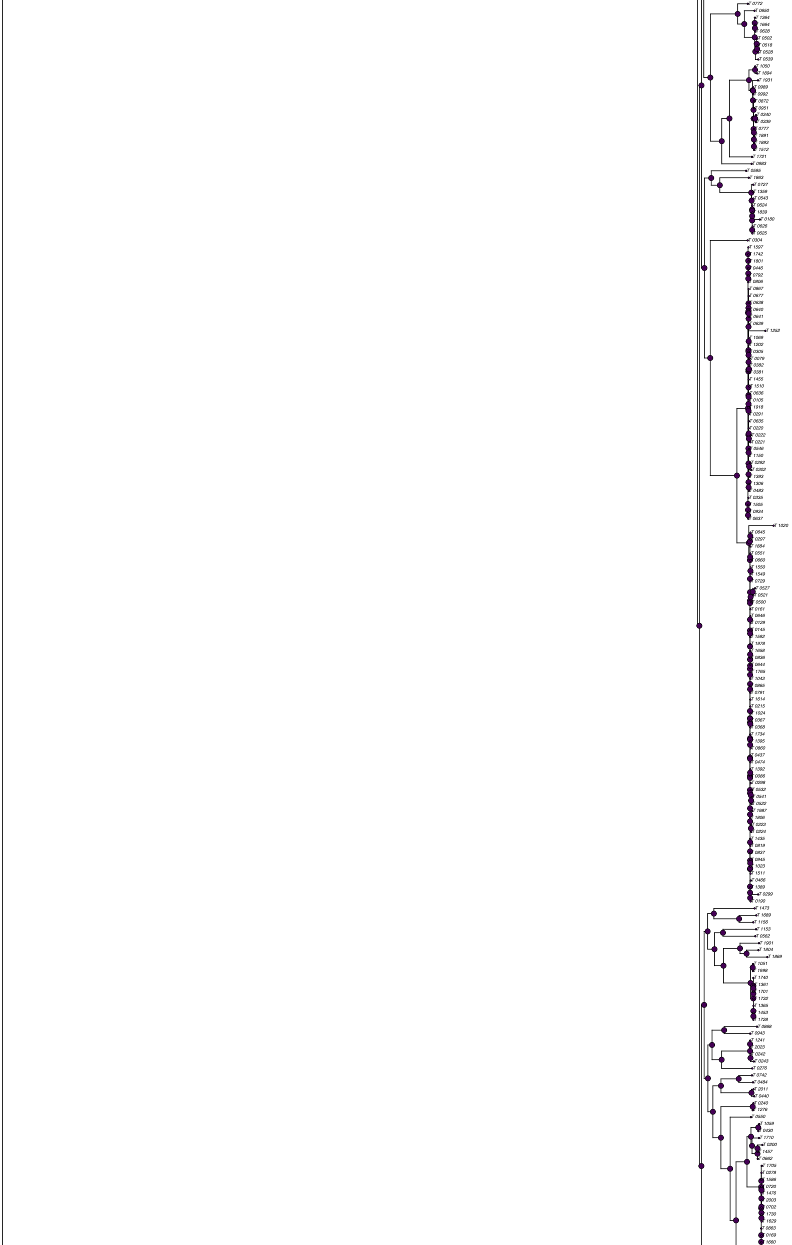
T 0772
T 0650
T 1364
1664
0628
T 0502
0518
T 0528
T 0539
T 1050
T 1894
T 1931
T 0989
0992
0872
T 0951
T 0340
T 0339
0777
1891
1893
1512
T 1721
T 0983
T 0595
T 1863
T 0727
T 1359
T 0543
T 0624
1839
T 0180
T 0626
T 0625
T 0304
T 1597
1742
1801
0446
0792
T 0806
T 0867
T 0677
0638
0640
0641
T 0639
T 1252
T 1069
T 1202
0305
T 0079
0382
0381
T 1455
1510
T 0636
0105
1918
T 0291
T 0635
T 0220
T 0222
T 0221
0546
T 1150
T 0292
T 0302
1393
1306
T 0483
T 0335
1505
0934
T 0637
T 1020
T 0645
0297
1884
T 0551
0660
T 1550
1549
0729
T 0527
T 0521
0500
T 0161
0646
0129
T 0145
T 1592
T 1978
T 1658
0836
0644
T 1765
T 1043
0865
T 0791
T 1614
T 0215
1024
0367
T 0368
T 1734
1395
T 0860
0437
T 0474
T 1392
0086
T 0298
T 0532
T 0541
T 0522
T 1987
1806
T 0223
T 0224
T 1435
T 0819
T 0837
0945
1023
T 1511
T 0466
1389
T 0299
T 0190
T 1473
T 1689
T 1156
T 1153
T 0562
T 1901
T 1804
T 1869
T 1051
T 1998
T 1740
1361
1701
1732
T 1365
1453
T 1728
T 0868
T 0943
T 1241
2023
0242
T 0243
T 0276
T 0742
T 0484
T 2011
T 0440
T 0240
T 1276
T 0550
T 1059
0430
T 1710
T 0200
1457
T 0662
T 1705
T 0278
1586
0720
T 1476
2003
0702
1730
T 1629
T 0863
0169
1660

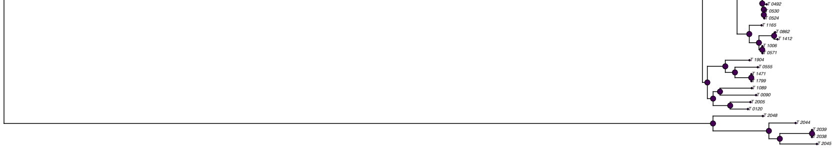
T 0492
T 0530
T 0524
T 1165
T 0862
T 1412
T 1006
T 0571
T 1904
T 0555
T 1471
T 1799
T 1089
T 0090
T 2005
T 0120
T 2048
T 2044
T 2039
T 2038
T 2045

Effector gene: pldB

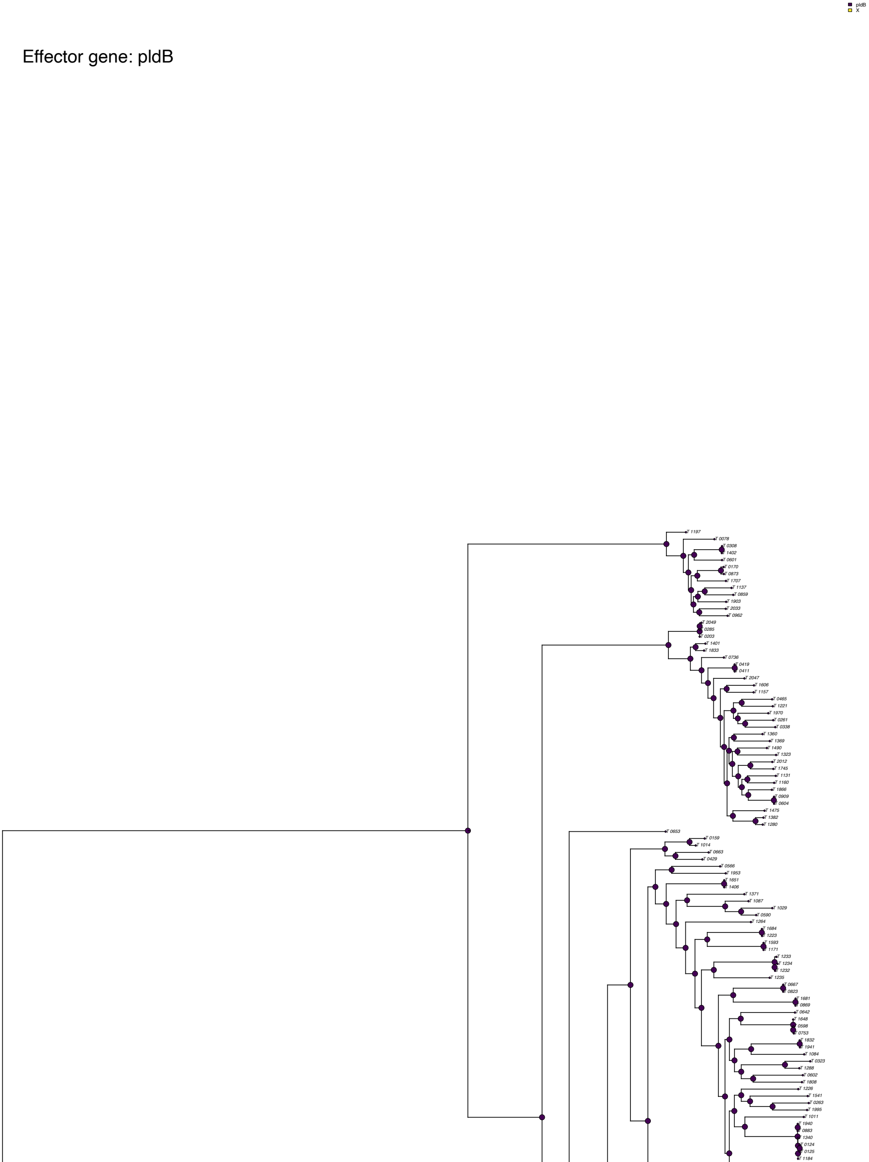

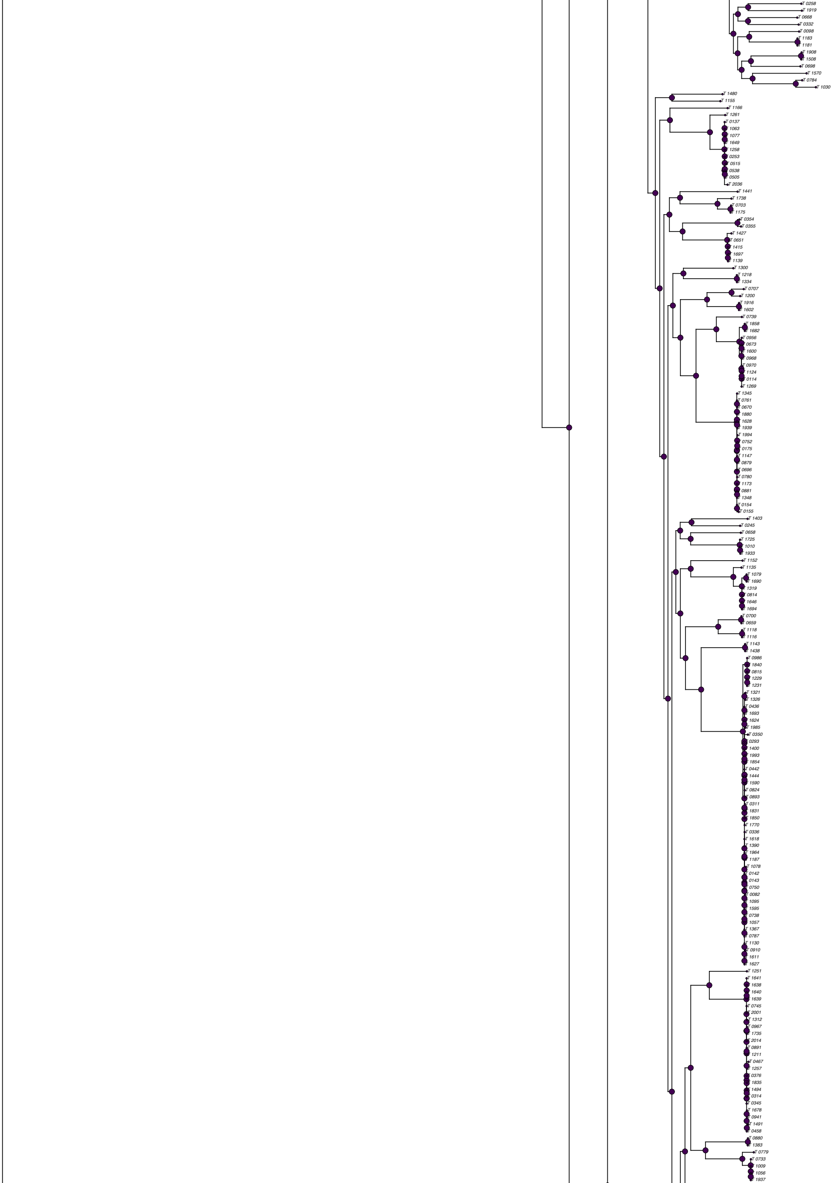
T 0258
T 1919
T 0668
T 0332
T 0098
T 1183
T 1181
T 1908
T 1508
T 0698
T 1570
T 0784
T 1030
T 1480
T 1155
T 1166
T 1261
T 0137
1063
1077
1649
1258
0253
0515
0538
0505
T 2036
T 1441
T 1738
T 0703
1175
T 0354
T 0355
T 1427
0651
1415
1697
1139
T 1300
T 1218
1334
T 0707
T 1200
T 1916
1602
T 0739
T 1858
1682
T 0956
0673
1600
0968
T 0970
1124
0114
T 1269
T 1345
T 0761
0670
1880
1628
1939
T 1994
0752
0175
T 1147
0879
0696
T 0780
1173
0881
1348
T 0154
T 0155
T 1403
T 0245
T 0658
T 1725
1010
1933
T 1152
T 1135
T 1079
1690
1319
0814
1646
1694
T 0700
0659
T 1118
1116
T 1143
1438
T 0986
1840
0815
1229
1231
T 1321
1326
0436
1693
1624
T 1985
T 0350
0293
1400
1993
1854
T 0442
1444
1590
T 0824
0893
T 0311
1831
1850
T 1770
T 0336
T 1618
T 1390
1964
1187
T 1078
0142
0143
0750
0082
1095
1595
0738
1057
T 1367
0787
T 1130
T 0910
1611
1627
T 1251
T 1641
1638
1640
1639
T 0745
2001
T 1312
T 0967
1735
2014
T 0891
1211
T 0467
1257
0376
1835
1494
0314
T 0345
T 1678
0941
T 1491
0458
T 0880
1383
T 0779
T 0733
1009
1056
1937

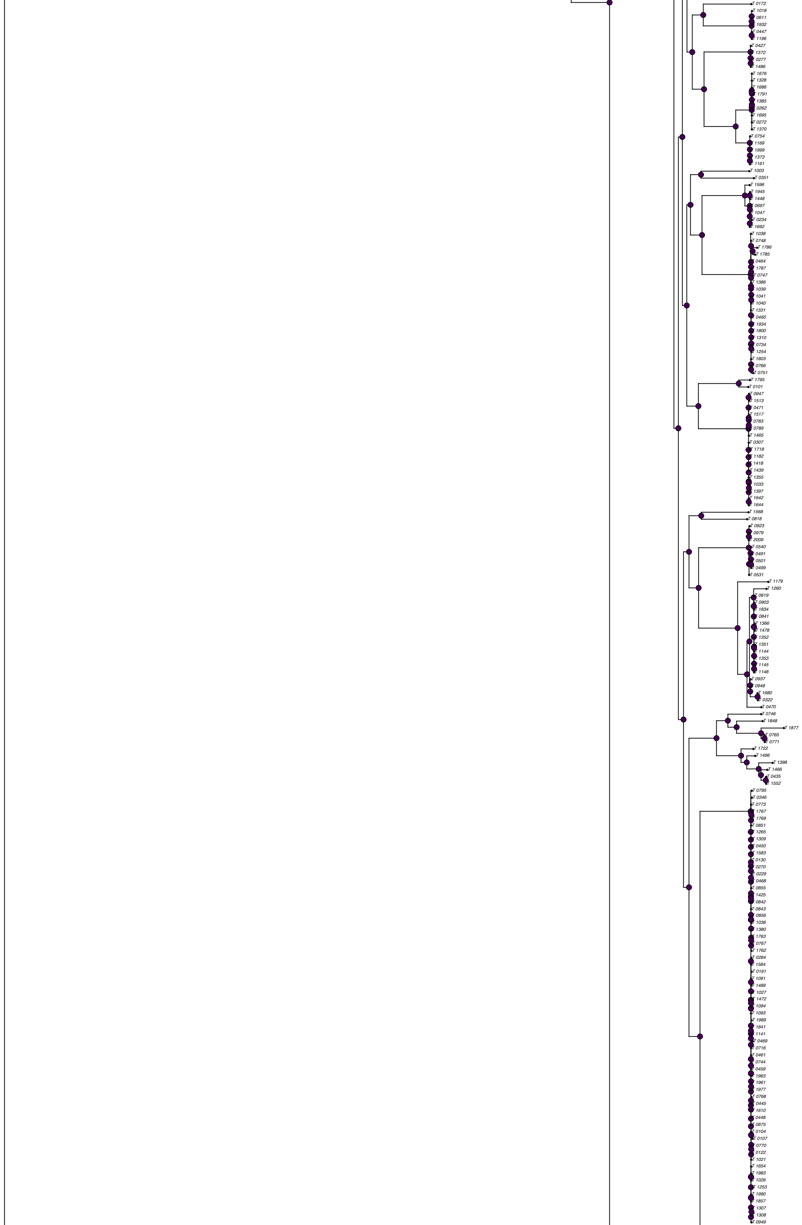
T 0172
T 1018
0611
1932
T 0447
T 1196
T 0427
1372
T 0277
T 1486
T 1676
T 1328
T 1686
1791
1385
0262
T 1695
T 0272
T 1370
T 0754
1169
1999
1373
T 1161
T 1003
T 0351
T 1596
T 1945
T 1448
0697
T 1047
T 0234
T 1692
T 1038
T 0748
T 1786
T 1785
0464
1787
T 0747
T 1386
1039
1041
1040
T 1331
0460
1934
1800
1310
0734
T 1254
T 1803
0766
T 0751
T 1795
T 0101
T 0947
T 1513
0471
T 1517
0783
0789
T 1465
T 0307
T 1718
T 1182
1418
T 1439
T 1355
1033
1397
T 1642
T 1644
T 1568
T 0818
T 0923
0979
T 2009
T 0540
0491
0501
T 0499
T 0531
T 1179
T 1260
0619
T 0903
T 1834
0841
T 1366
T 1478
1352
1351
1144
1353
1145
T 1148
T 0937
0948
T 1680
T 0322
T 0470
T 0746
T 1848
T 1877
T 0765
T 0771
T 1722
T 1496
T 1398
T 1466
T 0435
T 1552
T 0795
T 0346
T 0773
1767
1769
T 0851
1265
1309
0450
T 1583
T 0130
T 0270
T 0229
T 0468
T 0855
1425
0842
T 0843
0856
T 1036
1380
T 1763
0767
T 1762
T 0284
T 1584
T 0191
T 1091
T 1489
1027
T 1472
1094
T 1093
T 1989
1841
1141
T 0469
T 0716
T 0461
0744
T 0459
1963
1961
1977
T 0768
0445
1610
0448
T 0875
T 0104
T 0107
0770
0122
T 1021
T 1654
T 1983
1026
T 1253
T 1990
T 1857
T 1307
1308
T 0949

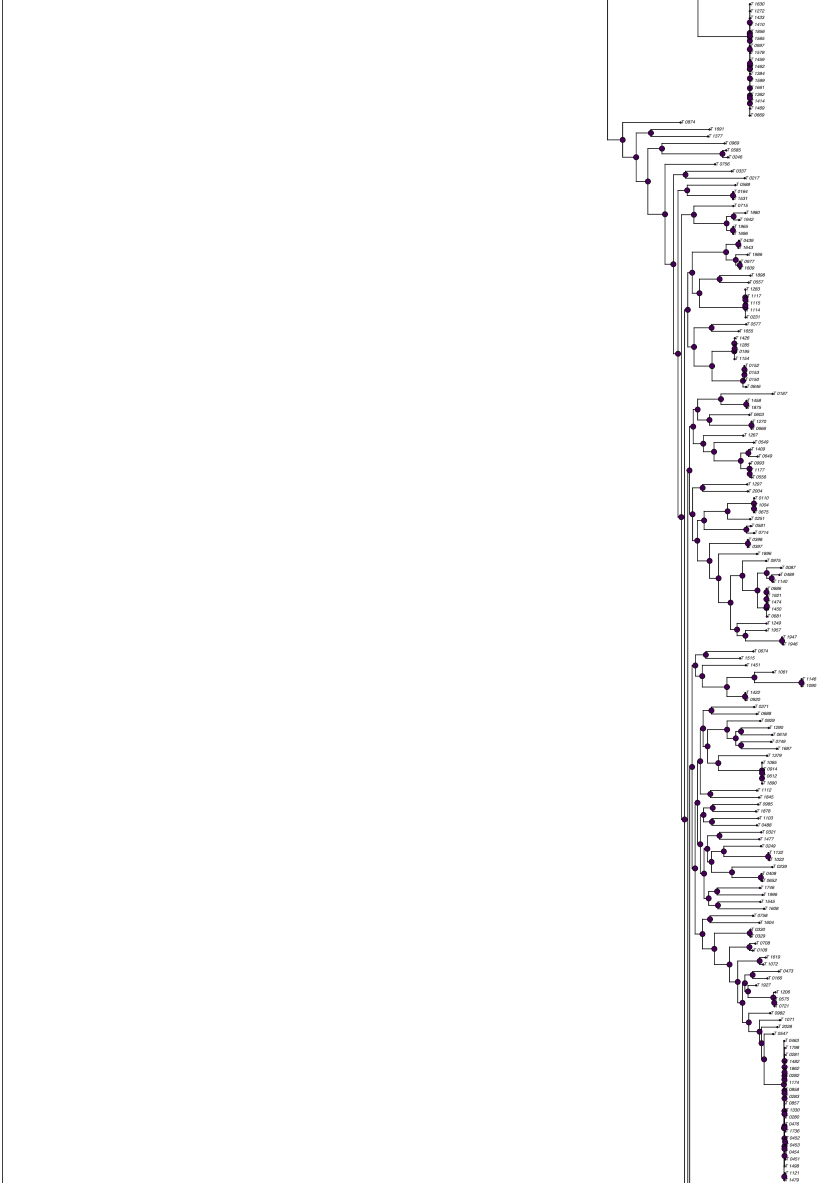

T 1630
T 1272
T 1433
T 1410
T 1856
T 1565
T 0997
T 1578
T 1459
T 1462
T 1384
T 1599
T 1661
T 1362
T 1414
T 1469
T 0669
T 0874
T 1691
T 1377
T 0969
T 0585
T 0246
T 0756
T 0337
T 0217
T 0588
T 0164
T 1531
T 0715
T 1980
T 1942
T 1965
T 1696
T 0439
T 1643
T 1986
T 0977
T 1609
T 1898
T 0557
T 1283
T 1117
T 1115
T 1114
T 0231
T 0577
T 1655
T 1426
T 1285
T 0195
T 1154
T 0152
T 0153
T 0150
T 0846
T 0187
T 1458
T 1875
T 0603
T 1270
T 0666
T 1267
T 0549
T 1409
T 0649
T 0993
T 1177
T 0556
T 1297
T 2004
T 0110
T 1004
T 0675
T 0251
T 0581
T 0714
T 0398
T 0397
T 1896
T 0975
T 0087
T 0489
T 1140
T 0686
T 1921
T 1474
T 1450
T 0681
T 1249
T 1957
T 1947
T 1946
T 0674
T 1515
T 1451
T 1061
T 1146
T 1090
T 1422
T 0920
T 0371
T 0688
T 0929
T 1290
T 0618
T 0749
T 1687
T 1379
T 1065
T 0914
T 0612
T 1890
T 1112
T 1845
T 0985
T 1878
T 1103
T 0488
T 0321
T 1477
T 0249
T 1132
T 1022
T 0239
T 0408
T 0652
T 1746
T 1996
T 1545
T 1608
T 0758
T 1604
T 0330
T 0329
T 0708
T 0108
T 1619
T 1072
T 0473
T 0166
T 1927
T 1206
T 0575
T 0721
T 0982
T 1071
T 2028
T 0547
T 0463
T 1798
T 0281
T 1482
T 1862
T 0282
T 1174
T 0858
T 0283
T 0857
T 1330
T 0280
T 0476
T 1736
T 0452
T 0453
T 0454
T 0451
T 1498
T 1121
T 1479

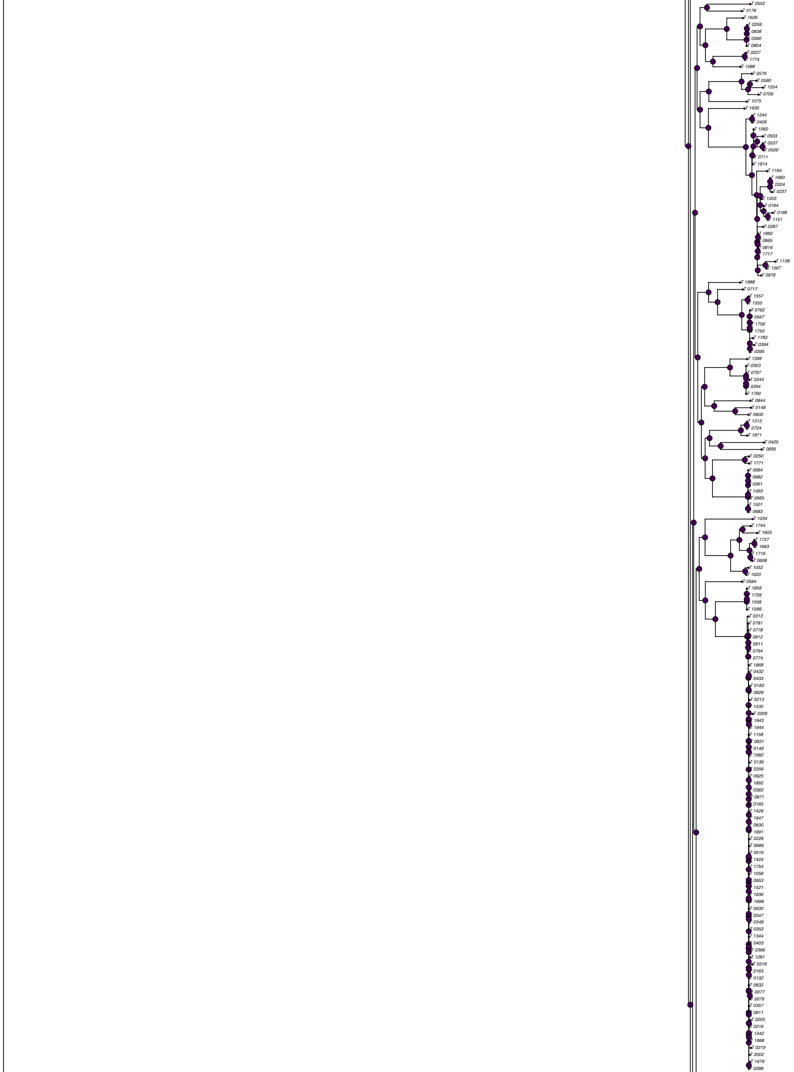
T 0552
T 0176
T 1626
T 0259
0838
0260
T 0804
T 0227
T 1774
T 1588
T 0579
T 0580
T 1554
T 0709
T 1075
T 1930
T 1244
T 0409
T 1082
T 0503
T 0537
T 0509
T 0711
T 1914
T 1164
T 1683
0324
T 0237
T 1203
T 0194
T 0186
T 1151
T 0287
T 1882
0665
T 0816
1717
T 1138
T 1567
T 0976
T 1988
T 0717
T 1557
T 1555
T 0762
0567
T 1756
1755
T 1783
T 0394
T 0395
T 1399
T 0303
0797
T 0244
0294
T 1760
T 0844
T 0148
T 0600
T 1313
T 0724
T 1871
T 0425
T 0695
T 0250
T 1771
T 0684
0682
0361
T 1493
T 0685
T 1501
T 0683
T 1034
T 1744
T 1603
T 1727
T 1663
T 1716
T 0608
T 1052
T 1620
T 0584
T 1859
1759
1558
T 1566
T 0313
T 0781
T 0778
0812
0911
T 0794
0774
T 1868
T 0432
0433
T 0183
T 0828
T 0213
1535
T 0209
1943
T 1944
T 1158
0831
0149
T 1982
T 0136
0356
T 0925
T 1892
0383
T 0871
0165
T 1429
T 1647
T 0830
1991
T 0226
T 0689
T 0516
1424
T 1754
T 1058
0953
1521
T 1936
1698
T 0930
0347
T 0348
T 0352
T 1344
0403
T 0396
T 1281
T 0318
0163
T 0132
T 0832
T 0077
T 0076
T 0357
0811
T 0205
T 0216
1342
T 1888
T 0219
T 2002
T 1679
T 0288

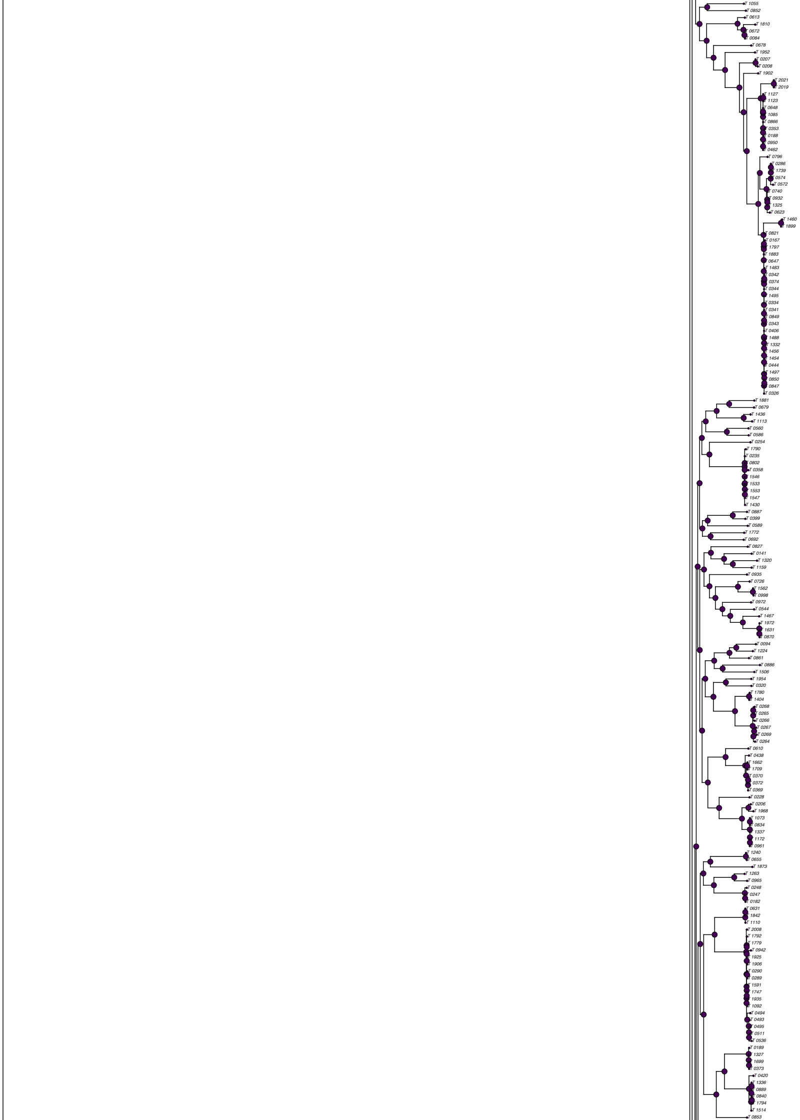
T 1055
T 0852
T 0613
T 1810
T 0672
T 0084
T 0678
T 1952
T 0207
T 0208
T 1902
T 2021
T 2019
T 1127
T 1123
T 0648
T 1085
T 0866
T 0353
T 0188
T 0950
T 0462
T 0796
T 0286
T 1739
T 0574
T 0572
T 0740
T 0932
T 1325
T 0623
T 1460
T 1899
T 0821
T 0167
T 1797
T 1883
T 0647
T 1483
T 0342
T 0374
T 0344
T 1495
T 0334
T 0341
T 0849
T 0343
T 0406
T 1488
T 1332
T 1456
T 1454
T 0444
T 1497
T 0850
T 0847
T 0326
T 1881
T 0679
T 1436
T 1113
T 0560
T 0586
T 0254
T 1790
T 0235
T 0802
T 0358
T 1546
T 1533
T 1553
T 1547
T 1430
T 0887
T 0399
T 0589
T 1772
T 0692
T 0827
T 0141
T 1320
T 1159
T 0935
T 0726
T 1562
T 0998
T 0972
T 0544
T 1467
T 1972
T 1631
T 0870
T 0094
T 1224
T 0861
T 0886
T 1506
T 1954
T 0320
T 1780
T 1404
T 0268
T 0265
T 0266
T 0267
T 0269
T 0264
T 0610
T 0438
T 1662
T 1709
T 0370
T 0372
T 0369
T 0228
T 0206
T 1968
T 1073
T 0834
T 1337
T 1172
T 0961
T 1240
T 0655
T 1873
T 1263
T 0965
T 0248
T 0247
T 0182
T 0631
T 1842
T 1110
T 2008
T 1792
T 1779
T 0942
T 1925
T 1906
T 0290
T 0289
T 1591
T 1747
T 1935
T 1092
T 0494
T 0493
T 0495
T 0511
T 0536
T 0189
T 1327
T 1699
T 0373
T 0420
T 1336
T 0889
T 0840
T 1794
T 1514
T 0853

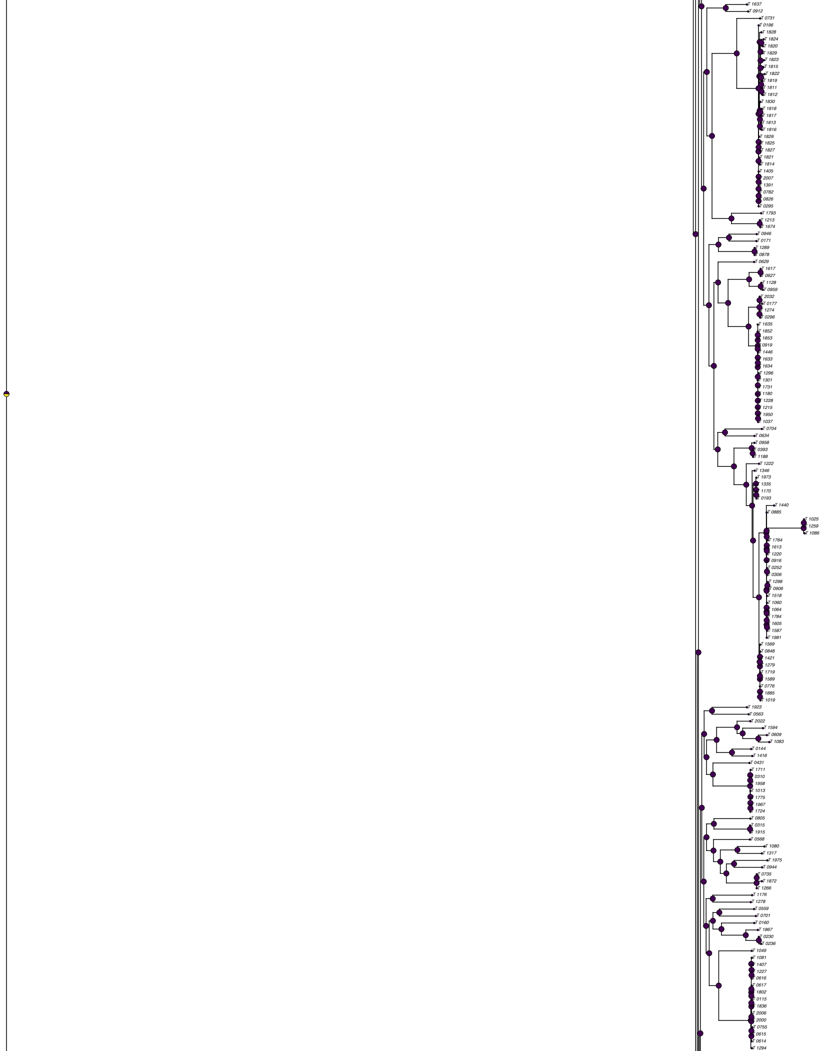
T 1637
T 0912
T 0731
T 0196
T 1828
T 1824
T 1820
T 1829
T 1823
T 1815
T 1822
T 1819
T 1811
T 1812
T 1830
T 1818
T 1817
T 1813
T 1816
T 1826
T 1825
T 1827
T 1821
T 1814
T 1405
2007
1391
0782
0826
T 0295
T 1793
T 1213
T 1674
T 0946
T 0171
T 1289
T 0878
T 0629
T 1617
T 0627
T 1128
T 0959
T 2032
T 0177
T 1274
T 0296
T 1635
T 1852
1853
0919
T 1446
1633
1634
T 1296
T 1301
T 1731
1180
T 1228
1215
1950
T 1037
T 0704
T 0634
T 0958
T 0393
T 1188
T 1222
T 1346
T 1973
1335
1170
T 0193
T 1440
T 0885
T 1025
1259
T 1086
T 1764
1613
T 1220
0916
T 0252
T 0306
T 1298
T 0906
T 1518
T 1060
1064
T 1784
1605
T 1587
T 1981
T 1569
T 0848
1421
1279
T 1719
1589
T 0776
1885
T 1019
T 1923
T 0563
T 2022
T 1594
T 0609
T 1083
T 0144
T 1416
T 0431
T 1711
0310
1958
T 1013
1775
1967
1724
T 0805
T 0315
1915
T 0568
T 1080
T 1317
T 1975
T 0944
T 0735
T 1872
T 1266
T 1176
T 1278
T 0559
T 0701
T 0160
T 1867
T 0230
T 0236
T 1049
T 1081
1407
1227
0616
T 0617
1802
T 0115
1836
T 2006
2000
T 0755
0615
T 0614
T 1294

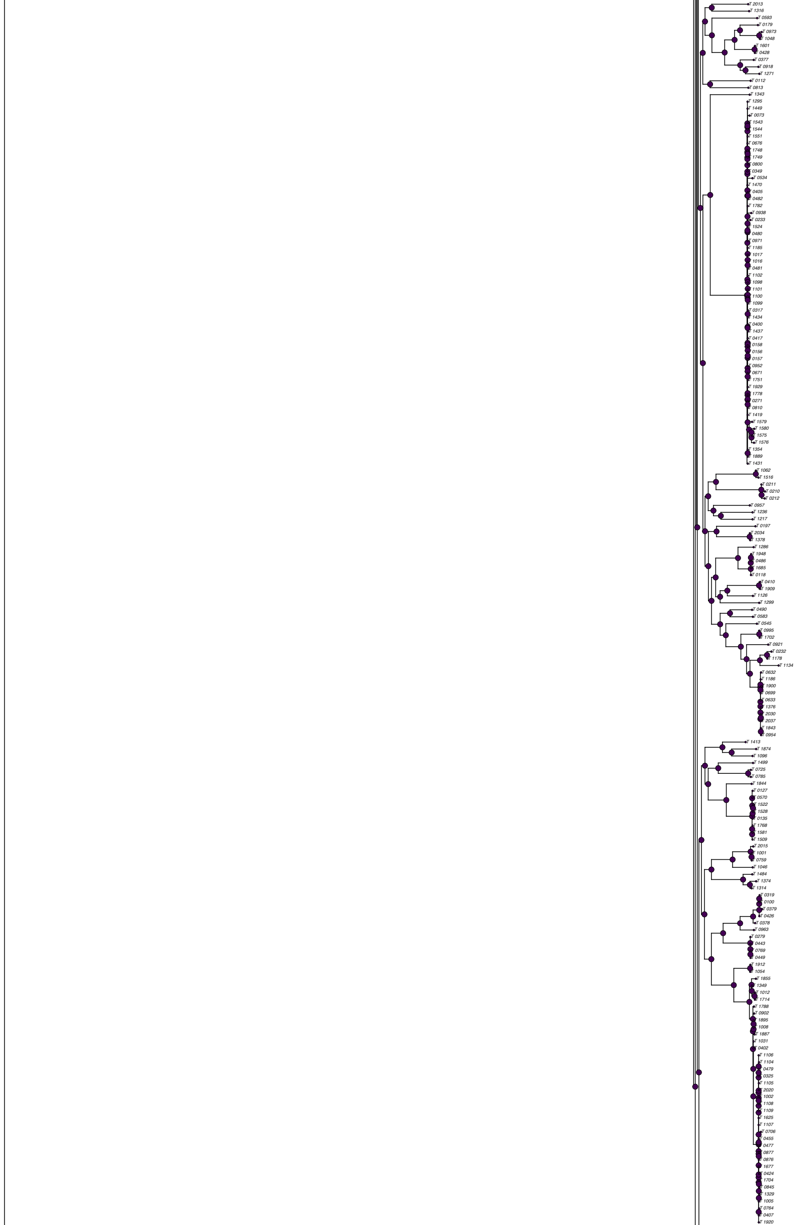

T 2013
T 1316
T 0593
T 0179
T 0973
T 1048
T 1601
T 0428
T 0377
T 0918
T 1271
T 0112
T 0813
T 1343
T 1295
T 1449
T 0073
1543
1544
T 1551
T 0676
1748
1749
0800
0349
T 0534
T 1470
0405
0482
T 1782
T 0938
T 0233
1524
0480
0971
1185
1017
1016
0481
1102
1098
1101
1100
1099
T 0317
1434
T 0400
1437
T 0417
0158
0156
0157
0952
0671
1751
T 1929
1778
0271
0810
T 1419
1579
T 1580
1575
T 1576
T 1354
1889
T 1431
T 1062
T 1516
T 0211
T 0210
T 0212
T 0957
T 1236
T 1217
T 0197
T 2034
T 1378
T 1286
T 1948
0486
1685
T 0118
T 0410
T 1909
T 1126
T 1299
T 0490
T 0583
T 0545
T 0995
T 1702
T 0921
T 0232
T 1178
T 1134
T 0632
T 1186
1900
0699
T 0633
1376
2030
2037
T 1843
T 0954
T 1413
T 1874
T 1096
T 1499
T 0725
T 0785
T 1844
T 0127
0570
1522
1528
0135
T 1768
1581
T 1509
T 2015
T 1001
T 0759
T 1046
T 1484
T 1374
T 1314
T 0319
0100
T 0379
T 0426
T 0378
T 0963
T 0279
0443
0769
0449
T 1912
1054
T 1855
1349
T 1012
T 1714
T 1788
T 0902
1895
1008
T 1887
T 1031
0402
T 1106
T 1104
0479
0325
T 1105
2020
1002
1108
1109
T 1625
T 1107
T 0706
T 0455
0477
0877
0876
1677
0424
1704
0845
1329
1005
T 0764
0407
T 1920

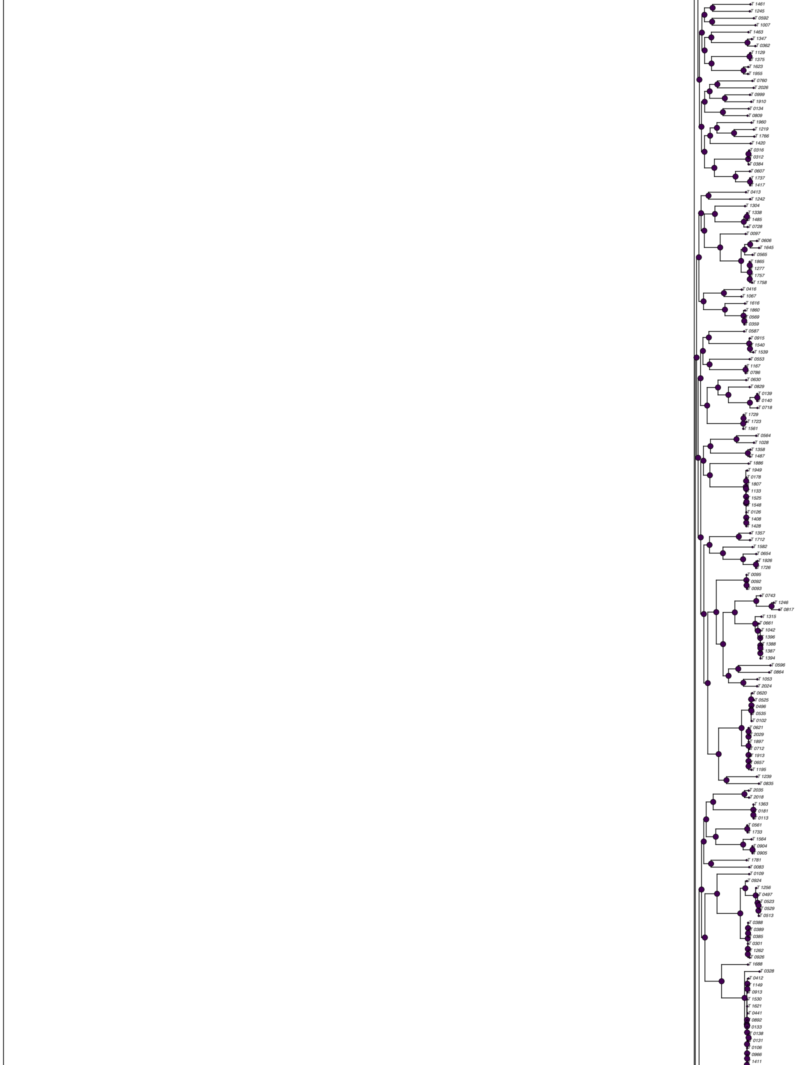
T 1461
T 1245
T 0592
T 1007
T 1463
T 1347
T 0362
T 1129
T 1375
T 1623
T 1955
T 0760
T 2026
T 0999
T 1910
T 0134
T 0809
T 1960
T 1219
T 1766
T 1420
T 0316
T 0312
T 0384
T 0607
T 1737
T 1417
T 0413
T 1242
T 1304
T 1338
T 1485
T 0728
T 0097
T 0606
T 1645
T 0565
T 1865
T 1277
T 1757
T 1758
T 0416
T 1067
T 1616
T 1860
T 0569
T 0359
T 0587
T 0915
T 1540
T 1539
T 0553
T 1167
T 0786
T 0630
T 0829
T 0139
T 0140
T 0718
T 1729
T 1723
T 1561
T 0564
T 1028
T 1358
T 1487
T 1886
T 1949
T 0178
T 1807
T 1133
T 1525
T 1548
T 0126
T 1408
T 1428
T 1357
T 1712
T 1582
T 0654
T 1926
T 1726
T 0095
T 0092
T 0093
T 0743
T 1246
T 0817
T 1315
T 0661
T 1042
T 1396
T 1388
T 1387
T 1394
T 0596
T 0864
T 1053
T 2024
T 0620
T 0525
T 0496
T 0535
T 0102
T 0621
T 2029
T 1897
T 0712
T 1913
T 0657
T 1195
T 1239
T 0835
T 2035
T 2018
T 1363
T 0181
T 0113
T 0561
T 1733
T 1564
T 0904
T 0905
T 1781
T 0083
T 0109
T 0924
T 1256
T 0497
T 0523
T 0529
T 0513
T 0388
T 0389
T 0385
T 0301
T 1262
T 0926
T 1688
T 0328
T 0412
T 1149
T 0913
T 1530
T 1621
T 0441
T 0892
T 0133
T 0138
T 0131
T 0106
T 0966
T 1411

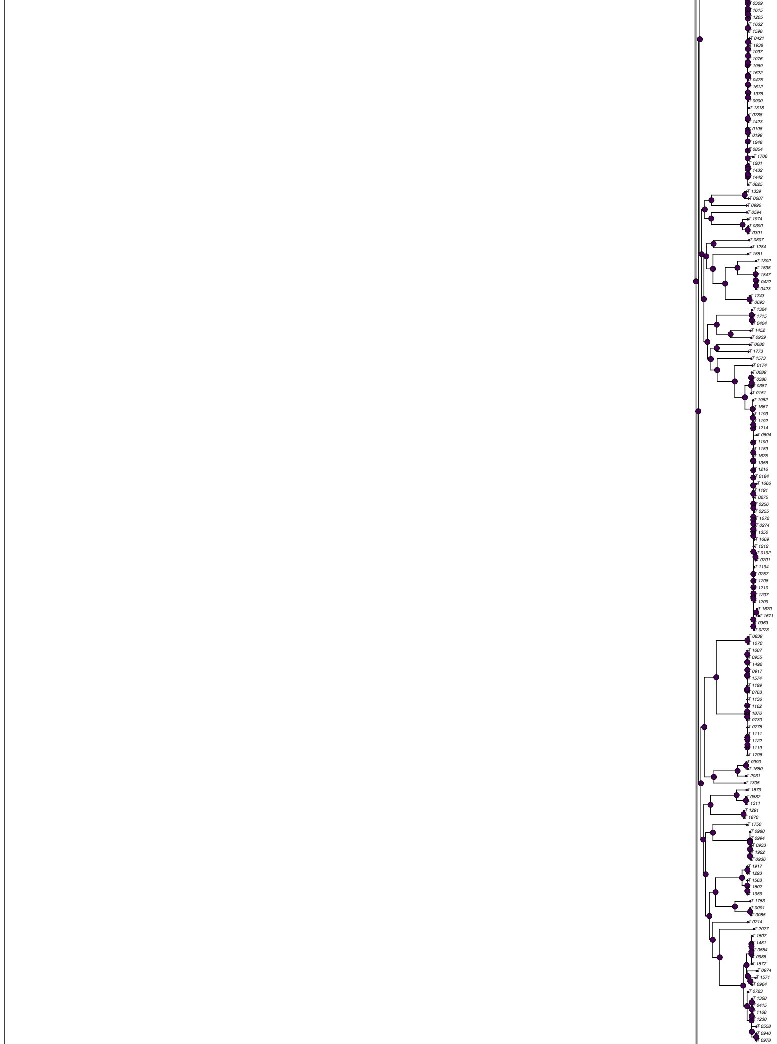
T 0309
T 1615
T 1205
T 1632
T 1598
T 0421
T 1938
T 1097
T 1076
T 1969
T 1622
T 0475
T 1612
T 1976
T 0900
T 1318
T 0788
T 1423
T 0198
T 0199
T 1248
T 0854
T 1706
T 1201
T 1432
T 1442
T 0825
T 1339
T 0687
T 0996
T 0594
T 1974
T 0390
T 0391
T 0807
T 1284
T 1851
T 1302
T 1838
T 1847
T 0422
T 0423
T 1743
T 0693
T 1324
T 1715
T 0404
T 1452
T 0939
T 0680
T 1773
T 1573
T 0174
T 0089
T 0386
T 0387
T 0151
T 1962
T 1667
T 1193
T 1192
T 1214
T 0694
T 1190
T 1189
T 1675
T 1356
T 1216
T 0184
T 1666
T 1191
T 0275
T 0256
T 0255
T 1672
T 0274
T 1350
T 1669
T 1212
T 0192
T 0201
T 1194
T 0257
T 1208
T 1210
T 1207
T 1209
T 1670
T 1671
T 0363
T 0273
T 0839
T 1070
T 1607
T 0955
T 1492
T 0917
T 1574
T 1199
T 0763
T 1136
T 1162
T 1876
T 0730
T 0775
T 1111
T 1122
T 1119
T 1796
T 0990
T 1650
T 2031
T 1305
T 1879
T 0882
T 1311
T 1291
T 1870
T 1750
T 0980
T 0994
T 0933
T 1922
T 0936
T 1917
T 1293
T 1563
T 1502
T 1959
T 1753
T 0091
T 0085
T 0214
T 2027
T 1507
T 1481
T 0554
T 0988
T 1577
T 0974
T 1571
T 0964
T 0723
T 1368
T 0415
T 1168
T 1230
T 0558
T 0940
T 0978

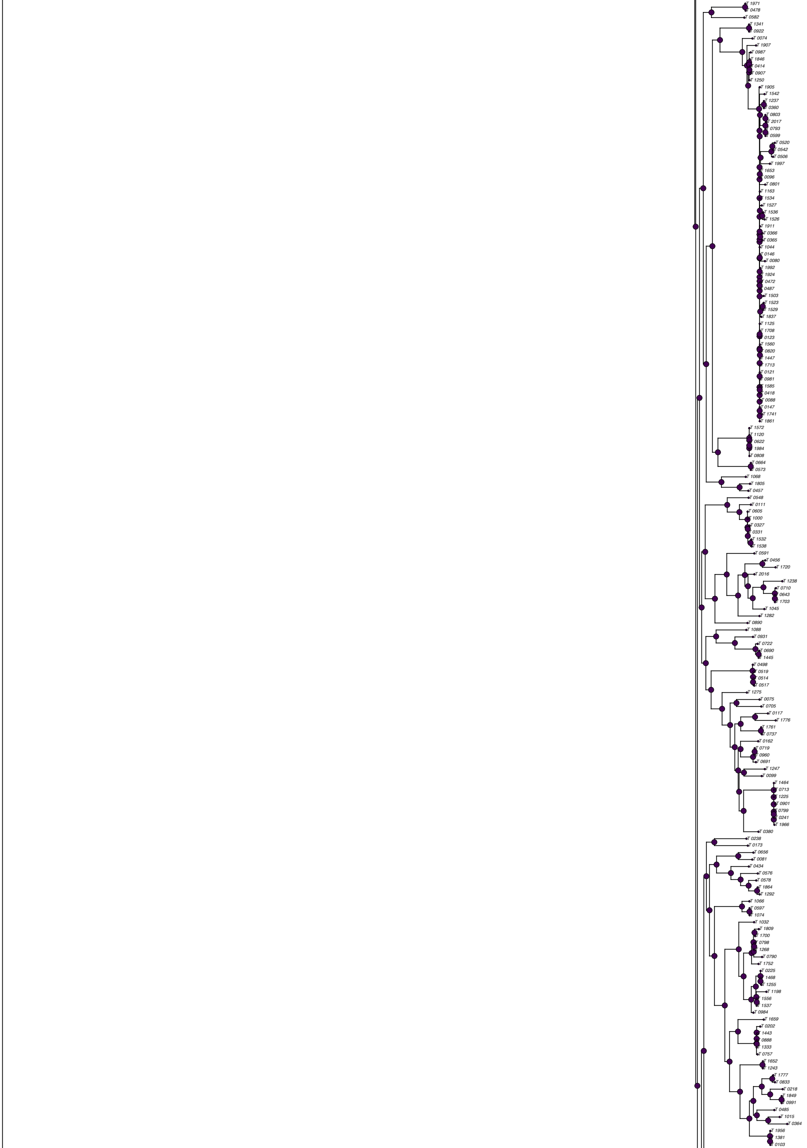
T 1971
T 0478
T 0582
T 1341
T 0922
T 0074
T 1907
T 0987
T 1846
T 0414
T 0907
T 1250
T 1905
T 1542
T 1237
T 0360
T 0803
T 2017
T 0793
T 0599
T 0520
T 0542
T 0506
T 1997
T 1653
T 0096
T 0801
T 1163
T 1534
T 1527
T 1536
T 1526
T 1911
T 0366
T 0365
T 1044
T 0146
T 0080
T 1992
T 1924
T 0472
T 0487
T 1503
T 1523
T 1529
T 1837
T 1125
T 1708
T 0123
T 1560
T 0820
T 1447
T 1713
T 0121
T 0981
T 1585
T 0418
T 0088
T 0147
T 1741
T 1861
T 1572
T 1120
T 0622
T 1984
T 0808
T 0664
T 0573
T 1068
T 1805
T 0457
T 0548
T 0111
T 0605
T 1000
T 0327
T 0331
T 1532
T 1538
T 0591
T 0456
T 1720
T 2016
T 1238
T 0710
T 0643
T 1703
T 1045
T 1282
T 0890
T 1088
T 0931
T 0722
T 0690
T 1445
T 0498
T 0519
T 0514
T 0517
T 1275
T 0075
T 0705
T 0117
T 1776
T 1761
T 0737
T 0162
T 0719
T 0960
T 0691
T 1247
T 0099
T 1464
T 0713
T 1225
T 0901
T 0799
T 0241
T 1966
T 0380
T 0238
T 0173
T 0656
T 0081
T 0434
T 0576
T 0578
T 1864
T 1292
T 1066
T 0597
T 1074
T 1032
T 1809
T 1700
T 0798
T 1268
T 0790
T 1752
T 0225
T 1468
T 1255
T 1198
T 1556
T 1537
T 0984
T 1659
T 0202
T 1443
T 0888
T 1333
T 0757
T 1652
T 1243
T 1777
T 0833
T 0218
T 1849
T 0991
T 0485
T 1015
T 0364
T 1956
T 1381
T 0103

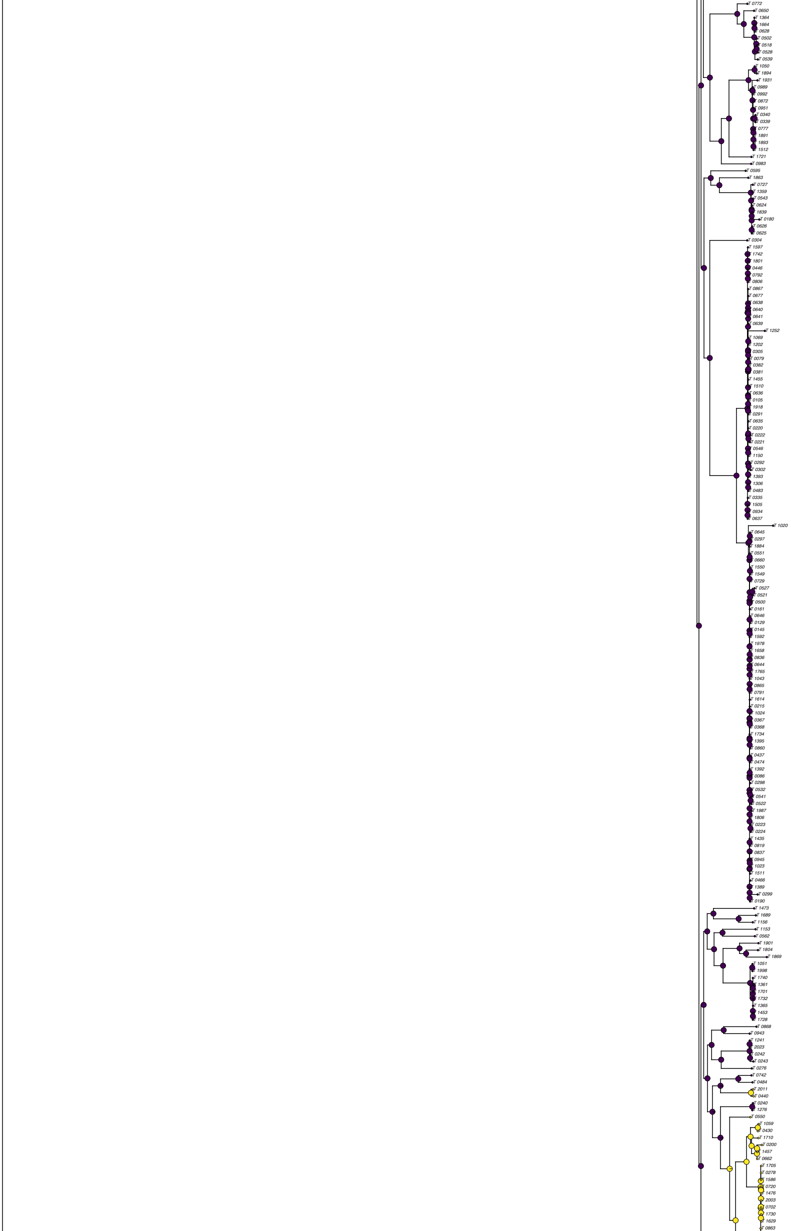
T 0772
T 0650
T 1364
T 1664
T 0628
T 0502
T 0518
T 0528
T 0539
T 1050
T 1894
T 1931
T 0989
T 0992
T 0872
T 0951
T 0340
T 0339
T 0777
T 1891
T 1893
T 1512
T 1721
T 0983
T 0595
T 1863
T 0727
T 1359
T 0543
T 0624
T 1839
T 0180
T 0626
T 0625
T 0304
T 1597
T 1742
T 1801
T 0446
T 0792
T 0806
T 0867
T 0677
T 0638
T 0640
T 0641
T 0639
T 1252
T 1069
T 1202
T 0305
T 0079
T 0382
T 0381
T 1455
T 1510
T 0636
T 0105
T 1918
T 0291
T 0635
T 0220
T 0222
T 0221
T 0546
T 1150
T 0292
T 0302
T 1393
T 1306
T 0483
T 0335
T 1505
T 0934
T 0637
T 1020
T 0645
T 0297
T 1884
T 0551
T 0660
T 1550
T 1549
T 0729
T 0527
T 0521
T 0500
T 0161
T 0646
T 0129
T 0145
T 1592
T 1978
T 1658
T 0836
T 0644
T 1765
T 1043
T 0865
T 0791
T 1614
T 0215
T 1024
T 0367
T 0368
T 1734
T 1395
T 0860
T 0437
T 0474
T 1392
T 0086
T 0298
T 0532
T 0541
T 0522
T 1987
T 1806
T 0223
T 0224
T 1435
T 0819
T 0837
T 0945
T 1023
T 1511
T 0466
T 1389
T 0299
T 0190
T 1473
T 1689
T 1156
T 1153
T 0562
T 1901
T 1804
T 1869
T 1051
T 1998
T 1740
T 1361
T 1701
T 1732
T 1365
T 1453
T 1728
T 0868
T 0943
T 1241
T 2023
T 0242
T 0243
T 0276
T 0742
T 0484
T 2011
T 0440
T 0240
T 1276
T 0550
T 1059
T 0430
T 1710
T 0200
T 1457
T 0662
T 1705
T 0278
T 1586
T 0720
T 1476
T 2003
T 0702
T 1730
T 1629
T 0863

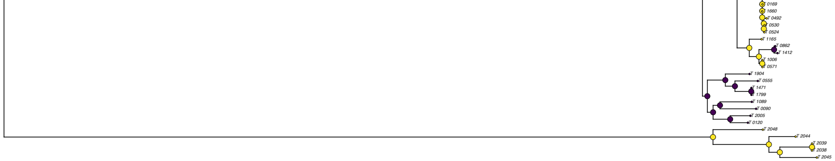
0169
1660
T 0492
T 0530
T 0524
T 1165
T 0862
T 1412
T 1006
T 0571
T 1904
T 0555
T 1471
T 1799
T 1089
T 0090
T 2005
T 0120
T 2048
T 2044
T 2039
T 2038
T 2045

Effector gene: tle1

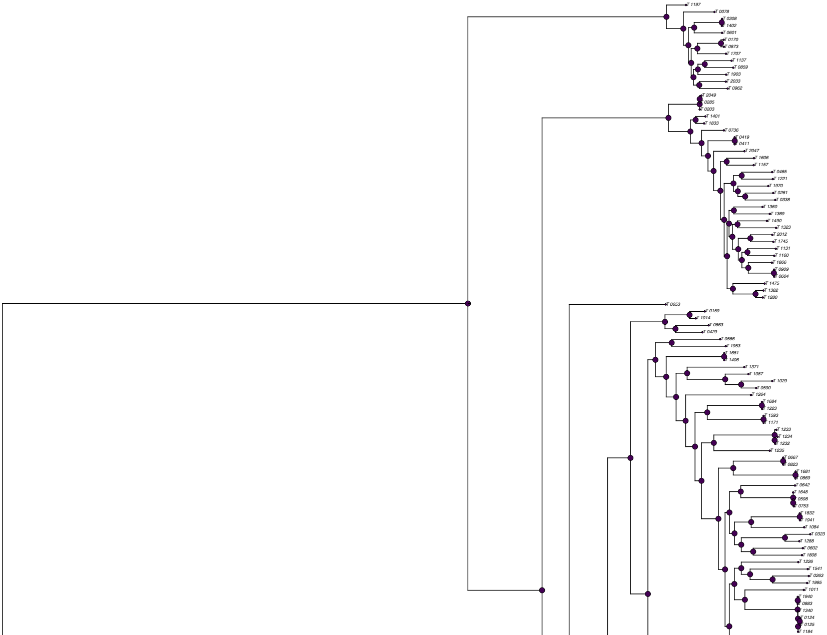

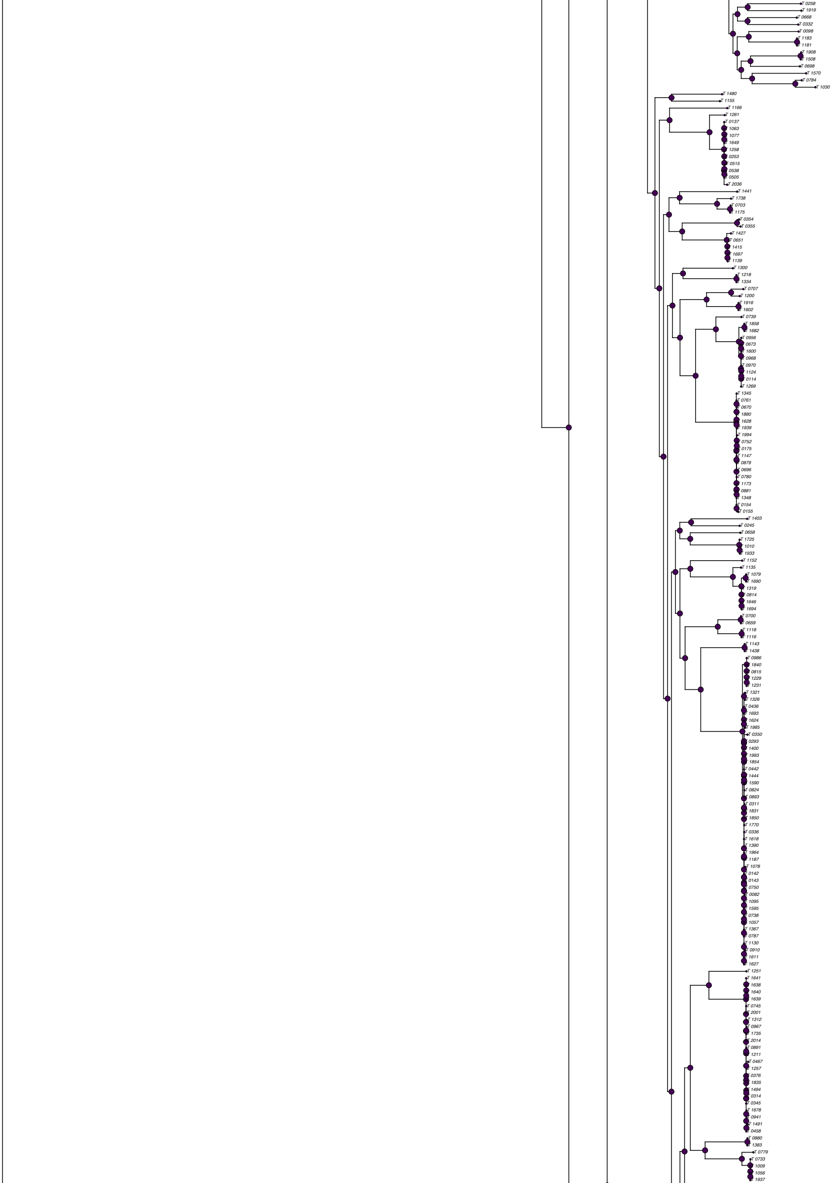
T 0258
T 1919
T 0668
T 0332
T 0098
T 1183
T 1181
T 1908
T 1508
T 0698
T 1570
T 0784
T 1030
T 1480
T 1155
T 1166
T 1261
T 0137
1063
1077
T 1649
1258
0253
0515
0538
0505
T 2036
T 1441
T 1738
T 0703
T 1175
T 0354
T 0355
T 1427
0651
1415
1697
1139
T 1300
T 1218
1334
T 0707
T 1200
T 1916
T 1602
T 0739
T 1858
1682
T 0956
0673
1600
0968
T 0970
1124
0114
T 1269
T 1345
T 0761
0670
1880
1628
1939
T 1994
0752
0175
T 1147
0879
0696
T 0780
1173
0881
1348
T 0154
T 0155
T 1403
T 0245
T 0658
T 1725
1010
1933
T 1152
T 1135
T 1079
T 1690
1319
0814
1646
1694
T 0700
T 0659
T 1118
T 1116
T 1143
1438
T 0986
1840
0815
1229
1231
T 1321
T 1326
0436
1693
1624
T 1985
T 0350
0293
1400
1993
1854
T 0442
1444
1590
T 0824
0893
T 0311
1831
1850
T 1770
T 0336
T 1618
1390
1964
1187
T 1078
0142
0143
0750
0082
1095
1595
0738
1057
T 1367
0787
T 1130
T 0910
1611
1627
T 1251
T 1641
1638
1640
1639
T 0745
2001
T 1312
T 0967
1735
2014
T 0891
1211
T 0467
1257
0376
1835
1494
0314
T 0345
T 1678
0941
T 1491
0458
T 0880
1383
T 0779
T 0733
1009
1056
1937

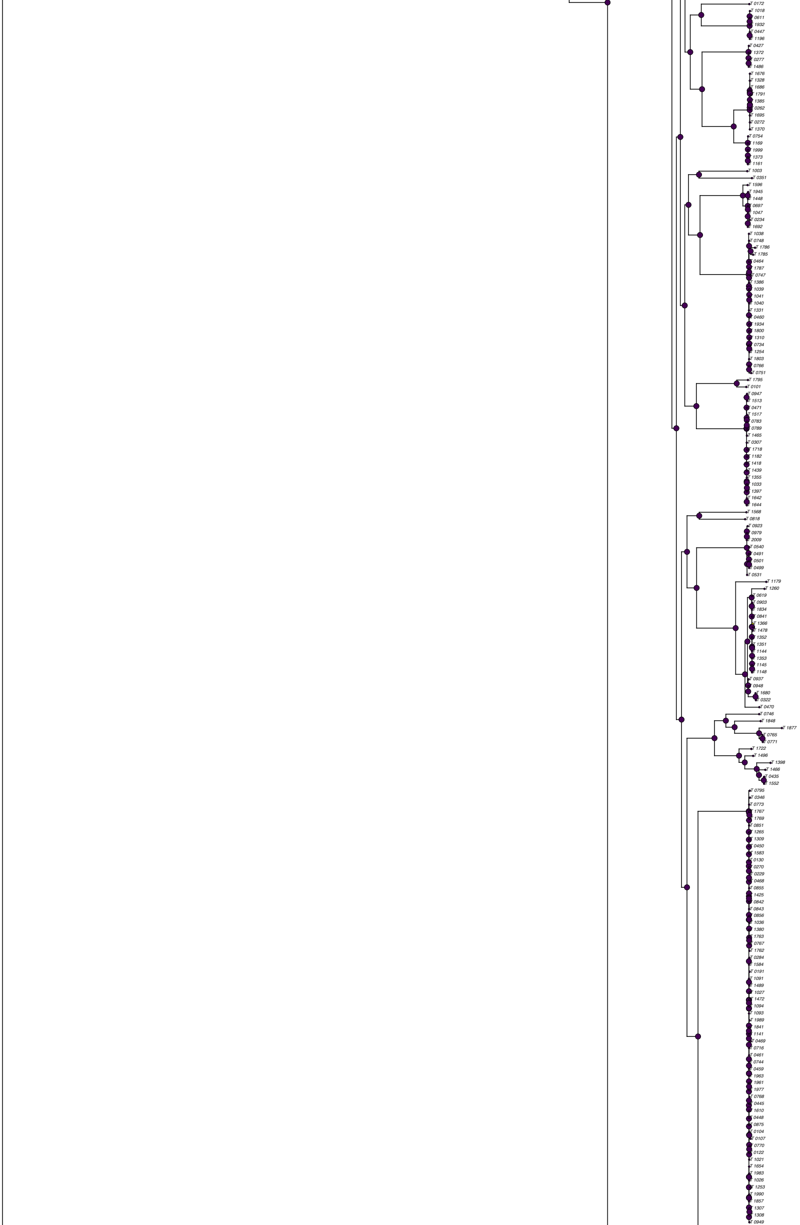

T 0172
T 1018
T 0611
T 1932
T 0447
T 1196
T 0427
T 1372
T 0277
T 1486
T 1676
T 1328
T 1686
T 1791
T 1385
T 0262
T 1695
T 0272
T 1370
T 0754
T 1169
T 1999
T 1373
T 1161
T 1003
T 0351
T 1596
T 1945
T 1448
T 0697
T 1047
T 0234
T 1692
T 1038
T 0748
T 1786
T 1785
T 0464
T 1787
T 0747
T 1386
T 1039
T 1041
T 1040
T 1331
T 0460
T 1934
T 1800
T 1310
T 0734
T 1254
T 1803
T 0766
T 0751
T 1795
T 0101
T 0947
T 1513
T 0471
T 1517
T 0783
T 0789
T 1465
T 0307
T 1718
T 1182
T 1418
T 1439
T 1355
T 1033
T 1397
T 1642
T 1644
T 1568
T 0818
T 0923
T 0979
T 2009
T 0540
T 0491
T 0501
T 0499
T 0531
T 1179
T 1260
T 0619
T 0903
T 1834
T 0841
T 1366
T 1478
T 1352
T 1351
T 1144
T 1353
T 1145
T 1148
T 0937
T 0948
T 1680
T 0322
T 0470
T 0746
T 1848
T 1877
T 0765
T 0771
T 1722
T 1496
T 1398
T 1466
T 0435
T 1552
T 0795
T 0346
T 0773
T 1767
T 1769
T 0851
T 1265
T 1309
T 0450
T 1583
T 0130
T 0270
T 0229
T 0468
T 0855
T 1425
T 0842
T 0843
T 0856
T 1036
T 1380
T 1763
T 0767
T 1762
T 0284
T 1584
T 0191
T 1091
T 1489
T 1027
T 1472
T 1094
T 1093
T 1989
T 1841
T 1141
T 0469
T 0716
T 0461
T 0744
T 0459
T 1963
T 1961
T 1977
T 0768
T 0445
T 1610
T 0448
T 0875
T 0104
T 0107
T 0770
T 0122
T 1021
T 1654
T 1983
T 1026
T 1253
T 1990
T 1857
T 1307
T 1308
T 0949

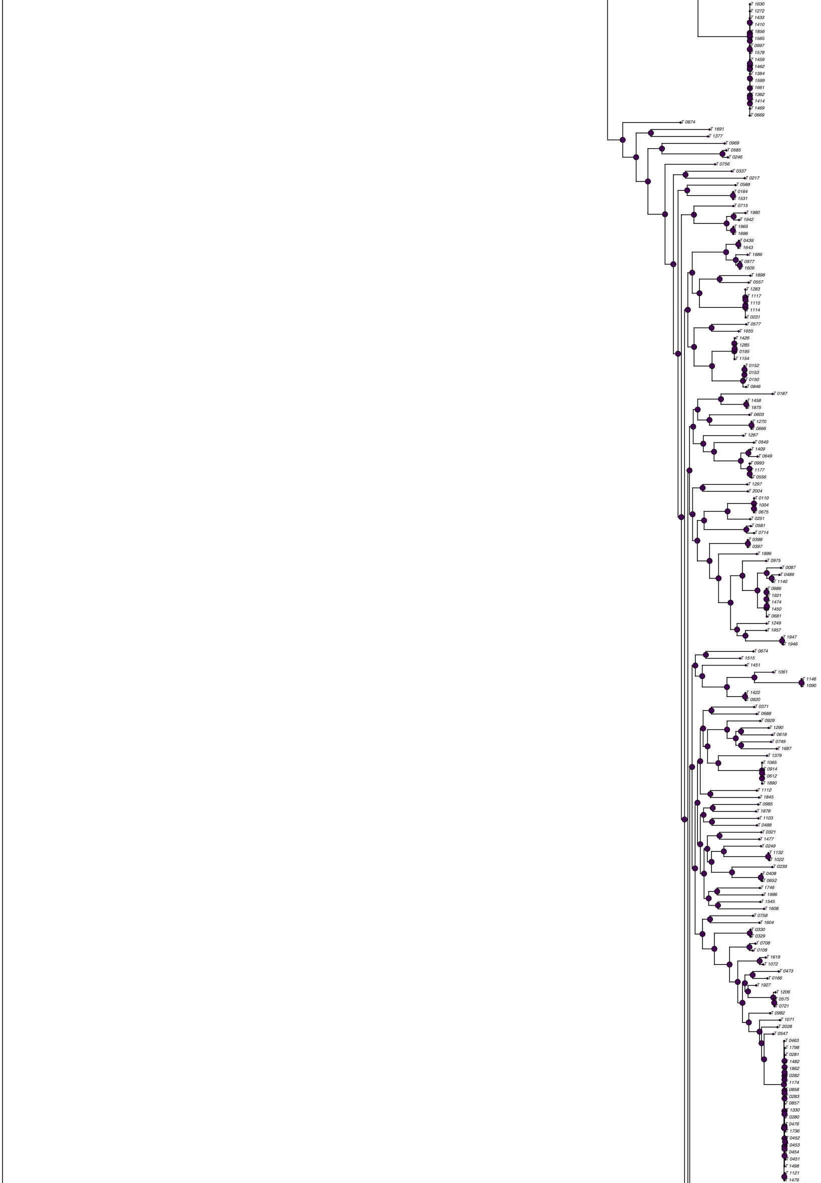
T 1630
T 1272
T 1433
T 1410
T 1856
T 1565
T 0997
T 1578
T 1459
T 1462
T 1384
T 1599
T 1661
T 1362
T 1414
T 1469
T 0669
T 0874
T 1691
T 1377
T 0969
T 0585
T 0246
T 0756
T 0337
T 0217
T 0588
T 0164
T 1531
T 0715
T 1980
T 1942
T 1965
T 1696
T 0439
T 1643
T 1986
T 0977
T 1609
T 1898
T 0557
T 1283
T 1117
T 1115
T 1114
T 0231
T 0577
T 1655
T 1426
T 1285
T 0195
T 1154
T 0152
T 0153
T 0150
T 0846
T 0187
T 1458
T 1875
T 0603
T 1270
T 0666
T 1267
T 0549
T 1409
T 0649
T 0993
T 1177
T 0556
T 1297
T 2004
T 0110
T 1004
T 0675
T 0251
T 0581
T 0714
T 0398
T 0397
T 1896
T 0975
T 0087
T 0489
T 1140
T 0686
T 1921
T 1474
T 1450
T 0681
T 1249
T 1957
T 1947
T 1946
T 0674
T 1515
T 1451
T 1061
T 1146
T 1090
T 1422
T 0920
T 0371
T 0688
T 0929
T 1290
T 0618
T 0749
T 1687
T 1379
T 1065
T 0914
T 0612
T 1890
T 1112
T 1845
T 0985
T 1878
T 1103
T 0488
T 0321
T 1477
T 0249
T 1132
T 1022
T 0239
T 0408
T 0652
T 1746
T 1996
T 1545
T 1608
T 0758
T 1604
T 0330
T 0329
T 0708
T 0108
T 1619
T 1072
T 0473
T 0166
T 1927
T 1206
T 0575
T 0721
T 0982
T 1071
T 2028
T 0547
T 0463
T 1798
T 0281
T 1482
T 1862
T 0282
T 1174
T 0858
T 0283
T 0857
T 1330
T 0280
T 0476
T 1736
T 0452
T 0453
T 0454
T 0451
T 1498
T 1121
T 1479

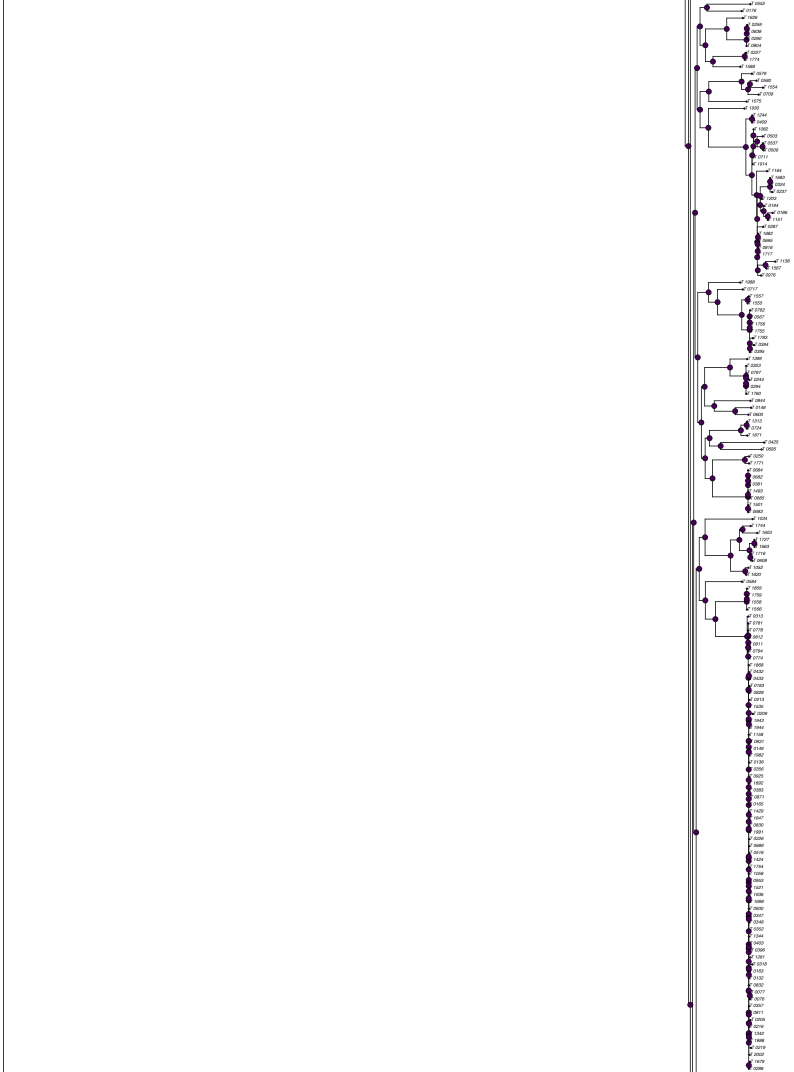
T 0552
T 0176
T 1626
T 0259
T 0838
T 0260
T 0804
T 0227
T 1774
T 1588
T 0579
T 0580
T 1554
T 0709
T 1075
T 1930
T 1244
T 0409
T 1082
T 0503
T 0537
T 0509
T 0711
T 1914
T 1164
T 1683
T 0324
T 0237
T 1203
T 0194
T 0186
T 1151
T 0287
T 1882
T 0665
T 0816
T 1717
T 1138
T 1567
T 0976
T 1988
T 0717
T 1557
T 1555
T 0762
T 0567
T 1756
T 1755
T 1783
T 0394
T 0395
T 1399
T 0303
T 0797
T 0244
T 0294
T 1760
T 0844
T 0148
T 0600
T 1313
T 0724
T 1871
T 0425
T 0695
T 0250
T 1771
T 0684
T 0682
T 0361
T 1493
T 0685
T 1501
T 0683
T 1034
T 1744
T 1603
T 1727
T 1663
T 1716
T 0608
T 1052
T 1620
T 0584
T 1859
T 1759
T 1558
T 1566
T 0313
T 0781
T 0778
T 0812
T 0911
T 0794
T 0774
T 1868
T 0432
T 0433
T 0183
T 0828
T 0213
T 1535
T 0209
T 1943
T 1944
T 1158
T 0831
T 0149
T 1982
T 0136
T 0356
T 0925
T 1892
T 0383
T 0871
T 0165
T 1429
T 1647
T 0830
T 1991
T 0226
T 0689
T 0516
T 1424
T 1754
T 1058
T 0953
T 1521
T 1936
T 1698
T 0930
T 0347
T 0348
T 0352
T 1344
T 0403
T 0396
T 1281
T 0318
T 0163
T 0132
T 0832
T 0077
T 0076
T 0357
T 0811
T 0205
T 0216
T 1342
T 1888
T 0219
T 2002
T 1679
T 0288

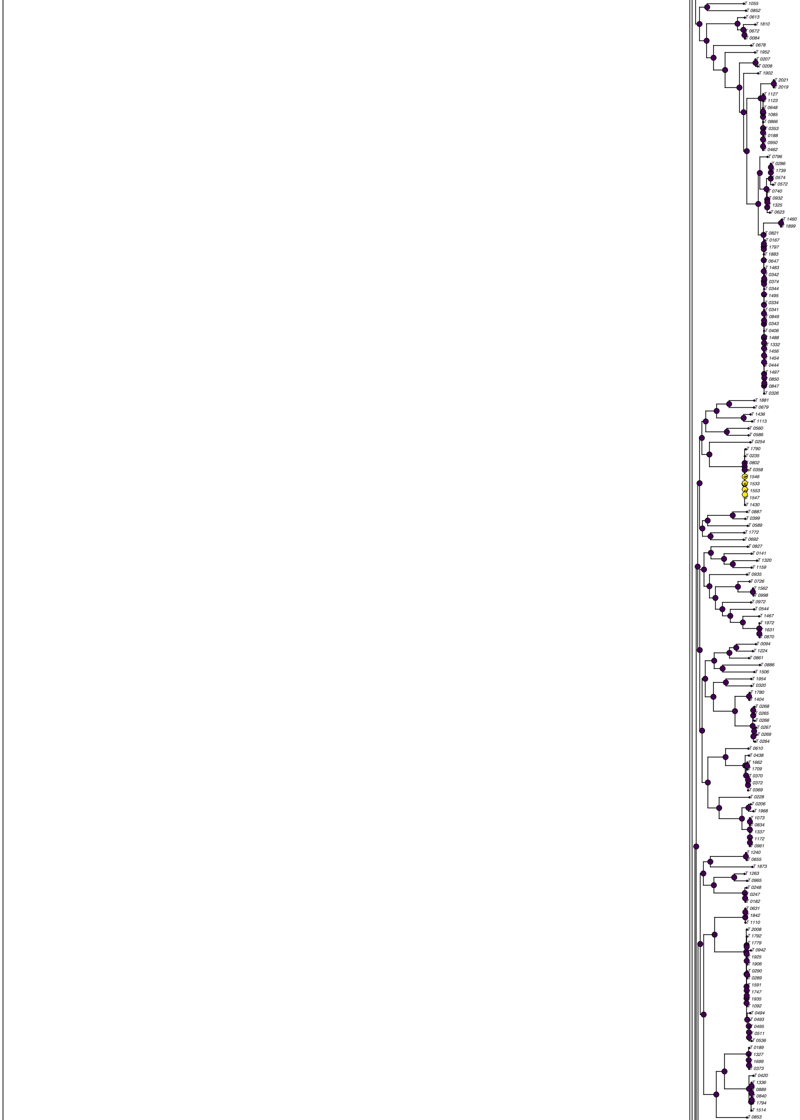
T 1055
T 0852
T 0613
T 1810
T 0672
T 0084
T 0678
T 1952
T 0207
T 0208
T 1902
T 2021
T 2019
T 1127
T 1123
T 0648
T 1085
T 0866
T 0353
T 0188
T 0950
T 0462
T 0796
T 0286
T 1739
T 0574
T 0572
T 0740
T 0932
T 1325
T 0623
T 1460
T 1899
T 0821
T 0167
T 1797
T 1883
T 0647
T 1483
T 0342
T 0374
T 0344
T 1495
T 0334
T 0341
T 0849
T 0343
T 0406
T 1488
T 1332
T 1456
T 1454
T 0444
T 1497
T 0850
T 0847
T 0326
T 1881
T 0679
T 1436
T 1113
T 0560
T 0586
T 0254
T 1790
T 0235
T 0802
T 0358
T 1546
T 1533
T 1553
T 1547
T 1430
T 0887
T 0399
T 0589
T 1772
T 0692
T 0827
T 0141
T 1320
T 1159
T 0935
T 0726
T 1562
T 0998
T 0972
T 0544
T 1467
T 1972
T 1631
T 0870
T 0094
T 1224
T 0861
T 0886
T 1506
T 1954
T 0320
T 1780
T 1404
T 0268
T 0265
T 0266
T 0267
T 0269
T 0264
T 0610
T 0438
T 1662
T 1709
T 0370
T 0372
T 0369
T 0228
T 0206
T 1968
T 1073
T 0834
T 1337
T 1172
T 0961
T 1240
T 0655
T 1873
T 1263
T 0965
T 0248
T 0247
T 0182
T 0631
T 1842
T 1110
T 2008
T 1792
T 1779
T 0942
T 1925
T 1906
T 0290
T 0289
T 1591
T 1747
T 1935
T 1092
T 0494
T 0493
T 0495
T 0511
T 0536
T 0189
T 1327
T 1699
T 0373
T 0420
T 1336
T 0889
T 0840
T 1794
T 1514
T 0853

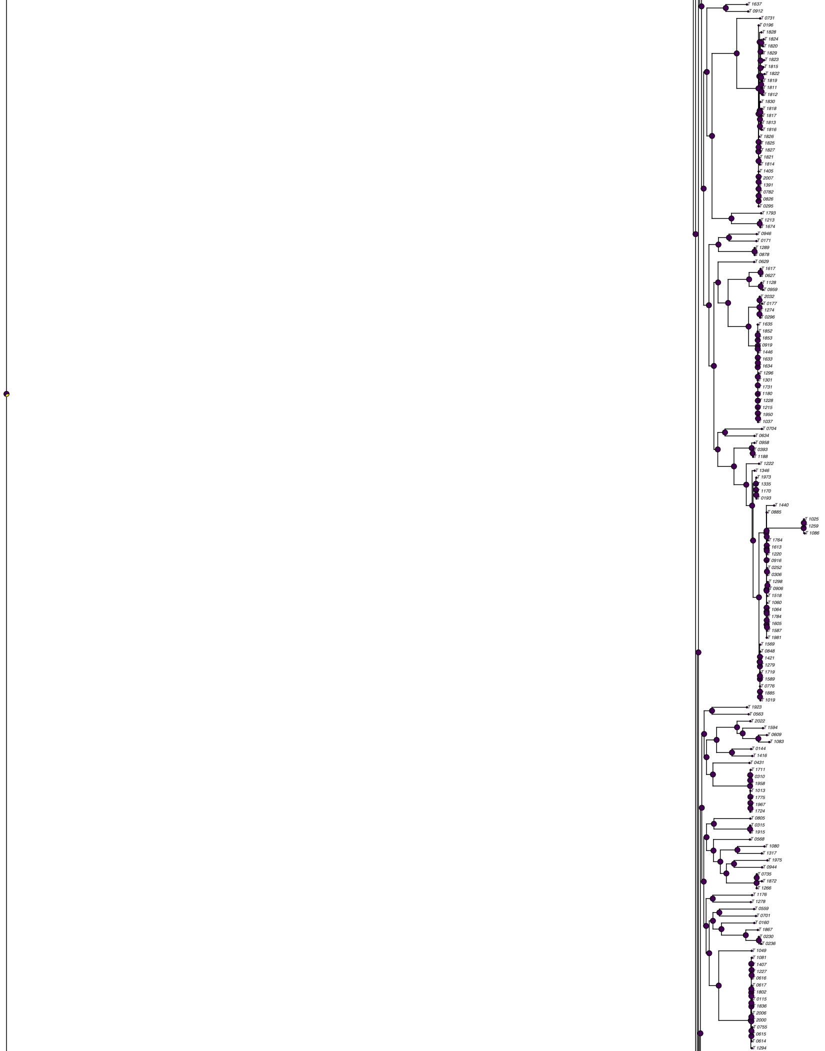
T 1637
T 0912
T 0731
T 0196
T 1828
T 1824
T 1820
T 1829
T 1823
T 1815
T 1822
T 1819
T 1811
T 1812
T 1830
T 1818
T 1817
T 1813
T 1816
T 1826
T 1825
T 1827
T 1821
T 1814
T 1405
2007
1391
0782
0826
T 0295
T 1793
T 1213
T 1674
T 0946
T 0171
T 1289
T 0878
T 0629
T 1617
T 0627
T 1128
T 0959
T 2032
T 0177
1274
T 0296
T 1635
1852
1853
0919
1446
1633
1634
1296
1301
1731
1180
1228
1215
1950
1037
T 0704
T 0634
T 0958
0393
1188
T 1222
T 1346
T 1973
1335
1170
0193
T 1440
T 0885
T 1025
1259
T 1086
T 1764
1613
1220
0916
T 0252
0306
T 1298
T 0906
T 1518
T 1060
1064
1784
1605
1587
T 1981
T 1569
T 0848
1421
1279
T 1719
1589
T 0776
1885
T 1019
T 1923
T 0563
T 2022
T 1594
T 0609
T 1083
T 0144
T 1416
T 0431
T 1711
0310
1958
1013
1775
1967
1724
T 0805
T 0315
1915
T 0568
T 1080
T 1317
T 1975
T 0944
T 0735
T 1872
T 1266
T 1176
T 1278
T 0559
T 0701
T 0160
T 1867
T 0230
T 0236
T 1049
T 1081
1407
1227
0616
T 0617
1802
0115
1836
T 2006
2000
T 0755
0615
T 0614
T 1294

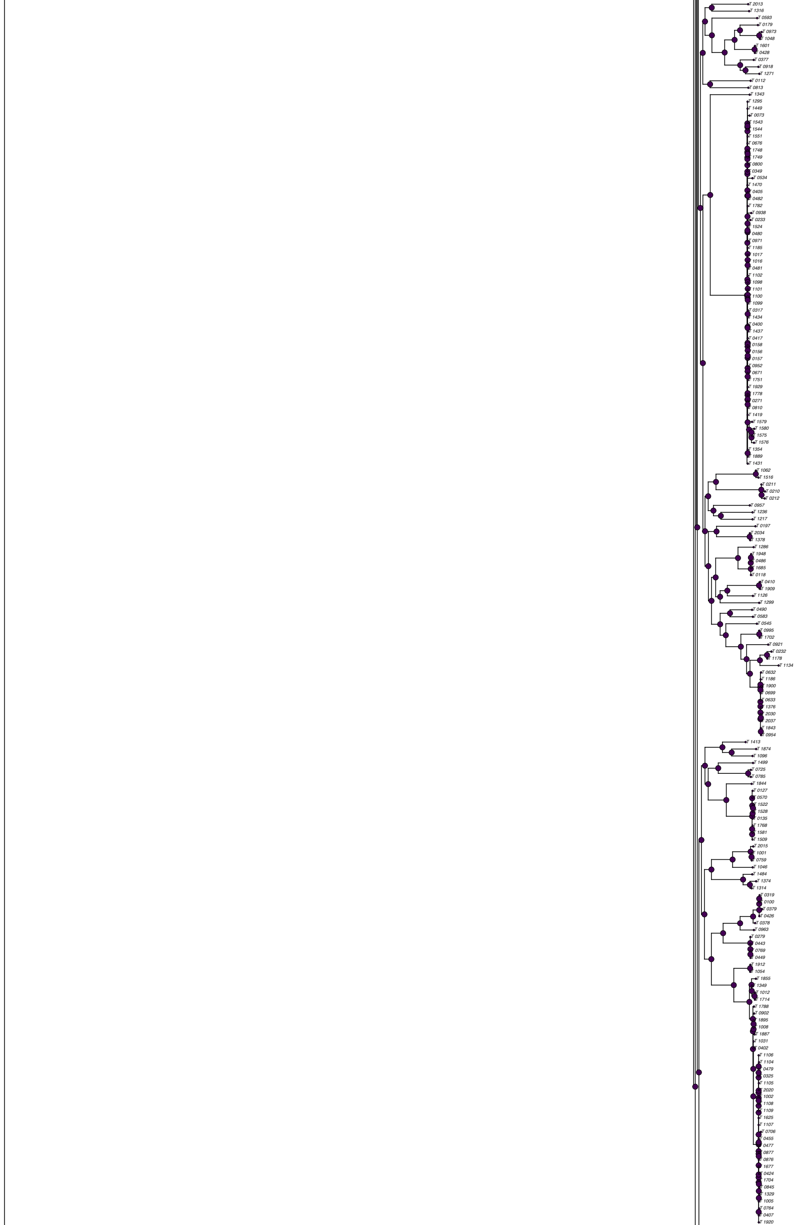
T 2013
T 1316
T 0593
T 0179
T 0973
T 1048
T 1601
T 0428
T 0377
T 0918
T 1271
T 0112
T 0813
T 1343
T 1295
T 1449
T 0073
T 1543
T 1544
T 1551
T 0676
T 1748
T 1749
T 0800
T 0349
T 0534
T 1470
T 0405
T 0482
T 1782
T 0938
T 0233
T 1524
T 0480
T 0971
T 1185
T 1017
T 1016
T 0481
T 1102
T 1098
T 1101
T 1100
T 1099
T 0317
T 1434
T 0400
T 1437
T 0417
T 0158
T 0156
T 0157
T 0952
T 0671
T 1751
T 1929
T 1778
T 0271
T 0810
T 1419
T 1579
T 1580
T 1575
T 1576
T 1354
T 1889
T 1431
T 1062
T 1516
T 0211
T 0210
T 0212
T 0957
T 1236
T 1217
T 0197
T 2034
T 1378
T 1286
T 1948
T 0486
T 1685
T 0118
T 0410
T 1909
T 1126
T 1299
T 0490
T 0583
T 0545
T 0995
T 1702
T 0921
T 0232
T 1178
T 1134
T 0632
T 1186
T 1900
T 0699
T 0633
T 1376
T 2030
T 2037
T 1843
T 0954
T 1413
T 1874
T 1096
T 1499
T 0725
T 0785
T 1844
T 0127
T 0570
T 1522
T 1528
T 0135
T 1768
T 1581
T 1509
T 2015
T 1001
T 0759
T 1046
T 1484
T 1374
T 1314
T 0319
T 0100
T 0379
T 0426
T 0378
T 0963
T 0279
T 0443
T 0769
T 0449
T 1912
T 1054
T 1855
T 1349
T 1012
T 1714
T 1788
T 0902
T 1895
T 1008
T 1887
T 1031
T 0402
T 1106
T 1104
T 0479
T 0325
T 1105
T 2020
T 1002
T 1108
T 1109
T 1625
T 1107
T 0706
T 0455
T 0477
T 0877
T 0876
T 1677
T 0424
T 1704
T 0845
T 1329
T 1005
T 0764
T 0407
T 1920

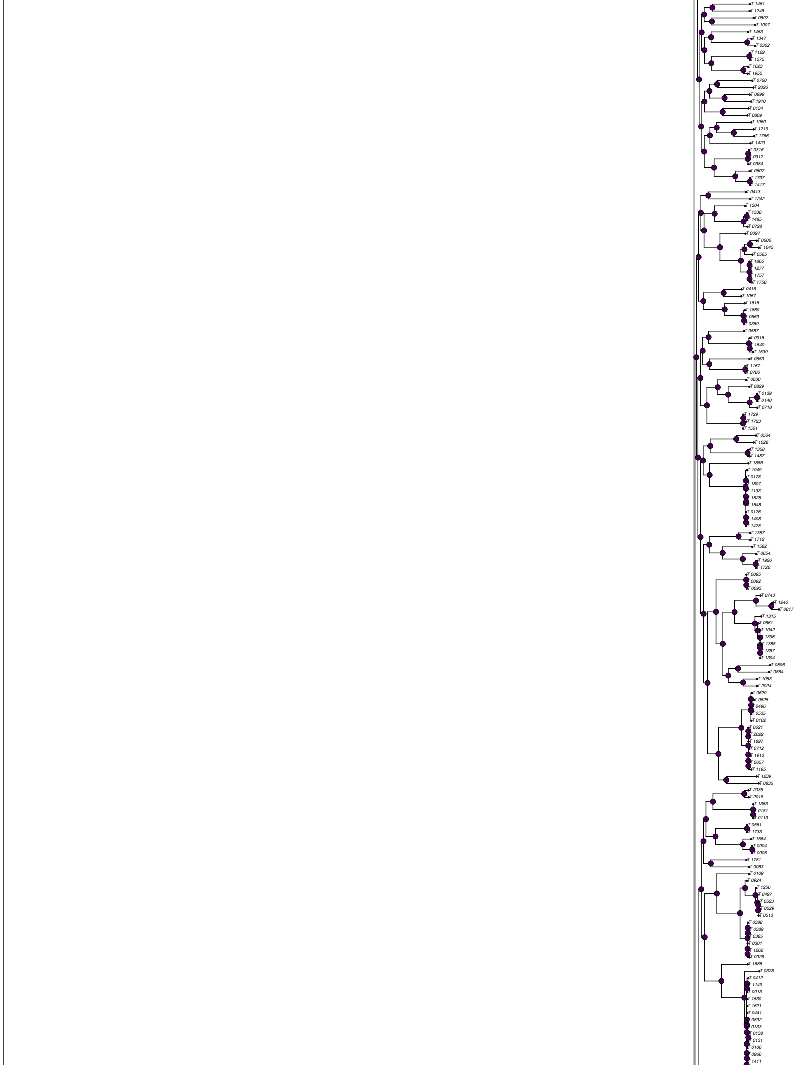

T 1461
T 1245
T 0592
T 1007
T 1463
T 1347
T 0362
T 1129
T 1375
T 1623
T 1955
T 0760
T 2026
T 0999
T 1910
T 0134
T 0809
T 1960
T 1219
T 1766
T 1420
T 0316
T 0312
T 0384
T 0607
T 1737
T 1417
T 0413
T 1242
T 1304
T 1338
T 1485
T 0728
T 0097
T 0606
T 1645
T 0565
T 1865
T 1277
T 1757
T 1758
T 0416
T 1067
T 1616
T 1860
T 0569
T 0359
T 0587
T 0915
T 1540
T 1539
T 0553
T 1167
T 0786
T 0630
T 0829
T 0139
T 0140
T 0718
T 1729
T 1723
T 1561
T 0564
T 1028
T 1358
T 1487
T 1886
T 1949
T 0178
T 1807
T 1133
T 1525
T 1548
T 0126
T 1408
T 1428
T 1357
T 1712
T 1582
T 0654
T 1926
T 1726
T 0095
T 0092
T 0093
T 0743
T 1246
T 0817
T 1315
T 0661
T 1042
T 1396
T 1388
T 1387
T 1394
T 0596
T 0864
T 1053
T 2024
T 0620
T 0525
T 0496
T 0535
T 0102
T 0621
T 2029
T 1897
T 0712
T 1913
T 0657
T 1195
T 1239
T 0835
T 2035
T 2018
T 1363
T 0181
T 0113
T 0561
T 1733
T 1564
T 0904
T 0905
T 1781
T 0083
T 0109
T 0924
T 1256
T 0497
T 0523
T 0529
T 0513
T 0388
T 0389
T 0385
T 0301
T 1262
T 0926
T 1688
T 0328
T 0412
T 1149
T 0913
T 1530
T 1621
T 0441
T 0892
T 0133
T 0138
T 0131
T 0106
T 0966
T 1411

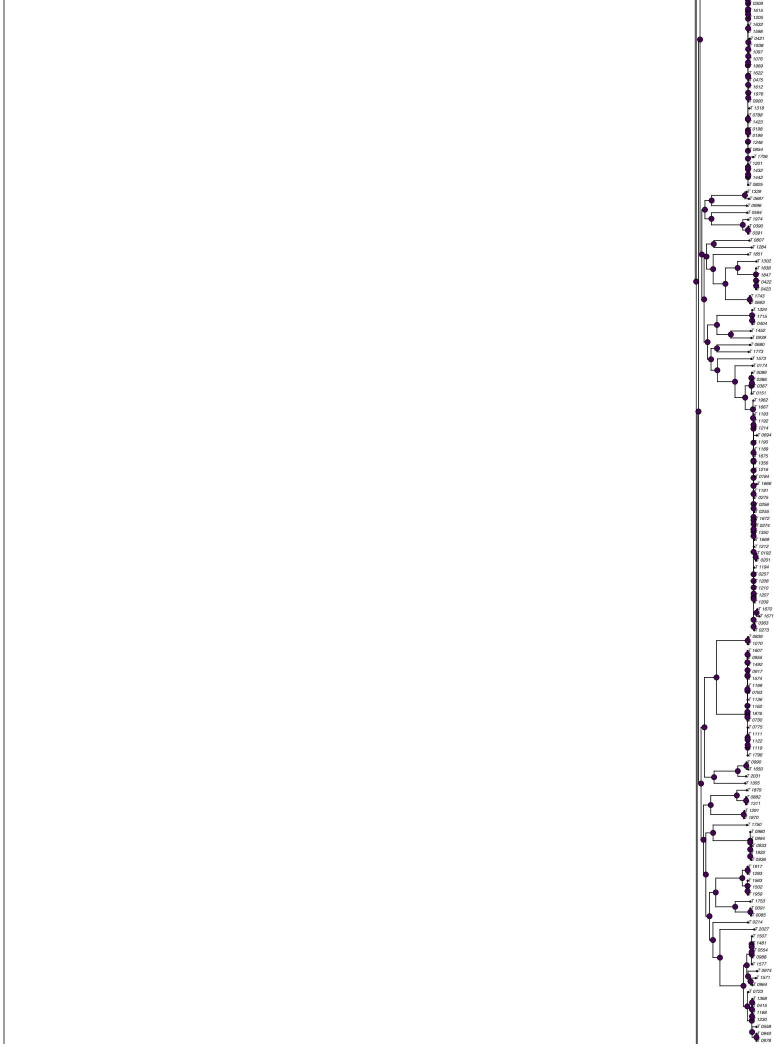
T 0309
T 1615
T 1205
T 1632
T 1598
T 0421
T 1938
T 1097
T 1076
T 1969
T 1622
T 0475
T 1612
T 1976
T 0900
T 1318
T 0788
T 1423
T 0198
T 0199
T 1248
T 0854
T 1706
T 1201
T 1432
T 1442
T 0825
T 1339
T 0687
T 0996
T 0594
T 1974
T 0390
T 0391
T 0807
T 1284
T 1851
T 1302
T 1838
T 1847
T 0422
T 0423
T 1743
T 0693
T 1324
T 1715
T 0404
T 1452
T 0939
T 0680
T 1773
T 1573
T 0174
T 0089
T 0386
T 0387
T 0151
T 1962
T 1667
T 1193
T 1192
T 1214
T 0694
T 1190
T 1189
T 1675
T 1356
T 1216
T 0184
T 1666
T 1191
T 0275
T 0256
T 0255
T 1672
T 0274
T 1350
T 1669
T 1212
T 0192
T 0201
T 1194
T 0257
T 1208
T 1210
T 1207
T 1209
T 1670
T 1671
T 0363
T 0273
T 0839
T 1070
T 1607
T 0955
T 1492
T 0917
T 1574
T 1199
T 0763
T 1136
T 1162
T 1876
T 0730
T 0775
T 1111
T 1122
T 1119
T 1796
T 0990
T 1650
T 2031
T 1305
T 1879
T 0882
T 1311
T 1291
T 1870
T 1750
T 0980
T 0994
T 0933
T 1922
T 0936
T 1917
T 1293
T 1563
T 1502
T 1959
T 1753
T 0091
T 0085
T 0214
T 2027
T 1507
T 1481
T 0554
T 0988
T 1577
T 0974
T 1571
T 0964
T 0723
T 1368
T 0415
T 1168
T 1230
T 0558
T 0940
T 0978

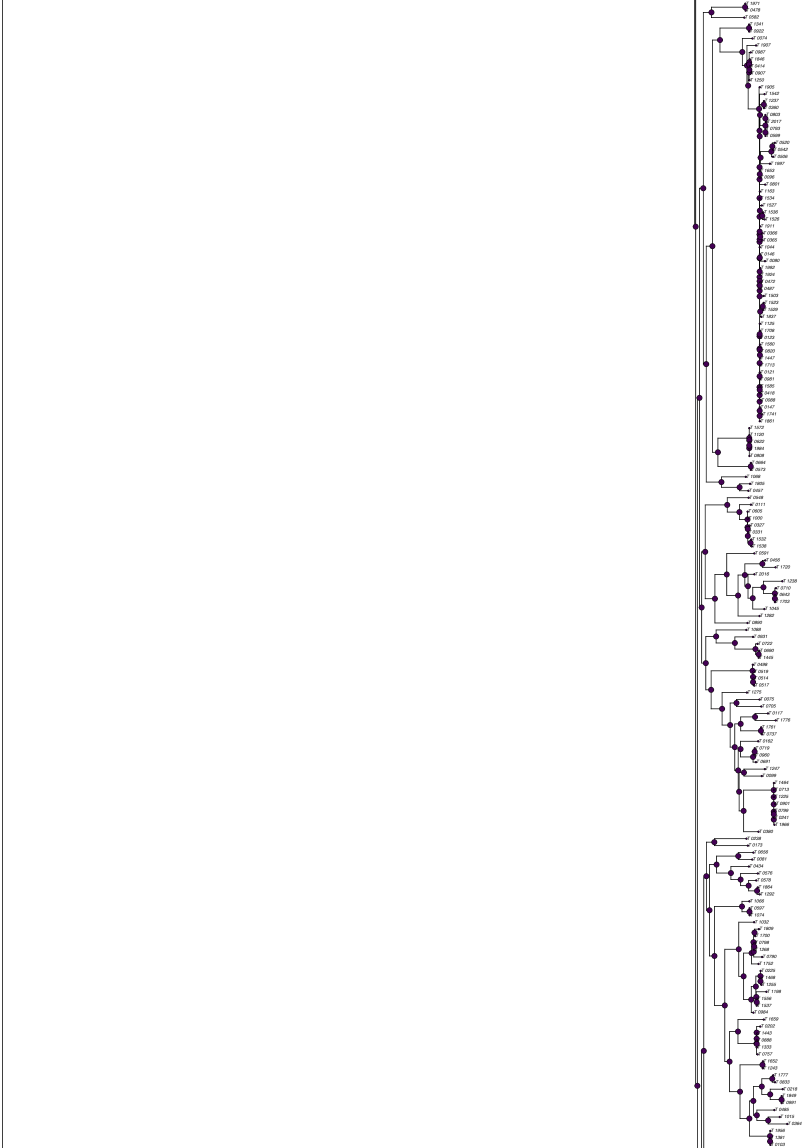

T 1971
T 0478
T 0582
T 1341
T 0922
T 0074
T 1907
T 0987
T 1846
T 0414
T 0907
T 1250
T 1905
T 1542
T 1237
T 0360
T 0803
T 2017
T 0793
T 0599
T 0520
T 0542
T 0506
T 1997
1653
0096
T 0801
T 1163
1534
T 1527
T 1536
T 1526
T 1911
T 0366
T 0365
T 1044
T 0146
T 0080
T 1992
1924
0472
0487
T 1503
T 1523
T 1529
T 1837
T 1125
T 1708
0123
T 1560
0820
T 1447
1713
T 0121
0981
1585
T 0418
T 0088
T 0147
T 1741
T 1861
T 1572
T 1120
0622
1984
T 0808
T 0664
T 0573
T 1068
T 1805
T 0457
T 0548
T 0111
T 0605
1000
T 0327
0331
T 1532
T 1538
T 0591
T 0456
T 1720
T 2016
T 1238
T 0710
0643
T 1703
T 1045
T 1282
T 0890
T 1088
T 0931
T 0722
T 0690
T 1445
T 0498
0519
0514
T 0517
T 1275
T 0075
T 0705
T 0117
T 1776
T 1761
T 0737
T 0162
T 0719
0960
T 0691
T 1247
T 0099
T 1464
0713
1225
0901
T 0799
0241
T 1966
T 0380
T 0238
T 0173
T 0656
T 0081
T 0434
T 0576
T 0578
T 1864
T 1292
T 1066
T 0597
T 1074
T 1032
T 1809
T 1700
0798
1268
T 0790
T 1752
T 0225
1468
T 1255
T 1198
1556
T 1537
T 0984
T 1659
T 0202
1443
0888
1333
T 0757
T 1652
T 1243
T 1777
T 0833
T 0218
T 1849
0991
T 0485
T 1015
T 0364
T 1956
1381
T 0103

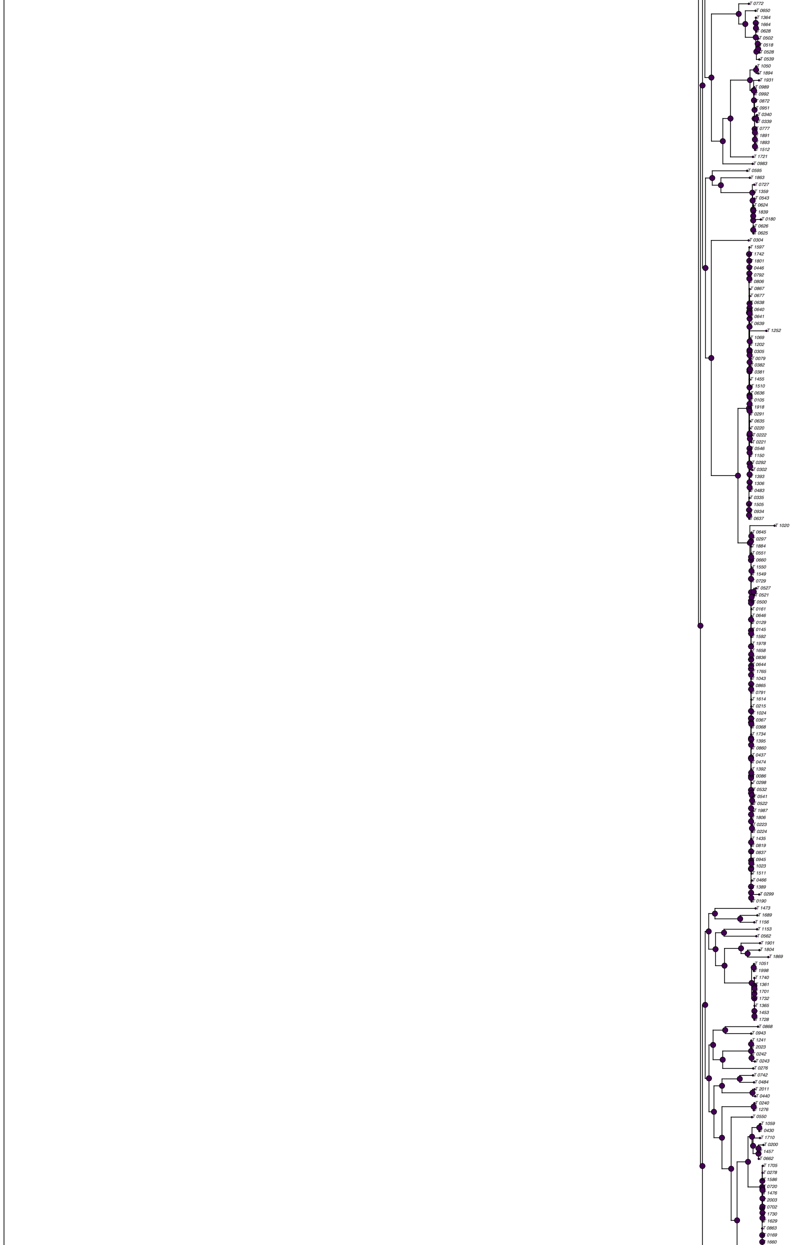
T 0772
T 0650
T 1364
T 1664
T 0628
T 0502
T 0518
T 0528
T 0539
T 1050
T 1894
T 1931
T 0989
T 0992
T 0872
T 0951
T 0340
T 0339
T 0777
T 1891
T 1893
T 1512
T 1721
T 0983
T 0595
T 1863
T 0727
T 1359
T 0543
T 0624
T 1839
T 0180
T 0626
T 0625
T 0304
T 1597
T 1742
T 1801
T 0446
T 0792
T 0806
T 0867
T 0677
T 0638
T 0640
T 0641
T 0639
T 1252
T 1069
T 1202
T 0305
T 0079
T 0382
T 0381
T 1455
T 1510
T 0636
T 0105
T 1918
T 0291
T 0635
T 0220
T 0222
T 0221
T 0546
T 1150
T 0292
T 0302
T 1393
T 1306
T 0483
T 0335
T 1505
T 0934
T 0637
T 1020
T 0645
T 0297
T 1884
T 0551
T 0660
T 1550
T 1549
T 0729
T 0527
T 0521
T 0500
T 0161
T 0646
T 0129
T 0145
T 1592
T 1978
T 1658
T 0836
T 0644
T 1765
T 1043
T 0865
T 0791
T 1614
T 0215
T 1024
T 0367
T 0368
T 1734
T 1395
T 0860
T 0437
T 0474
T 1392
T 0086
T 0298
T 0532
T 0541
T 0522
T 1987
T 1806
T 0223
T 0224
T 1435
T 0819
T 0837
T 0945
T 1023
T 1511
T 0466
T 1389
T 0299
T 0190
T 1473
T 1689
T 1156
T 1153
T 0562
T 1901
T 1804
T 1869
T 1051
T 1998
T 1740
T 1361
T 1701
T 1732
T 1365
T 1453
T 1728
T 0868
T 0943
T 1241
T 2023
T 0242
T 0243
T 0276
T 0742
T 0484
T 2011
T 0440
T 0240
T 1276
T 0550
T 1059
T 0430
T 1710
T 0200
T 1457
T 0662
T 1705
T 0278
T 1586
T 0720
T 1476
T 2003
T 0702
T 1730
T 1629
T 0863
T 0169
T 1660

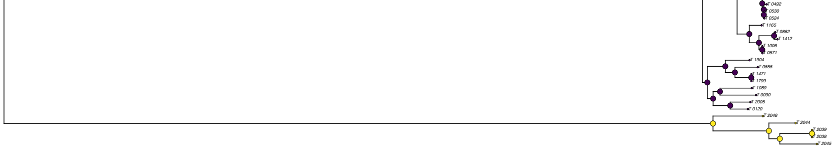
T 0492
T 0530
T 0524
T 1165
T 0862
T 1412
T 1006
T 0571
T 1904
T 0555
T 1471
T 1799
T 1089
T 0090
T 2005
T 0120
T 2048
T 2044
T 2039
T 2038
T 2045

Effector gene: tplE

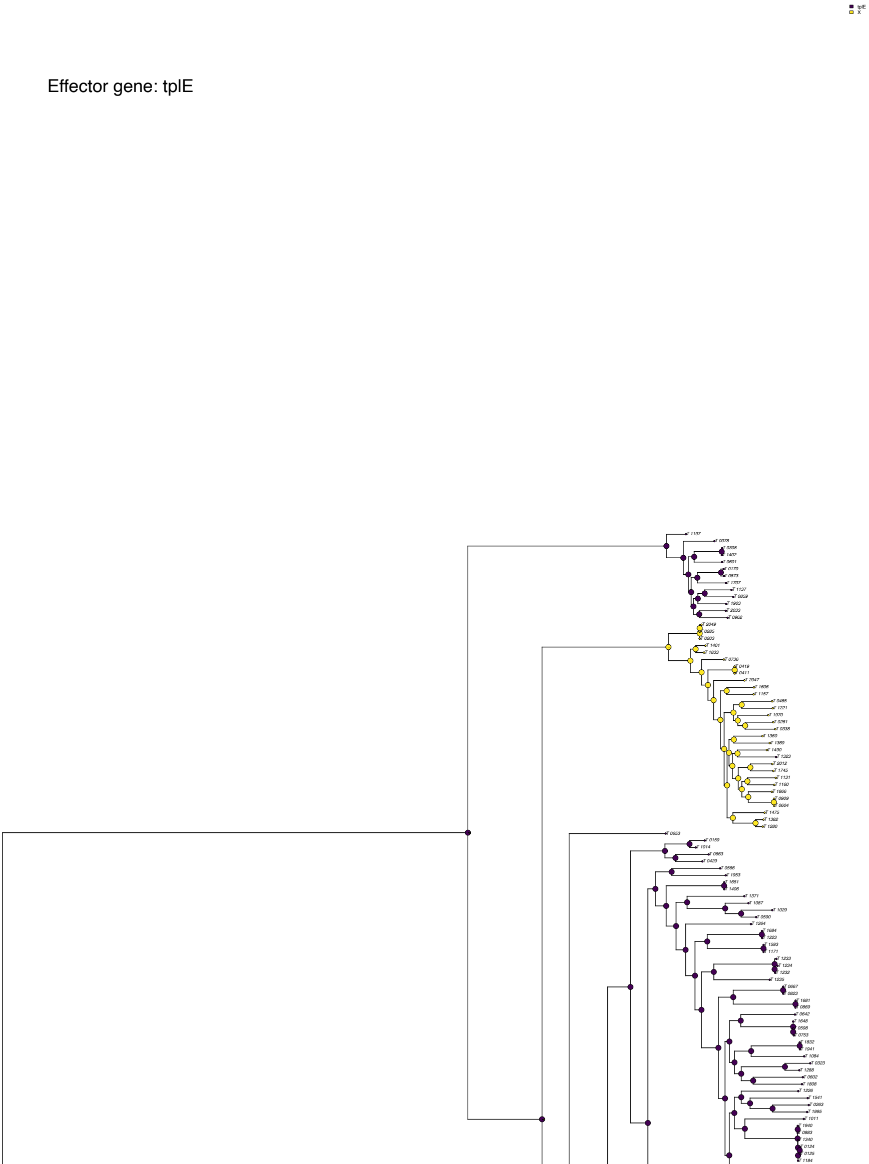

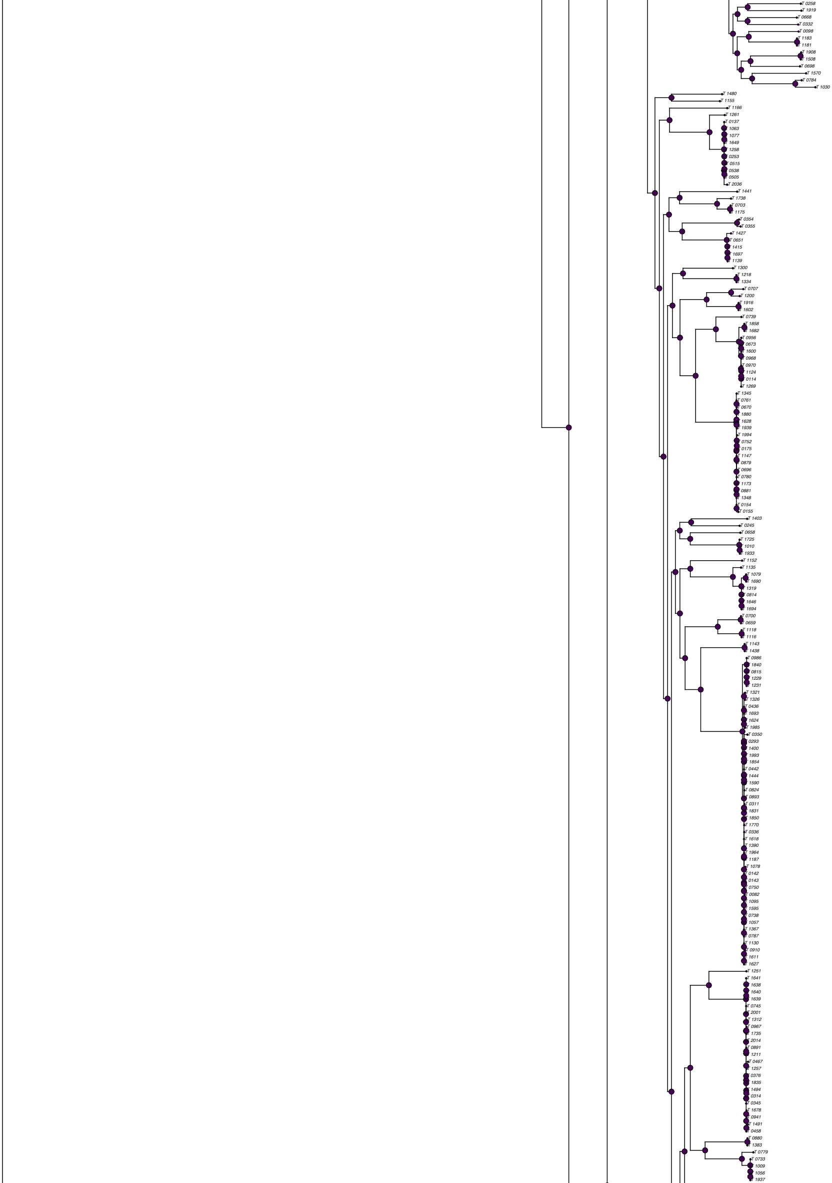
T 0258
T 1919
T 0668
T 0332
T 0098
T 1183
T 1181
T 1908
T 1508
T 0698
T 1570
T 0784
T 1030
T 1480
T 1155
T 1166
T 1261
T 0137
1063
1077
1649
1258
0253
0515
0538
0505
T 2036
T 1441
T 1738
T 0703
T 1175
T 0354
T 0355
T 1427
T 0651
1415
1697
1139
T 1300
T 1218
1334
T 0707
T 1200
T 1916
T 1602
T 0739
T 1858
T 1682
T 0956
0673
T 1600
T 0968
T 0970
T 1124
T 0114
T 1269
T 1345
T 0761
0670
1880
1628
1939
T 1994
0752
0175
T 1147
T 0879
T 0696
T 0780
1173
0881
T 1348
T 0154
T 0155
T 1403
T 0245
T 0658
T 1725
1010
T 1933
T 1152
T 1135
T 1079
T 1690
1319
0814
1646
T 1694
T 0700
T 0659
T 1118
T 1116
T 1143
T 1438
T 0986
1840
0815
1229
T 1231
T 1321
T 1326
T 0436
T 1693
T 1624
T 1985
T 0350
0293
1400
1993
1854
T 0442
1444
1590
T 0824
0893
T 0311
1831
1850
T 1770
T 0336
T 1618
T 1390
T 1964
1187
T 1078
0142
0143
0750
T 0082
1095
1595
T 0738
1057
T 1367
T 0787
T 1130
T 0910
1611
T 1627
T 1251
T 1641
1638
1640
1639
T 0745
T 2001
T 1312
T 0967
T 1735
2014
T 0891
T 1211
T 0467
1257
0376
1835
1494
0314
T 0345
T 1678
0941
T 1491
T 0458
T 0880
T 1383
T 0779
T 0733
1009
1056
1937

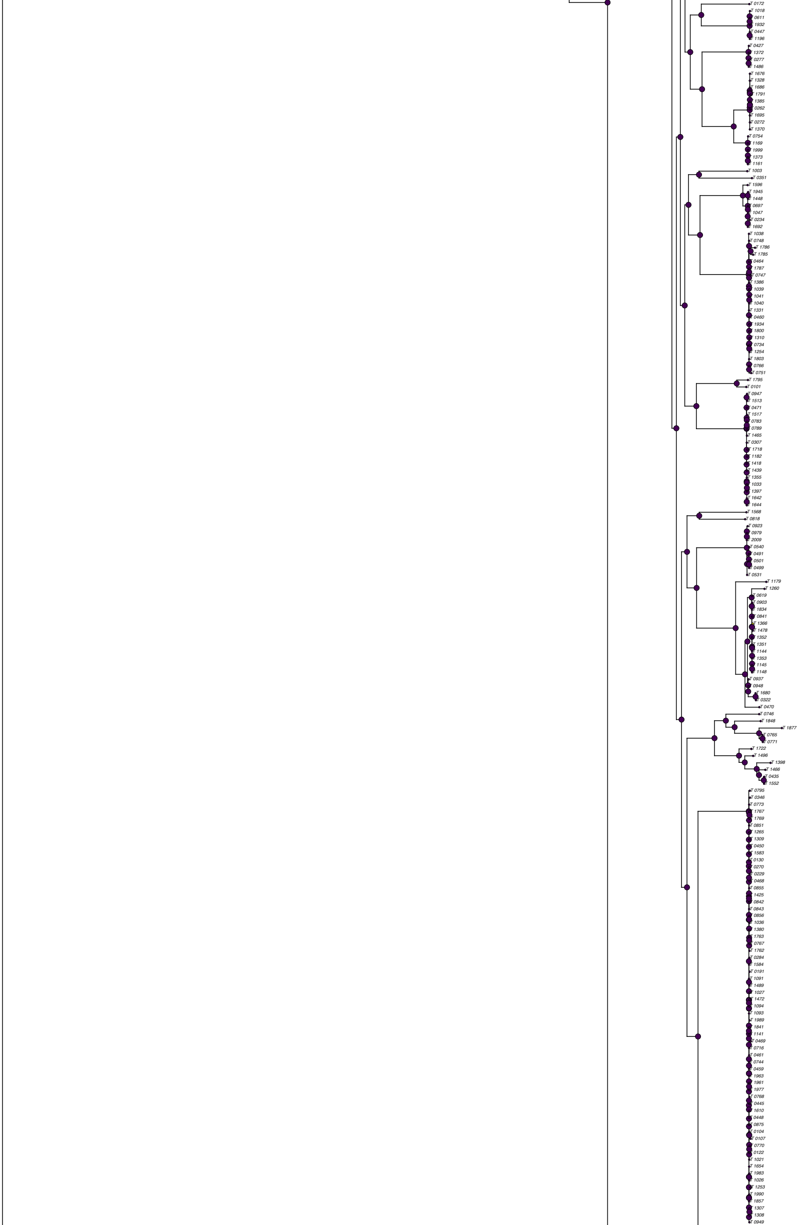
T 0172
T 1018
0611
1932
T 0447
T 1196
T 0427
1372
0277
T 1486
T 1676
T 1328
T 1686
1791
1385
0262
T 1695
T 0272
T 1370
T 0754
1169
1999
1373
T 1161
T 1003
T 0351
T 1596
T 1945
T 1448
0697
T 1047
T 0234
T 1692
T 1038
T 0748
T 1786
T 1785
0464
1787
T 0747
T 1386
1039
1041
1040
T 1331
0460
1934
1800
1310
0734
T 1254
T 1803
0766
T 0751
T 1795
T 0101
T 0947
T 1513
0471
T 1517
0783
0789
T 1465
T 0307
T 1718
T 1182
1418
T 1439
T 1355
1033
1397
T 1642
T 1644
T 1568
T 0818
T 0923
0979
T 2009
T 0540
0491
0501
T 0499
T 0531
T 1179
T 1260
0619
T 0903
T 1834
0841
T 1366
T 1478
1352
1351
1144
1353
1145
T 1148
T 0937
0948
T 1680
T 0322
T 0470
T 0746
T 1848
T 1877
T 0765
T 0771
T 1722
T 1496
T 1398
T 1466
T 0435
T 1552
T 0795
T 0346
T 0773
1767
1769
T 0851
1265
1309
0450
1583
0130
0270
T 0229
0468
T 0855
1425
0842
T 0843
0856
T 1036
1380
T 1763
0767
T 1762
T 0284
1584
T 0191
T 1091
T 1489
1027
T 1472
1094
T 1093
T 1989
1841
1141
T 0469
T 0716
T 0461
0744
0459
1963
1961
1977
T 0768
0445
1610
0448
T 0875
T 0104
T 0107
0770
0122
T 1021
T 1654
T 1983
1026
T 1253
T 1990
T 1857
T 1307
1308
T 0949

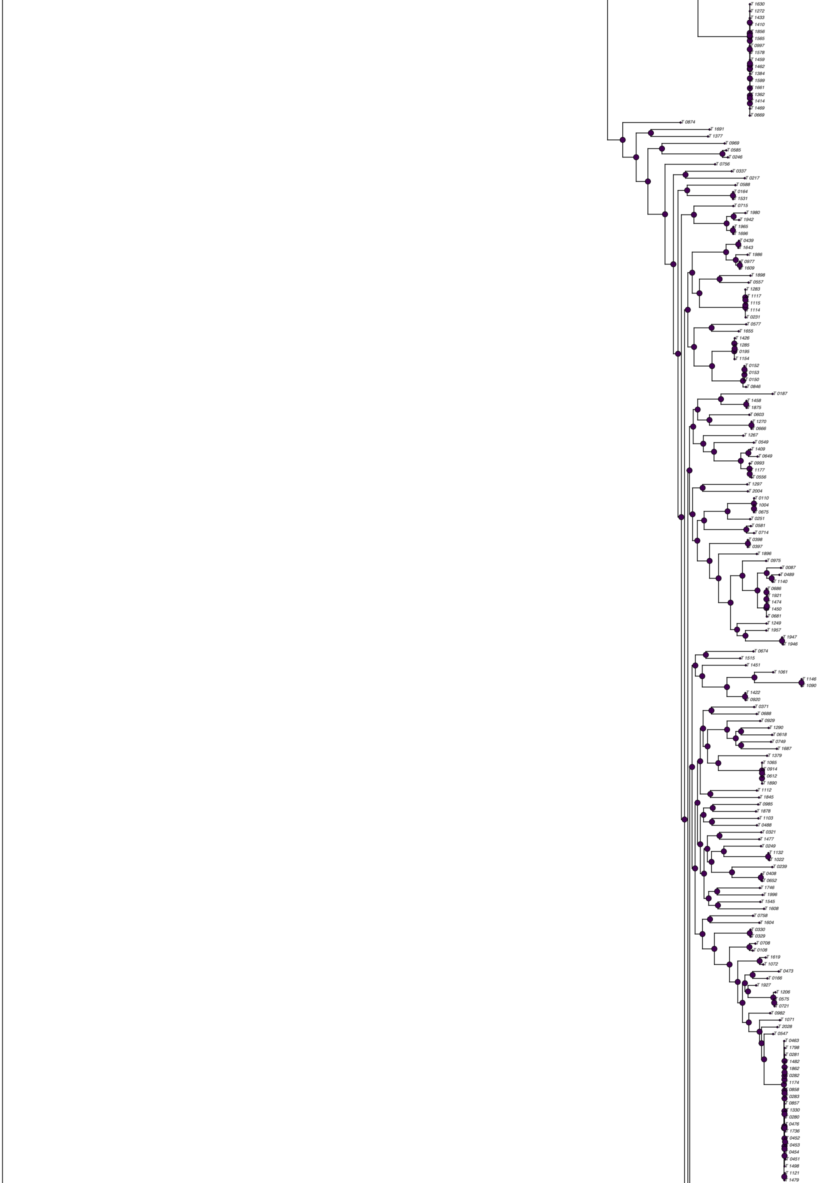
T 1630
T 1272
T 1433
T 1410
T 1856
T 1565
T 0997
T 1578
T 1459
T 1462
T 1384
T 1599
T 1661
T 1362
T 1414
T 1469
T 0669
T 0874
T 1691
T 1377
T 0969
T 0585
T 0246
T 0756
T 0337
T 0217
T 0588
T 0164
T 1531
T 0715
T 1980
T 1942
T 1965
T 1696
T 0439
T 1643
T 1986
T 0977
T 1609
T 1898
T 0557
T 1283
T 1117
T 1115
T 1114
T 0231
T 0577
T 1655
T 1426
T 1285
T 0195
T 1154
T 0152
T 0153
T 0150
T 0846
T 0187
T 1458
T 1875
T 0603
T 1270
T 0666
T 1267
T 0549
T 1409
T 0649
T 0993
T 1177
T 0556
T 1297
T 2004
T 0110
T 1004
T 0675
T 0251
T 0581
T 0714
T 0398
T 0397
T 1896
T 0975
T 0087
T 0489
T 1140
T 0686
T 1921
T 1474
T 1450
T 0681
T 1249
T 1957
T 1947
T 1946
T 0674
T 1515
T 1451
T 1061
T 1146
T 1090
T 1422
T 0920
T 0371
T 0688
T 0929
T 1290
T 0618
T 0749
T 1687
T 1379
T 1065
T 0914
T 0612
T 1890
T 1112
T 1845
T 0985
T 1878
T 1103
T 0488
T 0321
T 1477
T 0249
T 1132
T 1022
T 0239
T 0408
T 0652
T 1746
T 1996
T 1545
T 1608
T 0758
T 1604
T 0330
T 0329
T 0708
T 0108
T 1619
T 1072
T 0473
T 0166
T 1927
T 1206
T 0575
T 0721
T 0982
T 1071
T 2028
T 0547
T 0463
T 1798
T 0281
T 1482
T 1862
T 0282
T 1174
T 0858
T 0283
T 0857
T 1330
T 0280
T 0476
T 1736
T 0452
T 0453
T 0454
T 0451
T 1498
T 1121
T 1479

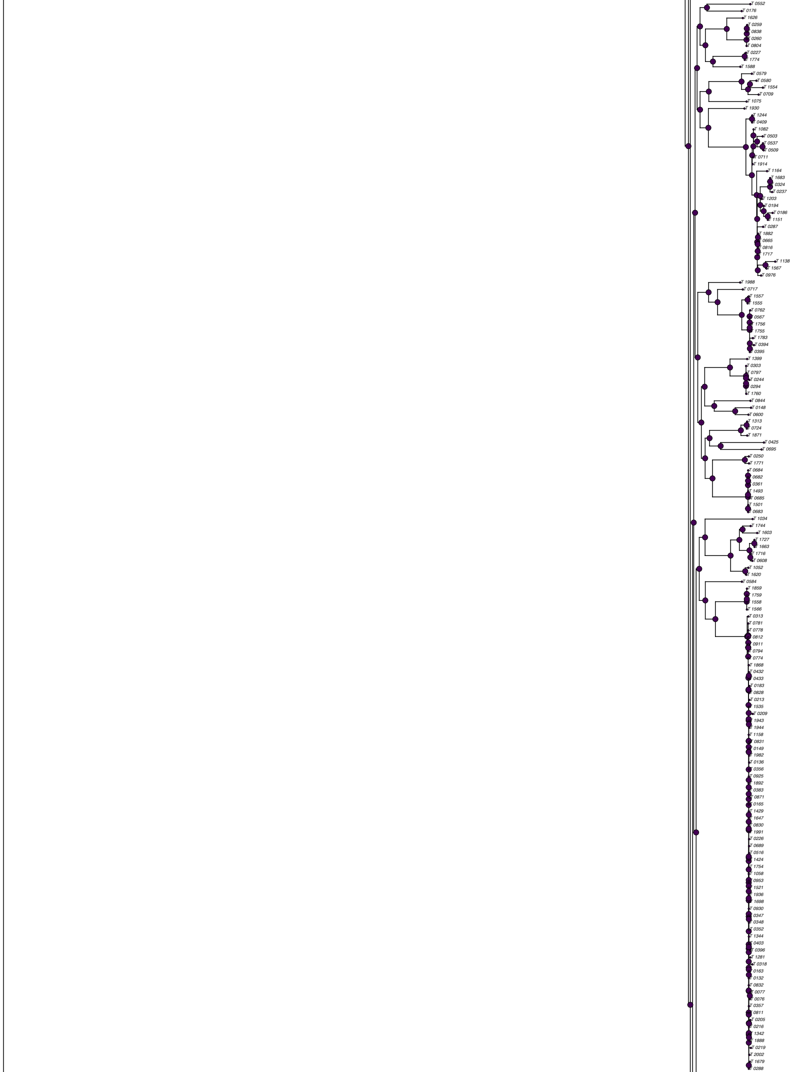
T 0552
T 0176
T 1626
T 0259
0838
0260
0804
T 0227
1774
T 1588
T 0579
T 0580
T 1554
T 0709
T 1075
T 1930
T 1244
0409
T 1082
T 0503
T 0537
T 0509
T 0711
T 1914
T 1164
T 1683
0324
T 0237
T 1203
T 0194
T 0186
1151
T 0287
T 1882
0665
0816
1717
T 1138
T 1567
T 0976
T 1988
T 0717
T 1557
1555
T 0762
0567
1756
1755
T 1783
T 0394
0395
T 1399
T 0303
0797
T 0244
0294
T 1760
T 0844
T 0148
T 0600
T 1313
0724
T 1871
T 0425
T 0695
T 0250
T 1771
T 0684
0682
0361
T 1493
T 0685
T 1501
0683
T 1034
T 1744
T 1603
T 1727
T 1663
T 1716
T 0608
T 1052
T 1620
T 0584
T 1859
1759
1558
T 1566
T 0313
T 0781
T 0778
0812
0911
0794
0774
T 1868
T 0432
0433
T 0183
0828
T 0213
1535
T 0209
1943
1944
T 1158
0831
0149
1982
T 0136
0356
T 0925
1892
0383
T 0871
0165
T 1429
1647
T 0830
1991
T 0226
T 0689
T 0516
1424
T 1754
T 1058
0953
1521
T 1936
1698
T 0930
0347
T 0348
T 0352
T 1344
0403
T 0396
T 1281
T 0318
0163
T 0132
T 0832
T 0077
0076
T 0357
0811
T 0205
T 0216
1342
T 1888
T 0219
T 2002
T 1679
0288

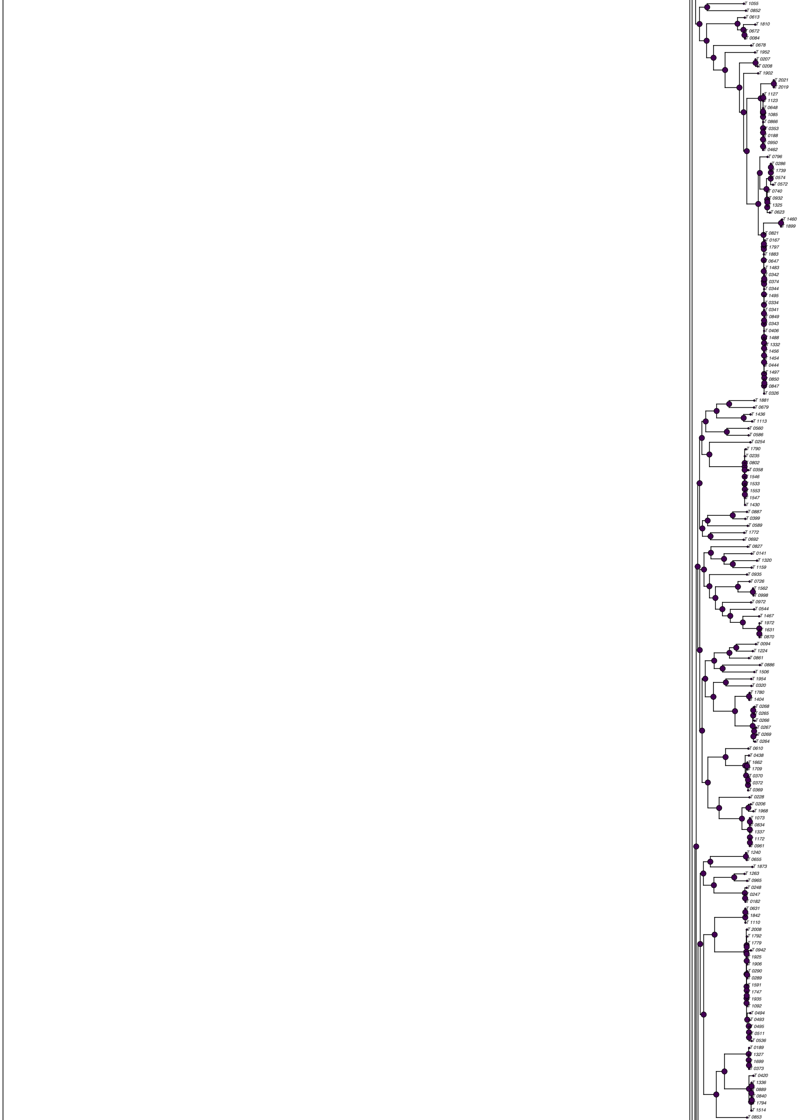
T 1055
T 0852
T 0613
T 1810
T 0672
T 0084
T 0678
T 1952
T 0207
T 0208
T 1902
T 2021
T 2019
T 1127
T 1123
T 0648
T 1085
T 0866
T 0353
T 0188
T 0950
T 0462
T 0796
T 0286
T 1739
T 0574
T 0572
T 0740
T 0932
T 1325
T 0623
T 1460
T 1899
T 0821
T 0167
T 1797
T 1883
T 0647
T 1483
T 0342
T 0374
T 0344
T 1495
T 0334
T 0341
T 0849
T 0343
T 0406
T 1488
T 1332
T 1456
T 1454
T 0444
T 1497
T 0850
T 0847
T 0326
T 1881
T 0679
T 1436
T 1113
T 0560
T 0586
T 0254
T 1790
T 0235
T 0802
T 0358
T 1546
T 1533
T 1553
T 1547
T 1430
T 0887
T 0399
T 0589
T 1772
T 0692
T 0827
T 0141
T 1320
T 1159
T 0935
T 0726
T 1562
T 0998
T 0972
T 0544
T 1467
T 1972
T 1631
T 0870
T 0094
T 1224
T 0861
T 0886
T 1506
T 1954
T 0320
T 1780
T 1404
T 0268
T 0265
T 0266
T 0267
T 0269
T 0264
T 0610
T 0438
T 1662
T 1709
T 0370
T 0372
T 0369
T 0228
T 0206
T 1968
T 1073
T 0834
T 1337
T 1172
T 0961
T 1240
T 0655
T 1873
T 1263
T 0965
T 0248
T 0247
T 0182
T 0631
T 1842
T 1110
T 2008
T 1792
T 1779
T 0942
T 1925
T 1906
T 0290
T 0289
T 1591
T 1747
T 1935
T 1092
T 0494
T 0493
T 0495
T 0511
T 0536
T 0189
T 1327
T 1699
T 0373
T 0420
T 1336
T 0889
T 0840
T 1794
T 1514
T 0853

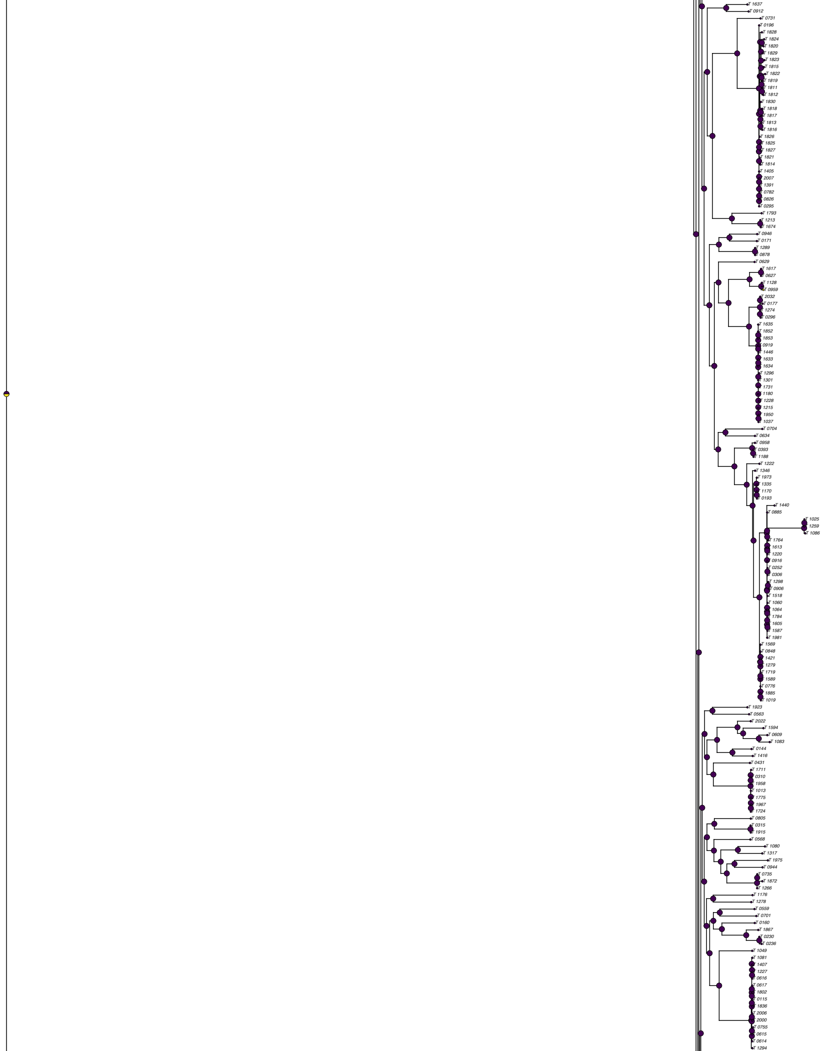

T 1637
T 0912
T 0731
T 0196
T 1828
T 1824
T 1820
T 1829
T 1823
T 1815
T 1822
T 1819
T 1811
T 1812
T 1830
T 1818
T 1817
T 1813
T 1816
T 1826
T 1825
T 1827
T 1821
T 1814
T 1405
2007
1391
0782
0826
T 0295
T 1793
T 1213
T 1674
T 0946
T 0171
T 1289
T 0878
T 0629
T 1617
T 0627
T 1128
T 0959
T 2032
T 0177
T 1274
T 0296
T 1635
T 1852
1853
0919
T 1446
1633
1634
T 1296
T 1301
1731
1180
T 1228
1215
1950
T 1037
T 0704
T 0634
T 0958
T 0393
T 1188
T 1222
T 1346
T 1973
1335
1170
T 0193
T 1440
T 0885
T 1025
1259
T 1086
T 1764
1613
T 1220
0916
T 0252
T 0306
T 1298
T 0906
T 1518
T 1060
1064
T 1784
1605
T 1587
T 1981
T 1569
T 0848
1421
1279
T 1719
1589
T 0776
1885
T 1019
T 1923
T 0563
T 2022
T 1594
T 0609
T 1083
T 0144
T 1416
T 0431
T 1711
0310
1958
T 1013
1775
1967
T 1724
T 0805
T 0315
T 1915
T 0568
T 1080
T 1317
T 1975
T 0944
T 0735
T 1872
T 1266
T 1176
T 1278
T 0559
T 0701
T 0160
T 1867
T 0230
T 0236
T 1049
T 1081
1407
1227
0616
T 0617
1802
T 0115
1836
T 2006
T 2000
T 0755
0615
T 0614
T 1294

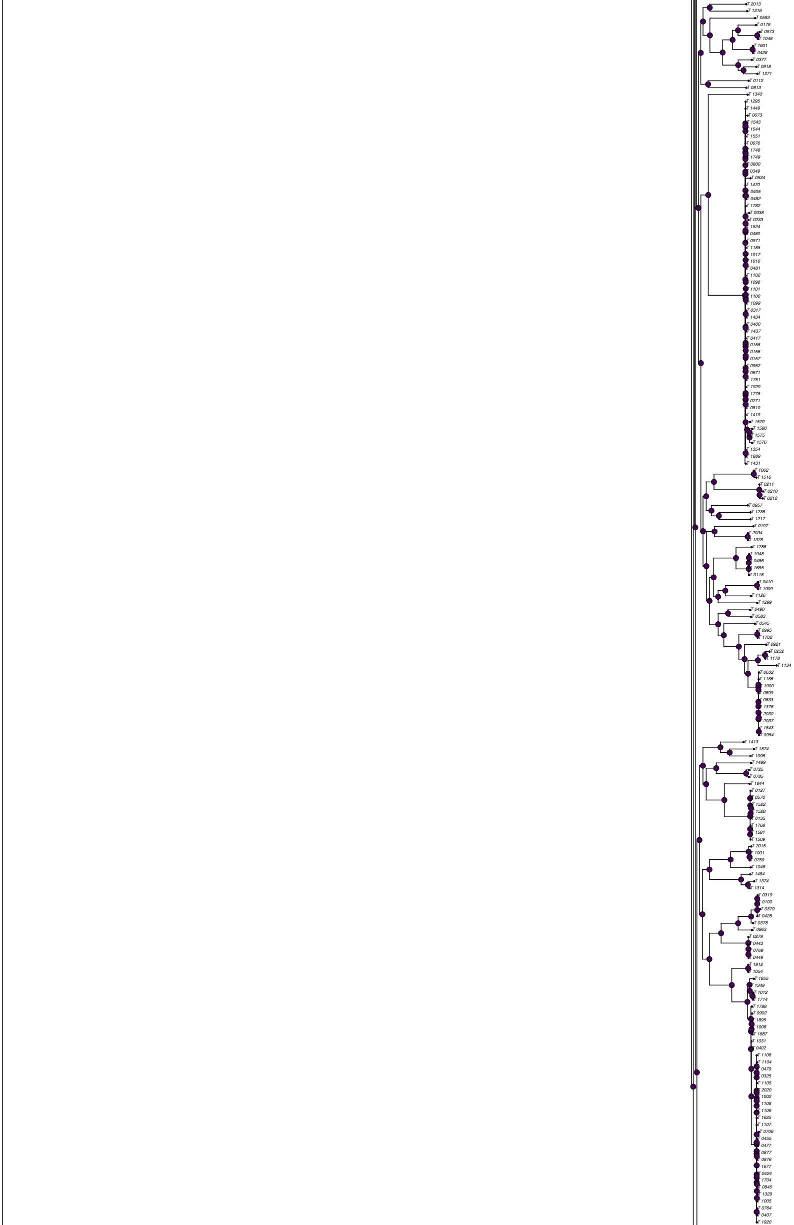

T 2013
T 1316
T 0593
T 0179
T 0973
T 1048
T 1601
T 0428
T 0377
T 0918
T 1271
T 0112
T 0813
T 1343
T 1295
T 1449
T 0073
1543
1544
T 1551
T 0676
1748
1749
0800
0349
T 0534
T 1470
0405
0482
T 1782
T 0938
T 0233
1524
0480
T 0971
T 1185
1017
1016
T 0481
T 1102
1098
1101
1100
T 1099
T 0317
T 1434
T 0400
T 1437
T 0417
0158
0156
0157
T 0952
0671
T 1751
T 1929
1778
0271
T 0810
T 1419
T 1579
T 1580
1575
T 1576
T 1354
T 1889
T 1431
T 1062
T 1516
T 0211
T 0210
T 0212
T 0957
T 1236
T 1217
T 0197
T 2034
T 1378
T 1286
T 1948
0486
1685
T 0118
T 0410
T 1909
T 1126
T 1299
T 0490
T 0583
T 0545
T 0995
T 1702
T 0921
T 0232
T 1178
T 1134
T 0632
T 1186
1900
T 0699
T 0633
1376
2030
2037
T 1843
T 0954
T 1413
T 1874
T 1096
T 1499
T 0725
T 0785
T 1844
T 0127
0570
1522
1528
T 0135
T 1768
1581
T 1509
T 2015
T 1001
T 0759
T 1046
T 1484
T 1374
T 1314
T 0319
0100
T 0379
T 0426
T 0378
T 0963
T 0279
0443
0769
T 0449
T 1912
T 1054
T 1855
1349
T 1012
T 1714
T 1788
T 0902
1895
1008
T 1887
T 1031
0402
T 1106
T 1104
0479
0325
T 1105
2020
1002
1108
T 1109
T 1625
T 1107
T 0706
T 0455
0477
T 0877
T 0876
1677
0424
1704
T 0845
T 1329
T 1005
T 0764
T 0407
T 1920

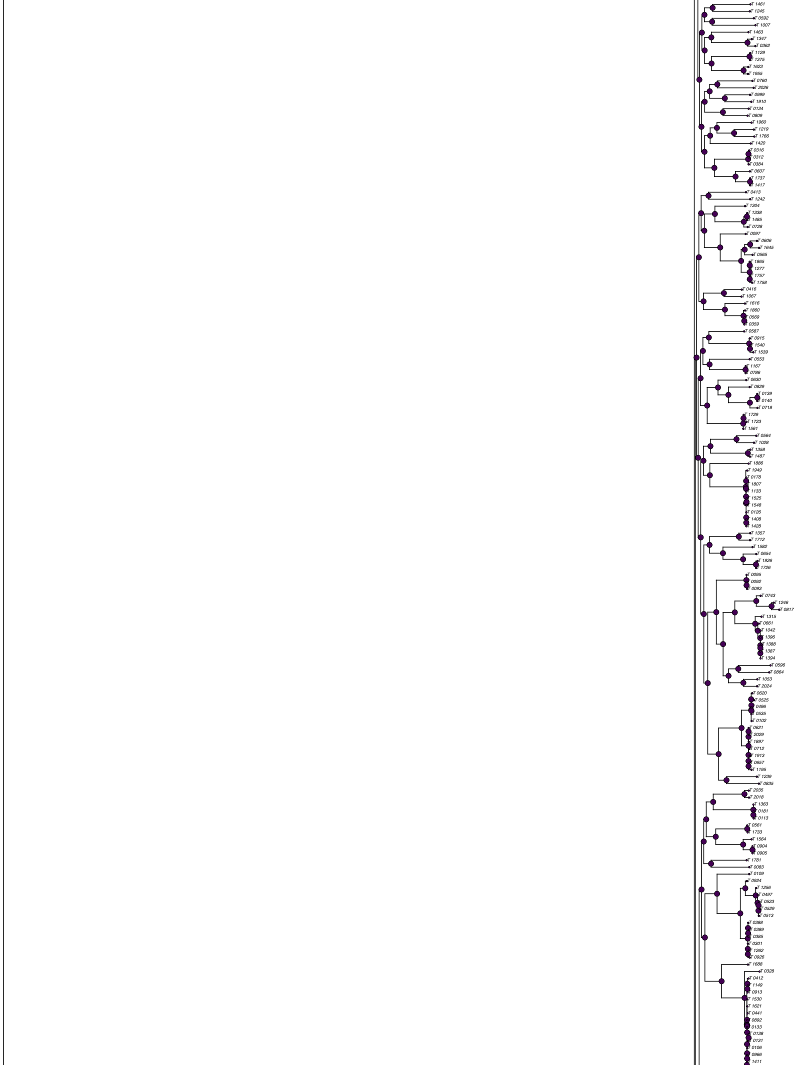
T 1461
T 1245
T 0592
T 1007
T 1463
T 1347
T 0362
T 1129
T 1375
T 1623
T 1955
T 0760
T 2026
T 0999
T 1910
T 0134
T 0809
T 1960
T 1219
T 1766
T 1420
T 0316
T 0312
T 0384
T 0607
T 1737
T 1417
T 0413
T 1242
T 1304
T 1338
T 1485
T 0728
T 0097
T 0606
T 1645
T 0565
T 1865
T 1277
T 1757
T 1758
T 0416
T 1067
T 1616
T 1860
T 0569
T 0359
T 0587
T 0915
T 1540
T 1539
T 0553
T 1167
T 0786
T 0630
T 0829
T 0139
T 0140
T 0718
T 1729
T 1723
T 1561
T 0564
T 1028
T 1358
T 1487
T 1886
T 1949
T 0178
1807
1133
1525
1548
T 0126
1408
T 1428
T 1357
T 1712
T 1582
T 0654
T 1926
T 1726
T 0095
0092
T 0093
T 0743
T 1246
T 0817
T 1315
T 0661
T 1042
1396
T 1388
1387
T 1394
T 0596
T 0864
T 1053
T 2024
T 0620
T 0525
0496
T 0535
T 0102
T 0621
2029
T 1897
T 0712
T 1913
T 0657
T 1195
T 1239
T 0835
T 2035
T 2018
T 1363
0181
T 0113
T 0561
T 1733
T 1564
T 0904
T 0905
T 1781
T 0083
T 0109
T 0924
T 1256
T 0497
T 0523
T 0529
T 0513
T 0388
T 0389
T 0385
T 0301
T 1262
T 0926
T 1688
T 0328
T 0412
1149
T 0913
T 1530
T 1621
T 0441
0892
0133
T 0138
T 0131
T 0106
0966
1411

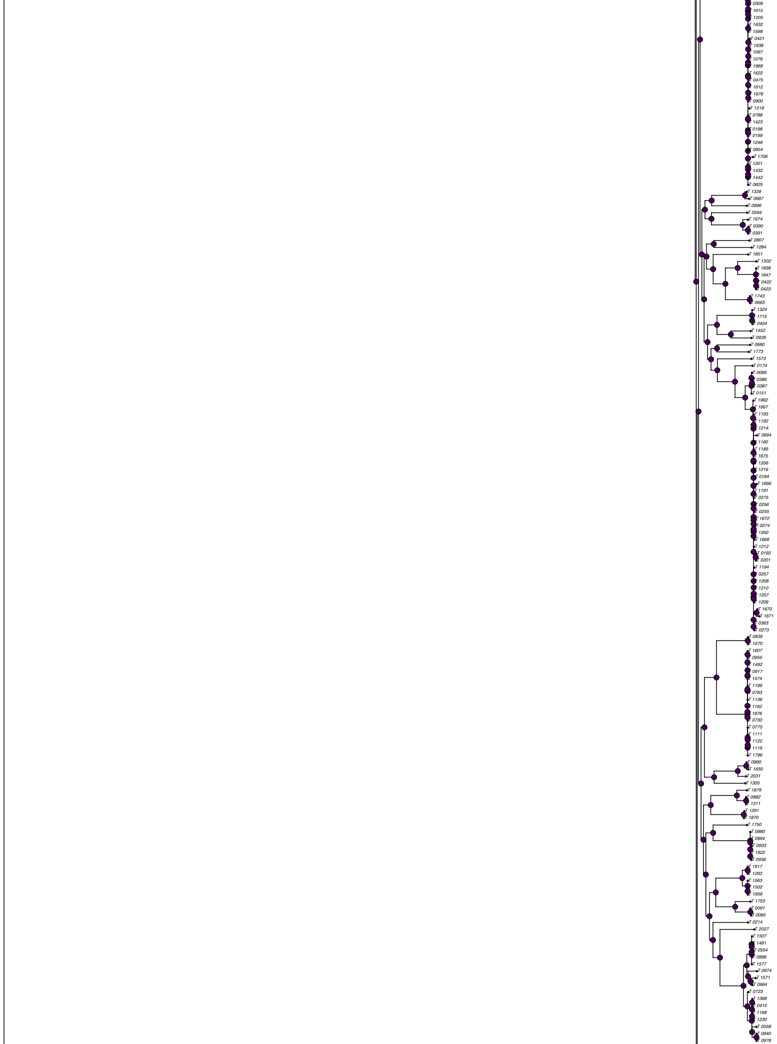
T 0309
T 1615
T 1205
T 1632
T 1598
T 0421
T 1938
T 1097
T 1076
T 1969
T 1622
T 0475
T 1612
T 1976
T 0900
T 1318
T 0788
T 1423
T 0198
T 0199
T 1248
T 0854
T 1706
T 1201
T 1432
T 1442
T 0825
T 1339
T 0687
T 0996
T 0594
T 1974
T 0390
T 0391
T 0807
T 1284
T 1851
T 1302
T 1838
T 1847
T 0422
T 0423
T 1743
T 0693
T 1324
T 1715
T 0404
T 1452
T 0939
T 0680
T 1773
T 1573
T 0174
T 0089
T 0386
T 0387
T 0151
T 1962
T 1667
T 1193
T 1192
T 1214
T 0694
T 1190
T 1189
T 1675
T 1356
T 1216
T 0184
T 1666
T 1191
T 0275
T 0256
T 0255
T 1672
T 0274
T 1350
T 1669
T 1212
T 0192
T 0201
T 1194
T 0257
T 1208
T 1210
T 1207
T 1209
T 1670
T 1671
T 0363
T 0273
T 0839
T 1070
T 1607
T 0955
T 1492
T 0917
T 1574
T 1199
T 0763
T 1136
T 1162
T 1876
T 0730
T 0775
T 1111
T 1122
T 1119
T 1796
T 0990
T 1650
T 2031
T 1305
T 1879
T 0882
T 1311
T 1291
T 1870
T 1750
T 0980
T 0994
T 0933
T 1922
T 0936
T 1917
T 1293
T 1563
T 1502
T 1959
T 1753
T 0091
T 0085
T 0214
T 2027
T 1507
T 1481
T 0554
T 0988
T 1577
T 0974
T 1571
T 0964
T 0723
T 1368
T 0415
T 1168
T 1230
T 0558
T 0940
T 0978

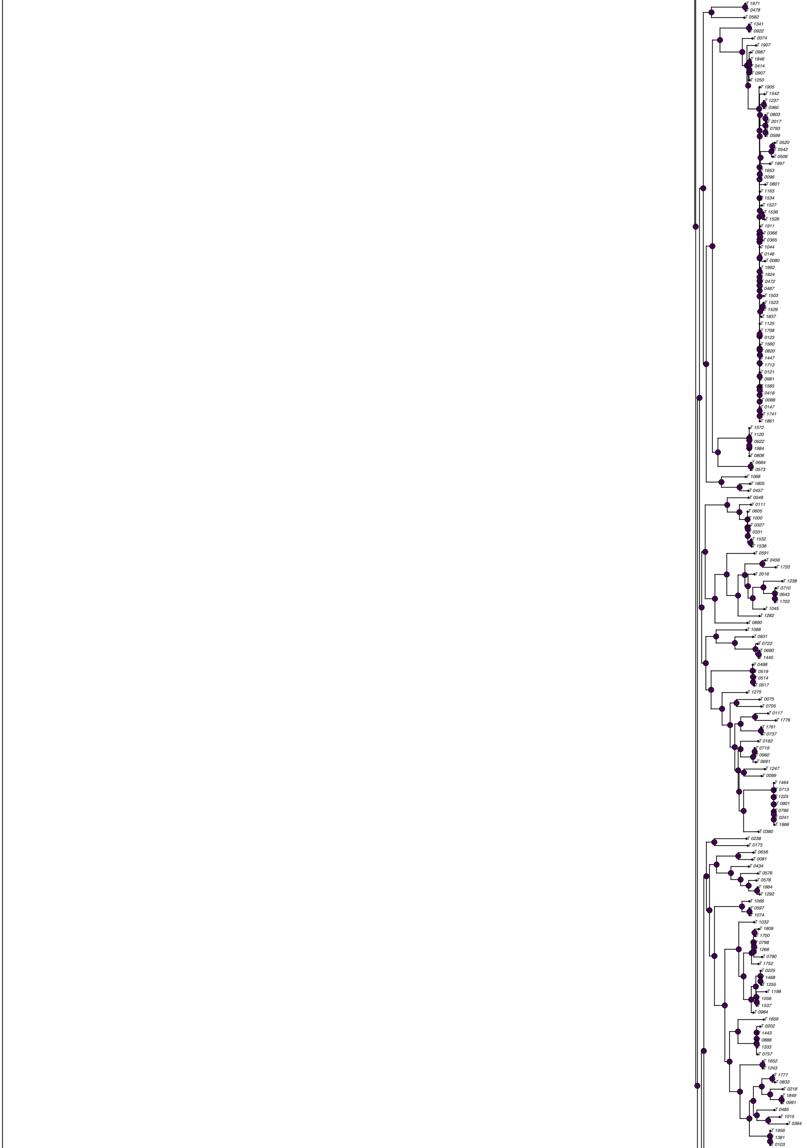
T 1971
T 0478
T 0582
T 1341
T 0922
T 0074
T 1907
T 0987
T 1846
T 0414
T 0907
T 1250
T 1905
T 1542
T 1237
T 0360
T 0803
T 2017
T 0793
T 0599
T 0520
T 0542
T 0506
T 1997
T 1653
T 0096
T 0801
T 1163
T 1534
T 1527
T 1536
T 1526
T 1911
T 0366
T 0365
T 1044
T 0146
T 0080
T 1992
T 1924
T 0472
T 0487
T 1503
T 1523
T 1529
T 1837
T 1125
T 1708
T 0123
T 1560
T 0820
T 1447
T 1713
T 0121
T 0981
T 1585
T 0418
T 0088
T 0147
T 1741
T 1861
T 1572
T 1120
T 0622
T 1984
T 0808
T 0664
T 0573
T 1068
T 1805
T 0457
T 0548
T 0111
T 0605
T 1000
T 0327
T 0331
T 1532
T 1538
T 0591
T 0456
T 1720
T 2016
T 1238
T 0710
T 0643
T 1703
T 1045
T 1282
T 0890
T 1088
T 0931
T 0722
T 0690
T 1445
T 0498
T 0519
T 0514
T 0517
T 1275
T 0075
T 0705
T 0117
T 1776
T 1761
T 0737
T 0162
T 0719
T 0960
T 0691
T 1247
T 0099
T 1464
T 0713
T 1225
T 0901
T 0799
T 0241
T 1966
T 0380
T 0238
T 0173
T 0656
T 0081
T 0434
T 0576
T 0578
T 1864
T 1292
T 1066
T 0597
T 1074
T 1032
T 1809
T 1700
T 0798
T 1268
T 0790
T 1752
T 0225
T 1468
T 1255
T 1198
T 1556
T 1537
T 0984
T 1659
T 0202
T 1443
T 0888
T 1333
T 0757
T 1652
T 1243
T 1777
T 0833
T 0218
T 1849
T 0991
T 0485
T 1015
T 0364
T 1956
T 1381
T 0103

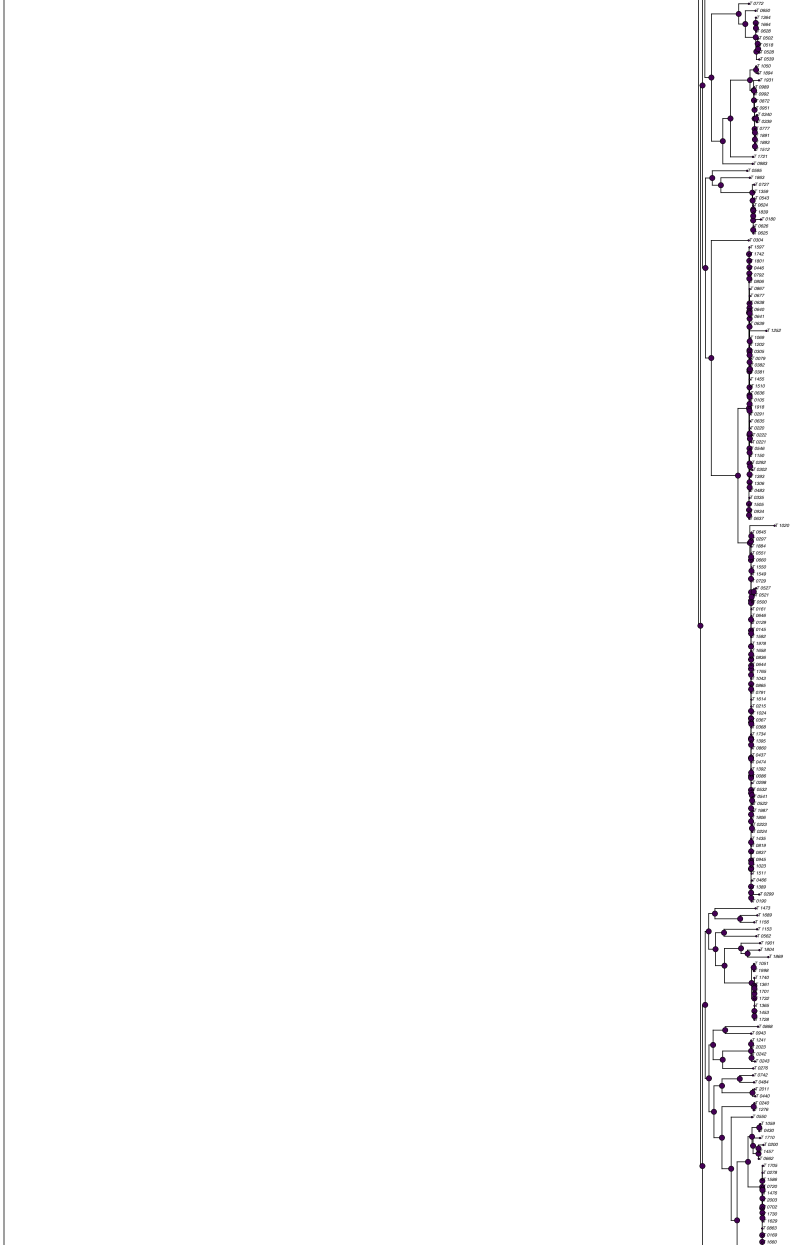

T 0772
T 0650
T 1364
1664
0628
T 0502
0518
T 0528
T 0539
T 1050
T 1894
T 1931
T 0989
0992
0872
T 0951
T 0340
T 0339
0777
1891
1893
T 1512
T 1721
T 0983
T 0595
T 1863
T 0727
T 1359
T 0543
T 0624
1839
T 0180
T 0626
T 0625
T 0304
T 1597
1742
1801
0446
0792
T 0806
T 0867
T 0677
0638
0640
0641
T 0639
T 1252
T 1069
T 1202
0305
T 0079
0382
0381
T 1455
1510
T 0636
0105
1918
T 0291
T 0635
T 0220
T 0222
T 0221
0546
T 1150
T 0292
T 0302
1393
1306
T 0483
T 0335
1505
0934
T 0637
T 1020
T 0645
0297
T 1884
T 0551
0660
T 1550
1549
0729
T 0527
T 0521
0500
T 0161
0646
T 0129
T 0145
T 1592
T 1978
1658
0836
0644
T 1765
T 1043
0865
T 0791
T 1614
T 0215
1024
0367
T 0368
T 1734
1395
T 0860
0437
T 0474
T 1392
0086
T 0298
T 0532
T 0541
T 0522
T 1987
1806
T 0223
T 0224
T 1435
T 0819
T 0837
0945
1023
T 1511
T 0466
1389
T 0299
T 0190
T 1473
T 1689
T 1156
T 1153
T 0562
T 1901
T 1804
T 1869
T 1051
T 1998
T 1740
1361
1701
1732
T 1365
1453
T 1728
T 0868
T 0943
T 1241
2023
0242
T 0243
T 0276
T 0742
T 0484
T 2011
T 0440
T 0240
T 1276
T 0550
T 1059
0430
T 1710
T 0200
1457
T 0662
T 1705
T 0278
1586
0720
T 1476
2003
0702
1730
T 1629
T 0863
0169
1660

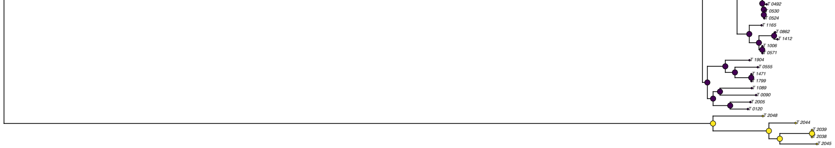
T 0492
T 0530
T 0524
T 1165
T 0862
T 1412
T 1006
T 0571
T 1904
T 0555
T 1471
T 1799
T 1089
T 0090
T 2005
T 0120
T 2048
T 2044
T 2039
T 2038
T 2045

tseT
X

Effector gene: tseT

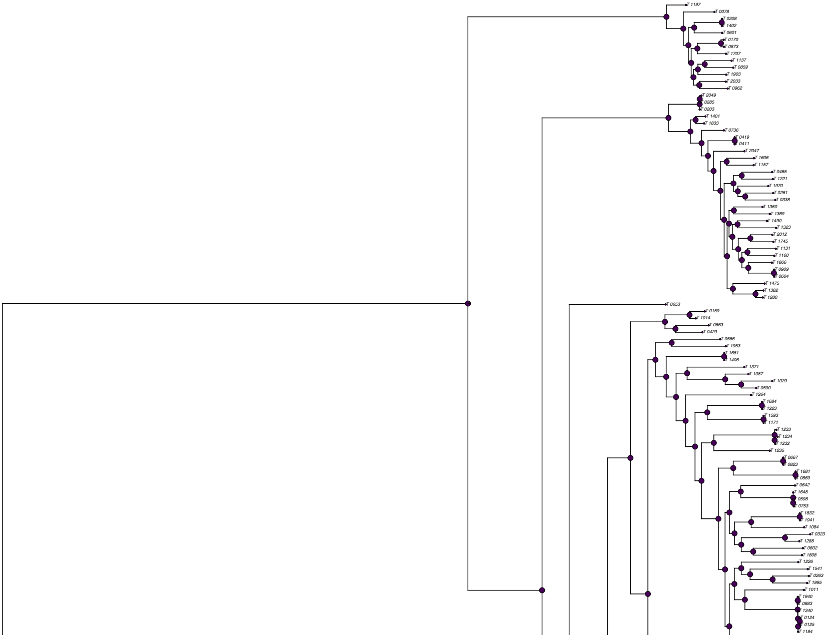

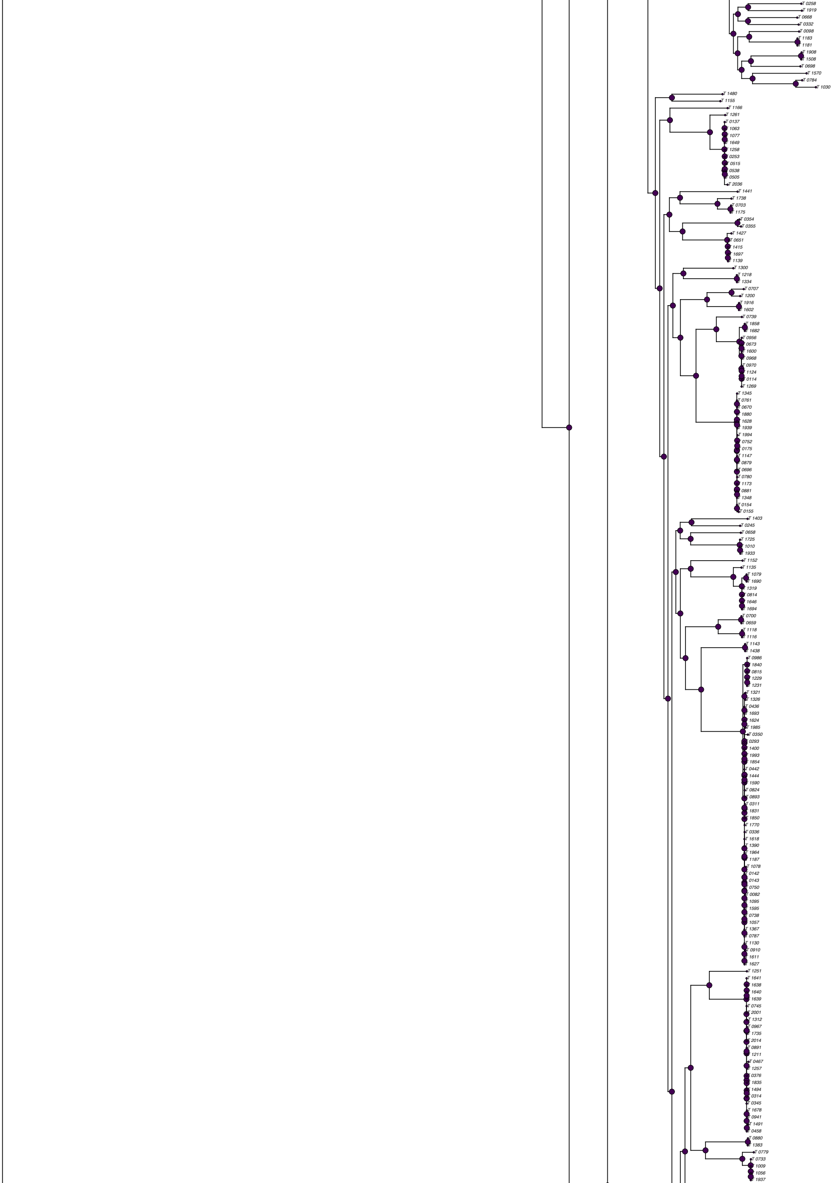
T 0258
T 1919
T 0668
T 0332
T 0098
T 1183
T 1181
T 1908
T 1508
T 0698
T 1570
T 0784
T 1030
T 1480
T 1155
T 1166
T 1261
T 0137
T 1063
T 1077
T 1649
T 1258
T 0253
T 0515
T 0538
T 0505
T 2036
T 1441
T 1738
T 0703
T 1175
T 0354
T 0355
T 1427
T 0651
T 1415
T 1697
T 1139
T 1300
T 1218
T 1334
T 0707
T 1200
T 1916
T 1602
T 0739
T 1858
T 1682
T 0956
T 0673
T 1600
T 0968
T 0970
T 1124
T 0114
T 1269
T 1345
T 0761
T 0670
T 1880
T 1628
T 1939
T 1994
T 0752
T 0175
T 1147
T 0879
T 0696
T 0780
T 1173
T 0881
T 1348
T 0154
T 0155
T 1403
T 0245
T 0658
T 1725
T 1010
T 1933
T 1152
T 1135
T 1079
T 1690
T 1319
T 0814
T 1646
T 1694
T 0700
T 0659
T 1118
T 1116
T 1143
T 1438
T 0986
T 1840
T 0815
T 1229
T 1231
T 1321
T 1326
T 0436
T 1693
T 1624
T 1985
T 0350
T 0293
T 1400
T 1993
T 1854
T 0442
T 1444
T 1590
T 0824
T 0893
T 0311
T 1831
T 1850
T 1770
T 0336
T 1618
T 1390
T 1964
T 1187
T 1078
T 0142
T 0143
T 0750
T 0082
T 1095
T 1595
T 0738
T 1057
T 1367
T 0787
T 1130
T 0910
T 1611
T 1627
T 1251
T 1641
T 1638
T 1640
T 1639
T 0745
T 2001
T 1312
T 0967
T 1735
T 2014
T 0891
T 1211
T 0467
T 1257
T 0376
T 1835
T 1494
T 0314
T 0345
T 1678
T 0941
T 1491
T 0458
T 0880
T 1383
T 0779
T 0733
T 1009
T 1056
T 1937

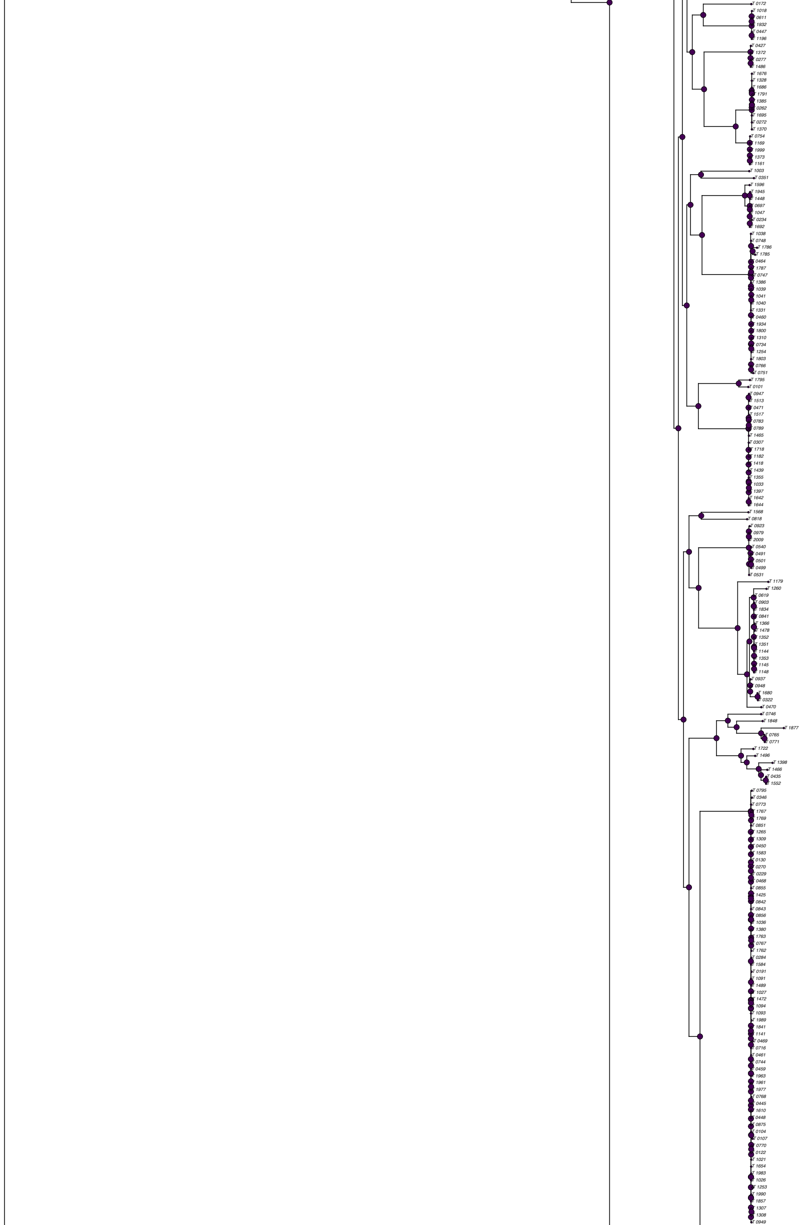
T 0172
T 1018
0611
1932
T 0447
T 1196
T 0427
1372
T 0277
T 1486
T 1676
T 1328
T 1686
1791
1385
T 0262
T 1695
T 0272
T 1370
T 0754
1169
1999
1373
T 1161
T 1003
T 0351
T 1596
T 1945
T 1448
0697
T 1047
T 0234
T 1692
T 1038
T 0748
T 1786
T 1785
0464
1787
T 0747
T 1386
1039
1041
1040
T 1331
0460
1934
1800
1310
0734
T 1254
T 1803
0766
T 0751
T 1795
T 0101
T 0947
T 1513
0471
T 1517
0783
0789
T 1465
T 0307
T 1718
T 1182
1418
T 1439
T 1355
1033
1397
T 1642
T 1644
T 1568
T 0818
T 0923
0979
T 2009
T 0540
0491
0501
T 0499
T 0531
T 1179
T 1260
0619
T 0903
T 1834
0841
T 1366
T 1478
1352
1351
1144
1353
1145
T 1148
T 0937
0948
T 1680
T 0322
T 0470
T 0746
T 1848
T 1877
T 0765
T 0771
T 1722
T 1496
T 1398
T 1466
T 0435
T 1552
T 0795
T 0346
T 0773
1767
1769
T 0851
1265
1309
0450
1583
T 0130
T 0270
T 0229
T 0468
T 0855
1425
0842
T 0843
0856
T 1036
1380
T 1763
0767
T 1762
T 0284
1584
T 0191
T 1091
1489
1027
T 1472
1094
T 1093
T 1989
1841
1141
T 0469
T 0716
T 0461
0744
T 0459
1963
1961
1977
T 0768
0445
1610
0448
T 0875
T 0104
T 0107
0770
0122
T 1021
T 1654
T 1983
1026
T 1253
T 1990
T 1857
T 1307
1308
T 0949

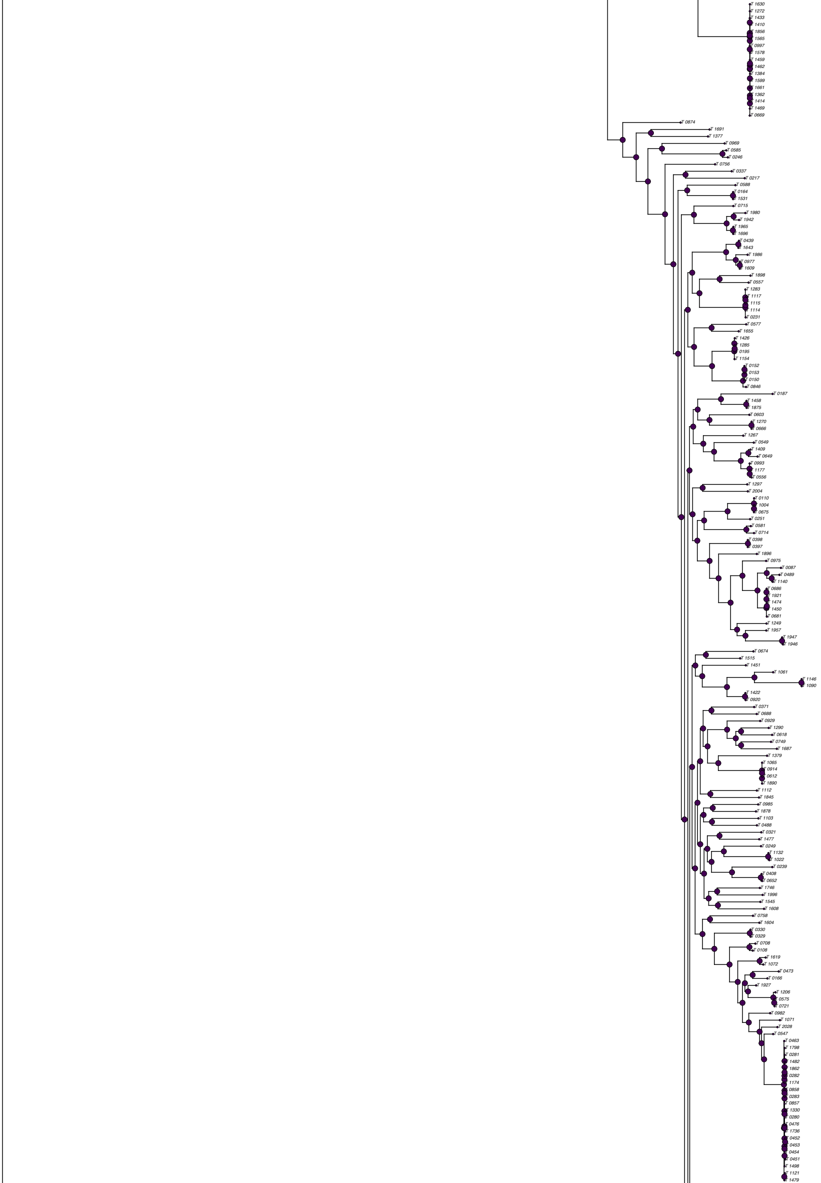
T 1630
T 1272
T 1433
T 1410
T 1856
T 1565
T 0997
T 1578
T 1459
T 1462
T 1384
T 1599
T 1661
T 1362
T 1414
T 1469
T 0669
T 0874
T 1691
T 1377
T 0969
T 0585
T 0246
T 0756
T 0337
T 0217
T 0588
T 0164
T 1531
T 0715
T 1980
T 1942
T 1965
T 1696
T 0439
T 1643
T 1986
T 0977
T 1609
T 1898
T 0557
T 1283
T 1117
T 1115
T 1114
T 0231
T 0577
T 1655
T 1426
T 1285
T 0195
T 1154
T 0152
T 0153
T 0150
T 0846
T 0187
T 1458
T 1875
T 0603
T 1270
T 0666
T 1267
T 0549
T 1409
T 0649
T 0993
T 1177
T 0556
T 1297
T 2004
T 0110
T 1004
T 0675
T 0251
T 0581
T 0714
T 0398
T 0397
T 1896
T 0975
T 0087
T 0489
T 1140
T 0686
T 1921
T 1474
T 1450
T 0681
T 1249
T 1957
T 1947
T 1946
T 0674
T 1515
T 1451
T 1061
T 1146
T 1090
T 1422
T 0920
T 0371
T 0688
T 0929
T 1290
T 0618
T 0749
T 1687
T 1379
T 1065
T 0914
T 0612
T 1890
T 1112
T 1845
T 0985
T 1878
T 1103
T 0488
T 0321
T 1477
T 0249
T 1132
T 1022
T 0239
T 0408
T 0652
T 1746
T 1996
T 1545
T 1608
T 0758
T 1604
T 0330
T 0329
T 0708
T 0108
T 1619
T 1072
T 0473
T 0166
T 1927
T 1206
T 0575
T 0721
T 0982
T 1071
T 2028
T 0547
T 0463
T 1798
T 0281
T 1482
T 1862
T 0282
T 1174
T 0858
T 0283
T 0857
T 1330
T 0280
T 0476
T 1736
T 0452
T 0453
T 0454
T 0451
T 1498
T 1121
T 1479

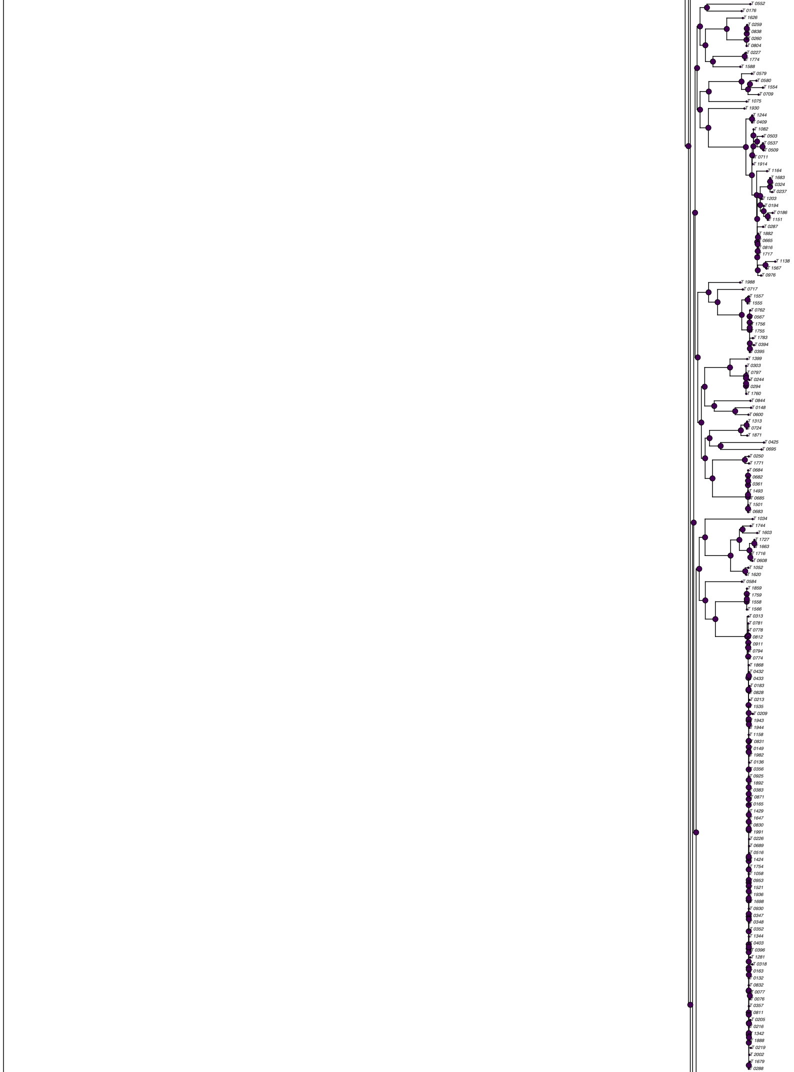

T 0552
T 0176
T 1626
T 0259
0838
0260
T 0804
T 0227
T 1774
T 1588
T 0579
T 0580
T 1554
T 0709
T 1075
T 1930
T 1244
T 0409
T 1082
T 0503
T 0537
T 0509
T 0711
T 1914
T 1164
T 1683
0324
T 0237
T 1203
T 0194
T 0186
T 1151
T 0287
T 1882
0665
T 0816
1717
T 1138
T 1567
T 0976
T 1988
T 0717
T 1557
T 1555
T 0762
0567
T 1756
1755
T 1783
T 0394
T 0395
T 1399
T 0303
0797
T 0244
0294
T 1760
T 0844
T 0148
T 0600
T 1313
T 0724
T 1871
T 0425
T 0695
T 0250
T 1771
T 0684
0682
0361
T 1493
T 0685
T 1501
T 0683
T 1034
T 1744
T 1603
T 1727
T 1663
T 1716
T 0608
T 1052
T 1620
T 0584
T 1859
1759
1558
T 1566
T 0313
T 0781
T 0778
0812
0911
T 0794
0774
T 1868
T 0432
0433
T 0183
T 0828
T 0213
1535
T 0209
1943
T 1944
T 1158
0831
0149
T 1982
T 0136
0356
T 0925
T 1892
0383
T 0871
0165
T 1429
T 1647
T 0830
1991
T 0226
T 0689
T 0516
1424
T 1754
T 1058
0953
1521
T 1936
1698
T 0930
0347
T 0348
T 0352
T 1344
T 0403
T 0396
T 1281
T 0318
0163
T 0132
T 0832
T 0077
T 0076
T 0357
0811
T 0205
T 0216
1342
T 1888
T 0219
T 2002
T 1679
T 0288

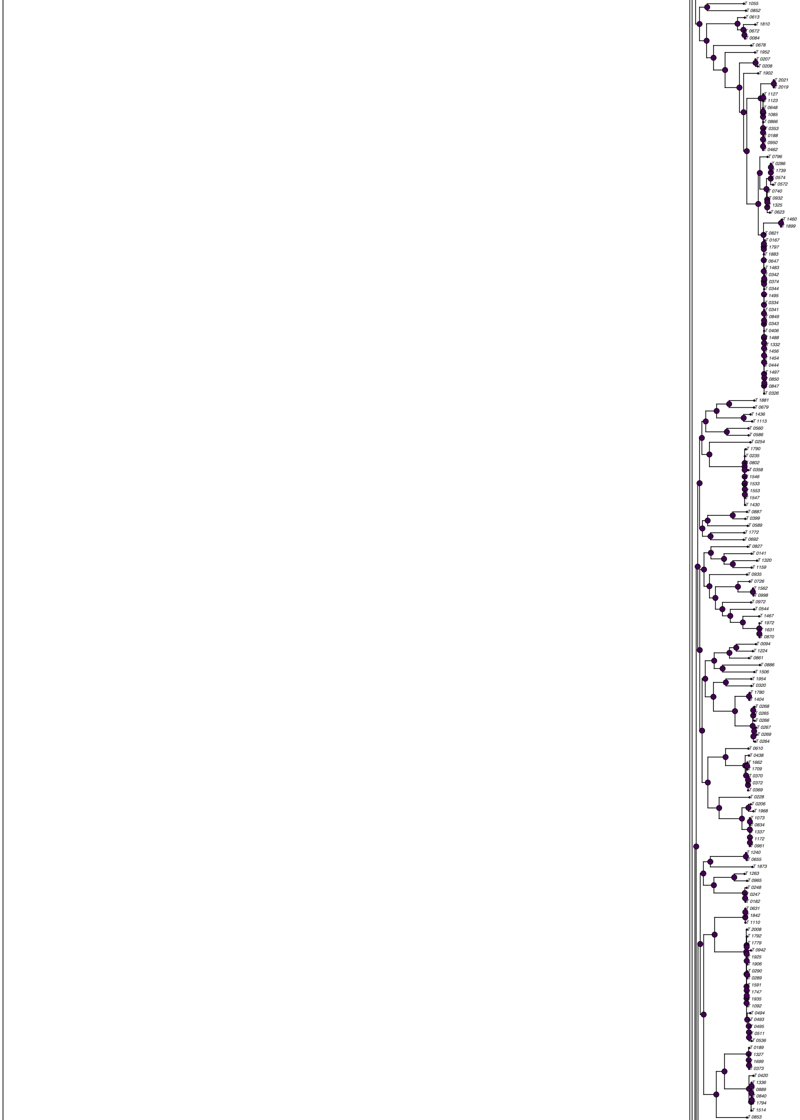
T 1055
T 0852
T 0613
T 1810
0672
T 0084
T 0678
T 1952
T 0207
T 0208
T 1902
T 2021
T 2019
T 1127
T 1123
T 0648
T 1085
T 0866
T 0353
T 0188
T 0950
T 0462
T 0796
T 0286
T 1739
T 0574
T 0572
T 0740
T 0932
T 1325
T 0623
T 1460
T 1899
T 0821
T 0167
T 1797
T 1883
T 0647
T 1483
T 0342
T 0374
T 0344
T 1495
T 0334
T 0341
T 0849
T 0343
T 0406
T 1488
T 1332
T 1456
T 1454
T 0444
T 1497
T 0850
T 0847
T 0326
T 1881
T 0679
T 1436
T 1113
T 0560
T 0586
T 0254
T 1790
T 0235
0802
T 0358
1546
1533
1553
T 1547
T 1430
T 0887
T 0399
T 0589
T 1772
T 0692
T 0827
T 0141
T 1320
T 1159
T 0935
T 0726
T 1562
T 0998
T 0972
T 0544
T 1467
T 1972
1631
T 0870
T 0094
T 1224
T 0861
T 0886
T 1506
T 1954
T 0320
T 1780
T 1404
T 0268
T 0265
T 0266
T 0267
T 0269
T 0264
T 0610
T 0438
T 1662
T 1709
T 0370
T 0372
T 0369
T 0228
T 0206
T 1968
T 1073
T 0834
T 1337
T 1172
T 0961
T 1240
T 0655
T 1873
T 1263
T 0965
T 0248
T 0247
T 0182
T 0631
T 1842
T 1110
T 2008
T 1792
T 1779
T 0942
T 1925
T 1906
T 0290
T 0289
T 1591
T 1747
T 1935
T 1092
T 0494
T 0493
T 0495
T 0511
T 0536
T 0189
T 1327
T 1699
T 0373
T 0420
T 1336
T 0889
T 0840
T 1794
T 1514
T 0853

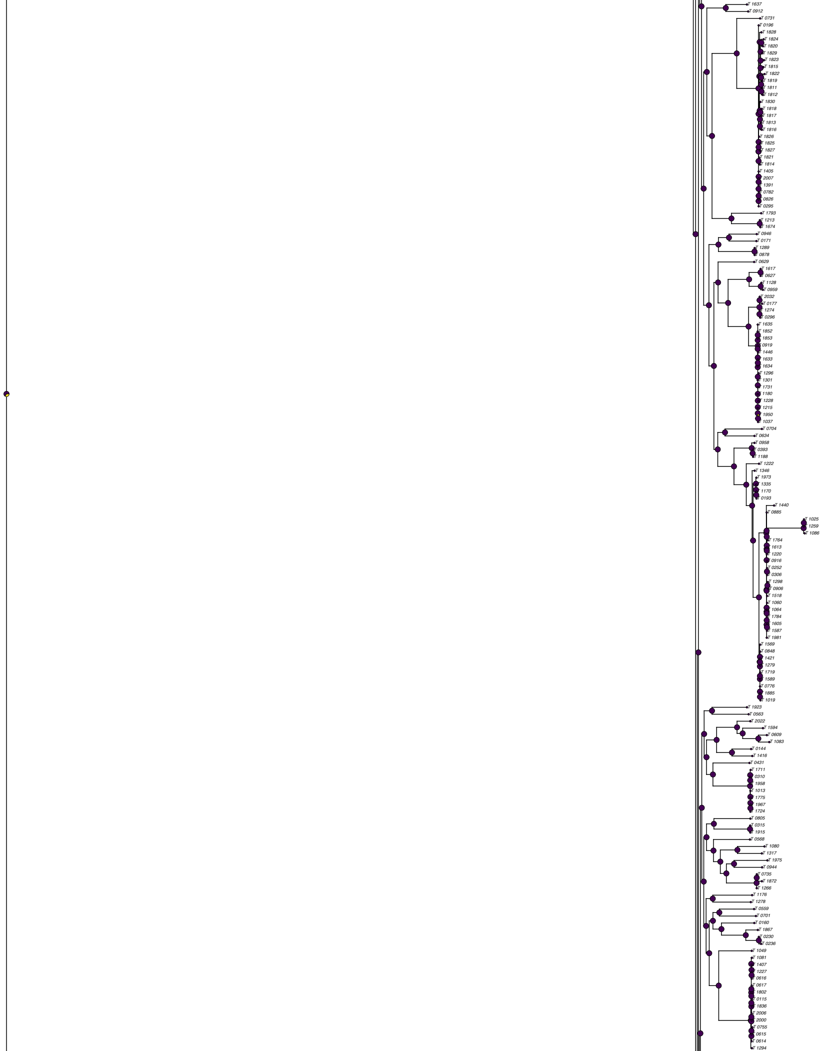
T 1637
T 0912
T 0731
T 0196
T 1828
T 1824
T 1820
T 1829
T 1823
T 1815
T 1822
T 1819
T 1811
T 1812
T 1830
T 1818
T 1817
T 1813
T 1816
T 1826
T 1825
T 1827
T 1821
T 1814
T 1405
2007
1391
0782
0826
T 0295
T 1793
T 1213
T 1674
T 0946
T 0171
T 1289
T 0878
T 0629
T 1617
T 0627
T 1128
T 0959
T 2032
T 0177
T 1274
T 0296
T 1635
T 1852
1853
0919
T 1446
1633
1634
T 1296
T 1301
1731
1180
T 1228
1215
1950
T 1037
T 0704
T 0634
T 0958
T 0393
T 1188
T 1222
T 1346
T 1973
1335
1170
T 0193
T 1440
T 0885
T 1025
1259
T 1086
T 1764
1613
T 1220
0916
T 0252
T 0306
T 1298
T 0906
T 1518
T 1060
1064
T 1784
1605
T 1587
T 1981
T 1569
T 0848
1421
1279
T 1719
1589
T 0776
1885
T 1019
T 1923
T 0563
T 2022
T 1594
T 0609
T 1083
T 0144
T 1416
T 0431
T 1711
0310
1958
T 1013
1775
1967
T 1724
T 0805
T 0315
T 1915
T 0568
T 1080
T 1317
T 1975
T 0944
T 0735
T 1872
T 1266
T 1176
T 1278
T 0559
T 0701
T 0160
T 1867
T 0230
T 0236
T 1049
T 1081
1407
1227
0616
T 0617
1802
0115
1836
T 2006
2000
T 0755
0615
T 0614
T 1294

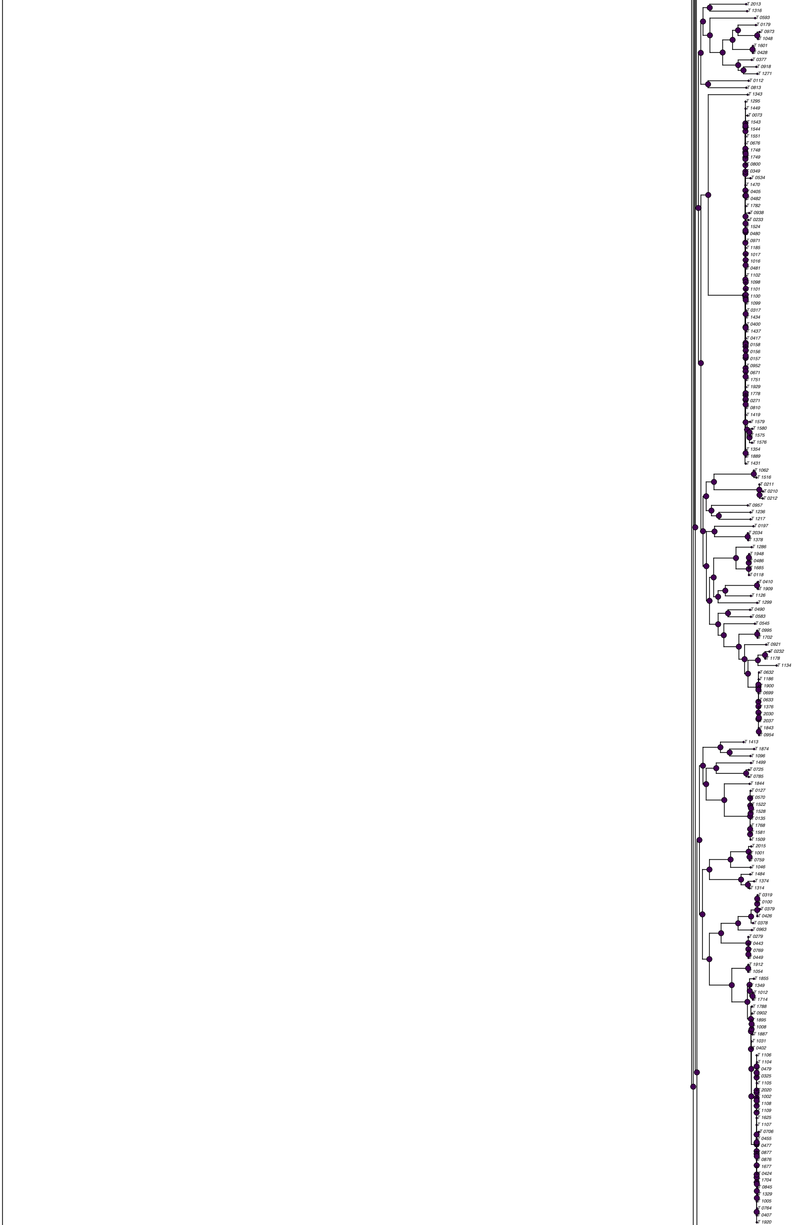
T 2013
T 1316
T 0593
T 0179
T 0973
T 1048
T 1601
T 0428
T 0377
T 0918
T 1271
T 0112
T 0813
T 1343
T 1295
T 1449
T 0073
1543
1544
T 1551
T 0676
1748
1749
0800
0349
T 0534
T 1470
0405
0482
T 1782
T 0938
T 0233
1524
0480
0971
T 1185
1017
1016
0481
T 1102
1098
1101
1100
1099
T 0317
1434
T 0400
1437
T 0417
0158
0156
0157
T 0952
0671
1751
T 1929
1778
0271
0810
T 1419
1579
T 1580
1575
T 1576
T 1354
1889
T 1431
T 1062
T 1516
T 0211
T 0210
T 0212
T 0957
T 1236
T 1217
T 0197
T 2034
T 1378
T 1286
T 1948
0486
1685
T 0118
T 0410
T 1909
T 1126
T 1299
T 0490
T 0583
T 0545
T 0995
T 1702
T 0921
T 0232
T 1178
T 1134
T 0632
T 1186
1900
0699
T 0633
1376
2030
2037
T 1843
T 0954
T 1413
T 1874
T 1096
T 1499
T 0725
T 0785
T 1844
T 0127
0570
1522
1528
0135
T 1768
1581
T 1509
T 2015
T 1001
T 0759
T 1046
T 1484
T 1374
T 1314
T 0319
0100
T 0379
T 0426
T 0378
T 0963
T 0279
0443
0769
0449
T 1912
1054
T 1855
1349
T 1012
T 1714
T 1788
T 0902
1895
1008
T 1887
T 1031
0402
T 1106
T 1104
0479
0325
T 1105
2020
1002
1108
1109
T 1625
T 1107
T 0706
T 0455
0477
0877
T 0876
1677
0424
1704
0845
T 1329
1005
T 0764
0407
T 1920

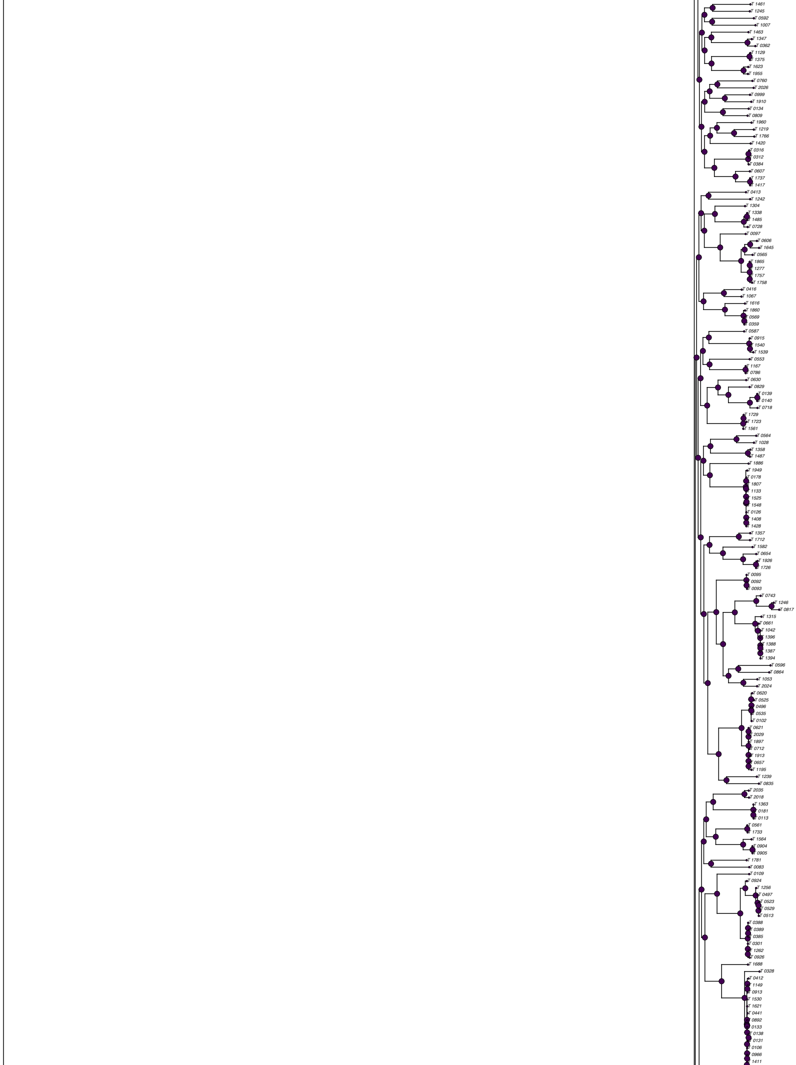
T 1461
T 1245
T 0592
T 1007
T 1463
T 1347
T 0362
T 1129
T 1375
T 1623
T 1955
T 0760
T 2026
T 0999
T 1910
T 0134
T 0809
T 1960
T 1219
T 1766
T 1420
T 0316
T 0312
T 0384
T 0607
T 1737
T 1417
T 0413
T 1242
T 1304
T 1338
T 1485
T 0728
T 0097
T 0606
T 1645
T 0565
T 1865
T 1277
T 1757
T 1758
T 0416
T 1067
T 1616
T 1860
T 0569
T 0359
T 0587
T 0915
T 1540
T 1539
T 0553
T 1167
T 0786
T 0630
T 0829
T 0139
T 0140
T 0718
T 1729
T 1723
T 1561
T 0564
T 1028
T 1358
T 1487
T 1886
T 1949
T 0178
T 1807
T 1133
T 1525
T 1548
T 0126
T 1408
T 1428
T 1357
T 1712
T 1582
T 0654
T 1926
T 1726
T 0095
T 0092
T 0093
T 0743
T 1246
T 0817
T 1315
T 0661
T 1042
T 1396
T 1388
T 1387
T 1394
T 0596
T 0864
T 1053
T 2024
T 0620
T 0525
T 0496
T 0535
T 0102
T 0621
T 2029
T 1897
T 0712
T 1913
T 0657
T 1195
T 1239
T 0835
T 2035
T 2018
T 1363
T 0181
T 0113
T 0561
T 1733
T 1564
T 0904
T 0905
T 1781
T 0083
T 0109
T 0924
T 1256
T 0497
T 0523
T 0529
T 0513
T 0388
T 0389
T 0385
T 0301
T 1262
T 0926
T 1688
T 0328
T 0412
T 1149
T 0913
T 1530
T 1621
T 0441
T 0892
T 0133
T 0138
T 0131
T 0106
T 0966
T 1411

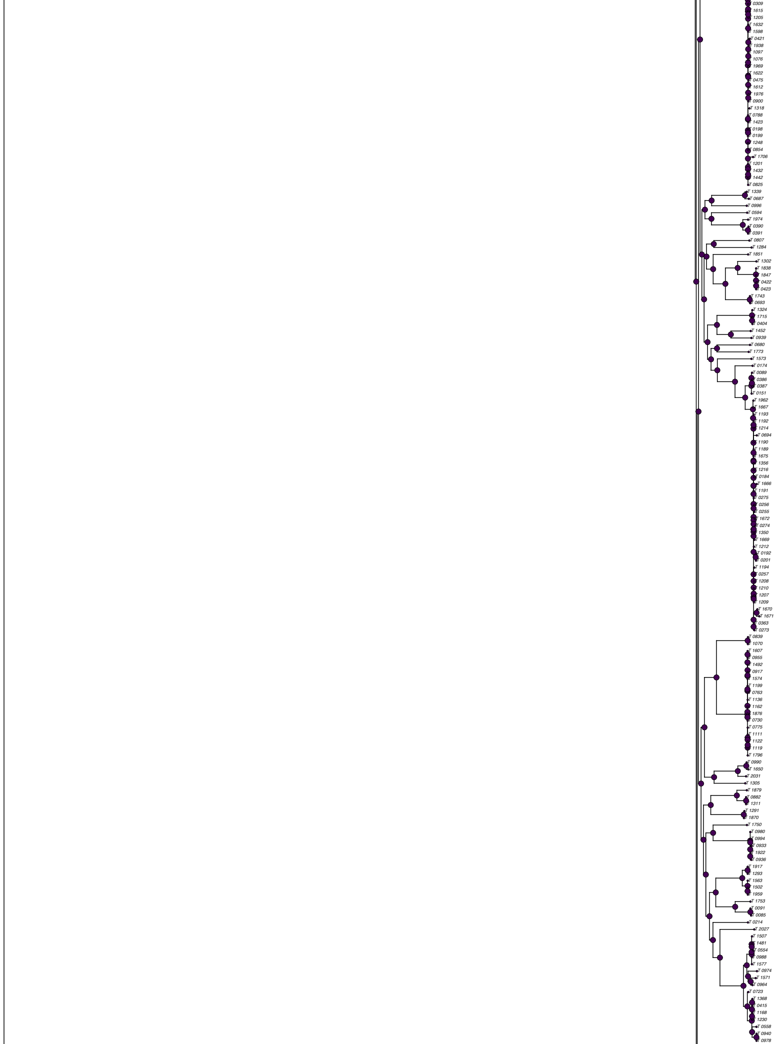
T 0309
T 1615
T 1205
T 1632
T 1598
T 0421
T 1938
T 1097
T 1076
T 1969
T 1622
T 0475
T 1612
T 1976
T 0900
T 1318
T 0788
T 1423
T 0198
T 0199
T 1248
T 0854
T 1706
T 1201
T 1432
T 1442
T 0825
T 1339
T 0687
T 0996
T 0594
T 1974
T 0390
T 0391
T 0807
T 1284
T 1851
T 1302
T 1838
T 1847
T 0422
T 0423
T 1743
T 0693
T 1324
T 1715
T 0404
T 1452
T 0939
T 0680
T 1773
T 1573
T 0174
T 0089
T 0386
T 0387
T 0151
T 1962
T 1667
T 1193
T 1192
T 1214
T 0694
T 1190
T 1189
T 1675
T 1356
T 1216
T 0184
T 1666
T 1191
T 0275
T 0256
T 0255
T 1672
T 0274
T 1350
T 1669
T 1212
T 0192
T 0201
T 1194
T 0257
T 1208
T 1210
T 1207
T 1209
T 1670
T 1671
T 0363
T 0273
T 0839
T 1070
T 1607
T 0955
T 1492
T 0917
T 1574
T 1199
T 0763
T 1136
T 1162
T 1876
T 0730
T 0775
T 1111
T 1122
T 1119
T 1796
T 0990
T 1650
T 2031
T 1305
T 1879
T 0882
T 1311
T 1291
T 1870
T 1750
T 0980
T 0994
T 0933
T 1922
T 0936
T 1917
T 1293
T 1563
T 1502
T 1959
T 1753
T 0091
T 0085
T 0214
T 2027
T 1507
T 1481
T 0554
T 0988
T 1577
T 0974
T 1571
T 0964
T 0723
T 1368
T 0415
T 1168
T 1230
T 0558
T 0940
T 0978

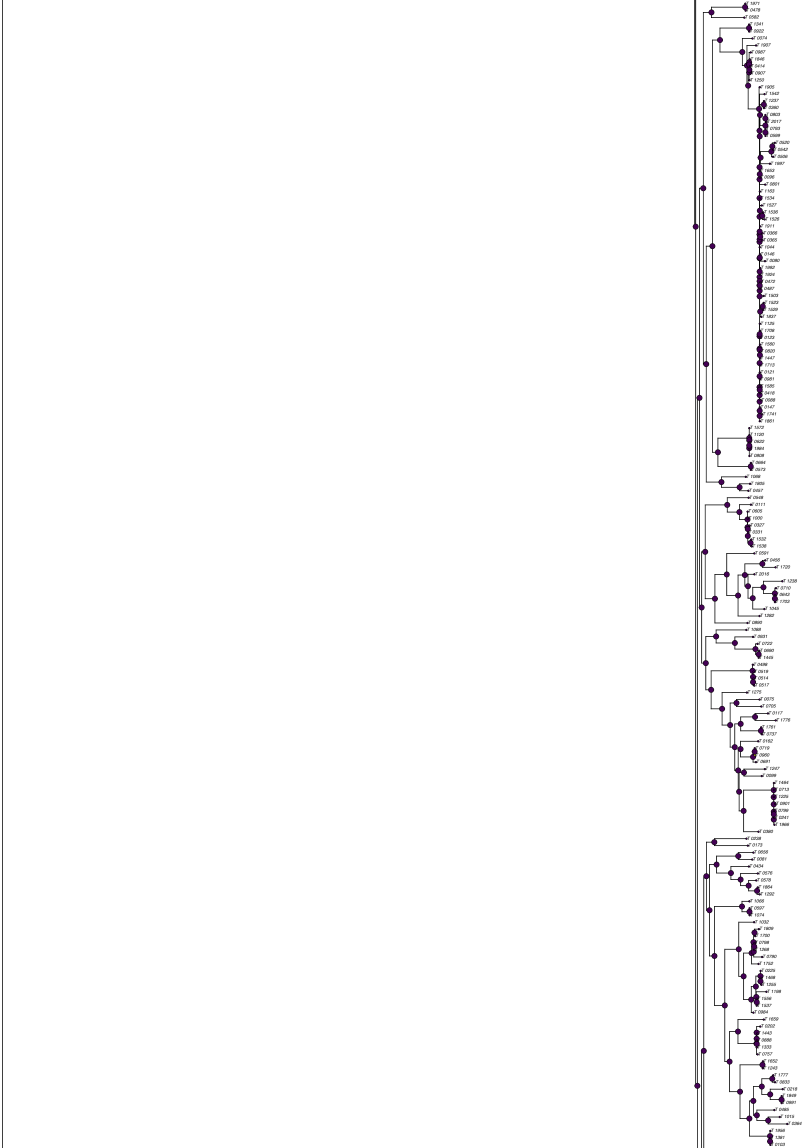

T 1971
T 0478
T 0582
T 1341
T 0922
T 0074
T 1907
T 0987
T 1846
T 0414
T 0907
T 1250
T 1905
T 1542
T 1237
T 0360
T 0803
T 2017
T 0793
T 0599
T 0520
T 0542
T 0506
T 1997
1653
0096
T 0801
T 1163
1534
T 1527
T 1536
T 1526
T 1911
T 0366
T 0365
T 1044
T 0146
T 0080
T 1992
1924
0472
0487
T 1503
T 1523
T 1529
T 1837
T 1125
T 1708
0123
T 1560
0820
1447
1713
T 0121
0981
1585
0418
T 0088
T 0147
T 1741
T 1861
T 1572
T 1120
0622
1984
T 0808
T 0664
T 0573
T 1068
T 1805
T 0457
T 0548
T 0111
T 0605
1000
T 0327
0331
T 1532
T 1538
T 0591
T 0456
T 1720
T 2016
T 1238
T 0710
0643
T 1703
T 1045
T 1282
T 0890
T 1088
T 0931
T 0722
T 0690
T 1445
T 0498
0519
0514
T 0517
T 1275
T 0075
T 0705
T 0117
T 1776
T 1761
T 0737
T 0162
T 0719
T 0960
T 0691
T 1247
T 0099
T 1464
0713
1225
T 0901
T 0799
0241
T 1966
T 0380
T 0238
T 0173
T 0656
T 0081
T 0434
T 0576
T 0578
T 1864
T 1292
T 1066
T 0597
T 1074
T 1032
T 1809
T 1700
T 0798
1268
T 0790
T 1752
T 0225
1468
T 1255
T 1198
1556
1537
T 0984
T 1659
T 0202
1443
0888
1333
T 0757
T 1652
T 1243
T 1777
T 0833
T 0218
T 1849
T 0991
T 0485
T 1015
T 0364
T 1956
1381
T 0103

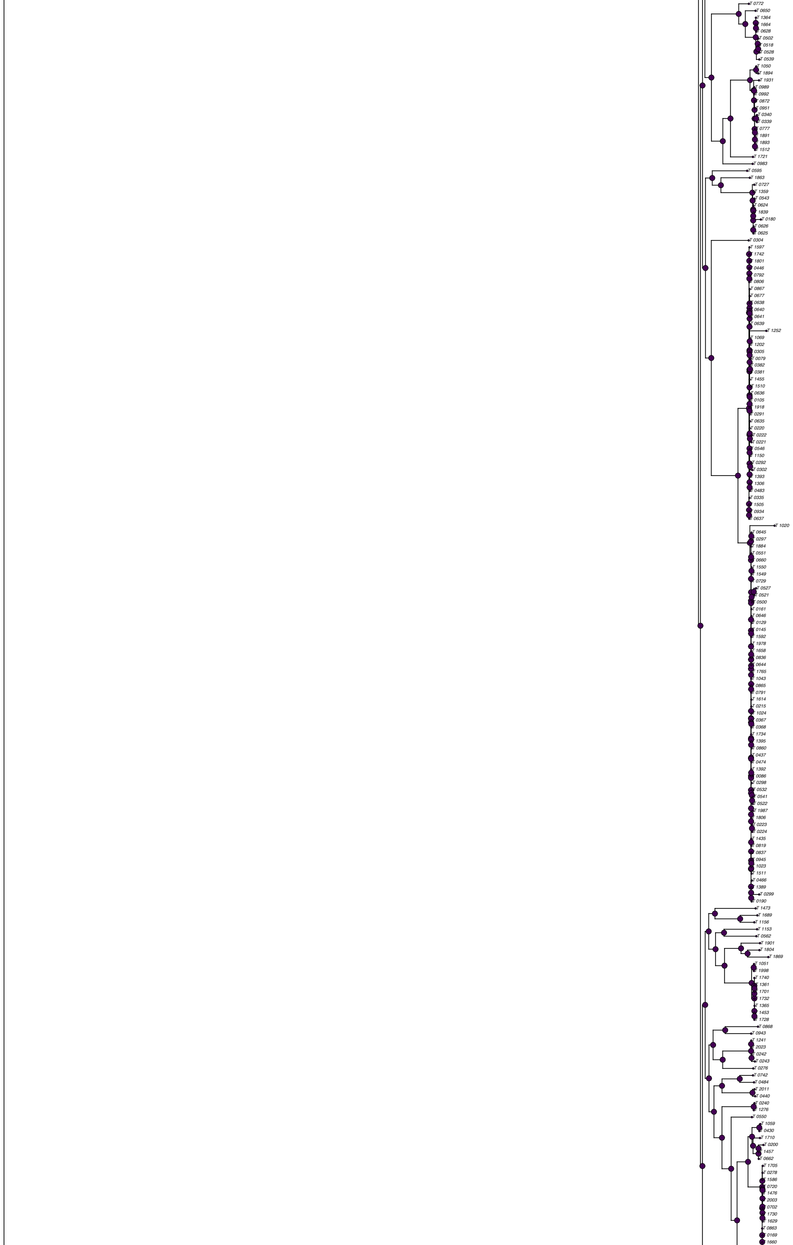
T 0772
T 0650
T 1364
1664
0628
T 0502
0518
T 0528
T 0539
T 1050
T 1894
T 1931
T 0989
0992
0872
T 0951
T 0340
T 0339
0777
1891
1893
T 1512
T 1721
T 0983
T 0595
T 1863
T 0727
T 1359
T 0543
T 0624
1839
T 0180
T 0626
T 0625
T 0304
T 1597
1742
1801
0446
0792
T 0806
T 0867
T 0677
0638
0640
0641
T 0639
T 1252
T 1069
T 1202
0305
T 0079
0382
0381
T 1455
1510
T 0636
0105
1918
T 0291
T 0635
T 0220
T 0222
T 0221
0546
T 1150
T 0292
T 0302
1393
1306
T 0483
T 0335
1505
0934
T 0637
T 1020
T 0645
0297
1884
T 0551
0660
T 1550
1549
0729
T 0527
T 0521
0500
T 0161
0646
T 0129
T 0145
T 1592
T 1978
1658
0836
0644
T 1765
T 1043
0865
T 0791
T 1614
T 0215
1024
0367
T 0368
T 1734
1395
T 0860
0437
T 0474
T 1392
0086
T 0298
T 0532
T 0541
T 0522
T 1987
1806
T 0223
T 0224
T 1435
T 0819
T 0837
0945
1023
T 1511
T 0466
1389
T 0299
T 0190
T 1473
T 1689
T 1156
T 1153
T 0562
T 1901
T 1804
T 1869
T 1051
T 1998
T 1740
1361
1701
1732
T 1365
1453
T 1728
T 0868
T 0943
T 1241
2023
0242
T 0243
T 0276
T 0742
T 0484
T 2011
T 0440
T 0240
T 1276
T 0550
T 1059
0430
T 1710
T 0200
1457
T 0662
T 1705
T 0278
1586
0720
T 1476
2003
0702
1730
T 1629
T 0863
0169
1660

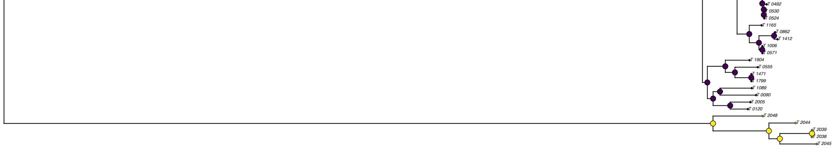

T 0492
T 0530
T 0524
T 1165
T 0862
T 1412
T 1006
T 0571
T 1904
T 0555
T 1471
T 1799
T 1089
T 0090
T 2005
T 0120
T 2048
T 2044
T 2039
T 2038
T 2045

Effector gene: tseV

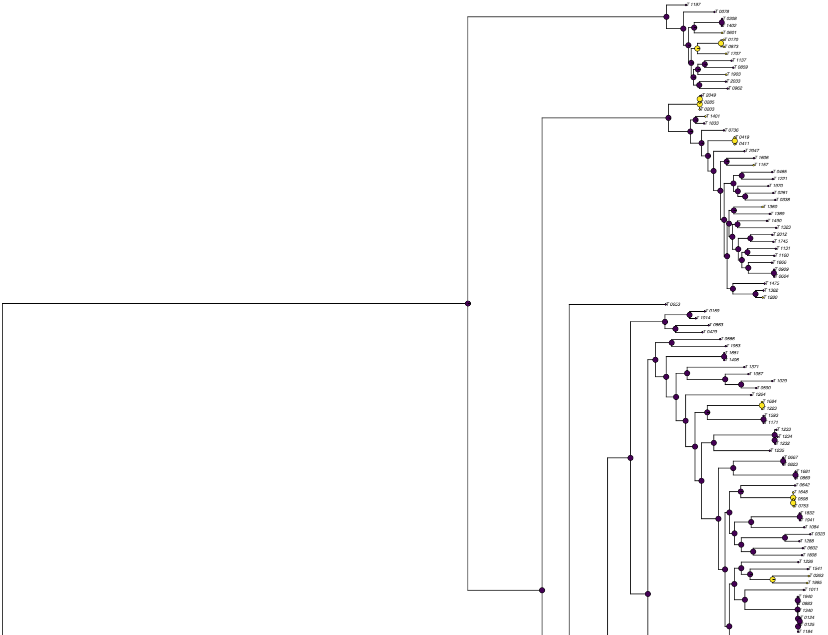

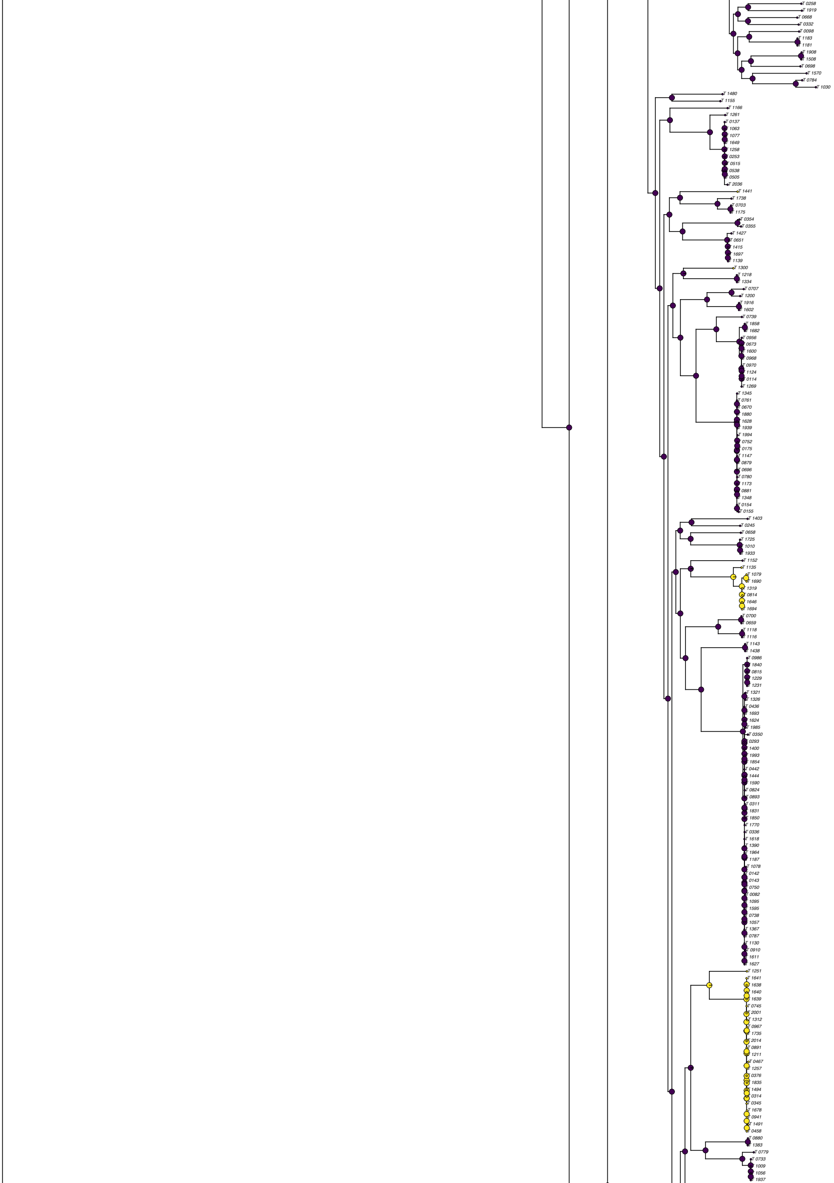

T 0258
T 1919
T 0668
T 0332
T 0098
T 1183
T 1181
T 1908
T 1508
T 0698
T 1570
T 0784
T 1030
T 1480
T 1155
T 1166
T 1261
T 0137
1063
1077
1649
1258
0253
0515
0538
0505
T 2036
T 1441
T 1738
T 0703
T 1175
T 0354
T 0355
T 1427
T 0651
1415
1697
1139
T 1300
T 1218
T 1334
T 0707
T 1200
T 1916
T 1602
T 0739
T 1858
T 1682
T 0956
0673
1600
0968
T 0970
T 1124
T 0114
T 1269
T 1345
T 0761
0670
1880
1628
1939
T 1994
0752
0175
T 1147
0879
0696
T 0780
1173
0881
T 1348
T 0154
T 0155
T 1403
T 0245
T 0658
T 1725
1010
T 1933
T 1152
T 1135
T 1079
T 1690
1319
0814
1646
T 1694
T 0700
T 0659
T 1118
T 1116
T 1143
1438
T 0986
1840
0815
1229
1231
T 1321
T 1326
T 0436
1693
1624
T 1985
T 0350
0293
1400
1993
1854
T 0442
1444
1590
T 0824
0893
T 0311
1831
1850
T 1770
T 0336
T 1618
1390
T 1964
1187
T 1078
0142
0143
0750
0082
1095
1595
0738
1057
T 1367
0787
T 1130
T 0910
1611
1627
T 1251
T 1641
1638
1640
1639
T 0745
2001
T 1312
T 0967
1735
2014
T 0891
1211
T 0467
1257
0376
1835
1494
0314
T 0345
T 1678
0941
T 1491
0458
T 0880
T 1383
T 0779
T 0733
1009
1056
T 1937

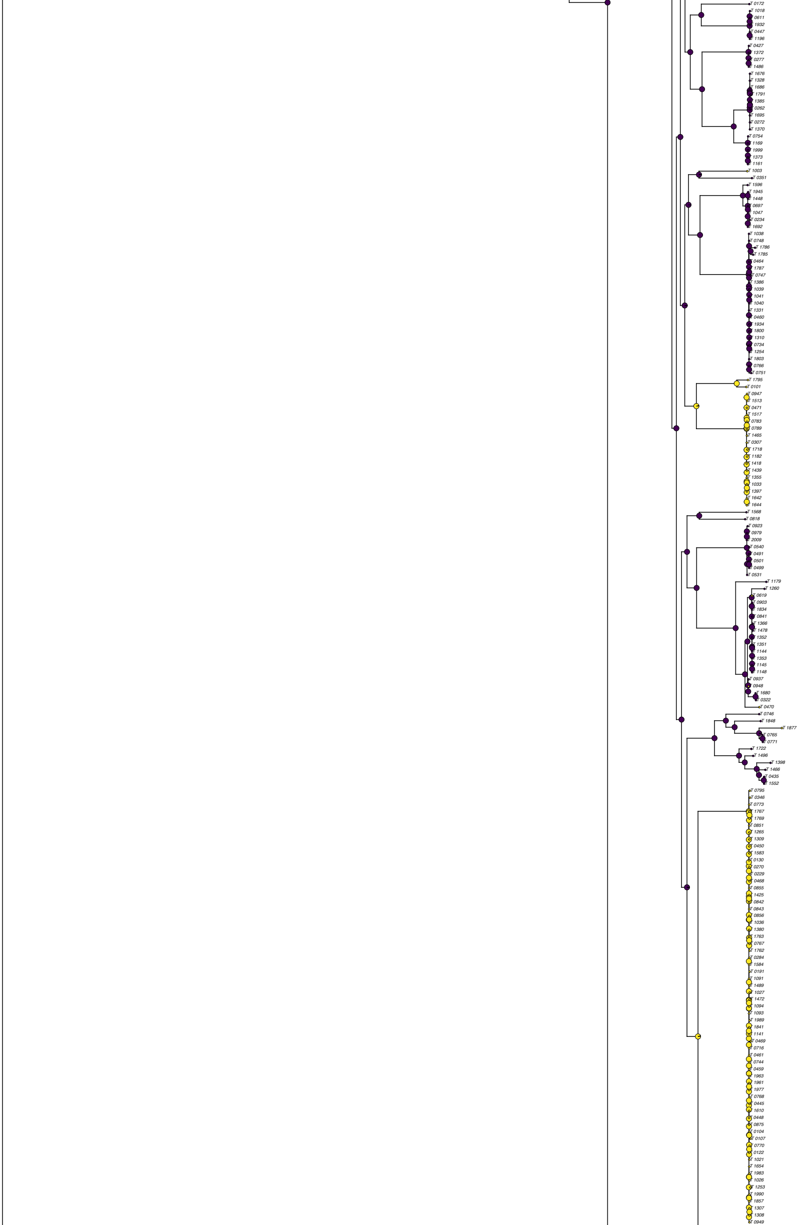
T 0172
T 1018
0611
1932
T 0447
T 1196
T 0427
1372
0277
T 1486
T 1676
T 1328
T 1686
1791
1385
0262
T 1695
T 0272
T 1370
T 0754
1169
1999
1373
T 1161
T 1003
T 0351
T 1596
T 1945
T 1448
0697
T 1047
T 0234
T 1692
T 1038
T 0748
T 1786
T 1785
0464
1787
T 0747
T 1386
1039
1041
1040
T 1331
0460
1934
1800
1310
0734
T 1254
T 1803
0766
T 0751
T 1795
T 0101
T 0947
T 1513
0471
T 1517
0783
0789
T 1465
T 0307
T 1718
T 1182
T 1418
T 1439
T 1355
1033
1397
T 1642
T 1644
T 1568
T 0818
T 0923
0979
T 2009
T 0540
0491
0501
T 0499
T 0531
T 1179
T 1260
T 0619
T 0903
T 1834
0841
T 1366
T 1478
1352
1351
1144
1353
1145
T 1148
T 0937
T 0948
T 1680
T 0322
T 0470
T 0746
T 1848
T 1877
T 0765
T 0771
T 1722
T 1496
T 1398
T 1466
T 0435
T 1552
T 0795
T 0346
T 0773
T 1767
T 1769
T 0851
T 1265
T 1309
T 0450
T 1583
T 0130
T 0270
T 0229
T 0468
T 0855
T 1425
T 0842
T 0843
T 0856
T 1036
T 1380
T 1763
T 0767
T 1762
T 0284
T 1584
T 0191
T 1091
T 1489
T 1027
T 1472
T 1094
T 1093
T 1989
T 1841
T 1141
T 0469
T 0716
T 0461
T 0744
T 0459
T 1963
T 1961
T 1977
T 0768
T 0445
T 1610
T 0448
T 0875
T 0104
T 0107
T 0770
T 0122
T 1021
T 1654
T 1983
T 1026
T 1253
T 1990
T 1857
T 1307
T 1308
T 0949

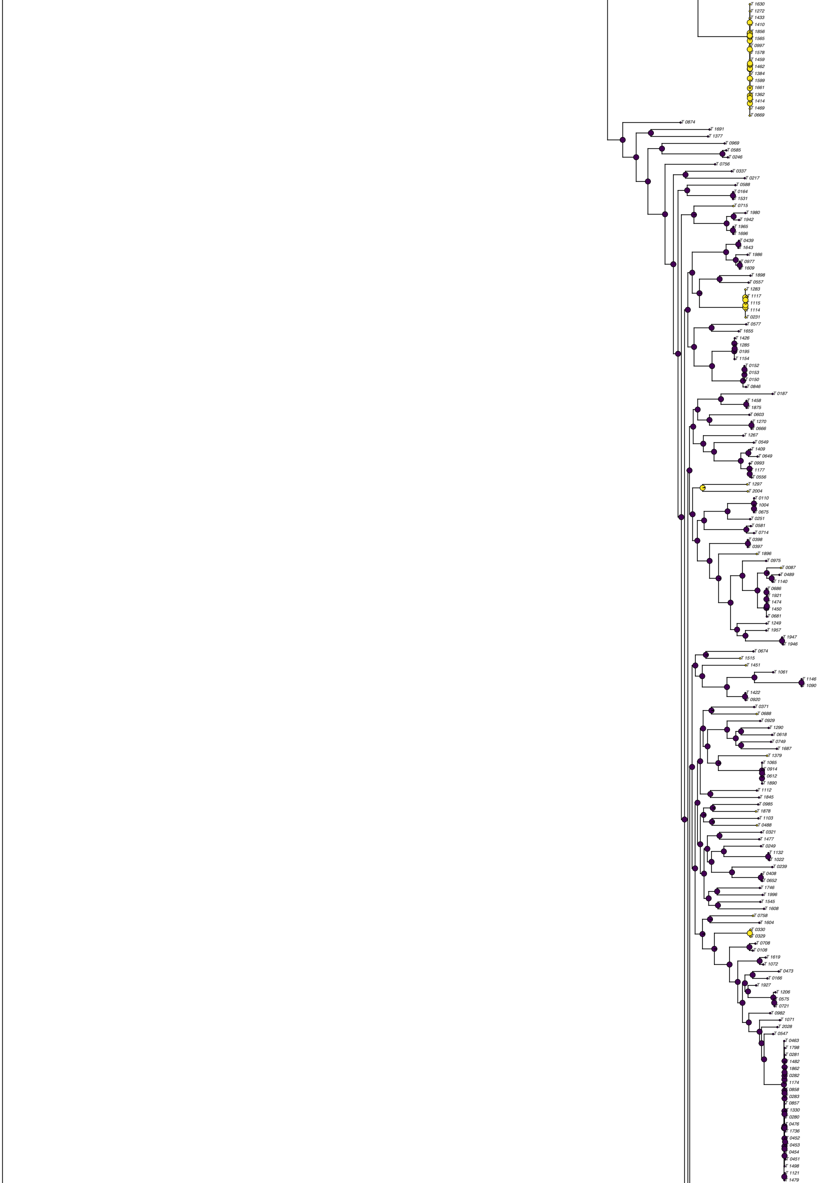

T 1630
T 1272
T 1433
T 1410
T 1856
T 1565
T 0997
T 1578
T 1459
T 1462
T 1384
T 1599
T 1661
T 1362
T 1414
T 1469
T 0669
T 0874
T 1691
T 1377
T 0969
T 0585
T 0246
T 0756
T 0337
T 0217
T 0588
T 0164
T 1531
T 0715
T 1980
T 1942
T 1965
T 1696
T 0439
T 1643
T 1986
T 0977
T 1609
T 1898
T 0557
T 1283
T 1117
T 1115
T 1114
T 0231
T 0577
T 1655
T 1426
T 1285
T 0195
T 1154
T 0152
T 0153
T 0150
T 0846
T 0187
T 1458
T 1875
T 0603
T 1270
T 0666
T 1267
T 0549
T 1409
T 0649
T 0993
T 1177
T 0556
T 1297
T 2004
T 0110
T 1004
T 0675
T 0251
T 0581
T 0714
T 0398
T 0397
T 1896
T 0975
T 0087
T 0489
T 1140
T 0686
T 1921
T 1474
T 1450
T 0681
T 1249
T 1957
T 1947
T 1946
T 0674
T 1515
T 1451
T 1061
T 1146
T 1090
T 1422
T 0920
T 0371
T 0688
T 0929
T 1290
T 0618
T 0749
T 1687
T 1379
T 1065
T 0914
T 0612
T 1890
T 1112
T 1845
T 0985
T 1878
T 1103
T 0488
T 0321
T 1477
T 0249
T 1132
T 1022
T 0239
T 0408
T 0652
T 1746
T 1996
T 1545
T 1608
T 0758
T 1604
T 0330
T 0329
T 0708
T 0108
T 1619
T 1072
T 0473
T 0166
T 1927
T 1206
T 0575
T 0721
T 0982
T 1071
T 2028
T 0547
T 0463
T 1798
T 0281
T 1482
T 1862
T 0282
T 1174
T 0858
T 0283
T 0857
T 1330
T 0280
T 0476
T 1736
T 0452
T 0453
T 0454
T 0451
T 1498
T 1121
T 1479

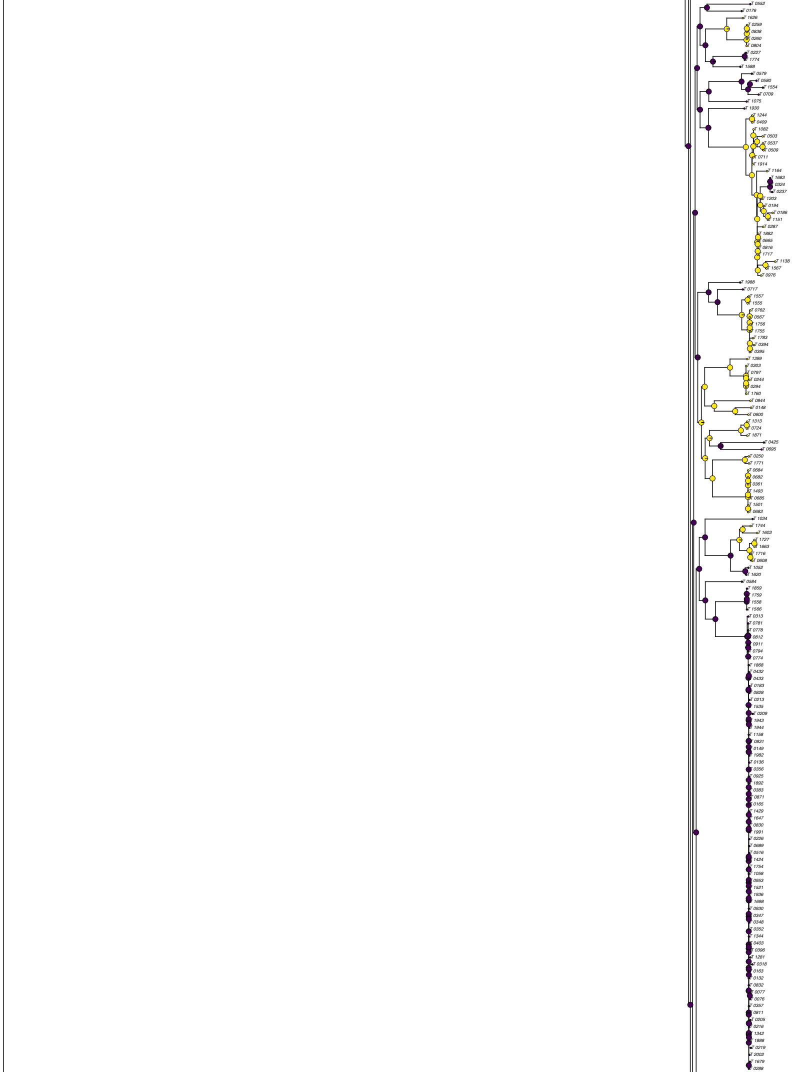
T 0552
T 0176
T 1626
T 0259
0838
0260
0804
T 0227
1774
T 1588
T 0579
T 0580
T 1554
T 0709
T 1075
T 1930
T 1244
0409
T 1082
T 0503
T 0537
T 0509
T 0711
T 1914
T 1164
T 1683
0324
T 0237
T 1203
T 0194
T 0186
1151
T 0287
T 1882
0665
0816
1717
T 1138
1567
T 0976
T 1988
T 0717
T 1557
1555
T 0762
0567
1756
1755
T 1783
T 0394
0395
T 1399
T 0303
0797
T 0244
0294
T 1760
T 0844
T 0148
T 0600
T 1313
0724
T 1871
T 0425
T 0695
T 0250
T 1771
T 0684
0682
0361
T 1493
T 0685
T 1501
0683
T 1034
T 1744
T 1603
T 1727
1663
T 1716
T 0608
T 1052
T 1620
T 0584
T 1859
1759
1558
T 1566
T 0313
T 0781
T 0778
0812
0911
T 0794
0774
T 1868
T 0432
0433
T 0183
T 0828
T 0213
1535
T 0209
1943
1944
T 1158
0831
0149
T 1982
T 0136
0356
T 0925
1892
0383
T 0871
0165
T 1429
T 1647
T 0830
1991
T 0226
T 0689
T 0516
1424
T 1754
T 1058
0953
1521
T 1936
1698
T 0930
0347
T 0348
T 0352
T 1344
0403
T 0396
T 1281
T 0318
0163
T 0132
T 0832
T 0077
T 0076
T 0357
0811
T 0205
T 0216
T 1342
T 1888
T 0219
T 2002
T 1679
T 0288

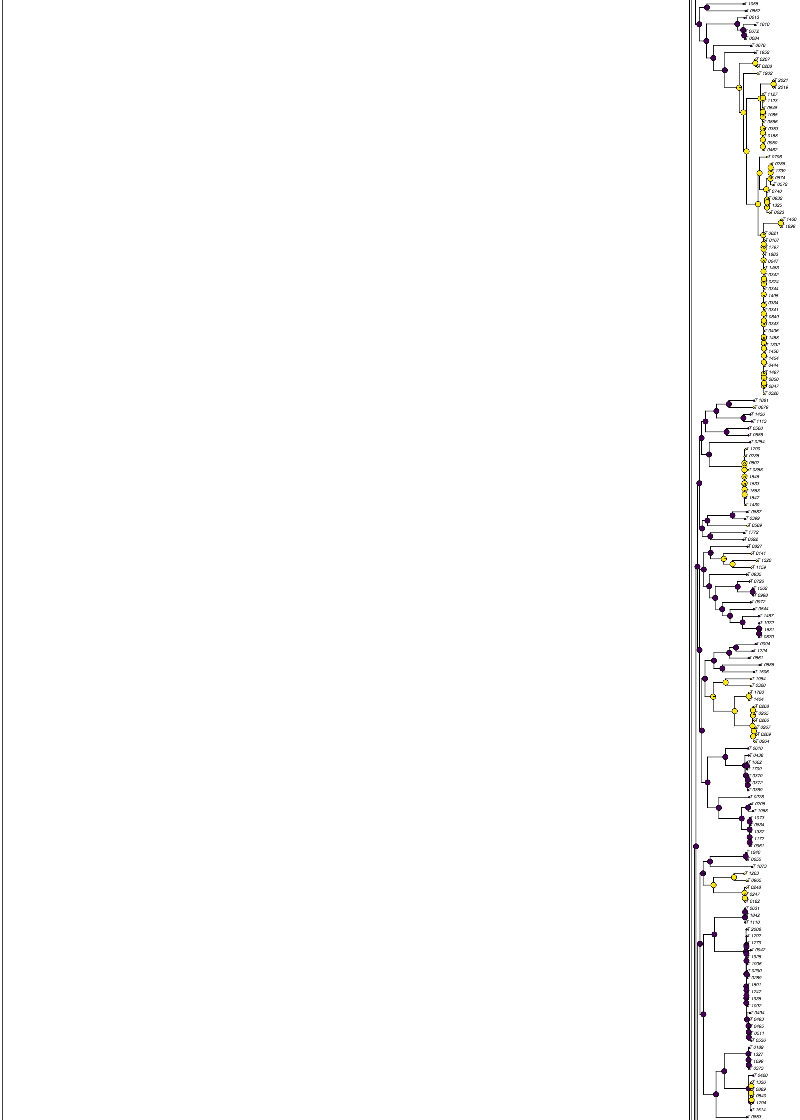
T 1055
T 0852
T 0613
T 1810
0672
T 0084
T 0678
T 1952
T 0207
T 0208
T 1902
T 2021
T 2019
T 1127
T 1123
T 0648
1085
T 0866
T 0353
0188
T 0950
0462
T 0796
T 0286
1739
0574
T 0572
T 0740
T 0932
1325
T 0623
T 1460
T 1899
T 0821
T 0167
1797
T 1883
0647
T 1483
T 0342
T 0374
T 0344
1495
T 0334
T 0341
T 0849
T 0343
T 0406
1488
T 1332
1456
1454
T 0444
T 1497
0850
T 0847
T 0326
T 1881
T 0679
T 1436
T 1113
T 0560
T 0586
T 0254
T 1790
T 0235
0802
T 0358
1546
1533
1553
T 1547
T 1430
T 0887
T 0399
T 0589
T 1772
T 0692
T 0827
T 0141
T 1320
T 1159
T 0935
T 0726
T 1562
T 0998
T 0972
T 0544
T 1467
T 1972
1631
T 0870
T 0094
T 1224
T 0861
T 0886
T 1506
T 1954
T 0320
T 1780
T 1404
T 0268
0265
T 0266
T 0267
T 0269
T 0264
T 0610
T 0438
T 1662
T 1709
T 0370
0372
T 0369
T 0228
T 0206
T 1968
T 1073
T 0834
1337
1172
T 0961
T 1240
T 0655
T 1873
T 1263
T 0965
T 0248
0247
T 0182
T 0631
1842
T 1110
T 2008
T 1792
T 1779
T 0942
T 1925
T 1906
T 0290
T 0289
T 1591
T 1747
T 1935
T 1092
T 0494
T 0493
0495
0511
T 0536
T 0189
T 1327
T 1699
T 0373
T 0420
T 1336
T 0889
T 0840
T 1794
T 1514
T 0853

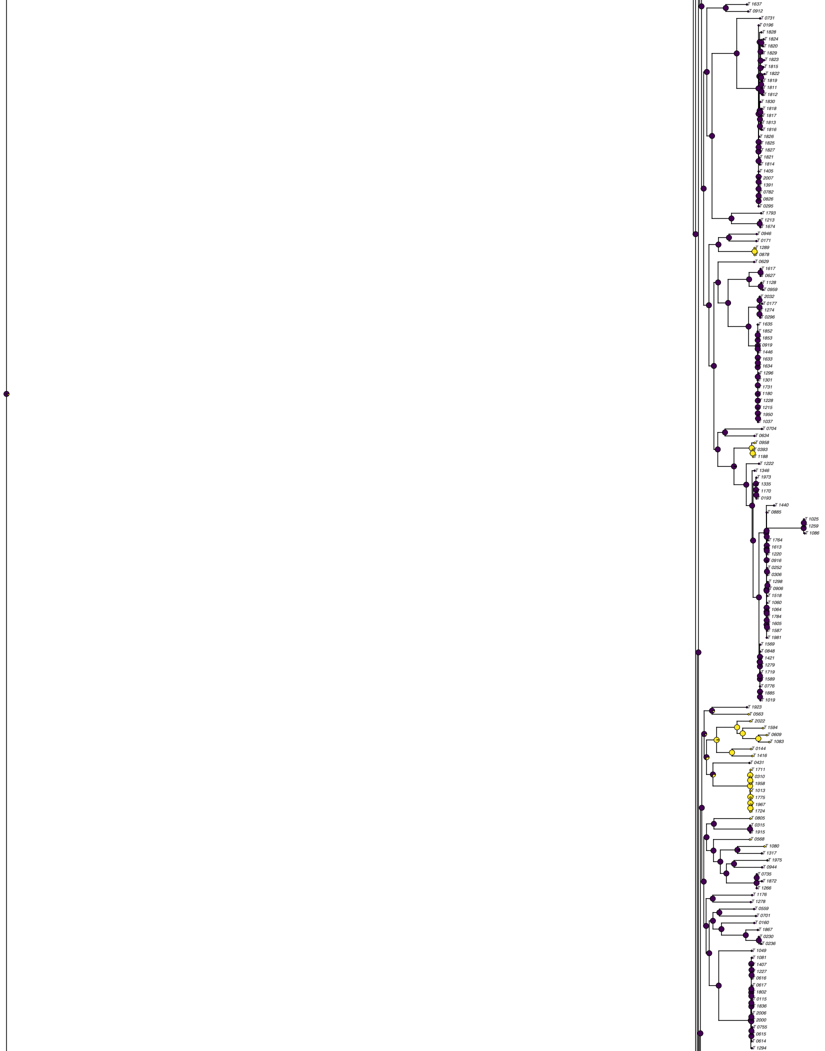
T 1637
T 0912
T 0731
T 0196
T 1828
T 1824
T 1820
T 1829
T 1823
T 1815
T 1822
T 1819
T 1811
T 1812
T 1830
T 1818
T 1817
T 1813
T 1816
T 1826
T 1825
T 1827
T 1821
T 1814
T 1405
2007
1391
0782
0826
T 0295
T 1793
T 1213
T 1674
T 0946
T 0171
T 1289
T 0878
T 0629
T 1617
T 0627
T 1128
T 0959
T 2032
T 0177
T 1274
T 0296
T 1635
T 1852
1853
0919
T 1446
1633
1634
T 1296
T 1301
1731
1180
1228
1215
1950
T 1037
T 0704
T 0634
T 0958
0393
T 1188
T 1222
T 1346
T 1973
1335
1170
T 0193
T 1440
T 0885
T 1025
1259
T 1086
T 1764
1613
T 1220
0916
T 0252
T 0306
T 1298
T 0906
T 1518
T 1060
1064
T 1784
1605
T 1587
T 1981
T 1569
T 0848
1421
1279
T 1719
1589
T 0776
1885
T 1019
T 1923
T 0563
T 2022
T 1594
T 0609
T 1083
T 0144
T 1416
T 0431
T 1711
0310
1958
T 1013
1775
1967
1724
T 0805
T 0315
1915
T 0568
T 1080
T 1317
T 1975
T 0944
T 0735
T 1872
T 1266
T 1176
T 1278
T 0559
T 0701
T 0160
T 1867
T 0230
T 0236
T 1049
T 1081
1407
1227
0616
T 0617
1802
T 0115
1836
T 2006
2000
T 0755
0615
T 0614
T 1294

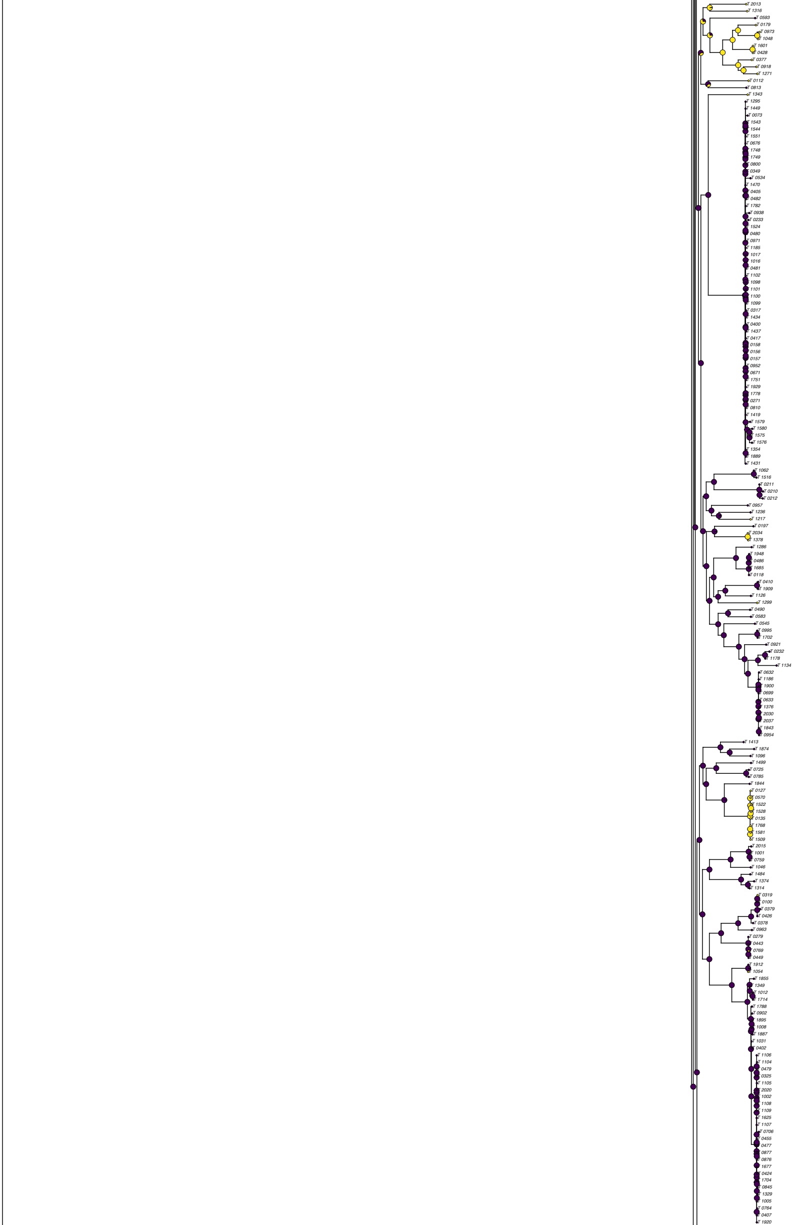
T 2013
T 1316
T 0593
T 0179
T 0973
T 1048
T 1601
T 0428
T 0377
T 0918
T 1271
T 0112
T 0813
T 1343
T 1295
T 1449
T 0073
T 1543
T 1544
T 1551
T 0676
T 1748
T 1749
T 0800
T 0349
T 0534
T 1470
T 0405
T 0482
T 1782
T 0938
T 0233
T 1524
T 0480
T 0971
T 1185
T 1017
T 1016
T 0481
T 1102
T 1098
T 1101
T 1100
T 1099
T 0317
T 1434
T 0400
T 1437
T 0417
T 0158
T 0156
T 0157
T 0952
T 0671
T 1751
T 1929
T 1778
T 0271
T 0810
T 1419
T 1579
T 1580
T 1575
T 1576
T 1354
T 1889
T 1431
T 1062
T 1516
T 0211
T 0210
T 0212
T 0957
T 1236
T 1217
T 0197
T 2034
T 1378
T 1286
T 1948
T 0486
T 1685
T 0118
T 0410
T 1909
T 1126
T 1299
T 0490
T 0583
T 0545
T 0995
T 1702
T 0921
T 0232
T 1178
T 1134
T 0632
T 1186
T 1900
T 0699
T 0633
T 1376
T 2030
T 2037
T 1843
T 0954
T 1413
T 1874
T 1096
T 1499
T 0725
T 0785
T 1844
T 0127
T 0570
T 1522
T 1528
T 0135
T 1768
T 1581
T 1509
T 2015
T 1001
T 0759
T 1046
T 1484
T 1374
T 1314
T 0319
T 0100
T 0379
T 0426
T 0378
T 0963
T 0279
T 0443
T 0769
T 0449
T 1912
T 1054
T 1855
T 1349
T 1012
T 1714
T 1788
T 0902
T 1895
T 1008
T 1887
T 1031
T 0402
T 1106
T 1104
T 0479
T 0325
T 1105
T 2020
T 1002
T 1108
T 1109
T 1625
T 1107
T 0706
T 0455
T 0477
T 0877
T 0876
T 1677
T 0424
T 1704
T 0845
T 1329
T 1005
T 0764
T 0407
T 1920

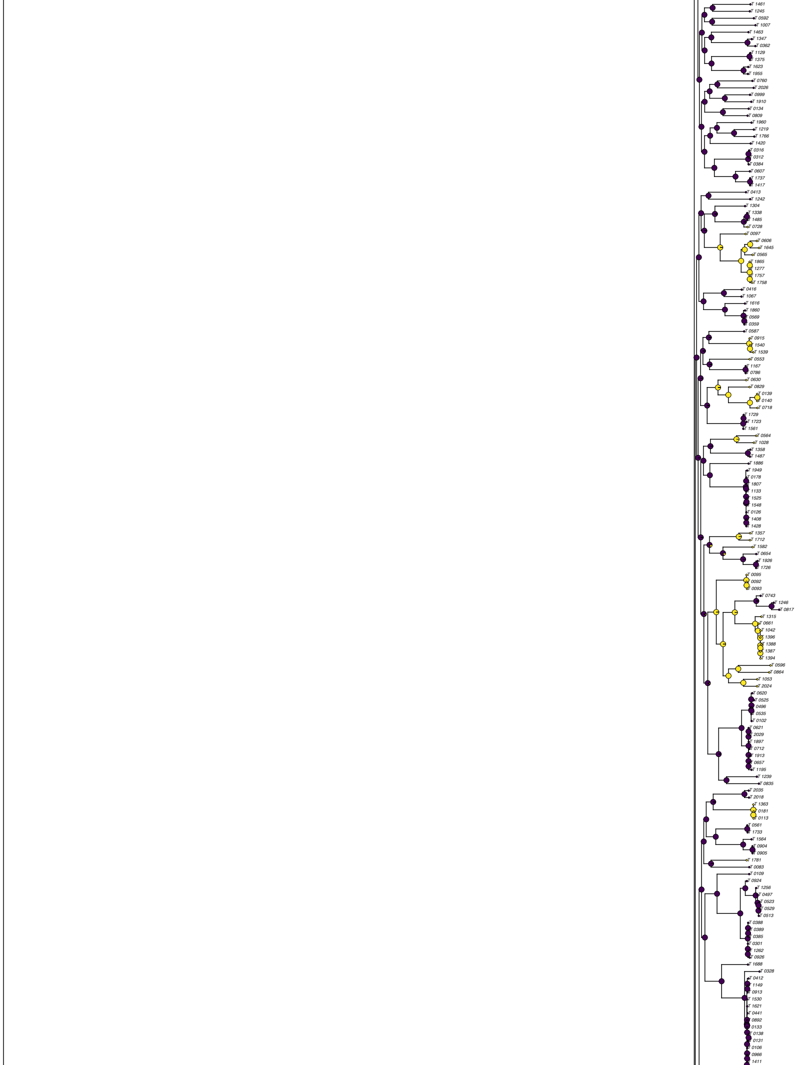
T 1461
T 1245
T 0592
T 1007
T 1463
T 1347
T 0362
T 1129
T 1375
T 1623
T 1955
T 0760
T 2026
T 0999
T 1910
T 0134
T 0809
T 1960
T 1219
T 1766
T 1420
T 0316
T 0312
T 0384
T 0607
T 1737
T 1417
T 0413
T 1242
T 1304
T 1338
T 1485
T 0728
T 0097
T 0606
T 1645
T 0565
T 1865
T 1277
T 1757
T 1758
T 0416
T 1067
T 1616
T 1860
T 0569
T 0359
T 0587
T 0915
T 1540
T 1539
T 0553
T 1167
T 0786
T 0630
T 0829
T 0139
T 0140
T 0718
T 1729
T 1723
T 1561
T 0564
T 1028
T 1358
T 1487
T 1886
T 1949
T 0178
T 1807
T 1133
T 1525
T 1548
T 0126
T 1408
T 1428
T 1357
T 1712
T 1582
T 0654
T 1926
T 1726
T 0095
T 0092
T 0093
T 0743
T 1246
T 0817
T 1315
T 0661
T 1042
T 1396
T 1388
T 1387
T 1394
T 0596
T 0864
T 1053
T 2024
T 0620
T 0525
T 0496
T 0535
T 0102
T 0621
T 2029
T 1897
T 0712
T 1913
T 0657
T 1195
T 1239
T 0835
T 2035
T 2018
T 1363
T 0181
T 0113
T 0561
T 1733
T 1564
T 0904
T 0905
T 1781
T 0083
T 0109
T 0924
T 1256
T 0497
T 0523
T 0529
T 0513
T 0388
T 0389
T 0385
T 0301
T 1262
T 0926
T 1688
T 0328
T 0412
T 1149
T 0913
T 1530
T 1621
T 0441
T 0892
T 0133
T 0138
T 0131
T 0106
T 0966
T 1411

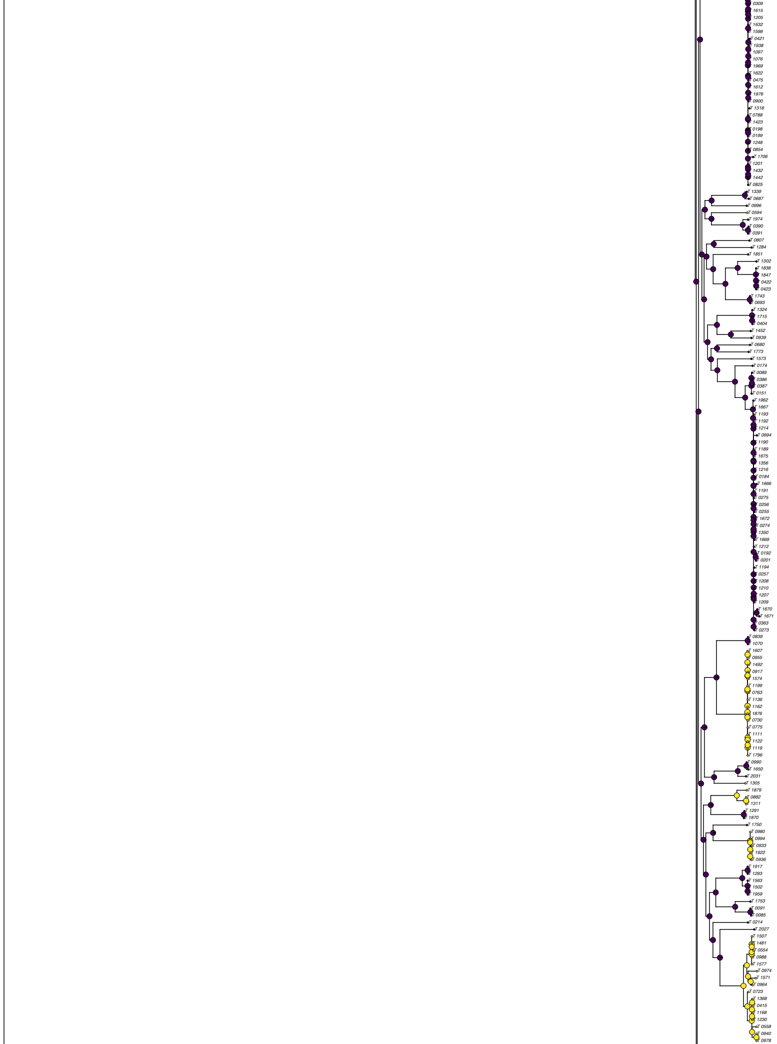
T 0309
T 1615
T 1205
T 1632
T 1598
T 0421
T 1938
T 1097
T 1076
T 1969
T 1622
T 0475
T 1612
T 1976
T 0900
T 1318
T 0788
T 1423
T 0198
T 0199
T 1248
T 0854
T 1706
T 1201
T 1432
T 1442
T 0825
T 1339
T 0687
T 0996
T 0594
T 1974
T 0390
T 0391
T 0807
T 1284
T 1851
T 1302
T 1838
T 1847
T 0422
T 0423
T 1743
T 0693
T 1324
T 1715
T 0404
T 1452
T 0939
T 0680
T 1773
T 1573
T 0174
T 0089
T 0386
T 0387
T 0151
T 1962
T 1667
T 1193
T 1192
T 1214
T 0694
T 1190
T 1189
T 1675
T 1356
T 1216
T 0184
T 1666
T 1191
T 0275
T 0256
T 0255
T 1672
T 0274
T 1350
T 1669
T 1212
T 0192
T 0201
T 1194
T 0257
T 1208
T 1210
T 1207
T 1209
T 1670
T 1671
T 0363
T 0273
T 0839
T 1070
T 1607
T 0955
T 1492
T 0917
T 1574
T 1199
T 0763
T 1136
T 1162
T 1876
T 0730
T 0775
T 1111
T 1122
T 1119
T 1796
T 0990
T 1650
T 2031
T 1305
T 1879
T 0882
T 1311
T 1291
T 1870
T 1750
T 0980
T 0994
T 0933
T 1922
T 0936
T 1917
T 1293
T 1563
T 1502
T 1959
T 1753
T 0091
T 0085
T 0214
T 2027
T 1507
T 1481
T 0554
T 0988
T 1577
T 0974
T 1571
T 0964
T 0723
T 1368
T 0415
T 1168
T 1230
T 0558
T 0940
T 0978

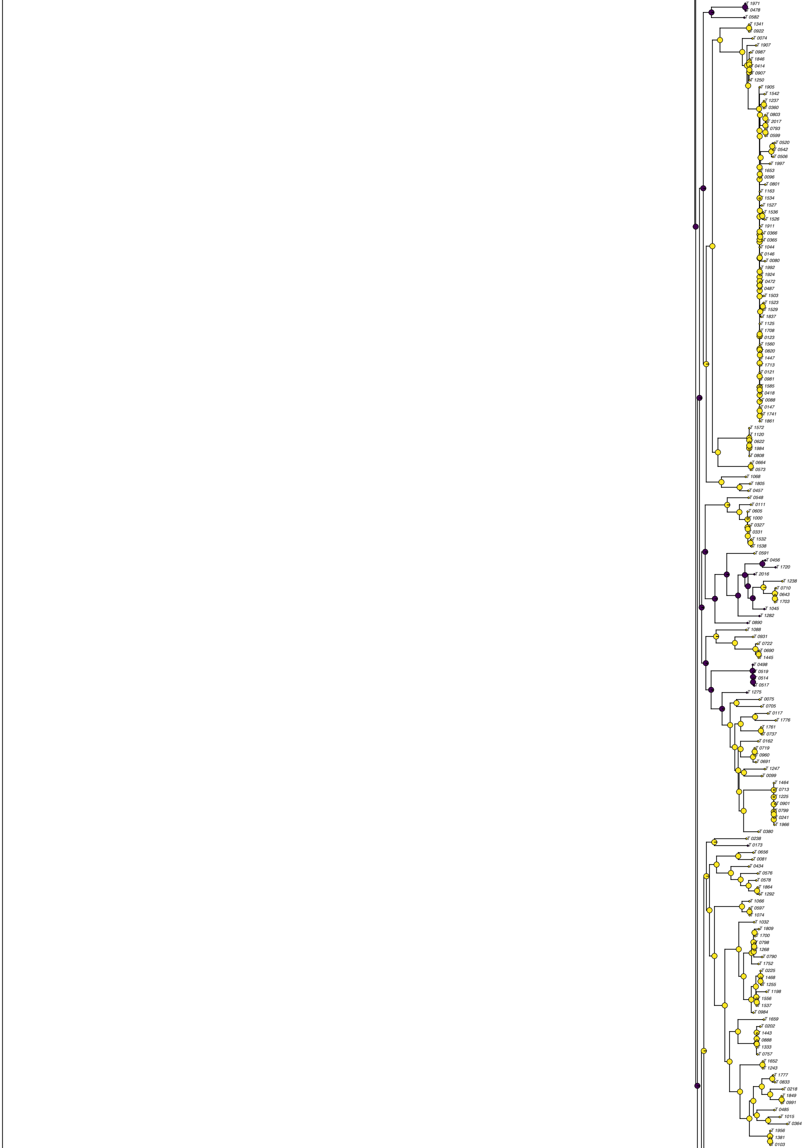
T 1971
T 0478
T 0582
T 1341
0922
T 0074
T 1907
T 0987
T 1846
T 0414
T 0907
T 1250
T 1905
T 1542
T 1237
T 0360
T 0803
T 2017
T 0793
T 0599
T 0520
T 0542
T 0506
T 1997
T 1653
0096
T 0801
T 1163
1534
T 1527
T 1536
T 1526
T 1911
T 0366
T 0365
T 1044
T 0146
T 0080
T 1992
T 1924
T 0472
0487
T 1503
T 1523
T 1529
T 1837
T 1125
T 1708
T 0123
T 1560
T 0820
T 1447
T 1713
T 0121
T 0981
T 1585
T 0418
T 0088
T 0147
T 1741
T 1861
T 1572
T 1120
T 0622
T 1984
T 0808
T 0664
T 0573
T 1068
T 1805
T 0457
T 0548
T 0111
T 0605
1000
T 0327
0331
T 1532
T 1538
T 0591
T 0456
T 1720
T 2016
T 1238
T 0710
0643
T 1703
T 1045
T 1282
T 0890
T 1088
T 0931
T 0722
T 0690
T 1445
T 0498
T 0519
T 0514
T 0517
T 1275
T 0075
T 0705
T 0117
T 1776
T 1761
T 0737
T 0162
T 0719
0960
T 0691
T 1247
T 0099
T 1464
T 0713
1225
T 0901
T 0799
0241
T 1966
T 0380
T 0238
T 0173
T 0656
T 0081
T 0434
T 0576
T 0578
T 1864
T 1292
T 1066
T 0597
1074
T 1032
T 1809
T 1700
T 0798
1268
T 0790
T 1752
T 0225
1468
T 1255
T 1198
1556
T 1537
T 0984
T 1659
T 0202
1443
T 0888
T 1333
T 0757
T 1652
T 1243
T 1777
T 0833
T 0218
T 1849
T 0991
T 0485
T 1015
T 0364
T 1956
1381
T 0103

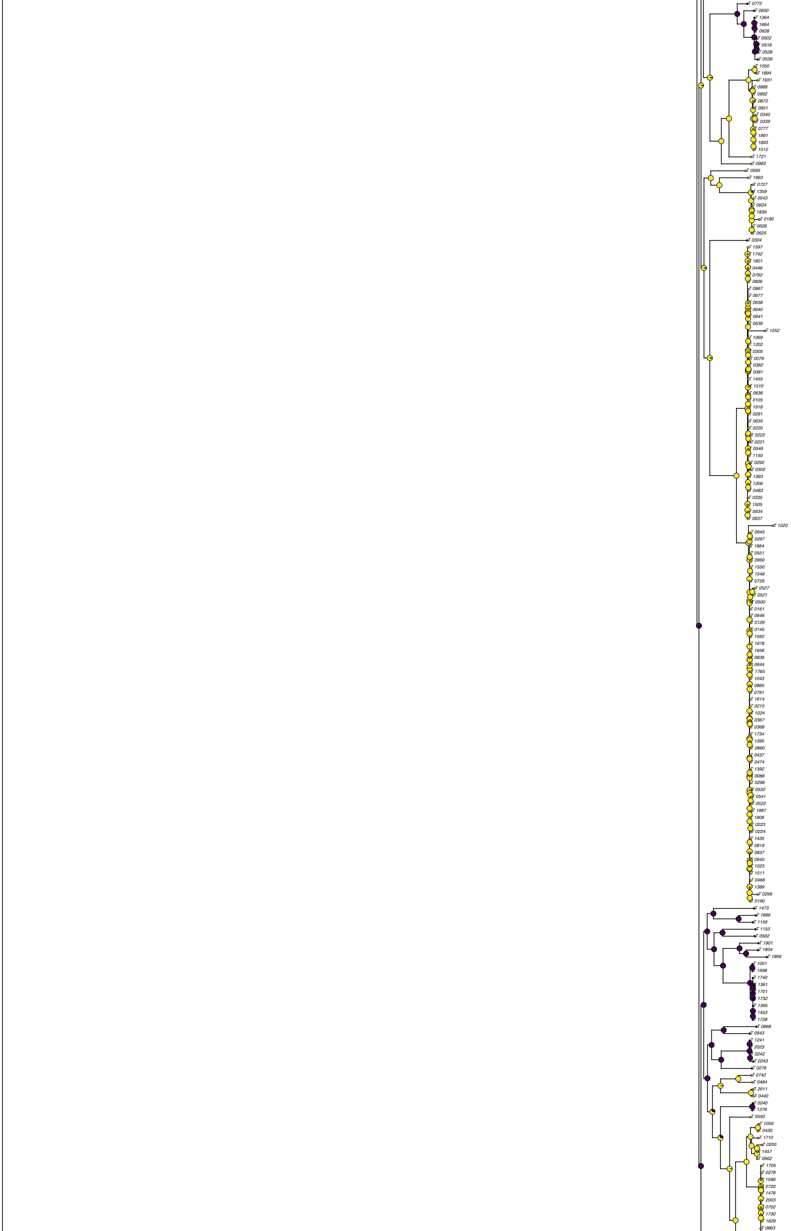

T 0772
T 0650
T 1364
T 1664
T 0628
T 0502
T 0518
T 0528
T 0539
T 1050
T 1894
T 1931
T 0989
T 0992
T 0872
T 0951
T 0340
T 0339
T 0777
T 1891
T 1893
T 1512
T 1721
T 0983
T 0595
T 1863
T 0727
T 1359
T 0543
T 0624
T 1839
T 0180
T 0626
T 0625
T 0304
T 1597
T 1742
T 1801
T 0446
T 0792
T 0806
T 0867
T 0677
T 0638
T 0640
T 0641
T 0639
T 1252
T 1069
T 1202
T 0305
T 0079
T 0382
T 0381
T 1455
T 1510
T 0636
T 0105
T 1918
T 0291
T 0635
T 0220
T 0222
T 0221
T 0546
T 1150
T 0292
T 0302
T 1393
T 1306
T 0483
T 0335
T 1505
T 0934
T 0637
T 1020
T 0645
T 0297
T 1884
T 0551
T 0660
T 1550
T 1549
T 0729
T 0527
T 0521
T 0500
T 0161
T 0646
T 0129
T 0145
T 1592
T 1978
T 1658
T 0836
T 0644
T 1765
T 1043
T 0865
T 0791
T 1614
T 0215
T 1024
T 0367
T 0368
T 1734
T 1395
T 0860
T 0437
T 0474
T 1392
T 0086
T 0298
T 0532
T 0541
T 0522
T 1987
T 1806
T 0223
T 0224
T 1435
T 0819
T 0837
T 0945
T 1023
T 1511
T 0466
T 1389
T 0299
T 0190
T 1473
T 1689
T 1156
T 1153
T 0562
T 1901
T 1804
T 1869
T 1051
T 1998
T 1740
T 1361
T 1701
T 1732
T 1365
T 1453
T 1728
T 0868
T 0943
T 1241
T 2023
T 0242
T 0243
T 0276
T 0742
T 0484
T 2011
T 0440
T 0240
T 1276
T 0550
T 1059
T 0430
T 1710
T 0200
T 1457
T 0662
T 1705
T 0278
T 1586
T 0720
T 1476
T 2003
T 0702
T 1730
T 1629
T 0863

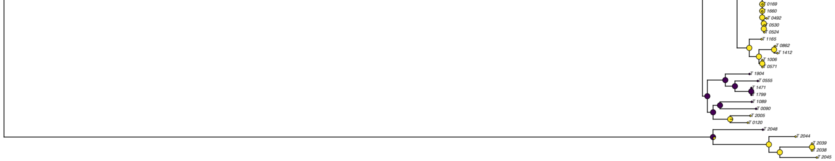
T 0169
T 1660
T 0492
T 0530
T 0524
T 1165
T 0862
T 1412
T 1006
T 0571
T 1904
T 0555
T 1471
T 1799
T 1089
T 0090
T 2005
T 0120
T 2048
T 2044
T 2039
T 2038
T 2045

Effector gene: pldA

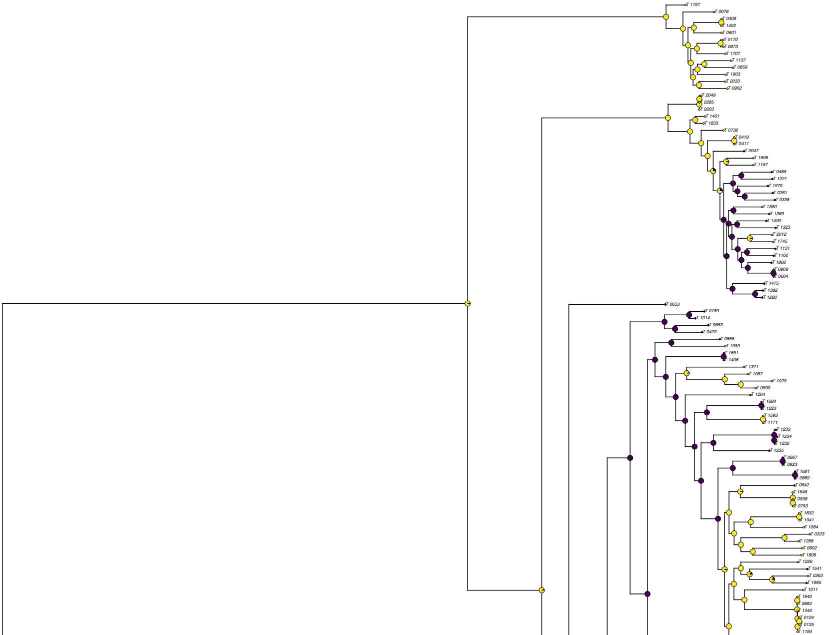

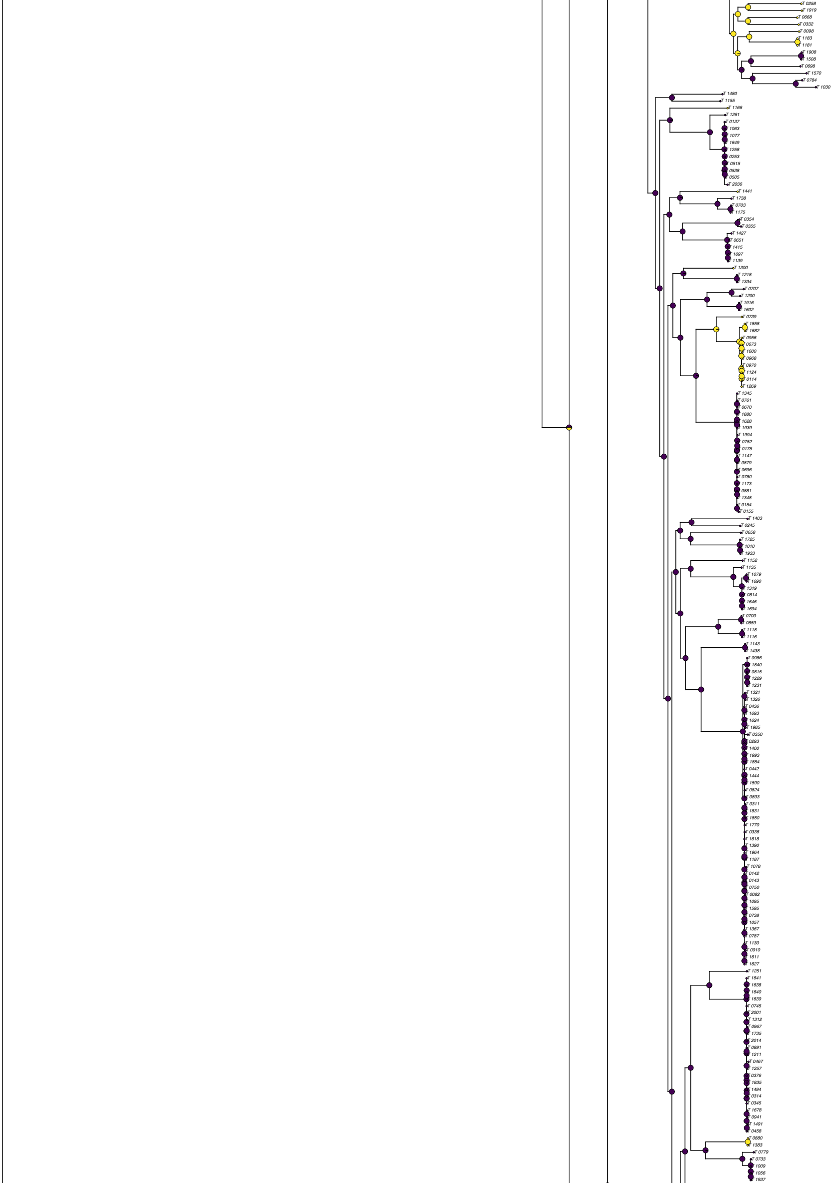
T 0258
T 1919
T 0668
T 0332
T 0098
T 1183
T 1181
T 1908
T 1508
T 0698
T 1570
T 0784
T 1030
T 1480
T 1155
T 1166
T 1261
T 0137
1063
1077
1649
1258
0253
0515
0538
0505
T 2036
T 1441
T 1738
T 0703
1175
T 0354
T 0355
T 1427
T 0651
1415
1697
1139
T 1300
T 1218
T 1334
T 0707
T 1200
T 1916
T 1602
T 0739
T 1858
1682
T 0956
0673
1600
0968
T 0970
1124
0114
T 1269
T 1345
T 0761
0670
1880
1628
1939
T 1994
0752
0175
T 1147
0879
0696
T 0780
1173
0881
T 1348
T 0154
T 0155
T 1403
T 0245
T 0658
T 1725
1010
1933
T 1152
T 1135
T 1079
1690
1319
0814
1646
1694
T 0700
T 0659
T 1118
T 1116
T 1143
1438
T 0986
1840
0815
1229
1231
T 1321
T 1326
T 0436
1693
1624
T 1985
T 0350
0293
1400
1993
1854
T 0442
1444
1590
T 0824
0893
T 0311
1831
1850
T 1770
T 0336
T 1618
T 1390
1964
1187
T 1078
0142
0143
0750
0082
1095
1595
0738
1057
T 1367
0787
T 1130
T 0910
1611
1627
T 1251
T 1641
1638
1640
1639
T 0745
2001
T 1312
T 0967
1735
2014
T 0891
1211
T 0467
1257
0376
1835
1494
0314
T 0345
T 1678
0941
T 1491
0458
T 0880
1383
T 0779
T 0733
1009
1056
1937

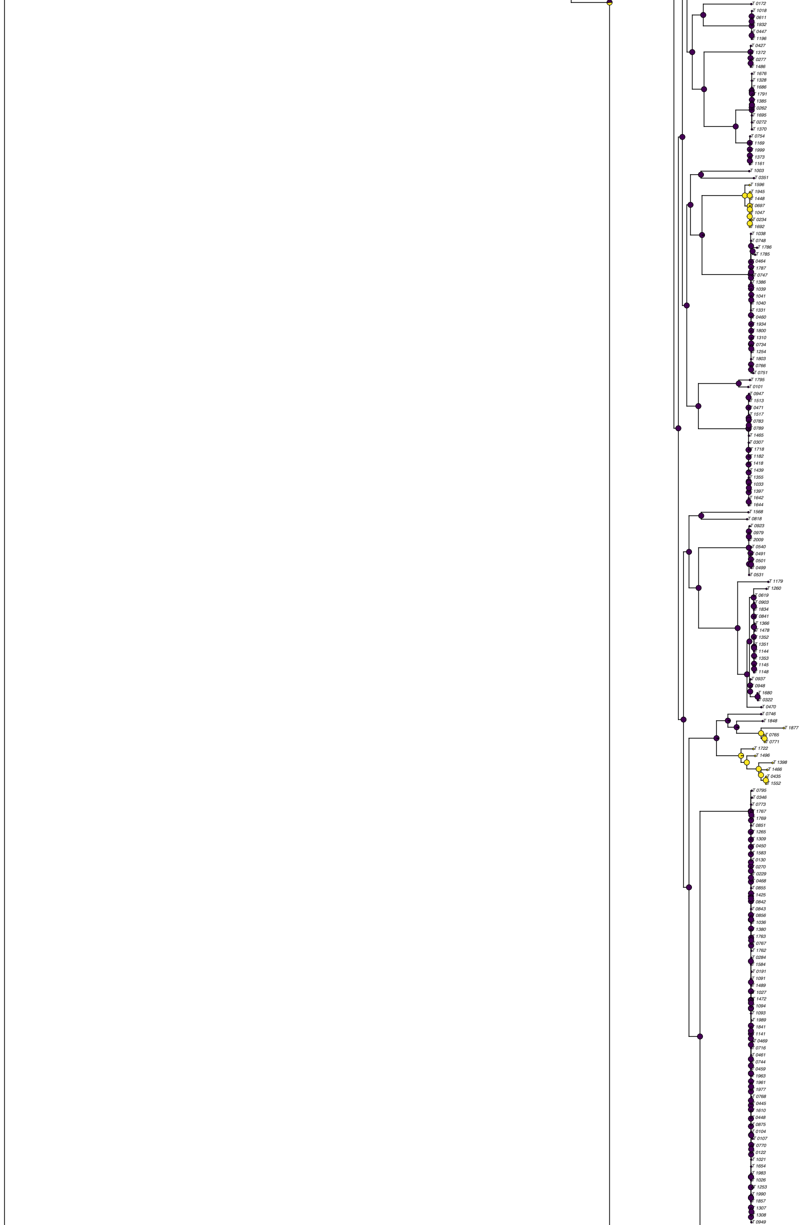

T 0172
T 1018
0611
1932
T 0447
T 1196
T 0427
1372
T 0277
T 1486
T 1676
T 1328
T 1686
1791
1385
0262
T 1695
T 0272
T 1370
T 0754
1169
1999
1373
T 1161
T 1003
T 0351
T 1596
T 1945
T 1448
0697
1047
T 0234
T 1692
T 1038
T 0748
T 1786
T 1785
0464
1787
T 0747
T 1386
1039
1041
1040
T 1331
0460
1934
1800
1310
0734
T 1254
T 1803
0766
T 0751
T 1795
T 0101
T 0947
T 1513
0471
T 1517
0783
0789
T 1465
T 0307
T 1718
T 1182
T 1418
T 1439
T 1355
1033
1397
T 1642
T 1644
T 1568
T 0818
T 0923
0979
T 2009
T 0540
0491
0501
T 0499
T 0531
T 1179
T 1260
T 0619
T 0903
T 1834
T 0841
T 1366
T 1478
1352
T 1351
1144
1353
1145
T 1148
T 0937
0948
T 1680
T 0322
T 0470
T 0746
T 1848
T 1877
T 0765
T 0771
T 1722
T 1496
T 1398
T 1466
T 0435
T 1552
T 0795
T 0346
T 0773
T 1767
T 1769
T 0851
T 1265
T 1309
T 0450
T 1583
T 0130
T 0270
T 0229
T 0468
T 0855
T 1425
T 0842
T 0843
T 0856
T 1036
T 1380
T 1763
T 0767
T 1762
T 0284
T 1584
T 0191
T 1091
T 1489
T 1027
T 1472
T 1094
T 1093
T 1989
T 1841
T 1141
T 0469
T 0716
T 0461
T 0744
T 0459
T 1963
T 1961
T 1977
T 0768
T 0445
T 1610
T 0448
T 0875
T 0104
T 0107
T 0770
T 0122
T 1021
T 1654
T 1983
T 1026
T 1253
T 1990
T 1857
T 1307
T 1308
T 0949

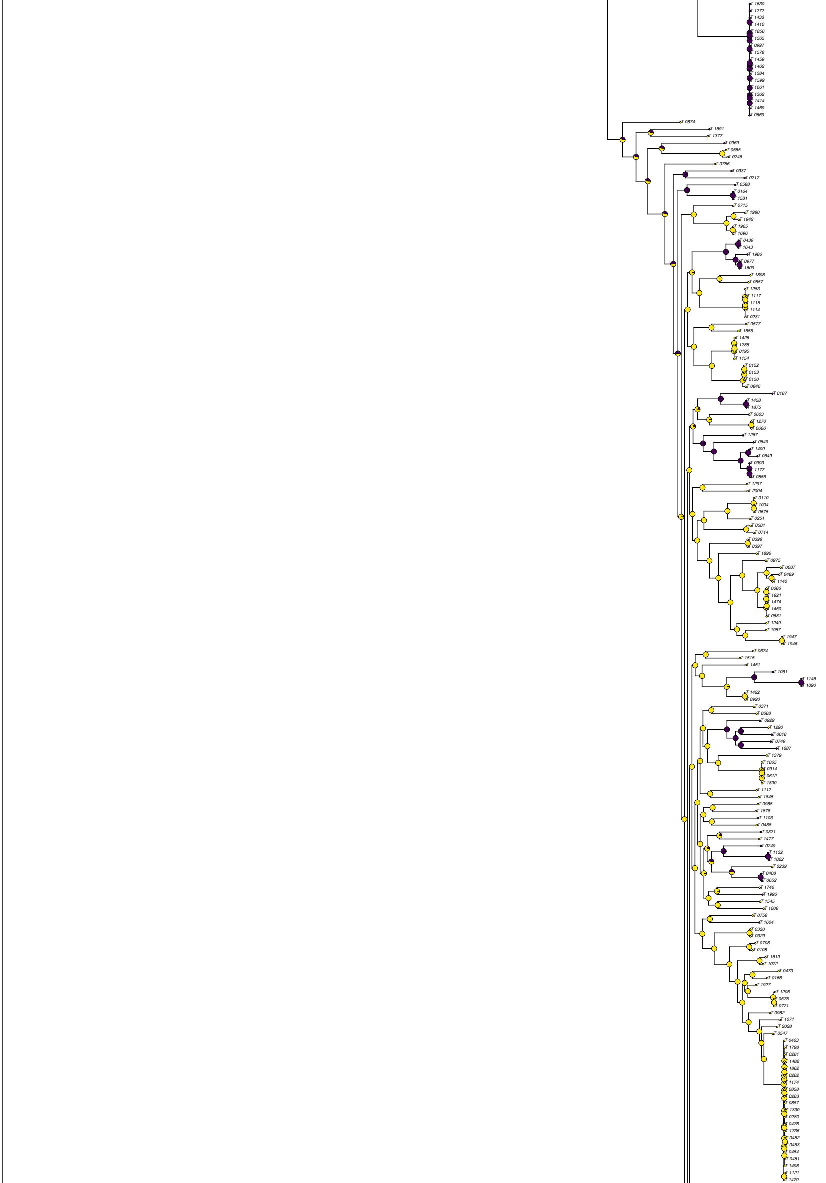
T 1630
T 1272
T 1433
T 1410
T 1856
T 1565
T 0997
T 1578
T 1459
T 1462
T 1384
T 1599
T 1661
T 1362
T 1414
T 1469
T 0669
T 0874
T 1691
T 1377
T 0969
T 0585
T 0246
T 0756
T 0337
T 0217
T 0588
T 0164
T 1531
T 0715
T 1980
T 1942
T 1965
T 1696
T 0439
T 1643
T 1986
T 0977
T 1609
T 1898
T 0557
T 1283
T 1117
T 1115
T 1114
T 0231
T 0577
T 1655
T 1426
T 1285
T 0195
T 1154
T 0152
T 0153
T 0150
T 0846
T 0187
T 1458
T 1875
T 0603
T 1270
T 0666
T 1267
T 0549
T 1409
T 0649
T 0993
T 1177
T 0556
T 1297
T 2004
T 0110
T 1004
T 0675
T 0251
T 0581
T 0714
T 0398
T 0397
T 1896
T 0975
T 0087
T 0489
T 1140
T 0686
T 1921
T 1474
T 1450
T 0681
T 1249
T 1957
T 1947
T 1946
T 0674
T 1515
T 1451
T 1061
T 1146
T 1090
T 1422
T 0920
T 0371
T 0688
T 0929
T 1290
T 0618
T 0749
T 1687
T 1379
T 1065
T 0914
T 0612
T 1890
T 1112
T 1845
T 0985
T 1878
T 1103
T 0488
T 0321
T 1477
T 0249
T 1132
T 1022
T 0239
T 0408
T 0652
T 1746
T 1996
T 1545
T 1608
T 0758
T 1604
T 0330
T 0329
T 0708
T 0108
T 1619
T 1072
T 0473
T 0166
T 1927
T 1206
T 0575
T 0721
T 0982
T 1071
T 2028
T 0547
T 0463
T 1798
T 0281
T 1482
T 1862
T 0282
T 1174
T 0858
T 0283
T 0857
T 1330
T 0280
T 0476
T 1736
T 0452
T 0453
T 0454
T 0451
T 1498
T 1121
T 1479

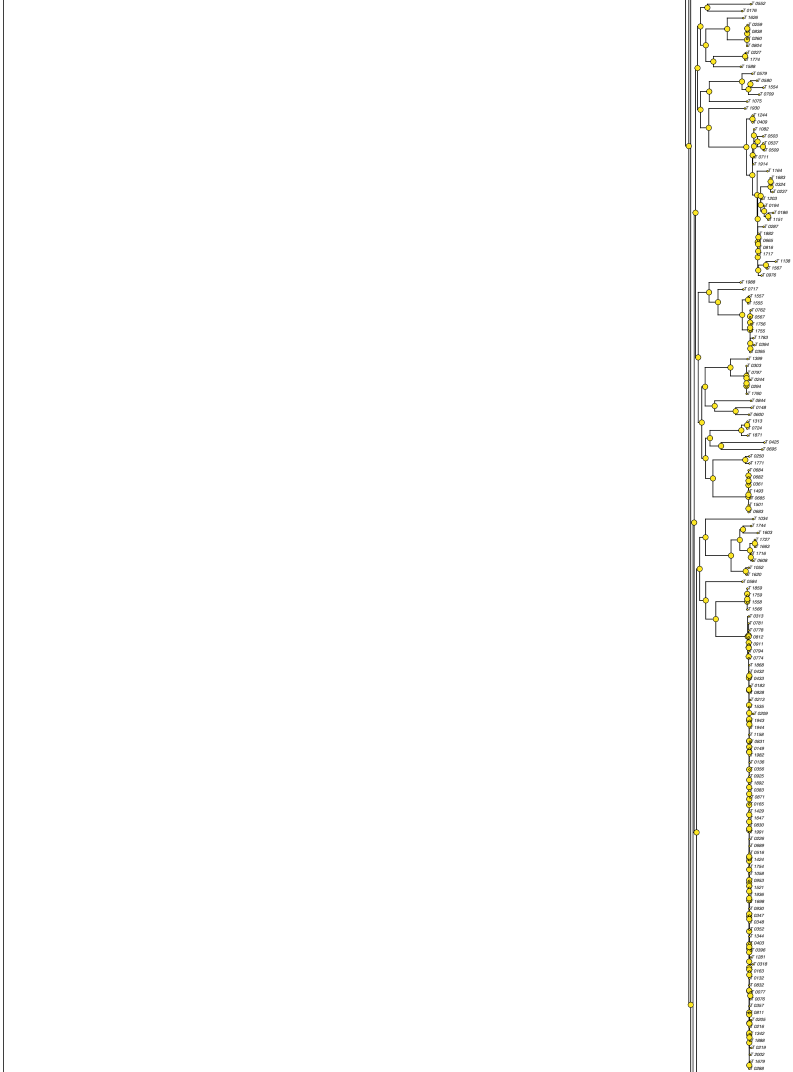
T 0552
T 0176
T 1626
T 0259
T 0838
T 0260
T 0804
T 0227
T 1774
T 1588
T 0579
T 0580
T 1554
T 0709
T 1075
T 1930
T 1244
T 0409
T 1082
T 0503
T 0537
T 0509
T 0711
T 1914
T 1164
T 1683
T 0324
T 0237
T 1203
T 0194
T 0186
T 1151
T 0287
T 1882
T 0665
T 0816
T 1717
T 1138
T 1567
T 0976
T 1988
T 0717
T 1557
T 1555
T 0762
T 0567
T 1756
T 1755
T 1783
T 0394
T 0395
T 1399
T 0303
T 0797
T 0244
T 0294
T 1760
T 0844
T 0148
T 0600
T 1313
T 0724
T 1871
T 0425
T 0695
T 0250
T 1771
T 0684
T 0682
T 0361
T 1493
T 0685
T 1501
T 0683
T 1034
T 1744
T 1603
T 1727
T 1663
T 1716
T 0608
T 1052
T 1620
T 0584
T 1859
T 1759
T 1558
T 1566
T 0313
T 0781
T 0778
T 0812
T 0911
T 0794
T 0774
T 1868
T 0432
T 0433
T 0183
T 0828
T 0213
T 1535
T 0209
T 1943
T 1944
T 1158
T 0831
T 0149
T 1982
T 0136
T 0356
T 0925
T 1892
T 0383
T 0871
T 0165
T 1429
T 1647
T 0830
T 1991
T 0226
T 0689
T 0516
T 1424
T 1754
T 1058
T 0953
T 1521
T 1936
T 1698
T 0930
T 0347
T 0348
T 0352
T 1344
T 0403
T 0396
T 1281
T 0318
T 0163
T 0132
T 0832
T 0077
T 0076
T 0357
T 0811
T 0205
T 0216
T 1342
T 1888
T 0219
T 2002
T 1679
T 0288

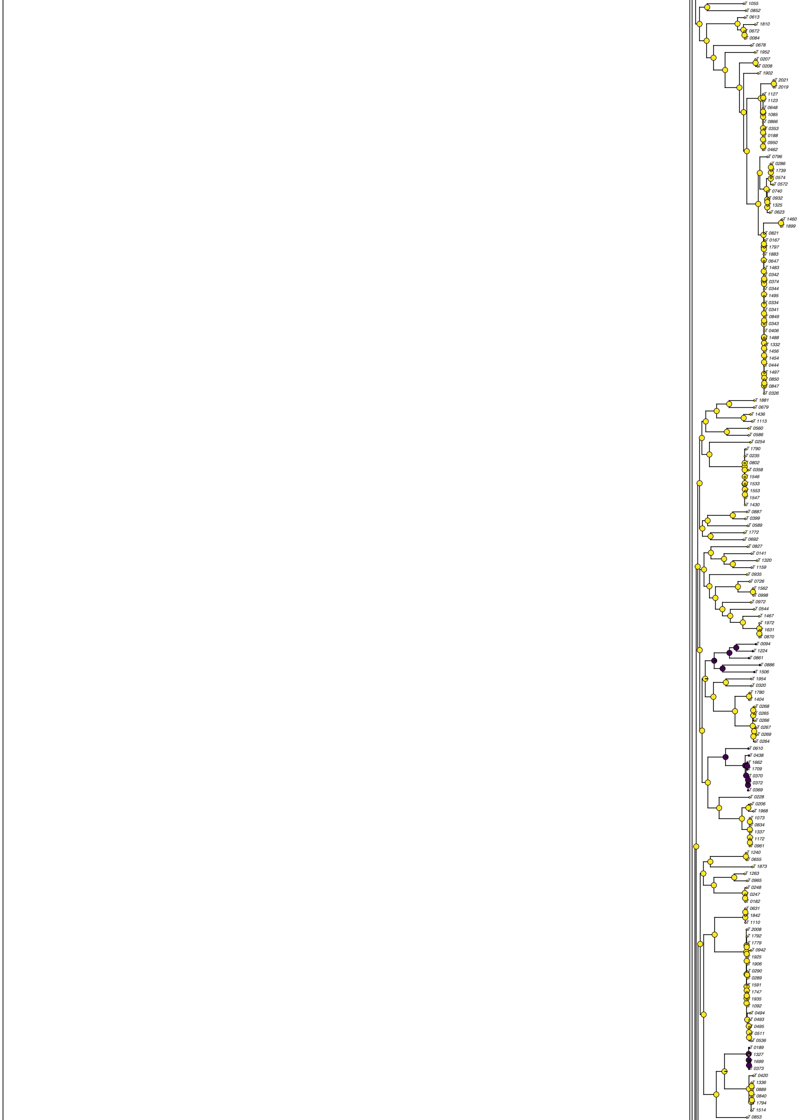
T 1055
T 0852
T 0613
T 1810
T 0672
T 0084
T 0678
T 1952
T 0207
T 0208
T 1902
T 2021
T 2019
T 1127
T 1123
T 0648
T 1085
T 0866
T 0353
T 0188
T 0950
T 0462
T 0796
T 0286
T 1739
T 0574
T 0572
T 0740
T 0932
T 1325
T 0623
T 1460
T 1899
T 0821
T 0167
T 1797
T 1883
T 0647
T 1483
T 0342
T 0374
T 0344
T 1495
T 0334
T 0341
T 0849
T 0343
T 0406
T 1488
T 1332
T 1456
T 1454
T 0444
T 1497
T 0850
T 0847
T 0326
T 1881
T 0679
T 1436
T 1113
T 0560
T 0586
T 0254
T 1790
T 0235
T 0802
T 0358
T 1546
T 1533
T 1553
T 1547
T 1430
T 0887
T 0399
T 0589
T 1772
T 0692
T 0827
T 0141
T 1320
T 1159
T 0935
T 0726
T 1562
T 0998
T 0972
T 0544
T 1467
T 1972
T 1631
T 0870
T 0094
T 1224
T 0861
T 0886
T 1506
T 1954
T 0320
T 1780
T 1404
T 0268
T 0265
T 0266
T 0267
T 0269
T 0264
T 0610
T 0438
T 1662
T 1709
T 0370
T 0372
T 0369
T 0228
T 0206
T 1968
T 1073
T 0834
T 1337
T 1172
T 0961
T 1240
T 0655
T 1873
T 1263
T 0965
T 0248
T 0247
T 0182
T 0631
T 1842
T 1110
T 2008
T 1792
T 1779
T 0942
T 1925
T 1906
T 0290
T 0289
T 1591
T 1747
T 1935
T 1092
T 0494
T 0493
T 0495
T 0511
T 0536
T 0189
T 1327
T 1699
T 0373
T 0420
T 1336
T 0889
T 0840
T 1794
T 1514
T 0853

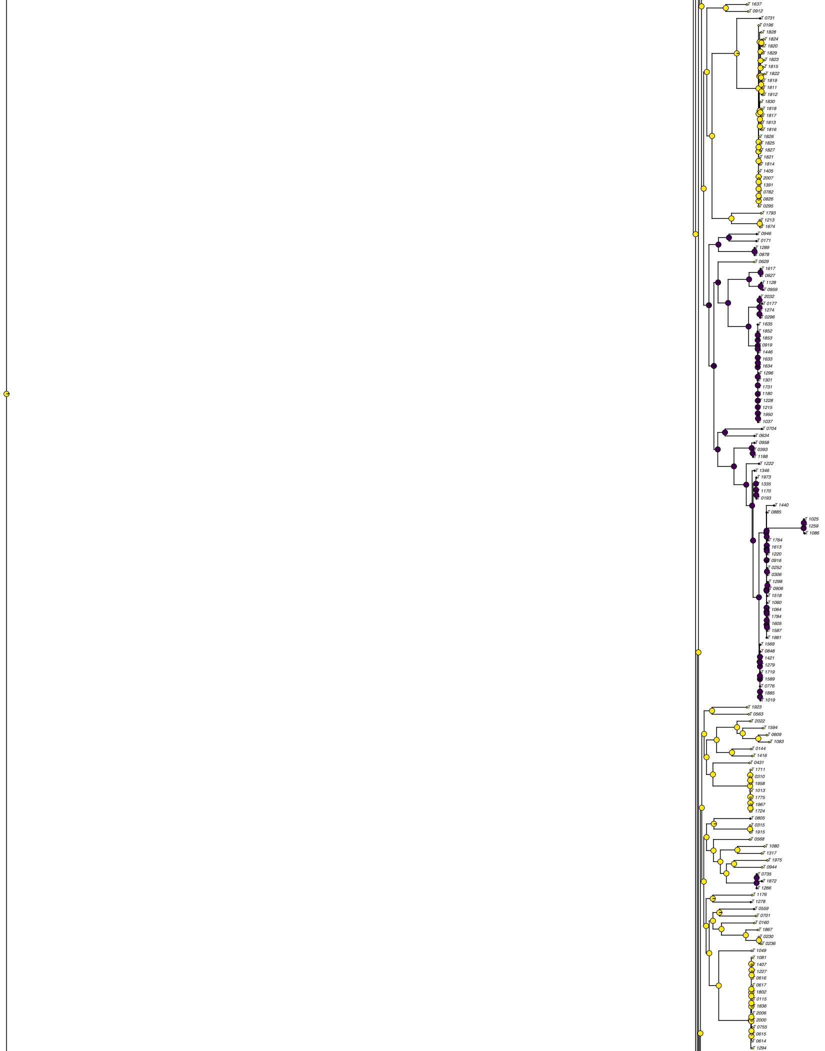

T 1637
T 0912
T 0731
T 0196
T 1828
T 1824
T 1820
T 1829
T 1823
T 1815
T 1822
T 1819
T 1811
T 1812
T 1830
T 1818
T 1817
T 1813
T 1816
T 1826
T 1825
T 1827
T 1821
T 1814
T 1405
2007
1391
0782
0826
T 0295
T 1793
T 1213
T 1674
T 0946
T 0171
T 1289
T 0878
T 0629
T 1617
T 0627
T 1128
T 0959
T 2032
T 0177
T 1274
T 0296
T 1635
T 1852
1853
0919
T 1446
1633
1634
T 1296
T 1301
T 1731
1180
T 1228
1215
1950
T 1037
T 0704
T 0634
T 0958
T 0393
T 1188
T 1222
T 1346
T 1973
1335
1170
T 0193
T 1440
T 0885
T 1025
1259
T 1086
T 1764
1613
T 1220
0916
T 0252
T 0306
T 1298
T 0906
T 1518
T 1060
1064
T 1784
1605
T 1587
T 1981
T 1569
T 0848
1421
1279
T 1719
1589
T 0776
1885
T 1019
T 1923
T 0563
T 2022
T 1594
T 0609
T 1083
T 0144
T 1416
T 0431
T 1711
0310
1958
T 1013
1775
1967
1724
T 0805
T 0315
T 1915
T 0568
T 1080
T 1317
T 1975
T 0944
T 0735
T 1872
T 1266
T 1176
T 1278
T 0559
T 0701
T 0160
T 1867
T 0230
T 0236
T 1049
T 1081
1407
1227
0616
T 0617
1802
T 0115
1836
T 2006
2000
T 0755
0615
T 0614
T 1294

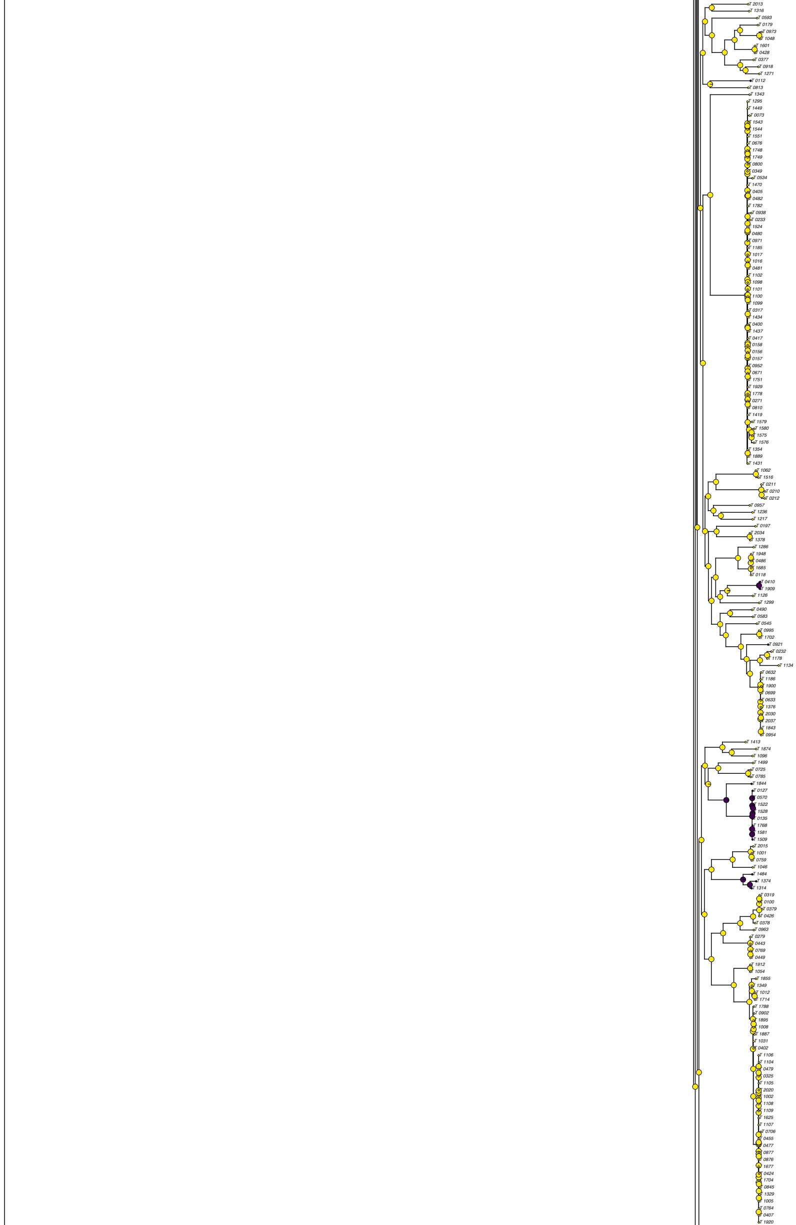
T 2013
T 1316
T 0593
T 0179
T 0973
T 1048
T 1601
T 0428
T 0377
T 0918
T 1271
T 0112
T 0813
T 1343
T 1295
T 1449
T 0073
T 1543
T 1544
T 1551
T 0676
T 1748
T 1749
T 0800
T 0349
T 0534
T 1470
T 0405
T 0482
T 1782
T 0938
T 0233
T 1524
T 0480
T 0971
T 1185
T 1017
T 1016
T 0481
T 1102
T 1098
T 1101
T 1100
T 1099
T 0317
T 1434
T 0400
T 1437
T 0417
T 0158
T 0156
T 0157
T 0952
T 0671
T 1751
T 1929
T 1778
T 0271
T 0810
T 1419
T 1579
T 1580
T 1575
T 1576
T 1354
T 1889
T 1431
T 1062
T 1516
T 0211
T 0210
T 0212
T 0957
T 1236
T 1217
T 0197
T 2034
T 1378
T 1286
T 1948
T 0486
T 1685
T 0118
T 0410
T 1909
T 1126
T 1299
T 0490
T 0583
T 0545
T 0995
T 1702
T 0921
T 0232
T 1178
T 1134
T 0632
T 1186
T 1900
T 0699
T 0633
T 1376
T 2030
T 2037
T 1843
T 0954
T 1413
T 1874
T 1096
T 1499
T 0725
T 0785
T 1844
T 0127
T 0570
T 1522
T 1528
T 0135
T 1768
T 1581
T 1509
T 2015
T 1001
T 0759
T 1046
T 1484
T 1374
T 1314
T 0319
T 0100
T 0379
T 0426
T 0378
T 0963
T 0279
T 0443
T 0769
T 0449
T 1912
T 1054
T 1855
T 1349
T 1012
T 1714
T 1788
T 0902
T 1895
T 1008
T 1887
T 1031
T 0402
T 1106
T 1104
T 0479
T 0325
T 1105
T 2020
T 1002
T 1108
T 1109
T 1625
T 1107
T 0706
T 0455
T 0477
T 0877
T 0876
T 1677
T 0424
T 1704
T 0845
T 1329
T 1005
T 0764
T 0407
T 1920

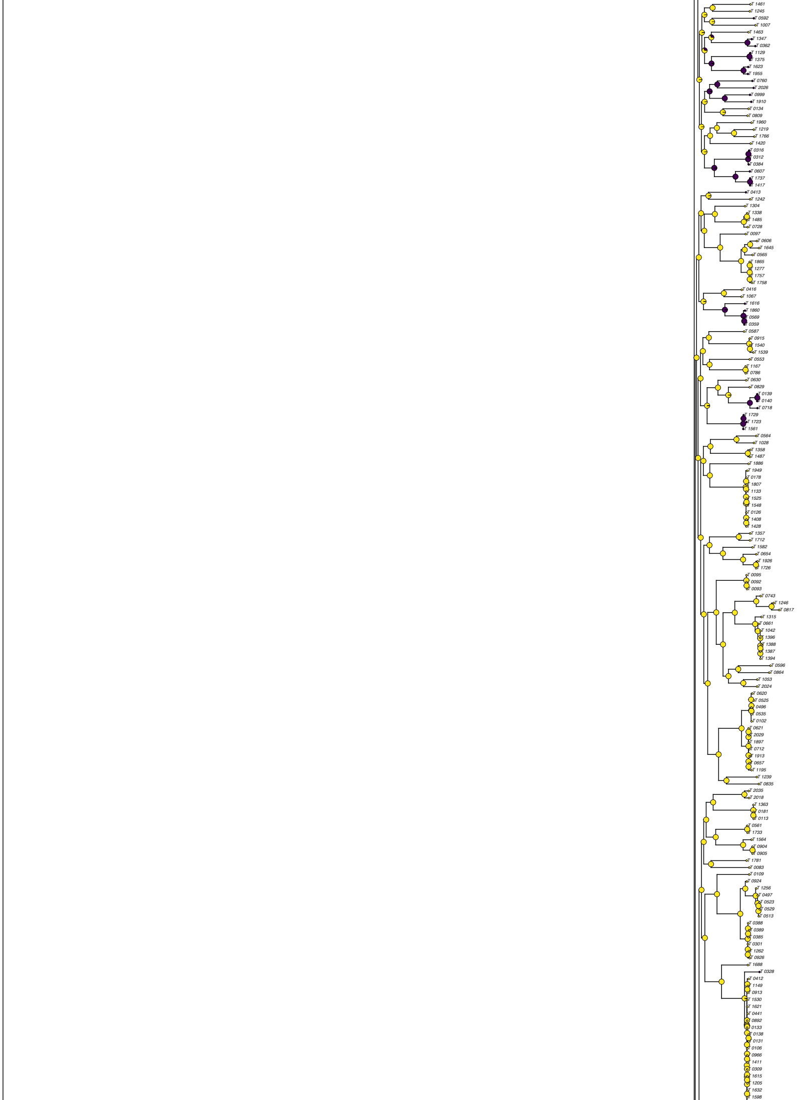
T 1461
T 1245
T 0592
T 1007
T 1463
T 1347
T 0362
T 1129
T 1375
T 1623
T 1955
T 0760
T 2026
T 0999
T 1910
T 0134
T 0809
T 1960
T 1219
T 1766
T 1420
T 0316
T 0312
T 0384
T 0607
T 1737
T 1417
T 0413
T 1242
T 1304
T 1338
T 1485
T 0728
T 0097
T 0606
T 1645
T 0565
T 1865
1277
1757
T 1758
T 0416
T 1067
T 1616
T 1860
T 0569
T 0359
T 0587
T 0915
T 1540
T 1539
T 0553
T 1167
T 0786
T 0630
T 0829
T 0139
T 0140
T 0718
T 1729
T 1723
T 1561
T 0564
T 1028
T 1358
T 1487
T 1886
T 1949
T 0178
T 1807
T 1133
T 1525
T 1548
T 0126
T 1408
T 1428
T 1357
T 1712
T 1582
T 0654
T 1926
1726
T 0095
0092
T 0093
T 0743
T 1246
T 0817
T 1315
T 0661
T 1042
1396
T 1388
1387
T 1394
T 0596
T 0864
T 1053
T 2024
T 0620
T 0525
0496
0535
T 0102
T 0621
2029
T 1897
T 0712
1913
T 0657
T 1195
T 1239
T 0835
T 2035
T 2018
T 1363
0181
T 0113
T 0561
T 1733
T 1564
T 0904
T 0905
T 1781
T 0083
T 0109
T 0924
T 1256
T 0497
T 0523
T 0529
T 0513
T 0388
0389
T 0385
T 0301
T 1262
T 0926
T 1688
T 0328
T 0412
1149
0913
T 1530
T 1621
T 0441
0892
0133
T 0138
T 0131
T 0106
0966
1411
0309
1615
1205
T 1632
T 1598

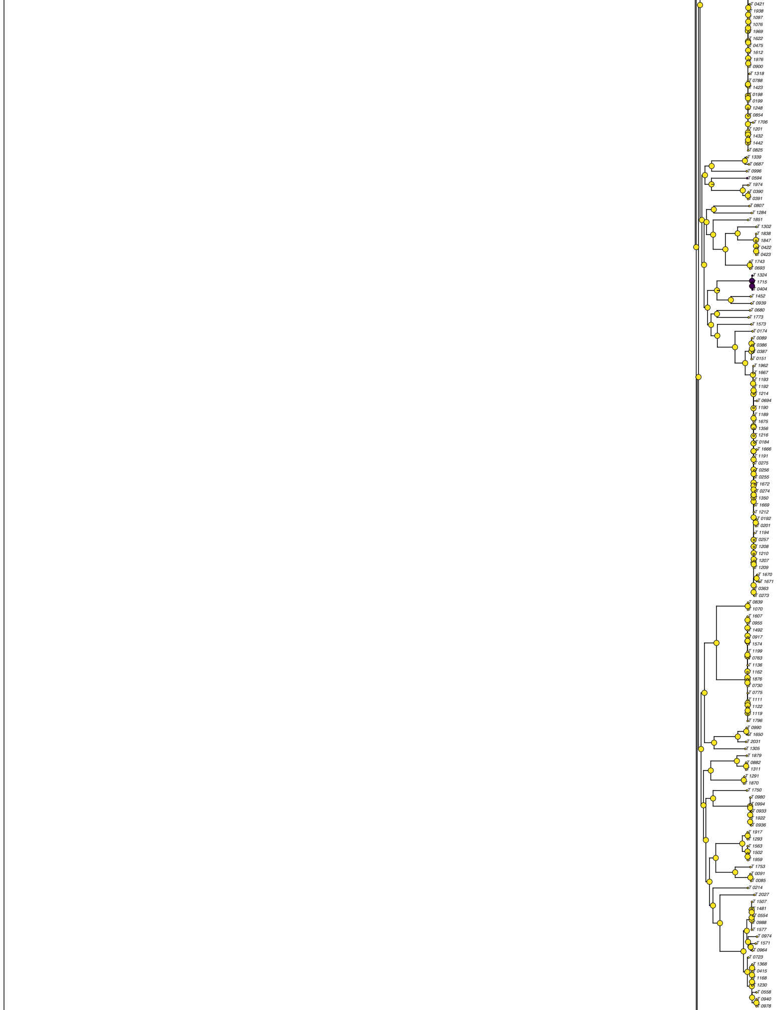
T 0421
T 1938
T 1097
T 1076
T 1969
T 1622
T 0475
T 1612
T 1976
T 0900
T 1318
T 0788
T 1423
T 0198
T 0199
T 1248
T 0854
T 1706
T 1201
T 1432
T 1442
T 0825
T 1339
T 0687
T 0996
T 0594
T 1974
T 0390
T 0391
T 0807
T 1284
T 1851
T 1302
T 1838
T 1847
T 0422
T 0423
T 1743
T 0693
T 1324
T 1715
T 0404
T 1452
T 0939
T 0680
T 1773
T 1573
T 0174
T 0089
T 0386
T 0387
T 0151
T 1962
T 1667
T 1193
T 1192
T 1214
T 0694
T 1190
T 1189
T 1675
T 1356
T 1216
T 0184
T 1666
T 1191
T 0275
T 0256
T 0255
T 1672
T 0274
T 1350
T 1669
T 1212
T 0192
T 0201
T 1194
T 0257
T 1208
T 1210
T 1207
T 1209
T 1670
T 1671
T 0363
T 0273
T 0839
T 1070
T 1607
T 0955
T 1492
T 0917
T 1574
T 1199
T 0763
T 1136
T 1162
T 1876
T 0730
T 0775
T 1111
T 1122
T 1119
T 1796
T 0990
T 1650
T 2031
T 1305
T 1879
T 0882
T 1311
T 1291
T 1870
T 1750
T 0980
T 0994
T 0933
T 1922
T 0936
T 1917
T 1293
T 1563
T 1502
T 1959
T 1753
T 0091
T 0085
T 0214
T 2027
T 1507
T 1481
T 0554
T 0988
T 1577
T 0974
T 1571
T 0964
T 0723
T 1368
T 0415
T 1168
T 1230
T 0558
T 0940
T 0978

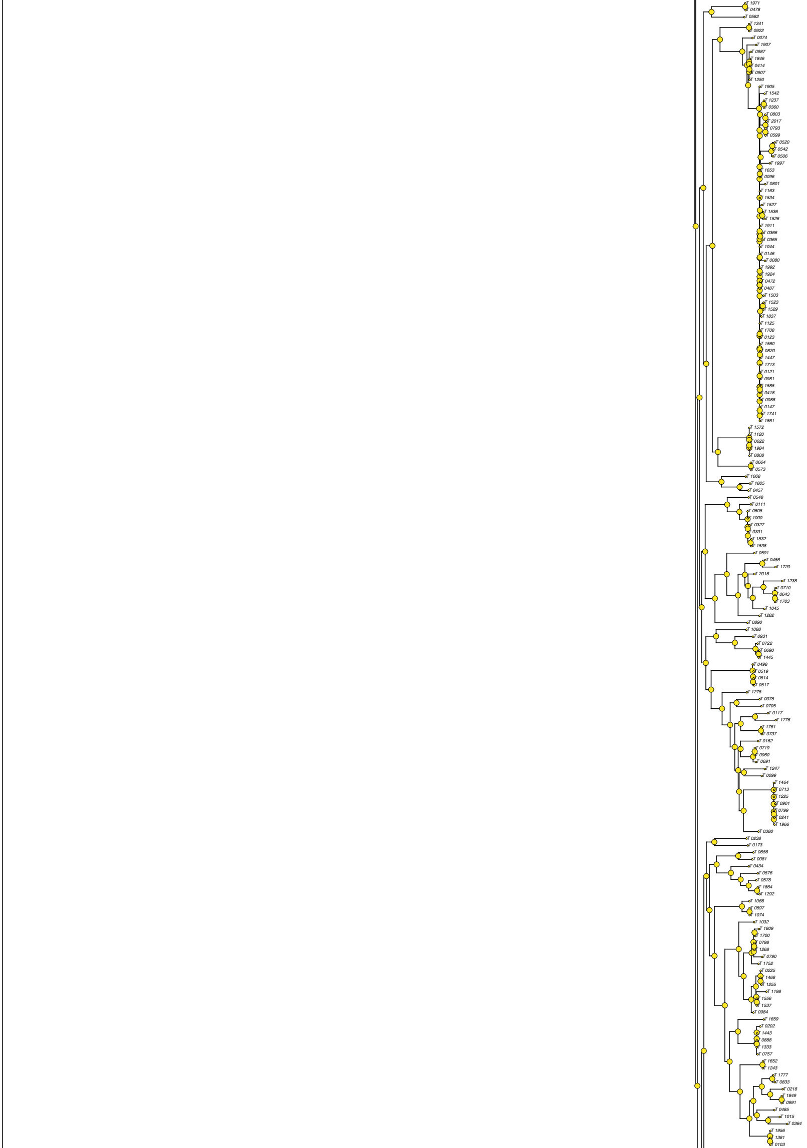

T 1971
T 0478
T 0582
T 1341
T 0922
T 0074
T 1907
T 0987
T 1846
T 0414
T 0907
T 1250
T 1905
T 1542
T 1237
T 0360
T 0803
T 2017
T 0793
T 0599
T 0520
T 0542
T 0506
T 1997
T 1653
T 0096
T 0801
T 1163
T 1534
T 1527
T 1536
T 1526
T 1911
T 0366
T 0365
T 1044
T 0146
T 0080
T 1992
T 1924
T 0472
T 0487
T 1503
T 1523
T 1529
T 1837
T 1125
T 1708
T 0123
T 1560
T 0820
T 1447
T 1713
T 0121
T 0981
T 1585
T 0418
T 0088
T 0147
T 1741
T 1861
T 1572
T 1120
T 0622
T 1984
T 0808
T 0664
T 0573
T 1068
T 1805
T 0457
T 0548
T 0111
T 0605
T 1000
T 0327
T 0331
T 1532
T 1538
T 0591
T 0456
T 1720
T 2016
T 1238
T 0710
T 0643
T 1703
T 1045
T 1282
T 0890
T 1088
T 0931
T 0722
T 0690
T 1445
T 0498
T 0519
T 0514
T 0517
T 1275
T 0075
T 0705
T 0117
T 1776
T 1761
T 0737
T 0162
T 0719
T 0960
T 0691
T 1247
T 0099
T 1464
T 0713
T 1225
T 0901
T 0799
T 0241
T 1966
T 0380
T 0238
T 0173
T 0656
T 0081
T 0434
T 0576
T 0578
T 1864
T 1292
T 1066
T 0597
T 1074
T 1032
T 1809
T 1700
T 0798
T 1268
T 0790
T 1752
T 0225
T 1468
T 1255
T 1198
T 1556
T 1537
T 0984
T 1659
T 0202
T 1443
T 0888
T 1333
T 0757
T 1652
T 1243
T 1777
T 0833
T 0218
T 1849
T 0991
T 0485
T 1015
T 0364
T 1956
T 1381
T 0103

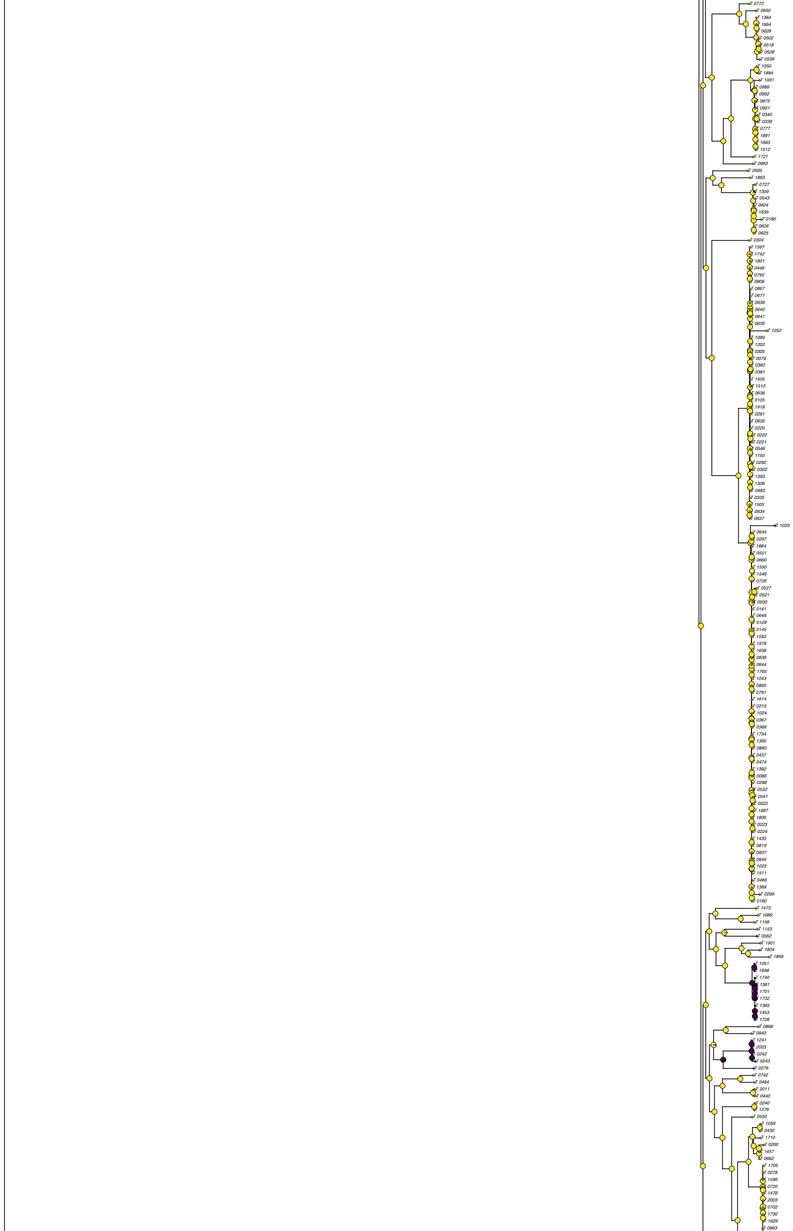
T 0772
T 0650
T 1364
T 1664
T 0628
T 0502
T 0518
T 0528
T 0539
T 1050
T 1894
T 1931
T 0989
T 0992
T 0872
T 0951
T 0340
T 0339
T 0777
T 1891
T 1893
T 1512
T 1721
T 0983
T 0595
T 1863
T 0727
T 1359
T 0543
T 0624
T 1839
T 0180
T 0626
T 0625
T 0304
T 1597
T 1742
T 1801
T 0446
T 0792
T 0806
T 0867
T 0677
T 0638
T 0640
T 0641
T 0639
T 1252
T 1069
T 1202
T 0305
T 0079
T 0382
T 0381
T 1455
T 1510
T 0636
T 0105
T 1918
T 0291
T 0635
T 0220
T 0222
T 0221
T 0546
T 1150
T 0292
T 0302
T 1393
T 1306
T 0483
T 0335
T 1505
T 0934
T 0637
T 1020
T 0645
T 0297
T 1884
T 0551
T 0660
T 1550
T 1549
T 0729
T 0527
T 0521
T 0500
T 0161
T 0646
T 0129
T 0145
T 1592
T 1978
T 1658
T 0836
T 0644
T 1765
T 1043
T 0865
T 0791
T 1614
T 0215
T 1024
T 0367
T 0368
T 1734
T 1395
T 0860
T 0437
T 0474
T 1392
T 0086
T 0298
T 0532
T 0541
T 0522
T 1987
T 1806
T 0223
T 0224
T 1435
T 0819
T 0837
T 0945
T 1023
T 1511
T 0466
T 1389
T 0299
T 0190
T 1473
T 1689
T 1156
T 1153
T 0562
T 1901
T 1804
T 1869
T 1051
T 1998
T 1740
T 1361
T 1701
T 1732
T 1365
T 1453
T 1728
T 0868
T 0943
T 1241
T 2023
T 0242
T 0243
T 0276
T 0742
T 0484
T 2011
T 0440
T 0240
T 1276
T 0550
T 1059
T 0430
T 1710
T 0200
T 1457
T 0662
T 1705
T 0278
T 1586
T 0720
T 1476
T 2003
T 0702
T 1730
T 1629
T 0863

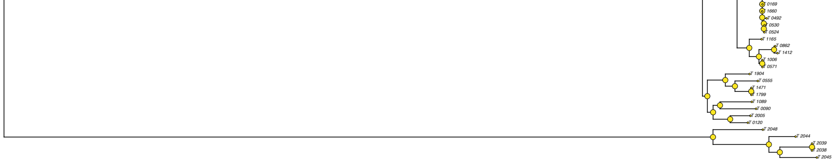

T 0169
T 1660
T 0492
T 0530
T 0524
T 1165
T 0862
T 1412
T 1006
T 0571
T 1904
T 0555
T 1471
T 1799
T 1089
T 0090
T 2005
T 0120
T 2048
T 2044
T 2039
T 2038
T 2045

Effector gene: rhsP2

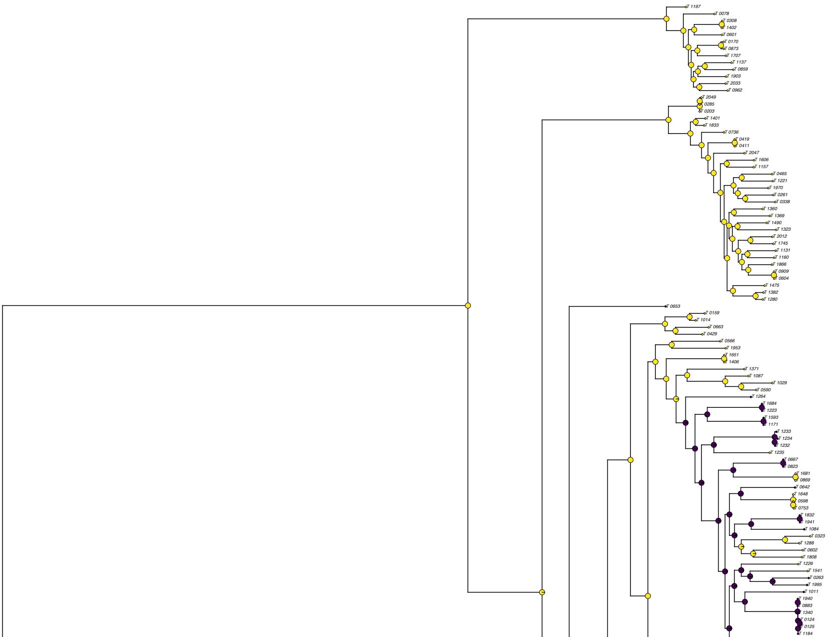

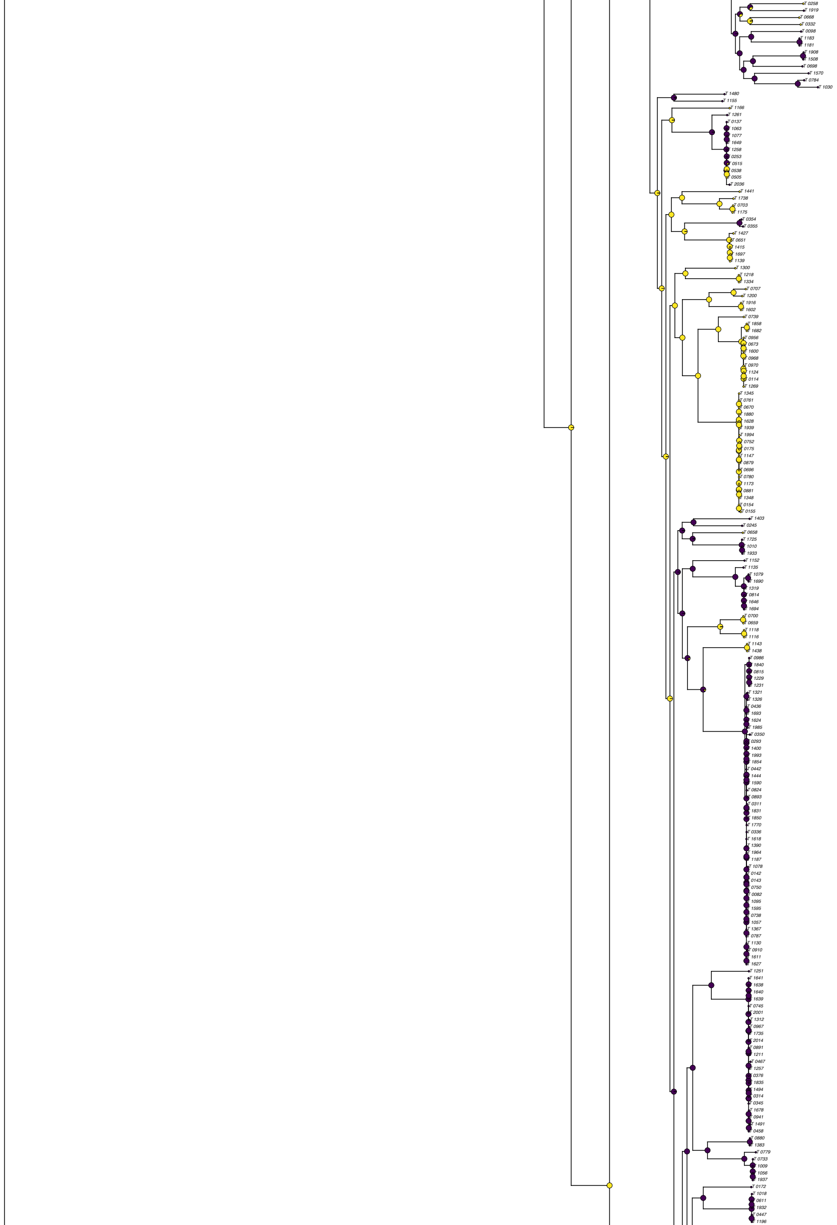
T 0258
T 1919
T 0668
T 0332
T 0098
T 1183
1181
T 1908
T 1508
T 0698
T 1570
T 0784
T 1030
T 1480
T 1155
T 1166
T 1261
T 0137
1063
1077
T 1649
1258
0253
0515
0538
0505
T 2036
T 1441
T 1738
T 0703
T 1175
T 0354
T 0355
T 1427
T 0651
1415
T 1697
1139
T 1300
T 1218
1334
T 0707
T 1200
T 1916
T 1602
T 0739
T 1858
T 1682
T 0956
0673
1600
0968
T 0970
1124
0114
T 1269
T 1345
T 0761
0670
1880
T 1628
1939
T 1994
T 0752
0175
T 1147
0879
T 0696
T 0780
1173
0881
1348
0154
T 0155
T 1403
T 0245
T 0658
T 1725
1010
T 1933
T 1152
T 1135
T 1079
T 1690
1319
0814
1646
T 1694
T 0700
0659
T 1118
T 1116
T 1143
1438
T 0986
1840
0815
1229
T 1231
T 1321
T 1326
T 0436
T 1693
1624
T 1985
T 0350
0293
1400
1993
1854
T 0442
1444
1590
T 0824
0893
T 0311
1831
1850
T 1770
T 0336
T 1618
T 1390
1964
1187
T 1078
0142
0143
0750
T 0082
1095
1595
T 0738
1057
T 1367
T 0787
T 1130
T 0910
1611
T 1627
T 1251
T 1641
1638
1640
1639
T 0745
T 2001
T 1312
T 0967
T 1735
2014
T 0891
1211
T 0467
T 1257
T 0376
1835
1494
0314
T 0345
T 1678
0941
T 1491
T 0458
T 0880
T 1383
T 0779
T 0733
1009
1056
T 1937
T 0172
T 1018
0611
1932
T 0447
T 1196

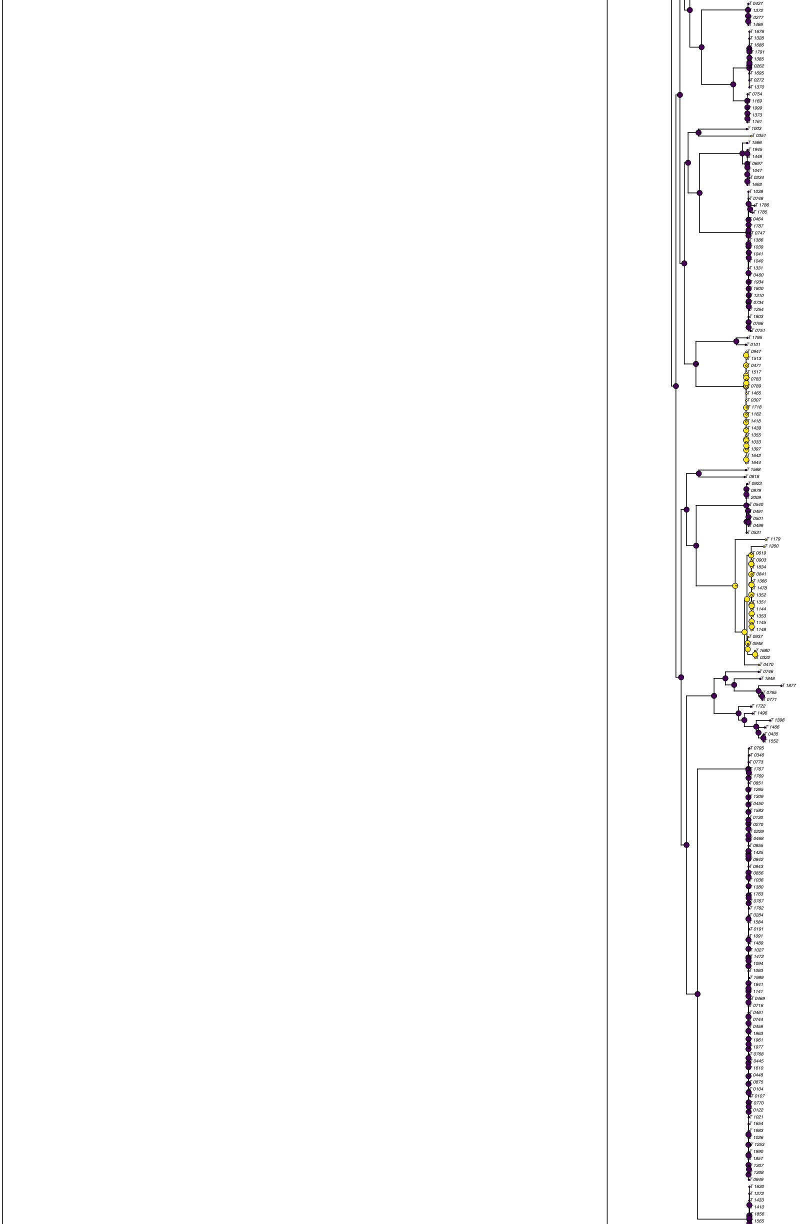
T 0427
1372
0277
1486
T 1676
T 1328
T 1686
1791
1385
0262
T 1695
T 0272
T 1370
T 0754
1169
1999
1373
T 1161
T 1003
T 0351
T 1596
T 1945
T 1448
0697
T 1047
T 0234
1692
T 1038
T 0748
T 1786
T 1785
0464
1787
T 0747
T 1386
1039
1041
T 1040
T 1331
0460
1934
1800
1310
0734
T 1254
T 1803
0766
T 0751
T 1795
T 0101
T 0947
T 1513
0471
T 1517
0783
0789
T 1465
T 0307
T 1718
1182
1418
1439
T 1355
1033
1397
T 1642
1644
T 1568
T 0818
T 0923
0979
T 2009
T 0540
0491
0501
T 0499
T 0531
T 1179
T 1260
T 0619
0903
1834
0841
T 1366
T 1478
1352
T 1351
T 1144
1353
T 1145
1148
T 0937
0948
T 1680
T 0322
T 0470
T 0746
T 1848
T 1877
T 0765
T 0771
T 1722
T 1496
T 1398
T 1466
T 0435
T 1552
T 0795
T 0346
T 0773
1767
1769
T 0851
1265
1309
0450
1583
T 0130
0270
T 0229
0468
T 0855
1425
0842
T 0843
0856
T 1036
1380
1763
0767
T 1762
T 0284
T 1584
T 0191
T 1091
1489
1027
T 1472
1094
T 1093
T 1989
1841
1141
T 0469
T 0716
T 0461
0744
T 0459
1963
1961
1977
T 0768
0445
1610
0448
T 0875
T 0104
T 0107
0770
0122
T 1021
T 1654
T 1983
T 1026
T 1253
T 1990
T 1857
1307
1308
T 0949
T 1630
T 1272
T 1433
1410
T 1856
1565

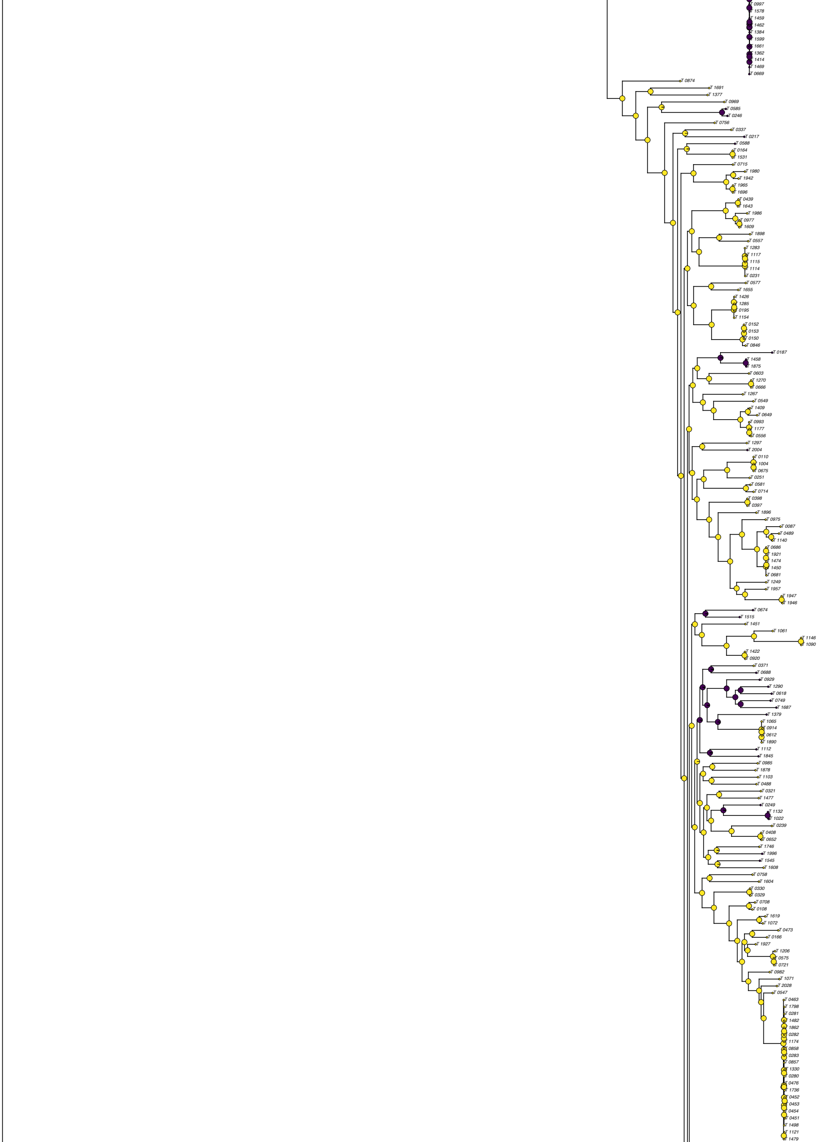
T 0997
T 1578
T 1459
T 1462
T 1384
T 1599
T 1661
T 1362
T 1414
T 1469
T 0669
T 0874
T 1691
T 1377
T 0969
T 0585
T 0246
T 0756
T 0337
T 0217
T 0588
T 0164
T 1531
T 0715
T 1980
T 1942
T 1965
T 1696
T 0439
T 1643
T 1986
T 0977
T 1609
T 1898
T 0557
T 1283
T 1117
T 1115
T 1114
T 0231
T 0577
T 1655
T 1426
T 1285
T 0195
T 1154
T 0152
T 0153
T 0150
T 0846
T 0187
T 1458
T 1875
T 0603
T 1270
T 0666
T 1267
T 0549
T 1409
T 0649
T 0993
T 1177
T 0556
T 1297
T 2004
T 0110
T 1004
T 0675
T 0251
T 0581
T 0714
T 0398
T 0397
T 1896
T 0975
T 0087
T 0489
T 1140
T 0686
T 1921
T 1474
T 1450
T 0681
T 1249
T 1957
T 1947
T 1946
T 0674
T 1515
T 1451
T 1061
T 1146
T 1090
T 1422
T 0920
T 0371
T 0688
T 0929
T 1290
T 0618
T 0749
T 1687
T 1379
T 1065
T 0914
T 0612
T 1890
T 1112
T 1845
T 0985
T 1878
T 1103
T 0488
T 0321
T 1477
T 0249
T 1132
T 1022
T 0239
T 0408
T 0652
T 1746
T 1996
T 1545
T 1608
T 0758
T 1604
T 0330
T 0329
T 0708
T 0108
T 1619
T 1072
T 0473
T 0166
T 1927
T 1206
T 0575
T 0721
T 0982
T 1071
T 2028
T 0547
T 0463
T 1798
T 0281
T 1482
T 1862
T 0282
T 1174
T 0858
T 0283
T 0857
T 1330
T 0280
T 0476
T 1736
T 0452
T 0453
T 0454
T 0451
T 1498
T 1121
T 1479

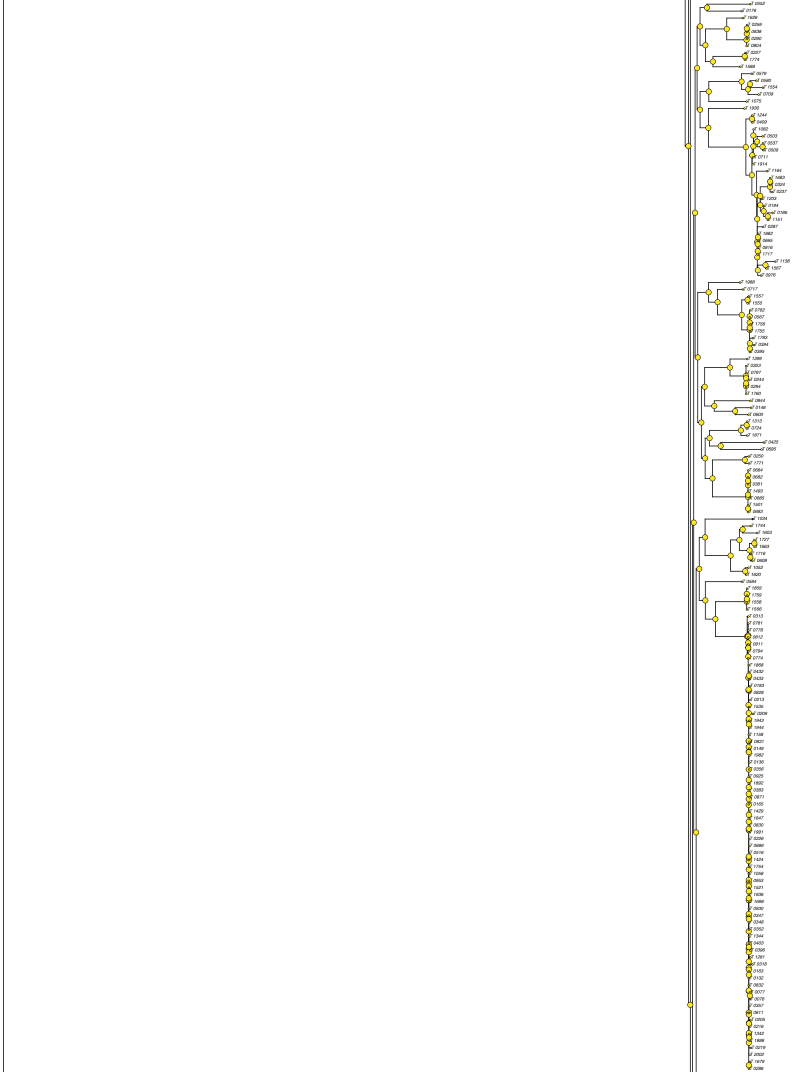
T 0552
T 0176
T 1626
T 0259
T 0838
T 0260
T 0804
T 0227
T 1774
T 1588
T 0579
T 0580
T 1554
T 0709
T 1075
T 1930
T 1244
T 0409
T 1082
T 0503
T 0537
T 0509
T 0711
T 1914
T 1164
T 1683
T 0324
T 0237
T 1203
T 0194
T 0186
T 1151
T 0287
T 1882
T 0665
T 0816
T 1717
T 1138
T 1567
T 0976
T 1988
T 0717
T 1557
T 1555
T 0762
T 0567
T 1756
T 1755
T 1783
T 0394
T 0395
T 1399
T 0303
T 0797
T 0244
T 0294
T 1760
T 0844
T 0148
T 0600
T 1313
T 0724
T 1871
T 0425
T 0695
T 0250
T 1771
T 0684
T 0682
T 0361
T 1493
T 0685
T 1501
T 0683
T 1034
T 1744
T 1603
T 1727
T 1663
T 1716
T 0608
T 1052
T 1620
T 0584
T 1859
T 1759
T 1558
T 1566
T 0313
T 0781
T 0778
T 0812
T 0911
T 0794
T 0774
T 1868
T 0432
T 0433
T 0183
T 0828
T 0213
T 1535
T 0209
T 1943
T 1944
T 1158
T 0831
T 0149
T 1982
T 0136
T 0356
T 0925
T 1892
T 0383
T 0871
T 0165
T 1429
T 1647
T 0830
T 1991
T 0226
T 0689
T 0516
T 1424
T 1754
T 1058
T 0953
T 1521
T 1936
T 1698
T 0930
T 0347
T 0348
T 0352
T 1344
T 0403
T 0396
T 1281
T 0318
T 0163
T 0132
T 0832
T 0077
T 0076
T 0357
T 0811
T 0205
T 0216
T 1342
T 1888
T 0219
T 2002
T 1679
T 0288

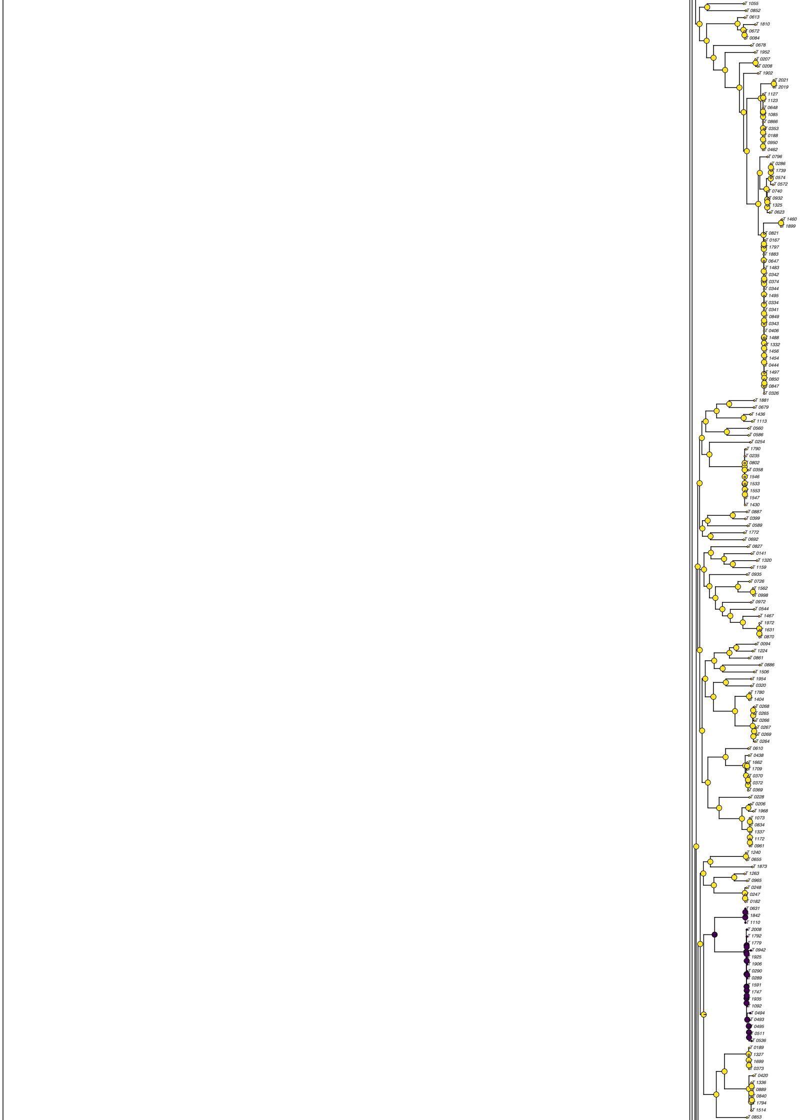

T 1055
T 0852
T 0613
T 1810
0672
T 0084
T 0678
T 1952
T 0207
T 0208
T 1902
T 2021
2019
T 1127
T 1123
T 0648
1085
T 0866
T 0353
0188
0950
0462
T 0796
T 0286
1739
0574
T 0572
T 0740
T 0932
1325
T 0623
T 1460
1899
T 0821
T 0167
1797
T 1883
0647
T 1483
T 0342
0374
T 0344
1495
T 0334
T 0341
T 0849
0343
T 0406
1488
T 1332
1456
1454
0444
T 1497
0850
0847
T 0326
T 1881
T 0679
T 1436
T 1113
T 0560
T 0586
T 0254
T 1790
T 0235
0802
T 0358
1546
1533
1553
1547
T 1430
T 0887
T 0399
T 0589
T 1772
T 0692
T 0827
T 0141
T 1320
T 1159
T 0935
T 0726
T 1562
T 0998
T 0972
T 0544
T 1467
T 1972
1631
T 0870
T 0094
T 1224
T 0861
T 0886
T 1506
T 1954
T 0320
T 1780
1404
T 0268
0265
T 0266
T 0267
T 0269
T 0264
T 0610
T 0438
T 1662
1709
T 0370
0372
T 0369
T 0228
T 0206
T 1968
T 1073
0834
1337
1172
0961
T 1240
T 0655
T 1873
T 1263
T 0965
T 0248
0247
T 0182
T 0631
1842
T 1110
T 2008
T 1792
T 1779
T 0942
T 1925
T 1906
T 0290
T 0289
T 1591
1747
T 1935
T 1092
T 0494
T 0493
0495
0511
T 0536
T 0189
1327
1699
T 0373
T 0420
T 1336
0889
T 0840
1794
T 1514
T 0853

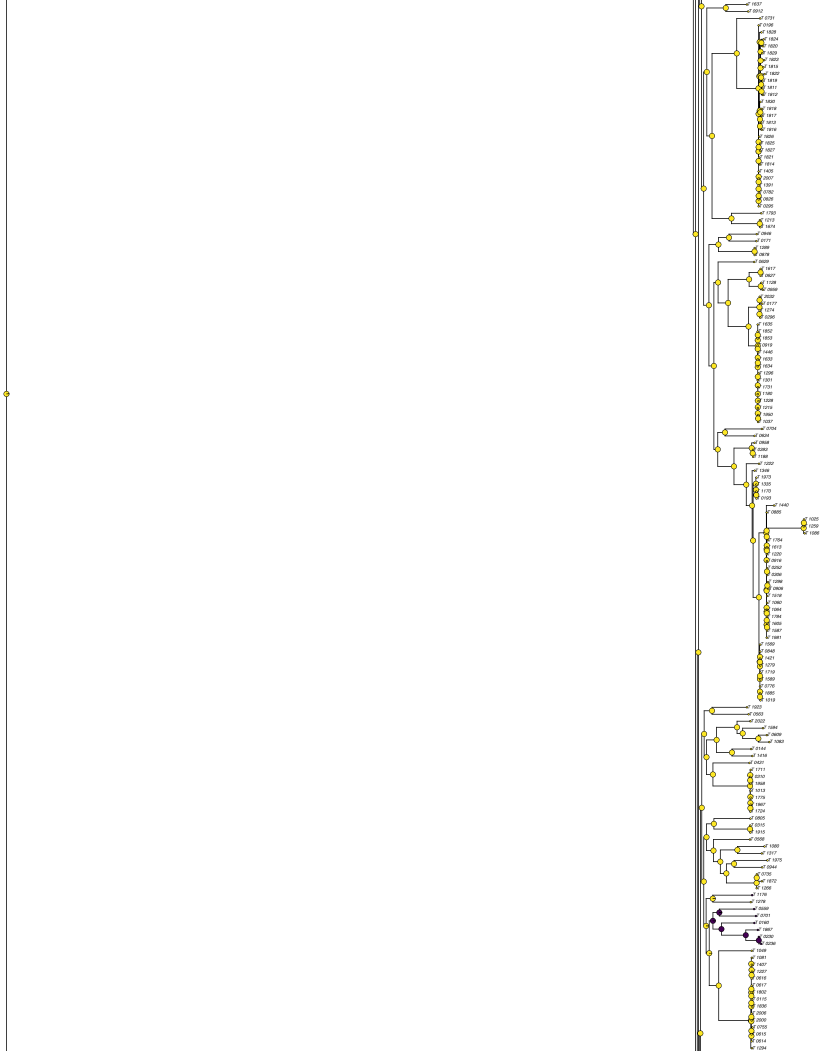
T 1637
T 0912
T 0731
T 0196
T 1828
T 1824
T 1820
T 1829
T 1823
T 1815
T 1822
T 1819
T 1811
T 1812
T 1830
T 1818
T 1817
T 1813
T 1816
T 1826
T 1825
T 1827
T 1821
T 1814
T 1405
T 2007
T 1391
T 0782
T 0826
T 0295
T 1793
T 1213
T 1674
T 0946
T 0171
T 1289
T 0878
T 0629
T 1617
T 0627
T 1128
T 0959
T 2032
T 0177
T 1274
T 0296
T 1635
T 1852
T 1853
T 0919
T 1446
T 1633
T 1634
T 1296
T 1301
T 1731
T 1180
T 1228
T 1215
T 1950
T 1037
T 0704
T 0634
T 0958
T 0393
T 1188
T 1222
T 1346
T 1973
T 1335
T 1170
T 0193
T 1440
T 0885
T 1025
T 1259
T 1086
T 1764
T 1613
T 1220
T 0916
T 0252
T 0306
T 1298
T 0906
T 1518
T 1060
T 1064
T 1784
T 1605
T 1587
T 1981
T 1569
T 0848
T 1421
T 1279
T 1719
T 1589
T 0776
T 1885
T 1019
T 1923
T 0563
T 2022
T 1594
T 0609
T 1083
T 0144
T 1416
T 0431
T 1711
T 0310
T 1958
T 1013
T 1775
T 1967
T 1724
T 0805
T 0315
T 1915
T 0568
T 1080
T 1317
T 1975
T 0944
T 0735
T 1872
T 1266
T 1176
T 1278
T 0559
T 0701
T 0160
T 1867
T 0230
T 0236
T 1049
T 1081
T 1407
T 1227
T 0616
T 0617
T 1802
T 0115
T 1836
T 2006
T 2000
T 0755
T 0615
T 0614
T 1294

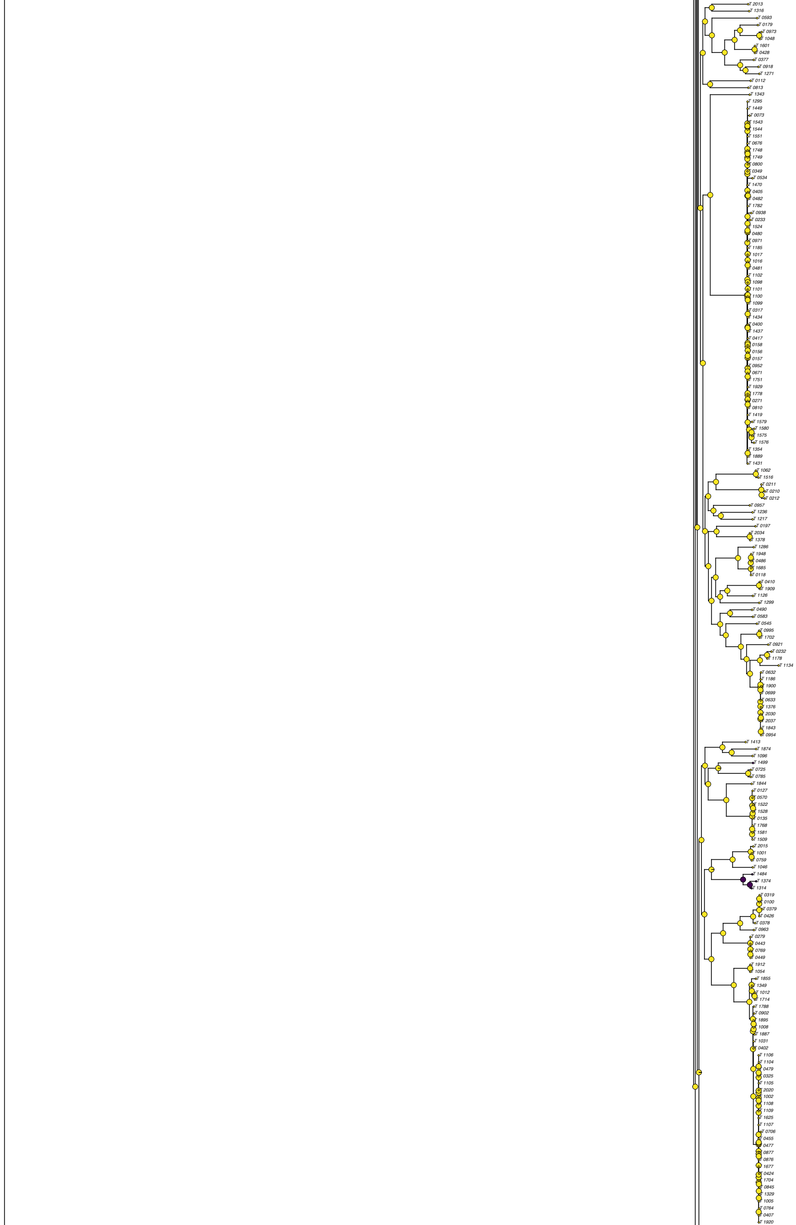
T 2013
T 1316
T 0593
T 0179
T 0973
T 1048
T 1601
T 0428
T 0377
T 0918
T 1271
T 0112
T 0813
T 1343
T 1295
T 1449
T 0073
T 1543
T 1544
T 1551
T 0676
T 1748
T 1749
T 0800
T 0349
T 0534
T 1470
T 0405
T 0482
T 1782
T 0938
T 0233
T 1524
T 0480
T 0971
T 1185
T 1017
T 1016
T 0481
T 1102
T 1098
T 1101
T 1100
T 1099
T 0317
T 1434
T 0400
T 1437
T 0417
T 0158
T 0156
T 0157
T 0952
T 0671
T 1751
T 1929
T 1778
T 0271
T 0810
T 1419
T 1579
T 1580
T 1575
T 1576
T 1354
T 1889
T 1431
T 1062
T 1516
T 0211
T 0210
T 0212
T 0957
T 1236
T 1217
T 0197
T 2034
T 1378
T 1286
T 1948
T 0486
T 1685
T 0118
T 0410
T 1909
T 1126
T 1299
T 0490
T 0583
T 0545
T 0995
T 1702
T 0921
T 0232
T 1178
T 1134
T 0632
T 1186
T 1900
T 0699
T 0633
T 1376
T 2030
T 2037
T 1843
T 0954
T 1413
T 1874
T 1096
T 1499
T 0725
T 0785
T 1844
T 0127
T 0570
T 1522
T 1528
T 0135
T 1768
T 1581
T 1509
T 2015
T 1001
T 0759
T 1046
T 1484
T 1374
T 1314
T 0319
T 0100
T 0379
T 0426
T 0378
T 0963
T 0279
T 0443
T 0769
T 0449
T 1912
T 1054
T 1855
T 1349
T 1012
T 1714
T 1788
T 0902
T 1895
T 1008
T 1887
T 1031
T 0402
T 1106
T 1104
T 0479
T 0325
T 1105
T 2020
T 1002
T 1108
T 1109
T 1625
T 1107
T 0706
T 0455
T 0477
T 0877
T 0876
T 1677
T 0424
T 1704
T 0845
T 1329
T 1005
T 0764
T 0407
T 1920

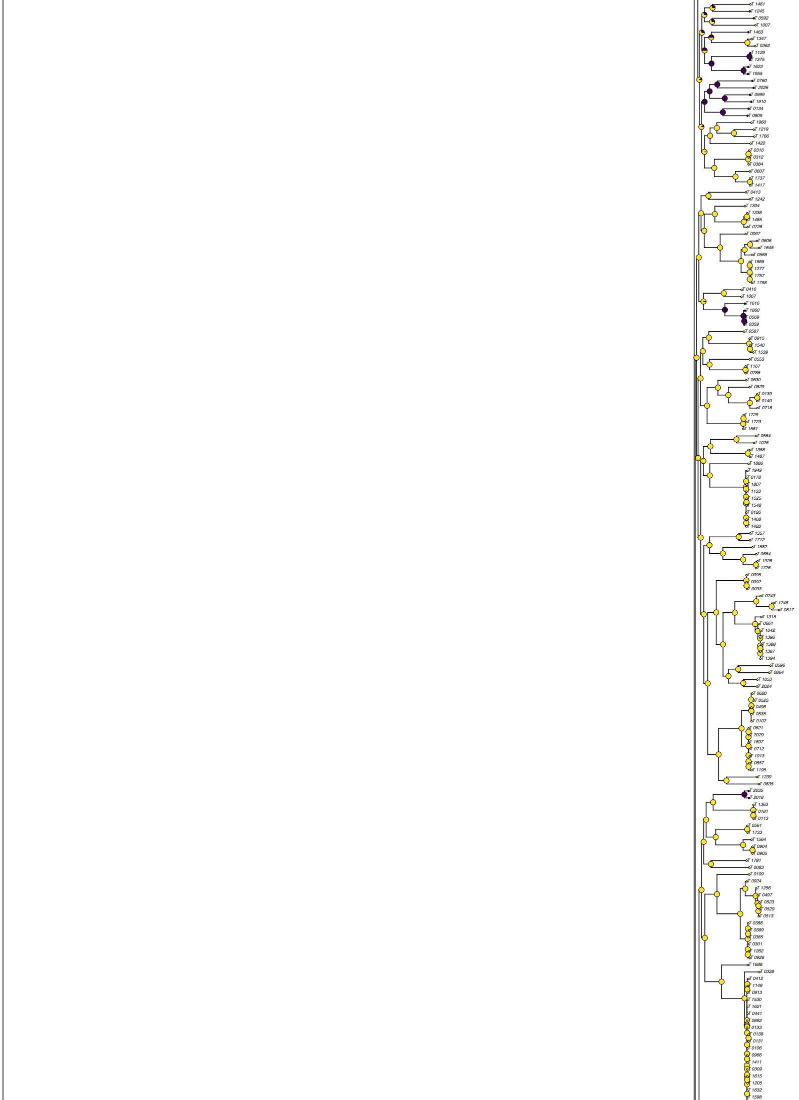
T 1461
T 1245
T 0592
T 1007
T 1463
T 1347
T 0362
T 1129
T 1375
T 1623
T 1955
T 0760
T 2026
T 0999
T 1910
T 0134
T 0809
T 1960
T 1219
T 1766
T 1420
T 0316
T 0312
T 0384
T 0607
T 1737
T 1417
T 0413
T 1242
T 1304
T 1338
T 1485
T 0728
T 0097
T 0606
T 1645
T 0565
T 1865
T 1277
T 1757
T 1758
T 0416
T 1067
T 1616
T 1860
T 0569
T 0359
T 0587
T 0915
T 1540
T 1539
T 0553
T 1167
T 0786
T 0630
T 0829
T 0139
T 0140
T 0718
T 1729
T 1723
T 1561
T 0564
T 1028
T 1358
T 1487
T 1886
T 1949
T 0178
T 1807
T 1133
T 1525
T 1548
T 0126
T 1408
T 1428
T 1357
T 1712
T 1582
T 0654
T 1926
T 1726
T 0095
T 0092
T 0093
T 0743
T 1246
T 0817
T 1315
T 0661
T 1042
T 1396
T 1388
T 1387
T 1394
T 0596
T 0864
T 1053
T 2024
T 0620
T 0525
T 0496
T 0535
T 0102
T 0621
T 2029
T 1897
T 0712
T 1913
T 0657
T 1195
T 1239
T 0835
T 2035
T 2018
T 1363
T 0181
T 0113
T 0561
T 1733
T 1564
T 0904
T 0905
T 1781
T 0083
T 0109
T 0924
T 1256
T 0497
T 0523
T 0529
T 0513
T 0388
T 0389
T 0385
T 0301
T 1262
T 0926
T 1688
T 0328
T 0412
T 1149
T 0913
T 1530
T 1621
T 0441
T 0892
T 0133
T 0138
T 0131
T 0106
T 0966
T 1411
T 0309
T 1615
T 1205
T 1632
T 1598

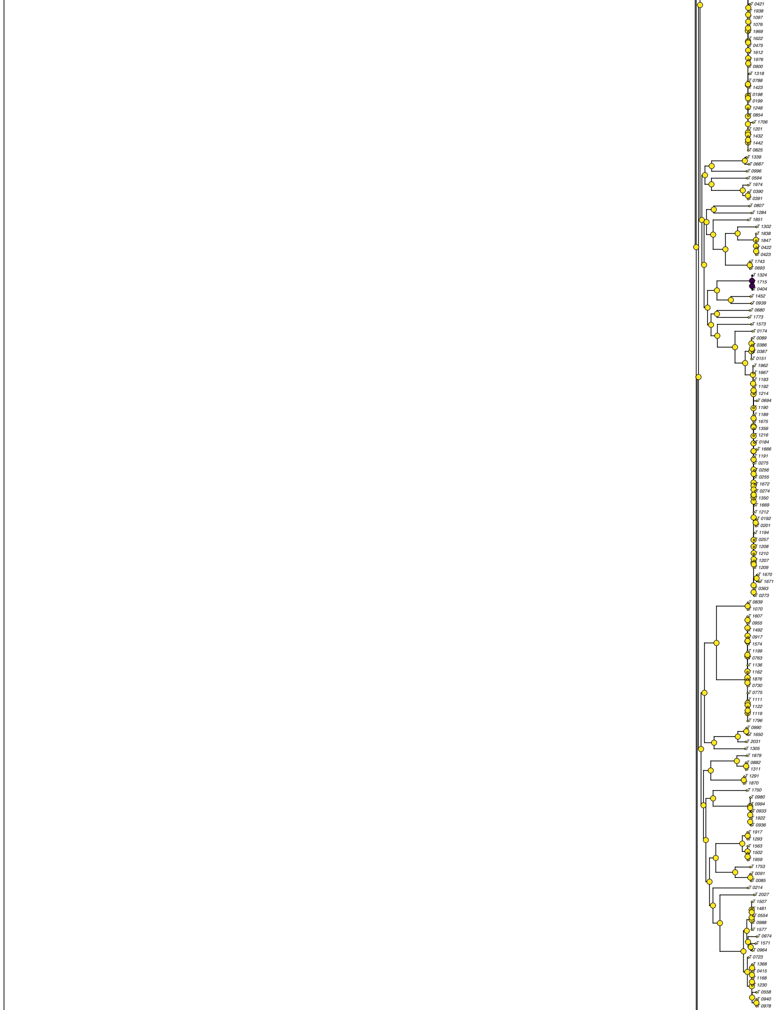
T 0421
T 1938
T 1097
T 1076
T 1969
T 1622
T 0475
T 1612
T 1976
T 0900
T 1318
T 0788
T 1423
T 0198
T 0199
T 1248
T 0854
T 1706
T 1201
T 1432
T 1442
T 0825
T 1339
T 0687
T 0996
T 0594
T 1974
T 0390
T 0391
T 0807
T 1284
T 1851
T 1302
T 1838
T 1847
T 0422
T 0423
T 1743
T 0693
T 1324
T 1715
T 0404
T 1452
T 0939
T 0680
T 1773
T 1573
T 0174
T 0089
T 0386
T 0387
T 0151
T 1962
T 1667
T 1193
T 1192
T 1214
T 0694
T 1190
T 1189
T 1675
T 1356
T 1216
T 0184
T 1666
T 1191
T 0275
T 0256
T 0255
T 1672
T 0274
T 1350
T 1669
T 1212
T 0192
T 0201
T 1194
T 0257
T 1208
T 1210
T 1207
T 1209
T 1670
T 1671
T 0363
T 0273
T 0839
T 1070
T 1607
T 0955
T 1492
T 0917
T 1574
T 1199
T 0763
T 1136
T 1162
T 1876
T 0730
T 0775
T 1111
T 1122
T 1119
T 1796
T 0990
T 1650
T 2031
T 1305
T 1879
T 0882
T 1311
T 1291
T 1870
T 1750
T 0980
T 0994
T 0933
T 1922
T 0936
T 1917
T 1293
T 1563
T 1502
T 1959
T 1753
T 0091
T 0085
T 0214
T 2027
T 1507
T 1481
T 0554
T 0988
T 1577
T 0974
T 1571
T 0964
T 0723
T 1368
T 0415
T 1168
T 1230
T 0558
T 0940
T 0978

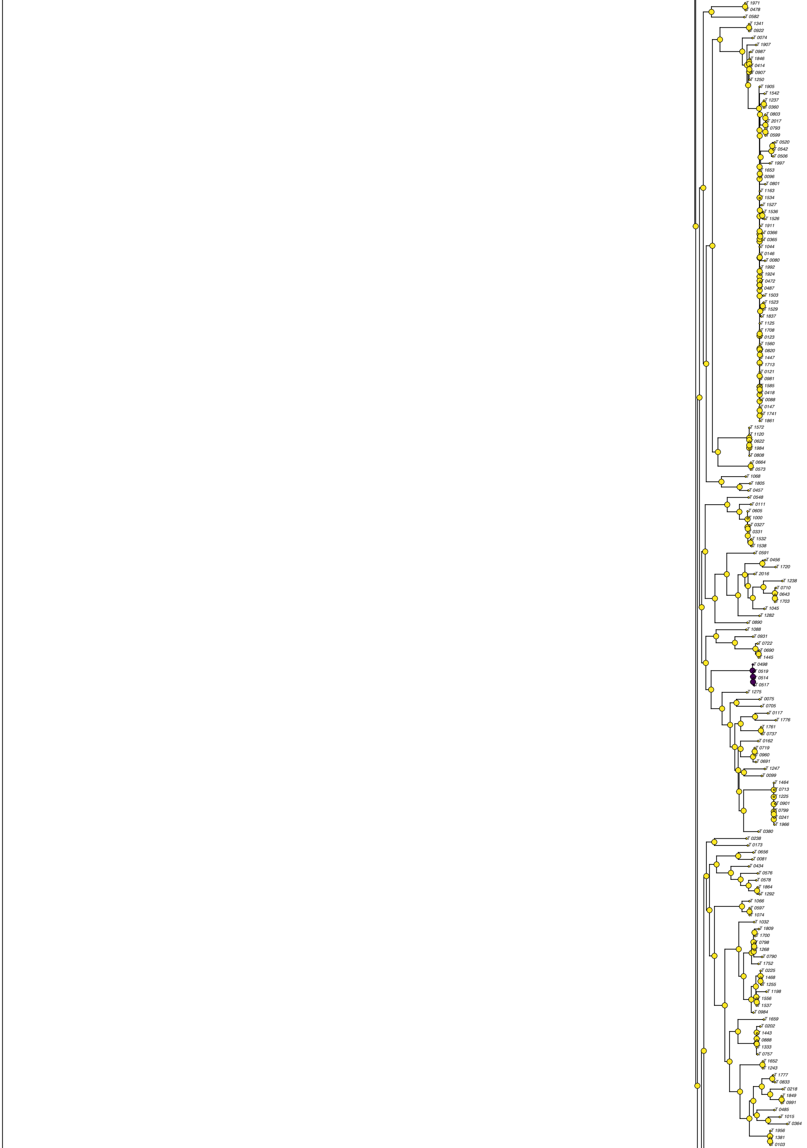

T 1971
T 0478
T 0582
T 1341
T 0922
T 0074
T 1907
T 0987
T 1846
T 0414
T 0907
T 1250
T 1905
T 1542
T 1237
T 0360
T 0803
T 2017
T 0793
T 0599
T 0520
T 0542
T 0506
T 1997
T 1653
T 0096
T 0801
T 1163
T 1534
T 1527
T 1536
T 1526
T 1911
T 0366
T 0365
T 1044
T 0146
T 0080
T 1992
T 1924
T 0472
T 0487
T 1503
T 1523
T 1529
T 1837
T 1125
T 1708
T 0123
T 1560
T 0820
T 1447
T 1713
T 0121
T 0981
T 1585
T 0418
T 0088
T 0147
T 1741
T 1861
T 1572
T 1120
T 0622
T 1984
T 0808
T 0664
T 0573
T 1068
T 1805
T 0457
T 0548
T 0111
T 0605
T 1000
T 0327
T 0331
T 1532
T 1538
T 0591
T 0456
T 1720
T 2016
T 1238
T 0710
T 0643
T 1703
T 1045
T 1282
T 0890
T 1088
T 0931
T 0722
T 0690
T 1445
T 0498
T 0519
T 0514
T 0517
T 1275
T 0075
T 0705
T 0117
T 1776
T 1761
T 0737
T 0162
T 0719
T 0960
T 0691
T 1247
T 0099
T 1464
T 0713
T 1225
T 0901
T 0799
T 0241
T 1966
T 0380
T 0238
T 0173
T 0656
T 0081
T 0434
T 0576
T 0578
T 1864
T 1292
T 1066
T 0597
T 1074
T 1032
T 1809
T 1700
T 0798
T 1268
T 0790
T 1752
T 0225
T 1468
T 1255
T 1198
T 1556
T 1537
T 0984
T 1659
T 0202
T 1443
T 0888
T 1333
T 0757
T 1652
T 1243
T 1777
T 0833
T 0218
T 1849
T 0991
T 0485
T 1015
T 0364
T 1956
T 1381
T 0103

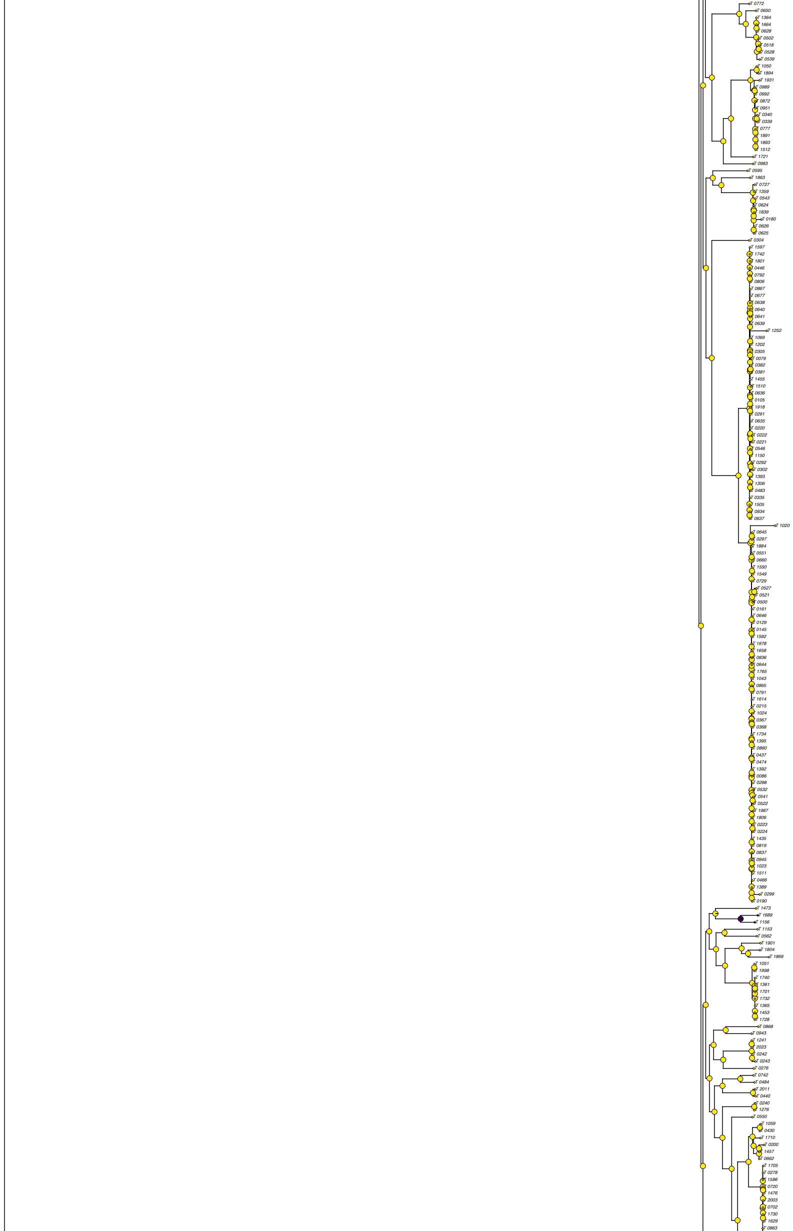
T 0772
T 0650
T 1364
T 1664
T 0628
T 0502
T 0518
T 0528
T 0539
T 1050
T 1894
T 1931
T 0989
T 0992
T 0872
T 0951
T 0340
T 0339
T 0777
T 1891
T 1893
T 1512
T 1721
T 0983
T 0595
T 1863
T 0727
T 1359
T 0543
T 0624
T 1839
T 0180
T 0626
T 0625
T 0304
T 1597
T 1742
T 1801
T 0446
T 0792
T 0806
T 0867
T 0677
T 0638
T 0640
T 0641
T 0639
T 1252
T 1069
T 1202
T 0305
T 0079
T 0382
T 0381
T 1455
T 1510
T 0636
T 0105
T 1918
T 0291
T 0635
T 0220
T 0222
T 0221
T 0546
T 1150
T 0292
T 0302
T 1393
T 1306
T 0483
T 0335
T 1505
T 0934
T 0637
T 1020
T 0645
T 0297
T 1884
T 0551
T 0660
T 1550
T 1549
T 0729
T 0527
T 0521
T 0500
T 0161
T 0646
T 0129
T 0145
T 1592
T 1978
T 1658
T 0836
T 0644
T 1765
T 1043
T 0865
T 0791
T 1614
T 0215
T 1024
T 0367
T 0368
T 1734
T 1395
T 0860
T 0437
T 0474
T 1392
T 0086
T 0298
T 0532
T 0541
T 0522
T 1987
T 1806
T 0223
T 0224
T 1435
T 0819
T 0837
T 0945
T 1023
T 1511
T 0466
T 1389
T 0299
T 0190
T 1473
T 1689
T 1156
T 1153
T 0562
T 1901
T 1804
T 1869
T 1051
T 1998
T 1740
T 1361
T 1701
T 1732
T 1365
T 1453
T 1728
T 0868
T 0943
T 1241
T 2023
T 0242
T 0243
T 0276
T 0742
T 0484
T 2011
T 0440
T 0240
T 1276
T 0550
T 1059
T 0430
T 1710
T 0200
T 1457
T 0662
T 1705
T 0278
T 1586
T 0720
T 1476
T 2003
T 0702
T 1730
T 1629
T 0863

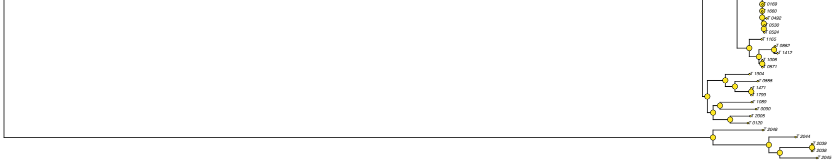

T 0169
T 1660
T 0492
T 0530
T 0524
T 1165
T 0862
T 1412
T 1006
T 0571
T 1904
T 0555
T 1471
T 1799
T 1089
T 0090
T 2005
T 0120
T 2048
T 2044
T 2039
T 2038
T 2045

Effector gene: tle2

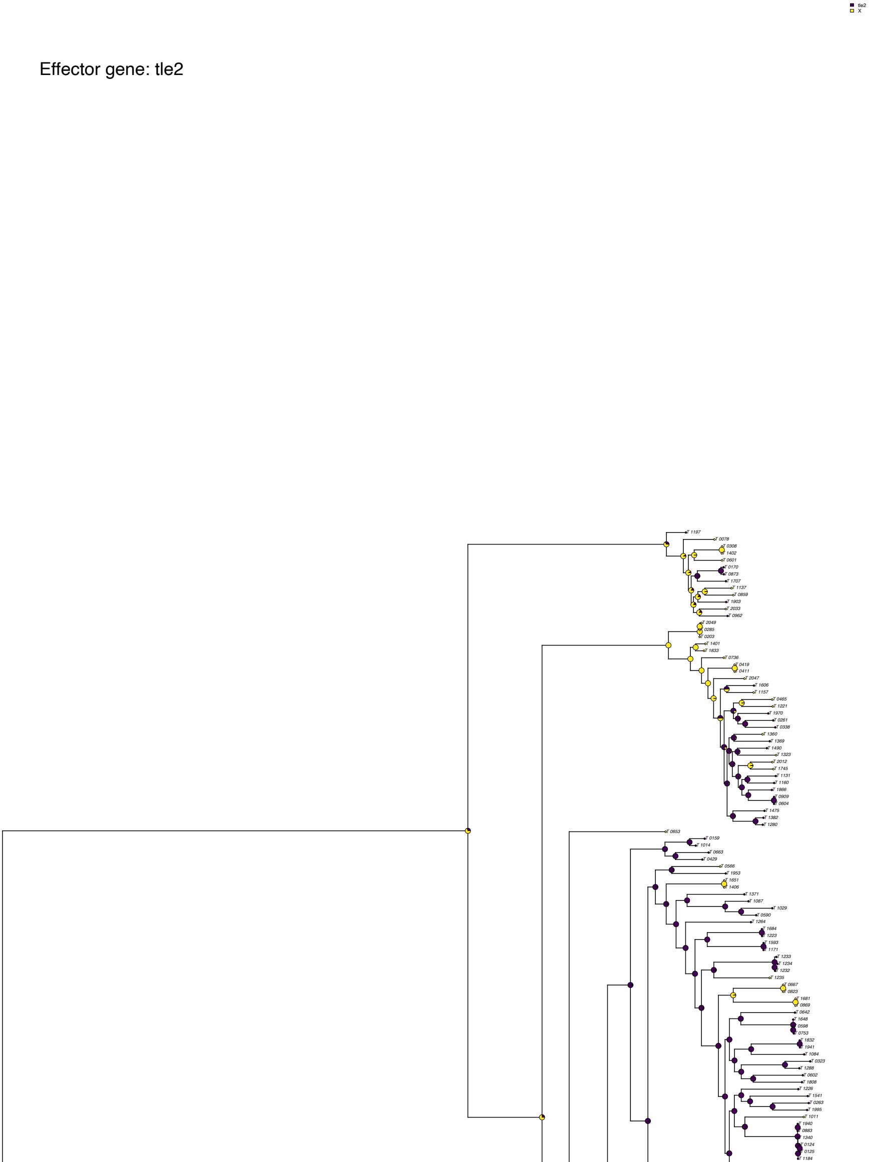

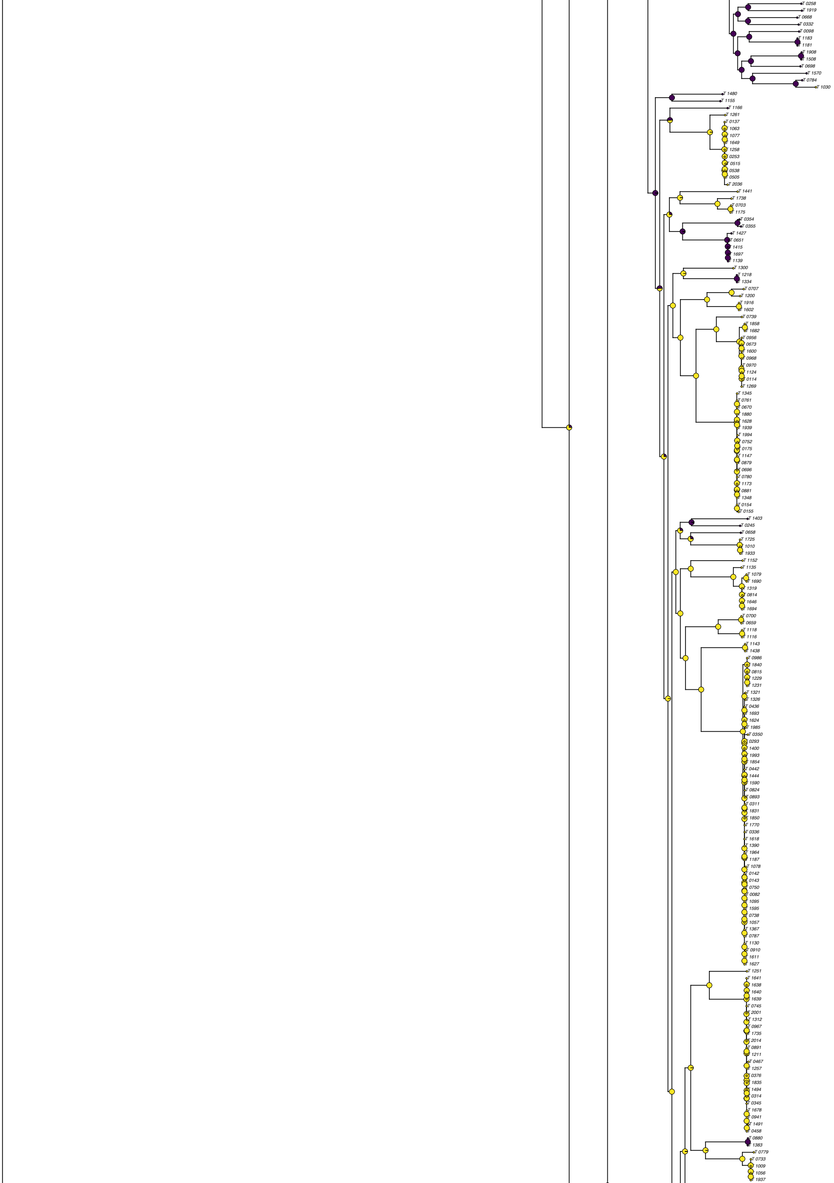
T 0258
T 1919
T 0668
T 0332
T 0098
T 1183
T 1181
T 1908
T 1508
T 0698
T 1570
T 0784
T 1030
T 1480
T 1155
T 1166
T 1261
T 0137
T 1063
T 1077
T 1649
T 1258
T 0253
T 0515
T 0538
T 0505
T 2036
T 1441
T 1738
T 0703
T 1175
T 0354
T 0355
T 1427
T 0651
T 1415
T 1697
T 1139
T 1300
T 1218
T 1334
T 0707
T 1200
T 1916
T 1602
T 0739
T 1858
T 1682
T 0956
T 0673
T 1600
T 0968
T 0970
T 1124
T 0114
T 1269
T 1345
T 0761
T 0670
T 1880
T 1628
T 1939
T 1994
T 0752
T 0175
T 1147
T 0879
T 0696
T 0780
T 1173
T 0881
T 1348
T 0154
T 0155
T 1403
T 0245
T 0658
T 1725
T 1010
T 1933
T 1152
T 1135
T 1079
T 1690
T 1319
T 0814
T 1646
T 1694
T 0700
T 0659
T 1118
T 1116
T 1143
T 1438
T 0986
T 1840
T 0815
T 1229
T 1231
T 1321
T 1326
T 0436
T 1693
T 1624
T 1985
T 0350
T 0293
T 1400
T 1993
T 1854
T 0442
T 1444
T 1590
T 0824
T 0893
T 0311
T 1831
T 1850
T 1770
T 0336
T 1618
T 1390
T 1964
T 1187
T 1078
T 0142
T 0143
T 0750
T 0082
T 1095
T 1595
T 0738
T 1057
T 1367
T 0787
T 1130
T 0910
T 1611
T 1627
T 1251
T 1641
T 1638
T 1640
T 1639
T 0745
T 2001
T 1312
T 0967
T 1735
T 2014
T 0891
T 1211
T 0467
T 1257
T 0376
T 1835
T 1494
T 0314
T 0345
T 1678
T 0941
T 1491
T 0458
T 0880
T 1383
T 0779
T 0733
T 1009
T 1056
T 1937

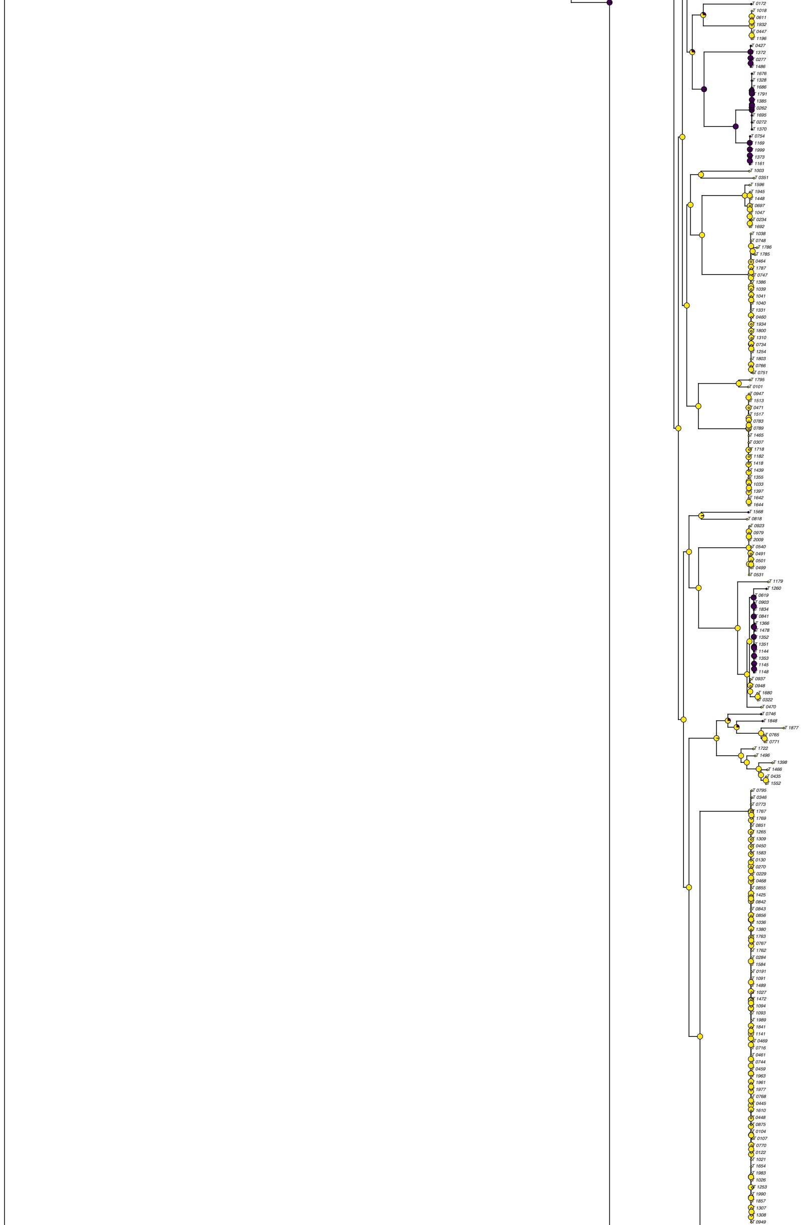
T 0172
T 1018
T 0611
T 1932
T 0447
T 1196
T 0427
T 1372
T 0277
T 1486
T 1676
T 1328
T 1686
T 1791
T 1385
T 0262
T 1695
T 0272
T 1370
T 0754
T 1169
T 1999
T 1373
T 1161
T 1003
T 0351
T 1596
T 1945
T 1448
T 0697
T 1047
T 0234
T 1692
T 1038
T 0748
T 1786
T 1785
T 0464
T 1787
T 0747
T 1386
T 1039
T 1041
T 1040
T 1331
T 0460
T 1934
T 1800
T 1310
T 0734
T 1254
T 1803
T 0766
T 0751
T 1795
T 0101
T 0947
T 1513
T 0471
T 1517
T 0783
T 0789
T 1465
T 0307
T 1718
T 1182
T 1418
T 1439
T 1355
T 1033
T 1397
T 1642
T 1644
T 1568
T 0818
T 0923
T 0979
T 2009
T 0540
T 0491
T 0501
T 0499
T 0531
T 1179
T 1260
T 0619
T 0903
T 1834
T 0841
T 1366
T 1478
T 1352
T 1351
T 1144
T 1353
T 1145
T 1148
T 0937
T 0948
T 1680
T 0322
T 0470
T 0746
T 1848
T 1877
T 0765
T 0771
T 1722
T 1496
T 1398
T 1466
T 0435
T 1552
T 0795
T 0346
T 0773
T 1767
T 1769
T 0851
T 1265
T 1309
T 0450
T 1583
T 0130
T 0270
T 0229
T 0468
T 0855
T 1425
T 0842
T 0843
T 0856
T 1036
T 1380
T 1763
T 0767
T 1762
T 0284
T 1584
T 0191
T 1091
T 1489
T 1027
T 1472
T 1094
T 1093
T 1989
T 1841
T 1141
T 0469
T 0716
T 0461
T 0744
T 0459
T 1963
T 1961
T 1977
T 0768
T 0445
T 1610
T 0448
T 0875
T 0104
T 0107
T 0770
T 0122
T 1021
T 1654
T 1983
T 1026
T 1253
T 1990
T 1857
T 1307
T 1308
T 0949

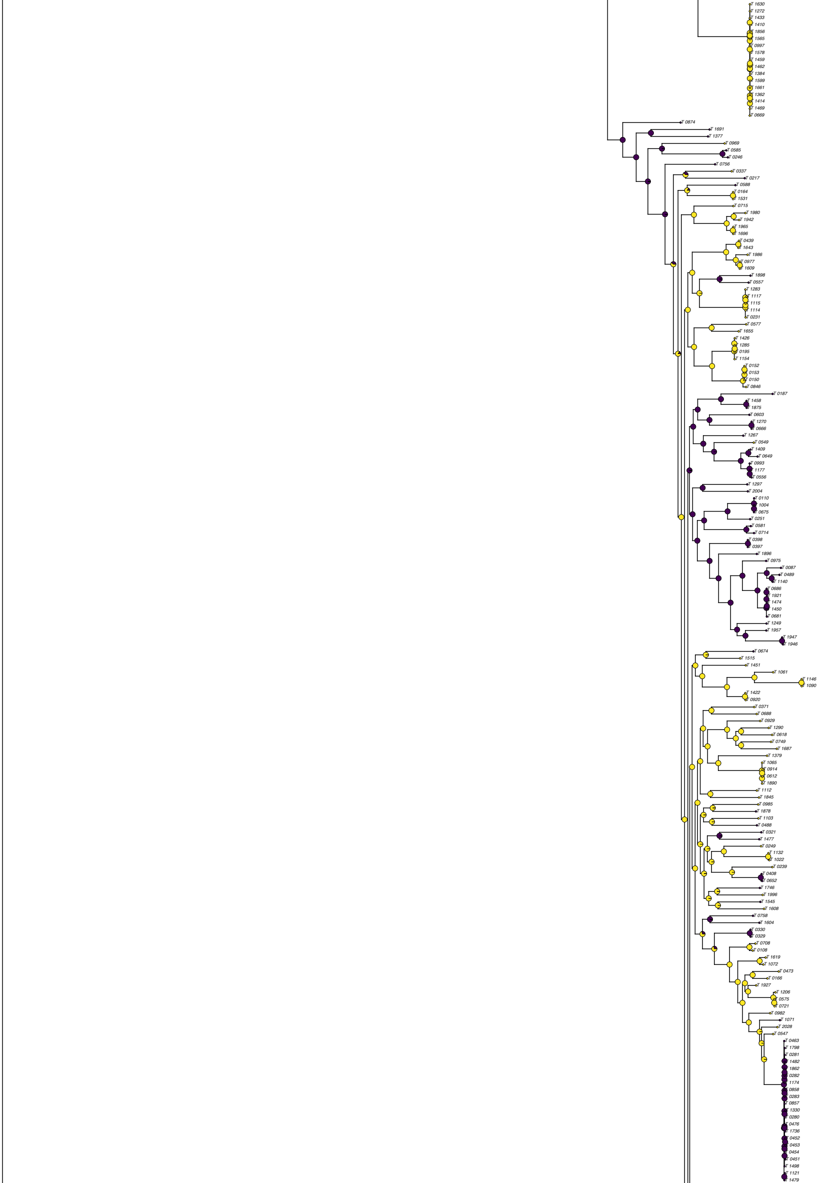
T 1630
T 1272
T 1433
T 1410
T 1856
T 1565
T 0997
T 1578
T 1459
T 1462
T 1384
T 1599
T 1661
T 1362
T 1414
T 1469
T 0669
T 0874
T 1691
T 1377
T 0969
T 0585
T 0246
T 0756
T 0337
T 0217
T 0588
T 0164
T 1531
T 0715
T 1980
T 1942
T 1965
T 1696
T 0439
T 1643
T 1986
T 0977
T 1609
T 1898
T 0557
T 1283
T 1117
T 1115
T 1114
T 0231
T 0577
T 1655
T 1426
T 1285
T 0195
T 1154
T 0152
T 0153
T 0150
T 0846
T 0187
T 1458
T 1875
T 0603
T 1270
T 0666
T 1267
T 0549
T 1409
T 0649
T 0993
T 1177
T 0556
T 1297
T 2004
T 0110
T 1004
T 0675
T 0251
T 0581
T 0714
T 0398
T 0397
T 1896
T 0975
T 0087
T 0489
T 1140
T 0686
T 1921
T 1474
T 1450
T 0681
T 1249
T 1957
T 1947
T 1946
T 0674
T 1515
T 1451
T 1061
T 1146
T 1090
T 1422
T 0920
T 0371
T 0688
T 0929
T 1290
T 0618
T 0749
T 1687
T 1379
T 1065
T 0914
T 0612
T 1890
T 1112
T 1845
T 0985
T 1878
T 1103
T 0488
T 0321
T 1477
T 0249
T 1132
T 1022
T 0239
T 0408
T 0652
T 1746
T 1996
T 1545
T 1608
T 0758
T 1604
T 0330
T 0329
T 0708
T 0108
T 1619
T 1072
T 0473
T 0166
T 1927
T 1206
T 0575
T 0721
T 0982
T 1071
T 2028
T 0547
T 0463
T 1798
T 0281
T 1482
T 1862
T 0282
T 1174
T 0858
T 0283
T 0857
T 1330
T 0280
T 0476
T 1736
T 0452
T 0453
T 0454
T 0451
T 1498
T 1121
T 1479

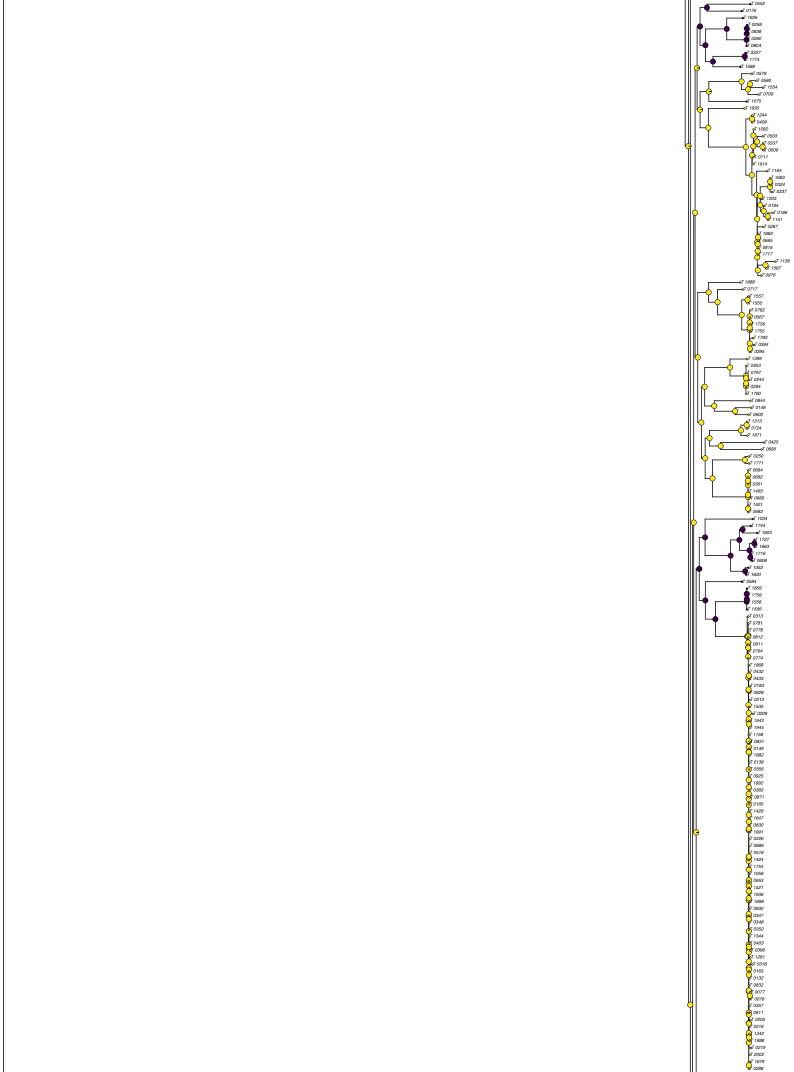

T 0552
T 0176
T 1626
T 0259
0838
0260
T 0804
T 0227
T 1774
T 1588
T 0579
T 0580
T 1554
T 0709
T 1075
T 1930
T 1244
T 0409
T 1082
T 0503
T 0537
T 0509
T 0711
T 1914
T 1164
T 1683
0324
T 0237
T 1203
T 0194
T 0186
1151
T 0287
T 1882
0665
T 0816
1717
T 1138
T 1567
T 0976
T 1988
T 0717
T 1557
1555
T 0762
0567
1756
1755
T 1783
T 0394
0395
T 1399
T 0303
T 0797
T 0244
0294
T 1760
T 0844
T 0148
T 0600
T 1313
T 0724
T 1871
T 0425
T 0695
T 0250
T 1771
T 0684
0682
0361
T 1493
T 0685
T 1501
0683
T 1034
T 1744
T 1603
T 1727
T 1663
T 1716
T 0608
T 1052
T 1620
T 0584
T 1859
1759
1558
T 1566
T 0313
T 0781
T 0778
0812
0911
T 0794
0774
T 1868
T 0432
0433
T 0183
T 0828
T 0213
1535
T 0209
1943
1944
T 1158
0831
0149
1982
T 0136
0356
T 0925
1892
0383
T 0871
0165
T 1429
1647
0830
T 1991
T 0226
T 0689
T 0516
1424
T 1754
T 1058
0953
1521
1936
1698
T 0930
0347
0348
T 0352
T 1344
T 0403
T 0396
T 1281
T 0318
0163
T 0132
T 0832
T 0077
T 0076
T 0357
0811
T 0205
T 0216
1342
1888
T 0219
T 2002
T 1679
T 0288

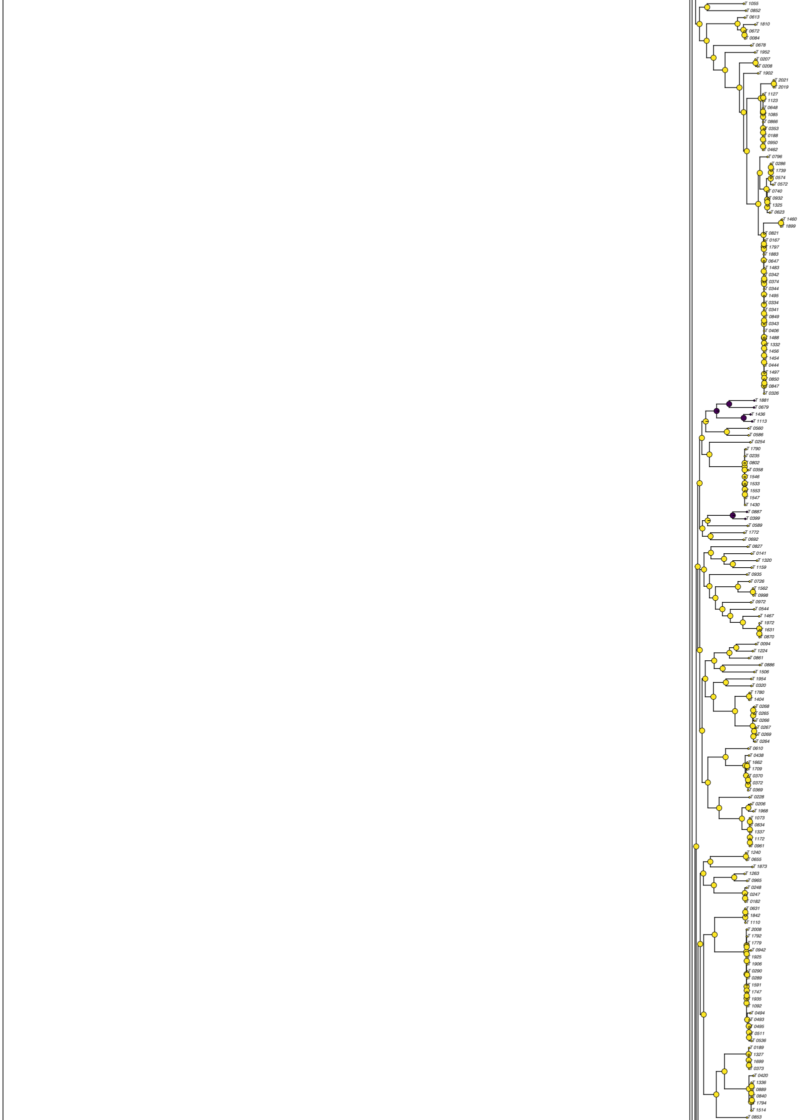
T 1055
T 0852
T 0613
T 1810
T 0672
T 0084
T 0678
T 1952
T 0207
T 0208
T 1902
T 2021
T 2019
T 1127
T 1123
T 0648
T 1085
T 0866
T 0353
T 0188
T 0950
T 0462
T 0796
T 0286
T 1739
T 0574
T 0572
T 0740
T 0932
T 1325
T 0623
T 1460
T 1899
T 0821
T 0167
T 1797
T 1883
T 0647
T 1483
T 0342
T 0374
T 0344
T 1495
T 0334
T 0341
T 0849
T 0343
T 0406
T 1488
T 1332
T 1456
T 1454
T 0444
T 1497
T 0850
T 0847
T 0326
T 1881
T 0679
T 1436
T 1113
T 0560
T 0586
T 0254
T 1790
T 0235
T 0802
T 0358
T 1546
T 1533
T 1553
T 1547
T 1430
T 0887
T 0399
T 0589
T 1772
T 0692
T 0827
T 0141
T 1320
T 1159
T 0935
T 0726
T 1562
T 0998
T 0972
T 0544
T 1467
T 1972
T 1631
T 0870
T 0094
T 1224
T 0861
T 0886
T 1506
T 1954
T 0320
T 1780
T 1404
T 0268
T 0265
T 0266
T 0267
T 0269
T 0264
T 0610
T 0438
T 1662
T 1709
T 0370
T 0372
T 0369
T 0228
T 0206
T 1968
T 1073
T 0834
T 1337
T 1172
T 0961
T 1240
T 0655
T 1873
T 1263
T 0965
T 0248
T 0247
T 0182
T 0631
T 1842
T 1110
T 2008
T 1792
T 1779
T 0942
T 1925
T 1906
T 0290
T 0289
T 1591
T 1747
T 1935
T 1092
T 0494
T 0493
T 0495
T 0511
T 0536
T 0189
T 1327
T 1699
T 0373
T 0420
T 1336
T 0889
T 0840
T 1794
T 1514
T 0853

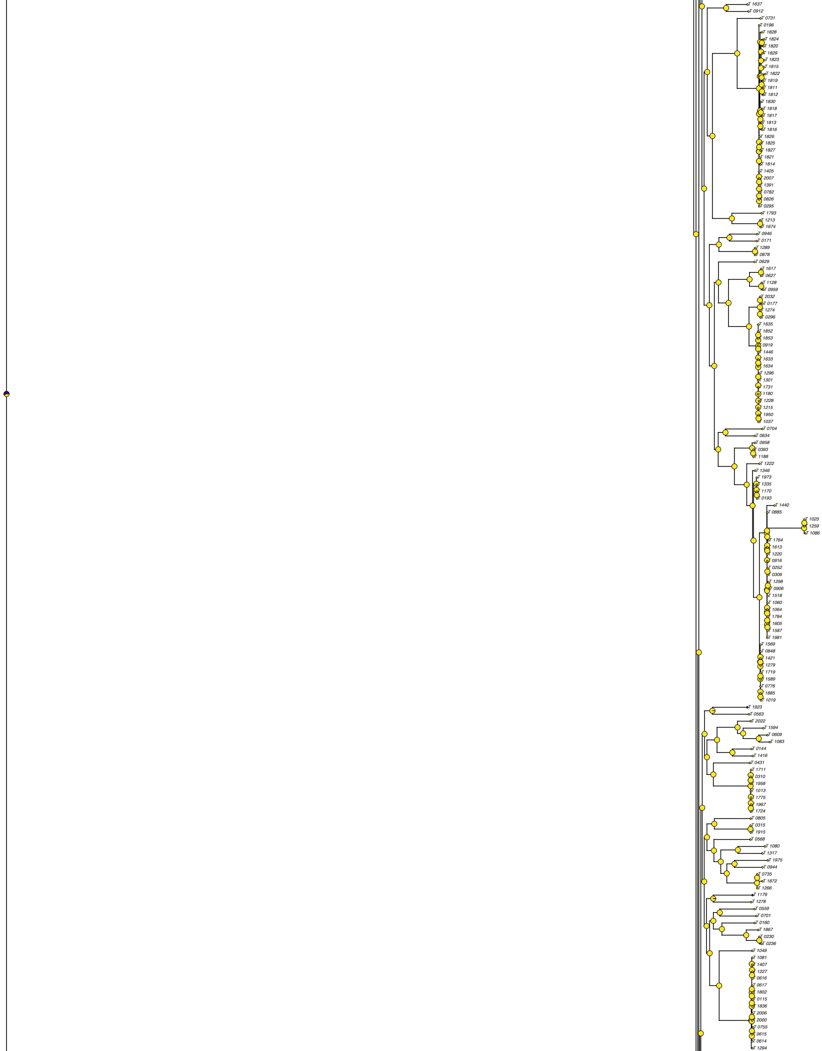
T 1637
T 0912
T 0731
T 0196
T 1828
T 1824
T 1820
T 1829
T 1823
T 1815
T 1822
T 1819
T 1811
T 1812
T 1830
T 1818
T 1817
T 1813
T 1816
T 1826
T 1825
T 1827
T 1821
T 1814
T 1405
T 2007
T 1391
T 0782
T 0826
T 0295
T 1793
T 1213
T 1674
T 0946
T 0171
T 1289
T 0878
T 0629
T 1617
T 0627
T 1128
T 0959
T 2032
T 0177
T 1274
T 0296
T 1635
T 1852
T 1853
T 0919
T 1446
T 1633
T 1634
T 1296
T 1301
T 1731
T 1180
T 1228
T 1215
T 1950
T 1037
T 0704
T 0634
T 0958
T 0393
T 1188
T 1222
T 1346
T 1973
T 1335
T 1170
T 0193
T 1440
T 0885
T 1025
T 1259
T 1086
T 1764
T 1613
T 1220
T 0916
T 0252
T 0306
T 1298
T 0906
T 1518
T 1060
T 1064
T 1784
T 1605
T 1587
T 1981
T 1569
T 0848
T 1421
T 1279
T 1719
T 1589
T 0776
T 1885
T 1019
T 1923
T 0563
T 2022
T 1594
T 0609
T 1083
T 0144
T 1416
T 0431
T 1711
T 0310
T 1958
T 1013
T 1775
T 1967
T 1724
T 0805
T 0315
T 1915
T 0568
T 1080
T 1317
T 1975
T 0944
T 0735
T 1872
T 1266
T 1176
T 1278
T 0559
T 0701
T 0160
T 1867
T 0230
T 0236
T 1049
T 1081
T 1407
T 1227
T 0616
T 0617
T 1802
T 0115
T 1836
T 2006
T 2000
T 0755
T 0615
T 0614
T 1294

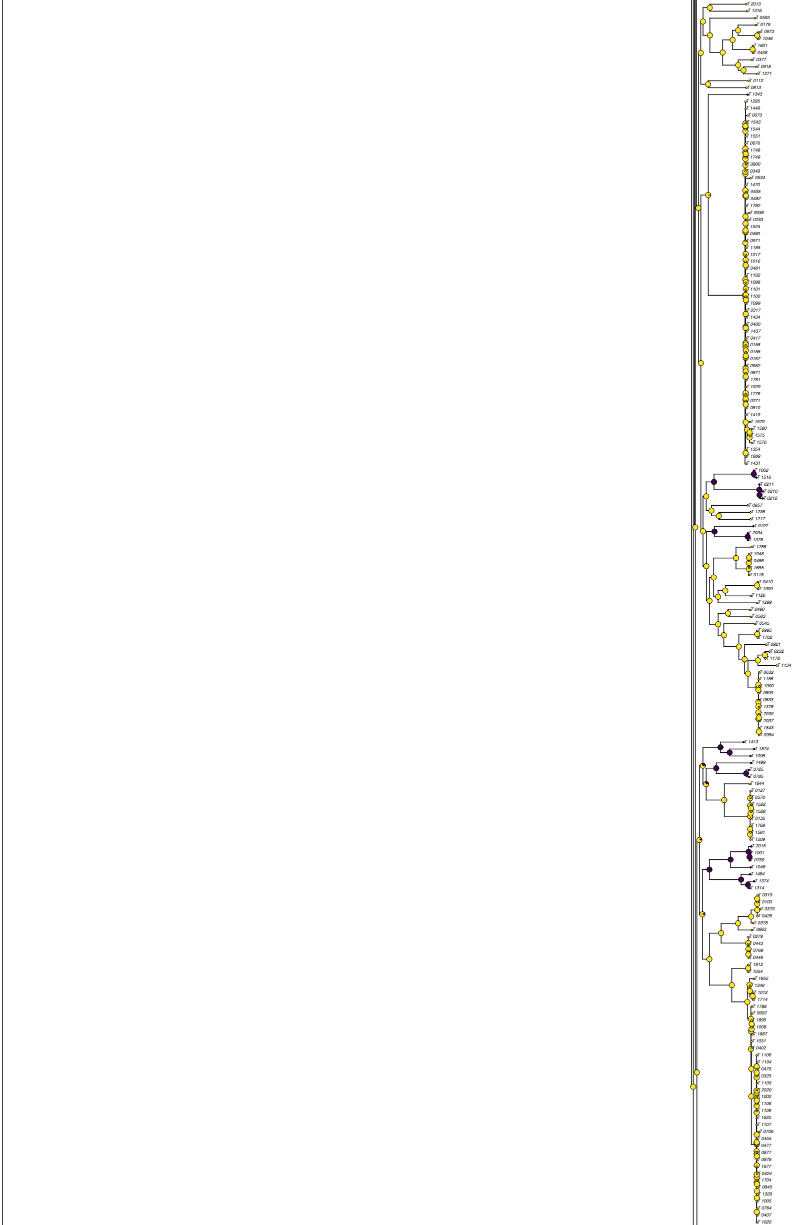

T 2013
T 1316
T 0593
T 0179
T 0973
T 1048
T 1601
T 0428
T 0377
T 0918
T 1271
T 0112
T 0813
T 1343
T 1295
T 1449
T 0073
T 1543
T 1544
T 1551
T 0676
T 1748
T 1749
T 0800
T 0349
T 0534
T 1470
T 0405
T 0482
T 1782
T 0938
T 0233
T 1524
T 0480
T 0971
T 1185
T 1017
T 1016
T 0481
T 1102
T 1098
T 1101
T 1100
T 1099
T 0317
T 1434
T 0400
T 1437
T 0417
T 0158
T 0156
T 0157
T 0952
T 0671
T 1751
T 1929
T 1778
T 0271
T 0810
T 1419
T 1579
T 1580
T 1575
T 1576
T 1354
T 1889
T 1431
T 1062
T 1516
T 0211
T 0210
T 0212
T 0957
T 1236
T 1217
T 0197
T 2034
T 1378
T 1286
T 1948
T 0486
T 1685
T 0118
T 0410
T 1909
T 1126
T 1299
T 0490
T 0583
T 0545
T 0995
T 1702
T 0921
T 0232
T 1178
T 1134
T 0632
T 1186
T 1900
T 0699
T 0633
T 1376
T 2030
T 2037
T 1843
T 0954
T 1413
T 1874
T 1096
T 1499
T 0725
T 0785
T 1844
T 0127
T 0570
T 1522
T 1528
T 0135
T 1768
T 1581
T 1509
T 2015
T 1001
T 0759
T 1046
T 1484
T 1374
T 1314
T 0319
T 0100
T 0379
T 0426
T 0378
T 0963
T 0279
T 0443
T 0769
T 0449
T 1912
T 1054
T 1855
T 1349
T 1012
T 1714
T 1788
T 0902
T 1895
T 1008
T 1887
T 1031
T 0402
T 1106
T 1104
T 0479
T 0325
T 1105
T 2020
T 1002
T 1108
T 1109
T 1625
T 1107
T 0706
T 0455
T 0477
T 0877
T 0876
T 1677
T 0424
T 1704
T 0845
T 1329
T 1005
T 0764
T 0407
T 1920

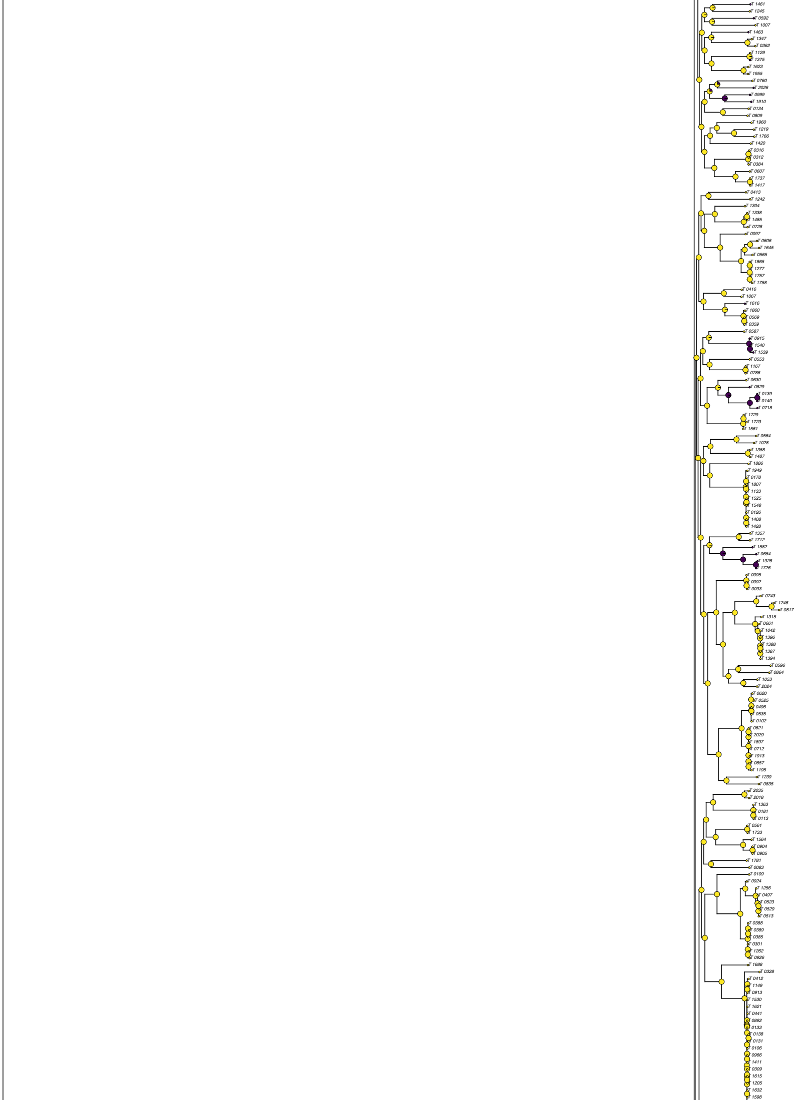

T 1461
T 1245
T 0592
T 1007
T 1463
T 1347
T 0362
T 1129
T 1375
T 1623
T 1955
T 0760
T 2026
T 0999
T 1910
T 0134
T 0809
T 1960
T 1219
T 1766
T 1420
T 0316
T 0312
T 0384
T 0607
T 1737
T 1417
T 0413
T 1242
T 1304
T 1338
T 1485
T 0728
T 0097
T 0606
T 1645
T 0565
T 1865
T 1277
T 1757
T 1758
T 0416
T 1067
T 1616
T 1860
T 0569
T 0359
T 0587
T 0915
T 1540
T 1539
T 0553
T 1167
T 0786
T 0630
T 0829
T 0139
T 0140
T 0718
T 1729
T 1723
T 1561
T 0564
T 1028
T 1358
T 1487
T 1886
T 1949
T 0178
T 1807
T 1133
T 1525
T 1548
T 0126
T 1408
T 1428
T 1357
T 1712
T 1582
T 0654
T 1926
T 1726
T 0095
T 0092
T 0093
T 0743
T 1246
T 0817
T 1315
T 0661
T 1042
T 1396
T 1388
T 1387
T 1394
T 0596
T 0864
T 1053
T 2024
T 0620
T 0525
T 0496
T 0535
T 0102
T 0621
T 2029
T 1897
T 0712
T 1913
T 0657
T 1195
T 1239
T 0835
T 2035
T 2018
T 1363
T 0181
T 0113
T 0561
T 1733
T 1564
T 0904
T 0905
T 1781
T 0083
T 0109
T 0924
T 1256
T 0497
T 0523
T 0529
T 0513
T 0388
T 0389
T 0385
T 0301
T 1262
T 0926
T 1688
T 0328
T 0412
T 1149
T 0913
T 1530
T 1621
T 0441
T 0892
T 0133
T 0138
T 0131
T 0106
T 0966
T 1411
T 0309
T 1615
T 1205
T 1632
T 1598

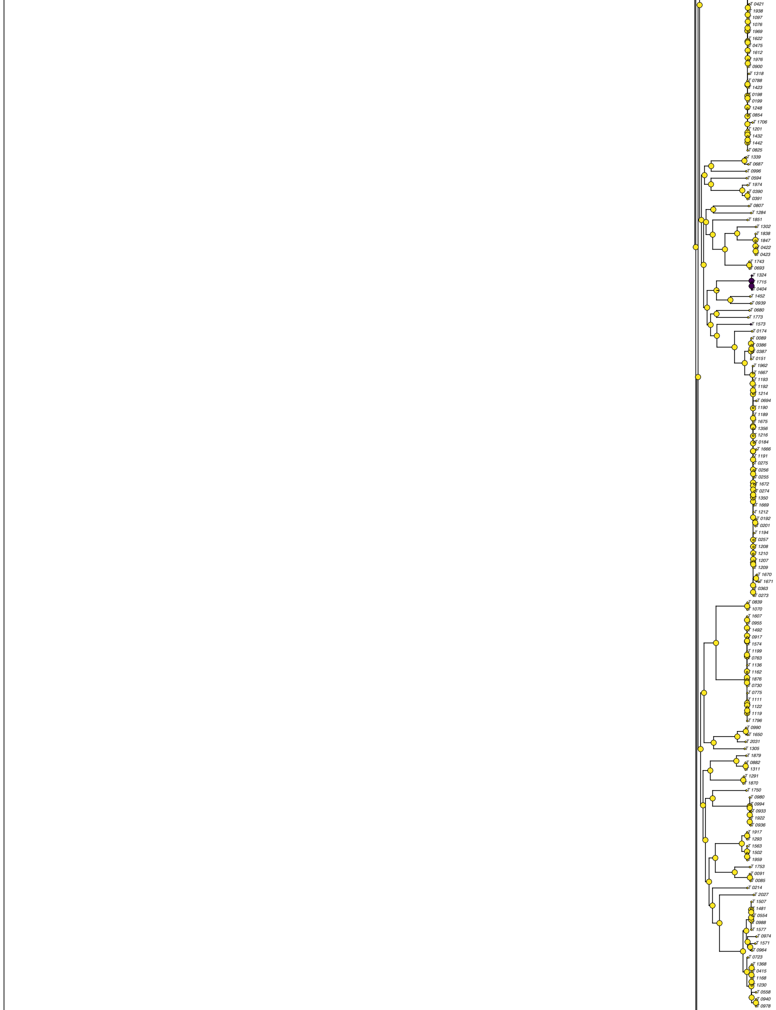
T 0421
T 1938
T 1097
T 1076
T 1969
T 1622
T 0475
T 1612
T 1976
T 0900
T 1318
T 0788
T 1423
T 0198
T 0199
T 1248
T 0854
T 1706
T 1201
T 1432
T 1442
T 0825
T 1339
T 0687
T 0996
T 0594
T 1974
T 0390
T 0391
T 0807
T 1284
T 1851
T 1302
T 1838
T 1847
T 0422
T 0423
T 1743
T 0693
T 1324
T 1715
T 0404
T 1452
T 0939
T 0680
T 1773
T 1573
T 0174
T 0089
T 0386
T 0387
T 0151
T 1962
T 1667
T 1193
T 1192
T 1214
T 0694
T 1190
T 1189
T 1675
T 1356
T 1216
T 0184
T 1666
T 1191
T 0275
T 0256
T 0255
T 1672
T 0274
T 1350
T 1669
T 1212
T 0192
T 0201
T 1194
T 0257
T 1208
T 1210
T 1207
T 1209
T 1670
T 1671
T 0363
T 0273
T 0839
T 1070
T 1607
T 0955
T 1492
T 0917
T 1574
T 1199
T 0763
T 1136
T 1162
T 1876
T 0730
T 0775
T 1111
T 1122
T 1119
T 1796
T 0990
T 1650
T 2031
T 1305
T 1879
T 0882
T 1311
T 1291
T 1870
T 1750
T 0980
T 0994
T 0933
T 1922
T 0936
T 1917
T 1293
T 1563
T 1502
T 1959
T 1753
T 0091
T 0085
T 0214
T 2027
T 1507
T 1481
T 0554
T 0988
T 1577
T 0974
T 1571
T 0964
T 0723
T 1368
T 0415
T 1168
T 1230
T 0558
T 0940
T 0978

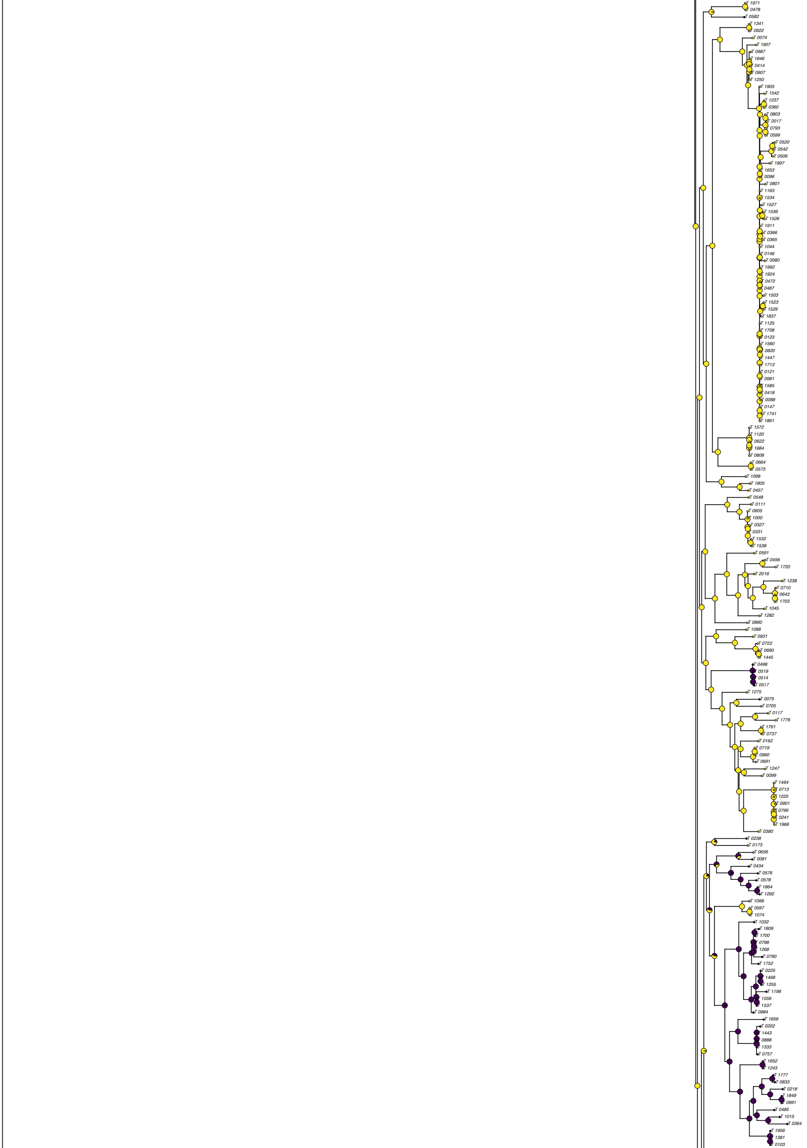
T 1971
T 0478
T 0582
T 1341
T 0922
T 0074
T 1907
T 0987
T 1846
T 0414
T 0907
T 1250
T 1905
T 1542
T 1237
T 0360
T 0803
T 2017
T 0793
T 0599
T 0520
T 0542
T 0506
T 1997
T 1653
T 0096
T 0801
T 1163
T 1534
T 1527
T 1536
T 1526
T 1911
T 0366
T 0365
T 1044
T 0146
T 0080
T 1992
T 1924
T 0472
T 0487
T 1503
T 1523
T 1529
T 1837
T 1125
T 1708
T 0123
T 1560
T 0820
T 1447
T 1713
T 0121
T 0981
T 1585
T 0418
T 0088
T 0147
T 1741
T 1861
T 1572
T 1120
T 0622
T 1984
T 0808
T 0664
T 0573
T 1068
T 1805
T 0457
T 0548
T 0111
T 0605
T 1000
T 0327
T 0331
T 1532
T 1538
T 0591
T 0456
T 1720
T 2016
T 1238
T 0710
T 0643
T 1703
T 1045
T 1282
T 0890
T 1088
T 0931
T 0722
T 0690
T 1445
T 0498
T 0519
T 0514
T 0517
T 1275
T 0075
T 0705
T 0117
T 1776
T 1761
T 0737
T 0162
T 0719
T 0960
T 0691
T 1247
T 0099
T 1464
T 0713
T 1225
T 0901
T 0799
T 0241
T 1966
T 0380
T 0238
T 0173
T 0656
T 0081
T 0434
T 0576
T 0578
T 1864
T 1292
T 1066
T 0597
T 1074
T 1032
T 1809
T 1700
T 0798
T 1268
T 0790
T 1752
T 0225
T 1468
T 1255
T 1198
T 1556
T 1537
T 0984
T 1659
T 0202
T 1443
T 0888
T 1333
T 0757
T 1652
T 1243
T 1777
T 0833
T 0218
T 1849
T 0991
T 0485
T 1015
T 0364
T 1956
T 1381
T 0103

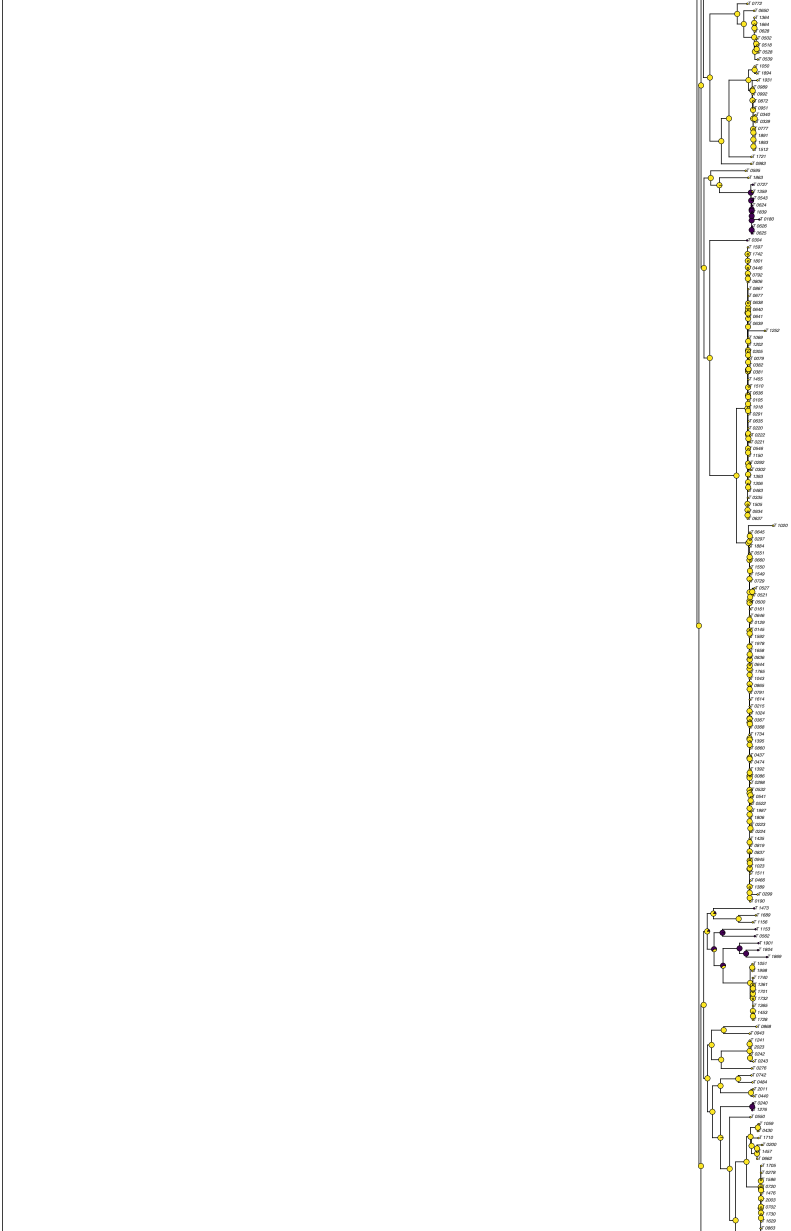
T 0772
T 0650
T 1364
T 1664
T 0628
T 0502
T 0518
T 0528
T 0539
T 1050
T 1894
T 1931
T 0989
T 0992
T 0872
T 0951
T 0340
T 0339
T 0777
T 1891
T 1893
T 1512
T 1721
T 0983
T 0595
T 1863
T 0727
T 1359
T 0543
T 0624
T 1839
T 0180
T 0626
T 0625
T 0304
T 1597
T 1742
T 1801
T 0446
T 0792
T 0806
T 0867
T 0677
T 0638
T 0640
T 0641
T 0639
T 1252
T 1069
T 1202
T 0305
T 0079
T 0382
T 0381
T 1455
T 1510
T 0636
T 0105
T 1918
T 0291
T 0635
T 0220
T 0222
T 0221
T 0546
T 1150
T 0292
T 0302
T 1393
T 1306
T 0483
T 0335
T 1505
T 0934
T 0637
T 1020
T 0645
T 0297
T 1884
T 0551
T 0660
T 1550
T 1549
T 0729
T 0527
T 0521
T 0500
T 0161
T 0646
T 0129
T 0145
T 1592
T 1978
T 1658
T 0836
T 0644
T 1765
T 1043
T 0865
T 0791
T 1614
T 0215
T 1024
T 0367
T 0368
T 1734
T 1395
T 0860
T 0437
T 0474
T 1392
T 0086
T 0298
T 0532
T 0541
T 0522
T 1987
T 1806
T 0223
T 0224
T 1435
T 0819
T 0837
T 0945
T 1023
T 1511
T 0466
T 1389
T 0299
T 0190
T 1473
T 1689
T 1156
T 1153
T 0562
T 1901
T 1804
T 1869
T 1051
T 1998
T 1740
T 1361
T 1701
T 1732
T 1365
T 1453
T 1728
T 0868
T 0943
T 1241
T 2023
T 0242
T 0243
T 0276
T 0742
T 0484
T 2011
T 0440
T 0240
T 1276
T 0550
T 1059
T 0430
T 1710
T 0200
T 1457
T 0662
T 1705
T 0278
T 1586
T 0720
T 1476
T 2003
T 0702
T 1730
T 1629
T 0863

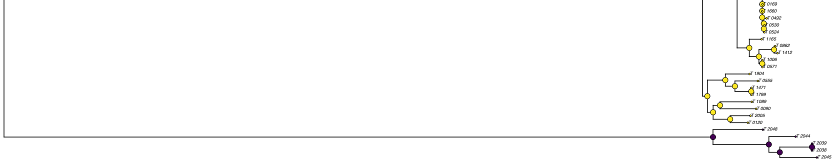
T 0169
T 1660
T 0492
T 0530
T 0524
T 1165
T 0862
T 1412
T 1006
T 0571
T 1904
T 0555
T 1471
T 1799
T 1089
T 0090
T 2005
T 0120
T 2048
T 2044
T 2039
T 2038
T 2045

Effector gene: tle3

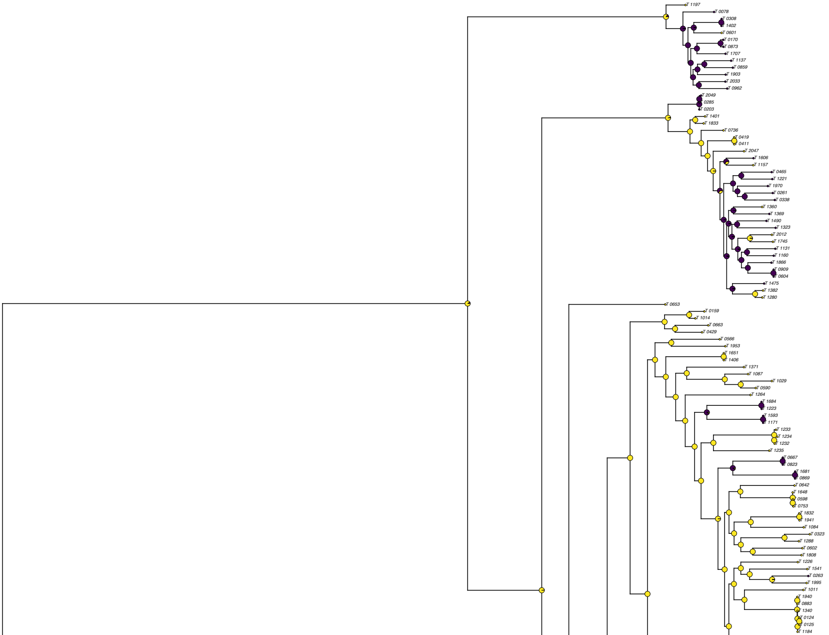

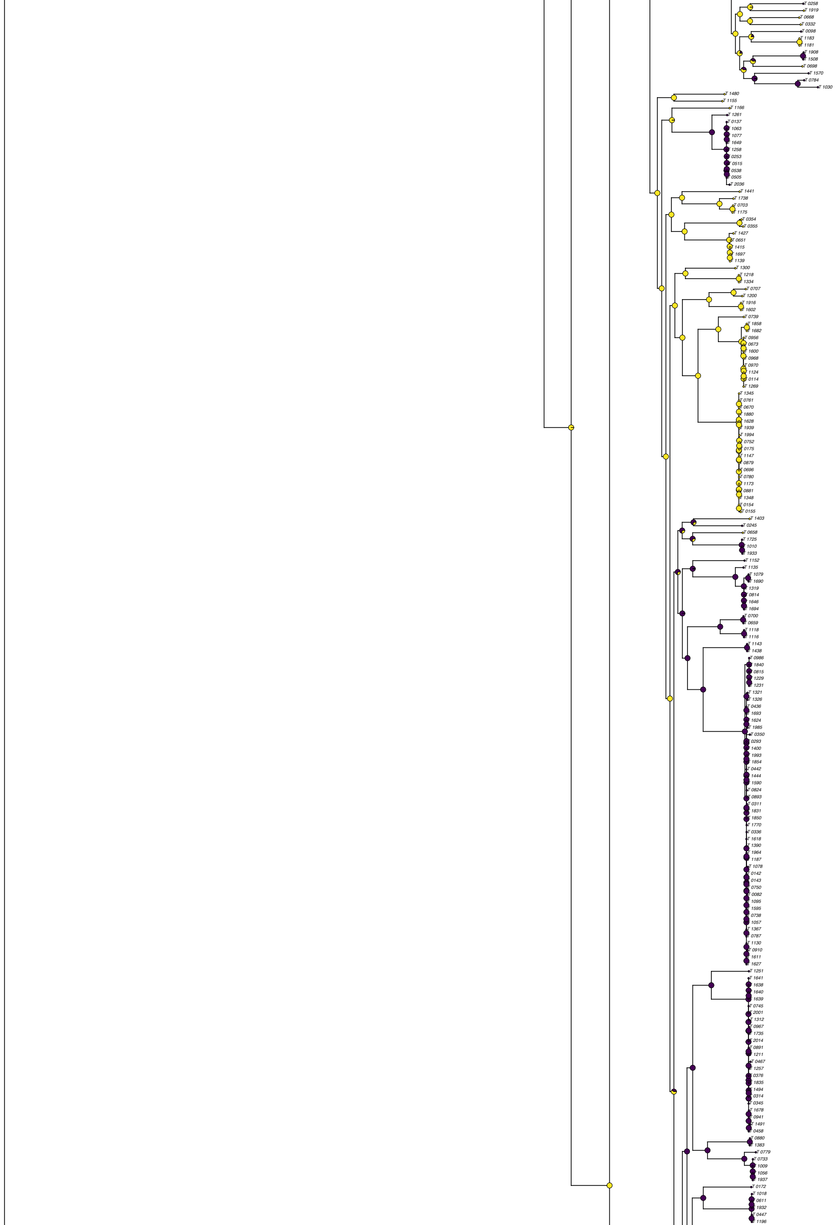
T 0258
T 1919
T 0668
T 0332
T 0098
T 1183
1181
T 1908
1508
T 0698
T 1570
T 0784
T 1030
T 1480
T 1155
T 1166
T 1261
T 0137
1063
1077
T 1649
1258
0253
0515
0538
T 0505
T 2036
T 1441
T 1738
T 0703
T 1175
T 0354
T 0355
T 1427
T 0651
1415
T 1697
1139
T 1300
T 1218
1334
T 0707
T 1200
T 1916
T 1602
T 0739
T 1858
1682
T 0956
0673
1600
0968
T 0970
1124
0114
T 1269
T 1345
T 0761
0670
1880
T 1628
1939
T 1994
0752
0175
T 1147
0879
T 0696
T 0780
1173
0881
1348
0154
T 0155
T 1403
T 0245
T 0658
T 1725
1010
T 1933
T 1152
T 1135
T 1079
T 1690
1319
0814
1646
T 1694
T 0700
T 0659
T 1118
T 1116
T 1143
1438
T 0986
1840
0815
1229
T 1231
T 1321
T 1326
T 0436
1693
1624
T 1985
T 0350
0293
1400
1993
1854
T 0442
1444
1590
T 0824
0893
T 0311
1831
1850
T 1770
T 0336
T 1618
T 1390
1964
1187
T 1078
0142
0143
0750
T 0082
1095
1595
0738
1057
T 1367
T 0787
T 1130
T 0910
1611
T 1627
T 1251
T 1641
1638
1640
1639
T 0745
T 2001
T 1312
T 0967
T 1735
2014
T 0891
1211
T 0467
T 1257
T 0376
T 1835
1494
0314
T 0345
T 1678
0941
T 1491
T 0458
T 0880
T 1383
T 0779
T 0733
1009
1056
T 1937
T 0172
T 1018
0611
1932
T 0447
T 1196

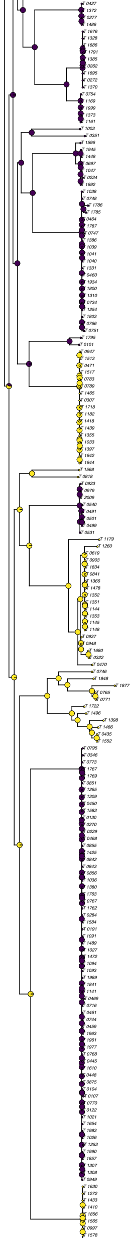
T 0427
1372
0277
1486
T 1676
T 1328
T 1686
1791
1385
0262
T 1695
T 0272
T 1370
T 0754
1169
1999
1373
T 1161
T 1003
T 0351
T 1596
T 1945
T 1448
0697
T 1047
T 0234
T 1692
T 1038
T 0748
T 1786
T 1785
0464
1787
T 0747
T 1386
1039
1041
T 1040
T 1331
0460
1934
1800
1310
0734
T 1254
T 1803
0766
T 0751
T 1795
T 0101
T 0947
T 1513
0471
T 1517
0783
0789
T 1465
T 0307
T 1718
T 1182
1418
T 1439
T 1355
1033
1397
T 1642
1644
T 1568
T 0818
T 0923
0979
T 2009
T 0540
0491
0501
T 0499
T 0531
T 1179
T 1260
T 0619
T 0903
T 1834
0841
T 1366
T 1478
T 1352
T 1351
T 1144
T 1353
T 1145
T 1148
T 0937
0948
T 1680
T 0322
T 0470
T 0746
T 1848
T 1877
T 0765
T 0771
T 1722
T 1496
T 1398
T 1466
T 0435
T 1552
T 0795
T 0346
T 0773
T 1767
T 1769
T 0851
T 1265
1309
0450
T 1583
T 0130
T 0270
T 0229
0468
T 0855
1425
0842
T 0843
0856
T 1036
1380
1763
0767
T 1762
T 0284
T 1584
T 0191
T 1091
T 1489
1027
1472
1094
T 1093
T 1989
1841
1141
T 0469
T 0716
T 0461
T 0744
0459
T 1963
1961
1977
T 0768
0445
1610
0448
T 0875
T 0104
T 0107
T 0770
0122
T 1021
T 1654
T 1983
1026
T 1253
T 1990
T 1857
1307
1308
T 0949
T 1630
T 1272
T 1433
1410
1856
1565
T 0997
1578

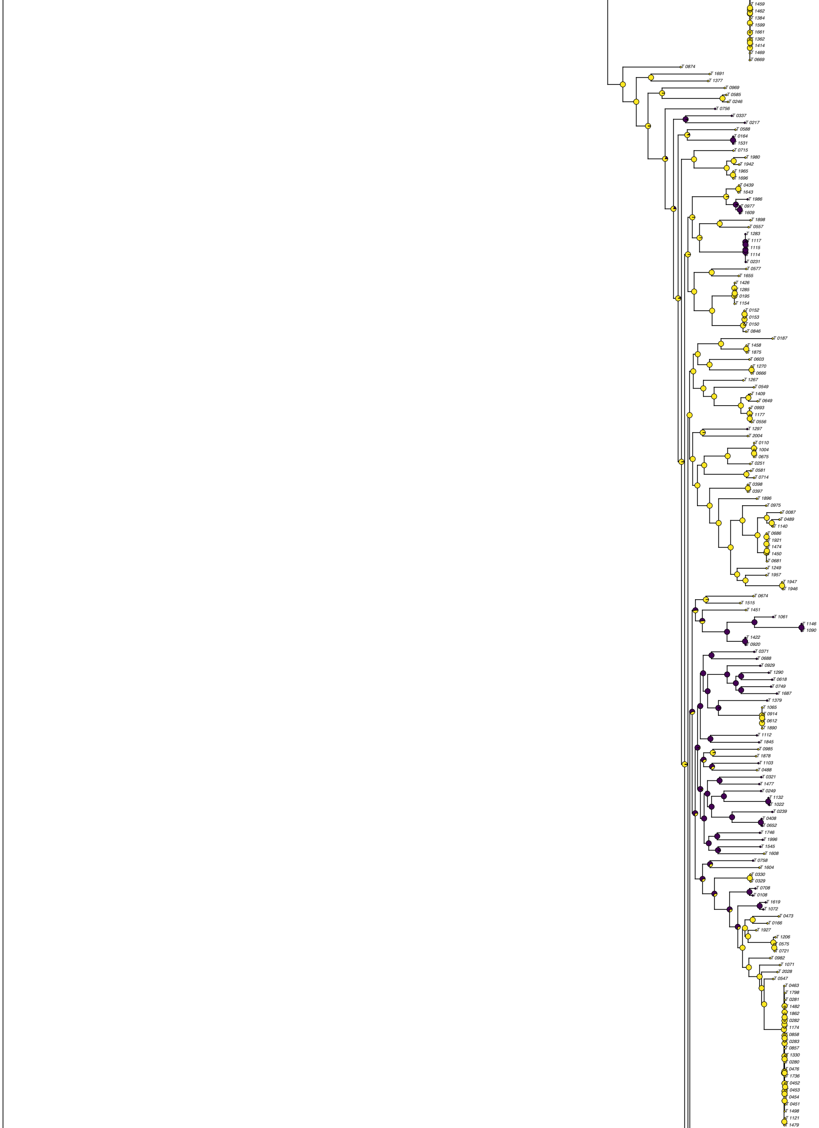
T 1459
T 1462
T 1384
T 1599
T 1661
T 1362
T 1414
T 1469
T 0669
T 0874
T 1691
T 1377
T 0969
T 0585
T 0246
T 0756
T 0337
T 0217
T 0588
T 0164
T 1531
T 0715
T 1980
T 1942
T 1965
T 1696
T 0439
T 1643
T 1986
T 0977
T 1609
T 1898
T 0557
T 1283
T 1117
T 1115
T 1114
T 0231
T 0577
T 1655
T 1426
T 1285
T 0195
T 1154
T 0152
T 0153
T 0150
T 0846
T 0187
T 1458
T 1875
T 0603
T 1270
T 0666
T 1267
T 0549
T 1409
T 0649
T 0993
T 1177
T 0556
T 1297
T 2004
T 0110
T 1004
T 0675
T 0251
T 0581
T 0714
T 0398
T 0397
T 1896
T 0975
T 0087
T 0489
T 1140
T 0686
T 1921
T 1474
T 1450
T 0681
T 1249
T 1957
T 1947
T 1946
T 0674
T 1515
T 1451
T 1061
T 1146
T 1090
T 1422
T 0920
T 0371
T 0688
T 0929
T 1290
T 0618
T 0749
T 1687
T 1379
T 1065
T 0914
T 0612
T 1890
T 1112
T 1845
T 0985
T 1878
T 1103
T 0488
T 0321
T 1477
T 0249
T 1132
T 1022
T 0239
T 0408
T 0652
T 1746
T 1996
T 1545
T 1608
T 0758
T 1604
T 0330
T 0329
T 0708
T 0108
T 1619
T 1072
T 0473
T 0166
T 1927
T 1206
T 0575
T 0721
T 0982
T 1071
T 2028
T 0547
T 0463
T 1798
T 0281
T 1482
T 1862
T 0282
T 1174
T 0858
T 0283
T 0857
T 1330
T 0280
T 0476
T 1736
T 0452
T 0453
T 0454
T 0451
T 1498
T 1121
T 1479

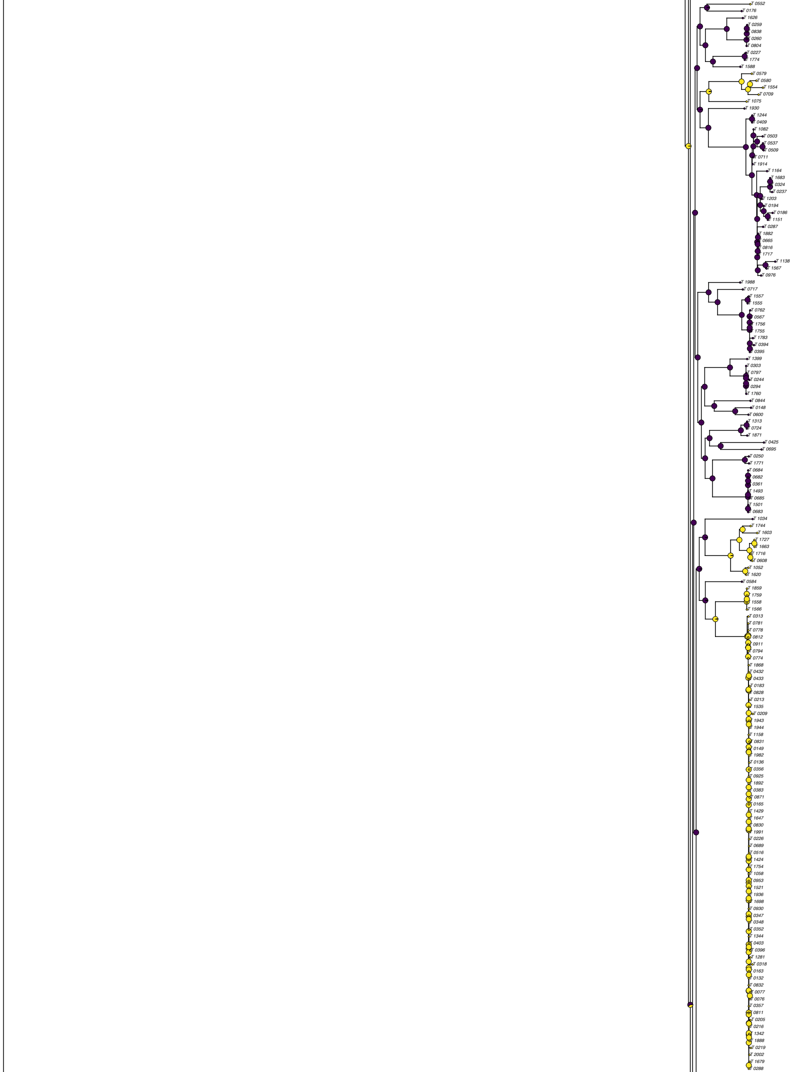
T 0552
T 0176
T 1626
T 0259
T 0838
T 0260
T 0804
T 0227
T 1774
T 1588
T 0579
T 0580
T 1554
T 0709
T 1075
T 1930
T 1244
T 0409
T 1082
T 0503
T 0537
T 0509
T 0711
T 1914
T 1164
T 1683
T 0324
T 0237
T 1203
T 0194
T 0186
T 1151
T 0287
T 1882
T 0665
T 0816
T 1717
T 1138
T 1567
T 0976
T 1988
T 0717
T 1557
T 1555
T 0762
T 0567
T 1756
T 1755
T 1783
T 0394
T 0395
T 1399
T 0303
T 0797
T 0244
T 0294
T 1760
T 0844
T 0148
T 0600
T 1313
T 0724
T 1871
T 0425
T 0695
T 0250
T 1771
T 0684
T 0682
T 0361
T 1493
T 0685
T 1501
T 0683
T 1034
T 1744
T 1603
T 1727
T 1663
T 1716
T 0608
T 1052
T 1620
T 0584
T 1859
T 1759
T 1558
T 1566
T 0313
T 0781
T 0778
T 0812
T 0911
T 0794
T 0774
T 1868
T 0432
T 0433
T 0183
T 0828
T 0213
T 1535
T 0209
T 1943
T 1944
T 1158
T 0831
T 0149
T 1982
T 0136
T 0356
T 0925
T 1892
T 0383
T 0871
T 0165
T 1429
T 1647
T 0830
T 1991
T 0226
T 0689
T 0516
T 1424
T 1754
T 1058
T 0953
T 1521
T 1936
T 1698
T 0930
T 0347
T 0348
T 0352
T 1344
T 0403
T 0396
T 1281
T 0318
T 0163
T 0132
T 0832
T 0077
T 0076
T 0357
T 0811
T 0205
T 0216
T 1342
T 1888
T 0219
T 2002
T 1679
T 0288

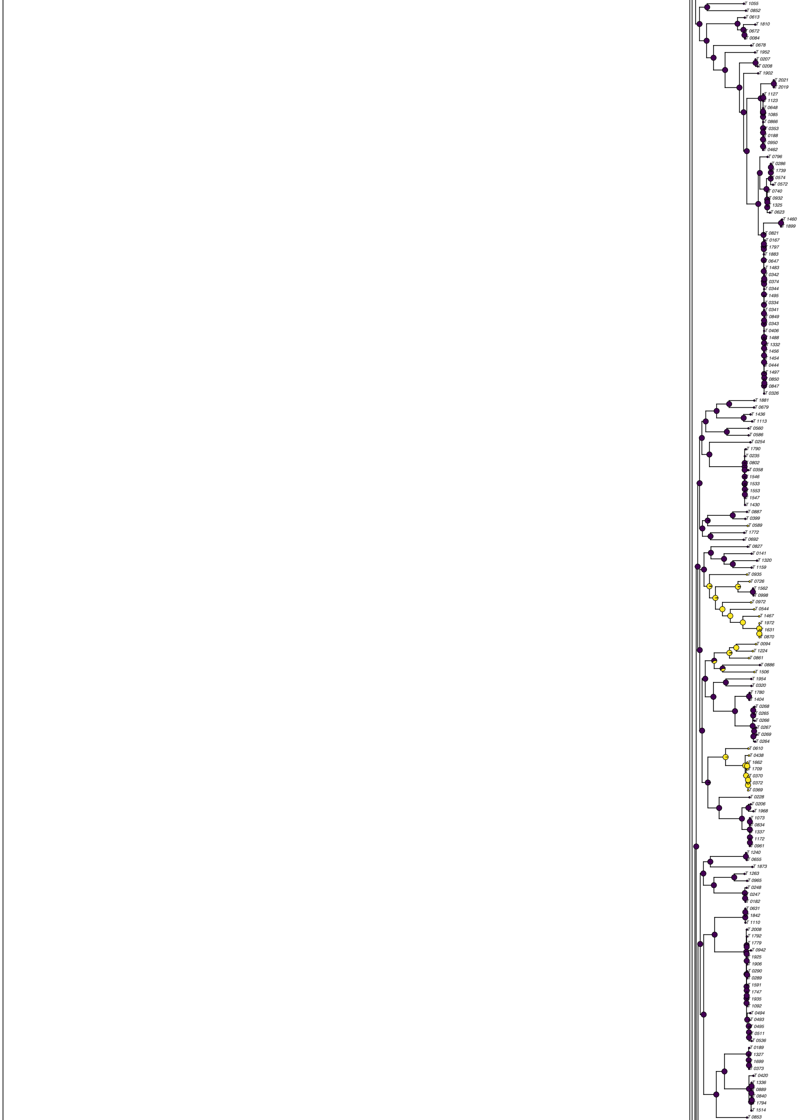

T 1055
T 0852
T 0613
T 1810
0672
T 0084
T 0678
T 1952
T 0207
T 0208
T 1902
T 2021
T 2019
T 1127
T 1123
T 0648
1085
T 0866
T 0353
T 0188
0950
T 0462
T 0796
T 0286
1739
0574
T 0572
T 0740
T 0932
1325
T 0623
T 1460
T 1899
T 0821
T 0167
1797
T 1883
0647
T 1483
T 0342
0374
T 0344
1495
T 0334
T 0341
T 0849
0343
T 0406
1488
T 1332
1456
1454
0444
1497
0850
0847
T 0326
T 1881
T 0679
T 1436
T 1113
T 0560
T 0586
T 0254
T 1790
T 0235
0802
T 0358
1546
1533
1553
T 1547
T 1430
T 0887
T 0399
T 0589
T 1772
T 0692
T 0827
T 0141
T 1320
T 1159
T 0935
T 0726
T 1562
T 0998
T 0972
T 0544
T 1467
T 1972
1631
0870
T 0094
T 1224
T 0861
T 0886
T 1506
T 1954
T 0320
T 1780
T 1404
T 0268
0265
T 0266
T 0267
T 0269
T 0264
T 0610
T 0438
T 1662
1709
T 0370
0372
T 0369
T 0228
T 0206
T 1968
T 1073
T 0834
1337
1172
T 0961
T 1240
T 0655
T 1873
T 1263
T 0965
T 0248
T 0247
T 0182
T 0631
1842
T 1110
T 2008
T 1792
T 1779
T 0942
T 1925
T 1906
T 0290
T 0289
T 1591
1747
1935
T 1092
T 0494
T 0493
0495
0511
T 0536
T 0189
1327
1699
T 0373
T 0420
T 1336
0889
T 0840
1794
T 1514
T 0853

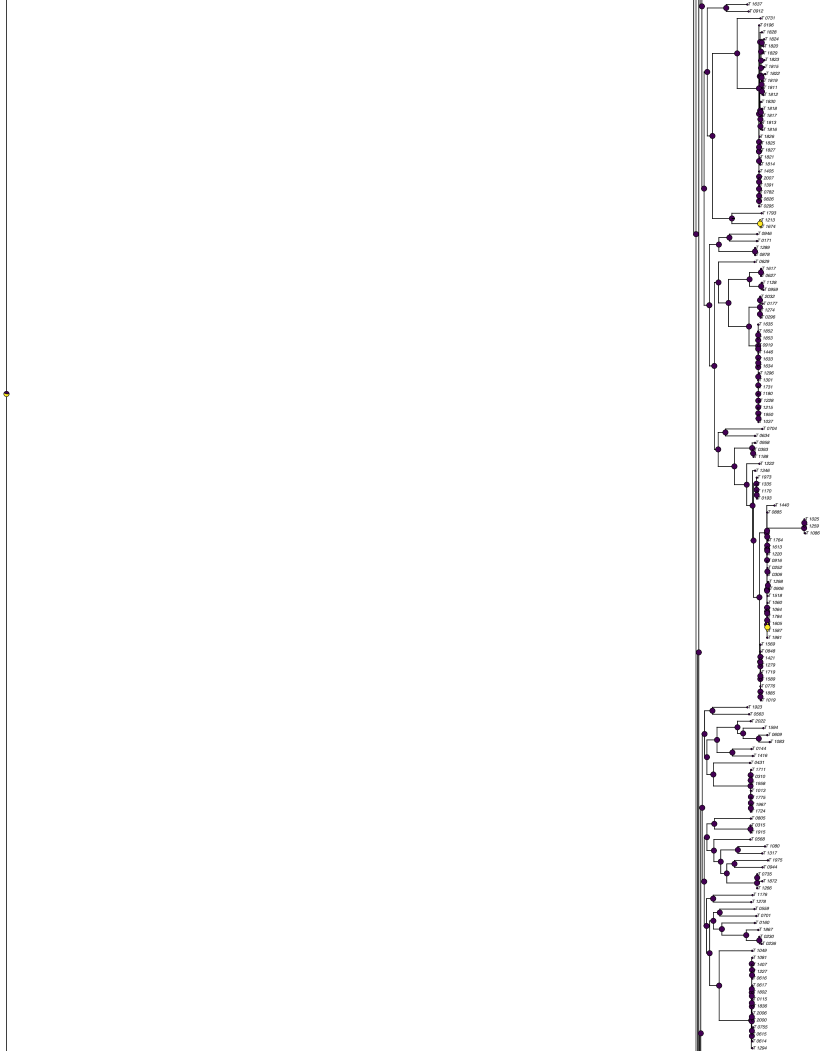
T 1637
T 0912
T 0731
T 0196
T 1828
T 1824
T 1820
T 1829
T 1823
T 1815
T 1822
T 1819
T 1811
T 1812
T 1830
T 1818
T 1817
T 1813
T 1816
T 1826
T 1825
T 1827
T 1821
T 1814
T 1405
T 2007
T 1391
T 0782
T 0826
T 0295
T 1793
T 1213
T 1674
T 0946
T 0171
T 1289
T 0878
T 0629
T 1617
T 0627
T 1128
T 0959
T 2032
T 0177
T 1274
T 0296
T 1635
T 1852
T 1853
T 0919
T 1446
T 1633
T 1634
T 1296
T 1301
T 1731
T 1180
T 1228
T 1215
T 1950
T 1037
T 0704
T 0634
T 0958
T 0393
T 1188
T 1222
T 1346
T 1973
T 1335
T 1170
T 0193
T 1440
T 0885
T 1025
T 1259
T 1086
T 1764
T 1613
T 1220
T 0916
T 0252
T 0306
T 1298
T 0906
T 1518
T 1060
T 1064
T 1784
T 1605
T 1587
T 1981
T 1569
T 0848
T 1421
T 1279
T 1719
T 1589
T 0776
T 1885
T 1019
T 1923
T 0563
T 2022
T 1594
T 0609
T 1083
T 0144
T 1416
T 0431
T 1711
T 0310
T 1958
T 1013
T 1775
T 1967
T 1724
T 0805
T 0315
T 1915
T 0568
T 1080
T 1317
T 1975
T 0944
T 0735
T 1872
T 1266
T 1176
T 1278
T 0559
T 0701
T 0160
T 1867
T 0230
T 0236
T 1049
T 1081
T 1407
T 1227
T 0616
T 0617
T 1802
T 0115
T 1836
T 2006
T 2000
T 0755
T 0615
T 0614
T 1294

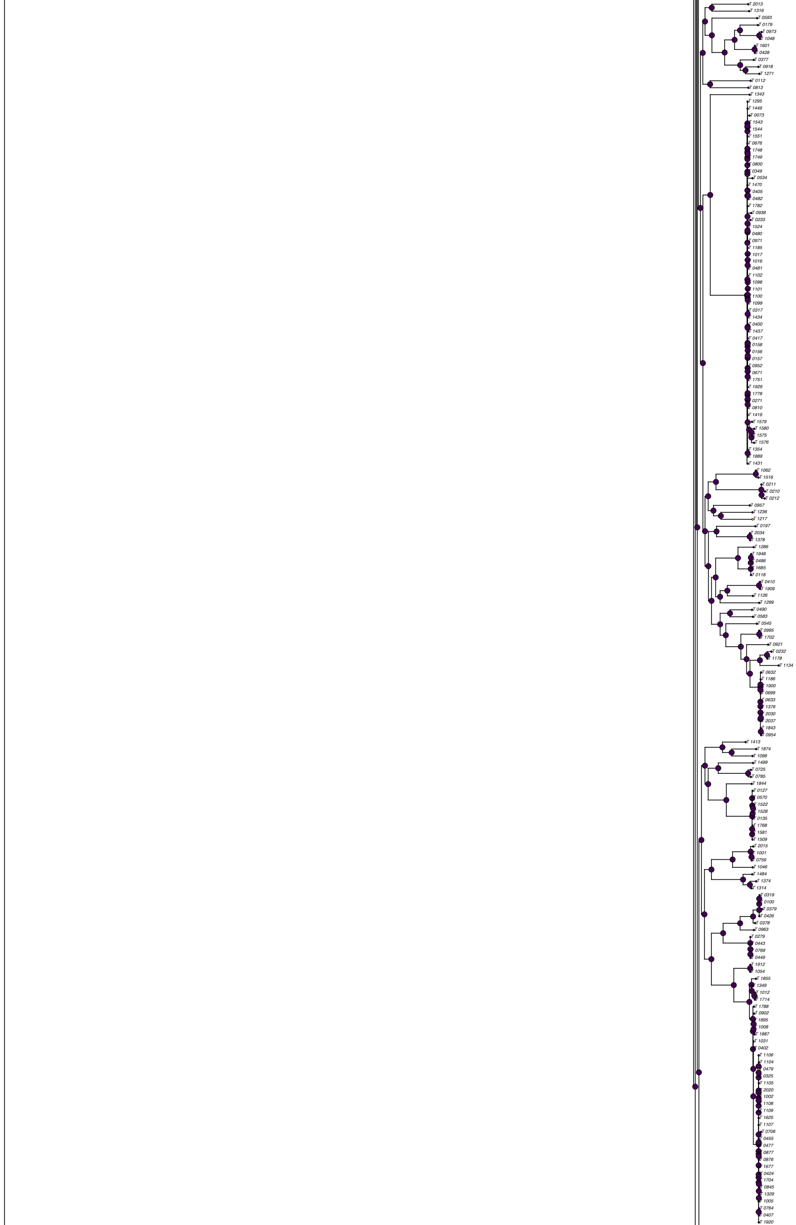
T 2013
T 1316
T 0593
T 0179
T 0973
T 1048
T 1601
T 0428
T 0377
T 0918
T 1271
T 0112
T 0813
T 1343
T 1295
T 1449
T 0073
T 1543
T 1544
T 1551
T 0676
T 1748
T 1749
T 0800
T 0349
T 0534
T 1470
T 0405
T 0482
T 1782
T 0938
T 0233
T 1524
T 0480
T 0971
T 1185
T 1017
T 1016
T 0481
T 1102
T 1098
T 1101
T 1100
T 1099
T 0317
T 1434
T 0400
T 1437
T 0417
T 0158
T 0156
T 0157
T 0952
T 0671
T 1751
T 1929
T 1778
T 0271
T 0810
T 1419
T 1579
T 1580
T 1575
T 1576
T 1354
T 1889
T 1431
T 1062
T 1516
T 0211
T 0210
T 0212
T 0957
T 1236
T 1217
T 0197
T 2034
T 1378
T 1286
T 1948
T 0486
T 1685
T 0118
T 0410
T 1909
T 1126
T 1299
T 0490
T 0583
T 0545
T 0995
T 1702
T 0921
T 0232
T 1178
T 1134
T 0632
T 1186
T 1900
T 0699
T 0633
T 1376
T 2030
T 2037
T 1843
T 0954
T 1413
T 1874
T 1096
T 1499
T 0725
T 0785
T 1844
T 0127
T 0570
T 1522
T 1528
T 0135
T 1768
T 1581
T 1509
T 2015
T 1001
T 0759
T 1046
T 1484
T 1374
T 1314
T 0319
T 0100
T 0379
T 0426
T 0378
T 0963
T 0279
T 0443
T 0769
T 0449
T 1912
T 1054
T 1855
T 1349
T 1012
T 1714
T 1788
T 0902
T 1895
T 1008
T 1887
T 1031
T 0402
T 1106
T 1104
T 0479
T 0325
T 1105
T 2020
T 1002
T 1108
T 1109
T 1625
T 1107
T 0706
T 0455
T 0477
T 0877
T 0876
T 1677
T 0424
T 1704
T 0845
T 1329
T 1005
T 0764
T 0407
T 1920

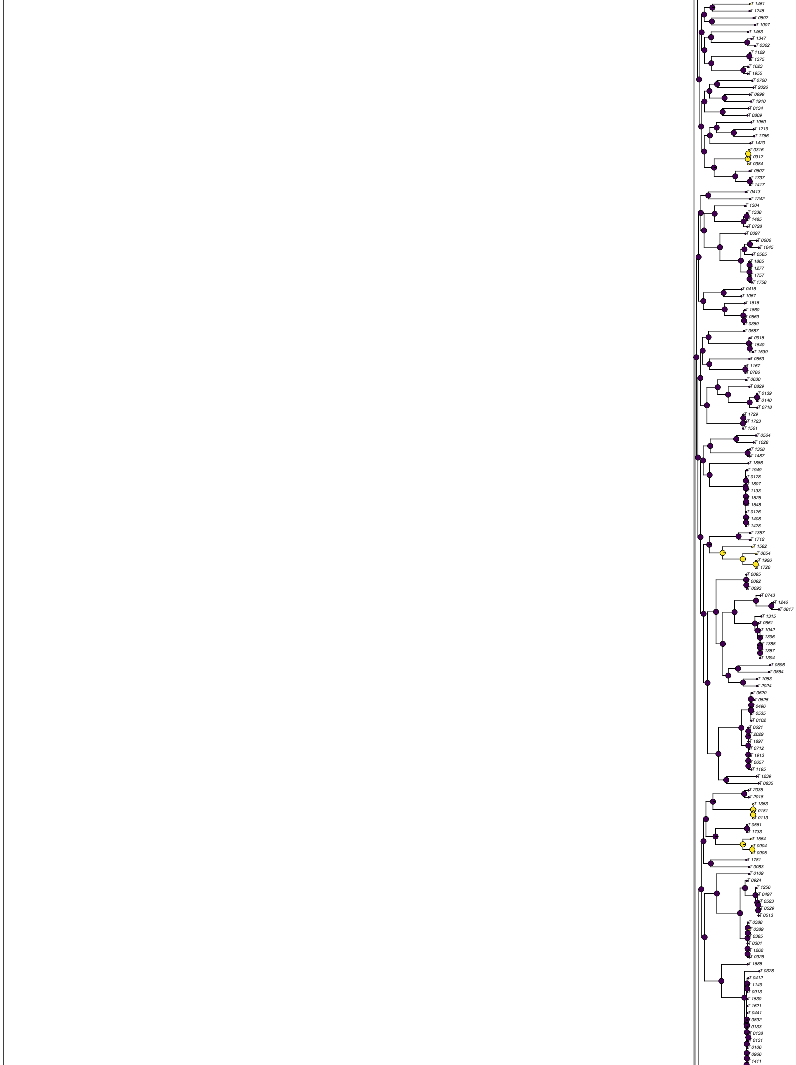

T 1461
T 1245
T 0592
T 1007
T 1463
T 1347
T 0362
T 1129
T 1375
T 1623
T 1955
T 0760
T 2026
T 0999
T 1910
T 0134
T 0809
T 1960
T 1219
T 1766
T 1420
T 0316
0312
T 0384
T 0607
T 1737
T 1417
T 0413
T 1242
T 1304
T 1338
T 1485
T 0728
T 0097
T 0606
T 1645
T 0565
T 1865
1277
1757
T 1758
T 0416
T 1067
T 1616
T 1860
0569
0359
T 0587
T 0915
1540
T 1539
T 0553
T 1167
0786
T 0630
T 0829
T 0139
0140
T 0718
T 1729
T 1723
T 1561
T 0564
T 1028
T 1358
T 1487
T 1886
T 1949
T 0178
1807
1133
1525
1548
T 0126
1408
1428
T 1357
T 1712
T 1582
T 0654
T 1926
1726
T 0095
0092
0093
T 0743
T 1246
T 0817
T 1315
T 0661
T 1042
1396
T 1388
1387
T 1394
T 0596
T 0864
T 1053
T 2024
T 0620
T 0525
0496
T 0535
T 0102
T 0621
2029
T 1897
T 0712
T 1913
0657
T 1195
T 1239
T 0835
T 2035
T 2018
T 1363
0181
0113
T 0561
T 1733
T 1564
T 0904
0905
T 1781
T 0083
T 0109
T 0924
T 1256
T 0497
T 0523
T 0529
T 0513
T 0388
T 0389
T 0385
T 0301
T 1262
T 0926
T 1688
T 0328
T 0412
1149
T 0913
T 1530
T 1621
T 0441
0892
0133
T 0138
0131
0106
0966
1411

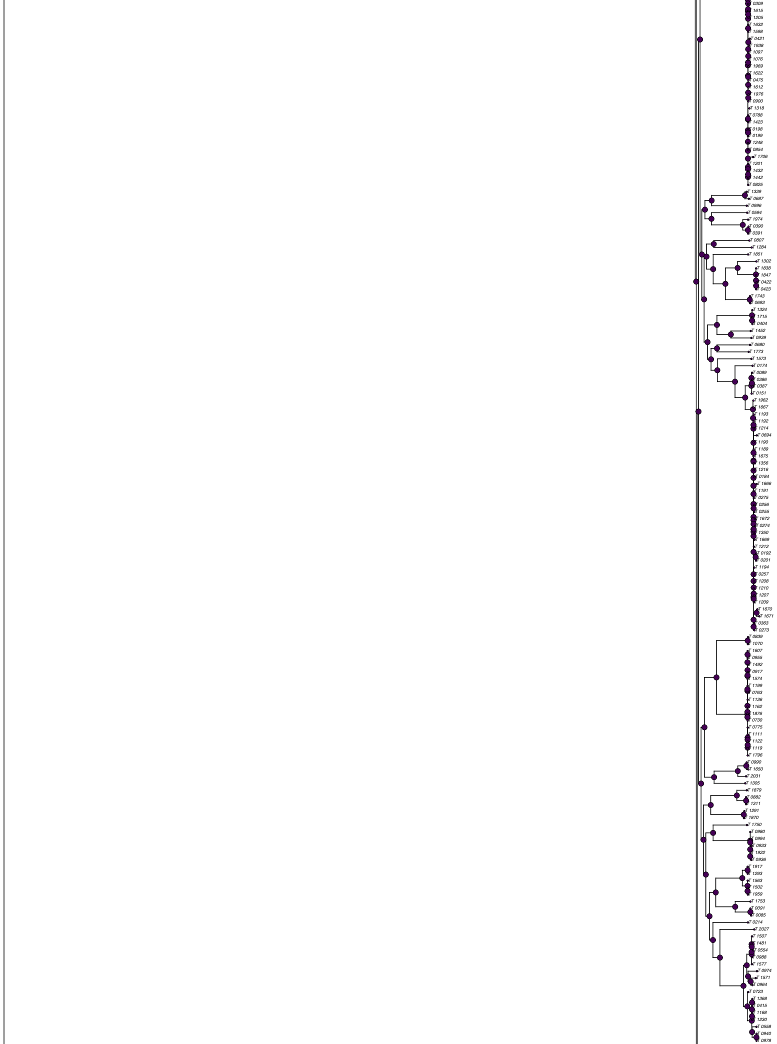

T 0309
T 1615
T 1205
T 1632
T 1598
T 0421
T 1938
T 1097
T 1076
T 1969
T 1622
T 0475
T 1612
T 1976
T 0900
T 1318
T 0788
T 1423
T 0198
T 0199
T 1248
T 0854
T 1706
T 1201
T 1432
T 1442
T 0825
T 1339
T 0687
T 0996
T 0594
T 1974
T 0390
T 0391
T 0807
T 1284
T 1851
T 1302
T 1838
T 1847
T 0422
T 0423
T 1743
T 0693
T 1324
T 1715
T 0404
T 1452
T 0939
T 0680
T 1773
T 1573
T 0174
T 0089
T 0386
T 0387
T 0151
T 1962
T 1667
T 1193
T 1192
T 1214
T 0694
T 1190
T 1189
T 1675
T 1356
T 1216
T 0184
T 1666
T 1191
T 0275
T 0256
T 0255
T 1672
T 0274
T 1350
T 1669
T 1212
T 0192
T 0201
T 1194
T 0257
T 1208
T 1210
T 1207
T 1209
T 1670
T 1671
T 0363
T 0273
T 0839
T 1070
T 1607
T 0955
T 1492
T 0917
T 1574
T 1199
T 0763
T 1136
T 1162
T 1876
T 0730
T 0775
T 1111
T 1122
T 1119
T 1796
T 0990
T 1650
T 2031
T 1305
T 1879
T 0882
T 1311
T 1291
T 1870
T 1750
T 0980
T 0994
T 0933
T 1922
T 0936
T 1917
T 1293
T 1563
T 1502
T 1959
T 1753
T 0091
T 0085
T 0214
T 2027
T 1507
T 1481
T 0554
T 0988
T 1577
T 0974
T 1571
T 0964
T 0723
T 1368
T 0415
T 1168
T 1230
T 0558
T 0940
T 0978

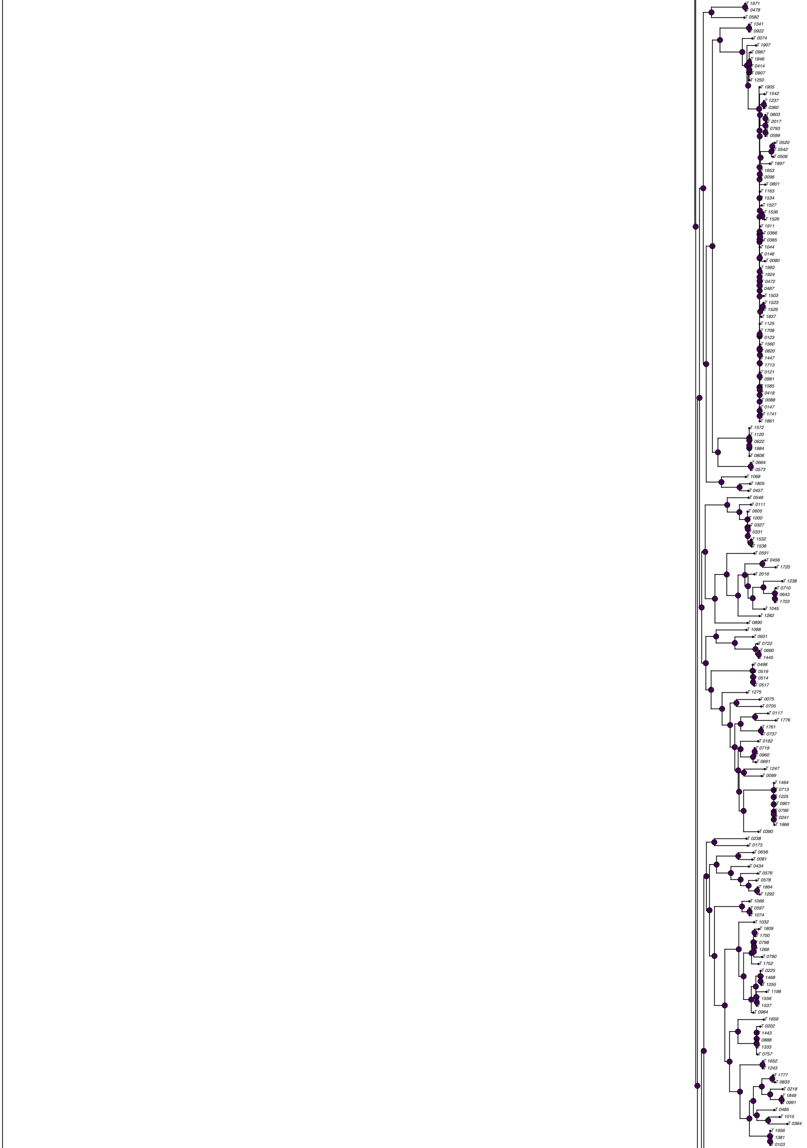
T 1971
T 0478
T 0582
T 1341
T 0922
T 0074
T 1907
T 0987
T 1846
T 0414
T 0907
T 1250
T 1905
T 1542
T 1237
T 0360
T 0803
T 2017
T 0793
T 0599
T 0520
T 0542
T 0506
T 1997
T 1653
T 0096
T 0801
T 1163
T 1534
T 1527
T 1536
T 1526
T 1911
T 0366
T 0365
T 1044
T 0146
T 0080
T 1992
T 1924
T 0472
T 0487
T 1503
T 1523
T 1529
T 1837
T 1125
T 1708
T 0123
T 1560
T 0820
T 1447
T 1713
T 0121
T 0981
T 1585
T 0418
T 0088
T 0147
T 1741
T 1861
T 1572
T 1120
T 0622
T 1984
T 0808
T 0664
T 0573
T 1068
T 1805
T 0457
T 0548
T 0111
T 0605
T 1000
T 0327
T 0331
T 1532
T 1538
T 0591
T 0456
T 1720
T 2016
T 1238
T 0710
T 0643
T 1703
T 1045
T 1282
T 0890
T 1088
T 0931
T 0722
T 0690
T 1445
T 0498
T 0519
T 0514
T 0517
T 1275
T 0075
T 0705
T 0117
T 1776
T 1761
T 0737
T 0162
T 0719
T 0960
T 0691
T 1247
T 0099
T 1464
T 0713
T 1225
T 0901
T 0799
T 0241
T 1966
T 0380
T 0238
T 0173
T 0656
T 0081
T 0434
T 0576
T 0578
T 1864
T 1292
T 1066
T 0597
T 1074
T 1032
T 1809
T 1700
T 0798
T 1268
T 0790
T 1752
T 0225
T 1468
T 1255
T 1198
T 1556
T 1537
T 0984
T 1659
T 0202
T 1443
T 0888
T 1333
T 0757
T 1652
T 1243
T 1777
T 0833
T 0218
T 1849
T 0991
T 0485
T 1015
T 0364
T 1956
T 1381
T 0103

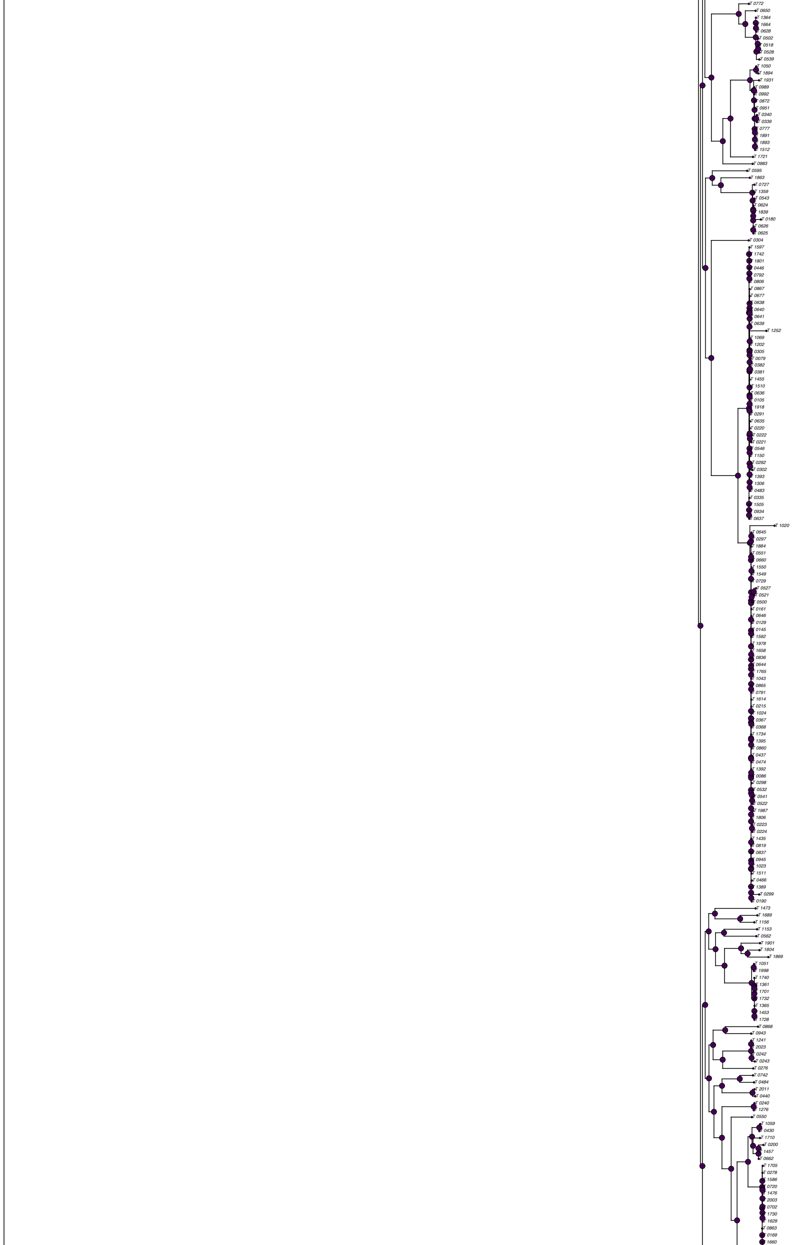
T 0772
T 0650
T 1364
1664
0628
T 0502
0518
T 0528
T 0539
T 1050
T 1894
T 1931
T 0989
0992
0872
T 0951
T 0340
T 0339
0777
1891
1893
T 1512
T 1721
T 0983
T 0595
T 1863
T 0727
T 1359
T 0543
T 0624
1839
T 0180
T 0626
T 0625
T 0304
T 1597
1742
1801
0446
0792
T 0806
T 0867
T 0677
0638
0640
0641
T 0639
T 1252
T 1069
T 1202
0305
T 0079
0382
0381
T 1455
1510
T 0636
0105
1918
T 0291
T 0635
T 0220
T 0222
T 0221
0546
T 1150
T 0292
T 0302
1393
1306
T 0483
T 0335
1505
0934
T 0637
T 1020
T 0645
0297
T 1884
T 0551
0660
T 1550
1549
0729
T 0527
T 0521
0500
T 0161
0646
T 0129
T 0145
T 1592
T 1978
T 1658
0836
0644
T 1765
T 1043
0865
T 0791
T 1614
T 0215
1024
0367
T 0368
T 1734
1395
T 0860
0437
T 0474
T 1392
0086
T 0298
T 0532
T 0541
T 0522
T 1987
1806
T 0223
T 0224
T 1435
T 0819
T 0837
0945
1023
T 1511
T 0466
1389
T 0299
T 0190
T 1473
T 1689
T 1156
T 1153
T 0562
T 1901
T 1804
T 1869
T 1051
T 1998
T 1740
1361
1701
1732
T 1365
1453
T 1728
T 0868
T 0943
T 1241
2023
0242
T 0243
T 0276
T 0742
T 0484
T 2011
T 0440
T 0240
T 1276
T 0550
T 1059
0430
T 1710
T 0200
1457
T 0662
T 1705
T 0278
1586
0720
T 1476
2003
0702
1730
T 1629
T 0863
0169
1660

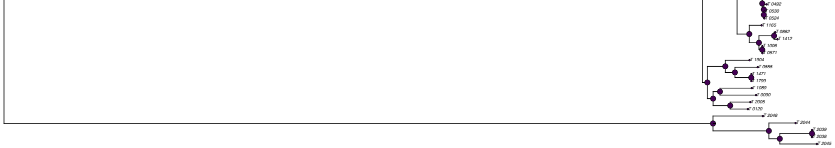
T 0492
T 0530
T 0524
T 1165
T 0862
T 1412
T 1006
T 0571
T 1904
T 0555
T 1471
T 1799
T 1089
T 0090
T 2005
T 0120
T 2048
T 2044
T 2039
T 2038
T 2045

Effector gene: tle4b

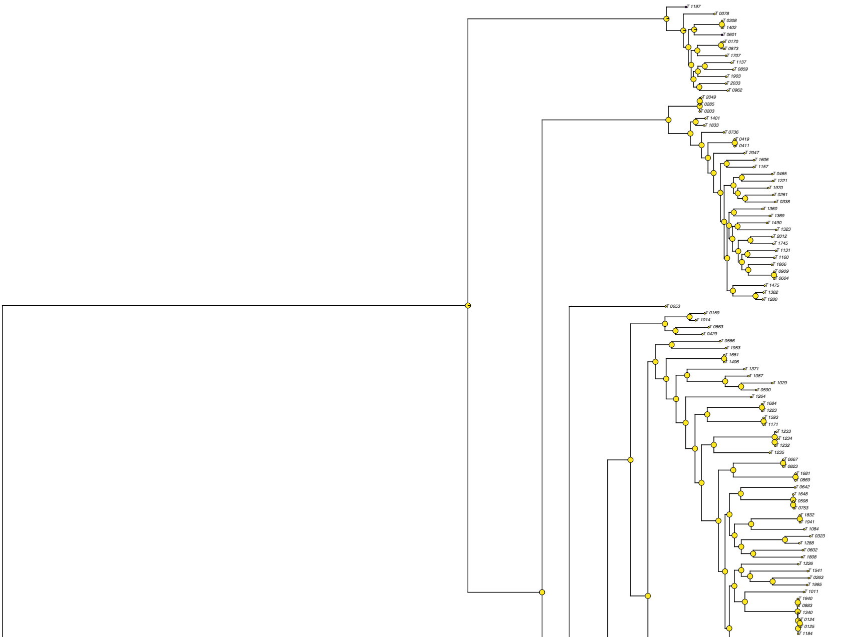

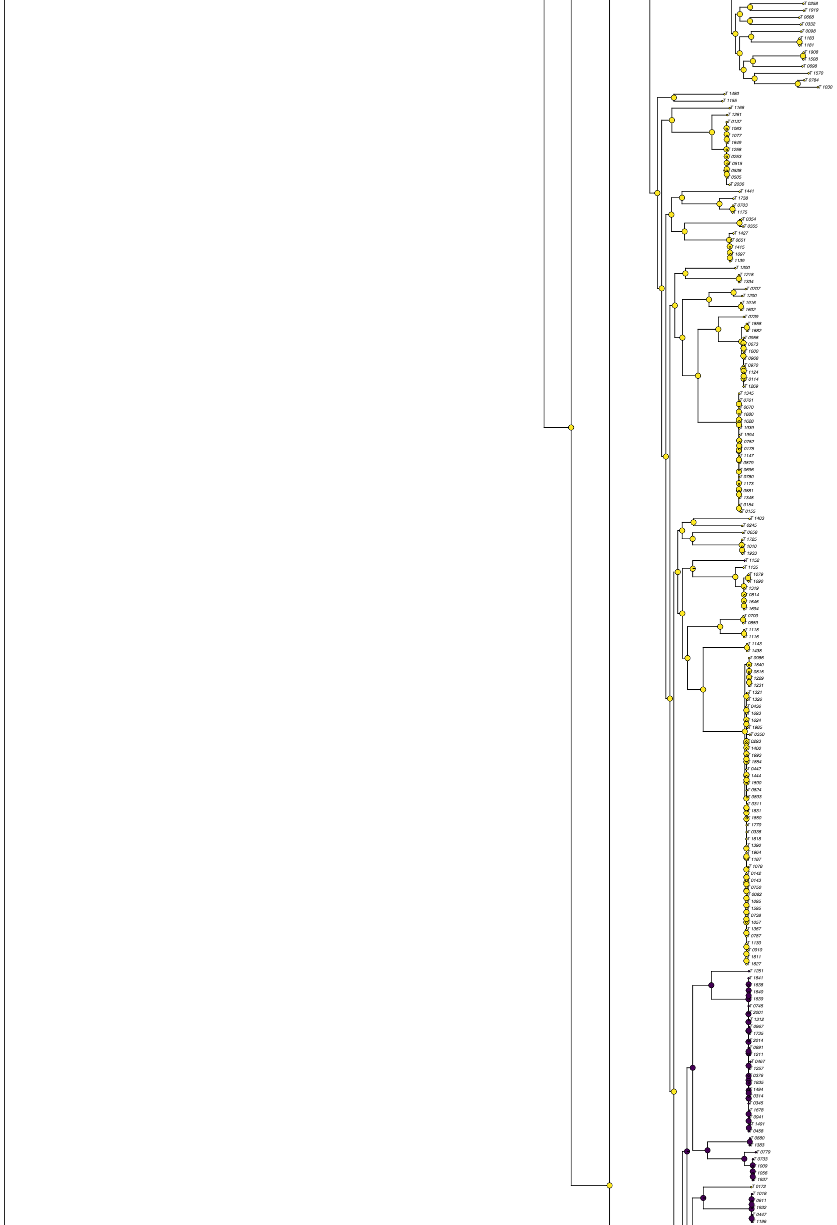

T 0258
T 1919
T 0668
T 0332
T 0098
T 1183
1181
T 1908
1508
T 0698
T 1570
T 0784
T 1030
T 1480
T 1155
T 1166
T 1261
T 0137
1063
1077
1649
1258
0253
0515
0538
0505
T 2036
T 1441
T 1738
T 0703
T 1175
T 0354
T 0355
T 1427
T 0651
1415
1697
1139
T 1300
T 1218
1334
T 0707
T 1200
T 1916
1602
T 0739
T 1858
1682
T 0956
0673
1600
0968
T 0970
1124
0114
T 1269
T 1345
T 0761
0670
1880
1628
1939
T 1994
0752
0175
T 1147
0879
T 0696
T 0780
1173
0881
1348
0154
T 0155
T 1403
T 0245
T 0658
T 1725
1010
1933
T 1152
T 1135
T 1079
1690
1319
0814
1646
1694
T 0700
0659
T 1118
1116
T 1143
1438
T 0986
1840
0815
1229
1231
T 1321
1326
T 0436
1693
1624
T 1985
T 0350
0293
1400
1993
1854
T 0442
1444
1590
T 0824
0893
T 0311
1831
1850
T 1770
T 0336
T 1618
T 1390
1964
1187
T 1078
0142
0143
0750
T 0082
1095
1595
0738
1057
T 1367
0787
T 1130
T 0910
1611
1627
T 1251
T 1641
1638
1640
1639
T 0745
2001
T 1312
T 0967
T 1735
2014
T 0891
1211
T 0467
T 1257
T 0376
T 1835
1494
0314
T 0345
T 1678
0941
T 1491
0458
T 0880
T 1383
T 0779
T 0733
1009
1056
1937
T 0172
T 1018
0611
1932
T 0447
T 1196

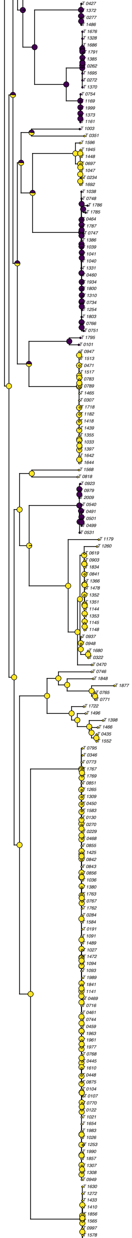
T 0427
T 1372
T 0277
T 1486
T 1676
T 1328
T 1686
T 1791
T 1385
T 0262
T 1695
T 0272
T 1370
T 0754
T 1169
T 1999
T 1373
T 1161
T 1003
T 0351
T 1596
T 1945
T 1448
T 0697
T 1047
T 0234
T 1692
T 1038
T 0748
T 1786
T 1785
T 0464
T 1787
T 0747
T 1386
T 1039
T 1041
T 1040
T 1331
T 0460
T 1934
T 1800
T 1310
T 0734
T 1254
T 1803
T 0766
T 0751
T 1795
T 0101
T 0947
T 1513
T 0471
T 1517
T 0783
T 0789
T 1465
T 0307
T 1718
T 1182
T 1418
T 1439
T 1355
T 1033
T 1397
T 1642
T 1644
T 1568
T 0818
T 0923
T 0979
T 2009
T 0540
T 0491
T 0501
T 0499
T 0531
T 1179
T 1260
T 0619
T 0903
T 1834
T 0841
T 1366
T 1478
T 1352
T 1351
T 1144
T 1353
T 1145
T 1148
T 0937
T 0948
T 1680
T 0322
T 0470
T 0746
T 1848
T 1877
T 0765
T 0771
T 1722
T 1496
T 1398
T 1466
T 0435
T 1552
T 0795
T 0346
T 0773
T 1767
T 1769
T 0851
T 1265
T 1309
T 0450
T 1583
T 0130
T 0270
T 0229
T 0468
T 0855
T 1425
T 0842
T 0843
T 0856
T 1036
T 1380
T 1763
T 0767
T 1762
T 0284
T 1584
T 0191
T 1091
T 1489
T 1027
T 1472
T 1094
T 1093
T 1989
T 1841
T 1141
T 0469
T 0716
T 0461
T 0744
T 0459
T 1963
T 1961
T 1977
T 0768
T 0445
T 1610
T 0448
T 0875
T 0104
T 0107
T 0770
T 0122
T 1021
T 1654
T 1983
T 1026
T 1253
T 1990
T 1857
T 1307
T 1308
T 0949
T 1630
T 1272
T 1433
T 1410
T 1856
T 1565
T 0997
T 1578

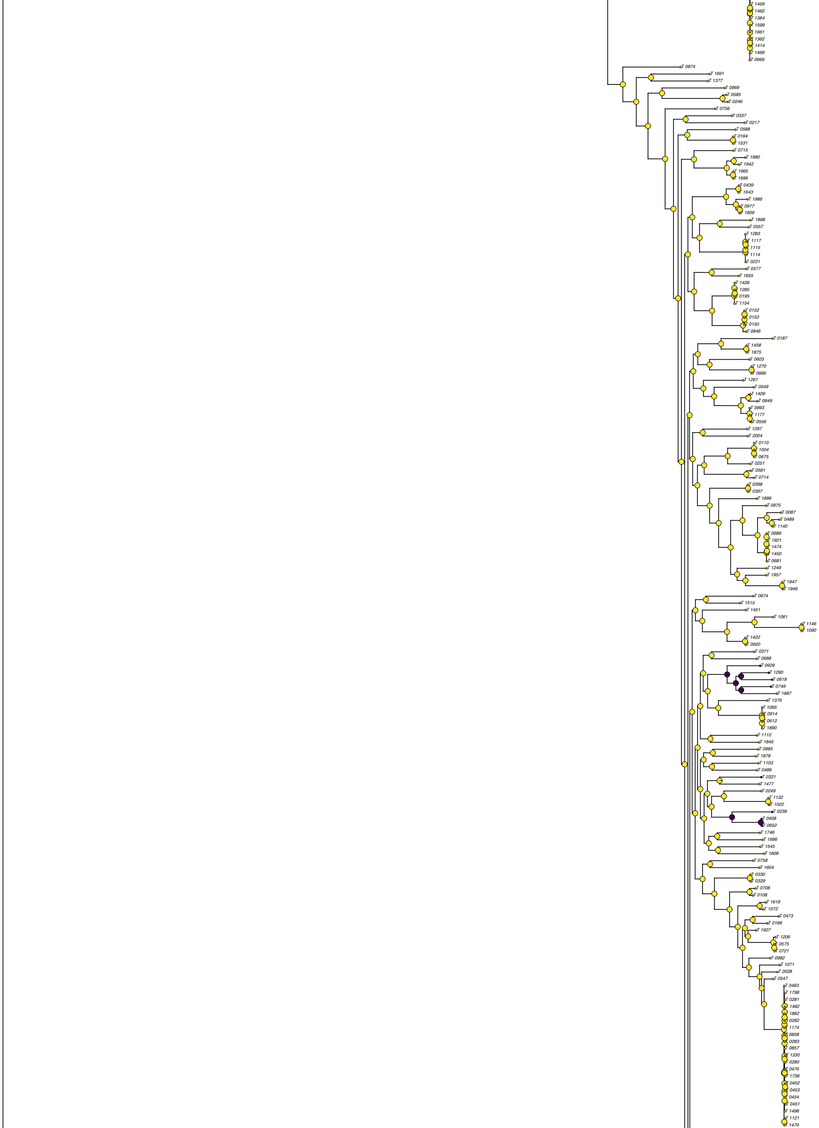
T 1459
T 1462
T 1384
T 1599
T 1661
T 1362
T 1414
T 1469
T 0669
T 0874
T 1691
T 1377
T 0969
T 0585
T 0246
T 0756
T 0337
T 0217
T 0588
T 0164
T 1531
T 0715
T 1980
T 1942
T 1965
T 1696
T 0439
T 1643
T 1986
T 0977
T 1609
T 1898
T 0557
T 1283
T 1117
T 1115
T 1114
T 0231
T 0577
T 1655
T 1426
T 1285
T 0195
T 1154
T 0152
T 0153
T 0150
T 0846
T 0187
T 1458
T 1875
T 0603
T 1270
T 0666
T 1267
T 0549
T 1409
T 0649
T 0993
T 1177
T 0556
T 1297
T 2004
T 0110
T 1004
T 0675
T 0251
T 0581
T 0714
T 0398
T 0397
T 1896
T 0975
T 0087
T 0489
T 1140
T 0686
T 1921
T 1474
T 1450
T 0681
T 1249
T 1957
T 1947
T 1946
T 0674
T 1515
T 1451
T 1061
T 1146
T 1090
T 1422
T 0920
T 0371
T 0688
T 0929
T 1290
T 0618
T 0749
T 1687
T 1379
T 1065
T 0914
T 0612
T 1890
T 1112
T 1845
T 0985
T 1878
T 1103
T 0488
T 0321
T 1477
T 0249
T 1132
T 1022
T 0239
T 0408
T 0652
T 1746
T 1996
T 1545
T 1608
T 0758
T 1604
T 0330
T 0329
T 0708
T 0108
T 1619
T 1072
T 0473
T 0166
T 1927
T 1206
T 0575
T 0721
T 0982
T 1071
T 2028
T 0547
T 0463
T 1798
T 0281
T 1482
T 1862
T 0282
T 1174
T 0858
T 0283
T 0857
T 1330
T 0280
T 0476
T 1736
T 0452
T 0453
T 0454
T 0451
T 1498
T 1121
T 1479

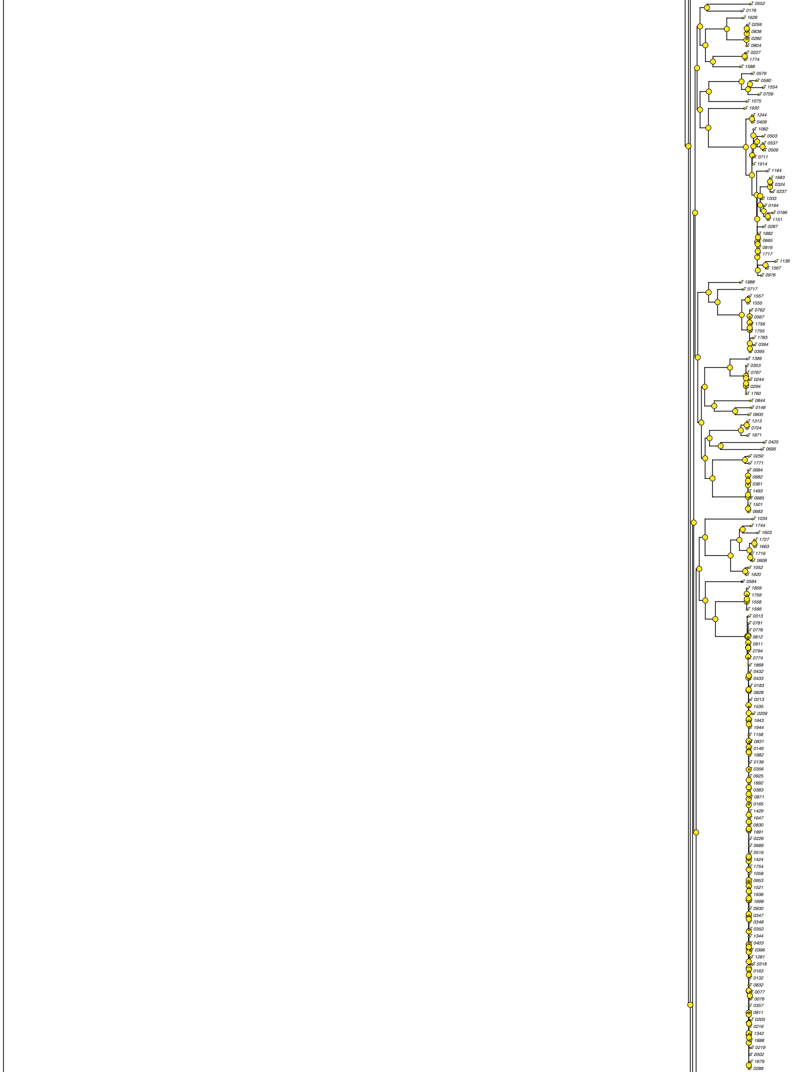
T 0552
T 0176
T 1626
T 0259
0838
0260
T 0804
T 0227
T 1774
T 1588
T 0579
T 0580
T 1554
T 0709
T 1075
T 1930
T 1244
T 0409
T 1082
T 0503
T 0537
T 0509
T 0711
T 1914
T 1164
T 1683
0324
T 0237
T 1203
T 0194
T 0186
T 1151
T 0287
T 1882
0665
T 0816
1717
T 1138
T 1567
T 0976
T 1988
T 0717
T 1557
T 1555
T 0762
0567
T 1756
1755
T 1783
T 0394
T 0395
T 1399
T 0303
T 0797
T 0244
0294
T 1760
T 0844
T 0148
T 0600
T 1313
T 0724
T 1871
T 0425
T 0695
T 0250
T 1771
T 0684
0682
0361
T 1493
T 0685
T 1501
0683
T 1034
T 1744
T 1603
T 1727
T 1663
T 1716
T 0608
T 1052
T 1620
T 0584
T 1859
1759
1558
T 1566
T 0313
T 0781
T 0778
0812
0911
T 0794
0774
T 1868
T 0432
0433
T 0183
T 0828
T 0213
1535
T 0209
1943
T 1944
T 1158
0831
0149
T 1982
T 0136
0356
T 0925
1892
0383
T 0871
0165
T 1429
T 1647
T 0830
T 1991
T 0226
T 0689
T 0516
1424
T 1754
T 1058
0953
1521
T 1936
1698
T 0930
0347
0348
T 0352
T 1344
T 0403
T 0396
T 1281
T 0318
0163
T 0132
T 0832
T 0077
T 0076
T 0357
0811
T 0205
T 0216
1342
T 1888
T 0219
T 2002
T 1679
T 0288

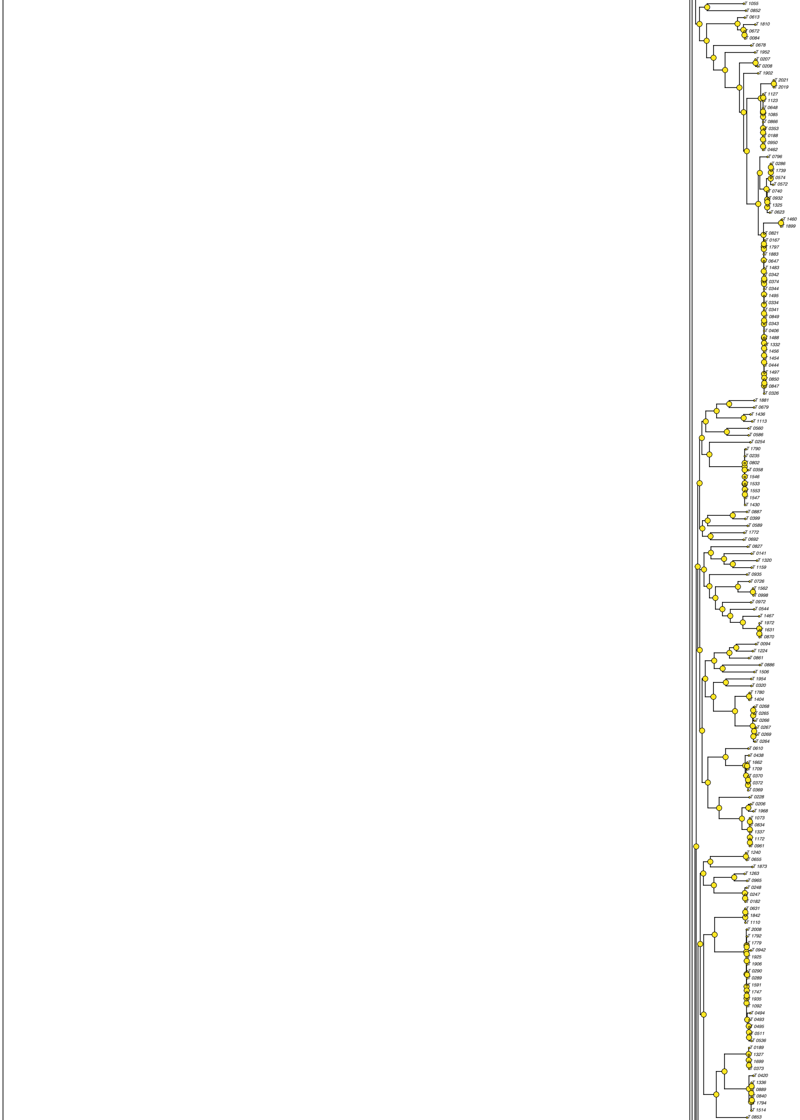
T 1055
T 0852
T 0613
T 1810
T 0672
T 0084
T 0678
T 1952
T 0207
T 0208
T 1902
T 2021
T 2019
T 1127
T 1123
T 0648
T 1085
T 0866
T 0353
T 0188
T 0950
T 0462
T 0796
T 0286
T 1739
T 0574
T 0572
T 0740
T 0932
T 1325
T 0623
T 1460
T 1899
T 0821
T 0167
T 1797
T 1883
T 0647
T 1483
T 0342
T 0374
T 0344
T 1495
T 0334
T 0341
T 0849
T 0343
T 0406
T 1488
T 1332
T 1456
T 1454
T 0444
T 1497
T 0850
T 0847
T 0326
T 1881
T 0679
T 1436
T 1113
T 0560
T 0586
T 0254
T 1790
T 0235
T 0802
T 0358
T 1546
T 1533
T 1553
T 1547
T 1430
T 0887
T 0399
T 0589
T 1772
T 0692
T 0827
T 0141
T 1320
T 1159
T 0935
T 0726
T 1562
T 0998
T 0972
T 0544
T 1467
T 1972
T 1631
T 0870
T 0094
T 1224
T 0861
T 0886
T 1506
T 1954
T 0320
T 1780
T 1404
T 0268
T 0265
T 0266
T 0267
T 0269
T 0264
T 0610
T 0438
T 1662
T 1709
T 0370
T 0372
T 0369
T 0228
T 0206
T 1968
T 1073
T 0834
T 1337
T 1172
T 0961
T 1240
T 0655
T 1873
T 1263
T 0965
T 0248
T 0247
T 0182
T 0631
T 1842
T 1110
T 2008
T 1792
T 1779
T 0942
T 1925
T 1906
T 0290
T 0289
T 1591
T 1747
T 1935
T 1092
T 0494
T 0493
T 0495
T 0511
T 0536
T 0189
T 1327
T 1699
T 0373
T 0420
T 1336
T 0889
T 0840
T 1794
T 1514
T 0853

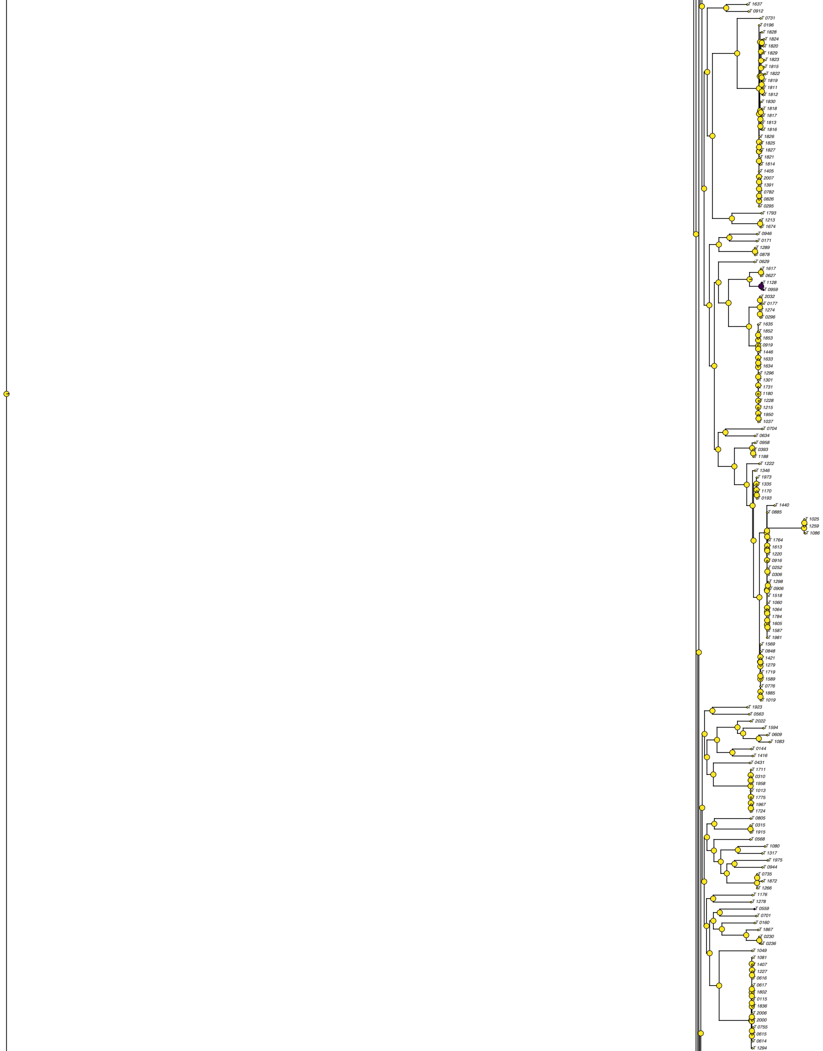
T 1637
T 0912
T 0731
T 0196
T 1828
T 1824
T 1820
T 1829
T 1823
T 1815
T 1822
T 1819
T 1811
T 1812
T 1830
T 1818
T 1817
T 1813
T 1816
T 1826
T 1825
T 1827
T 1821
T 1814
T 1405
T 2007
T 1391
T 0782
T 0826
T 0295
T 1793
T 1213
T 1674
T 0946
T 0171
T 1289
T 0878
T 0629
T 1617
T 0627
T 1128
T 0959
T 2032
T 0177
T 1274
T 0296
T 1635
T 1852
T 1853
T 0919
T 1446
T 1633
T 1634
T 1296
T 1301
T 1731
T 1180
T 1228
T 1215
T 1950
T 1037
T 0704
T 0634
T 0958
T 0393
T 1188
T 1222
T 1346
T 1973
T 1335
T 1170
T 0193
T 1440
T 0885
T 1025
T 1259
T 1086
T 1764
T 1613
T 1220
T 0916
T 0252
T 0306
T 1298
T 0906
T 1518
T 1060
T 1064
T 1784
T 1605
T 1587
T 1981
T 1569
T 0848
T 1421
T 1279
T 1719
T 1589
T 0776
T 1885
T 1019
T 1923
T 0563
T 2022
T 1594
T 0609
T 1083
T 0144
T 1416
T 0431
T 1711
T 0310
T 1958
T 1013
T 1775
T 1967
T 1724
T 0805
T 0315
T 1915
T 0568
T 1080
T 1317
T 1975
T 0944
T 0735
T 1872
T 1266
T 1176
T 1278
T 0559
T 0701
T 0160
T 1867
T 0230
T 0236
T 1049
T 1081
T 1407
T 1227
T 0616
T 0617
T 1802
T 0115
T 1836
T 2006
T 2000
T 0755
T 0615
T 0614
T 1294

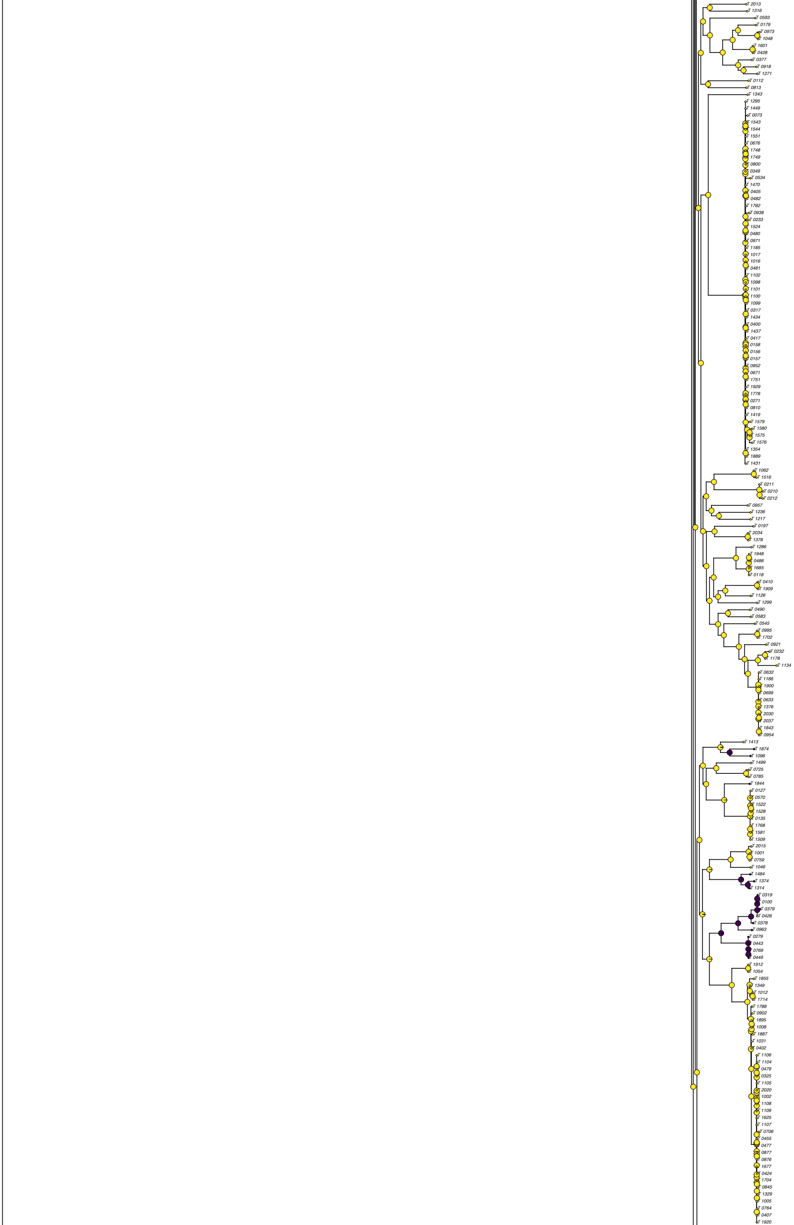
T 2013
T 1316
T 0593
T 0179
T 0973
T 1048
T 1601
T 0428
T 0377
T 0918
T 1271
T 0112
T 0813
T 1343
T 1295
T 1449
T 0073
T 1543
1544
T 1551
T 0676
T 1748
T 1749
0800
0349
T 0534
T 1470
0405
0482
T 1782
T 0938
T 0233
T 1524
0480
T 0971
T 1185
1017
1016
T 0481
T 1102
1098
T 1101
1100
T 1099
T 0317
T 1434
T 0400
T 1437
T 0417
0158
0156
0157
T 0952
0671
T 1751
T 1929
1778
0271
0810
T 1419
T 1579
T 1580
T 1575
T 1576
T 1354
T 1889
T 1431
T 1062
T 1516
T 0211
T 0210
T 0212
T 0957
T 1236
T 1217
T 0197
T 2034
T 1378
T 1286
T 1948
0486
1685
T 0118
T 0410
T 1909
T 1126
T 1299
T 0490
T 0583
T 0545
T 0995
T 1702
T 0921
T 0232
T 1178
T 1134
T 0632
T 1186
T 1900
T 0699
T 0633
T 1376
T 2030
T 2037
T 1843
T 0954
T 1413
T 1874
T 1096
T 1499
T 0725
T 0785
T 1844
T 0127
0570
T 1522
T 1528
T 0135
T 1768
1581
T 1509
T 2015
T 1001
T 0759
T 1046
T 1484
T 1374
T 1314
T 0319
0100
T 0379
T 0426
T 0378
T 0963
T 0279
0443
0769
T 0449
T 1912
T 1054
T 1855
1349
T 1012
T 1714
T 1788
T 0902
1895
1008
T 1887
T 1031
0402
T 1106
T 1104
0479
0325
T 1105
2020
1002
1108
1109
T 1625
T 1107
T 0706
0455
0477
0877
0876
1677
0424
1704
0845
1329
1005
T 0764
0407
T 1920

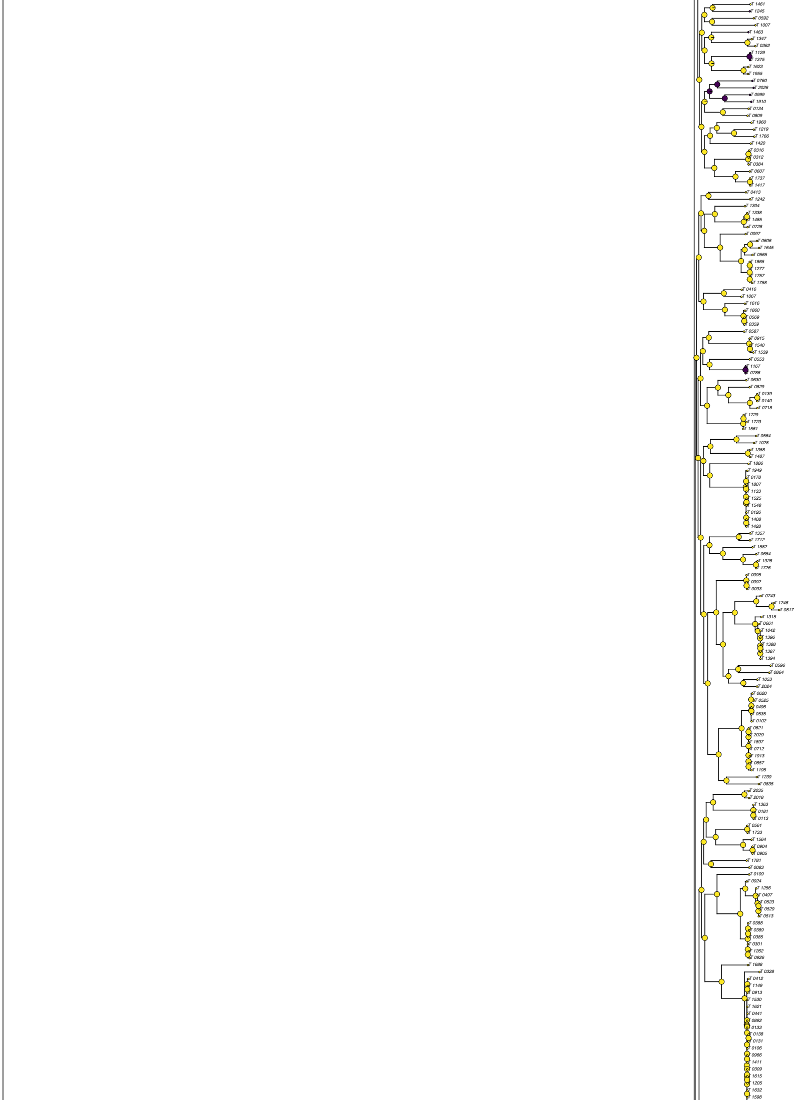
T 1461
T 1245
T 0592
T 1007
T 1463
T 1347
T 0362
T 1129
T 1375
T 1623
T 1955
T 0760
T 2026
T 0999
T 1910
T 0134
T 0809
T 1960
T 1219
T 1766
T 1420
T 0316
T 0312
T 0384
T 0607
T 1737
T 1417
T 0413
T 1242
T 1304
T 1338
T 1485
T 0728
T 0097
T 0606
T 1645
T 0565
T 1865
T 1277
T 1757
T 1758
T 0416
T 1067
T 1616
T 1860
T 0569
T 0359
T 0587
T 0915
T 1540
T 1539
T 0553
T 1167
T 0786
T 0630
T 0829
T 0139
T 0140
T 0718
T 1729
T 1723
T 1561
T 0564
T 1028
T 1358
T 1487
T 1886
T 1949
T 0178
T 1807
T 1133
T 1525
T 1548
T 0126
T 1408
T 1428
T 1357
T 1712
T 1582
T 0654
T 1926
T 1726
T 0095
T 0092
T 0093
T 0743
T 1246
T 0817
T 1315
T 0661
T 1042
T 1396
T 1388
T 1387
T 1394
T 0596
T 0864
T 1053
T 2024
T 0620
T 0525
T 0496
T 0535
T 0102
T 0621
T 2029
T 1897
T 0712
T 1913
T 0657
T 1195
T 1239
T 0835
T 2035
T 2018
T 1363
T 0181
T 0113
T 0561
T 1733
T 1564
T 0904
T 0905
T 1781
T 0083
T 0109
T 0924
T 1256
T 0497
T 0523
T 0529
T 0513
T 0388
T 0389
T 0385
T 0301
T 1262
T 0926
T 1688
T 0328
T 0412
T 1149
T 0913
T 1530
T 1621
T 0441
T 0892
T 0133
T 0138
T 0131
T 0106
T 0966
T 1411
T 0309
T 1615
T 1205
T 1632
T 1598

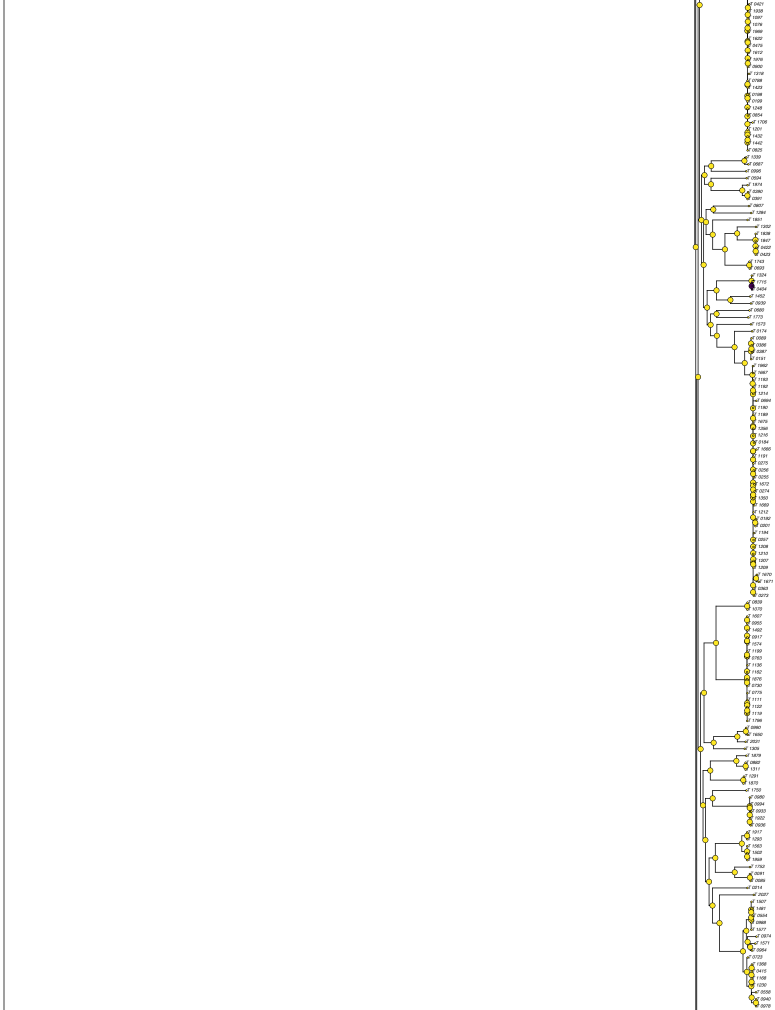
T 0421
T 1938
T 1097
T 1076
T 1969
T 1622
T 0475
T 1612
T 1976
T 0900
T 1318
T 0788
T 1423
T 0198
T 0199
T 1248
T 0854
T 1706
T 1201
T 1432
T 1442
T 0825
T 1339
T 0687
T 0996
T 0594
T 1974
T 0390
T 0391
T 0807
T 1284
T 1851
T 1302
T 1838
T 1847
T 0422
T 0423
T 1743
T 0693
T 1324
T 1715
T 0404
T 1452
T 0939
T 0680
T 1773
T 1573
T 0174
T 0089
T 0386
T 0387
T 0151
T 1962
T 1667
T 1193
T 1192
T 1214
T 0694
T 1190
T 1189
T 1675
T 1356
T 1216
T 0184
T 1666
T 1191
T 0275
T 0256
T 0255
T 1672
T 0274
T 1350
T 1669
T 1212
T 0192
T 0201
T 1194
T 0257
T 1208
T 1210
T 1207
T 1209
T 1670
T 1671
T 0363
T 0273
T 0839
T 1070
T 1607
T 0955
T 1492
T 0917
T 1574
T 1199
T 0763
T 1136
T 1162
T 1876
T 0730
T 0775
T 1111
T 1122
T 1119
T 1796
T 0990
T 1650
T 2031
T 1305
T 1879
T 0882
T 1311
T 1291
T 1870
T 1750
T 0980
T 0994
T 0933
T 1922
T 0936
T 1917
T 1293
T 1563
T 1502
T 1959
T 1753
T 0091
T 0085
T 0214
T 2027
T 1507
T 1481
T 0554
T 0988
T 1577
T 0974
T 1571
T 0964
T 0723
T 1368
T 0415
T 1168
T 1230
T 0558
T 0940
T 0978

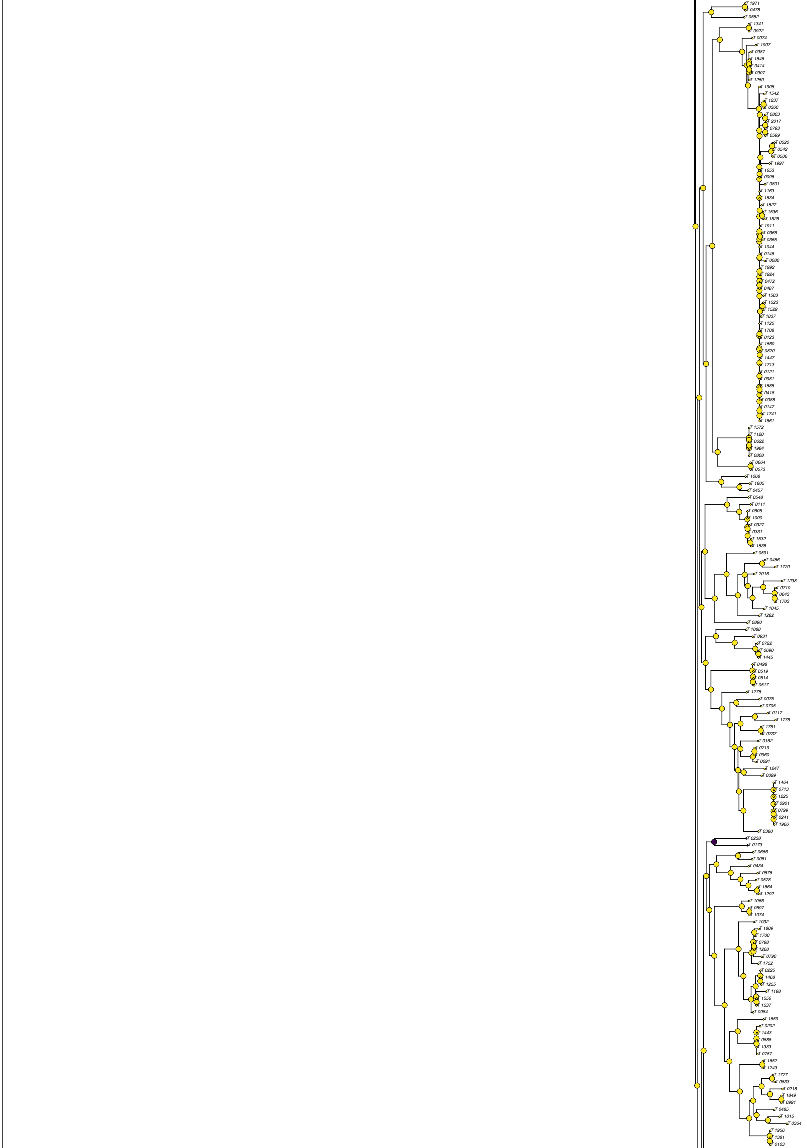
T 1971
T 0478
T 0582
T 1341
T 0922
T 0074
T 1907
T 0987
T 1846
T 0414
T 0907
T 1250
T 1905
T 1542
T 1237
T 0360
T 0803
T 2017
T 0793
T 0599
T 0520
T 0542
T 0506
T 1997
T 1653
T 0096
T 0801
T 1163
T 1534
T 1527
T 1536
T 1526
T 1911
T 0366
T 0365
T 1044
T 0146
T 0080
T 1992
T 1924
T 0472
T 0487
T 1503
T 1523
T 1529
T 1837
T 1125
T 1708
T 0123
T 1560
T 0820
T 1447
T 1713
T 0121
T 0981
T 1585
T 0418
T 0088
T 0147
T 1741
T 1861
T 1572
T 1120
T 0622
T 1984
T 0808
T 0664
T 0573
T 1068
T 1805
T 0457
T 0548
T 0111
T 0605
T 1000
T 0327
T 0331
T 1532
T 1538
T 0591
T 0456
T 1720
T 2016
T 1238
T 0710
T 0643
T 1703
T 1045
T 1282
T 0890
T 1088
T 0931
T 0722
T 0690
T 1445
T 0498
T 0519
T 0514
T 0517
T 1275
T 0075
T 0705
T 0117
T 1776
T 1761
T 0737
T 0162
T 0719
T 0960
T 0691
T 1247
T 0099
T 1464
T 0713
T 1225
T 0901
T 0799
T 0241
T 1966
T 0380
T 0238
T 0173
T 0656
T 0081
T 0434
T 0576
T 0578
T 1864
T 1292
T 1066
T 0597
T 1074
T 1032
T 1809
T 1700
T 0798
T 1268
T 0790
T 1752
T 0225
T 1468
T 1255
T 1198
T 1556
T 1537
T 0984
T 1659
T 0202
T 1443
T 0888
T 1333
T 0757
T 1652
T 1243
T 1777
T 0833
T 0218
T 1849
T 0991
T 0485
T 1015
T 0364
T 1956
T 1381
T 0103

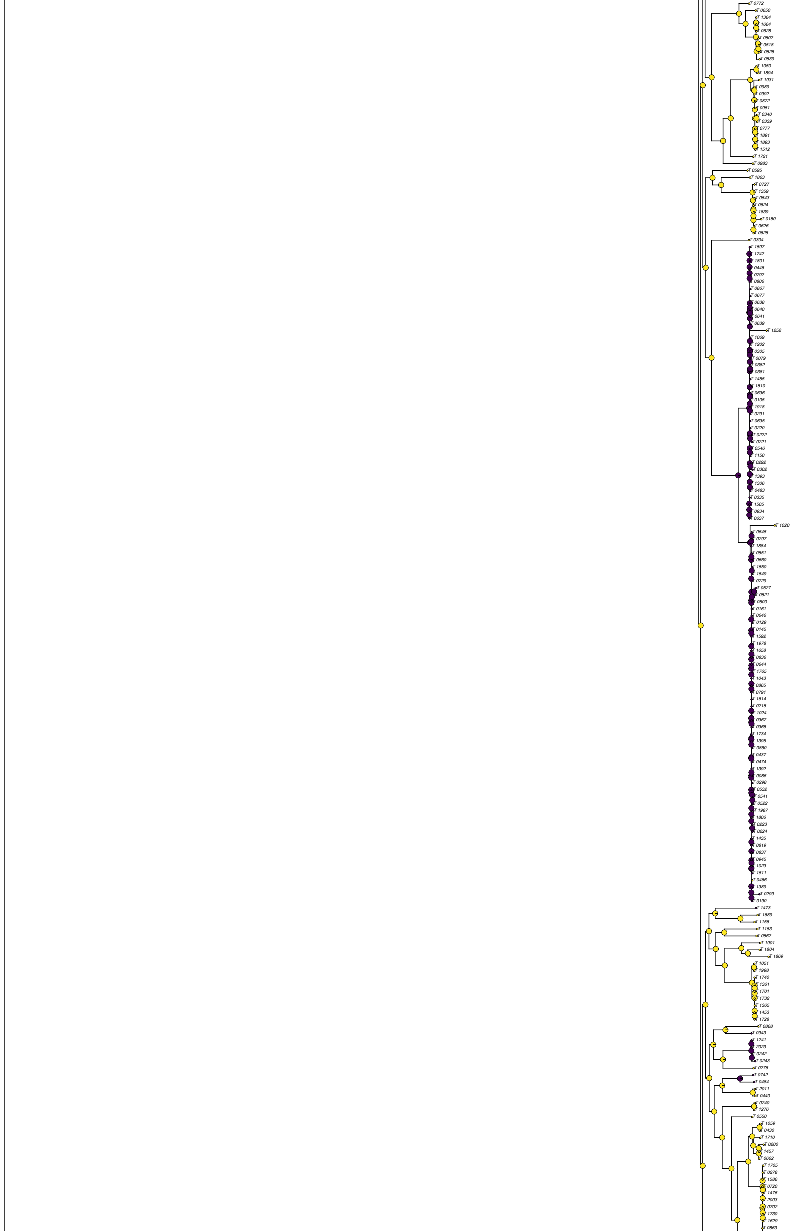
T 0772
T 0650
T 1364
T 1664
T 0628
T 0502
T 0518
T 0528
T 0539
T 1050
T 1894
T 1931
T 0989
T 0992
T 0872
T 0951
T 0340
T 0339
T 0777
T 1891
T 1893
T 1512
T 1721
T 0983
T 0595
T 1863
T 0727
T 1359
T 0543
T 0624
T 1839
T 0180
T 0626
T 0625
T 0304
T 1597
T 1742
T 1801
T 0446
T 0792
T 0806
T 0867
T 0677
T 0638
T 0640
T 0641
T 0639
T 1252
T 1069
T 1202
T 0305
T 0079
T 0382
T 0381
T 1455
T 1510
T 0636
T 0105
T 1918
T 0291
T 0635
T 0220
T 0222
T 0221
T 0546
T 1150
T 0292
T 0302
T 1393
T 1306
T 0483
T 0335
T 1505
T 0934
T 0637
T 1020
T 0645
T 0297
T 1884
T 0551
T 0660
T 1550
T 1549
T 0729
T 0527
T 0521
T 0500
T 0161
T 0646
T 0129
T 0145
T 1592
T 1978
T 1658
T 0836
T 0644
T 1765
T 1043
T 0865
T 0791
T 1614
T 0215
T 1024
T 0367
T 0368
T 1734
T 1395
T 0860
T 0437
T 0474
T 1392
T 0086
T 0298
T 0532
T 0541
T 0522
T 1987
T 1806
T 0223
T 0224
T 1435
T 0819
T 0837
T 0945
T 1023
T 1511
T 0466
T 1389
T 0299
T 0190
T 1473
T 1689
T 1156
T 1153
T 0562
T 1901
T 1804
T 1869
T 1051
T 1998
T 1740
T 1361
T 1701
T 1732
T 1365
T 1453
T 1728
T 0868
T 0943
T 1241
T 2023
T 0242
T 0243
T 0276
T 0742
T 0484
T 2011
T 0440
T 0240
T 1276
T 0550
T 1059
T 0430
T 1710
T 0200
T 1457
T 0662
T 1705
T 0278
T 1586
T 0720
T 1476
T 2003
T 0702
T 1730
T 1629
T 0863

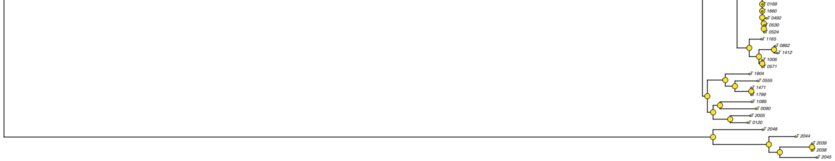
0169
1660
T 0492
T 0530
T 0524
T 1165
T 0862
T 1412
T 1006
T 0571
T 1904
T 0555
T 1471
T 1799
T 1089
T 0090
T 2005
T 0120
T 2048
T 2044
T 2039
T 2038
T 2045

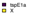

Effector gene: tspE1a

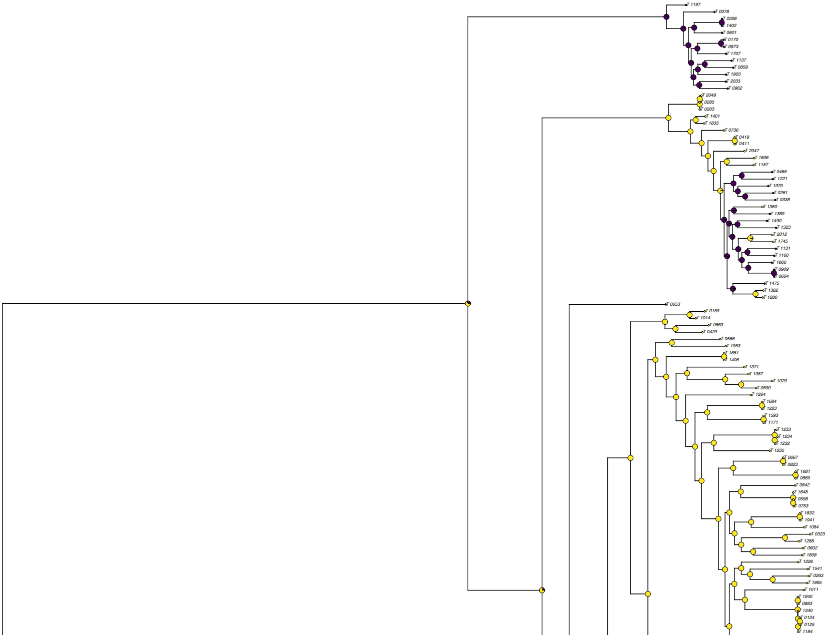

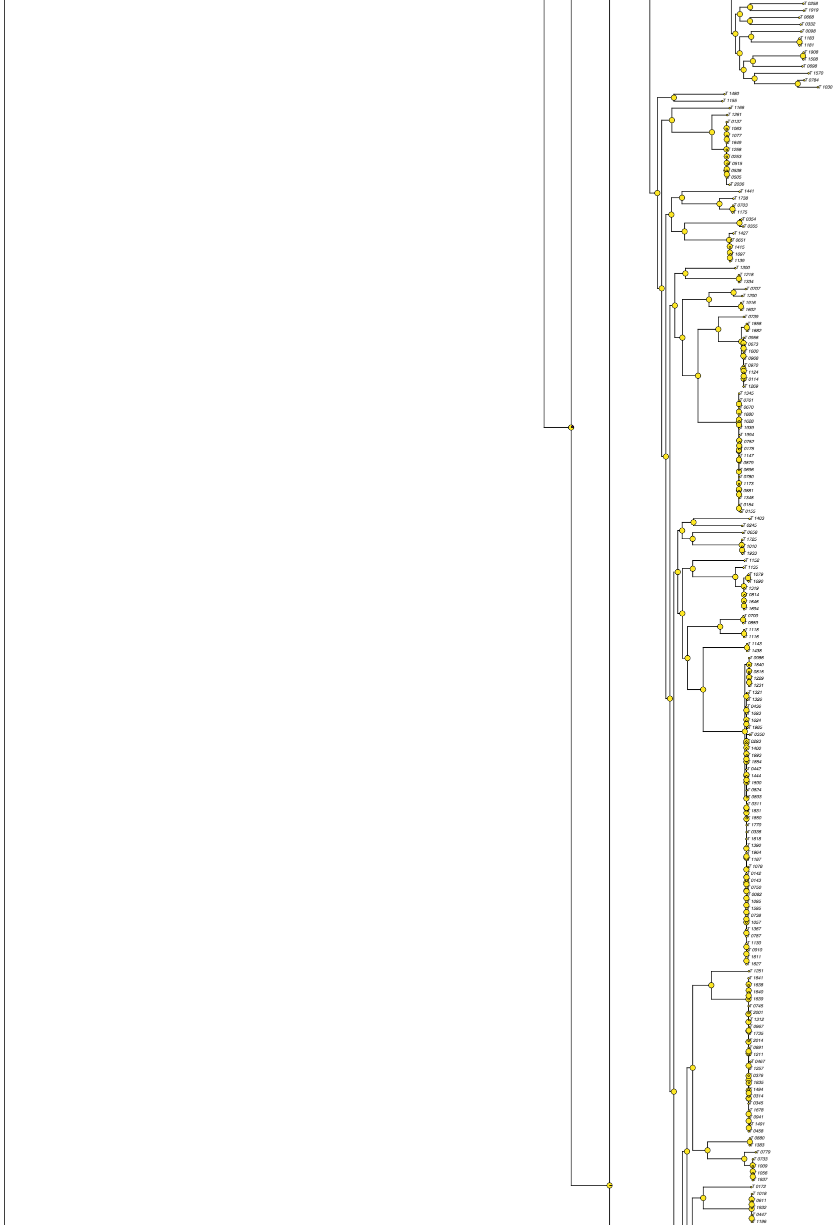
T 0258
T 1919
T 0668
T 0332
T 0098
T 1183
T 1181
T 1908
T 1508
T 0698
T 1570
T 0784
T 1030
T 1480
T 1155
T 1166
T 1261
T 0137
T 1063
T 1077
T 1649
T 1258
T 0253
T 0515
T 0538
T 0505
T 2036
T 1441
T 1738
T 0703
T 1175
T 0354
T 0355
T 1427
T 0651
T 1415
T 1697
T 1139
T 1300
T 1218
T 1334
T 0707
T 1200
T 1916
T 1602
T 0739
T 1858
T 1682
T 0956
T 0673
T 1600
T 0968
T 0970
T 1124
T 0114
T 1269
T 1345
T 0761
T 0670
T 1880
T 1628
T 1939
T 1994
T 0752
T 0175
T 1147
T 0879
T 0696
T 0780
T 1173
T 0881
T 1348
T 0154
T 0155
T 1403
T 0245
T 0658
T 1725
T 1010
T 1933
T 1152
T 1135
T 1079
T 1690
T 1319
T 0814
T 1646
T 1694
T 0700
T 0659
T 1118
T 1116
T 1143
T 1438
T 0986
T 1840
T 0815
T 1229
T 1231
T 1321
T 1326
T 0436
T 1693
T 1624
T 1985
T 0350
T 0293
T 1400
T 1993
T 1854
T 0442
T 1444
T 1590
T 0824
T 0893
T 0311
T 1831
T 1850
T 1770
T 0336
T 1618
T 1390
T 1964
T 1187
T 1078
T 0142
T 0143
T 0750
T 0082
T 1095
T 1595
T 0738
T 1057
T 1367
T 0787
T 1130
T 0910
T 1611
T 1627
T 1251
T 1641
T 1638
T 1640
T 1639
T 0745
T 2001
T 1312
T 0967
T 1735
T 2014
T 0891
T 1211
T 0467
T 1257
T 0376
T 1835
T 1494
T 0314
T 0345
T 1678
T 0941
T 1491
T 0458
T 0880
T 1383
T 0779
T 0733
T 1009
T 1056
T 1937
T 0172
T 1018
T 0611
T 1932
T 0447
T 1196

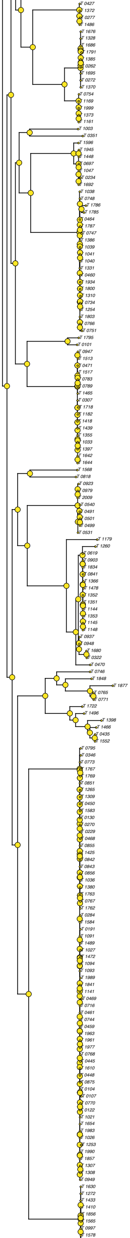
T 0427
T 1372
T 0277
T 1486
T 1676
T 1328
T 1686
T 1791
T 1385
T 0262
T 1695
T 0272
T 1370
T 0754
T 1169
T 1999
T 1373
T 1161
T 1003
T 0351
T 1596
T 1945
T 1448
T 0697
T 1047
T 0234
T 1692
T 1038
T 0748
T 1786
T 1785
T 0464
T 1787
T 0747
T 1386
T 1039
T 1041
T 1040
T 1331
T 0460
T 1934
T 1800
T 1310
T 0734
T 1254
T 1803
T 0766
T 0751
T 1795
T 0101
T 0947
T 1513
T 0471
T 1517
T 0783
T 0789
T 1465
T 0307
T 1718
T 1182
T 1418
T 1439
T 1355
T 1033
T 1397
T 1642
T 1644
T 1568
T 0818
T 0923
T 0979
T 2009
T 0540
T 0491
T 0501
T 0499
T 0531
T 1179
T 1260
T 0619
T 0903
T 1834
T 0841
T 1366
T 1478
T 1352
T 1351
T 1144
T 1353
T 1145
T 1148
T 0937
T 0948
T 1680
T 0322
T 0470
T 0746
T 1848
T 1877
T 0765
T 0771
T 1722
T 1496
T 1398
T 1466
T 0435
T 1552
T 0795
T 0346
T 0773
T 1767
T 1769
T 0851
T 1265
T 1309
T 0450
T 1583
T 0130
T 0270
T 0229
T 0468
T 0855
T 1425
T 0842
T 0843
T 0856
T 1036
T 1380
T 1763
T 0767
T 1762
T 0284
T 1584
T 0191
T 1091
T 1489
T 1027
T 1472
T 1094
T 1093
T 1989
T 1841
T 1141
T 0469
T 0716
T 0461
T 0744
T 0459
T 1963
T 1961
T 1977
T 0768
T 0445
T 1610
T 0448
T 0875
T 0104
T 0107
T 0770
T 0122
T 1021
T 1654
T 1983
T 1026
T 1253
T 1990
T 1857
T 1307
T 1308
T 0949
T 1630
T 1272
T 1433
T 1410
T 1856
T 1565
T 0997
T 1578

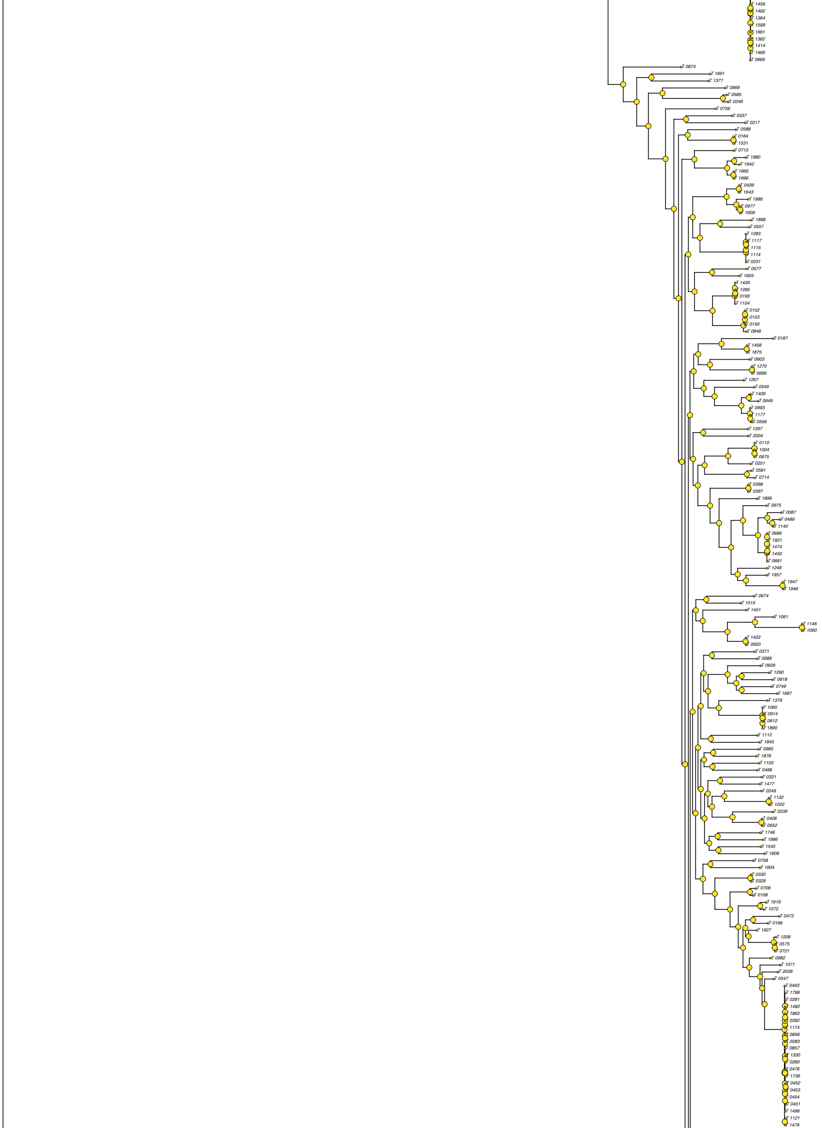
T 1459
T 1462
T 1384
T 1599
T 1661
T 1362
T 1414
T 1469
T 0669
T 0874
T 1691
T 1377
T 0969
T 0585
T 0246
T 0756
T 0337
T 0217
T 0588
T 0164
T 1531
T 0715
T 1980
T 1942
T 1965
T 1696
T 0439
T 1643
T 1986
T 0977
T 1609
T 1898
T 0557
T 1283
T 1117
T 1115
T 1114
T 0231
T 0577
T 1655
T 1426
T 1285
T 0195
T 1154
T 0152
T 0153
T 0150
T 0846
T 0187
T 1458
T 1875
T 0603
T 1270
T 0666
T 1267
T 0549
T 1409
T 0649
T 0993
T 1177
T 0556
T 1297
T 2004
T 0110
T 1004
T 0675
T 0251
T 0581
T 0714
T 0398
T 0397
T 1896
T 0975
T 0087
T 0489
T 1140
T 0686
T 1921
T 1474
T 1450
T 0681
T 1249
T 1957
T 1947
T 1946
T 0674
T 1515
T 1451
T 1061
T 1146
T 1090
T 1422
T 0920
T 0371
T 0688
T 0929
T 1290
T 0618
T 0749
T 1687
T 1379
T 1065
T 0914
T 0612
T 1890
T 1112
T 1845
T 0985
T 1878
T 1103
T 0488
T 0321
T 1477
T 0249
T 1132
T 1022
T 0239
T 0408
T 0652
T 1746
T 1996
T 1545
T 1608
T 0758
T 1604
T 0330
T 0329
T 0708
T 0108
T 1619
T 1072
T 0473
T 0166
T 1927
T 1206
T 0575
T 0721
T 0982
T 1071
T 2028
T 0547
T 0463
T 1798
T 0281
T 1482
T 1862
T 0282
T 1174
T 0858
T 0283
T 0857
T 1330
T 0280
T 0476
T 1736
T 0452
T 0453
T 0454
T 0451
T 1498
T 1121
T 1479

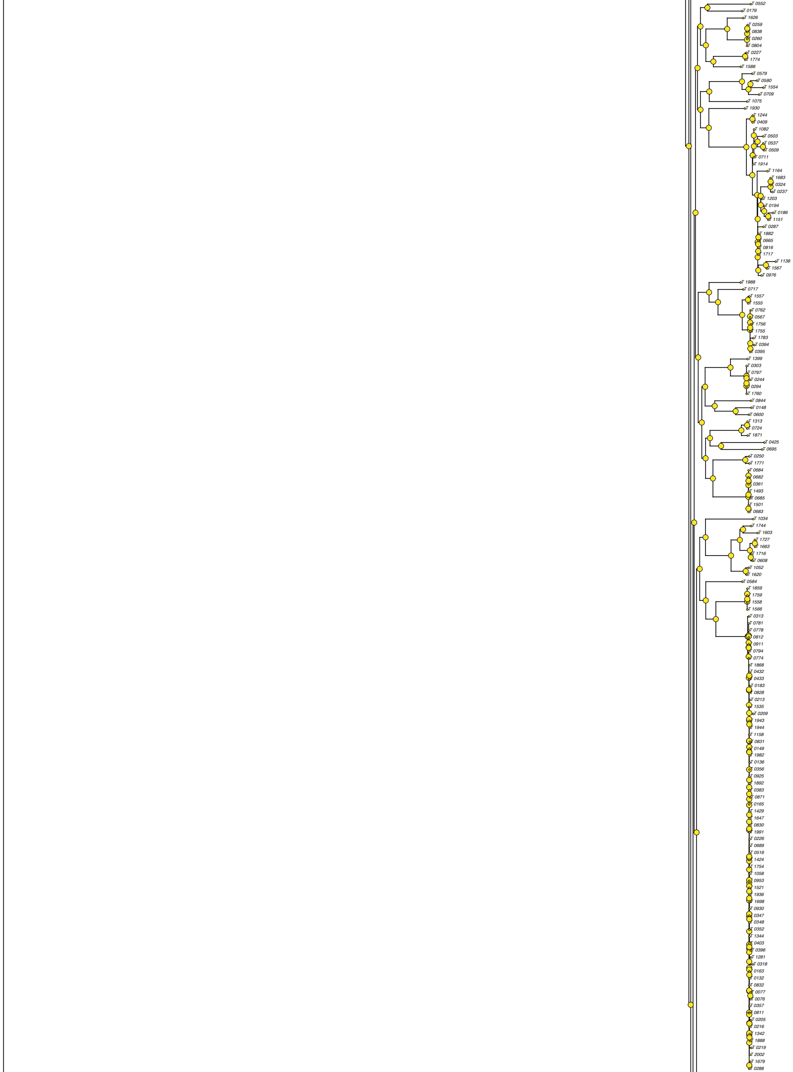
T 0552
T 0176
T 1626
T 0259
0838
0260
T 0804
T 0227
1774
T 1588
T 0579
T 0580
T 1554
T 0709
T 1075
T 1930
T 1244
T 0409
T 1082
T 0503
T 0537
T 0509
T 0711
T 1914
T 1164
T 1683
0324
T 0237
T 1203
T 0194
T 0186
1151
T 0287
T 1882
0665
T 0816
1717
T 1138
1567
T 0976
T 1988
T 0717
T 1557
1555
T 0762
0567
1756
1755
T 1783
T 0394
0395
T 1399
T 0303
0797
T 0244
0294
T 1760
T 0844
T 0148
T 0600
T 1313
0724
T 1871
T 0425
T 0695
T 0250
T 1771
T 0684
0682
0361
T 1493
T 0685
T 1501
0683
T 1034
T 1744
T 1603
T 1727
1663
T 1716
T 0608
T 1052
1620
T 0584
T 1859
1759
1558
1566
T 0313
T 0781
T 0778
0812
0911
0794
0774
T 1868
T 0432
0433
T 0183
0828
T 0213
1535
T 0209
1943
1944
T 1158
0831
0149
1982
T 0136
0356
T 0925
1892
0383
T 0871
0165
T 1429
1647
0830
1991
T 0226
T 0689
T 0516
1424
T 1754
T 1058
0953
1521
1936
1698
T 0930
0347
0348
T 0352
T 1344
T 0403
T 0396
T 1281
T 0318
0163
0132
T 0832
T 0077
T 0076
T 0357
0811
T 0205
T 0216
1342
1888
T 0219
T 2002
T 1679
0288

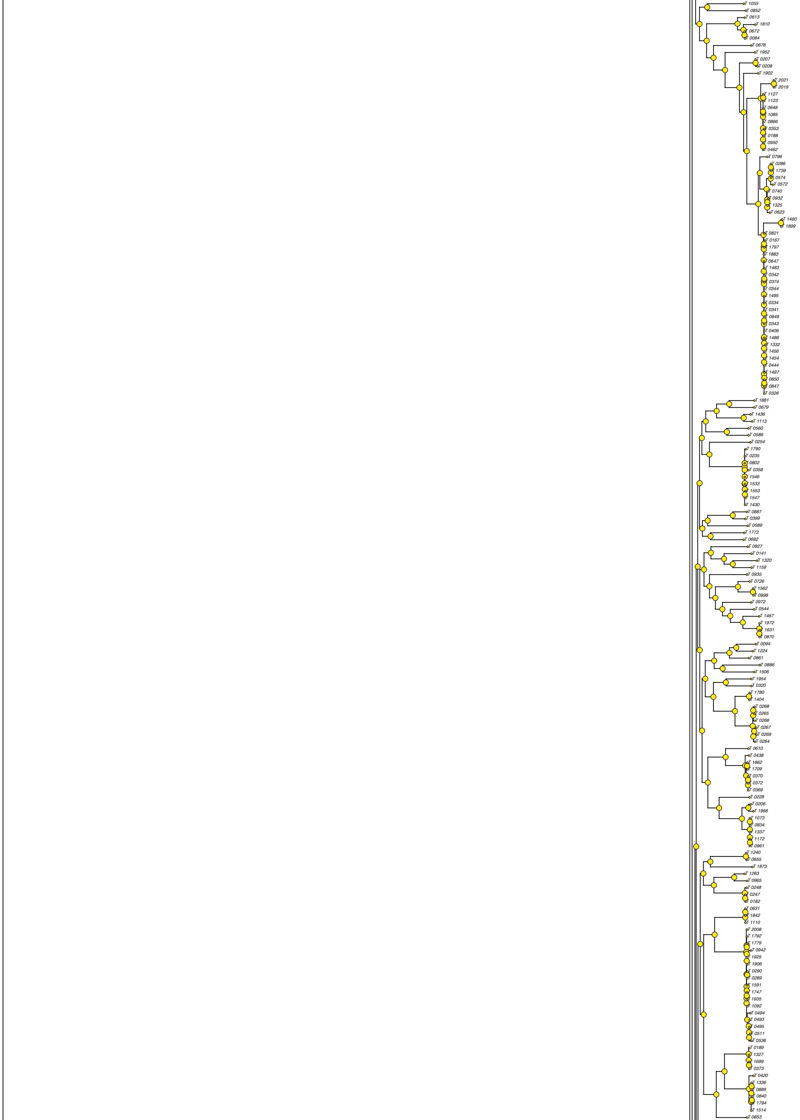
T 1055
T 0852
T 0613
T 1810
T 0672
T 0084
T 0678
T 1952
T 0207
T 0208
T 1902
T 2021
T 2019
T 1127
T 1123
T 0648
T 1085
T 0866
T 0353
T 0188
T 0950
T 0462
T 0796
T 0286
T 1739
T 0574
T 0572
T 0740
T 0932
T 1325
T 0623
T 1460
T 1899
T 0821
T 0167
T 1797
T 1883
T 0647
T 1483
T 0342
T 0374
T 0344
T 1495
T 0334
T 0341
T 0849
T 0343
T 0406
T 1488
T 1332
T 1456
T 1454
T 0444
T 1497
T 0850
T 0847
T 0326
T 1881
T 0679
T 1436
T 1113
T 0560
T 0586
T 0254
T 1790
T 0235
T 0802
T 0358
T 1546
T 1533
T 1553
T 1547
T 1430
T 0887
T 0399
T 0589
T 1772
T 0692
T 0827
T 0141
T 1320
T 1159
T 0935
T 0726
T 1562
T 0998
T 0972
T 0544
T 1467
T 1972
T 1631
T 0870
T 0094
T 1224
T 0861
T 0886
T 1506
T 1954
T 0320
T 1780
T 1404
T 0268
T 0265
T 0266
T 0267
T 0269
T 0264
T 0610
T 0438
T 1662
T 1709
T 0370
T 0372
T 0369
T 0228
T 0206
T 1968
T 1073
T 0834
T 1337
T 1172
T 0961
T 1240
T 0655
T 1873
T 1263
T 0965
T 0248
T 0247
T 0182
T 0631
T 1842
T 1110
T 2008
T 1792
T 1779
T 0942
T 1925
T 1906
T 0290
T 0289
T 1591
T 1747
T 1935
T 1092
T 0494
T 0493
T 0495
T 0511
T 0536
T 0189
T 1327
T 1699
T 0373
T 0420
T 1336
T 0889
T 0840
T 1794
T 1514
T 0853

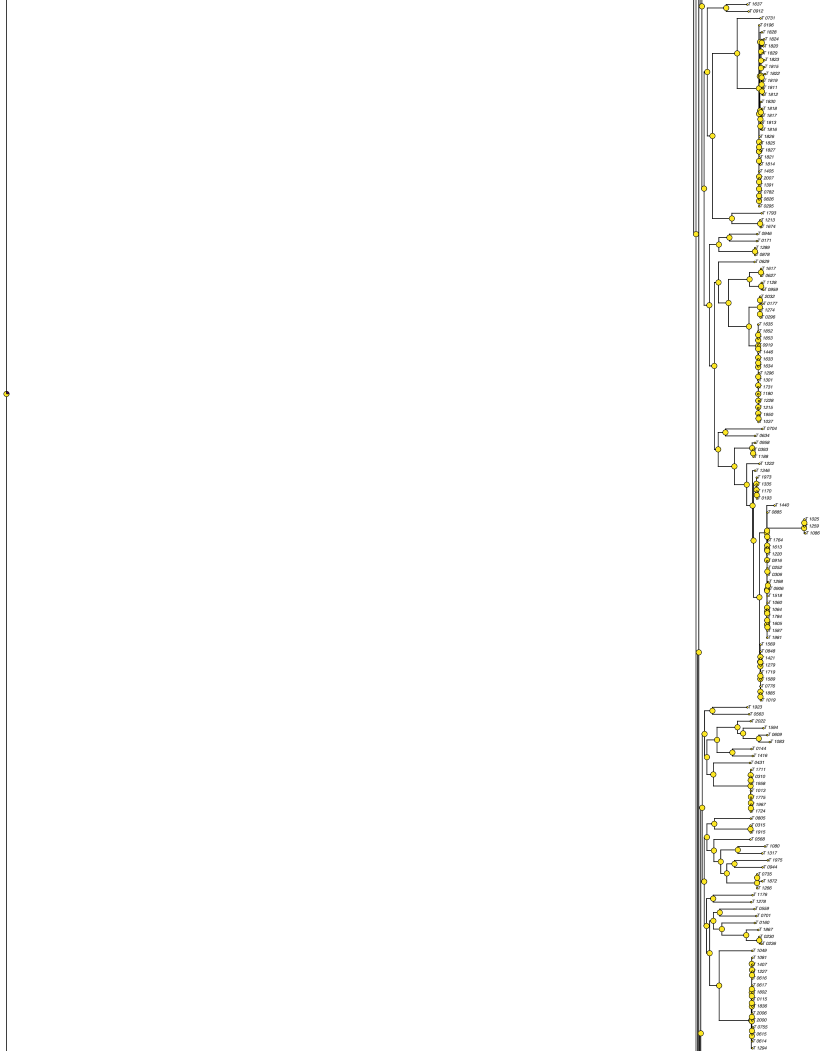
T 1637
T 0912
T 0731
T 0196
T 1828
T 1824
T 1820
T 1829
T 1823
T 1815
T 1822
T 1819
T 1811
T 1812
T 1830
T 1818
T 1817
T 1813
T 1816
T 1826
T 1825
T 1827
T 1821
T 1814
T 1405
T 2007
T 1391
T 0782
T 0826
T 0295
T 1793
T 1213
T 1674
T 0946
T 0171
T 1289
T 0878
T 0629
T 1617
T 0627
T 1128
T 0959
T 2032
T 0177
T 1274
T 0296
T 1635
T 1852
T 1853
T 0919
T 1446
T 1633
T 1634
T 1296
T 1301
T 1731
T 1180
T 1228
T 1215
T 1950
T 1037
T 0704
T 0634
T 0958
T 0393
T 1188
T 1222
T 1346
T 1973
T 1335
T 1170
T 0193
T 1440
T 0885
T 1025
T 1259
T 1086
T 1764
T 1613
T 1220
T 0916
T 0252
T 0306
T 1298
T 0906
T 1518
T 1060
T 1064
T 1784
T 1605
T 1587
T 1981
T 1569
T 0848
T 1421
T 1279
T 1719
T 1589
T 0776
T 1885
T 1019
T 1923
T 0563
T 2022
T 1594
T 0609
T 1083
T 0144
T 1416
T 0431
T 1711
T 0310
T 1958
T 1013
T 1775
T 1967
T 1724
T 0805
T 0315
T 1915
T 0568
T 1080
T 1317
T 1975
T 0944
T 0735
T 1872
T 1266
T 1176
T 1278
T 0559
T 0701
T 0160
T 1867
T 0230
T 0236
T 1049
T 1081
T 1407
T 1227
T 0616
T 0617
T 1802
T 0115
T 1836
T 2006
T 2000
T 0755
T 0615
T 0614
T 1294

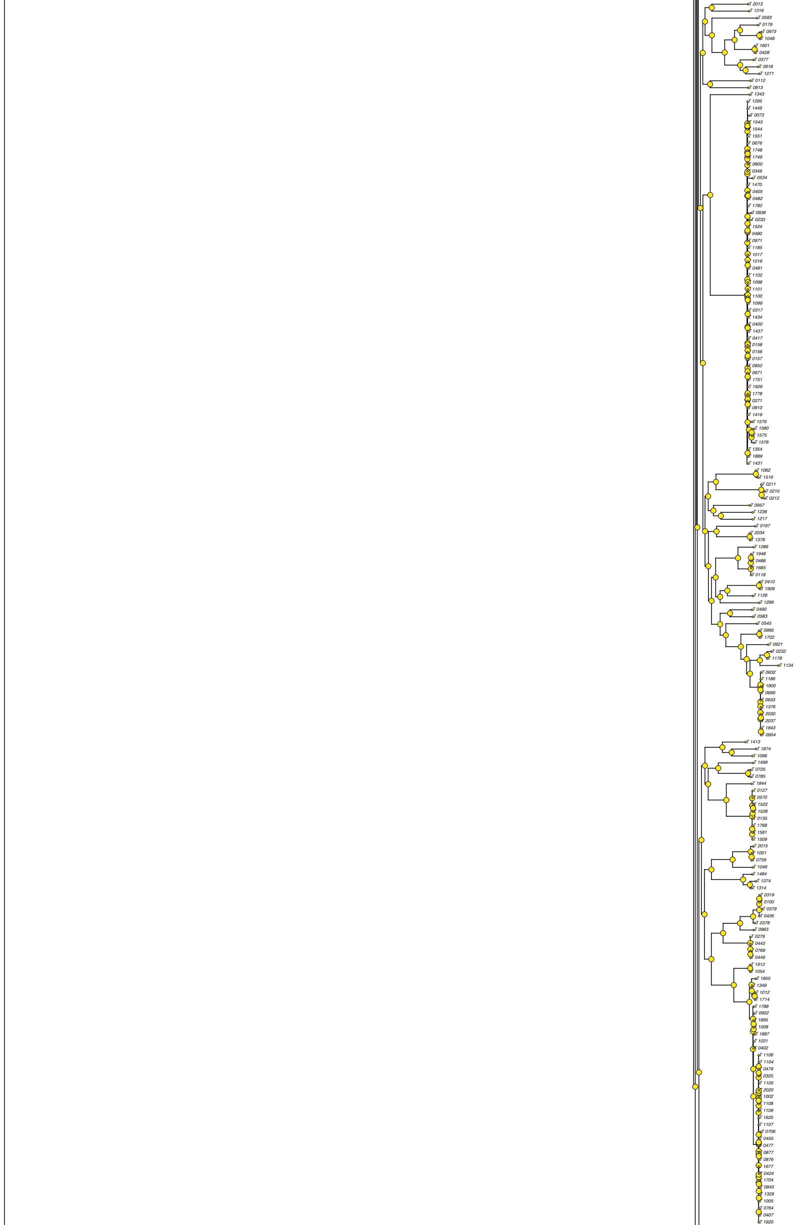
T 2013
T 1316
T 0593
T 0179
T 0973
T 1048
T 1601
T 0428
T 0377
T 0918
T 1271
T 0112
T 0813
T 1343
T 1295
T 1449
T 0073
T 1543
T 1544
T 1551
T 0676
T 1748
T 1749
T 0800
T 0349
T 0534
T 1470
T 0405
T 0482
T 1782
T 0938
T 0233
T 1524
T 0480
T 0971
T 1185
T 1017
T 1016
T 0481
T 1102
T 1098
T 1101
T 1100
T 1099
T 0317
T 1434
T 0400
T 1437
T 0417
T 0158
T 0156
T 0157
T 0952
T 0671
T 1751
T 1929
T 1778
T 0271
T 0810
T 1419
T 1579
T 1580
T 1575
T 1576
T 1354
T 1889
T 1431
T 1062
T 1516
T 0211
T 0210
T 0212
T 0957
T 1236
T 1217
T 0197
T 2034
T 1378
T 1286
T 1948
T 0486
T 1685
T 0118
T 0410
T 1909
T 1126
T 1299
T 0490
T 0583
T 0545
T 0995
T 1702
T 0921
T 0232
T 1178
T 1134
T 0632
T 1186
T 1900
T 0699
T 0633
T 1376
T 2030
T 2037
T 1843
T 0954
T 1413
T 1874
T 1096
T 1499
T 0725
T 0785
T 1844
T 0127
T 0570
T 1522
T 1528
T 0135
T 1768
T 1581
T 1509
T 2015
T 1001
T 0759
T 1046
T 1484
T 1374
T 1314
T 0319
T 0100
T 0379
T 0426
T 0378
T 0963
T 0279
T 0443
T 0769
T 0449
T 1912
T 1054
T 1855
T 1349
T 1012
T 1714
T 1788
T 0902
T 1895
T 1008
T 1887
T 1031
T 0402
T 1106
T 1104
T 0479
T 0325
T 1105
T 2020
T 1002
T 1108
T 1109
T 1625
T 1107
T 0706
T 0455
T 0477
T 0877
T 0876
T 1677
T 0424
T 1704
T 0845
T 1329
T 1005
T 0764
T 0407
T 1920

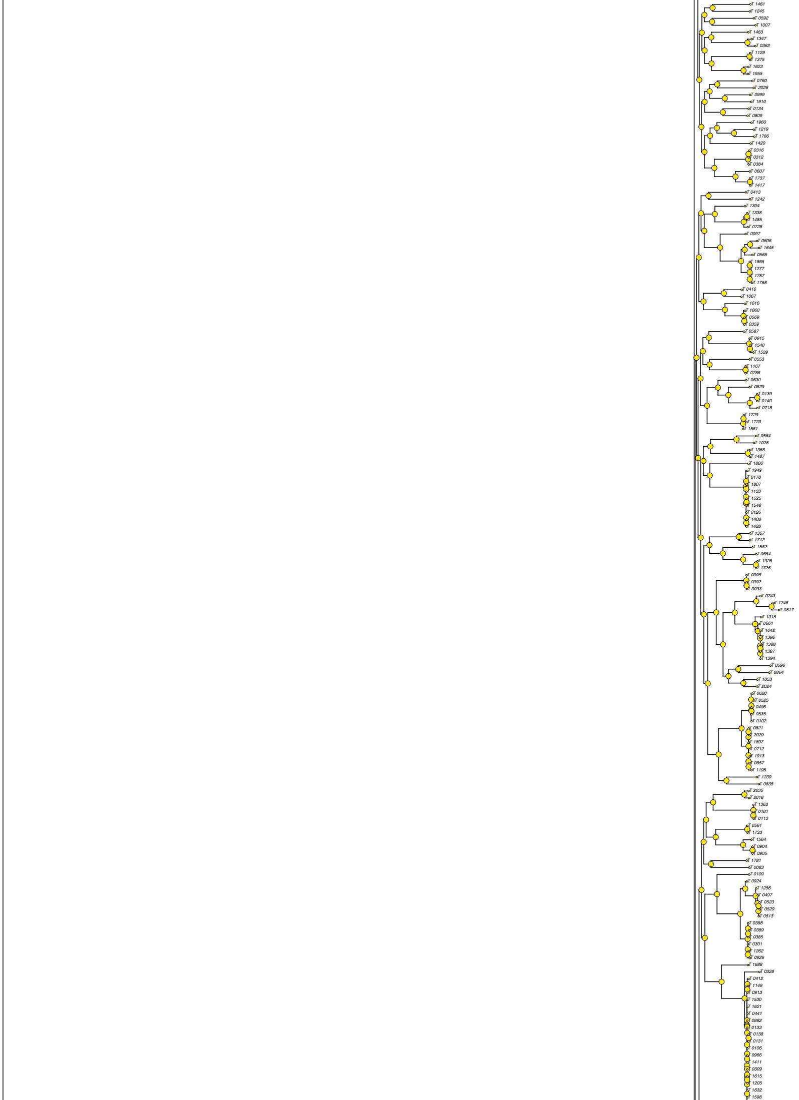

T 1461
T 1245
T 0592
T 1007
T 1463
T 1347
T 0362
T 1129
T 1375
T 1623
T 1955
T 0760
T 2026
T 0999
T 1910
T 0134
T 0809
T 1960
T 1219
T 1766
T 1420
T 0316
T 0312
T 0384
T 0607
T 1737
T 1417
T 0413
T 1242
T 1304
T 1338
T 1485
T 0728
T 0097
T 0606
T 1645
T 0565
T 1865
T 1277
T 1757
T 1758
T 0416
T 1067
T 1616
T 1860
T 0569
T 0359
T 0587
T 0915
T 1540
T 1539
T 0553
T 1167
T 0786
T 0630
T 0829
T 0139
T 0140
T 0718
T 1729
T 1723
T 1561
T 0564
T 1028
T 1358
T 1487
T 1886
T 1949
T 0178
T 1807
T 1133
T 1525
T 1548
T 0126
T 1408
T 1428
T 1357
T 1712
T 1582
T 0654
T 1926
T 1726
T 0095
T 0092
T 0093
T 0743
T 1246
T 0817
T 1315
T 0661
T 1042
T 1396
T 1388
T 1387
T 1394
T 0596
T 0864
T 1053
T 2024
T 0620
T 0525
T 0496
T 0535
T 0102
T 0621
T 2029
T 1897
T 0712
T 1913
T 0657
T 1195
T 1239
T 0835
T 2035
T 2018
T 1363
T 0181
T 0113
T 0561
T 1733
T 1564
T 0904
T 0905
T 1781
T 0083
T 0109
T 0924
T 1256
T 0497
T 0523
T 0529
T 0513
T 0388
T 0389
T 0385
T 0301
T 1262
T 0926
T 1688
T 0328
T 0412
T 1149
T 0913
T 1530
T 1621
T 0441
T 0892
T 0133
T 0138
T 0131
T 0106
T 0966
T 1411
T 0309
T 1615
T 1205
T 1632
T 1598

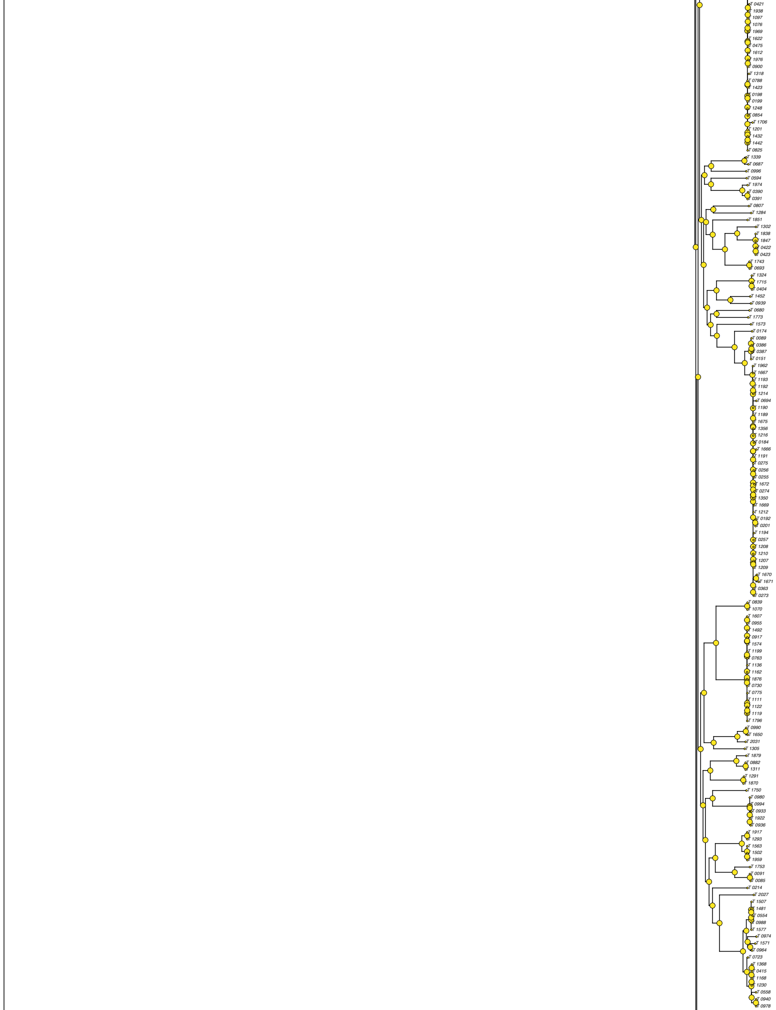
T 0421
T 1938
T 1097
T 1076
T 1969
T 1622
T 0475
T 1612
T 1976
T 0900
T 1318
T 0788
T 1423
T 0198
T 0199
T 1248
T 0854
T 1706
T 1201
T 1432
T 1442
T 0825
T 1339
T 0687
T 0996
T 0594
T 1974
T 0390
T 0391
T 0807
T 1284
T 1851
T 1302
T 1838
T 1847
T 0422
T 0423
T 1743
T 0693
T 1324
T 1715
T 0404
T 1452
T 0939
T 0680
T 1773
T 1573
T 0174
T 0089
T 0386
T 0387
T 0151
T 1962
T 1667
T 1193
T 1192
T 1214
T 0694
T 1190
T 1189
T 1675
T 1356
T 1216
T 0184
T 1666
T 1191
T 0275
T 0256
T 0255
T 1672
T 0274
T 1350
T 1669
T 1212
T 0192
T 0201
T 1194
T 0257
T 1208
T 1210
T 1207
T 1209
T 1670
T 1671
T 0363
T 0273
T 0839
T 1070
T 1607
T 0955
T 1492
T 0917
T 1574
T 1199
T 0763
T 1136
T 1162
T 1876
T 0730
T 0775
T 1111
T 1122
T 1119
T 1796
T 0990
T 1650
T 2031
T 1305
T 1879
T 0882
T 1311
T 1291
T 1870
T 1750
T 0980
T 0994
T 0933
T 1922
T 0936
T 1917
T 1293
T 1563
T 1502
T 1959
T 1753
T 0091
T 0085
T 0214
T 2027
T 1507
T 1481
T 0554
T 0988
T 1577
T 0974
T 1571
T 0964
T 0723
T 1368
T 0415
T 1168
T 1230
T 0558
T 0940
T 0978

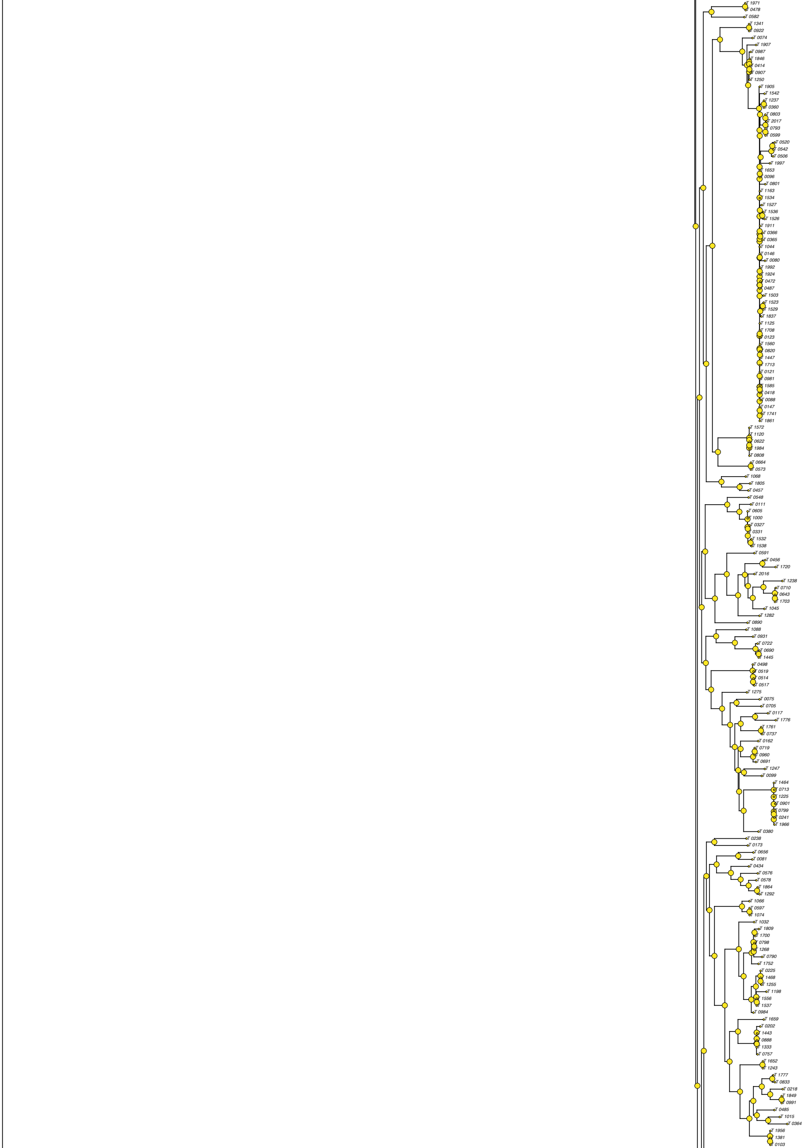
T 1971
T 0478
T 0582
T 1341
T 0922
T 0074
T 1907
T 0987
T 1846
T 0414
T 0907
T 1250
T 1905
T 1542
T 1237
T 0360
T 0803
T 2017
T 0793
T 0599
T 0520
T 0542
T 0506
T 1997
T 1653
0096
T 0801
T 1163
1534
T 1527
T 1536
T 1526
T 1911
T 0366
T 0365
T 1044
T 0146
T 0080
T 1992
T 1924
T 0472
T 0487
T 1503
T 1523
T 1529
T 1837
T 1125
T 1708
T 0123
T 1560
T 0820
T 1447
T 1713
T 0121
T 0981
T 1585
T 0418
T 0088
T 0147
T 1741
T 1861
T 1572
T 1120
T 0622
T 1984
T 0808
T 0664
T 0573
T 1068
T 1805
T 0457
T 0548
T 0111
T 0605
1000
T 0327
0331
T 1532
T 1538
T 0591
T 0456
T 1720
T 2016
T 1238
T 0710
0643
T 1703
T 1045
T 1282
T 0890
T 1088
T 0931
T 0722
T 0690
T 1445
T 0498
T 0519
T 0514
T 0517
T 1275
T 0075
T 0705
T 0117
T 1776
T 1761
T 0737
T 0162
T 0719
T 0960
T 0691
T 1247
T 0099
T 1464
T 0713
1225
T 0901
T 0799
T 0241
T 1966
T 0380
T 0238
T 0173
T 0656
T 0081
T 0434
T 0576
T 0578
T 1864
T 1292
T 1066
T 0597
T 1074
T 1032
T 1809
T 1700
T 0798
T 1268
T 0790
T 1752
T 0225
T 1468
T 1255
T 1198
T 1556
T 1537
T 0984
T 1659
T 0202
T 1443
T 0888
T 1333
T 0757
T 1652
T 1243
T 1777
T 0833
T 0218
T 1849
T 0991
T 0485
T 1015
T 0364
T 1956
T 1381
T 0103

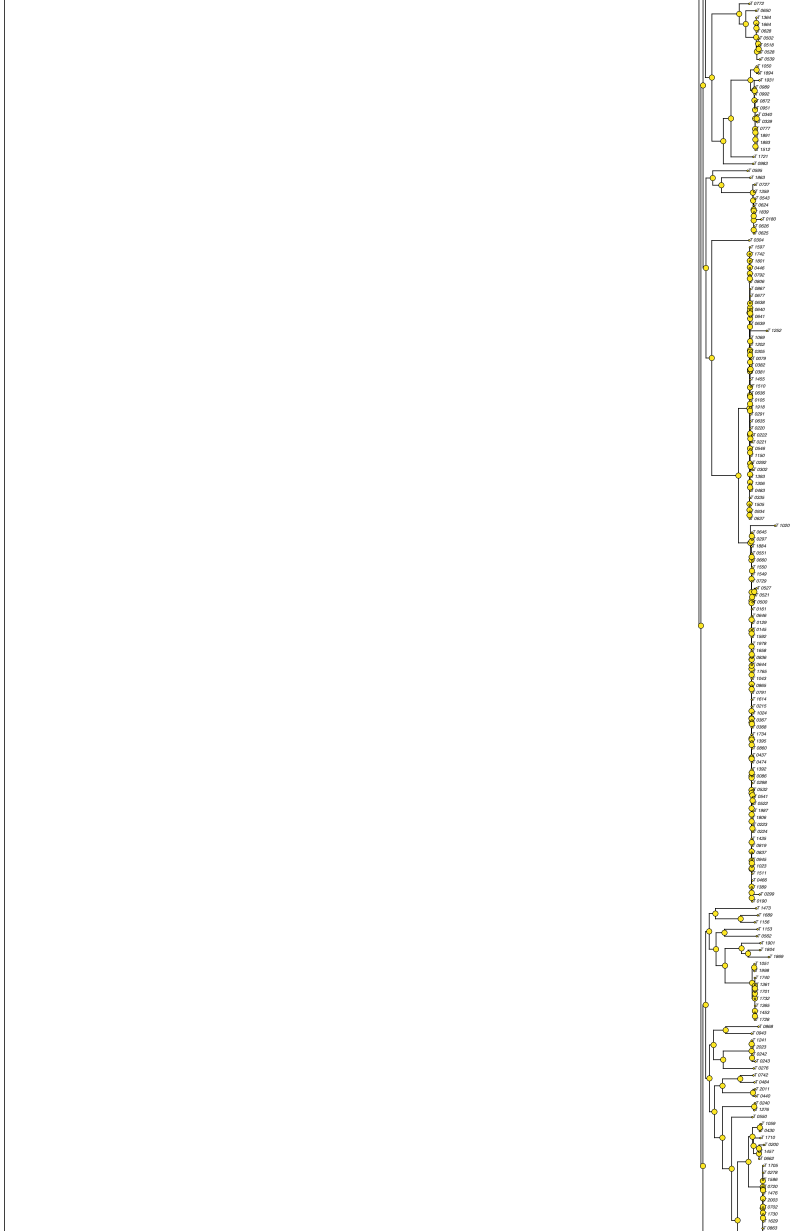
T 0772
T 0650
T 1364
T 1664
T 0628
T 0502
T 0518
T 0528
T 0539
T 1050
T 1894
T 1931
T 0989
T 0992
T 0872
T 0951
T 0340
T 0339
T 0777
T 1891
T 1893
T 1512
T 1721
T 0983
T 0595
T 1863
T 0727
T 1359
T 0543
T 0624
T 1839
T 0180
T 0626
T 0625
T 0304
T 1597
T 1742
T 1801
T 0446
T 0792
T 0806
T 0867
T 0677
T 0638
T 0640
T 0641
T 0639
T 1252
T 1069
T 1202
T 0305
T 0079
T 0382
T 0381
T 1455
T 1510
T 0636
T 0105
T 1918
T 0291
T 0635
T 0220
T 0222
T 0221
T 0546
T 1150
T 0292
T 0302
T 1393
T 1306
T 0483
T 0335
T 1505
T 0934
T 0637
T 1020
T 0645
T 0297
T 1884
T 0551
T 0660
T 1550
T 1549
T 0729
T 0527
T 0521
T 0500
T 0161
T 0646
T 0129
T 0145
T 1592
T 1978
T 1658
T 0836
T 0644
T 1765
T 1043
T 0865
T 0791
T 1614
T 0215
T 1024
T 0367
T 0368
T 1734
T 1395
T 0860
T 0437
T 0474
T 1392
T 0086
T 0298
T 0532
T 0541
T 0522
T 1987
T 1806
T 0223
T 0224
T 1435
T 0819
T 0837
T 0945
T 1023
T 1511
T 0466
T 1389
T 0299
T 0190
T 1473
T 1689
T 1156
T 1153
T 0562
T 1901
T 1804
T 1869
T 1051
T 1998
T 1740
T 1361
T 1701
T 1732
T 1365
T 1453
T 1728
T 0868
T 0943
T 1241
T 2023
T 0242
T 0243
T 0276
T 0742
T 0484
T 2011
T 0440
T 0240
T 1276
T 0550
T 1059
T 0430
T 1710
T 0200
T 1457
T 0662
T 1705
T 0278
T 1586
T 0720
T 1476
T 2003
T 0702
T 1730
T 1629
T 0863

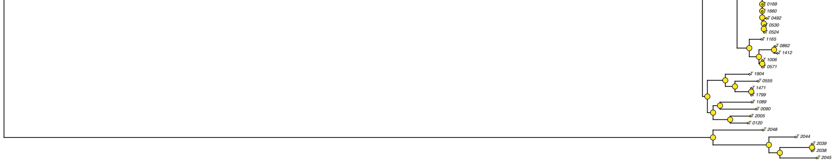
T 0169
T 1660
T 0492
T 0530
T 0524
T 1165
T 0862
T 1412
T 1006
T 0571
T 1904
T 0555
T 1471
T 1799
T 1089
T 0090
T 2005
T 0120
T 2048
T 2044
T 2039
T 2038
T 2045

Effector gene: tspE1b

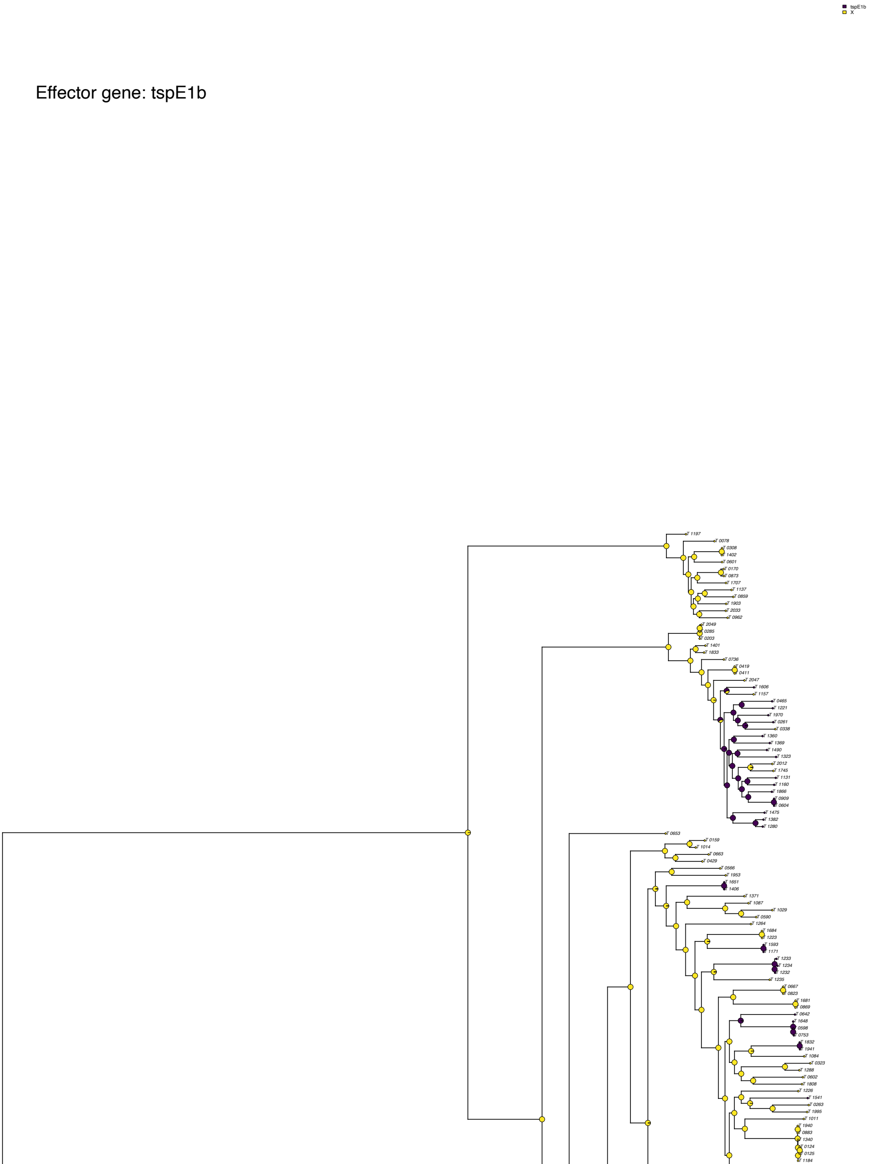

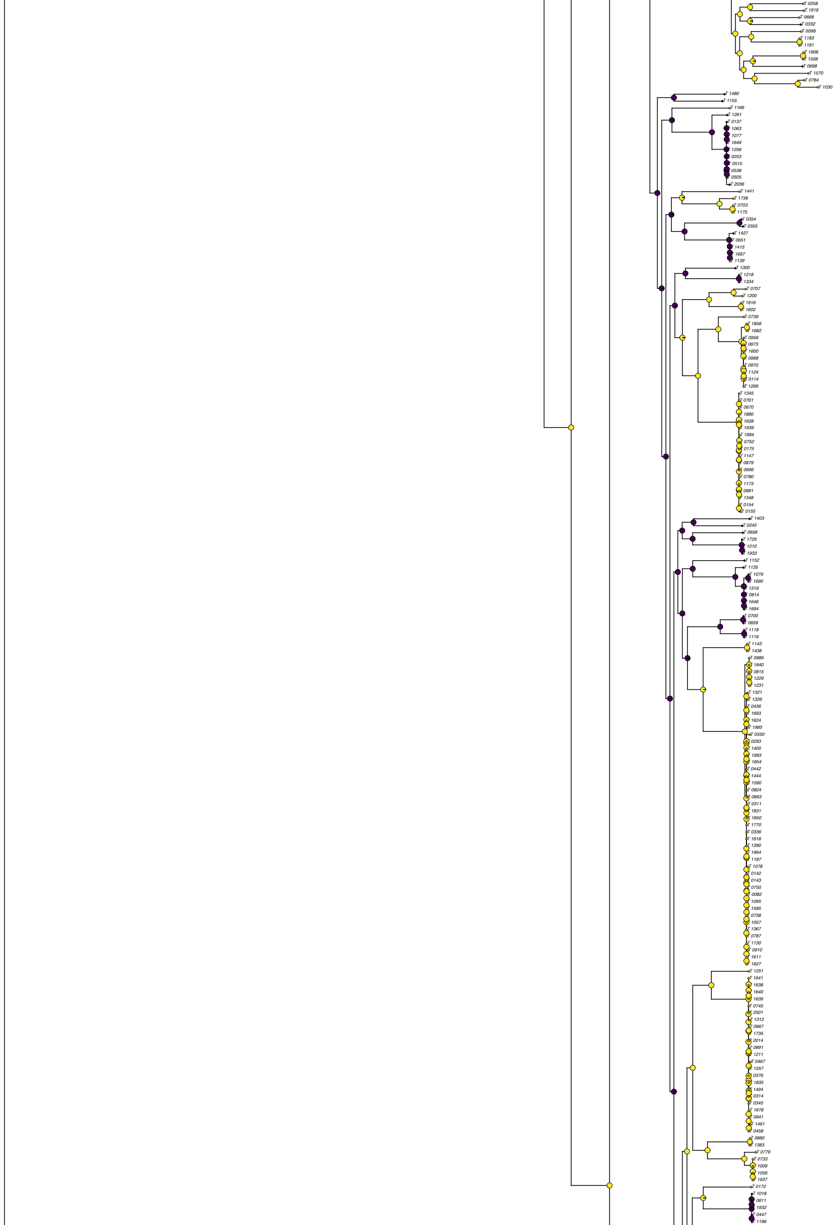

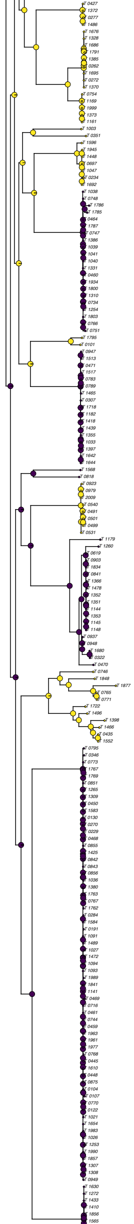
T 0427
T 1372
T 0277
T 1486
T 1676
T 1328
T 1686
T 1791
T 1385
T 0262
T 1695
T 0272
T 1370
T 0754
T 1169
T 1999
T 1373
T 1161
T 1003
T 0351
T 1596
T 1945
T 1448
T 0697
T 1047
T 0234
T 1692
T 1038
T 0748
T 1786
T 1785
T 0464
T 1787
T 0747
T 1386
T 1039
T 1041
T 1040
T 1331
T 0460
T 1934
T 1800
T 1310
T 0734
T 1254
T 1803
T 0766
T 0751
T 1795
T 0101
T 0947
T 1513
T 0471
T 1517
T 0783
T 0789
T 1465
T 0307
T 1718
T 1182
T 1418
T 1439
T 1355
T 1033
T 1397
T 1642
T 1644
T 1568
T 0818
T 0923
T 0979
T 2009
T 0540
T 0491
T 0501
T 0499
T 0531
T 1179
T 1260
T 0619
T 0903
T 1834
T 0841
T 1366
T 1478
T 1352
T 1351
T 1144
T 1353
T 1145
T 1148
T 0937
T 0948
T 1680
T 0322
T 0470
T 0746
T 1848
T 1877
T 0765
T 0771
T 1722
T 1496
T 1398
T 1466
T 0435
T 1552
T 0795
T 0346
T 0773
T 1767
T 1769
T 0851
T 1265
T 1309
T 0450
T 1583
T 0130
T 0270
T 0229
T 0468
T 0855
T 1425
T 0842
T 0843
T 0856
T 1036
T 1380
T 1763
T 0767
T 1762
T 0284
T 1584
T 0191
T 1091
T 1489
T 1027
T 1472
T 1094
T 1093
T 1989
T 1841
T 1141
T 0469
T 0716
T 0461
T 0744
T 0459
T 1963
T 1961
T 1977
T 0768
T 0445
T 1610
T 0448
T 0875
T 0104
T 0107
T 0770
T 0122
T 1021
T 1654
T 1983
T 1026
T 1253
T 1990
T 1857
T 1307
T 1308
T 0949
T 1630
T 1272
T 1433
T 1410
T 1856
T 1565

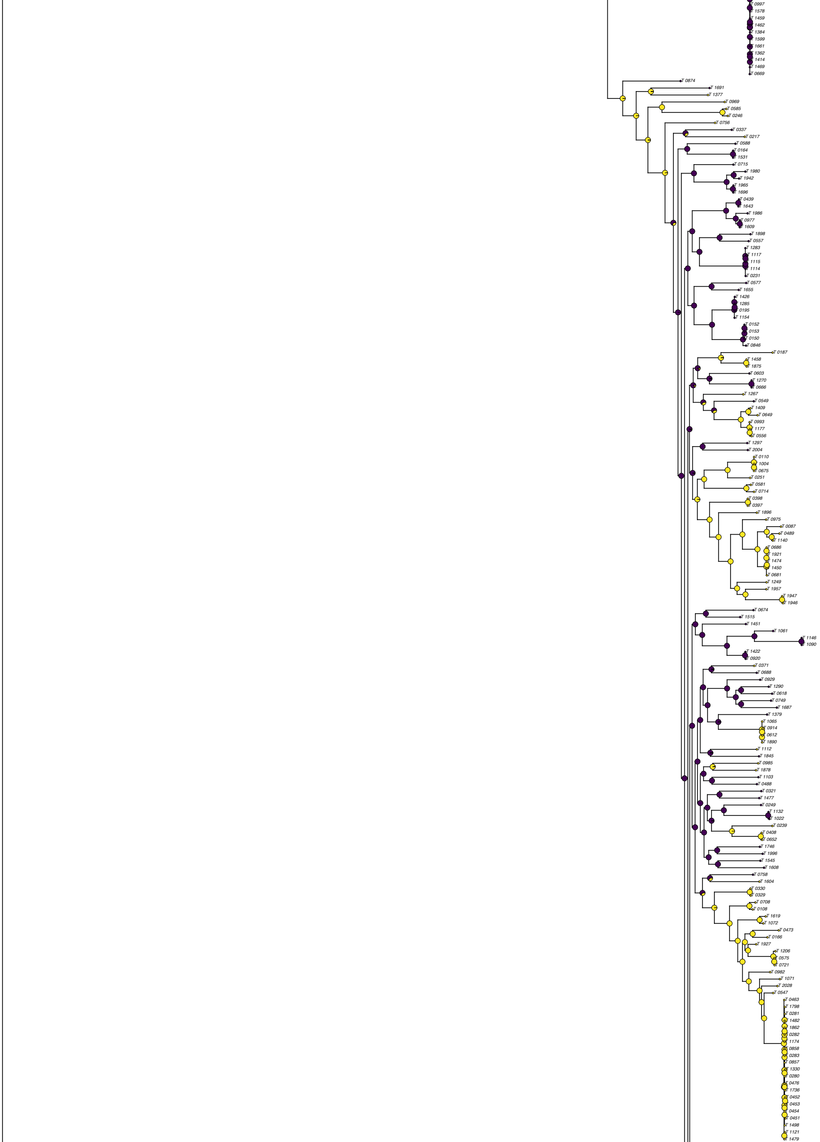
T 0997
T 1578
T 1459
T 1462
T 1384
T 1599
T 1661
T 1362
T 1414
T 1469
T 0669
T 0874
T 1691
T 1377
T 0969
T 0585
T 0246
T 0756
T 0337
T 0217
T 0588
T 0164
T 1531
T 0715
T 1980
T 1942
T 1965
T 1696
T 0439
T 1643
T 1986
T 0977
T 1609
T 1898
T 0557
T 1283
T 1117
T 1115
T 1114
T 0231
T 0577
T 1655
T 1426
T 1285
T 0195
T 1154
T 0152
T 0153
T 0150
T 0846
T 0187
T 1458
T 1875
T 0603
T 1270
T 0666
T 1267
T 0549
T 1409
T 0649
T 0993
T 1177
T 0556
T 1297
T 2004
T 0110
T 1004
T 0675
T 0251
T 0581
T 0714
T 0398
T 0397
T 1896
T 0975
T 0087
T 0489
T 1140
T 0686
T 1921
T 1474
T 1450
T 0681
T 1249
T 1957
T 1947
T 1946
T 0674
T 1515
T 1451
T 1061
T 1146
T 1090
T 1422
T 0920
T 0371
T 0688
T 0929
T 1290
T 0618
T 0749
T 1687
T 1379
T 1065
T 0914
T 0612
T 1890
T 1112
T 1845
T 0985
T 1878
T 1103
T 0488
T 0321
T 1477
T 0249
T 1132
T 1022
T 0239
T 0408
T 0652
T 1746
T 1996
T 1545
T 1608
T 0758
T 1604
T 0330
T 0329
T 0708
T 0108
T 1619
T 1072
T 0473
T 0166
T 1927
T 1206
T 0575
T 0721
T 0982
T 1071
T 2028
T 0547
T 0463
T 1798
T 0281
T 1482
T 1862
T 0282
T 1174
T 0858
T 0283
T 0857
T 1330
T 0280
T 0476
T 1736
T 0452
T 0453
T 0454
T 0451
T 1498
T 1121
T 1479

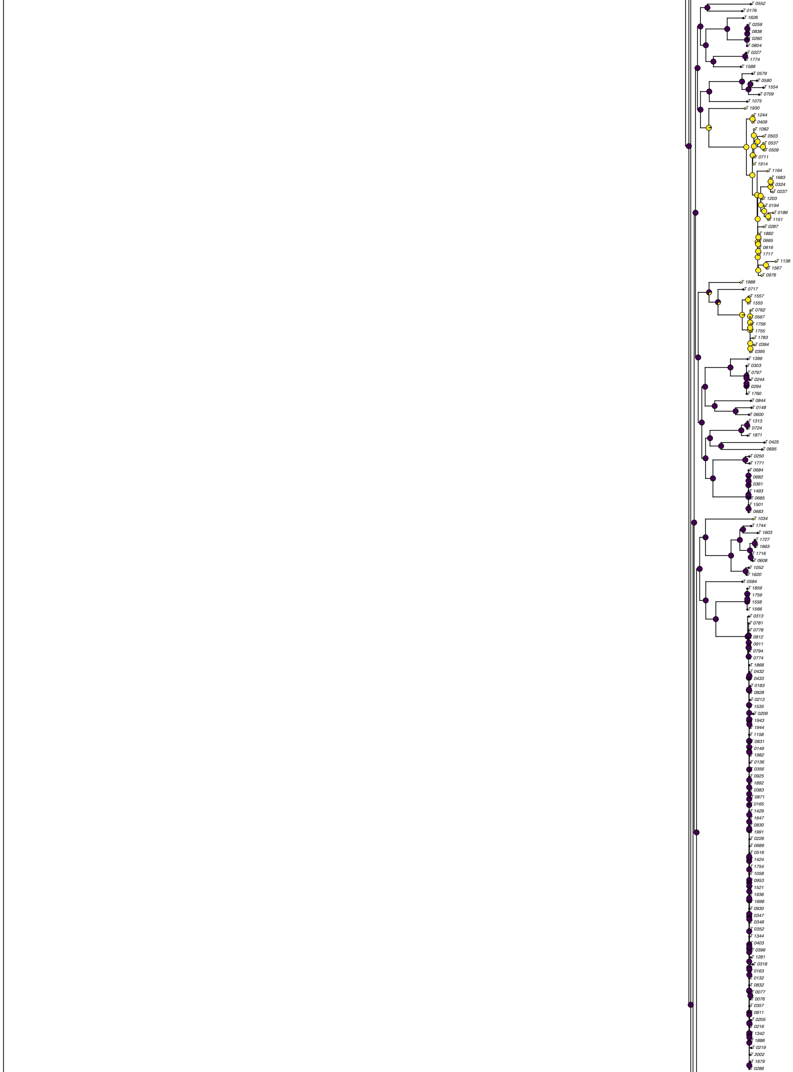
T 0552
T 0176
T 1626
T 0259
0838
T 0260
T 0804
T 0227
T 1774
T 1588
T 0579
T 0580
T 1554
T 0709
T 1075
T 1930
T 1244
T 0409
T 1082
T 0503
T 0537
T 0509
T 0711
T 1914
T 1164
T 1683
0324
T 0237
T 1203
T 0194
T 0186
T 1151
T 0287
T 1882
0665
T 0816
1717
T 1138
T 1567
T 0976
T 1988
T 0717
T 1557
1555
T 0762
0567
1756
1755
T 1783
T 0394
0395
T 1399
T 0303
T 0797
T 0244
0294
T 1760
T 0844
T 0148
T 0600
T 1313
0724
T 1871
T 0425
T 0695
T 0250
T 1771
T 0684
0682
0361
T 1493
T 0685
T 1501
0683
T 1034
T 1744
T 1603
T 1727
T 1663
T 1716
T 0608
T 1052
T 1620
T 0584
T 1859
1759
1558
T 1566
T 0313
T 0781
T 0778
0812
0911
T 0794
0774
T 1868
T 0432
0433
T 0183
T 0828
T 0213
1535
T 0209
1943
1944
T 1158
0831
0149
T 1982
T 0136
0356
T 0925
T 1892
0383
T 0871
0165
T 1429
T 1647
T 0830
1991
T 0226
T 0689
T 0516
1424
T 1754
T 1058
0953
1521
T 1936
1698
T 0930
0347
T 0348
0352
T 1344
0403
T 0396
T 1281
T 0318
0163
T 0132
T 0832
T 0077
T 0076
T 0357
0811
T 0205
T 0216
1342
T 1888
T 0219
T 2002
T 1679
T 0288

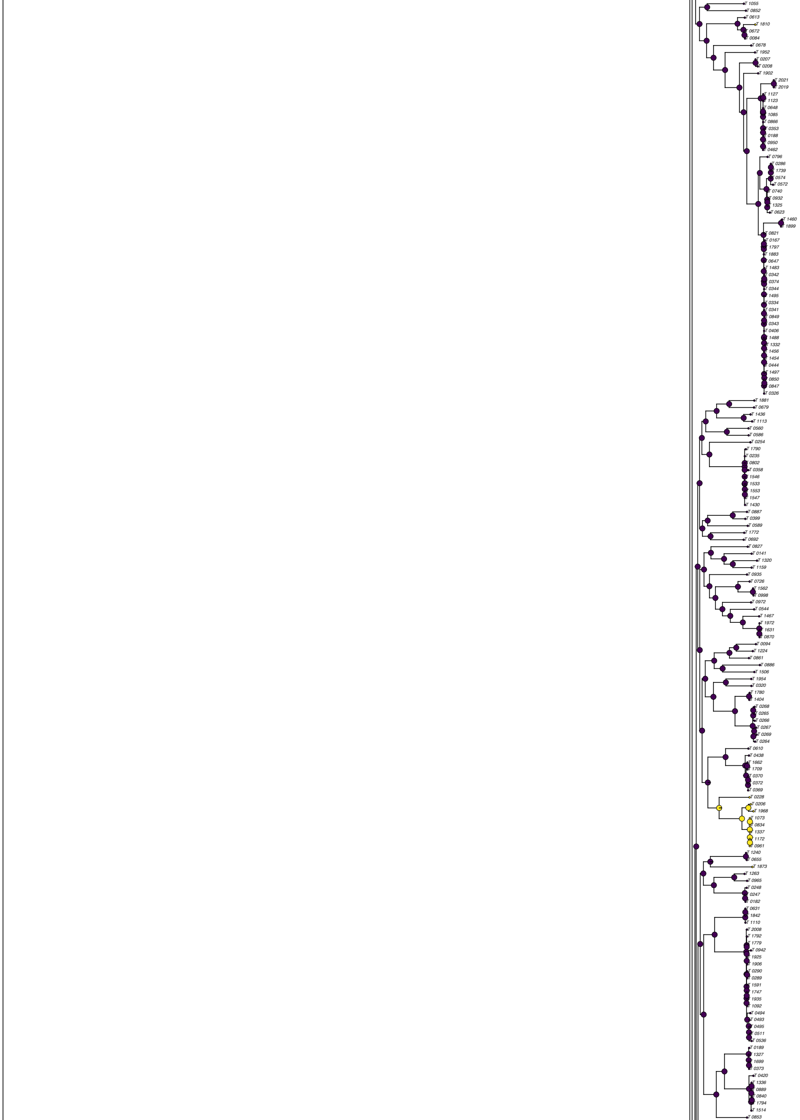
T 1055
T 0852
T 0613
T 1810
0672
T 0084
T 0678
T 1952
T 0207
T 0208
T 1902
T 2021
T 2019
T 1127
T 1123
T 0648
1085
T 0866
T 0353
T 0188
0950
T 0462
T 0796
T 0286
T 1739
0574
T 0572
T 0740
T 0932
1325
T 0623
T 1460
T 1899
0821
T 0167
T 1797
T 1883
0647
T 1483
T 0342
0374
T 0344
1495
T 0334
T 0341
T 0849
0343
T 0406
1488
T 1332
1456
T 1454
0444
T 1497
0850
0847
T 0326
T 1881
T 0679
T 1436
T 1113
T 0560
T 0586
T 0254
T 1790
T 0235
0802
T 0358
1546
1533
1553
T 1547
T 1430
T 0887
T 0399
T 0589
T 1772
T 0692
T 0827
T 0141
T 1320
T 1159
T 0935
T 0726
T 1562
T 0998
T 0972
T 0544
T 1467
T 1972
1631
T 0870
T 0094
T 1224
T 0861
T 0886
T 1506
T 1954
T 0320
T 1780
T 1404
T 0268
0265
T 0266
T 0267
T 0269
T 0264
T 0610
T 0438
T 1662
T 1709
T 0370
0372
T 0369
T 0228
T 0206
T 1968
T 1073
T 0834
T 1337
1172
T 0961
T 1240
T 0655
T 1873
T 1263
T 0965
T 0248
0247
T 0182
T 0631
1842
T 1110
T 2008
T 1792
T 1779
T 0942
T 1925
T 1906
T 0290
T 0289
T 1591
1747
1935
T 1092
T 0494
T 0493
0495
0511
T 0536
T 0189
1327
1699
T 0373
T 0420
T 1336
0889
T 0840
1794
T 1514
T 0853

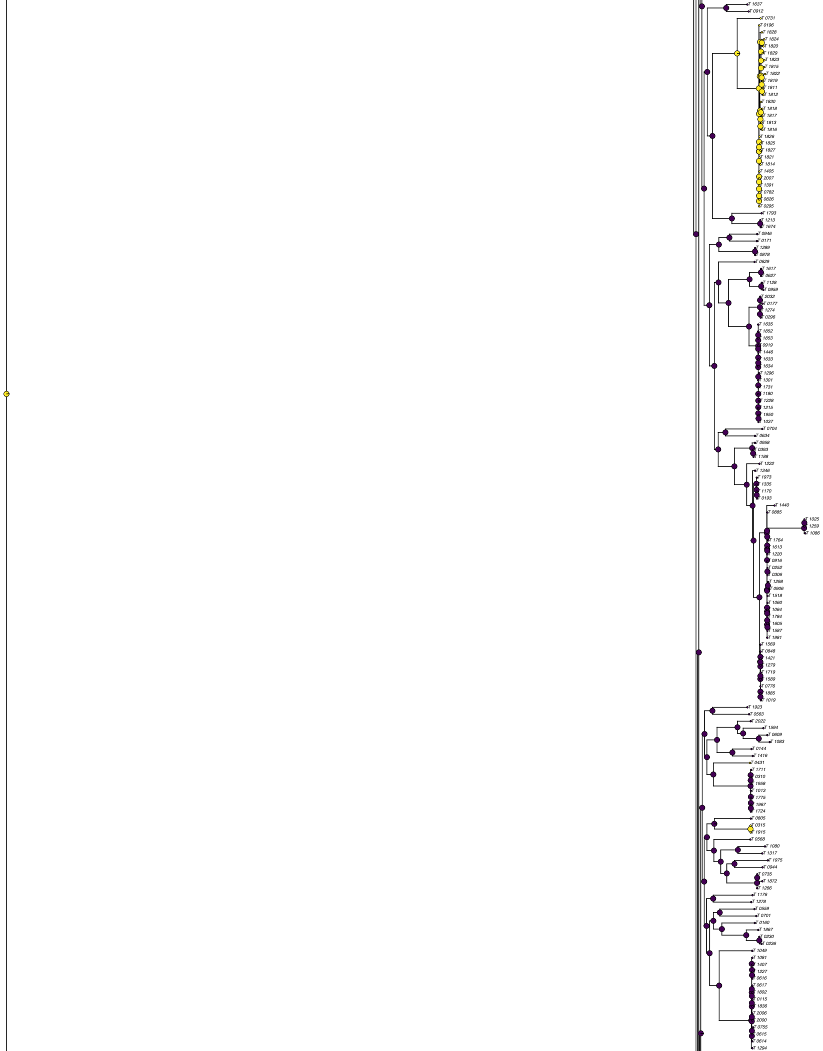
T 1637
T 0912
T 0731
T 0196
T 1828
T 1824
T 1820
T 1829
T 1823
T 1815
T 1822
T 1819
T 1811
T 1812
T 1830
T 1818
T 1817
T 1813
T 1816
T 1826
T 1825
T 1827
T 1821
T 1814
T 1405
2007
1391
0782
0826
T 0295
T 1793
T 1213
T 1674
T 0946
T 0171
T 1289
T 0878
T 0629
T 1617
T 0627
T 1128
T 0959
T 2032
T 0177
T 1274
T 0296
T 1635
T 1852
1853
0919
T 1446
1633
1634
T 1296
T 1301
1731
1180
1228
1215
1950
T 1037
T 0704
T 0634
T 0958
T 0393
T 1188
T 1222
T 1346
T 1973
1335
1170
T 0193
T 1440
T 0885
T 1025
1259
T 1086
T 1764
1613
T 1220
0916
T 0252
T 0306
T 1298
T 0906
T 1518
T 1060
1064
T 1784
1605
T 1587
T 1981
T 1569
T 0848
1421
1279
T 1719
1589
T 0776
1885
T 1019
T 1923
T 0563
T 2022
T 1594
T 0609
T 1083
T 0144
T 1416
T 0431
T 1711
0310
1958
T 1013
1775
1967
T 1724
T 0805
T 0315
T 1915
T 0568
T 1080
T 1317
T 1975
T 0944
T 0735
T 1872
T 1266
T 1176
T 1278
T 0559
T 0701
T 0160
T 1867
T 0230
T 0236
T 1049
T 1081
1407
1227
0616
T 0617
1802
T 0115
1836
T 2006
2000
T 0755
0615
T 0614
T 1294

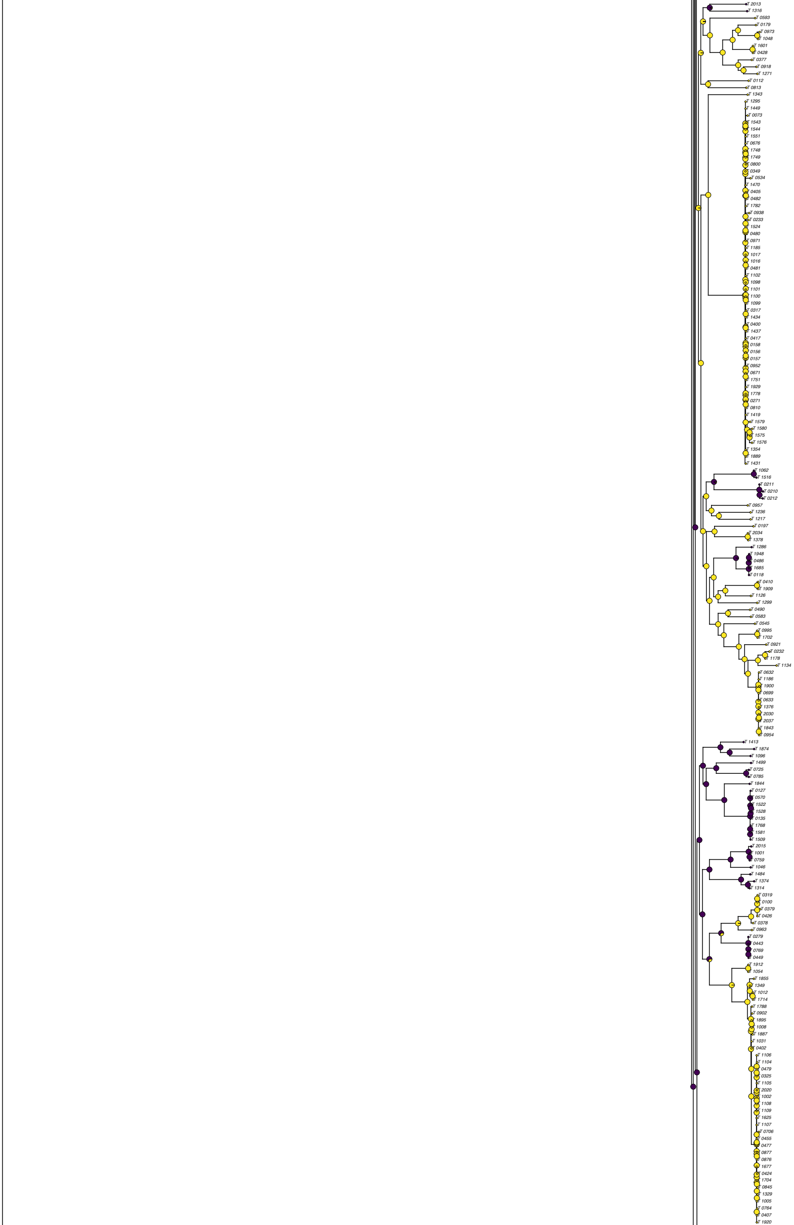
T 2013
T 1316
T 0593
T 0179
T 0973
T 1048
T 1601
T 0428
T 0377
T 0918
T 1271
T 0112
T 0813
T 1343
T 1295
T 1449
T 0073
T 1543
T 1544
T 1551
T 0676
T 1748
T 1749
T 0800
T 0349
T 0534
T 1470
T 0405
T 0482
T 1782
T 0938
T 0233
T 1524
T 0480
T 0971
T 1185
T 1017
T 1016
T 0481
T 1102
T 1098
T 1101
T 1100
T 1099
T 0317
T 1434
T 0400
T 1437
T 0417
T 0158
T 0156
T 0157
T 0952
T 0671
T 1751
T 1929
T 1778
T 0271
T 0810
T 1419
T 1579
T 1580
T 1575
T 1576
T 1354
T 1889
T 1431
T 1062
T 1516
T 0211
T 0210
T 0212
T 0957
T 1236
T 1217
T 0197
T 2034
T 1378
T 1286
T 1948
T 0486
T 1685
T 0118
T 0410
T 1909
T 1126
T 1299
T 0490
T 0583
T 0545
T 0995
T 1702
T 0921
T 0232
T 1178
T 1134
T 0632
T 1186
T 1900
T 0699
T 0633
T 1376
T 2030
T 2037
T 1843
T 0954
T 1413
T 1874
T 1096
T 1499
T 0725
T 0785
T 1844
T 0127
T 0570
T 1522
T 1528
T 0135
T 1768
T 1581
T 1509
T 2015
T 1001
T 0759
T 1046
T 1484
T 1374
T 1314
T 0319
T 0100
T 0379
T 0426
T 0378
T 0963
T 0279
T 0443
T 0769
T 0449
T 1912
T 1054
T 1855
T 1349
T 1012
T 1714
T 1788
T 0902
T 1895
T 1008
T 1887
T 1031
T 0402
T 1106
T 1104
T 0479
T 0325
T 1105
T 2020
T 1002
T 1108
T 1109
T 1625
T 1107
T 0706
T 0455
T 0477
T 0877
T 0876
T 1677
T 0424
T 1704
T 0845
T 1329
T 1005
T 0764
T 0407
T 1920

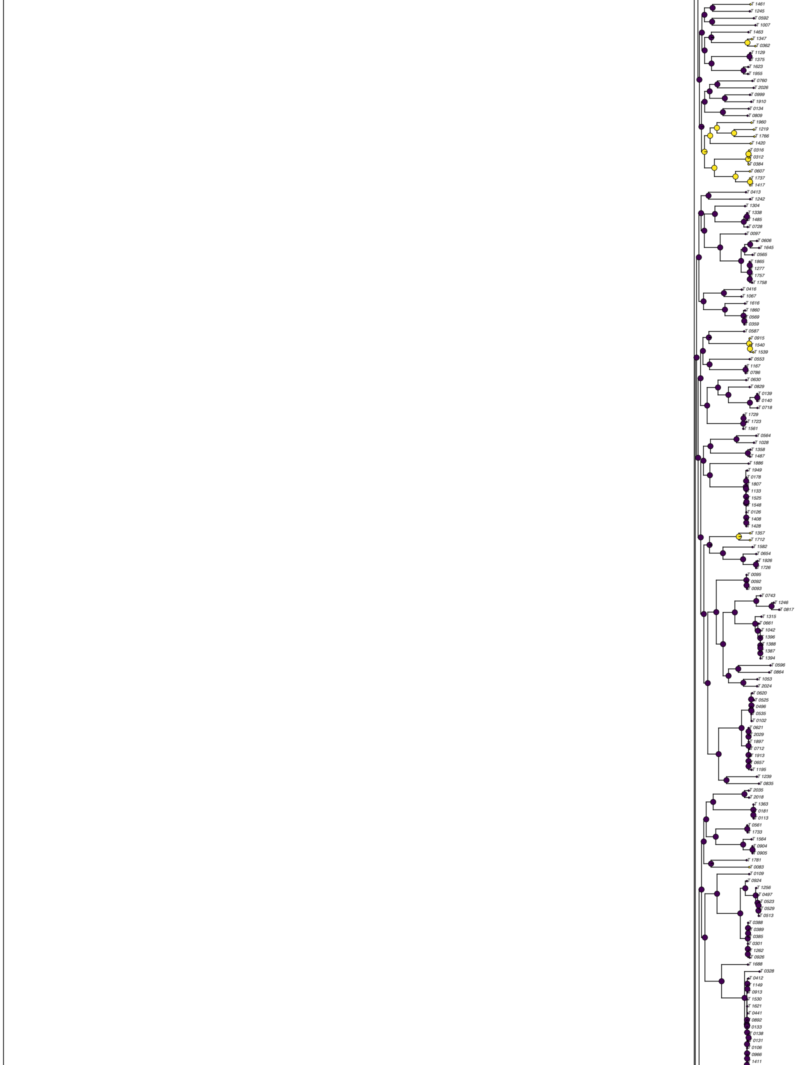

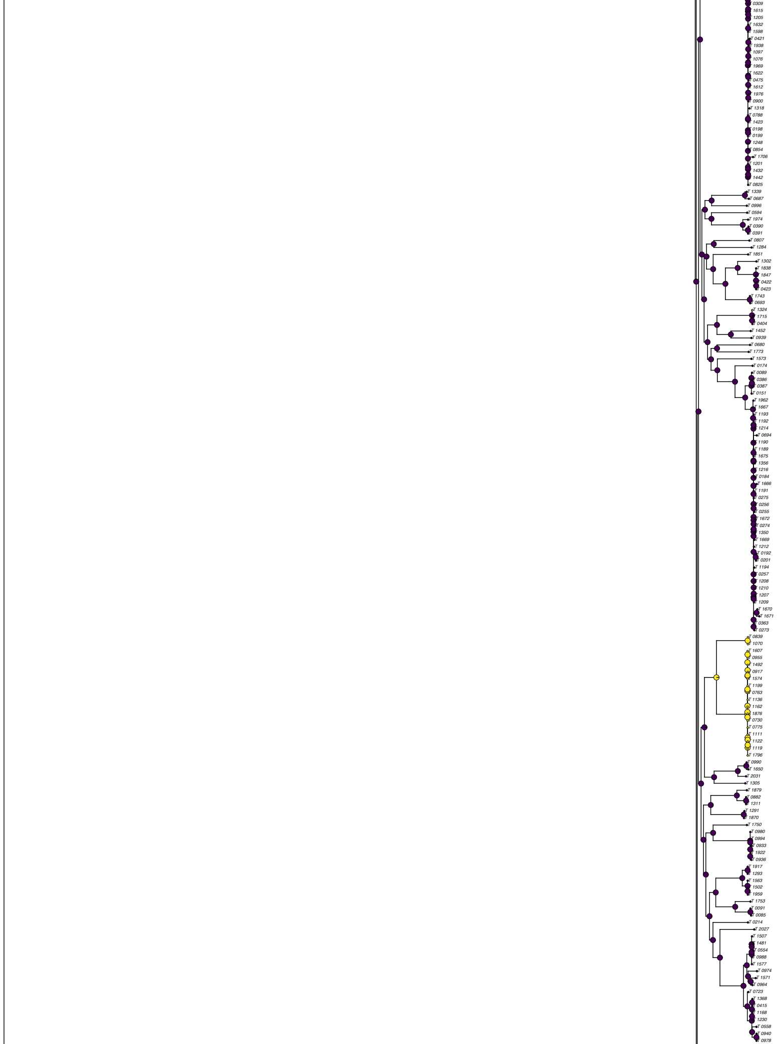
T 0309
T 1615
T 1205
T 1632
T 1598
T 0421
T 1938
T 1097
T 1076
T 1969
T 1622
T 0475
T 1612
T 1976
T 0900
T 1318
T 0788
T 1423
T 0198
T 0199
T 1248
T 0854
T 1706
T 1201
T 1432
T 1442
T 0825
T 1339
T 0687
T 0996
T 0594
T 1974
T 0390
T 0391
T 0807
T 1284
T 1851
T 1302
T 1838
T 1847
T 0422
T 0423
T 1743
T 0693
T 1324
T 1715
T 0404
T 1452
T 0939
T 0680
T 1773
T 1573
T 0174
T 0089
T 0386
T 0387
T 0151
T 1962
T 1667
T 1193
T 1192
T 1214
T 0694
T 1190
T 1189
T 1675
T 1356
T 1216
T 0184
T 1666
T 1191
T 0275
T 0256
T 0255
T 1672
T 0274
T 1350
T 1669
T 1212
T 0192
T 0201
T 1194
T 0257
T 1208
T 1210
T 1207
T 1209
T 1670
T 1671
T 0363
T 0273
T 0839
T 1070
T 1607
T 0955
T 1492
T 0917
T 1574
T 1199
T 0763
T 1136
T 1162
T 1876
T 0730
T 0775
T 1111
T 1122
T 1119
T 1796
T 0990
T 1650
T 2031
T 1305
T 1879
T 0882
T 1311
T 1291
T 1870
T 1750
T 0980
T 0994
T 0933
T 1922
T 0936
T 1917
T 1293
T 1563
T 1502
T 1959
T 1753
T 0091
T 0085
T 0214
T 2027
T 1507
T 1481
T 0554
T 0988
T 1577
T 0974
T 1571
T 0964
T 0723
T 1368
T 0415
T 1168
T 1230
T 0558
T 0940
T 0978

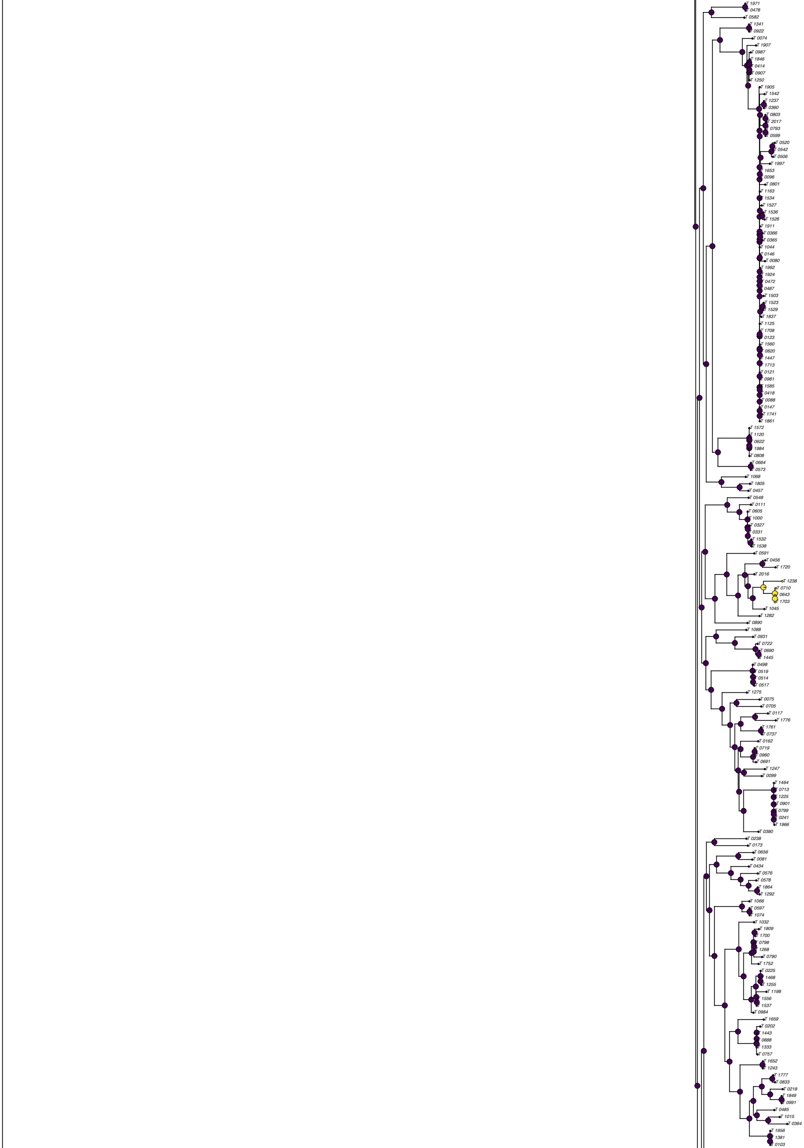
T 1971
T 0478
T 0582
T 1341
T 0922
T 0074
T 1907
T 0987
T 1846
T 0414
T 0907
T 1250
T 1905
T 1542
T 1237
T 0360
T 0803
T 2017
T 0793
T 0599
T 0520
T 0542
T 0506
T 1997
1653
0096
T 0801
T 1163
1534
T 1527
T 1536
T 1526
T 1911
T 0366
T 0365
T 1044
T 0146
T 0080
T 1992
1924
0472
0487
T 1503
T 1523
T 1529
T 1837
T 1125
T 1708
0123
T 1560
0820
T 1447
1713
T 0121
0981
1585
T 0418
T 0088
T 0147
T 1741
T 1861
T 1572
T 1120
T 0622
1984
T 0808
T 0664
T 0573
T 1068
T 1805
T 0457
T 0548
T 0111
T 0605
1000
T 0327
0331
T 1532
T 1538
T 0591
T 0456
T 1720
T 2016
T 1238
T 0710
0643
T 1703
T 1045
T 1282
T 0890
T 1088
T 0931
T 0722
T 0690
T 1445
T 0498
0519
0514
T 0517
T 1275
T 0075
T 0705
T 0117
T 1776
T 1761
T 0737
T 0162
T 0719
0960
T 0691
T 1247
T 0099
T 1464
0713
1225
0901
0799
0241
T 1966
T 0380
T 0238
T 0173
T 0656
T 0081
T 0434
T 0576
T 0578
T 1864
T 1292
T 1066
T 0597
T 1074
T 1032
T 1809
T 1700
0798
1268
T 0790
T 1752
T 0225
1468
T 1255
T 1198
1556
T 1537
T 0984
T 1659
T 0202
1443
0888
T 1333
T 0757
T 1652
T 1243
T 1777
T 0833
T 0218
T 1849
0991
T 0485
T 1015
T 0364
T 1956
1381
T 0103

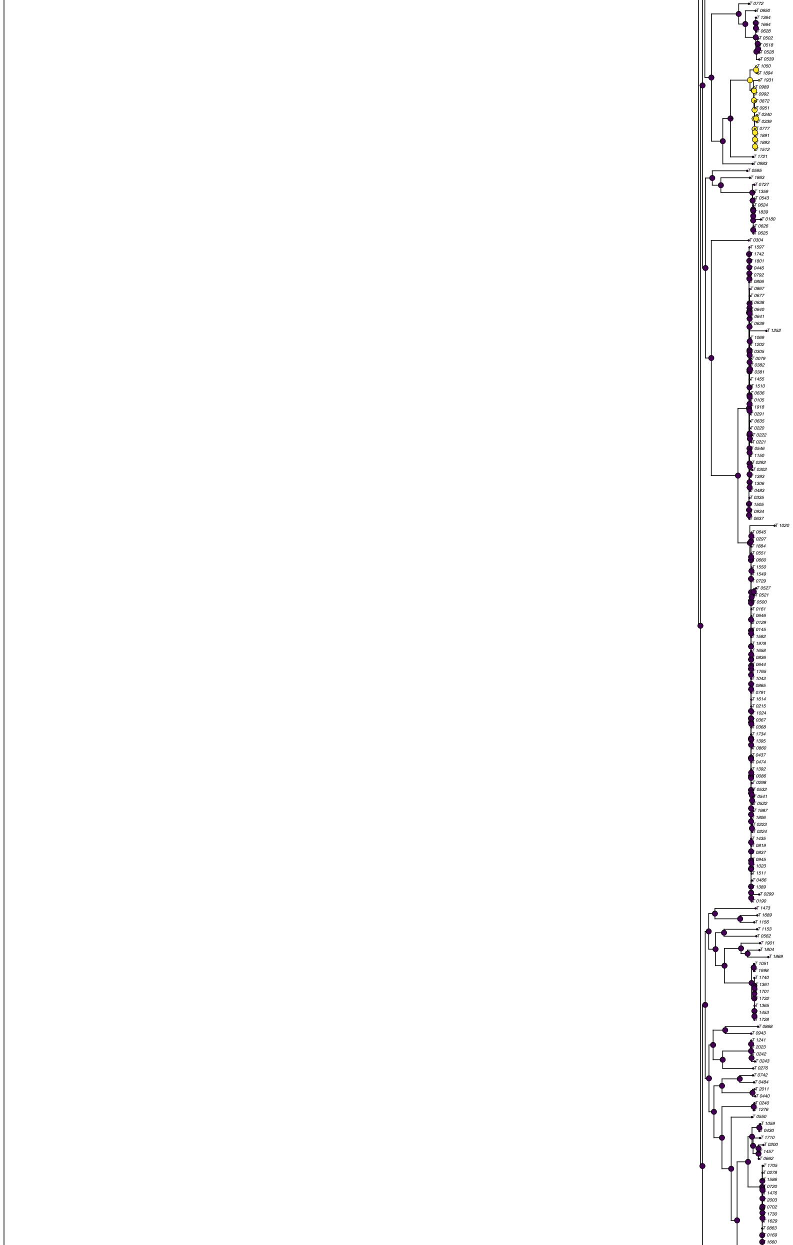
T 0772
T 0650
T 1364
1664
0628
T 0502
0518
T 0528
T 0539
T 1050
T 1894
T 1931
T 0989
0992
T 0872
0951
T 0340
0339
0777
1891
1893
1512
T 1721
T 0983
T 0595
T 1863
T 0727
T 1359
T 0543
T 0624
1839
T 0180
T 0626
T 0625
T 0304
T 1597
1742
1801
0446
0792
0806
T 0867
T 0677
0638
0640
0641
0639
T 1252
T 1069
T 1202
0305
T 0079
0382
0381
T 1455
1510
T 0636
0105
1918
T 0291
T 0635
T 0220
T 0222
T 0221
0546
1150
T 0292
T 0302
1393
1306
T 0483
T 0335
1505
0934
T 0637
T 1020
T 0645
0297
1884
T 0551
0660
T 1550
1549
0729
T 0527
T 0521
0500
T 0161
0646
T 0129
T 0145
T 1592
T 1978
T 1658
0836
0644
T 1765
T 1043
0865
T 0791
T 1614
T 0215
T 1024
0367
T 0368
T 1734
1395
T 0860
0437
T 0474
T 1392
0086
T 0298
T 0532
T 0541
T 0522
T 1987
1806
T 0223
T 0224
T 1435
T 0819
0837
0945
1023
T 1511
T 0466
1389
T 0299
T 0190
T 1473
T 1689
T 1156
T 1153
T 0562
T 1901
T 1804
T 1869
T 1051
T 1998
T 1740
1361
1701
1732
T 1365
1453
T 1728
T 0868
T 0943
T 1241
2023
0242
T 0243
T 0276
T 0742
T 0484
T 2011
T 0440
T 0240
T 1276
T 0550
T 1059
T 0430
T 1710
T 0200
T 1457
T 0662
T 1705
T 0278
1586
0720
T 1476
2003
0702
T 1730
T 1629
T 0863
T 0169
1660

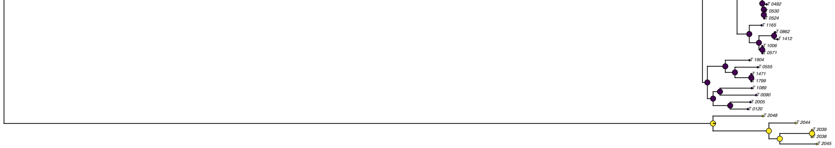

T 0492
T 0530
T 0524
T 1165
T 0862
T 1412
T 1006
T 0571
T 1904
T 0555
T 1471
T 1799
T 1089
T 0090
T 2005
T 0120
T 2048
T 2044
T 2039
T 2038
T 2045

Effector gene: tspE1c

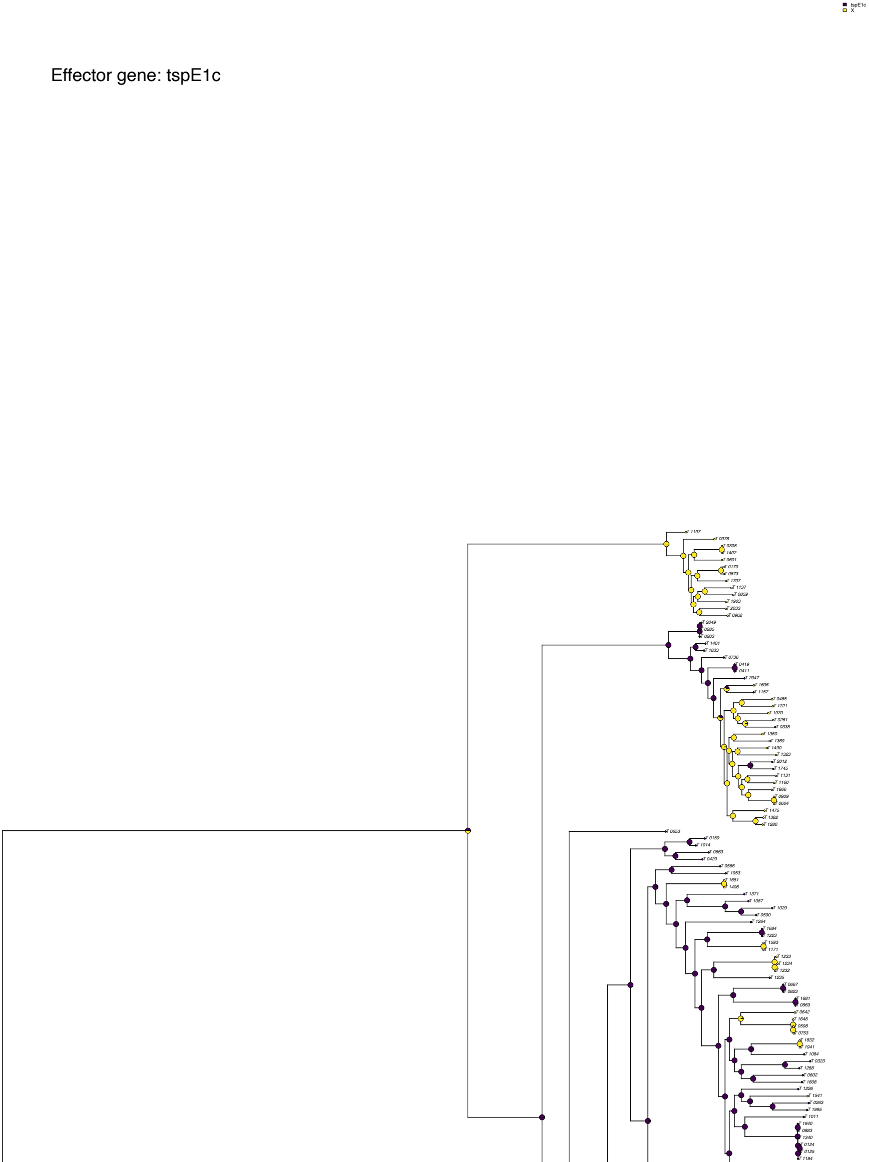

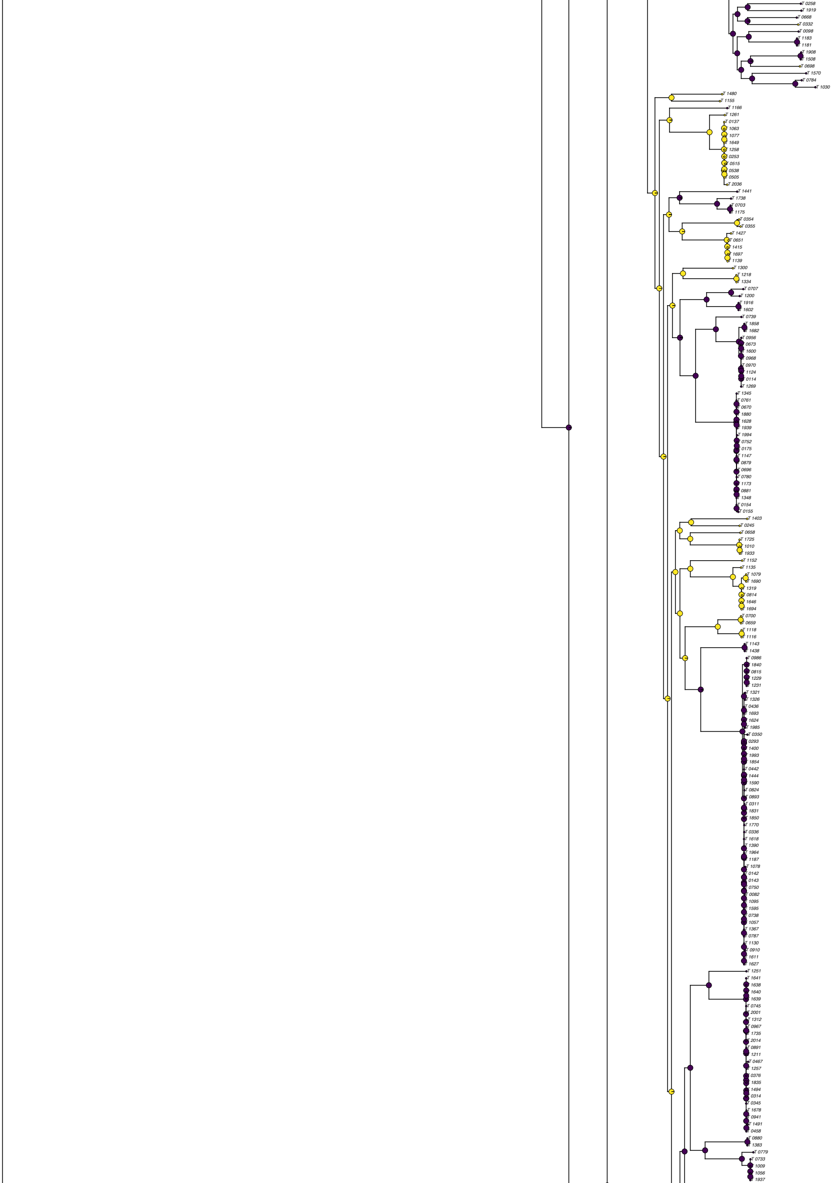

T 0258
T 1919
T 0668
T 0332
T 0098
T 1183
T 1181
T 1908
T 1508
T 0698
T 1570
T 0784
T 1030
T 1480
T 1155
T 1166
T 1261
T 0137
T 1063
T 1077
T 1649
T 1258
T 0253
T 0515
T 0538
T 0505
T 2036
T 1441
T 1738
T 0703
T 1175
T 0354
T 0355
T 1427
T 0651
T 1415
T 1697
T 1139
T 1300
T 1218
T 1334
T 0707
T 1200
T 1916
T 1602
T 0739
T 1858
T 1682
T 0956
T 0673
T 1600
T 0968
T 0970
T 1124
T 0114
T 1269
T 1345
T 0761
T 0670
T 1880
T 1628
T 1939
T 1994
T 0752
T 0175
T 1147
T 0879
T 0696
T 0780
T 1173
T 0881
T 1348
T 0154
T 0155
T 1403
T 0245
T 0658
T 1725
T 1010
T 1933
T 1152
T 1135
T 1079
T 1690
T 1319
T 0814
T 1646
T 1694
T 0700
T 0659
T 1118
T 1116
T 1143
T 1438
T 0986
T 1840
T 0815
T 1229
T 1231
T 1321
T 1326
T 0436
T 1693
T 1624
T 1985
T 0350
T 0293
T 1400
T 1993
T 1854
T 0442
T 1444
T 1590
T 0824
T 0893
T 0311
T 1831
T 1850
T 1770
T 0336
T 1618
T 1390
T 1964
T 1187
T 1078
T 0142
T 0143
T 0750
T 0082
T 1095
T 1595
T 0738
T 1057
T 1367
T 0787
T 1130
T 0910
T 1611
T 1627
T 1251
T 1641
T 1638
T 1640
T 1639
T 0745
T 2001
T 1312
T 0967
T 1735
T 2014
T 0891
T 1211
T 0467
T 1257
T 0376
T 1835
T 1494
T 0314
T 0345
T 1678
T 0941
T 1491
T 0458
T 0880
T 1383
T 0779
T 0733
T 1009
T 1056
T 1937

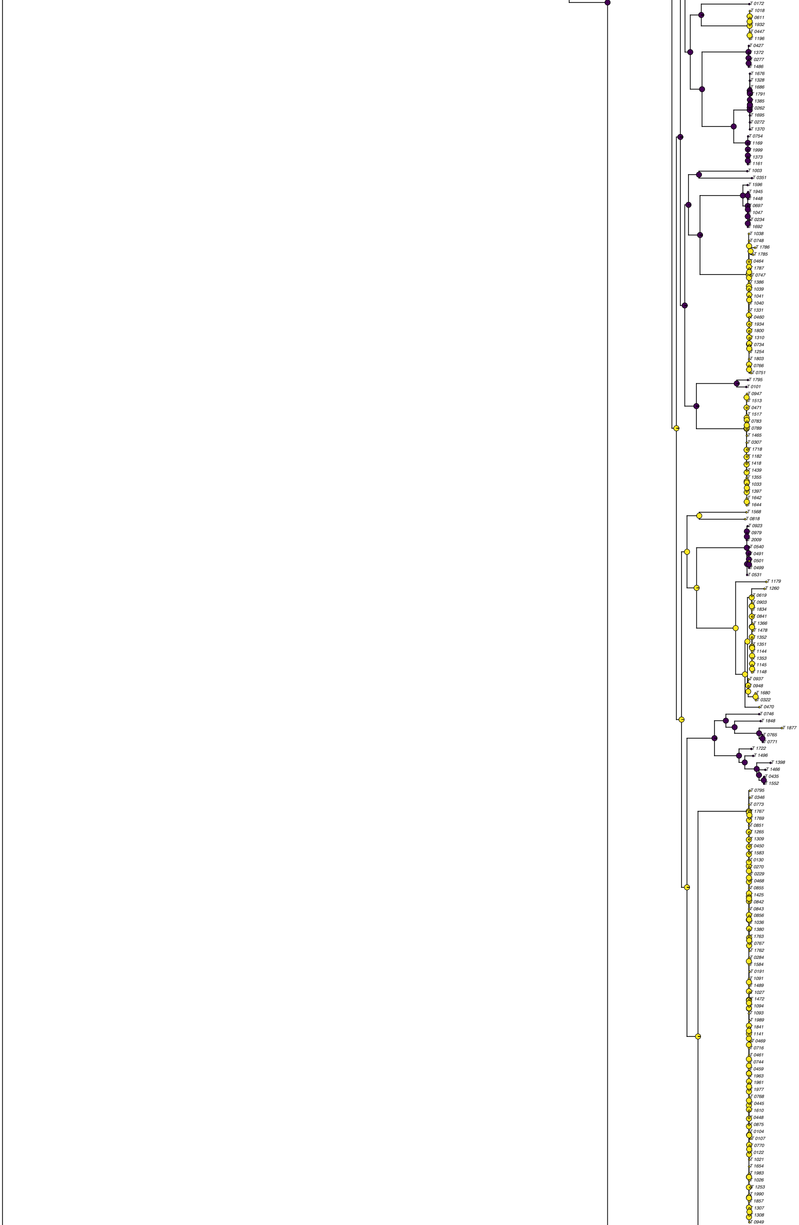
T 0172
T 1018
T 0611
T 1932
T 0447
T 1196
T 0427
T 1372
T 0277
T 1486
T 1676
T 1328
T 1686
T 1791
T 1385
T 0262
T 1695
T 0272
T 1370
T 0754
T 1169
T 1999
T 1373
T 1161
T 1003
T 0351
T 1596
T 1945
T 1448
T 0697
T 1047
T 0234
T 1692
T 1038
T 0748
T 1786
T 1785
T 0464
T 1787
T 0747
T 1386
T 1039
T 1041
T 1040
T 1331
T 0460
T 1934
T 1800
T 1310
T 0734
T 1254
T 1803
T 0766
T 0751
T 1795
T 0101
T 0947
T 1513
T 0471
T 1517
T 0783
T 0789
T 1465
T 0307
T 1718
T 1182
T 1418
T 1439
T 1355
T 1033
T 1397
T 1642
T 1644
T 1568
T 0818
T 0923
T 0979
T 2009
T 0540
T 0491
T 0501
T 0499
T 0531
T 1179
T 1260
T 0619
T 0903
T 1834
T 0841
T 1366
T 1478
T 1352
T 1351
T 1144
T 1353
T 1145
T 1148
T 0937
T 0948
T 1680
T 0322
T 0470
T 0746
T 1848
T 1877
T 0765
T 0771
T 1722
T 1496
T 1398
T 1466
T 0435
T 1552
T 0795
T 0346
T 0773
T 1767
T 1769
T 0851
T 1265
T 1309
T 0450
T 1583
T 0130
T 0270
T 0229
T 0468
T 0855
T 1425
T 0842
T 0843
T 0856
T 1036
T 1380
T 1763
T 0767
T 1762
T 0284
T 1584
T 0191
T 1091
T 1489
T 1027
T 1472
T 1094
T 1093
T 1989
T 1841
T 1141
T 0469
T 0716
T 0461
T 0744
T 0459
T 1963
T 1961
T 1977
T 0768
T 0445
T 1610
T 0448
T 0875
T 0104
T 0107
T 0770
T 0122
T 1021
T 1654
T 1983
T 1026
T 1253
T 1990
T 1857
T 1307
T 1308
T 0949

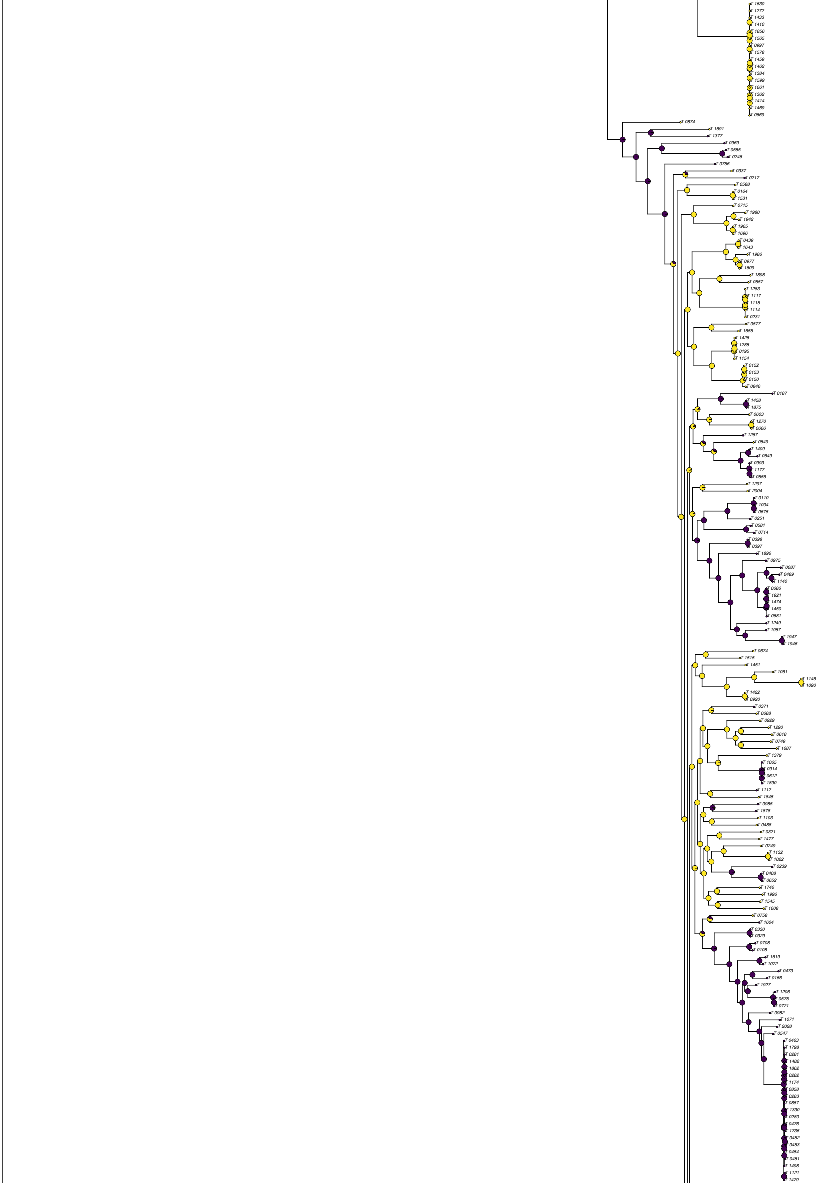
T 1630
T 1272
T 1433
T 1410
T 1856
T 1565
T 0997
T 1578
T 1459
T 1462
T 1384
T 1599
T 1661
T 1362
T 1414
T 1469
T 0669
T 0874
T 1691
T 1377
T 0969
T 0585
T 0246
T 0756
T 0337
T 0217
T 0588
T 0164
T 1531
T 0715
T 1980
T 1942
T 1965
T 1696
T 0439
T 1643
T 1986
T 0977
T 1609
T 1898
T 0557
T 1283
T 1117
T 1115
T 1114
T 0231
T 0577
T 1655
T 1426
T 1285
T 0195
T 1154
T 0152
T 0153
T 0150
T 0846
T 0187
T 1458
T 1875
T 0603
T 1270
T 0666
T 1267
T 0549
T 1409
T 0649
T 0993
T 1177
T 0556
T 1297
T 2004
T 0110
T 1004
T 0675
T 0251
T 0581
T 0714
T 0398
T 0397
T 1896
T 0975
T 0087
T 0489
T 1140
T 0686
T 1921
T 1474
T 1450
T 0681
T 1249
T 1957
T 1947
T 1946
T 0674
T 1515
T 1451
T 1061
T 1146
T 1090
T 1422
T 0920
T 0371
T 0688
T 0929
T 1290
T 0618
T 0749
T 1687
T 1379
T 1065
T 0914
T 0612
T 1890
T 1112
T 1845
T 0985
T 1878
T 1103
T 0488
T 0321
T 1477
T 0249
T 1132
T 1022
T 0239
T 0408
T 0652
T 1746
T 1996
T 1545
T 1608
T 0758
T 1604
T 0330
T 0329
T 0708
T 0108
T 1619
T 1072
T 0473
T 0166
T 1927
T 1206
T 0575
T 0721
T 0982
T 1071
T 2028
T 0547
T 0463
T 1798
T 0281
T 1482
T 1862
T 0282
T 1174
T 0858
T 0283
T 0857
T 1330
T 0280
T 0476
T 1736
T 0452
T 0453
T 0454
T 0451
T 1498
T 1121
T 1479

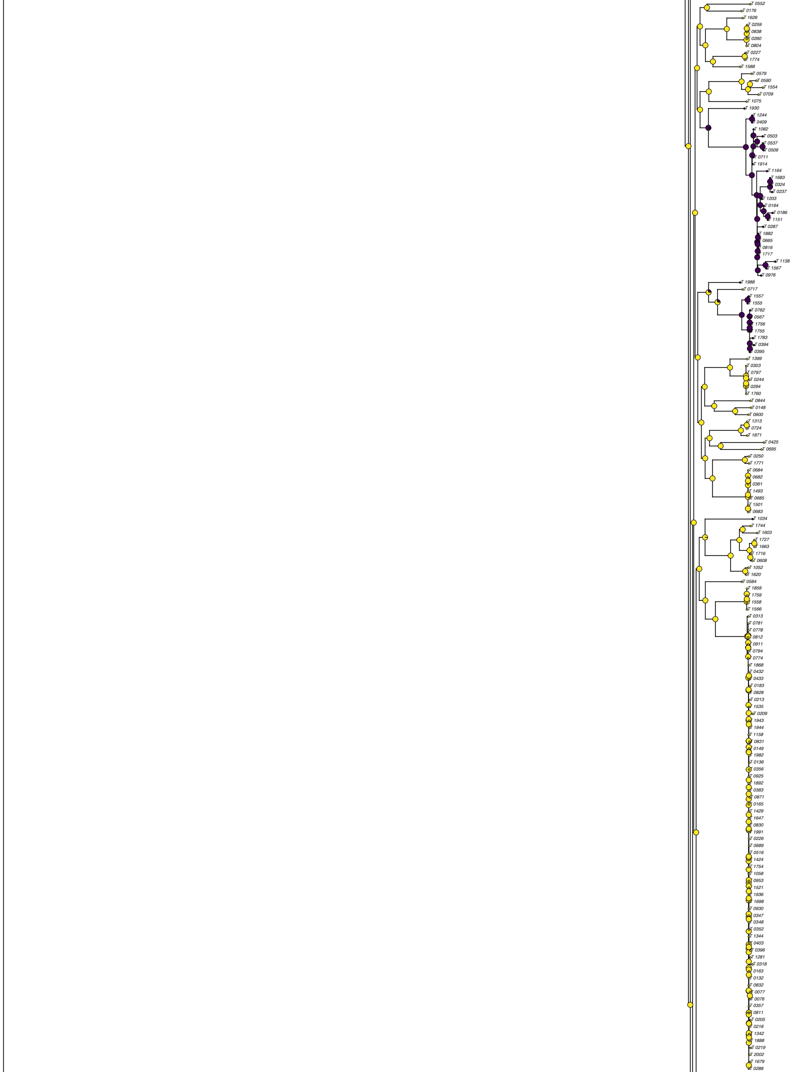

T 0552
T 0176
T 1626
T 0259
0838
0260
T 0804
T 0227
1774
T 1588
T 0579
T 0580
T 1554
T 0709
T 1075
T 1930
T 1244
T 0409
T 1082
T 0503
T 0537
T 0509
T 0711
T 1914
T 1164
T 1683
0324
T 0237
T 1203
T 0194
T 0186
1151
T 0287
T 1882
0665
T 0816
1717
T 1138
T 1567
T 0976
T 1988
T 0717
T 1557
1555
T 0762
0567
T 1756
1755
T 1783
T 0394
T 0395
T 1399
T 0303
T 0797
T 0244
0294
T 1760
T 0844
T 0148
T 0600
T 1313
T 0724
T 1871
T 0425
T 0695
T 0250
T 1771
T 0684
0682
0361
T 1493
T 0685
T 1501
0683
T 1034
T 1744
T 1603
T 1727
T 1663
T 1716
T 0608
T 1052
T 1620
T 0584
T 1859
1759
1558
T 1566
T 0313
T 0781
T 0778
0812
0911
T 0794
0774
T 1868
T 0432
0433
T 0183
T 0828
T 0213
1535
T 0209
1943
1944
T 1158
0831
0149
T 1982
T 0136
0356
T 0925
1892
0383
T 0871
0165
T 1429
1647
0830
1991
T 0226
T 0689
T 0516
1424
T 1754
T 1058
0953
1521
1936
1698
T 0930
0347
0348
T 0352
T 1344
T 0403
T 0396
T 1281
T 0318
0163
T 0132
T 0832
T 0077
T 0076
T 0357
0811
T 0205
T 0216
1342
1888
T 0219
T 2002
T 1679
0288

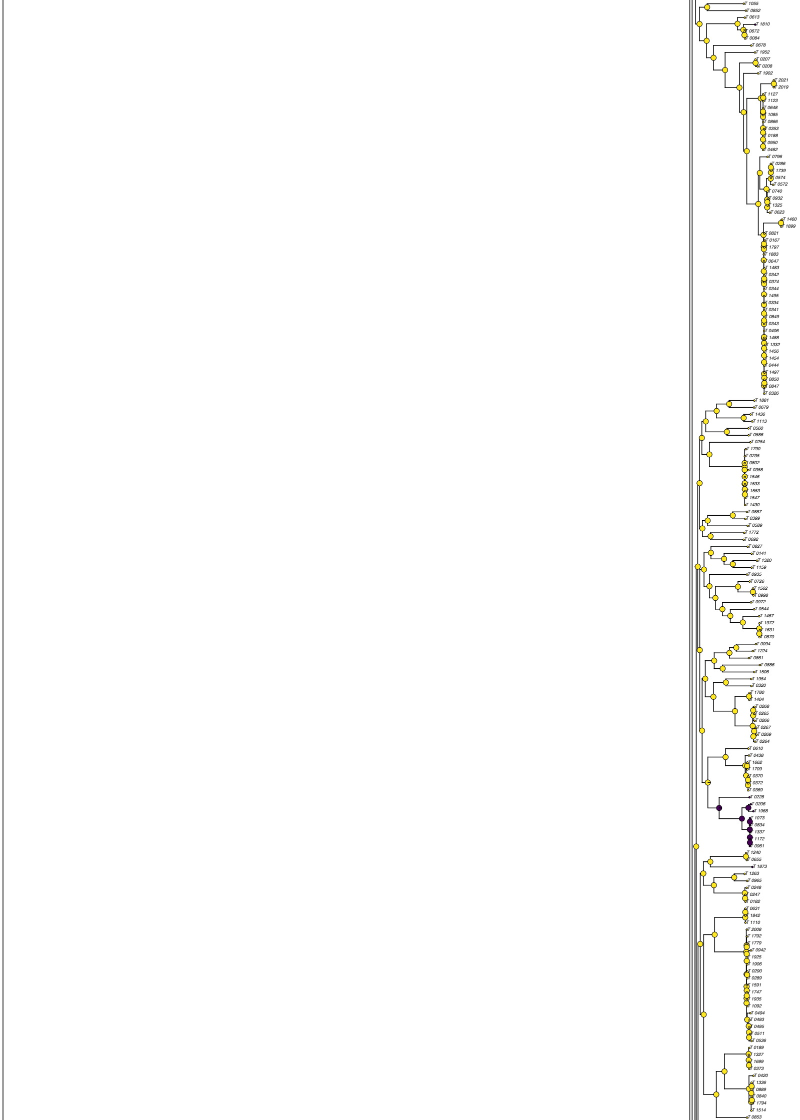
T 1055
T 0852
T 0613
T 1810
T 0672
T 0084
T 0678
T 1952
T 0207
T 0208
T 1902
T 2021
T 2019
T 1127
T 1123
T 0648
T 1085
T 0866
T 0353
T 0188
T 0950
T 0462
T 0796
T 0286
T 1739
T 0574
T 0572
T 0740
T 0932
T 1325
T 0623
T 1460
T 1899
T 0821
T 0167
T 1797
T 1883
T 0647
T 1483
T 0342
T 0374
T 0344
T 1495
T 0334
T 0341
T 0849
T 0343
T 0406
T 1488
T 1332
T 1456
T 1454
T 0444
T 1497
T 0850
T 0847
T 0326
T 1881
T 0679
T 1436
T 1113
T 0560
T 0586
T 0254
T 1790
T 0235
T 0802
T 0358
T 1546
T 1533
T 1553
T 1547
T 1430
T 0887
T 0399
T 0589
T 1772
T 0692
T 0827
T 0141
T 1320
T 1159
T 0935
T 0726
T 1562
T 0998
T 0972
T 0544
T 1467
T 1972
T 1631
T 0870
T 0094
T 1224
T 0861
T 0886
T 1506
T 1954
T 0320
T 1780
T 1404
T 0268
T 0265
T 0266
T 0267
T 0269
T 0264
T 0610
T 0438
T 1662
T 1709
T 0370
T 0372
T 0369
T 0228
T 0206
T 1968
T 1073
T 0834
T 1337
T 1172
T 0961
T 1240
T 0655
T 1873
T 1263
T 0965
T 0248
T 0247
T 0182
T 0631
T 1842
T 1110
T 2008
T 1792
T 1779
T 0942
T 1925
T 1906
T 0290
T 0289
T 1591
T 1747
T 1935
T 1092
T 0494
T 0493
T 0495
T 0511
T 0536
T 0189
T 1327
T 1699
T 0373
T 0420
T 1336
T 0889
T 0840
T 1794
T 1514
T 0853

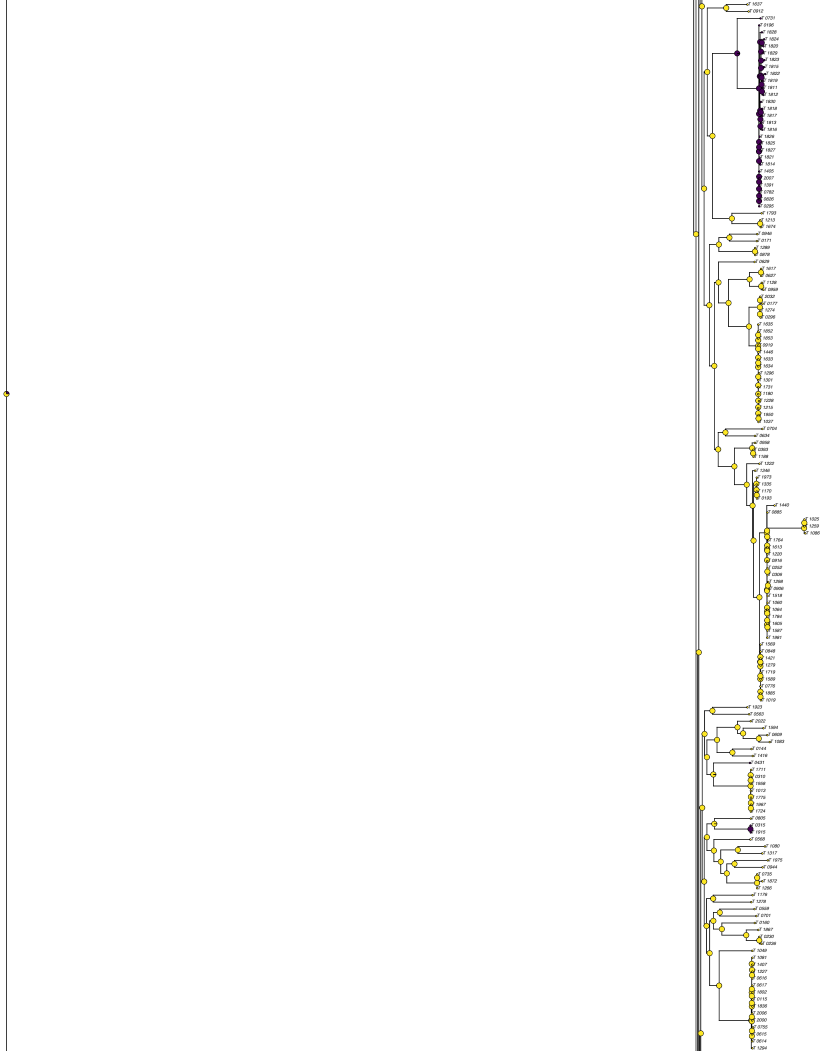
T 1637
T 0912
T 0731
T 0196
T 1828
T 1824
T 1820
T 1829
T 1823
T 1815
T 1822
T 1819
T 1811
T 1812
T 1830
T 1818
T 1817
T 1813
T 1816
T 1826
T 1825
T 1827
T 1821
T 1814
T 1405
T 2007
T 1391
T 0782
T 0826
T 0295
T 1793
T 1213
T 1674
T 0946
T 0171
T 1289
T 0878
T 0629
T 1617
T 0627
T 1128
T 0959
T 2032
T 0177
T 1274
T 0296
T 1635
T 1852
T 1853
T 0919
T 1446
T 1633
T 1634
T 1296
T 1301
T 1731
T 1180
T 1228
T 1215
T 1950
T 1037
T 0704
T 0634
T 0958
T 0393
T 1188
T 1222
T 1346
T 1973
T 1335
T 1170
T 0193
T 1440
T 0885
T 1025
T 1259
T 1086
T 1764
T 1613
T 1220
T 0916
T 0252
T 0306
T 1298
T 0906
T 1518
T 1060
T 1064
T 1784
T 1605
T 1587
T 1981
T 1569
T 0848
T 1421
T 1279
T 1719
T 1589
T 0776
T 1885
T 1019
T 1923
T 0563
T 2022
T 1594
T 0609
T 1083
T 0144
T 1416
T 0431
T 1711
T 0310
T 1958
T 1013
T 1775
T 1967
T 1724
T 0805
T 0315
T 1915
T 0568
T 1080
T 1317
T 1975
T 0944
T 0735
T 1872
T 1266
T 1176
T 1278
T 0559
T 0701
T 0160
T 1867
T 0230
T 0236
T 1049
T 1081
T 1407
T 1227
T 0616
T 0617
T 1802
T 0115
T 1836
T 2006
T 2000
T 0755
T 0615
T 0614
T 1294

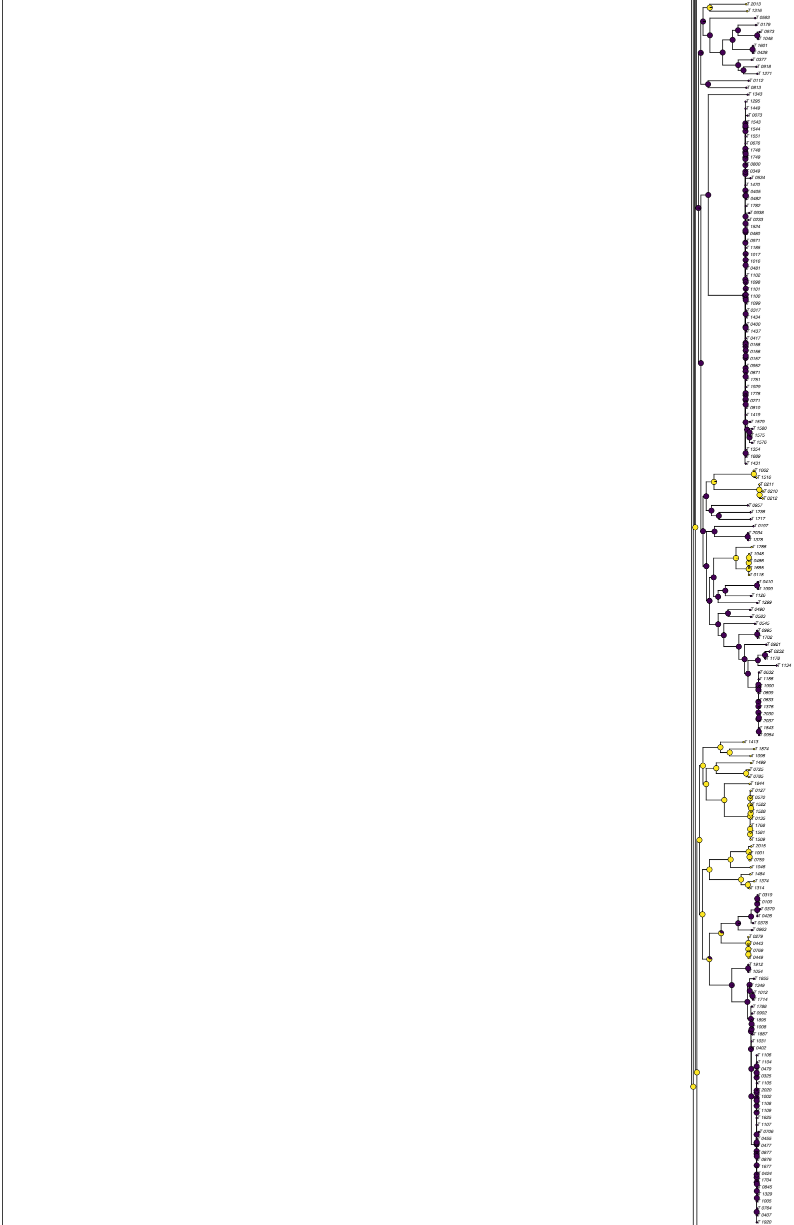
T 2013
T 1316
T 0593
T 0179
T 0973
T 1048
T 1601
T 0428
T 0377
T 0918
T 1271
T 0112
T 0813
T 1343
T 1295
T 1449
T 0073
T 1543
T 1544
T 1551
T 0676
T 1748
T 1749
T 0800
T 0349
T 0534
T 1470
T 0405
T 0482
T 1782
T 0938
T 0233
T 1524
T 0480
T 0971
T 1185
T 1017
T 1016
T 0481
T 1102
T 1098
T 1101
T 1100
T 1099
T 0317
T 1434
T 0400
T 1437
T 0417
T 0158
T 0156
T 0157
T 0952
T 0671
T 1751
T 1929
T 1778
T 0271
T 0810
T 1419
T 1579
T 1580
T 1575
T 1576
T 1354
T 1889
T 1431
T 1062
T 1516
T 0211
T 0210
T 0212
T 0957
T 1236
T 1217
T 0197
T 2034
T 1378
T 1286
T 1948
T 0486
T 1685
T 0118
T 0410
T 1909
T 1126
T 1299
T 0490
T 0583
T 0545
T 0995
T 1702
T 0921
T 0232
T 1178
T 1134
T 0632
T 1186
T 1900
T 0699
T 0633
T 1376
T 2030
T 2037
T 1843
T 0954
T 1413
T 1874
T 1096
T 1499
T 0725
T 0785
T 1844
T 0127
T 0570
T 1522
T 1528
T 0135
T 1768
T 1581
T 1509
T 2015
T 1001
T 0759
T 1046
T 1484
T 1374
T 1314
T 0319
T 0100
T 0379
T 0426
T 0378
T 0963
T 0279
T 0443
T 0769
T 0449
T 1912
T 1054
T 1855
T 1349
T 1012
T 1714
T 1788
T 0902
T 1895
T 1008
T 1887
T 1031
T 0402
T 1106
T 1104
T 0479
T 0325
T 1105
T 2020
T 1002
T 1108
T 1109
T 1625
T 1107
T 0706
T 0455
T 0477
T 0877
T 0876
T 1677
T 0424
T 1704
T 0845
T 1329
T 1005
T 0764
T 0407
T 1920

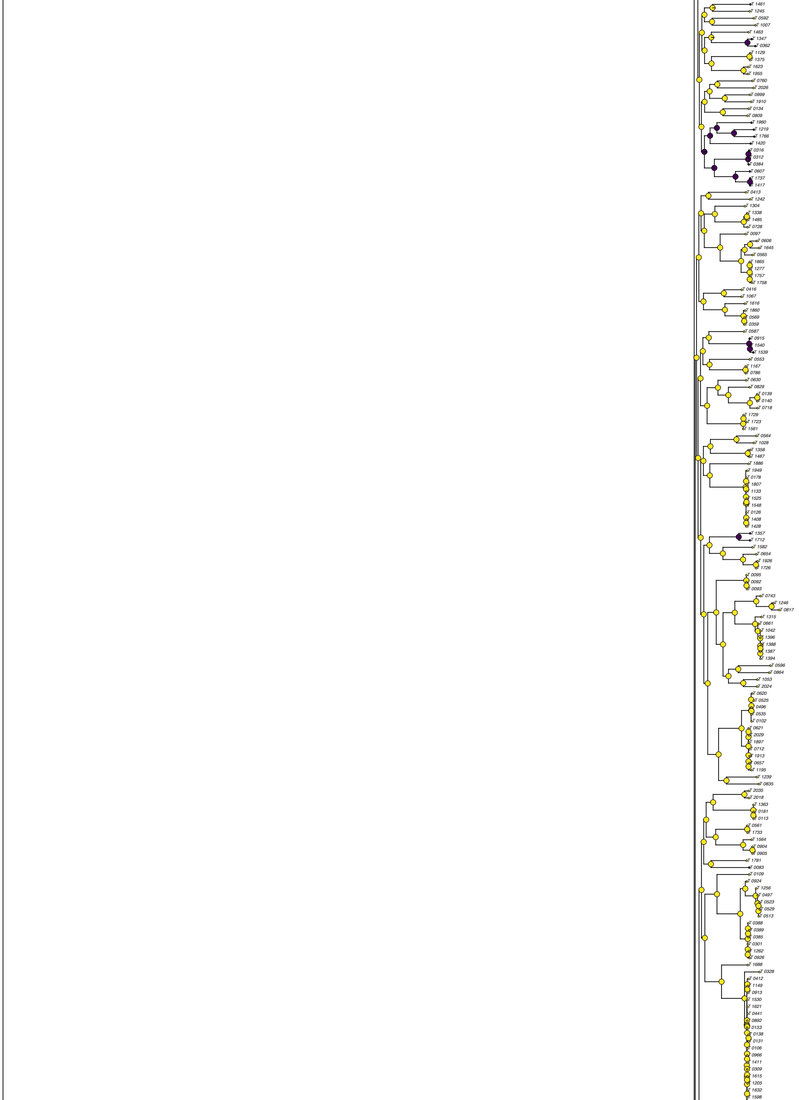
T 1461
T 1245
T 0592
T 1007
T 1463
T 1347
T 0362
T 1129
T 1375
T 1623
T 1955
T 0760
T 2026
T 0999
T 1910
T 0134
T 0809
T 1960
T 1219
T 1766
T 1420
T 0316
T 0312
T 0384
T 0607
T 1737
T 1417
T 0413
T 1242
T 1304
T 1338
T 1485
T 0728
T 0097
T 0606
T 1645
T 0565
T 1865
T 1277
T 1757
T 1758
T 0416
T 1067
T 1616
T 1860
T 0569
T 0359
T 0587
T 0915
T 1540
T 1539
T 0553
T 1167
T 0786
T 0630
T 0829
T 0139
T 0140
T 0718
T 1729
T 1723
T 1561
T 0564
T 1028
T 1358
T 1487
T 1886
T 1949
T 0178
T 1807
T 1133
T 1525
T 1548
T 0126
T 1408
T 1428
T 1357
T 1712
T 1582
T 0654
T 1926
T 1726
T 0095
T 0092
T 0093
T 0743
T 1246
T 0817
T 1315
T 0661
T 1042
T 1396
T 1388
T 1387
T 1394
T 0596
T 0864
T 1053
T 2024
T 0620
T 0525
T 0496
T 0535
T 0102
T 0621
T 2029
T 1897
T 0712
T 1913
T 0657
T 1195
T 1239
T 0835
T 2035
T 2018
T 1363
T 0181
T 0113
T 0561
T 1733
T 1564
T 0904
T 0905
T 1781
T 0083
T 0109
T 0924
T 1256
T 0497
T 0523
T 0529
T 0513
T 0388
T 0389
T 0385
T 0301
T 1262
T 0926
T 1688
T 0328
T 0412
T 1149
T 0913
T 1530
T 1621
T 0441
T 0892
T 0133
T 0138
T 0131
T 0106
T 0966
T 1411
T 0309
T 1615
T 1205
T 1632
T 1598

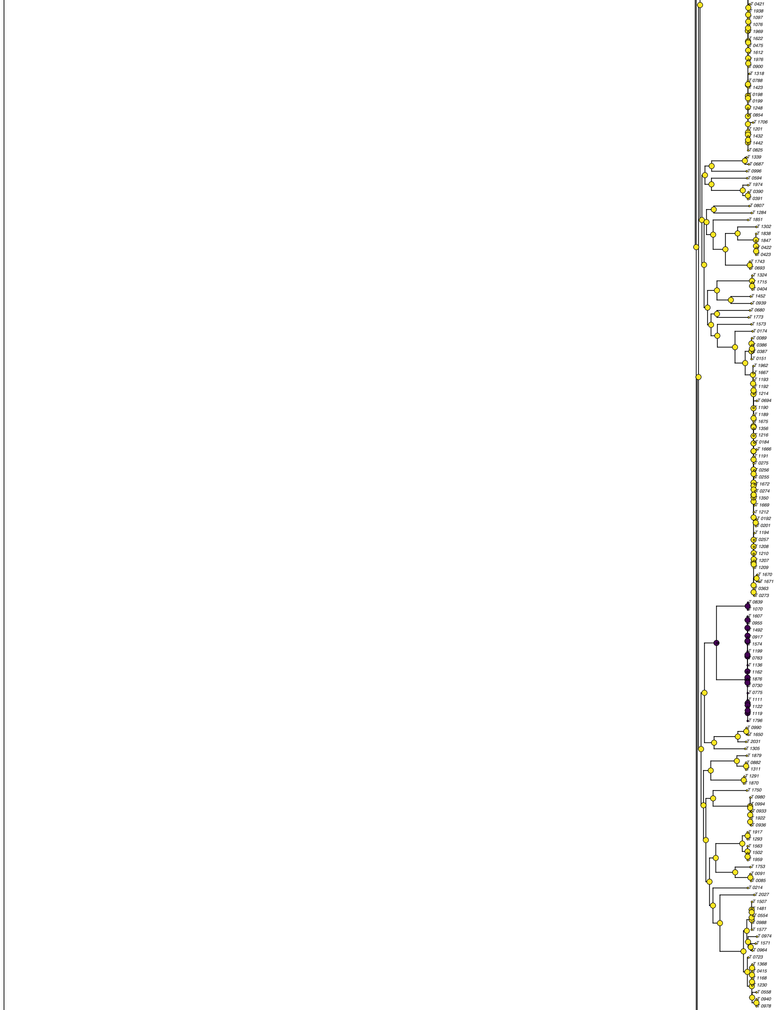
T 0421
T 1938
T 1097
T 1076
T 1969
T 1622
T 0475
T 1612
T 1976
T 0900
T 1318
T 0788
T 1423
T 0198
T 0199
T 1248
T 0854
T 1706
T 1201
T 1432
T 1442
T 0825
T 1339
T 0687
T 0996
T 0594
T 1974
T 0390
T 0391
T 0807
T 1284
T 1851
T 1302
T 1838
T 1847
T 0422
T 0423
T 1743
T 0693
T 1324
T 1715
T 0404
T 1452
T 0939
T 0680
T 1773
T 1573
T 0174
T 0089
T 0386
T 0387
T 0151
T 1962
T 1667
T 1193
T 1192
T 1214
T 0694
T 1190
T 1189
T 1675
T 1356
T 1216
T 0184
T 1666
T 1191
T 0275
T 0256
T 0255
T 1672
T 0274
T 1350
T 1669
T 1212
T 0192
T 0201
T 1194
T 0257
T 1208
T 1210
T 1207
T 1209
T 1670
T 1671
T 0363
T 0273
T 0839
T 1070
T 1607
T 0955
T 1492
T 0917
T 1574
T 1199
T 0763
T 1136
T 1162
T 1876
T 0730
T 0775
T 1111
T 1122
T 1119
T 1796
T 0990
T 1650
T 2031
T 1305
T 1879
T 0882
T 1311
T 1291
T 1870
T 1750
T 0980
T 0994
T 0933
T 1922
T 0936
T 1917
T 1293
T 1563
T 1502
T 1959
T 1753
T 0091
T 0085
T 0214
T 2027
T 1507
T 1481
T 0554
T 0988
T 1577
T 0974
T 1571
T 0964
T 0723
T 1368
T 0415
T 1168
T 1230
T 0558
T 0940
T 0978

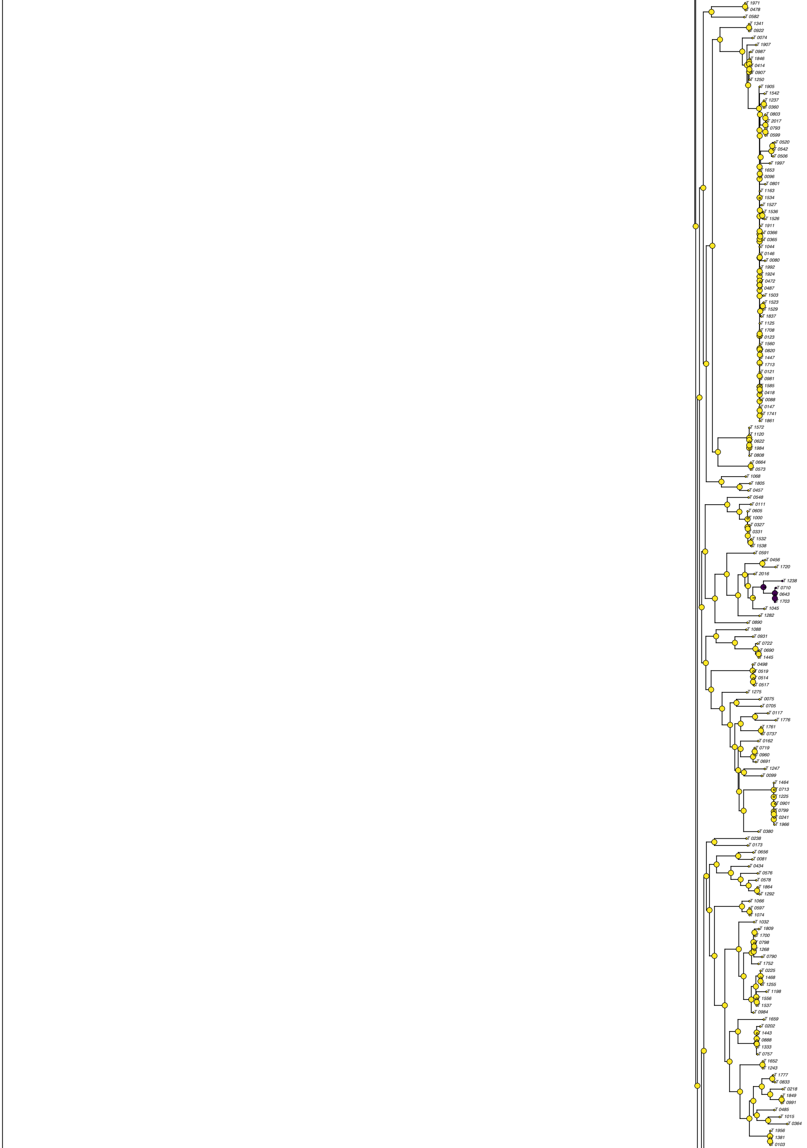
T 1971
T 0478
T 0582
T 1341
T 0922
T 0074
T 1907
T 0987
T 1846
T 0414
T 0907
T 1250
T 1905
T 1542
T 1237
T 0360
T 0803
T 2017
T 0793
T 0599
T 0520
T 0542
T 0506
T 1997
T 1653
T 0096
T 0801
T 1163
T 1534
T 1527
T 1536
T 1526
T 1911
T 0366
T 0365
T 1044
T 0146
T 0080
T 1992
T 1924
T 0472
T 0487
T 1503
T 1523
T 1529
T 1837
T 1125
T 1708
T 0123
T 1560
T 0820
T 1447
T 1713
T 0121
T 0981
T 1585
T 0418
T 0088
T 0147
T 1741
T 1861
T 1572
T 1120
T 0622
T 1984
T 0808
T 0664
T 0573
T 1068
T 1805
T 0457
T 0548
T 0111
T 0605
T 1000
T 0327
T 0331
T 1532
T 1538
T 0591
T 0456
T 1720
T 2016
T 1238
T 0710
T 0643
T 1703
T 1045
T 1282
T 0890
T 1088
T 0931
T 0722
T 0690
T 1445
T 0498
T 0519
T 0514
T 0517
T 1275
T 0075
T 0705
T 0117
T 1776
T 1761
T 0737
T 0162
T 0719
T 0960
T 0691
T 1247
T 0099
T 1464
T 0713
T 1225
T 0901
T 0799
T 0241
T 1966
T 0380
T 0238
T 0173
T 0656
T 0081
T 0434
T 0576
T 0578
T 1864
T 1292
T 1066
T 0597
T 1074
T 1032
T 1809
T 1700
T 0798
T 1268
T 0790
T 1752
T 0225
T 1468
T 1255
T 1198
T 1556
T 1537
T 0984
T 1659
T 0202
T 1443
T 0888
T 1333
T 0757
T 1652
T 1243
T 1777
T 0833
T 0218
T 1849
T 0991
T 0485
T 1015
T 0364
T 1956
T 1381
T 0103

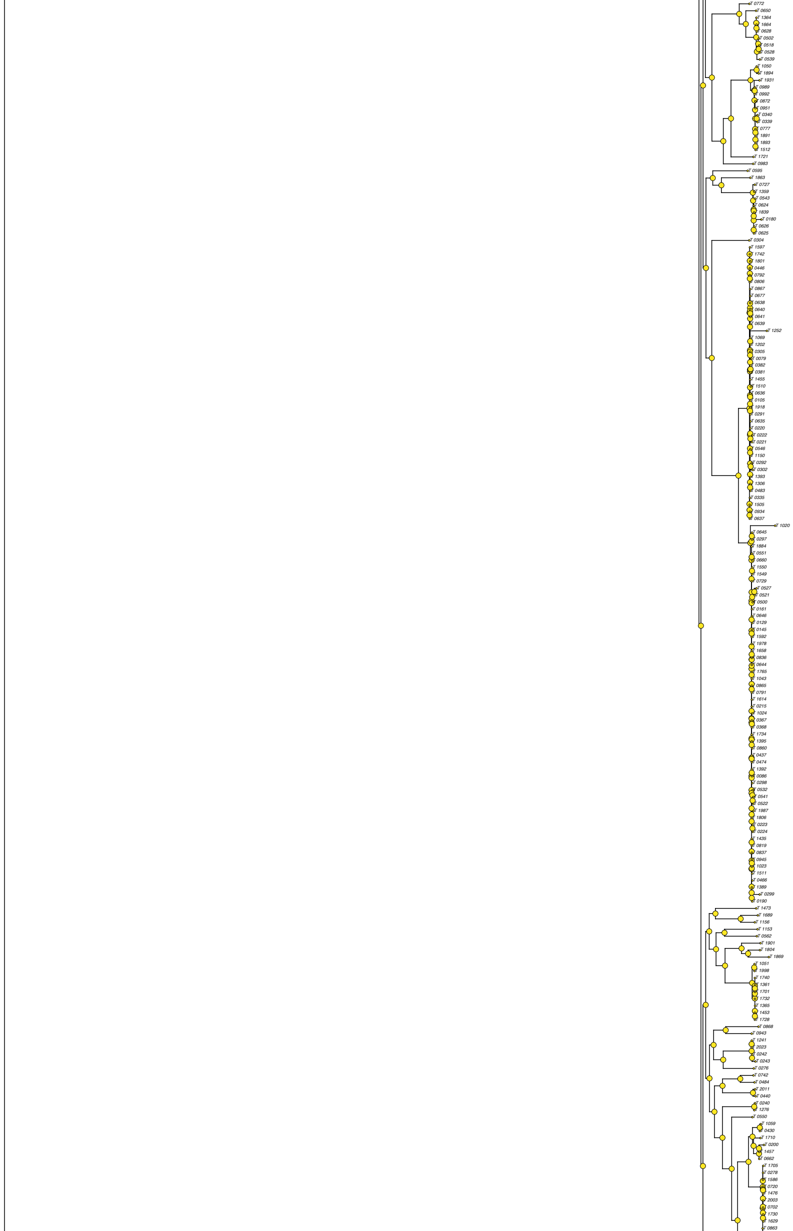
T 0772
T 0650
T 1364
T 1664
T 0628
T 0502
T 0518
T 0528
T 0539
T 1050
T 1894
T 1931
T 0989
T 0992
T 0872
T 0951
T 0340
T 0339
T 0777
T 1891
T 1893
T 1512
T 1721
T 0983
T 0595
T 1863
T 0727
T 1359
T 0543
T 0624
T 1839
T 0180
T 0626
T 0625
T 0304
T 1597
T 1742
T 1801
T 0446
T 0792
T 0806
T 0867
T 0677
T 0638
T 0640
T 0641
T 0639
T 1252
T 1069
T 1202
T 0305
T 0079
T 0382
T 0381
T 1455
T 1510
T 0636
T 0105
T 1918
T 0291
T 0635
T 0220
T 0222
T 0221
T 0546
T 1150
T 0292
T 0302
T 1393
T 1306
T 0483
T 0335
T 1505
T 0934
T 0637
T 1020
T 0645
T 0297
T 1884
T 0551
T 0660
T 1550
T 1549
T 0729
T 0527
T 0521
T 0500
T 0161
T 0646
T 0129
T 0145
T 1592
T 1978
T 1658
T 0836
T 0644
T 1765
T 1043
T 0865
T 0791
T 1614
T 0215
T 1024
T 0367
T 0368
T 1734
T 1395
T 0860
T 0437
T 0474
T 1392
T 0086
T 0298
T 0532
T 0541
T 0522
T 1987
T 1806
T 0223
T 0224
T 1435
T 0819
T 0837
T 0945
T 1023
T 1511
T 0466
T 1389
T 0299
T 0190
T 1473
T 1689
T 1156
T 1153
T 0562
T 1901
T 1804
T 1869
T 1051
T 1998
T 1740
T 1361
T 1701
T 1732
T 1365
T 1453
T 1728
T 0868
T 0943
T 1241
T 2023
T 0242
T 0243
T 0276
T 0742
T 0484
T 2011
T 0440
T 0240
T 1276
T 0550
T 1059
T 0430
T 1710
T 0200
T 1457
T 0662
T 1705
T 0278
T 1586
T 0720
T 1476
T 2003
T 0702
T 1730
T 1629
T 0863

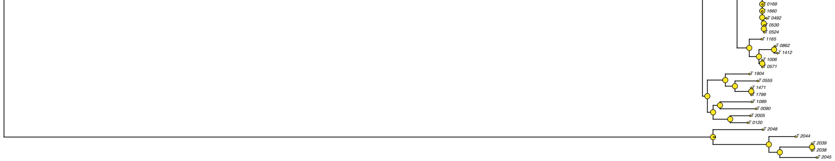
T 0169
T 1660
T 0492
T 0530
T 0524
T 1165
T 0862
T 1412
T 1006
T 0571
T 1904
T 0555
T 1471
T 1799
T 1089
T 0090
T 2005
T 0120
T 2048
T 2044
T 2039
T 2038
T 2045

Effector gene: vgrG2b

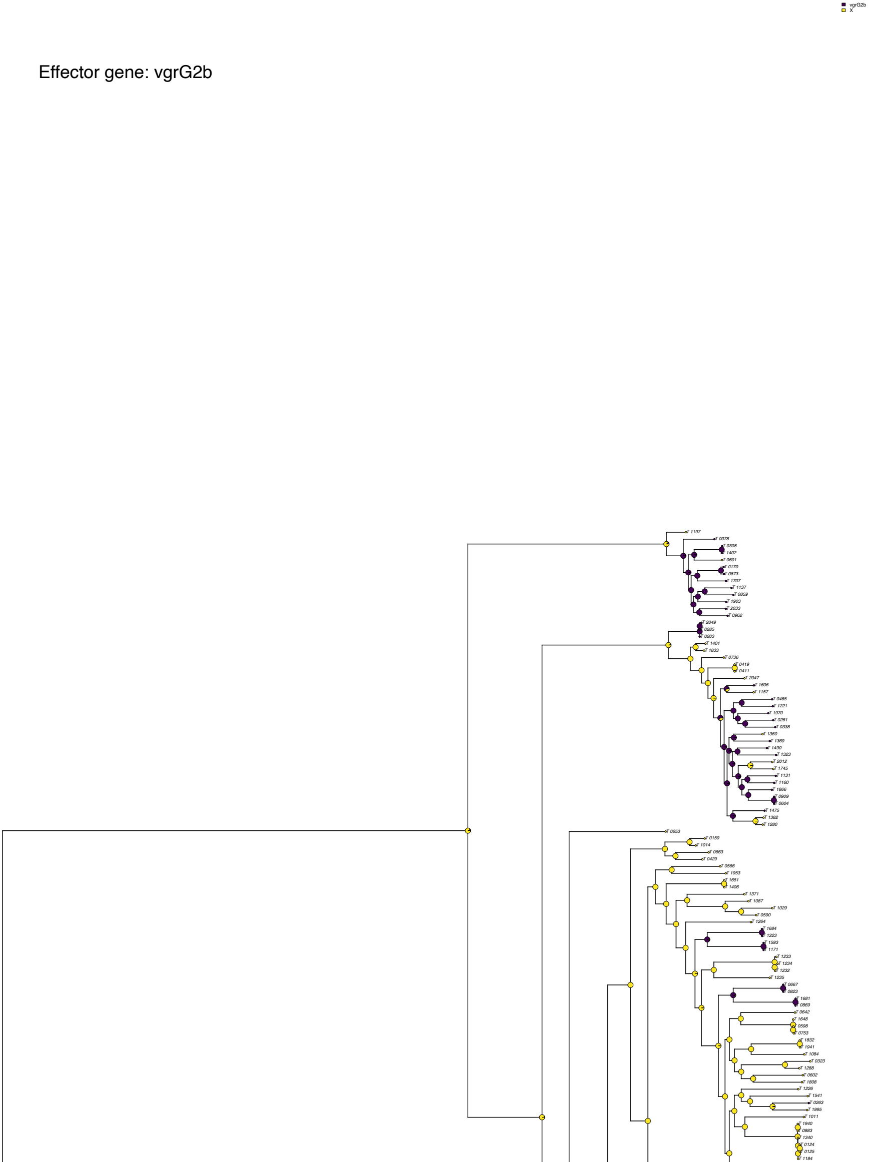

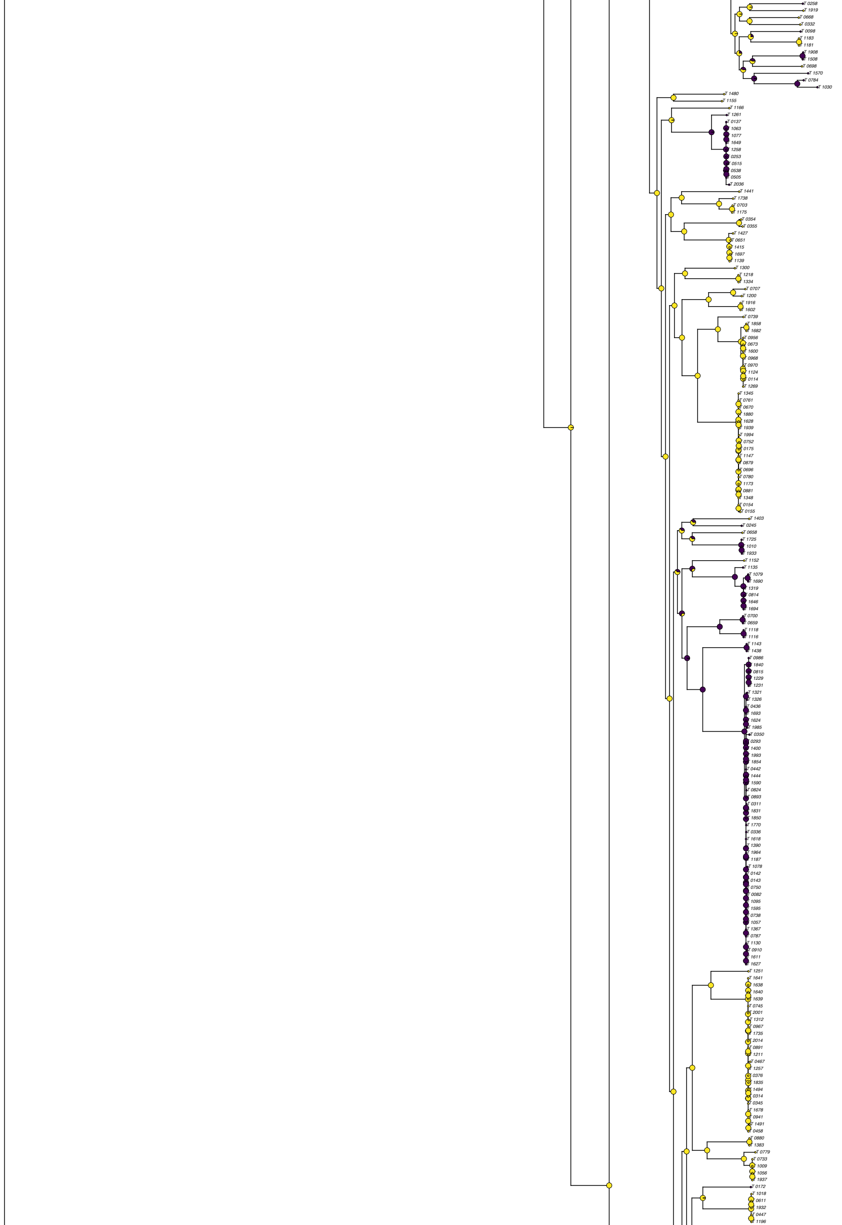
T 0258
T 1919
T 0668
T 0332
T 0098
T 1183
1181
T 1908
1508
T 0698
T 1570
T 0784
T 1030
T 1480
T 1155
T 1166
T 1261
T 0137
1063
1077
1649
1258
0253
0515
0538
0505
T 2036
T 1441
T 1738
T 0703
T 1175
T 0354
T 0355
T 1427
T 0651
1415
1697
1139
T 1300
T 1218
1334
T 0707
T 1200
T 1916
T 1602
T 0739
T 1858
T 1682
T 0956
0673
1600
0968
T 0970
1124
0114
T 1269
T 1345
T 0761
0670
1880
1628
1939
T 1994
0752
0175
T 1147
0879
T 0696
T 0780
1173
0881
1348
0154
T 0155
T 1403
T 0245
T 0658
T 1725
1010
T 1933
T 1152
T 1135
T 1079
T 1690
1319
0814
1646
1694
T 0700
T 0659
T 1118
T 1116
T 1143
T 1438
T 0986
1840
0815
1229
T 1231
T 1321
T 1326
T 0436
1693
1624
T 1985
T 0350
0293
1400
1993
1854
T 0442
1444
1590
T 0824
0893
T 0311
1831
1850
T 1770
T 0336
T 1618
T 1390
1964
1187
T 1078
0142
0143
0750
0082
1095
1595
0738
1057
T 1367
0787
T 1130
T 0910
1611
1627
T 1251
T 1641
1638
1640
1639
T 0745
2001
T 1312
T 0967
1735
2014
T 0891
1211
T 0467
T 1257
0376
1835
1494
0314
0345
T 1678
0941
T 1491
0458
T 0880
1383
T 0779
T 0733
1009
1056
1937
T 0172
T 1018
0611
1932
T 0447
1196

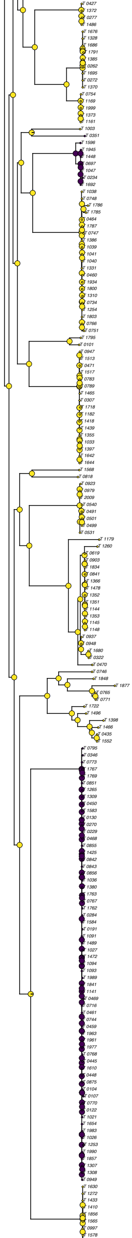
T 0427
T 1372
T 0277
T 1486
T 1676
T 1328
T 1686
T 1791
T 1385
T 0262
T 1695
T 0272
T 1370
T 0754
T 1169
T 1999
T 1373
T 1161
T 1003
T 0351
T 1596
T 1945
T 1448
T 0697
T 1047
T 0234
T 1692
T 1038
T 0748
T 1786
T 1785
T 0464
T 1787
T 0747
T 1386
T 1039
T 1041
T 1040
T 1331
T 0460
T 1934
T 1800
T 1310
T 0734
T 1254
T 1803
T 0766
T 0751
T 1795
T 0101
T 0947
T 1513
T 0471
T 1517
T 0783
T 0789
T 1465
T 0307
T 1718
T 1182
T 1418
T 1439
T 1355
T 1033
T 1397
T 1642
T 1644
T 1568
T 0818
T 0923
T 0979
T 2009
T 0540
T 0491
T 0501
T 0499
T 0531
T 1179
T 1260
T 0619
T 0903
T 1834
T 0841
T 1366
T 1478
T 1352
T 1351
T 1144
T 1353
T 1145
T 1148
T 0937
T 0948
T 1680
T 0322
T 0470
T 0746
T 1848
T 1877
T 0765
T 0771
T 1722
T 1496
T 1398
T 1466
T 0435
T 1552
T 0795
T 0346
T 0773
T 1767
T 1769
T 0851
T 1265
T 1309
T 0450
T 1583
T 0130
T 0270
T 0229
T 0468
T 0855
T 1425
T 0842
T 0843
T 0856
T 1036
T 1380
T 1763
T 0767
T 1762
T 0284
T 1584
T 0191
T 1091
T 1489
T 1027
T 1472
T 1094
T 1093
T 1989
T 1841
T 1141
T 0469
T 0716
T 0461
T 0744
T 0459
T 1963
T 1961
T 1977
T 0768
T 0445
T 1610
T 0448
T 0875
T 0104
T 0107
T 0770
T 0122
T 1021
T 1654
T 1983
T 1026
T 1253
T 1990
T 1857
T 1307
T 1308
T 0949
T 1630
T 1272
T 1433
T 1410
T 1856
T 1565
T 0997
T 1578

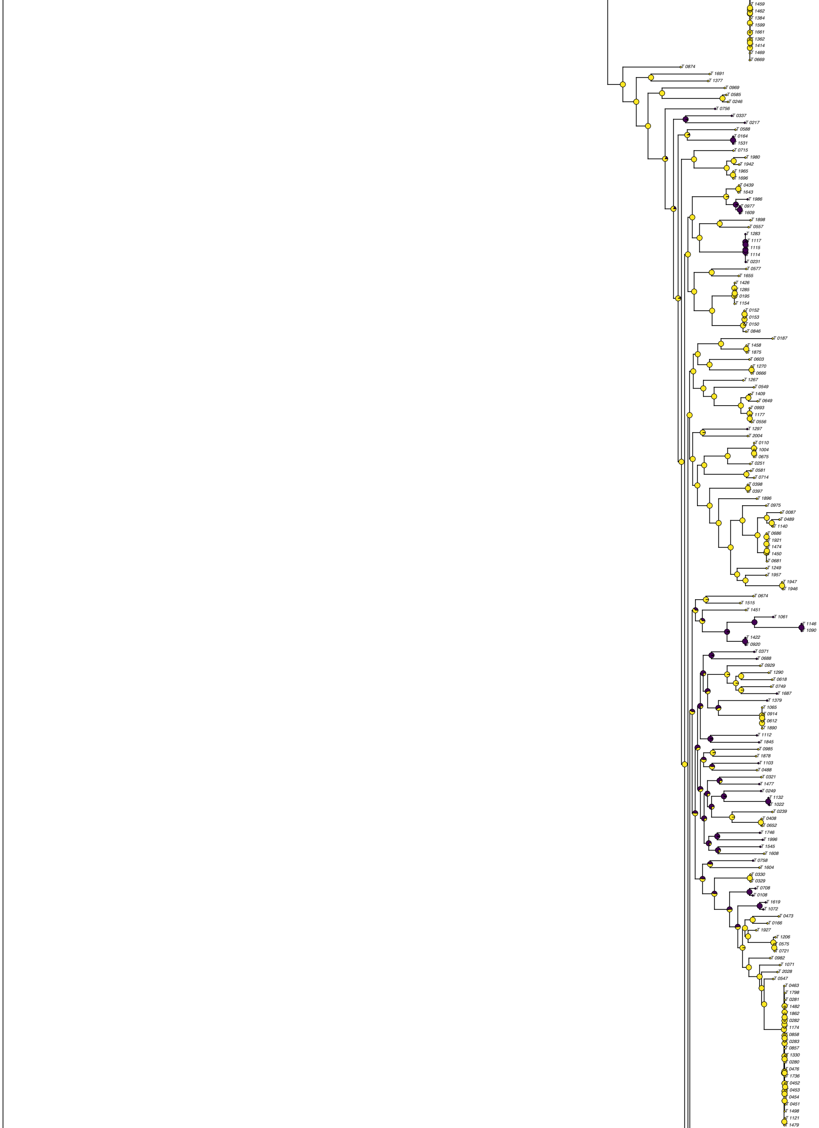
T 1459
T 1462
T 1384
T 1599
T 1661
T 1362
T 1414
T 1469
T 0669
T 0874
T 1691
T 1377
T 0969
T 0585
T 0246
T 0756
T 0337
T 0217
T 0588
T 0164
T 1531
T 0715
T 1980
T 1942
T 1965
T 1696
T 0439
T 1643
T 1986
T 0977
T 1609
T 1898
T 0557
T 1283
T 1117
T 1115
T 1114
T 0231
T 0577
T 1655
T 1426
T 1285
T 0195
T 1154
T 0152
T 0153
T 0150
T 0846
T 0187
T 1458
T 1875
T 0603
T 1270
T 0666
T 1267
T 0549
T 1409
T 0649
T 0993
T 1177
T 0556
T 1297
T 2004
T 0110
T 1004
T 0675
T 0251
T 0581
T 0714
T 0398
T 0397
T 1896
T 0975
T 0087
T 0489
T 1140
T 0686
T 1921
T 1474
T 1450
T 0681
T 1249
T 1957
T 1947
T 1946
T 0674
T 1515
T 1451
T 1061
T 1146
T 1090
T 1422
T 0920
T 0371
T 0688
T 0929
T 1290
T 0618
T 0749
T 1687
T 1379
T 1065
T 0914
T 0612
T 1890
T 1112
T 1845
T 0985
T 1878
T 1103
T 0488
T 0321
T 1477
T 0249
T 1132
T 1022
T 0239
T 0408
T 0652
T 1746
T 1996
T 1545
T 1608
T 0758
T 1604
T 0330
T 0329
T 0708
T 0108
T 1619
T 1072
T 0473
T 0166
T 1927
T 1206
T 0575
T 0721
T 0982
T 1071
T 2028
T 0547
T 0463
T 1798
T 0281
T 1482
T 1862
T 0282
T 1174
T 0858
T 0283
T 0857
T 1330
T 0280
T 0476
T 1736
T 0452
T 0453
T 0454
T 0451
T 1498
T 1121
T 1479

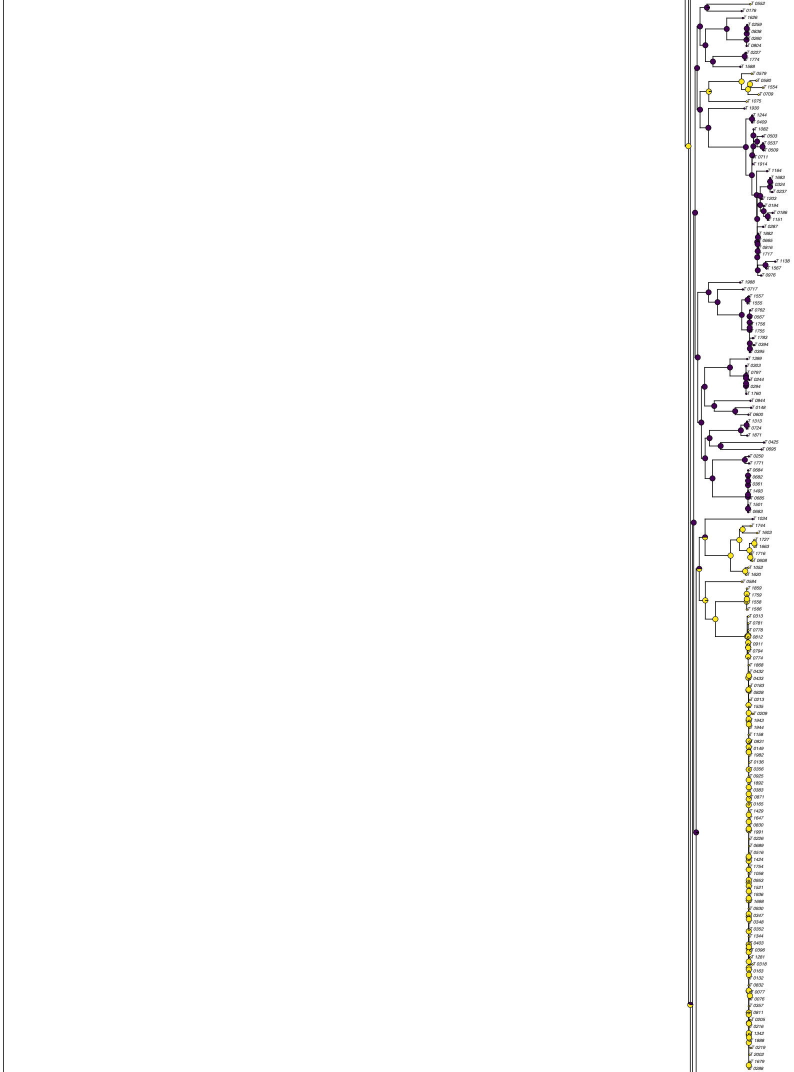
T 0552
T 0176
T 1626
T 0259
0838
0260
T 0804
T 0227
T 1774
T 1588
T 0579
T 0580
T 1554
T 0709
T 1075
T 1930
T 1244
T 0409
T 1082
T 0503
T 0537
T 0509
T 0711
T 1914
T 1164
T 1683
0324
T 0237
T 1203
T 0194
T 0186
T 1151
T 0287
T 1882
0665
T 0816
1717
T 1138
T 1567
T 0976
T 1988
T 0717
T 1557
1555
T 0762
0567
T 1756
1755
T 1783
T 0394
0395
T 1399
T 0303
T 0797
T 0244
0294
T 1760
T 0844
T 0148
T 0600
T 1313
T 0724
T 1871
T 0425
T 0695
T 0250
T 1771
T 0684
0682
0361
T 1493
T 0685
T 1501
T 0683
T 1034
T 1744
T 1603
T 1727
T 1663
T 1716
T 0608
T 1052
T 1620
T 0584
T 1859
1759
1558
T 1566
T 0313
T 0781
T 0778
0812
0911
T 0794
0774
T 1868
T 0432
0433
T 0183
T 0828
T 0213
1535
T 0209
1943
1944
T 1158
0831
0149
1982
T 0136
0356
T 0925
1892
0383
T 0871
0165
T 1429
1647
0830
1991
T 0226
T 0689
T 0516
1424
T 1754
T 1058
0953
1521
1936
1698
T 0930
0347
0348
0352
T 1344
T 0403
T 0396
T 1281
T 0318
0163
T 0132
T 0832
T 0077
0076
T 0357
0811
T 0205
T 0216
1342
1888
T 0219
T 2002
T 1679
0288

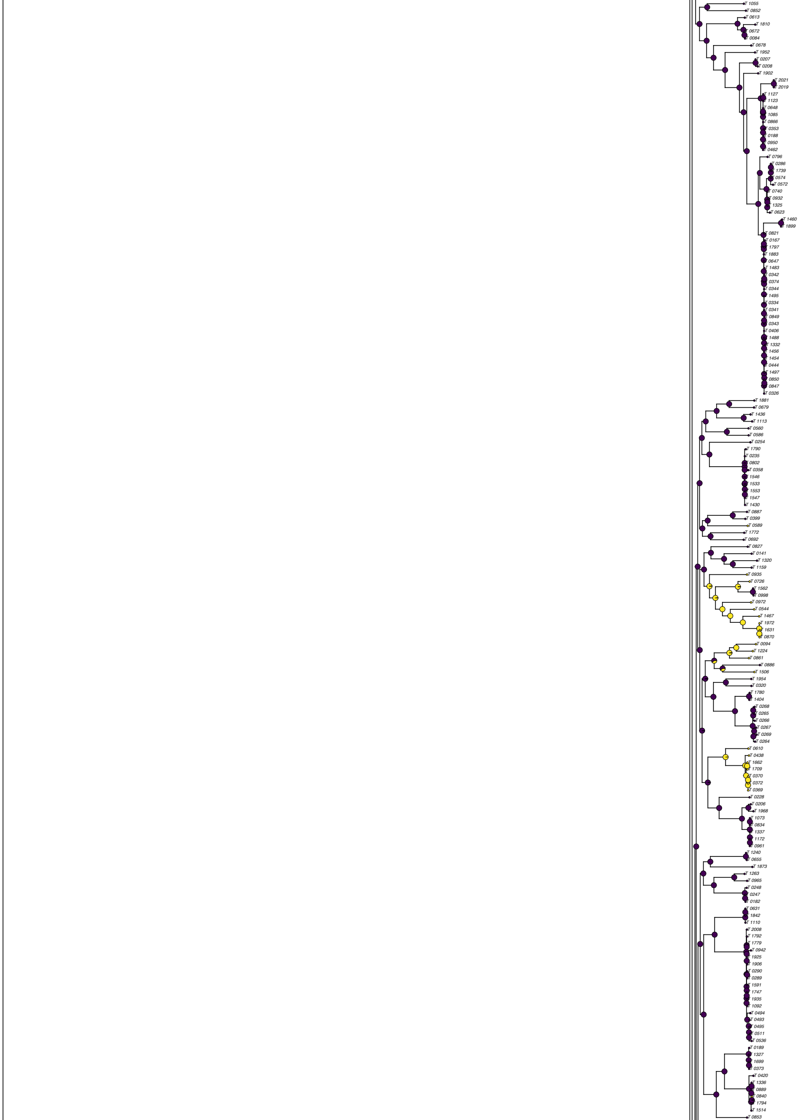
T 1055
T 0852
T 0613
T 1810
T 0672
T 0084
T 0678
T 1952
T 0207
T 0208
T 1902
T 2021
T 2019
T 1127
T 1123
T 0648
T 1085
T 0866
T 0353
T 0188
T 0950
T 0462
T 0796
T 0286
T 1739
T 0574
T 0572
T 0740
T 0932
T 1325
T 0623
T 1460
T 1899
T 0821
T 0167
T 1797
T 1883
T 0647
T 1483
T 0342
T 0374
T 0344
T 1495
T 0334
T 0341
T 0849
T 0343
T 0406
T 1488
T 1332
T 1456
T 1454
T 0444
T 1497
T 0850
T 0847
T 0326
T 1881
T 0679
T 1436
T 1113
T 0560
T 0586
T 0254
T 1790
T 0235
T 0802
T 0358
T 1546
T 1533
T 1553
T 1547
T 1430
T 0887
T 0399
T 0589
T 1772
T 0692
T 0827
T 0141
T 1320
T 1159
T 0935
T 0726
T 1562
T 0998
T 0972
T 0544
T 1467
T 1972
T 1631
T 0870
T 0094
T 1224
T 0861
T 0886
T 1506
T 1954
T 0320
T 1780
T 1404
T 0268
T 0265
T 0266
T 0267
T 0269
T 0264
T 0610
T 0438
T 1662
T 1709
T 0370
T 0372
T 0369
T 0228
T 0206
T 1968
T 1073
T 0834
T 1337
T 1172
T 0961
T 1240
T 0655
T 1873
T 1263
T 0965
T 0248
T 0247
T 0182
T 0631
T 1842
T 1110
T 2008
T 1792
T 1779
T 0942
T 1925
T 1906
T 0290
T 0289
T 1591
T 1747
T 1935
T 1092
T 0494
T 0493
T 0495
T 0511
T 0536
T 0189
T 1327
T 1699
T 0373
T 0420
T 1336
T 0889
T 0840
T 1794
T 1514
T 0853

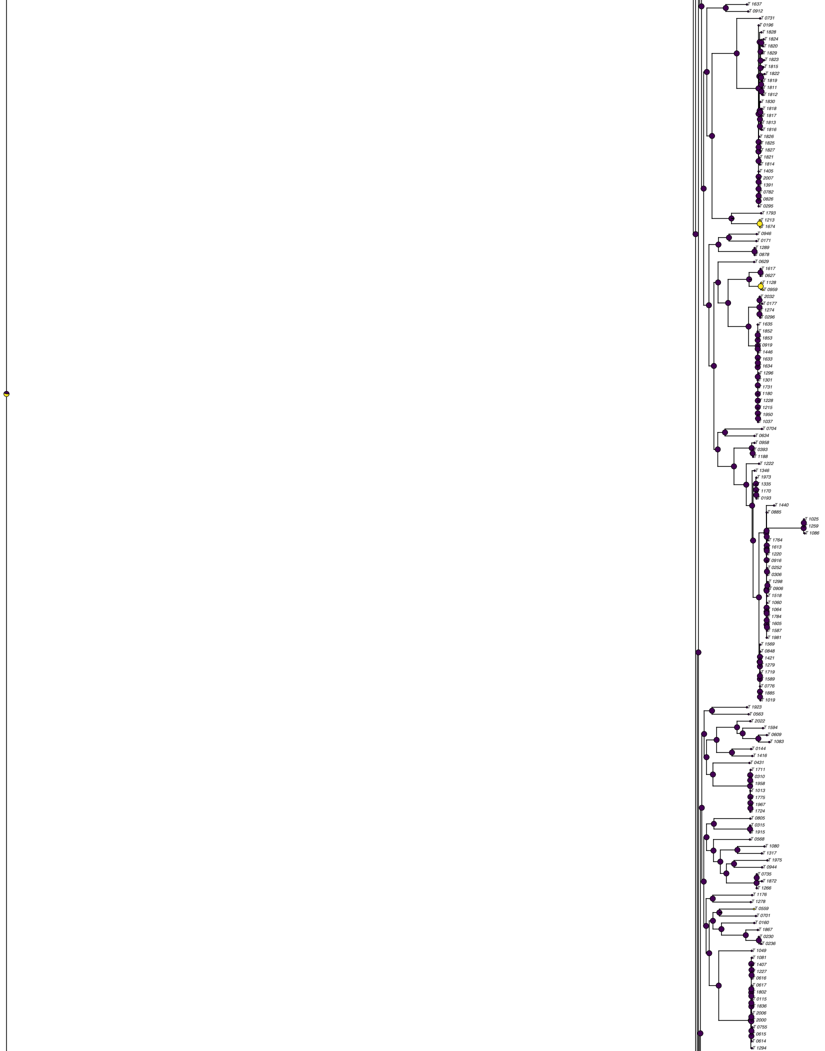
T 1637
T 0912
T 0731
T 0196
T 1828
T 1824
T 1820
T 1829
T 1823
T 1815
T 1822
T 1819
T 1811
T 1812
T 1830
T 1818
T 1817
T 1813
T 1816
T 1826
T 1825
T 1827
T 1821
T 1814
T 1405
2007
1391
0782
0826
T 0295
T 1793
T 1213
T 1674
T 0946
T 0171
T 1289
T 0878
T 0629
T 1617
T 0627
T 1128
T 0959
T 2032
T 0177
T 1274
T 0296
T 1635
T 1852
1853
0919
T 1446
1633
1634
T 1296
T 1301
T 1731
1180
T 1228
1215
1950
T 1037
T 0704
T 0634
T 0958
T 0393
T 1188
T 1222
T 1346
T 1973
1335
1170
T 0193
T 1440
T 0885
T 1025
1259
T 1086
T 1764
1613
T 1220
0916
T 0252
T 0306
T 1298
T 0906
T 1518
T 1060
1064
T 1784
1605
T 1587
T 1981
T 1569
T 0848
1421
1279
T 1719
1589
T 0776
1885
T 1019
T 1923
T 0563
T 2022
T 1594
T 0609
T 1083
T 0144
T 1416
T 0431
T 1711
0310
1958
T 1013
1775
1967
T 1724
T 0805
T 0315
T 1915
T 0568
T 1080
T 1317
T 1975
T 0944
T 0735
T 1872
T 1266
T 1176
T 1278
T 0559
T 0701
T 0160
T 1867
T 0230
T 0236
T 1049
T 1081
1407
1227
0616
T 0617
1802
T 0115
1836
T 2006
2000
T 0755
0615
T 0614
T 1294

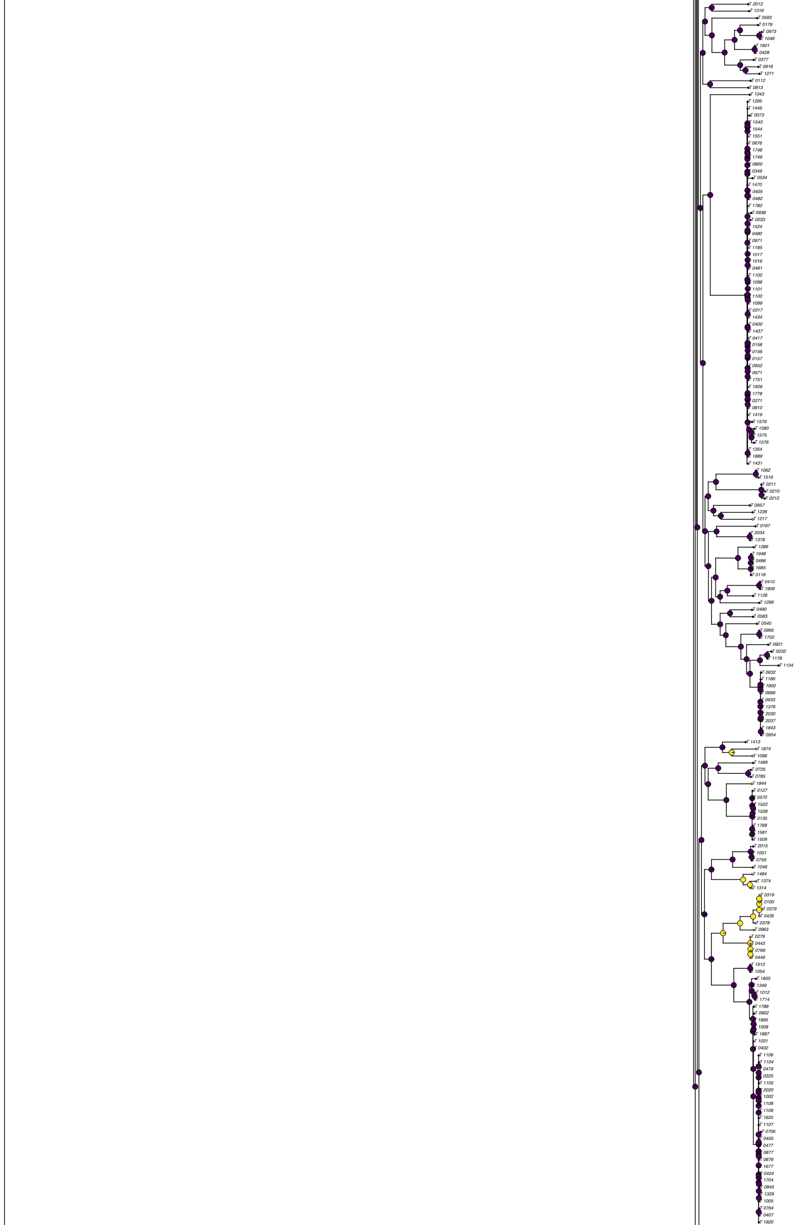
T 2013
T 1316
T 0593
T 0179
T 0973
T 1048
T 1601
T 0428
T 0377
T 0918
T 1271
T 0112
T 0813
T 1343
T 1295
T 1449
T 0073
T 1543
T 1544
T 1551
T 0676
T 1748
T 1749
T 0800
T 0349
T 0534
T 1470
T 0405
T 0482
T 1782
T 0938
T 0233
T 1524
T 0480
T 0971
T 1185
T 1017
T 1016
T 0481
T 1102
T 1098
T 1101
T 1100
T 1099
T 0317
T 1434
T 0400
T 1437
T 0417
T 0158
T 0156
T 0157
T 0952
T 0671
T 1751
T 1929
T 1778
T 0271
T 0810
T 1419
T 1579
T 1580
T 1575
T 1576
T 1354
T 1889
T 1431
T 1062
T 1516
T 0211
T 0210
T 0212
T 0957
T 1236
T 1217
T 0197
T 2034
T 1378
T 1286
T 1948
T 0486
T 1685
T 0118
T 0410
T 1909
T 1126
T 1299
T 0490
T 0583
T 0545
T 0995
T 1702
T 0921
T 0232
T 1178
T 1134
T 0632
T 1186
T 1900
T 0699
T 0633
T 1376
T 2030
T 2037
T 1843
T 0954
T 1413
T 1874
T 1096
T 1499
T 0725
T 0785
T 1844
T 0127
T 0570
T 1522
T 1528
T 0135
T 1768
T 1581
T 1509
T 2015
T 1001
T 0759
T 1046
T 1484
T 1374
T 1314
T 0319
T 0100
T 0379
T 0426
T 0378
T 0963
T 0279
T 0443
T 0769
T 0449
T 1912
T 1054
T 1855
T 1349
T 1012
T 1714
T 1788
T 0902
T 1895
T 1008
T 1887
T 1031
T 0402
T 1106
T 1104
T 0479
T 0325
T 1105
T 2020
T 1002
T 1108
T 1109
T 1625
T 1107
T 0706
T 0455
T 0477
T 0877
T 0876
T 1677
T 0424
T 1704
T 0845
T 1329
T 1005
T 0764
T 0407
T 1920

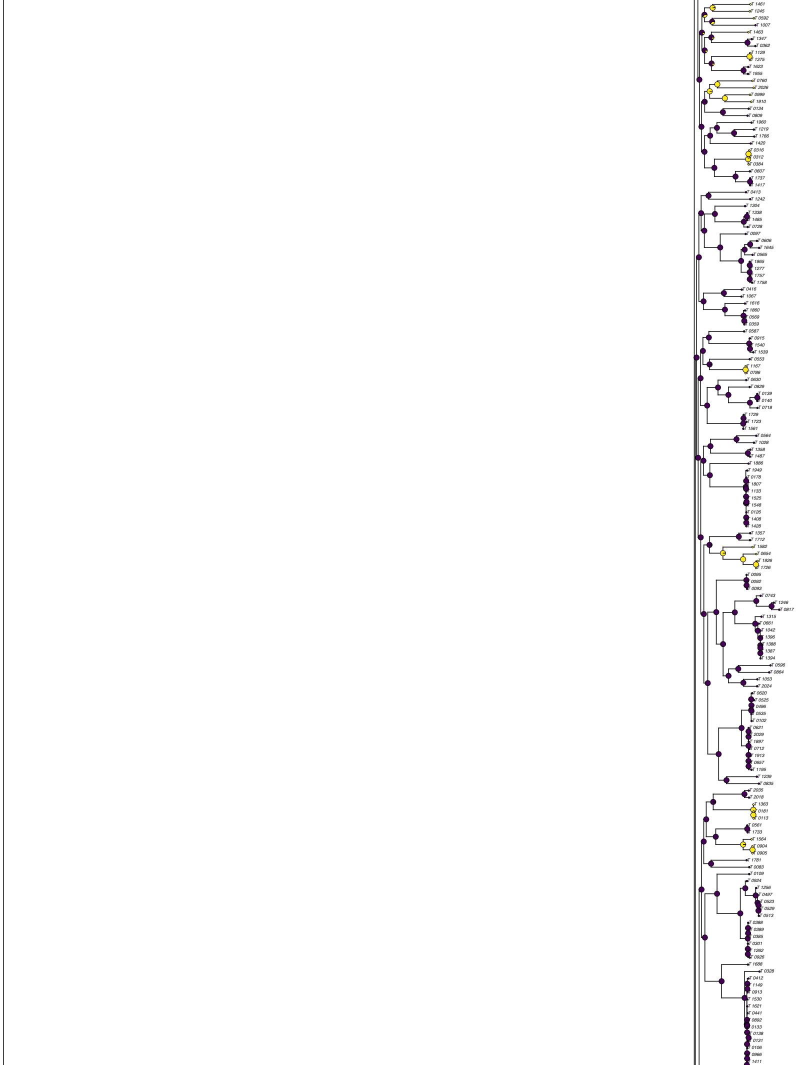
T 1461
T 1245
T 0592
T 1007
T 1463
T 1347
T 0362
T 1129
T 1375
T 1623
T 1955
T 0760
T 2026
T 0999
T 1910
T 0134
T 0809
T 1960
T 1219
T 1766
T 1420
T 0316
T 0312
T 0384
T 0607
T 1737
T 1417
T 0413
T 1242
T 1304
T 1338
T 1485
T 0728
T 0097
T 0606
T 1645
T 0565
T 1865
T 1277
T 1757
T 1758
T 0416
T 1067
T 1616
T 1860
T 0569
T 0359
T 0587
T 0915
T 1540
T 1539
T 0553
T 1167
T 0786
T 0630
T 0829
T 0139
T 0140
T 0718
T 1729
T 1723
T 1561
T 0564
T 1028
T 1358
T 1487
T 1886
T 1949
T 0178
T 1807
T 1133
T 1525
T 1548
T 0126
T 1408
T 1428
T 1357
T 1712
T 1582
T 0654
T 1926
T 1726
T 0095
T 0092
T 0093
T 0743
T 1246
T 0817
T 1315
T 0661
T 1042
T 1396
T 1388
T 1387
T 1394
T 0596
T 0864
T 1053
T 2024
T 0620
T 0525
T 0496
T 0535
T 0102
T 0621
T 2029
T 1897
T 0712
T 1913
T 0657
T 1195
T 1239
T 0835
T 2035
T 2018
T 1363
T 0181
T 0113
T 0561
T 1733
T 1564
T 0904
T 0905
T 1781
T 0083
T 0109
T 0924
T 1256
T 0497
T 0523
T 0529
T 0513
T 0388
T 0389
T 0385
T 0301
T 1262
T 0926
T 1688
T 0328
T 0412
T 1149
T 0913
T 1530
T 1621
T 0441
T 0892
T 0133
T 0138
T 0131
T 0106
T 0966
T 1411

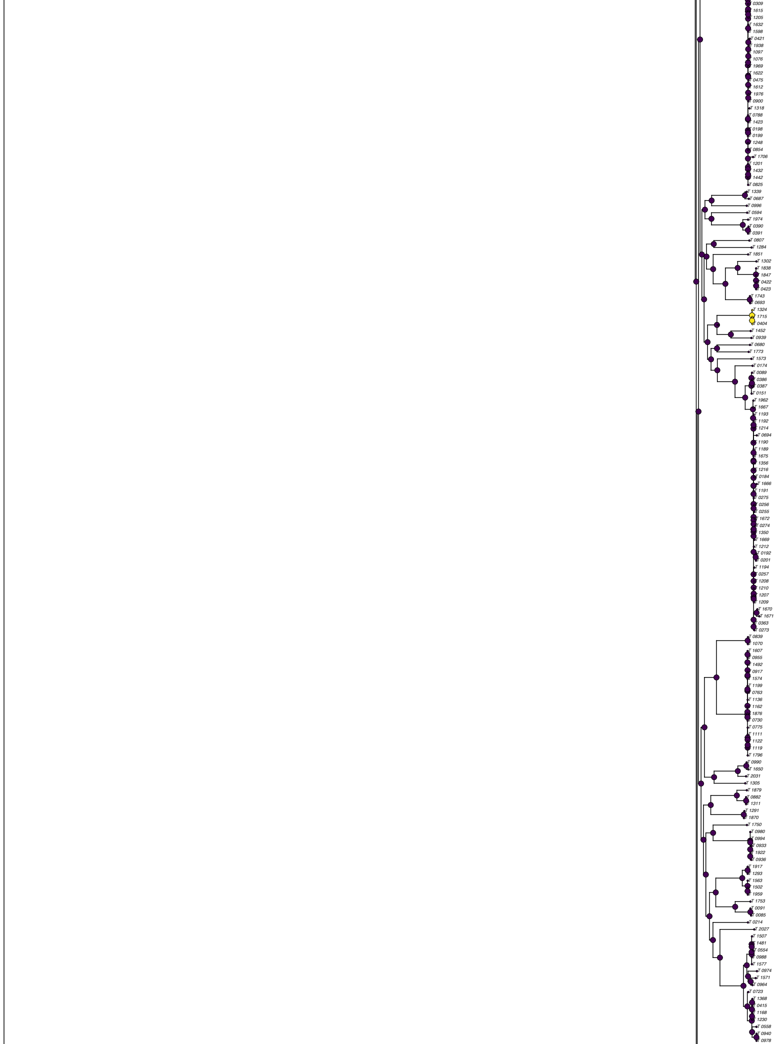
0309
T 1615
T 1205
T 1632
T 1598
T 0421
T 1938
T 1097
T 1076
T 1969
T 1622
T 0475
T 1612
T 1976
T 0900
T 1318
T 0788
T 1423
T 0198
T 0199
T 1248
T 0854
T 1706
T 1201
T 1432
T 1442
T 0825
T 1339
T 0687
T 0996
T 0594
T 1974
T 0390
T 0391
T 0807
T 1284
T 1851
T 1302
T 1838
T 1847
T 0422
T 0423
T 1743
T 0693
T 1324
T 1715
T 0404
T 1452
T 0939
T 0680
T 1773
T 1573
T 0174
T 0089
T 0386
T 0387
T 0151
T 1962
T 1667
T 1193
T 1192
T 1214
T 0694
T 1190
T 1189
T 1675
T 1356
T 1216
T 0184
T 1666
T 1191
T 0275
T 0256
T 0255
T 1672
T 0274
T 1350
T 1669
T 1212
T 0192
T 0201
T 1194
T 0257
T 1208
T 1210
T 1207
T 1209
T 1670
T 1671
T 0363
T 0273
T 0839
T 1070
T 1607
T 0955
T 1492
T 0917
T 1574
T 1199
T 0763
T 1136
T 1162
T 1876
T 0730
T 0775
T 1111
T 1122
T 1119
T 1796
T 0990
T 1650
T 2031
T 1305
T 1879
T 0882
T 1311
T 1291
T 1870
T 1750
T 0980
T 0994
T 0933
T 1922
T 0936
T 1917
T 1293
T 1563
T 1502
T 1959
T 1753
T 0091
T 0085
T 0214
T 2027
T 1507
T 1481
T 0554
T 0988
T 1577
T 0974
T 1571
T 0964
T 0723
T 1368
T 0415
T 1168
T 1230
T 0558
T 0940
T 0978

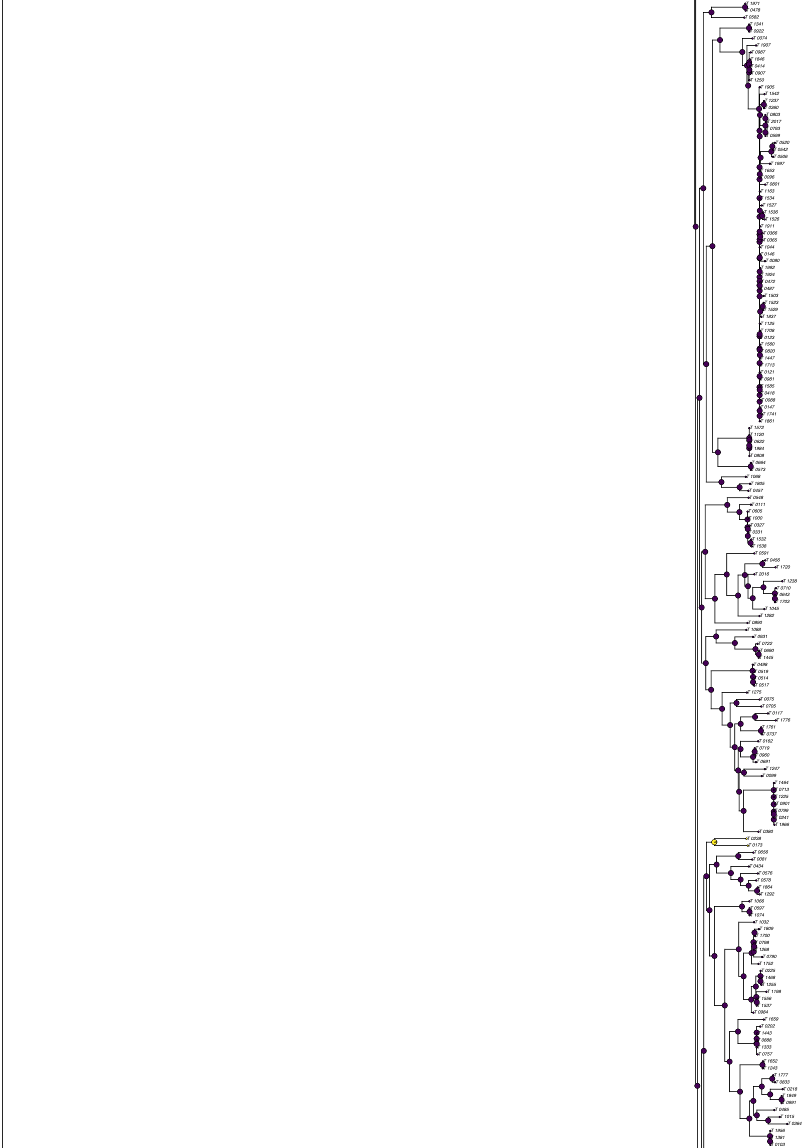
T 1971
T 0478
T 0582
T 1341
T 0922
T 0074
T 1907
T 0987
T 1846
T 0414
T 0907
T 1250
T 1905
T 1542
T 1237
T 0360
T 0803
T 2017
T 0793
T 0599
T 0520
T 0542
T 0506
T 1997
T 1653
T 0096
T 0801
T 1163
T 1534
T 1527
T 1536
T 1526
T 1911
T 0366
T 0365
T 1044
T 0146
T 0080
T 1992
T 1924
T 0472
T 0487
T 1503
T 1523
T 1529
T 1837
T 1125
T 1708
T 0123
T 1560
T 0820
T 1447
T 1713
T 0121
T 0981
T 1585
T 0418
T 0088
T 0147
T 1741
T 1861
T 1572
T 1120
T 0622
T 1984
T 0808
T 0664
T 0573
T 1068
T 1805
T 0457
T 0548
T 0111
T 0605
T 1000
T 0327
T 0331
T 1532
T 1538
T 0591
T 0456
T 1720
T 2016
T 1238
T 0710
T 0643
T 1703
T 1045
T 1282
T 0890
T 1088
T 0931
T 0722
T 0690
T 1445
T 0498
T 0519
T 0514
T 0517
T 1275
T 0075
T 0705
T 0117
T 1776
T 1761
T 0737
T 0162
T 0719
T 0960
T 0691
T 1247
T 0099
T 1464
T 0713
T 1225
T 0901
T 0799
T 0241
T 1966
T 0380
T 0238
T 0173
T 0656
T 0081
T 0434
T 0576
T 0578
T 1864
T 1292
T 1066
T 0597
T 1074
T 1032
T 1809
T 1700
T 0798
T 1268
T 0790
T 1752
T 0225
T 1468
T 1255
T 1198
T 1556
T 1537
T 0984
T 1659
T 0202
T 1443
T 0888
T 1333
T 0757
T 1652
T 1243
T 1777
T 0833
T 0218
T 1849
T 0991
T 0485
T 1015
T 0364
T 1956
T 1381
T 0103

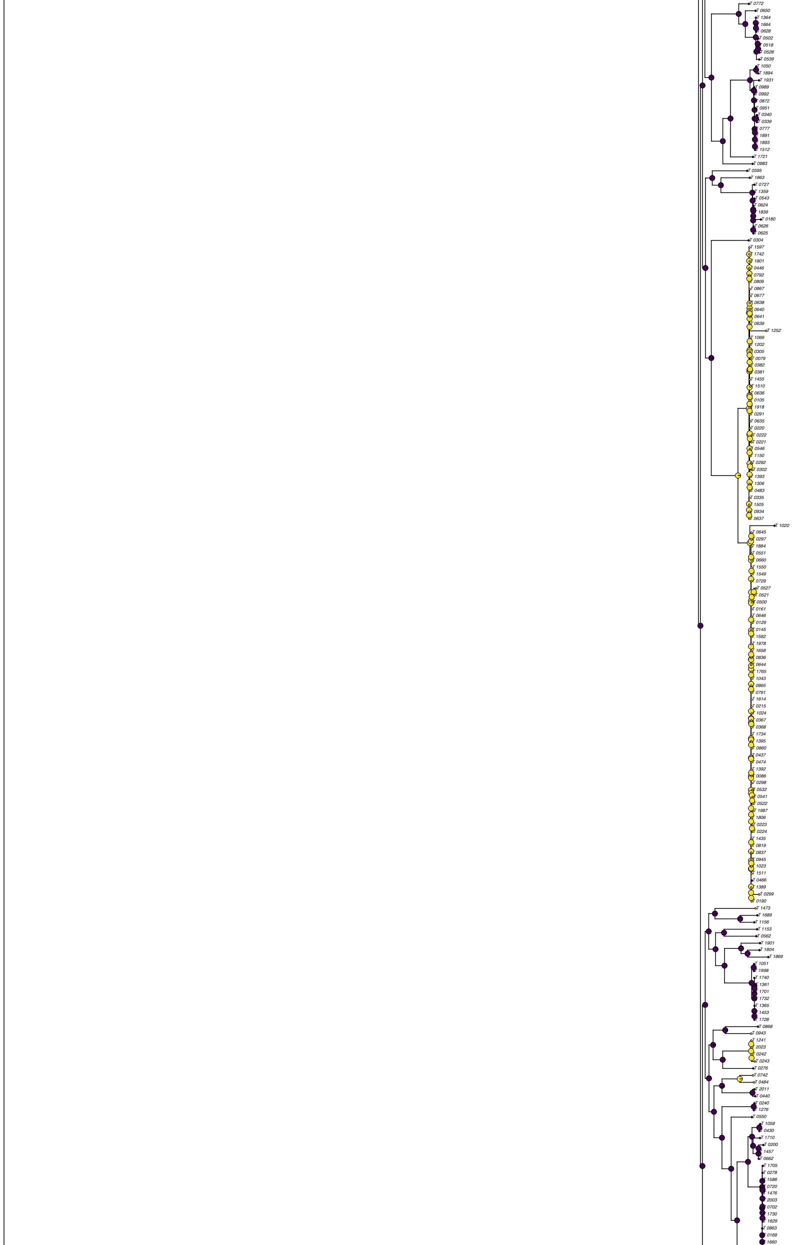
T 0772
T 0650
T 1364
T 1664
T 0628
T 0502
T 0518
T 0528
T 0539
T 1050
T 1894
T 1931
T 0989
T 0992
T 0872
T 0951
T 0340
T 0339
T 0777
T 1891
T 1893
T 1512
T 1721
T 0983
T 0595
T 1863
T 0727
T 1359
T 0543
T 0624
T 1839
T 0180
T 0626
T 0625
T 0304
T 1597
T 1742
T 1801
T 0446
T 0792
T 0806
T 0867
T 0677
T 0638
T 0640
T 0641
T 0639
T 1252
T 1069
T 1202
T 0305
T 0079
T 0382
T 0381
T 1455
T 1510
T 0636
T 0105
T 1918
T 0291
T 0635
T 0220
T 0222
T 0221
T 0546
T 1150
T 0292
T 0302
T 1393
T 1306
T 0483
T 0335
T 1505
T 0934
T 0637
T 1020
T 0645
T 0297
T 1884
T 0551
T 0660
T 1550
T 1549
T 0729
T 0527
T 0521
T 0500
T 0161
T 0646
T 0129
T 0145
T 1592
T 1978
T 1658
T 0836
T 0644
T 1765
T 1043
T 0865
T 0791
T 1614
T 0215
T 1024
T 0367
T 0368
T 1734
T 1395
T 0860
T 0437
T 0474
T 1392
T 0086
T 0298
T 0532
T 0541
T 0522
T 1987
T 1806
T 0223
T 0224
T 1435
T 0819
T 0837
T 0945
T 1023
T 1511
T 0466
T 1389
T 0299
T 0190
T 1473
T 1689
T 1156
T 1153
T 0562
T 1901
T 1804
T 1869
T 1051
T 1998
T 1740
T 1361
T 1701
T 1732
T 1365
T 1453
T 1728
T 0868
T 0943
T 1241
T 2023
T 0242
T 0243
T 0276
T 0742
T 0484
T 2011
T 0440
T 0240
T 1276
T 0550
T 1059
T 0430
T 1710
T 0200
T 1457
T 0662
T 1705
T 0278
T 1586
T 0720
T 1476
T 2003
T 0702
T 1730
T 1629
T 0863
T 0169
T 1660

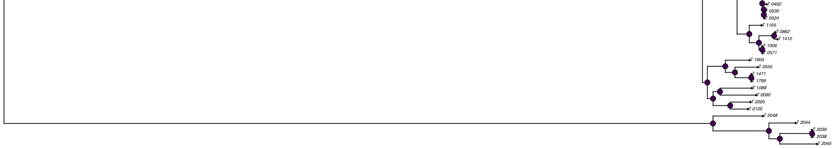
T 0492
T 0530
T 0524
T 1165
T 0862
T 1412
T 1006
T 0571
T 1904
T 0555
T 1471
T 1799
T 1089
T 0090
T 2005
T 0120
T 2048
T 2044
T 2039
T 2038
T 2045

Effector gene: PA0256

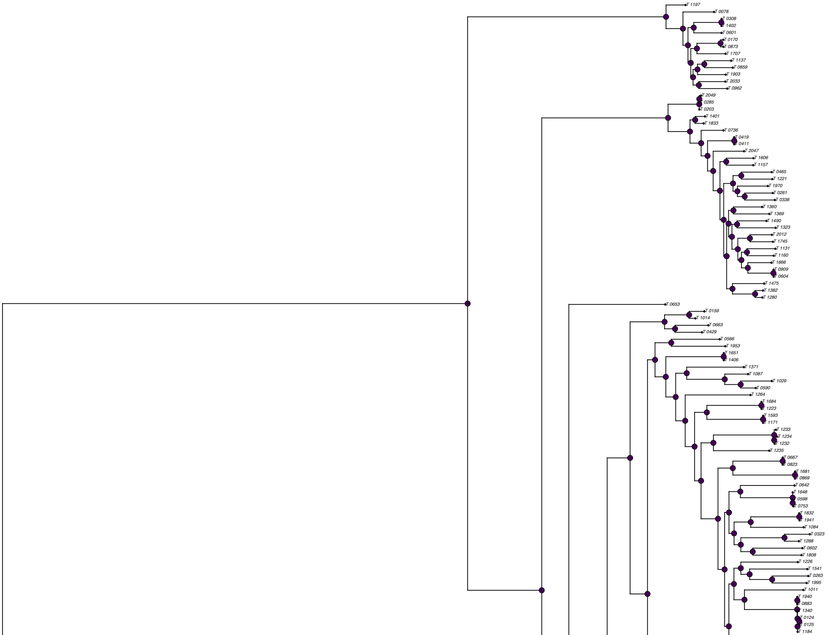

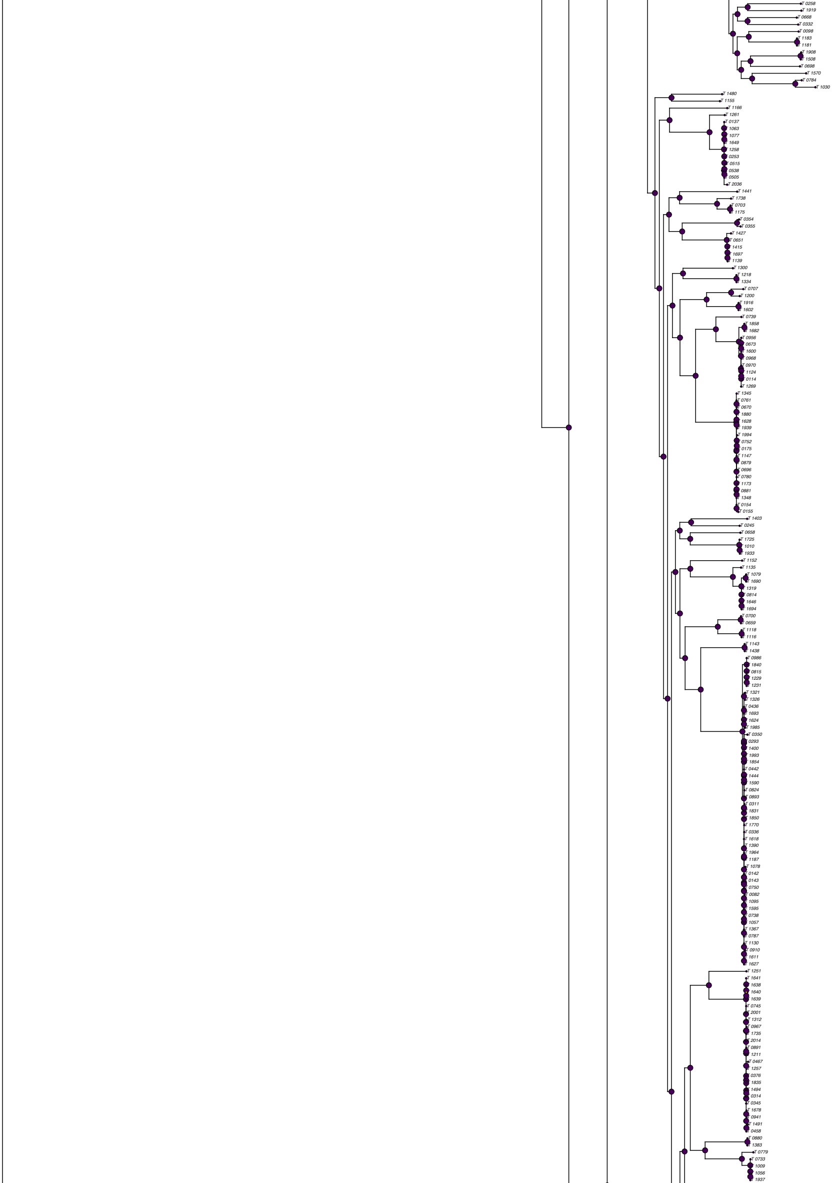

T 0258
T 1919
T 0668
T 0332
T 0098
T 1183
T 1181
T 1908
T 1508
T 0698
T 1570
T 0784
T 1030
T 1480
T 1155
T 1166
T 1261
T 0137
T 1063
T 1077
T 1649
T 1258
T 0253
T 0515
T 0538
T 0505
T 2036
T 1441
T 1738
T 0703
T 1175
T 0354
T 0355
T 1427
T 0651
T 1415
T 1697
T 1139
T 1300
T 1218
T 1334
T 0707
T 1200
T 1916
T 1602
T 0739
T 1858
T 1682
T 0956
T 0673
T 1600
T 0968
T 0970
T 1124
T 0114
T 1269
T 1345
T 0761
T 0670
T 1880
T 1628
T 1939
T 1994
T 0752
T 0175
T 1147
T 0879
T 0696
T 0780
T 1173
T 0881
T 1348
T 0154
T 0155
T 1403
T 0245
T 0658
T 1725
T 1010
T 1933
T 1152
T 1135
T 1079
T 1690
T 1319
T 0814
T 1646
T 1694
T 0700
T 0659
T 1118
T 1116
T 1143
T 1438
T 0986
T 1840
T 0815
T 1229
T 1231
T 1321
T 1326
T 0436
T 1693
T 1624
T 1985
T 0350
T 0293
T 1400
T 1993
T 1854
T 0442
T 1444
T 1590
T 0824
T 0893
T 0311
T 1831
T 1850
T 1770
T 0336
T 1618
T 1390
T 1964
T 1187
T 1078
T 0142
T 0143
T 0750
T 0082
T 1095
T 1595
T 0738
T 1057
T 1367
T 0787
T 1130
T 0910
T 1611
T 1627
T 1251
T 1641
T 1638
T 1640
T 1639
T 0745
T 2001
T 1312
T 0967
T 1735
T 2014
T 0891
T 1211
T 0467
T 1257
T 0376
T 1835
T 1494
T 0314
T 0345
T 1678
T 0941
T 1491
T 0458
T 0880
T 1383
T 0779
T 0733
T 1009
T 1056
T 1937

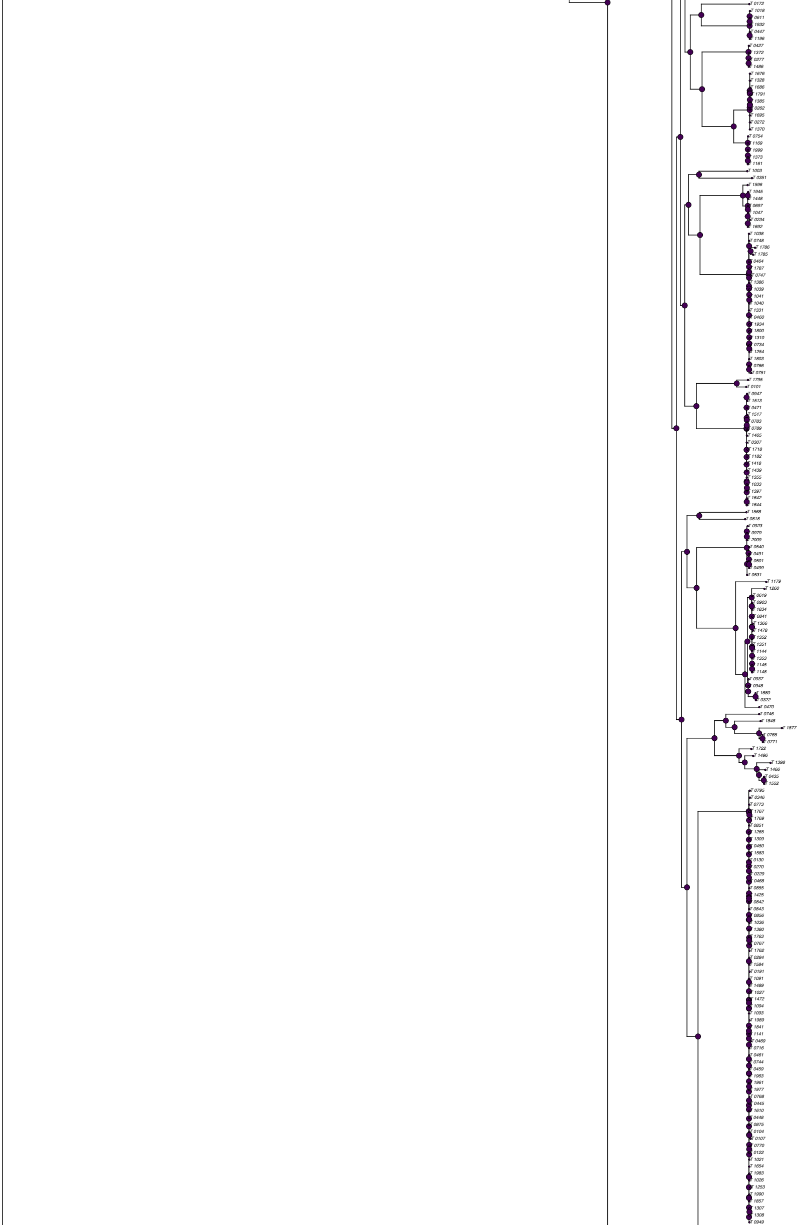
T 0172
T 1018
0611
1932
T 0447
T 1196
T 0427
1372
T 0277
T 1486
T 1676
T 1328
T 1686
1791
1385
T 0262
T 1695
T 0272
T 1370
T 0754
1169
1999
1373
T 1161
T 1003
T 0351
T 1596
T 1945
T 1448
0697
T 1047
T 0234
T 1692
T 1038
T 0748
T 1786
T 1785
0464
1787
T 0747
T 1386
1039
1041
1040
T 1331
0460
1934
1800
1310
0734
T 1254
T 1803
0766
T 0751
T 1795
T 0101
T 0947
T 1513
0471
T 1517
0783
0789
T 1465
T 0307
T 1718
T 1182
1418
T 1439
T 1355
1033
1397
T 1642
T 1644
T 1568
T 0818
T 0923
0979
T 2009
T 0540
0491
0501
T 0499
T 0531
T 1179
T 1260
0619
T 0903
T 1834
0841
T 1366
T 1478
1352
1351
1144
1353
1145
T 1148
T 0937
0948
T 1680
T 0322
T 0470
T 0746
T 1848
T 1877
T 0765
T 0771
T 1722
T 1496
T 1398
T 1466
T 0435
T 1552
T 0795
T 0346
T 0773
1767
1769
T 0851
1265
1309
0450
T 1583
T 0130
0270
T 0229
T 0468
T 0855
1425
0842
T 0843
0856
T 1036
1380
T 1763
0767
T 1762
T 0284
1584
T 0191
T 1091
1489
1027
T 1472
T 1094
T 1093
T 1989
1841
1141
T 0469
T 0716
T 0461
0744
T 0459
1963
1961
1977
T 0768
0445
1610
0448
T 0875
T 0104
T 0107
0770
0122
T 1021
T 1654
T 1983
1026
T 1253
T 1990
T 1857
T 1307
1308
T 0949

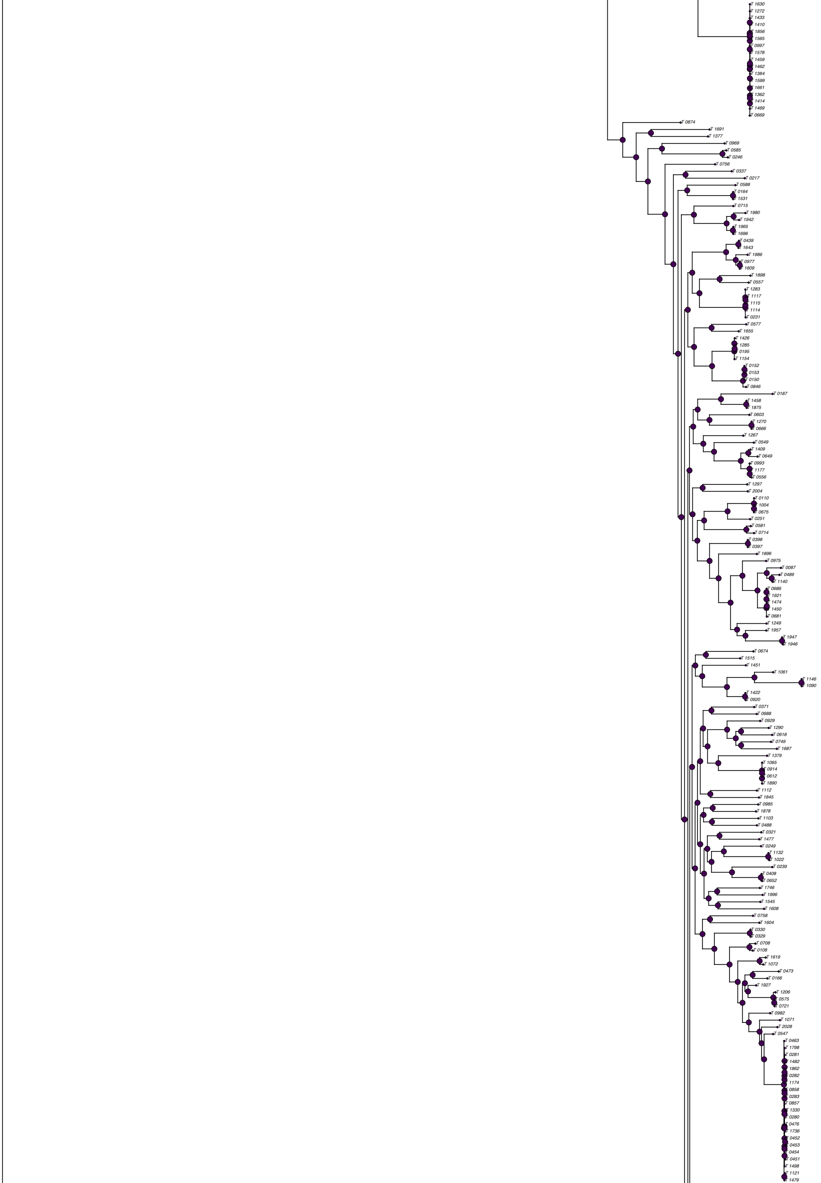
T 1630
T 1272
T 1433
T 1410
T 1856
T 1565
T 0997
T 1578
T 1459
T 1462
T 1384
T 1599
T 1661
T 1362
T 1414
T 1469
T 0669
T 0874
T 1691
T 1377
T 0969
T 0585
T 0246
T 0756
T 0337
T 0217
T 0588
T 0164
T 1531
T 0715
T 1980
T 1942
T 1965
T 1696
T 0439
T 1643
T 1986
T 0977
T 1609
T 1898
T 0557
T 1283
T 1117
T 1115
T 1114
T 0231
T 0577
T 1655
T 1426
T 1285
T 0195
T 1154
T 0152
T 0153
T 0150
T 0846
T 0187
T 1458
T 1875
T 0603
T 1270
T 0666
T 1267
T 0549
T 1409
T 0649
T 0993
T 1177
T 0556
T 1297
T 2004
T 0110
T 1004
T 0675
T 0251
T 0581
T 0714
T 0398
T 0397
T 1896
T 0975
T 0087
T 0489
T 1140
T 0686
T 1921
T 1474
T 1450
T 0681
T 1249
T 1957
T 1947
T 1946
T 0674
T 1515
T 1451
T 1061
T 1146
T 1090
T 1422
T 0920
T 0371
T 0688
T 0929
T 1290
T 0618
T 0749
T 1687
T 1379
T 1065
T 0914
T 0612
T 1890
T 1112
T 1845
T 0985
T 1878
T 1103
T 0488
T 0321
T 1477
T 0249
T 1132
T 1022
T 0239
T 0408
T 0652
T 1746
T 1996
T 1545
T 1608
T 0758
T 1604
T 0330
T 0329
T 0708
T 0108
T 1619
T 1072
T 0473
T 0166
T 1927
T 1206
T 0575
T 0721
T 0982
T 1071
T 2028
T 0547
T 0463
T 1798
T 0281
T 1482
T 1862
T 0282
T 1174
T 0858
T 0283
T 0857
T 1330
T 0280
T 0476
T 1736
T 0452
T 0453
T 0454
T 0451
T 1498
T 1121
T 1479

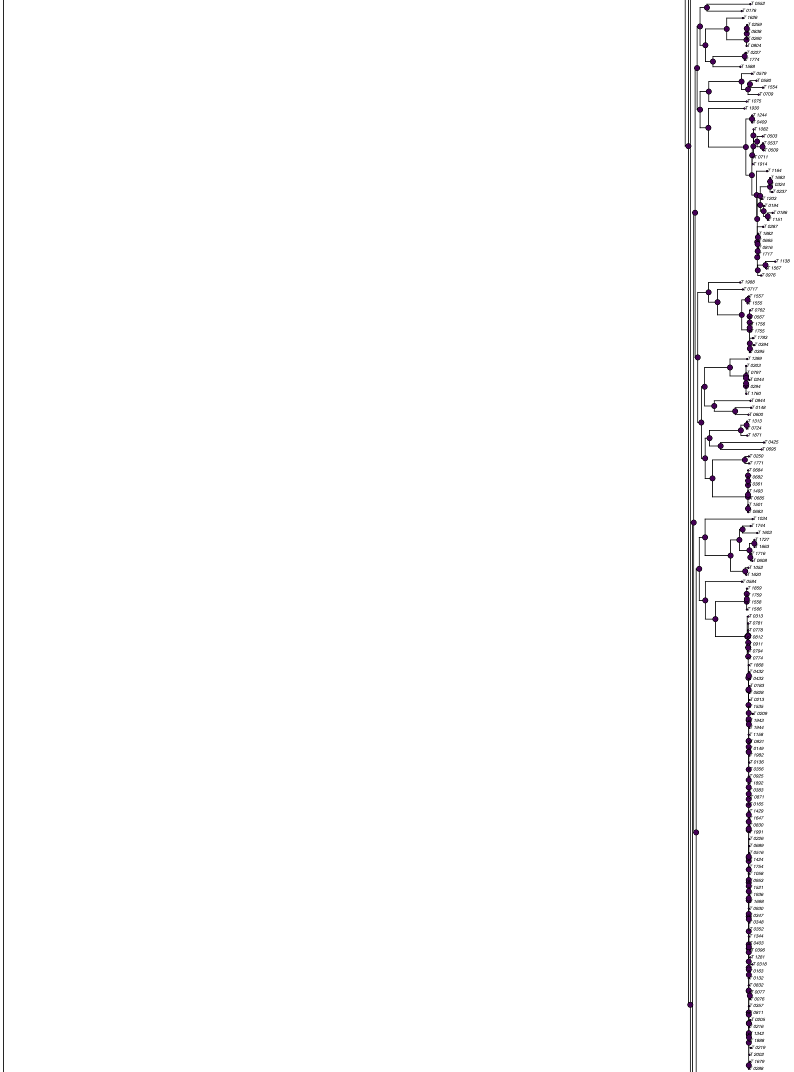
T 0552
T 0176
T 1626
T 0259
0838
0260
T 0804
T 0227
T 1774
T 1588
T 0579
T 0580
T 1554
T 0709
T 1075
T 1930
T 1244
T 0409
T 1082
T 0503
T 0537
T 0509
T 0711
T 1914
T 1164
T 1683
0324
T 0237
T 1203
T 0194
T 0186
T 1151
T 0287
T 1882
0665
T 0816
1717
T 1138
T 1567
T 0976
T 1988
T 0717
T 1557
T 1555
T 0762
0567
T 1756
T 1755
T 1783
T 0394
T 0395
T 1399
T 0303
T 0797
T 0244
0294
T 1760
T 0844
T 0148
T 0600
T 1313
T 0724
T 1871
T 0425
T 0695
T 0250
T 1771
T 0684
0682
0361
T 1493
T 0685
T 1501
T 0683
T 1034
T 1744
T 1603
T 1727
T 1663
T 1716
T 0608
T 1052
T 1620
T 0584
T 1859
1759
1558
T 1566
T 0313
T 0781
T 0778
0812
0911
T 0794
0774
T 1868
T 0432
0433
T 0183
T 0828
T 0213
1535
T 0209
1943
T 1944
T 1158
0831
0149
T 1982
T 0136
0356
T 0925
T 1892
0383
T 0871
0165
T 1429
T 1647
T 0830
1991
T 0226
T 0689
T 0516
1424
T 1754
T 1058
0953
1521
T 1936
1698
T 0930
0347
T 0348
T 0352
T 1344
0403
T 0396
T 1281
T 0318
0163
T 0132
T 0832
T 0077
T 0076
T 0357
0811
T 0205
T 0216
1342
T 1888
T 0219
T 2002
T 1679
T 0288

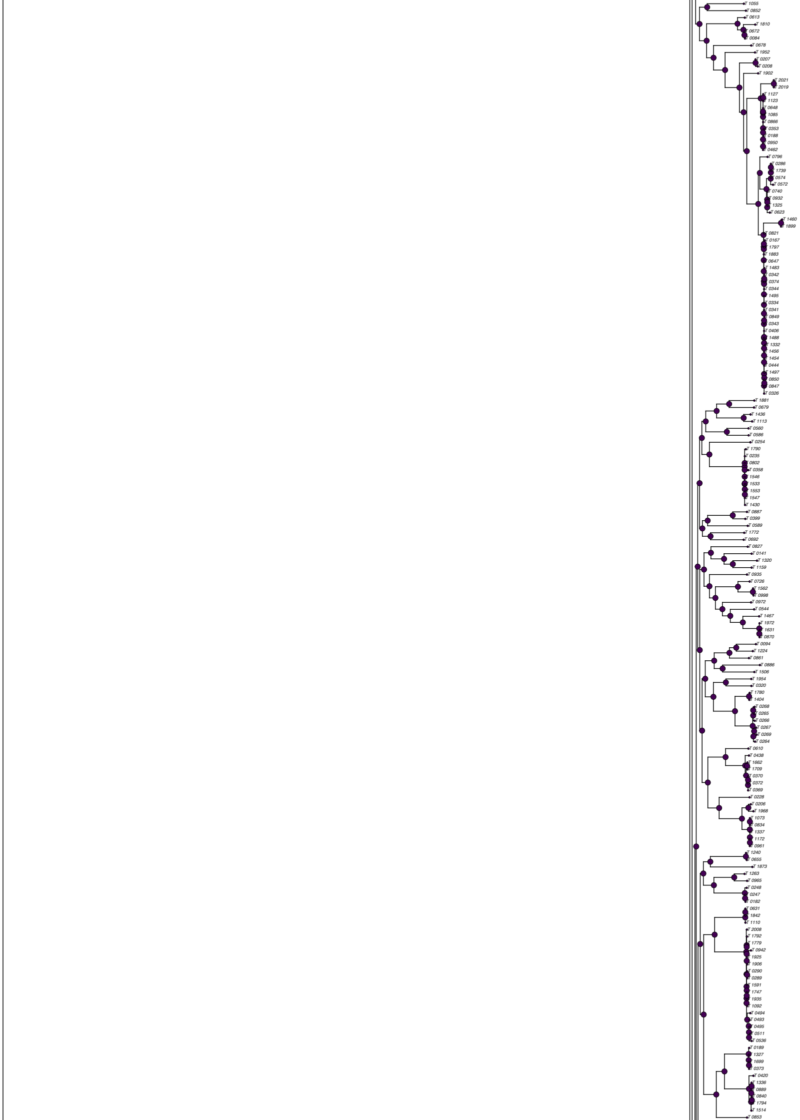
T 1055
T 0852
T 0613
T 1810
0672
T 0084
T 0678
T 1952
T 0207
T 0208
T 1902
T 2021
T 2019
T 1127
T 1123
T 0648
T 1085
T 0866
T 0353
T 0188
T 0950
T 0462
T 0796
T 0286
T 1739
T 0574
T 0572
T 0740
T 0932
T 1325
T 0623
T 1460
T 1899
T 0821
T 0167
T 1797
T 1883
0647
T 1483
T 0342
T 0374
T 0344
1495
T 0334
T 0341
T 0849
T 0343
T 0406
1488
T 1332
T 1456
T 1454
T 0444
T 1497
T 0850
T 0847
T 0326
T 1881
T 0679
T 1436
T 1113
T 0560
T 0586
T 0254
T 1790
T 0235
0802
T 0358
1546
1533
1553
T 1547
T 1430
T 0887
T 0399
T 0589
T 1772
T 0692
T 0827
T 0141
T 1320
T 1159
T 0935
T 0726
T 1562
T 0998
T 0972
T 0544
T 1467
T 1972
1631
T 0870
T 0094
T 1224
T 0861
T 0886
T 1506
T 1954
T 0320
T 1780
T 1404
T 0268
T 0265
T 0266
T 0267
T 0269
T 0264
T 0610
T 0438
T 1662
T 1709
T 0370
T 0372
T 0369
T 0228
T 0206
T 1968
T 1073
T 0834
T 1337
T 1172
T 0961
T 1240
T 0655
T 1873
T 1263
T 0965
T 0248
T 0247
T 0182
T 0631
T 1842
T 1110
T 2008
T 1792
T 1779
T 0942
T 1925
T 1906
T 0290
T 0289
T 1591
T 1747
T 1935
T 1092
T 0494
T 0493
T 0495
T 0511
T 0536
T 0189
T 1327
T 1699
T 0373
T 0420
T 1336
T 0889
T 0840
T 1794
T 1514
T 0853

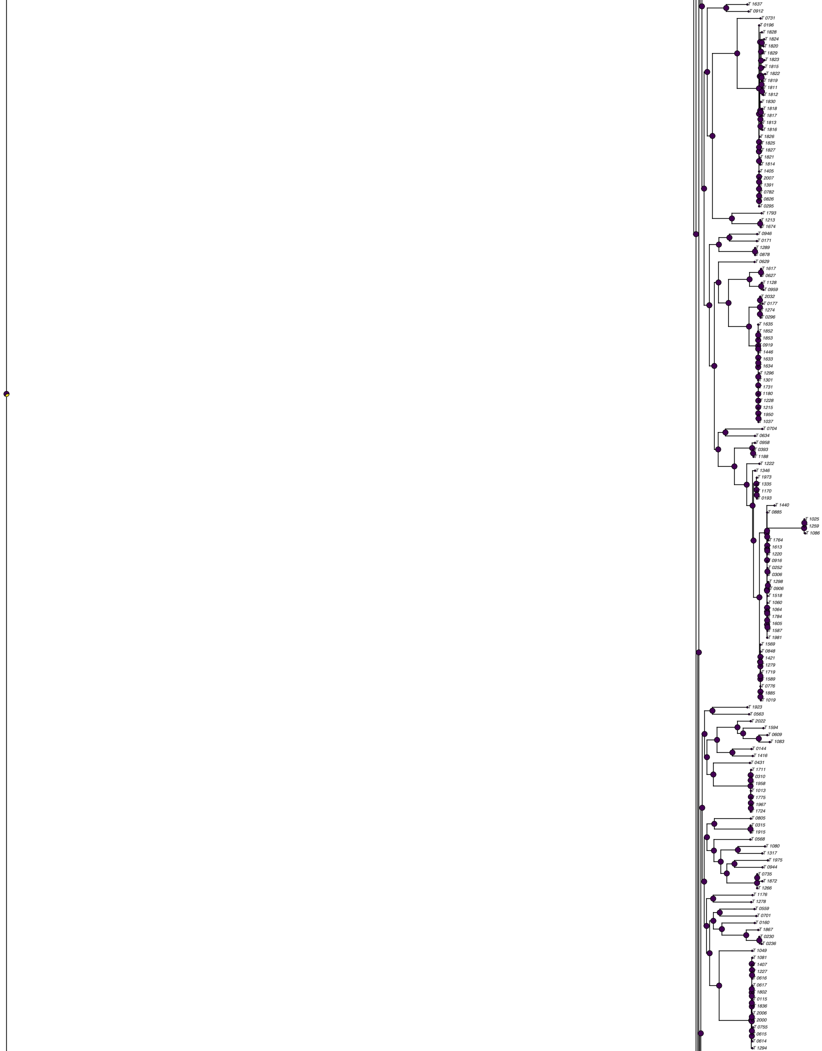
T 1637
T 0912
T 0731
T 0196
T 1828
T 1824
T 1820
T 1829
T 1823
T 1815
T 1822
T 1819
T 1811
T 1812
T 1830
T 1818
T 1817
T 1813
T 1816
T 1826
T 1825
T 1827
T 1821
T 1814
T 1405
2007
1391
0782
0826
T 0295
T 1793
T 1213
T 1674
T 0946
T 0171
T 1289
T 0878
T 0629
T 1617
T 0627
T 1128
T 0959
T 2032
T 0177
T 1274
T 0296
T 1635
T 1852
1853
0919
T 1446
1633
1634
T 1296
T 1301
1731
1180
T 1228
1215
1950
T 1037
T 0704
T 0634
T 0958
T 0393
T 1188
T 1222
T 1346
T 1973
1335
1170
T 0193
T 1440
T 0885
T 1025
1259
T 1086
T 1764
1613
T 1220
0916
T 0252
T 0306
T 1298
T 0906
T 1518
T 1060
1064
T 1784
1605
T 1587
T 1981
T 1569
T 0848
1421
1279
T 1719
1589
T 0776
1885
T 1019
T 1923
T 0563
T 2022
T 1594
T 0609
T 1083
T 0144
T 1416
T 0431
T 1711
0310
1958
T 1013
1775
1967
1724
T 0805
T 0315
T 1915
T 0568
T 1080
T 1317
T 1975
T 0944
T 0735
T 1872
T 1266
T 1176
T 1278
T 0559
T 0701
T 0160
T 1867
T 0230
T 0236
T 1049
T 1081
1407
1227
0616
T 0617
1802
T 0115
1836
T 2006
2000
T 0755
0615
T 0614
T 1294

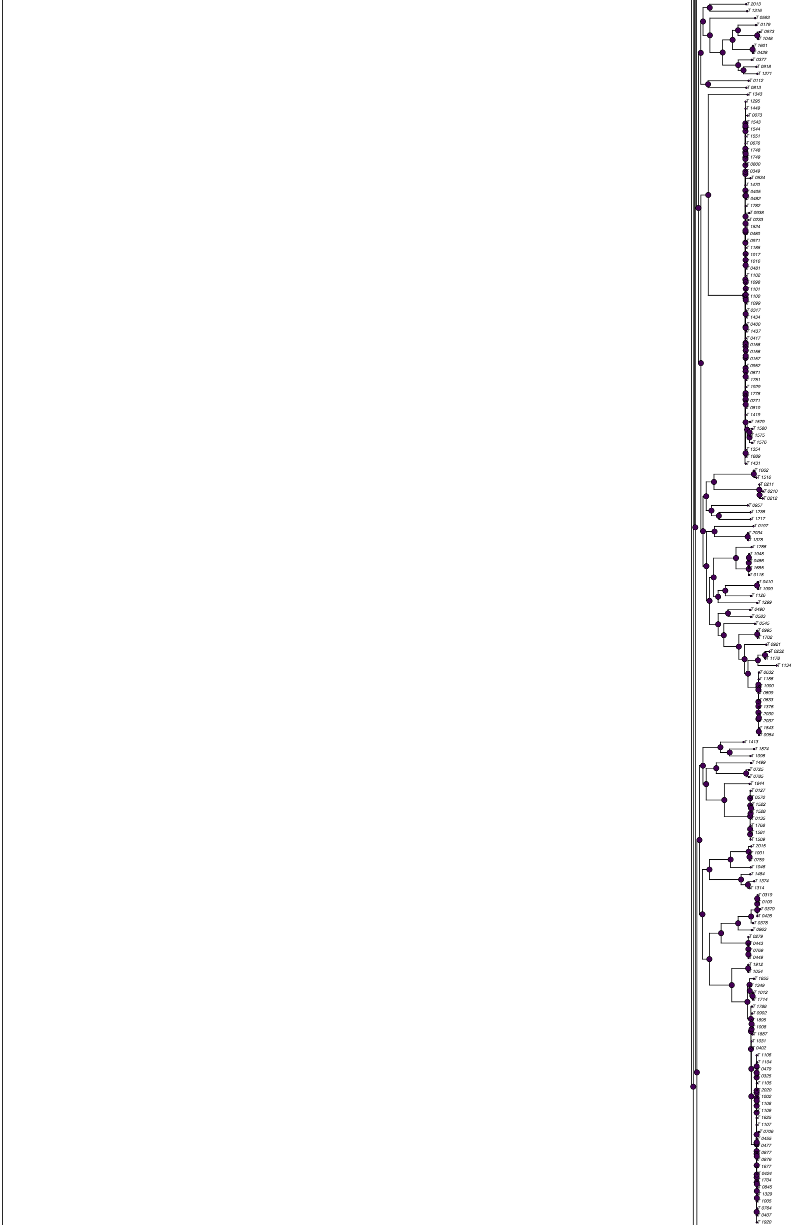
T 2013
T 1316
T 0593
T 0179
T 0973
T 1048
T 1601
T 0428
T 0377
T 0918
T 1271
T 0112
T 0813
T 1343
T 1295
T 1449
T 0073
T 1543
1544
T 1551
T 0676
1748
1749
0800
0349
T 0534
T 1470
0405
0482
T 1782
T 0938
T 0233
1524
0480
T 0971
T 1185
1017
1016
0481
T 1102
1098
1101
1100
T 1099
T 0317
1434
T 0400
1437
T 0417
0158
0156
0157
T 0952
0671
T 1751
T 1929
1778
0271
T 0810
T 1419
T 1579
T 1580
1575
T 1576
T 1354
T 1889
T 1431
T 1062
T 1516
T 0211
T 0210
T 0212
T 0957
T 1236
T 1217
T 0197
T 2034
T 1378
T 1286
T 1948
0486
T 1685
T 0118
T 0410
T 1909
T 1126
T 1299
T 0490
T 0583
T 0545
T 0995
T 1702
T 0921
T 0232
T 1178
T 1134
T 0632
T 1186
T 1900
T 0699
T 0633
T 1376
T 2030
T 2037
T 1843
T 0954
T 1413
T 1874
T 1096
T 1499
T 0725
T 0785
T 1844
T 0127
T 0570
T 1522
T 1528
T 0135
T 1768
T 1581
T 1509
T 2015
T 1001
T 0759
T 1046
T 1484
T 1374
T 1314
T 0319
T 0100
T 0379
T 0426
T 0378
T 0963
T 0279
T 0443
T 0769
T 0449
T 1912
T 1054
T 1855
T 1349
T 1012
T 1714
T 1788
T 0902
T 1895
T 1008
T 1887
T 1031
T 0402
T 1106
T 1104
T 0479
T 0325
T 1105
T 2020
T 1002
T 1108
T 1109
T 1625
T 1107
T 0706
T 0455
T 0477
T 0877
T 0876
T 1677
T 0424
T 1704
T 0845
T 1329
T 1005
T 0764
T 0407
T 1920

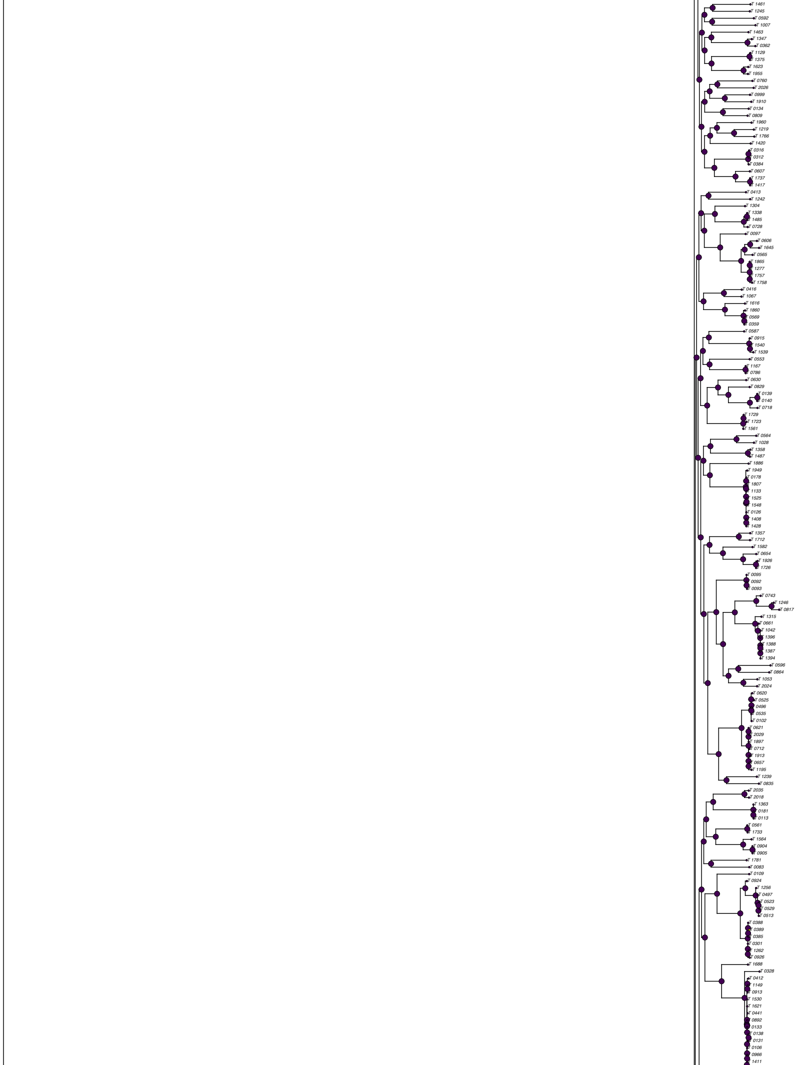

T 1461
T 1245
T 0592
T 1007
T 1463
T 1347
T 0362
T 1129
T 1375
T 1623
T 1955
T 0760
T 2026
T 0999
T 1910
T 0134
T 0809
T 1960
T 1219
T 1766
T 1420
T 0316
T 0312
T 0384
T 0607
T 1737
T 1417
T 0413
T 1242
T 1304
T 1338
T 1485
T 0728
T 0097
T 0606
T 1645
T 0565
T 1865
1277
1757
T 1758
T 0416
T 1067
T 1616
T 1860
0569
0359
T 0587
T 0915
1540
T 1539
T 0553
T 1167
0786
T 0630
T 0829
T 0139
0140
T 0718
T 1729
T 1723
T 1561
T 0564
T 1028
T 1358
T 1487
T 1886
T 1949
T 0178
1807
1133
1525
1548
T 0126
1408
1428
T 1357
T 1712
T 1582
T 0654
T 1926
T 1726
T 0095
0092
T 0093
T 0743
T 1246
T 0817
T 1315
T 0661
T 1042
1396
T 1388
1387
T 1394
T 0596
T 0864
T 1053
T 2024
T 0620
T 0525
0496
T 0535
T 0102
T 0621
2029
T 1897
T 0712
T 1913
0657
T 1195
T 1239
T 0835
T 2035
T 2018
T 1363
0181
T 0113
T 0561
T 1733
T 1564
T 0904
T 0905
T 1781
T 0083
T 0109
T 0924
T 1256
T 0497
T 0523
T 0529
T 0513
T 0388
T 0389
T 0385
T 0301
T 1262
T 0926
T 1688
T 0328
T 0412
1149
T 0913
T 1530
T 1621
T 0441
0892
0133
T 0138
0131
0106
0966
1411

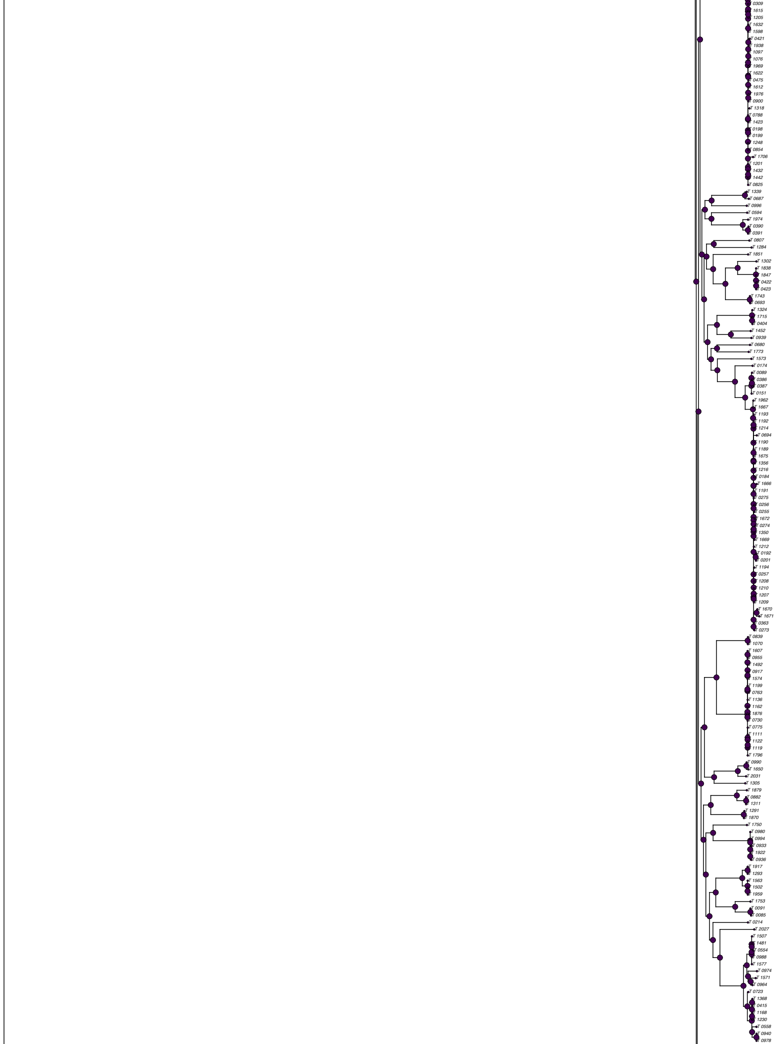
T 0309
T 1615
T 1205
T 1632
T 1598
T 0421
T 1938
T 1097
T 1076
T 1969
T 1622
T 0475
T 1612
T 1976
T 0900
T 1318
T 0788
T 1423
T 0198
T 0199
T 1248
T 0854
T 1706
T 1201
T 1432
T 1442
T 0825
T 1339
T 0687
T 0996
T 0594
T 1974
T 0390
T 0391
T 0807
T 1284
T 1851
T 1302
T 1838
T 1847
T 0422
T 0423
T 1743
T 0693
T 1324
T 1715
T 0404
T 1452
T 0939
T 0680
T 1773
T 1573
T 0174
T 0089
T 0386
T 0387
T 0151
T 1962
T 1667
T 1193
T 1192
T 1214
T 0694
T 1190
T 1189
T 1675
T 1356
T 1216
T 0184
T 1666
T 1191
T 0275
T 0256
T 0255
T 1672
T 0274
T 1350
T 1669
T 1212
T 0192
T 0201
T 1194
T 0257
T 1208
T 1210
T 1207
T 1209
T 1670
T 1671
T 0363
T 0273
T 0839
T 1070
T 1607
T 0955
T 1492
T 0917
T 1574
T 1199
T 0763
T 1136
T 1162
T 1876
T 0730
T 0775
T 1111
T 1122
T 1119
T 1796
T 0990
T 1650
T 2031
T 1305
T 1879
T 0882
T 1311
T 1291
T 1870
T 1750
T 0980
T 0994
T 0933
T 1922
T 0936
T 1917
T 1293
T 1563
T 1502
T 1959
T 1753
T 0091
T 0085
T 0214
T 2027
T 1507
T 1481
T 0554
T 0988
T 1577
T 0974
T 1571
T 0964
T 0723
T 1368
T 0415
T 1168
T 1230
T 0558
T 0940
T 0978

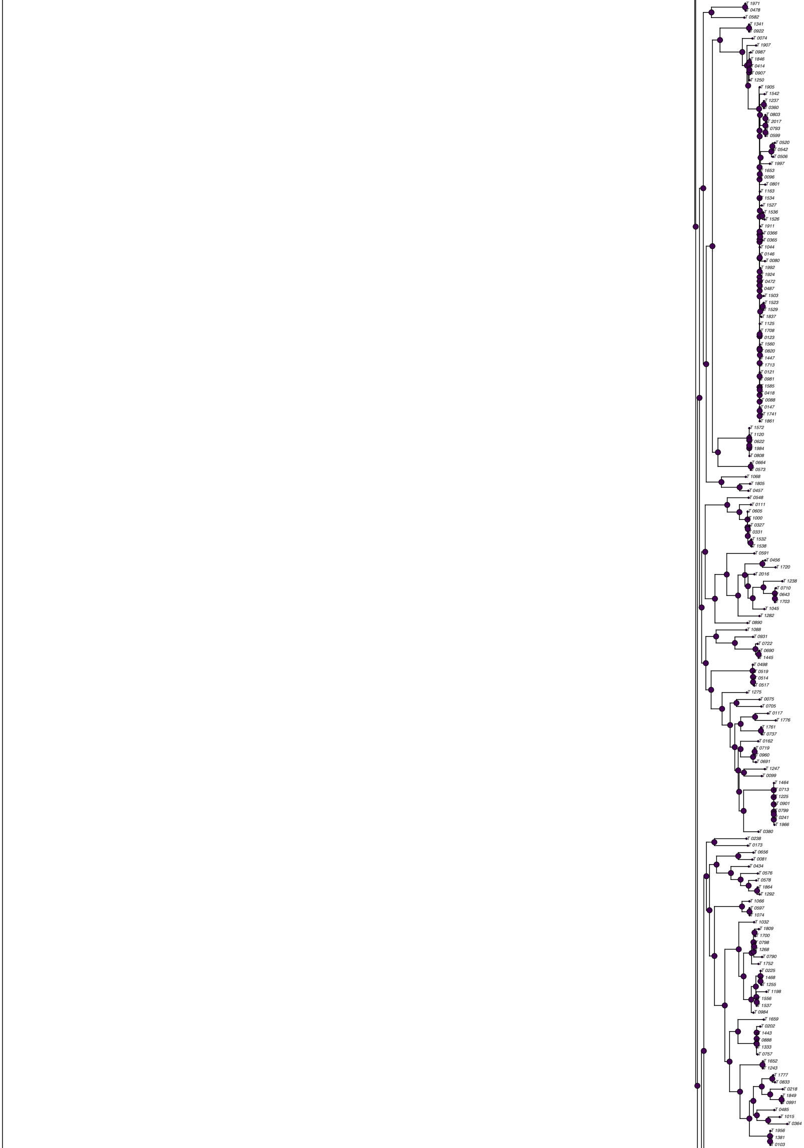
T 1971
T 0478
T 0582
T 1341
T 0922
T 0074
T 1907
T 0987
T 1846
T 0414
T 0907
T 1250
T 1905
T 1542
T 1237
T 0360
T 0803
T 2017
T 0793
T 0599
T 0520
T 0542
T 0506
T 1997
T 1653
T 0096
T 0801
T 1163
T 1534
T 1527
T 1536
T 1526
T 1911
T 0366
T 0365
T 1044
T 0146
T 0080
T 1992
T 1924
T 0472
T 0487
T 1503
T 1523
T 1529
T 1837
T 1125
T 1708
T 0123
T 1560
T 0820
T 1447
T 1713
T 0121
T 0981
T 1585
T 0418
T 0088
T 0147
T 1741
T 1861
T 1572
T 1120
T 0622
T 1984
T 0808
T 0664
T 0573
T 1068
T 1805
T 0457
T 0548
T 0111
T 0605
T 1000
T 0327
T 0331
T 1532
T 1538
T 0591
T 0456
T 1720
T 2016
T 1238
T 0710
T 0643
T 1703
T 1045
T 1282
T 0890
T 1088
T 0931
T 0722
T 0690
T 1445
T 0498
T 0519
T 0514
T 0517
T 1275
T 0075
T 0705
T 0117
T 1776
T 1761
T 0737
T 0162
T 0719
T 0960
T 0691
T 1247
T 0099
T 1464
T 0713
T 1225
T 0901
T 0799
T 0241
T 1966
T 0380
T 0238
T 0173
T 0656
T 0081
T 0434
T 0576
T 0578
T 1864
T 1292
T 1066
T 0597
T 1074
T 1032
T 1809
T 1700
T 0798
T 1268
T 0790
T 1752
T 0225
T 1468
T 1255
T 1198
T 1556
T 1537
T 0984
T 1659
T 0202
T 1443
T 0888
T 1333
T 0757
T 1652
T 1243
T 1777
T 0833
T 0218
T 1849
T 0991
T 0485
T 1015
T 0364
T 1956
T 1381
T 0103

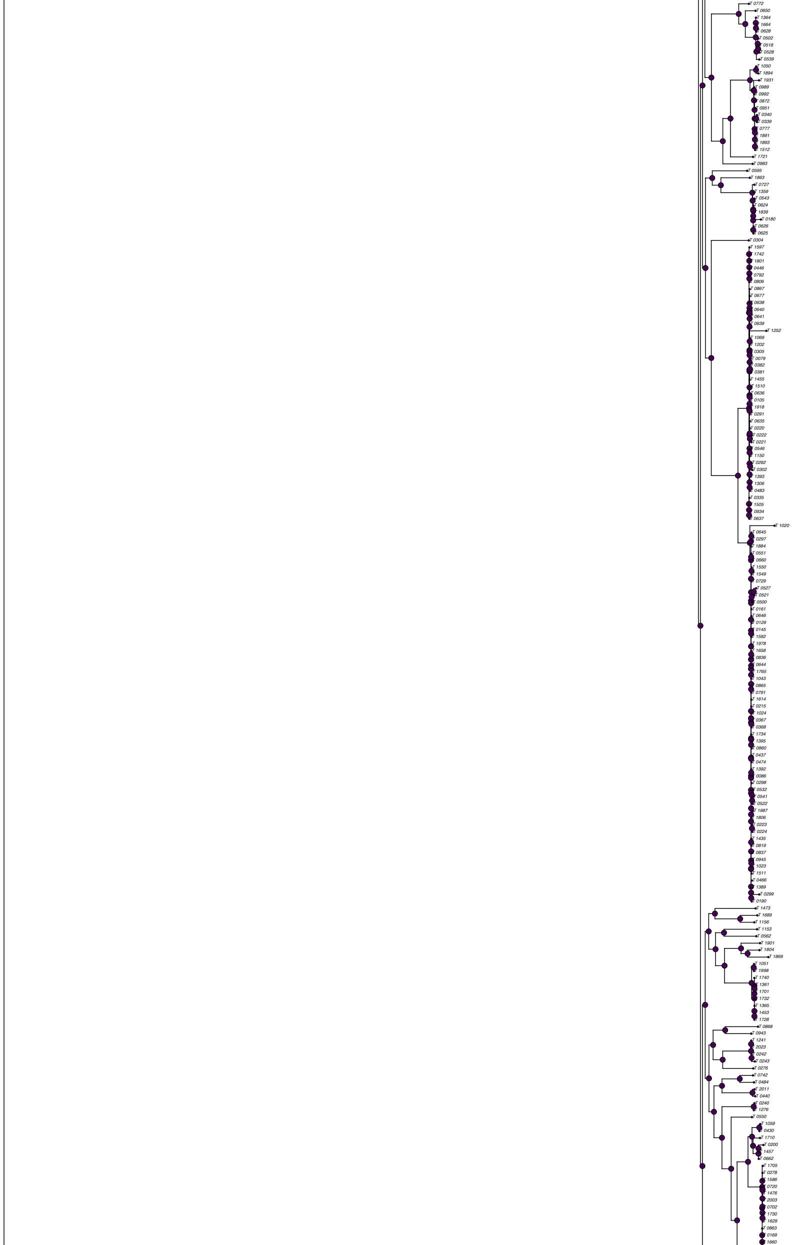
T 0772
T 0650
T 1364
1664
0628
T 0502
0518
T 0528
T 0539
T 1050
T 1894
T 1931
T 0989
0992
T 0872
T 0951
T 0340
T 0339
T 0777
1891
1893
T 1512
T 1721
T 0983
T 0595
T 1863
T 0727
T 1359
T 0543
T 0624
1839
T 0180
T 0626
T 0625
T 0304
T 1597
1742
1801
0446
0792
T 0806
T 0867
T 0677
0638
0640
0641
0639
T 1252
T 1069
T 1202
0305
T 0079
0382
0381
T 1455
1510
T 0636
0105
1918
T 0291
T 0635
T 0220
T 0222
T 0221
0546
T 1150
T 0292
T 0302
1393
1306
T 0483
T 0335
1505
0934
T 0637
T 1020
T 0645
0297
T 1884
T 0551
0660
T 1550
1549
0729
T 0527
T 0521
0500
T 0161
0646
T 0129
T 0145
T 1592
T 1978
T 1658
0836
0644
T 1765
T 1043
0865
T 0791
T 1614
T 0215
1024
0367
T 0368
T 1734
1395
T 0860
0437
T 0474
T 1392
0086
T 0298
T 0532
T 0541
T 0522
T 1987
1806
T 0223
T 0224
T 1435
T 0819
T 0837
0945
T 1023
T 1511
T 0466
1389
T 0299
T 0190
T 1473
T 1689
T 1156
T 1153
T 0562
T 1901
T 1804
T 1869
T 1051
T 1998
T 1740
1361
1701
1732
T 1365
1453
T 1728
T 0868
T 0943
T 1241
2023
0242
T 0243
T 0276
T 0742
T 0484
T 2011
T 0440
T 0240
T 1276
T 0550
T 1059
0430
T 1710
T 0200
1457
T 0662
T 1705
T 0278
1586
T 0720
T 1476
2003
0702
T 1730
T 1629
T 0863
T 0169
1660

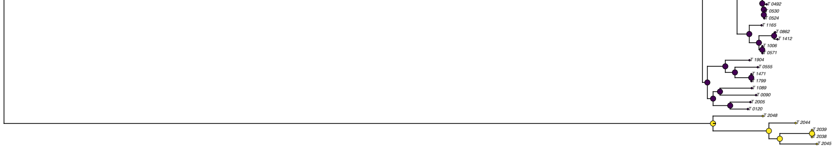
T 0492
T 0530
T 0524
T 1165
T 0862
T 1412
T 1006
T 0571
T 1904
T 0555
T 1471
T 1799
T 1089
T 0090
T 2005
T 0120
T 2048
T 2044
T 2039
T 2038
T 2045

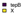

Effector gene: tepB

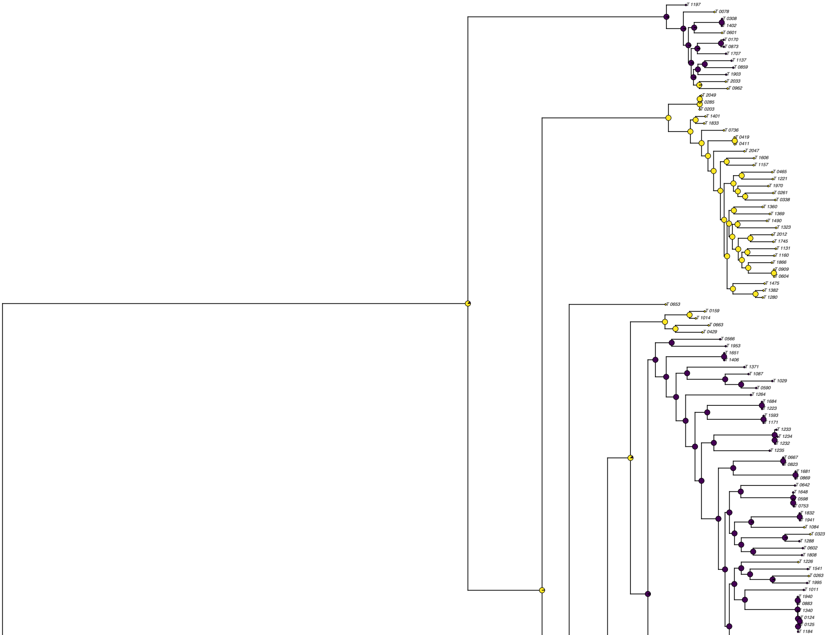

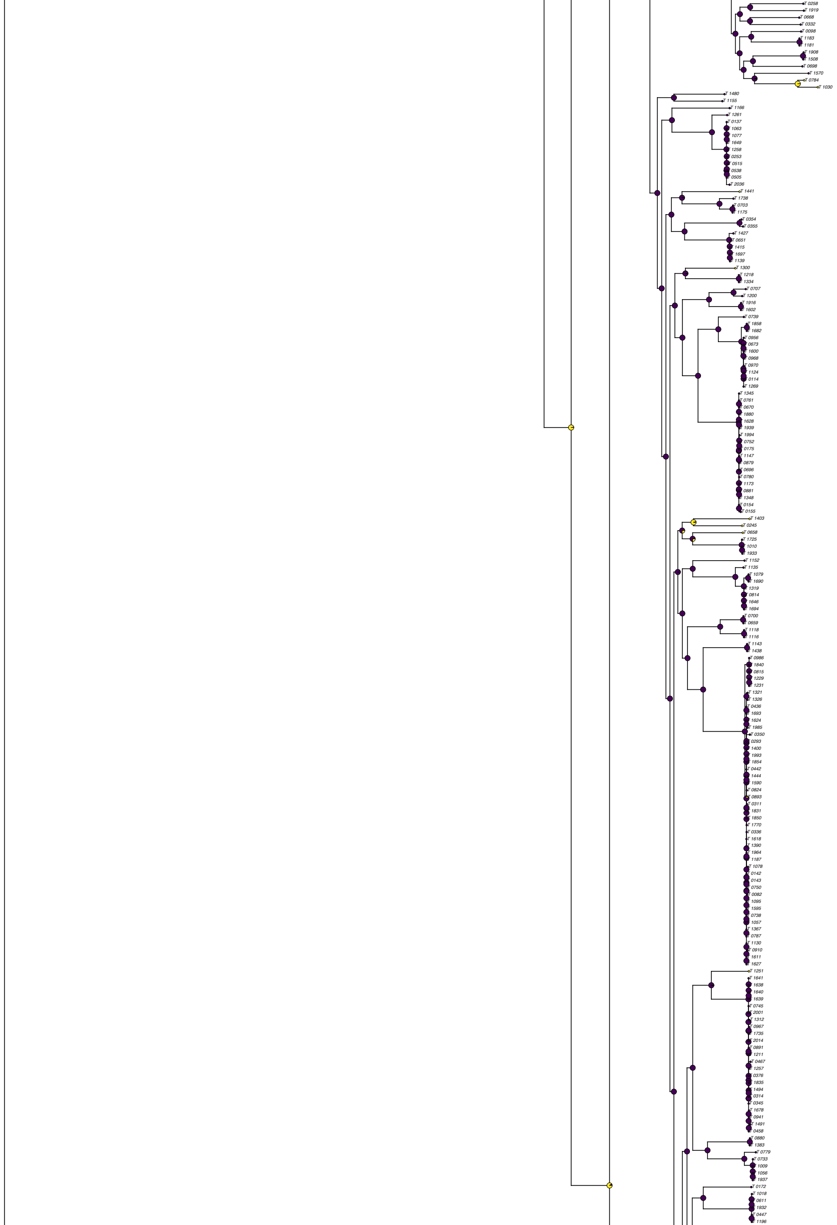

T 0258
T 1919
T 0668
T 0332
T 0098
T 1183
1181
T 1908
T 1508
T 0698
T 1570
T 0784
T 1030
T 1480
T 1155
T 1166
T 1261
T 0137
1063
1077
T 1649
1258
0253
T 0515
0538
T 0505
T 2036
T 1441
T 1738
T 0703
T 1175
T 0354
T 0355
T 1427
T 0651
1415
1697
T 1139
T 1300
T 1218
1334
T 0707
T 1200
T 1916
T 1602
T 0739
T 1858
T 1682
T 0956
0673
T 1600
0968
T 0970
T 1124
0114
T 1269
T 1345
T 0761
0670
1880
T 1628
1939
T 1994
T 0752
T 0175
T 1147
0879
T 0696
T 0780
T 1173
0881
T 1348
T 0154
T 0155
T 1403
T 0245
T 0658
T 1725
1010
T 1933
T 1152
T 1135
T 1079
T 1690
1319
0814
1646
T 1694
T 0700
T 0659
T 1118
T 1116
T 1143
T 1438
T 0986
1840
0815
1229
T 1231
T 1321
T 1326
T 0436
1693
1624
T 1985
T 0350
0293
1400
1993
1854
T 0442
1444
1590
T 0824
T 0893
T 0311
1831
1850
T 1770
T 0336
T 1618
T 1390
1964
1187
T 1078
0142
0143
0750
T 0082
1095
1595
0738
1057
T 1367
T 0787
T 1130
T 0910
1611
T 1627
T 1251
T 1641
1638
1640
1639
T 0745
T 2001
T 1312
T 0967
T 1735
2014
T 0891
1211
T 0467
T 1257
T 0376
1835
1494
0314
T 0345
T 1678
0941
T 1491
T 0458
T 0880
T 1383
T 0779
T 0733
1009
1056
T 1937
T 0172
T 1018
0611
1932
T 0447
T 1196

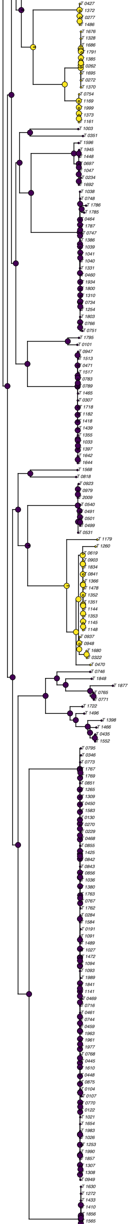
T 0427
T 1372
T 0277
T 1486
T 1676
T 1328
T 1686
T 1791
T 1385
T 0262
T 1695
T 0272
T 1370
T 0754
T 1169
T 1999
T 1373
T 1161
T 1003
T 0351
T 1596
T 1945
T 1448
T 0697
T 1047
T 0234
T 1692
T 1038
T 0748
T 1786
T 1785
T 0464
T 1787
T 0747
T 1386
T 1039
T 1041
T 1040
T 1331
T 0460
T 1934
T 1800
T 1310
T 0734
T 1254
T 1803
T 0766
T 0751
T 1795
T 0101
T 0947
T 1513
T 0471
T 1517
T 0783
T 0789
T 1465
T 0307
T 1718
T 1182
T 1418
T 1439
T 1355
T 1033
T 1397
T 1642
T 1644
T 1568
T 0818
T 0923
T 0979
T 2009
T 0540
T 0491
T 0501
T 0499
T 0531
T 1179
T 1260
T 0619
T 0903
T 1834
T 0841
T 1366
T 1478
T 1352
T 1351
T 1144
T 1353
T 1145
T 1148
T 0937
T 0948
T 1680
T 0322
T 0470
T 0746
T 1848
T 1877
T 0765
T 0771
T 1722
T 1496
T 1398
T 1466
T 0435
T 1552
T 0795
T 0346
T 0773
T 1767
T 1769
T 0851
T 1265
T 1309
T 0450
T 1583
T 0130
T 0270
T 0229
T 0468
T 0855
T 1425
T 0842
T 0843
T 0856
T 1036
T 1380
T 1763
T 0767
T 1762
T 0284
T 1584
T 0191
T 1091
T 1489
T 1027
T 1472
T 1094
T 1093
T 1989
T 1841
T 1141
T 0469
T 0716
T 0461
T 0744
T 0459
T 1963
T 1961
T 1977
T 0768
T 0445
T 1610
T 0448
T 0875
T 0104
T 0107
T 0770
T 0122
T 1021
T 1654
T 1983
T 1026
T 1253
T 1990
T 1857
T 1307
T 1308
T 0949
T 1630
T 1272
T 1433
T 1410
T 1856
T 1565

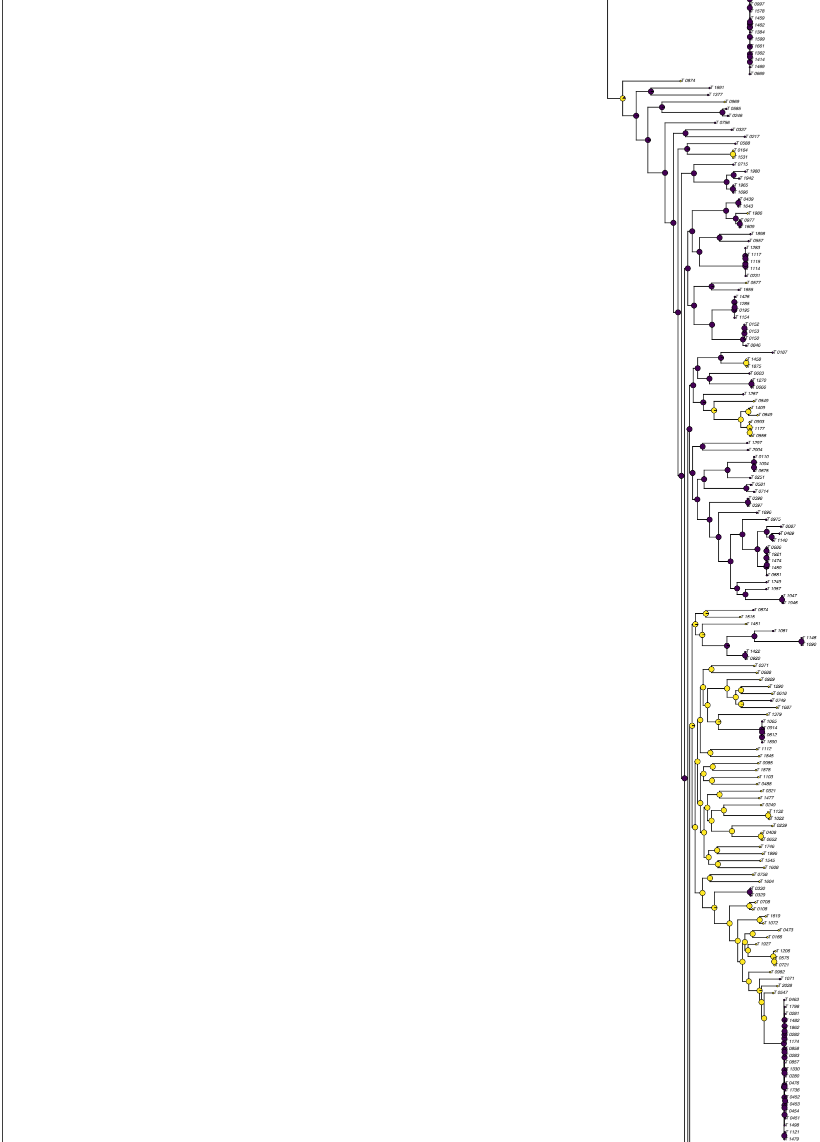
T 0997
T 1578
T 1459
T 1462
T 1384
T 1599
T 1661
T 1362
T 1414
T 1469
T 0669
T 0874
T 1691
T 1377
T 0969
T 0585
T 0246
T 0756
T 0337
T 0217
T 0588
T 0164
T 1531
T 0715
T 1980
T 1942
T 1965
T 1696
T 0439
T 1643
T 1986
T 0977
T 1609
T 1898
T 0557
T 1283
T 1117
T 1115
T 1114
T 0231
T 0577
T 1655
T 1426
T 1285
T 0195
T 1154
T 0152
T 0153
T 0150
T 0846
T 0187
T 1458
T 1875
T 0603
T 1270
T 0666
T 1267
T 0549
T 1409
T 0649
T 0993
T 1177
T 0556
T 1297
T 2004
T 0110
T 1004
T 0675
T 0251
T 0581
T 0714
T 0398
T 0397
T 1896
T 0975
T 0087
T 0489
T 1140
T 0686
T 1921
T 1474
T 1450
T 0681
T 1249
T 1957
T 1947
T 1946
T 0674
T 1515
T 1451
T 1061
T 1146
T 1090
T 1422
T 0920
T 0371
T 0688
T 0929
T 1290
T 0618
T 0749
T 1687
T 1379
T 1065
T 0914
T 0612
T 1890
T 1112
T 1845
T 0985
T 1878
T 1103
T 0488
T 0321
T 1477
T 0249
T 1132
T 1022
T 0239
T 0408
T 0652
T 1746
T 1996
T 1545
T 1608
T 0758
T 1604
T 0330
T 0329
T 0708
T 0108
T 1619
T 1072
T 0473
T 0166
T 1927
T 1206
T 0575
T 0721
T 0982
T 1071
T 2028
T 0547
T 0463
T 1798
T 0281
T 1482
T 1862
T 0282
T 1174
T 0858
T 0283
T 0857
T 1330
T 0280
T 0476
T 1736
T 0452
T 0453
T 0454
T 0451
T 1498
T 1121
T 1479

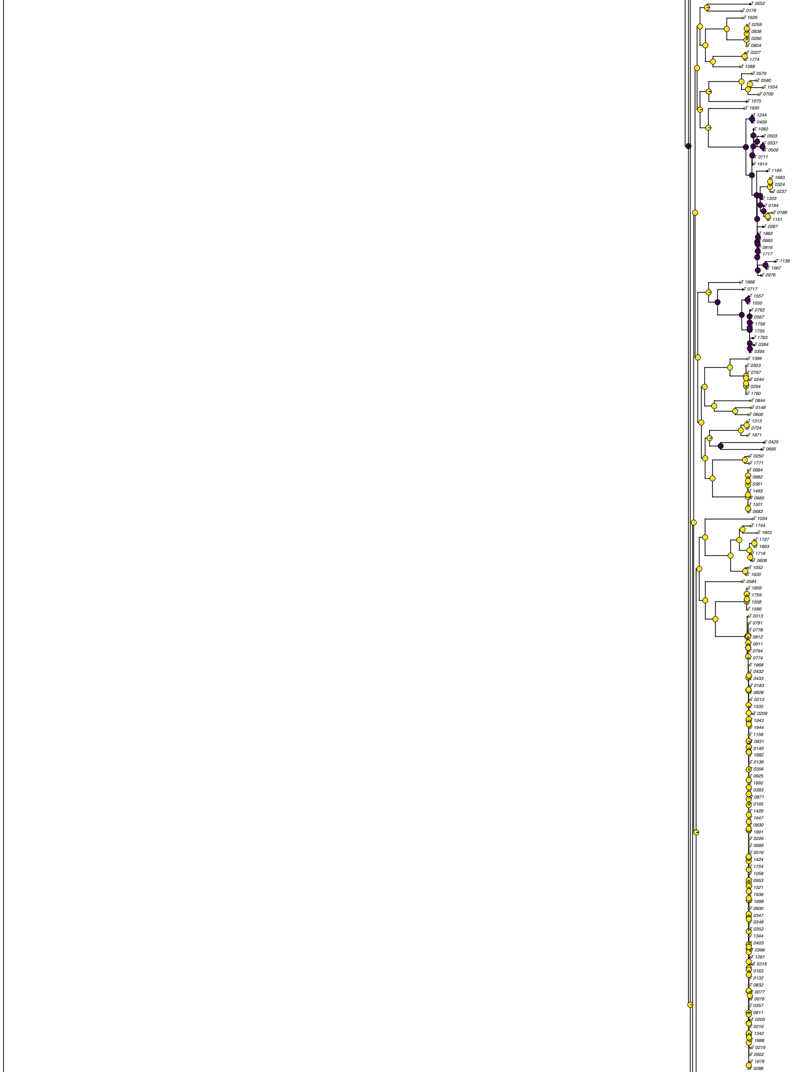

T 0552
T 0176
T 1626
T 0259
0838
0260
T 0804
T 0227
1774
T 1588
T 0579
T 0580
T 1554
T 0709
T 1075
T 1930
T 1244
T 0409
T 1082
T 0503
T 0537
T 0509
T 0711
T 1914
T 1164
T 1683
0324
T 0237
T 1203
T 0194
T 0186
1151
T 0287
T 1882
0665
T 0816
1717
T 1138
T 1567
T 0976
T 1988
T 0717
T 1557
1555
T 0762
0567
T 1756
1755
T 1783
T 0394
T 0395
T 1399
T 0303
T 0797
T 0244
0294
T 1760
T 0844
T 0148
T 0600
T 1313
0724
T 1871
T 0425
T 0695
T 0250
T 1771
T 0684
0682
0361
T 1493
T 0685
T 1501
0683
T 1034
T 1744
T 1603
T 1727
T 1663
T 1716
T 0608
T 1052
T 1620
T 0584
T 1859
1759
1558
T 1566
T 0313
T 0781
T 0778
0812
0911
T 0794
0774
T 1868
T 0432
0433
T 0183
T 0828
T 0213
1535
T 0209
1943
1944
T 1158
0831
0149
T 1982
T 0136
0356
T 0925
1892
0383
T 0871
0165
T 1429
1647
T 0830
1991
T 0226
T 0689
T 0516
1424
T 1754
T 1058
0953
1521
T 1936
1698
T 0930
0347
0348
T 0352
T 1344
0403
T 0396
T 1281
T 0318
0163
T 0132
T 0832
T 0077
T 0076
T 0357
T 0811
T 0205
T 0216
1342
T 1888
T 0219
T 2002
T 1679
T 0288

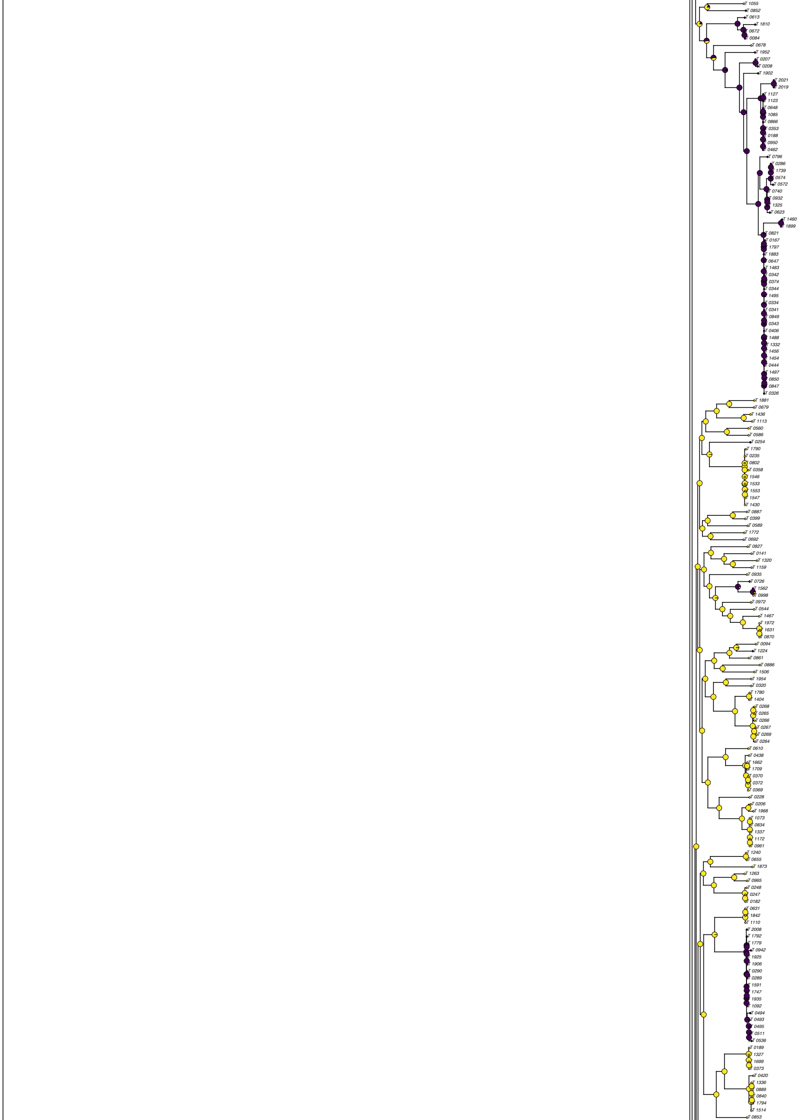
T 1055
T 0852
T 0613
T 1810
T 0672
T 0084
T 0678
T 1952
T 0207
T 0208
T 1902
T 2021
T 2019
T 1127
T 1123
T 0648
T 1085
T 0866
T 0353
T 0188
T 0950
T 0462
T 0796
T 0286
T 1739
T 0574
T 0572
T 0740
T 0932
T 1325
T 0623
T 1460
T 1899
T 0821
T 0167
T 1797
T 1883
T 0647
T 1483
T 0342
T 0374
T 0344
T 1495
T 0334
T 0341
T 0849
T 0343
T 0406
T 1488
T 1332
T 1456
T 1454
T 0444
T 1497
T 0850
T 0847
T 0326
T 1881
T 0679
T 1436
T 1113
T 0560
T 0586
T 0254
T 1790
T 0235
T 0802
T 0358
T 1546
T 1533
T 1553
T 1547
T 1430
T 0887
T 0399
T 0589
T 1772
T 0692
T 0827
T 0141
T 1320
T 1159
T 0935
T 0726
T 1562
T 0998
T 0972
T 0544
T 1467
T 1972
T 1631
T 0870
T 0094
T 1224
T 0861
T 0886
T 1506
T 1954
T 0320
T 1780
T 1404
T 0268
T 0265
T 0266
T 0267
T 0269
T 0264
T 0610
T 0438
T 1662
T 1709
T 0370
T 0372
T 0369
T 0228
T 0206
T 1968
T 1073
T 0834
T 1337
T 1172
T 0961
T 1240
T 0655
T 1873
T 1263
T 0965
T 0248
T 0247
T 0182
T 0631
T 1842
T 1110
T 2008
T 1792
T 1779
T 0942
T 1925
T 1906
T 0290
T 0289
T 1591
T 1747
T 1935
T 1092
T 0494
T 0493
T 0495
T 0511
T 0536
T 0189
T 1327
T 1699
T 0373
T 0420
T 1336
T 0889
T 0840
T 1794
T 1514
T 0853

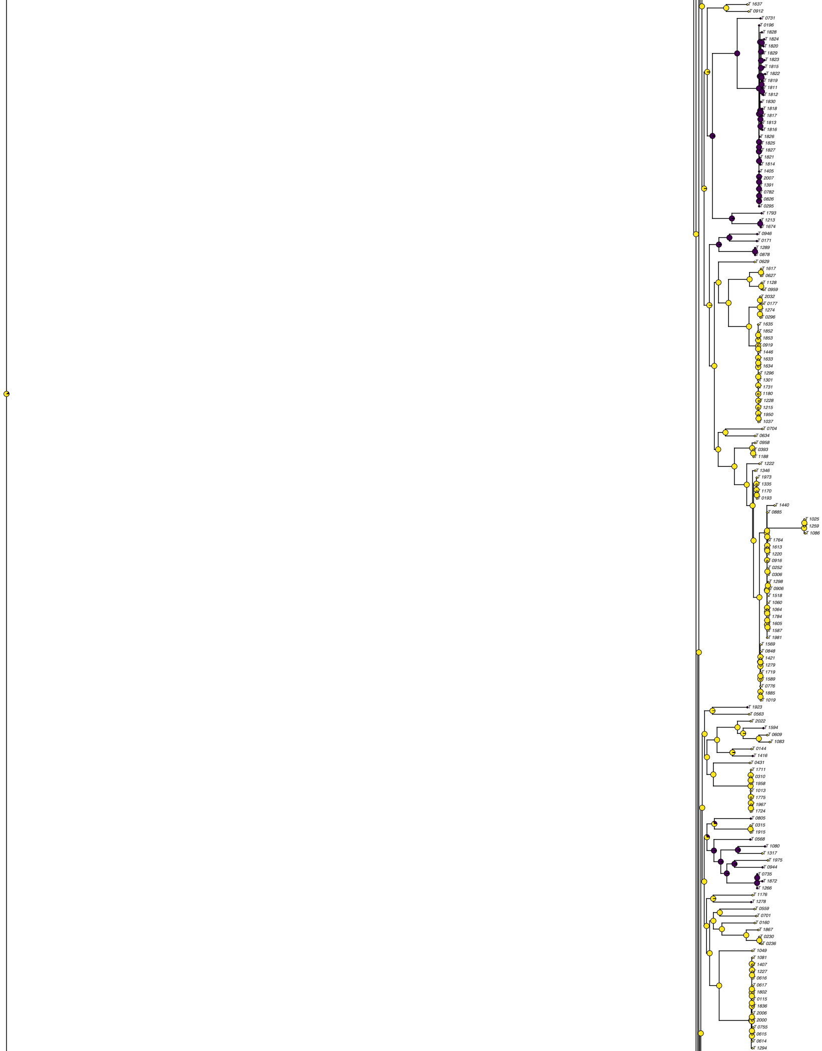

T 1637
T 0912
T 0731
T 0196
T 1828
T 1824
T 1820
T 1829
T 1823
T 1815
T 1822
T 1819
T 1811
T 1812
T 1830
T 1818
T 1817
T 1813
T 1816
T 1826
T 1825
T 1827
T 1821
T 1814
T 1405
2007
1391
0782
0826
T 0295
T 1793
T 1213
T 1674
T 0946
T 0171
T 1289
T 0878
T 0629
T 1617
T 0627
T 1128
T 0959
T 2032
T 0177
T 1274
T 0296
T 1635
T 1852
1853
0919
T 1446
1633
1634
T 1296
T 1301
1731
1180
T 1228
1215
1950
T 1037
T 0704
T 0634
T 0958
0393
T 1188
T 1222
T 1346
T 1973
1335
1170
T 0193
T 1440
T 0885
T 1025
1259
T 1086
T 1764
1613
T 1220
0916
T 0252
0306
T 1298
T 0906
T 1518
T 1060
1064
1784
1605
T 1587
T 1981
T 1569
T 0848
1421
1279
T 1719
1589
T 0776
1885
T 1019
T 1923
T 0563
T 2022
T 1594
T 0609
T 1083
T 0144
T 1416
T 0431
T 1711
0310
1958
T 1013
1775
1967
T 1724
T 0805
T 0315
T 1915
T 0568
T 1080
T 1317
T 1975
T 0944
T 0735
T 1872
T 1266
T 1176
T 1278
T 0559
T 0701
T 0160
T 1867
T 0230
T 0236
T 1049
T 1081
1407
1227
0616
T 0617
1802
T 0115
1836
T 2006
2000
T 0755
0615
T 0614
T 1294

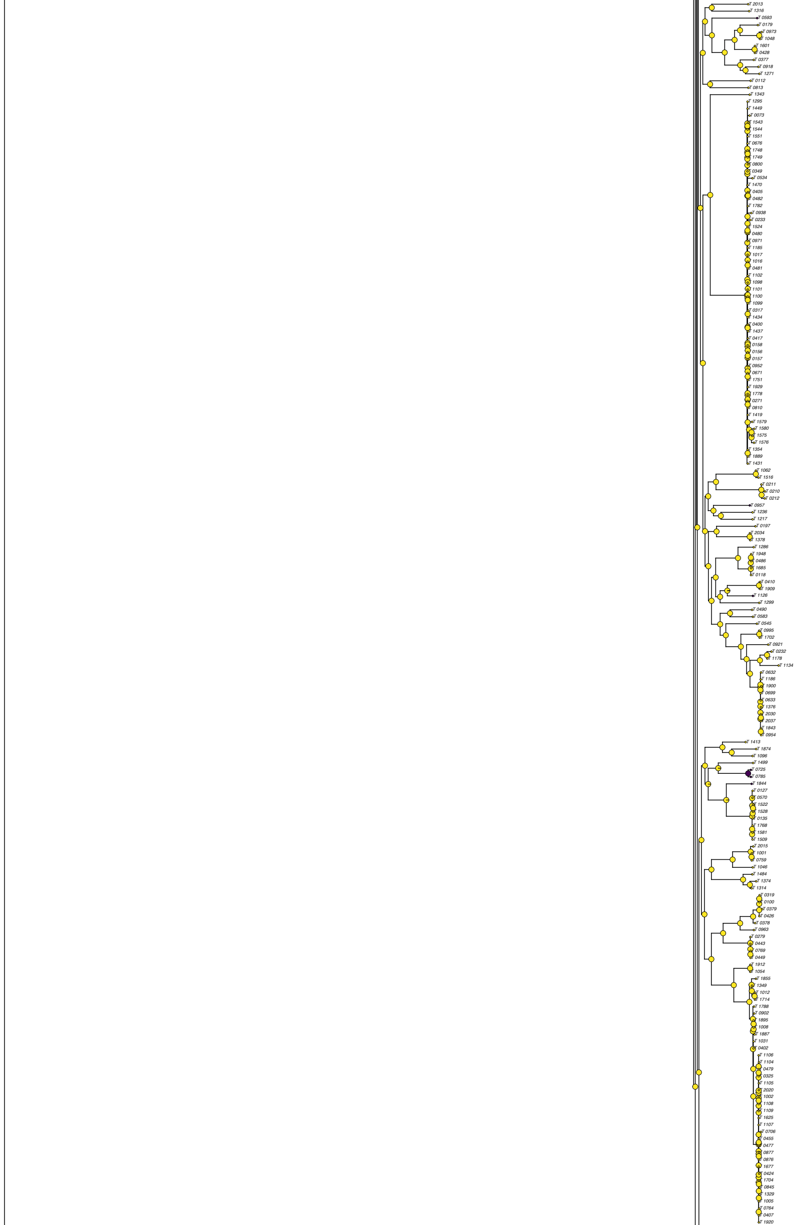
T 2013
T 1316
T 0593
T 0179
T 0973
T 1048
T 1601
T 0428
T 0377
T 0918
T 1271
T 0112
T 0813
T 1343
T 1295
T 1449
T 0073
T 1543
T 1544
T 1551
T 0676
T 1748
T 1749
T 0800
T 0349
T 0534
T 1470
T 0405
T 0482
T 1782
T 0938
T 0233
T 1524
T 0480
T 0971
T 1185
T 1017
T 1016
T 0481
T 1102
T 1098
T 1101
T 1100
T 1099
T 0317
T 1434
T 0400
T 1437
T 0417
T 0158
T 0156
T 0157
T 0952
T 0671
T 1751
T 1929
T 1778
T 0271
T 0810
T 1419
T 1579
T 1580
T 1575
T 1576
T 1354
T 1889
T 1431
T 1062
T 1516
T 0211
T 0210
T 0212
T 0957
T 1236
T 1217
T 0197
T 2034
T 1378
T 1286
T 1948
T 0486
T 1685
T 0118
T 0410
T 1909
T 1126
T 1299
T 0490
T 0583
T 0545
T 0995
T 1702
T 0921
T 0232
T 1178
T 1134
T 0632
T 1186
T 1900
T 0699
T 0633
T 1376
T 2030
T 2037
T 1843
T 0954
T 1413
T 1874
T 1096
T 1499
T 0725
T 0785
T 1844
T 0127
T 0570
T 1522
T 1528
T 0135
T 1768
T 1581
T 1509
T 2015
T 1001
T 0759
T 1046
T 1484
T 1374
T 1314
T 0319
T 0100
T 0379
T 0426
T 0378
T 0963
T 0279
T 0443
T 0769
T 0449
T 1912
T 1054
T 1855
T 1349
T 1012
T 1714
T 1788
T 0902
T 1895
T 1008
T 1887
T 1031
T 0402
T 1106
T 1104
T 0479
T 0325
T 1105
T 2020
T 1002
T 1108
T 1109
T 1625
T 1107
T 0706
T 0455
T 0477
T 0877
T 0876
T 1677
T 0424
T 1704
T 0845
T 1329
T 1005
T 0764
T 0407
T 1920

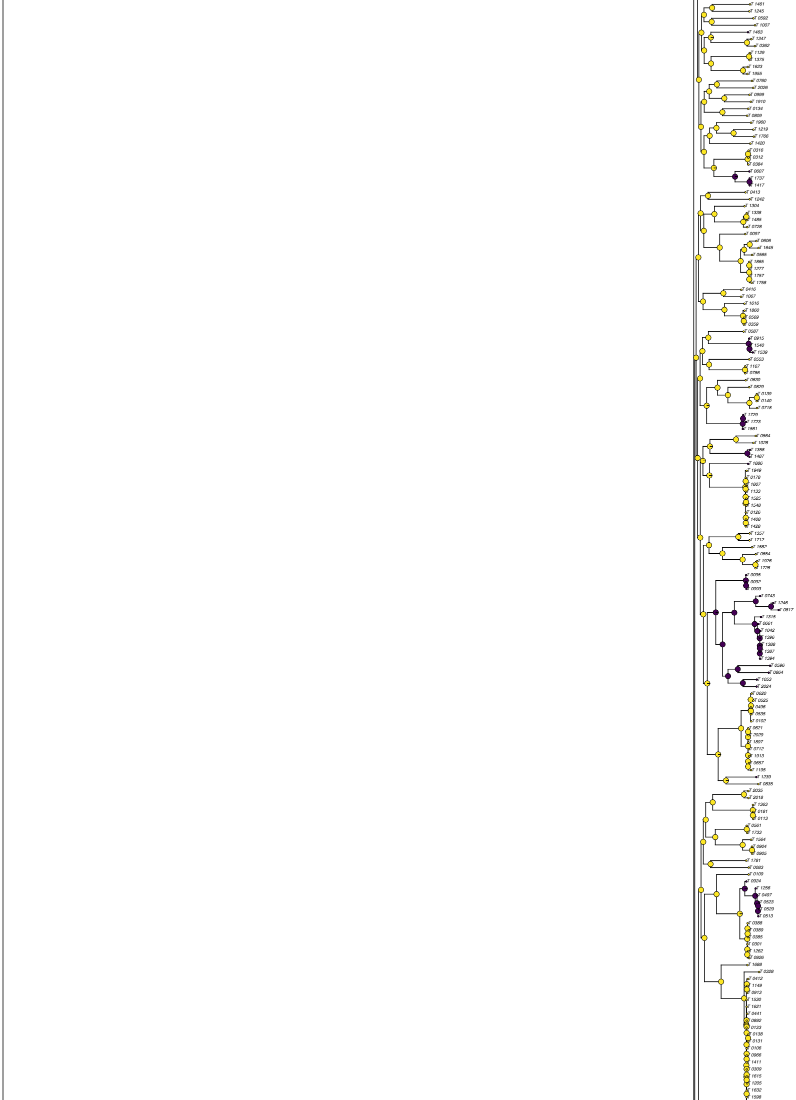
T 1461
T 1245
T 0592
T 1007
T 1463
T 1347
T 0362
T 1129
T 1375
T 1623
T 1955
T 0760
T 2026
T 0999
T 1910
T 0134
T 0809
T 1960
T 1219
T 1766
T 1420
T 0316
T 0312
T 0384
T 0607
T 1737
T 1417
T 0413
T 1242
T 1304
T 1338
T 1485
T 0728
T 0097
T 0606
T 1645
T 0565
T 1865
T 1277
T 1757
T 1758
T 0416
T 1067
T 1616
T 1860
T 0569
T 0359
T 0587
T 0915
T 1540
T 1539
T 0553
T 1167
T 0786
T 0630
T 0829
T 0139
T 0140
T 0718
T 1729
T 1723
T 1561
T 0564
T 1028
T 1358
T 1487
T 1886
T 1949
T 0178
T 1807
T 1133
T 1525
T 1548
T 0126
T 1408
T 1428
T 1357
T 1712
T 1582
T 0654
T 1926
T 1726
T 0095
T 0092
T 0093
T 0743
T 1246
T 0817
T 1315
T 0661
T 1042
T 1396
T 1388
T 1387
T 1394
T 0596
T 0864
T 1053
T 2024
T 0620
T 0525
T 0496
T 0535
T 0102
T 0621
T 2029
T 1897
T 0712
T 1913
T 0657
T 1195
T 1239
T 0835
T 2035
T 2018
T 1363
T 0181
T 0113
T 0561
T 1733
T 1564
T 0904
T 0905
T 1781
T 0083
T 0109
T 0924
T 1256
T 0497
T 0523
T 0529
T 0513
T 0388
T 0389
T 0385
T 0301
T 1262
T 0926
T 1688
T 0328
T 0412
T 1149
T 0913
T 1530
T 1621
T 0441
T 0892
T 0133
T 0138
T 0131
T 0106
T 0966
T 1411
T 0309
T 1615
T 1205
T 1632
T 1598

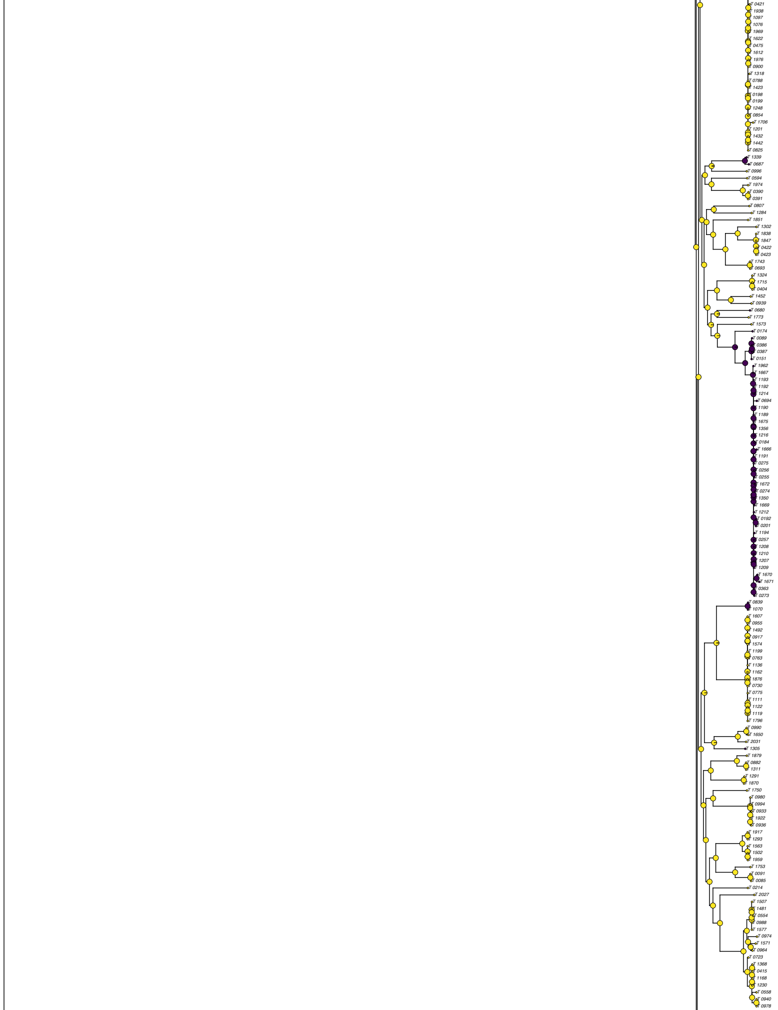
T 0421
T 1938
T 1097
T 1076
T 1969
T 1622
T 0475
T 1612
T 1976
T 0900
T 1318
T 0788
T 1423
T 0198
T 0199
T 1248
T 0854
T 1706
T 1201
T 1432
T 1442
T 0825
T 1339
T 0687
T 0996
T 0594
T 1974
T 0390
T 0391
T 0807
T 1284
T 1851
T 1302
T 1838
T 1847
T 0422
T 0423
T 1743
T 0693
T 1324
T 1715
T 0404
T 1452
T 0939
T 0680
T 1773
T 1573
T 0174
T 0089
T 0386
T 0387
T 0151
T 1962
T 1667
T 1193
T 1192
T 1214
T 0694
T 1190
T 1189
T 1675
T 1356
T 1216
T 0184
T 1666
T 1191
T 0275
T 0256
T 0255
T 1672
T 0274
T 1350
T 1669
T 1212
T 0192
T 0201
T 1194
T 0257
T 1208
T 1210
T 1207
T 1209
T 1670
T 1671
T 0363
T 0273
T 0839
T 1070
T 1607
T 0955
T 1492
T 0917
T 1574
T 1199
T 0763
T 1136
T 1162
T 1876
T 0730
T 0775
T 1111
T 1122
T 1119
T 1796
T 0990
T 1650
T 2031
T 1305
T 1879
T 0882
T 1311
T 1291
T 1870
T 1750
T 0980
T 0994
T 0933
T 1922
T 0936
T 1917
T 1293
T 1563
T 1502
T 1959
T 1753
T 0091
T 0085
T 0214
T 2027
T 1507
T 1481
T 0554
T 0988
T 1577
T 0974
T 1571
T 0964
T 0723
T 1368
T 0415
T 1168
T 1230
T 0558
T 0940
T 0978

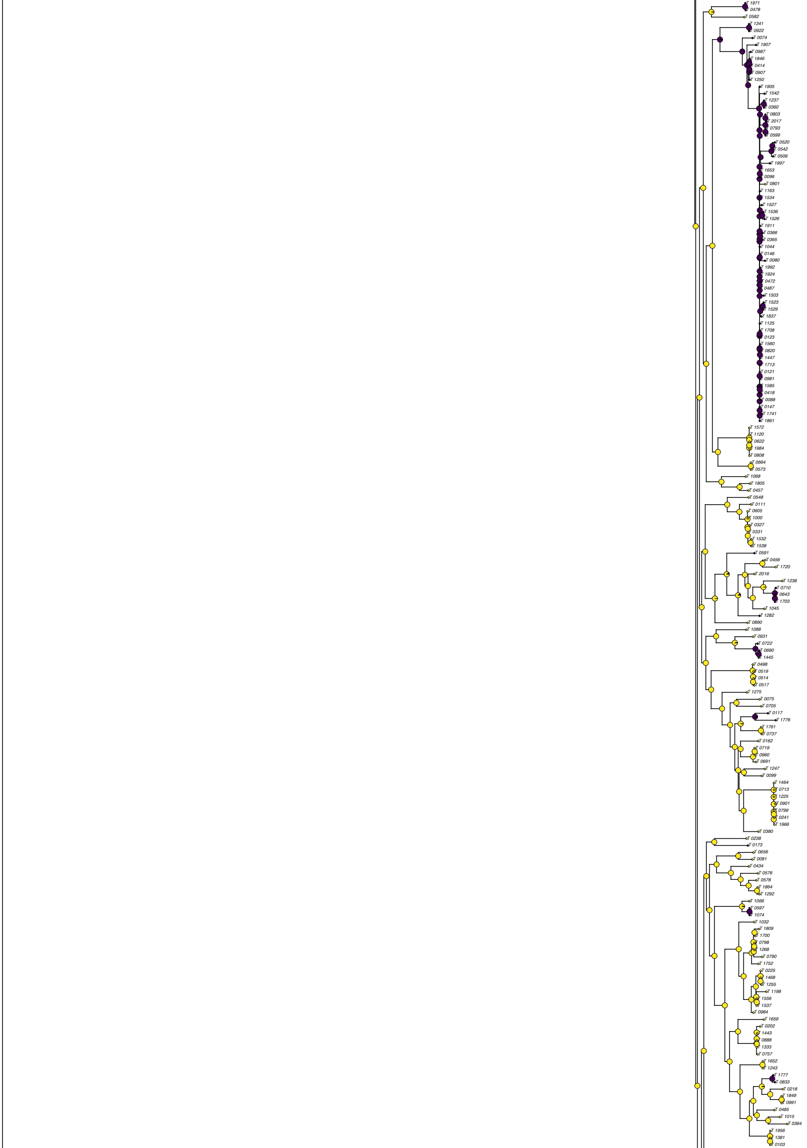
T 1971
T 0478
T 0582
T 1341
T 0922
T 0074
T 1907
T 0987
T 1846
T 0414
T 0907
T 1250
T 1905
T 1542
T 1237
T 0360
T 0803
T 2017
T 0793
T 0599
T 0520
T 0542
T 0506
T 1997
T 1653
T 0096
T 0801
T 1163
T 1534
T 1527
T 1536
T 1526
T 1911
T 0366
T 0365
T 1044
T 0146
T 0080
T 1992
T 1924
T 0472
T 0487
T 1503
T 1523
T 1529
T 1837
T 1125
T 1708
T 0123
T 1560
T 0820
T 1447
T 1713
T 0121
T 0981
T 1585
T 0418
T 0088
T 0147
T 1741
T 1861
T 1572
T 1120
T 0622
T 1984
T 0808
T 0664
T 0573
T 1068
T 1805
T 0457
T 0548
T 0111
T 0605
T 1000
T 0327
T 0331
T 1532
T 1538
T 0591
T 0456
T 1720
T 2016
T 1238
T 0710
T 0643
T 1703
T 1045
T 1282
T 0890
T 1088
T 0931
T 0722
T 0690
T 1445
T 0498
T 0519
T 0514
T 0517
T 1275
T 0075
T 0705
T 0117
T 1776
T 1761
T 0737
T 0162
T 0719
T 0960
T 0691
T 1247
T 0099
T 1464
T 0713
T 1225
T 0901
T 0799
T 0241
T 1966
T 0380
T 0238
T 0173
T 0656
T 0081
T 0434
T 0576
T 0578
T 1864
T 1292
T 1066
T 0597
T 1074
T 1032
T 1809
T 1700
T 0798
T 1268
T 0790
T 1752
T 0225
T 1468
T 1255
T 1198
T 1556
T 1537
T 0984
T 1659
T 0202
T 1443
T 0888
T 1333
T 0757
T 1652
T 1243
T 1777
T 0833
T 0218
T 1849
T 0991
T 0485
T 1015
T 0364
T 1956
T 1381
T 0103

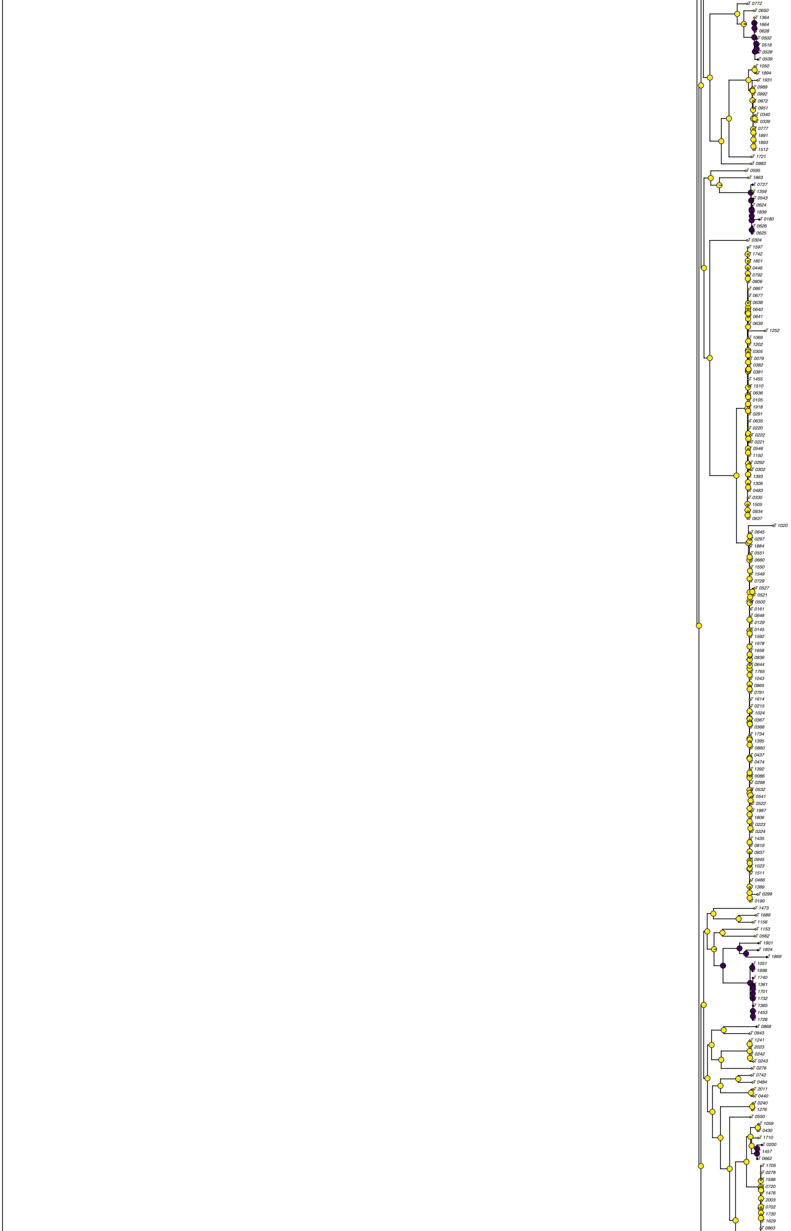

T 0772
T 0650
T 1364
1664
0628
T 0502
T 0518
T 0528
T 0539
T 1050
T 1894
T 1931
0989
0992
0872
0951
T 0340
0339
T 0777
1891
1893
1512
T 1721
T 0983
T 0595
T 1863
T 0727
T 1359
T 0543
T 0624
1839
T 0180
T 0626
0625
T 0304
T 1597
1742
1801
0446
0792
0806
T 0867
T 0677
0638
0640
0641
0639
T 1252
T 1069
T 1202
0305
0079
0382
0381
T 1455
1510
T 0636
0105
1918
T 0291
T 0635
T 0220
T 0222
T 0221
0546
1150
0292
T 0302
1393
1306
0483
T 0335
1505
0934
0637
T 1020
T 0645
0297
T 1884
T 0551
0660
T 1550
1549
0729
T 0527
T 0521
T 0500
T 0161
T 0646
0129
0145
1592
T 1978
1658
0836
0644
1765
T 1043
0865
0791
T 1614
T 0215
1024
0367
0368
T 1734
1395
0860
T 0437
T 0474
T 1392
0086
T 0298
0532
0541
T 0522
T 1987
1806
0223
0224
T 1435
0819
0837
0945
1023
T 1511
T 0466
1389
T 0299
0190
T 1473
T 1689
T 1156
T 1153
T 0562
T 1901
T 1804
T 1869
T 1051
1998
T 1740
1361
1701
1732
T 1365
1453
T 1728
T 0868
T 0943
T 1241
2023
0242
T 0243
T 0276
T 0742
T 0484
T 2011
T 0440
T 0240
1276
T 0550
T 1059
0430
T 1710
T 0200
1457
T 0662
T 1705
T 0278
1586
0720
1476
2003
0702
1730
1629
T 0863

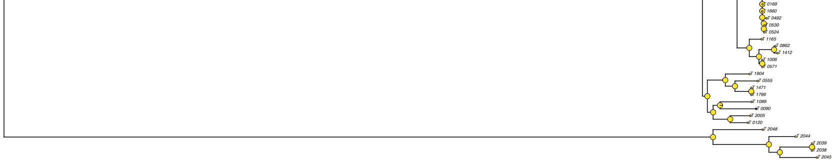
T 0169
T 1660
T 0492
T 0530
T 0524
T 1165
T 0862
T 1412
T 1006
T 0571
T 1904
T 0555
T 1471
T 1799
T 1089
T 0090
T 2005
T 0120
T 2048
T 2044
T 2039
T 2038
T 2045

tseF
X

Effector gene: tseF

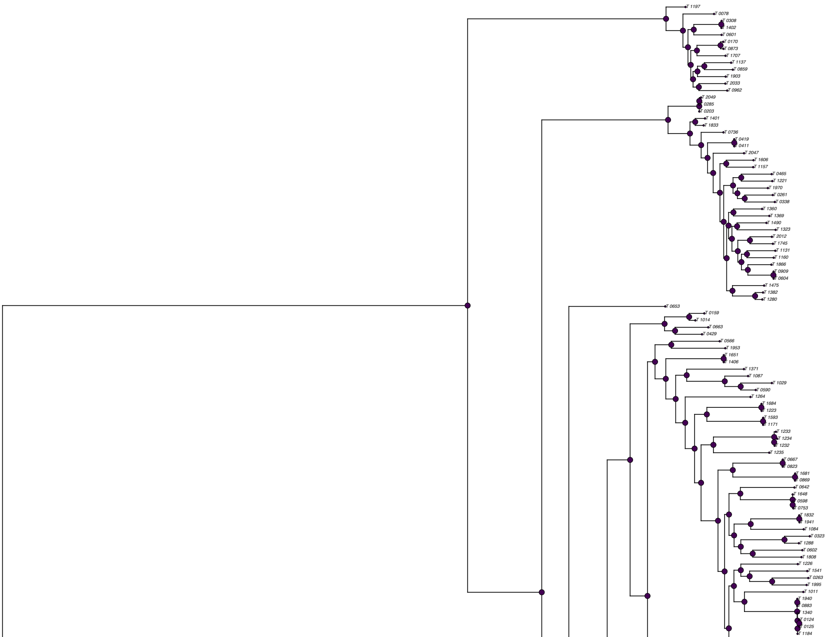

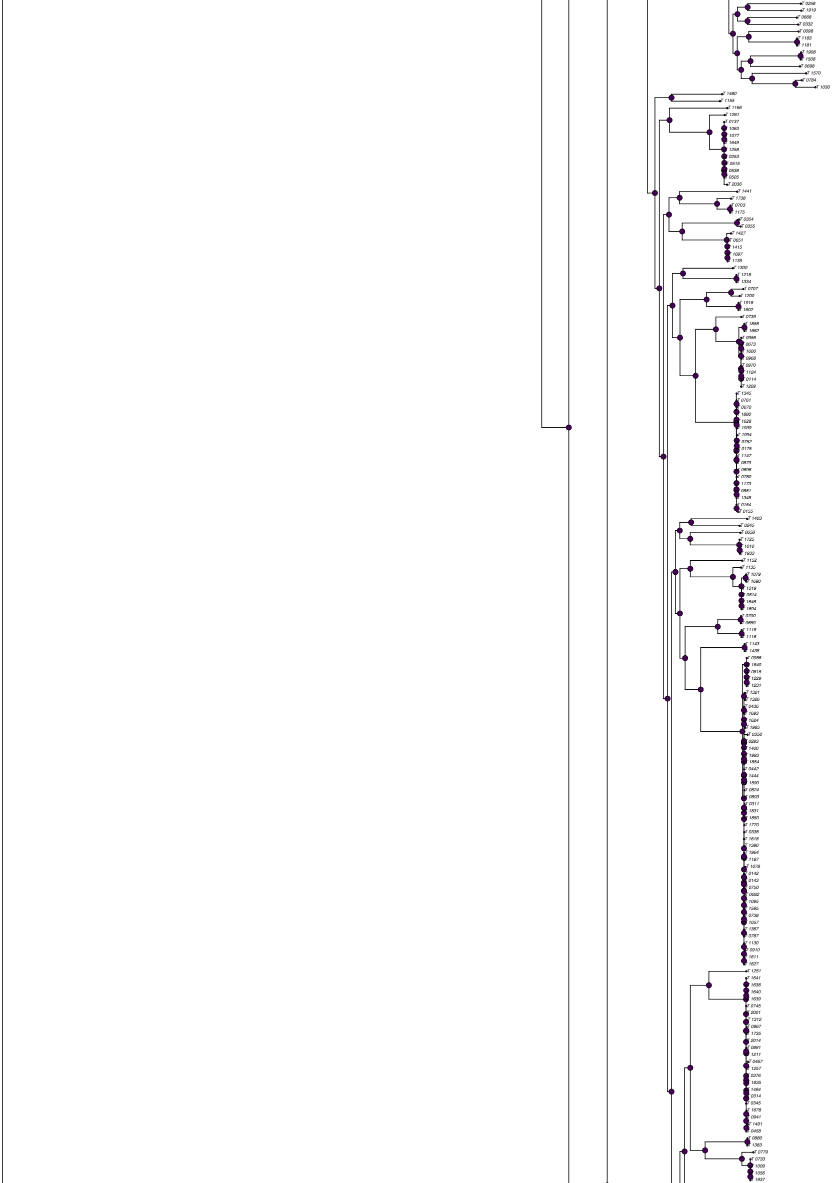
T 0258
T 1919
T 0668
T 0332
T 0098
T 1183
T 1181
T 1908
T 1508
T 0698
T 1570
T 0784
T 1030
T 1480
T 1155
T 1166
T 1261
T 0137
1063
1077
T 1649
1258
0253
0515
0538
0505
T 2036
T 1441
T 1738
T 0703
T 1175
T 0354
T 0355
T 1427
T 0651
1415
1697
T 1139
T 1300
T 1218
T 1334
T 0707
T 1200
T 1916
T 1602
T 0739
T 1858
T 1682
T 0956
0673
T 1600
T 0968
T 0970
T 1124
T 0114
T 1269
T 1345
T 0761
T 0670
1880
1628
T 1939
T 1994
0752
0175
T 1147
T 0879
T 0696
T 0780
1173
0881
T 1348
T 0154
T 0155
T 1403
T 0245
T 0658
T 1725
1010
T 1933
T 1152
T 1135
T 1079
T 1690
1319
0814
1646
T 1694
T 0700
T 0659
T 1118
T 1116
T 1143
T 1438
T 0986
1840
0815
1229
T 1231
T 1321
T 1326
T 0436
1693
1624
T 1985
T 0350
0293
1400
1993
1854
T 0442
1444
1590
T 0824
0893
T 0311
1831
1850
T 1770
T 0336
T 1618
T 1390
1964
1187
T 1078
0142
0143
0750
T 0082
1095
1595
T 0738
1057
T 1367
T 0787
T 1130
T 0910
1611
T 1627
T 1251
T 1641
1638
1640
1639
T 0745
2001
T 1312
T 0967
T 1735
2014
T 0891
1211
T 0467
1257
0376
1835
1494
0314
T 0345
T 1678
0941
T 1491
T 0458
T 0880
T 1383
T 0779
T 0733
1009
1056
T 1937

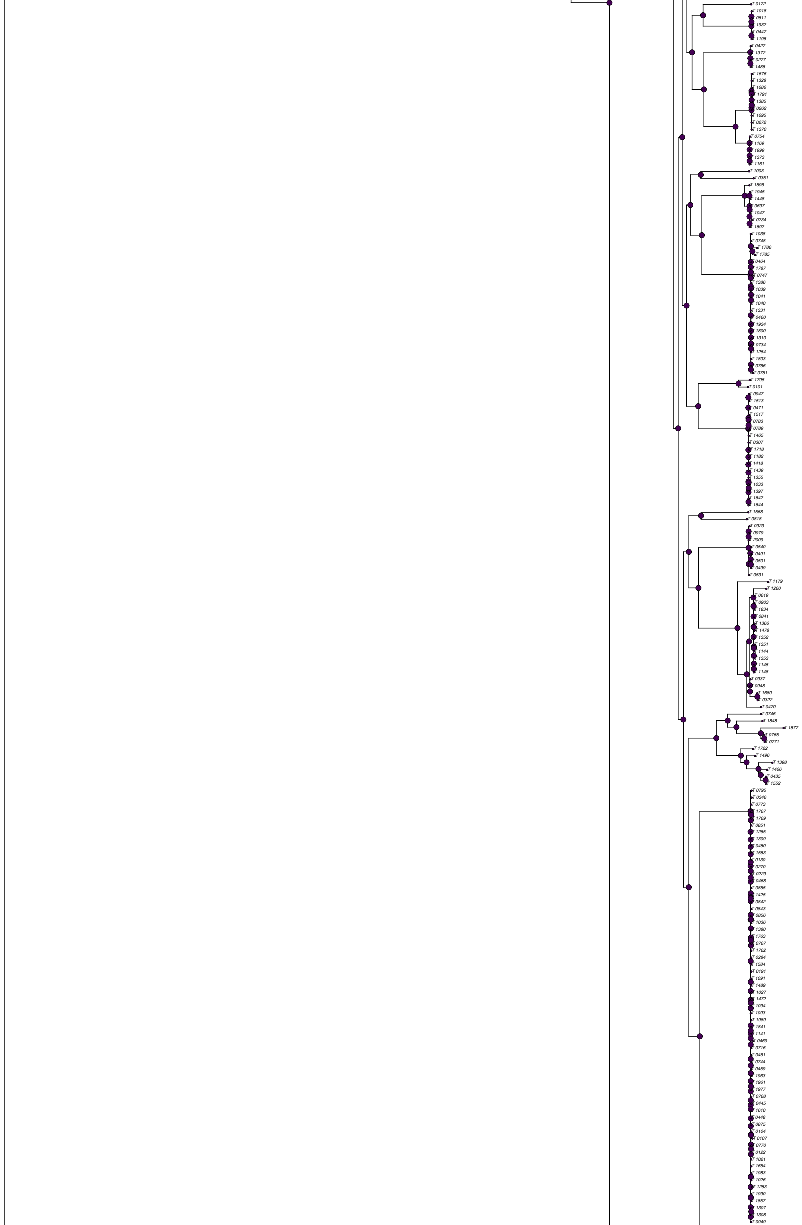
T 0172
T 1018
0611
1932
T 0447
T 1196
T 0427
1372
0277
T 1486
T 1676
T 1328
T 1686
1791
1385
0262
T 1695
T 0272
T 1370
T 0754
1169
1999
1373
T 1161
T 1003
T 0351
T 1596
T 1945
T 1448
0697
T 1047
T 0234
T 1692
T 1038
T 0748
T 1786
T 1785
0464
1787
T 0747
T 1386
1039
1041
1040
T 1331
0460
1934
1800
1310
0734
T 1254
T 1803
0766
T 0751
T 1795
T 0101
T 0947
T 1513
0471
T 1517
0783
0789
T 1465
T 0307
T 1718
T 1182
1418
T 1439
T 1355
1033
1397
T 1642
T 1644
T 1568
T 0818
T 0923
0979
T 2009
T 0540
0491
0501
T 0499
T 0531
T 1179
T 1260
0619
T 0903
T 1834
0841
T 1366
T 1478
1352
1351
1144
1353
1145
T 1148
T 0937
0948
T 1680
T 0322
T 0470
T 0746
T 1848
T 1877
T 0765
T 0771
T 1722
T 1496
T 1398
T 1466
T 0435
T 1552
T 0795
T 0346
T 0773
1767
1769
T 0851
1265
1309
0450
1583
0130
0270
T 0229
0468
T 0855
1425
0842
T 0843
0856
T 1036
1380
T 1763
0767
T 1762
T 0284
T 1584
T 0191
T 1091
1489
1027
T 1472
1094
T 1093
T 1989
1841
1141
T 0469
T 0716
T 0461
0744
T 0459
1963
1961
1977
T 0768
0445
1610
0448
T 0875
T 0104
T 0107
0770
0122
T 1021
T 1654
T 1983
1026
T 1253
T 1990
T 1857
T 1307
1308
T 0949

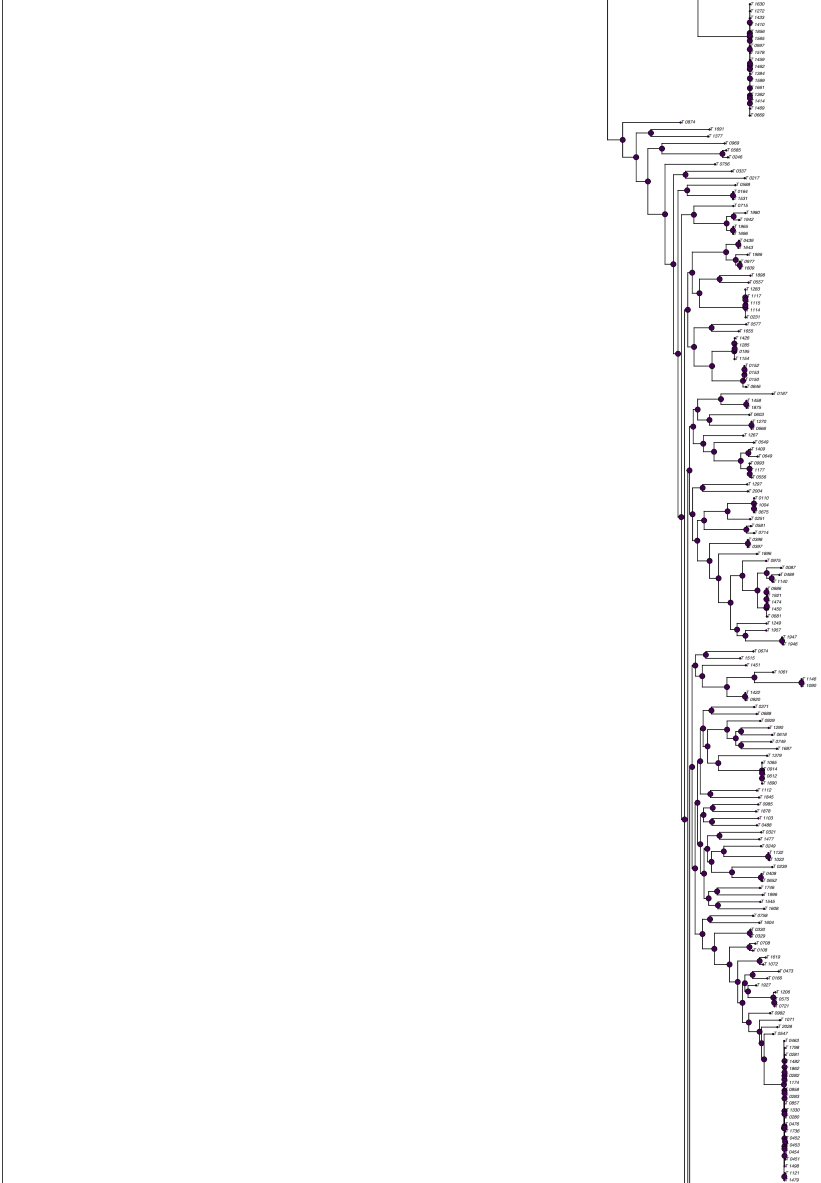
T 1630
T 1272
T 1433
T 1410
1856
1565
T 0997
T 1578
T 1459
1462
T 1384
1599
1661
1362
1414
T 1469
T 0669
T 0874
T 1691
T 1377
T 0969
T 0585
T 0246
T 0756
T 0337
T 0217
T 0588
T 0164
T 1531
T 0715
T 1980
T 1942
T 1965
T 1696
T 0439
T 1643
T 1986
T 0977
T 1609
T 1898
T 0557
T 1283
T 1117
T 1115
T 1114
T 0231
T 0577
T 1655
T 1426
1285
0195
T 1154
T 0152
0153
T 0150
T 0846
T 0187
T 1458
T 1875
T 0603
T 1270
T 0666
T 1267
T 0549
T 1409
T 0649
T 0993
1177
T 0556
T 1297
T 2004
T 0110
1004
T 0675
T 0251
T 0581
T 0714
T 0398
T 0397
T 1896
T 0975
T 0087
T 0489
T 1140
T 0686
T 1921
1474
1450
T 0681
T 1249
T 1957
T 1947
T 1946
T 0674
T 1515
T 1451
T 1061
T 1146
T 1090
T 1422
T 0920
T 0371
T 0688
T 0929
T 1290
T 0618
T 0749
T 1687
T 1379
T 1065
0914
0612
T 1890
T 1112
T 1845
T 0985
T 1878
T 1103
T 0488
T 0321
T 1477
T 0249
T 1132
T 1022
T 0239
T 0408
T 0652
T 1746
T 1996
T 1545
T 1608
T 0758
T 1604
T 0330
T 0329
T 0708
T 0108
T 1619
T 1072
T 0473
T 0166
T 1927
T 1206
0575
T 0721
T 0982
T 1071
T 2028
T 0547
T 0463
T 1798
T 0281
1482
1862
0282
1174
0858
0283
T 0857
1330
0280
T 0476
T 1736
0452
0453
0454
T 0451
T 1498
T 1121
T 1479

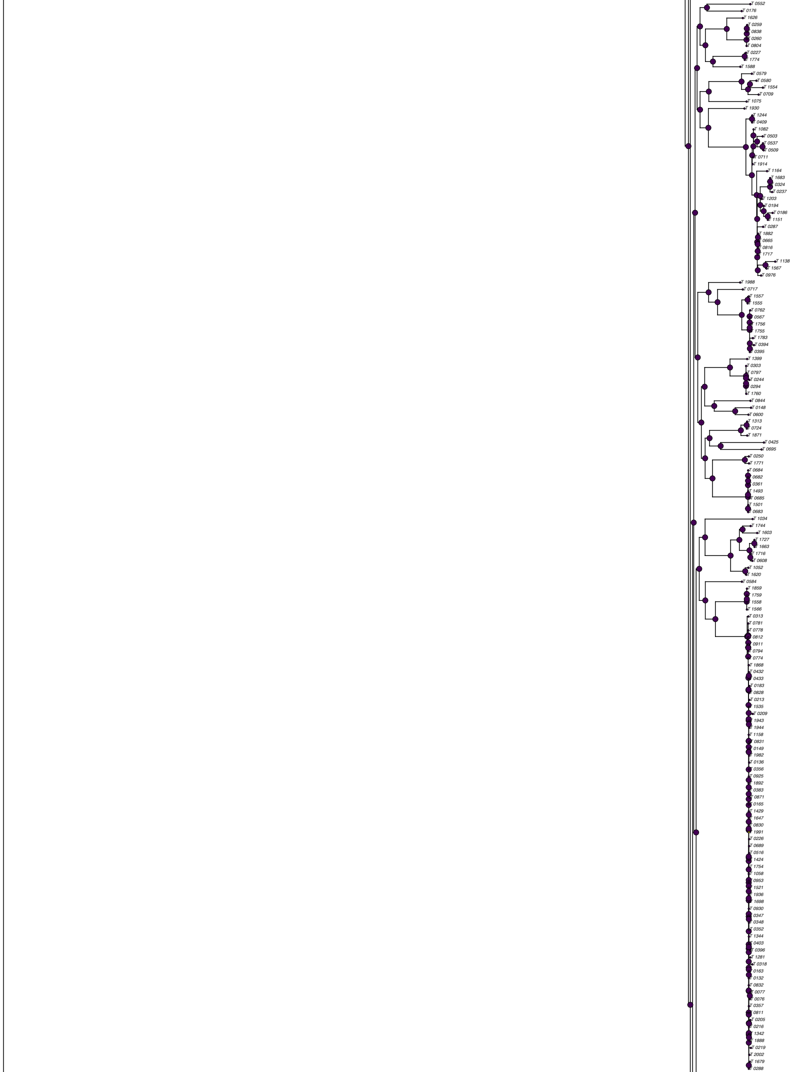
T 0552
T 0176
T 1626
T 0259
0838
0260
T 0804
T 0227
T 1774
T 1588
T 0579
T 0580
T 1554
T 0709
T 1075
T 1930
T 1244
T 0409
T 1082
T 0503
T 0537
T 0509
T 0711
T 1914
T 1164
T 1683
0324
T 0237
T 1203
T 0194
T 0186
T 1151
T 0287
T 1882
0665
T 0816
1717
T 1138
T 1567
T 0976
T 1988
T 0717
T 1557
T 1555
T 0762
0567
T 1756
1755
T 1783
T 0394
T 0395
T 1399
T 0303
T 0797
T 0244
0294
T 1760
T 0844
T 0148
T 0600
T 1313
T 0724
T 1871
T 0425
T 0695
T 0250
T 1771
T 0684
0682
0361
T 1493
T 0685
T 1501
T 0683
T 1034
T 1744
T 1603
T 1727
T 1663
T 1716
T 0608
T 1052
T 1620
T 0584
T 1859
1759
1558
T 1566
T 0313
T 0781
T 0778
0812
0911
T 0794
0774
T 1868
T 0432
0433
T 0183
T 0828
T 0213
1535
T 0209
1943
T 1944
T 1158
0831
0149
T 1982
T 0136
0356
T 0925
T 1892
0383
T 0871
0165
T 1429
T 1647
T 0830
T 1991
T 0226
T 0689
T 0516
1424
T 1754
T 1058
0953
1521
T 1936
1698
T 0930
0347
T 0348
T 0352
T 1344
T 0403
T 0396
T 1281
T 0318
0163
T 0132
T 0832
T 0077
T 0076
T 0357
0811
T 0205
T 0216
1342
T 1888
T 0219
T 2002
T 1679
T 0288

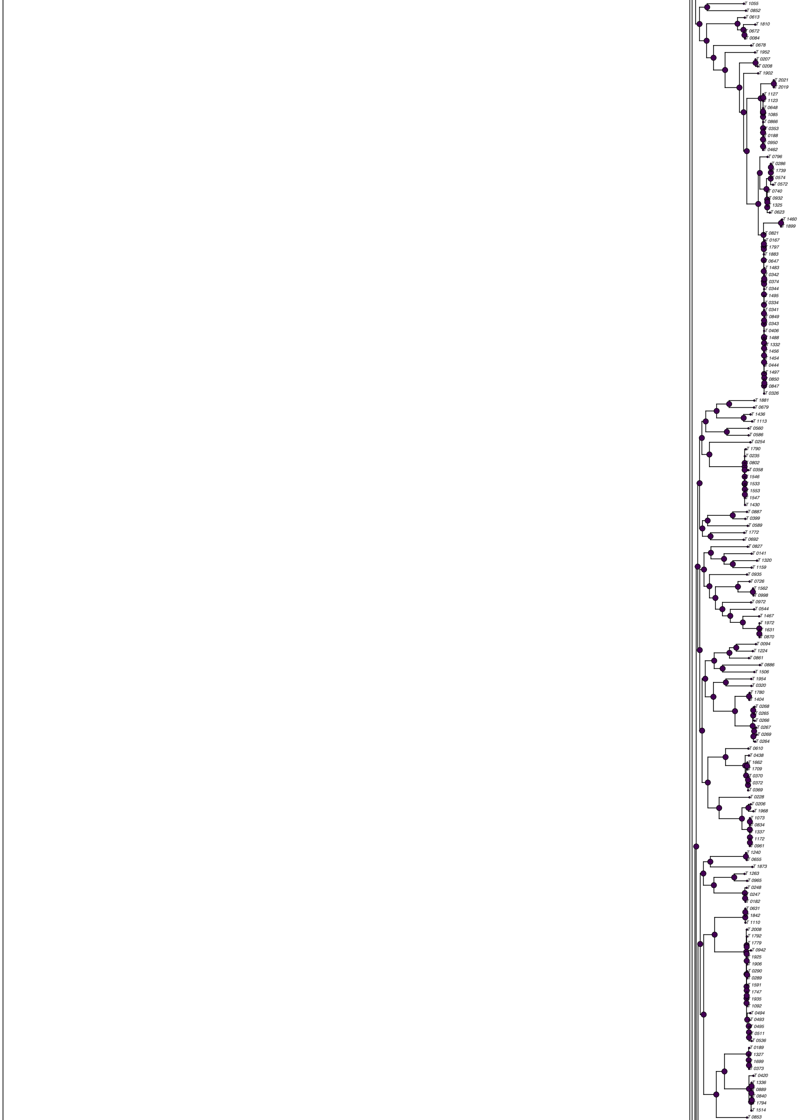
T 1055
T 0852
T 0613
T 1810
0672
T 0084
T 0678
T 1952
T 0207
T 0208
T 1902
T 2021
T 2019
T 1127
T 1123
T 0648
1085
T 0866
T 0353
T 0188
T 0950
T 0462
T 0796
T 0286
T 1739
T 0574
T 0572
T 0740
T 0932
T 1325
T 0623
T 1460
T 1899
T 0821
T 0167
T 1797
T 1883
0647
T 1483
T 0342
T 0374
T 0344
T 1495
T 0334
T 0341
T 0849
T 0343
T 0406
T 1488
T 1332
T 1456
T 1454
T 0444
T 1497
T 0850
T 0847
T 0326
T 1881
T 0679
T 1436
T 1113
T 0560
T 0586
T 0254
T 1790
T 0235
0802
T 0358
1546
1533
1553
T 1547
T 1430
T 0887
T 0399
T 0589
T 1772
T 0692
T 0827
T 0141
T 1320
T 1159
T 0935
T 0726
T 1562
T 0998
T 0972
T 0544
T 1467
T 1972
1631
T 0870
T 0094
T 1224
T 0861
T 0886
T 1506
T 1954
T 0320
T 1780
T 1404
T 0268
T 0265
T 0266
T 0267
T 0269
T 0264
T 0610
T 0438
T 1662
T 1709
T 0370
T 0372
T 0369
T 0228
T 0206
T 1968
T 1073
T 0834
T 1337
T 1172
T 0961
T 1240
T 0655
T 1873
T 1263
T 0965
T 0248
T 0247
T 0182
T 0631
T 1842
T 1110
T 2008
T 1792
T 1779
T 0942
T 1925
T 1906
T 0290
T 0289
T 1591
T 1747
T 1935
T 1092
T 0494
T 0493
T 0495
T 0511
T 0536
T 0189
T 1327
T 1699
T 0373
T 0420
T 1336
T 0889
T 0840
T 1794
T 1514
T 0853

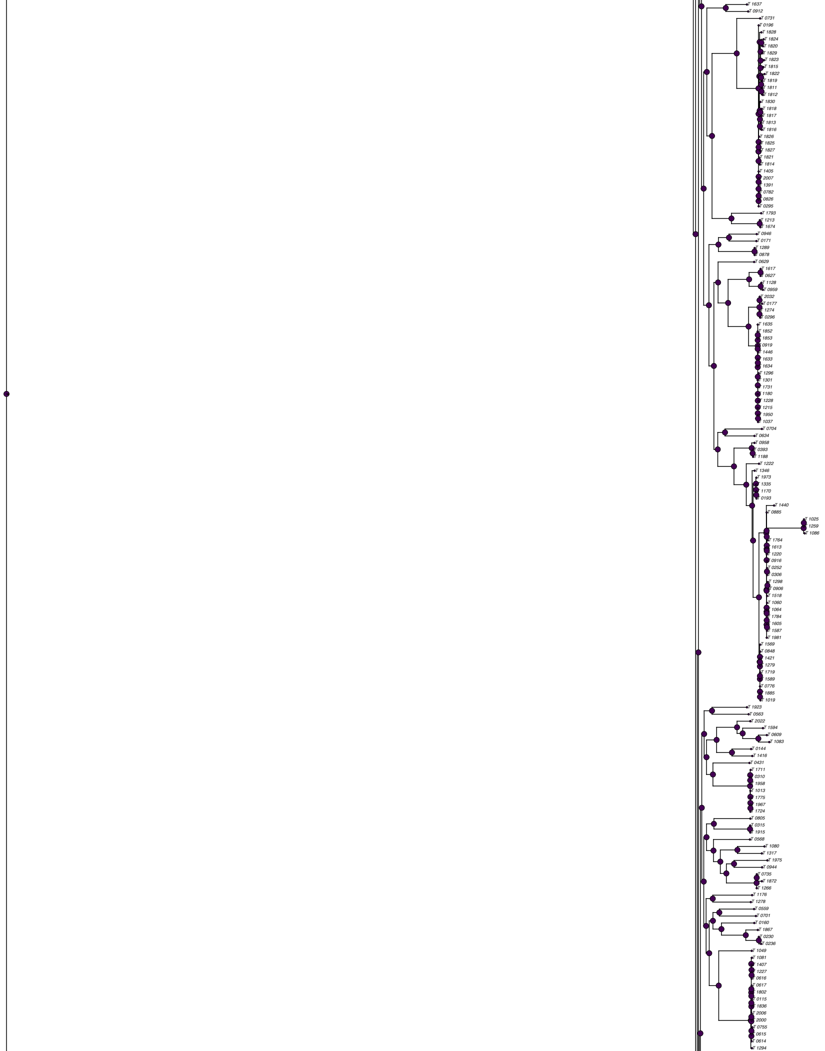
T 1637
T 0912
T 0731
T 0196
T 1828
T 1824
T 1820
T 1829
T 1823
T 1815
T 1822
T 1819
T 1811
T 1812
T 1830
T 1818
T 1817
T 1813
T 1816
T 1826
T 1825
T 1827
T 1821
T 1814
T 1405
2007
1391
0782
0826
T 0295
T 1793
T 1213
T 1674
T 0946
T 0171
T 1289
T 0878
T 0629
T 1617
T 0627
T 1128
T 0959
T 2032
T 0177
T 1274
T 0296
T 1635
T 1852
1853
0919
T 1446
1633
1634
T 1296
T 1301
1731
1180
1228
1215
1950
T 1037
T 0704
T 0634
T 0958
T 0393
T 1188
T 1222
T 1346
T 1973
1335
1170
T 0193
T 1440
T 0885
T 1025
1259
T 1086
T 1764
1613
T 1220
0916
T 0252
T 0306
T 1298
T 0906
T 1518
T 1060
1064
T 1784
1605
T 1587
T 1981
T 1569
T 0848
1421
1279
T 1719
1589
T 0776
1885
T 1019
T 1923
T 0563
T 2022
T 1594
T 0609
T 1083
T 0144
T 1416
T 0431
T 1711
0310
1958
T 1013
1775
1967
1724
T 0805
T 0315
T 1915
T 0568
T 1080
T 1317
T 1975
T 0944
T 0735
T 1872
T 1266
T 1176
T 1278
T 0559
T 0701
T 0160
T 1867
T 0230
T 0236
T 1049
T 1081
1407
1227
0616
T 0617
1802
0115
1836
T 2006
2000
T 0755
0615
T 0614
T 1294

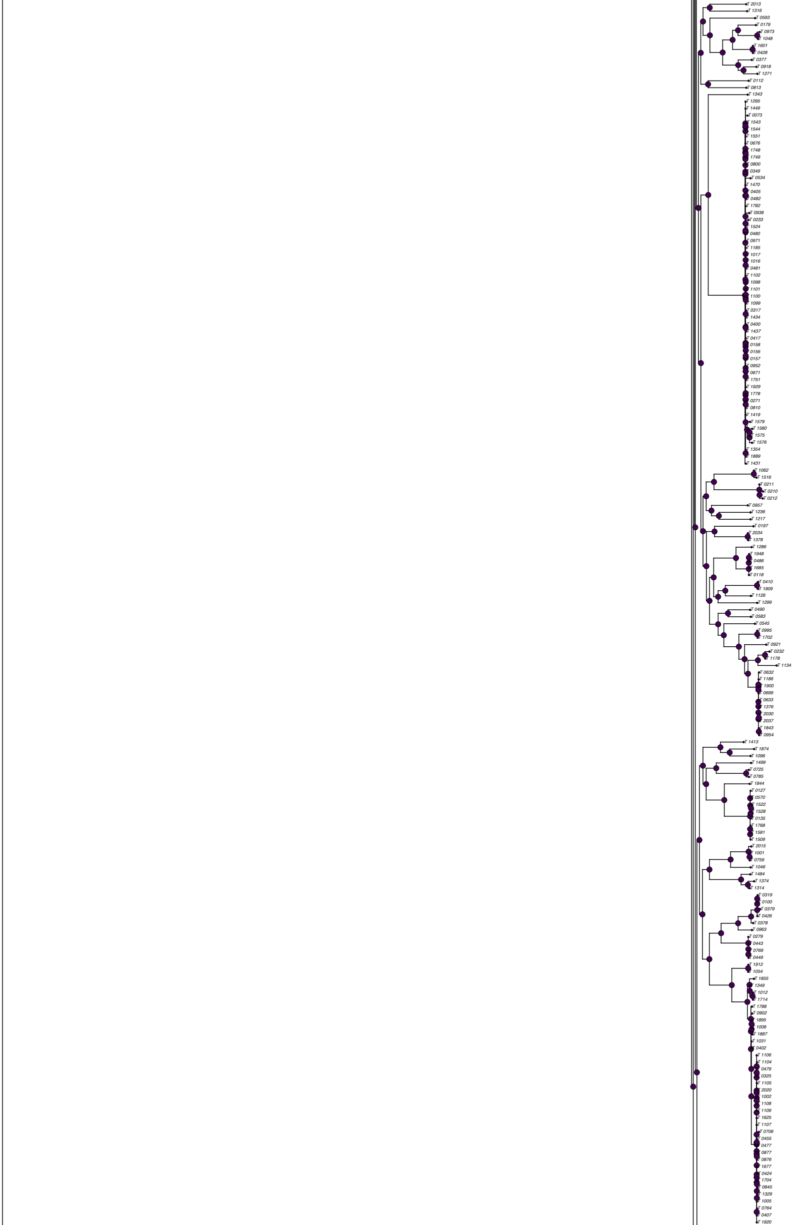
T 2013
T 1316
T 0593
T 0179
T 0973
T 1048
T 1601
T 0428
T 0377
T 0918
T 1271
T 0112
T 0813
T 1343
T 1295
T 1449
T 0073
T 1543
T 1544
T 1551
T 0676
T 1748
T 1749
T 0800
T 0349
T 0534
T 1470
T 0405
T 0482
T 1782
T 0938
T 0233
T 1524
T 0480
T 0971
T 1185
T 1017
T 1016
T 0481
T 1102
T 1098
T 1101
T 1100
T 1099
T 0317
T 1434
T 0400
T 1437
T 0417
T 0158
T 0156
T 0157
T 0952
T 0671
T 1751
T 1929
T 1778
T 0271
T 0810
T 1419
T 1579
T 1580
T 1575
T 1576
T 1354
T 1889
T 1431
T 1062
T 1516
T 0211
T 0210
T 0212
T 0957
T 1236
T 1217
T 0197
T 2034
T 1378
T 1286
T 1948
T 0486
T 1685
T 0118
T 0410
T 1909
T 1126
T 1299
T 0490
T 0583
T 0545
T 0995
T 1702
T 0921
T 0232
T 1178
T 1134
T 0632
T 1186
T 1900
T 0699
T 0633
T 1376
T 2030
T 2037
T 1843
T 0954
T 1413
T 1874
T 1096
T 1499
T 0725
T 0785
T 1844
T 0127
T 0570
T 1522
T 1528
T 0135
T 1768
T 1581
T 1509
T 2015
T 1001
T 0759
T 1046
T 1484
T 1374
T 1314
T 0319
T 0100
T 0379
T 0426
T 0378
T 0963
T 0279
T 0443
T 0769
T 0449
T 1912
T 1054
T 1855
T 1349
T 1012
T 1714
T 1788
T 0902
T 1895
T 1008
T 1887
T 1031
T 0402
T 1106
T 1104
T 0479
T 0325
T 1105
T 2020
T 1002
T 1108
T 1109
T 1625
T 1107
T 0706
T 0455
T 0477
T 0877
T 0876
T 1677
T 0424
T 1704
T 0845
T 1329
T 1005
T 0764
T 0407
T 1920

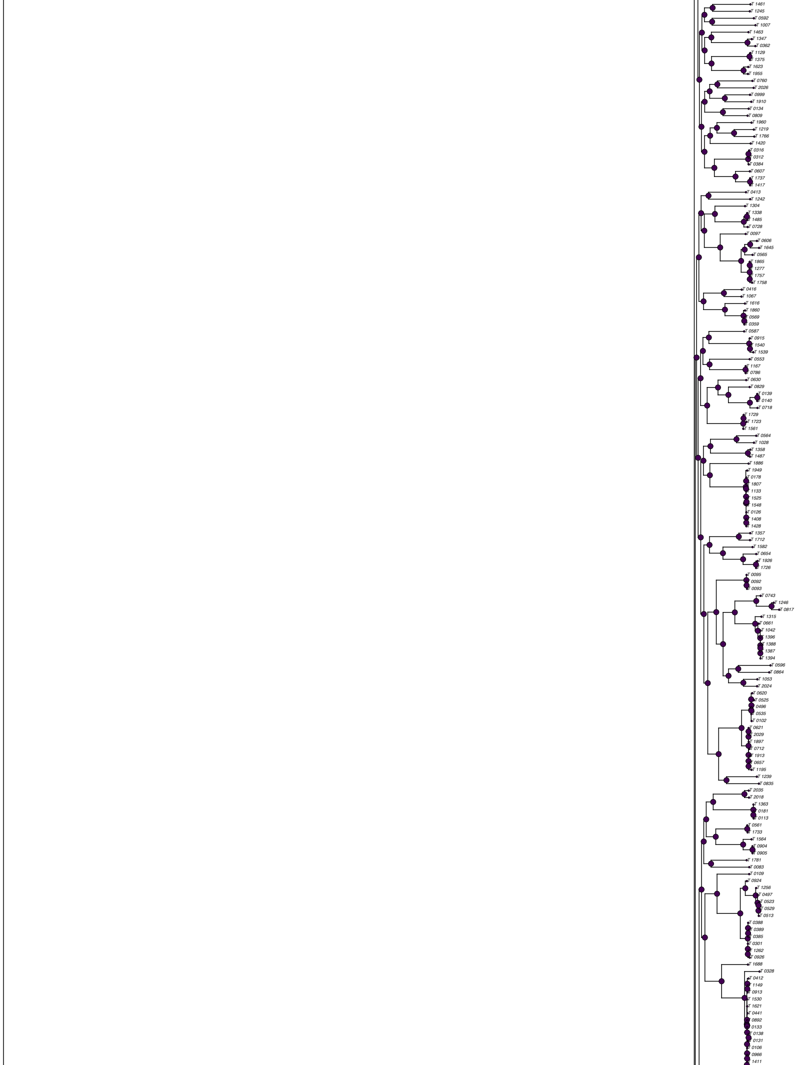

T 1461
T 1245
T 0592
T 1007
T 1463
T 1347
T 0362
T 1129
T 1375
T 1623
T 1955
T 0760
T 2026
T 0999
T 1910
T 0134
T 0809
T 1960
T 1219
T 1766
T 1420
T 0316
T 0312
T 0384
T 0607
T 1737
T 1417
T 0413
T 1242
T 1304
T 1338
T 1485
T 0728
T 0097
T 0606
T 1645
T 0565
T 1865
T 1277
T 1757
T 1758
T 0416
T 1067
T 1616
T 1860
T 0569
T 0359
T 0587
T 0915
T 1540
T 1539
T 0553
T 1167
T 0786
T 0630
T 0829
T 0139
T 0140
T 0718
T 1729
T 1723
T 1561
T 0564
T 1028
T 1358
T 1487
T 1886
T 1949
T 0178
1807
1133
1525
1548
T 0126
1408
T 1428
T 1357
T 1712
T 1582
T 0654
T 1926
T 1726
T 0095
0092
T 0093
T 0743
T 1246
T 0817
T 1315
T 0661
T 1042
1396
T 1388
1387
T 1394
T 0596
T 0864
T 1053
T 2024
T 0620
T 0525
0496
T 0535
T 0102
T 0621
2029
T 1897
T 0712
T 1913
T 0657
T 1195
T 1239
T 0835
T 2035
T 2018
T 1363
0181
T 0113
T 0561
T 1733
T 1564
T 0904
T 0905
T 1781
T 0083
T 0109
T 0924
T 1256
T 0497
T 0523
T 0529
T 0513
T 0388
T 0389
T 0385
T 0301
T 1262
T 0926
T 1688
T 0328
T 0412
1149
T 0913
T 1530
T 1621
T 0441
0892
0133
T 0138
0131
T 0106
0966
1411

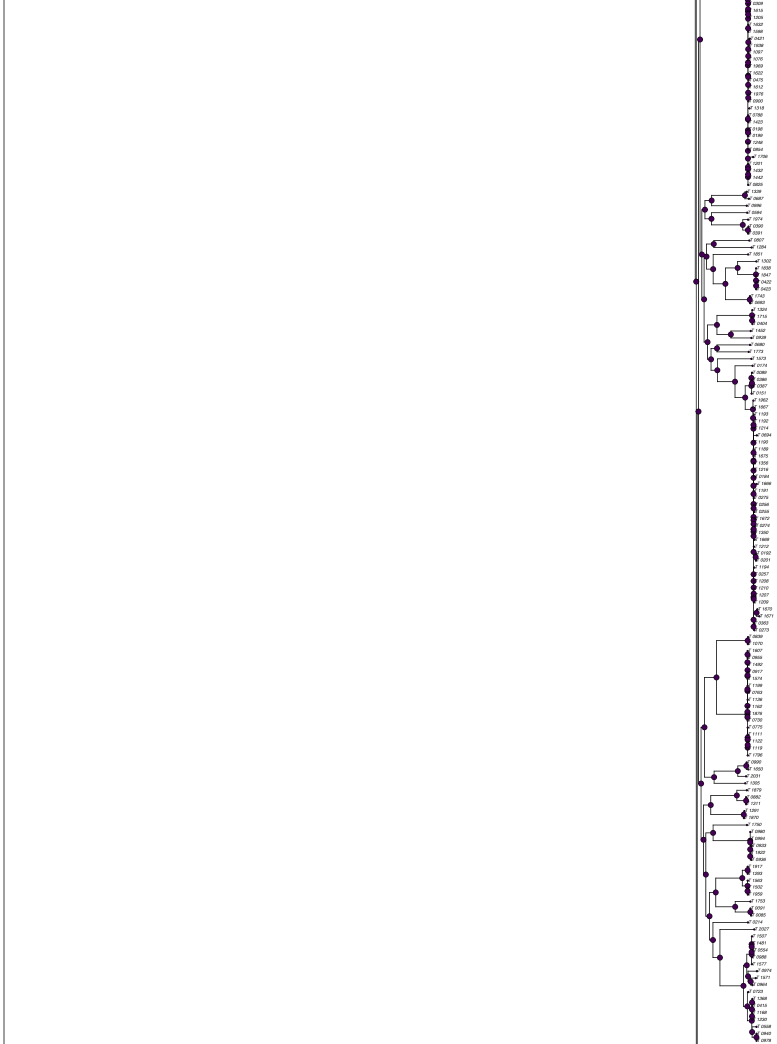
T 0309
T 1615
T 1205
T 1632
T 1598
T 0421
T 1938
T 1097
T 1076
T 1969
T 1622
T 0475
T 1612
T 1976
T 0900
T 1318
T 0788
T 1423
T 0198
T 0199
T 1248
T 0854
T 1706
T 1201
T 1432
T 1442
T 0825
T 1339
T 0687
T 0996
T 0594
T 1974
T 0390
T 0391
T 0807
T 1284
T 1851
T 1302
T 1838
T 1847
T 0422
T 0423
T 1743
T 0693
T 1324
T 1715
T 0404
T 1452
T 0939
T 0680
T 1773
T 1573
T 0174
T 0089
T 0386
T 0387
T 0151
T 1962
T 1667
T 1193
T 1192
T 1214
T 0694
T 1190
T 1189
T 1675
T 1356
T 1216
T 0184
T 1666
T 1191
T 0275
T 0256
T 0255
T 1672
T 0274
T 1350
T 1669
T 1212
T 0192
T 0201
T 1194
T 0257
T 1208
T 1210
T 1207
T 1209
T 1670
T 1671
T 0363
T 0273
T 0839
T 1070
T 1607
T 0955
T 1492
T 0917
T 1574
T 1199
T 0763
T 1136
T 1162
T 1876
T 0730
T 0775
T 1111
T 1122
T 1119
T 1796
T 0990
T 1650
T 2031
T 1305
T 1879
T 0882
T 1311
T 1291
T 1870
T 1750
T 0980
T 0994
T 0933
T 1922
T 0936
T 1917
T 1293
T 1563
T 1502
T 1959
T 1753
T 0091
T 0085
T 0214
T 2027
T 1507
T 1481
T 0554
T 0988
T 1577
T 0974
T 1571
T 0964
T 0723
T 1368
T 0415
T 1168
T 1230
T 0558
T 0940
T 0978

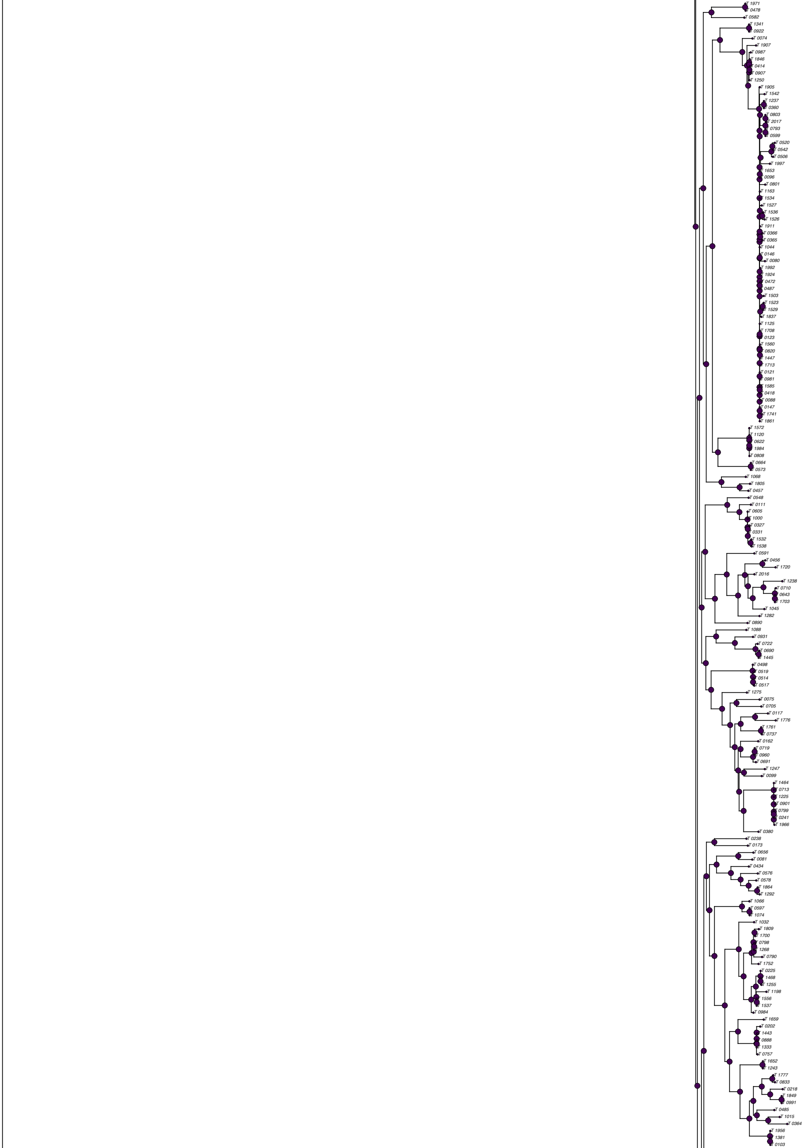
T 1971
T 0478
T 0582
T 1341
T 0922
T 0074
T 1907
T 0987
T 1846
T 0414
T 0907
T 1250
T 1905
T 1542
T 1237
T 0360
T 0803
T 2017
T 0793
T 0599
T 0520
T 0542
T 0506
T 1997
T 1653
T 0096
T 0801
T 1163
T 1534
T 1527
T 1536
T 1526
T 1911
T 0366
T 0365
T 1044
T 0146
T 0080
T 1992
T 1924
T 0472
T 0487
T 1503
T 1523
T 1529
T 1837
T 1125
T 1708
T 0123
T 1560
T 0820
T 1447
T 1713
T 0121
T 0981
T 1585
T 0418
T 0088
T 0147
T 1741
T 1861
T 1572
T 1120
T 0622
T 1984
T 0808
T 0664
T 0573
T 1068
T 1805
T 0457
T 0548
T 0111
T 0605
T 1000
T 0327
T 0331
T 1532
T 1538
T 0591
T 0456
T 1720
T 2016
T 1238
T 0710
T 0643
T 1703
T 1045
T 1282
T 0890
T 1088
T 0931
T 0722
T 0690
T 1445
T 0498
T 0519
T 0514
T 0517
T 1275
T 0075
T 0705
T 0117
T 1776
T 1761
T 0737
T 0162
T 0719
T 0960
T 0691
T 1247
T 0099
T 1464
T 0713
T 1225
T 0901
T 0799
T 0241
T 1966
T 0380
T 0238
T 0173
T 0656
T 0081
T 0434
T 0576
T 0578
T 1864
T 1292
T 1066
T 0597
T 1074
T 1032
T 1809
T 1700
T 0798
T 1268
T 0790
T 1752
T 0225
T 1468
T 1255
T 1198
T 1556
T 1537
T 0984
T 1659
T 0202
T 1443
T 0888
T 1333
T 0757
T 1652
T 1243
T 1777
T 0833
T 0218
T 1849
T 0991
T 0485
T 1015
T 0364
T 1956
T 1381
T 0103

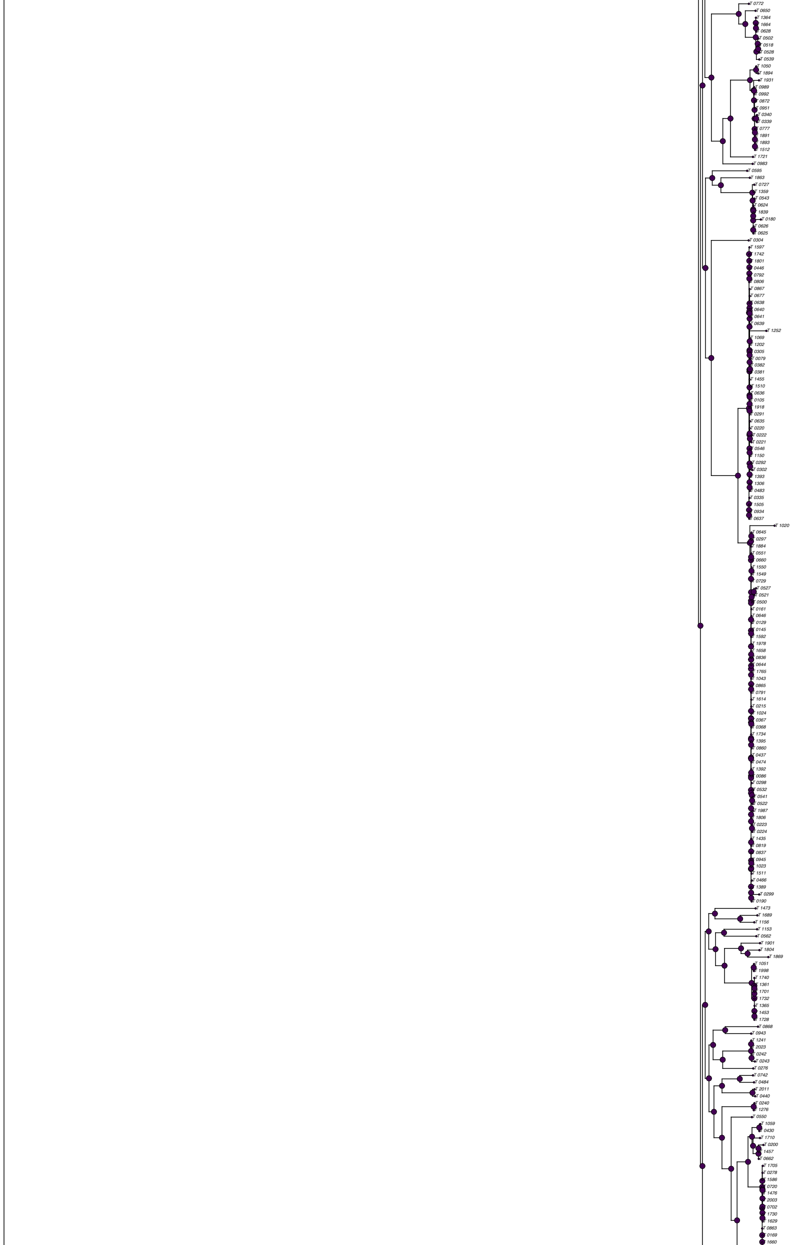
T 0772
T 0650
T 1364
1664
0628
T 0502
0518
T 0528
T 0539
T 1050
T 1894
T 1931
T 0989
0992
0872
T 0951
T 0340
T 0339
0777
1891
1893
T 1512
T 1721
T 0983
T 0595
T 1863
T 0727
T 1359
T 0543
T 0624
1839
T 0180
T 0626
T 0625
T 0304
T 1597
1742
1801
0446
0792
T 0806
T 0867
T 0677
0638
0640
0641
0639
T 1252
T 1069
T 1202
0305
T 0079
0382
0381
T 1455
1510
T 0636
0105
1918
T 0291
T 0635
T 0220
T 0222
T 0221
0546
1150
T 0292
T 0302
1393
1306
T 0483
T 0335
1505
0934
T 0637
T 1020
T 0645
0297
1884
T 0551
0660
T 1550
1549
0729
T 0527
T 0521
0500
T 0161
0646
0129
T 0145
1592
1978
1658
0836
0644
1765
T 1043
0865
T 0791
T 1614
T 0215
1024
0367
T 0368
T 1734
1395
T 0860
0437
T 0474
1392
0086
T 0298
T 0532
T 0541
T 0522
T 1987
1806
T 0223
T 0224
1435
T 0819
T 0837
0945
1023
T 1511
T 0466
1389
T 0299
T 0190
T 1473
T 1689
T 1156
T 1153
T 0562
T 1901
T 1804
T 1869
T 1051
T 1998
T 1740
1361
1701
1732
T 1365
1453
T 1728
T 0868
T 0943
T 1241
2023
0242
T 0243
T 0276
T 0742
T 0484
T 2011
T 0440
T 0240
1276
T 0550
T 1059
0430
T 1710
T 0200
1457
T 0662
T 1705
T 0278
1586
0720
1476
2003
0702
1730
1629
T 0863
0169
1660

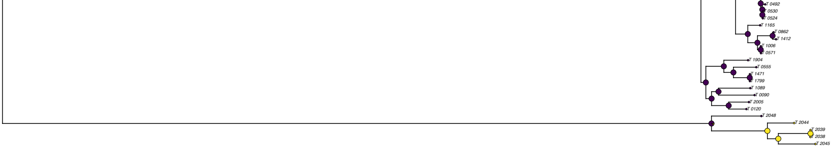
T 0492
T 0530
T 0524
T 1165
T 0862
T 1412
T 1006
T 0571
T 1904
T 0555
T 1471
T 1799
T 1089
T 0090
T 2005
T 0120
T 2048
T 2044
T 2039
T 2038
T 2045

tepBa
X

Effector gene: tepBa

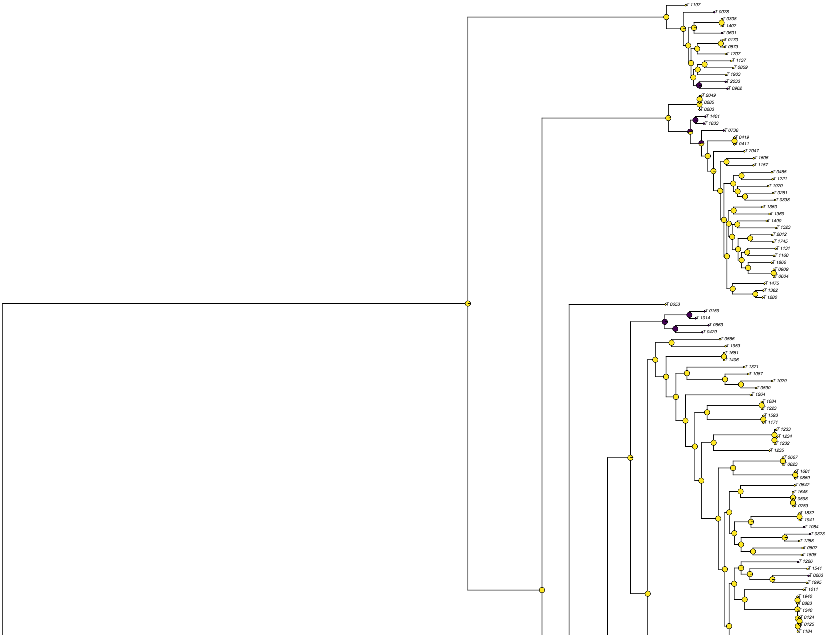

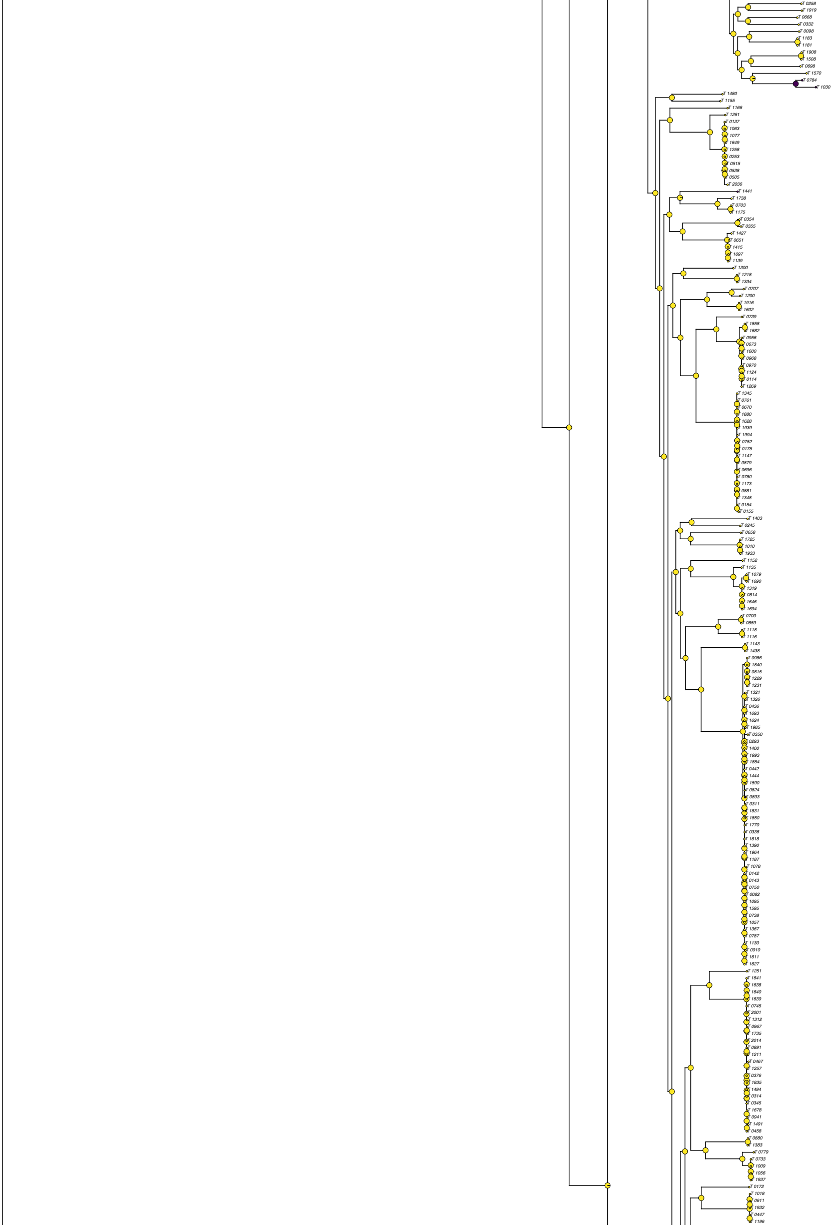

T 0258
T 1919
T 0668
T 0332
T 0098
T 1183
1181
T 1908
1508
T 0698
T 1570
T 0784
T 1030
T 1480
T 1155
T 1166
T 1261
T 0137
1063
1077
1649
1258
0253
0515
0538
0505
T 2036
T 1441
T 1738
T 0703
1175
T 0354
T 0355
T 1427
T 0651
1415
1697
1139
T 1300
T 1218
1334
T 0707
T 1200
T 1916
1602
T 0739
1858
1682
T 0956
0673
1600
0968
T 0970
1124
0114
T 1269
T 1345
T 0761
0670
1880
1628
1939
T 1994
0752
0175
T 1147
0879
T 0696
T 0780
1173
0881
1348
0154
T 0155
T 1403
T 0245
T 0658
T 1725
1010
1933
T 1152
T 1135
T 1079
1690
1319
0814
1646
1694
T 0700
0659
T 1118
1116
T 1143
1438
T 0986
1840
0815
1229
1231
T 1321
1326
T 0436
1693
1624
T 1985
T 0350
0293
1400
1993
1854
T 0442
1444
1590
T 0824
0893
T 0311
1831
1850
T 1770
T 0336
T 1618
1390
1964
1187
T 1078
0142
0143
0750
0082
1095
1595
0738
1057
T 1367
0787
T 1130
T 0910
1611
1627
T 1251
T 1641
1638
1640
1639
T 0745
T 2001
T 1312
T 0967
1735
2014
T 0891
1211
T 0467
1257
0376
1835
1494
0314
0345
T 1678
0941
T 1491
0458
T 0880
1383
T 0779
T 0733
1009
1056
1937
T 0172
T 1018
0611
1932
T 0447
1196

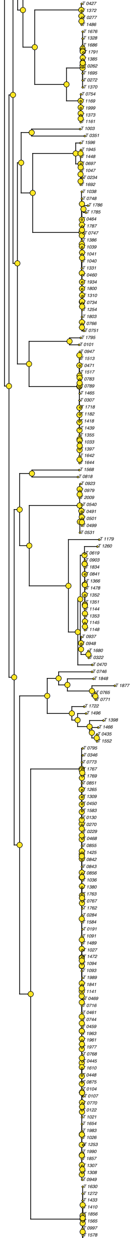
T 0427
T 1372
T 0277
T 1486
T 1676
T 1328
T 1686
T 1791
T 1385
T 0262
T 1695
T 0272
T 1370
T 0754
T 1169
T 1999
T 1373
T 1161
T 1003
T 0351
T 1596
T 1945
T 1448
T 0697
T 1047
T 0234
T 1692
T 1038
T 0748
T 1786
T 1785
T 0464
T 1787
T 0747
T 1386
T 1039
T 1041
T 1040
T 1331
T 0460
T 1934
T 1800
T 1310
T 0734
T 1254
T 1803
T 0766
T 0751
T 1795
T 0101
T 0947
T 1513
T 0471
T 1517
T 0783
T 0789
T 1465
T 0307
T 1718
T 1182
T 1418
T 1439
T 1355
T 1033
T 1397
T 1642
T 1644
T 1568
T 0818
T 0923
T 0979
T 2009
T 0540
T 0491
T 0501
T 0499
T 0531
T 1179
T 1260
T 0619
T 0903
T 1834
T 0841
T 1366
T 1478
T 1352
T 1351
T 1144
T 1353
T 1145
T 1148
T 0937
T 0948
T 1680
T 0322
T 0470
T 0746
T 1848
T 1877
T 0765
T 0771
T 1722
T 1496
T 1398
T 1466
T 0435
T 1552
T 0795
T 0346
T 0773
T 1767
T 1769
T 0851
T 1265
T 1309
T 0450
T 1583
T 0130
T 0270
T 0229
T 0468
T 0855
T 1425
T 0842
T 0843
T 0856
T 1036
T 1380
T 1763
T 0767
T 1762
T 0284
T 1584
T 0191
T 1091
T 1489
T 1027
T 1472
T 1094
T 1093
T 1989
T 1841
T 1141
T 0469
T 0716
T 0461
T 0744
T 0459
T 1963
T 1961
T 1977
T 0768
T 0445
T 1610
T 0448
T 0875
T 0104
T 0107
T 0770
T 0122
T 1021
T 1654
T 1983
T 1026
T 1253
T 1990
T 1857
T 1307
T 1308
T 0949
T 1630
T 1272
T 1433
T 1410
T 1856
T 1565
T 0997
T 1578

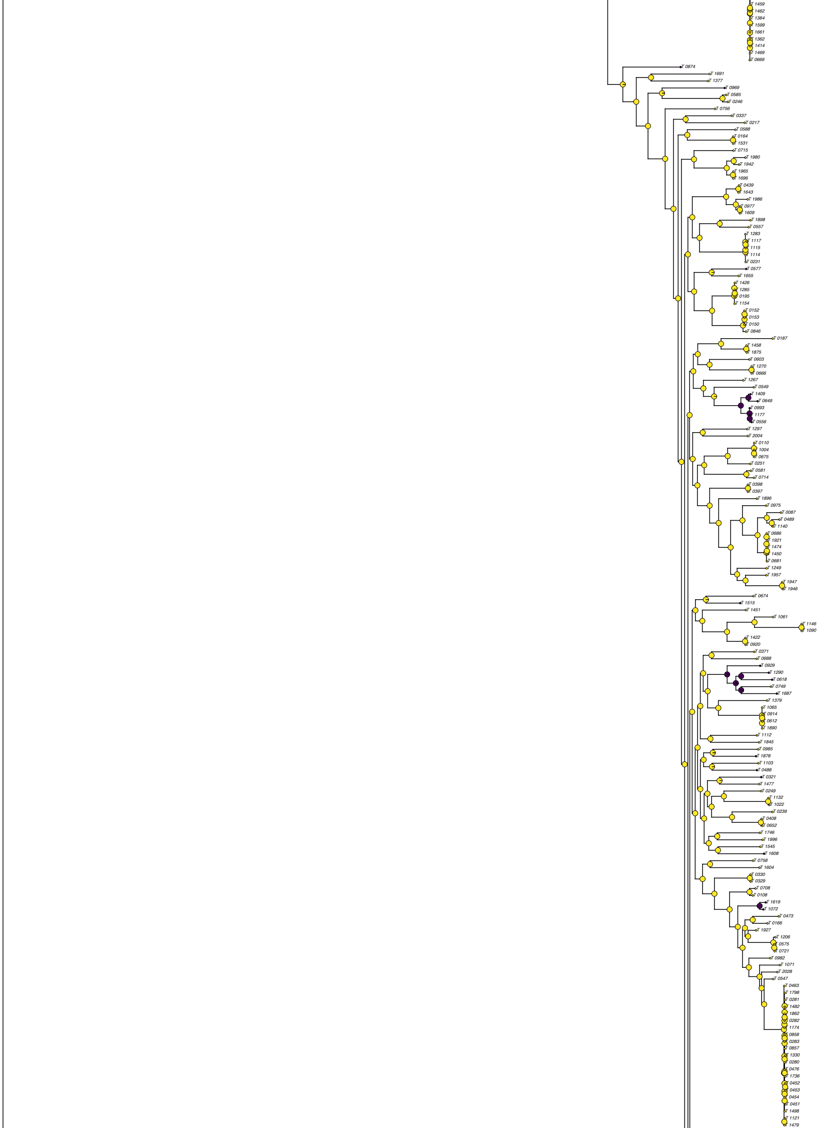
T 1459
T 1462
T 1384
T 1599
T 1661
T 1362
T 1414
T 1469
T 0669
T 0874
T 1691
T 1377
T 0969
T 0585
T 0246
T 0756
T 0337
T 0217
T 0588
T 0164
T 1531
T 0715
T 1980
T 1942
T 1965
T 1696
T 0439
T 1643
T 1986
T 0977
T 1609
T 1898
T 0557
T 1283
T 1117
T 1115
T 1114
T 0231
T 0577
T 1655
T 1426
T 1285
T 0195
T 1154
T 0152
T 0153
T 0150
T 0846
T 0187
T 1458
T 1875
T 0603
T 1270
T 0666
T 1267
T 0549
T 1409
T 0649
T 0993
T 1177
T 0556
T 1297
T 2004
T 0110
T 1004
T 0675
T 0251
T 0581
T 0714
T 0398
T 0397
T 1896
T 0975
T 0087
T 0489
T 1140
T 0686
T 1921
T 1474
T 1450
T 0681
T 1249
T 1957
T 1947
T 1946
T 0674
T 1515
T 1451
T 1061
T 1146
T 1090
T 1422
T 0920
T 0371
T 0688
T 0929
T 1290
T 0618
T 0749
T 1687
T 1379
T 1065
T 0914
T 0612
T 1890
T 1112
T 1845
T 0985
T 1878
T 1103
T 0488
T 0321
T 1477
T 0249
T 1132
T 1022
T 0239
T 0408
T 0652
T 1746
T 1996
T 1545
T 1608
T 0758
T 1604
T 0330
T 0329
T 0708
T 0108
T 1619
T 1072
T 0473
T 0166
T 1927
T 1206
T 0575
T 0721
T 0982
T 1071
T 2028
T 0547
T 0463
T 1798
T 0281
T 1482
T 1862
T 0282
T 1174
T 0858
T 0283
T 0857
T 1330
T 0280
T 0476
T 1736
T 0452
T 0453
T 0454
T 0451
T 1498
T 1121
T 1479

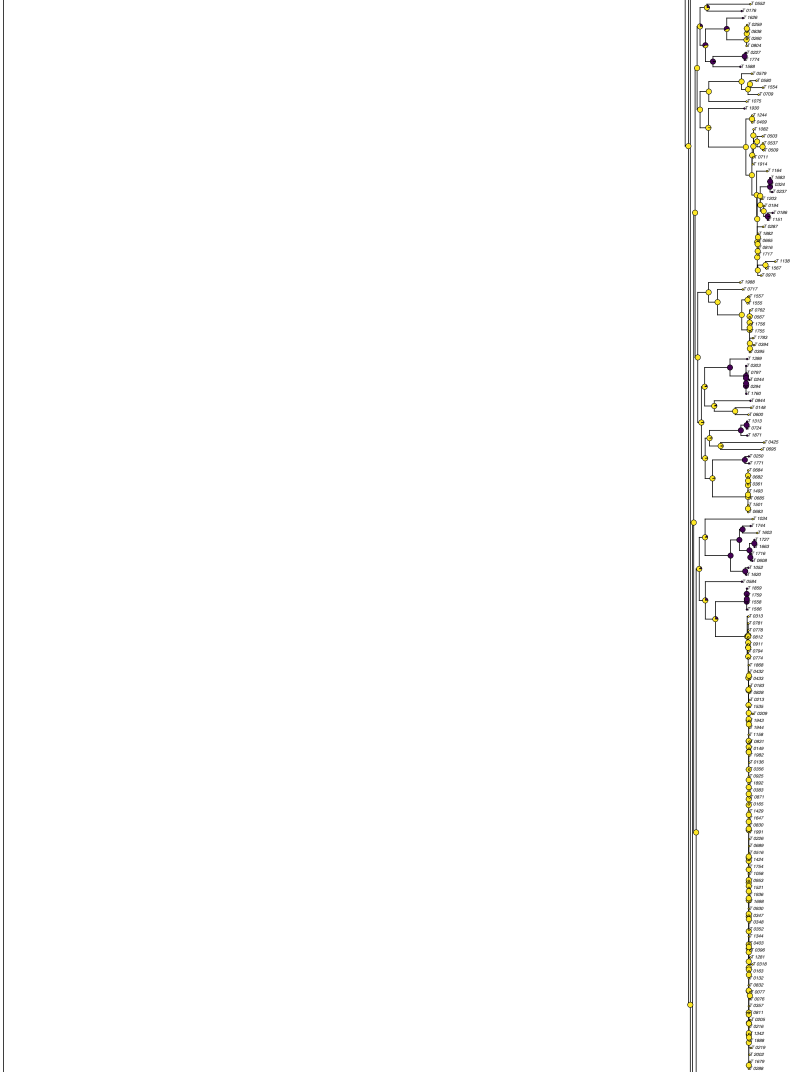
T 0552
T 0176
T 1626
T 0259
T 0838
T 0260
T 0804
T 0227
T 1774
T 1588
T 0579
T 0580
T 1554
T 0709
T 1075
T 1930
T 1244
T 0409
T 1082
T 0503
T 0537
T 0509
T 0711
T 1914
T 1164
T 1683
T 0324
T 0237
T 1203
T 0194
T 0186
T 1151
T 0287
T 1882
T 0665
T 0816
T 1717
T 1138
T 1567
T 0976
T 1988
T 0717
T 1557
T 1555
T 0762
T 0567
T 1756
T 1755
T 1783
T 0394
T 0395
T 1399
T 0303
T 0797
T 0244
T 0294
T 1760
T 0844
T 0148
T 0600
T 1313
T 0724
T 1871
T 0425
T 0695
T 0250
T 1771
T 0684
T 0682
T 0361
T 1493
T 0685
T 1501
T 0683
T 1034
T 1744
T 1603
T 1727
T 1663
T 1716
T 0608
T 1052
T 1620
T 0584
T 1859
T 1759
T 1558
T 1566
T 0313
T 0781
T 0778
T 0812
T 0911
T 0794
T 0774
T 1868
T 0432
T 0433
T 0183
T 0828
T 0213
T 1535
T 0209
T 1943
T 1944
T 1158
T 0831
T 0149
T 1982
T 0136
T 0356
T 0925
T 1892
T 0383
T 0871
T 0165
T 1429
T 1647
T 0830
T 1991
T 0226
T 0689
T 0516
T 1424
T 1754
T 1058
T 0953
T 1521
T 1936
T 1698
T 0930
T 0347
T 0348
T 0352
T 1344
T 0403
T 0396
T 1281
T 0318
T 0163
T 0132
T 0832
T 0077
T 0076
T 0357
T 0811
T 0205
T 0216
T 1342
T 1888
T 0219
T 2002
T 1679
T 0288

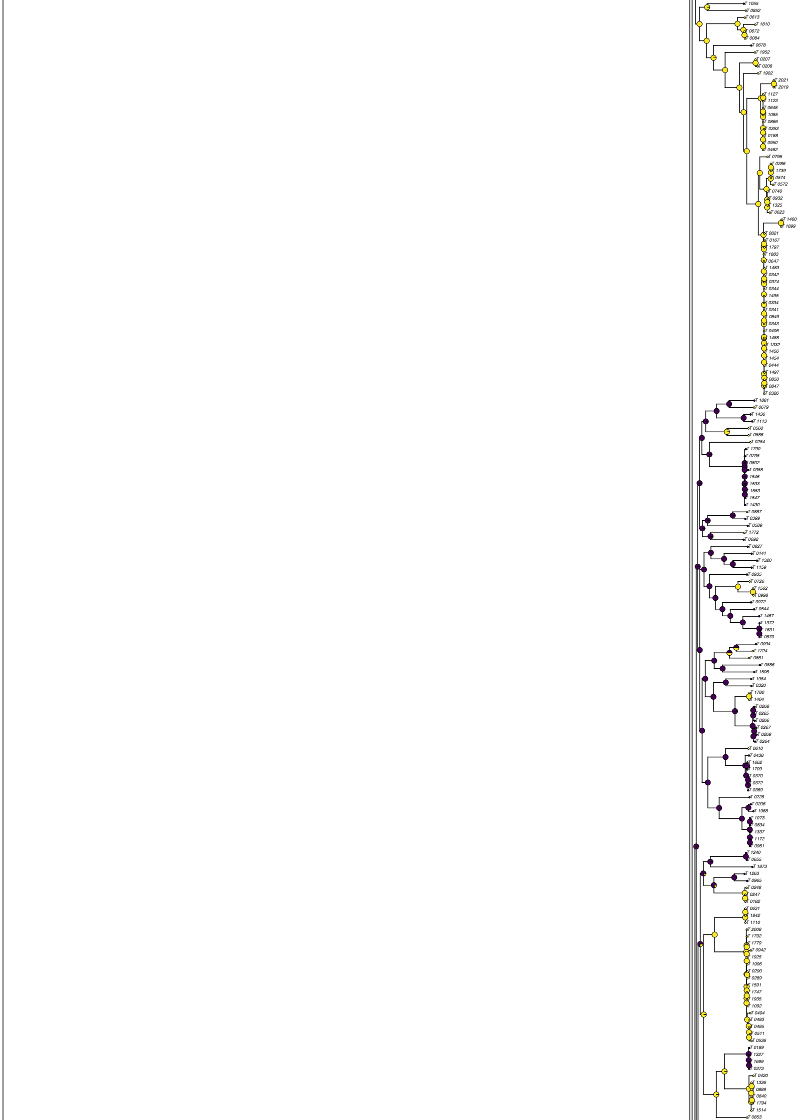
T 1055
T 0852
T 0613
T 1810
T 0672
T 0084
T 0678
T 1952
T 0207
T 0208
T 1902
T 2021
T 2019
T 1127
T 1123
T 0648
T 1085
T 0866
T 0353
T 0188
T 0950
T 0462
T 0796
T 0286
T 1739
T 0574
T 0572
T 0740
T 0932
T 1325
T 0623
T 1460
T 1899
T 0821
T 0167
T 1797
T 1883
T 0647
T 1483
T 0342
T 0374
T 0344
T 1495
T 0334
T 0341
T 0849
T 0343
T 0406
T 1488
T 1332
T 1456
T 1454
T 0444
T 1497
T 0850
T 0847
T 0326
T 1881
T 0679
T 1436
T 1113
T 0560
T 0586
T 0254
T 1790
T 0235
T 0802
T 0358
T 1546
T 1533
T 1553
T 1547
T 1430
T 0887
T 0399
T 0589
T 1772
T 0692
T 0827
T 0141
T 1320
T 1159
T 0935
T 0726
T 1562
T 0998
T 0972
T 0544
T 1467
T 1972
T 1631
T 0870
T 0094
T 1224
T 0861
T 0886
T 1506
T 1954
T 0320
T 1780
T 1404
T 0268
T 0265
T 0266
T 0267
T 0269
T 0264
T 0610
T 0438
T 1662
T 1709
T 0370
T 0372
T 0369
T 0228
T 0206
T 1968
T 1073
T 0834
T 1337
T 1172
T 0961
T 1240
T 0655
T 1873
T 1263
T 0965
T 0248
T 0247
T 0182
T 0631
T 1842
T 1110
T 2008
T 1792
T 1779
T 0942
T 1925
T 1906
T 0290
T 0289
T 1591
T 1747
T 1935
T 1092
T 0494
T 0493
T 0495
T 0511
T 0536
T 0189
T 1327
T 1699
T 0373
T 0420
T 1336
T 0889
T 0840
T 1794
T 1514
T 0853

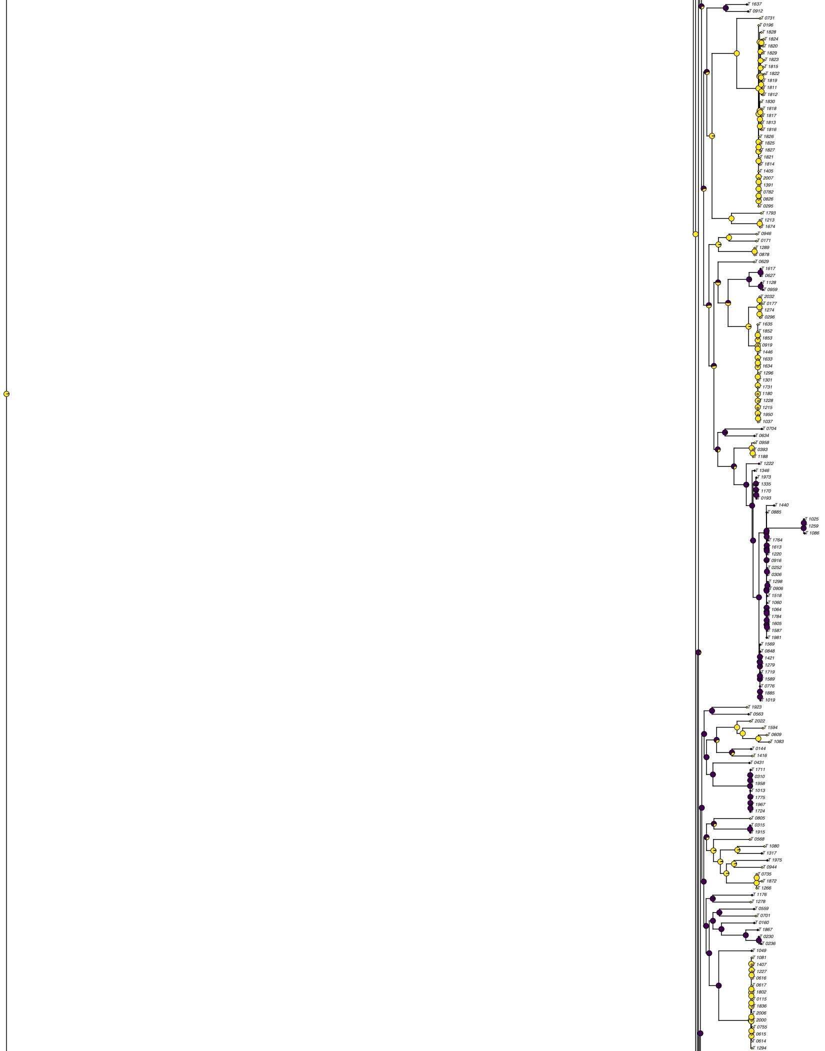
T 1637
T 0912
T 0731
T 0196
T 1828
T 1824
T 1820
T 1829
T 1823
T 1815
T 1822
T 1819
T 1811
T 1812
T 1830
T 1818
T 1817
T 1813
T 1816
T 1826
T 1825
T 1827
T 1821
T 1814
T 1405
2007
1391
0782
0826
T 0295
T 1793
T 1213
T 1674
T 0946
T 0171
T 1289
T 0878
T 0629
T 1617
T 0627
T 1128
T 0959
T 2032
T 0177
T 1274
T 0296
T 1635
T 1852
1853
0919
1446
1633
1634
T 1296
1301
1731
1180
1228
1215
1950
T 1037
T 0704
T 0634
T 0958
0393
T 1188
T 1222
T 1346
T 1973
1335
1170
T 0193
T 1440
T 0885
T 1025
1259
T 1086
T 1764
1613
T 1220
0916
T 0252
T 0306
T 1298
T 0906
T 1518
T 1060
1064
T 1784
1605
T 1587
T 1981
T 1569
T 0848
1421
1279
T 1719
1589
T 0776
1885
T 1019
T 1923
T 0563
T 2022
T 1594
T 0609
T 1083
T 0144
T 1416
T 0431
T 1711
0310
1958
T 1013
1775
1967
T 1724
T 0805
T 0315
T 1915
T 0568
T 1080
T 1317
T 1975
T 0944
T 0735
T 1872
T 1266
T 1176
T 1278
T 0559
T 0701
T 0160
T 1867
T 0230
T 0236
T 1049
T 1081
1407
1227
0616
T 0617
1802
T 0115
1836
T 2006
2000
T 0755
0615
T 0614
T 1294

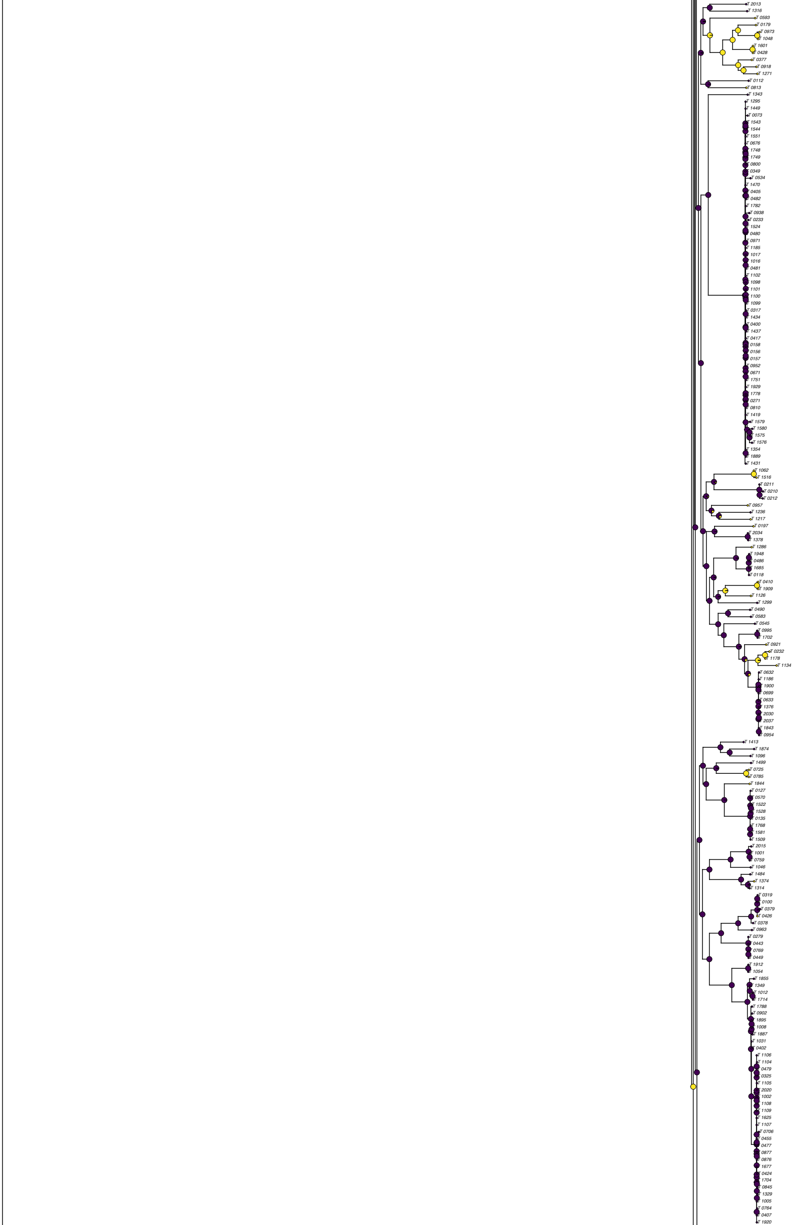

T 2013
T 1316
T 0593
T 0179
T 0973
T 1048
T 1601
T 0428
T 0377
T 0918
T 1271
T 0112
T 0813
T 1343
T 1295
T 1449
T 0073
1543
1544
T 1551
T 0676
1748
1749
0800
0349
T 0534
T 1470
0405
0482
T 1782
T 0938
T 0233
1524
0480
0971
T 1185
1017
1016
0481
T 1102
1098
1101
1100
T 1099
T 0317
1434
T 0400
T 1437
T 0417
0158
0156
0157
T 0952
0671
T 1751
T 1929
1778
0271
T 0810
T 1419
T 1579
T 1580
1575
T 1576
T 1354
T 1889
T 1431
T 1062
T 1516
T 0211
T 0210
T 0212
T 0957
T 1236
T 1217
T 0197
T 2034
T 1378
T 1286
T 1948
0486
1685
T 0118
T 0410
T 1909
T 1126
T 1299
T 0490
T 0583
T 0545
T 0995
T 1702
T 0921
T 0232
T 1178
T 1134
T 0632
T 1186
T 1900
T 0699
T 0633
T 1376
2030
2037
T 1843
T 0954
T 1413
T 1874
T 1096
T 1499
T 0725
T 0785
T 1844
T 0127
0570
T 1522
1528
T 0135
T 1768
1581
T 1509
T 2015
T 1001
T 0759
T 1046
T 1484
T 1374
T 1314
T 0319
0100
T 0379
T 0426
T 0378
T 0963
T 0279
0443
0769
T 0449
T 1912
T 1054
T 1855
1349
T 1012
T 1714
T 1788
T 0902
1895
1008
T 1887
T 1031
0402
T 1106
T 1104
0479
0325
T 1105
2020
1002
1108
T 1109
T 1625
T 1107
T 0706
T 0455
0477
0877
T 0876
1677
T 0424
1704
T 0845
T 1329
T 1005
T 0764
T 0407
T 1920

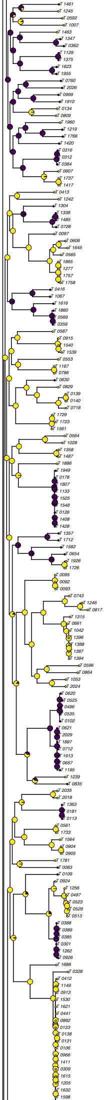
T 1461
T 1245
T 0592
T 1007
T 1463
T 1347
T 0362
T 1129
T 1375
T 1623
T 1955
T 0760
T 2026
T 0999
T 1910
T 0134
T 0809
T 1960
T 1219
T 1766
T 1420
T 0316
T 0312
T 0384
T 0607
T 1737
T 1417
T 0413
T 1242
T 1304
T 1338
T 1485
T 0728
T 0097
T 0606
T 1645
T 0565
T 1865
T 1277
T 1757
T 1758
T 0416
T 1067
T 1616
T 1860
T 0569
T 0359
T 0587
T 0915
T 1540
T 1539
T 0553
T 1167
T 0786
T 0630
T 0829
T 0139
T 0140
T 0718
T 1729
T 1723
T 1561
T 0564
T 1028
T 1358
T 1487
T 1886
T 1949
T 0178
T 1807
T 1133
T 1525
T 1548
T 0126
T 1408
T 1428
T 1357
T 1712
T 1582
T 0654
T 1926
T 1726
T 0095
T 0092
T 0093
T 0743
T 1246
T 0817
T 1315
T 0661
T 1042
T 1396
T 1388
T 1387
T 1394
T 0596
T 0864
T 1053
T 2024
T 0620
T 0525
T 0496
T 0535
T 0102
T 0621
T 2029
T 1897
T 0712
T 1913
T 0657
T 1195
T 1239
T 0835
T 2035
T 2018
T 1363
T 0181
T 0113
T 0561
T 1733
T 1564
T 0904
T 0905
T 1781
T 0083
T 0109
T 0924
T 1256
T 0497
T 0523
T 0529
T 0513
T 0388
T 0389
T 0385
T 0301
T 1262
T 0926
T 1688
T 0328
T 0412
T 1149
T 0913
T 1530
T 1621
T 0441
T 0892
T 0133
T 0138
T 0131
T 0106
T 0966
T 1411
T 0309
T 1615
T 1205
T 1632
T 1598

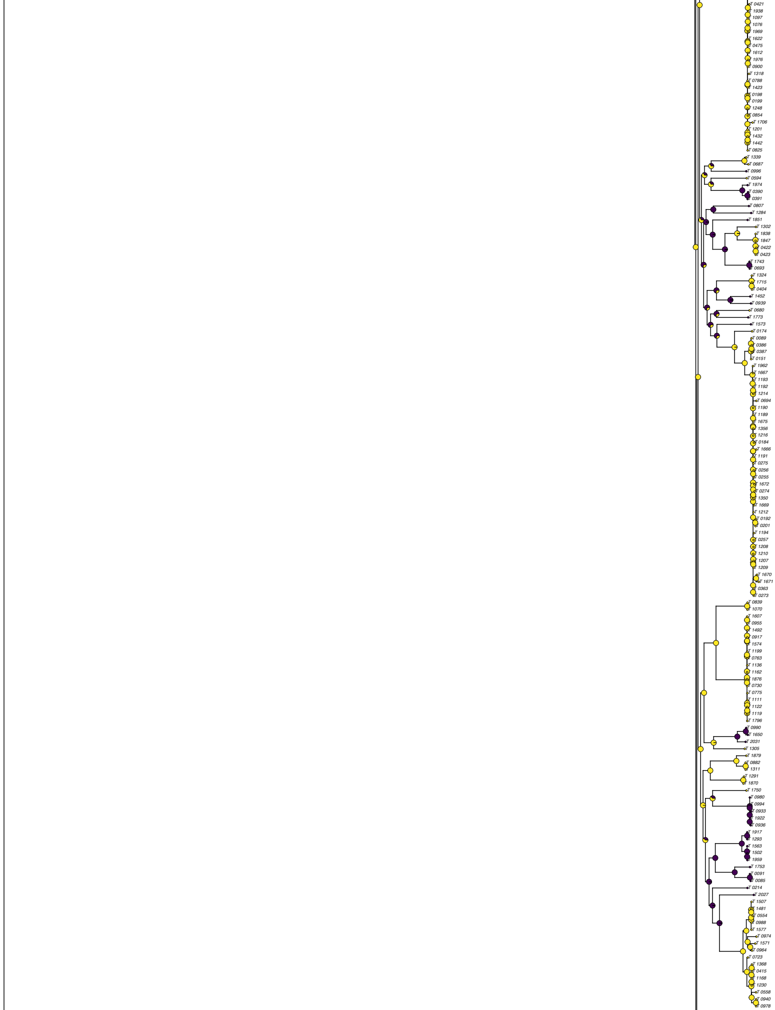
T 0421
T 1938
T 1097
T 1076
T 1969
T 1622
T 0475
T 1612
T 1976
T 0900
T 1318
T 0788
T 1423
T 0198
T 0199
T 1248
T 0854
T 1706
T 1201
T 1432
T 1442
T 0825
T 1339
T 0687
T 0996
T 0594
T 1974
T 0390
T 0391
T 0807
T 1284
T 1851
T 1302
T 1838
T 1847
T 0422
T 0423
T 1743
T 0693
T 1324
T 1715
T 0404
T 1452
T 0939
T 0680
T 1773
T 1573
T 0174
T 0089
T 0386
T 0387
T 0151
T 1962
T 1667
T 1193
T 1192
T 1214
T 0694
T 1190
T 1189
T 1675
T 1356
T 1216
T 0184
T 1666
T 1191
T 0275
T 0256
T 0255
T 1672
T 0274
T 1350
T 1669
T 1212
T 0192
T 0201
T 1194
T 0257
T 1208
T 1210
T 1207
T 1209
T 1670
T 1671
T 0363
T 0273
T 0839
T 1070
T 1607
T 0955
T 1492
T 0917
T 1574
T 1199
T 0763
T 1136
T 1162
T 1876
T 0730
T 0775
T 1111
T 1122
T 1119
T 1796
T 0990
T 1650
T 2031
T 1305
T 1879
T 0882
T 1311
T 1291
T 1870
T 1750
T 0980
T 0994
T 0933
T 1922
T 0936
T 1917
T 1293
T 1563
T 1502
T 1959
T 1753
T 0091
T 0085
T 0214
T 2027
T 1507
T 1481
T 0554
T 0988
T 1577
T 0974
T 1571
T 0964
T 0723
T 1368
T 0415
T 1168
T 1230
T 0558
T 0940
T 0978

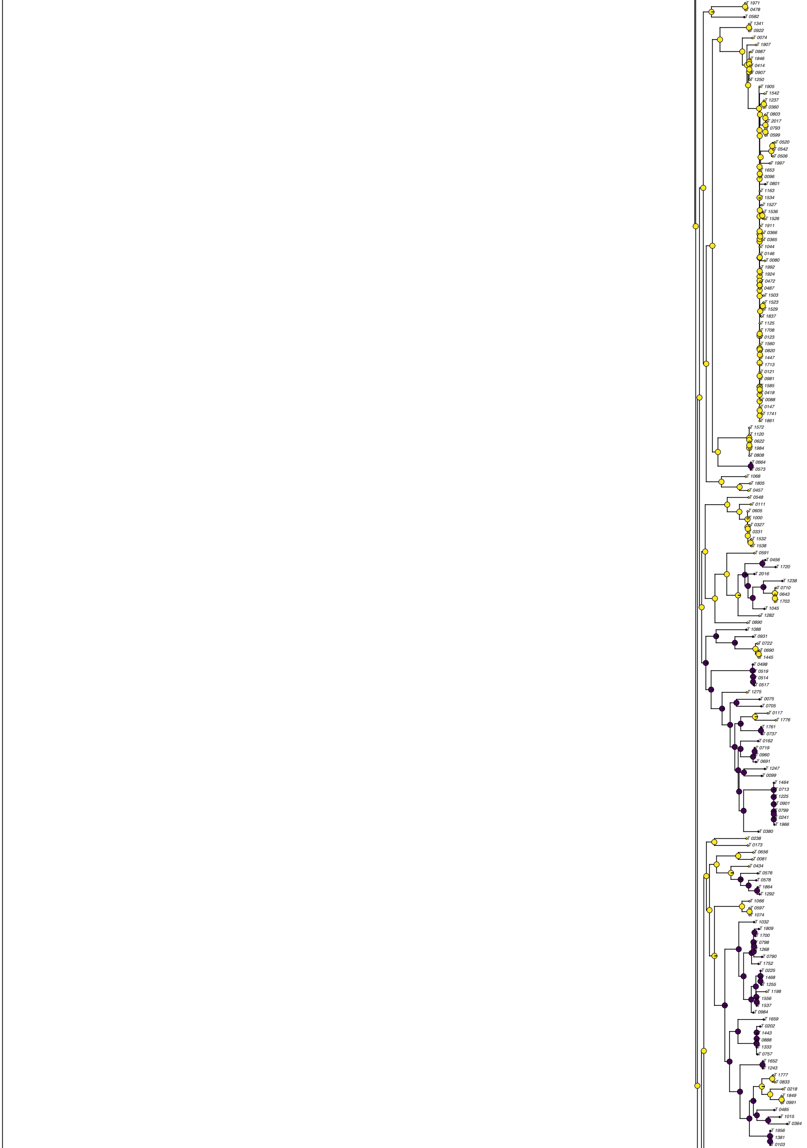
T 1971
T 0478
T 0582
T 1341
T 0922
T 0074
T 1907
T 0987
T 1846
T 0414
T 0907
T 1250
T 1905
T 1542
T 1237
T 0360
T 0803
T 2017
T 0793
T 0599
T 0520
T 0542
T 0506
T 1997
T 1653
T 0096
T 0801
T 1163
T 1534
T 1527
T 1536
T 1526
T 1911
T 0366
T 0365
T 1044
T 0146
T 0080
T 1992
T 1924
T 0472
T 0487
T 1503
T 1523
T 1529
T 1837
T 1125
T 1708
T 0123
T 1560
T 0820
T 1447
T 1713
T 0121
T 0981
T 1585
T 0418
T 0088
T 0147
T 1741
T 1861
T 1572
T 1120
T 0622
T 1984
T 0808
T 0664
T 0573
T 1068
T 1805
T 0457
T 0548
T 0111
T 0605
T 1000
T 0327
T 0331
T 1532
T 1538
T 0591
T 0456
T 1720
T 2016
T 1238
T 0710
T 0643
T 1703
T 1045
T 1282
T 0890
T 1088
T 0931
T 0722
T 0690
T 1445
T 0498
T 0519
T 0514
T 0517
T 1275
T 0075
T 0705
T 0117
T 1776
T 1761
T 0737
T 0162
T 0719
T 0960
T 0691
T 1247
T 0099
T 1464
T 0713
T 1225
T 0901
T 0799
T 0241
T 1966
T 0380
T 0238
T 0173
T 0656
T 0081
T 0434
T 0576
T 0578
T 1864
T 1292
T 1066
T 0597
T 1074
T 1032
T 1809
T 1700
T 0798
T 1268
T 0790
T 1752
T 0225
T 1468
T 1255
T 1198
T 1556
T 1537
T 0984
T 1659
T 0202
T 1443
T 0888
T 1333
T 0757
T 1652
T 1243
T 1777
T 0833
T 0218
T 1849
T 0991
T 0485
T 1015
T 0364
T 1956
T 1381
T 0103

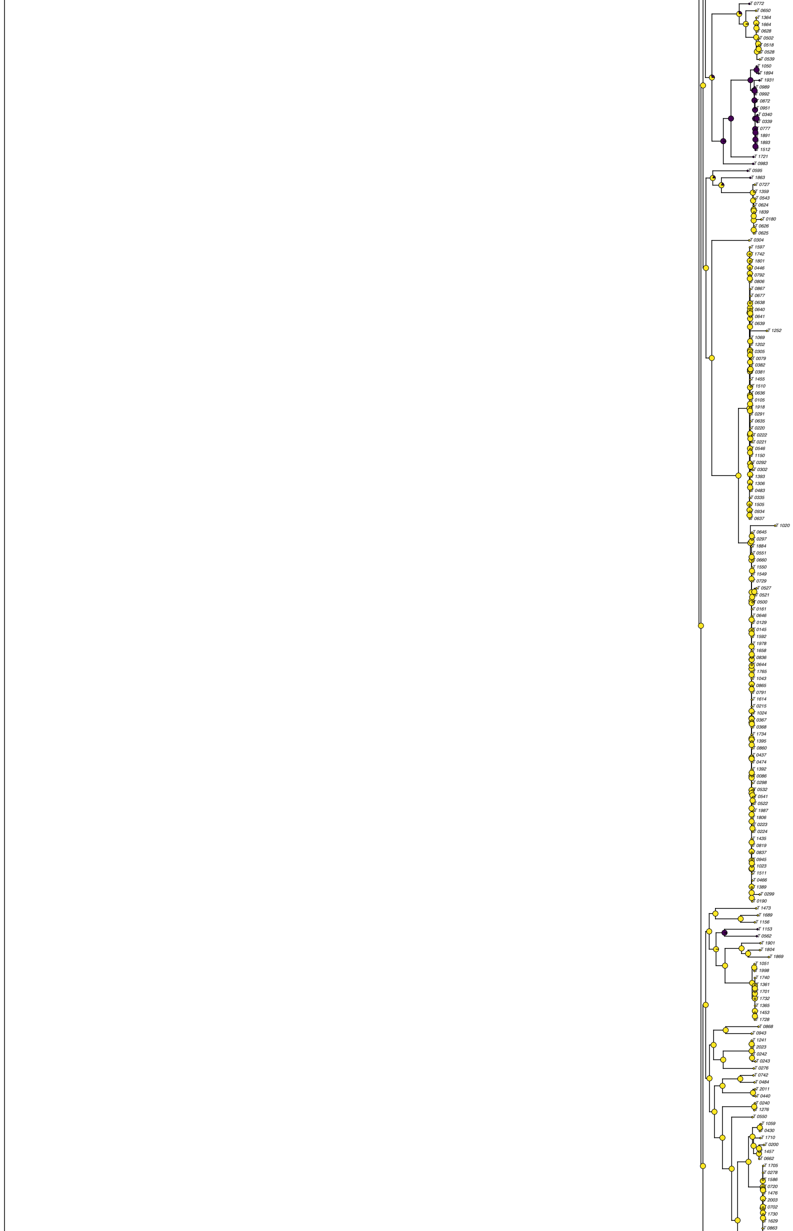
T 0772
T 0650
T 1364
T 1664
T 0628
T 0502
T 0518
T 0528
T 0539
T 1050
T 1894
T 1931
T 0989
T 0992
T 0872
T 0951
T 0340
T 0339
T 0777
T 1891
T 1893
T 1512
T 1721
T 0983
T 0595
T 1863
T 0727
T 1359
T 0543
T 0624
T 1839
T 0180
T 0626
T 0625
T 0304
T 1597
T 1742
T 1801
T 0446
T 0792
T 0806
T 0867
T 0677
T 0638
T 0640
T 0641
T 0639
T 1252
T 1069
T 1202
T 0305
T 0079
T 0382
T 0381
T 1455
T 1510
T 0636
T 0105
T 1918
T 0291
T 0635
T 0220
T 0222
T 0221
T 0546
T 1150
T 0292
T 0302
T 1393
T 1306
T 0483
T 0335
T 1505
T 0934
T 0637
T 1020
T 0645
T 0297
T 1884
T 0551
T 0660
T 1550
T 1549
T 0729
T 0527
T 0521
T 0500
T 0161
T 0646
T 0129
T 0145
T 1592
T 1978
T 1658
T 0836
T 0644
T 1765
T 1043
T 0865
T 0791
T 1614
T 0215
T 1024
T 0367
T 0368
T 1734
T 1395
T 0860
T 0437
T 0474
T 1392
T 0086
T 0298
T 0532
T 0541
T 0522
T 1987
T 1806
T 0223
T 0224
T 1435
T 0819
T 0837
T 0945
T 1023
T 1511
T 0466
T 1389
T 0299
T 0190
T 1473
T 1689
T 1156
T 1153
T 0562
T 1901
T 1804
T 1869
T 1051
T 1998
T 1740
T 1361
T 1701
T 1732
T 1365
T 1453
T 1728
T 0868
T 0943
T 1241
T 2023
T 0242
T 0243
T 0276
T 0742
T 0484
T 2011
T 0440
T 0240
T 1276
T 0550
T 1059
T 0430
T 1710
T 0200
T 1457
T 0662
T 1705
T 0278
T 1586
T 0720
T 1476
T 2003
T 0702
T 1730
T 1629
T 0863

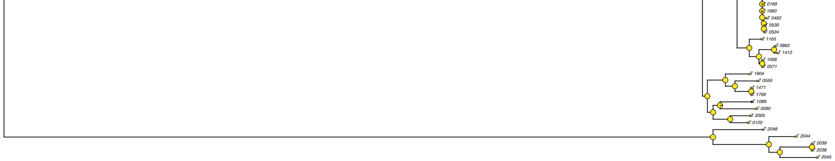
T 0169
T 1660
T 0492
T 0530
T 0524
T 1165
T 0862
T 1412
T 1006
T 0571
T 1904
T 0555
T 1471
T 1799
T 1089
T 0090
T 2005
T 0120
T 2048
T 2044
T 2039
T 2038
T 2045

Effector gene: tepBb

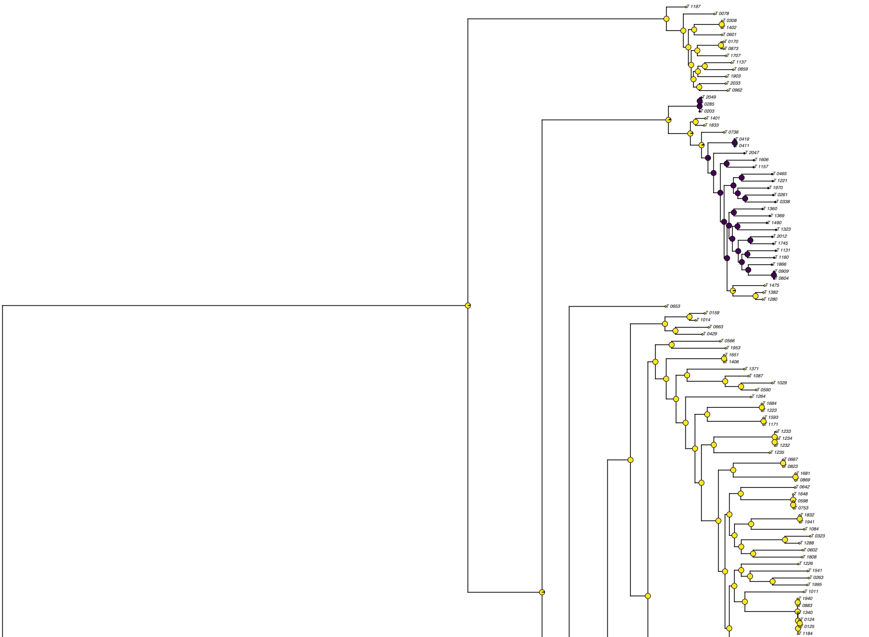

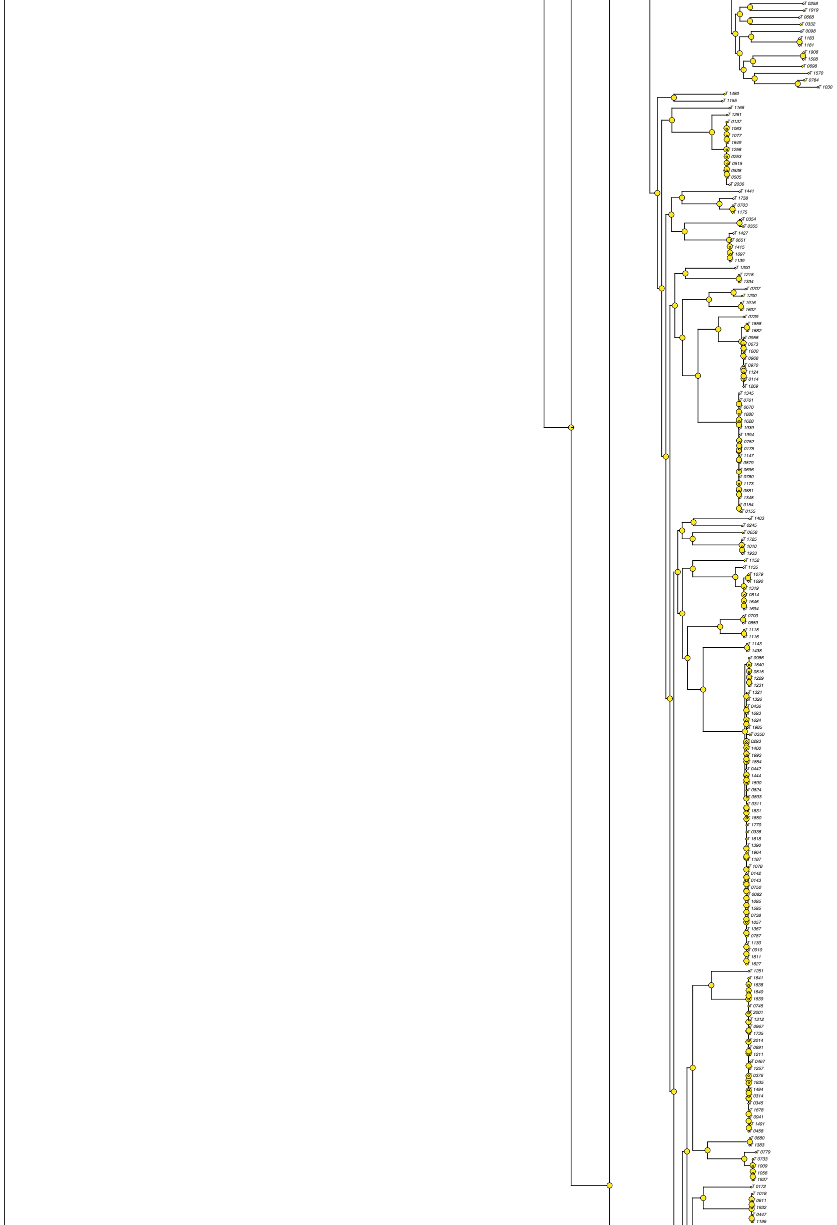

T 0258
T 1919
T 0668
T 0332
T 0098
T 1183
1181
T 1908
1508
T 0698
T 1570
T 0784
T 1030
T 1480
T 1155
T 1166
T 1261
T 0137
1063
1077
1649
1258
0253
0515
0538
0505
T 2036
T 1441
T 1738
T 0703
T 1175
T 0354
T 0355
T 1427
T 0651
1415
1697
1139
T 1300
T 1218
1334
T 0707
T 1200
T 1916
1602
T 0739
T 1858
1682
T 0956
0673
1600
0968
T 0970
1124
0114
T 1269
T 1345
0761
0670
1880
1628
1939
T 1994
0752
0175
T 1147
0879
0696
0780
1173
0881
1348
0154
T 0155
T 1403
T 0245
T 0658
T 1725
1010
1933
T 1152
T 1135
T 1079
1690
1319
0814
1646
1694
T 0700
0659
T 1118
1116
T 1143
1438
T 0986
1840
0815
1229
1231
T 1321
1326
0436
1693
1624
T 1985
T 0350
0293
1400
1993
1854
T 0442
1444
1590
T 0824
0893
0311
1831
1850
T 1770
T 0336
T 1618
1390
1964
1187
T 1078
0142
0143
0750
0082
1095
1595
0738
1057
T 1367
0787
T 1130
0910
1611
1627
T 1251
T 1641
1638
1640
1639
T 0745
2001
T 1312
T 0967
1735
2014
T 0891
1211
T 0467
1257
0376
1835
1494
0314
0345
T 1678
0941
T 1491
0458
T 0880
1383
T 0779
T 0733
1009
1056
1937
T 0172
T 1018
0611
1932
T 0447
1196

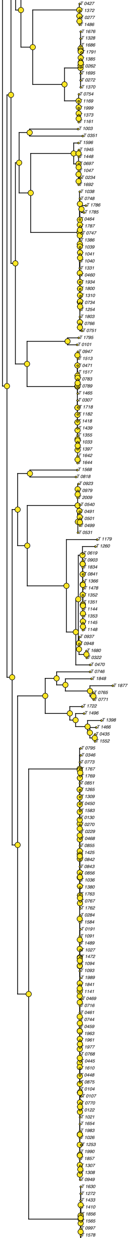
T 0427
T 1372
T 0277
T 1486
T 1676
T 1328
T 1686
T 1791
T 1385
T 0262
T 1695
T 0272
T 1370
T 0754
T 1169
T 1999
T 1373
T 1161
T 1003
T 0351
T 1596
T 1945
T 1448
T 0697
T 1047
T 0234
T 1692
T 1038
T 0748
T 1786
T 1785
T 0464
T 1787
T 0747
T 1386
T 1039
T 1041
T 1040
T 1331
T 0460
T 1934
T 1800
T 1310
T 0734
T 1254
T 1803
T 0766
T 0751
T 1795
T 0101
T 0947
T 1513
T 0471
T 1517
T 0783
T 0789
T 1465
T 0307
T 1718
T 1182
T 1418
T 1439
T 1355
T 1033
T 1397
T 1642
T 1644
T 1568
T 0818
T 0923
T 0979
T 2009
T 0540
T 0491
T 0501
T 0499
T 0531
T 1179
T 1260
T 0619
T 0903
T 1834
T 0841
T 1366
T 1478
T 1352
T 1351
T 1144
T 1353
T 1145
T 1148
T 0937
T 0948
T 1680
T 0322
T 0470
T 0746
T 1848
T 1877
T 0765
T 0771
T 1722
T 1496
T 1398
T 1466
T 0435
T 1552
T 0795
T 0346
T 0773
T 1767
T 1769
T 0851
T 1265
T 1309
T 0450
T 1583
T 0130
T 0270
T 0229
T 0468
T 0855
T 1425
T 0842
T 0843
T 0856
T 1036
T 1380
T 1763
T 0767
T 1762
T 0284
T 1584
T 0191
T 1091
T 1489
T 1027
T 1472
T 1094
T 1093
T 1989
T 1841
T 1141
T 0469
T 0716
T 0461
T 0744
T 0459
T 1963
T 1961
T 1977
T 0768
T 0445
T 1610
T 0448
T 0875
T 0104
T 0107
T 0770
T 0122
T 1021
T 1654
T 1983
T 1026
T 1253
T 1990
T 1857
T 1307
T 1308
T 0949
T 1630
T 1272
T 1433
T 1410
T 1856
T 1565
T 0997
T 1578

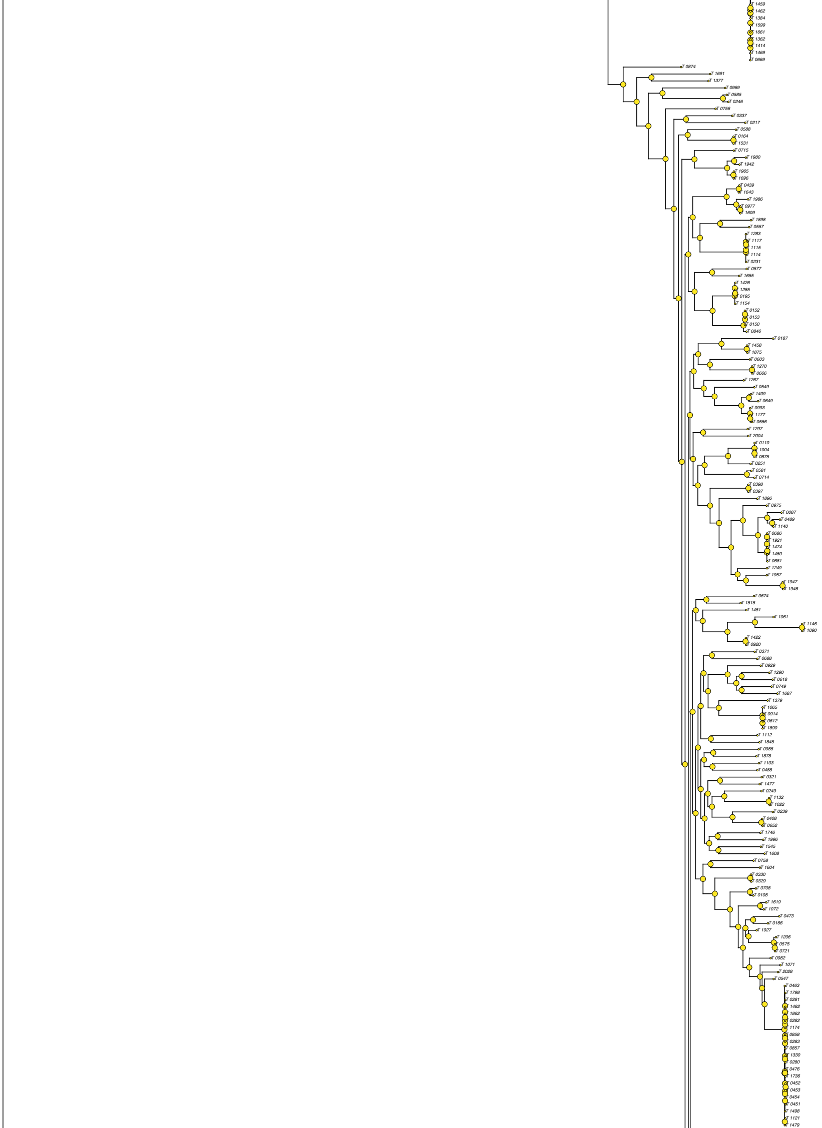
T 1459
T 1462
T 1384
T 1599
T 1661
T 1362
T 1414
T 1469
T 0669
T 0874
T 1691
T 1377
T 0969
T 0585
T 0246
T 0756
T 0337
T 0217
T 0588
T 0164
T 1531
T 0715
T 1980
T 1942
T 1965
T 1696
T 0439
T 1643
T 1986
T 0977
T 1609
T 1898
T 0557
T 1283
T 1117
T 1115
T 1114
T 0231
T 0577
T 1655
T 1426
T 1285
T 0195
T 1154
T 0152
T 0153
T 0150
T 0846
T 0187
T 1458
T 1875
T 0603
T 1270
T 0666
T 1267
T 0549
T 1409
T 0649
T 0993
T 1177
T 0556
T 1297
T 2004
T 0110
T 1004
T 0675
T 0251
T 0581
T 0714
T 0398
T 0397
T 1896
T 0975
T 0087
T 0489
T 1140
T 0686
T 1921
T 1474
T 1450
T 0681
T 1249
T 1957
T 1947
T 1946
T 0674
T 1515
T 1451
T 1061
T 1146
T 1090
T 1422
T 0920
T 0371
T 0688
T 0929
T 1290
T 0618
T 0749
T 1687
T 1379
T 1065
T 0914
T 0612
T 1890
T 1112
T 1845
T 0985
T 1878
T 1103
T 0488
T 0321
T 1477
T 0249
T 1132
T 1022
T 0239
T 0408
T 0652
T 1746
T 1996
T 1545
T 1608
T 0758
T 1604
T 0330
T 0329
T 0708
T 0108
T 1619
T 1072
T 0473
T 0166
T 1927
T 1206
T 0575
T 0721
T 0982
T 1071
T 2028
T 0547
T 0463
T 1798
T 0281
T 1482
T 1862
T 0282
T 1174
T 0858
T 0283
T 0857
T 1330
T 0280
T 0476
T 1736
T 0452
T 0453
T 0454
T 0451
T 1498
T 1121
T 1479

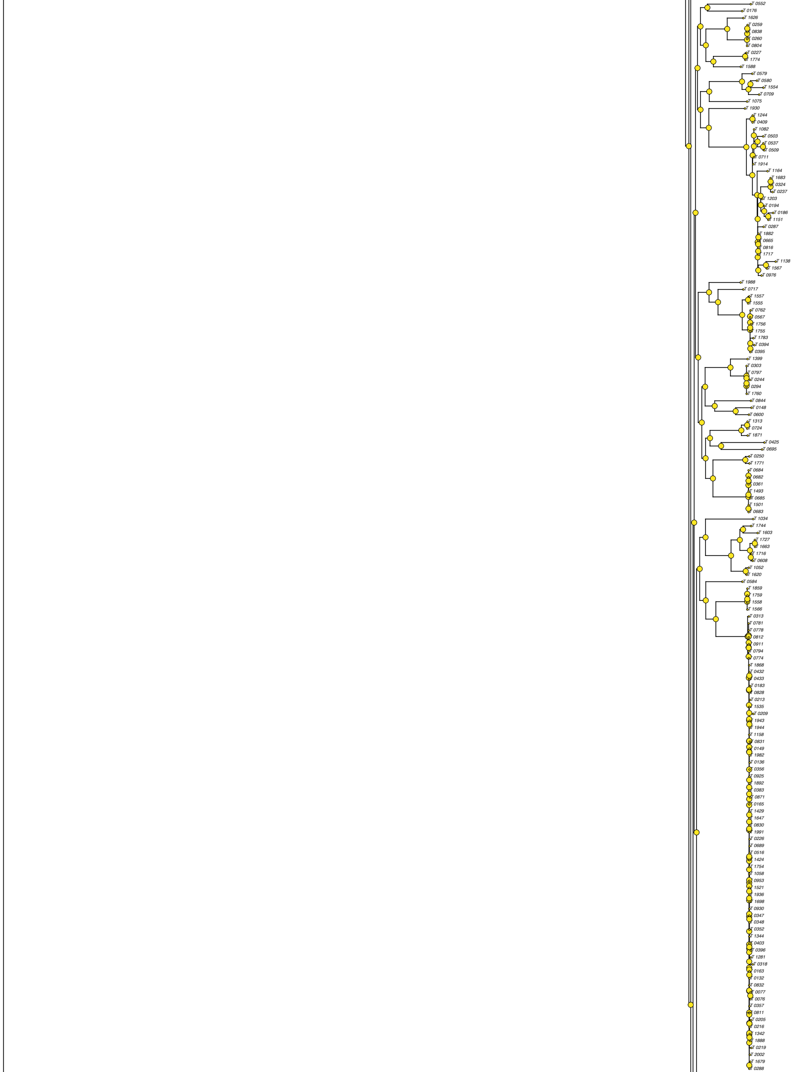
T 0552
T 0176
T 1626
T 0259
0838
0260
0804
T 0227
1774
T 1588
T 0579
T 0580
T 1554
T 0709
T 1075
T 1930
T 1244
T 0409
T 1082
T 0503
T 0537
T 0509
T 0711
T 1914
T 1164
T 1683
0324
T 0237
T 1203
T 0194
T 0186
1151
T 0287
T 1882
0665
0816
1717
T 1138
T 1567
T 0976
T 1988
T 0717
T 1557
1555
T 0762
0567
1756
1755
T 1783
T 0394
0395
T 1399
T 0303
0797
T 0244
0294
T 1760
T 0844
T 0148
T 0600
T 1313
0724
T 1871
T 0425
T 0695
T 0250
T 1771
T 0684
0682
0361
T 1493
T 0685
T 1501
0683
T 1034
T 1744
T 1603
T 1727
T 1663
T 1716
T 0608
T 1052
T 1620
T 0584
T 1859
1759
1558
T 1566
T 0313
T 0781
T 0778
0812
0911
0794
0774
T 1868
T 0432
0433
T 0183
0828
T 0213
1535
T 0209
1943
1944
T 1158
0831
0149
1982
T 0136
0356
T 0925
1892
0383
T 0871
0165
T 1429
1647
0830
1991
T 0226
T 0689
T 0516
1424
T 1754
T 1058
0953
1521
1936
1698
T 0930
0347
0348
T 0352
T 1344
T 0403
T 0396
T 1281
T 0318
0163
0132
T 0832
T 0077
T 0076
T 0357
0811
T 0205
T 0216
1342
1888
T 0219
T 2002
T 1679
0288

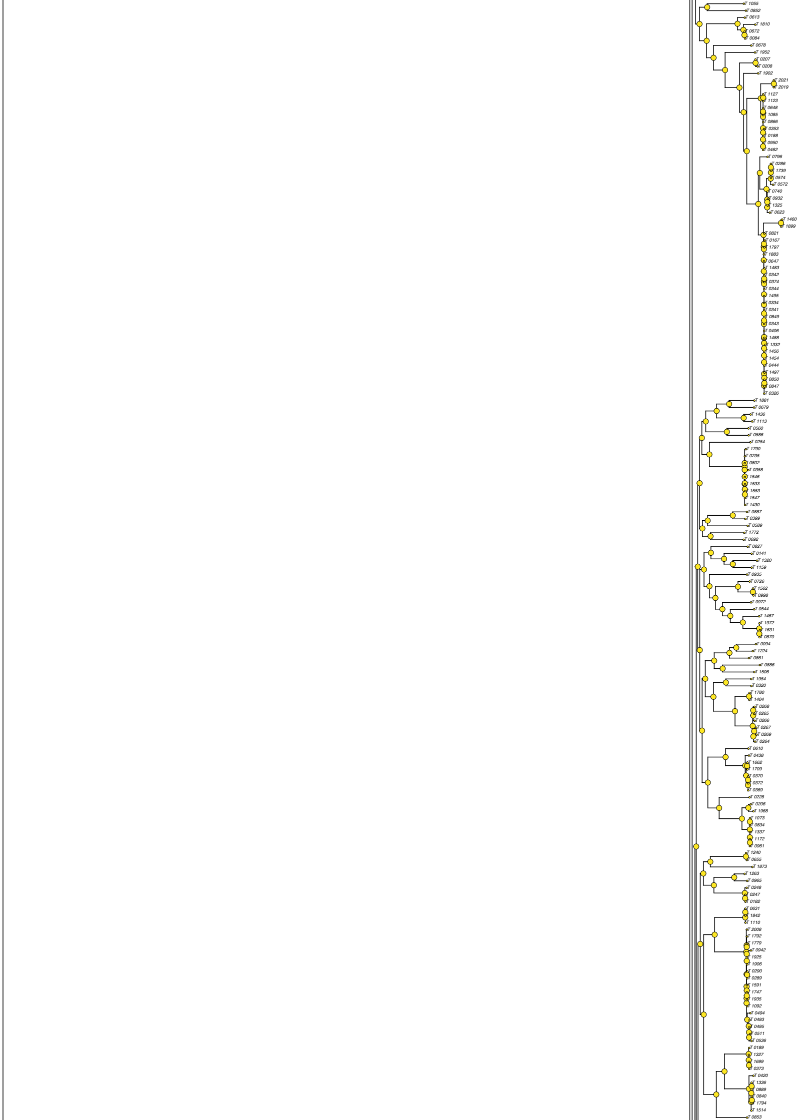

T 1055
T 0852
T 0613
T 1810
T 0672
T 0084
T 0678
T 1952
T 0207
T 0208
T 1902
T 2021
T 2019
T 1127
T 1123
T 0648
T 1085
T 0866
T 0353
T 0188
T 0950
T 0462
T 0796
T 0286
T 1739
T 0574
T 0572
T 0740
T 0932
T 1325
T 0623
T 1460
T 1899
T 0821
T 0167
T 1797
T 1883
T 0647
T 1483
T 0342
T 0374
T 0344
T 1495
T 0334
T 0341
T 0849
T 0343
T 0406
T 1488
T 1332
T 1456
T 1454
T 0444
T 1497
T 0850
T 0847
T 0326
T 1881
T 0679
T 1436
T 1113
T 0560
T 0586
T 0254
T 1790
T 0235
T 0802
T 0358
T 1546
T 1533
T 1553
T 1547
T 1430
T 0887
T 0399
T 0589
T 1772
T 0692
T 0827
T 0141
T 1320
T 1159
T 0935
T 0726
T 1562
T 0998
T 0972
T 0544
T 1467
T 1972
T 1631
T 0870
T 0094
T 1224
T 0861
T 0886
T 1506
T 1954
T 0320
T 1780
T 1404
T 0268
T 0265
T 0266
T 0267
T 0269
T 0264
T 0610
T 0438
T 1662
T 1709
T 0370
T 0372
T 0369
T 0228
T 0206
T 1968
T 1073
T 0834
T 1337
T 1172
T 0961
T 1240
T 0655
T 1873
T 1263
T 0965
T 0248
T 0247
T 0182
T 0631
T 1842
T 1110
T 2008
T 1792
T 1779
T 0942
T 1925
T 1906
T 0290
T 0289
T 1591
T 1747
T 1935
T 1092
T 0494
T 0493
T 0495
T 0511
T 0536
T 0189
T 1327
T 1699
T 0373
T 0420
T 1336
T 0889
T 0840
T 1794
T 1514
T 0853

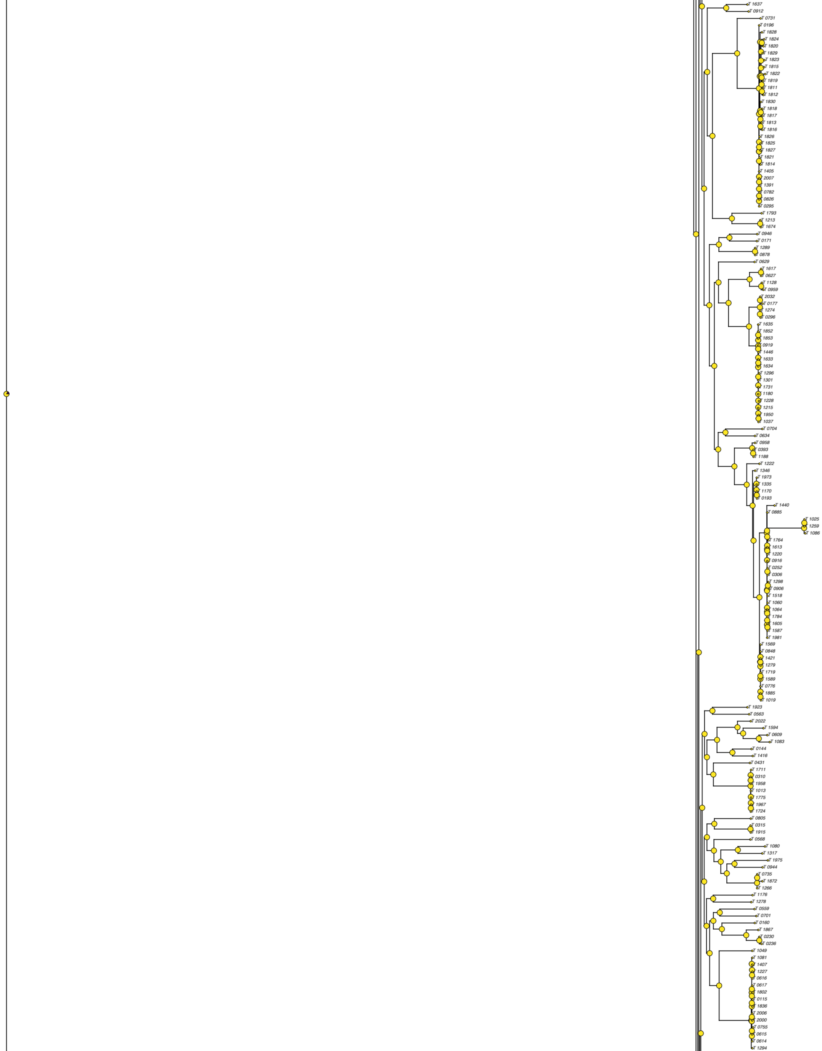
T 1637
T 0912
T 0731
T 0196
T 1828
T 1824
T 1820
T 1829
T 1823
T 1815
T 1822
T 1819
T 1811
T 1812
T 1830
T 1818
T 1817
T 1813
T 1816
T 1826
T 1825
T 1827
T 1821
T 1814
T 1405
T 2007
T 1391
T 0782
T 0826
T 0295
T 1793
T 1213
T 1674
T 0946
T 0171
T 1289
T 0878
T 0629
T 1617
T 0627
T 1128
T 0959
T 2032
T 0177
T 1274
T 0296
T 1635
T 1852
T 1853
T 0919
T 1446
T 1633
T 1634
T 1296
T 1301
T 1731
T 1180
T 1228
T 1215
T 1950
T 1037
T 0704
T 0634
T 0958
T 0393
T 1188
T 1222
T 1346
T 1973
T 1335
T 1170
T 0193
T 1440
T 0885
T 1025
T 1259
T 1086
T 1764
T 1613
T 1220
T 0916
T 0252
T 0306
T 1298
T 0906
T 1518
T 1060
T 1064
T 1784
T 1605
T 1587
T 1981
T 1569
T 0848
T 1421
T 1279
T 1719
T 1589
T 0776
T 1885
T 1019
T 1923
T 0563
T 2022
T 1594
T 0609
T 1083
T 0144
T 1416
T 0431
T 1711
T 0310
T 1958
T 1013
T 1775
T 1967
T 1724
T 0805
T 0315
T 1915
T 0568
T 1080
T 1317
T 1975
T 0944
T 0735
T 1872
T 1266
T 1176
T 1278
T 0559
T 0701
T 0160
T 1867
T 0230
T 0236
T 1049
T 1081
T 1407
T 1227
T 0616
T 0617
T 1802
T 0115
T 1836
T 2006
T 2000
T 0755
T 0615
T 0614
T 1294

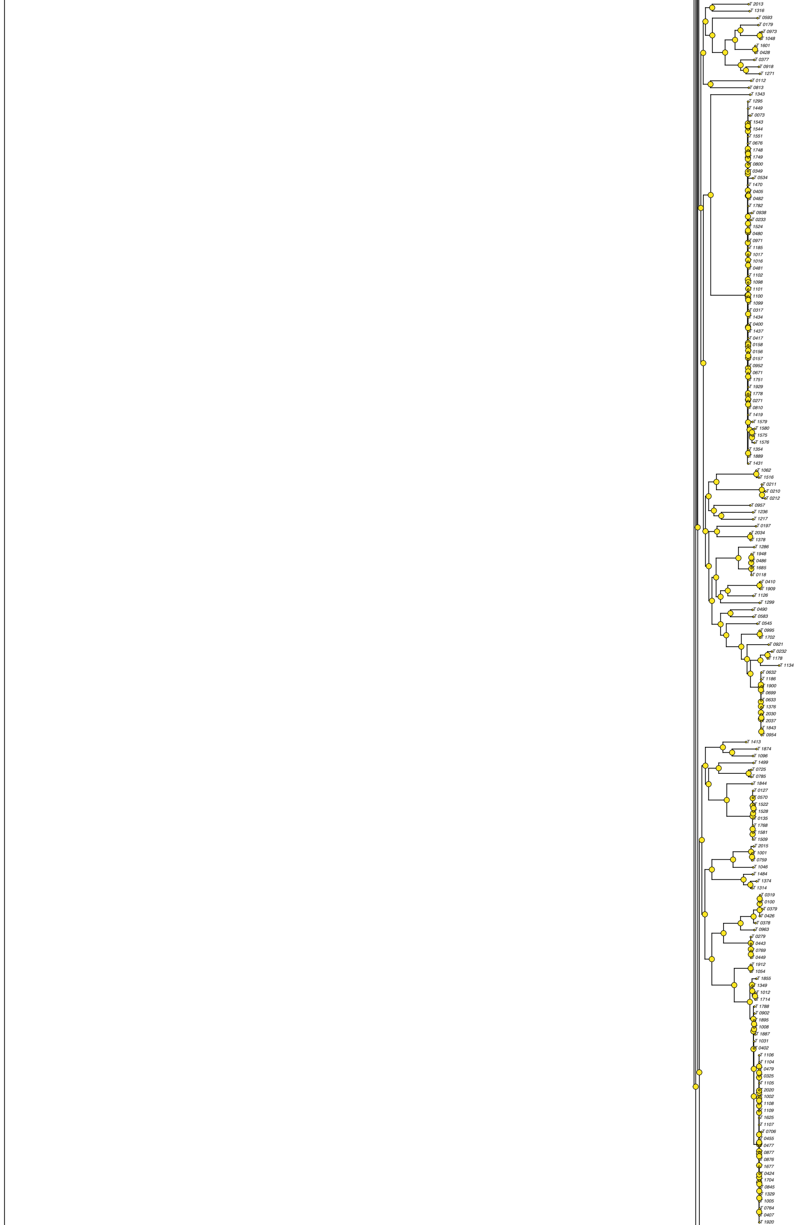
T 2013
T 1316
T 0593
T 0179
T 0973
T 1048
T 1601
T 0428
T 0377
T 0918
T 1271
T 0112
T 0813
T 1343
T 1295
T 1449
T 0073
T 1543
T 1544
T 1551
T 0676
T 1748
T 1749
T 0800
T 0349
T 0534
T 1470
T 0405
T 0482
T 1782
T 0938
T 0233
T 1524
T 0480
T 0971
T 1185
T 1017
T 1016
T 0481
T 1102
T 1098
T 1101
T 1100
T 1099
T 0317
T 1434
T 0400
T 1437
T 0417
T 0158
T 0156
T 0157
T 0952
T 0671
T 1751
T 1929
T 1778
T 0271
T 0810
T 1419
T 1579
T 1580
T 1575
T 1576
T 1354
T 1889
T 1431
T 1062
T 1516
T 0211
T 0210
T 0212
T 0957
T 1236
T 1217
T 0197
T 2034
T 1378
T 1286
T 1948
T 0486
T 1685
T 0118
T 0410
T 1909
T 1126
T 1299
T 0490
T 0583
T 0545
T 0995
T 1702
T 0921
T 0232
T 1178
T 1134
T 0632
T 1186
T 1900
T 0699
T 0633
T 1376
T 2030
T 2037
T 1843
T 0954
T 1413
T 1874
T 1096
T 1499
T 0725
T 0785
T 1844
T 0127
T 0570
T 1522
T 1528
T 0135
T 1768
T 1581
T 1509
T 2015
T 1001
T 0759
T 1046
T 1484
T 1374
T 1314
T 0319
T 0100
T 0379
T 0426
T 0378
T 0963
T 0279
T 0443
T 0769
T 0449
T 1912
T 1054
T 1855
T 1349
T 1012
T 1714
T 1788
T 0902
T 1895
T 1008
T 1887
T 1031
T 0402
T 1106
T 1104
T 0479
T 0325
T 1105
T 2020
T 1002
T 1108
T 1109
T 1625
T 1107
T 0706
T 0455
T 0477
T 0877
T 0876
T 1677
T 0424
T 1704
T 0845
T 1329
T 1005
T 0764
T 0407
T 1920

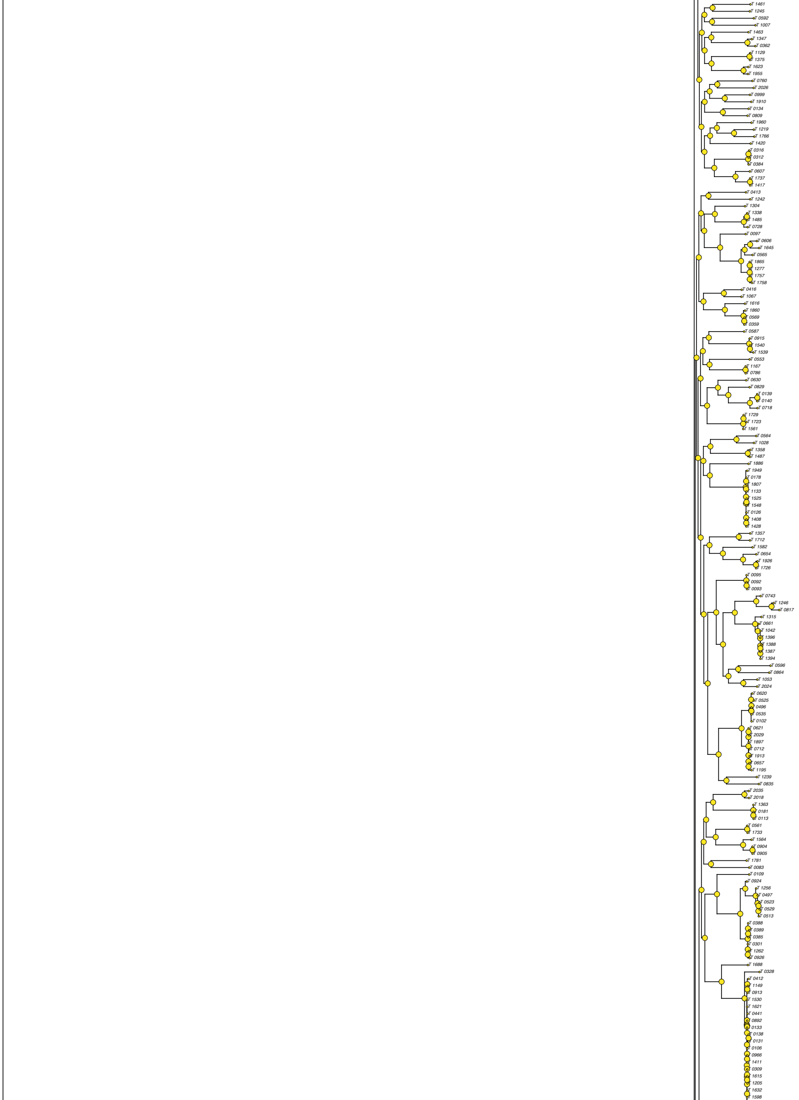
T 1461
T 1245
T 0592
T 1007
T 1463
T 1347
T 0362
T 1129
T 1375
T 1623
T 1955
T 0760
T 2026
T 0999
T 1910
T 0134
T 0809
T 1960
T 1219
T 1766
T 1420
T 0316
T 0312
T 0384
T 0607
T 1737
T 1417
T 0413
T 1242
T 1304
T 1338
T 1485
T 0728
T 0097
T 0606
T 1645
T 0565
T 1865
T 1277
T 1757
T 1758
T 0416
T 1067
T 1616
T 1860
T 0569
T 0359
T 0587
T 0915
T 1540
T 1539
T 0553
T 1167
T 0786
T 0630
T 0829
T 0139
T 0140
T 0718
T 1729
T 1723
T 1561
T 0564
T 1028
T 1358
T 1487
T 1886
T 1949
T 0178
T 1807
T 1133
T 1525
T 1548
T 0126
T 1408
T 1428
T 1357
T 1712
T 1582
T 0654
T 1926
T 1726
T 0095
T 0092
T 0093
T 0743
T 1246
T 0817
T 1315
T 0661
T 1042
T 1396
T 1388
T 1387
T 1394
T 0596
T 0864
T 1053
T 2024
T 0620
T 0525
T 0496
T 0535
T 0102
T 0621
T 2029
T 1897
T 0712
T 1913
T 0657
T 1195
T 1239
T 0835
T 2035
T 2018
T 1363
T 0181
T 0113
T 0561
T 1733
T 1564
T 0904
T 0905
T 1781
T 0083
T 0109
T 0924
T 1256
T 0497
T 0523
T 0529
T 0513
T 0388
T 0389
T 0385
T 0301
T 1262
T 0926
T 1688
T 0328
T 0412
T 1149
T 0913
T 1530
T 1621
T 0441
T 0892
T 0133
T 0138
T 0131
T 0106
T 0966
T 1411
T 0309
T 1615
T 1205
T 1632
T 1598

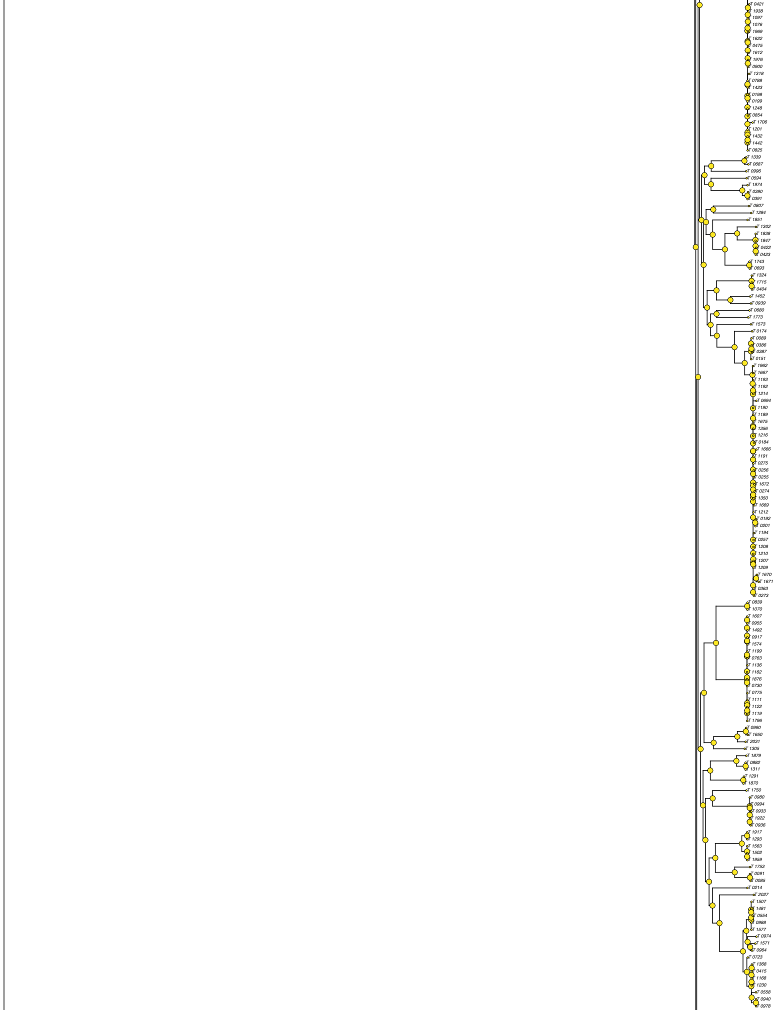
T 0421
T 1938
T 1097
T 1076
T 1969
T 1622
T 0475
T 1612
T 1976
T 0900
T 1318
T 0788
T 1423
T 0198
T 0199
T 1248
T 0854
T 1706
T 1201
T 1432
T 1442
T 0825
T 1339
T 0687
T 0996
T 0594
T 1974
T 0390
T 0391
T 0807
T 1284
T 1851
T 1302
T 1838
T 1847
T 0422
T 0423
T 1743
T 0693
T 1324
T 1715
T 0404
T 1452
T 0939
T 0680
T 1773
T 1573
T 0174
T 0089
T 0386
T 0387
T 0151
T 1962
T 1667
T 1193
T 1192
T 1214
T 0694
T 1190
T 1189
T 1675
T 1356
T 1216
T 0184
T 1666
T 1191
T 0275
T 0256
T 0255
T 1672
T 0274
T 1350
T 1669
T 1212
T 0192
T 0201
T 1194
T 0257
T 1208
T 1210
T 1207
T 1209
T 1670
T 1671
T 0363
T 0273
T 0839
T 1070
T 1607
T 0955
T 1492
T 0917
T 1574
T 1199
T 0763
T 1136
T 1162
T 1876
T 0730
T 0775
T 1111
T 1122
T 1119
T 1796
T 0990
T 1650
T 2031
T 1305
T 1879
T 0882
T 1311
T 1291
T 1870
T 1750
T 0980
T 0994
T 0933
T 1922
T 0936
T 1917
T 1293
T 1563
T 1502
T 1959
T 1753
T 0091
T 0085
T 0214
T 2027
T 1507
T 1481
T 0554
T 0988
T 1577
T 0974
T 1571
T 0964
T 0723
T 1368
T 0415
T 1168
T 1230
T 0558
T 0940
T 0978

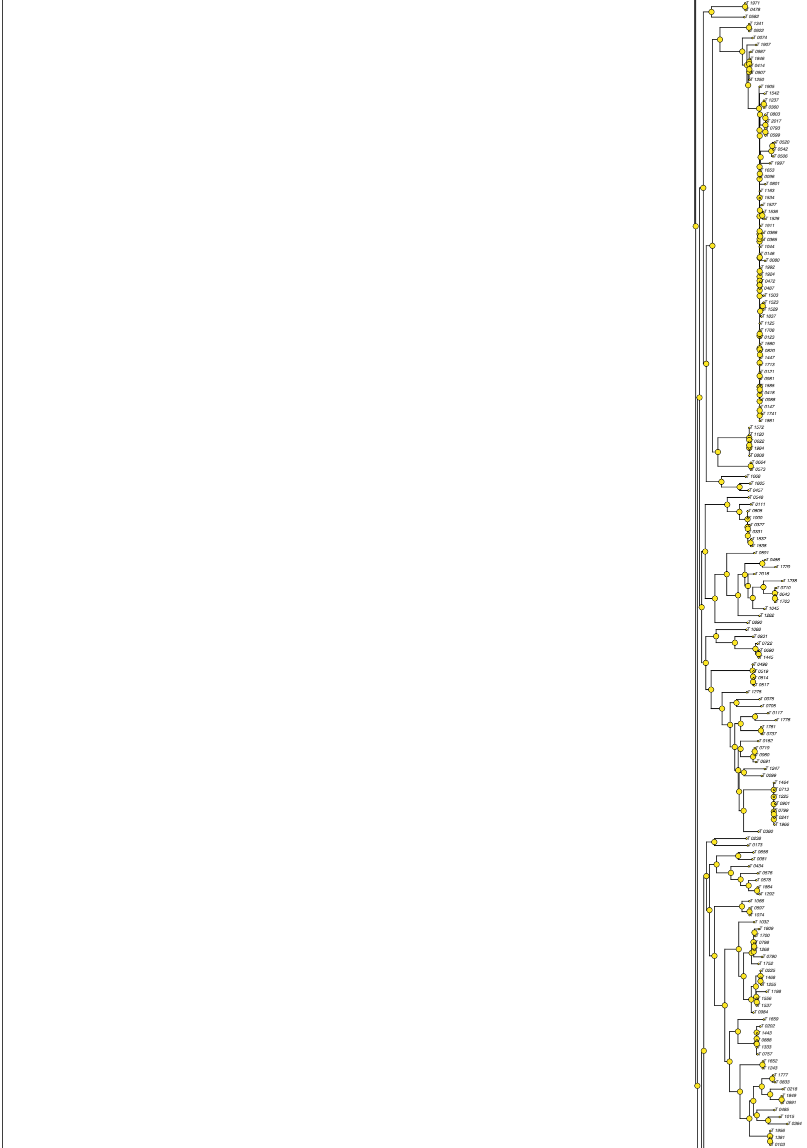

T 1971
T 0478
T 0582
T 1341
T 0922
T 0074
T 1907
T 0987
T 1846
T 0414
T 0907
T 1250
T 1905
T 1542
T 1237
T 0360
T 0803
T 2017
T 0793
T 0599
T 0520
T 0542
T 0506
T 1997
T 1653
T 0096
T 0801
T 1163
T 1534
T 1527
T 1536
T 1526
T 1911
T 0366
T 0365
T 1044
T 0146
T 0080
T 1992
T 1924
T 0472
T 0487
T 1503
T 1523
T 1529
T 1837
T 1125
T 1708
T 0123
T 1560
T 0820
T 1447
T 1713
T 0121
T 0981
T 1585
T 0418
T 0088
T 0147
T 1741
T 1861
T 1572
T 1120
T 0622
T 1984
T 0808
T 0664
T 0573
T 1068
T 1805
T 0457
T 0548
T 0111
T 0605
T 1000
T 0327
T 0331
T 1532
T 1538
T 0591
T 0456
T 1720
T 2016
T 1238
T 0710
T 0643
T 1703
T 1045
T 1282
T 0890
T 1088
T 0931
T 0722
T 0690
T 1445
T 0498
T 0519
T 0514
T 0517
T 1275
T 0075
T 0705
T 0117
T 1776
T 1761
T 0737
T 0162
T 0719
T 0960
T 0691
T 1247
T 0099
T 1464
T 0713
T 1225
T 0901
T 0799
T 0241
T 1966
T 0380
T 0238
T 0173
T 0656
T 0081
T 0434
T 0576
T 0578
T 1864
T 1292
T 1066
T 0597
T 1074
T 1032
T 1809
T 1700
T 0798
T 1268
T 0790
T 1752
T 0225
T 1468
T 1255
T 1198
T 1556
T 1537
T 0984
T 1659
T 0202
T 1443
T 0888
T 1333
T 0757
T 1652
T 1243
T 1777
T 0833
T 0218
T 1849
T 0991
T 0485
T 1015
T 0364
T 1956
T 1381
T 0103

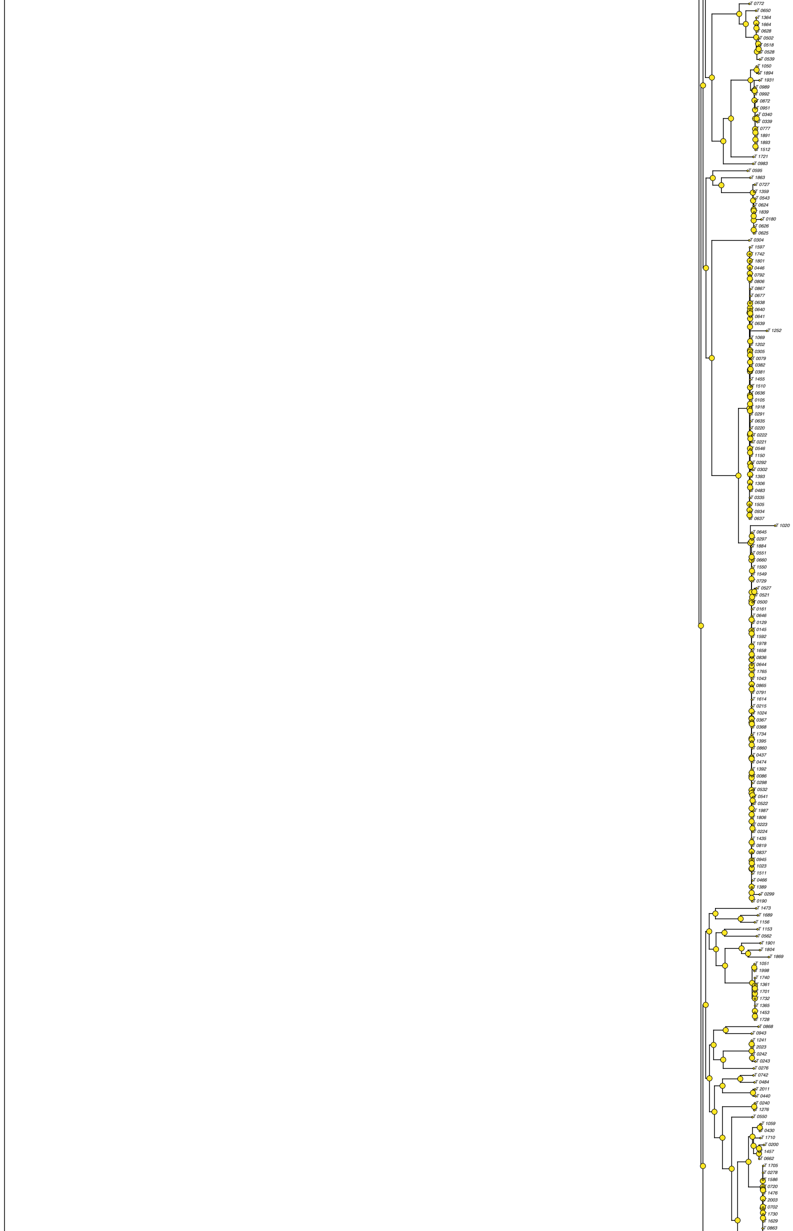
T 0772
T 0650
T 1364
T 1664
T 0628
T 0502
T 0518
T 0528
T 0539
T 1050
T 1894
T 1931
T 0989
T 0992
T 0872
T 0951
T 0340
T 0339
T 0777
T 1891
T 1893
T 1512
T 1721
T 0983
T 0595
T 1863
T 0727
T 1359
T 0543
T 0624
T 1839
T 0180
T 0626
T 0625
T 0304
T 1597
T 1742
T 1801
T 0446
T 0792
T 0806
T 0867
T 0677
T 0638
T 0640
T 0641
T 0639
T 1252
T 1069
T 1202
T 0305
T 0079
T 0382
T 0381
T 1455
T 1510
T 0636
T 0105
T 1918
T 0291
T 0635
T 0220
T 0222
T 0221
T 0546
T 1150
T 0292
T 0302
T 1393
T 1306
T 0483
T 0335
T 1505
T 0934
T 0637
T 1020
T 0645
T 0297
T 1884
T 0551
T 0660
T 1550
T 1549
T 0729
T 0527
T 0521
T 0500
T 0161
T 0646
T 0129
T 0145
T 1592
T 1978
T 1658
T 0836
T 0644
T 1765
T 1043
T 0865
T 0791
T 1614
T 0215
T 1024
T 0367
T 0368
T 1734
T 1395
T 0860
T 0437
T 0474
T 1392
T 0086
T 0298
T 0532
T 0541
T 0522
T 1987
T 1806
T 0223
T 0224
T 1435
T 0819
T 0837
T 0945
T 1023
T 1511
T 0466
T 1389
T 0299
T 0190
T 1473
T 1689
T 1156
T 1153
T 0562
T 1901
T 1804
T 1869
T 1051
T 1998
T 1740
T 1361
T 1701
T 1732
T 1365
T 1453
T 1728
T 0868
T 0943
T 1241
T 2023
T 0242
T 0243
T 0276
T 0742
T 0484
T 2011
T 0440
T 0240
T 1276
T 0550
T 1059
T 0430
T 1710
T 0200
T 1457
T 0662
T 1705
T 0278
T 1586
T 0720
T 1476
T 2003
T 0702
T 1730
T 1629
T 0863

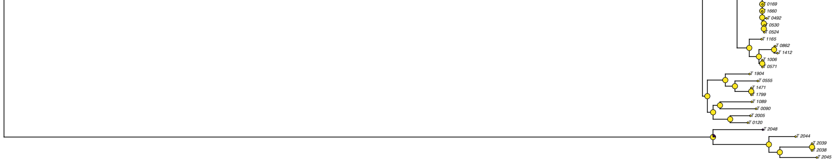
T 0169
T 1660
T 0492
T 0530
T 0524
T 1165
T 0862
T 1412
T 1006
T 0571
T 1904
T 0555
T 1471
T 1799
T 1089
T 0090
T 2005
T 0120
T 2048
T 2044
T 2039
T 2038
T 2045
