# Supplementary material for: Distribution of the four type VI secretion systems in Pseudomonas aeruginosa and classification of their core and accessory effectors
Source: Nat Commun. 2025 Jan 21;16:888. doi: 10.1038/s41467-024-54649-5 (PMC11751169; doi:10.1038/s41467-024-54649-5)
Supplement: Supplementary file 24 — Supplementary Dataset 22 [file 41467_2024_54649_MOESM24_ESM.pdf]

Distribution of the four type VI secretion systems in *Pseudomonas aeruginosa* and classification of their core and accessory effectors

Supplementary Data 22

To view this file, open in Preview (MacOS) or Microsoft Edge (Windows).

This file contains the results of the stochastic mapping of *tle3* and *vgrG2b-C-ter* at the two different loci. The respective effector gene and the locus are indicated at the top left corner of the page. The posterior probabilities of the stochastic mapping are indicated on each node of the phylogenetic tree. The tree is based on a core genome alignment of *P. aeruginosa* (n=1912) and *P. paraeruginosa* (n=5). Seven strains of the species *P. delhiensis, P. knackmussii, P. humi, P. jinjuensis, P. multiresinivorans, P. nitroreducens,* and *P. panipatensis* were used as an outgroup (not shown in the tree). The tree is based on the HKY+F+I model, rooted to the outgroup and distances are shown in substitutions per site.

Effector gene: tle3
Locus 1

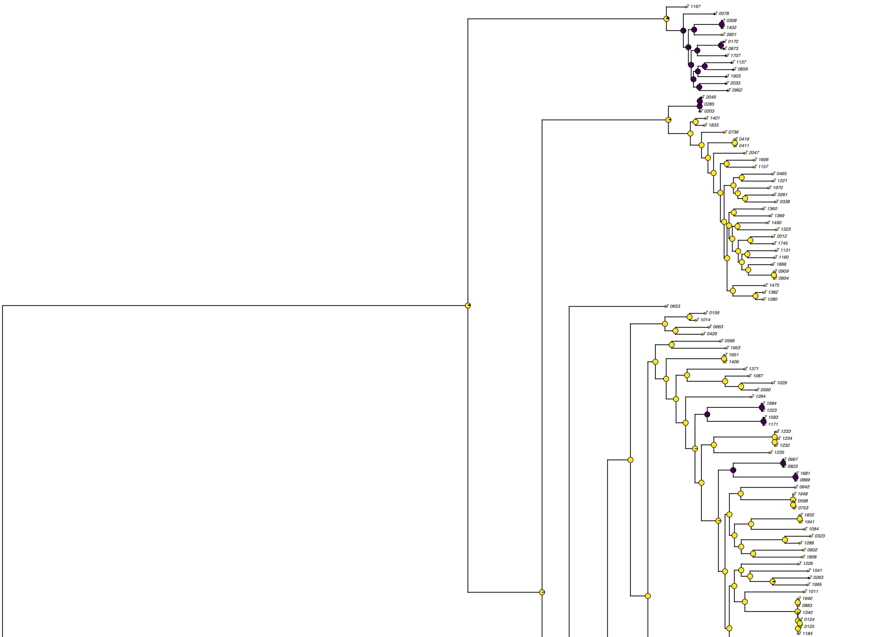

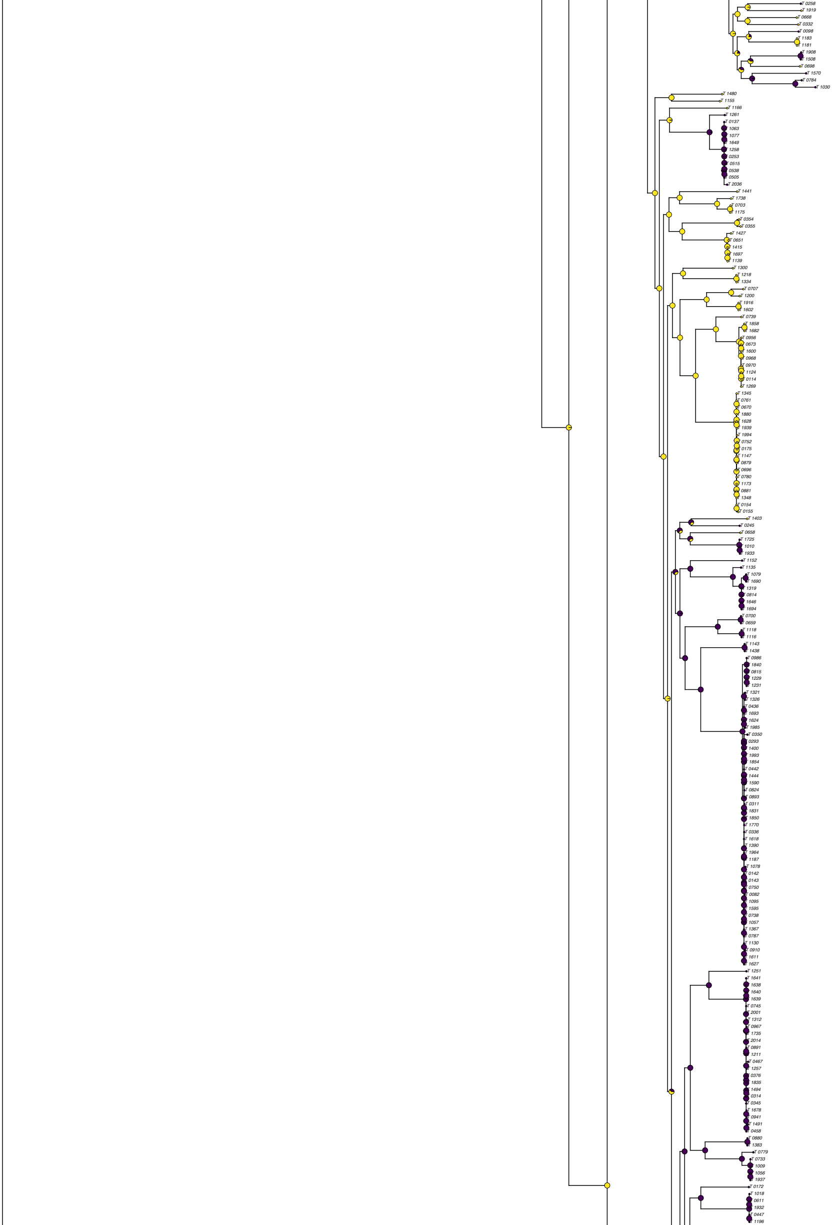
T 0258
T 1919
T 0668
T 0332
T 0098
T 1183
1181
T 1908
T 1508
T 0698
T 1570
T 0784
T 1030
T 1480
T 1155
T 1166
T 1261
T 0137
1063
1077
1649
1258
0253
0515
0538
0505
T 2036
T 1441
T 1738
T 0703
T 1175
T 0354
T 0355
T 1427
T 0651
1415
1697
1139
T 1300
T 1218
1334
T 0707
T 1200
T 1916
T 1602
T 0739
T 1858
T 1682
T 0956
0673
1600
0968
T 0970
1124
0114
T 1269
T 1345
T 0761
0670
1880
1628
1939
T 1994
0752
0175
T 1147
0879
T 0696
T 0780
1173
0881
1348
0154
T 0155
T 1403
T 0245
T 0658
T 1725
1010
T 1933
T 1152
T 1135
T 1079
T 1690
1319
0814
1646
1694
T 0700
T 0659
T 1118
T 1116
T 1143
T 1438
T 0986
1840
0815
1229
T 1231
T 1321
T 1326
T 0436
1693
1624
T 1985
T 0350
0293
1400
1993
1854
T 0442
1444
1590
T 0824
0893
T 0311
1831
1850
T 1770
T 0336
T 1618
T 1390
1964
1187
T 1078
0142
0143
0750
T 0082
1095
1595
0738
1057
T 1367
0787
T 1130
T 0910
1611
1627
T 1251
T 1641
1638
1640
1639
T 0745
2001
T 1312
T 0967
T 1735
2014
T 0891
1211
T 0467
T 1257
0376
1835
1494
0314
T 0345
T 1678
0941
T 1491
T 0458
T 0880
T 1383
T 0779
T 0733
1009
1056
T 1937
T 0172
T 1018
0611
1932
T 0447
T 1196

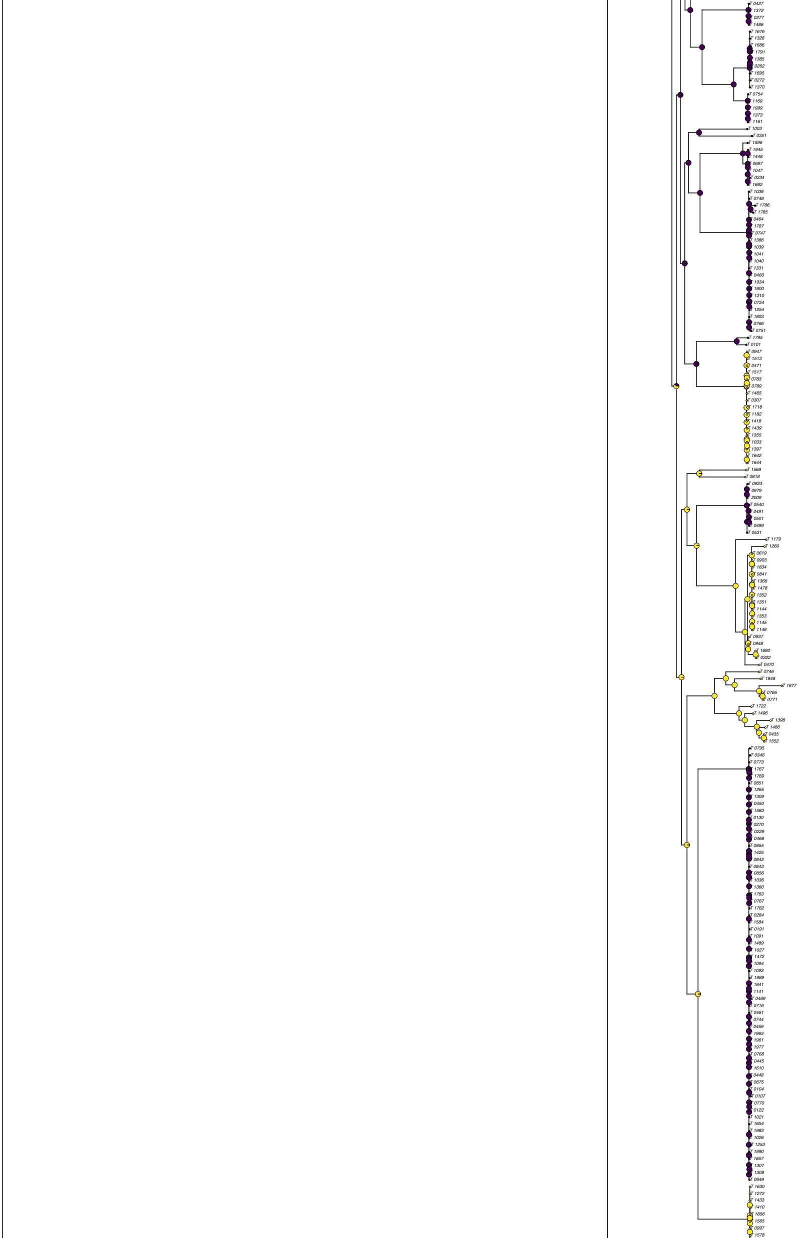

T 0427
1372
0277
T 1486
T 1676
T 1328
T 1686
1791
1385
0262
T 1695
T 0272
T 1370
T 0754
1169
1999
1373
T 1161
T 1003
T 0351
T 1596
T 1945
T 1448
0697
T 1047
T 0234
T 1692
T 1038
T 0748
T 1786
T 1785
0464
1787
T 0747
T 1386
1039
1041
T 1040
T 1331
0460
1934
1800
1310
0734
T 1254
T 1803
0766
T 0751
T 1795
T 0101
T 0947
T 1513
0471
T 1517
0783
0789
T 1465
T 0307
T 1718
1182
1418
T 1439
T 1355
1033
1397
T 1642
1644
T 1568
T 0818
T 0923
0979
T 2009
T 0540
0491
0501
T 0499
T 0531
T 1179
T 1260
0619
T 0903
1834
0841
T 1366
T 1478
1352
T 1351
1144
1353
1145
1148
T 0937
0948
T 1680
T 0322
T 0470
T 0746
T 1848
T 1877
T 0765
T 0771
T 1722
T 1496
T 1398
T 1466
T 0435
T 1552
T 0795
T 0346
T 0773
1767
T 1769
T 0851
1265
1309
0450
T 1583
T 0130
T 0270
T 0229
0468
T 0855
1425
0842
T 0843
0856
T 1036
1380
T 1763
0767
T 1762
T 0284
T 1584
T 0191
T 1091
T 1489
1027
T 1472
1094
T 1093
T 1989
1841
1141
T 0469
T 0716
T 0461
0744
0459
1963
1961
1977
T 0768
0445
1610
0448
T 0875
T 0104
T 0107
0770
0122
T 1021
T 1654
T 1983
1026
T 1253
T 1990
T 1857
1307
1308
T 0949
T 1630
T 1272
T 1433
1410
1856
1565
T 0997
1578

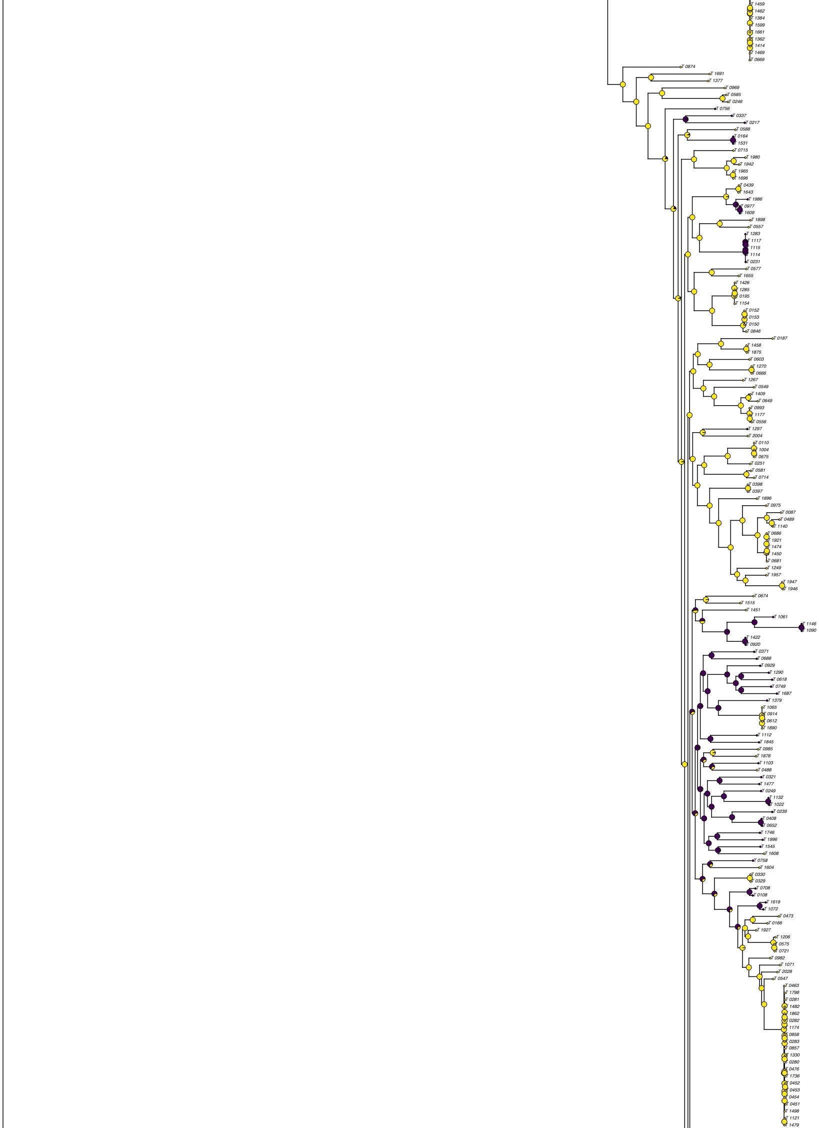
T 1459
T 1462
T 1384
T 1599
T 1661
T 1362
T 1414
T 1469
T 0669
T 0874
T 1691
T 1377
T 0969
T 0585
T 0246
T 0756
T 0337
T 0217
T 0588
T 0164
T 1531
T 0715
T 1980
T 1942
T 1965
T 1696
T 0439
T 1643
T 1986
T 0977
T 1609
T 1898
T 0557
T 1283
T 1117
T 1115
T 1114
T 0231
T 0577
T 1655
T 1426
T 1285
T 0195
T 1154
T 0152
T 0153
T 0150
T 0846
T 0187
T 1458
T 1875
T 0603
T 1270
T 0666
T 1267
T 0549
T 1409
T 0649
T 0993
T 1177
T 0556
T 1297
T 2004
T 0110
T 1004
T 0675
T 0251
T 0581
T 0714
T 0398
T 0397
T 1896
T 0975
T 0087
T 0489
T 1140
T 0686
T 1921
T 1474
T 1450
T 0681
T 1249
T 1957
T 1947
T 1946
T 0674
T 1515
T 1451
T 1061
T 1146
T 1090
T 1422
T 0920
T 0371
T 0688
T 0929
T 1290
T 0618
T 0749
T 1687
T 1379
T 1065
T 0914
T 0612
T 1890
T 1112
T 1845
T 0985
T 1878
T 1103
T 0488
T 0321
T 1477
T 0249
T 1132
T 1022
T 0239
T 0408
T 0652
T 1746
T 1996
T 1545
T 1608
T 0758
T 1604
T 0330
T 0329
T 0708
T 0108
T 1619
T 1072
T 0473
T 0166
T 1927
T 1206
T 0575
T 0721
T 0982
T 1071
T 2028
T 0547
T 0463
T 1798
T 0281
T 1482
T 1862
T 0282
T 1174
T 0858
T 0283
T 0857
T 1330
T 0280
T 0476
T 1736
T 0452
T 0453
T 0454
T 0451
T 1498
T 1121
T 1479

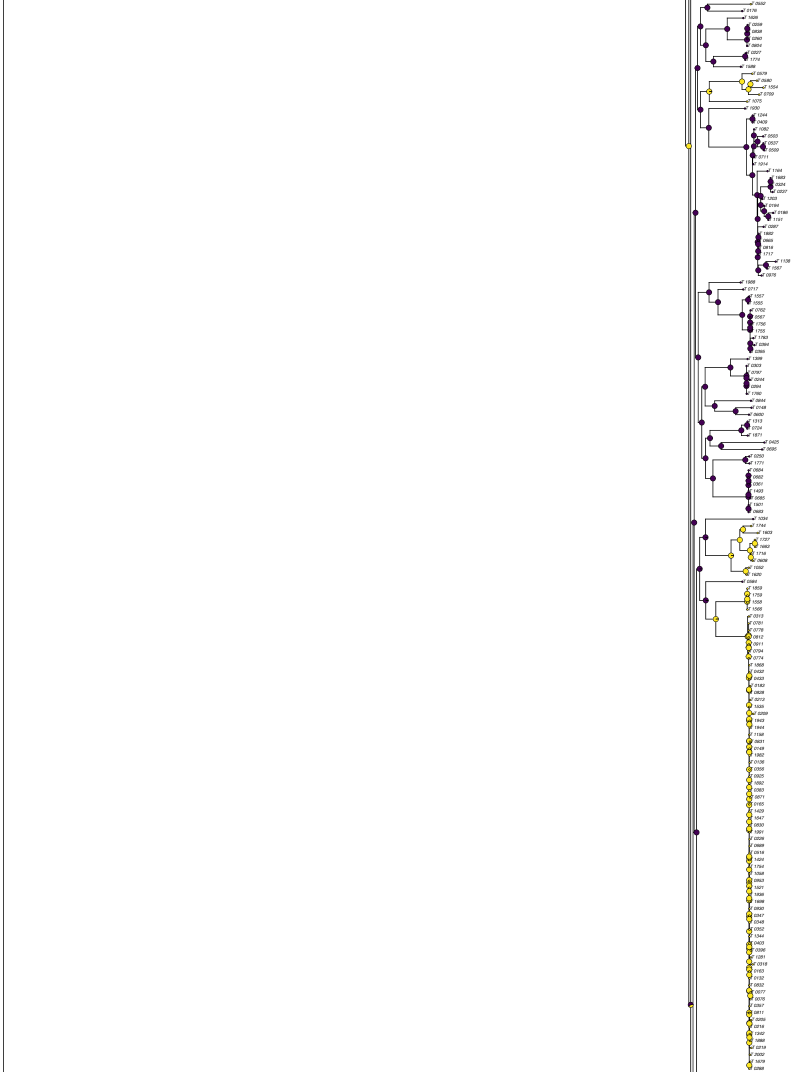
T 0552
T 0176
T 1626
T 0259
0838
0260
T 0804
T 0227
T 1774
T 1588
T 0579
T 0580
T 1554
T 0709
T 1075
T 1930
T 1244
T 0409
T 1082
T 0503
T 0537
T 0509
T 0711
T 1914
T 1164
T 1683
0324
T 0237
T 1203
T 0194
T 0186
T 1151
T 0287
T 1882
0665
T 0816
1717
T 1138
T 1567
T 0976
T 1988
T 0717
T 1557
1555
T 0762
0567
T 1756
1755
T 1783
T 0394
T 0395
T 1399
T 0303
T 0797
T 0244
0294
T 1760
T 0844
T 0148
T 0600
T 1313
T 0724
T 1871
T 0425
T 0695
T 0250
T 1771
T 0684
0682
0361
T 1493
T 0685
T 1501
T 0683
T 1034
T 1744
T 1603
T 1727
T 1663
T 1716
T 0608
T 1052
T 1620
T 0584
T 1859
1759
1558
T 1566
T 0313
T 0781
T 0778
0812
0911
T 0794
0774
T 1868
T 0432
0433
T 0183
T 0828
T 0213
1535
T 0209
1943
1944
T 1158
0831
0149
T 1982
T 0136
0356
T 0925
1892
0383
T 0871
0165
T 1429
1647
0830
T 1991
T 0226
T 0689
T 0516
1424
T 1754
T 1058
0953
1521
T 1936
T 1698
T 0930
T 0347
0348
0352
T 1344
T 0403
T 0396
T 1281
T 0318
0163
T 0132
T 0832
T 0077
T 0076
T 0357
0811
T 0205
T 0216
1342
T 1888
T 0219
T 2002
T 1679
T 0288

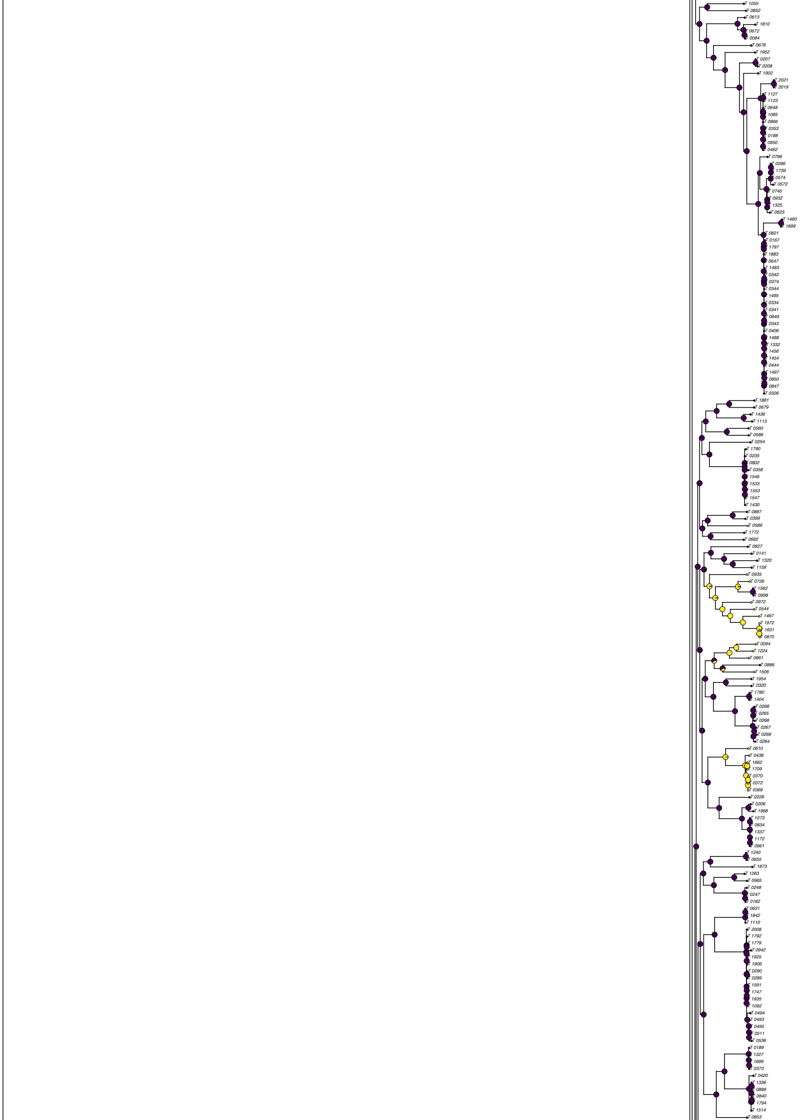
T 1055
T 0852
T 0613
T 1810
T 0672
T 0084
T 0678
T 1952
T 0207
T 0208
T 1902
T 2021
T 2019
T 1127
T 1123
T 0648
T 1085
T 0866
T 0353
T 0188
T 0950
T 0462
T 0796
T 0286
T 1739
T 0574
T 0572
T 0740
T 0932
T 1325
T 0623
T 1460
T 1899
T 0821
T 0167
T 1797
T 1883
T 0647
T 1483
T 0342
T 0374
T 0344
T 1495
T 0334
T 0341
T 0849
T 0343
T 0406
T 1488
T 1332
T 1456
T 1454
T 0444
T 1497
T 0850
T 0847
T 0326
T 1881
T 0679
T 1436
T 1113
T 0560
T 0586
T 0254
T 1790
T 0235
T 0802
T 0358
T 1546
T 1533
T 1553
T 1547
T 1430
T 0887
T 0399
T 0589
T 1772
T 0692
T 0827
T 0141
T 1320
T 1159
T 0935
T 0726
T 1562
T 0998
T 0972
T 0544
T 1467
T 1972
T 1631
T 0870
T 0094
T 1224
T 0861
T 0886
T 1506
T 1954
T 0320
T 1780
T 1404
T 0268
T 0265
T 0266
T 0267
T 0269
T 0264
T 0610
T 0438
T 1662
T 1709
T 0370
T 0372
T 0369
T 0228
T 0206
T 1968
T 1073
T 0834
T 1337
T 1172
T 0961
T 1240
T 0655
T 1873
T 1263
T 0965
T 0248
T 0247
T 0182
T 0631
T 1842
T 1110
T 2008
T 1792
T 1779
T 0942
T 1925
T 1906
T 0290
T 0289
T 1591
T 1747
T 1935
T 1092
T 0494
T 0493
T 0495
T 0511
T 0536
T 0189
T 1327
T 1699
T 0373
T 0420
T 1336
T 0889
T 0840
T 1794
T 1514
T 0853

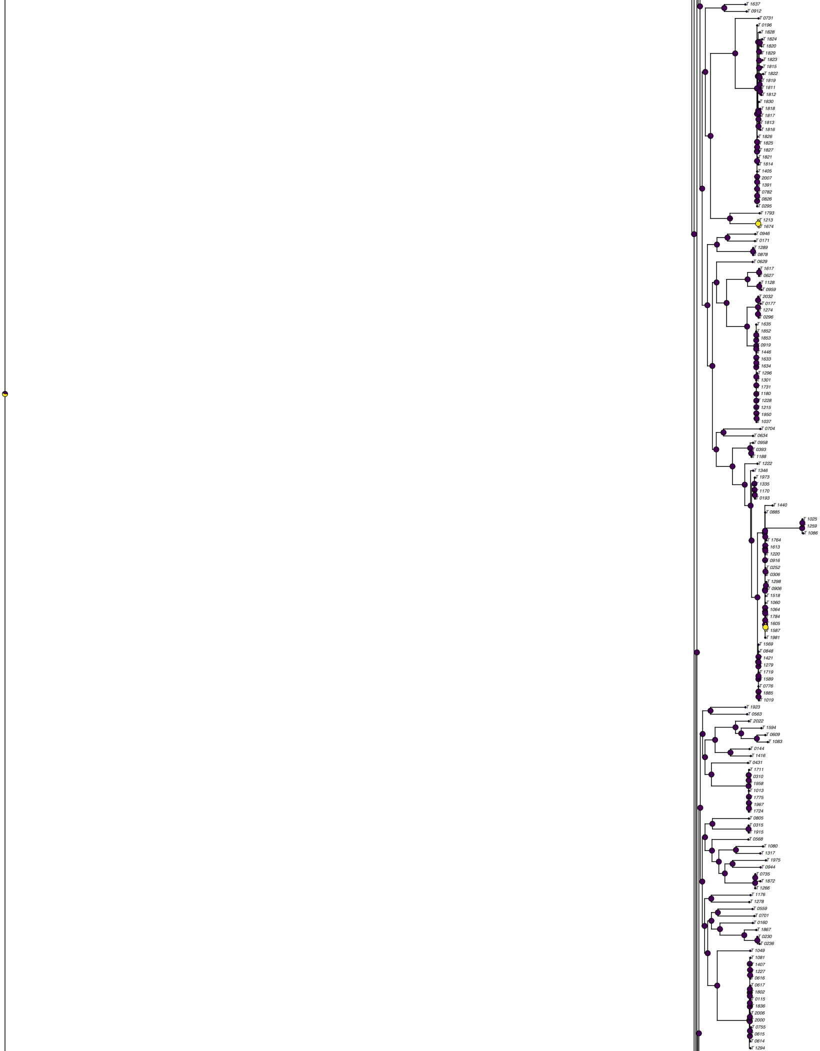
T 1637
T 0912
T 0731
T 0196
T 1828
T 1824
T 1820
T 1829
T 1823
T 1815
T 1822
T 1819
T 1811
T 1812
T 1830
T 1818
T 1817
T 1813
T 1816
T 1826
T 1825
T 1827
T 1821
T 1814
T 1405
T 2007
T 1391
T 0782
T 0826
T 0295
T 1793
T 1213
T 1674
T 0946
T 0171
T 1289
T 0878
T 0629
T 1617
T 0627
T 1128
T 0959
T 2032
T 0177
T 1274
T 0296
T 1635
T 1852
T 1853
T 0919
T 1446
T 1633
T 1634
T 1296
T 1301
T 1731
T 1180
T 1228
T 1215
T 1950
T 1037
T 0704
T 0634
T 0958
T 0393
T 1188
T 1222
T 1346
T 1973
T 1335
T 1170
T 0193
T 1440
T 0885
T 1025
T 1259
T 1086
T 1764
T 1613
T 1220
T 0916
T 0252
T 0306
T 1298
T 0906
T 1518
T 1060
T 1064
T 1784
T 1605
T 1587
T 1981
T 1569
T 0848
T 1421
T 1279
T 1719
T 1589
T 0776
T 1885
T 1019
T 1923
T 0563
T 2022
T 1594
T 0609
T 1083
T 0144
T 1416
T 0431
T 1711
T 0310
T 1958
T 1013
T 1775
T 1967
T 1724
T 0805
T 0315
T 1915
T 0568
T 1080
T 1317
T 1975
T 0944
T 0735
T 1872
T 1266
T 1176
T 1278
T 0559
T 0701
T 0160
T 1867
T 0230
T 0236
T 1049
T 1081
T 1407
T 1227
T 0616
T 0617
T 1802
T 0115
T 1836
T 2006
T 2000
T 0755
T 0615
T 0614
T 1294

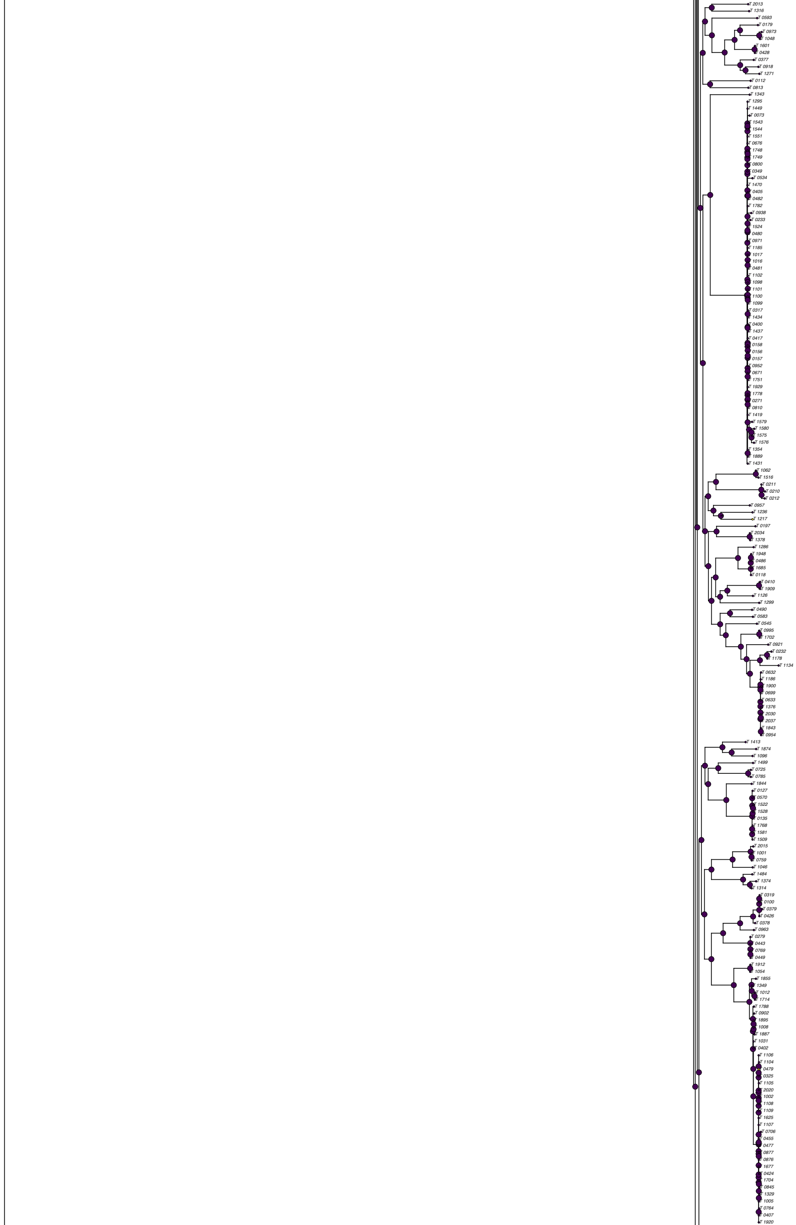
T 2013
T 1316
T 0593
T 0179
T 0973
T 1048
T 1601
T 0428
T 0377
T 0918
T 1271
T 0112
T 0813
T 1343
T 1295
T 1449
T 0073
1543
1544
T 1551
T 0676
1748
1749
0800
0349
T 0534
T 1470
0405
0482
T 1782
T 0938
T 0233
1524
0480
T 0971
T 1185
1017
1016
T 0481
T 1102
1098
1101
1100
T 1099
T 0317
T 1434
T 0400
T 1437
T 0417
0158
0156
0157
T 0952
0671
T 1751
T 1929
1778
0271
T 0810
T 1419
T 1579
T 1580
1575
T 1576
T 1354
T 1889
T 1431
T 1062
T 1516
T 0211
T 0210
T 0212
T 0957
T 1236
T 1217
T 0197
T 2034
T 1378
T 1286
T 1948
0486
1685
T 0118
T 0410
T 1909
T 1126
T 1299
T 0490
T 0583
T 0545
T 0995
T 1702
T 0921
T 0232
T 1178
T 1134
T 0632
T 1186
1900
T 0699
T 0633
1376
2030
2037
T 1843
T 0954
T 1413
T 1874
T 1096
T 1499
T 0725
T 0785
T 1844
T 0127
0570
T 1522
1528
T 0135
T 1768
1581
T 1509
T 2015
T 1001
T 0759
T 1046
T 1484
T 1374
T 1314
T 0319
0100
T 0379
T 0426
T 0378
T 0963
T 0279
0443
0769
T 0449
T 1912
T 1054
T 1855
1349
T 1012
T 1714
T 1788
T 0902
1895
1008
T 1887
T 1031
0402
T 1106
T 1104
0479
0325
T 1105
2020
1002
1108
1109
T 1625
T 1107
T 0706
T 0455
0477
0877
T 0876
1677
0424
1704
T 0845
T 1329
T 1005
T 0764
T 0407
T 1920

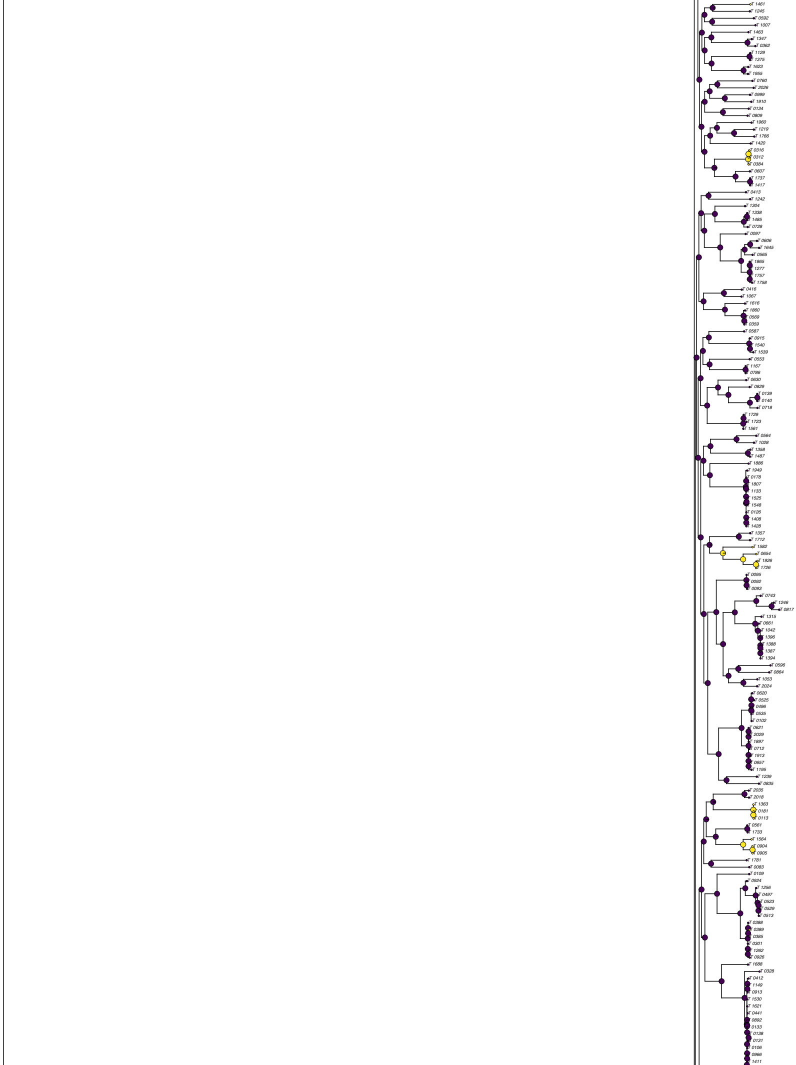
T 1461
T 1245
T 0592
T 1007
T 1463
T 1347
T 0362
T 1129
T 1375
T 1623
T 1955
T 0760
T 2026
T 0999
T 1910
T 0134
T 0809
T 1960
T 1219
T 1766
T 1420
T 0316
0312
T 0384
T 0607
T 1737
T 1417
T 0413
T 1242
T 1304
T 1338
T 1485
T 0728
T 0097
T 0606
T 1645
T 0565
T 1865
1277
1757
T 1758
T 0416
T 1067
T 1616
T 1860
0569
0359
T 0587
T 0915
1540
T 1539
T 0553
T 1167
0786
T 0630
T 0829
T 0139
0140
T 0718
T 1729
T 1723
T 1561
T 0564
T 1028
T 1358
T 1487
T 1886
T 1949
T 0178
1807
1133
1525
1548
T 0126
1408
1428
T 1357
T 1712
T 1582
T 0654
T 1926
1726
T 0095
0092
T 0093
T 0743
T 1246
T 0817
T 1315
T 0661
T 1042
1396
T 1388
1387
T 1394
T 0596
T 0864
T 1053
T 2024
T 0620
T 0525
0496
T 0535
T 0102
T 0621
2029
T 1897
T 0712
T 1913
0657
T 1195
T 1239
T 0835
T 2035
T 2018
T 1363
0181
0113
T 0561
T 1733
T 1564
T 0904
0905
T 1781
T 0083
T 0109
T 0924
T 1256
T 0497
T 0523
T 0529
T 0513
T 0388
T 0389
T 0385
T 0301
T 1262
T 0926
T 1688
T 0328
T 0412
1149
T 0913
T 1530
T 1621
T 0441
0892
0133
T 0138
0131
T 0106
0966
1411

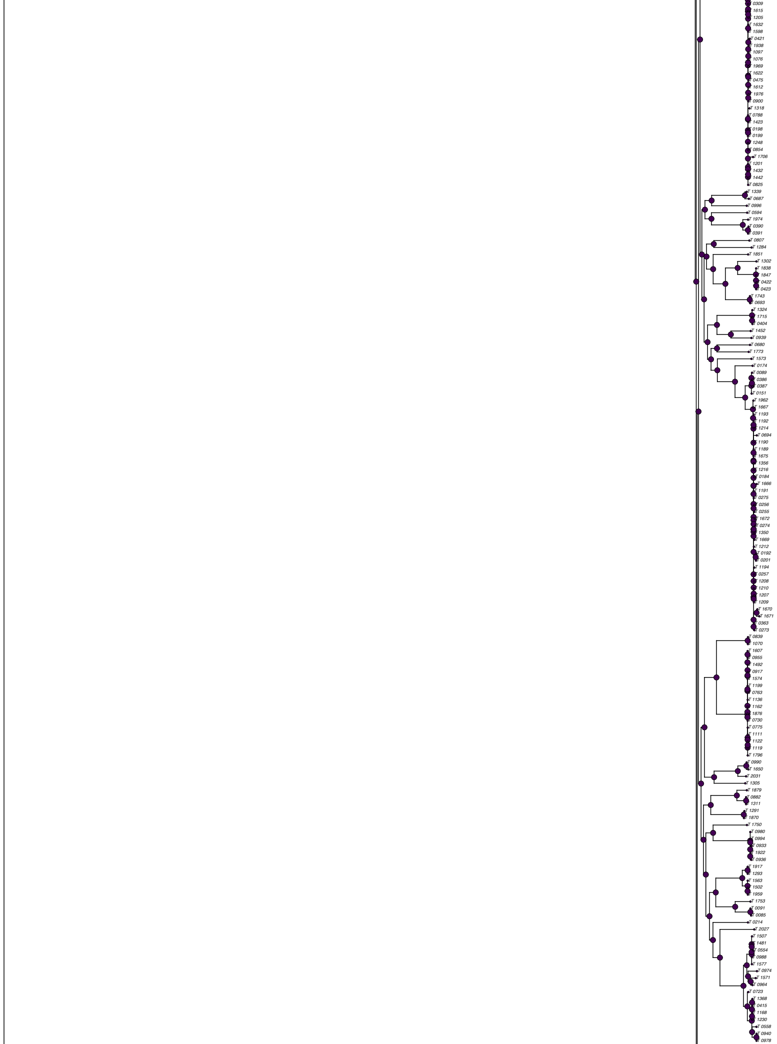
0309
1615
1205
T 1632
T 1598
T 0421
T 1938
T 1097
T 1076
T 1969
T 1622
T 0475
T 1612
T 1976
T 0900
T 1318
T 0788
T 1423
T 0198
T 0199
1248
0854
T 1706
T 1201
1432
1442
T 0825
T 1339
T 0687
T 0996
T 0594
T 1974
T 0390
T 0391
T 0807
T 1284
T 1851
T 1302
T 1838
1847
T 0422
T 0423
T 1743
T 0693
T 1324
1715
T 0404
T 1452
T 0939
T 0680
T 1773
T 1573
T 0174
T 0089
0386
0387
T 0151
T 1962
T 1667
T 1193
T 1192
1214
T 0694
1190
T 1189
T 1675
1356
1216
T 0184
T 1666
T 1191
T 0275
T 0256
T 0255
T 1672
T 0274
T 1350
T 1669
T 1212
T 0192
T 0201
T 1194
T 0257
T 1208
T 1210
T 1207
T 1209
T 1670
T 1671
T 0363
T 0273
T 0839
T 1070
T 1607
T 0955
T 1492
0917
T 1574
T 1199
T 0763
T 1136
1162
1876
T 0730
T 0775
T 1111
1122
1119
T 1796
T 0990
T 1650
T 2031
T 1305
T 1879
T 0882
T 1311
T 1291
T 1870
T 1750
T 0980
T 0994
T 0933
T 1922
T 0936
T 1917
T 1293
T 1563
1502
T 1959
T 1753
T 0091
T 0085
T 0214
T 2027
T 1507
1481
T 0554
T 0988
T 1577
T 0974
T 1571
T 0964
T 0723
T 1368
T 0415
1168
1230
T 0558
T 0940
T 0978

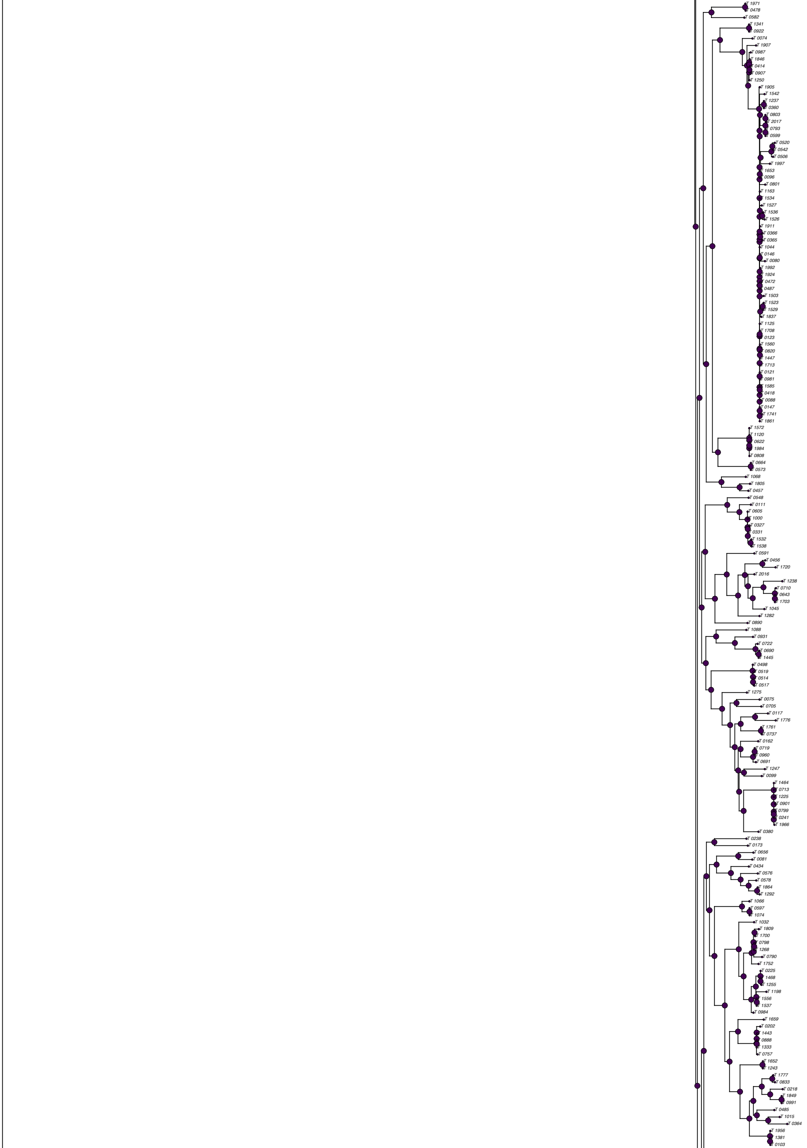
T 1971
T 0478
T 0582
T 1341
T 0922
T 0074
T 1907
T 0987
T 1846
T 0414
T 0907
T 1250
T 1905
T 1542
T 1237
T 0360
T 0803
T 2017
T 0793
T 0599
T 0520
T 0542
T 0506
T 1997
T 1653
T 0096
T 0801
T 1163
T 1534
T 1527
T 1536
T 1526
T 1911
T 0366
T 0365
T 1044
T 0146
T 0080
T 1992
T 1924
T 0472
T 0487
T 1503
T 1523
T 1529
T 1837
T 1125
T 1708
T 0123
T 1560
T 0820
T 1447
T 1713
T 0121
T 0981
T 1585
T 0418
T 0088
T 0147
T 1741
T 1861
T 1572
T 1120
T 0622
T 1984
T 0808
T 0664
T 0573
T 1068
T 1805
T 0457
T 0548
T 0111
T 0605
T 1000
T 0327
T 0331
T 1532
T 1538
T 0591
T 0456
T 1720
T 2016
T 1238
T 0710
T 0643
T 1703
T 1045
T 1282
T 0890
T 1088
T 0931
T 0722
T 0690
T 1445
T 0498
T 0519
T 0514
T 0517
T 1275
T 0075
T 0705
T 0117
T 1776
T 1761
T 0737
T 0162
T 0719
T 0960
T 0691
T 1247
T 0099
T 1464
T 0713
T 1225
T 0901
T 0799
T 0241
T 1966
T 0380
T 0238
T 0173
T 0656
T 0081
T 0434
T 0576
T 0578
T 1864
T 1292
T 1066
T 0597
T 1074
T 1032
T 1809
T 1700
T 0798
T 1268
T 0790
T 1752
T 0225
T 1468
T 1255
T 1198
T 1556
T 1537
T 0984
T 1659
T 0202
T 1443
T 0888
T 1333
T 0757
T 1652
T 1243
T 1777
T 0833
T 0218
T 1849
T 0991
T 0485
T 1015
T 0364
T 1956
T 1381
T 0103

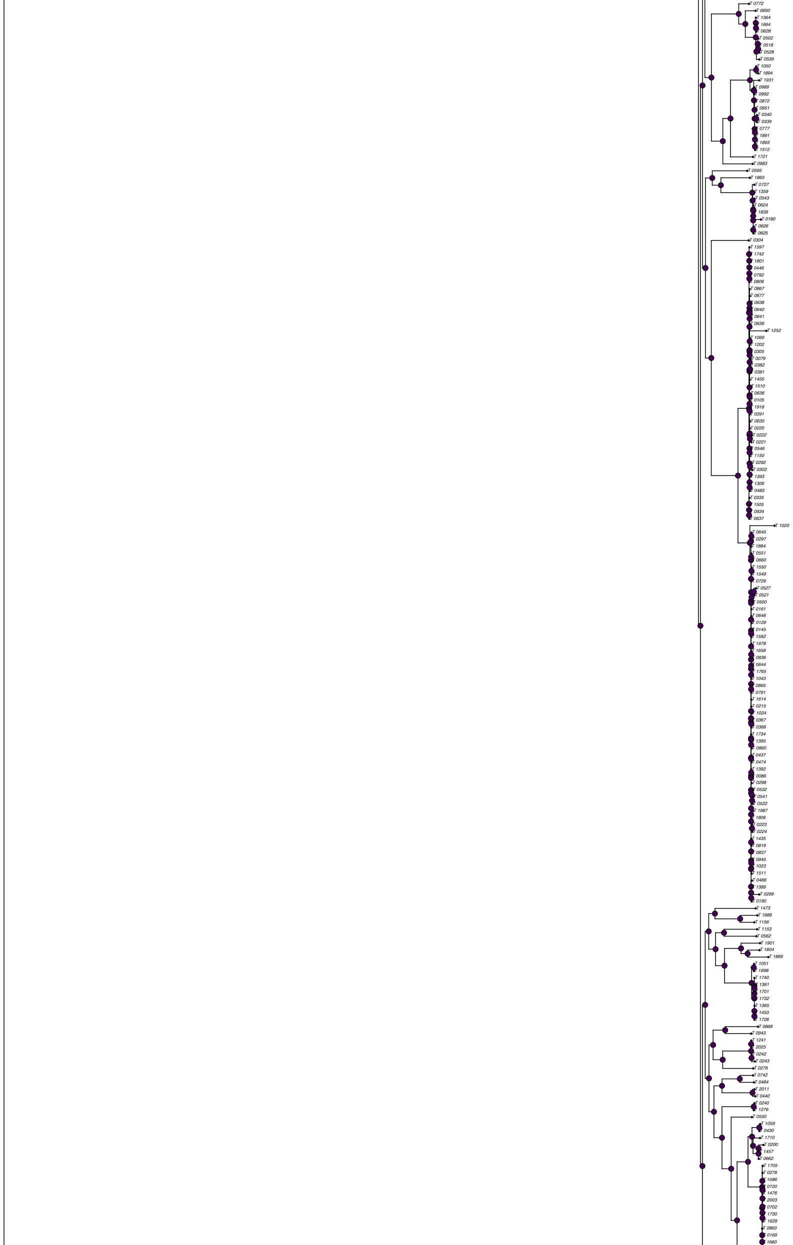
T 0772
T 0650
T 1364
1664
0628
T 0502
0518
T 0528
T 0539
T 1050
T 1894
T 1931
T 0989
0992
0872
0951
T 0340
T 0339
0777
1891
1893
1512
T 1721
T 0983
T 0595
T 1863
T 0727
T 1359
T 0543
T 0624
1839
T 0180
T 0626
T 0625
T 0304
T 1597
1742
1801
0446
0792
0806
T 0867
T 0677
0638
0640
0641
0639
T 1252
T 1069
1202
0305
T 0079
0382
0381
T 1455
1510
0636
0105
1918
T 0291
T 0635
T 0220
T 0222
T 0221
0546
1150
T 0292
T 0302
1393
1306
T 0483
T 0335
1505
0934
T 0637
T 1020
T 0645
0297
1884
T 0551
0660
T 1550
1549
0729
T 0527
T 0521
0500
T 0161
0646
0129
0145
1592
1978
1658
0836
0644
1765
1043
0865
0791
T 1614
T 0215
1024
0367
T 0368
T 1734
1395
T 0860
0437
T 0474
1392
0086
T 0298
0532
0541
0522
T 1987
1806
0223
0224
1435
0819
0837
0945
1023
T 1511
T 0466
1389
T 0299
T 0190
T 1473
T 1689
T 1156
T 1153
T 0562
T 1901
T 1804
T 1869
T 1051
1998
T 1740
1361
1701
1732
T 1365
1453
T 1728
T 0868
T 0943
T 1241
2023
0242
T 0243
T 0276
T 0742
T 0484
T 2011
T 0440
T 0240
1276
T 0550
T 1059
0430
T 1710
T 0200
1457
T 0662
T 1705
T 0278
1586
0720
1476
2003
0702
1730
1629
T 0863
0169
1660

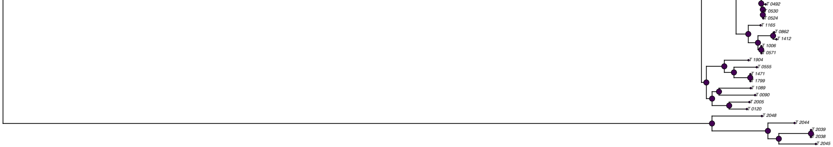

T 0492
T 0530
T 0524
T 1165
T 0862
T 1412
T 1006
T 0571
T 1904
T 0555
T 1471
T 1799
T 1089
T 0090
T 2005
T 0120
T 2048
T 2044
T 2039
T 2038
T 2045

Effector gene: vgrG2b
Locus 1

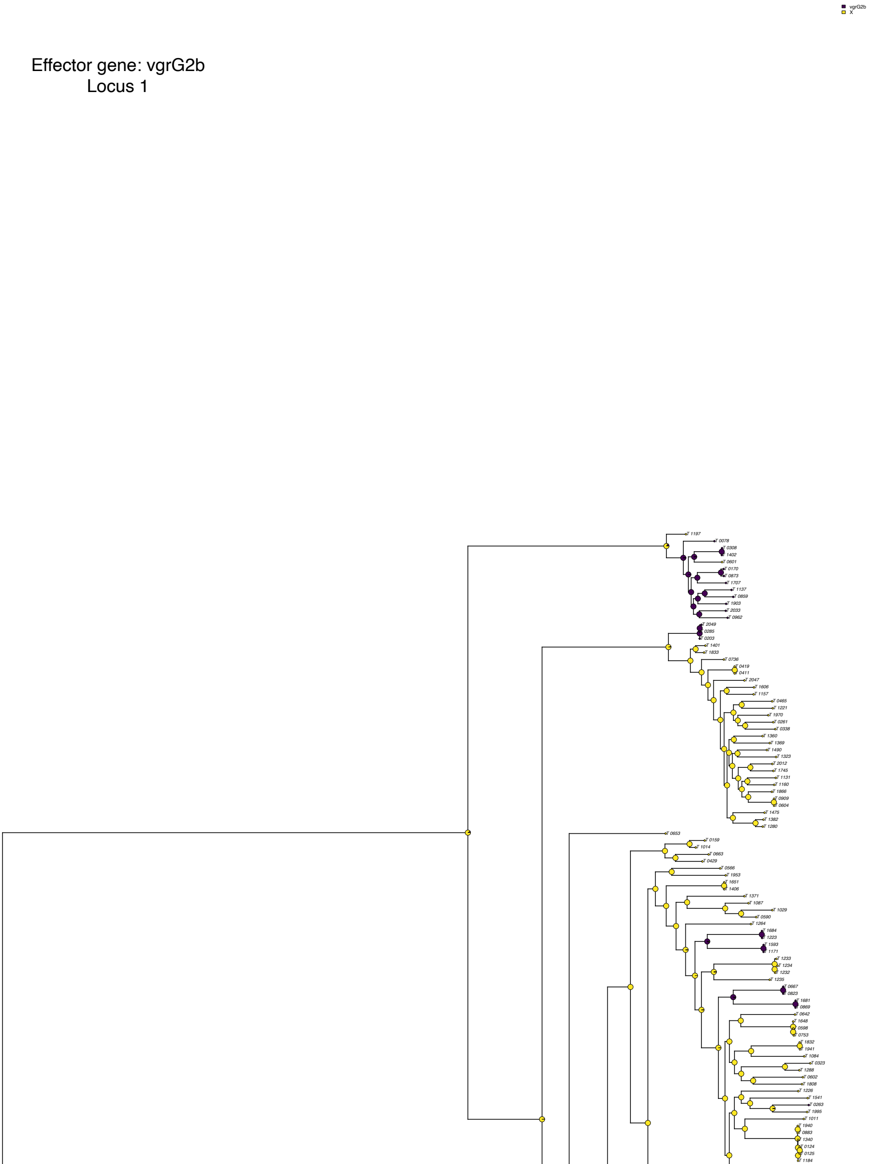

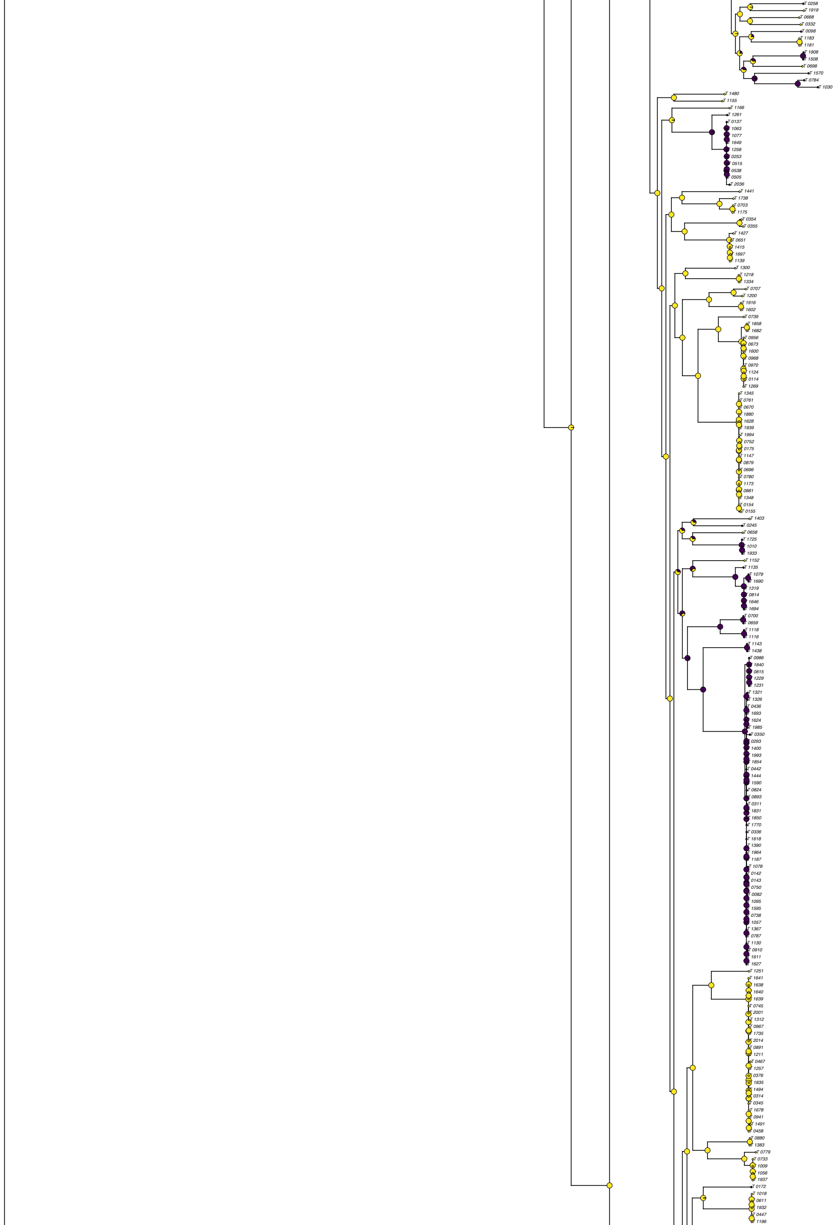

T 0258
T 1919
T 0668
T 0332
T 0098
T 1183
1181
T 1908
T 1508
T 0698
T 1570
T 0784
T 1030
T 1480
T 1155
T 1166
T 1261
T 0137
1063
1077
1649
1258
0253
0515
0538
0505
T 2036
T 1441
T 1738
T 0703
1175
T 0354
T 0355
T 1427
T 0651
1415
1697
1139
T 1300
T 1218
1334
T 0707
T 1200
T 1916
1602
T 0739
T 1858
1682
T 0956
0673
1600
0968
T 0970
1124
0114
T 1269
T 1345
T 0761
0670
1880
1628
1939
T 1994
0752
0175
T 1147
0879
T 0696
T 0780
1173
0881
1348
0154
T 0155
T 1403
T 0245
T 0658
T 1725
1010
1933
T 1152
T 1135
T 1079
T 1690
1319
0814
1646
1694
T 0700
T 0659
T 1118
1116
T 1143
1438
T 0986
1840
0815
1229
1231
T 1321
T 1326
T 0436
1693
1624
1985
T 0350
0293
1400
1993
1854
T 0442
1444
1590
T 0824
0893
T 0311
1831
1850
T 1770
T 0336
T 1618
T 1390
1964
1187
T 1078
0142
0143
0750
0082
1095
1595
0738
1057
T 1367
0787
T 1130
0910
1611
1627
T 1251
T 1641
1638
1640
1639
T 0745
2001
T 1312
0967
1735
2014
T 0891
1211
T 0467
1257
0376
1835
1494
0314
0345
T 1678
0941
T 1491
0458
T 0880
1383
T 0779
T 0733
1009
1056
1937
T 0172
T 1018
0611
1932
T 0447
1196

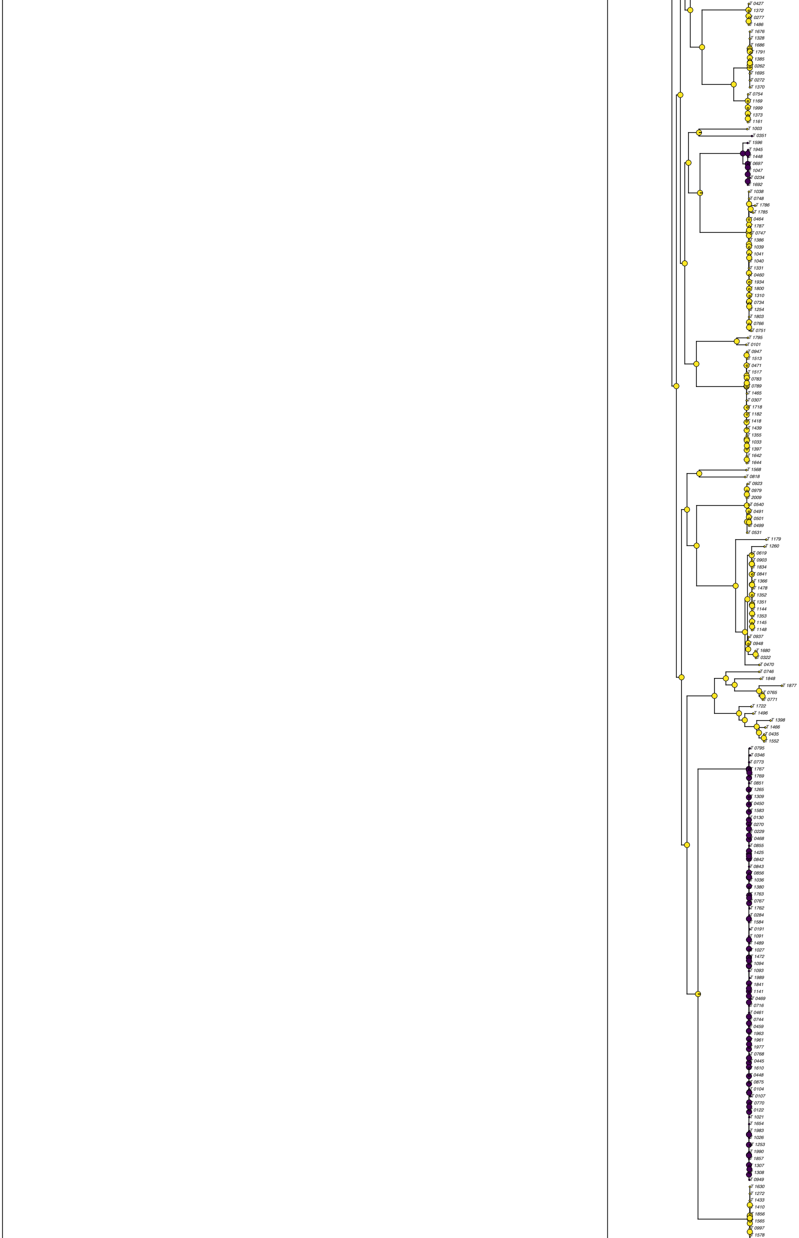
T 0427
T 1372
T 0277
T 1486
T 1676
T 1328
T 1686
T 1791
T 1385
T 0262
T 1695
T 0272
T 1370
T 0754
T 1169
T 1999
T 1373
T 1161
T 1003
T 0351
T 1596
T 1945
T 1448
T 0697
T 1047
T 0234
T 1692
T 1038
T 0748
T 1786
T 1785
T 0464
T 1787
T 0747
T 1386
T 1039
T 1041
T 1040
T 1331
T 0460
T 1934
T 1800
T 1310
T 0734
T 1254
T 1803
T 0766
T 0751
T 1795
T 0101
T 0947
T 1513
T 0471
T 1517
T 0783
T 0789
T 1465
T 0307
T 1718
T 1182
T 1418
T 1439
T 1355
T 1033
T 1397
T 1642
T 1644
T 1568
T 0818
T 0923
T 0979
T 2009
T 0540
T 0491
T 0501
T 0499
T 0531
T 1179
T 1260
T 0619
T 0903
T 1834
T 0841
T 1366
T 1478
T 1352
T 1351
T 1144
T 1353
T 1145
T 1148
T 0937
T 0948
T 1680
T 0322
T 0470
T 0746
T 1848
T 1877
T 0765
T 0771
T 1722
T 1496
T 1398
T 1466
T 0435
T 1552
T 0795
T 0346
T 0773
T 1767
T 1769
T 0851
T 1265
T 1309
T 0450
T 1583
T 0130
T 0270
T 0229
T 0468
T 0855
T 1425
T 0842
T 0843
T 0856
T 1036
T 1380
T 1763
T 0767
T 1762
T 0284
T 1584
T 0191
T 1091
T 1489
T 1027
T 1472
T 1094
T 1093
T 1989
T 1841
T 1141
T 0469
T 0716
T 0461
T 0744
T 0459
T 1963
T 1961
T 1977
T 0768
T 0445
T 1610
T 0448
T 0875
T 0104
T 0107
T 0770
T 0122
T 1021
T 1654
T 1983
T 1026
T 1253
T 1990
T 1857
T 1307
T 1308
T 0949
T 1630
T 1272
T 1433
T 1410
T 1856
T 1565
T 0997
T 1578

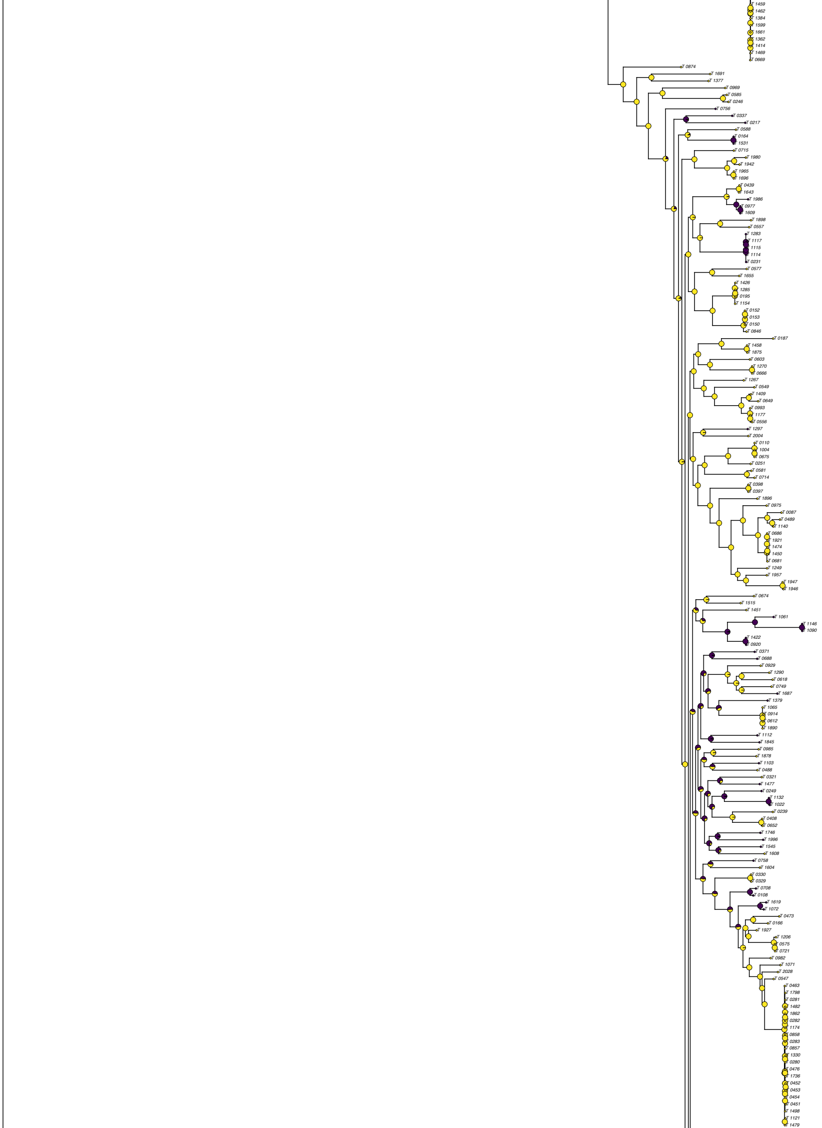
T 1459
T 1462
T 1384
T 1599
T 1661
T 1362
T 1414
T 1469
T 0669
T 0874
T 1691
T 1377
T 0969
T 0585
T 0246
T 0756
T 0337
T 0217
T 0588
T 0164
T 1531
T 0715
T 1980
T 1942
T 1965
T 1696
T 0439
T 1643
T 1986
T 0977
T 1609
T 1898
T 0557
T 1283
T 1117
T 1115
T 1114
T 0231
T 0577
T 1655
T 1426
T 1285
T 0195
T 1154
T 0152
T 0153
T 0150
T 0846
T 0187
T 1458
T 1875
T 0603
T 1270
T 0666
T 1267
T 0549
T 1409
T 0649
T 0993
T 1177
T 0556
T 1297
T 2004
T 0110
T 1004
T 0675
T 0251
T 0581
T 0714
T 0398
T 0397
T 1896
T 0975
T 0087
T 0489
T 1140
T 0686
T 1921
T 1474
T 1450
T 0681
T 1249
T 1957
T 1947
T 1946
T 0674
T 1515
T 1451
T 1061
T 1146
T 1090
T 1422
T 0920
T 0371
T 0688
T 0929
T 1290
T 0618
T 0749
T 1687
T 1379
T 1065
T 0914
T 0612
T 1890
T 1112
T 1845
T 0985
T 1878
T 1103
T 0488
T 0321
T 1477
T 0249
T 1132
T 1022
T 0239
T 0408
T 0652
T 1746
T 1996
T 1545
T 1608
T 0758
T 1604
T 0330
T 0329
T 0708
T 0108
T 1619
T 1072
T 0473
T 0166
T 1927
T 1206
T 0575
T 0721
T 0982
T 1071
T 2028
T 0547
T 0463
T 1798
T 0281
T 1482
T 1862
T 0282
T 1174
T 0858
T 0283
T 0857
T 1330
T 0280
T 0476
T 1736
T 0452
T 0453
T 0454
T 0451
T 1498
T 1121
T 1479

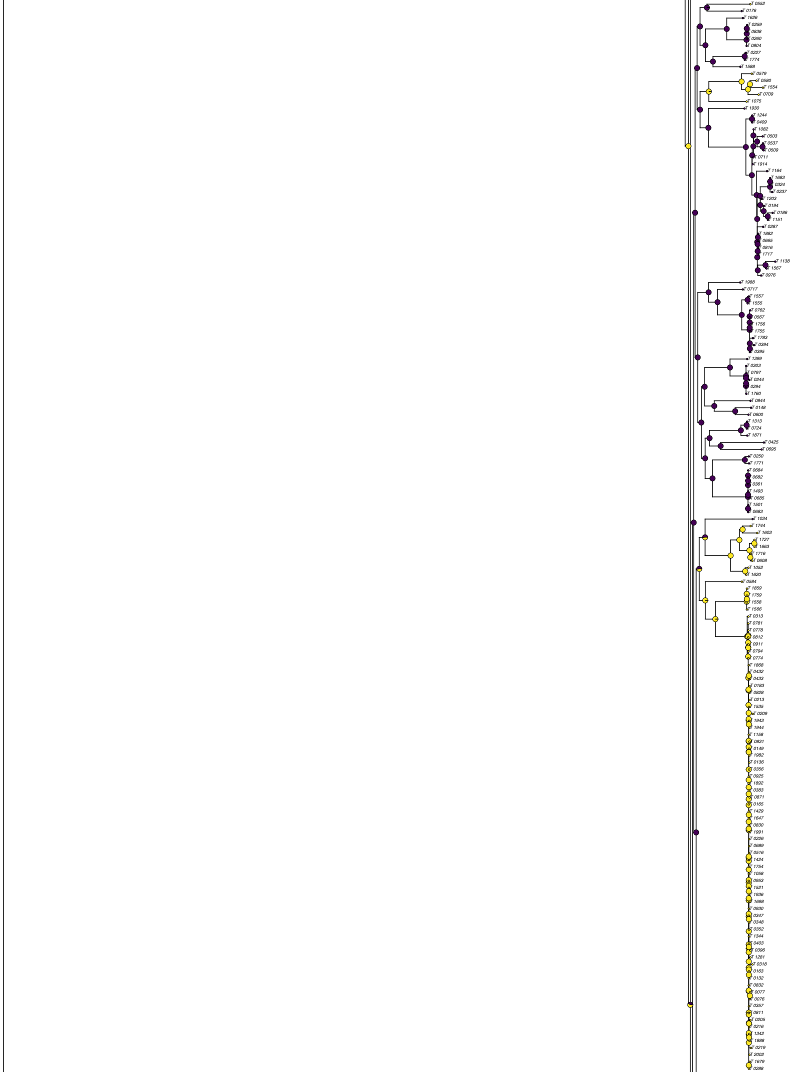
T 0552
T 0176
T 1626
T 0259
0838
0260
T 0804
T 0227
T 1774
T 1588
T 0579
T 0580
T 1554
T 0709
T 1075
T 1930
T 1244
T 0409
T 1082
T 0503
T 0537
T 0509
T 0711
T 1914
T 1164
T 1683
0324
T 0237
T 1203
T 0194
T 0186
T 1151
T 0287
T 1882
0665
T 0816
1717
T 1138
T 1567
T 0976
T 1988
T 0717
T 1557
T 1555
T 0762
0567
T 1756
T 1755
T 1783
T 0394
T 0395
T 1399
T 0303
T 0797
T 0244
0294
T 1760
T 0844
T 0148
T 0600
T 1313
T 0724
T 1871
T 0425
T 0695
T 0250
T 1771
T 0684
0682
0361
T 1493
T 0685
T 1501
T 0683
T 1034
T 1744
T 1603
T 1727
T 1663
T 1716
T 0608
T 1052
T 1620
T 0584
T 1859
1759
1558
T 1566
T 0313
T 0781
T 0778
0812
0911
T 0794
0774
T 1868
T 0432
0433
T 0183
T 0828
T 0213
1535
T 0209
1943
T 1944
T 1158
0831
0149
T 1982
T 0136
0356
T 0925
1892
0383
T 0871
0165
T 1429
T 1647
T 0830
T 1991
T 0226
T 0689
T 0516
1424
T 1754
T 1058
0953
1521
T 1936
T 1698
T 0930
0347
T 0348
0352
T 1344
T 0403
T 0396
T 1281
T 0318
0163
T 0132
T 0832
T 0077
T 0076
T 0357
0811
T 0205
T 0216
1342
T 1888
T 0219
T 2002
T 1679
T 0288

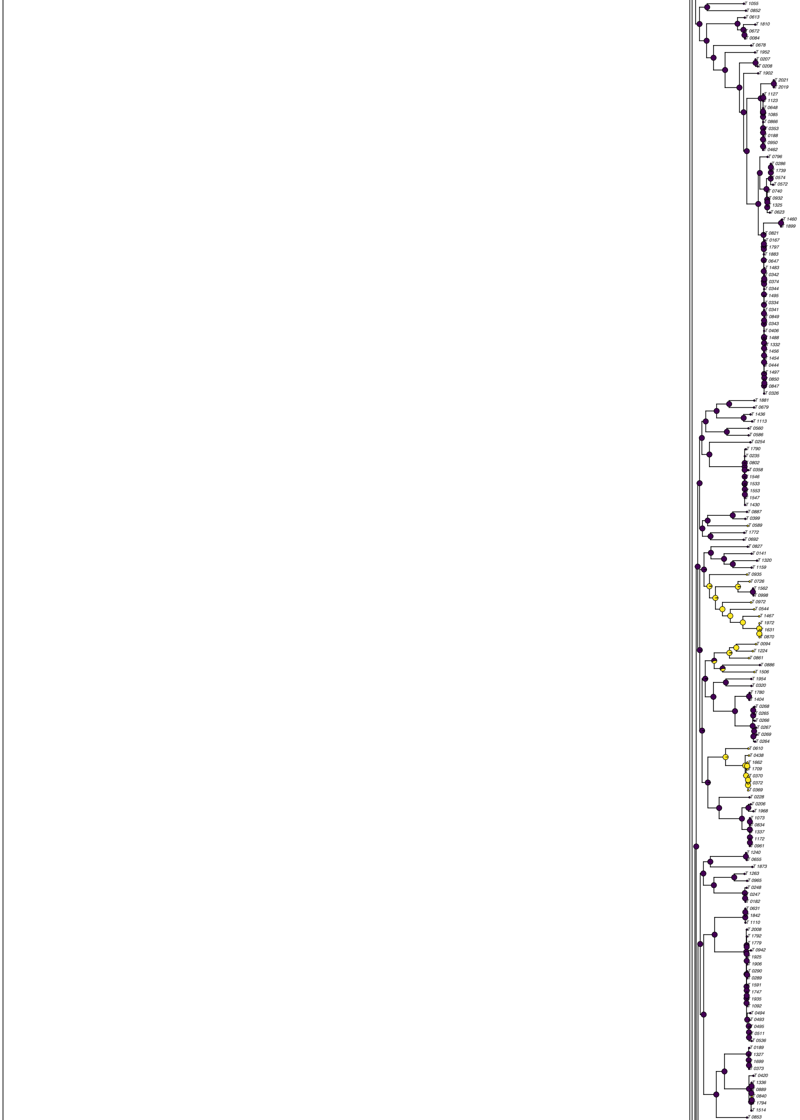

T 1055
T 0852
T 0613
T 1810
T 0672
T 0084
T 0678
T 1952
T 0207
T 0208
T 1902
T 2021
T 2019
T 1127
T 1123
T 0648
T 1085
T 0866
T 0353
T 0188
T 0950
T 0462
T 0796
T 0286
T 1739
T 0574
T 0572
T 0740
T 0932
T 1325
T 0623
T 1460
T 1899
T 0821
T 0167
T 1797
T 1883
T 0647
T 1483
T 0342
T 0374
T 0344
T 1495
T 0334
T 0341
T 0849
T 0343
T 0406
T 1488
T 1332
T 1456
T 1454
T 0444
T 1497
T 0850
T 0847
T 0326
T 1881
T 0679
T 1436
T 1113
T 0560
T 0586
T 0254
T 1790
T 0235
T 0802
T 0358
T 1546
T 1533
T 1553
T 1547
T 1430
T 0887
T 0399
T 0589
T 1772
T 0692
T 0827
T 0141
T 1320
T 1159
T 0935
T 0726
T 1562
T 0998
T 0972
T 0544
T 1467
T 1972
T 1631
T 0870
T 0094
T 1224
T 0861
T 0886
T 1506
T 1954
T 0320
T 1780
T 1404
T 0268
T 0265
T 0266
T 0267
T 0269
T 0264
T 0610
T 0438
T 1662
T 1709
T 0370
T 0372
T 0369
T 0228
T 0206
T 1968
T 1073
T 0834
T 1337
T 1172
T 0961
T 1240
T 0655
T 1873
T 1263
T 0965
T 0248
T 0247
T 0182
T 0631
T 1842
T 1110
T 2008
T 1792
T 1779
T 0942
T 1925
T 1906
T 0290
T 0289
T 1591
T 1747
T 1935
T 1092
T 0494
T 0493
T 0495
T 0511
T 0536
T 0189
T 1327
T 1699
T 0373
T 0420
T 1336
T 0889
T 0840
T 1794
T 1514
T 0853

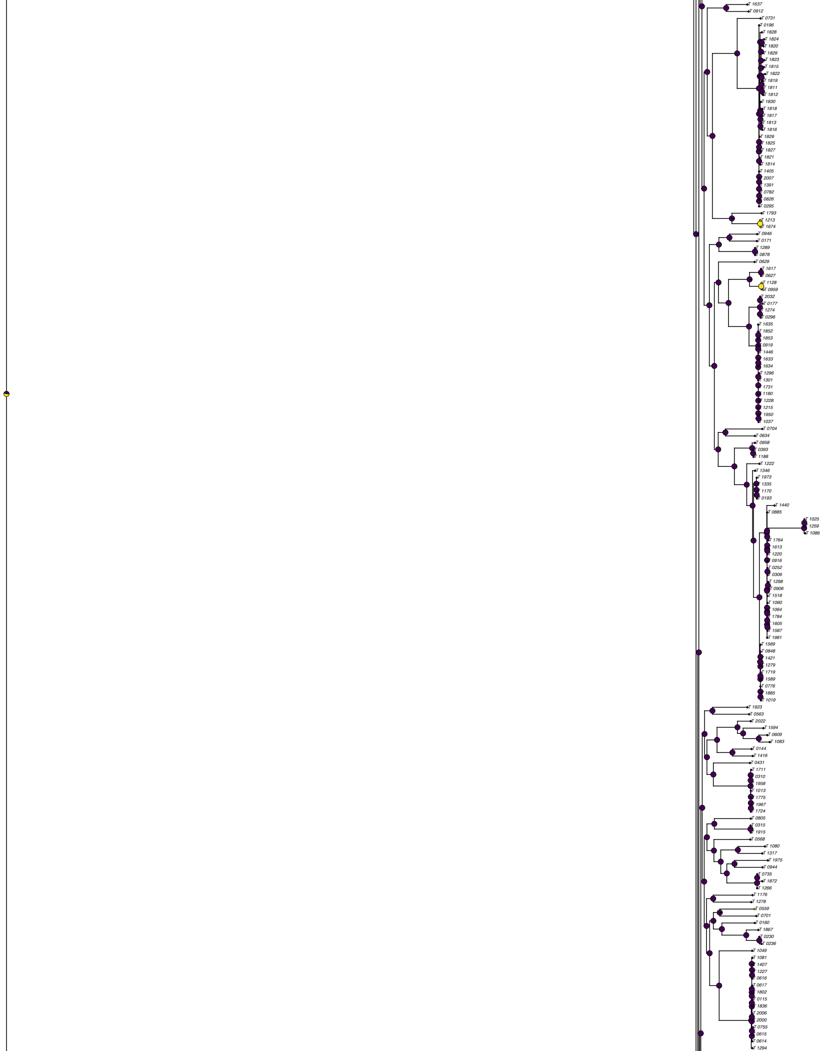
T 1637
T 0912
T 0731
T 0196
T 1828
T 1824
T 1820
T 1829
T 1823
T 1815
T 1822
T 1819
T 1811
T 1812
T 1830
T 1818
T 1817
T 1813
T 1816
T 1826
T 1825
T 1827
T 1821
T 1814
T 1405
T 2007
T 1391
T 0782
T 0826
T 0295
T 1793
T 1213
T 1674
T 0946
T 0171
T 1289
T 0878
T 0629
T 1617
T 0627
T 1128
T 0959
T 2032
T 0177
T 1274
T 0296
T 1635
T 1852
T 1853
T 0919
T 1446
T 1633
T 1634
T 1296
T 1301
T 1731
T 1180
T 1228
T 1215
T 1950
T 1037
T 0704
T 0634
T 0958
T 0393
T 1188
T 1222
T 1346
T 1973
T 1335
T 1170
T 0193
T 1440
T 0885
T 1025
T 1259
T 1086
T 1764
T 1613
T 1220
T 0916
T 0252
T 0306
T 1298
T 0906
T 1518
T 1060
T 1064
T 1784
T 1605
T 1587
T 1981
T 1569
T 0848
T 1421
T 1279
T 1719
T 1589
T 0776
T 1885
T 1019
T 1923
T 0563
T 2022
T 1594
T 0609
T 1083
T 0144
T 1416
T 0431
T 1711
T 0310
T 1958
T 1013
T 1775
T 1967
T 1724
T 0805
T 0315
T 1915
T 0568
T 1080
T 1317
T 1975
T 0944
T 0735
T 1872
T 1266
T 1176
T 1278
T 0559
T 0701
T 0160
T 1867
T 0230
T 0236
T 1049
T 1081
T 1407
T 1227
T 0616
T 0617
T 1802
T 0115
T 1836
T 2006
T 2000
T 0755
T 0615
T 0614
T 1294

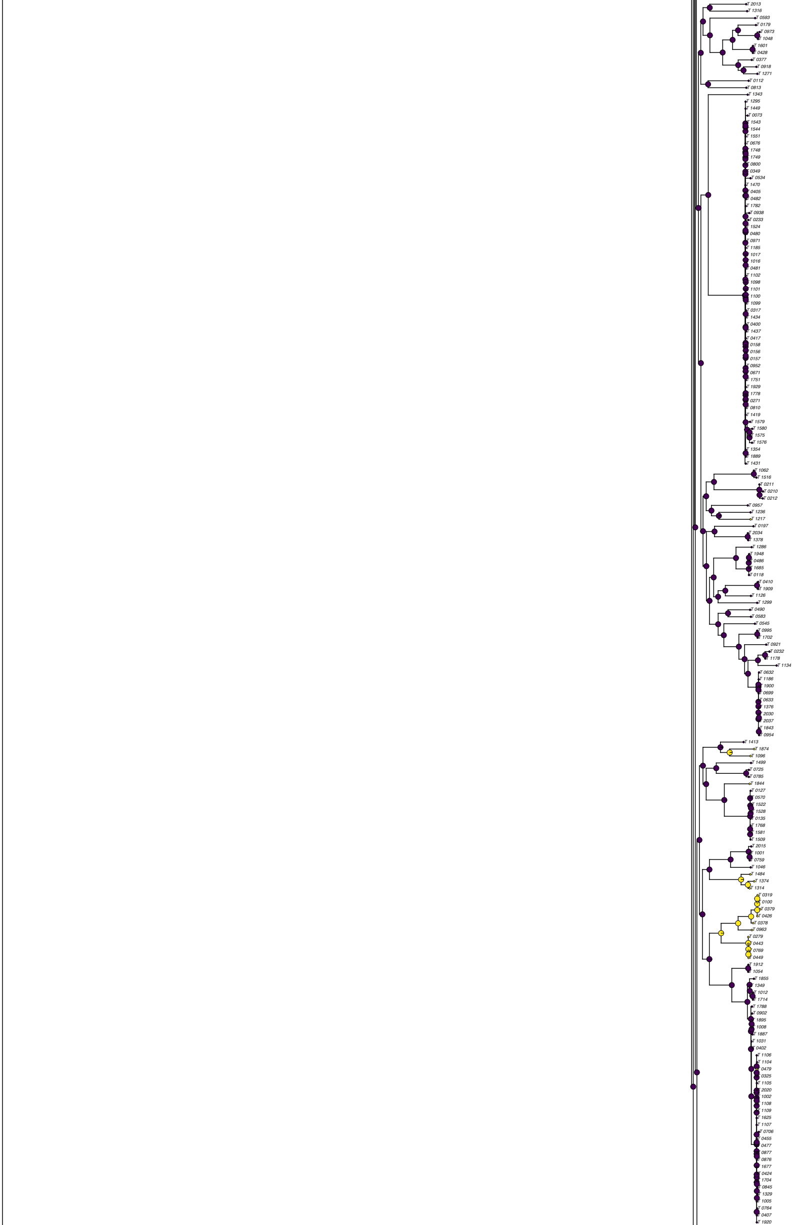
T 2013
T 1316
T 0593
T 0179
T 0973
T 1048
T 1601
T 0428
T 0377
T 0918
T 1271
T 0112
T 0813
T 1343
T 1295
T 1449
T 0073
T 1543
T 1544
T 1551
T 0676
T 1748
T 1749
T 0800
T 0349
T 0534
T 1470
T 0405
T 0482
T 1782
T 0938
T 0233
T 1524
T 0480
T 0971
T 1185
T 1017
T 1016
T 0481
T 1102
T 1098
T 1101
T 1100
T 1099
T 0317
T 1434
T 0400
T 1437
T 0417
T 0158
T 0156
T 0157
T 0952
T 0671
T 1751
T 1929
T 1778
T 0271
T 0810
T 1419
T 1579
T 1580
T 1575
T 1576
T 1354
T 1889
T 1431
T 1062
T 1516
T 0211
T 0210
T 0212
T 0957
T 1236
T 1217
T 0197
T 2034
T 1378
T 1286
T 1948
T 0486
T 1685
T 0118
T 0410
T 1909
T 1126
T 1299
T 0490
T 0583
T 0545
T 0995
T 1702
T 0921
T 0232
T 1178
T 1134
T 0632
T 1186
T 1900
T 0699
T 0633
T 1376
T 2030
T 2037
T 1843
T 0954
T 1413
T 1874
T 1096
T 1499
T 0725
T 0785
T 1844
T 0127
T 0570
T 1522
T 1528
T 0135
T 1768
T 1581
T 1509
T 2015
T 1001
T 0759
T 1046
T 1484
T 1374
T 1314
T 0319
T 0100
T 0379
T 0426
T 0378
T 0963
T 0279
T 0443
T 0769
T 0449
T 1912
T 1054
T 1855
T 1349
T 1012
T 1714
T 1788
T 0902
T 1895
T 1008
T 1887
T 1031
T 0402
T 1106
T 1104
T 0479
T 0325
T 1105
T 2020
T 1002
T 1108
T 1109
T 1625
T 1107
T 0706
T 0455
T 0477
T 0877
T 0876
T 1677
T 0424
T 1704
T 0845
T 1329
T 1005
T 0764
T 0407
T 1920

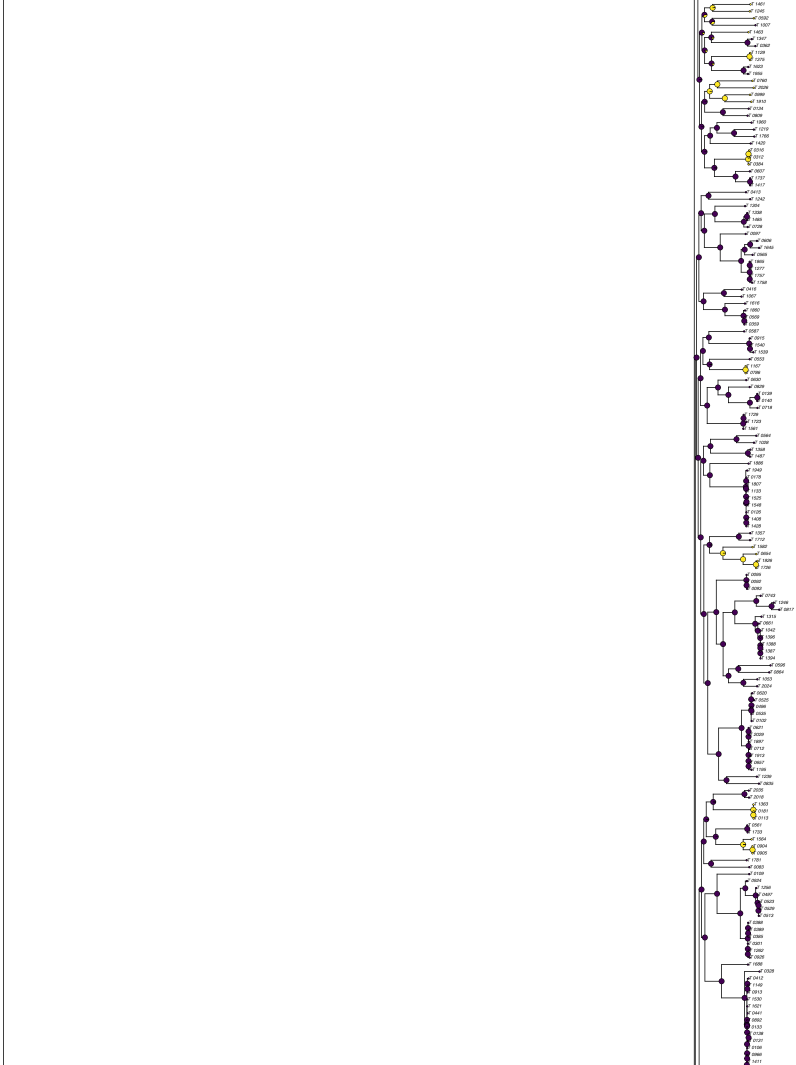
T 1461
T 1245
T 0592
T 1007
T 1463
T 1347
T 0362
T 1129
T 1375
T 1623
T 1955
T 0760
T 2026
T 0999
T 1910
T 0134
T 0809
T 1960
T 1219
T 1766
T 1420
T 0316
T 0312
T 0384
T 0607
T 1737
T 1417
T 0413
T 1242
T 1304
T 1338
T 1485
T 0728
T 0097
T 0606
T 1645
T 0565
T 1865
T 1277
T 1757
T 1758
T 0416
T 1067
T 1616
T 1860
T 0569
T 0359
T 0587
T 0915
T 1540
T 1539
T 0553
T 1167
T 0786
T 0630
T 0829
T 0139
T 0140
T 0718
T 1729
T 1723
T 1561
T 0564
T 1028
T 1358
T 1487
T 1886
T 1949
T 0178
T 1807
T 1133
T 1525
T 1548
T 0126
T 1408
T 1428
T 1357
T 1712
T 1582
T 0654
T 1926
T 1726
T 0095
T 0092
T 0093
T 0743
T 1246
T 0817
T 1315
T 0661
T 1042
T 1396
T 1388
T 1387
T 1394
T 0596
T 0864
T 1053
T 2024
T 0620
T 0525
T 0496
T 0535
T 0102
T 0621
T 2029
T 1897
T 0712
T 1913
T 0657
T 1195
T 1239
T 0835
T 2035
T 2018
T 1363
T 0181
T 0113
T 0561
T 1733
T 1564
T 0904
T 0905
T 1781
T 0083
T 0109
T 0924
T 1256
T 0497
T 0523
T 0529
T 0513
T 0388
T 0389
T 0385
T 0301
T 1262
T 0926
T 1688
T 0328
T 0412
T 1149
T 0913
T 1530
T 1621
T 0441
T 0892
T 0133
T 0138
T 0131
T 0106
T 0966
T 1411

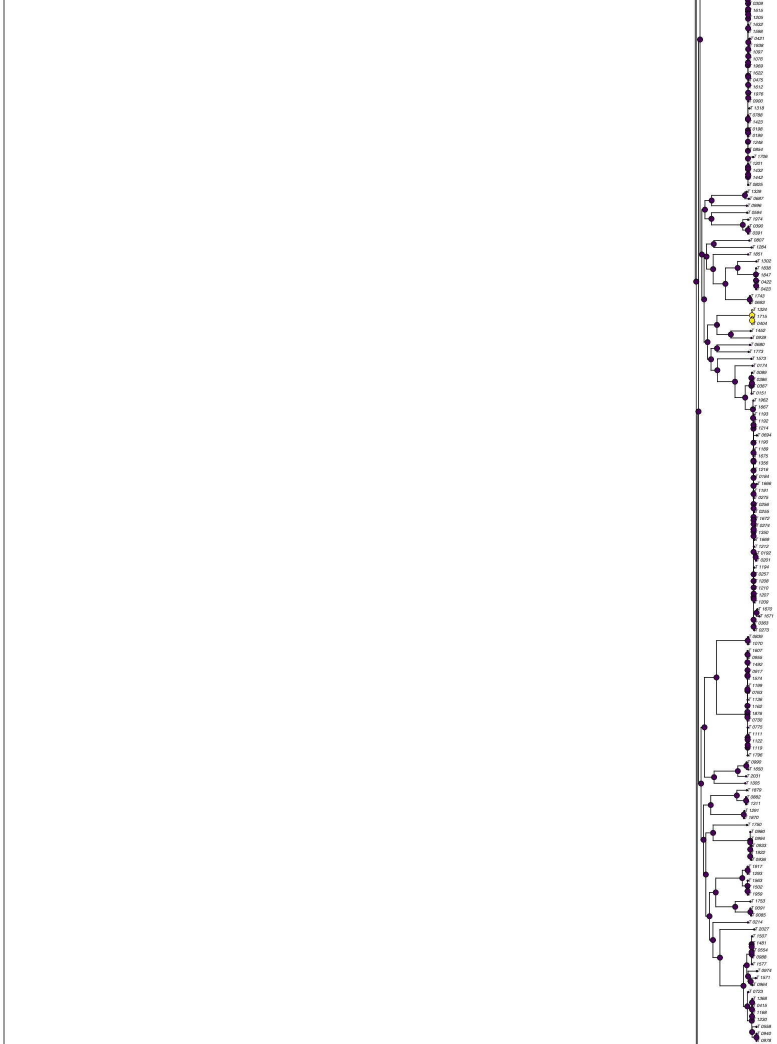

0309
1615
1205
T 1632
T 1598
T 0421
T 1938
T 1097
T 1076
T 1969
T 1622
T 0475
T 1612
T 1976
T 0900
T 1318
T 0788
T 1423
T 0198
T 0199
T 1248
T 0854
T 1706
T 1201
T 1432
T 1442
T 0825
T 1339
T 0687
T 0996
T 0594
T 1974
T 0390
T 0391
T 0807
T 1284
T 1851
T 1302
T 1838
T 1847
T 0422
T 0423
T 1743
T 0693
T 1324
T 1715
T 0404
T 1452
T 0939
T 0680
T 1773
T 1573
T 0174
T 0089
T 0386
T 0387
T 0151
T 1962
T 1667
T 1193
T 1192
T 1214
T 0694
T 1190
T 1189
T 1675
T 1356
T 1216
T 0184
T 1666
T 1191
T 0275
T 0256
T 0255
T 1672
T 0274
T 1350
T 1669
T 1212
T 0192
T 0201
T 1194
T 0257
T 1208
T 1210
T 1207
T 1209
T 1670
T 1671
T 0363
T 0273
T 0839
T 1070
T 1607
T 0955
T 1492
T 0917
T 1574
T 1199
T 0763
T 1136
T 1162
T 1876
T 0730
T 0775
T 1111
T 1122
T 1119
T 1796
T 0990
T 1650
T 2031
T 1305
T 1879
T 0882
T 1311
T 1291
T 1870
T 1750
T 0980
T 0994
T 0933
T 1922
T 0936
T 1917
T 1293
T 1563
T 1502
T 1959
T 1753
T 0091
T 0085
T 0214
T 2027
T 1507
T 1481
T 0554
T 0988
T 1577
T 0974
T 1571
T 0964
T 0723
T 1368
T 0415
T 1168
T 1230
T 0558
T 0940
T 0978

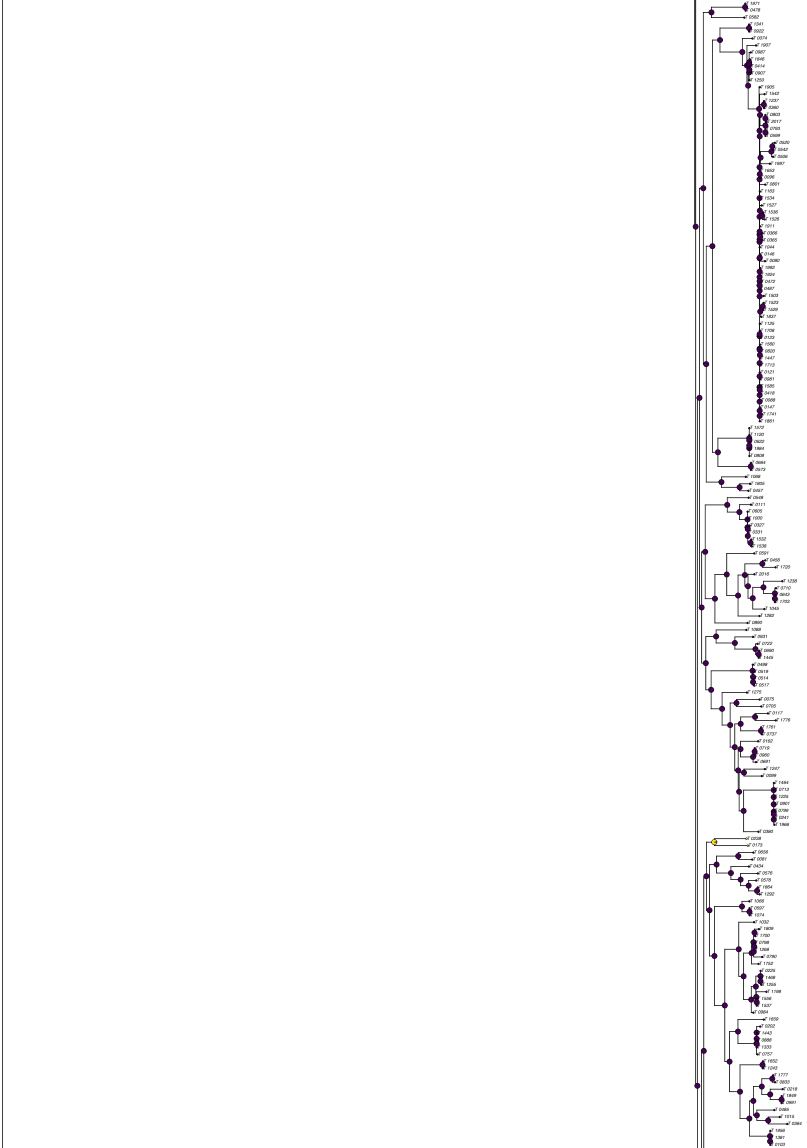
T 1971
T 0478
T 0582
T 1341
T 0922
T 0074
T 1907
T 0987
T 1846
T 0414
T 0907
T 1250
T 1905
T 1542
T 1237
T 0360
T 0803
T 2017
T 0793
T 0599
T 0520
T 0542
T 0506
T 1997
T 1653
T 0096
T 0801
T 1163
T 1534
T 1527
T 1536
T 1526
T 1911
T 0366
T 0365
T 1044
T 0146
T 0080
T 1992
T 1924
T 0472
T 0487
T 1503
T 1523
T 1529
T 1837
T 1125
T 1708
T 0123
T 1560
T 0820
T 1447
T 1713
T 0121
T 0981
T 1585
T 0418
T 0088
T 0147
T 1741
T 1861
T 1572
T 1120
T 0622
T 1984
T 0808
T 0664
T 0573
T 1068
T 1805
T 0457
T 0548
T 0111
T 0605
T 1000
T 0327
T 0331
T 1532
T 1538
T 0591
T 0456
T 1720
T 2016
T 1238
T 0710
T 0643
T 1703
T 1045
T 1282
T 0890
T 1088
T 0931
T 0722
T 0690
T 1445
T 0498
T 0519
T 0514
T 0517
T 1275
T 0075
T 0705
T 0117
T 1776
T 1761
T 0737
T 0162
T 0719
T 0960
T 0691
T 1247
T 0099
T 1464
T 0713
T 1225
T 0901
T 0799
T 0241
T 1966
T 0380
T 0238
T 0173
T 0656
T 0081
T 0434
T 0576
T 0578
T 1864
T 1292
T 1066
T 0597
T 1074
T 1032
T 1809
T 1700
T 0798
T 1268
T 0790
T 1752
T 0225
T 1468
T 1255
T 1198
T 1556
T 1537
T 0984
T 1659
T 0202
T 1443
T 0888
T 1333
T 0757
T 1652
T 1243
T 1777
T 0833
T 0218
T 1849
T 0991
T 0485
T 1015
T 0364
T 1956
T 1381
T 0103

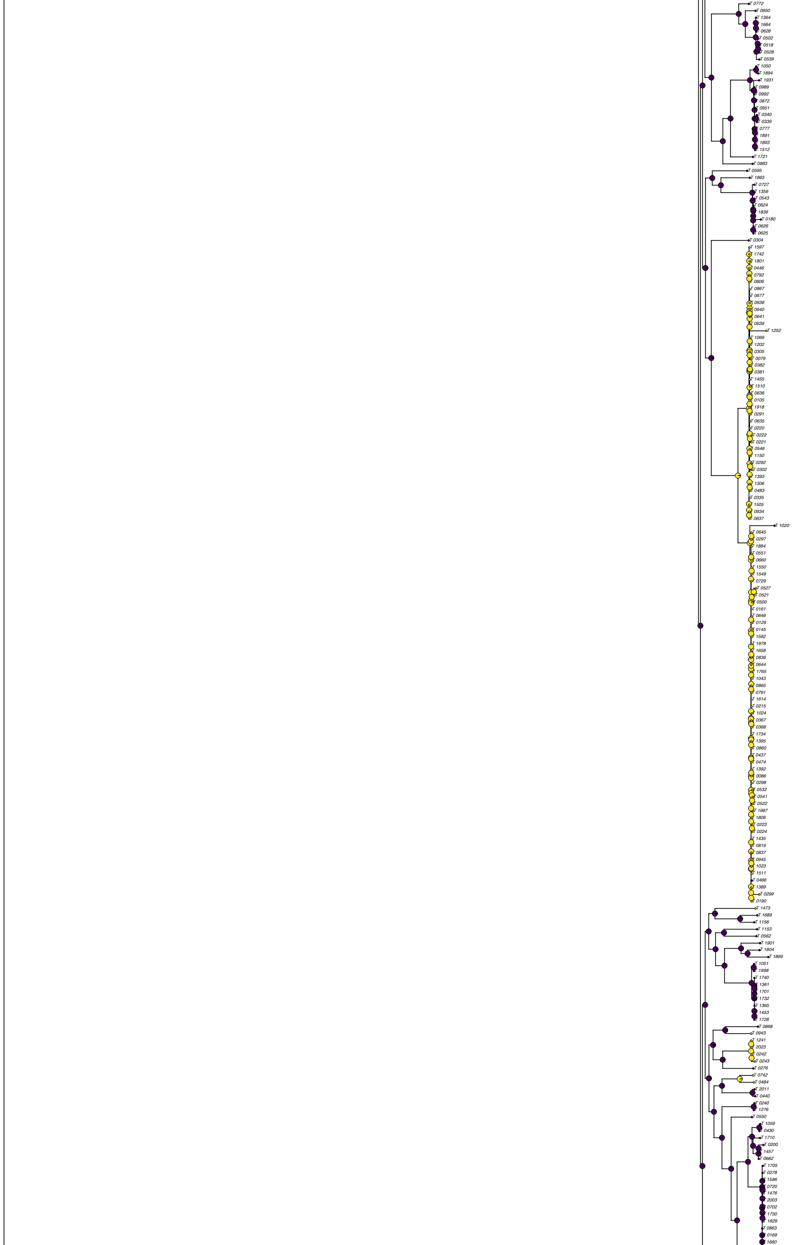
T 0772
T 0650
T 1364
T 1664
T 0628
T 0502
T 0518
T 0528
T 0539
T 1050
T 1894
T 1931
T 0989
T 0992
T 0872
T 0951
T 0340
T 0339
T 0777
T 1891
T 1893
T 1512
T 1721
T 0983
T 0595
T 1863
T 0727
T 1359
T 0543
T 0624
T 1839
T 0180
T 0626
T 0625
T 0304
T 1597
T 1742
T 1801
T 0446
T 0792
T 0806
T 0867
T 0677
T 0638
T 0640
T 0641
T 0639
T 1252
T 1069
T 1202
T 0305
T 0079
T 0382
T 0381
T 1455
T 1510
T 0636
T 0105
T 1918
T 0291
T 0635
T 0220
T 0222
T 0221
T 0546
T 1150
T 0292
T 0302
T 1393
T 1306
T 0483
T 0335
T 1505
T 0934
T 0637
T 1020
T 0645
T 0297
T 1884
T 0551
T 0660
T 1550
T 1549
T 0729
T 0527
T 0521
T 0500
T 0161
T 0646
T 0129
T 0145
T 1592
T 1978
T 1658
T 0836
T 0644
T 1765
T 1043
T 0865
T 0791
T 1614
T 0215
T 1024
T 0367
T 0368
T 1734
T 1395
T 0860
T 0437
T 0474
T 1392
T 0086
T 0298
T 0532
T 0541
T 0522
T 1987
T 1806
T 0223
T 0224
T 1435
T 0819
T 0837
T 0945
T 1023
T 1511
T 0466
T 1389
T 0299
T 0190
T 1473
T 1689
T 1156
T 1153
T 0562
T 1901
T 1804
T 1869
T 1051
T 1998
T 1740
T 1361
T 1701
T 1732
T 1365
T 1453
T 1728
T 0868
T 0943
T 1241
T 2023
T 0242
T 0243
T 0276
T 0742
T 0484
T 2011
T 0440
T 0240
T 1276
T 0550
T 1059
T 0430
T 1710
T 0200
T 1457
T 0662
T 1705
T 0278
T 1586
T 0720
T 1476
T 2003
T 0702
T 1730
T 1629
T 0863
T 0169
T 1660

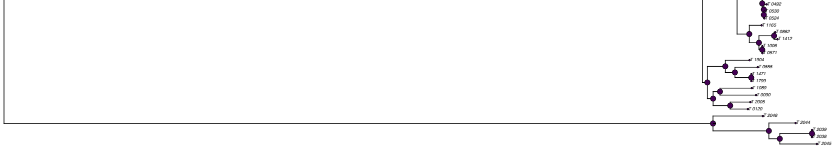
T 0492
T 0530
T 0524
T 1165
T 0862
T 1412
T 1006
T 0571
T 1904
T 0555
T 1471
T 1799
T 1089
T 0090
T 2005
T 0120
T 2048
T 2044
T 2039
T 2038
T 2045

Effector gene: tle3
Locus 2

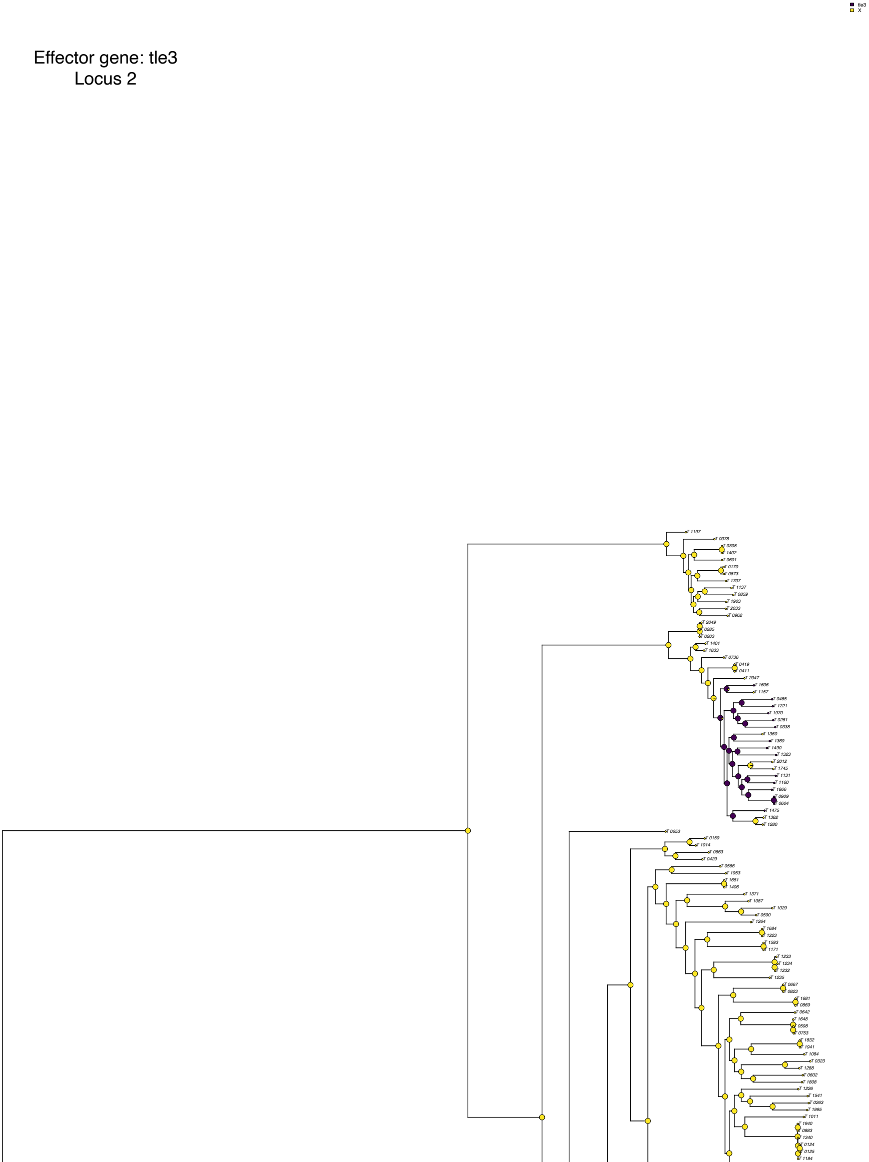

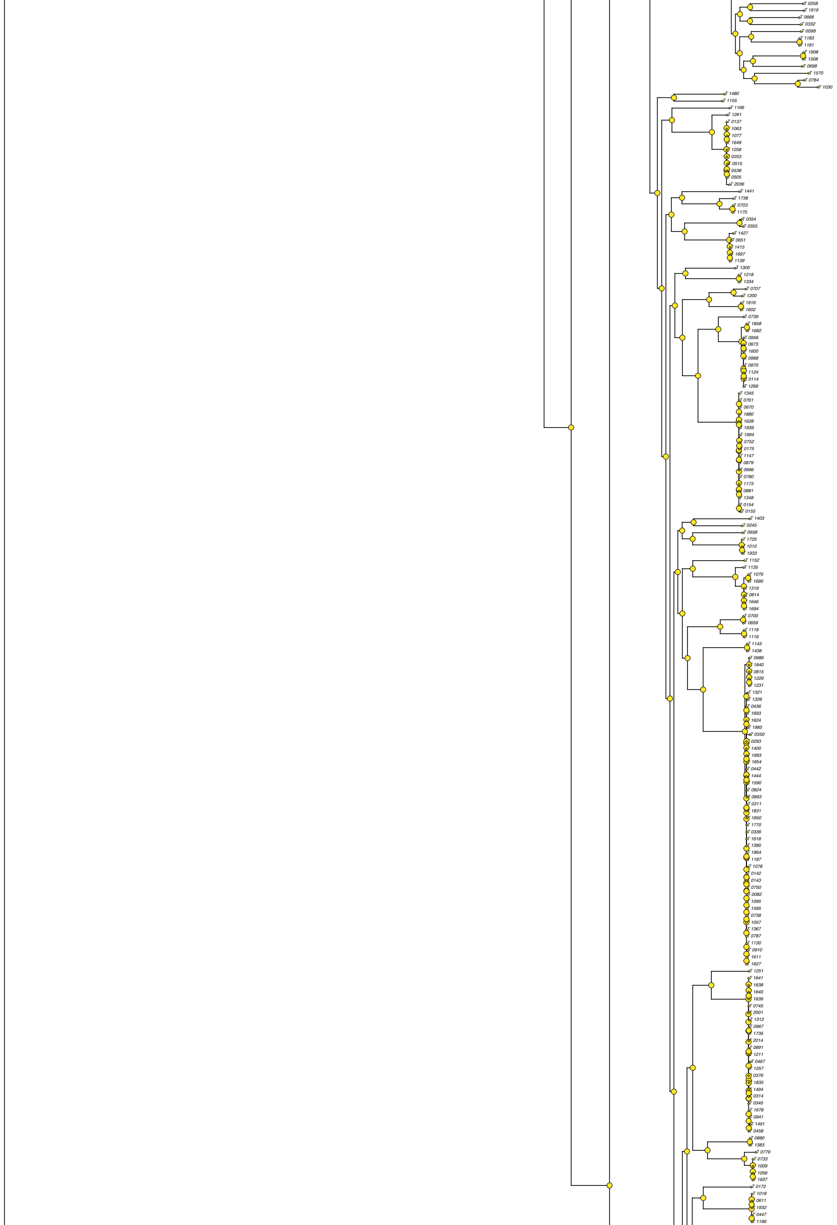
T 0258
T 1919
T 0668
T 0332
T 0098
T 1183
1181
T 1908
1508
T 0698
T 1570
T 0784
T 1030
T 1480
T 1155
T 1166
T 1261
T 0137
1063
1077
1649
1258
0253
0515
0538
0505
T 2036
T 1441
T 1738
T 0703
T 1175
T 0354
T 0355
T 1427
T 0651
1415
1697
1139
T 1300
T 1218
1334
T 0707
T 1200
T 1916
1602
T 0739
T 1858
1682
T 0956
0673
1600
0968
T 0970
1124
0114
T 1269
T 1345
0761
0670
1880
1628
1939
T 1994
0752
0175
T 1147
0879
T 0696
T 0780
1173
0881
1348
0154
T 0155
T 1403
T 0245
T 0658
T 1725
1010
1933
T 1152
T 1135
T 1079
1690
1319
0814
1646
1694
T 0700
0659
T 1118
1116
T 1143
1438
T 0986
1840
0815
1229
1231
T 1321
1326
T 0436
1693
1624
T 1985
T 0350
0293
1400
1993
1854
T 0442
1444
1590
T 0824
0893
T 0311
1831
1850
T 1770
T 0336
T 1618
1390
1964
1187
T 1078
0142
0143
0750
0082
1095
1595
0738
1057
T 1367
0787
T 1130
0910
1611
1627
T 1251
T 1641
1638
1640
1639
T 0745
T 2001
T 1312
T 0967
1735
2014
T 0891
1211
T 0467
1257
0376
1835
1494
0314
0345
T 1678
0941
T 1491
0458
T 0880
1383
T 0779
T 0733
1009
1056
1937
T 0172
T 1018
0611
1932
T 0447
1196

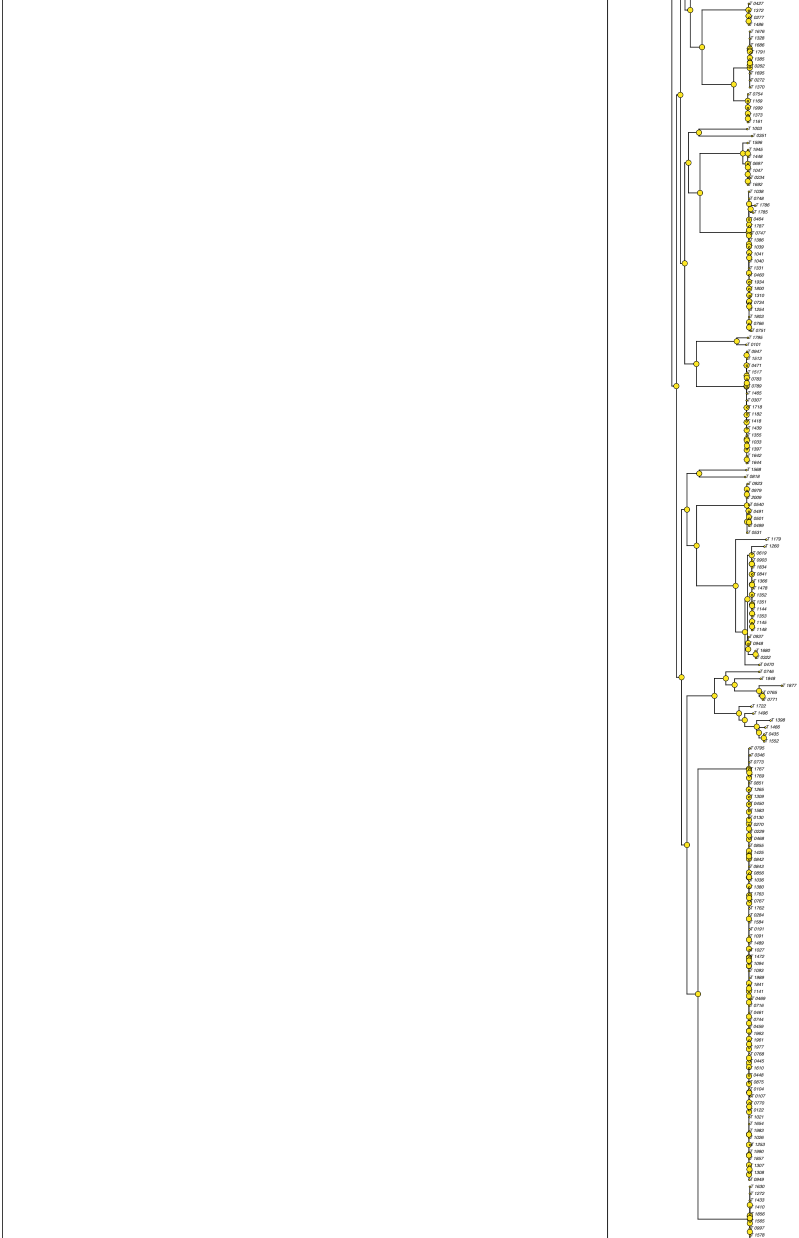
T 0427
T 1372
T 0277
T 1486
T 1676
T 1328
T 1686
T 1791
T 1385
T 0262
T 1695
T 0272
T 1370
T 0754
T 1169
T 1999
T 1373
T 1161
T 1003
T 0351
T 1596
T 1945
T 1448
T 0697
T 1047
T 0234
T 1692
T 1038
T 0748
T 1786
T 1785
T 0464
T 1787
T 0747
T 1386
T 1039
T 1041
T 1040
T 1331
T 0460
T 1934
T 1800
T 1310
T 0734
T 1254
T 1803
T 0766
T 0751
T 1795
T 0101
T 0947
T 1513
T 0471
T 1517
T 0783
T 0789
T 1465
T 0307
T 1718
T 1182
T 1418
T 1439
T 1355
T 1033
T 1397
T 1642
T 1644
T 1568
T 0818
T 0923
T 0979
T 2009
T 0540
T 0491
T 0501
T 0499
T 0531
T 1179
T 1260
T 0619
T 0903
T 1834
T 0841
T 1366
T 1478
T 1352
T 1351
T 1144
T 1353
T 1145
T 1148
T 0937
T 0948
T 1680
T 0322
T 0470
T 0746
T 1848
T 1877
T 0765
T 0771
T 1722
T 1496
T 1398
T 1466
T 0435
T 1552
T 0795
T 0346
T 0773
T 1767
T 1769
T 0851
T 1265
T 1309
T 0450
T 1583
T 0130
T 0270
T 0229
T 0468
T 0855
T 1425
T 0842
T 0843
T 0856
T 1036
T 1380
T 1763
T 0767
T 1762
T 0284
T 1584
T 0191
T 1091
T 1489
T 1027
T 1472
T 1094
T 1093
T 1989
T 1841
T 1141
T 0469
T 0716
T 0461
T 0744
T 0459
T 1963
T 1961
T 1977
T 0768
T 0445
T 1610
T 0448
T 0875
T 0104
T 0107
T 0770
T 0122
T 1021
T 1654
T 1983
T 1026
T 1253
T 1990
T 1857
T 1307
T 1308
T 0949
T 1630
T 1272
T 1433
T 1410
T 1856
T 1565
T 0997
T 1578

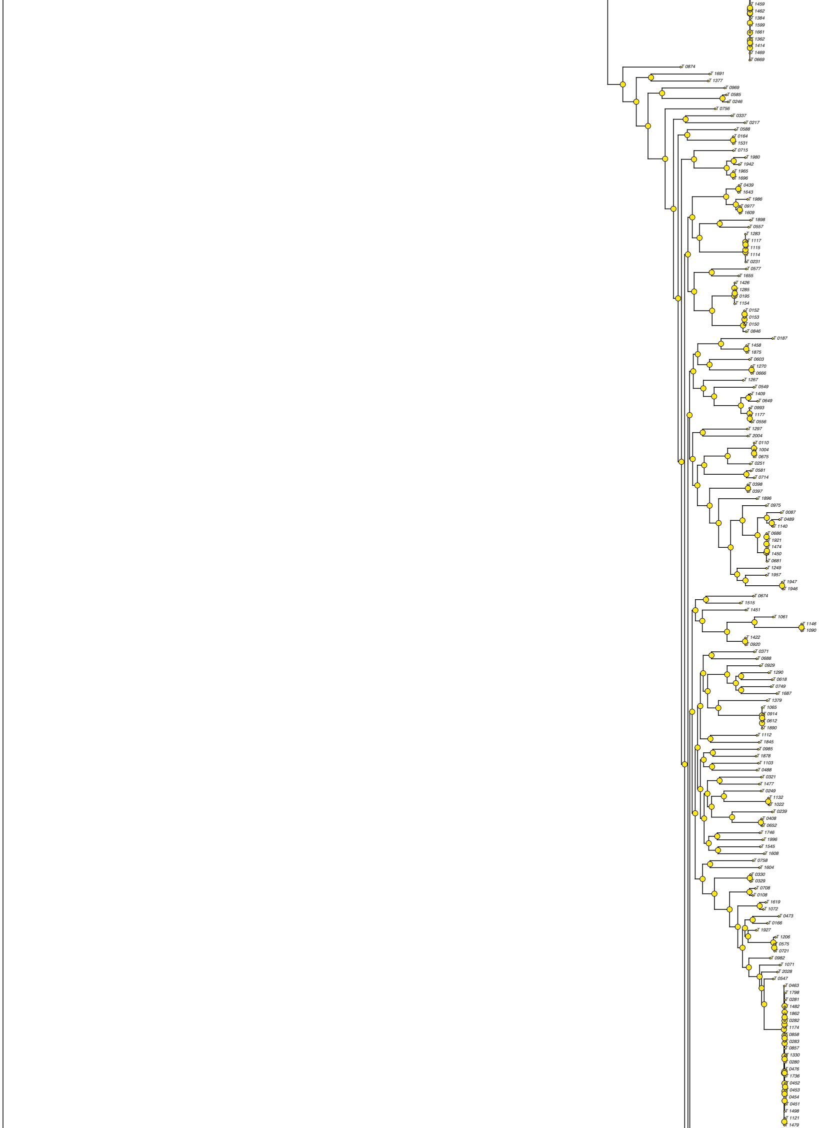

T 1459
T 1462
T 1384
T 1599
T 1661
T 1362
T 1414
T 1469
T 0669
T 0874
T 1691
T 1377
T 0969
T 0585
T 0246
T 0756
T 0337
T 0217
T 0588
T 0164
T 1531
T 0715
T 1980
T 1942
T 1965
T 1696
T 0439
T 1643
T 1986
T 0977
T 1609
T 1898
T 0557
T 1283
T 1117
T 1115
T 1114
T 0231
T 0577
T 1655
T 1426
T 1285
T 0195
T 1154
T 0152
T 0153
T 0150
T 0846
T 0187
T 1458
T 1875
T 0603
T 1270
T 0666
T 1267
T 0549
T 1409
T 0649
T 0993
T 1177
T 0556
T 1297
T 2004
T 0110
T 1004
T 0675
T 0251
T 0581
T 0714
T 0398
T 0397
T 1896
T 0975
T 0087
T 0489
T 1140
T 0686
T 1921
T 1474
T 1450
T 0681
T 1249
T 1957
T 1947
T 1946
T 0674
T 1515
T 1451
T 1061
T 1146
T 1090
T 1422
T 0920
T 0371
T 0688
T 0929
T 1290
T 0618
T 0749
T 1687
T 1379
T 1065
T 0914
T 0612
T 1890
T 1112
T 1845
T 0985
T 1878
T 1103
T 0488
T 0321
T 1477
T 0249
T 1132
T 1022
T 0239
T 0408
T 0652
T 1746
T 1996
T 1545
T 1608
T 0758
T 1604
T 0330
T 0329
T 0708
T 0108
T 1619
T 1072
T 0473
T 0166
T 1927
T 1206
T 0575
T 0721
T 0982
T 1071
T 2028
T 0547
T 0463
T 1798
T 0281
T 1482
T 1862
T 0282
T 1174
T 0858
T 0283
T 0857
T 1330
T 0280
T 0476
T 1736
T 0452
T 0453
T 0454
T 0451
T 1498
T 1121
T 1479

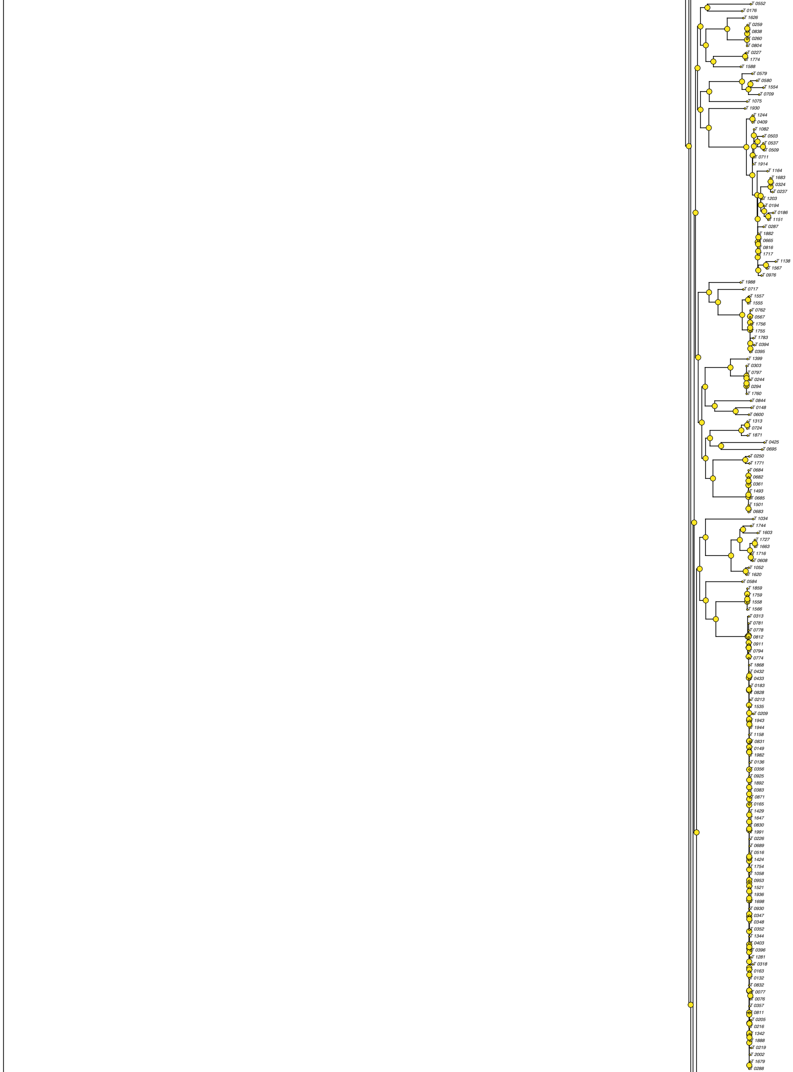
T 0552
T 0176
T 1626
T 0259
T 0838
T 0260
T 0804
T 0227
T 1774
T 1588
T 0579
T 0580
T 1554
T 0709
T 1075
T 1930
T 1244
T 0409
T 1082
T 0503
T 0537
T 0509
T 0711
T 1914
T 1164
T 1683
T 0324
T 0237
T 1203
T 0194
T 0186
T 1151
T 0287
T 1882
T 0665
T 0816
T 1717
T 1138
T 1567
T 0976
T 1988
T 0717
T 1557
T 1555
T 0762
T 0567
T 1756
T 1755
T 1783
T 0394
T 0395
T 1399
T 0303
T 0797
T 0244
T 0294
T 1760
T 0844
T 0148
T 0600
T 1313
T 0724
T 1871
T 0425
T 0695
T 0250
T 1771
T 0684
T 0682
T 0361
T 1493
T 0685
T 1501
T 0683
T 1034
T 1744
T 1603
T 1727
T 1663
T 1716
T 0608
T 1052
T 1620
T 0584
T 1859
T 1759
T 1558
T 1566
T 0313
T 0781
T 0778
T 0812
T 0911
T 0794
T 0774
T 1868
T 0432
T 0433
T 0183
T 0828
T 0213
T 1535
T 0209
T 1943
T 1944
T 1158
T 0831
T 0149
T 1982
T 0136
T 0356
T 0925
T 1892
T 0383
T 0871
T 0165
T 1429
T 1647
T 0830
T 1991
T 0226
T 0689
T 0516
T 1424
T 1754
T 1058
T 0953
T 1521
T 1936
T 1698
T 0930
T 0347
T 0348
T 0352
T 1344
T 0403
T 0396
T 1281
T 0318
T 0163
T 0132
T 0832
T 0077
T 0076
T 0357
T 0811
T 0205
T 0216
T 1342
T 1888
T 0219
T 2002
T 1679
T 0288

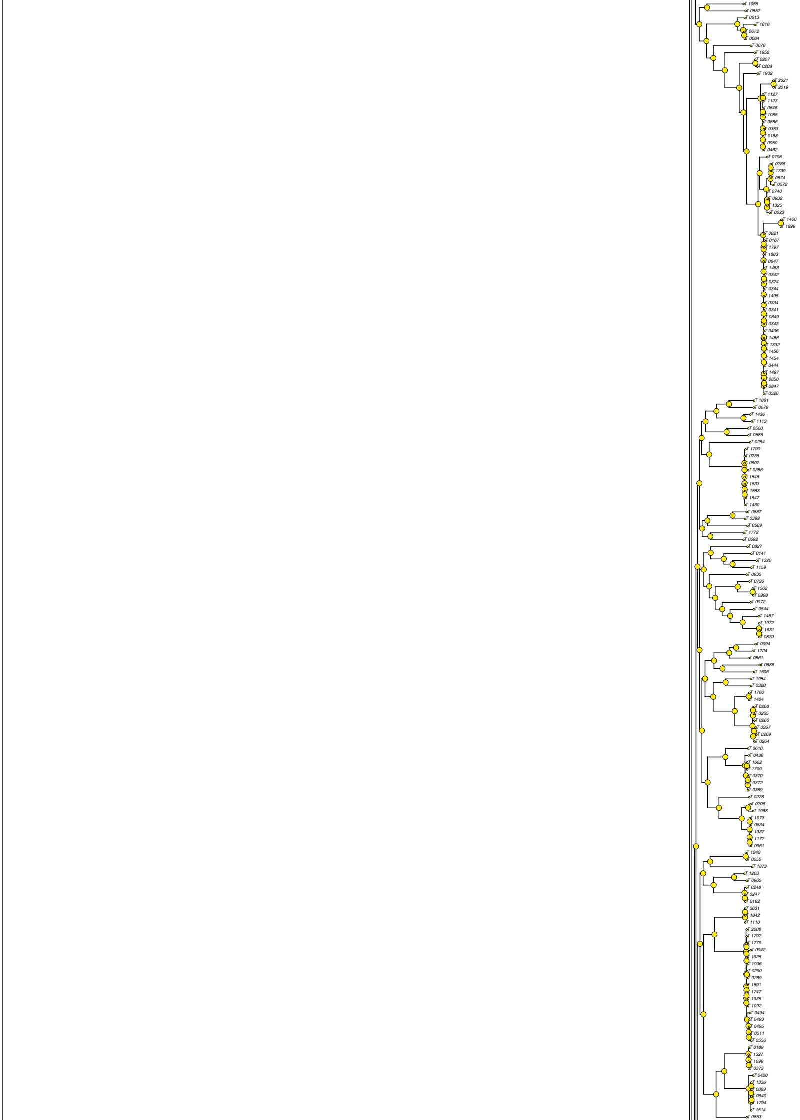
T 1055
T 0852
T 0613
T 1810
T 0672
T 0084
T 0678
T 1952
T 0207
T 0208
T 1902
T 2021
T 2019
T 1127
T 1123
T 0648
T 1085
T 0866
T 0353
T 0188
T 0950
T 0462
T 0796
T 0286
T 1739
T 0574
T 0572
T 0740
T 0932
T 1325
T 0623
T 1460
T 1899
T 0821
T 0167
T 1797
T 1883
T 0647
T 1483
T 0342
T 0374
T 0344
T 1495
T 0334
T 0341
T 0849
T 0343
T 0406
T 1488
T 1332
T 1456
T 1454
T 0444
T 1497
T 0850
T 0847
T 0326
T 1881
T 0679
T 1436
T 1113
T 0560
T 0586
T 0254
T 1790
T 0235
T 0802
T 0358
T 1546
T 1533
T 1553
T 1547
T 1430
T 0887
T 0399
T 0589
T 1772
T 0692
T 0827
T 0141
T 1320
T 1159
T 0935
T 0726
T 1562
T 0998
T 0972
T 0544
T 1467
T 1972
T 1631
T 0870
T 0094
T 1224
T 0861
T 0886
T 1506
T 1954
T 0320
T 1780
T 1404
T 0268
T 0265
T 0266
T 0267
T 0269
T 0264
T 0610
T 0438
T 1662
T 1709
T 0370
T 0372
T 0369
T 0228
T 0206
T 1968
T 1073
T 0834
T 1337
T 1172
T 0961
T 1240
T 0655
T 1873
T 1263
T 0965
T 0248
T 0247
T 0182
T 0631
T 1842
T 1110
T 2008
T 1792
T 1779
T 0942
T 1925
T 1906
T 0290
T 0289
T 1591
T 1747
T 1935
T 1092
T 0494
T 0493
T 0495
T 0511
T 0536
T 0189
T 1327
T 1699
T 0373
T 0420
T 1336
T 0889
T 0840
T 1794
T 1514
T 0853

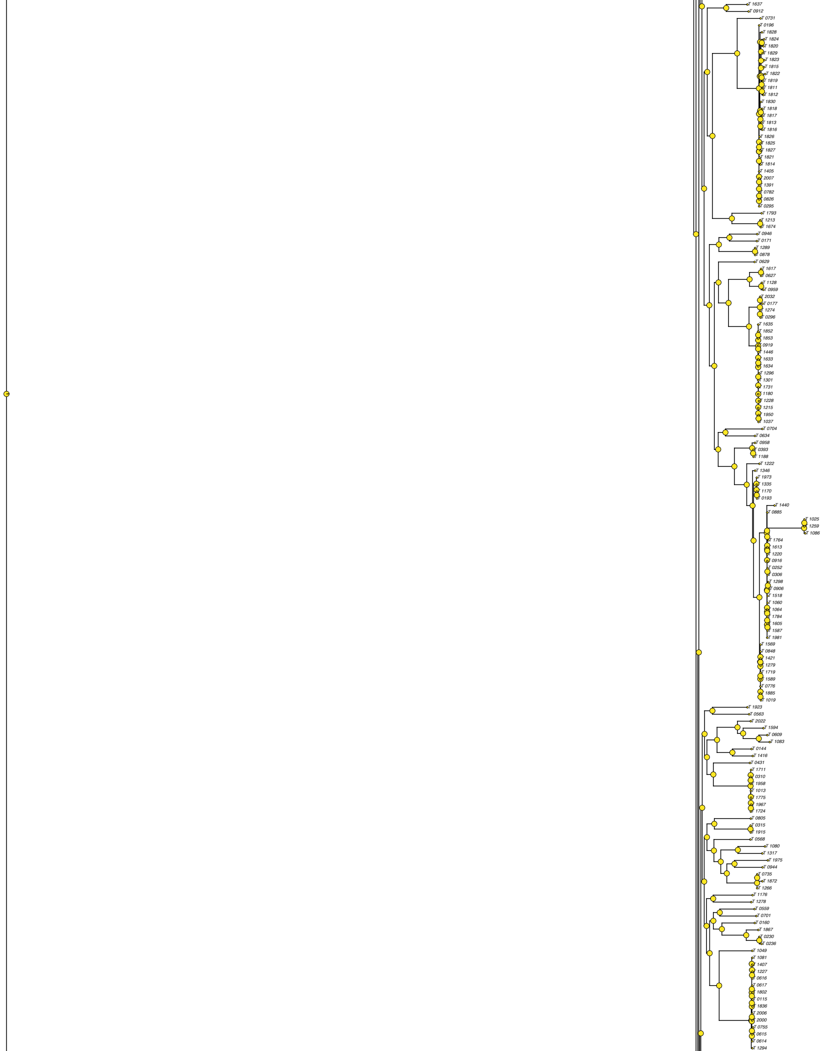
T 1637
T 0912
T 0731
T 0196
T 1828
T 1824
T 1820
T 1829
T 1823
T 1815
T 1822
T 1819
T 1811
T 1812
T 1830
T 1818
T 1817
T 1813
T 1816
T 1826
T 1825
T 1827
T 1821
T 1814
T 1405
T 2007
T 1391
T 0782
T 0826
T 0295
T 1793
T 1213
T 1674
T 0946
T 0171
T 1289
T 0878
T 0629
T 1617
T 0627
T 1128
T 0959
T 2032
T 0177
T 1274
T 0296
T 1635
T 1852
T 1853
T 0919
T 1446
T 1633
T 1634
T 1296
T 1301
T 1731
T 1180
T 1228
T 1215
T 1950
T 1037
T 0704
T 0634
T 0958
T 0393
T 1188
T 1222
T 1346
T 1973
T 1335
T 1170
T 0193
T 1440
T 0885
T 1025
T 1259
T 1086
T 1764
T 1613
T 1220
T 0916
T 0252
T 0306
T 1298
T 0906
T 1518
T 1060
T 1064
T 1784
T 1605
T 1587
T 1981
T 1569
T 0848
T 1421
T 1279
T 1719
T 1589
T 0776
T 1885
T 1019
T 1923
T 0563
T 2022
T 1594
T 0609
T 1083
T 0144
T 1416
T 0431
T 1711
T 0310
T 1958
T 1013
T 1775
T 1967
T 1724
T 0805
T 0315
T 1915
T 0568
T 1080
T 1317
T 1975
T 0944
T 0735
T 1872
T 1266
T 1176
T 1278
T 0559
T 0701
T 0160
T 1867
T 0230
T 0236
T 1049
T 1081
T 1407
T 1227
T 0616
T 0617
T 1802
T 0115
T 1836
T 2006
T 2000
T 0755
T 0615
T 0614
T 1294

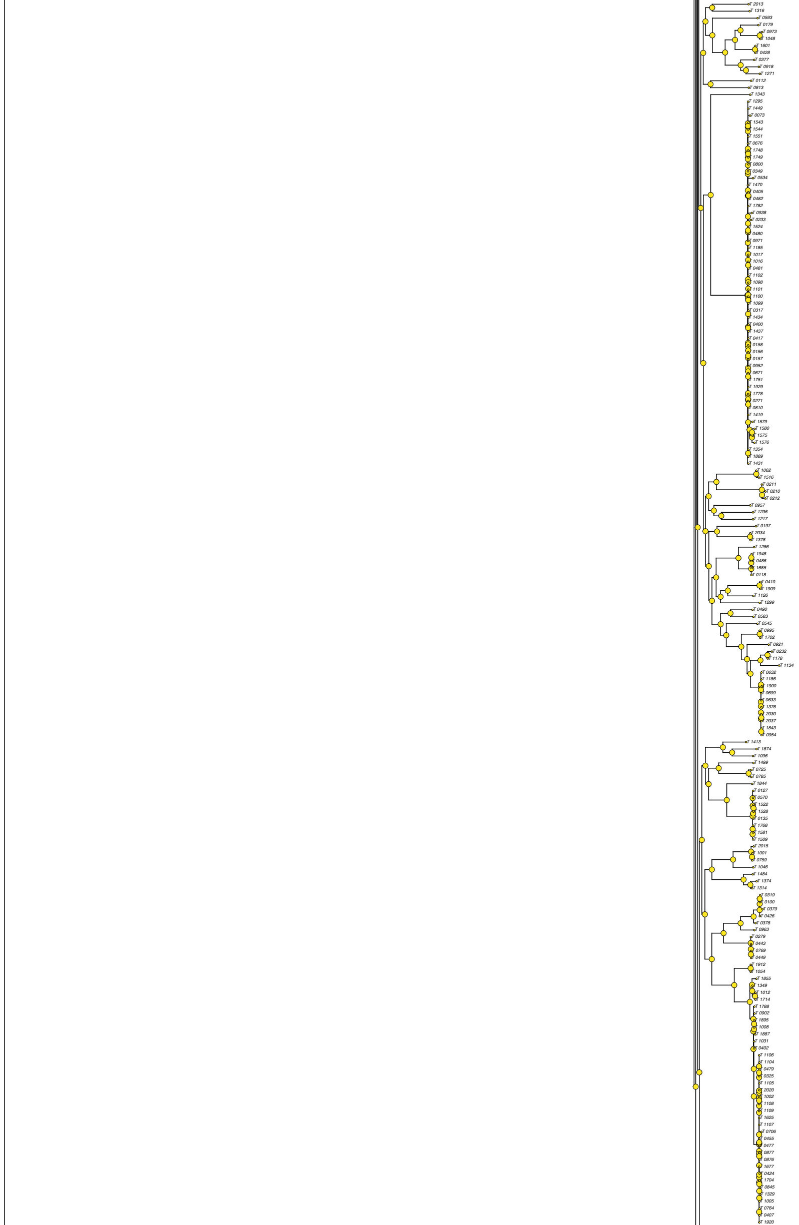
T 2013
T 1316
T 0593
T 0179
T 0973
T 1048
T 1601
T 0428
T 0377
T 0918
T 1271
T 0112
T 0813
T 1343
T 1295
T 1449
T 0073
T 1543
T 1544
T 1551
T 0676
T 1748
T 1749
T 0800
T 0349
T 0534
T 1470
T 0405
T 0482
T 1782
T 0938
T 0233
T 1524
T 0480
T 0971
T 1185
T 1017
T 1016
T 0481
T 1102
T 1098
T 1101
T 1100
T 1099
T 0317
T 1434
T 0400
T 1437
T 0417
T 0158
T 0156
T 0157
T 0952
T 0671
T 1751
T 1929
T 1778
T 0271
T 0810
T 1419
T 1579
T 1580
T 1575
T 1576
T 1354
T 1889
T 1431
T 1062
T 1516
T 0211
T 0210
T 0212
T 0957
T 1236
T 1217
T 0197
T 2034
T 1378
T 1286
T 1948
T 0486
T 1685
T 0118
T 0410
T 1909
T 1126
T 1299
T 0490
T 0583
T 0545
T 0995
T 1702
T 0921
T 0232
T 1178
T 1134
T 0632
T 1186
T 1900
T 0699
T 0633
T 1376
T 2030
T 2037
T 1843
T 0954
T 1413
T 1874
T 1096
T 1499
T 0725
T 0785
T 1844
T 0127
T 0570
T 1522
T 1528
T 0135
T 1768
T 1581
T 1509
T 2015
T 1001
T 0759
T 1046
T 1484
T 1374
T 1314
T 0319
T 0100
T 0379
T 0426
T 0378
T 0963
T 0279
T 0443
T 0769
T 0449
T 1912
T 1054
T 1855
T 1349
T 1012
T 1714
T 1788
T 0902
T 1895
T 1008
T 1887
T 1031
T 0402
T 1106
T 1104
T 0479
T 0325
T 1105
T 2020
T 1002
T 1108
T 1109
T 1625
T 1107
T 0706
T 0455
T 0477
T 0877
T 0876
T 1677
T 0424
T 1704
T 0845
T 1329
T 1005
T 0764
T 0407
T 1920

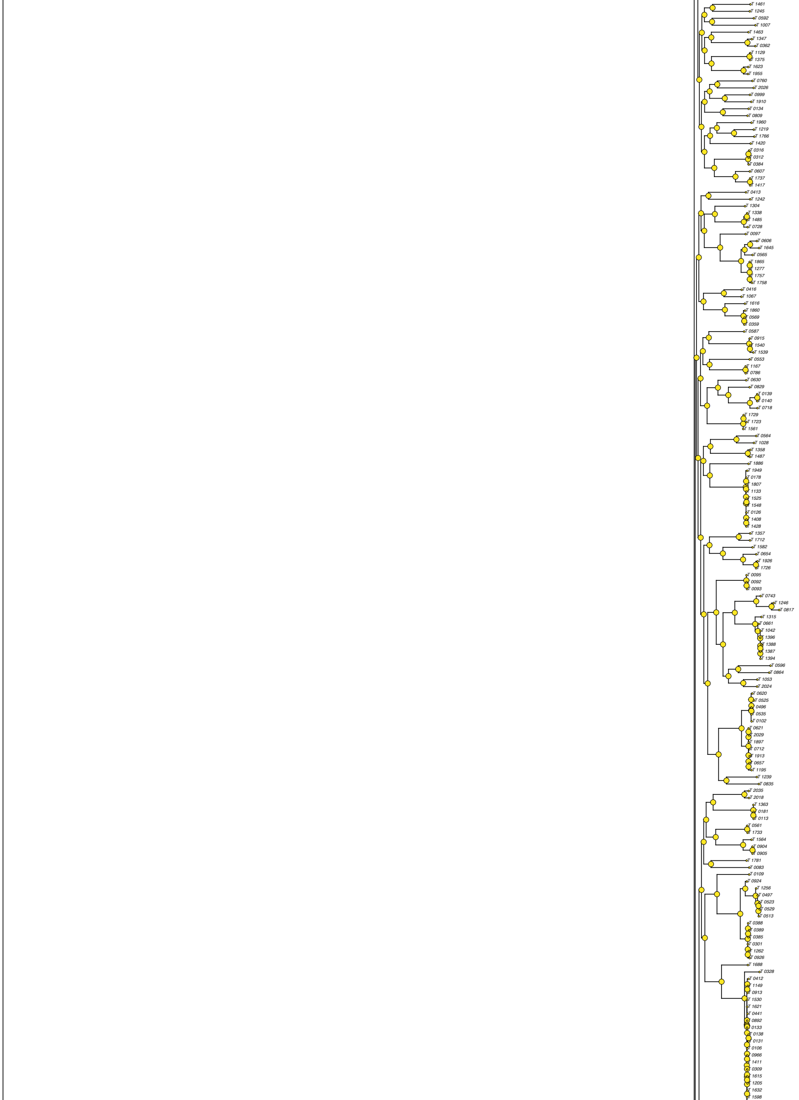
T 1461
T 1245
T 0592
T 1007
T 1463
T 1347
T 0362
T 1129
T 1375
T 1623
T 1955
T 0760
T 2026
T 0999
T 1910
T 0134
T 0809
T 1960
T 1219
T 1766
T 1420
T 0316
T 0312
T 0384
T 0607
T 1737
T 1417
T 0413
T 1242
T 1304
T 1338
T 1485
T 0728
T 0097
T 0606
T 1645
T 0565
T 1865
T 1277
T 1757
T 1758
T 0416
T 1067
T 1616
T 1860
T 0569
T 0359
T 0587
T 0915
T 1540
T 1539
T 0553
T 1167
T 0786
T 0630
T 0829
T 0139
T 0140
T 0718
T 1729
T 1723
T 1561
T 0564
T 1028
T 1358
T 1487
T 1886
T 1949
T 0178
T 1807
T 1133
T 1525
T 1548
T 0126
T 1408
T 1428
T 1357
T 1712
T 1582
T 0654
T 1926
T 1726
T 0095
T 0092
T 0093
T 0743
T 1246
T 0817
T 1315
T 0661
T 1042
T 1396
T 1388
T 1387
T 1394
T 0596
T 0864
T 1053
T 2024
T 0620
T 0525
T 0496
T 0535
T 0102
T 0621
T 2029
T 1897
T 0712
T 1913
T 0657
T 1195
T 1239
T 0835
T 2035
T 2018
T 1363
T 0181
T 0113
T 0561
T 1733
T 1564
T 0904
T 0905
T 1781
T 0083
T 0109
T 0924
T 1256
T 0497
T 0523
T 0529
T 0513
T 0388
T 0389
T 0385
T 0301
T 1262
T 0926
T 1688
T 0328
T 0412
T 1149
T 0913
T 1530
T 1621
T 0441
T 0892
T 0133
T 0138
T 0131
T 0106
T 0966
T 1411
T 0309
T 1615
T 1205
T 1632
T 1598

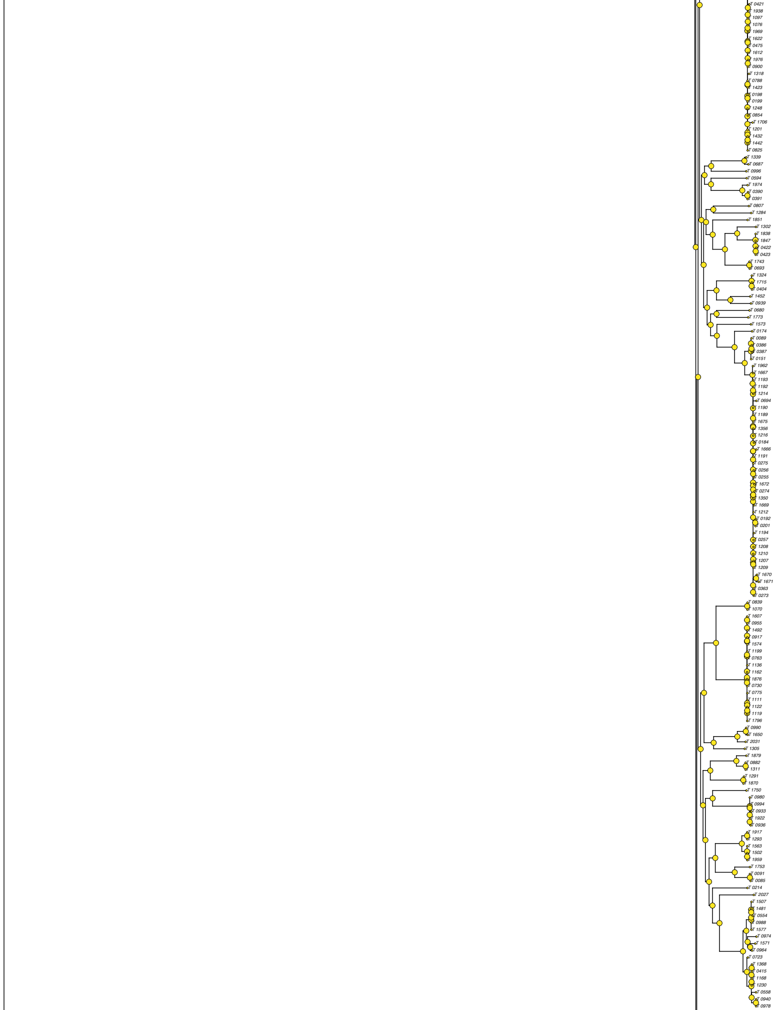
T 0421
T 1938
T 1097
T 1076
T 1969
T 1622
T 0475
T 1612
T 1976
T 0900
T 1318
T 0788
T 1423
T 0198
T 0199
T 1248
T 0854
T 1706
T 1201
T 1432
T 1442
T 0825
T 1339
T 0687
T 0996
T 0594
T 1974
T 0390
T 0391
T 0807
T 1284
T 1851
T 1302
T 1838
T 1847
T 0422
T 0423
T 1743
T 0693
T 1324
T 1715
T 0404
T 1452
T 0939
T 0680
T 1773
T 1573
T 0174
T 0089
T 0386
T 0387
T 0151
T 1962
T 1667
T 1193
T 1192
T 1214
T 0694
T 1190
T 1189
T 1675
T 1356
T 1216
T 0184
T 1666
T 1191
T 0275
T 0256
T 0255
T 1672
T 0274
T 1350
T 1669
T 1212
T 0192
T 0201
T 1194
T 0257
T 1208
T 1210
T 1207
T 1209
T 1670
T 1671
T 0363
T 0273
T 0839
T 1070
T 1607
T 0955
T 1492
T 0917
T 1574
T 1199
T 0763
T 1136
T 1162
T 1876
T 0730
T 0775
T 1111
T 1122
T 1119
T 1796
T 0990
T 1650
T 2031
T 1305
T 1879
T 0882
T 1311
T 1291
T 1870
T 1750
T 0980
T 0994
T 0933
T 1922
T 0936
T 1917
T 1293
T 1563
T 1502
T 1959
T 1753
T 0091
T 0085
T 0214
T 2027
T 1507
T 1481
T 0554
T 0988
T 1577
T 0974
T 1571
T 0964
T 0723
T 1368
T 0415
T 1168
T 1230
T 0558
T 0940
T 0978

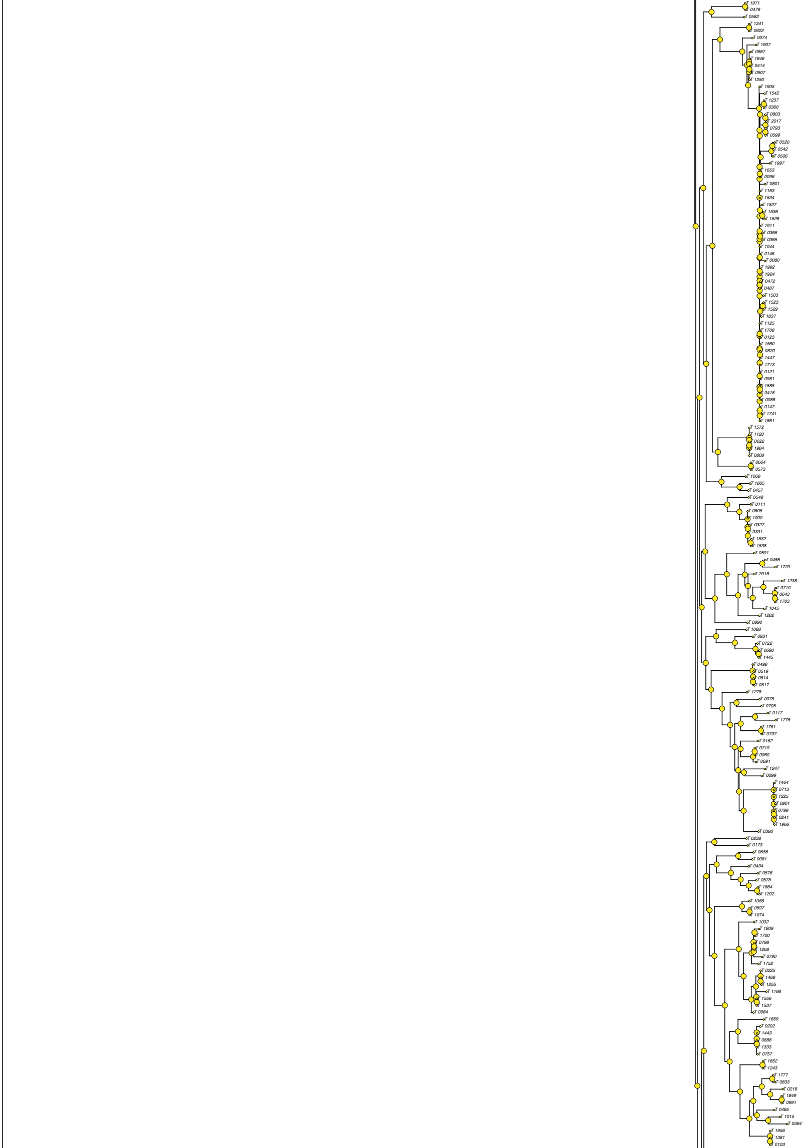
T 1971
T 0478
T 0582
T 1341
T 0922
T 0074
T 1907
T 0987
T 1846
T 0414
T 0907
T 1250
T 1905
T 1542
T 1237
T 0360
T 0803
T 2017
T 0793
T 0599
T 0520
T 0542
T 0506
T 1997
T 1653
0096
T 0801
T 1163
1534
T 1527
T 1536
T 1526
T 1911
T 0366
T 0365
T 1044
T 0146
T 0080
T 1992
T 1924
T 0472
T 0487
T 1503
T 1523
T 1529
T 1837
T 1125
T 1708
0123
T 1560
0820
T 1447
T 1713
T 0121
T 0981
1585
0418
T 0088
T 0147
T 1741
T 1861
T 1572
T 1120
T 0622
T 1984
T 0808
T 0664
T 0573
T 1068
T 1805
T 0457
T 0548
T 0111
T 0605
1000
T 0327
0331
T 1532
T 1538
T 0591
T 0456
T 1720
T 2016
T 1238
T 0710
0643
T 1703
T 1045
T 1282
T 0890
T 1088
T 0931
T 0722
T 0690
T 1445
T 0498
T 0519
T 0514
T 0517
T 1275
T 0075
T 0705
T 0117
T 1776
T 1761
T 0737
T 0162
T 0719
0960
T 0691
T 1247
T 0099
T 1464
0713
1225
T 0901
T 0799
T 0241
T 1966
T 0380
T 0238
T 0173
T 0656
T 0081
T 0434
T 0576
T 0578
T 1864
T 1292
T 1066
T 0597
1074
T 1032
T 1809
T 1700
T 0798
1268
T 0790
T 1752
T 0225
1468
T 1255
T 1198
1556
T 1537
T 0984
T 1659
T 0202
T 1443
T 0888
1333
T 0757
T 1652
T 1243
T 1777
T 0833
T 0218
T 1849
T 0991
T 0485
T 1015
T 0364
T 1956
T 1381
0103

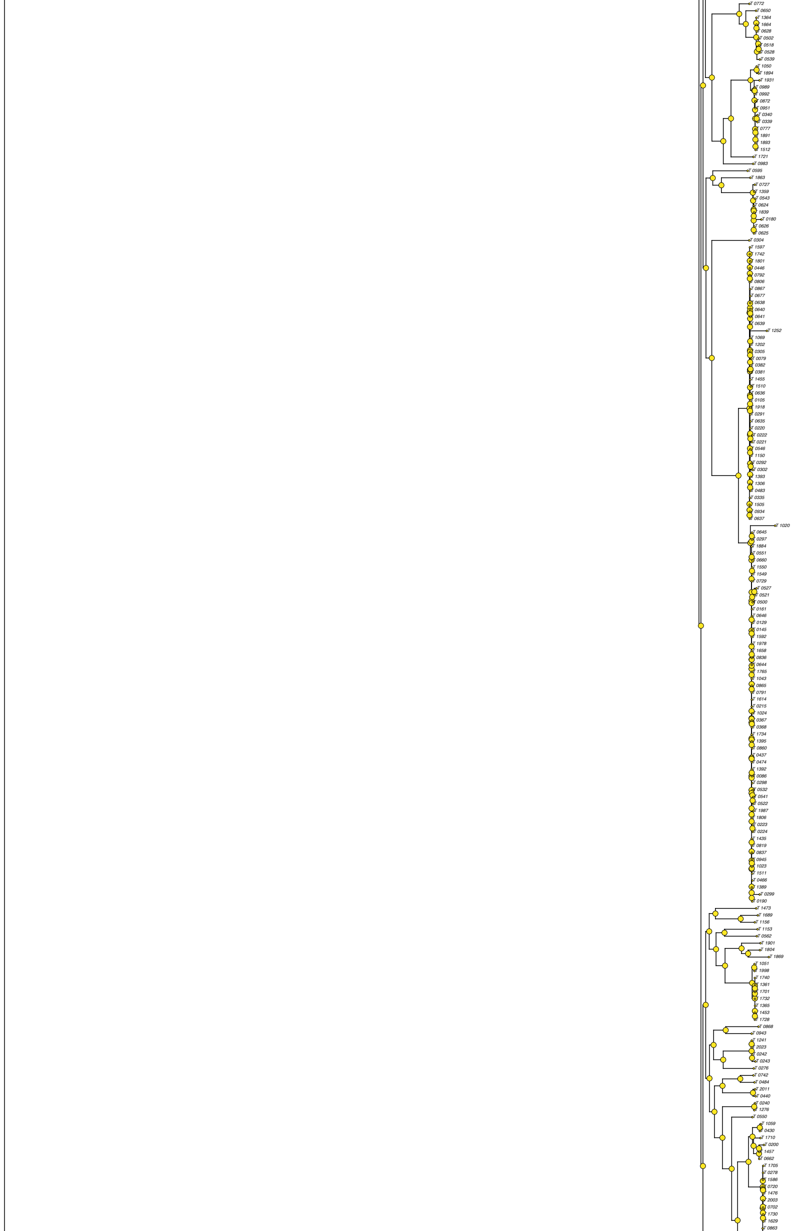
T 0772
T 0650
T 1364
T 1664
T 0628
T 0502
T 0518
T 0528
T 0539
T 1050
T 1894
T 1931
T 0989
T 0992
T 0872
T 0951
T 0340
T 0339
T 0777
T 1891
T 1893
T 1512
T 1721
T 0983
T 0595
T 1863
T 0727
T 1359
T 0543
T 0624
T 1839
T 0180
T 0626
T 0625
T 0304
T 1597
T 1742
T 1801
T 0446
T 0792
T 0806
T 0867
T 0677
T 0638
T 0640
T 0641
T 0639
T 1252
T 1069
T 1202
T 0305
T 0079
T 0382
T 0381
T 1455
T 1510
T 0636
T 0105
T 1918
T 0291
T 0635
T 0220
T 0222
T 0221
T 0546
T 1150
T 0292
T 0302
T 1393
T 1306
T 0483
T 0335
T 1505
T 0934
T 0637
T 1020
T 0645
T 0297
T 1884
T 0551
T 0660
T 1550
T 1549
T 0729
T 0527
T 0521
T 0500
T 0161
T 0646
T 0129
T 0145
T 1592
T 1978
T 1658
T 0836
T 0644
T 1765
T 1043
T 0865
T 0791
T 1614
T 0215
T 1024
T 0367
T 0368
T 1734
T 1395
T 0860
T 0437
T 0474
T 1392
T 0086
T 0298
T 0532
T 0541
T 0522
T 1987
T 1806
T 0223
T 0224
T 1435
T 0819
T 0837
T 0945
T 1023
T 1511
T 0466
T 1389
T 0299
T 0190
T 1473
T 1689
T 1156
T 1153
T 0562
T 1901
T 1804
T 1869
T 1051
T 1998
T 1740
T 1361
T 1701
T 1732
T 1365
T 1453
T 1728
T 0868
T 0943
T 1241
T 2023
T 0242
T 0243
T 0276
T 0742
T 0484
T 2011
T 0440
T 0240
T 1276
T 0550
T 1059
T 0430
T 1710
T 0200
T 1457
T 0662
T 1705
T 0278
T 1586
T 0720
T 1476
T 2003
T 0702
T 1730
T 1629
T 0863

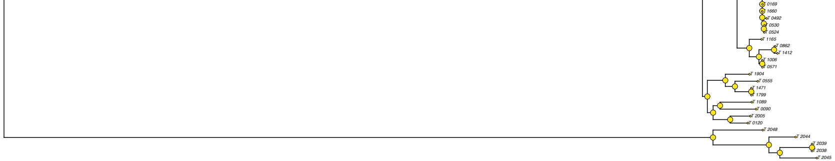
T 0169
T 1660
T 0492
T 0530
T 0524
T 1165
T 0862
T 1412
T 1006
T 0571
T 1904
T 0555
T 1471
T 1799
T 1089
T 0090
T 2005
T 0120
T 2048
T 2044
T 2039
T 2038
T 2045

Effector gene: vgrG2b
Locus 2

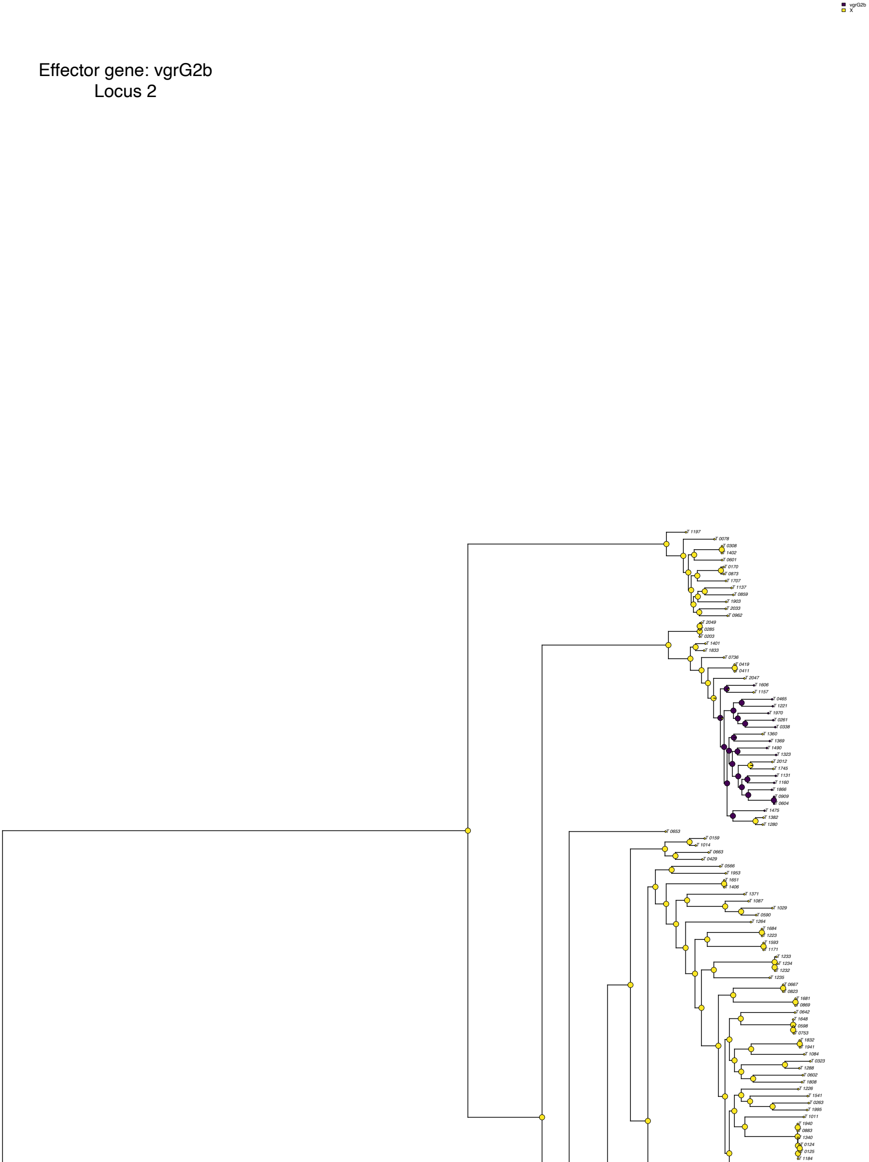

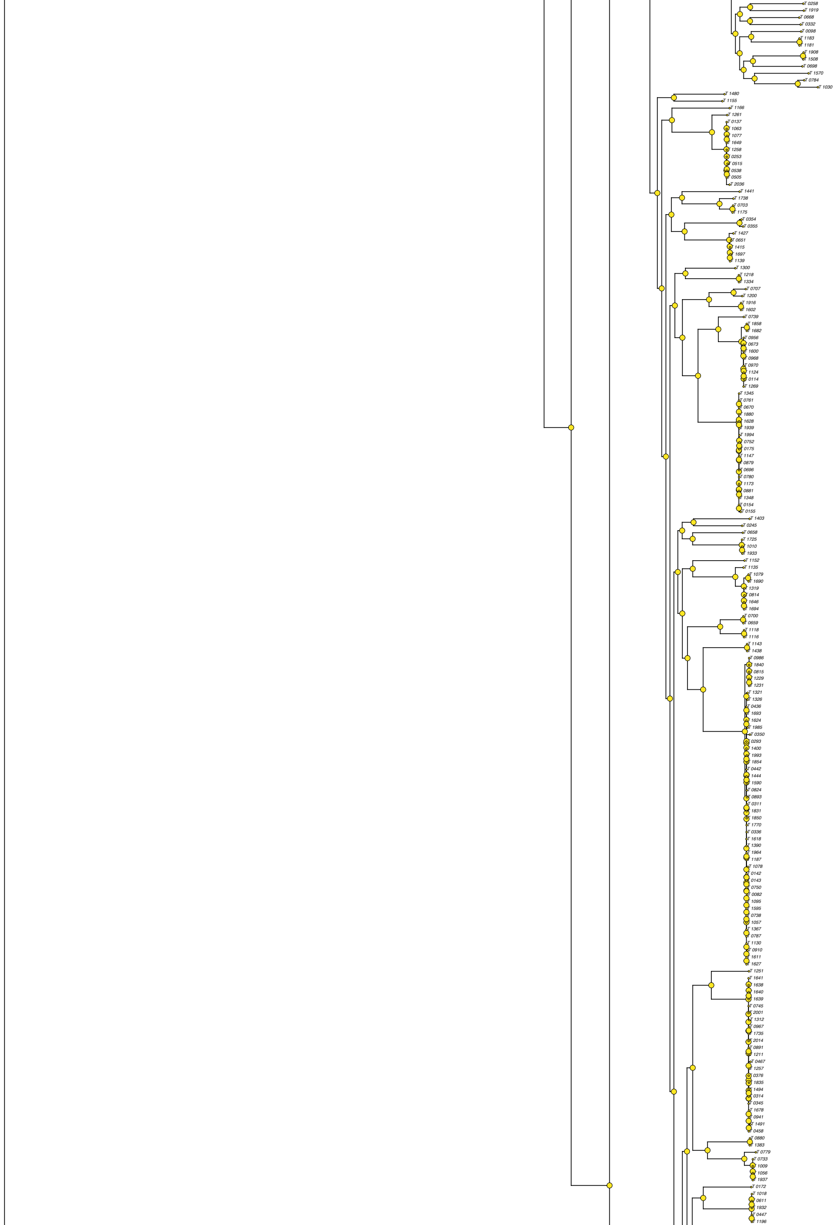
T 0258
T 1919
T 0668
T 0332
T 0098
T 1183
1181
T 1908
1508
T 0698
T 1570
T 0784
T 1030
T 1480
T 1155
T 1166
T 1261
T 0137
1063
1077
1649
1258
0253
0515
0538
0505
T 2036
T 1441
T 1738
T 0703
1175
T 0354
T 0355
T 1427
T 0651
1415
1697
1139
T 1300
T 1218
1334
T 0707
T 1200
T 1916
1602
T 0739
T 1858
1682
T 0956
0673
1600
0968
T 0970
1124
0114
T 1269
T 1345
0761
0670
1880
1628
1939
T 1994
0752
0175
T 1147
0879
T 0696
T 0780
1173
0881
1348
0154
T 0155
T 1403
T 0245
T 0658
T 1725
1010
1933
T 1152
T 1135
T 1079
1690
1319
0814
1646
1694
T 0700
0659
T 1118
1116
T 1143
1438
T 0986
1840
0815
1229
1231
T 1321
1326
T 0436
1693
1624
T 1985
T 0350
0293
1400
1993
1854
T 0442
1444
1590
T 0824
0893
T 0311
1831
1850
T 1770
T 0336
T 1618
T 1390
1964
1187
T 1078
0142
0143
0750
0082
1095
1595
0738
1057
T 1367
0787
T 1130
0910
1611
1627
T 1251
T 1641
1638
1640
1639
T 0745
T 2001
T 1312
T 0967
1735
2014
T 0891
1211
T 0467
1257
0376
1835
1494
0314
0345
T 1678
0941
T 1491
0458
T 0880
1383
T 0779
T 0733
1009
1056
1937
T 0172
T 1018
0611
1932
T 0447
1196

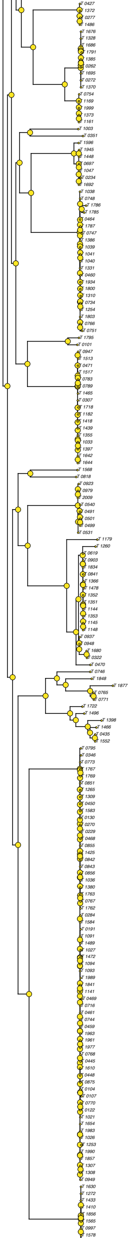
T 0427
T 1372
T 0277
T 1486
T 1676
T 1328
T 1686
T 1791
T 1385
T 0262
T 1695
T 0272
T 1370
T 0754
T 1169
T 1999
T 1373
T 1161
T 1003
T 0351
T 1596
T 1945
T 1448
T 0697
T 1047
T 0234
T 1692
T 1038
T 0748
T 1786
T 1785
T 0464
T 1787
T 0747
T 1386
T 1039
T 1041
T 1040
T 1331
T 0460
T 1934
T 1800
T 1310
T 0734
T 1254
T 1803
T 0766
T 0751
T 1795
T 0101
T 0947
T 1513
T 0471
T 1517
T 0783
T 0789
T 1465
T 0307
T 1718
T 1182
T 1418
T 1439
T 1355
T 1033
T 1397
T 1642
T 1644
T 1568
T 0818
T 0923
T 0979
T 2009
T 0540
T 0491
T 0501
T 0499
T 0531
T 1179
T 1260
T 0619
T 0903
T 1834
T 0841
T 1366
T 1478
T 1352
T 1351
T 1144
T 1353
T 1145
T 1148
T 0937
T 0948
T 1680
T 0322
T 0470
T 0746
T 1848
T 1877
T 0765
T 0771
T 1722
T 1496
T 1398
T 1466
T 0435
T 1552
T 0795
T 0346
T 0773
T 1767
T 1769
T 0851
T 1265
T 1309
T 0450
T 1583
T 0130
T 0270
T 0229
T 0468
T 0855
T 1425
T 0842
T 0843
T 0856
T 1036
T 1380
T 1763
T 0767
T 1762
T 0284
T 1584
T 0191
T 1091
T 1489
T 1027
T 1472
T 1094
T 1093
T 1989
T 1841
T 1141
T 0469
T 0716
T 0461
T 0744
T 0459
T 1963
T 1961
T 1977
T 0768
T 0445
T 1610
T 0448
T 0875
T 0104
T 0107
T 0770
T 0122
T 1021
T 1654
T 1983
T 1026
T 1253
T 1990
T 1857
T 1307
T 1308
T 0949
T 1630
T 1272
T 1433
T 1410
T 1856
T 1565
T 0997
T 1578

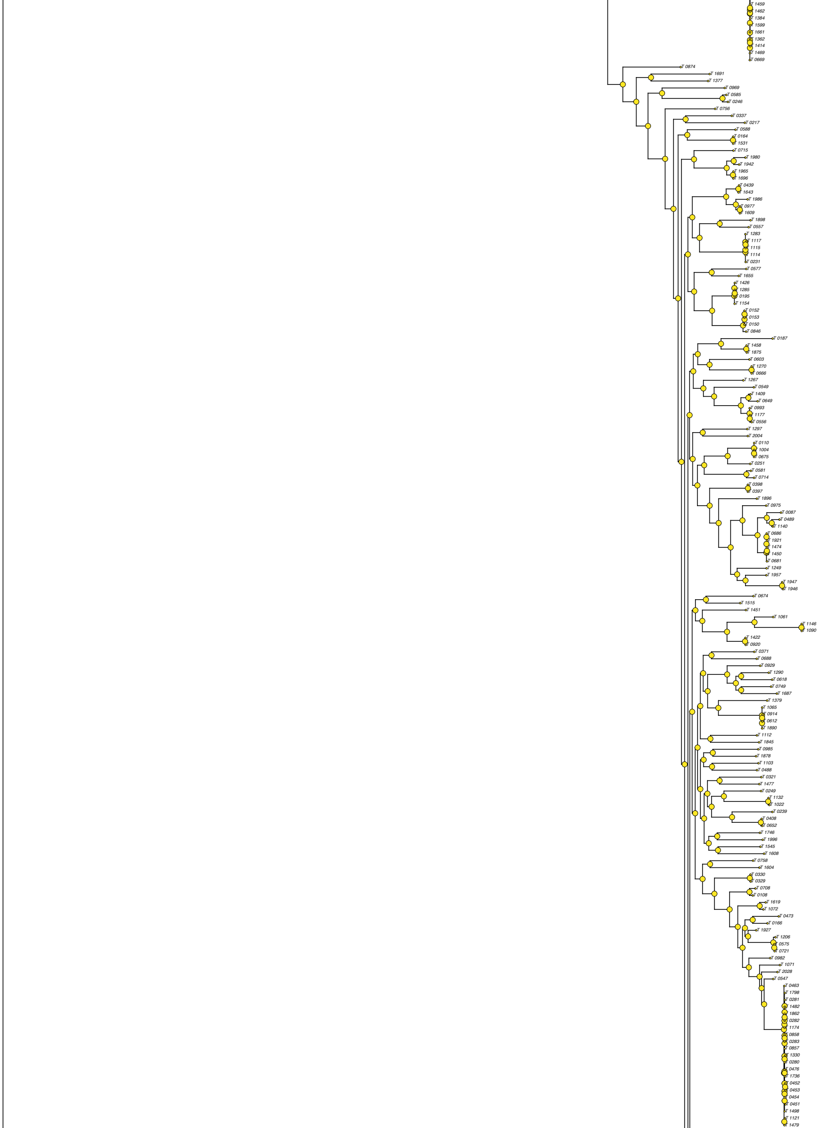
T 1459
T 1462
T 1384
T 1599
T 1661
T 1362
T 1414
T 1469
T 0669
T 0874
T 1691
T 1377
T 0969
T 0585
T 0246
T 0756
T 0337
T 0217
T 0588
T 0164
T 1531
T 0715
T 1980
T 1942
T 1965
T 1696
T 0439
T 1643
T 1986
T 0977
T 1609
T 1898
T 0557
T 1283
T 1117
T 1115
T 1114
T 0231
T 0577
T 1655
T 1426
T 1285
T 0195
T 1154
T 0152
T 0153
T 0150
T 0846
T 0187
T 1458
T 1875
T 0603
T 1270
T 0666
T 1267
T 0549
T 1409
T 0649
T 0993
T 1177
T 0556
T 1297
T 2004
T 0110
T 1004
T 0675
T 0251
T 0581
T 0714
T 0398
T 0397
T 1896
T 0975
T 0087
T 0489
T 1140
T 0686
T 1921
T 1474
T 1450
T 0681
T 1249
T 1957
T 1947
T 1946
T 0674
T 1515
T 1451
T 1061
T 1146
T 1090
T 1422
T 0920
T 0371
T 0688
T 0929
T 1290
T 0618
T 0749
T 1687
T 1379
T 1065
T 0914
T 0612
T 1890
T 1112
T 1845
T 0985
T 1878
T 1103
T 0488
T 0321
T 1477
T 0249
T 1132
T 1022
T 0239
T 0408
T 0652
T 1746
T 1996
T 1545
T 1608
T 0758
T 1604
T 0330
T 0329
T 0708
T 0108
T 1619
T 1072
T 0473
T 0166
T 1927
T 1206
T 0575
T 0721
T 0982
T 1071
T 2028
T 0547
T 0463
T 1798
T 0281
T 1482
T 1862
T 0282
T 1174
T 0858
T 0283
T 0857
T 1330
T 0280
T 0476
T 1736
T 0452
T 0453
T 0454
T 0451
T 1498
T 1121
T 1479

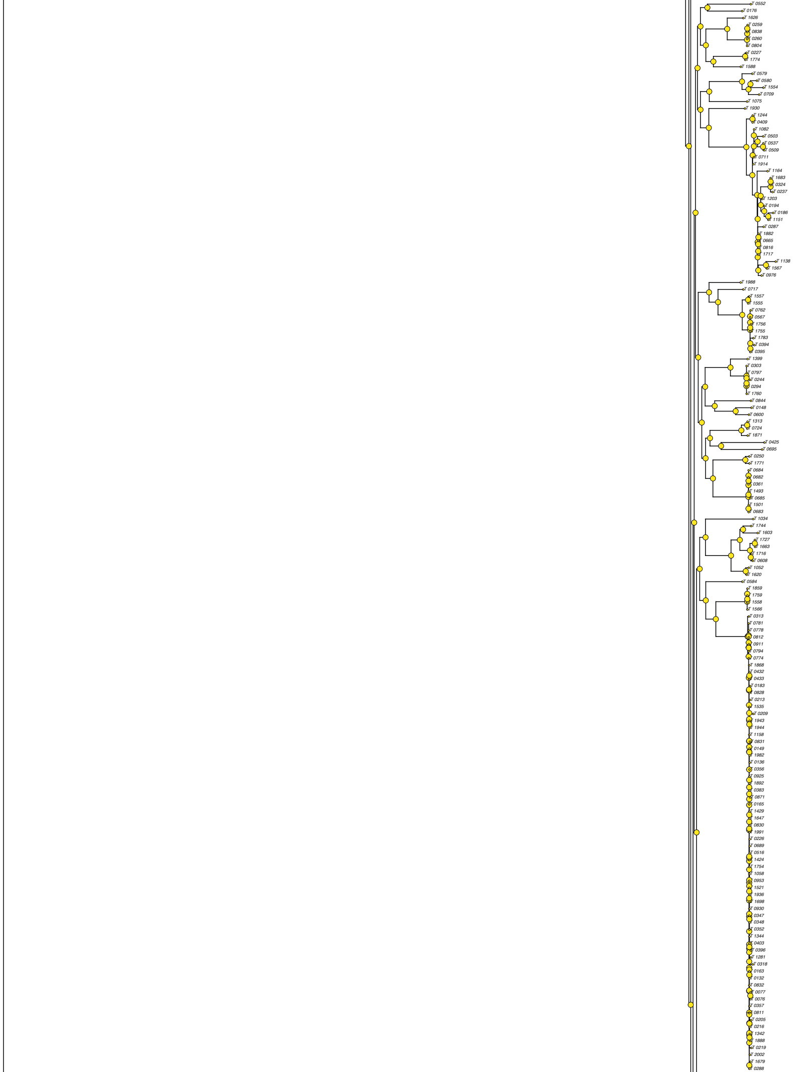
T 0552
T 0176
T 1626
T 0259
T 0838
T 0260
T 0804
T 0227
T 1774
T 1588
T 0579
T 0580
T 1554
T 0709
T 1075
T 1930
T 1244
T 0409
T 1082
T 0503
T 0537
T 0509
T 0711
T 1914
T 1164
T 1683
T 0324
T 0237
T 1203
T 0194
T 0186
T 1151
T 0287
T 1882
T 0665
T 0816
T 1717
T 1138
T 1567
T 0976
T 1988
T 0717
T 1557
T 1555
T 0762
T 0567
T 1756
T 1755
T 1783
T 0394
T 0395
T 1399
T 0303
T 0797
T 0244
T 0294
T 1760
T 0844
T 0148
T 0600
T 1313
T 0724
T 1871
T 0425
T 0695
T 0250
T 1771
T 0684
T 0682
T 0361
T 1493
T 0685
T 1501
T 0683
T 1034
T 1744
T 1603
T 1727
T 1663
T 1716
T 0608
T 1052
T 1620
T 0584
T 1859
T 1759
T 1558
T 1566
T 0313
T 0781
T 0778
T 0812
T 0911
T 0794
T 0774
T 1868
T 0432
T 0433
T 0183
T 0828
T 0213
T 1535
T 0209
T 1943
T 1944
T 1158
T 0831
T 0149
T 1982
T 0136
T 0356
T 0925
T 1892
T 0383
T 0871
T 0165
T 1429
T 1647
T 0830
T 1991
T 0226
T 0689
T 0516
T 1424
T 1754
T 1058
T 0953
T 1521
T 1936
T 1698
T 0930
T 0347
T 0348
T 0352
T 1344
T 0403
T 0396
T 1281
T 0318
T 0163
T 0132
T 0832
T 0077
T 0076
T 0357
T 0811
T 0205
T 0216
T 1342
T 1888
T 0219
T 2002
T 1679
T 0288

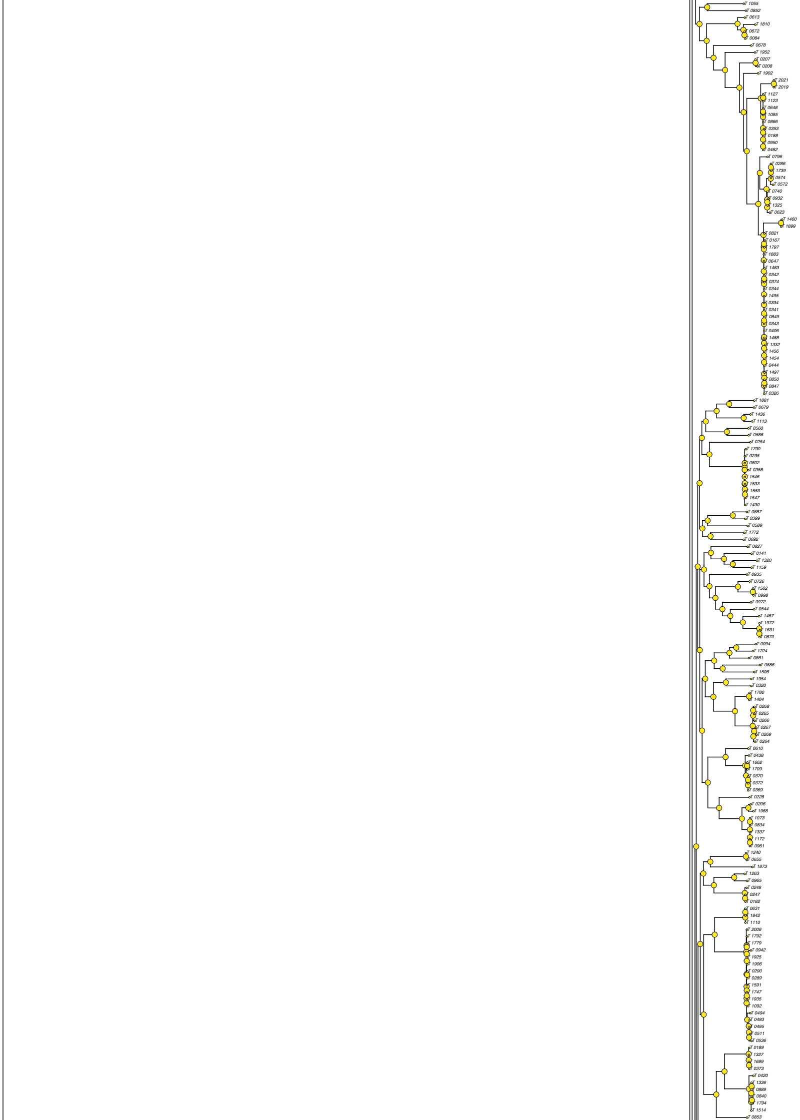
T 1055
T 0852
T 0613
T 1810
T 0672
T 0084
T 0678
T 1952
T 0207
T 0208
T 1902
T 2021
T 2019
T 1127
T 1123
T 0648
T 1085
T 0866
T 0353
T 0188
T 0950
T 0462
T 0796
T 0286
T 1739
T 0574
T 0572
T 0740
T 0932
T 1325
T 0623
T 1460
T 1899
T 0821
T 0167
T 1797
T 1883
T 0647
T 1483
T 0342
T 0374
T 0344
T 1495
T 0334
T 0341
T 0849
T 0343
T 0406
T 1488
T 1332
T 1456
T 1454
T 0444
T 1497
T 0850
T 0847
T 0326
T 1881
T 0679
T 1436
T 1113
T 0560
T 0586
T 0254
T 1790
T 0235
T 0802
T 0358
T 1546
T 1533
T 1553
T 1547
T 1430
T 0887
T 0399
T 0589
T 1772
T 0692
T 0827
T 0141
T 1320
T 1159
T 0935
T 0726
T 1562
T 0998
T 0972
T 0544
T 1467
T 1972
T 1631
T 0870
T 0094
T 1224
T 0861
T 0886
T 1506
T 1954
T 0320
T 1780
T 1404
T 0268
T 0265
T 0266
T 0267
T 0269
T 0264
T 0610
T 0438
T 1662
T 1709
T 0370
T 0372
T 0369
T 0228
T 0206
T 1968
T 1073
T 0834
T 1337
T 1172
T 0961
T 1240
T 0655
T 1873
T 1263
T 0965
T 0248
T 0247
T 0182
T 0631
T 1842
T 1110
T 2008
T 1792
T 1779
T 0942
T 1925
T 1906
T 0290
T 0289
T 1591
T 1747
T 1935
T 1092
T 0494
T 0493
T 0495
T 0511
T 0536
T 0189
T 1327
T 1699
T 0373
T 0420
T 1336
T 0889
T 0840
T 1794
T 1514
T 0853

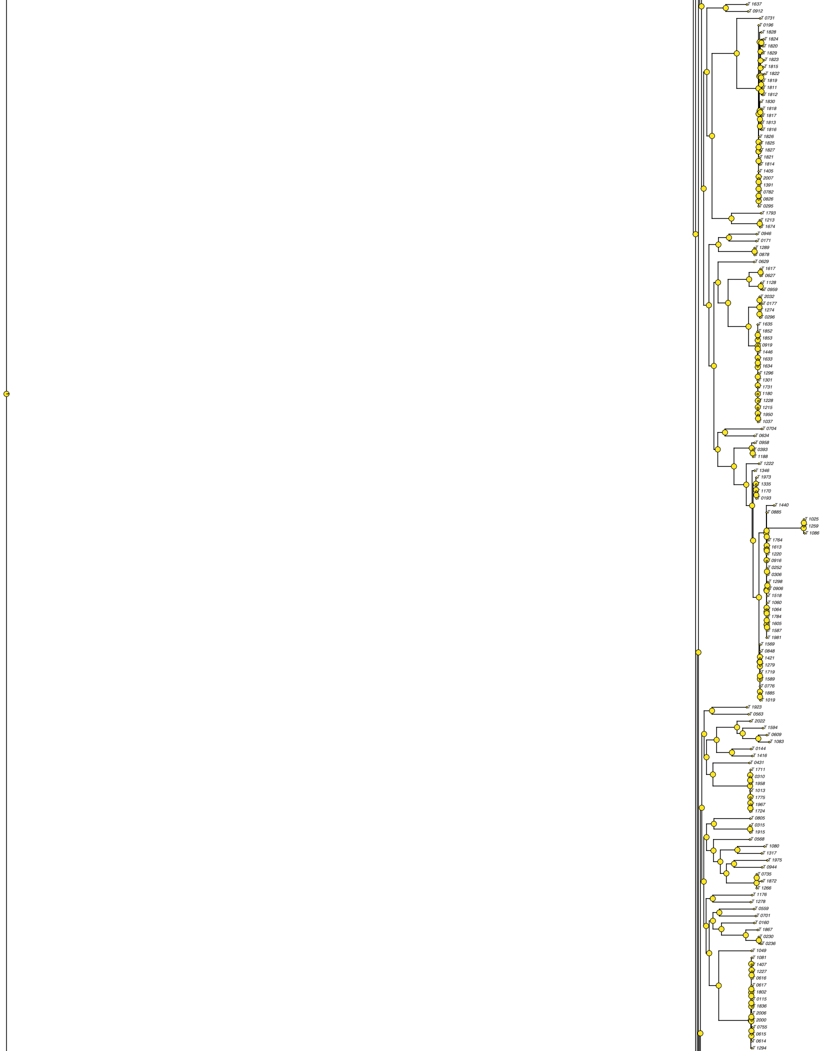
T 1637
T 0912
T 0731
T 0196
T 1828
T 1824
T 1820
T 1829
T 1823
T 1815
T 1822
T 1819
T 1811
T 1812
T 1830
T 1818
T 1817
T 1813
T 1816
T 1826
T 1825
T 1827
T 1821
T 1814
T 1405
2007
1391
0782
0826
T 0295
T 1793
T 1213
T 1674
T 0946
T 0171
T 1289
0878
T 0629
T 1617
T 0627
T 1128
T 0959
T 2032
T 0177
T 1274
T 0296
T 1635
T 1852
1853
0919
T 1446
1633
1634
T 1296
T 1301
1731
1180
1228
1215
1950
T 1037
T 0704
T 0634
T 0958
0393
T 1188
T 1222
T 1346
T 1973
1335
1170
0193
T 1440
T 0885
T 1025
1259
T 1086
T 1764
1613
T 1220
0916
T 0252
0306
T 1298
T 0906
T 1518
T 1060
1064
1784
1605
T 1587
T 1981
T 1569
T 0848
1421
1279
T 1719
1589
T 0776
1885
T 1019
T 1923
T 0563
T 2022
T 1594
T 0609
T 1083
T 0144
T 1416
T 0431
T 1711
0310
1958
T 1013
1775
1967
1724
T 0805
T 0315
1915
T 0568
T 1080
T 1317
T 1975
T 0944
T 0735
T 1872
T 1266
T 1176
T 1278
T 0559
T 0701
T 0160
T 1867
T 0230
T 0236
T 1049
T 1081
1407
1227
0616
T 0617
1802
T 0115
1836
T 2006
2000
T 0755
0615
T 0614
T 1294

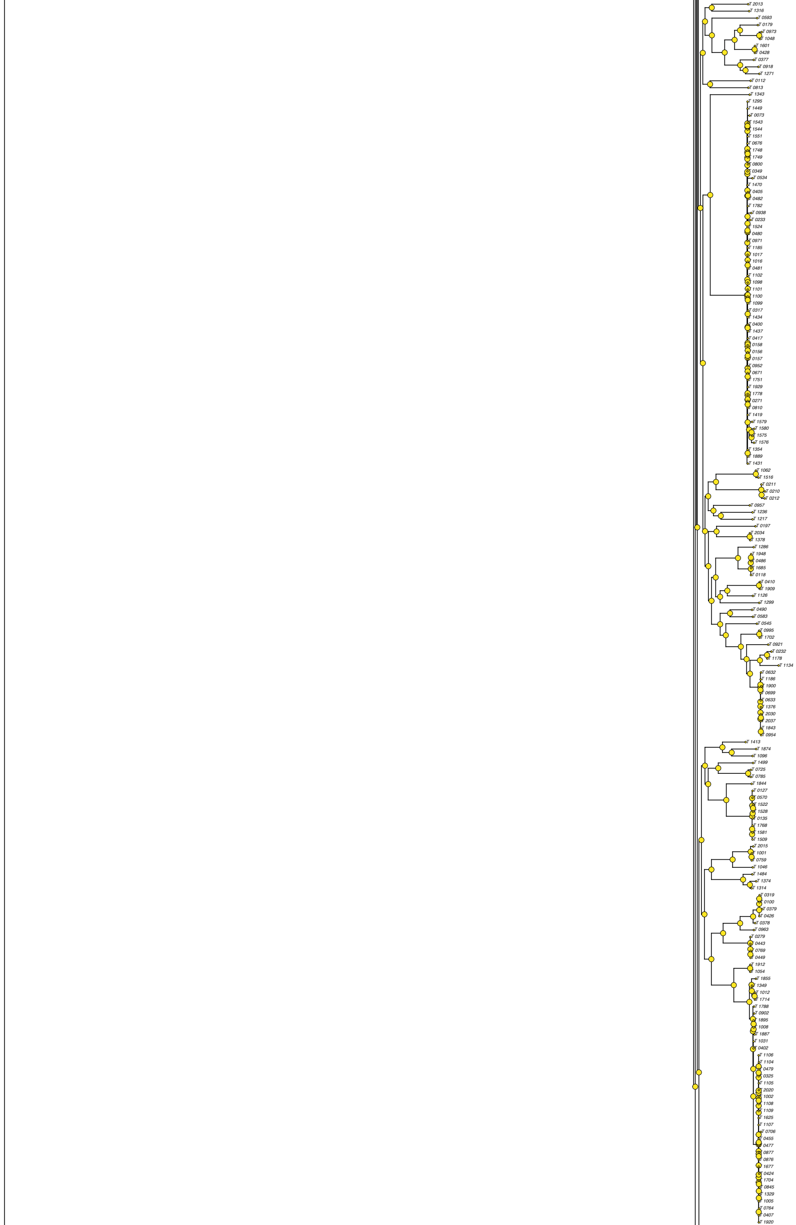

T 2013
T 1316
T 0593
T 0179
T 0973
T 1048
T 1601
T 0428
T 0377
T 0918
T 1271
T 0112
T 0813
T 1343
T 1295
T 1449
T 0073
1543
1544
T 1551
T 0676
1748
1749
0800
0349
T 0534
T 1470
0405
0482
T 1782
T 0938
T 0233
1524
0480
0971
T 1185
1017
1016
T 0481
T 1102
1098
T 1101
1100
T 1099
T 0317
T 1434
T 0400
T 1437
T 0417
0158
0156
0157
T 0952
0671
T 1751
T 1929
1778
0271
T 0810
T 1419
T 1579
T 1580
1575
T 1576
T 1354
T 1889
T 1431
T 1062
T 1516
T 0211
T 0210
T 0212
T 0957
T 1236
T 1217
T 0197
T 2034
T 1378
T 1286
T 1948
0486
1685
T 0118
T 0410
T 1909
T 1126
T 1299
T 0490
T 0583
T 0545
T 0995
T 1702
T 0921
T 0232
T 1178
T 1134
T 0632
T 1186
T 1900
T 0699
T 0633
T 1376
2030
T 2037
T 1843
T 0954
T 1413
T 1874
T 1096
T 1499
T 0725
T 0785
T 1844
T 0127
0570
T 1522
T 1528
T 0135
T 1768
1581
T 1509
T 2015
T 1001
T 0759
T 1046
T 1484
T 1374
T 1314
T 0319
0100
T 0379
T 0426
T 0378
T 0963
T 0279
0443
T 0769
T 0449
T 1912
T 1054
T 1855
1349
T 1012
T 1714
T 1788
T 0902
1895
1008
T 1887
T 1031
0402
T 1106
T 1104
0479
0325
T 1105
2020
1002
1108
1109
T 1625
T 1107
T 0706
0455
0477
0877
0876
1677
T 0424
1704
T 0845
T 1329
1005
T 0764
0407
T 1920

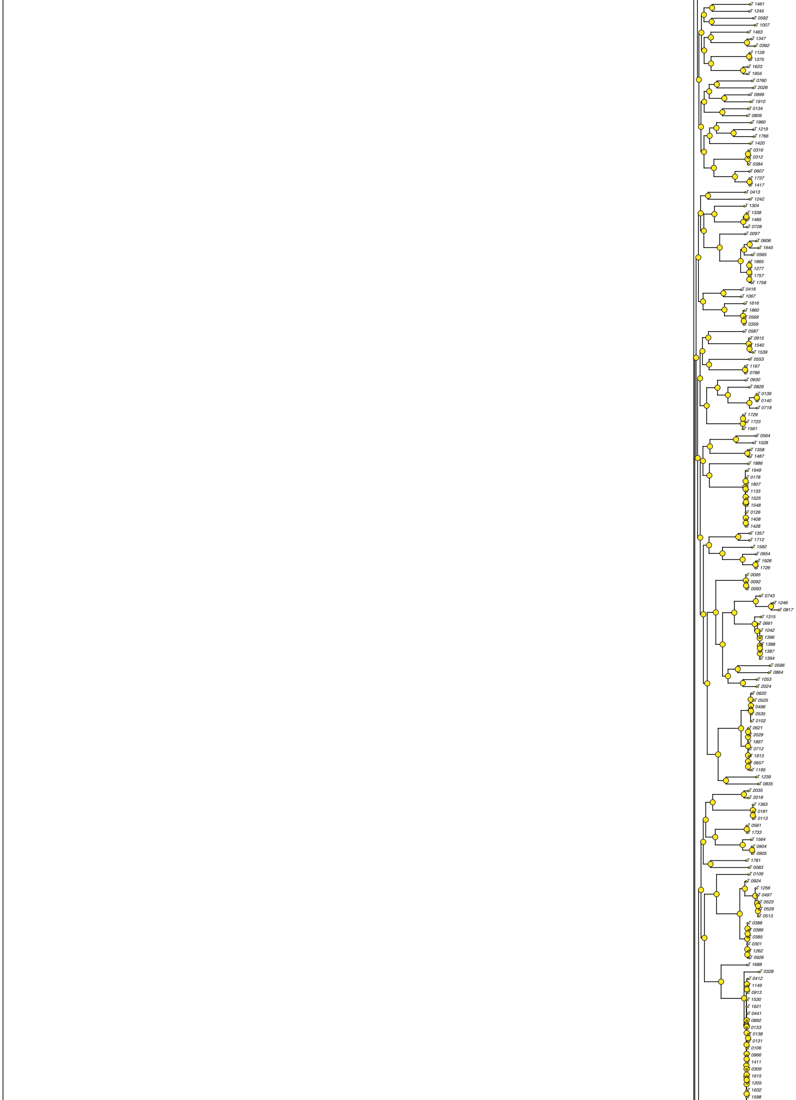
T 1461
T 1245
T 0592
T 1007
T 1463
T 1347
T 0362
T 1129
T 1375
T 1623
T 1955
T 0760
T 2026
T 0999
T 1910
T 0134
T 0809
T 1960
T 1219
T 1766
T 1420
T 0316
T 0312
T 0384
T 0607
T 1737
T 1417
T 0413
T 1242
T 1304
T 1338
T 1485
T 0728
T 0097
T 0606
T 1645
T 0565
T 1865
T 1277
T 1757
T 1758
T 0416
T 1067
T 1616
T 1860
T 0569
T 0359
T 0587
T 0915
T 1540
T 1539
T 0553
T 1167
T 0786
T 0630
T 0829
T 0139
T 0140
T 0718
T 1729
T 1723
T 1561
T 0564
T 1028
T 1358
T 1487
T 1886
T 1949
T 0178
T 1807
T 1133
T 1525
T 1548
T 0126
T 1408
T 1428
T 1357
T 1712
T 1582
T 0654
T 1926
T 1726
T 0095
T 0092
T 0093
T 0743
T 1246
T 0817
T 1315
T 0661
T 1042
T 1396
T 1388
T 1387
T 1394
T 0596
T 0864
T 1053
T 2024
T 0620
T 0525
T 0496
T 0535
T 0102
T 0621
T 2029
T 1897
T 0712
T 1913
T 0657
T 1195
T 1239
T 0835
T 2035
T 2018
T 1363
T 0181
T 0113
T 0561
T 1733
T 1564
T 0904
T 0905
T 1781
T 0083
T 0109
T 0924
T 1256
T 0497
T 0523
T 0529
T 0513
T 0388
T 0389
T 0385
T 0301
T 1262
T 0926
T 1688
T 0328
T 0412
T 1149
T 0913
T 1530
T 1621
T 0441
T 0892
T 0133
T 0138
T 0131
T 0106
T 0966
T 1411
T 0309
T 1615
T 1205
T 1632
T 1598

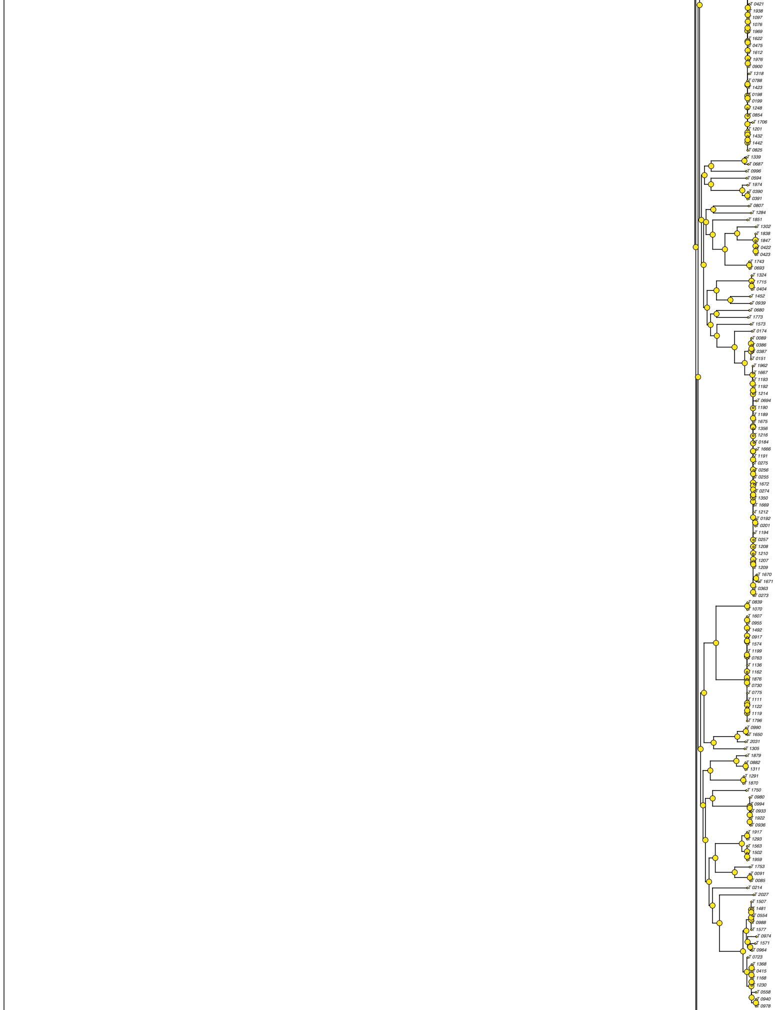
T 0421
T 1938
T 1097
T 1076
T 1969
T 1622
T 0475
T 1612
T 1976
T 0900
T 1318
T 0788
T 1423
T 0198
T 0199
T 1248
T 0854
T 1706
T 1201
T 1432
T 1442
T 0825
T 1339
T 0687
T 0996
T 0594
T 1974
T 0390
T 0391
T 0807
T 1284
T 1851
T 1302
T 1838
T 1847
T 0422
T 0423
T 1743
T 0693
T 1324
T 1715
T 0404
T 1452
T 0939
T 0680
T 1773
T 1573
T 0174
T 0089
T 0386
T 0387
T 0151
T 1962
T 1667
T 1193
T 1192
T 1214
T 0694
T 1190
T 1189
T 1675
T 1356
T 1216
T 0184
T 1666
T 1191
T 0275
T 0256
T 0255
T 1672
T 0274
T 1350
T 1669
T 1212
T 0192
T 0201
T 1194
T 0257
T 1208
T 1210
T 1207
T 1209
T 1670
T 1671
T 0363
T 0273
T 0839
T 1070
T 1607
T 0955
T 1492
T 0917
T 1574
T 1199
T 0763
T 1136
T 1162
T 1876
T 0730
T 0775
T 1111
T 1122
T 1119
T 1796
T 0990
T 1650
T 2031
T 1305
T 1879
T 0882
T 1311
T 1291
T 1870
T 1750
T 0980
T 0994
T 0933
T 1922
T 0936
T 1917
T 1293
T 1563
T 1502
T 1959
T 1753
T 0091
T 0085
T 0214
T 2027
T 1507
T 1481
T 0554
T 0988
T 1577
T 0974
T 1571
T 0964
T 0723
T 1368
T 0415
T 1168
T 1230
T 0558
T 0940
T 0978

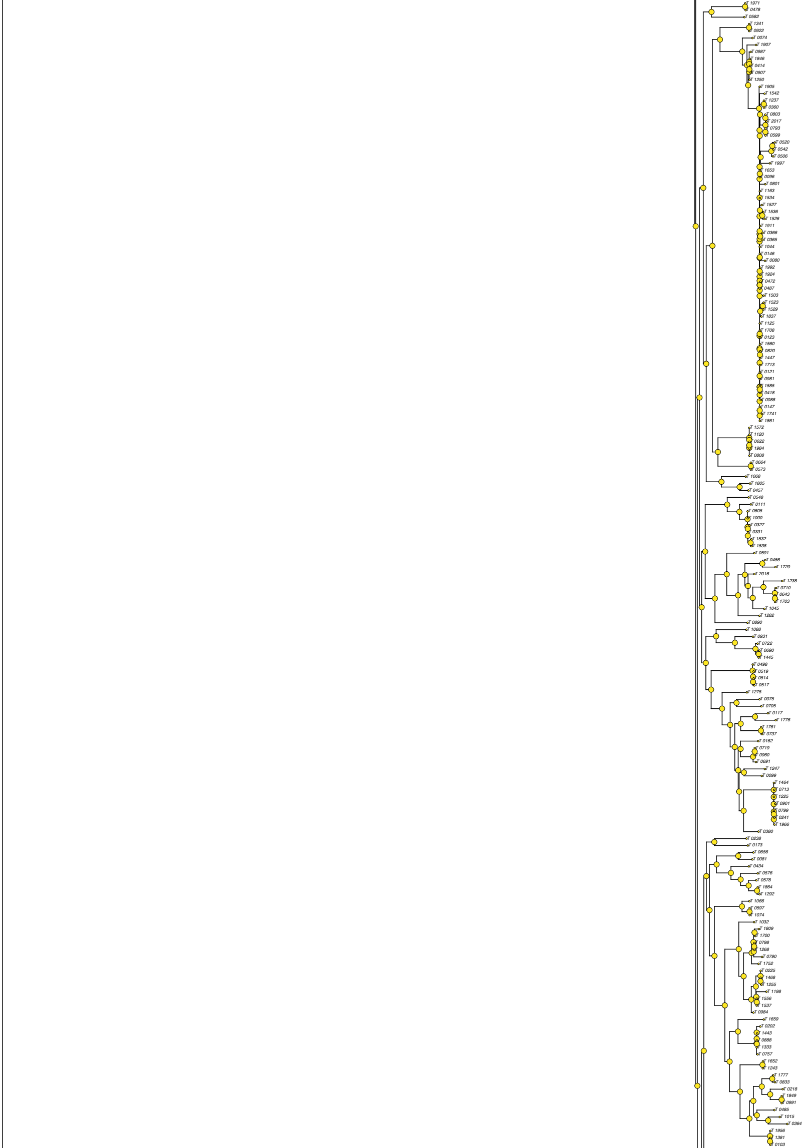
T 1971
T 0478
T 0582
T 1341
T 0922
T 0074
T 1907
T 0987
T 1846
T 0414
T 0907
T 1250
T 1905
T 1542
T 1237
T 0360
T 0803
T 2017
T 0793
T 0599
T 0520
T 0542
T 0506
T 1997
T 1653
T 0096
T 0801
T 1163
T 1534
T 1527
T 1536
T 1526
T 1911
T 0366
T 0365
T 1044
T 0146
T 0080
T 1992
T 1924
T 0472
T 0487
T 1503
T 1523
T 1529
T 1837
T 1125
T 1708
T 0123
T 1560
T 0820
T 1447
T 1713
T 0121
T 0981
T 1585
T 0418
T 0088
T 0147
T 1741
T 1861
T 1572
T 1120
T 0622
T 1984
T 0808
T 0664
T 0573
T 1068
T 1805
T 0457
T 0548
T 0111
T 0605
T 1000
T 0327
T 0331
T 1532
T 1538
T 0591
T 0456
T 1720
T 2016
T 1238
T 0710
T 0643
T 1703
T 1045
T 1282
T 0890
T 1088
T 0931
T 0722
T 0690
T 1445
T 0498
T 0519
T 0514
T 0517
T 1275
T 0075
T 0705
T 0117
T 1776
T 1761
T 0737
T 0162
T 0719
T 0960
T 0691
T 1247
T 0099
T 1464
T 0713
T 1225
T 0901
T 0799
T 0241
T 1966
T 0380
T 0238
T 0173
T 0656
T 0081
T 0434
T 0576
T 0578
T 1864
T 1292
T 1066
T 0597
T 1074
T 1032
T 1809
T 1700
T 0798
T 1268
T 0790
T 1752
T 0225
T 1468
T 1255
T 1198
T 1556
T 1537
T 0984
T 1659
T 0202
T 1443
T 0888
T 1333
T 0757
T 1652
T 1243
T 1777
T 0833
T 0218
T 1849
T 0991
T 0485
T 1015
T 0364
T 1956
T 1381
T 0103

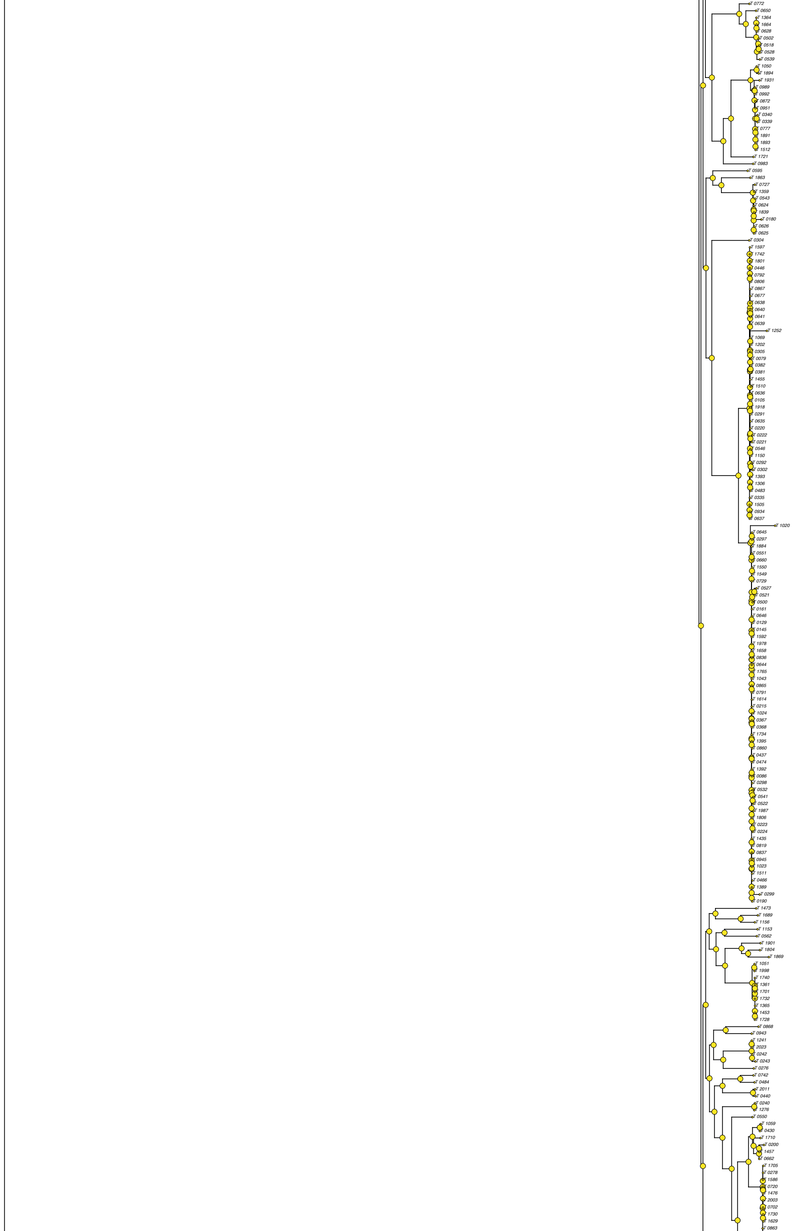
T 0772
T 0650
T 1364
T 1664
T 0628
T 0502
T 0518
T 0528
T 0539
T 1050
T 1894
T 1931
T 0989
T 0992
T 0872
T 0951
T 0340
T 0339
T 0777
T 1891
T 1893
T 1512
T 1721
T 0983
T 0595
T 1863
T 0727
T 1359
T 0543
T 0624
T 1839
T 0180
T 0626
T 0625
T 0304
T 1597
T 1742
T 1801
T 0446
T 0792
T 0806
T 0867
T 0677
T 0638
T 0640
T 0641
T 0639
T 1252
T 1069
T 1202
T 0305
T 0079
T 0382
T 0381
T 1455
T 1510
T 0636
T 0105
T 1918
T 0291
T 0635
T 0220
T 0222
T 0221
T 0546
T 1150
T 0292
T 0302
T 1393
T 1306
T 0483
T 0335
T 1505
T 0934
T 0637
T 1020
T 0645
T 0297
T 1884
T 0551
T 0660
T 1550
T 1549
T 0729
T 0527
T 0521
T 0500
T 0161
T 0646
T 0129
T 0145
T 1592
T 1978
T 1658
T 0836
T 0644
T 1765
T 1043
T 0865
T 0791
T 1614
T 0215
T 1024
T 0367
T 0368
T 1734
T 1395
T 0860
T 0437
T 0474
T 1392
T 0086
T 0298
T 0532
T 0541
T 0522
T 1987
T 1806
T 0223
T 0224
T 1435
T 0819
T 0837
T 0945
T 1023
T 1511
T 0466
T 1389
T 0299
T 0190
T 1473
T 1689
T 1156
T 1153
T 0562
T 1901
T 1804
T 1869
T 1051
T 1998
T 1740
T 1361
T 1701
T 1732
T 1365
T 1453
T 1728
T 0868
T 0943
T 1241
T 2023
T 0242
T 0243
T 0276
T 0742
T 0484
T 2011
T 0440
T 0240
T 1276
T 0550
T 1059
T 0430
T 1710
T 0200
T 1457
T 0662
T 1705
T 0278
T 1586
T 0720
T 1476
T 2003
T 0702
T 1730
T 1629
T 0863

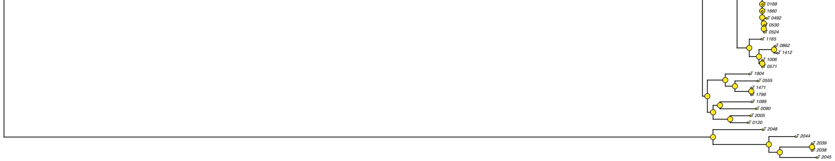
0169
1660
T 0492
T 0530
T 0524
T 1165
T 0862
T 1412
T 1006
T 0571
T 1904
T 0555
T 1471
T 1799
T 1089
T 0090
T 2005
T 0120
T 2048
T 2044
T 2039
T 2038
T 2045
