# Supplementary material for: Distribution of the four type VI secretion systems in Pseudomonas aeruginosa and classification of their core and accessory effectors
Source: Nat Commun. 2025 Jan 21;16:888. doi: 10.1038/s41467-024-54649-5 (PMC11751169; doi:10.1038/s41467-024-54649-5)

Distribution of the four type VI secretion systems in *Pseudomonas aeruginosa* and classification of their core and accessory effectors

Supplementary Data 23

To view this file, open in Preview (MacOS) or Microsoft Edge (Windows).

This file contains the results of the stochastic mapping of loci with mutually exclusive effectors. The respective effector genes and the locus are indicated at the top left corner of the page. The posterior probabilities of the stochastic mapping are indicated on each node of the phylogenetic tree. The tree is based on a core genome alignment of *P. aeruginosa* (n=1912) and *P. paraeruginosa* (n=5). Seven strains of the species *P. delhiensis, P. knackmussii, P. humi, P. jinjuensis, P. multiresinivorans, P. nitroreducens,* and *P. panipatensis* were used as an outgroup (not shown in the tree). The tree is based on the HKY+F+I model, rooted to the outgroup and distances are shown in substitutions per site.

Locus: PA0093

Effector genes: tas1, tse6, tne3 (tse6a)

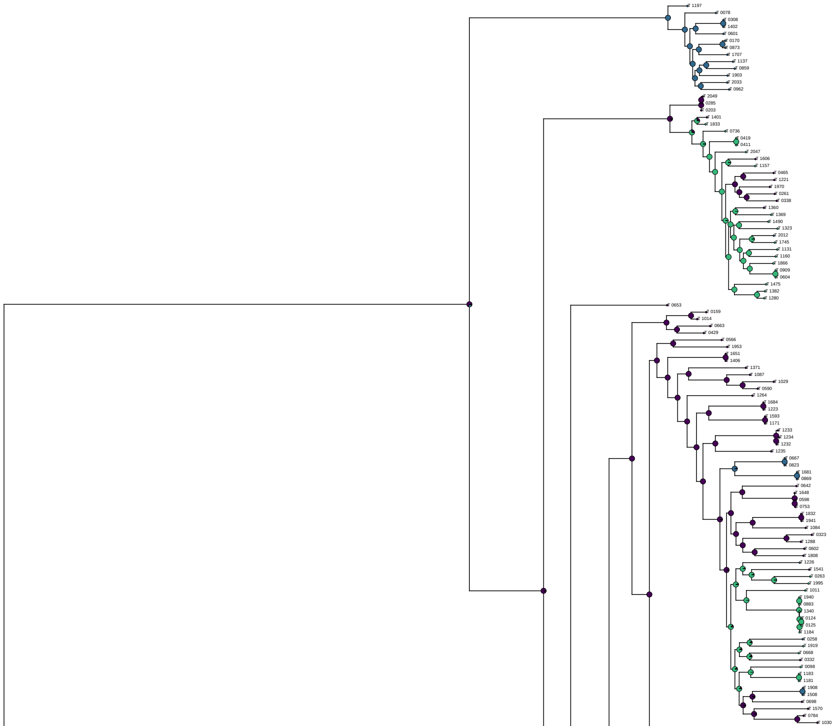

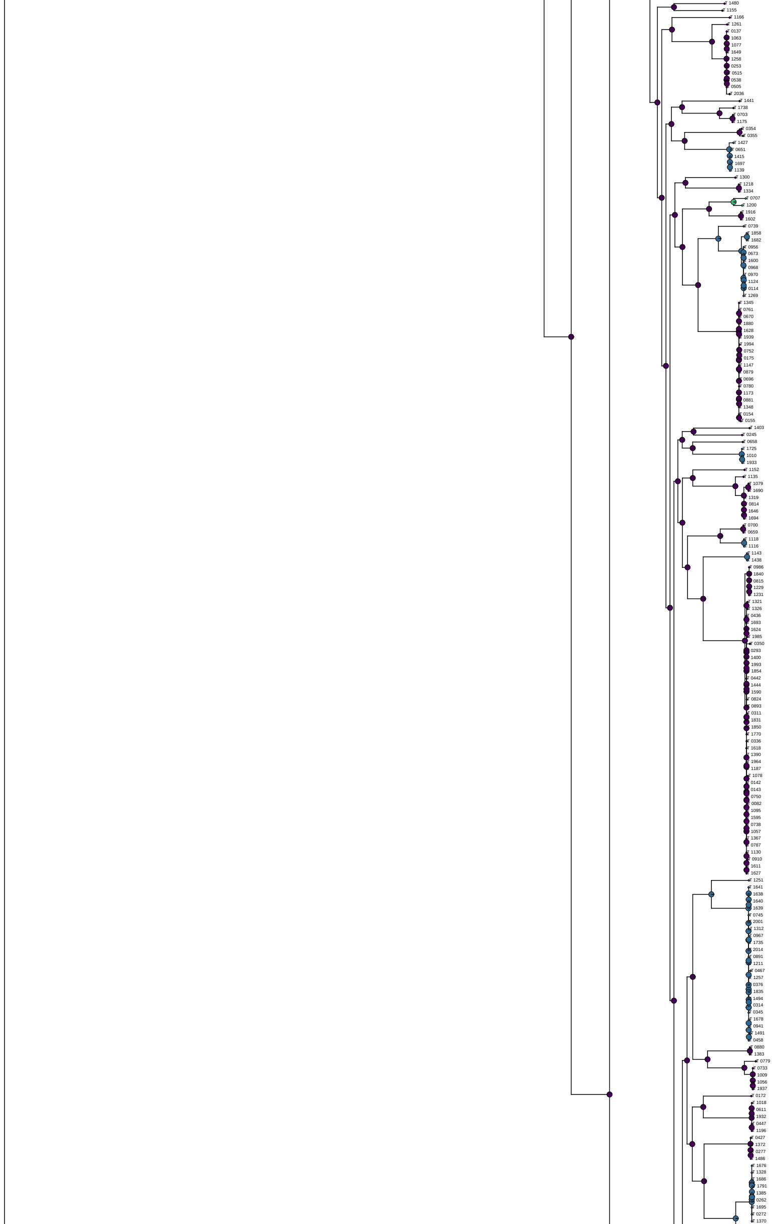
1480
1155
1166
1261
0137
1063
1077
1649
1258
0253
0515
0538
0505
2036
1441
1738
0703
1175
0354
0355
1427
0651
1415
1697
1139
1300
1218
1334
0707
1200
1916
1602
0739
1858
1682
0956
0673
1600
0968
0970
1124
0114
1269
1345
0761
0670
1880
1628
1939
1994
0752
0175
1147
0879
0696
0780
1173
0881
1348
0154
0155
1403
0245
0658
1725
1010
1933
1152
1135
1079
1690
1319
0814
1646
1694
0700
0659
1118
1116
1143
1438
0986
1840
0815
1229
1231
1321
1326
0436
1693
1624
1985
0350
0293
1400
1993
1854
0442
1444
1590
0824
0893
0311
1831
1850
1770
0336
1618
1390
1964
1187
1078
0142
0143
0750
0082
1095
1595
0738
1057
1367
0787
1130
0910
1611
1627
1251
1641
1638
1640
1639
0745
2001
1312
0967
1735
2014
0891
1211
0467
1257
0376
1835
1494
0314
0345
1678
0941
1491
0458
0880
1383
0779
0733
1009
1056
1937
0172
1018
0611
1932
0447
1196
0427
1372
0277
1486
1676
1328
1686
1791
1385
0262
1695
0272
1370

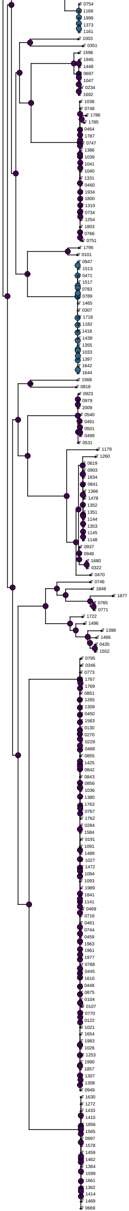
T 0754
1169
1999
1373
T 1161
T 1003
T 0351
T 1596
T 1945
T 1448
T 0697
T 1047
T 0234
T 1692
T 1038
T 0748
T 1786
T 1785
0464
1787
T 0747
T 1386
1039
1041
T 1040
T 1331
0460
1934
1800
1310
0734
T 1254
T 1803
0766
T 0751
T 1795
T 0101
T 0947
T 1513
0471
T 1517
0783
0789
T 1465
T 0307
T 1718
1182
1418
T 1439
T 1355
1033
1397
T 1642
T 1644
T 1568
T 0818
T 0923
0979
T 2009
T 0540
0491
0501
T 0499
T 0531
T 1179
T 1260
T 0619
T 0903
T 1834
0841
T 1366
T 1478
1352
1351
1144
1353
1145
T 1148
T 0937
0948
T 1680
T 0322
T 0470
T 0746
T 1848
T 1877
T 0765
T 0771
T 1722
T 1496
T 1398
T 1466
T 0435
T 1552
T 0795
T 0346
T 0773
1767
1769
T 0851
1265
1309
0450
1583
T 0130
0270
0229
0468
T 0855
1425
0842
T 0843
0856
1036
1380
1763
0767
T 1762
T 0284
T 1584
T 0191
T 1091
1489
1027
1472
1094
T 1093
T 1989
1841
1141
T 0469
0716
T 0461
0744
T 0459
1963
1961
1977
T 0768
0445
1610
0448
0875
T 0104
T 0107
0770
0122
T 1021
T 1654
T 1983
1026
T 1253
T 1990
1857
1307
1308
T 0949
T 1630
T 1272
T 1433
1410
1856
1565
T 0997
T 1578
T 1459
1462
T 1384
1599
1661
1362
1414
T 1469
T 0669

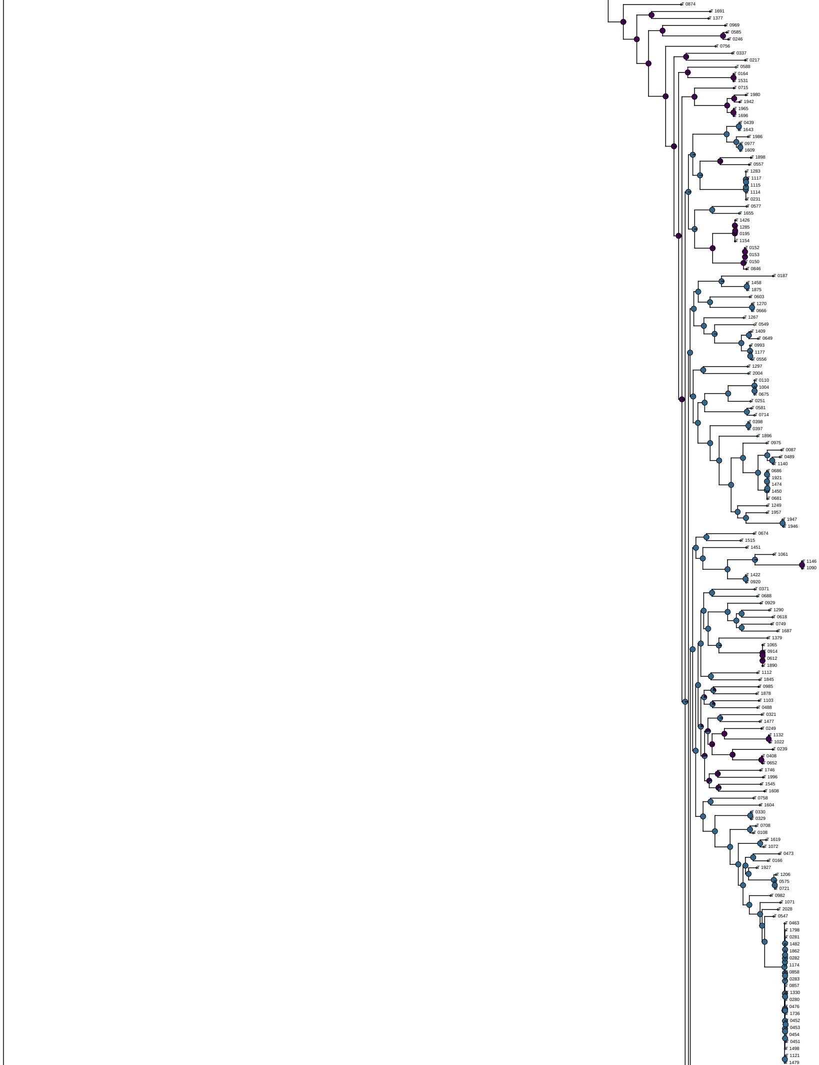
T 0874
T 1691
T 1377
T 0969
T 0585
T 0246
T 0756
T 0337
T 0217
T 0588
T 0164
T 1531
T 0715
T 1980
T 1942
T 1965
T 1696
T 0439
T 1643
T 1986
T 0977
T 1609
T 1898
T 0557
T 1283
T 1117
1115
T 1114
T 0231
T 0577
T 1655
T 1426
1285
0195
T 1154
T 0152
0153
T 0150
T 0846
T 0187
T 1458
T 1875
T 0603
T 1270
T 0666
T 1267
T 0549
T 1409
T 0649
T 0993
1177
T 0556
T 1297
T 2004
T 0110
1004
T 0675
T 0251
T 0581
T 0714
T 0398
T 0397
T 1896
T 0975
T 0087
T 0489
T 1140
T 0686
T 1921
1474
1450
T 0681
T 1249
T 1957
T 1947
T 1946
T 0674
T 1515
T 1451
T 1061
T 1146
T 1090
T 1422
T 0920
T 0371
T 0688
T 0929
T 1290
T 0618
T 0749
T 1687
T 1379
T 1065
T 0914
0612
T 1890
T 1112
T 1845
T 0985
T 1878
T 1103
T 0488
T 0321
T 1477
T 0249
T 1132
T 1022
T 0239
T 0408
T 0652
T 1746
T 1996
T 1545
T 1608
T 0758
T 1604
T 0330
0329
T 0708
T 0108
T 1619
T 1072
T 0473
T 0166
T 1927
T 1206
0575
T 0721
T 0982
T 1071
T 2028
T 0547
T 0463
T 1798
T 0281
1482
1862
0282
1174
0858
0283
T 0857
1330
0280
0476
T 1736
0452
0453
0454
0451
T 1498
T 1121
T 1479

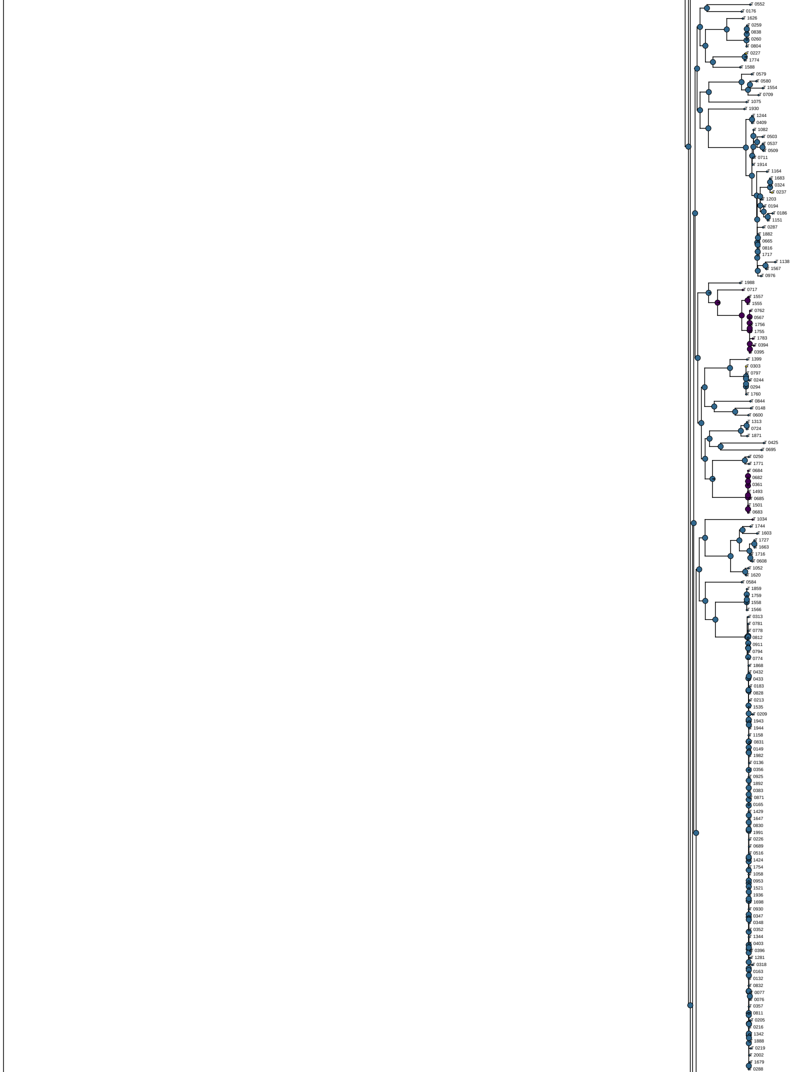
T 0552
T 0176
T 1626
T 0259
0838
0260
0804
T 0227
T 1774
T 1588
T 0579
T 0580
T 1554
T 0709
T 1075
T 1930
T 1244
T 0409
T 1082
T 0503
T 0537
T 0509
T 0711
T 1914
T 1164
T 1683
0324
T 0237
T 1203
T 0194
T 0186
1151
T 0287
T 1882
0665
0816
1717
T 1138
T 1567
T 0976
T 1988
T 0717
T 1557
1555
T 0762
0567
1756
1755
T 1783
T 0394
0395
T 1399
0303
0797
T 0244
0294
T 1760
T 0844
T 0148
T 0600
T 1313
0724
T 1871
T 0425
T 0695
T 0250
T 1771
T 0684
0682
0361
T 1493
T 0685
T 1501
0683
T 1034
T 1744
T 1603
T 1727
T 1663
T 1716
T 0608
T 1052
T 1620
T 0584
T 1859
1759
1558
T 1566
T 0313
T 0781
T 0778
0812
0911
0794
0774
T 1868
T 0432
0433
T 0183
0828
T 0213
1535
T 0209
1943
1944
T 1158
0831
0149
1982
T 0136
0356
T 0925
1892
0383
T 0871
0165
T 1429
1647
0830
1991
T 0226
T 0689
T 0516
1424
T 1754
T 1058
0953
1521
T 1936
1698
T 0930
0347
T 0348
T 0352
T 1344
0403
T 0396
T 1281
T 0318
0163
0132
T 0832
0077
T 0076
T 0357
0811
T 0205
0216
1342
T 1888
T 0219
T 2002
T 1679
T 0288

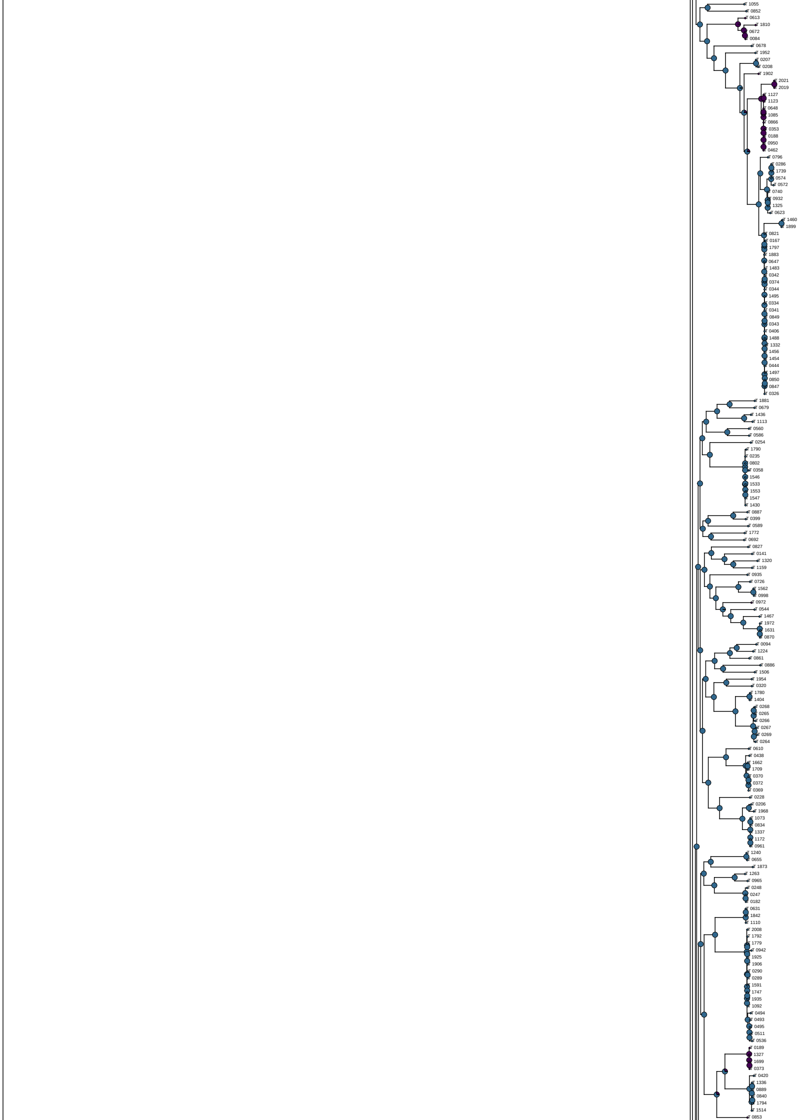
T 1055
T 0852
T 0613
T 1810
0672
T 0084
T 0678
T 1952
T 0207
T 0208
T 1902
T 2021
T 2019
T 1127
T 1123
T 0648
1085
T 0866
T 0353
T 0188
T 0950
T 0462
T 0796
T 0286
1739
0574
T 0572
0740
0932
1325
T 0623
T 1460
T 1899
T 0821
T 0167
1797
T 1883
0647
T 1483
T 0342
T 0374
T 0344
1495
T 0334
T 0341
T 0849
T 0343
T 0406
1488
T 1332
T 1456
T 1454
T 0444
T 1497
0850
T 0847
T 0326
T 1881
T 0679
T 1436
T 1113
T 0560
T 0586
T 0254
T 1790
T 0235
0802
T 0358
1546
1533
1553
1547
T 1430
T 0887
T 0399
T 0589
T 1772
T 0692
T 0827
T 0141
T 1320
T 1159
T 0935
T 0726
T 1562
T 0998
T 0972
T 0544
T 1467
T 1972
1631
T 0870
T 0094
T 1224
T 0861
T 0886
T 1506
T 1954
T 0320
T 1780
T 1404
T 0268
0265
T 0266
T 0267
T 0269
T 0264
T 0610
T 0438
T 1662
1709
T 0370
0372
T 0369
T 0228
T 0206
T 1968
T 1073
T 0834
1337
T 1172
T 0961
T 1240
T 0655
T 1873
T 1263
T 0965
T 0248
0247
T 0182
T 0631
1842
T 1110
T 2008
T 1792
T 1779
T 0942
1925
T 1906
T 0290
T 0289
T 1591
T 1747
T 1935
T 1092
T 0494
T 0493
T 0495
T 0511
T 0536
T 0189
1327
1699
T 0373
T 0420
T 1336
0889
T 0840
T 1794
T 1514
T 0853

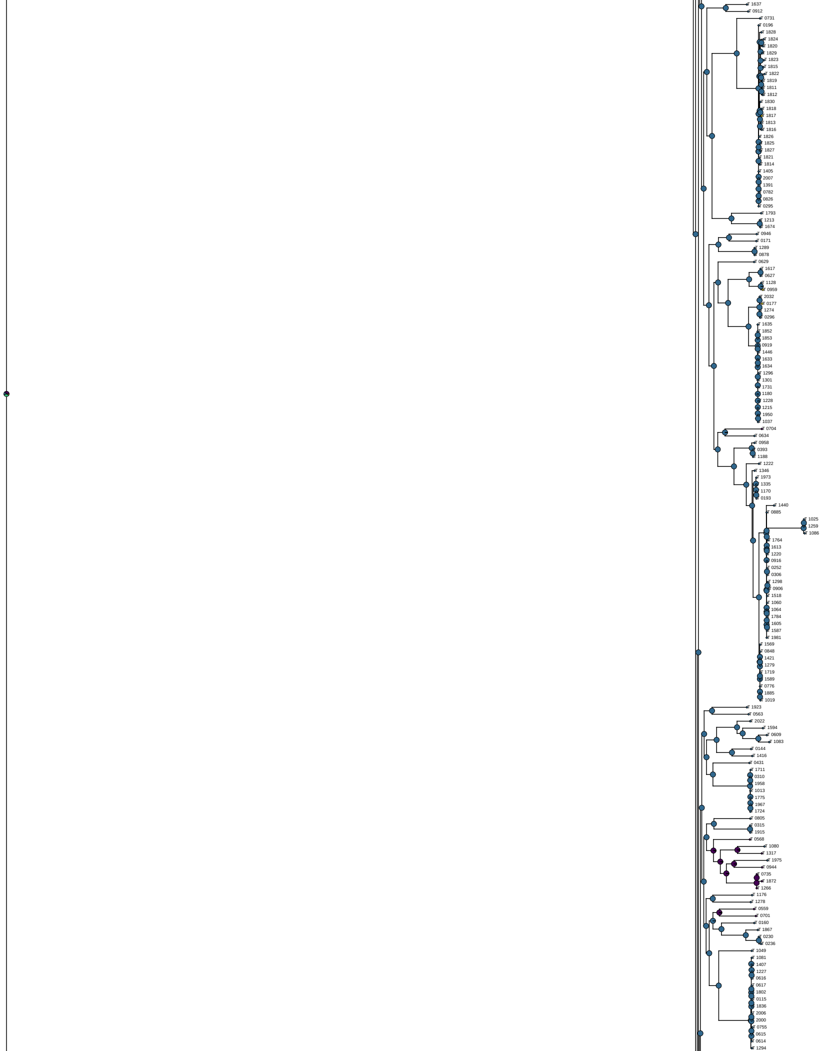
T 1637
T 0912
T 0731
T 0196
T 1828
T 1824
T 1820
T 1829
T 1823
T 1815
T 1822
T 1819
T 1811
T 1812
T 1830
T 1818
T 1817
T 1813
T 1816
T 1826
T 1825
T 1827
T 1821
T 1814
T 1405
T 2007
T 1391
T 0782
T 0826
T 0295
T 1793
T 1213
T 1674
T 0946
T 0171
T 1289
T 0878
T 0629
T 1617
T 0627
T 1128
T 0959
T 2032
T 0177
T 1274
T 0296
T 1635
T 1852
T 1853
T 0919
T 1446
T 1633
T 1634
T 1296
T 1301
T 1731
T 1180
T 1228
T 1215
T 1950
T 1037
T 0704
T 0634
T 0958
T 0393
T 1188
T 1222
T 1346
T 1973
T 1335
T 1170
T 0193
T 1440
T 0885
T 1025
T 1259
T 1086
T 1764
T 1613
T 1220
T 0916
T 0252
T 0306
T 1298
T 0906
T 1518
T 1060
T 1064
T 1784
T 1605
T 1587
T 1981
T 1569
T 0848
T 1421
T 1279
T 1719
T 1589
T 0776
T 1885
T 1019
T 1923
T 0563
T 2022
T 1594
T 0609
T 1083
T 0144
T 1416
T 0431
T 1711
T 0310
T 1958
T 1013
T 1775
T 1967
T 1724
T 0805
T 0315
T 1915
T 0568
T 1080
T 1317
T 1975
T 0944
T 0735
T 1872
T 1266
T 1176
T 1278
T 0559
T 0701
T 0160
T 1867
T 0230
T 0236
T 1049
T 1081
T 1407
T 1227
T 0616
T 0617
T 1802
T 0115
T 1836
T 2006
T 2000
T 0755
T 0615
T 0614
T 1294

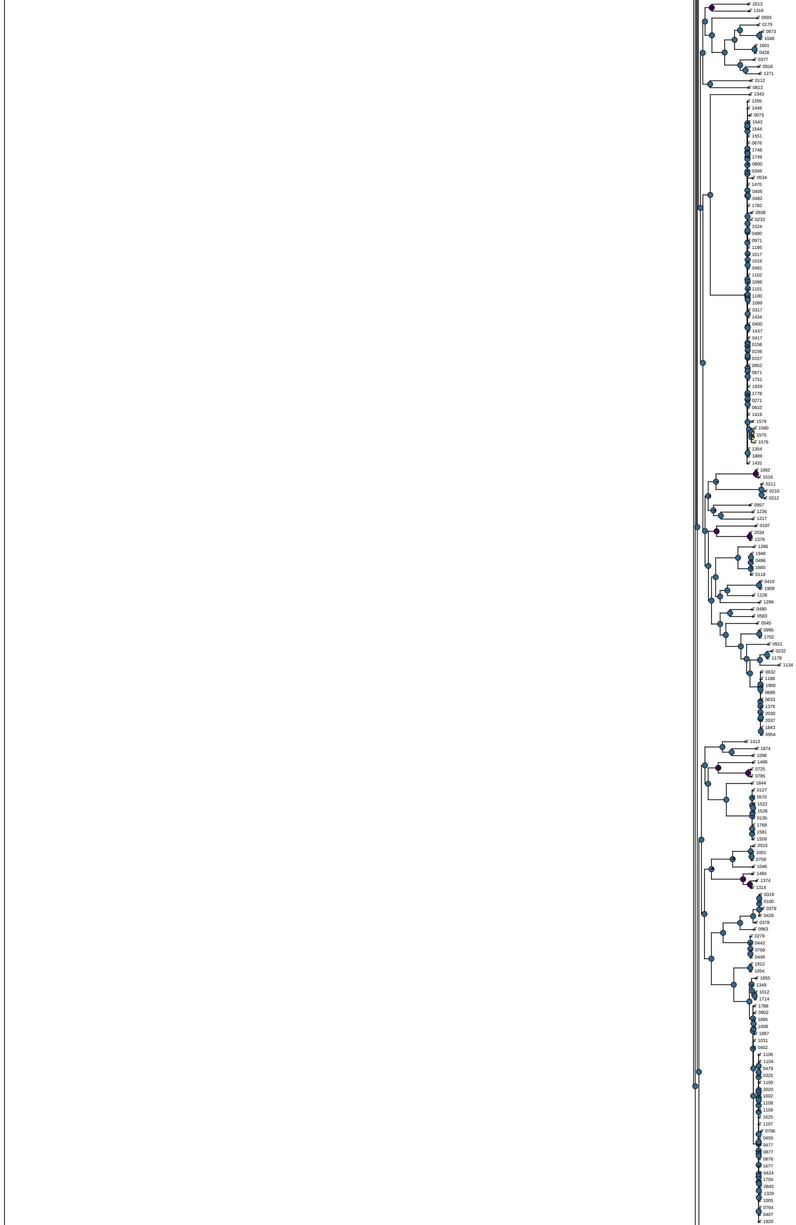
T 2013
T 1316
T 0593
T 0179
T 0973
T 1048
T 1601
T 0428
T 0377
T 0918
T 1271
T 0112
T 0813
T 1343
T 1295
T 1449
T 0073
T 1543
T 1544
T 1551
T 0676
T 1748
T 1749
T 0800
T 0349
T 0534
T 1470
T 0405
T 0482
T 1782
T 0938
T 0233
T 1524
T 0480
T 0971
T 1185
T 1017
T 1016
T 0481
T 1102
T 1098
T 1101
T 1100
T 1099
T 0317
T 1434
T 0400
T 1437
T 0417
T 0158
T 0156
T 0157
T 0952
T 0671
T 1751
T 1929
T 1778
T 0271
T 0810
T 1419
T 1579
T 1580
T 1575
T 1576
T 1354
T 1889
T 1431
T 1062
T 1516
T 0211
T 0210
T 0212
T 0957
T 1236
T 1217
T 0197
T 2034
T 1378
T 1286
T 1948
T 0486
T 1685
T 0118
T 0410
T 1909
T 1126
T 1299
T 0490
T 0583
T 0545
T 0995
T 1702
T 0921
T 0232
T 1178
T 1134
T 0632
T 1186
T 1900
T 0699
T 0633
T 1376
T 2030
T 2037
T 1843
T 0954
T 1413
T 1874
T 1096
T 1499
T 0725
T 0785
T 1844
T 0127
T 0570
T 1522
T 1528
T 0135
T 1768
T 1581
T 1509
T 2015
T 1001
T 0759
T 1046
T 1484
T 1374
T 1314
T 0319
T 0100
T 0379
T 0426
T 0378
T 0963
T 0279
T 0443
T 0769
T 0449
T 1912
T 1054
T 1855
T 1349
T 1012
T 1714
T 1788
T 0902
T 1895
T 1008
T 1887
T 1031
T 0402
T 1106
T 1104
T 0479
T 0325
T 1105
T 2020
T 1002
T 1108
T 1109
T 1625
T 1107
T 0706
T 0455
T 0477
T 0877
T 0876
T 1677
T 0424
T 1704
T 0845
T 1329
T 1005
T 0764
T 0407
T 1920

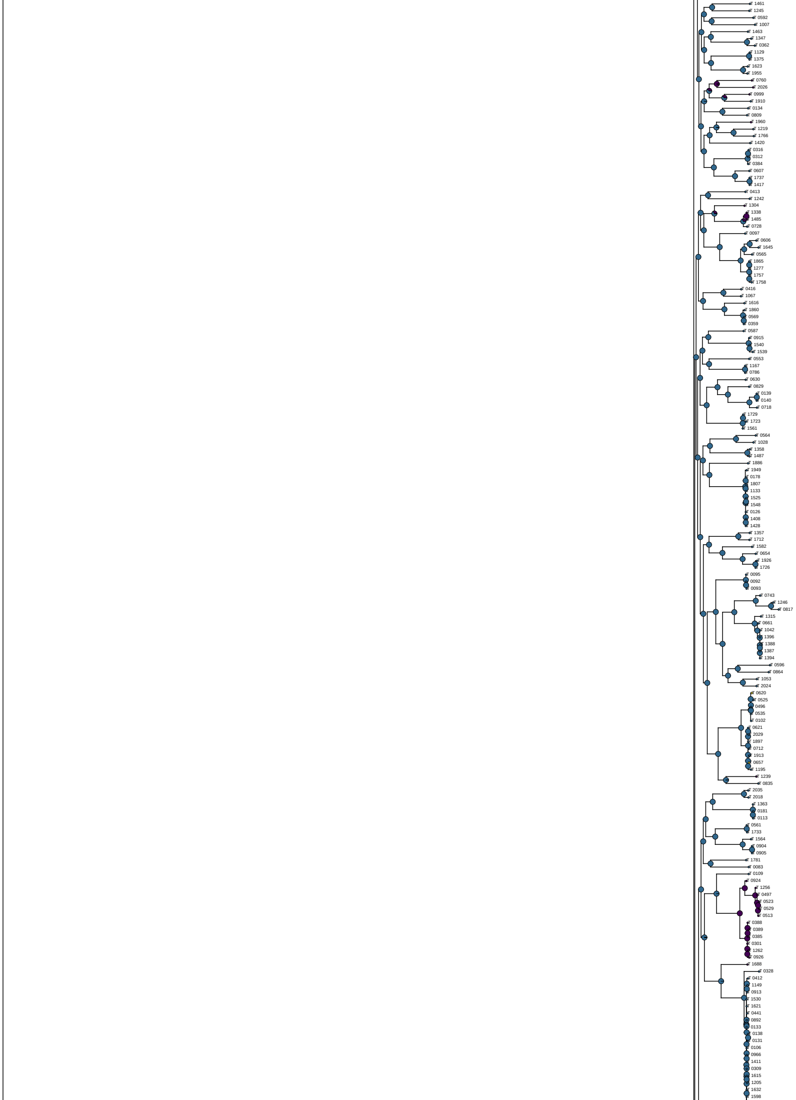

T 1461
T 1245
T 0592
T 1007
T 1463
T 1347
T 0362
T 1129
T 1375
T 1623
T 1955
T 0760
T 2026
T 0999
T 1910
T 0134
T 0809
T 1960
T 1219
T 1766
T 1420
T 0316
T 0312
T 0384
T 0607
T 1737
T 1417
T 0413
T 1242
T 1304
T 1338
T 1485
T 0728
T 0097
T 0606
T 1645
T 0565
T 1865
1277
T 1757
T 1758
T 0416
T 1067
T 1616
T 1860
0569
0359
T 0587
T 0915
1540
T 1539
T 0553
T 1167
T 0786
T 0630
T 0829
T 0139
T 0140
T 0718
T 1729
T 1723
T 1561
T 0564
T 1028
T 1358
T 1487
T 1886
T 1949
T 0178
1807
1133
1525
1548
T 0126
1408
1428
T 1357
T 1712
T 1582
T 0654
T 1926
1726
T 0095
0092
T 0093
T 0743
T 1246
T 0817
T 1315
T 0661
T 1042
1396
T 1388
1387
T 1394
T 0596
T 0864
T 1053
T 2024
T 0620
T 0525
0496
0535
T 0102
T 0621
2029
1897
T 0712
T 1913
0657
T 1195
T 1239
T 0835
T 2035
T 2018
T 1363
0181
T 0113
T 0561
T 1733
T 1564
T 0904
T 0905
T 1781
T 0083
T 0109
T 0924
T 1256
T 0497
T 0523
0529
T 0513
T 0388
0389
0385
T 0301
1262
T 0926
T 1688
T 0328
T 0412
1149
0913
T 1530
T 1621
T 0441
0892
0133
T 0138
T 0131
T 0106
0966
1411
0309
1615
1205
T 1632
T 1598

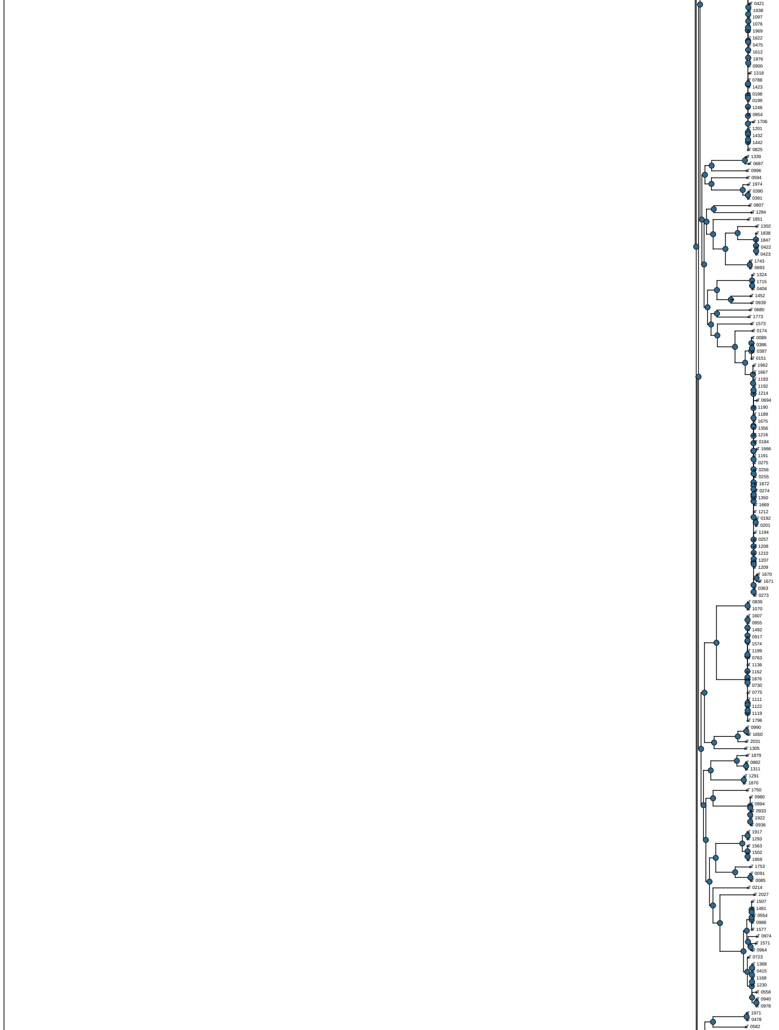
T 0421
T 1938
T 1097
T 1076
T 1969
T 1622
T 0475
T 1612
T 1976
T 0900
T 1318
T 0788
T 1423
T 0198
T 0199
T 1248
T 0854
T 1706
T 1201
T 1432
T 1442
T 0825
T 1339
T 0687
T 0996
T 0594
T 1974
T 0390
T 0391
T 0807
T 1284
T 1851
T 1302
T 1838
T 1847
T 0422
T 0423
T 1743
T 0693
T 1324
T 1715
T 0404
T 1452
T 0939
T 0680
T 1773
T 1573
T 0174
T 0089
T 0386
T 0387
T 0151
T 1962
T 1667
T 1193
T 1192
T 1214
T 0694
T 1190
T 1189
T 1675
T 1356
T 1216
T 0184
T 1666
T 1191
T 0275
T 0256
T 0255
T 1672
T 0274
T 1350
T 1669
T 1212
T 0192
T 0201
T 1194
T 0257
T 1208
T 1210
T 1207
T 1209
T 1670
T 1671
T 0363
T 0273
T 0839
T 1070
T 1607
T 0955
T 1492
T 0917
T 1574
T 1199
T 0763
T 1136
T 1162
T 1876
T 0730
T 0775
T 1111
T 1122
T 1119
T 1796
T 0990
T 1650
T 2031
T 1305
T 1879
T 0882
T 1311
T 1291
T 1870
T 1750
T 0980
T 0994
T 0933
T 1922
T 0936
T 1917
T 1293
T 1563
T 1502
T 1959
T 1753
T 0091
T 0085
T 0214
T 2027
T 1507
T 1481
T 0554
T 0988
T 1577
T 0974
T 1571
T 0964
T 0723
T 1368
T 0415
T 1168
T 1230
T 0558
T 0940
T 0978
T 1971
T 0478
T 0582

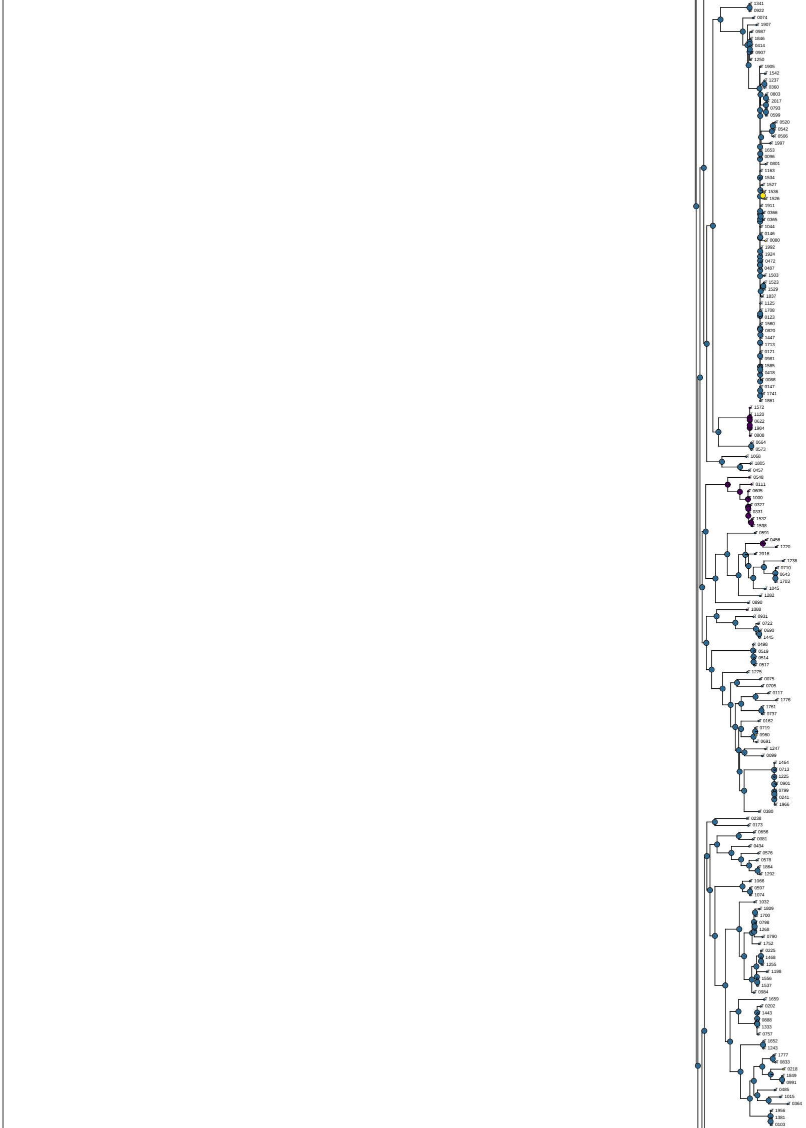
T 1341
T 0922
T 0074
T 1907
T 0987
T 1846
T 0414
T 0907
T 1250
T 1905
T 1542
T 1237
T 0360
T 0803
T 2017
T 0793
T 0599
T 0520
T 0542
T 0506
T 1997
T 1653
T 0096
T 0801
T 1163
T 1534
T 1527
T 1536
T 1526
T 1911
T 0366
T 0365
T 1044
T 0146
T 0080
T 1992
T 1924
T 0472
T 0487
T 1503
T 1523
T 1529
T 1837
T 1125
T 1708
T 0123
T 1560
T 0820
T 1447
T 1713
T 0121
T 0981
T 1585
T 0418
T 0088
T 0147
T 1741
T 1861
T 1572
T 1120
T 0622
T 1984
T 0808
T 0664
T 0573
T 1068
T 1805
T 0457
T 0548
T 0111
T 0605
T 1000
T 0327
T 0331
T 1532
T 1538
T 0591
T 0456
T 1720
T 2016
T 1238
T 0710
T 0643
T 1703
T 1045
T 1282
T 0890
T 1088
T 0931
T 0722
T 0690
T 1445
T 0498
T 0519
T 0514
T 0517
T 1275
T 0075
T 0705
T 0117
T 1776
T 1761
T 0737
T 0162
T 0719
T 0960
T 0691
T 1247
T 0099
T 1464
T 0713
T 1225
T 0901
T 0799
T 0241
T 1966
T 0380
T 0238
T 0173
T 0656
T 0081
T 0434
T 0576
T 0578
T 1864
T 1292
T 1066
T 0597
T 1074
T 1032
T 1809
T 1700
T 0798
T 1268
T 0790
T 1752
T 0225
T 1468
T 1255
T 1198
T 1556
T 1537
T 0984
T 1659
T 0202
T 1443
T 0888
T 1333
T 0757
T 1652
T 1243
T 1777
T 0833
T 0218
T 1849
T 0991
T 0485
T 1015
T 0364
T 1956
T 1381
T 0103

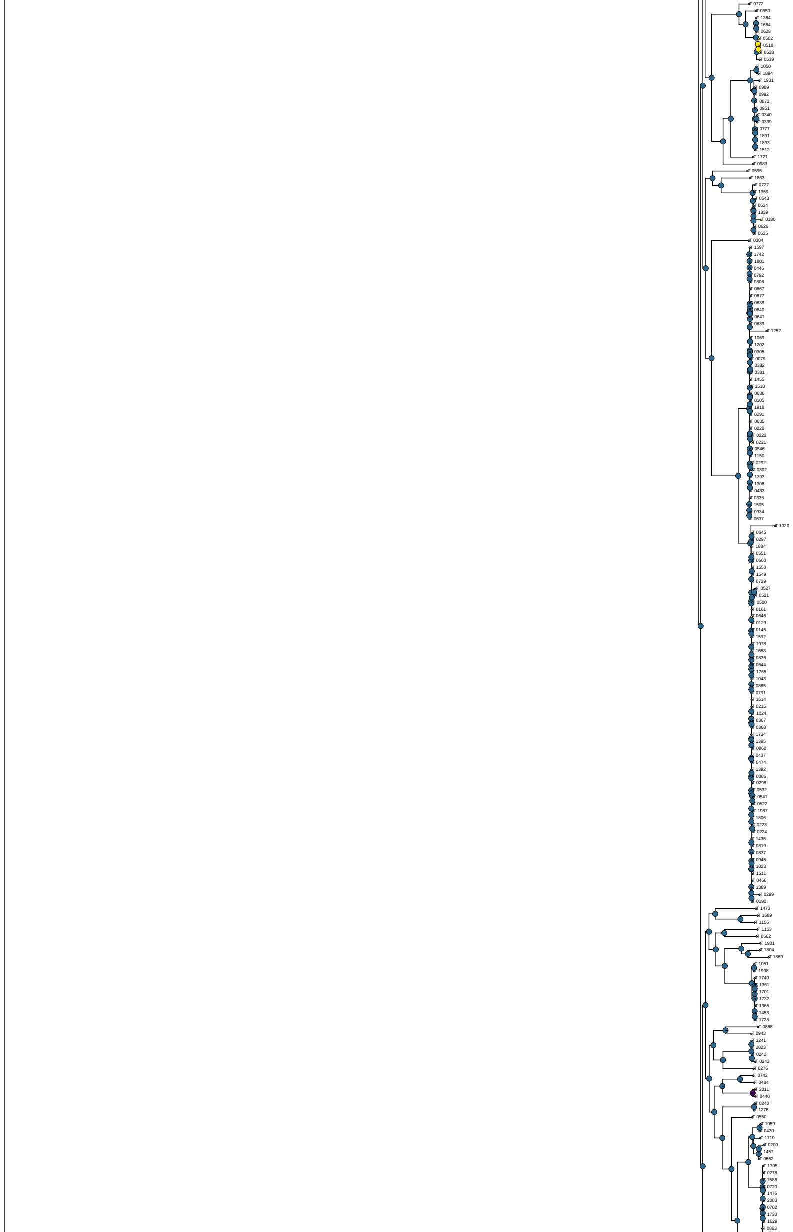
T 0772
T 0650
T 1364
1664
0628
T 0502
0518
T 0528
T 0539
T 1050
T 1894
T 1931
T 0989
0992
0872
0951
T 0340
T 0339
0777
1891
1893
1512
T 1721
T 0983
T 0595
T 1863
T 0727
T 1359
T 0543
0624
1839
T 0180
T 0626
0625
T 0304
T 1597
1742
1801
0446
0792
0806
T 0867
T 0677
0638
0640
0641
T 0639
T 1252
T 1069
T 1202
0305
T 0079
T 0382
0381
T 1455
T 1510
T 0636
0105
1918
T 0291
T 0635
T 0220
T 0222
T 0221
0546
1150
T 0292
T 0302
1393
1306
T 0483
T 0335
1505
0934
T 0637
T 1020
T 0645
0297
T 1884
T 0551
0660
T 1550
1549
0729
T 0527
T 0521
T 0500
T 0161
T 0646
0129
0145
1592
T 1978
1658
0836
0644
1765
T 1043
0865
T 0791
T 1614
T 0215
1024
0367
0368
T 1734
1395
T 0860
T 0437
T 0474
T 1392
0086
T 0298
T 0532
0541
T 0522
T 1987
1806
0223
T 0224
T 1435
T 0819
0837
0945
1023
T 1511
T 0466
1389
T 0299
T 0190
T 1473
T 1689
T 1156
T 1153
T 0562
T 1901
T 1804
T 1869
T 1051
1998
T 1740
1361
1701
1732
T 1365
1453
T 1728
T 0868
T 0943
T 1241
2023
0242
T 0243
T 0276
T 0742
T 0484
T 2011
T 0440
T 0240
1276
T 0550
T 1059
T 0430
T 1710
T 0200
1457
T 0662
T 1705
T 0278
1586
0720
1476
2003
0702
1730
1629
T 0863

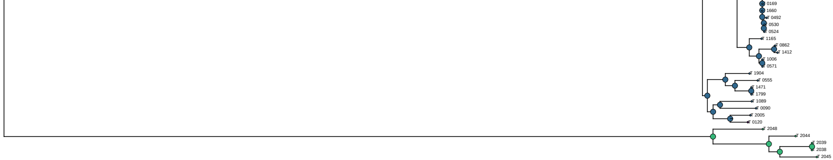

0169
1660
T 0492
T 0530
T 0524
T 1165
T 0862
T 1412
T 1006
T 0571
T 1904
T 0555
T 1471
T 1799
T 1089
T 0090
T 2005
T 0120
T 2048
T 2044
T 2039
T 2038
T 2045

Locus: PA0099

Effector genes: tse7, tse7a, tsd1 (tse7b), tse7c, tse7d, tse7e

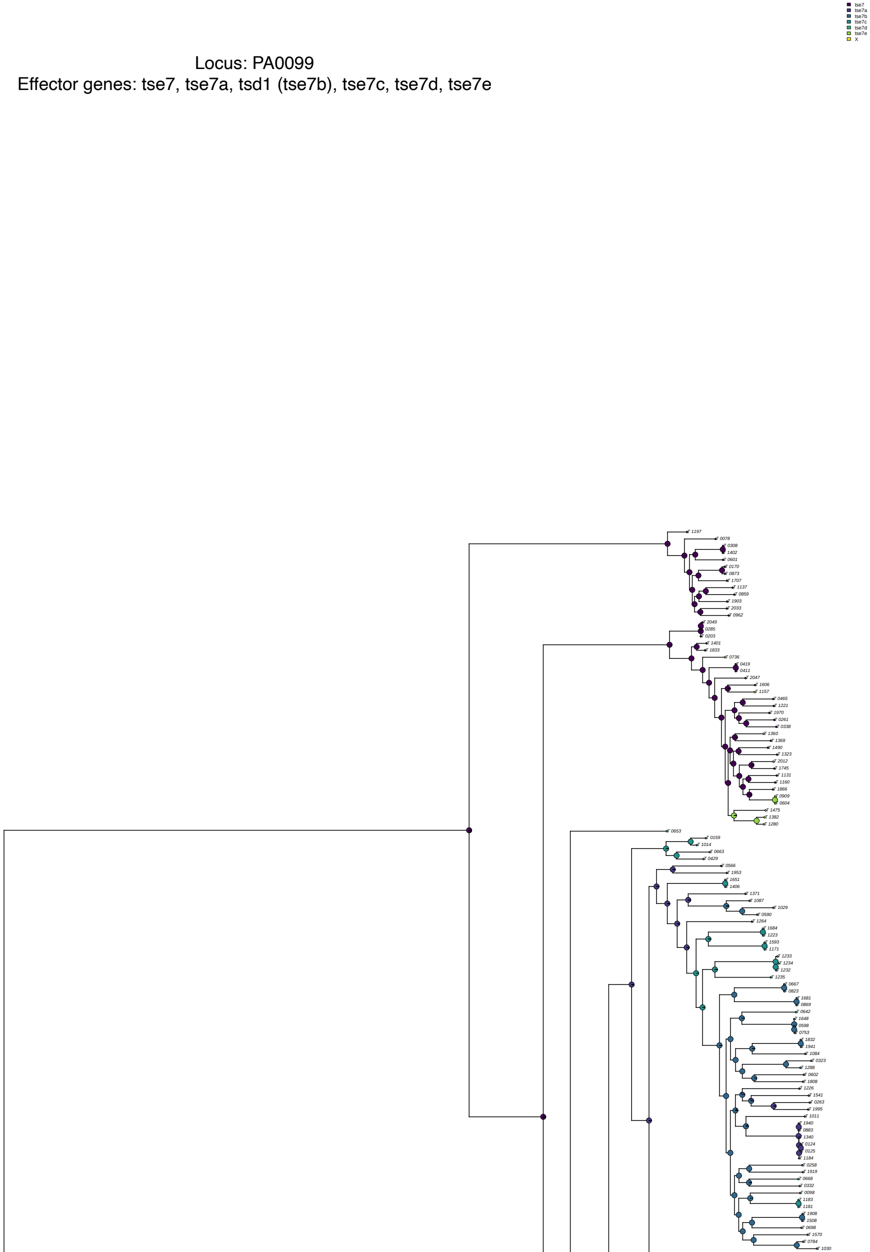

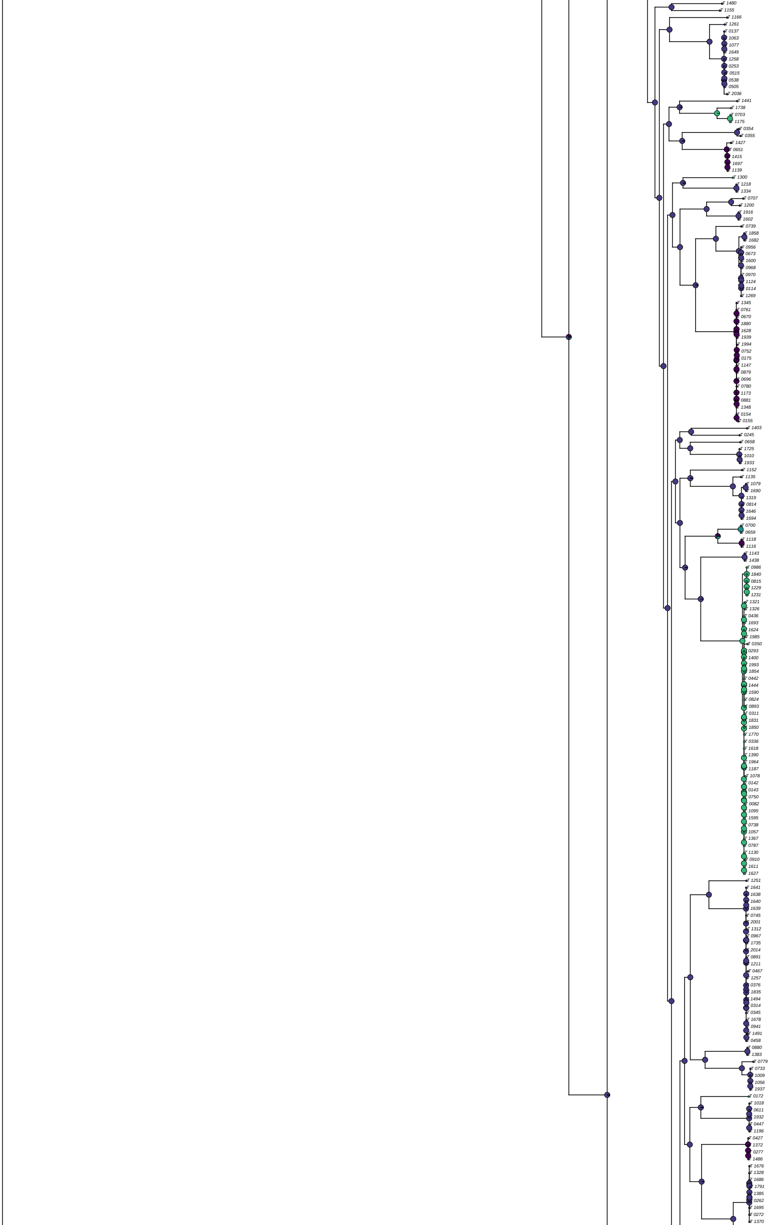

1480
1155
1166
1261
0137
1063
1077
1649
1258
0253
0515
0538
0505
2036
1441
1738
0703
1175
0354
0355
1427
0651
1415
1697
1139
1300
1218
1334
0707
1200
1916
1602
0739
1858
1682
0956
0673
1600
0968
0970
1124
0114
1269
1345
0761
0670
1880
1628
1939
1994
0752
0175
1147
0879
0696
0780
1173
0881
1348
0154
0155
1403
0245
0658
1725
1010
1933
1152
1135
1079
1690
1319
0814
1646
1694
0700
0659
1118
1116
1143
1438
0986
1840
0815
1229
1231
1321
1326
0436
1693
1624
1985
0350
0293
1400
1993
1854
0442
1444
1590
0824
0893
0311
1831
1850
1770
0336
1618
1390
1964
1187
1078
0142
0143
0750
0082
1095
1595
0738
1057
1367
0787
1130
0910
1611
1627
1251
1641
1638
1640
1639
0745
2001
1312
0967
1735
2014
0891
1211
0467
1257
0376
1835
1494
0314
0345
1678
0941
1491
0458
0880
1383
0779
0733
1009
1056
1937
0172
1018
0611
1932
0447
1196
0427
1372
0277
1486
1676
1328
1686
1791
1385
0262
1695
0272
1370

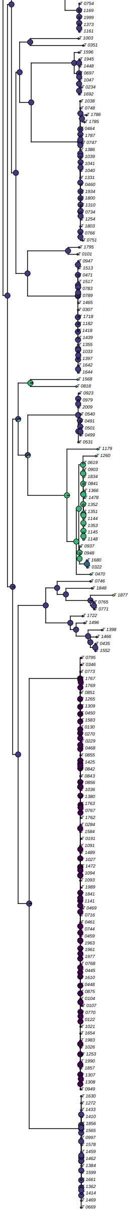
T 0754
T 1169
T 1999
T 1373
T 1161
T 1003
T 0351
T 1596
T 1945
T 1448
T 0697
T 1047
T 0234
T 1692
T 1038
T 0748
T 1786
T 1785
T 0464
T 1787
T 0747
T 1386
T 1039
T 1041
T 1040
T 1331
T 0460
T 1934
T 1800
T 1310
T 0734
T 1254
T 1803
T 0766
T 0751
T 1795
T 0101
T 0947
T 1513
T 0471
T 1517
T 0783
T 0789
T 1465
T 0307
T 1718
T 1182
T 1418
T 1439
T 1355
T 1033
T 1397
T 1642
T 1644
T 1568
T 0818
T 0923
T 0979
T 2009
T 0540
T 0491
T 0501
T 0499
T 0531
T 1179
T 1260
T 0619
T 0903
T 1834
T 0841
T 1366
T 1478
T 1352
T 1351
T 1144
T 1353
T 1145
T 1148
T 0937
T 0948
T 1680
T 0322
T 0470
T 0746
T 1848
T 1877
T 0765
T 0771
T 1722
T 1496
T 1398
T 1466
T 0435
T 1552
T 0795
T 0346
T 0773
T 1767
T 1769
T 0851
T 1265
T 1309
T 0450
T 1583
T 0130
T 0270
T 0229
T 0468
T 0855
T 1425
T 0842
T 0843
T 0856
T 1036
T 1380
T 1763
T 0767
T 1762
T 0284
T 1584
T 0191
T 1091
T 1489
T 1027
T 1472
T 1094
T 1093
T 1989
T 1841
T 1141
T 0469
T 0716
T 0461
T 0744
T 0459
T 1963
T 1961
T 1977
T 0768
T 0445
T 1610
T 0448
T 0875
T 0104
T 0107
T 0770
T 0122
T 1021
T 1654
T 1983
T 1026
T 1253
T 1990
T 1857
T 1307
T 1308
T 0949
T 1630
T 1272
T 1433
T 1410
T 1856
T 1565
T 0997
T 1578
T 1459
T 1462
T 1384
T 1599
T 1661
T 1362
T 1414
T 1469
T 0669

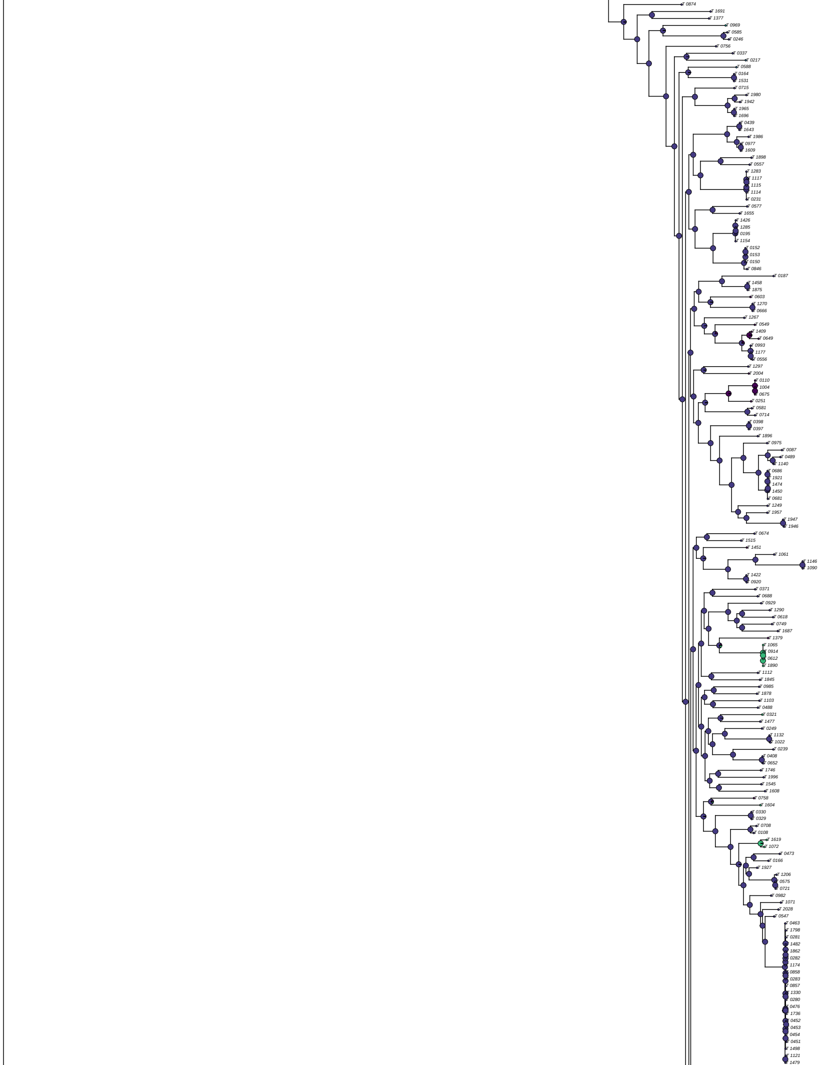
T 0874
T 1691
T 1377
T 0969
T 0585
T 0246
T 0756
T 0337
T 0217
T 0588
T 0164
T 1531
T 0715
T 1980
T 1942
T 1965
T 1696
T 0439
T 1643
T 1986
T 0977
T 1609
T 1898
T 0557
T 1283
T 1117
T 1115
T 1114
T 0231
T 0577
T 1655
T 1426
T 1285
T 0195
T 1154
T 0152
T 0153
T 0150
T 0846
T 0187
T 1458
T 1875
T 0603
T 1270
T 0666
T 1267
T 0549
T 1409
T 0649
T 0993
T 1177
T 0556
T 1297
T 2004
T 0110
T 1004
T 0675
T 0251
T 0581
T 0714
T 0398
T 0397
T 1896
T 0975
T 0087
T 0489
T 1140
T 0686
T 1921
T 1474
T 1450
T 0681
T 1249
T 1957
T 1947
T 1946
T 0674
T 1515
T 1451
T 1061
T 1146
T 1090
T 1422
T 0920
T 0371
T 0688
T 0929
T 1290
T 0618
T 0749
T 1687
T 1379
T 1065
T 0914
T 0612
T 1890
T 1112
T 1845
T 0985
T 1878
T 1103
T 0488
T 0321
T 1477
T 0249
T 1132
T 1022
T 0239
T 0408
T 0652
T 1746
T 1996
T 1545
T 1608
T 0758
T 1604
T 0330
T 0329
T 0708
T 0108
T 1619
T 1072
T 0473
T 0166
T 1927
T 1206
T 0575
T 0721
T 0982
T 1071
T 2028
T 0547
T 0463
T 1798
T 0281
T 1482
T 1862
T 0282
T 1174
T 0858
T 0283
T 0857
T 1330
T 0280
T 0476
T 1736
T 0452
T 0453
T 0454
T 0451
T 1498
T 1121
T 1479

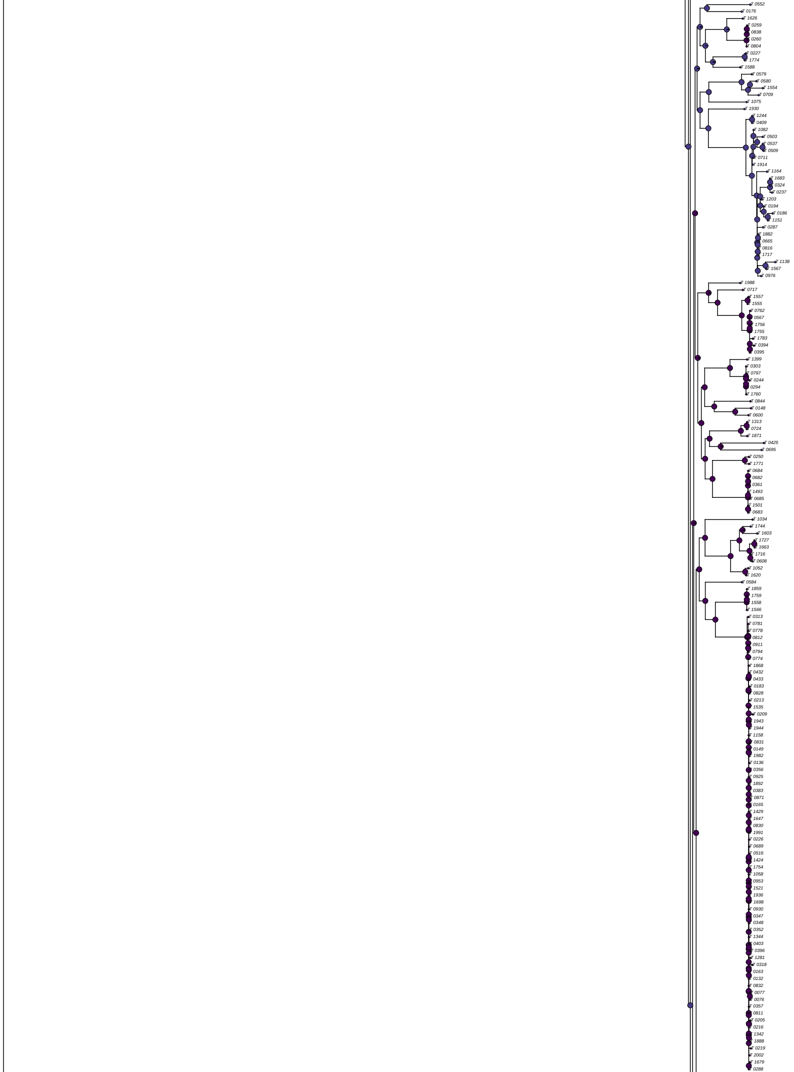
T 0552
T 0176
T 1626
T 0259
0838
0260
T 0804
T 0227
T 1774
T 1588
T 0579
T 0580
T 1554
T 0709
T 1075
T 1930
T 1244
T 0409
T 1082
T 0503
T 0537
T 0509
T 0711
T 1914
T 1164
T 1683
0324
T 0237
T 1203
T 0194
T 0186
1151
T 0287
T 1882
0665
T 0816
1717
T 1138
T 1567
T 0976
T 1988
T 0717
T 1557
T 1555
T 0762
0567
1756
1755
T 1783
T 0394
T 0395
T 1399
T 0303
T 0797
T 0244
0294
T 1760
T 0844
T 0148
T 0600
T 1313
T 0724
T 1871
T 0425
T 0695
T 0250
T 1771
T 0684
0682
0361
T 1493
T 0685
T 1501
T 0683
T 1034
T 1744
T 1603
T 1727
T 1663
T 1716
T 0608
T 1052
T 1620
T 0584
T 1859
1759
1558
T 1566
T 0313
T 0781
T 0778
0812
0911
T 0794
0774
T 1868
T 0432
0433
T 0183
0828
T 0213
1535
T 0209
1943
T 1944
T 1158
0831
0149
T 1982
T 0136
0356
T 0925
1892
0383
T 0871
0165
T 1429
1647
0830
1991
T 0226
T 0689
T 0516
1424
T 1754
T 1058
0953
1521
1936
1698
T 0930
0347
0348
0352
T 1344
0403
T 0396
T 1281
T 0318
0163
0132
T 0832
T 0077
T 0076
T 0357
0811
T 0205
T 0216
1342
T 1888
T 0219
T 2002
T 1679
T 0288

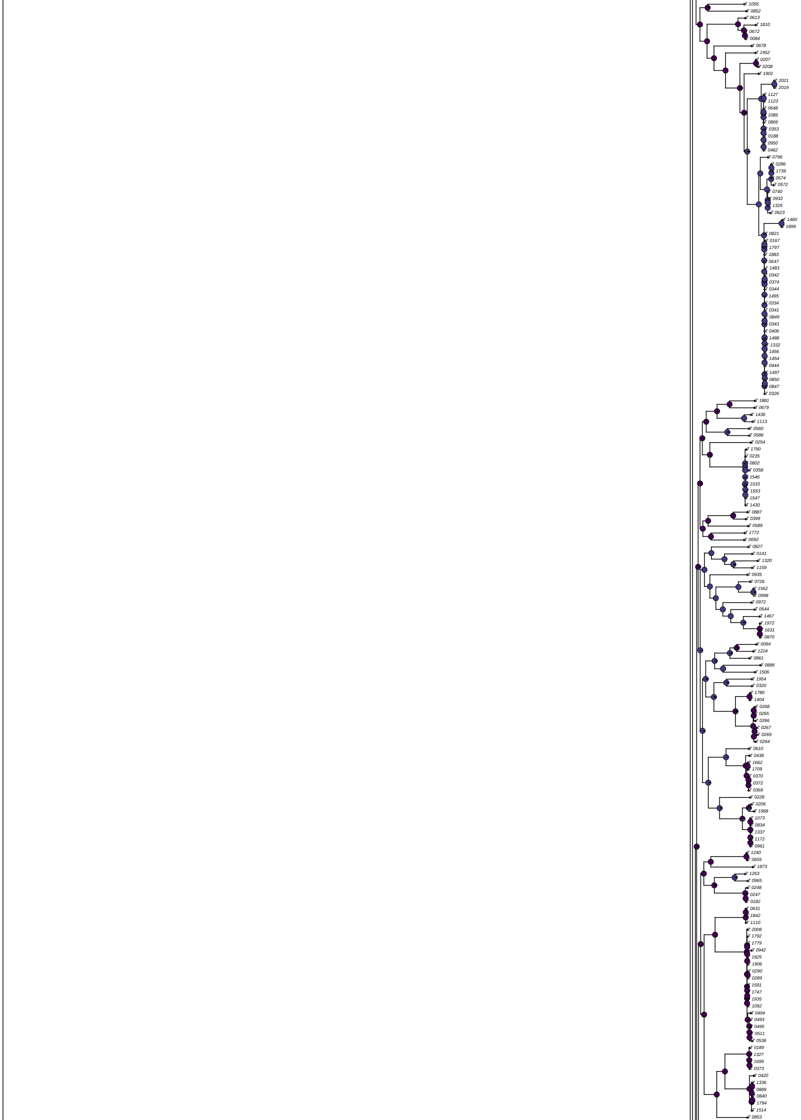
T 1055
T 0852
T 0613
T 1810
0672
T 0084
T 0678
T 1952
T 0207
T 0208
T 1902
T 2021
T 2019
T 1127
1123
T 0648
1085
T 0866
T 0353
T 0188
0950
T 0462
T 0796
T 0286
1739
T 0574
T 0572
T 0740
T 0932
1325
T 0623
T 1460
T 1899
T 0821
T 0167
1797
T 1883
0647
T 1483
T 0342
T 0374
T 0344
1495
T 0334
T 0341
T 0849
T 0343
T 0406
1488
T 1332
1456
T 1454
T 0444
T 1497
T 0850
T 0847
T 0326
T 1881
T 0679
T 1436
T 1113
T 0560
T 0586
T 0254
T 1790
T 0235
0802
T 0358
1546
1533
1553
T 1547
T 1430
T 0887
T 0399
T 0589
T 1772
T 0692
T 0827
T 0141
T 1320
T 1159
T 0935
T 0726
T 1562
T 0998
T 0972
T 0544
T 1467
T 1972
1631
T 0870
T 0094
T 1224
T 0861
T 0886
T 1506
T 1954
T 0320
T 1780
T 1404
T 0268
T 0265
T 0266
T 0267
T 0269
T 0264
T 0610
T 0438
T 1662
T 1709
T 0370
0372
T 0369
T 0228
T 0206
T 1968
T 1073
T 0834
1337
1172
T 0961
T 1240
T 0655
T 1873
T 1263
T 0965
T 0248
0247
T 0182
T 0631
1842
T 1110
T 2008
T 1792
T 1779
T 0942
1925
T 1906
T 0290
T 0289
T 1591
T 1747
T 1935
T 1092
T 0494
T 0493
T 0495
T 0511
T 0536
T 0189
1327
1699
T 0373
T 0420
T 1336
T 0889
T 0840
T 1794
T 1514
T 0853

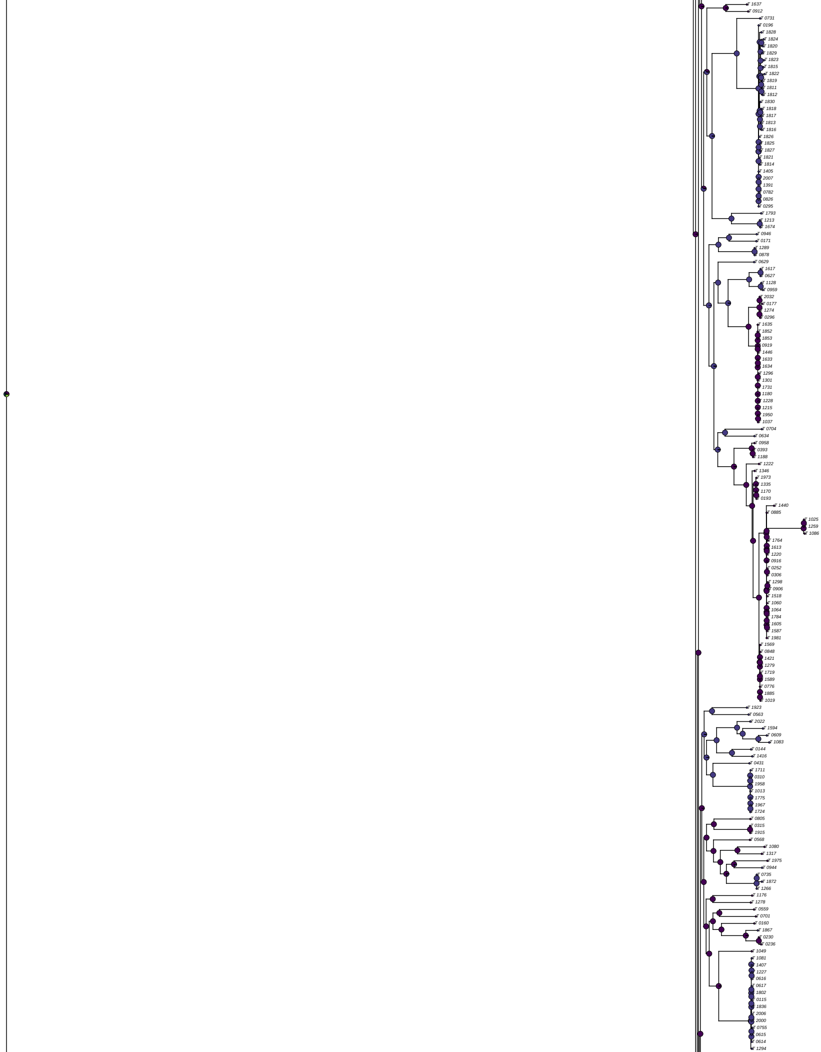

T 1637
T 0912
T 0731
T 0196
T 1828
T 1824
T 1820
T 1829
T 1823
T 1815
T 1822
T 1819
T 1811
T 1812
T 1830
T 1818
T 1817
T 1813
T 1816
T 1826
T 1825
T 1827
T 1821
T 1814
T 1405
2007
1391
0782
0826
T 0295
T 1793
T 1213
T 1674
T 0946
T 0171
T 1289
T 0878
T 0629
T 1617
T 0627
T 1128
T 0959
T 2032
T 0177
T 1274
T 0296
T 1635
T 1852
1853
0919
T 1446
1633
1634
T 1296
T 1301
1731
1180
T 1228
1215
1950
T 1037
T 0704
T 0634
T 0958
0393
T 1188
T 1222
T 1346
T 1973
1335
1170
T 0193
T 1440
T 0885
T 1025
1259
T 1086
T 1764
1613
T 1220
0916
T 0252
0306
T 1298
T 0906
T 1518
T 1060
1064
T 1784
1605
T 1587
T 1981
T 1569
T 0848
1421
1279
T 1719
1589
T 0776
1885
T 1019
T 1923
T 0563
T 2022
T 1594
T 0609
T 1083
T 0144
T 1416
T 0431
T 1711
0310
1958
T 1013
1775
1967
1724
T 0805
T 0315
T 1915
T 0568
T 1080
T 1317
T 1975
T 0944
T 0735
T 1872
T 1266
T 1176
T 1278
T 0559
T 0701
T 0160
T 1867
T 0230
T 0236
T 1049
T 1081
1407
1227
0616
T 0617
1802
T 0115
1836
T 2006
2000
T 0755
0615
T 0614
T 1294

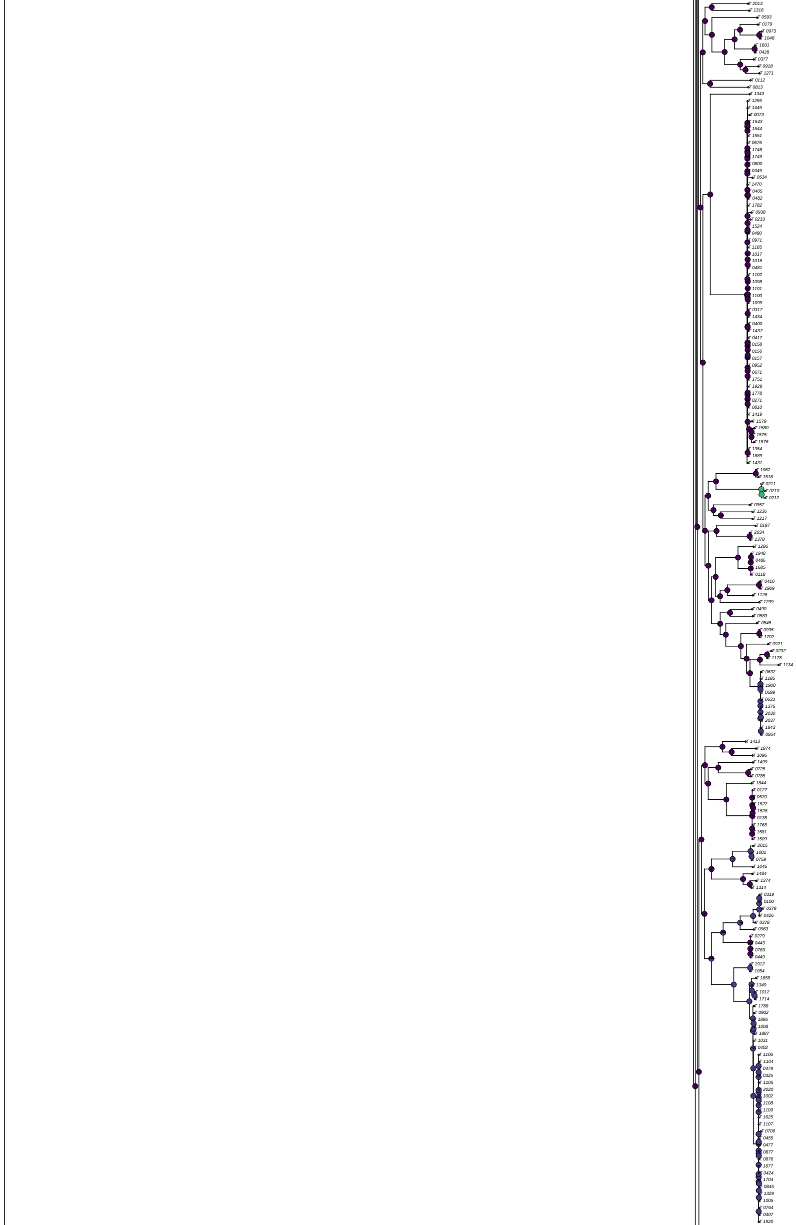

T 2013
T 1316
T 0593
T 0179
T 0973
1048
T 1601
0428
T 0377
T 0918
T 1271
T 0112
T 0813
T 1343
T 1295
T 1449
T 0073
1543
1544
T 1551
T 0676
1748
1749
0800
0349
T 0534
T 1470
0405
0482
T 1782
T 0938
T 0233
1524
0480
0971
T 1185
1017
1016
0481
T 1102
1098
1101
1100
1099
T 0317
1434
T 0400
1437
T 0417
0158
0156
0157
T 0952
0671
1751
T 1929
1778
0271
0810
T 1419
T 1579
T 1580
1575
T 1576
T 1354
1889
T 1431
T 1062
T 1516
T 0211
T 0210
T 0212
T 0957
T 1236
T 1217
T 0197
T 2034
1378
T 1286
T 1948
0486
1685
T 0118
T 0410
T 1909
T 1126
T 1299
T 0490
T 0583
T 0545
T 0995
T 1702
T 0921
T 0232
T 1178
T 1134
T 0632
T 1186
T 1900
T 0699
T 0633
T 1376
T 2030
T 2037
T 1843
T 0954
T 1413
T 1874
T 1096
T 1499
T 0725
T 0785
T 1844
T 0127
0570
1522
1528
0135
T 1768
1581
T 1509
T 2015
T 1001
0759
T 1046
T 1484
T 1374
T 1314
T 0319
0100
T 0379
T 0426
T 0378
T 0963
T 0279
0443
0769
0449
T 1912
1054
T 1855
1349
T 1012
T 1714
T 1788
T 0902
T 1895
1008
T 1887
T 1031
T 0402
T 1106
T 1104
0479
0325
T 1105
2020
1002
1108
1109
T 1625
T 1107
T 0706
0455
0477
0877
T 0876
1677
0424
1704
T 0845
T 1329
T 1005
T 0764
T 0407
T 1920

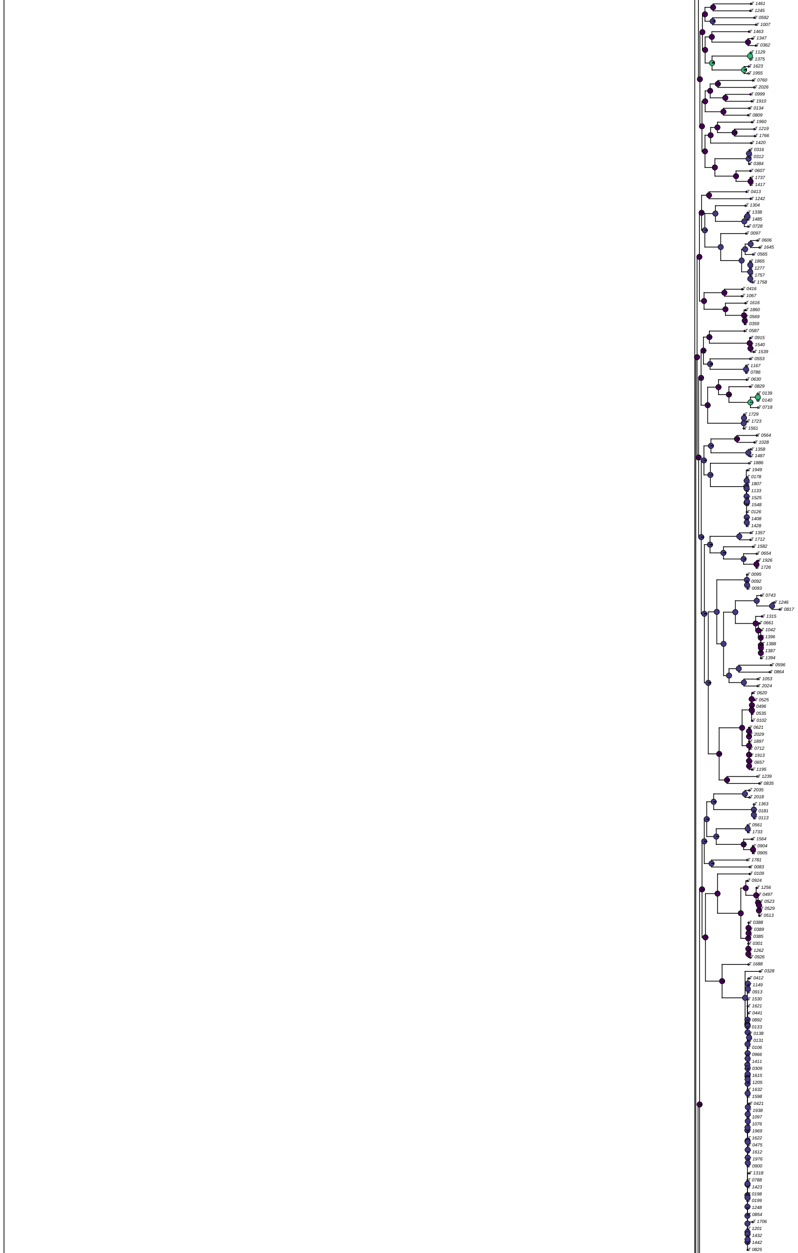
T 1461
T 1245
T 0592
T 1007
T 1463
T 1347
T 0362
T 1129
T 1375
T 1623
T 1955
T 0760
T 2026
T 0999
T 1910
T 0134
T 0809
T 1960
T 1219
T 1766
T 1420
T 0316
0312
T 0384
T 0607
T 1737
T 1417
T 0413
T 1242
T 1304
T 1338
T 1485
T 0728
T 0097
T 0606
T 1645
T 0565
T 1865
1277
T 1757
T 1758
T 0416
T 1067
T 1616
T 1860
0569
0359
T 0587
T 0915
1540
T 1539
T 0553
T 1167
0786
T 0630
T 0829
T 0139
0140
T 0718
T 1729
T 1723
T 1561
T 0564
T 1028
T 1358
T 1487
T 1886
T 1949
T 0178
1807
1133
1525
1548
T 0126
1408
1428
T 1357
T 1712
T 1582
T 0654
T 1926
1726
T 0095
0092
0093
T 0743
T 1246
T 0817
T 1315
T 0661
T 1042
1396
1388
1387
T 1394
T 0596
T 0864
T 1053
T 2024
T 0620
T 0525
0496
T 0535
T 0102
T 0621
2029
T 1897
T 0712
1913
0657
T 1195
T 1239
T 0835
T 2035
T 2018
T 1363
0181
0113
T 0561
1733
T 1564
T 0904
T 0905
T 1781
T 0083
T 0109
T 0924
T 1256
T 0497
T 0523
T 0529
T 0513
T 0388
0389
0385
T 0301
T 1262
T 0926
T 1688
T 0328
T 0412
1149
0913
T 1530
T 1621
T 0441
0892
0133
T 0138
0131
T 0106
0966
1411
0309
1615
1205
T 1632
1598
T 0421
1938
1097
1076
1969
1622
0475
1612
1976
0900
T 1318
T 0788
1423
0198
0199
1248
0854
T 1706
1201
1432
1442
T 0825

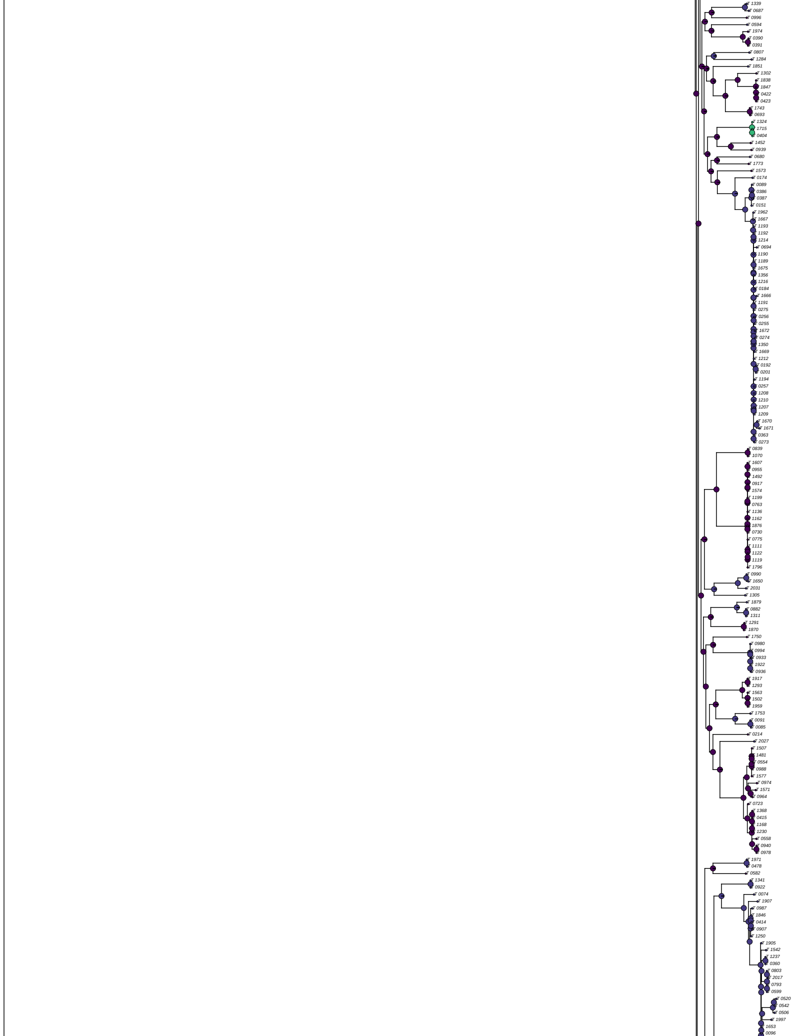
T 1339
T 0687
T 0996
T 0594
T 1974
T 0390
T 0391
T 0807
T 1284
T 1851
T 1302
T 1838
T 1847
T 0422
T 0423
T 1743
T 0693
T 1324
T 1715
T 0404
T 1452
T 0939
T 0680
T 1773
T 1573
T 0174
T 0089
T 0386
T 0387
T 0151
T 1962
T 1667
T 1193
T 1192
T 1214
T 0694
T 1190
T 1189
T 1675
T 1356
T 1216
T 0184
T 1666
T 1191
T 0275
T 0256
T 0255
T 1672
T 0274
T 1350
T 1669
T 1212
T 0192
T 0201
T 1194
T 0257
T 1208
T 1210
T 1207
T 1209
T 1670
T 1671
T 0363
T 0273
T 0839
T 1070
T 1607
T 0955
T 1492
T 0917
T 1574
T 1199
T 0763
T 1136
T 1162
T 1876
T 0730
T 0775
T 1111
T 1122
T 1119
T 1796
T 0990
T 1650
T 2031
T 1305
T 1879
T 0882
T 1311
T 1291
T 1870
T 1750
T 0980
T 0994
T 0933
T 1922
T 0936
T 1917
T 1293
T 1563
T 1502
T 1959
T 1753
T 0091
T 0085
T 0214
T 2027
T 1507
T 1481
T 0554
T 0988
T 1577
T 0974
T 1571
T 0964
T 0723
T 1368
T 0415
T 1168
T 1230
T 0558
T 0940
T 0978
T 1971
T 0478
T 0582
T 1341
T 0922
T 0074
T 1907
T 0987
T 1846
T 0414
T 0907
T 1250
T 1905
T 1542
T 1237
T 0360
T 0803
T 2017
T 0793
T 0599
T 0520
T 0542
T 0506
T 1997
T 1653
T 0096

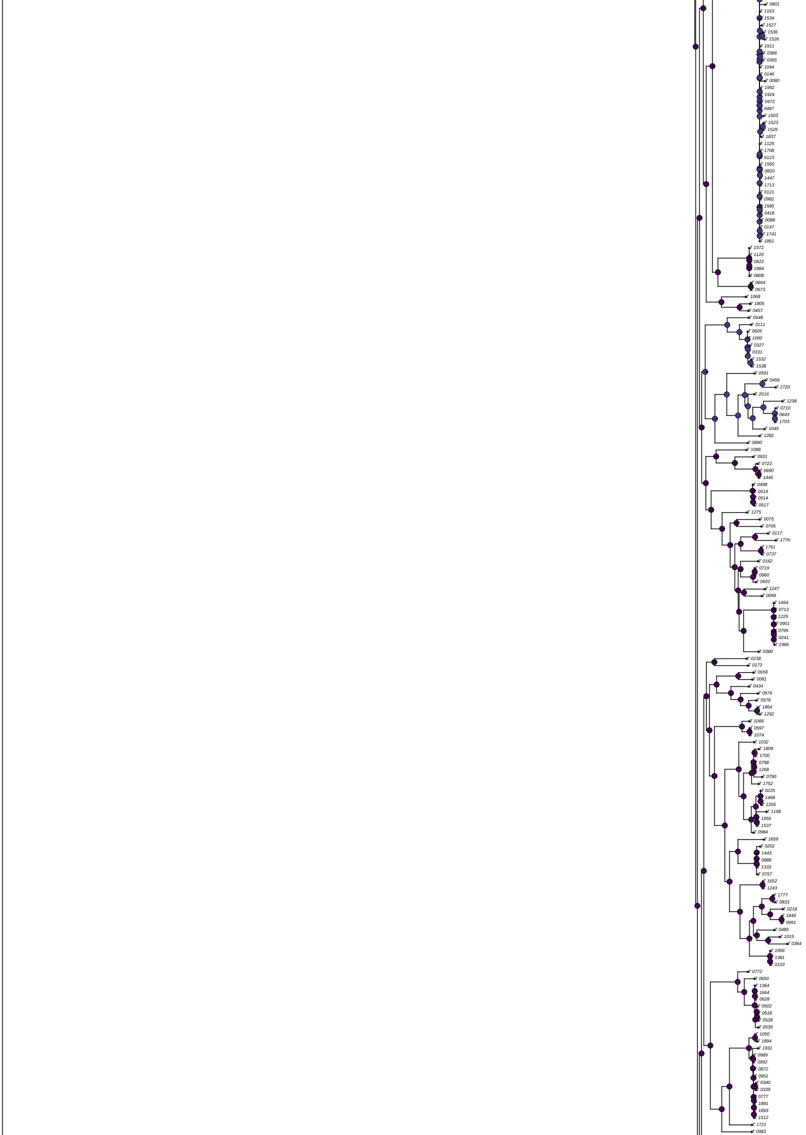

T 0801
T 1163
T 1534
T 1527
T 1536
T 1526
T 1911
T 0366
T 0365
T 1044
T 0146
T 0080
T 1992
T 1924
T 0472
T 0487
T 1503
T 1523
T 1529
T 1837
T 1125
T 1708
T 0123
T 1560
T 0820
T 1447
T 1713
T 0121
T 0981
T 1585
T 0418
T 0088
T 0147
T 1741
T 1861
T 1572
T 1120
T 0622
T 1984
T 0808
T 0664
T 0573
T 1068
T 1805
T 0457
T 0548
T 0111
T 0605
T 1000
T 0327
T 0331
T 1532
T 1538
T 0591
T 0456
T 1720
T 2016
T 1238
T 0710
T 0643
T 1703
T 1045
T 1282
T 0890
T 1088
T 0931
T 0722
T 0690
T 1445
T 0498
T 0519
T 0514
T 0517
T 1275
T 0075
T 0705
T 0117
T 1776
T 1761
T 0737
T 0162
T 0719
T 0960
T 0691
T 1247
T 0099
T 1464
T 0713
T 1225
T 0901
T 0799
T 0241
T 1966
T 0380
T 0238
T 0173
T 0656
T 0081
T 0434
T 0576
T 0578
T 1864
T 1292
T 1066
T 0597
T 1074
T 1032
T 1809
T 1700
T 0798
T 1268
T 0790
T 1752
T 0225
T 1468
T 1255
T 1198
T 1556
T 1537
T 0984
T 1659
T 0202
T 1443
T 0888
T 1333
T 0757
T 1652
T 1243
T 1777
T 0833
T 0218
T 1849
T 0991
T 0485
T 1015
T 0364
T 1956
T 1381
T 0103
T 0772
T 0650
T 1364
T 1664
T 0628
T 0502
T 0518
T 0528
T 0539
T 1050
T 1894
T 1931
T 0989
T 0992
T 0872
T 0951
T 0340
T 0339
T 0777
T 1891
T 1893
T 1512
T 1721
T 0983

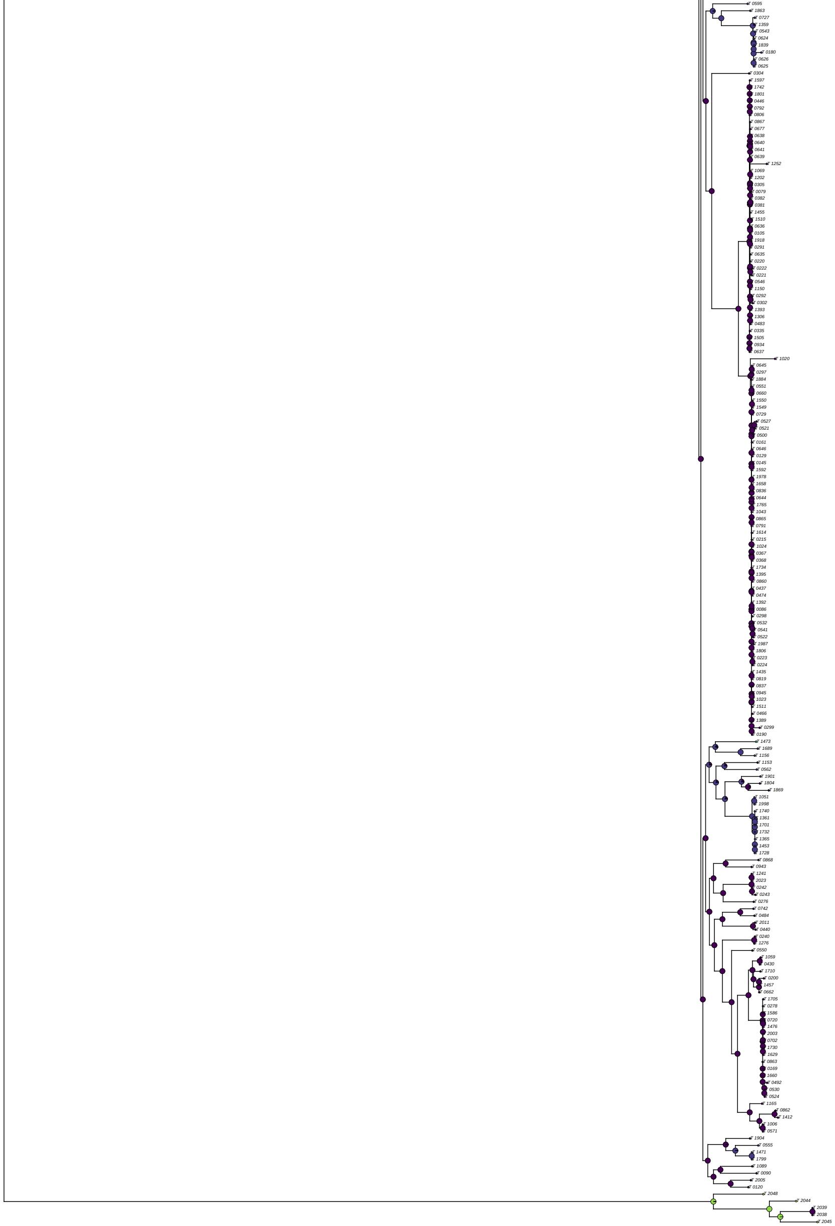

T 0595
T 1863
T 0727
T 1359
T 0543
T 0624
T 1839
T 0180
T 0626
T 0625
T 0304
T 1597
T 1742
T 1801
T 0446
T 0792
T 0806
T 0867
T 0677
T 0638
T 0640
T 0641
T 0639
T 1252
T 1069
T 1202
T 0305
T 0079
T 0382
T 0381
T 1455
T 1510
T 0636
T 0105
T 1918
T 0291
T 0635
T 0220
T 0222
T 0221
T 0546
T 1150
T 0292
T 0302
T 1393
T 1306
T 0483
T 0335
T 1505
T 0934
T 0637
T 1020
T 0645
T 0297
T 1884
T 0551
T 0660
T 1550
T 1549
T 0729
T 0527
T 0521
T 0500
T 0161
T 0646
T 0129
T 0145
T 1592
T 1978
T 1658
T 0836
T 0644
T 1765
T 1043
T 0865
T 0791
T 1614
T 0215
T 1024
T 0367
T 0368
T 1734
T 1395
T 0860
T 0437
T 0474
T 1392
T 0086
T 0298
T 0532
T 0541
T 0522
T 1987
T 1806
T 0223
T 0224
T 1435
T 0819
T 0837
T 0945
T 1023
T 1511
T 0466
T 1389
T 0299
T 0190
T 1473
T 1689
T 1156
T 1153
T 0562
T 1901
T 1804
T 1869
T 1051
T 1998
T 1740
T 1361
T 1701
T 1732
T 1365
T 1453
T 1728
T 0868
T 0943
T 1241
T 2023
T 0242
T 0243
T 0276
T 0742
T 0484
T 2011
T 0440
T 0240
T 1276
T 0550
T 1059
T 0430
T 1710
T 0200
T 1457
T 0662
T 1705
T 0278
T 1586
T 0720
T 1476
T 2003
T 0702
T 1730
T 1629
T 0863
T 0169
T 1660
T 0492
T 0530
T 0524
T 1165
T 0862
T 1412
T 1006
T 0571
T 1904
T 0555
T 1471
T 1799
T 1089
T 0090
T 2005
T 0120
T 2048
T 2044
T 2039
T 2038
T 2045

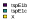

Locus: PA5265

Effector genes: tspE1b, tspE1c

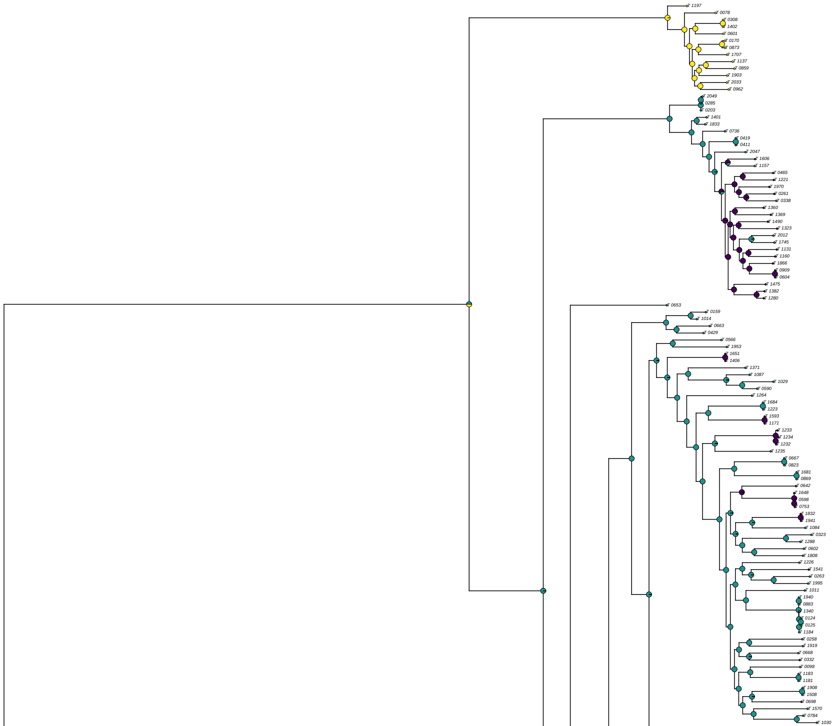

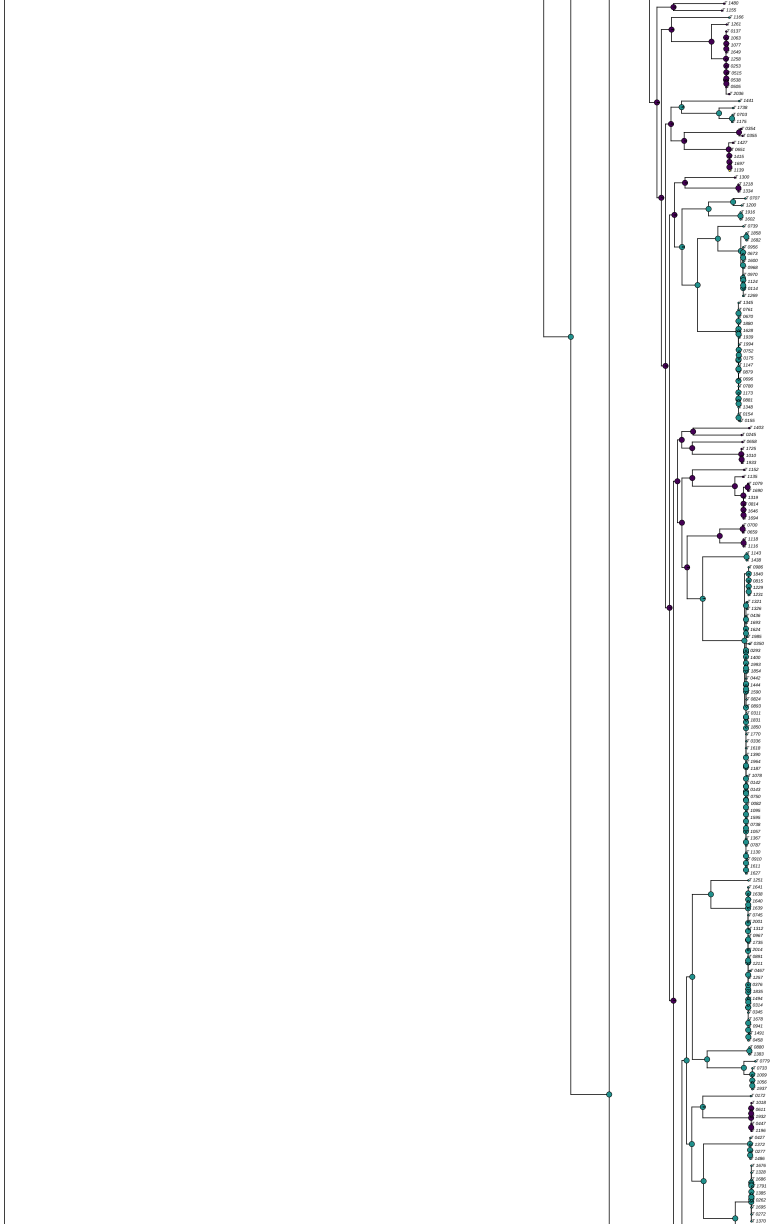
T 1480
T 1155
T 1166
T 1261
T 0137
1063
1077
1649
1258
0253
0515
0538
0505
T 2036
T 1441
T 1738
T 0703
1175
T 0354
T 0355
T 1427
T 0651
1415
1697
1139
T 1300
T 1218
T 1334
T 0707
T 1200
T 1916
T 1602
T 0739
T 1858
1682
T 0956
0673
1600
0968
T 0970
1124
0114
T 1269
T 1345
T 0761
0670
1880
1628
1939
T 1994
0752
0175
T 1147
0879
0696
T 0780
1173
0881
1348
T 0154
T 0155
T 1403
T 0245
T 0658
T 1725
1010
1933
T 1152
T 1135
T 1079
T 1690
1319
0814
1646
1694
T 0700
0659
T 1118
1116
T 1143
1438
T 0986
1840
0815
1229
1231
T 1321
1326
0436
1693
1624
T 1985
T 0350
0293
1400
1993
1854
T 0442
1444
1590
T 0824
0893
T 0311
1831
1850
T 1770
T 0336
T 1618
T 1390
T 1964
1187
T 1078
0142
0143
0750
0082
1095
1595
0738
1057
T 1367
0787
T 1130
T 0910
1611
1627
T 1251
T 1641
1638
1640
1639
T 0745
2001
T 1312
T 0967
1735
2014
T 0891
1211
T 0467
1257
0376
1835
1494
0314
T 0345
T 1678
0941
T 1491
0458
T 0880
1383
T 0779
T 0733
1009
1056
1937
T 0172
T 1018
0611
1932
T 0447
1196
T 0427
1372
0277
1486
T 1676
T 1328
T 1686
1791
1385
0262
T 1695
T 0272
T 1370

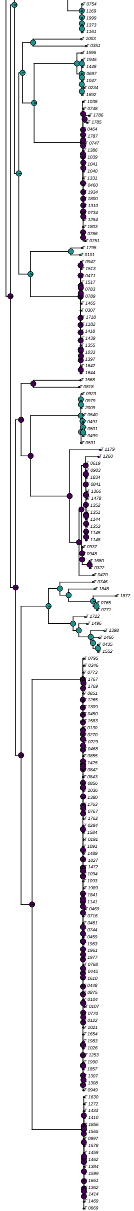
T 0754
T 1169
T 1999
T 1373
T 1161
T 1003
T 0351
T 1596
T 1945
T 1448
T 0697
T 1047
T 0234
T 1692
T 1038
T 0748
T 1786
T 1785
T 0464
T 1787
T 0747
T 1386
T 1039
T 1041
T 1040
T 1331
T 0460
T 1934
T 1800
T 1310
T 0734
T 1254
T 1803
T 0766
T 0751
T 1795
T 0101
T 0947
T 1513
T 0471
T 1517
T 0783
T 0789
T 1465
T 0307
T 1718
T 1182
T 1418
T 1439
T 1355
T 1033
T 1397
T 1642
T 1644
T 1568
T 0818
T 0923
T 0979
T 2009
T 0540
T 0491
T 0501
T 0499
T 0531
T 1179
T 1260
T 0619
T 0903
T 1834
T 0841
T 1366
T 1478
T 1352
T 1351
T 1144
T 1353
T 1145
T 1148
T 0937
T 0948
T 1680
T 0322
T 0470
T 0746
T 1848
T 1877
T 0765
T 0771
T 1722
T 1496
T 1398
T 1466
T 0435
T 1552
T 0795
T 0346
T 0773
T 1767
T 1769
T 0851
T 1265
T 1309
T 0450
T 1583
T 0130
T 0270
T 0229
T 0468
T 0855
T 1425
T 0842
T 0843
T 0856
T 1036
T 1380
T 1763
T 0767
T 1762
T 0284
T 1584
T 0191
T 1091
T 1489
T 1027
T 1472
T 1094
T 1093
T 1989
T 1841
T 1141
T 0469
T 0716
T 0461
T 0744
T 0459
T 1963
T 1961
T 1977
T 0768
T 0445
T 1610
T 0448
T 0875
T 0104
T 0107
T 0770
T 0122
T 1021
T 1654
T 1983
T 1026
T 1253
T 1990
T 1857
T 1307
T 1308
T 0949
T 1630
T 1272
T 1433
T 1410
T 1856
T 1565
T 0997
T 1578
T 1459
T 1462
T 1384
T 1599
T 1661
T 1362
T 1414
T 1469
T 0669

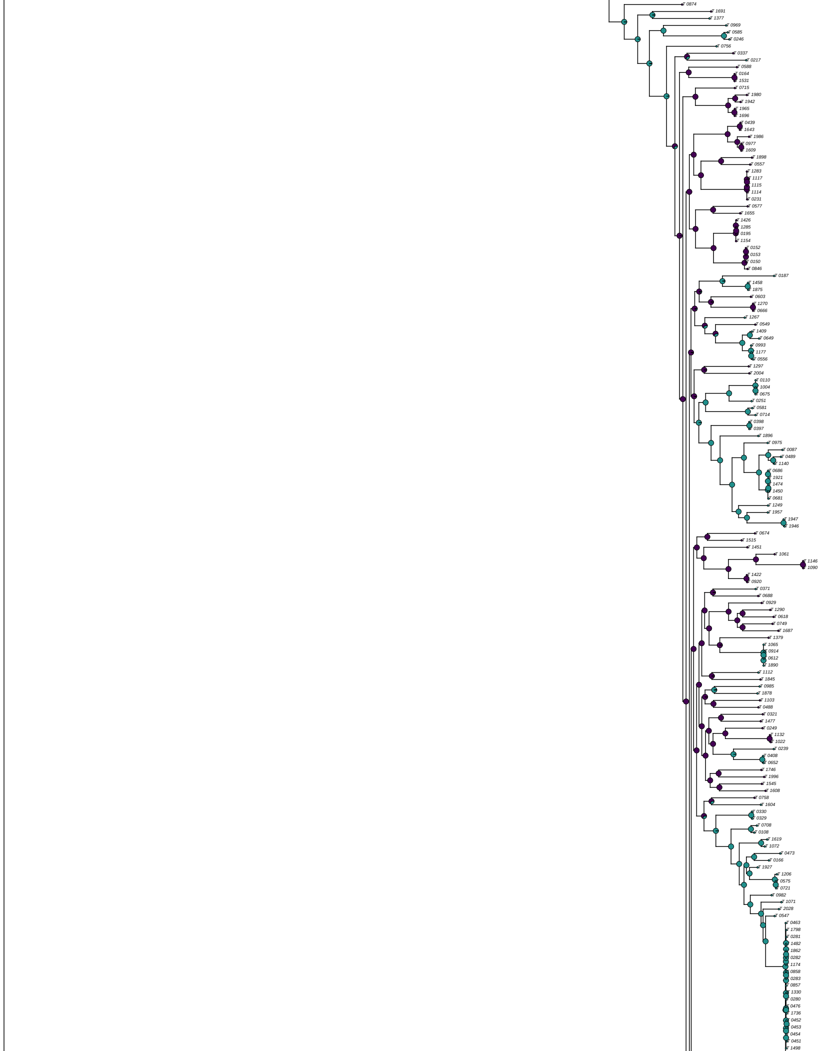
T 0874
T 1691
T 1377
T 0969
T 0585
T 0246
T 0756
T 0337
T 0217
T 0588
T 0164
T 1531
T 0715
T 1980
T 1942
T 1965
T 1696
T 0439
T 1643
T 1986
T 0977
T 1609
T 1898
T 0557
T 1283
T 1117
T 1115
T 1114
T 0231
T 0577
T 1655
T 1426
T 1285
T 0195
T 1154
T 0152
T 0153
T 0150
T 0846
T 0187
T 1458
T 1875
T 0603
T 1270
T 0666
T 1267
T 0549
T 1409
T 0649
T 0993
T 1177
T 0556
T 1297
T 2004
T 0110
T 1004
T 0675
T 0251
T 0581
T 0714
T 0398
T 0397
T 1896
T 0975
T 0087
T 0489
T 1140
T 0686
T 1921
T 1474
T 1450
T 0681
T 1249
T 1957
T 1947
T 1946
T 0674
T 1515
T 1451
T 1061
T 1146
T 1090
T 1422
T 0920
T 0371
T 0688
T 0929
T 1290
T 0618
T 0749
T 1687
T 1379
T 1065
T 0914
T 0612
T 1890
T 1112
T 1845
T 0985
T 1878
T 1103
T 0488
T 0321
T 1477
T 0249
T 1132
T 1022
T 0239
T 0408
T 0652
T 1746
T 1996
T 1545
T 1608
T 0758
T 1604
T 0330
T 0329
T 0708
T 0108
T 1619
T 1072
T 0473
T 0166
T 1927
T 1206
T 0575
T 0721
T 0982
T 1071
T 2028
T 0547
T 0463
T 1798
T 0281
T 1482
T 1862
T 0282
T 1174
T 0858
T 0283
T 0857
T 1330
T 0280
T 0476
T 1736
T 0452
T 0453
T 0454
T 0451
T 1498

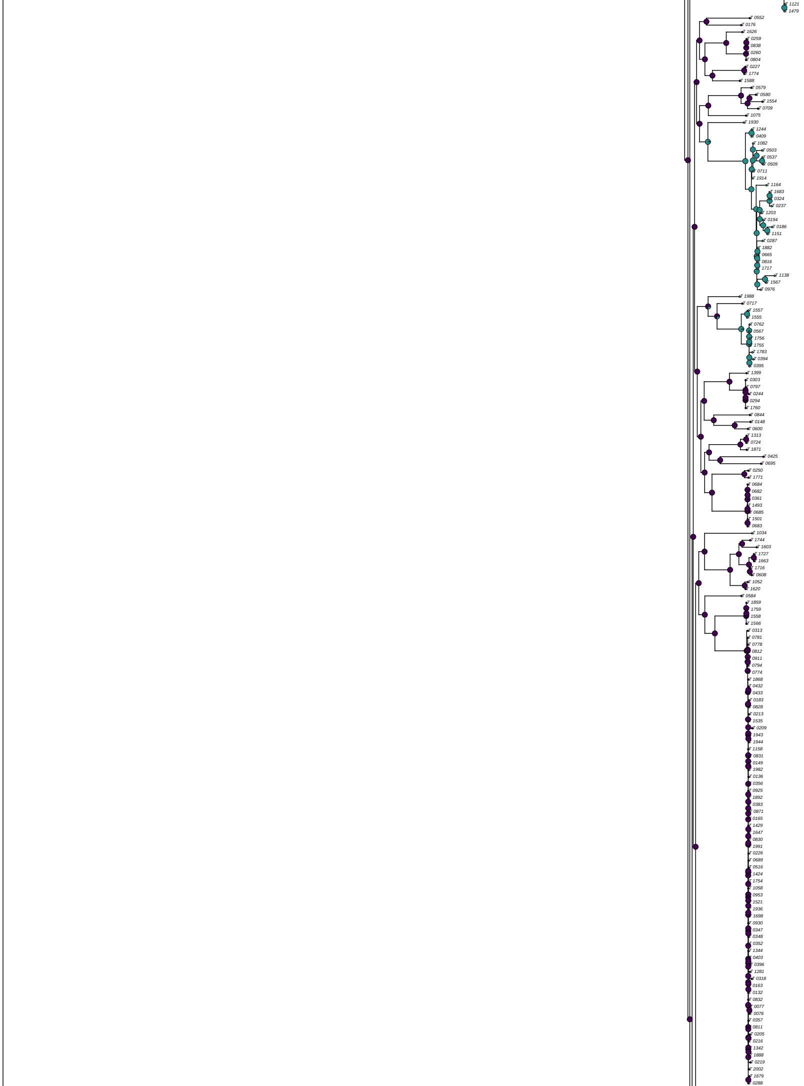
T 1121
T 1479
T 0552
T 0176
T 1626
T 0259
T 0838
T 0260
T 0804
T 0227
T 1774
T 1588
T 0579
T 0580
T 1554
T 0709
T 1075
T 1930
T 1244
T 0409
T 1082
T 0503
T 0537
T 0509
T 0711
T 1914
T 1164
T 1683
T 0324
T 0237
T 1203
T 0194
T 0186
T 1151
T 0287
T 1882
T 0665
T 0816
T 1717
T 1138
T 1567
T 0976
T 1988
T 0717
T 1557
T 1555
T 0762
T 0567
T 1756
T 1755
T 1783
T 0394
T 0395
T 1399
T 0303
T 0797
T 0244
T 0294
T 1760
T 0844
T 0148
T 0600
T 1313
T 0724
T 1871
T 0425
T 0695
T 0250
T 1771
T 0684
T 0682
T 0361
T 1493
T 0685
T 1501
T 0683
T 1034
T 1744
T 1603
T 1727
T 1663
T 1716
T 0608
T 1052
T 1620
T 0584
T 1859
T 1759
T 1558
T 1566
T 0313
T 0781
T 0778
T 0812
T 0911
T 0794
T 0774
T 1868
T 0432
T 0433
T 0183
T 0828
T 0213
T 1535
T 0209
T 1943
T 1944
T 1158
T 0831
T 0149
T 1982
T 0136
T 0356
T 0925
T 1892
T 0383
T 0871
T 0165
T 1429
T 1647
T 0830
T 1991
T 0226
T 0689
T 0516
T 1424
T 1754
T 1058
T 0953
T 1521
T 1936
T 1698
T 0930
T 0347
T 0348
T 0352
T 1344
T 0403
T 0396
T 1281
T 0318
T 0163
T 0132
T 0832
T 0077
T 0076
T 0357
T 0811
T 0205
T 0216
T 1342
T 1888
T 0219
T 2002
T 1679
T 0288

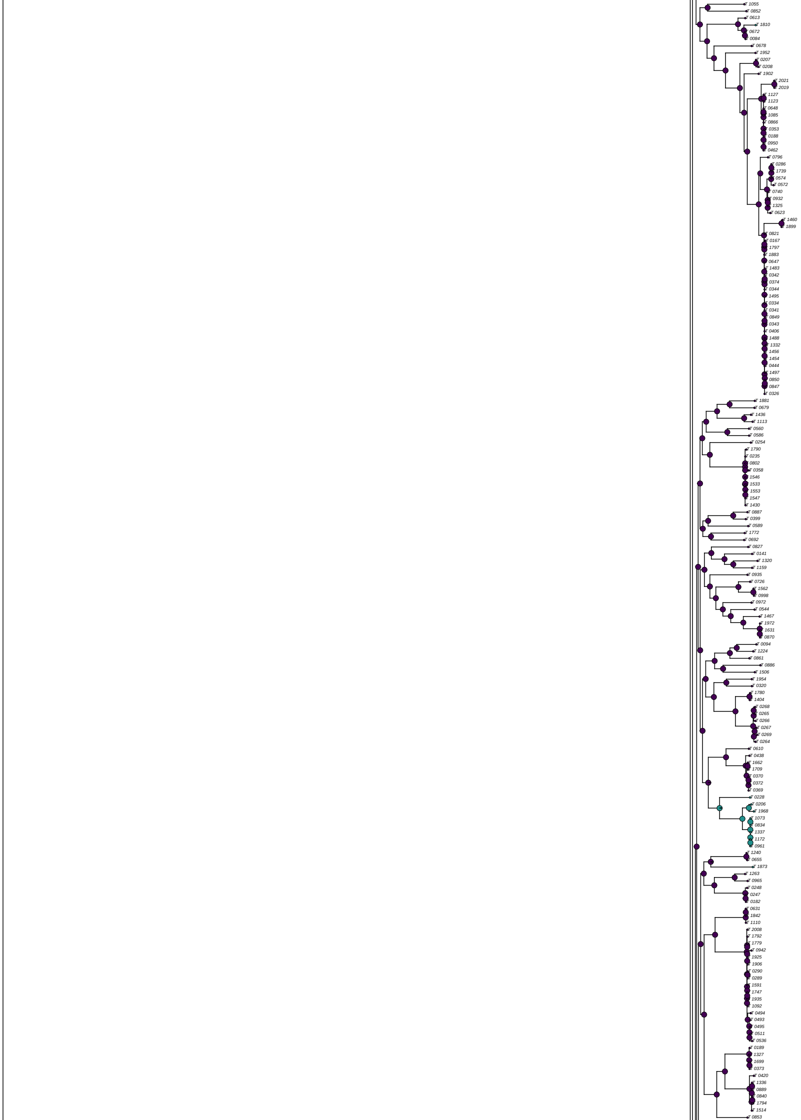
T 1055
T 0852
T 0613
T 1810
T 0672
T 0084
T 0678
T 1952
T 0207
T 0208
T 1902
T 2021
T 2019
T 1127
T 1123
T 0648
T 1085
T 0866
T 0353
T 0188
T 0950
T 0462
T 0796
T 0286
T 1739
T 0574
T 0572
T 0740
T 0932
T 1325
T 0623
T 1460
T 1899
T 0821
T 0167
T 1797
T 1883
T 0647
T 1483
T 0342
T 0374
T 0344
T 1495
T 0334
T 0341
T 0849
T 0343
T 0406
T 1488
T 1332
T 1456
T 1454
T 0444
T 1497
T 0850
T 0847
T 0326
T 1881
T 0679
T 1436
T 1113
T 0560
T 0586
T 0254
T 1790
T 0235
T 0802
T 0358
T 1546
T 1533
T 1553
T 1547
T 1430
T 0887
T 0399
T 0589
T 1772
T 0692
T 0827
T 0141
T 1320
T 1159
T 0935
T 0726
T 1562
T 0998
T 0972
T 0544
T 1467
T 1972
T 1631
T 0870
T 0094
T 1224
T 0861
T 0886
T 1506
T 1954
T 0320
T 1780
T 1404
T 0268
T 0265
T 0266
T 0267
T 0269
T 0264
T 0610
T 0438
T 1662
T 1709
T 0370
T 0372
T 0369
T 0228
T 0206
T 1968
T 1073
T 0834
T 1337
T 1172
T 0961
T 1240
T 0655
T 1873
T 1263
T 0965
T 0248
T 0247
T 0182
T 0631
T 1842
T 1110
T 2008
T 1792
T 1779
T 0942
T 1925
T 1906
T 0290
T 0289
T 1591
T 1747
T 1935
T 1092
T 0494
T 0493
T 0495
T 0511
T 0536
T 0189
T 1327
T 1699
T 0373
T 0420
T 1336
T 0889
T 0840
T 1794
T 1514
T 0853

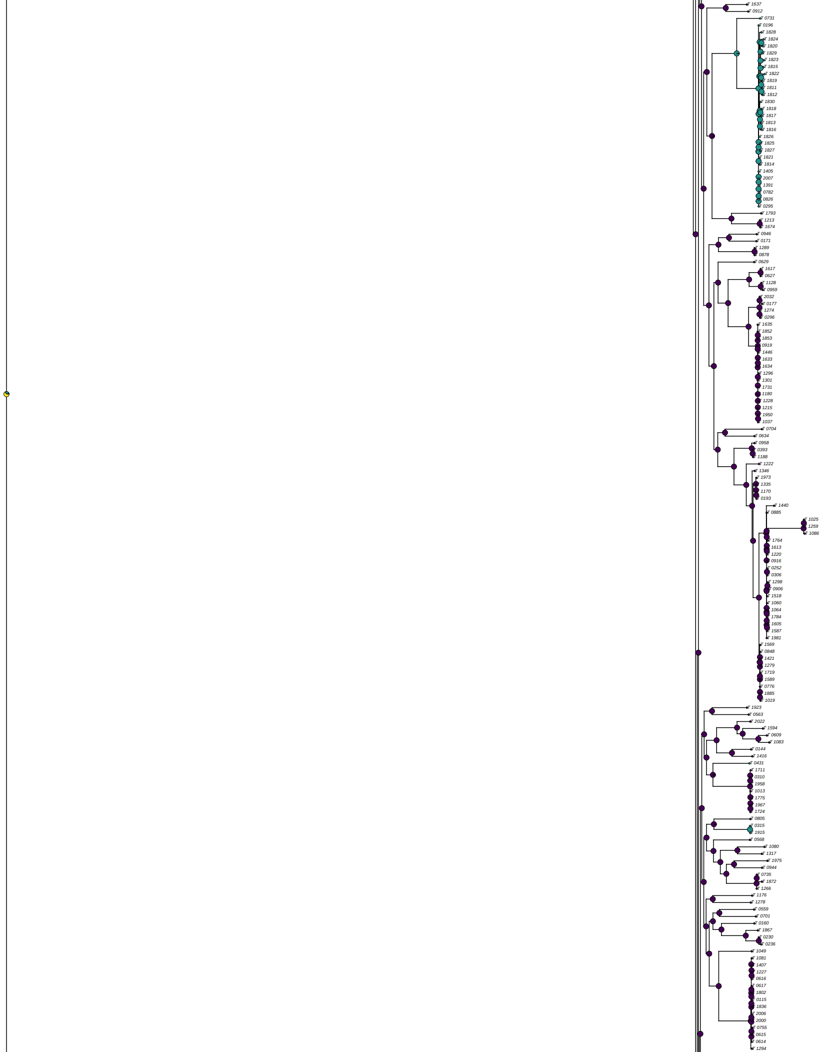
T 1637
T 0912
T 0731
T 0196
T 1828
T 1824
T 1820
T 1829
T 1823
T 1815
T 1822
T 1819
T 1811
T 1812
T 1830
T 1818
T 1817
T 1813
T 1816
T 1826
T 1825
T 1827
T 1821
T 1814
T 1405
T 2007
T 1391
T 0782
T 0826
T 0295
T 1793
T 1213
T 1674
T 0946
T 0171
T 1289
T 0878
T 0629
T 1617
T 0627
T 1128
T 0959
T 2032
T 0177
T 1274
T 0296
T 1635
T 1852
T 1853
T 0919
T 1446
T 1633
T 1634
T 1296
T 1301
T 1731
T 1180
T 1228
T 1215
T 1950
T 1037
T 0704
T 0634
T 0958
T 0393
T 1188
T 1222
T 1346
T 1973
T 1335
T 1170
T 0193
T 1440
T 0885
T 1025
T 1259
T 1086
T 1764
T 1613
T 1220
T 0916
T 0252
T 0306
T 1298
T 0906
T 1518
T 1060
T 1064
T 1784
T 1605
T 1587
T 1981
T 1569
T 0848
T 1421
T 1279
T 1719
T 1589
T 0776
T 1885
T 1019
T 1923
T 0563
T 2022
T 1594
T 0609
T 1083
T 0144
T 1416
T 0431
T 1711
T 0310
T 1958
T 1013
T 1775
T 1967
T 1724
T 0805
T 0315
T 1915
T 0568
T 1080
T 1317
T 1975
T 0944
T 0735
T 1872
T 1266
T 1176
T 1278
T 0559
T 0701
T 0160
T 1867
T 0230
T 0236
T 1049
T 1081
T 1407
T 1227
T 0616
T 0617
T 1802
T 0115
T 1836
T 2006
T 2000
T 0755
T 0615
T 0614
T 1294

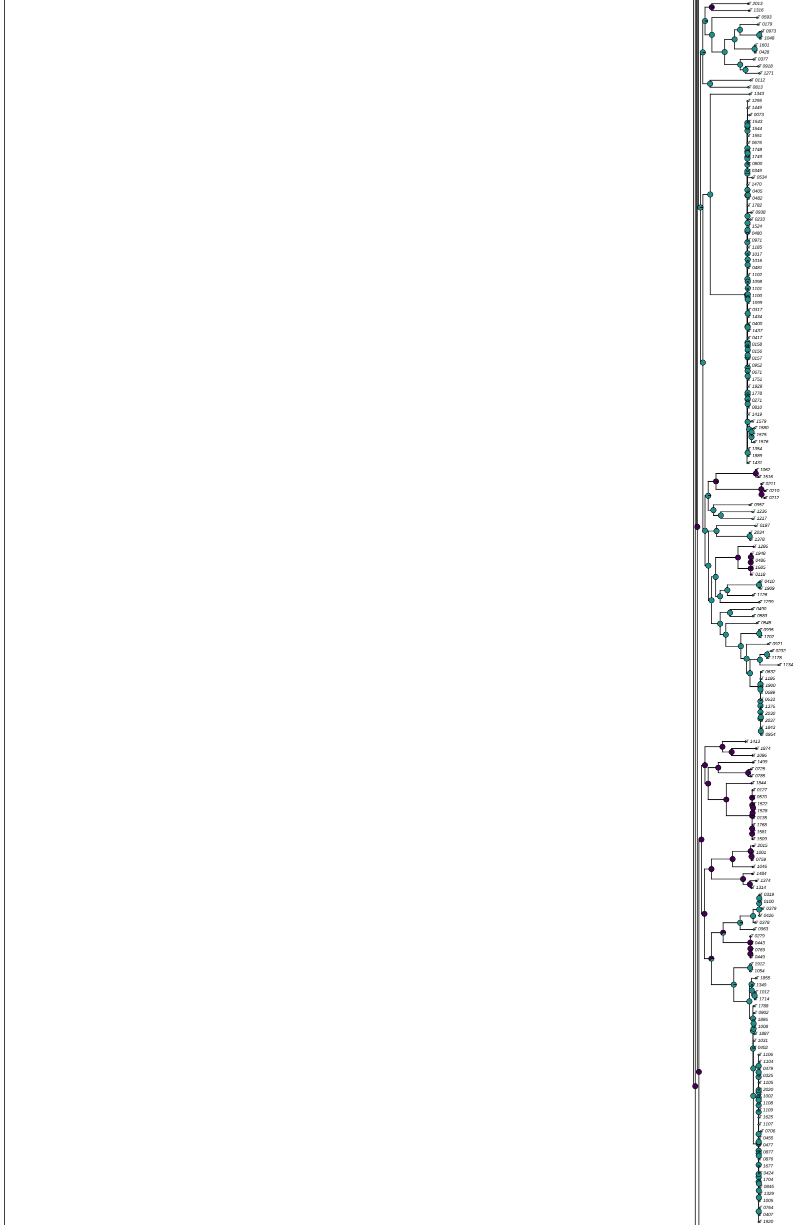
T 2013
T 1316
T 0593
T 0179
T 0973
T 1048
T 1601
T 0428
T 0377
T 0918
T 1271
T 0112
T 0813
T 1343
T 1295
T 1449
T 0073
T 1543
T 1544
T 1551
T 0676
T 1748
T 1749
T 0800
T 0349
T 0534
T 1470
T 0405
T 0482
T 1782
T 0938
T 0233
T 1524
T 0480
T 0971
T 1185
T 1017
T 1016
T 0481
T 1102
T 1098
T 1101
T 1100
T 1099
T 0317
T 1434
T 0400
T 1437
T 0417
T 0158
T 0156
T 0157
T 0952
T 0671
T 1751
T 1929
T 1778
T 0271
T 0810
T 1419
T 1579
T 1580
T 1575
T 1576
T 1354
T 1889
T 1431
T 1062
T 1516
T 0211
T 0210
T 0212
T 0957
T 1236
T 1217
T 0197
T 2034
T 1378
T 1286
T 1948
T 0486
T 1685
T 0118
T 0410
T 1909
T 1126
T 1299
T 0490
T 0583
T 0545
T 0995
T 1702
T 0921
T 0232
T 1178
T 1134
T 0632
T 1186
T 1900
T 0699
T 0633
T 1376
T 2030
T 2037
T 1843
T 0954
T 1413
T 1874
T 1096
T 1499
T 0725
T 0785
T 1844
T 0127
T 0570
T 1522
T 1528
T 0135
T 1768
T 1581
T 1509
T 2015
T 1001
T 0759
T 1046
T 1484
T 1374
T 1314
T 0319
T 0100
T 0379
T 0426
T 0378
T 0963
T 0279
T 0443
T 0769
T 0449
T 1912
T 1054
T 1855
T 1349
T 1012
T 1714
T 1788
T 0902
T 1895
T 1008
T 1887
T 1031
T 0402
T 1106
T 1104
T 0479
T 0325
T 1105
T 2020
T 1002
T 1108
T 1109
T 1625
T 1107
T 0706
T 0455
T 0477
T 0877
T 0876
T 1677
T 0424
T 1704
T 0845
T 1329
T 1005
T 0764
T 0407
T 1920

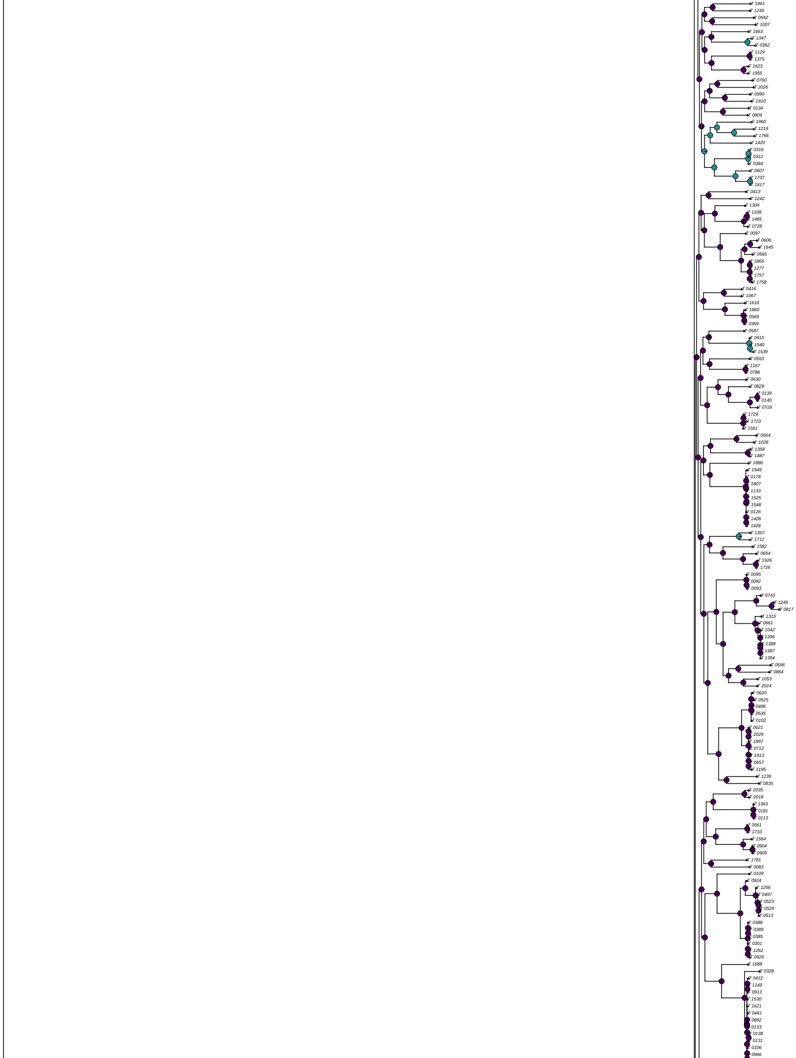
T 1461
T 1245
T 0592
T 1007
T 1463
T 1347
T 0362
T 1129
T 1375
T 1623
T 1955
T 0760
T 2026
T 0999
T 1910
T 0134
T 0809
T 1960
T 1219
T 1766
T 1420
T 0316
T 0312
T 0384
T 0607
T 1737
T 1417
T 0413
T 1242
T 1304
T 1338
T 1485
T 0728
T 0097
T 0606
T 1645
T 0565
T 1865
1277
T 1757
T 1758
T 0416
T 1067
T 1616
T 1860
0569
T 0359
T 0587
T 0915
1540
T 1539
T 0553
T 1167
T 0786
T 0630
T 0829
T 0139
T 0140
T 0718
T 1729
T 1723
T 1561
T 0564
T 1028
T 1358
T 1487
T 1886
T 1949
T 0178
1807
1133
1525
1548
T 0126
1408
1428
T 1357
T 1712
T 1582
T 0654
T 1926
T 1726
T 0095
0092
T 0093
T 0743
T 1246
T 0817
T 1315
T 0661
T 1042
1396
T 1388
1387
T 1394
T 0596
T 0864
T 1053
T 2024
T 0620
T 0525
0496
T 0535
T 0102
T 0621
2029
T 1897
T 0712
1913
0657
T 1195
T 1239
T 0835
T 2035
T 2018
T 1363
0181
0113
T 0561
T 1733
T 1564
T 0904
T 0905
T 1781
T 0083
T 0109
T 0924
T 1256
T 0497
T 0523
T 0529
T 0513
T 0388
T 0389
T 0385
T 0301
T 1262
T 0926
T 1688
T 0328
T 0412
1149
T 0913
T 1530
T 1621
T 0441
0892
0133
T 0138
T 0131
T 0106
0966

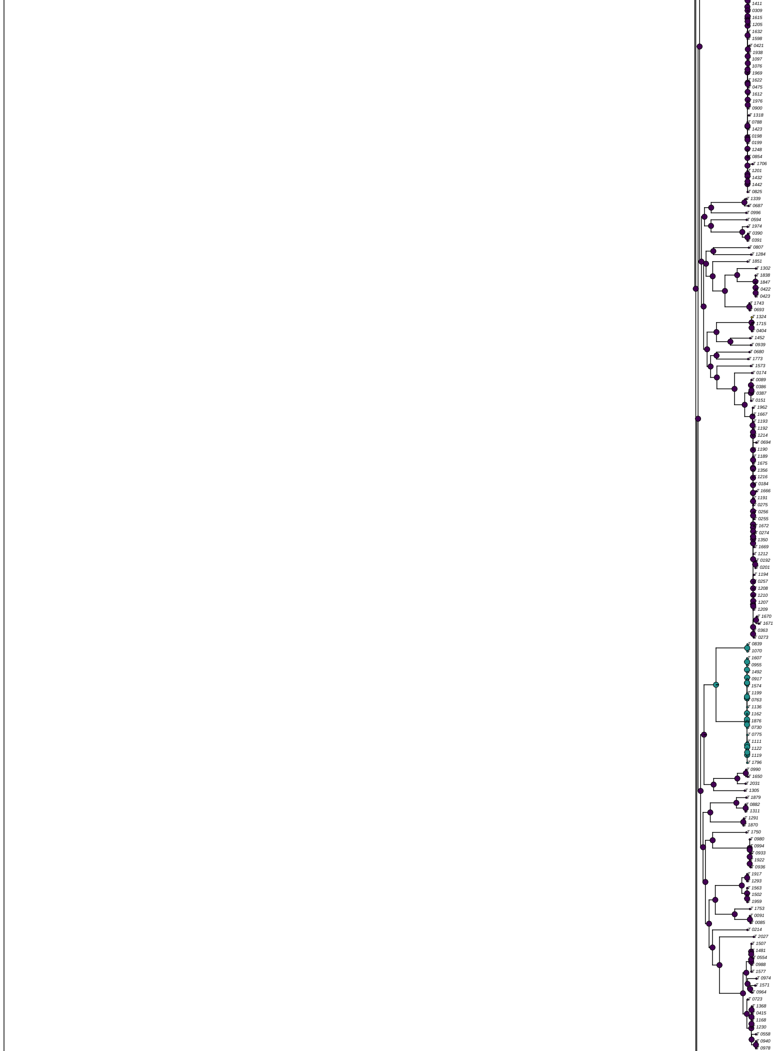
1411
0309
1615
1205
1632
1598
0421
1938
1097
1076
1969
1622
0475
1612
1976
0900
1318
0788
1423
0198
0199
1248
0854
1706
1201
1432
1442
0825
1339
0687
0996
0594
1974
0390
0391
0807
1284
1851
1302
1838
1847
0422
0423
1743
0693
1324
1715
0404
1452
0939
0680
1773
1573
0174
0089
0386
0387
0151
1962
1667
1193
1192
1214
0694
1190
1189
1675
1356
1216
0184
1666
1191
0275
0256
0255
1672
0274
1350
1669
1212
0192
0201
1194
0257
1208
1210
1207
1209
1670
1671
0363
0273
0839
1070
1607
0955
1492
0917
1574
1199
0763
1136
1162
1876
0730
0775
1111
1122
1119
1796
0990
1650
2031
1305
1879
0882
1311
1291
1870
1750
0980
0994
0933
1922
0936
1917
1293
1563
1502
1959
1753
0091
0085
0214
2027
1507
1481
0554
0988
1577
0974
1571
0964
0723
1368
0415
1168
1230
0558
0940
0978

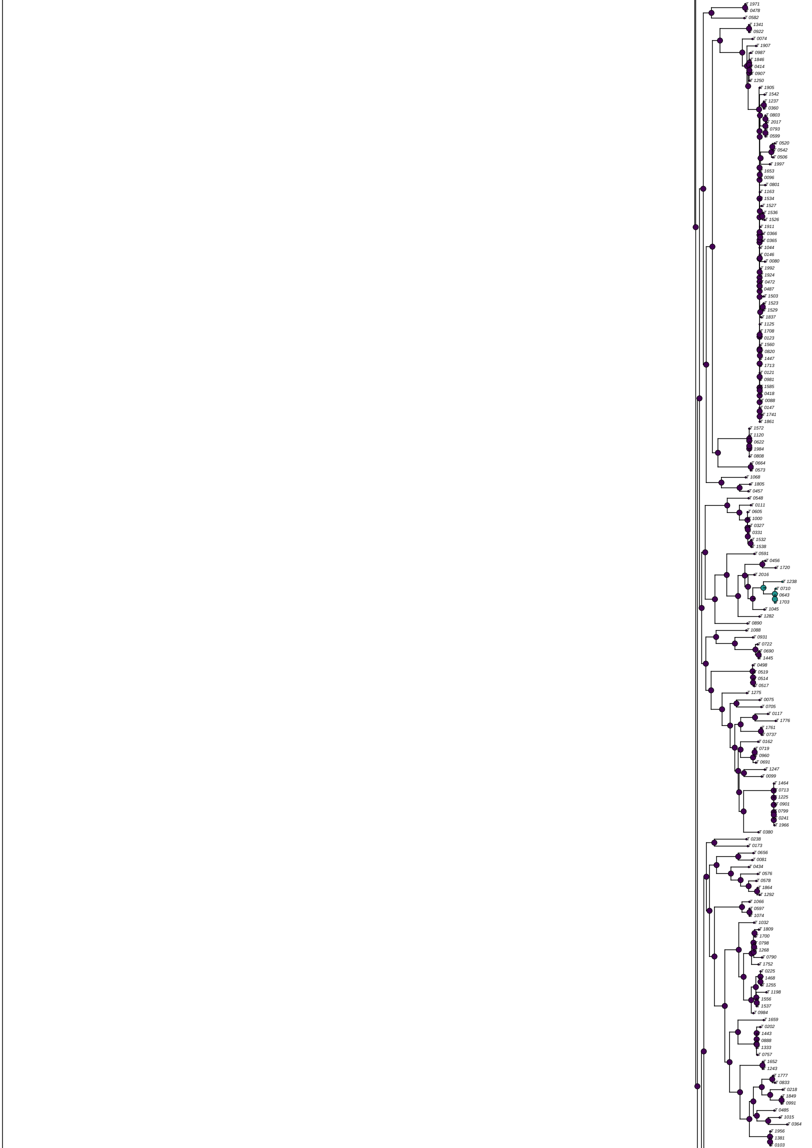

T 1971
T 0478
T 0582
T 1341
T 0922
T 0074
T 1907
T 0987
T 1846
T 0414
T 0907
T 1250
T 1905
T 1542
T 1237
T 0360
T 0803
T 2017
T 0793
T 0599
T 0520
T 0542
T 0506
T 1997
1653
0096
T 0801
T 1163
1534
T 1527
T 1536
T 1526
T 1911
T 0366
T 0365
T 1044
T 0146
T 0080
T 1992
1924
0472
0487
T 1503
T 1523
T 1529
T 1837
T 1125
T 1708
0123
T 1560
0820
1447
1713
T 0121
0981
1585
0418
0088
T 0147
T 1741
T 1861
T 1572
T 1120
0622
1984
T 0808
T 0664
T 0573
T 1068
T 1805
T 0457
T 0548
T 0111
T 0605
1000
T 0327
0331
T 1532
T 1538
T 0591
T 0456
T 1720
T 2016
T 1238
T 0710
0643
T 1703
T 1045
T 1282
T 0890
T 1088
T 0931
T 0722
T 0690
T 1445
T 0498
0519
0514
T 0517
T 1275
T 0075
T 0705
T 0117
T 1776
T 1761
T 0737
T 0162
T 0719
0960
T 0691
T 1247
T 0099
T 1464
T 0713
1225
T 0901
0799
0241
T 1966
T 0380
T 0238
T 0173
T 0656
T 0081
T 0434
T 0576
T 0578
T 1864
T 1292
T 1066
T 0597
T 1074
T 1032
T 1809
T 1700
0798
1268
T 0790
T 1752
T 0225
1468
T 1255
T 1198
1556
1537
T 0984
T 1659
T 0202
1443
0888
1333
T 0757
T 1652
T 1243
T 1777
T 0833
T 0218
T 1849
T 0991
T 0485
T 1015
T 0364
T 1956
1381
T 0103

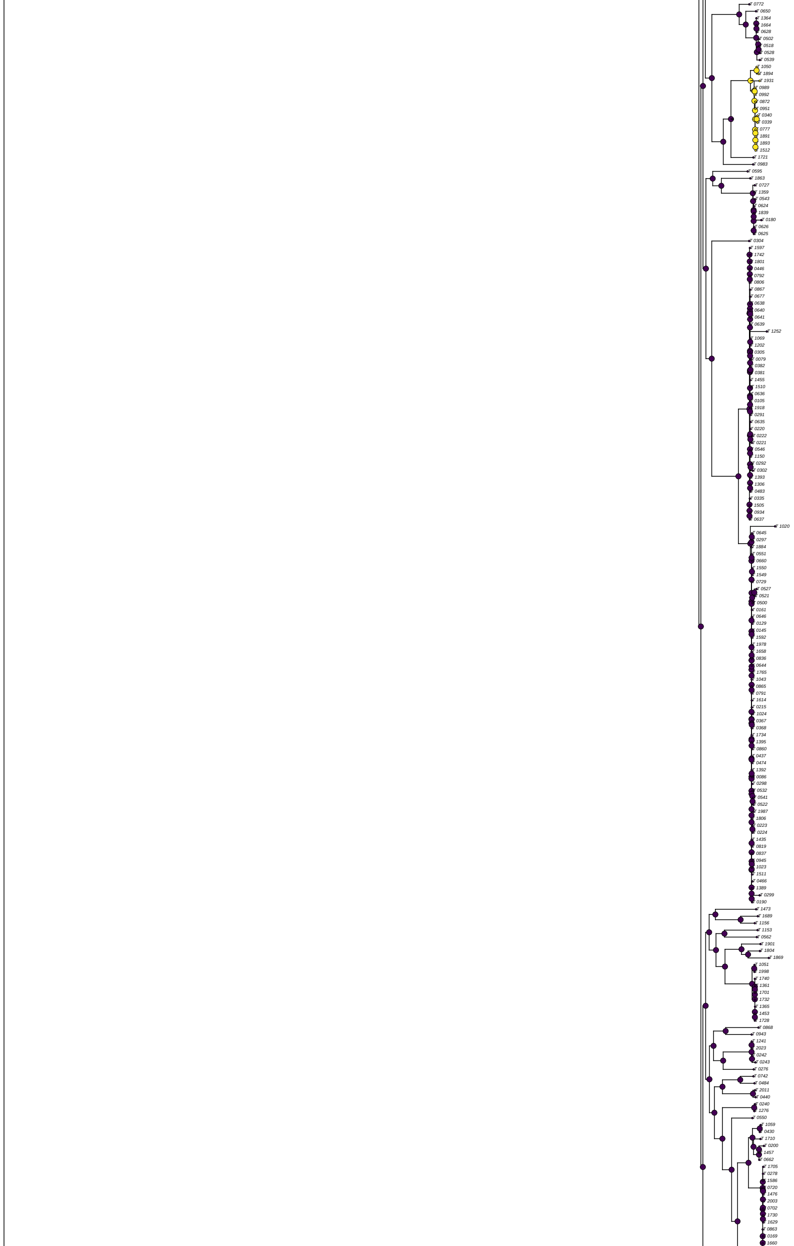
T 0772
T 0650
T 1364
T 1664
T 0628
T 0502
T 0518
T 0528
T 0539
T 1050
T 1894
T 1931
T 0989
T 0992
T 0872
T 0951
T 0340
T 0339
T 0777
T 1891
T 1893
T 1512
T 1721
T 0983
T 0595
T 1863
T 0727
T 1359
T 0543
T 0624
T 1839
T 0180
T 0626
T 0625
T 0304
T 1597
T 1742
T 1801
T 0446
T 0792
T 0806
T 0867
T 0677
T 0638
T 0640
T 0641
T 0639
T 1252
T 1069
T 1202
T 0305
T 0079
T 0382
T 0381
T 1455
T 1510
T 0636
T 0105
T 1918
T 0291
T 0635
T 0220
T 0222
T 0221
T 0546
T 1150
T 0292
T 0302
T 1393
T 1306
T 0483
T 0335
T 1505
T 0934
T 0637
T 1020
T 0645
T 0297
T 1884
T 0551
T 0660
T 1550
T 1549
T 0729
T 0527
T 0521
T 0500
T 0161
T 0646
T 0129
T 0145
T 1592
T 1978
T 1658
T 0836
T 0644
T 1765
T 1043
T 0865
T 0791
T 1614
T 0215
T 1024
T 0367
T 0368
T 1734
T 1395
T 0860
T 0437
T 0474
T 1392
T 0086
T 0298
T 0532
T 0541
T 0522
T 1987
T 1806
T 0223
T 0224
T 1435
T 0819
T 0837
T 0945
T 1023
T 1511
T 0466
T 1389
T 0299
T 0190
T 1473
T 1689
T 1156
T 1153
T 0562
T 1901
T 1804
T 1869
T 1051
T 1998
T 1740
T 1361
T 1701
T 1732
T 1365
T 1453
T 1728
T 0868
T 0943
T 1241
T 2023
T 0242
T 0243
T 0276
T 0742
T 0484
T 2011
T 0440
T 0240
T 1276
T 0550
T 1059
T 0430
T 1710
T 0200
T 1457
T 0662
T 1705
T 0278
T 1586
T 0720
T 1476
T 2003
T 0702
T 1730
T 1629
T 0863
T 0169
T 1660

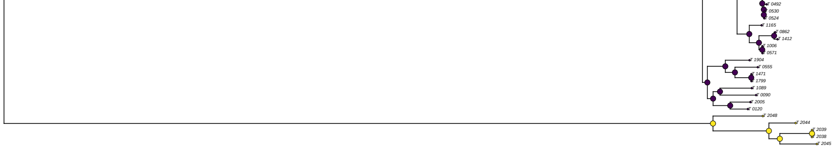
T 0492
0530
T 0524
T 1165
T 0862
T 1412
T 1006
T 0571
T 1904
T 0555
T 1471
T 1799
T 1089
T 0090
T 2005
T 0120
T 2048
T 2044
T 2039
T 2038
T 2045

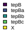

Locus: PA14-33970
Effector genes: tepB, tepBa, tepBb

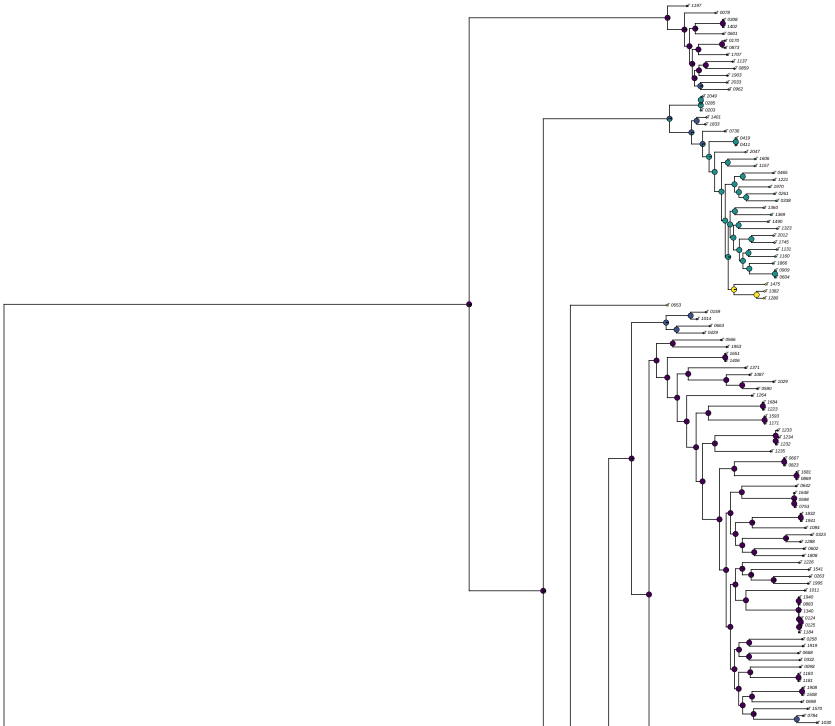

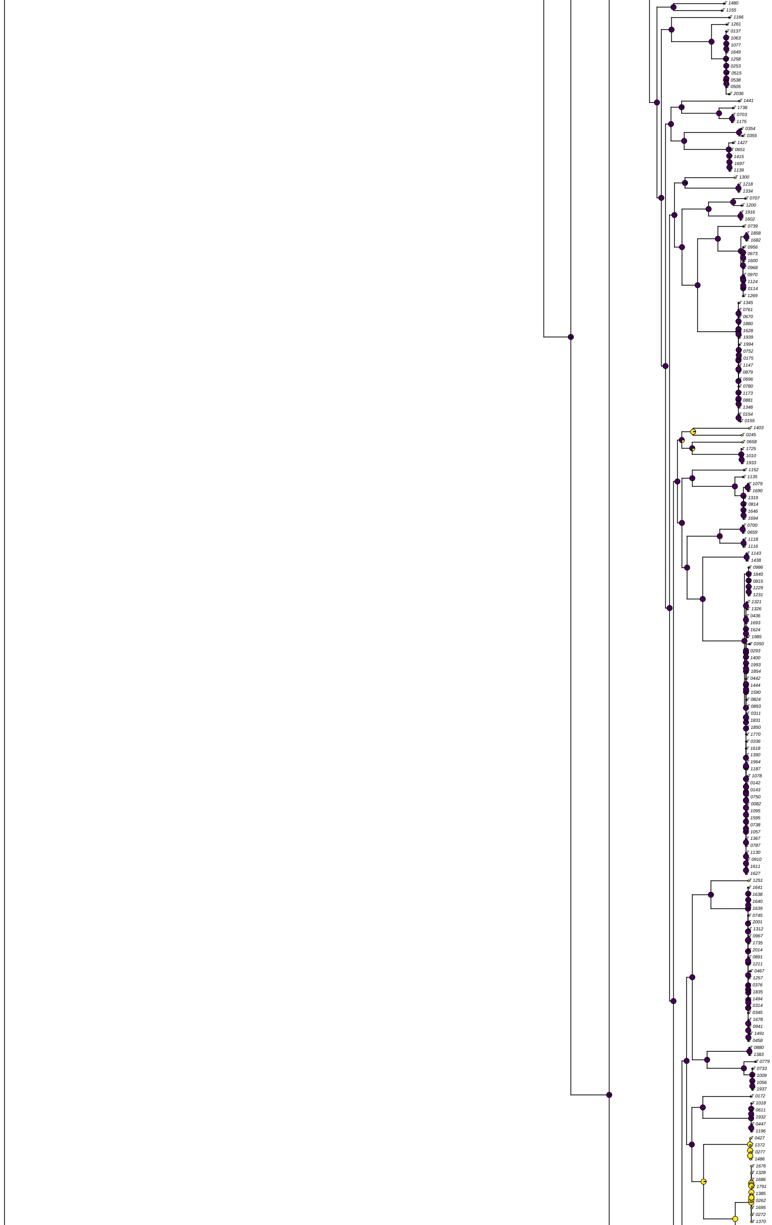

T 1480
T 1155
T 1166
T 1261
T 0137
1063
1077
T 1649
1258
0253
T 0515
0538
T 0505
T 2036
T 1441
T 1738
T 0703
T 1175
T 0354
T 0355
T 1427
T 0651
1415
1697
T 1139
T 1300
T 1218
T 1334
T 0707
T 1200
T 1916
T 1602
T 0739
T 1858
T 1682
T 0956
0673
T 1600
0968
T 0970
T 1124
T 0114
T 1269
T 1345
T 0761
0670
1880
1628
1939
T 1994
0752
0175
T 1147
0879
T 0696
T 0780
1173
0881
T 1348
T 0154
T 0155
T 1403
T 0245
T 0658
T 1725
1010
T 1933
T 1152
T 1135
T 1079
T 1690
1319
0814
1646
T 1694
T 0700
T 0659
T 1118
T 1116
T 1143
T 1438
T 0986
1840
0815
1229
T 1231
T 1321
T 1326
T 0436
1693
1624
T 1985
T 0350
0293
1400
1993
1854
T 0442
1444
1590
T 0824
0893
T 0311
1831
1850
T 1770
T 0336
T 1618
T 1390
T 1964
1187
T 1078
T 0142
0143
0750
0082
1095
1595
0738
1057
T 1367
0787
T 1130
T 0910
1611
T 1627
T 1251
T 1641
1638
1640
1639
T 0745
2001
T 1312
T 0967
T 1735
2014
T 0891
1211
T 0467
T 1257
0376
1835
1494
0314
T 0345
T 1678
T 0941
T 1491
T 0458
T 0880
T 1383
T 0779
T 0733
1009
1056
T 1937
T 0172
T 1018
0611
1932
T 0447
T 1196
T 0427
1372
0277
T 1486
T 1676
T 1328
T 1686
1791
1385
0262
T 1695
T 0272
T 1370

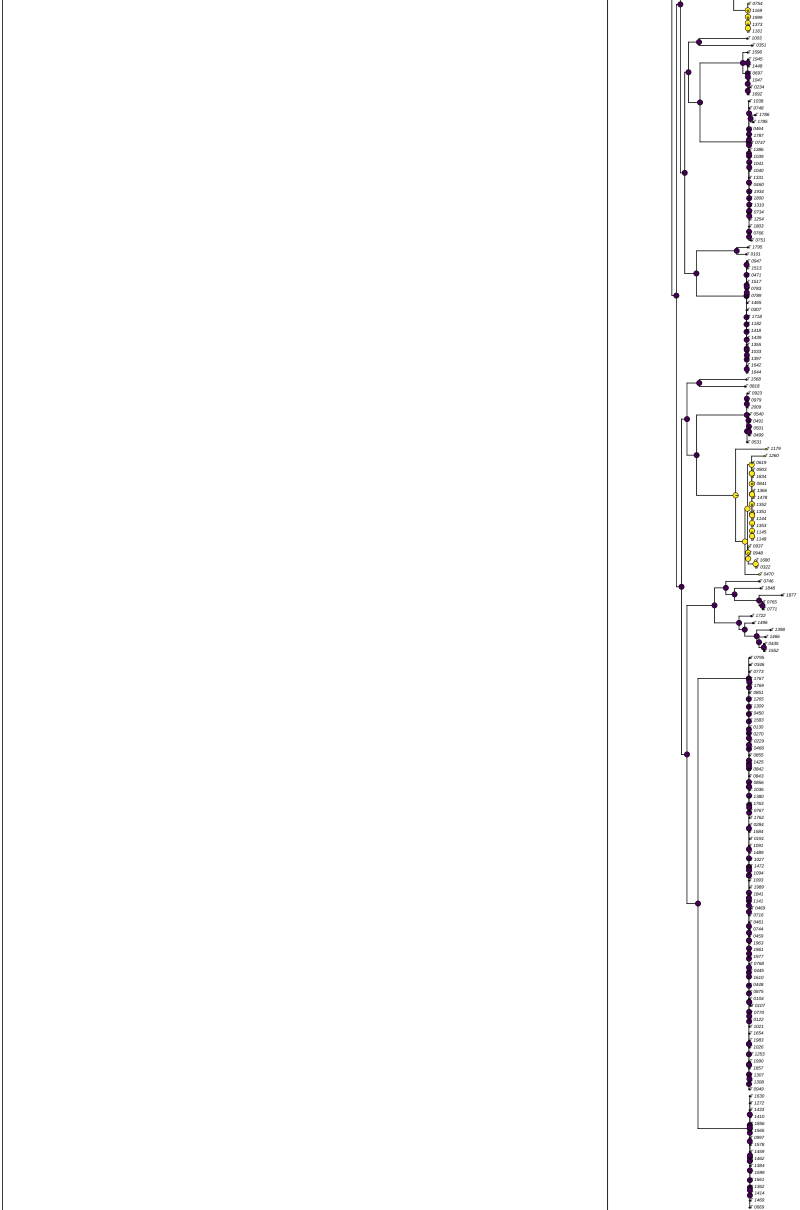
0754
1169
1999
1373
1161
1003
0351
1596
1945
1448
0697
1047
0234
1692
1038
0748
1786
1785
0464
1787
0747
1386
1039
1041
1040
1331
0460
1934
1800
1310
0734
1254
1803
0766
0751
1795
0101
0947
1513
0471
1517
0783
0789
1465
0307
1718
1182
1418
1439
1355
1033
1397
1642
1644
1568
0818
0923
0979
2009
0540
0491
0501
0499
0531
1179
1260
0619
0903
1834
0841
1366
1478
1352
1351
1144
1353
1145
1148
0937
0948
1680
0322
0470
0746
1848
1877
0765
0771
1722
1496
1398
1466
0435
1552
0795
0346
0773
1767
1769
0851
1265
1309
0450
1583
0130
0270
0229
0468
0855
1425
0842
0843
0856
1036
1380
1763
0767
1762
0284
1584
0191
1091
1489
1027
1472
1094
1093
1989
1841
1141
0469
0716
0461
0744
0459
1963
1961
1977
0768
0445
1610
0448
0875
0104
0107
0770
0122
1021
1654
1983
1026
1253
1990
1857
1307
1308
0949
1630
1272
1433
1410
1856
1565
0997
1578
1459
1462
1384
1599
1661
1362
1414
1469
0669

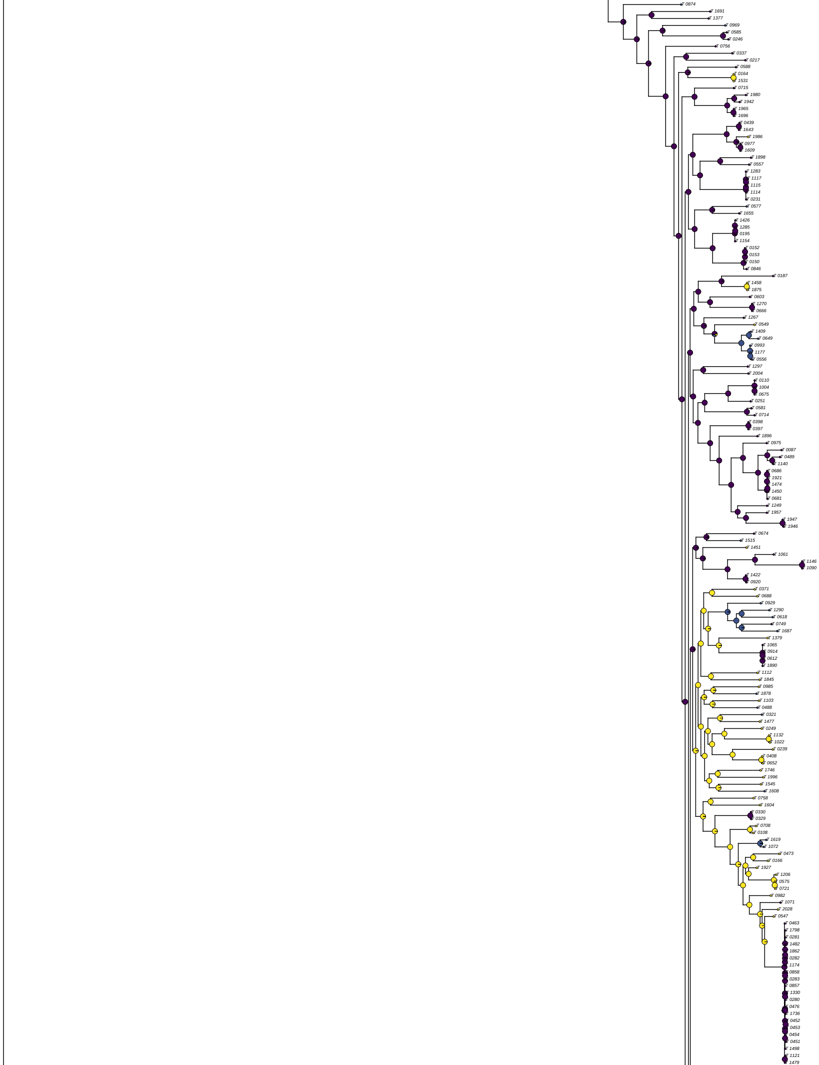
T 0874
T 1691
T 1377
T 0969
T 0585
T 0246
T 0756
T 0337
T 0217
T 0588
T 0164
T 1531
T 0715
T 1980
T 1942
T 1965
T 1696
T 0439
T 1643
T 1986
T 0977
T 1609
T 1898
T 0557
T 1283
T 1117
T 1115
T 1114
T 0231
T 0577
T 1655
T 1426
T 1285
T 0195
T 1154
T 0152
T 0153
T 0150
T 0846
T 0187
T 1458
T 1875
T 0603
T 1270
T 0666
T 1267
T 0549
T 1409
T 0649
T 0993
T 1177
T 0556
T 1297
T 2004
T 0110
T 1004
T 0675
T 0251
T 0581
T 0714
T 0398
T 0397
T 1896
T 0975
T 0087
T 0489
T 1140
T 0686
T 1921
T 1474
T 1450
T 0681
T 1249
T 1957
T 1947
T 1946
T 0674
T 1515
T 1451
T 1061
T 1146
T 1090
T 1422
T 0920
T 0371
T 0688
T 0929
T 1290
T 0618
T 0749
T 1687
T 1379
T 1065
T 0914
T 0612
T 1890
T 1112
T 1845
T 0985
T 1878
T 1103
T 0488
T 0321
T 1477
T 0249
T 1132
T 1022
T 0239
T 0408
T 0652
T 1746
T 1996
T 1545
T 1608
T 0758
T 1604
T 0330
T 0329
T 0708
T 0108
T 1619
T 1072
T 0473
T 0166
T 1927
T 1206
T 0575
T 0721
T 0982
T 1071
T 2028
T 0547
T 0463
T 1798
T 0281
1482
1862
0282
1174
0858
0283
T 0857
1330
T 0280
T 0476
T 1736
0452
0453
0454
T 0451
T 1498
T 1121
T 1479

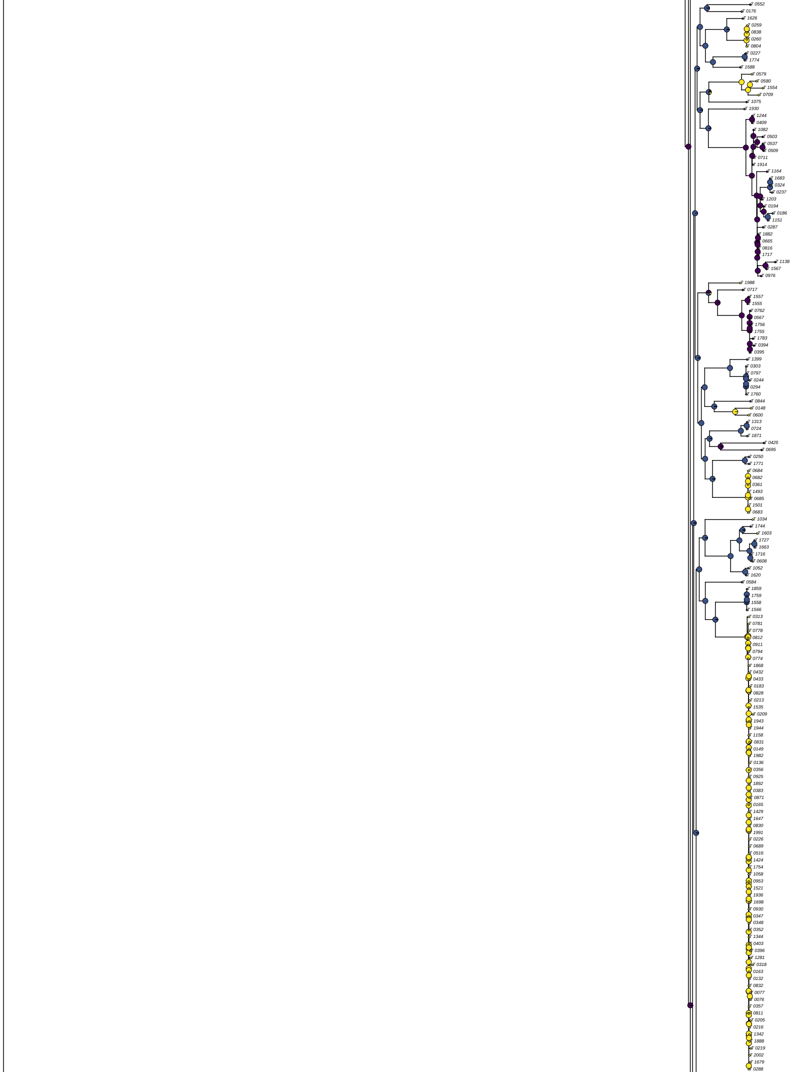
T 0552
T 0176
T 1626
T 0259
T 0838
T 0260
T 0804
T 0227
T 1774
T 1588
T 0579
T 0580
T 1554
T 0709
T 1075
T 1930
T 1244
T 0409
T 1082
T 0503
T 0537
T 0509
T 0711
T 1914
T 1164
T 1683
T 0324
T 0237
T 1203
T 0194
T 0186
T 1151
T 0287
T 1882
T 0665
T 0816
T 1717
T 1138
T 1567
T 0976
T 1988
T 0717
T 1557
T 1555
T 0762
T 0567
T 1756
T 1755
T 1783
T 0394
T 0395
T 1399
T 0303
T 0797
T 0244
T 0294
T 1760
T 0844
T 0148
T 0600
T 1313
T 0724
T 1871
T 0425
T 0695
T 0250
T 1771
T 0684
T 0682
T 0361
T 1493
T 0685
T 1501
T 0683
T 1034
T 1744
T 1603
T 1727
T 1663
T 1716
T 0608
T 1052
T 1620
T 0584
T 1859
T 1759
T 1558
T 1566
T 0313
T 0781
T 0778
T 0812
T 0911
T 0794
T 0774
T 1868
T 0432
T 0433
T 0183
T 0828
T 0213
T 1535
T 0209
T 1943
T 1944
T 1158
T 0831
T 0149
T 1982
T 0136
T 0356
T 0925
T 1892
T 0383
T 0871
T 0165
T 1429
T 1647
T 0830
T 1991
T 0226
T 0689
T 0516
T 1424
T 1754
T 1058
T 0953
T 1521
T 1936
T 1698
T 0930
T 0347
T 0348
T 0352
T 1344
T 0403
T 0396
T 1281
T 0318
T 0163
T 0132
T 0832
T 0077
T 0076
T 0357
T 0811
T 0205
T 0216
T 1342
T 1888
T 0219
T 2002
T 1679
T 0288

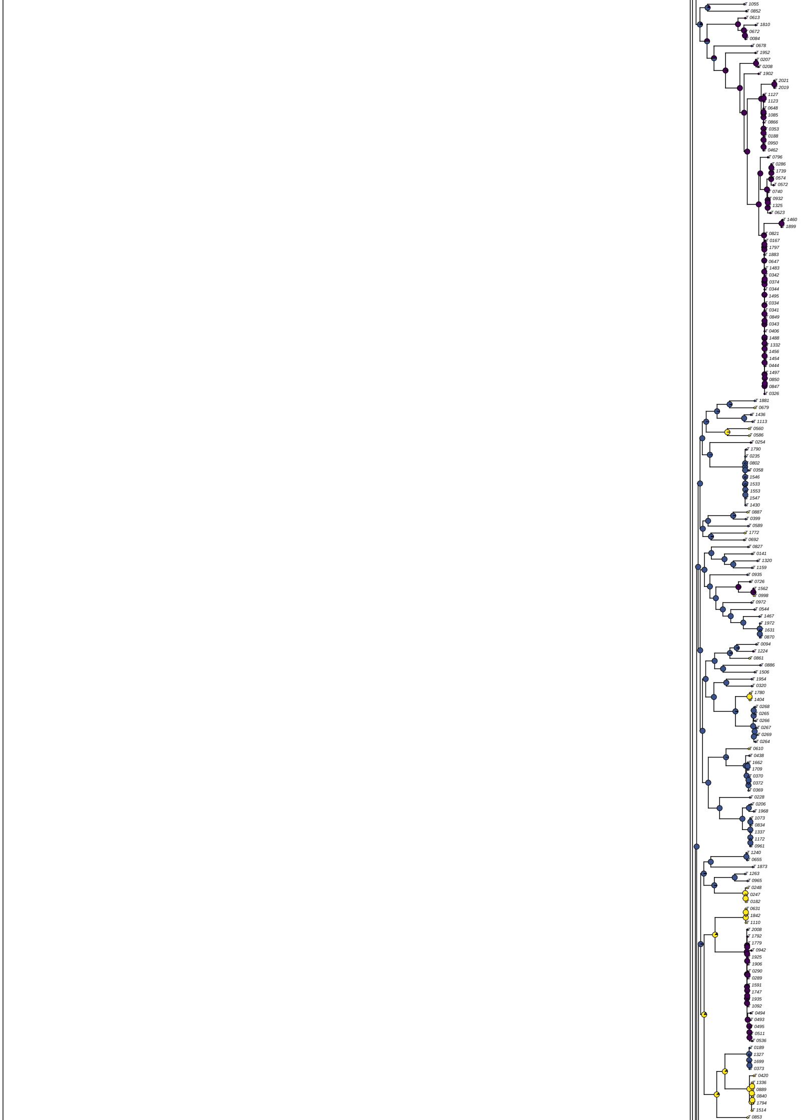

T 1055
T 0852
T 0613
T 1810
0672
T 0084
T 0678
T 1952
T 0207
T 0208
T 1902
T 2021
T 2019
T 1127
1123
T 0648
1085
T 0866
T 0353
T 0188
T 0950
T 0462
T 0796
T 0286
1739
T 0574
T 0572
0740
T 0932
1325
T 0623
T 1460
T 1899
T 0821
T 0167
T 1797
T 1883
0647
T 1483
T 0342
T 0374
T 0344
1495
T 0334
T 0341
T 0849
0343
T 0406
1488
T 1332
T 1456
T 1454
T 0444
T 1497
T 0850
T 0847
T 0326
T 1881
T 0679
T 1436
T 1113
T 0560
T 0586
T 0254
T 1790
T 0235
0802
T 0358
1546
1533
1553
T 1547
T 1430
T 0887
T 0399
T 0589
T 1772
T 0692
T 0827
T 0141
T 1320
T 1159
T 0935
T 0726
T 1562
T 0998
T 0972
T 0544
T 1467
T 1972
T 1631
T 0870
T 0094
T 1224
T 0861
T 0886
T 1506
T 1954
T 0320
T 1780
T 1404
T 0268
T 0265
T 0266
T 0267
T 0269
T 0264
T 0610
T 0438
T 1662
T 1709
T 0370
0372
T 0369
T 0228
T 0206
T 1968
T 1073
T 0834
1337
1172
T 0961
T 1240
T 0655
T 1873
T 1263
T 0965
T 0248
0247
T 0182
T 0631
1842
T 1110
T 2008
T 1792
T 1779
T 0942
1925
T 1906
T 0290
T 0289
1591
1747
1935
T 1092
T 0494
T 0493
0495
0511
T 0536
T 0189
1327
1699
T 0373
T 0420
T 1336
0889
T 0840
T 1794
T 1514
T 0853

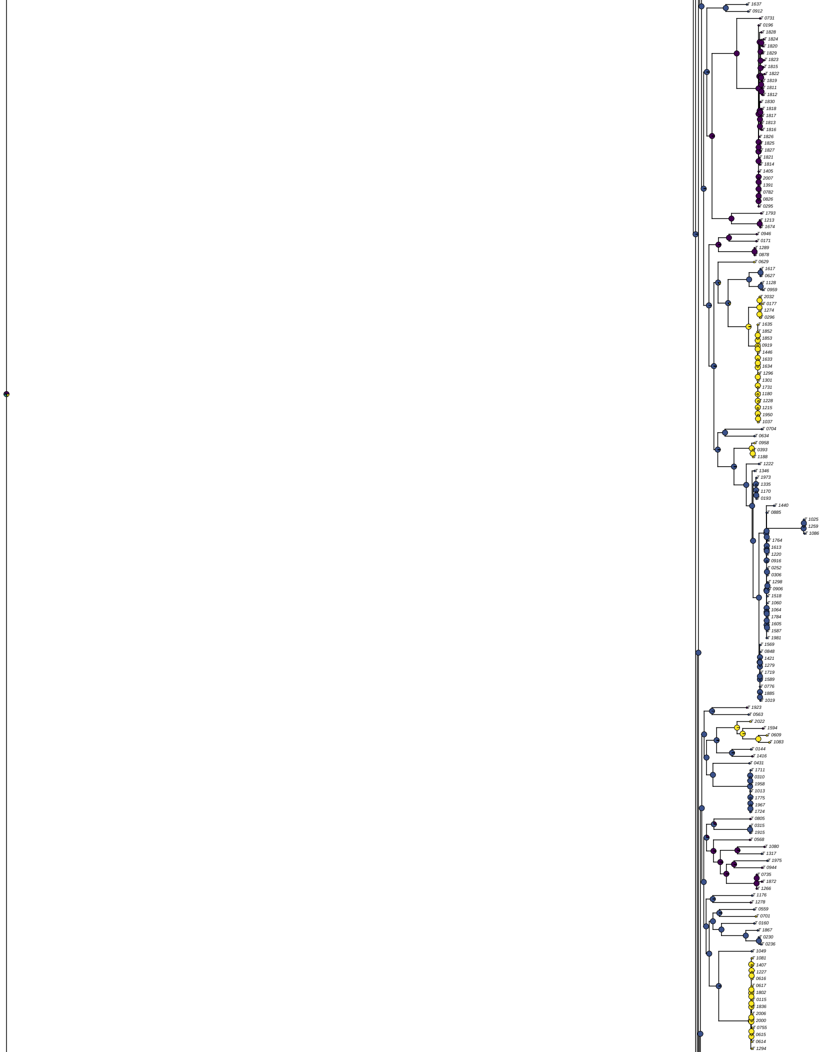
T 1637
T 0912
T 0731
T 0196
T 1828
T 1824
T 1820
T 1829
T 1823
T 1815
T 1822
T 1819
T 1811
T 1812
T 1830
T 1818
T 1817
T 1813
T 1816
T 1826
T 1825
T 1827
T 1821
T 1814
T 1405
2007
1391
0782
0826
T 0295
T 1793
T 1213
T 1674
T 0946
T 0171
T 1289
T 0878
T 0629
T 1617
T 0627
T 1128
T 0959
T 2032
T 0177
T 1274
T 0296
T 1635
T 1852
1853
0919
1446
1633
1634
T 1296
1301
1731
1180
1228
1215
1950
1037
T 0704
T 0634
T 0958
0393
1188
T 1222
T 1346
T 1973
1335
1170
0193
T 1440
T 0885
T 1025
1259
T 1086
T 1764
1613
T 1220
0916
T 0252
0306
T 1298
T 0906
T 1518
T 1060
1064
T 1784
1605
T 1587
T 1981
T 1569
T 0848
1421
1279
T 1719
1589
T 0776
1885
T 1019
T 1923
T 0563
T 2022
T 1594
T 0609
T 1083
T 0144
T 1416
T 0431
T 1711
0310
1958
T 1013
1775
1967
1724
T 0805
T 0315
1915
T 0568
T 1080
T 1317
T 1975
T 0944
T 0735
T 1872
T 1266
T 1176
T 1278
T 0559
T 0701
T 0160
T 1867
T 0230
T 0236
T 1049
T 1081
1407
T 1227
0616
T 0617
1802
T 0115
1836
T 2006
2000
T 0755
0615
T 0614
T 1294

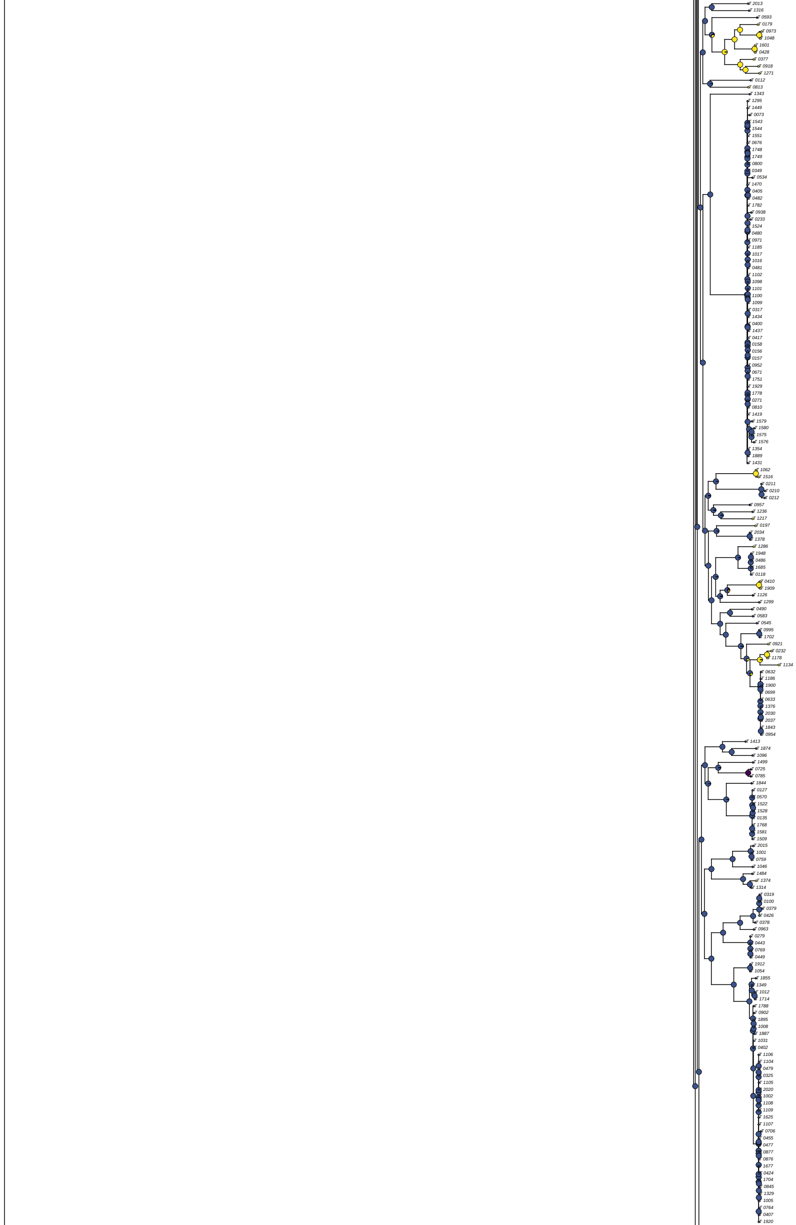
T 2013
T 1316
T 0593
T 0179
T 0973
T 1048
T 1601
T 0428
T 0377
T 0918
T 1271
T 0112
T 0813
T 1343
T 1295
T 1449
T 0073
T 1543
T 1544
T 1551
T 0676
T 1748
T 1749
T 0800
T 0349
T 0534
T 1470
T 0405
T 0482
T 1782
T 0938
T 0233
T 1524
T 0480
T 0971
T 1185
T 1017
T 1016
T 0481
T 1102
T 1098
T 1101
T 1100
T 1099
T 0317
T 1434
T 0400
T 1437
T 0417
T 0158
T 0156
T 0157
T 0952
T 0671
T 1751
T 1929
T 1778
T 0271
T 0810
T 1419
T 1579
T 1580
T 1575
T 1576
T 1354
T 1889
T 1431
T 1062
T 1516
T 0211
T 0210
T 0212
T 0957
T 1236
T 1217
T 0197
T 2034
T 1378
T 1286
T 1948
T 0486
T 1685
T 0118
T 0410
T 1909
T 1126
T 1299
T 0490
T 0583
T 0545
T 0995
T 1702
T 0921
T 0232
T 1178
T 1134
T 0632
T 1186
T 1900
T 0699
T 0633
T 1376
T 2030
T 2037
T 1843
T 0954
T 1413
T 1874
T 1096
T 1499
T 0725
T 0785
T 1844
T 0127
T 0570
T 1522
T 1528
T 0135
T 1768
T 1581
T 1509
T 2015
T 1001
T 0759
T 1046
T 1484
T 1374
T 1314
T 0319
T 0100
T 0379
T 0426
T 0378
T 0963
T 0279
T 0443
T 0769
T 0449
T 1912
T 1054
T 1855
T 1349
T 1012
T 1714
T 1788
T 0902
T 1895
T 1008
T 1887
T 1031
T 0402
T 1106
T 1104
T 0479
T 0325
T 1105
T 2020
T 1002
T 1108
T 1109
T 1625
T 1107
T 0706
T 0455
T 0477
T 0877
T 0876
T 1677
T 0424
T 1704
T 0845
T 1329
T 1005
T 0764
T 0407
T 1920

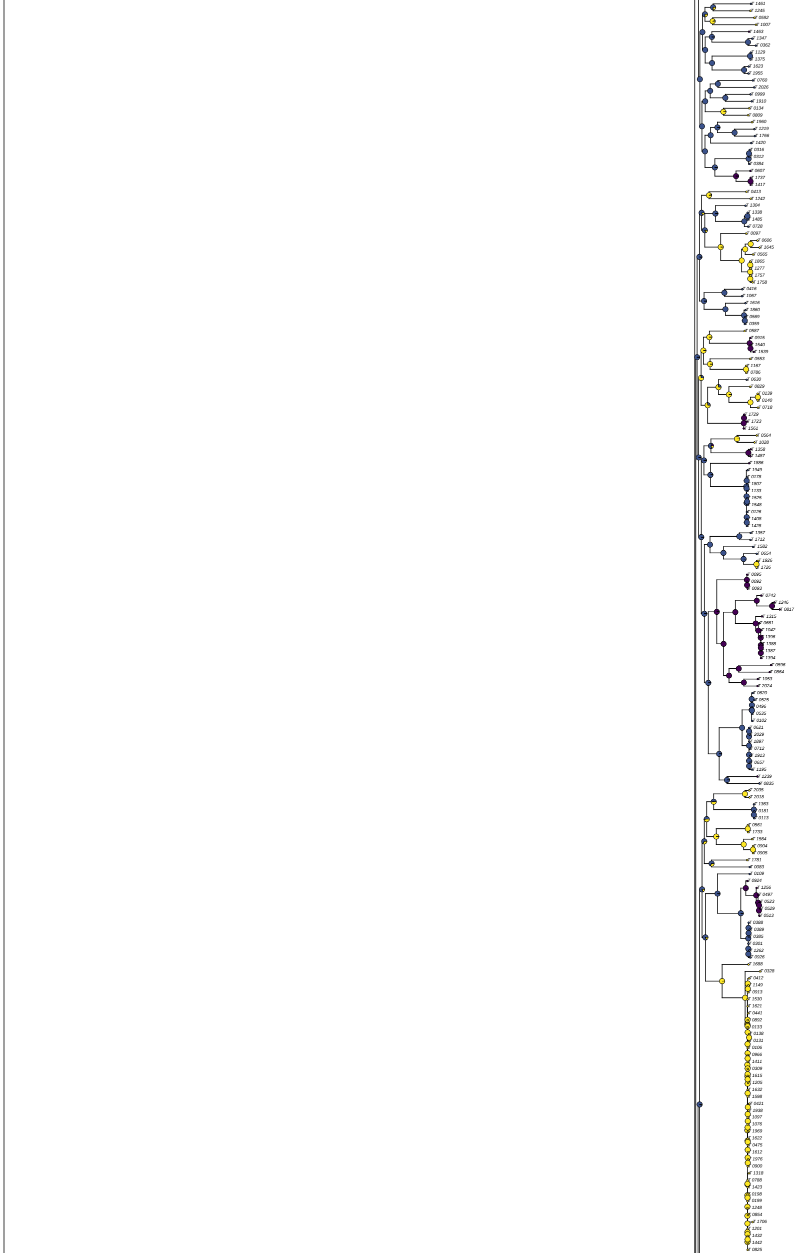

T 1461
T 1245
T 0592
T 1007
T 1463
T 1347
T 0362
T 1129
T 1375
T 1623
T 1955
T 0760
T 2026
T 0999
T 1910
T 0134
T 0809
T 1960
T 1219
T 1766
T 1420
T 0316
0312
T 0384
T 0607
T 1737
T 1417
T 0413
T 1242
T 1304
T 1338
T 1485
T 0728
T 0097
T 0606
T 1645
T 0565
T 1865
1277
T 1757
T 1758
T 0416
T 1067
T 1616
T 1860
0569
0359
T 0587
T 0915
1540
T 1539
T 0553
T 1167
0786
T 0630
T 0829
T 0139
0140
T 0718
T 1729
T 1723
T 1561
T 0564
T 1028
T 1358
T 1487
T 1886
T 1949
T 0178
1807
1133
1525
1548
T 0126
1408
1428
T 1357
T 1712
T 1582
T 0654
T 1926
1726
T 0095
0092
0093
T 0743
T 1246
T 0817
T 1315
T 0661
T 1042
1396
1388
1387
T 1394
T 0596
T 0864
T 1053
T 2024
T 0620
T 0525
0496
T 0535
T 0102
T 0621
2029
T 1897
T 0712
1913
0657
T 1195
T 1239
T 0835
T 2035
T 2018
T 1363
0181
0113
T 0561
1733
T 1564
T 0904
0905
T 1781
T 0083
T 0109
T 0924
T 1256
T 0497
T 0523
T 0529
T 0513
T 0388
0389
0385
T 0301
T 1262
T 0926
T 1688
T 0328
T 0412
1149
0913
T 1530
T 1621
T 0441
0892
0133
T 0138
0131
0106
0966
1411
0309
1615
1205
T 1632
1598
T 0421
1938
1097
1076
1969
1622
0475
1612
1976
0900
T 1318
T 0788
1423
0198
0199
1248
0854
T 1706
T 1201
1432
1442
T 0825

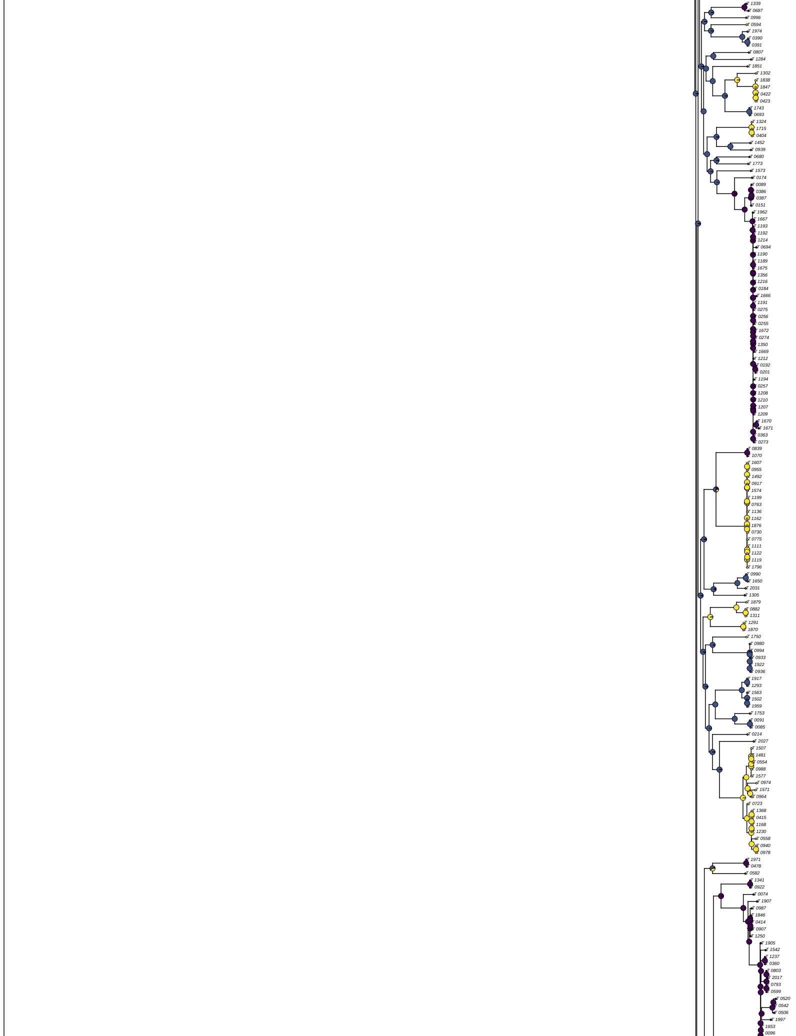
T 1339
T 0687
T 0996
T 0594
T 1974
T 0390
T 0391
T 0807
T 1284
T 1851
T 1302
T 1838
T 1847
T 0422
T 0423
T 1743
T 0693
T 1324
T 1715
T 0404
T 1452
T 0939
T 0680
T 1773
T 1573
T 0174
T 0089
T 0386
T 0387
T 0151
T 1962
T 1667
T 1193
T 1192
T 1214
T 0694
T 1190
T 1189
T 1675
T 1356
T 1216
T 0184
T 1666
T 1191
T 0275
T 0256
T 0255
T 1672
T 0274
T 1350
T 1669
T 1212
T 0192
T 0201
T 1194
T 0257
T 1208
T 1210
T 1207
T 1209
T 1670
T 1671
T 0363
T 0273
T 0839
T 1070
T 1607
T 0955
T 1492
T 0917
T 1574
T 1199
T 0763
T 1136
T 1162
T 1876
T 0730
T 0775
T 1111
T 1122
T 1119
T 1796
T 0990
T 1650
T 2031
T 1305
T 1879
T 0882
T 1311
T 1291
T 1870
T 1750
T 0980
T 0994
T 0933
T 1922
T 0936
T 1917
T 1293
T 1563
T 1502
T 1959
T 1753
T 0091
T 0085
T 0214
T 2027
T 1507
T 1481
T 0554
T 0988
T 1577
T 0974
T 1571
T 0964
T 0723
T 1368
T 0415
T 1168
T 1230
T 0558
T 0940
T 0978
T 1971
T 0478
T 0582
T 1341
T 0922
T 0074
T 1907
T 0987
T 1846
T 0414
T 0907
T 1250
T 1905
T 1542
T 1237
T 0360
T 0803
T 2017
T 0793
T 0599
T 0520
T 0542
T 0506
T 1997
T 1653
T 0096

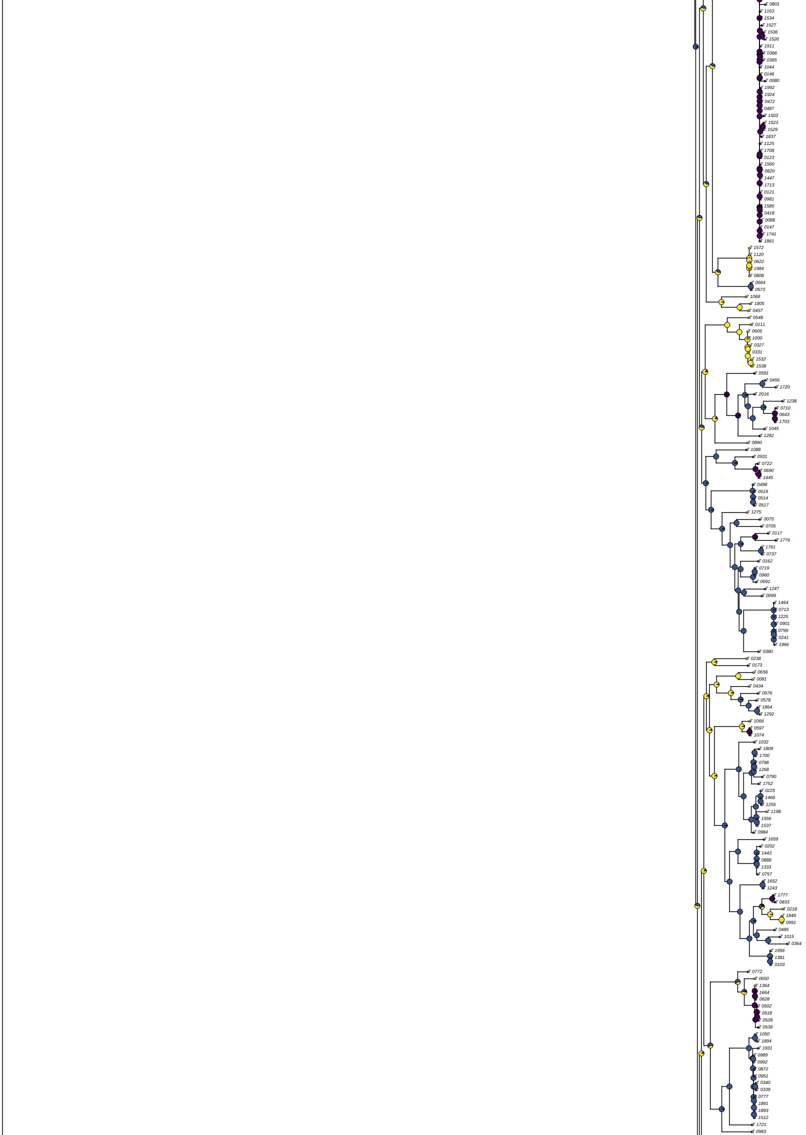

T 0801
T 1163
1534
T 1527
T 1536
T 1526
T 1911
T 0366
T 0365
T 1044
T 0146
T 0080
T 1992
1924
T 0472
0487
T 1503
T 1523
T 1529
T 1837
T 1125
T 1708
0123
T 1560
0820
T 1447
T 1713
T 0121
0981
T 1585
T 0418
T 0088
T 0147
T 1741
T 1861
T 1572
T 1120
0622
1984
T 0808
T 0664
T 0573
T 1068
T 1805
T 0457
T 0548
T 0111
T 0605
T 1000
T 0327
0331
T 1532
T 1538
T 0591
T 0456
T 1720
T 2016
T 1238
T 0710
0643
T 1703
T 1045
T 1282
T 0890
T 1088
T 0931
T 0722
T 0690
1445
T 0498
T 0519
0514
T 0517
T 1275
T 0075
T 0705
T 0117
T 1776
T 1761
T 0737
T 0162
T 0719
T 0960
T 0691
T 1247
T 0099
T 1464
0713
1225
T 0901
0799
0241
T 1966
T 0380
T 0238
T 0173
T 0656
T 0081
T 0434
T 0576
T 0578
T 1864
T 1292
T 1066
T 0597
T 1074
T 1032
T 1809
T 1700
T 0798
T 1268
T 0790
T 1752
T 0225
1468
T 1255
T 1198
1556
T 1537
T 0984
T 1659
T 0202
1443
0888
T 1333
T 0757
T 1652
T 1243
T 1777
T 0833
T 0218
T 1849
T 0991
T 0485
T 1015
T 0364
T 1956
1381
T 0103
T 0772
T 0650
T 1364
T 1664
T 0628
T 0502
T 0518
T 0528
T 0539
T 1050
T 1894
T 1931
0989
T 0992
T 0872
T 0951
T 0340
T 0339
T 0777
T 1891
T 1893
T 1512
T 1721
T 0983

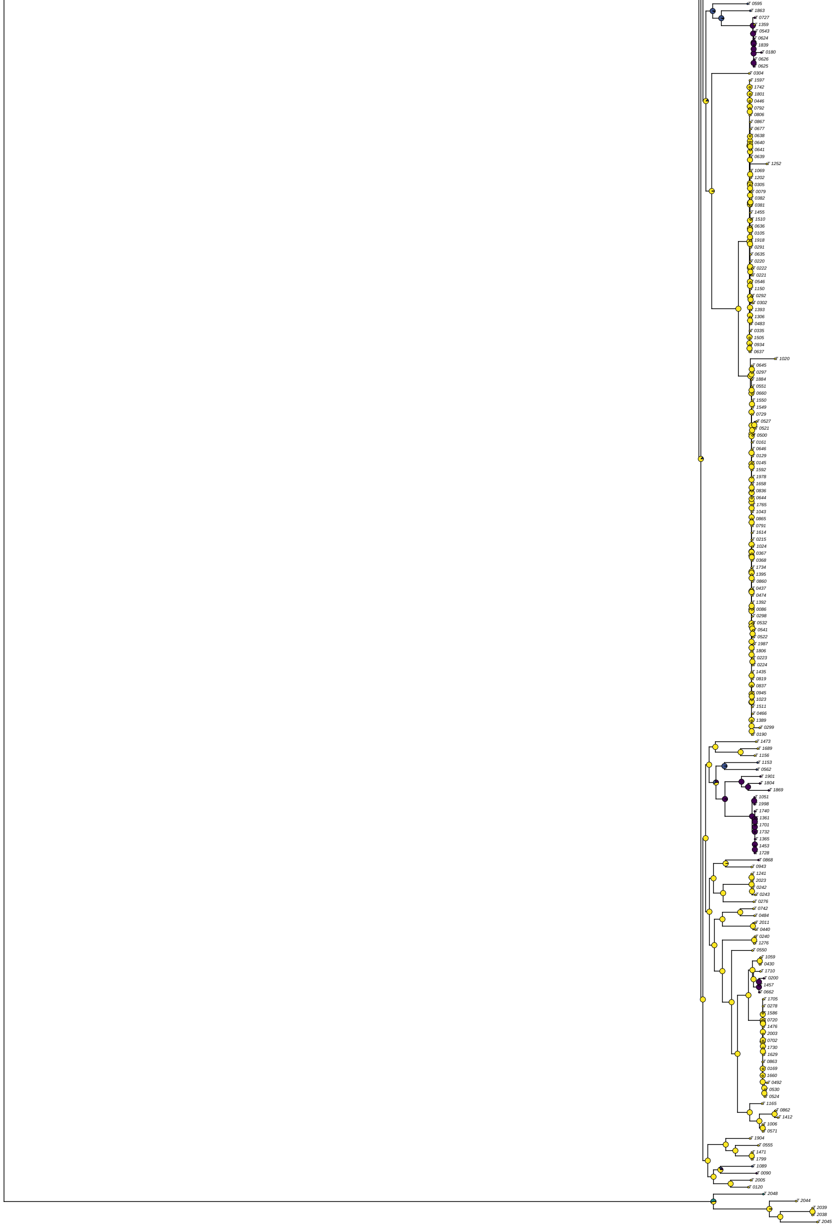

Supplement: Supplementary file 25 — Supplementary Dataset 23 [file 41467_2024_54649_MOESM25_ESM.pdf]
